# Enantioselective Organocatalytic Conjugate Addition of Malonates to β,β-Disubstituted β-Trifluoromethyl Enones under High Pressure

**DOI:** 10.1021/acs.orglett.5c00065

**Published:** 2025-02-24

**Authors:** Alicja J. Połosak, Michał P. Głowacki, Piotr Kwiatkowski

**Affiliations:** Faculty of Chemistry, Biological and Chemical Research Centre, University of Warsaw, Żwirki i Wigury 101, 02-089 Warsaw, Poland

## Abstract

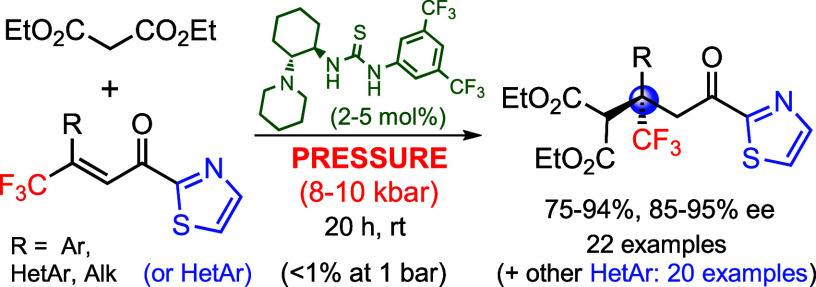

The first enantioselective Michael addition of malonates to acyclic β,β-disubstituted enones has been developed. Sterically hindered β-trifluoromethyl α,β-unsaturated 2-acyl thiazoles and benzothiazoles were found to be the most reactive groups of enones in the reaction catalyzed by bifunctional tertiary amine–thioureas (2–5 mol %). However, application of hyperbaric conditions (8–10 kbar) was required. The adducts containing quaternary stereogenic centers with a CF_3_ group were obtained in high yields (vs <1% at 1 bar) with enantiomeric excesses up to 95%.

The development of new methods for enantioselective construction of all-carbon quaternary stereogenic centers is still one of the most challenging directions in asymmetric catalysis.^1^ Among different strategies to achieve this goal, one possibility relies on the conjugate addition of carbon nucleophiles to β,β-disubstituted Michael acceptors (Scheme 1a). However, this approach is still of limited applicability and generally difficult due to the significantly lower reactivity of sterically demanding trisubstituted electron-deficient alkenes.

**Scheme 1:**
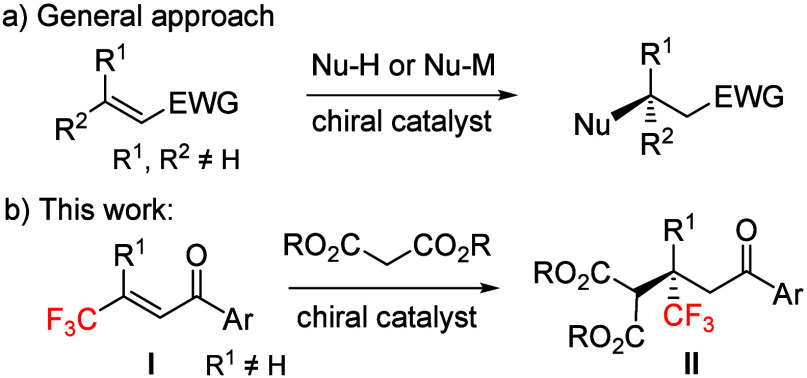
Construction of Quaternary Stereocenters at the β-Position via Conjugate Addition

Among enantioselective conjugate additions to acyclic β,β-disubstituted α,β-unsaturated carbonyl compounds generating all-carbon quaternary stereogenic centers,^1b,1c^ in the literature prevail examples of reactions catalyzed by transition metal complexes, including organometallic^2^ and selected non-organometallic nucleophiles.^3^ The organocatalytic variant is mostly limited to reactions with β,β-disubstituted nitroalkenes^4,5^ and cyclic enones. More challenging and rare are corresponding organocatalytic 1,4-additions to β,β-disubstituted enals^6^ and acyclic enones,^7^ so far reported mainly with smaller C-nucleophiles, e.g. nitromethane.

We focused our attention on organocatalytic reactions of β-trifluoromethyl β,β-disubstituted enones of type **I**(7b−7d) (Scheme 1b) with malonates, enabling the formation of adducts **II** with quaternary trifluoromethylated stereogenic centers.^8^ Asymmetric conjugate addition of malonates to β-monosubstituted enones was extensively investigated over the last two decades, especially organocatalytic versions.^9^ However, no effective methods were developed for the corresponding reaction with β,β-disubstituted enones.^10^ Only single protocols for the asymmetric addition of malonates to specific β,β-disubstituted enals^6b^ and nitroolefins^5a^ have been reported. An alternative strategy is based on the use of more reactive thiomalonates and β-disubstituted nitroalkenes.^5b,5c^ The use of β,β-disubstituted β-CF_3_ enones of type **I** in conjugated addition reactions, in particular in organocatalytic variants, is still rare and challenging.^7b−7d^ We demonstrated efficient enantioselective addition of nitromethane to enones **I** in the presence of chiral tertiary amine–thioureas (e.g., **1a** and **1e**, Figure 1) under high-pressure conditions (8–10 kbar).^7d,11^

**Figure 1:**
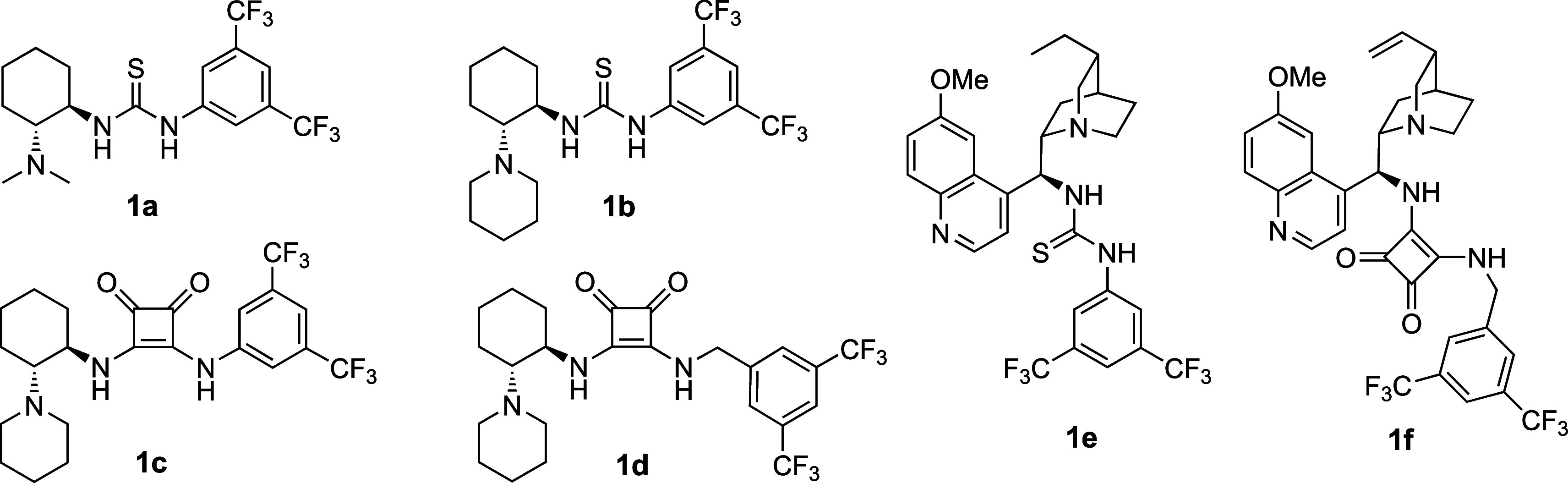
Organocatalysts examined in the studies.

Herein, we report the first highly enantioselective Michael addition of malonates to acyclic β,β-disubstituted enones. The initial studies with β-trifluoromethyl chalcone **2a** (Scheme 2, R = Ph), diethyl malonate, and amine–thioureas **1a** and **1e** (Figure 1) were unsuccessful even under high pressure.^11^ Product **3a** was formed at 10 kbar only in trace amounts (∼2%, Scheme 2). In contrast, the analogous reaction of MeNO_2_ with **2a** afforded the γ-nitroketone in high yield (>95%).^7d^ During our further studies, it turned out that the type of substituent on the carbonyl is of key importance, and the presence of some heteroaromatic groups allowed for significant yield improvement (Scheme 2).

**Scheme 2:**
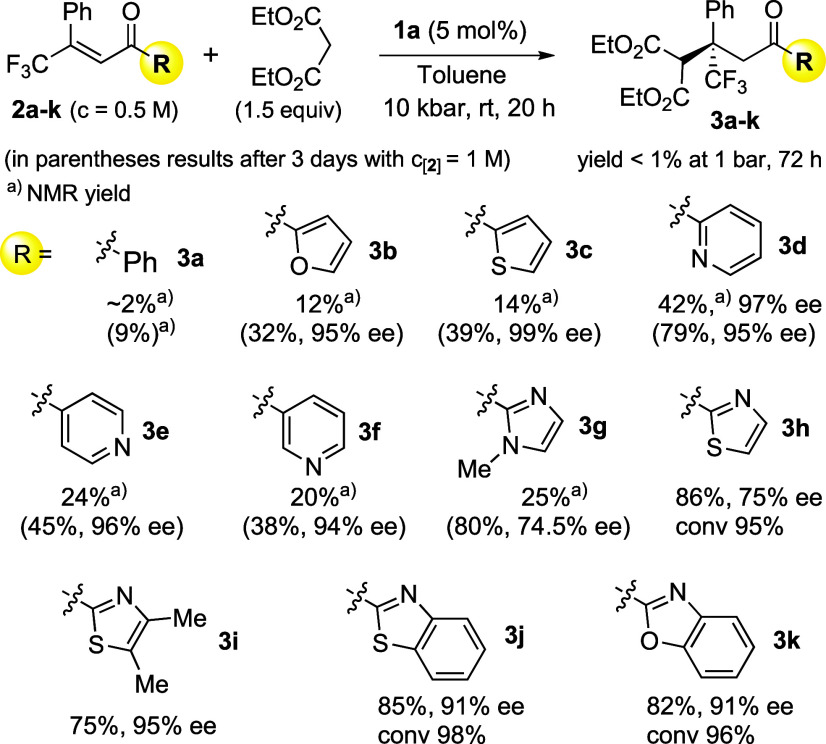
Reactivity of Enones and Effect of the Acyl Group

More promising results and high enantioselectivity were observed with enones containing 2-furyl (**3b**) and 2-thienyl (**3c**) substituents (up to 39% yield). Under the same reaction conditions, an enone bearing a 2-pyridyl group (**2d**) allowed us to obtain the product **3d** in acceptable yield with high enantioselectivity. However, changing the position of nitrogen in the pyridine ring (**3e** and **3f**) resulted in a significant yield decrease. Further modifications of substituents on the carbonyl group were focused on five-membered heterocycles containing at least one nitrogen atom. It turned out that thiazolyl enone **2h** showed significantly higher activity compared to the previously tested Michael acceptors. Product **3h** was obtained in high yield but with moderate enantioselectivity (75% ee). A similar level of enantioselectivity was observed for acyl imidazole acceptor **2g**, but it showed significantly lower reactivity. Finally, the use of substituted thiazole derivatives, including benzothiazole **2j** as well as benzoxazole **2k**, allowed over 90% ee and good yields to be obtained (see **3i**–**3k**). So far, only a few single examples of the application of α,β-unsaturated 2-acyl thiazoles in enantioselective conjugate additions have been described in the literature.^12^

The results presented in Scheme 2 allowed us to select a group of enones that offer high enantioselectivities and acceptable yields (see **3d**, **3i**, **3j**, and **3k**), but we were particularly interested in the possibility of using a simple thiazole acceptor (**2h**) because of several arguments: more efficient and easier synthesis,^13,14^ better solubility in less polar organic solvents, and well-described procedures for converting the simple thiazole ring into a formyl group,^15^ which was applied in the synthesis of carbohydrates.^16^ Moreover, many thiazole derivatives exhibit interesting properties, especially related to biological activity.^17^

In the course of further research with **2h**, it turned out that a simple modification of the Takemoto catalyst^18^ in the amine part (**1b**) resulted in high enantioselectivity (92% ee; Table 1, entry 2) under 10 kbar. Other tested catalysts (**1d**–**1f**) were less effective in terms of enantioselectivity.^14^ Moreover, in all control experiments under atmospheric pressure after 7 days, only traces of product were detected (<0.5%).

**Table 1:**
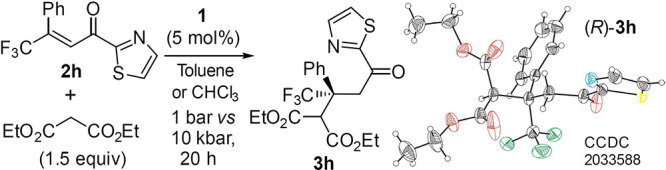
Catalyst Screening in the Model Reaction^a^

		1 bar (7 days)	10 kbar (20 h)	
entry	catalyst (5 mol %)	yield [%]^b^	yield [%]^b^,^c^	ee [%]^d^ (config.)
1	**1a**	0.3	91 (86)	75 (*R*)
2	**1b**	0.4	95 (88)	92 (*R*)
3^e^	**1b**	0.2	90	85 (*R*)
4^e^	**1c**^f^	<0.1	<1	–
5^e^	**1d**^g^	<0.1	90	42 (*R*)
6	**1e**	0.3	80	62 (*S*)^h^
7^e^	**1f**	0.1	85	77 (*S*)^h^
8	quinidine	<0.1	25	17 (*R*)

a Reaction conditions: **2a** (*E*/*Z* ≥ 98:2, 0.2 mmol, *c* = 0.5 mol/L), diethyl malonate (0.3 mmol, 1.5 equiv), and catalyst **1** (0.01 mmol, 5 mol %) in toluene (ca. 0.3 mL) at 20–25 °C.

b Determined by ^19^F NMR analysis.

c Numbers in parentheses are isolated yields.

d Determined by HPLC analysis using a Chiralpak IC column.

e Reaction in CHCl_3_.

f Very low solubility of **1c**.

g Incomplete solubility of **1d**.

h Product **3h** with opposite absolute configuration.

Further optimization studies (Table 2) indicated that the reaction of enones **2h** and **2j** is very difficult to perform under atmospheric pressure even at elevated temperature (entries 1 and 13; up to 3% for **3j** at 50 °C after 7 days). High pressure has a huge impact on the course of these reactions, and 8 kbar is required to obtain high yields (>90%; entry 3). For comparison, at 6 kbar products **3h** and **3j** were formed in moderate yields (65% and 50%; entries 2 and 14).^14^ The best results in terms of enantioselectivity (up to 94% ee) and yield were obtained under a pressure of 9–10 kbar. Benzothiazole enone **2j** is more reactive than **2h** (entry 16 vs 9, *c* = 0.5 M), but in other aspects thiazole enones are more attractive. Higher concentrations of enone **2h** (1.0 M vs 0.5 M) significantly improved the reaction yield (entries 8 and 9). 1,4-Addition of dimethyl malonate (entry 5) was slightly less enantioselective compared to the ethyl ester. Typical high-pressure experiments presented so far were carried out for 20 h; however, a shorter time (e.g., 5 h) also allowed the product to be obtained in good yields (>80%; entries 6 and 15). The results were also satisfactory when the catalyst loading was reduced to 2 mol % at 9 kbar for 20 h (entries 8, 16, and 17).

**Table 2:** Optimization of the Reaction Conditions^a^

entry	enone (mol/L)	catalyst (mol %)	pressure (kbar)	time (h)	yield [%]^b^^,^^c^	ee [%]^d^^,^^e^
1	**2h** (1.0)	**1b** (5)	0.001^f^	168	0.6	–
2	**2h** (1.0)	**1b** (5)	6	20	65	89
3	**2h** (1.0)	**1b** (5)	8	20	93	90
4	**2h** (1.0)	**1b** (5)	9	20	96 (90)^g^	93^e^
5^h^	**2h** (1.0)^i^	**1b** (5)	9	20	86 (80)	87
6	**2h** (1.0)	**1b** (5)	9	5	82	95
7	**2h** (1.0)	**1b** (5)	9	3	68	94
8	**2h** (1.0)	**1b** (2)	9	20	90 (85)	94
9	**2h** (0.5)	**1b** (2)	9	20	55	93
10	**2h** (1.0)^j^	**1b** (2)	10	20	94	92
11	**2h** (1.0)^k^	**1b** (1)	10	72	85	94
12^l^	(*Z*)-**2h** (0.5)	**1b** (5)	9	20	84	26^e^
13^m^	**2j** (1.0)	**1a** (5)	0.001^f^	168	∼3	–
14	**2j** (0.5)	**1a** (5)	6	20	50	90
15	**2j** (0.5)	**1a** (5)	9	5	87	91
16	**2j** (0.5)	**1a** (2)	9	20	91 (84)	93
17	**2j** (0.5)	**1b** (2)	9	20	93	92

a Reaction conditions: **2h** (*E*/*Z* ≥ 98:2, 0.2–0.4 mmol scale; 2–5 mmol scale for isolated yield) or **2j** (*E*/*Z* ≥ 98:2, 0.2 mmol scale, up to 1 mmol scale for isolated yield), diethyl malonate (1.5 equiv), and catalyst **1b** or **1a** (1–5 mol %) in toluene at 20–25 °C.

b Determined by NMR analysis.

c Numbers in parentheses are isolated yields.

d Determined by HPLC using a Chiralpak IC column.

e The absolute configuration of the main enantiomer of **3h** was (*R*).

f The reaction was performed at 50 °C.

g 5 mmol reaction scale: 73% isolated yield (purified by filtration and crystallization from EtOH) with 98% ee.

h Reaction with dimethyl malonate: product **3l** was formed.

i For *c*_**2h**_ = 0.5 mol/L: 63% yield with 79% ee (**3l**).

j For *c*_**2h**_ = 0.5 mol/L: 76% yield with 92% ee.

k For *c*_**2h**_ = 0.5 mol/L: 56% yield with 93% ee.

l Isomer (*Z*)-**2h** was used (*E/Z* ratio 1:9).

m ∼5% yield after 21 days (12 days at 50 °C, then 9 days at 70 °C) and significant catalyst decomposition was observed.

The absolute configuration of adduct **3h** was confirmed by X-ray crystallographic analysis (Table 1).^14^ The use of catalyst (1*R*,2*R*)-**1b** led to enantiomerically enriched product (*R*)-**3h**.^19^ An important issue affecting the enantioselectivity is the *E*/*Z* isomer ratio of the enone. So far, **2h** containing at least 98% *E* isomer was used. In the experiment with the isomer (*Z*)-**2h**, the same direction of asymmetric induction was observed, but the enantioselectivity was very low (26% ee; Table 2, entry 12). The explanation for this is the relatively fast isomerization of the less stable (*Z*)-enone in the presence of the catalyst. It was confirmed by mixing the *Z* isomer (95%) of **2h** with **1b** (5 mol %), and within 1 h (at 1 bar) the *E* isomer was formed in about 25% yield (6 h, *E*/*Z* 7:3; 24 h, *E*/*Z* 94:6).^14^ In the analogous experiment with pure *E* isomer, the *Z* isomer was detected in about 3% after 1 h and ∼5% after equilibrium was reached.

To evaluate the usefulness of this reaction, a series of β-trifluoromethyl α,β-unsaturated 2-acyl thiazoles were obtained by the Wittig reaction of trifluoromethyl ketones with the stabilized ylide prepared from commercially available 2-acetylthiazole.^13,14^ The scope of β-trifluoromethyl 2-thiazolyl enones (**4**, *c* = 1 M) in the reaction with diethyl malonate is presented in Scheme 3a. The additions were carried out with 5 mol % catalyst **1b** at pressures in the range of 9–10 kbar. This procedure works very well for products with *para-* and *meta-*substituted phenyls at the β-position (**5a**–**5k**, Scheme 3a). Unfortunately, *ortho*-substituted enones are unreactive (see *o*-OMe, **4l**). Practically full conversions were obtained for enones containing various heteroaromatic (**5m**–**5p**) and aliphatic (**5q**–**5u**) groups at the β-position. In general, β-alkyl enones are more reactive than β-aryl analogs, and for acceptors **4q**–**4s**, 8 kbar is sufficient to obtain high yields. In most cases, the observed enantioselectivity was in the range of 88–95% ee.

**Scheme 3:**
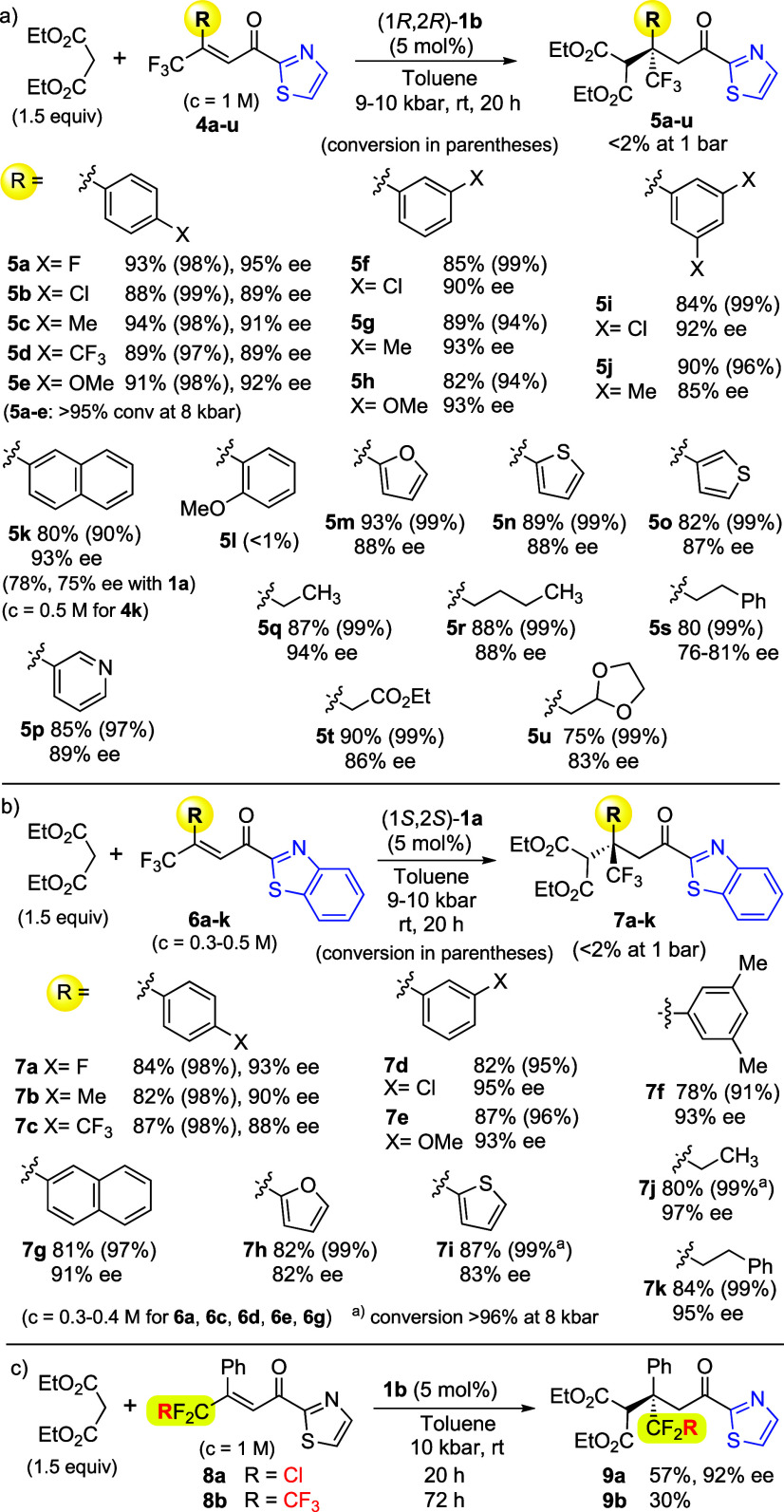
Scope of the β,β-Disubstituted Thiazolyl Enones

We also examined selected benzothiazole^15b^ enones **6a**–**6k** with ethyl malonate (Scheme 3b).^20^ These reactions are effective in the presence of commercially available Takemoto catalyst (**1a**) under a pressure of 9–10 kbar. The observed enantiomeric excesses for β-aryl derivatives (**7a**–**7g**) are generally similar to those of thiazole adducts. However, for products with heteroaromatic substituents (**7h** and **7i**) the ee is slightly lower, while in the case of β-alkyl groups (**7j** and **7k**), an improvement in enantioselectivity was observed. Finally, enones with other fluorinated groups were tested, e.g., CF_2_Cl and CF_2_CF_3_ (Scheme 3c). Reactions with **8a** and **8b** turned out to be more difficult compared to the model acceptor **2h**. In the case of the chlorodifluoromethyl derivative (**9a**), an acceptable yield and high enantiomeric excess were obtained; however, with a perfluoroethyl group (**9b**), the yield did not exceed 30%.

The thiazole ring is a very important motif in many biologically active compounds (e.g., thiamine, epothilone)^17^ and is used in synthesis as a masked equivalent of the formyl group.^15,16^Scheme 4 presents selected transformations of the thiazole adduct **3h** to interesting δ-keto acid **10a**, the corresponding δ-keto ester **10b**, and other cyclization products **10c**–**10e**. Direct Krapcho decarboxylation of **3h** was unsuccessful, and predominantly retro-Michael reaction was observed. Acid **10a** can be efficiently converted into 3,4-dihydropyran-2-one **10c**. Cyclopropane **10d** was obtained from adduct **3h** in the presence of iodine and DBU with moderate diastereoselectivity. Finally, **3h** heated with ammonium acetate cyclizes with decarboxylation to give trifluoromethylated 3,4-dihydro-2-pyridone **10e**. In addition, using **10e**, we presented the possibility of converting a thiazole substituent to a formyl group (product **10f**) according to the procedures developed by Dondoni.^15c^ The aldehyde, finally, was converted to α,β-unsaturated ester **10g**.

**Scheme 4:**
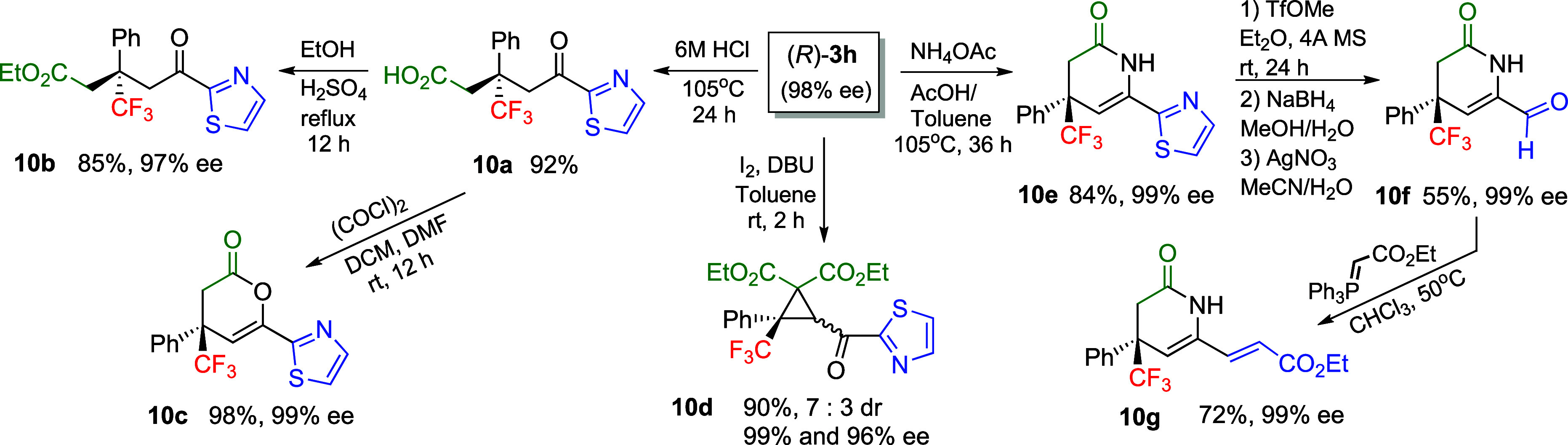
Synthetic Applications of Thiazole Adduct **3h**

In conclusion, we have developed the first highly enantioselective Michael addition of malonates to acyclic β,β-disubstituted enones. Furthermore, we have found that α,β-unsaturated 2-acyl thiazoles and benzothiazoles exhibit significantly higher activity in the organocatalytic Michael reaction compared to phenyl (**2a**) as well as other heterocyclic analogues (**2b**–**2g**). However, high-pressure conditions are required for successful 1,4-addition. This work demonstrates a very significant effect of hydrostatic pressure on the rate of the demanding organocatalytic reaction while retaining very high enantioselectivity. The reaction of selected sterically hindered β-trifluoromethyl enones with only 1.5 equiv of malonate is effectively accelerated under 8–10 kbar in the presence of bifunctional tertiary amine–thioureas (2–5 mol %) but practically does not occur at atmospheric pressure (<3%). The high-pressure approach allows for a very efficient asymmetric synthesis of 1,5-keto diesters containing an all-carbon quaternary stereogenic center with high enantioselectivity (up to 95% ee). Moreover 2-thiazolyl adducts **3h** and **5** are interesting precursors for the synthesis of cyclic derivatives **10c**–**10f**, including the possibility of converting a thiazole ring to a formyl group.

## Data Availability

The data underlying this study are available in the published article and its Supporting Information.

## Supporting Information Available

Experimental procedures, characterization data of new compounds, copies of NMR spectra and HPLC chromatograms, crystallographic data for **2h** and (*R*)-**3h**, and additional references (PDF)
