# Supplementary material for: Enantioselective Organocatalytic Conjugate Addition of Malonates to β,β-Disubstituted β-Trifluoromethyl Enones under High Pressure
Source: Org Lett. 2025 Feb 24;27(9):2087–92. doi: 10.1021/acs.orglett.5c00065 (PMC11894651; doi:10.1021/acs.orglett.5c00065)
Supplement: Supplementary file 1 — ol5c00065_si_001.pdf [file ol5c00065_si_001.pdf]

Enantioselective Organocatalytic Conjugate Addition of Malonates to β,β-Disubstituted β-Trifluoromethyl Enones under High-Pressure

Alicja J. Połosak, Michał P. Głowacki, and Piotr Kwiatkowski*

Faculty of Chemistry, Biological and Chemical Research Centre, University of Warsaw, Żwirki i Wigury 101, 02-089 Warsaw, Poland

pkwiat@chem.uw.edu.pl

Supporting Information

Table of Contents

**General Information**	S-2
**Starting materials**	S-3
**Enantioselective addition of diethyl malonate to enone 2h and 2j - additional information**	S-8
**General synthetic procedures**	S-10
**Analytical data of enones 2e-2k, 4a-4u, 6a-6k, 8a and 8b**	S-13
**Analytical data of products of malonate addition to enones under high pressure: 3a-3l, 5a-5k, 5m-5u, 7a-7k, 9a and 9b**	S-26
**Analytical data of compounds 10a-10g obtained from adduct 3h**	S-48
**HPLC chromatograms: 3b-3l, 5a-5k, 5m-5u, 7a-7k, 9a, 10b-10g**	S-53
**Crystallographic data: *(E)*-2h and *(R)*-3h**	S-78
**Copies of NMR spectra (^{1}H NMR + ^{19}F NMR miniature, ^{13}C NMR)**	S-86
**Copies of NMR spectra – enones 2e-2k, 4a-4u, 6a-6k, 8a and 8b**	S-86
**Copies of NMR spectra - products of malonate addition to enones: 3a-3l, 5a-5k, 5m-5u, 7a-7k, 9a, 9b**	S-129
**Copies of NMR spectra - compounds 10a-10g, obtained from adduct 3h**	S-219
**Additional copies of NMR spectra - ^{19}F NMR and DEPT 135**	S-235
**Copies of ^{19}F NMR spectra - enones 2e-2k, 4a-4u, 6a-6k, 8a and 8b**	S-235
**Copies of DEPT 135 spectra - enones 2e-2k, 4a-4u, 6a-6k, 8a and 8b**	S-256
**Copies of ^{19}F NMR spectra - malonate adducts 3b-3l, 5a-5k, 5m-5u, 7a-7k, 9a, 9b**	S-278
**Copies of DEPT 135 spectra - malonate adducts 3b-3l, 5a-5k, 5m-5u, 7a-7k, 9a, 9b**	S-300
**Copies of ^{19}F NMR spectra - compounds 10a-10g, obtained from adduct 3h**	S-322
**Copies of DEPT 135 spectra - compounds 10a-10g, obtained from adduct 3h**	S-326

General Information:

All solvents were used as received, unless otherwise noted. Purification of products was performed using flash chromatography on silica gel (Merck Kieselgel 60 or SiliaFlash P60, 230-400 mesh) with mixtures of hexane fraction from petroleum/ethyl acetate, or hexane fraction from petroleum/dichloromethane as eluents. Thin-layer chromatography (TLC) was performed on silica gel plates (Merck Kieselgel 60 F_{254}). Visualization of the developed chromatogram was accomplished using UV light or *p*-anisaldehyde and cerium molybdate stains.

All reported NMR spectra were recorded in $CDCl_3$ using Agilent 400 MHz spectrometer. Chemical shifts of ^{1}H NMR and ^{13}C NMR are reported as δ values relative to TMS (δ=0.00) and $CDCl_3$ (δ=77.0), respectively. Chemical shifts of ^{19}F NMR are relative to hexafluorobenzene (C_6F_6, δ= -161.64) as an internal standard in $CDCl_3$.[21] The following abbreviations are used to indicate the multiplicity: s - singlet; d - doublet; t - triplet; q - quartet; m – multiplet; dm **-** doublet of multiplets**.**

Mass spectra were measured on a Q Exactive unit equipped with Orbitrap mass analyzer, using HR ESI technique. Optical rotation was recorded on a Perkin Elmer 241 polarimeter. The X-ray measurements were undertaken on a SuperNova Dual Source (Agilent Technologies) by dr Damian Trzybiński (Biological and Chemical Research Centre of the University of Warsaw).

Enantiomeric ratios of the products were determined using high performance liquid chromatography (HPLC) techniques. HPLC analyses were performed on a Merck chromatograph equipped with the diode-array detector (DAD) and Chiralpak® IA (25 cm x 0.46 cm, 5 μm), Chiralpak® IB (25 cm x 0.46 cm, 5 μm) or Chiralpak® IC (25 cm x 0.46 cm, 5 μm) columns eluted with *iso*-propanol (3-20%) in hexane (flow rate = 1.0 mL/min).

High pressure experiments were performed at room temperature using a direct, single-stage piston-cylinder apparatus with a hydraulic press from Unipress (Warsaw, Poland) equipped with a liquid piston vessel LV/30/16 and laboratory hydraulic press U101. Experiments were conducted in 0.2-0.25 mL, 0.5 mL, 0.7 mL, 0.9-1.0 mL, 1.2 mL, 1.5 mL, 2.0-2.2 mL and 5.0 mL Teflon ampoules inserted into the high-pressure vessel filled with hexane as a transmission medium. In one high-pressure experiment up to 24 parallel reactions in 0.2-0.25 mL ampoules are possible.

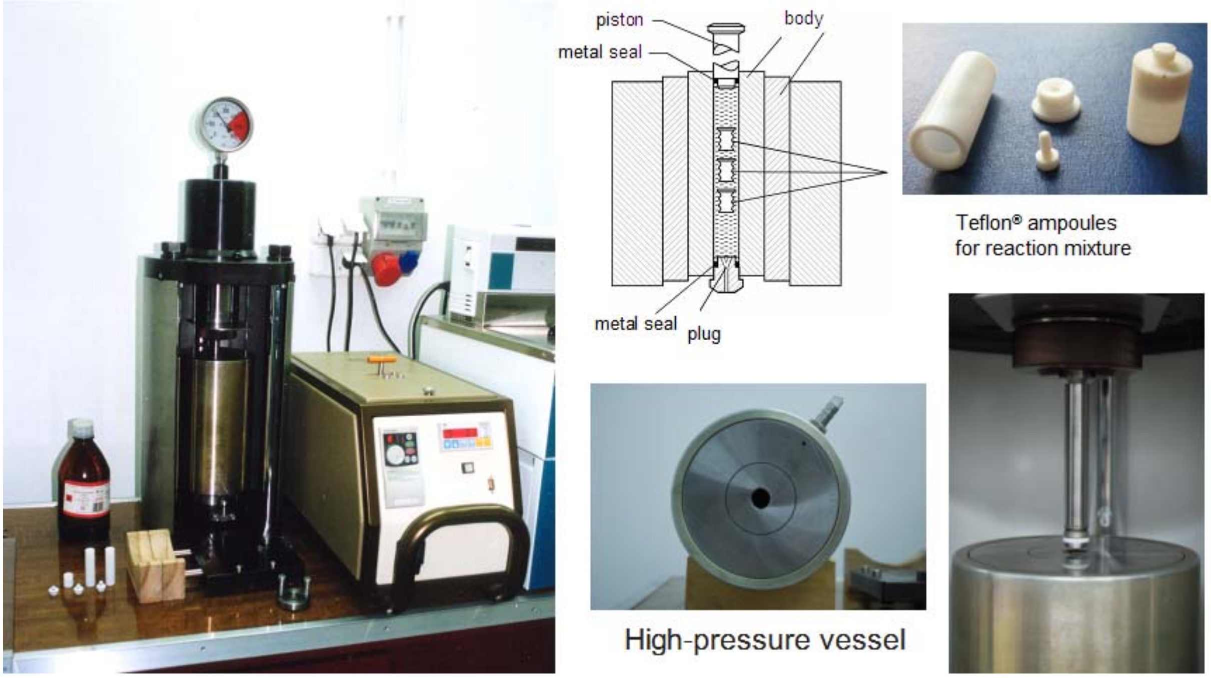

[21] C. P. Rosenau, B. J. Jelier, A. D. Gossert, A. Togni, *Angew. Chem. Int. Ed.* **2018**, *57*, 9528–9533.

Starting materials

All commercially available chemicals were used as received, unless otherwise noted.

Catalysts: Takemoto catalyst (**1a**) and quinidine were purchased from commercial suppliers (Strem, TCI) and used as received.
(1*R*,2*R*)-2-(Piperidin-1-yl)cyclohexanamine[22] based catalysts **1b**,[23] **1c**,[24] **1d**,[25] were prepared according to the literature methods. Cinchona alkaloid-thiourea **1e**[26] and corresponding squaramide **1f**[27] and 9-*epi*-amino-cinchona alkaloid[28] were prepared according to the literature methods.

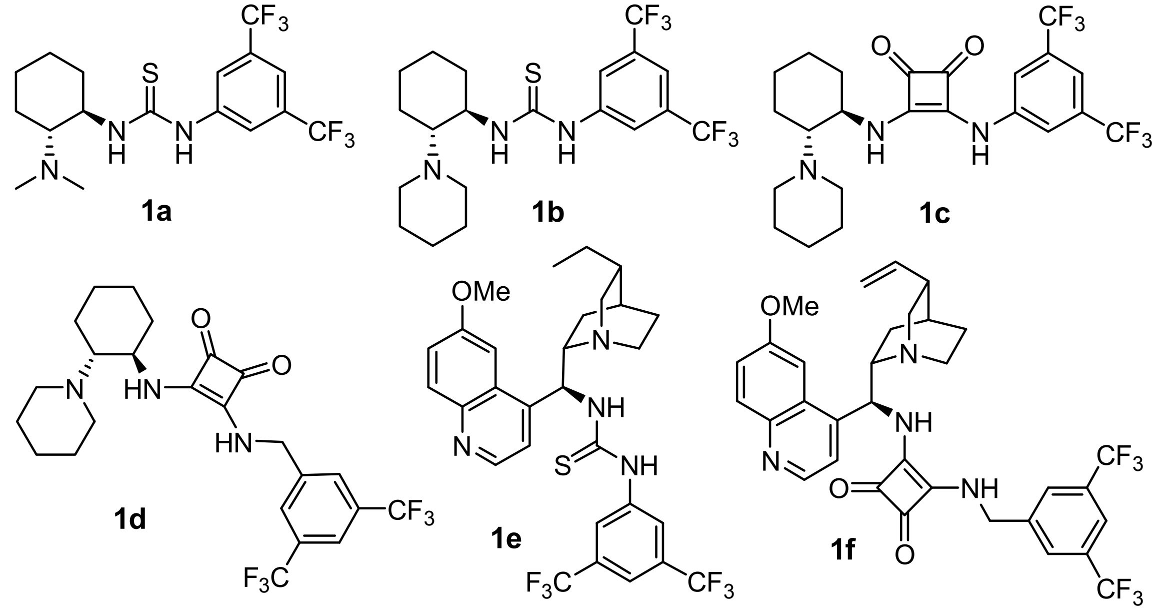

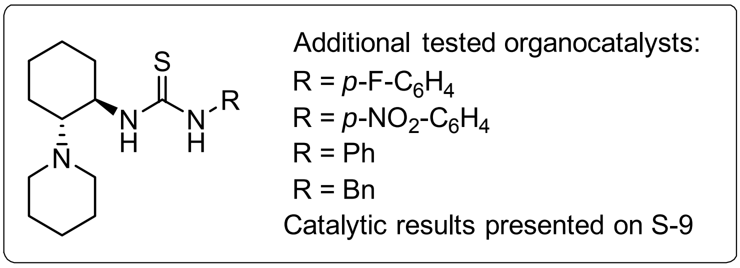

[22] Zhu, Y.; Malerich, J. P.; Rawal, V. H. *Angew. Chem. Int. Ed.* **2010**, *49*, 153–156.

[23] a) Jing, Z.; Bai, X.; Chen, W.; Zhang, G.; Zhu, B.; Jiang, Z. *Org. Lett.* **2016**, *18*, 260–263. b) Kurimoto, Y.; Nasu, T.; Fujii, Y.; Asano, K.; Matsubara, S. *Org. Lett.* **2019**, *21*, 2156–2160.

[24] Yang, W.; Du, D. *Adv. Synth. Catal.* **2011**, *353*, 1241–1246.

[25] Iriarte, I.; Olaizola, O.; Vera, S.; Gamboa, I.; Oiarbide, M.; Palomo, C. *Angew. Chem. Int. Ed.* **2017**, *56*, 8860–8864.

[26] a) B. Vakulya, S. Varga, A. Csámpai, T. Soós, *Org. Lett.*, **2005**, *7*, 1967–1969; b) B.-J. Li, L. Jiang, M. Liu, Y.-C. Chen, L.-S. Ding, Y. Wu, *Synlett*, **2005**, 603–606; c) S. H. McCooey, S. J. Connon, *Angew. Chem. Int. Ed.* **2005**, *44*, 6367-6370.

[27] W. Yang, D.-M. Du *Org. Lett.* **2010**, *12*, 5450–5453.

[28] a) H. Brunner, J. Buegler, B. Nuber, *Tetrahedron: Asymmetry* **1995**, *6*, 1699-1702. b) H. Brunner, P. Schmidt, *Eur. J. Org. Chem.* **2000**, 2119-2133. c) S. H. McCooey, S. J. Connon, *Org. Lett.* **2007**, *9*, 599-602.

β-Trifluoromethyl Enones:

Enones **2a-2h**, **2j**, **4a-4u**, **6a-6k**, (and **8a**, **8b**) were prepared from corresponding trifluoromethyl ketones (**8a**, **8b** from ClF_2CCOPh and F_3CF_2CCOPh respectively), and coresponding ylides (1.2-1.5 equiv) (Scheme S1). Ylides were obtained from α-bromomethyl ketones (2-bromoacetophenone, 2-(2-bromoacetyl)furan, 2-(2-bromoacetyl)tiophene, 2-(2-bromoacetyl)pyridine hydrobromide, 4-(2-bromoacetyl)pyridine hydrobromide, 3-(2-bromoacetyl)pyridine hydrobromide, 2-(2-bromoacetyl)-1-methylimidazole hydrobromide, 2-(2-bromoacetyl)thiazole hydrobromide, 2-(2-bromoacetyl)benzothiazole) and triphenylphosphine.

Enones were purified by column chromatography on silica gel using a mixture of hexane fraction from petroleum/DCM or hexane fraction from petroleum /AcOEt (gradient). For more polar enones (e.g. **2d**, **2e**, **2f**) eluent containing AcOEt was used.

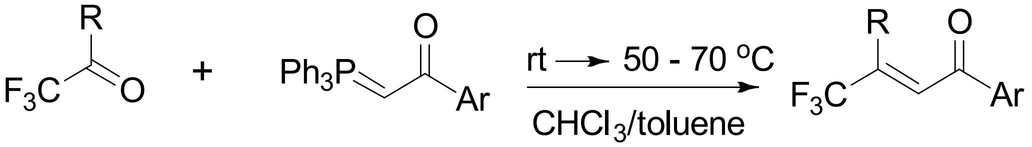

Scheme S1. General method of β-CF_3-enone synthesis

Enones **2i** and **2k** were prepared *via* metaloogranic addition to α,β-unsaturated Weinreb amide (Scheme S2).

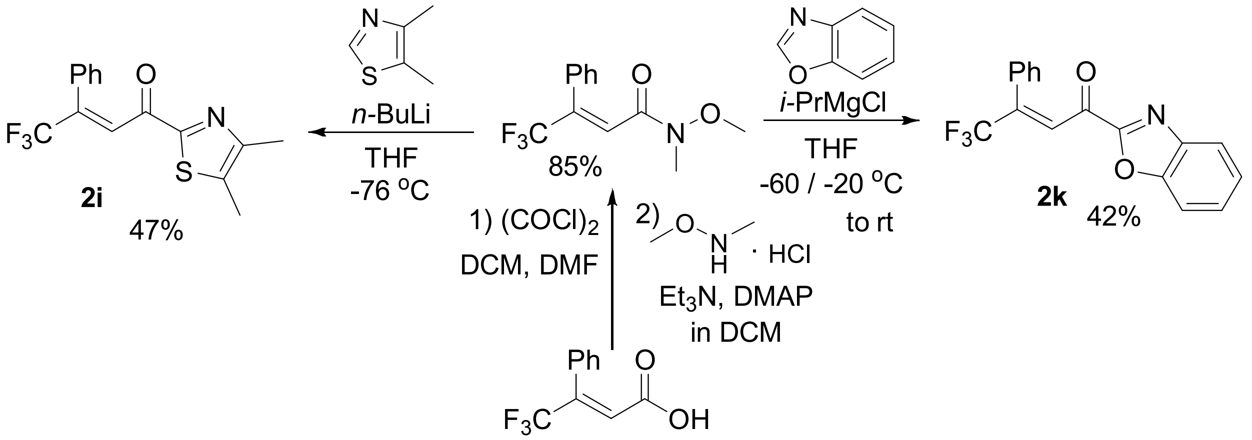

Scheme S2. Synthesis of enones **2i** and **2k**

Enones **2a**,[29] [7d] **2b**,[7d] **2c**,[7d] **2d**,[7d] and **2h** [7d] were previously synthesized in our laboratory, reported and characterized (NMR, MS).

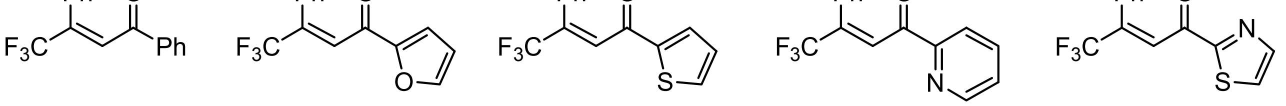

2a **2b** **2c** **2d** **2h**

[29] K. Matoba, H. Kawai, T. Furukawa, A. Kusuda, E. Tokunaga, S. Nakamura, M. Shiro, N. Shibata, *Angew. Chem. Int. Ed.* **2010**, *49*, 5762-5766.

[7d] P. Kwiatkowski, A. Cholewiak, A. Kasztelan, *Org. Lett.* **2014**, *16*, 5930–5933.

New enones:

Various β-trifluoromethyl-β-phenyl enones modified at the carbonyl group:

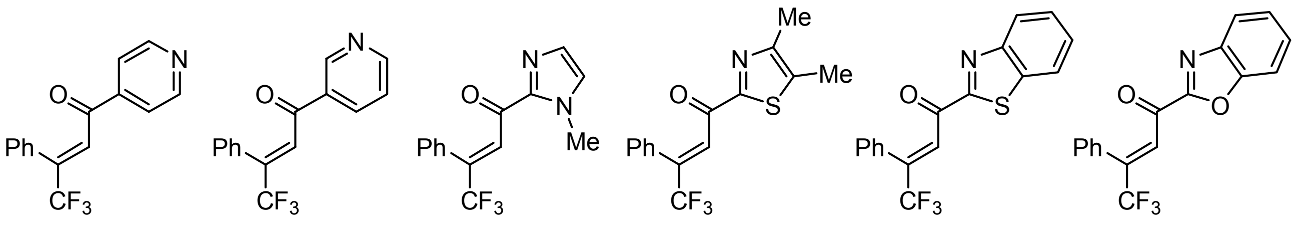

2e **2f** **2g** **2i** **2j** **2k**

β-Trifluoromethyl 2-thiazolyl enones **2h**, **4a-4u** were obtained starting from commercially available 2-acetylthiazole (Scheme S3). The initial steps of this synthesis are reported in the literature: bromination of 2-acetylthiazole[30] and preparation of α-(triphenylphosphoranylidene)-2-acetylthiazole.[31] We carried out these steps of synthesis with minor modifications. In the first step (bromination of 2-acetylthiazole) a smaller amount of HBr (0.2 equiv.) can be used instead of equimolar reported in the literature procedure.[30]

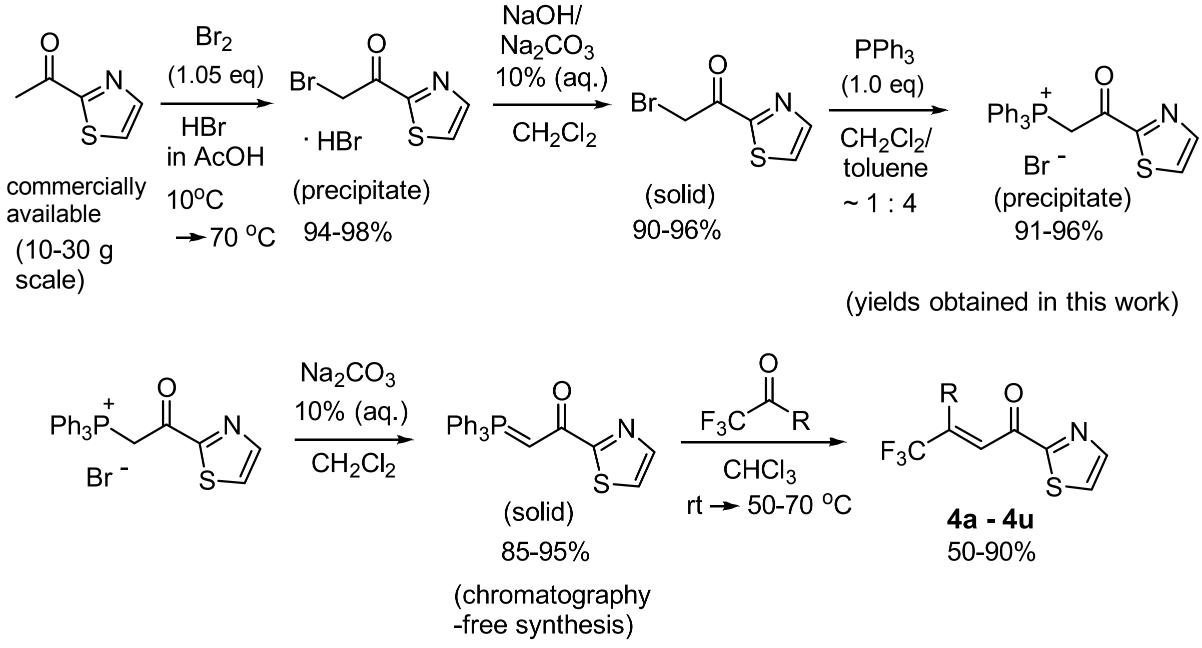

Scheme S3. Synthesis of thiazole-enones **4a-4u**.

[30] a) D. Dou, G. He, Y. Li, Z. Lai, L. Wei, K. R. Alliston, G. H. Lushington, D. M. Eichhorn, W. C. Groutas, *Bioorganic. Med. Chem.* **2010**, *18*, 1093–1102. b) J. M. Keith, L. A. Gomez, A. J. Barbier, S. J. Wilson, J. D. Boggs, B. Lord, C. Mazur, L. Aluisio, T. W. Lovenberg, N. I. Carruthers, *Bioorganic Med. Chem. Letters* **2007**, *17*, 4374–4377.

[31] a) A. Dondoni, P. Merino, J. Orduna, *Tetrahedron Letters* **1991**, *32*, 3247–3250. b) A. Dondoni, A. Marra, P. Merino, *J. Am. Chem. Soc.* **1994**, *116*, 3324–3336.

New thiazole-enones **4a**-**4u** and **8a**, **8b**:

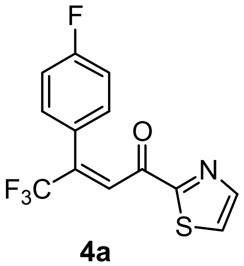

4a

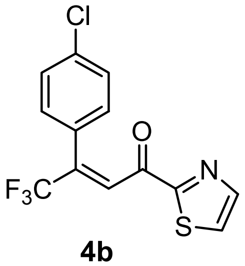

4b

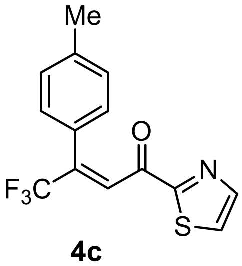

4c

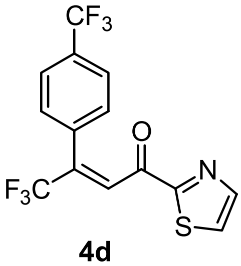

4d

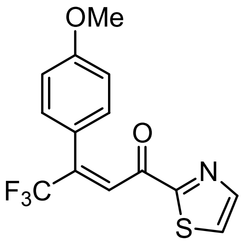

4e

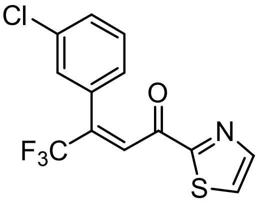

4f

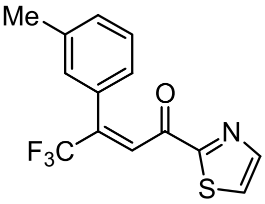

4g

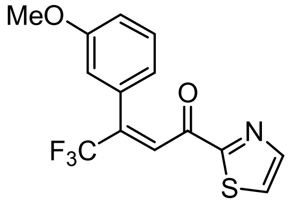

4h

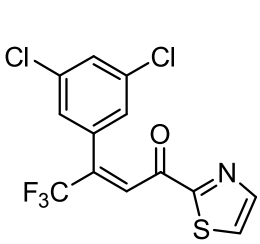

4i

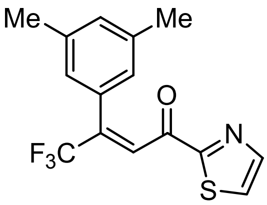

4j

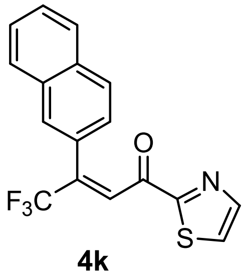

4k

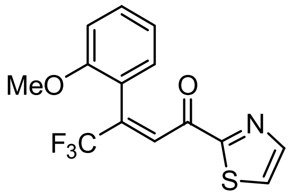

4l

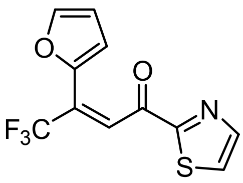

4m

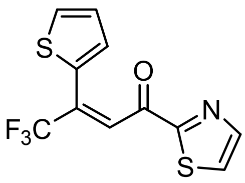

4n

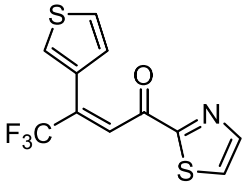

4o

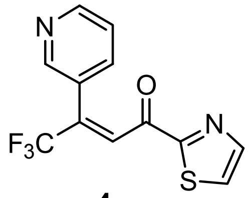

4p

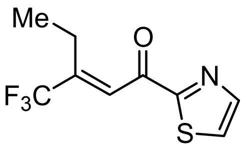

4q

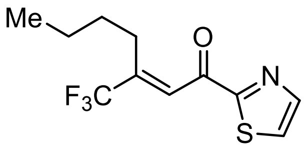

4r

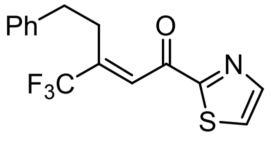

4s

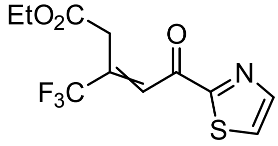

4t

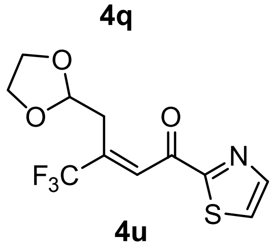

4u

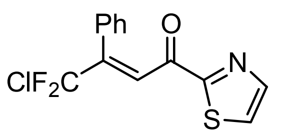

8a

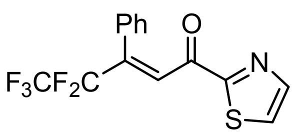

8b

β-Trifluoromethyl benzothiazolyl enones **2j**, **6a-6k** were obtained starting from benzothiazole (Scheme S4). 2-Acetylbenzothiazole[32] and 2-(2-bromoacetyl)benzothiazole[33] were obtained according to literature procedures.

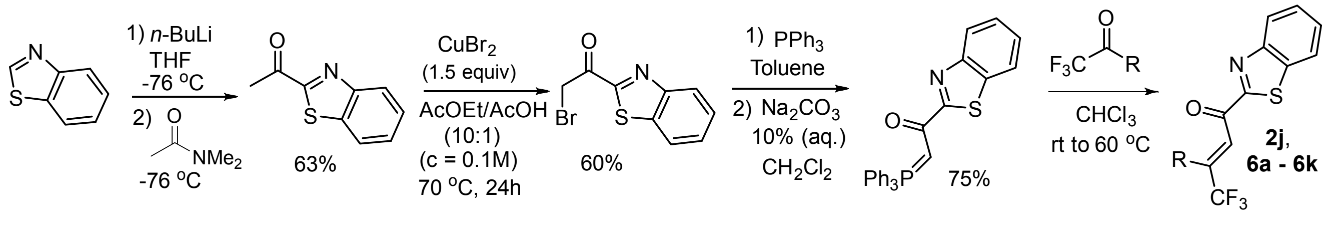

Scheme S4. Synthesis of benzothiazole-enones **2j**, **6a-6k**.

New benzothiazole-enones **6a-6k** (and **2j**):

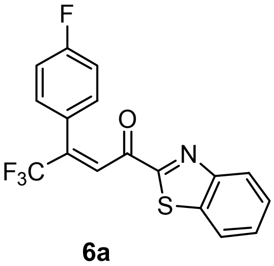

6a

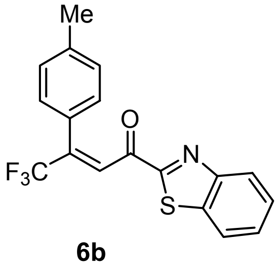

6b

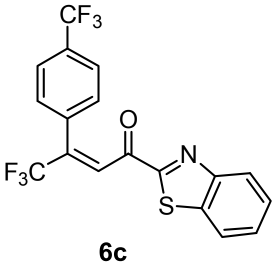

6c

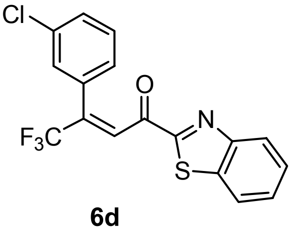

6d

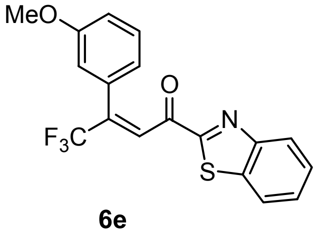

6e

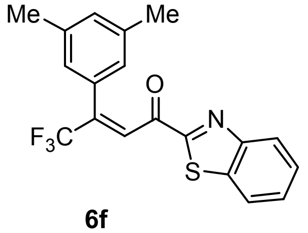

6f

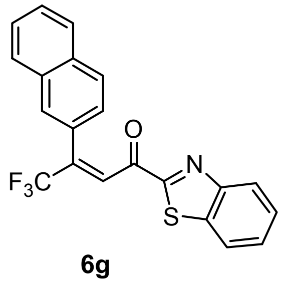

6g

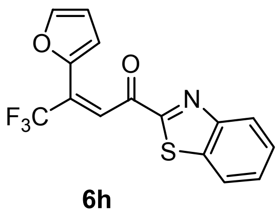

6h

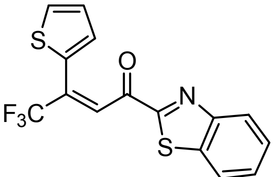

6i

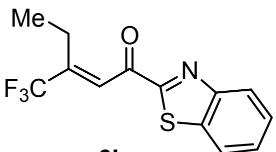

6j

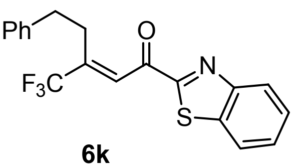

6k

[32] a) H. Chikashita, M. Ishibaba, K. Ori, K. Itoh, *Bull. Chem. Soc.* **1988**, *61*, 3637-3648; b) H. Yang, N. Huo, P. Yang, H. Pei, H. Lv, X. Zhang, *Org. Lett.* **2015**, *17*, 4144–4147.

[33] P. V. Podea, C. Paizs, M. I. Toşa, F. D. Irimie, *Tetrahedron: Asymmetry* **2008**, *19*, 1959–1964.

Enantioselective addition of diethyl malonate to enone 2h and 2j - additional information

Table S1. Effect of pressure on the Michael reaction of **2h** and **2j**

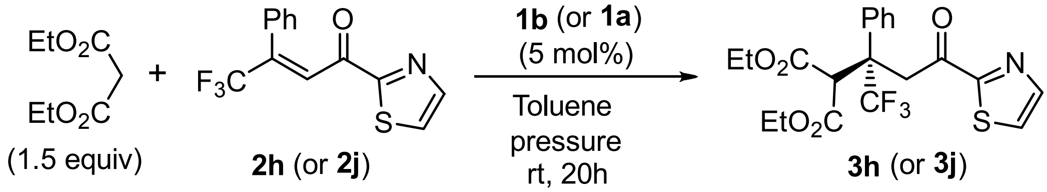

	**2h** (0.5 M) with **1b**		**2h** (1 M) with **1b**		**2j** (0.5 M) with **1a**	
Pressure	Yield (%) (NMR)	Ee (%)	Yield (%) (NMR)	Ee (%)	Yield (%) (NMR)	Ee (%)
1 bar	>0.1 %	-	>0.1 %	-	>0.1 %	-
3 kbar	2	-	5	-	ND	-
5 kbar	15	89	39	89	36	91
6 kbar	33	88	66	89	51	90
7 kbar	45	88	78	89	77	91
8 kbar	74	89	95	90	94	93
9 kbar	94	91	98	93	98	92
10 kbar	96	92	99	92	99	91

Figure S1: Effect of pressure on the Michael reaction of **2h** and **2j**

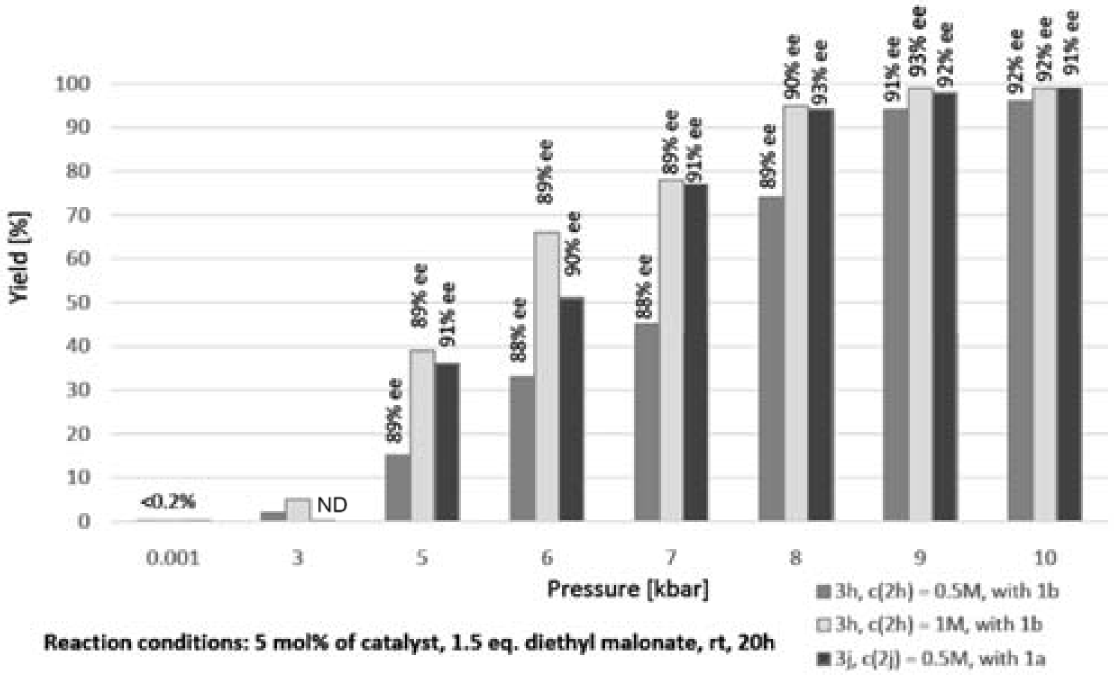

Table S1 and Fig. S1: Reaction of **2h** (c=0.5 and 1.0 M) in the presence of catalyst **1b** (5 mol%) as well as for benzothiazole enone **2j** (c=0.5 M) with Takemoto catalyst (**1a**). The results show that at higher pressure (8-10 kbar) the enantiomeric excess increases slightly. Higher concentration of enone **2h** (1.0 M vs 0.5 M) significantly improves the reaction efficiency in the range of 5-7 kbar. Benzothiazole enone **2j** is more reactive than thiazole one, however, its use has other limitations (e.g. lower solubility in toluene; more demanding synthesis). Finally to obtain high yield (>90%) application of pressure at least 8 kbar is required.

Catalyst Screening in the Model Reaction – Additional Examples:

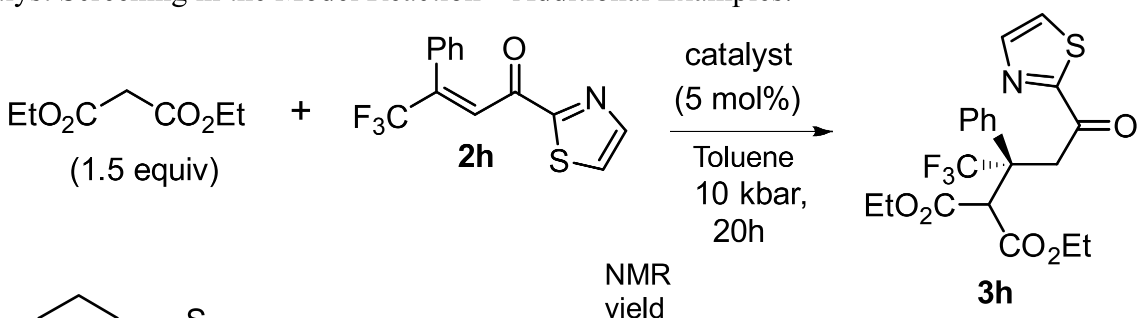

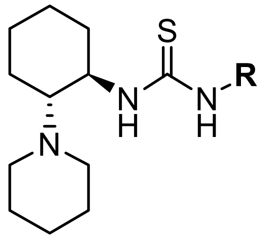

R	NMR yield	ee
R = *p*-F-C_6H_4	40%,	92% ee (*R*)
R = *p*-NO_2-C_6H_4	75%,	92% ee (*R*)
R = Ph	25%,	92% ee (*R*)
R = Bn	62%,	91% ee (*R*)
**1b**	95%,	92% ee (*R*)

1 bar, 7 days
yields < 0.2%

Changes in the structure of the **R** substituent in the catalyst, compared to **1b**, practically have no effect on the enantioselectivity, but significantly affect the yield (75-25% vs 95% for **1b**).

Isomerisation of enone 2h

In the Wittig raction, the (*E*)-**2h** isomer is formed with high selectivity (>90%), however we have isolated the enone (*Z*)-**2h** in a small amount (purity >90%, (*Z*) is slightly more polar).
We observed that (*Z*)-**2h** isomerizes in the presence of catalyst **1b** at atmospheric pressure to (*E*)-**2h**. At the equilibrium, the mixture contains (*E*)-**2h**: (*Z*)-**2h** in a ratio: 94:6.

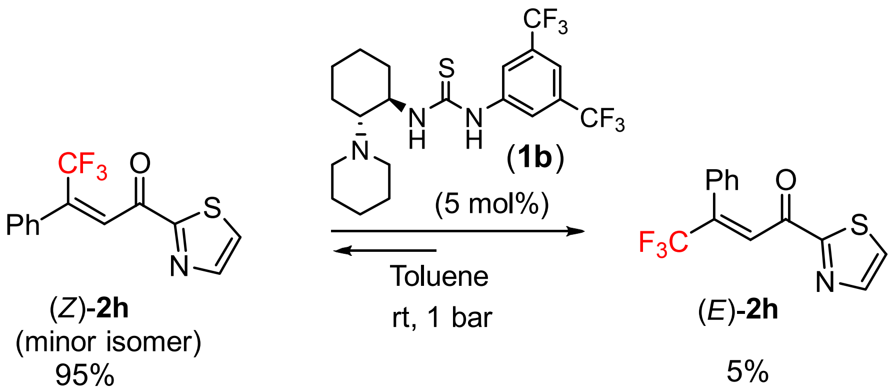

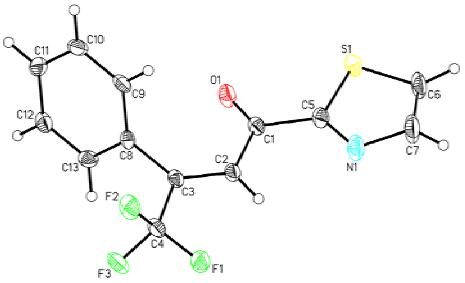

(*Z*)-2h	time	(*E*)-2h
83%	30 min.	17%
72%	1h	28%
56%	2h	44%
30%	6h	70%
6%	24h	94%

Enantioselective addition of diethyl malonate to enone (*Z*)-**2h**

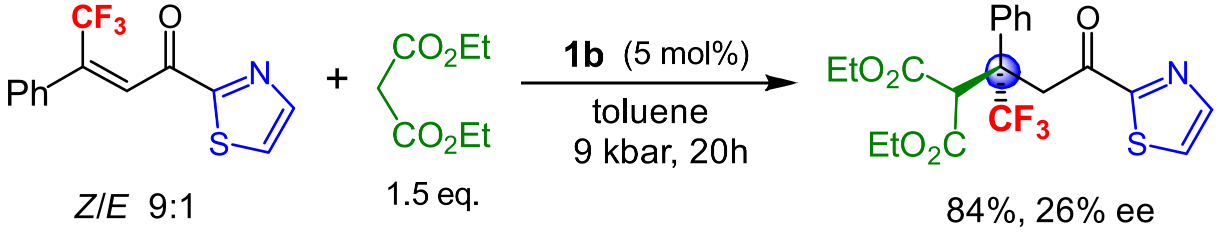

Proposed simplified stereochemical model

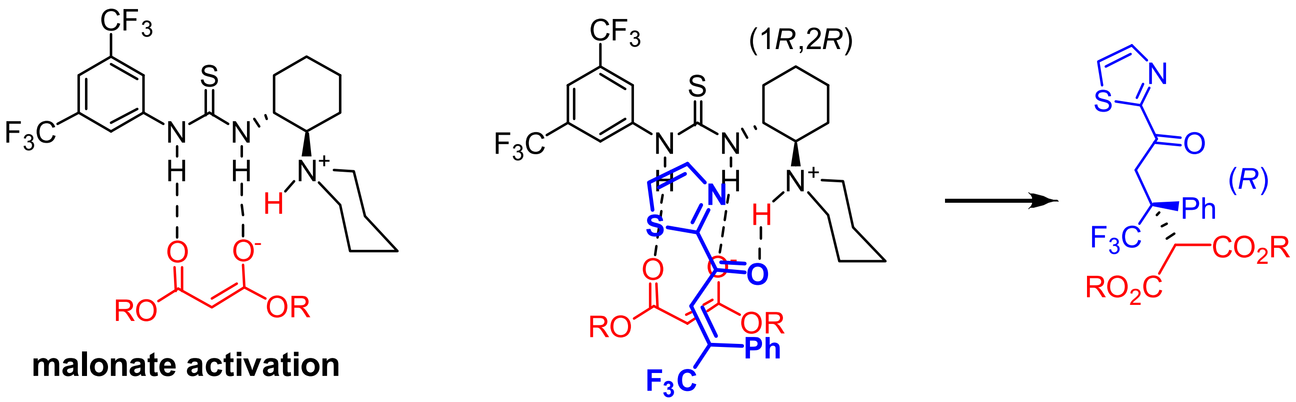

The simplified stereochemical model was proposed on the basis of computational studies for related / similar types of reactions:

- Addition of malonates, acetylacetone to nitrostyrenes catalyzed by Takemoto catalyst [34]
- Michael addition of nitromethane to chalcones in the presence of cinchona-thiourea catalysts.[35] [36]

General synthetic procedures:

A typical procedure for β-CF_3-enone synthesis:

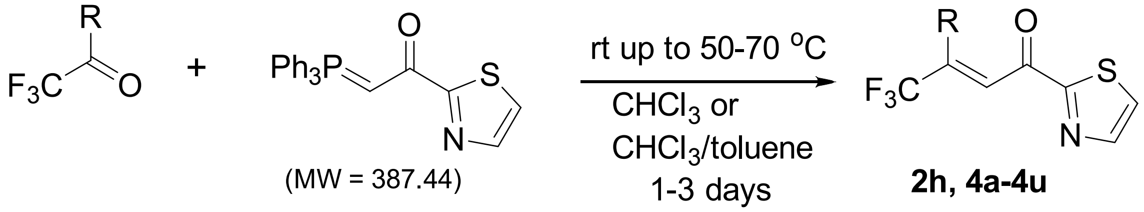

To α-(triphenylphosphoranylidene)-2-acetylthiazole[a)] (1.39 g – 1.74 g, 3.6–4.5 mmole, 1.2–1.5 equiv) in 10-20 ml screw cap tube was added chloroform (3–5 ml)[b)] and 3 mmol[c)] of corresponding trifluoromethyl ketone (alternatively in toluene; 1–1.5 ml). The tube with the reaction muxture was sealed and heated using a thermoblock to 50-70 °C, usually for 1–3 days. The progress of the reaction was monitored by silica gel thin layer chromatography (TLC), ^{19}F NMR or GC.[d)]

The reaction mixture was concentrated under reduced pressure, diluted with toluene and purified by column chromatography on silica gel usually using a mixture of hexane fraction from petroleum/DCM (or hexane fraction from petroleum/AcOEt). In most cases β-CF_3-enones were isolated with good yield (usually 60-90%) and *E*/*Z* ratio usually ≥ 97:3.

[a)] The reactions were carried out in an analogous manner with other stabilized ylides.

---

[34] A. Hamza, G. Schubert, T. Soós, I. Pápai, *J. Am. Chem. Soc.* **2006**, *128*, 13151–13160; b) J. A. Izzo, Y. Myshchuk, J. S. Hirschi, M. J. Vetticatt, *Org. Biomol. Chem.* **2019**, *17*, 3934–3939.

[35] a) M. N. Grayson, *J. Org. Chem.*, **2017**, *82*, 4396–4441. b) M. N. Grayson, K. N. Houk, *J. Am. Chem. Soc.* **2016**, *138*, 9041–9044. c) M. N. Grayson, K. N. Houk, *J. Am. Chem. Soc.*, **2016**, *138*, 1170–1173.

[36] For review articles see: a) M. Žabka, R. Šebesta, *Molecules* **2015**, *20*, 15500–15524. b) A. M. F. Phillips, M. H. G. Prechtl, A. J. L. Pombeiro, *Catalysts* **2021**, *11*, 569.

[b)] In some cases a solvent mixture of $CHCl_3$-toluene was used.

[c)] Depending on the enone example, Wittig reactions were carried out on a scale of 2-6 mmol. The exception is the model enone **2h** where the reaction scale reached 20 mmol.

[d)] In case of low conversion or incomplete consumption of trifluoromethyl ketone, ~0.5 equiv of ylide was added to the reaction muxture and heating continued until disappearance of trifluoromethyl ketone or to achieve a good conversion.

The model reaction optimization study:

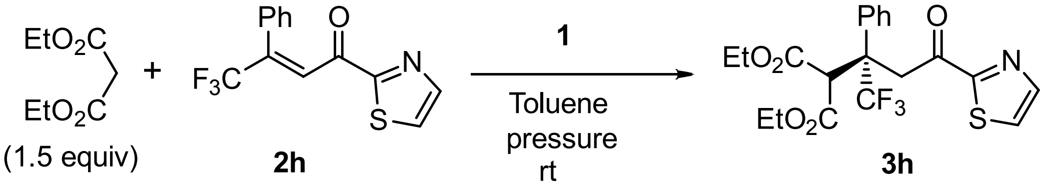

In some optimization studies stock solution of reagents was used, *e.g.*: enone **2h** (567 mg, 2.0 mmol, c=1.0 mol/L), diethyl malonate (480 mg, 3.0 mmol, 1.5 equiv), CF_3-internal standard (for ^{19}F NMR, α,α,α-trifluorotoluene or 1,4-bis(trifluoromethyl)benzene) and toluene in 2.0 mL volumetric flask.

General experimental procedure for the results presented in **Table 1** of the manuscript. A 1.5 mL vial was charged with catalyst **1** (0.01 mmol, 5 mol%), followed by 200 μL of stock solution of reagents (0.2 mmol of enone **2h**, 0.3 mmol of diethyl malonate in toluene), and diluted with 200 μL of toluene. Part of the reaction mixture was transferred to 0.20-0.25 mL Teflon ampoule and compressed in high-pressure apparatus (10 kbar for 20h). The yield and conversion of the reactions carried out under high pressure (~0.2 mL) and atmospheric pressure (remaining *ca.* 0.2 mL) were determined by ^{19}F NMR (with internal standard: α,α,α-trifluorotoluene or 1,4-bis(trifluoromethyl)benzene). During the analysis of the reaction mixtures using gas chromatography, we observed a partial decomposition of the product. The enantiomeric excess was determined by HPLC analysis on Chiralpak® IC chiral column after filtration through a short pad of silica gel with DCM/Et_2O and concentration.

General experimental procedure for the results presented in **Table 2** of the manuscript. (e.g. Entries 1 and 2). A 1.5 mL vial was charged with 9.1 mg of catalyst **1b** (0.02 mmol, 5 mol%), followed by 400 μL of stock solution of reagents (0.4 mmol of enone **2h**, 0.6 mmol of diethyl malonate in toluene). Part of the reaction mixture was transferred to 0.2-0.25 mL Teflon ampoule and compressed in high-pressure apparatus (9 kbar for 20h). Reaction mixtures were analyzed as described above (Table 1).

In one high-pressure experiment up to 24 parallel reactions in 0.2-0.25 mL ampoules were carried out.

General procedure for asymmetric high-pressure addition of diethyl malonate to β-CF_3-2-thiazolyl enones

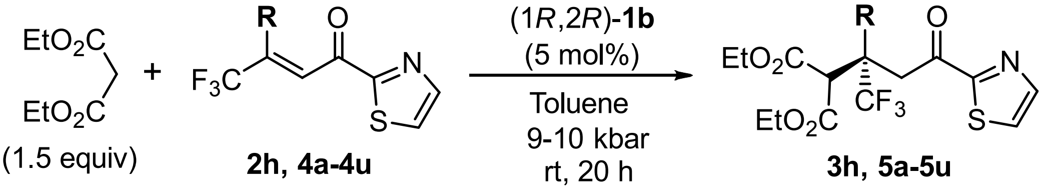

A 1.2 mL[a)] Teflon ampoule was charged with 5 mol% of catalyst **1b** (22.7–27.2 mg, 0.05–0.06 mmol), 1.0–1.2 mmol[a)] of enone (**2h**, **4a–4u**),[b)] 1.5 equiv of diethyl malonate (240–290 mg, 1.5–1.8 mmol), filled up with toluene[c)] (*ca.* 0.8–0.7 ml) and closed. Then the Teflon ampoule with homogenous reaction mixture was placed in a high-pressure chamber filled with the inert liquid (hexane or petroleum ether) and the pressure was slowly increased to 10 kbar at ambient temperature (20–25 °C) by hexane compressing. After the pressure was stabilized (10–9.5 kbar), the reaction mixture was kept under these conditions for 20 h. After decompression, the reaction mixture was flash-chromatographed on a silica gel using hexane fraction from petroleum/DCM (or hexane fraction from petroleum/AcOEt) as an eluent to afford adducts **3h**, **5a-5u**. The enantioselectivity was determined by HPLC analysis.

[a)] In some cases the scale was lowered to 0.9–0.6 mmol and experiments carried out in Teflon ampoules, 0.9 mL and 0.7 mL, respectively. Scale-up experiments for the model reaction (product **3h**) were carried out with 2 mmol, 3 mmol or 5 mmol of enone **2h**, in 2 mL, 3 mL and 5 mL Teflon vessels, respectively.

[b)] The reactions were carried out in an analogous manner with other enones (**2a-2g**, **2i-2k**, **6a-6k**, **8a**, **8b**). However, differences may concern the enone concentration, catalyst content and type (**1a** instead of **1b**), reaction time and scale.

[c)] Part of the toluene (~0.5 ml) can be added initially to the catalyst and enone to dissolve them. Then, after adding diethyl malonate, the Teflon vessel is filled up with the remaining volume of toluene and closed.

Preparation of racemates: Racemic samples of adducts **3**, **5**, **7** and **9a** were obtained from corresponding enones and diethyl malonate with catalytic amount (~5 mol%) of racemic **1b** in toluene under pressure 9-10 kbar. Alternatively, 1-(2-diethylamino-ethyl)-3-phenyl-thiourea or DBU can be used as a catalyst under high-pressure conditions, although they are less effective.

Analytical data of enones 2e-2k, 4a-4u, 6a-6k, 8a and 8b:

Enones **2a**, **2b**, **2c**, **2d** and **2h** were previously synthesized using the Wittig reaction in our laboratory, reported and characterized.[7d]

All enones were purified by column chromatography on silica gel using a mixture of hexane fraction from petroleum/DCM or hexane fraction from petroleum/AcOEt (gradient). For more polar enones (e.g. **2e**, **2f**) eluent containing AcOEt was used.

(*E*)-4,4,4-Trifluoro-3-phenyl-1-(pyridin-4-yl)but-2-en-1-one (2e):

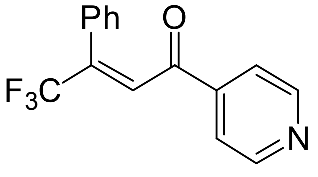

Yield: 67%, 559 mg (3 mmol scale Wittig reaction); column chromatography purification using a mixture of hexane fraction from petroleum/AcOEt; light gray solid; ^{1}H NMR (400 MHz, $CDCl_3$) δ 8.70 (dd, *J* = 4.4, 1.7 Hz, 2H), 7.53 (dd, *J* = 4.4, 1.7 Hz, 2H), 7.33 – 7.21 (m, 5H), 7.20 (q, *J* = 1.4 Hz, 1H); ^{13}C NMR (100 MHz, $CDCl_3$) δ 191.6 (C=O), 150.7 (2 x CH), 141.8 (C), 140.9 (q, *J* = 31.1 Hz, <u>C</u>-CF_3), 130.3 (C), 129.8 (CH), 129.1 (q, *J* = 5.2 Hz, CH), 128.9 (2 x CH), 128.5 (2 x CH), 122.5 (q, *J* = 275.0 Hz, CF_3), 121.2 (2 x CH); ^{19}F NMR (376 MHz, $CDCl_3$) δ -66.3 (s); HRMS (ESI) m/z: $[M + H]^+$ Calcd for $C_{15}H_{11}F_3NO$ 278.0787; Found 278.0788.
The enone contains ~0.2% of (Z)-isomer: ^{19}F NMR (376 MHz, $CDCl_3$) δ -59.0 (s).

(*E*)-4,4,4-Trifluoro-3-phenyl-1-(pyridin-3-yl)but-2-en-1-one (2f): [37]

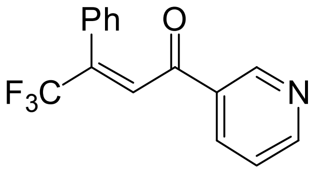

Yield: 84%, 696 mg (3 mmol scale Wittig reaction); column chromatography purification using a mixture of hexane fraction from petroleum/AcOEt; light yellow solid; ^{1}H NMR (400 MHz, $CDCl_3$) δ 8.98 (dm, *J* = 2.3 Hz, 1H), 8.68 (dm, *J* = 4.8 Hz, 1H), 8.03 (dm, *J* = 8.0 Hz, 1H), 7.32 – 7.24 (m, 6H), 7.21 (q, *J* = 1.4 Hz, 1H); ^{13}C NMR (100 MHz, $CDCl_3$) δ 191.1 (C=O), 153.8 (CH), 150.3 (CH), 140.1 (q, *J* = 31.1 Hz, <u>C</u>-CF_3), 135.7 (CH), 131.4 (C), 130.4 (C), 129.7 (CH), 129.6 (q, *J* = 5.1 Hz, CH), 128.9 (2 x CH), 128.5 (2 x CH), 123.4 (CH), 122.6 (q, *J* = 275.0 Hz, CF_3); ^{19}F NMR (376 MHz, $CDCl_3$) δ -66.3 (s); HRMS (ESI) m/z: $[M + H]^+$ Calcd for $C_{15}H_{11}F_3NO$ 278.0787; Found 278.0787.
The enone contains ~0.2% of (Z)-isomer: ^{19}F NMR (376 MHz, $CDCl_3$) δ -59.1 (s).

(*E*)-4,4,4-Trifluoro-1-(1-methyl-1H-imidazol-2-yl)-3-phenylbut-2-en-1-one (2g): [38]

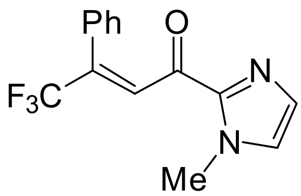

Yield: 65%, 735 mg (50°C, 3 days, 4 mmol scale Wittig reaction); light yellow solid; ^{1}H NMR (400 MHz, $CDCl_3$) δ 7.97 (q, *J* = 1.4 Hz, 1H), 7.43 – 7.36 (m, 3H), 7.33 – 7.29 (m, 2H), 7.21 (d, *J* = 0.8 Hz, 1H), 7.04 (bs, 1H), 3.88 (s, 3H); ^{13}C NMR (100 MHz, $CDCl_3$) δ 179.6 (C=O), 143.5 (C), 141.2 (q, *J* = 30.8 Hz, <u>C</u>-CF_3), 131.5 (C), 129.9 (CH), 129.0 (CH), 128.8 (2 x CH), 128.3 (q, *J* = 5.2 Hz, CH), 128.2 (2 x CH), 127.8 (CH), 122.7 (q, *J* = 275.0 Hz, CF_3), 36.1 (CH_3); ^{19}F NMR (376 MHz, $CDCl_3$) δ -67.1 (s); HRMS (ESI) m/z: $[M + H]^+$ Calcd for $C_{14}H_{12}F_3N_2O$ 281.0896; Found 281.0896.
The enone contains ~0.1% of (*Z*)-isomer: ^{19}F NMR (376 MHz, $CDCl_3$) δ -58.6 (s).

[7d] P. Kwiatkowski, A. Cholewiak, A. Kasztelan, *Org. Lett.* **2014**, *16*, 5930–5933.

[37] Sun, L.-W.; Hu, Y.-F.; Ji, W.-J.; Zhang, P.-Y.; Ma, M.; Shen, Z.-L.; Chu, X.-Q. *Org. Lett.* **2023**, *25*, 3745–3749.

[38] Zhang, H.; Luo, Y.; Zhu, C.; Dong, S.; Liu, X.; Feng, X. *Org. Lett.* **2020**, *22*, 5217–5222.

(*E*)-4,4,4-Trifluoro-3-phenyl-1-(thiazol-2-yl)but-2-en-1-one (2h): [7d]

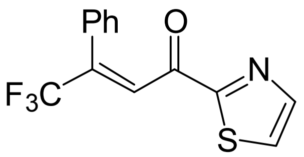

Yield: 85%, 4.80 g (20 mmol scale Wittig reaction); yellow solid; ^1H NMR (400 MHz, $CDCl_3$) δ 8.05 (d, *J* = 3.0 Hz, 1H), 7.92 (qd, *J* = 1.4, 0.7 Hz, 1H), 7.70 (dd, *J* = 3.0, 0.7 Hz, 1H), 7.45–7.37 (m, 3H), 7.35 – 7.31 (m, 2H); ^{13}C NMR (100 MHz, $CDCl_3$) δ 181.3 (C=O), 167.1 (C), 145.0 (CH), 143.3 (q, *J* = 31.0 Hz, C-CF_3), 130.9 (C), 129.4 (CH), 128.7 (2 × CH), 128.2 (2 × CH), 127.4 (CH), 126.1 (q, *J* = 5.3 Hz, CH), 122.5 (q, *J* = 275.1 Hz, CF_3); ^{19}F NMR (376 MHz, $CDCl_3$) δ -67.3 (s, 3F); HRMS (ESI) m/z: $[M + H]^+$ Calcd for $C_{13}H_9F_3NOS$ 284.0352; Found 284.0348.

The structure of (*E*)-**2h** (crystallized from AcOEt) was confirmed by X-ray crystallographic analysis.

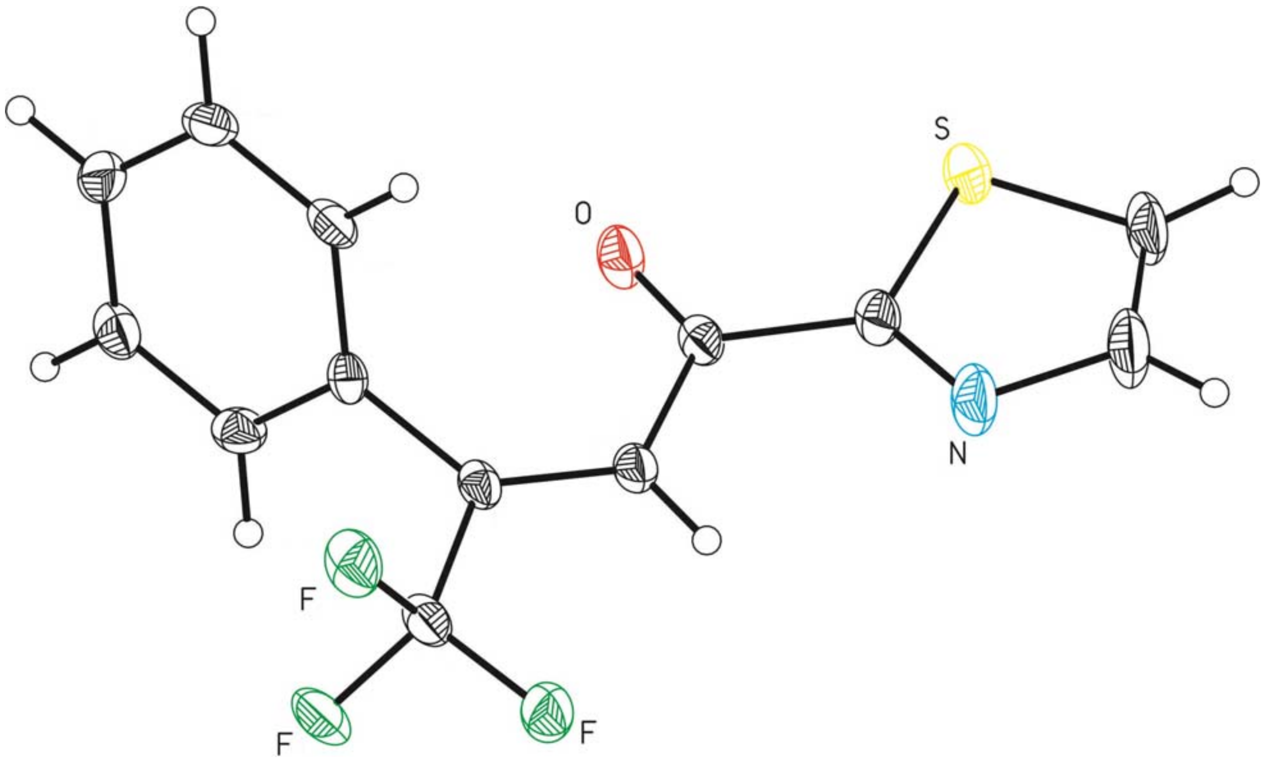

ORTEP of compound (*E*)-**2h** (CCDC Deposition Number 2033577)

(*Z*)-4,4,4-Trifluoro-3-phenyl-1-(thiazol-2-yl)but-2-en-1-one, minor isomer, ((*Z*)-2h):
(the content of isomers in the Wittig reaction mixture: ((*Z*)-**2h**/(*E*)-**2h** ~5:95)

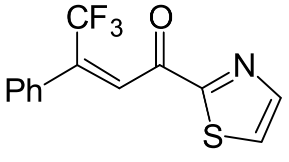

yellow oil, 140 mg; ^1H NMR (400 MHz, $CDCl_3$) δ 8.06 (d, *J* = 3.0 Hz, 1H), 7.77 (d, *J* = 3.0 Hz, 1H), 7.59 – 7.52 (m, 2H), 7.48 – 7.39 (m, 3H), 7.25 (s, 1H); ^{13}C NMR (100 MHz, $CDCl_3$) δ 183.0 (C=O), 166.4 (C), 145.2 (CH), 139.3 (q, *J* = 32.2 Hz, C-CF_3), 133.9 (q, *J* = 1.4 Hz, C), 131.9 (q, *J* = 3.1 Hz, CH), 129.7 (CH), 128.7 (2 x CH), 128.0 (q, *J* = 0.7 Hz, 2 x CH), 127.2 (CH), 122.2 (q, *J* = 276.4 Hz, CF_3); ^{19}F NMR (376 MHz, $CDCl_3$) δ -58.9 (s); HRMS (ESI) m/z: $[M + H]^+$ Calcd for $C_{13}H_9F_3NOS$ 284.0352; Found 284.0349.

(*E*)-1-(4,5-Dimethylthiazol-2-yl)-4,4,4-trifluoro-3-phenylbut-2-en-1-one (**2i**):

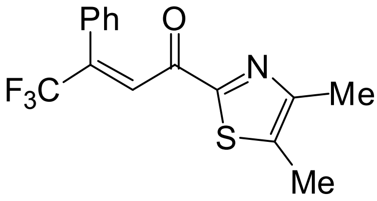

A solution of *n*-butyllithium in hexane (2.0 mL of 2.5 M, 5.0 mmol) was added dropwise into the solution of 4,5-dimethylthiazole (454 mg, 4.0 mmol) in tetrahydrofuran (20 mL) under inert atmosphere at -76 °C. After the reaction mixture had been stirred for 1 hour at a temperature below -60 °C, a solution of Weinreb amide - (*E*)-4,4,4-trifluoro-*N*-methoxy-*N*-methyl-3-phenylbut-2-enamide (1.04 g, 4.0 mmol, (see Scheme S2)) in tetrahydrofuran (4 mL) was added at -76 °C, and stirring was continued for 2 hours at this temperature. After the reaction mixture was warmed to room temperature, saturated aqueous ammonium chloride solution was added, then extracted with ethyl acetate (3 x 20 mL), dried over $MgSO_4$, filtered and concentrated under reduced pressure. The residue was purified by column chromatography (eluting with hexane fraction from petroleum/EtOAc, 9:1) on silica gel to give the enone **2i** as yellow oil (0.59 g) in 47% yield.

2i: yellow oil; ^{1}H NMR (400 MHz, $CDCl_3$) δ 7.87 (q, *J* = 1.2 Hz, 1H), 7.45 – 7.36 (m, 3H), 7.34 – 7.29 (m, 2H), 2.43 (s, 3H), 2.42 (s, 3H); ^{13}C NMR (100 MHz, $CDCl_3$) δ 181.0 (C=O), 162.0 (C), 152.1 (C), 142.5 (q, *J* = 30.7 Hz, C-CF_3), 137.7 (C), 131.3 (C), 129.2 (CH), 128.8 (2 x CH), 128.2 (2 x CH), 126.3 (q, *J* = 5.2 Hz, CH), 122.7 (q, *J* = 275.1 Hz, CF_3), 14.9 (CH_3), 12.2 (CH_3); ^{19}F NMR (376 MHz, $CDCl_3$) δ -67.2 (s); HRMS (ESI) m/z: $[M + H]^+$ Calcd for $C_{15}H_{13}F_3NOS$ 312.0664; Fund 312.0664.
The enone contains 1.3% of (*Z*)-isomer: ^{19}F NMR (376 MHz, $CDCl_3$) δ -58.8 (s).

(*E*)-4,4,4-trifluoro-*N*-methoxy-*N*-methyl-3-phenylbut-2-enamide (Weinreb amide used in the synthesis of enone **2i** and **2k**; see Scheme S2)

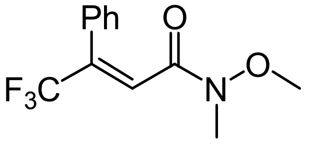

(*E*)-4,4,4-Trifluoro-3-phenylbut-2-enoic acid (β-trifluoromethyl-cinnamic acid) (4.36g, 20.2 mmol) was dissolved in DCM (25 mL) and the reaction mixture was cooled to 0 °C. Oxalyl chloride (2.5 mL, 29 mmol) was added followed by three drops of DMF. The reaction mixture was allowed to stir at room temperature for 15 h. Excess of oxalyl chloride and DCM was removed under reduced pressure and remaining crude β-trifluoromethyl cinnamoyl chloride was used for the next step without purification.

To a cooled and stirred suspension of *N*,O-dimethylhydroxylamine hydrochloride (2.6 g, 26.7 mmol) in anhydrous DCM (80 mL) at 0 °C was added dropwise triethylamine (8.2 mL, 58.5 mmol) and DMAP (243 mg, 2.0 mmol). After 0.5 hours of continuous agitation, a solution of crude β-trifluoromethyl cinnamoyl chloride (~20 mmol) in DCM (15 mL) was added and the reaction mixture was stirred overnight at room temperature. Then the reaction mixture was quenched with water (60 mL), organic phase was washed with brine and dried over Na_2SO_4. The solvent was removed in vacuo and the crude material was purified by column chromatography (95:5 → 75:25 hexane fraction from petroleum/EtOAc) on silica gel to afford correspnding Weinreb amide as a colourless oil (4.44 g) in 85% yield.
^{1}H NMR (400 MHz, $CDCl_3$) δ 7.44 – 7.31 (m, 5H), 6.97 (s, 1H), 3.67 (s, 3H), 3.11 (s, 3H); ^{13}C NMR (100 MHz, $CDCl_3$) δ 164.9 (C=O), 138.7 (q, *J* = 30.3 Hz, C-CF_3), 131.1 (C), 129.1 (CH), 128.7 (2 x CH), 128.1 (2 x CH), 125.2 (q, *J* = 5.2 Hz, =CH), 122.7 (q, *J* = 274.4 Hz, CF_3), 61.8 (CH_3), 32.0 (CH_3); ^{19}F NMR (376 MHz, $CDCl_3$) δ –66.5 (s); HRMS (ESI) m/z: $[M + H]^+$ Calcd for $C_{12}H_{13}F_3NO_2$ 260.0893; Found 260.0891.

(*E*)-1-(Benzo[d]thiazol-2-yl)-4,4,4-trifluoro-3-phenylbut-2-en-1-one (**2j**):

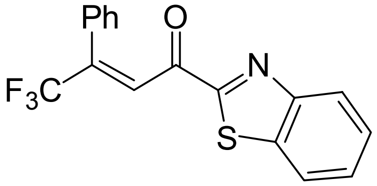

To 1.58 g α-(triphenylphosphoranylidene)-2-acetylbenzothiazole (3.6 mmole, 1.2 equiv) in 10 ml screw cap tube was added chloroform (5 ml) and 525 mg of trifluoroacetophenone (3.0 mmole, 1 equiv). The tube with the reaction muxture was sealed and heated to 50 °C for 3 days. The reaction mixture was concentrated under reduced pressure, diluted with toluene and purified by column chromatography on silica gel using a mixture of hexane fraction from petroleum/DCM (8:2) to give the enone **2j** as yellow solid (603 mg) in 60% yield.

2j: yellow solid; ^{1}H NMR (400 MHz, $CDCl_3$) δ 8.23 (ddd, *J* = 8.3, 1.3, 0.7 Hz, 1H), 8.06 (q, *J* = 1.3 Hz, 1H), 7.95 (ddd, *J* = 7.9, 1.4, 0.7 Hz, 1H), 7.63 – 7.53 (m, 2H), 7.46 – 7.39 (m, 3H), 7.37 – 7.32 (m, 2H); ^{13}C NMR (100 MHz, $CDCl_3$) δ 182.7 (C=O), 166.5 (C), 153.4 (C), 143.8 (q, *J* = 31.1 Hz, C-CF_3), 137.6 (C), 130.9 (C), 129.5 (CH), 128.7 (2 x CH), 128.3 (2 x CH), 128.2 (CH), 127.3 (CH), 125.8 (CH), 125.8 (q, *J* = 5.2 Hz, CH), 122.5 (q, *J* = 275.3 Hz, CF_3), 122.4 (CH); ^{19}F NMR (376 MHz, $CDCl_3$) δ -67.3; HRMS (ESI) m/z: $[M + H]^+$ Calcd for $C_{17}H_{11}F_3NOS$ 334.0508; Found 334.0507.

The enone contains ~0.5% of (*Z*)-isomer: ^{19}F NMR (376 MHz, $CDCl_3$) δ -58.8 (s).

(*E*)-1-(Benzo[d]oxazol-2-yl)-4,4,4-trifluoro-3-phenylbut-2-en-1-one (**2k**):

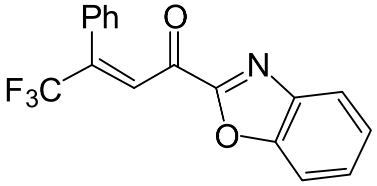

A solution of isopropylmagnesium chloride in THF (8.0 mL of 2.0 M, 16 mmol) was added dropwise into the solution of benzoxazole (1.72 g, 14.4 mmol) in dry tetrahydrofuran (30 mL) under inert atmosphere at -60 °C. Then the content of the flask was warmed to -20 °C, and after about 20 min cooled again to -60 °C and a solution of Weinreb amide - (*E*)-4,4,4-trifluoro-N-methoxy-N-methyl-3-phenylbut-2-enamide (2.40 g, 9.3 mmol; see Scheme S2) in tetrahydrofuran (4 mL) was addend. The cooling bath was removed and the reaction mixture was allowed to warm to room temperature and stirred overnight. Then the reaction mixture was quenched with saturated aqueous ammonium chloride solution, extracted with ethyl acetate (3 x 20 mL), dried over $MgSO_4$, filtered and concentrated under reduced pressure. The residue was purified by column chromatography (eluting with hexane fraction from petroleum/DCM, 7:3) on silica gel to give the enone **2k** as pale yellow solid (1.25 g) in 42% yield.

2k: pale yellow solid; ^{1}H NMR (400 MHz, $CDCl_3$) δ 7.92 (ddd, *J* = 8.0, 1.3, 0.7 Hz, 1H), 7.89 (q, *J* = 1.4 Hz, 1H), 7.62 (dm, *J* = 8.2 Hz, 1H), 7.57 – 7.52 (m, 1H), 7.50 – 7.45 (m, 1H), 7.43 – 7.37 (m, 3H), 7.36 – 7.32 (m, 2H); ^{13}C NMR (100 MHz, $CDCl_3$) δ 177.4 (C=O), 157.5 (C), 150.9 (C), 144.4 (q, *J* = 31.0 Hz, C-CF_3), 140.4 (C), 130.5 (C), 129.6 (CH), 129.1 (CH), 128.7 (2 x CH), 128.5 (2 x CH), 126.2 (q, *J* = 5.3 Hz, CH), 126.1 (CH), 122.5 (CH), 122.4 (q, *J* = 275.2 Hz, CF_3), 111.9 (CH); ^{19}F NMR (376 MHz, $CDCl_3$) δ -67.1 (s); HRMS (ESI) m/z: $[M + H]^+$ Calcd for $C_{17}H_{11}F_3NO_2$ 318.0736; Found 318.0736.

The enone contains <0.1% of (*Z*)-isomer: ^{19}F NMR (376 MHz, $CDCl_3$) δ -58.8 (s).

(*E*)-4,4,4-Trifluoro-3-(4-fluorophenyl)-1-(thiazol-2-yl)but-2-en-1-one (**4a**):

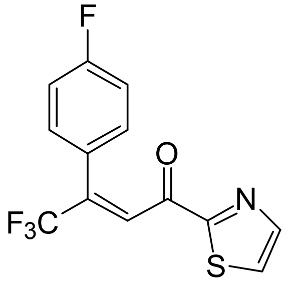

Yield: 90%, 1.62 g (6 mmol scale Wittig reaction); yellow oil (solidified upon long standing); ^{1}H NMR (400 MHz, $CDCl_3$) δ 8.05 (d, *J* = 3.0 Hz, 1H), 7.93 (q, *J* = 1.4 Hz, 1H), 7.71 (d, *J* = 3.0 Hz, 1H), 7.35 – 7.29 (m, 2H), 7.12 – 7.05 (m, 2H); ^{13}C NMR (100 MHz, $CDCl_3$) δ 181.2 (C=O), 167.0 (C), 163.3 (d, *J* = 249.6 Hz, C-F), 145.1 (CH), 142.2 (q, *J* = 31.2 Hz, C-CF_3), 130.9 (d, *J* = 8.5 Hz, 2× CH), 127.6 (CH), 126.7 (d, *J* = 3.7 Hz, C), 126.5 (q, *J* = 5.2 Hz, CH), 122.4 (q, *J* = 275.0 Hz, CF_3), 115.5 (d, *J* = 21.9 Hz, CH × 2); ^{19}F NMR (376 MHz, $CDCl_3$) δ -67.4 (s), -111.0 – -111.1 (m); HRMS (ESI) m/z: $[M + H]^+$ Calcd for $C_{13}H_8F_4NOS$ 302.0257; Found 302.0258.

(*E*)-3-(4-Chlorophenyl)-4,4,4-trifluoro-1-(thiazol-2-yl)but-2-en-1-one (**4b**):

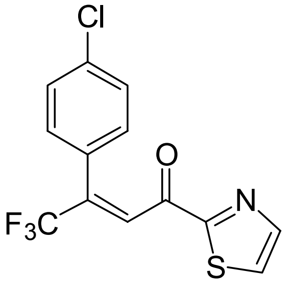

Yield: 78%, 1.48 g (6 mmol scale Wittig reaction); yellow oil; ^{1}H NMR (400 MHz, $CDCl_3$) δ 8.05 (d, *J* = 3.0 Hz, 1H), 7.95 (q, *J* = 1.4 Hz, 1H), 7.72 (d, *J* = 3.0 Hz, 1H), 7.42 – 7.35 (m, 2H), 7.27 (d, *J* = 8.3 Hz, 2H); ^{13}C NMR (100 MHz, $CDCl_3$) δ 181.1 (C=O), 167.0 (C), 145.2 (CH), 142.1 (q, *J* = 31.2 Hz, C-CF_3), 135.7 (C), 130.2 (2 x CH), 129.3 (C), 128.6 (2 x CH), 127.7 (CH), 126.6 (q, *J* = 5.2 Hz, CH), 122.3 (q, *J* = 275.2 Hz, CF_3); ^{19}F NMR (376 MHz, $CDCl_3$) δ -67.3 (s); HRMS (ESI) m/z: $[M + H]^+$ Calcd for $C_{13}H_8ClF_3NOS$ 317.9962; Found 317.9963.

(*E*)-4,4,4-Trifluoro-1-(thiazol-2-yl)-3-(*p*-tolyl)but-2-en-1-one (**4c**):

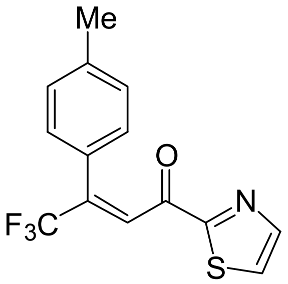

Yield: 81%, 1.20 g (5 mmol scale Wittig reaction); yellow oil (solidified upon long standing); ^{1}H NMR (400 MHz, $CDCl_3$) δ 8.04 (d, *J* = 3.0 Hz, 1H), 7.90 (m, 1H), 7.70 (d, *J* = 3.0 Hz, 1H), 7.25 – 7.17 (m, 4H), 2.38 (s, 3H); ^{13}C NMR (100 MHz, $CDCl_3$) δ 181.4 (C=O), 167.3 (C), 145.0 (CH), 143.5 (q, *J* = 30.8 Hz, C-CF_3), 139.5 (C), 129.0 (2 x CH), 128.6 (2 x CH), 127.9 (C), 127.4 (CH), 125.8 (q, *J* = 5.2 Hz, CH), 122.6 (q, *J* = 275.3 Hz, CF_3), 21.3 (CH_3); ^{19}F NMR (376 MHz, $CDCl_3$) δ -67.3 (s); HRMS (ESI) m/z: $[M + H]^+$ Calcd for $C_{14}H_{11}F_3NOS$ 298.0508; Found 298.0509.

(*E*)-4,4,4-Trifluoro-1-(thiazol-2-yl)-3-(4-(trifluoromethyl)phenyl)but-2-en-1-one (**4d**):

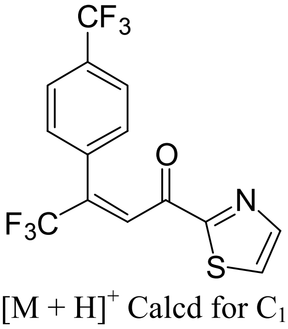

Yield: 74%, 1.55 g (6 mmol scale Wittig reaction); yellow oil; ^{1}H NMR (400 MHz, $CDCl_3$) δ 8.07 (d, *J* = 3.0 Hz, 1H), 8.03 (m, 1H), 7.75 (dd, *J* = 3.0, 0.9 Hz, 1H), 7.70 – 7.64 (m, 2H), 7.45 (d, *J* = 8.1 Hz, 2H); ^{13}C NMR (100 MHz, $CDCl_3$) δ 180.9 (C=O), 166.8 (C), 145.2 (CH), 142.0 (q, *J* = 31.1 Hz, C-CF_3), 134.7 (C), 131.4 (q, *J* = 32.8 Hz, C-CF_3), 129.3 (2 x CH), 127.9 (CH), 126.9 (q, *J* = 5.2 Hz, =CH-CO), 125.3 (q, *J* = 3.8 Hz, 2 x CH), 123.8 (q, *J* = 272.4 Hz, CF_3). 122.2 (q, *J* = 275.3 Hz, CF_3); ^{19}F NMR (376 MHz, $CDCl_3$) δ -62.8 (s, 3F), -67.3 (s, 3F); HRMS (ESI) m/z: $[M + H]^+$ Calcd for $C_{14}H_8F_6NOS$ 352.0225; Found 352.0226.

(*E*)-4,4,4-Trifluoro-3-(4-methoxyphenyl)-1-(thiazol-2-yl)but-2-en-1-one (**4e**):

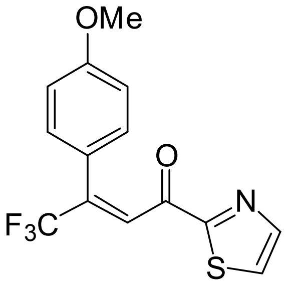

Yield: 65%, 1.22 g (6 mmol scale Wittig reaction); yellow solid; ^{1}H NMR (400 MHz, $CDCl_3$) δ 8.04 (d, *J* = 3.0 Hz, 1H), 7.86 (q, *J* = 1.3 Hz, 1H), 7.70 (d, *J* = 3.0 Hz, 1H), 7.31 – 7.27 (m, 2H), 6.93 – 6.89 (m, 2H), 3.82 (s, 3H); ^{13}C NMR (100 MHz, $CDCl_3$) δ 181.6 (C=O), 167.4 (C), 160.6 (C), 145.1 (CH), 143.1 (q, *J* = 30.7 Hz, $\underline{C}$-CF_3), 130.5 (2 x CH), 127.4 (CH), 125.5 (q, *J* = 5.2 Hz, CH), 122.9 (C), 122.7 (q, *J* = 275.4 Hz, CF_3), 113.7 (2 x CH_3), 55.2 (CH_3); ^{19}F NMR (376 MHz, $CDCl_3$) δ -67.0 (s); HRMS (ESI) m/z: $[M + H]^+$ Calcd for $C_{14}H_{11}F_3NO_2S$ 314.0457; Found 314.0458.

(*E*)-3-(3-Chlorophenyl)-4,4,4-trifluoro-1-(thiazol-2-yl)but-2-en-1-one (**4f**):

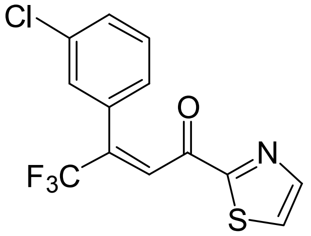

Yield: 75%, 1.43 g (6 mmol scale Wittig reaction); yellow oil; ^{1}H NMR (400 MHz, $CDCl_3$) δ 8.06 (d, *J* = 3.0 Hz, 1H), 7.96 (q, *J* = 1.3 Hz, 1H), 7.73 (dd, *J* = 3.0, 0.6 Hz, 1H), 7.41 (ddd, *J* = 8.1, 2.0, 1.2 Hz, 1H), 7.35 (d, *J* = 7.7 Hz, 1H), 7.32 (bs, 1H), 7.21 (d, *J* = 7.6 Hz, 1H); ^{13}C NMR (100 MHz, $CDCl_3$) δ 180.9 (C=O), 166.9 (C), 145.2 (CH), 141.8 (q, *J* = 30.7 Hz, C-CF_3), 134.3 (C), 132.6 (C), 129.6 (2 x CH), 128.7 (CH), 127.7 (CH), 127.1 (CH), 126.8 (q, *J* = 5.1 Hz, CH), 122.2 (q, *J* = 275.2 Hz, CF_3); ^{19}F NMR (376 MHz, $CDCl_3$) δ -67.3 (s); HRMS (ESI) m/z: $[M + H]^+$ Calcd for $C_{13}H_8ClF_3NOS$ 317.9962; Found 317.9963.

(*E*)-4,4,4-Trifluoro-1-(thiazol-2-yl)-3-(*m*-tolyl)but-2-en-1-one (**4g**):

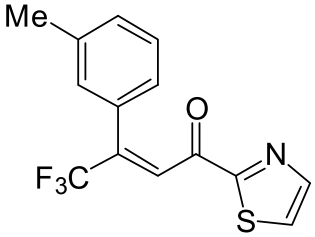

Yield: 80%, 1.55 g (6.5 mmol scale Wittig reaction); yellow oil (solidified upon standing); ^{1}H NMR (400 MHz, $CDCl_3$) δ 8.04 (d, *J* = 3.0 Hz, 1H), 7.89 (q, *J* = 1.3 Hz, 1H), 7.70 (d, *J* = 3.0 Hz, 1H), 7.31 – 7.26 (m, 1H), 7.23 (dm, *J* = 7.7 Hz, 1H), 7.15 – 7.09 (m, 2H), 2.36 (s, 3H); ^{13}C NMR (100 MHz, $CDCl_3$) δ 181.4 (C=O), 167.3 (C), 145.1 (CH), 143.5 (q, *J* = 30.8 Hz, $\underline{C}$-CF_3), 138.0 (C), 130.9 (C), 130.2 (CH), 129.1 (CH), 128.2 (CH), 127.4 (CH), 126.0 (q, *J* = 5.2 Hz, CH), 125.9 (CH), 122.6 (q, *J* = 275.1 Hz, CF_3), 21.4 (CH_3); ^{19}F NMR (376 MHz, $CDCl_3$) δ -67.3 (s); HRMS (ESI) m/z: $[M + H]^+$ Calcd for $C_{14}H_{11}F_3NOS$ 298.0508; Found 298.0510.

(*E*)-4,4,4-Trifluoro-3-(3-methoxyphenyl)-1-(thiazol-2-yl)but-2-en-1-one (**4h**):

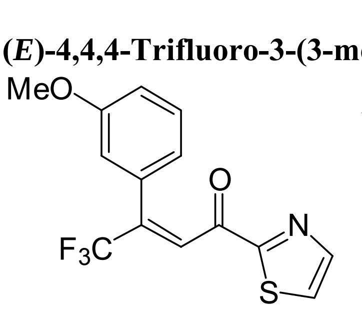

Yield: 76%, 1.43 g (6 mmol scale Wittig reaction); yellow solid; ^{1}H NMR (400 MHz, $CDCl_3$) δ 8.05 (d, *J* = 3.0 Hz, 1H), 7.88 (q, *J* = 1.4 Hz, 1H), 7.71 (d, *J* = 3.0 Hz, 1H), 7.30 (ddd, *J* = 8.1, 7.6, 0.4 Hz, 1H), 6.95 (ddd, *J* = 8.4, 2.6, 0.9 Hz, 1H), 6.89 (dm, *J* = 7.6 Hz, 1H), 6.85 (bs, 1H), 3.79 (s, 3H); ^{13}C NMR (100 MHz, $CDCl_3$) δ 181.4 (C=O), 167.0 (C), 159.2 (C), 145.0 (CH), 142.7 (q, *J* = 30.9 Hz, $\underline{C}$-CF_3), 132.1 (C), 129.4 (CH), 127.4 (CH), 126.4 (q, *J* = 5.2 Hz, CH), 122.4 (q, *J* = 275.1 Hz, CF_3), 121.0 (CH), 114.8 (CH), 114.4 (CH), 55.1 (CH_3); ^{19}F NMR (376 MHz, $CDCl_3$) δ -67.2 (s); HRMS (ESI) m/z: $[M + H]^+$ Calcd for $C_{14}H_{11}F_3NO_2S$ 314.0457; Found 314.0458.

(*E*)-3-(3,5-Dichlorophenyl)-4,4,4-trifluoro-1-(thiazol-2-yl)but-2-en-1-one (**4i**):

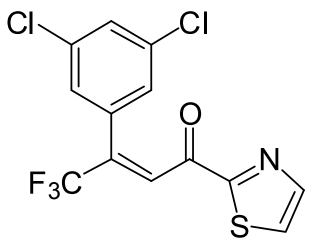

Yield: 76%, 805 mg (3 mmol scale Wittig reaction); yellow solid; ^{1}H NMR (400 MHz, $CDCl_3$) δ 8.08 (d, *J* = 3.0 Hz, 1H), 7.99 (q, *J* = 1.3 Hz, 1H), 7.76 (d, *J* = 3.0 Hz, 1H), 7.43 (t, *J* = 1.9 Hz, 1H), 7.22 – 7.19 (m, 2H); ^{13}C NMR (100 MHz, $CDCl_3$) δ 180.5 (C=O), 166.7 (C), 145.3 (CH), 140.4 (q, *J* = 31.7 Hz, $\underline{C}$-CF_3), 135.0 (2 × C), 133.6 (C), 129.6 (CH), 128.0 (CH), 127.4 (q, *J* = 5.1 Hz, CH), 127.3 (2 × CH), 122.0 (q, *J* = 275.1 Hz, CF_3); ^{19}F NMR (376 MHz, $CDCl_3$) δ -67.3 (s); HRMS (ESI) m/z: $[M + H]^+$ Calcd for $C_{13}H_7Cl_2F_3NOS$ 351.9572; Found 351.9573.

(*E*)-3-(3,5-Dimethylphenyl)-4,4,4-trifluoro-1-(thiazol-2-yl)but-2-en-1-one (**4j**):

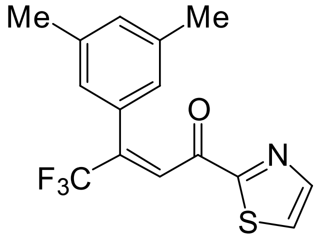

Yield: 65%, 930 mg (4.6 mmol scale Wittig reaction); pale yellow solid; ^{1}H NMR (400 MHz, $CDCl_3$) δ 8.04 (d, *J* = 3.0 Hz, 1H), 7.87 (q, *J* = 1.2 Hz, 1H), 7.69 (d, *J* = 3.0 Hz, 1H), 7.04 (s, 1H), 6.92 (s, 2H), 2.31 (s, 6H); ^{13}C NMR (100 MHz, $CDCl_3$) δ 181.4 (C=O), 167.3 (C), 145.0 (CH), 143.7 (q, *J* = 30.5 Hz, $\underline{C}$-CF_3), 137.8 (2 × C), 131.2 (CH), 130.8 (C), 127.3 (CH), 126.3 (2 × CH), 125.8 (q, *J* = 5.2 Hz, CH), 122.5 (q, *J* = 275.1 Hz, CF_3), 21.2 (2 × CH_3); ^{19}F NMR (376 MHz, $CDCl_3$) δ -67.2 (s); HRMS (ESI) m/z: $[M + H]^+$ Calcd for $C_{15}H_{13}F_3NOS$ 312.0664; Found 312.0665.

(*E*)-4,4,4-Trifluoro-3-(naphthalen-2-yl)-1-(thiazol-2-yl)but-2-en-1-one (**4k**):

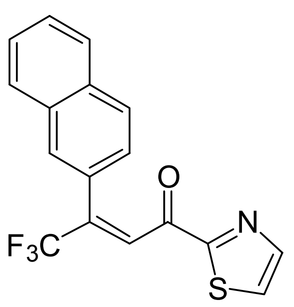

Yield: 90%, 1.53 g (5.1 mmol scale Wittig reaction); yellow solid; ^{1}H NMR (400 MHz, $CDCl_3$) δ 8.04 (d, *J* = 3.0 Hz, 1H), 8.00 (q, *J* = 1.3 Hz, 1H), 7.89 – 7.80 (m, 4H), 7.67 (d, *J* = 3.0 Hz, 1H), 7.55 – 7.47 (m, 2H), 7.40 (dd, *J* = 8.5, 1.5 Hz, 1H); ^{13}C NMR (100 MHz, $CDCl_3$) δ 181.3 (C=O), 167.1 (C), 145.0 (CH), 143.3 (q, *J* = 31.0 Hz, $\underline{C}$-CF_3), 133.4 (C), 132.7 (C), 128.4 (CH), 128.3 (CH), 127.9 (CH), 127.7 (CH), 127.5 (CH), 127.0 (CH), 126.5 (CH), 126.4 (q, *J* = 5.2 Hz, CH), 126.1 (CH), 122.7 (q, *J* = 275.4 Hz, CF_3); ^{19}F NMR (376 MHz, $CDCl_3$) δ -66.9 (s); HRMS (ESI) m/z: $[M + H]^+$ Calcd for $C_{17}H_{11}F_3NOS$ 334.0508; Found 334.0509.

(*E*)-4,4,4-Trifluoro-3-(2-methoxyphenyl)-1-(thiazol-2-yl)but-2-en-1-one (**4l**):

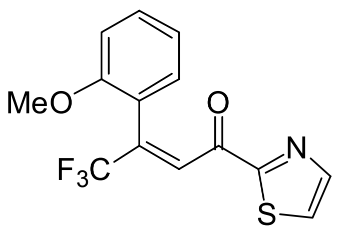

Yield: 50%, 670 mg (4.3 mmol scale Wittig reaction; reaction conditions: 4 days at 50°C); yellow oil; ^{1}H NMR (400 MHz, $CDCl_3$) δ 8.04 (d, *J* = 3.0 Hz, 1H), 8.01 (q, *J* = 1.4 Hz, 1H), 7.68 (d, *J* = 3.0 Hz, 1H), 7.39 (ddd, *J* = 8.4, 7.5, 1.7 Hz, 1H), 7.21 (dm, *J* = 7.6, Hz, 1H), 7.00 (td, *J* = 7.5, 1.0 Hz, 1H), 6.93 (dd, *J* = 8.4, 0.9 Hz, 1H), 3.68 (s, 3H); ^{13}C NMR (100 MHz, $CDCl_3$) δ 180.9 (C=O), 167.4 (C), 156.9 (C), 145.0 (CH), 139.6 (q, *J* = 31.5 Hz, $\underline{C}$-CF_3), 130.8 (CH), 129.3 (CH), 127.1 (CH), 126.8 (q, *J* = 5.1 Hz, CH), 122.6 (q, *J* = 275.4 Hz, CF_3), 120.5 (CH), 120.3 (C), 111.0 (CH), 55.5 (CH_3); ^{19}F NMR (376 MHz, $CDCl_3$) δ -67.2 (s); HRMS (ESI) m/z: $[M + H]^+$ Calcd for $C_{14}H_{11}F_3NO_2S$ 314.0457; Found 314.0458.

(*E*)-4,4,4-Trifluoro-3-(furan-2-yl)-1-(thiazol-2-yl)but-2-en-1-one (**4m**):

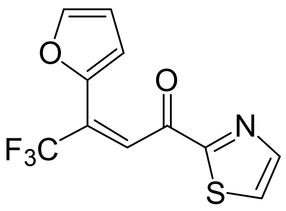

Yield: 70%, 1.05 g (5.5 mmol scale Wittig reaction); yellow oil (solidified at –20°C); ^{1}H NMR (400 MHz, $CDCl_3$) δ 8.02 (d, *J* = 3.0 Hz, 1H), 7.73 (d, *J* = 3.0 Hz, 1H), 7.45 – 7.40 (m, 2H), 7.11 (d, *J* = 3.5 Hz, 1H), 6.49 (dd, *J* = 3.6, 1.8 Hz, 1H); ^{13}C NMR (100 MHz, $CDCl_3$) δ 182.6 (C=O), 167.0 (C), 145.1 (CH), 145.0 (CH), 129.2 (q, *J* = 31.8 Hz, C-CF_3), 126.9 (CH), 122.9 (q, *J* = 5.5 Hz, CH), 122.2 (q, *J* = 275.3 Hz, CF_3), 116.2 (q, *J* = 1.5 Hz, CH), 112.0 (CH); ^{19}F NMR (376 MHz, $CDCl_3$) δ -64.7 (s); HRMS (ESI) m/z: $[M + H]^+$ Calcd for $C_{11}H_7F_3NO_2S$ 274.0144; Found 274.0141.

(*Z*)-4,4,4-Trifluoro-1-(thiazol-2-yl)-3-(thiophen-2-yl)but-2-en-1-one (**4n**):

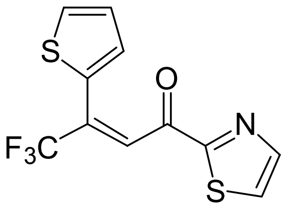

Yield: 88%, 815 mg (3.2 mmol scale Wittig reaction); yellow oil (solidified at 0°C); ^{1}H NMR (400 MHz, $CDCl_3$) δ 8.04 (d, *J* = 3.0 Hz, 1H), 7.80 (bs, 1H), 7.72 (d, *J* = 3.0 Hz, 1H), 7.49 (dd, *J* = 5.1, 0.8 Hz, 1H), 7.40 (d, *J* = 3.7 Hz, 1H), 7.07 (dd, *J* = 5.0, 3.9 Hz, 1H); ^{13}C NMR (100 MHz, $CDCl_3$) δ 181.6 (C=O), 167.1 (C), 145.1 (CH), 135.4 (q, *J* = 31.4 Hz, C-CF_3), 131.8 (CH), 130.3 (C), 129.7 (CH), 127.4 (CH), 127.1 (CH), 125.6 (q, *J* = 5.2 Hz, CH), 122.3 (q, *J* = 276.2 Hz, CF_3); ^{19}F NMR (376 MHz, $CDCl_3$) δ -66.2 (s); HRMS (ESI) m/z: $[M + H]^+$ Calcd for $C_{11}H_7F_3NOS_2$ 289.9916; Found 289.9913.

(*E*)-4,4,4-Trifluoro-1-(thiazol-2-yl)-3-(thiophen-3-yl)but-2-en-1-one (**4o**):

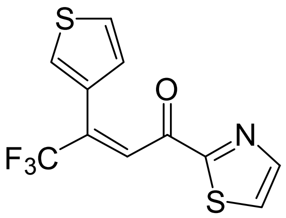

Yield: 81%, 795 mg (3.4 mmol scale Wittig reaction); yellow solid; ^{1}H NMR (400 MHz, $CDCl_3$) δ 8.04 (d, *J* = 3.0 Hz, 1H), 7.82 (q, *J* = 1.4 Hz, 1H), 7.71 (d, *J* = 3.0 Hz, 1H), 7.61 – 7.58 (m, 1H), 7.33 (dd, *J* = 5.1, 3.0 Hz, 1H), 7.15 (dm, *J* = 5.1 Hz, 1H); ^{13}C NMR (100 MHz, $CDCl_3$) δ 181.7 (C=O), 167.1 (C), 145.1 (CH), 137.6 (q, *J* = 31.2 Hz, C-CF_3), 130.0 (C), 128.4 (CH), 128.0 (q, *J* = 0.8 Hz, CH), 127.4 (CH), 125.6 (q, *J* = 5.4 Hz, CH), 125.4 (CH), 122.5 (q, *J* = 275.4 Hz, CF_3); ^{19}F NMR (376 MHz, $CDCl_3$) δ -66.7 (s); HRMS (ESI) m/z: $[M + H]^+$ Calcd for $C_{11}H_7F_3NOS_2$ 289.9916; Found 289.9910.

(*E*)-4,4,4-Trifluoro-3-(pyridin-3-yl)-1-(thiazol-2-yl)but-2-en-1-one (**4p**):

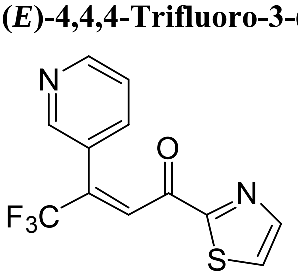

Yield: 69%, 940 mg (4.8 mmol scale Wittig reaction); yellow solid; ^{1}H NMR (400 MHz, $CDCl_3$) δ 8.67 (dd, *J* = 4.9, 1.7 Hz, 1H), 8.55 (d, *J* = 2.2 Hz, 1H), 8.08 (d, *J* = 3.0 Hz, 1H), 8.06 (q, *J* = 1.4 Hz, 1H), 7.75 (d, *J* = 3.0 Hz, 1H), 7.70 (dm, *J* = 7.9 Hz, 1H), 7.37 (ddd, *J* = 7.9, 4.9, 0.9 Hz, 1H); ^{13}C NMR (100 MHz, $CDCl_3$) δ 180.7 (C=O), 166.7 (C), 150.3 (CH), 149.1 (CH), 145.2 (CH), 139.9 (q, *J* = 31.8 Hz, C-CF_3), 136.4 (CH), 127.9 (CH), 127.5 (q, *J* = 5.1 Hz, CH), 127.3 (C), 122.9 (CH), 122.2 (q, *J* = 275.1 Hz, CF_3); ^{19}F NMR (376 MHz, $CDCl_3$) δ -67.4 (s); HRMS (ESI) m/z: $[M + H]^+$ Calcd for $C_{12}H_8F_3N_2OS$ 285.0304; Found 285.0301.

(*E*)-1-(Thiazol-2-yl)-3-(trifluoromethyl)pent-2-en-1-one (**4q**):

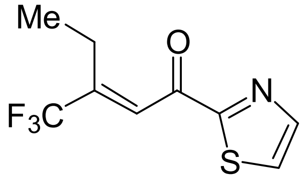

Yield: 59%, 623 mg (4.5 mmol scale Wittig reaction; reaction conditions: 5 days at 50°C); pale yellow oil (solid in the refrigerator); ^{1}H NMR (400 MHz, $CDCl_3$) δ 8.05 (d, *J* = 3.0 Hz, 1H), 7.81 (q, *J* = 1.4 Hz, 1H), 7.74 (d, *J* = 3.0 Hz, 1H), 2.79 (q, *J* = 7.5 Hz, 2H), 1.25 (t, *J* = 7.5 Hz, 3H); ^{13}C NMR (100 MHz, $CDCl_3$) δ 181.9 (C=O), 167.9 (C), 149.3 (q, *J* = 28.8 Hz, C-CF_3), 145.0 (CH), 127.3 (CH), 123.6 (q, *J* = 275.7 Hz, CF_3), 122.7 (q, *J* = 5.9 Hz, CH), 20.8 (CH_2), 13.2 (CH_3); ^{19}F NMR (376 MHz, $CDCl_3$) δ -69.1 (s); HRMS (ESI) m/z: $[M + H]^+$ Calcd for $C_9H_9F_3NOS$ 236.0351; Found 236.0350.

(*E*)-1-(Thiazol-2-yl)-3-(trifluoromethyl)hept-2-en-1-one (**4r**):

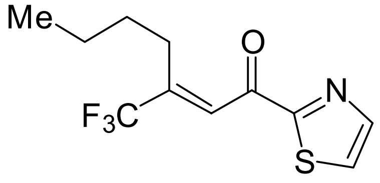

Yield: 55%, 580 mg (4 mmol scale Wittig reaction); pale yellow oil; ^{1}H NMR (400 MHz, $CDCl_3$) δ 8.05 (d, *J* = 2.9 Hz, 1H), 7.82 (bs, 1H), 7.74 (d, *J* = 2.9 Hz, 1H), 2.79 – 2.71 (m, 2H), 1.65 – 1.55 (m, 2H), 1.51 – 1.40 (m, 2H), 0.96 (t, *J* = 7.3 Hz, 3H); ^{13}C NMR (100 MHz, $CDCl_3$) δ 181.9 (C=O), 167.9 (C), 148.4 (q, *J* = 29.0 Hz, C-CF_3), 145.0 (CH), 127.3 (CH), 123.5 (q, *J* = 275.7 Hz, CF_3), 122.8 (q, *J* = 5.8 Hz, CH), 30.9 (CH_2), 27.3 (CH_2), 23.0 (CH_2), 13.6 (CH_3); ^{19}F NMR (376 MHz, $CDCl_3$) δ -69.0 (s); HRMS (ESI) m/z: $[M + H]^+$ Calcd for $C_{11}H_{13}F_3NOS$ 264.0664; Found 264.0661.

(*E*)-5-Phenyl-1-(thiazol-2-yl)-3-(trifluoromethyl)pent-2-en-1-one (**4s**):

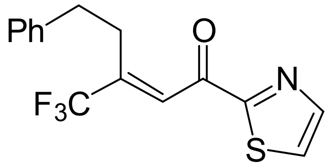

Yield: 88%, 820 mg (3 mmol scale Wittig reaction); pale yellow oil (solidified upon standing); ^{1}H NMR (400 MHz, $CDCl_3$) δ 8.05 (d, *J* = 3.0 Hz, 1H), 7.87 (d, *J* = 1.4 Hz, 1H), 7.73 (d, *J* = 3.0 Hz, 1H), 7.36 – 7.26 (m, 4H), 7.20 – 7.14 (m, 1H), 3.06 – 3.00 (m, 2H), 2.93 – 2.86 (m, 2H); ^{13}C NMR (100 MHz, $CDCl_3$) δ 181.8 (C=O), 167.7 (C), 146.5 (q, *J* = 29.3 Hz, C-CF_3), 145.0 (CH), 140.6 (C), 128.5 (2 x CH), 128.4 (2 x CH), 127.4 (CH), 126.2 (CH), 123.7 (q, *J* = 5.8 Hz, CH), 123.5 (q, *J* = 275.6 Hz, CF_3), 34.9 (CH_2), 29.8 (CH_2); ^{19}F NMR (376 MHz, $CDCl_3$) δ -69.0 (s); HRMS (ESI) m/z: $[M + H]^+$ Calcd for $C_{15}H_{13}F_3NOS$ 312.0664; Found 312.0661.

(*E*/*Z*)-Ethyl 5-oxo-5-(thiazol-2-yl)-3-(trifluoromethyl)pent-3-enoate (**4t**):

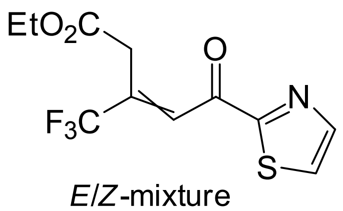

E/*Z*-mixture

Yield: 82%, 1.20 g (5 mmol scale Wittig reaction); light yellow oil; *E*/*Z* mixture ~ 1:1; ^{1}H NMR (400 MHz, $CDCl_3$) δ 8.07 (d, *J* = 3.0 Hz, 1H, (*E*)), 8.05 (bs, 1H, (*E*)), 8.04 (d, *J* = 3.0 Hz, 1H, (*Z*)), 7.77 (d, *J* = 3.0 Hz, 1H, (*E*)), 7.73 (d, *J* = 3.0 Hz, 1H, (*Z*)), 6.66 (bs, 1H, (*Z*)), 4.63 (s, 2H, (*Z*)), 4.20 (q, *J* = 7.3 Hz, 2H, (*E*)), 4.17 (q, *J* = 7.2 Hz, 2H, (*Z*)), 3.82 (s, 2H, (*E*)), 1.26 (t, *J* = 7.7 Hz, (*E*)), 1.24 (t, *J* = 7.7 Hz, (*Z*)); ^{13}C NMR (101 MHz, $CDCl_3$) δ 186.5 and 181.6 (*Z/E*, C=O), 167.8 and 167.1 (*E*/*Z*, C), 165.5 and 164.3 (*Z/E*, C), 145.1 and 144.8 (*E*/*Z*, CH), 138.5 (q, *J* = 31.4 Hz) and 137.8 (q, *J* = 31.5 Hz) (*E*/*Z*, C-CF_3), 127.8 and 126.7 (*E*/*Z*, CH), 125.8 (q, *J* = 5.5 Hz) and 125.6 (q, *J* = 5.8 Hz) (*E*/*Z*, CH), 122.8 (q, *J* = 274.4 Hz) and 122.7 (q, *J* = 274.0 Hz) (*E*/*Z*, CF_3), 61.4 and 61.2 (*E*/*Z*, CH_2), 36.6 and 32.6 (*E*/*Z*, CH_2), 14.0 and 13.9 (*E*/*Z*, CH_3); ^{19}F NMR (376 MHz, $CDCl_3$) δ -70.1 (s) and -70.3 (s); HRMS (ESI) m/z: $[M + H]^+$ Calcd for $C_{11}H_{11}F_3NO_3S$ 294.0406; Found 294.0402.

(*E*)-3-((1,3-Dioxolan-2-yl)methyl)-4,4,4-trifluoro-1-(thiazol-2-yl)but-2-en-1-one (**4u**):

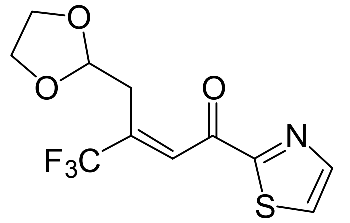

Yield: 55%, 810 mg (5 mmol scale Wittig reaction); light yellow oil; ^{1}H NMR (400 MHz, $CDCl_3$) δ 8.04 (d, *J* = 3.0 Hz, 1H), 7.87 (bs, 1H), 7.74 (d, *J* = 3.0 Hz, 1H), 5.22 (t, *J* = 5.0 Hz, 1H), 3.97 – 3.87 (m, 2H), 3.85 – 3.76 (m, 2H), 3.15 (d, *J* = 5.0 Hz, 2H); ^{13}C NMR (100 MHz, $CDCl_3$) δ 182.0 (C=O), 167.5 (C), 145.0 (CH), 140.2 (q, *J* = 30.5 Hz, C-CF_3), 127.3 (CH), 126.6 (q, *J* = 5.7 Hz, CH), 123.1 (q, *J* = 275.1 Hz, CF_3), 102.3 (CH), 64.8 (2 × CH_2), 31.6 (CH_2); ^{19}F NMR (376 MHz, $CDCl_3$) δ -68.3 (s); HRMS (ESI) m/z: $[M + H]^+$ Calcd for $C_{11}H_{11}F_3NO_3S$ 294.0406; Found 294.0403.

(*E*)-1-(Benzo[d]thiazol-2-yl)-4,4,4-trifluoro-3-(4-fluorophenyl)but-2-en-1-one (**6a**):

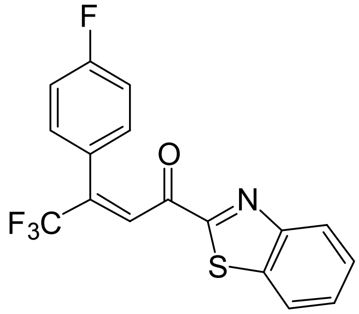

Yield: 57%, 502 mg (2.5 mmol scale Wittig reaction); yellow solid; ^{1}H NMR (400 MHz, $CDCl_3$) δ 8.23 (dm, *J* = 8.1 Hz, 1H), 8.08 (q, *J* = 1.3 Hz, 1H), 7.96 (dm, *J* = 7.7 Hz, 1H), 7.64 – 7.53 (m, 2H), 7.37 – 7.31 (m, 2H), 7.14 – 7.06 (m, 2H); ^{13}C NMR (100 MHz, $CDCl_3$) δ 182.6 (C=O), 166.4 (C), 163.4 (d, *J* = 249.8 Hz, C-F), 153.4 (C), 142.7 (q, *J* = 31.2 Hz, C-CF_3), 137.6 (C), 130.9 (d, *J* = 8.5 Hz, 2 × CH), 128.3 (CH), 127.3 (CH), 126.7 (d, *J* = 3.6 Hz, C), 126.1 (q, *J* = 5.1 Hz, CH), 125.8 (CH), 122.44 (q, *J* = 276.2 Hz, CF_3), 122.43 (CH), 115.6 (d, *J* = 21.9 Hz, 2 × CH); ^{19}F NMR (376 MHz, $CDCl_3$) δ -67.4 (s, 3F), -110.8 – -111.0 (m, 1F); HRMS (ESI) m/z: $[M + H]^+$ Calcd for $C_{17}H_{10}F_4NOS$ 352.0414; Found 352.0411.

(*E*)-1-(Benzo[d]thiazol-2-yl)-4,4,4-trifluoro-3-(*p*-tolyl)but-2-en-1-one (**6b**):

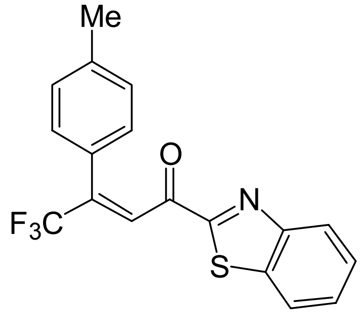

Yield: 48%, 399 mg (2.4 mmol scale Wittig reaction); yellow solid; ^{1}H NMR (400 MHz, $CDCl_3$) δ 8.23 (dm, *J* = 8.3 Hz, 1H), 8.04 (q, *J* = 1.3 Hz, 1H), 7.95 (dm, *J* = 7.9 Hz, 1H), 7.63 – 7.52 (m, 2H), 7.27 – 7.19 (m, 4H), 2.38 (s, 3H); ^{13}C NMR (100 MHz, $CDCl_3$) δ 182.8 (C=O), 166.7 (C), 153.4 (C), 144.1 (q, *J* = 30.8 Hz, C-CF_3), 139.6 (C), 137.6 (C), 129.1 (2 x CH), 128.7 (2 x CH), 128.1 (CH), 127.9 (C), 127.2 (CH), 125.8 (CH), 125.4 (q, *J* = 5.2 Hz, CH), 122.6 (q, *J* = 275.4 Hz, CF_3), 122.4 (CH), 21.4 (CH_3); ^{19}F NMR (376 MHz, $CDCl_3$) δ -67.2 (s); HRMS (ESI) m/z: $[M + H]^+$ Calcd for $C_{18}H_{13}F_3NOS$ 348.0664; Found 348.0662.

(*E*)-1-(Benzo[d]thiazol-2-yl)-4,4,4-trifluoro-3-(4-(trifluoromethyl)phenyl)but-2-en-1-one (**6c**):

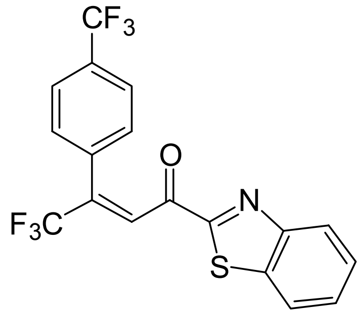

Yield: 58%, 468 mg (2.0 mmol scale Wittig reaction); yellow solid; ^{1}H NMR (400 MHz, $CDCl_3$) δ 8.24 (d, *J* = 7.6 Hz, 1H), 8.17 (q, *J* = 1.3 Hz, 1H), 7.96 (d, *J* = 7.6 Hz, 1H), 7.68 (d, *J* = 8.1 Hz, 2H), 7.64 – 7.53 (m, 2H), 7.47 (d, *J* = 8.1 Hz, 2H); ^{13}C NMR (100 MHz, $CDCl_3$) δ 182.3 (C=O), 166.1 (C), 153.4 (C), 142.4 (q, *J* = 31.4 Hz, C-CF_3), 137.7 (C), 134.7 (C), 131.5 (q, *J* = 32.8 Hz, C-CF_3), 129.3 (2 x CH), 128.4 (CH), 127.4 (CH), 126.5 (q, *J* = 5.1 Hz, CH), 125.9 (CH), 125.4 (q, *J* = 3.8 Hz, 2 x CH), 123.8 (q, *J* = 272.3 Hz, CF_3), 122.5 (CH), 122.2 (q, *J* = 275.2 Hz, CF_3); ^{19}F NMR (376 MHz, $CDCl_3$) δ -62.8 (s, 3F), -67.2 (s, 3F); HRMS (ESI) m/z: $[M + H]^+$ Calcd for $C_{18}H_{10}F_6NOS$ 402.0382; Found 402.0379.

(*E*)-1-(Benzo[d]thiazol-2-yl)-3-(3-chlorophenyl)-4,4,4-trifluorobut-2-en-1-one (**6d**):

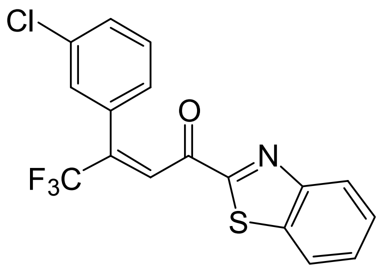

Yield: 50%, 462 mg (2.5 mmol scale Wittig reaction); light yellow solid; ^{1}H NMR (400 MHz, CDCl$_3$) δ 8.22 (ddd, *J* = 8.3, 1.3, 0.7 Hz, 1H), 8.09 (q, *J* = 1.3 Hz, 1H), 7.94 (ddd, *J* = 7.8, 1.3, 0.6 Hz, 1H), 7.62 – 7.51 (m, 2H), 7.40 (ddd, *J* = 8.0, 2.1, 1.2 Hz, 1H), 7.36 – 7.31 (m, 2H), 7.22 (dm, *J* = 7.6 Hz, 1H); ^{13}C NMR (100 MHz, CDCl$_3$) δ 182.3 (C=O), 166.2 (C), 153.4 (C), 142.2 (q, *J* = 31.4 Hz, C-CF$_3$), 137.6 (C), 134.3 (C), 132.6 (C), 129.7 (CH), 129.6 (CH), 128.7 (CH), 128.3 (CH), 127.4 (CH), 127.1 (CH), 126.4 (q, *J* = 5.1 Hz, CH), 125.9 (CH), 122.4 (CH), 122.3 (q, *J* = 275.3 Hz, CF$_3$); ^{19}F NMR (376 MHz, CDCl$_3$) δ -67.3 (s); HRMS (ESI) m/z: [M + H]$^+$ Calcd for $C_{17}H_{10}ClF_3NOS$ 368.0118; Found 368.0117.

(*E*)-1-(Benzo[d]thiazol-2-yl)-4,4,4-trifluoro-3-(3-methoxyphenyl)but-2-en-1-one (**6e**):

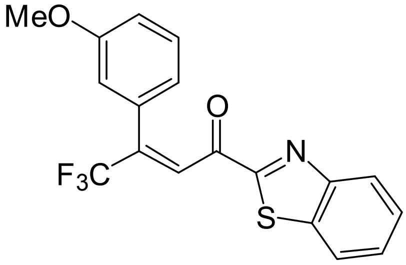

Yield: 56%, 510 mg (2.5 mmol scale Wittig reaction); yellow solid; ^{1}H NMR (400 MHz, CDCl$_3$) δ 8.23 (ddd, *J* = 8.2, 1.4, 0.7 Hz, 1H), 8.03 (q, *J* = 1.4 Hz, 1H), 7.96 (ddd, *J* = 7.9, 1.4, 0.7 Hz, 1H), 7.63 – 7.52 (m, 2H), 7.32 (ddd, *J* = 8.3, 7.6, 0.4 Hz, 1H), 6.96 (ddd, *J* = 8.4, 2.6, 0.9 Hz, 1H), 6.91 (dm, *J* = 7.6 Hz, 1H), 6.88 – 6.86 (m, 1H), 3.79 (s, 3H); ^{13}C NMR (100 MHz, CDCl$_3$) δ 182.8 (C=O), 166.5 (C), 159.3 (C), 153.4 (C), 143.4 (q, *J* = 31.1 Hz, C-CF$_3$), 137.6 (C), 132.1 (C), 129.5 (CH), 128.2 (CH), 127.3 (CH), 126.1 (q, *J* = 5.1 Hz, CH), 125.8 (CH), 122.5 (q, *J* = 274.8 Hz, CF$_3$), 122.4 (CH), 121.1 (CH), 115.0 (CH), 114.4 (CH), 55.2 (CH$_3$); ^{19}F NMR (376 MHz, CDCl$_3$) δ -67.2 (s); HRMS (ESI) m/z: [M + H]$^+$ Calcd for $C_{18}H_{13}F_3NO_2S$ 364.0614; Found 364.0611.

(*E*)-1-(Benzo[d]thiazol-2-yl)-3-(3,5-dimethylphenyl)-4,4,4-trifluorobut-2-en-1-one (**6f**):

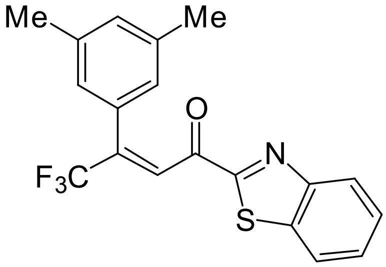

Yield: 59%, 509 mg (2.4 mmol scale Wittig reaction); yellow solid; ^{1}H NMR (400 MHz, CDCl$_3$) δ 8.22 (d, *J* = 8.2 Hz, 1H), 8.00 (q, *J* = 1.1 Hz, 1H), 7.94 (dm, *J* = 8.0 Hz, 1H), 7.61 – 7.50 (m, 2H), 7.03 (s, 1H), 6.94 (s, 2H), 2.31 (s, 6H); ^{13}C NMR (100 MHz, CDCl$_3$) δ 182.9 (C=O), 166.6 (C), 153.4 (C), 144.2 (q, *J* = 31.0 Hz, C-CF$_3$), 137.9 (2 x C), 137.5 (C), 131.2 (CH), 130.8 (C), 128.1 (CH), 127.2 (CH), 126.3 (2 x CH), 125.8 (CH), 125.5 (q, *J* = 5.2 Hz, CH), 122.6 (q, *J* = 275.0 Hz, CF$_3$), 122.4 (CH), 21.2 (2 × CH$_3$); ^{19}F NMR (376 MHz, CDCl$_3$) δ -67.2 (s); HRMS (ESI) m/z: [M + H]$^+$ Calcd for $C_{19}H_{15}F_3NOS$ 362.0821; Found 362.0818.

(*E*)-1-(Benzo[d]thiazol-2-yl)-4,4,4-trifluoro-3-(naphthalen-2-yl)but-2-en-1-one (**6g**):

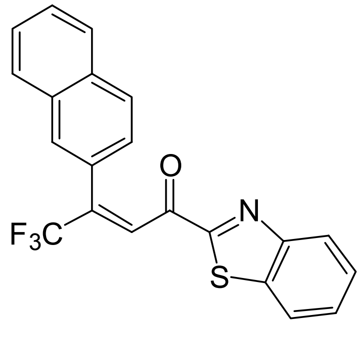

Yield: 54%, 415 mg (2.0 mmol scale Wittig reaction); yellow solid; ^{1}H NMR (400 MHz, CDCl$_3$) δ 8.22 (ddd, *J* = 8.3, 1.2, 0.7 Hz, 1H), 8.15 (q, *J* = 1.3 Hz, 1H), 7.89 (ddd, *J* = 8.0, 1.3, 0.7 Hz, 1H), 7.87 – 7.79 (m, 4H), 7.59 – 7.54 (m, 1H), 7.53 – 7.45 (m, 3H), 7.42 (dd, *J* = 8.6, 1.2 Hz, 1H); ^{13}C NMR (100 MHz, CDCl$_3$) δ 182.6 (C=O), 166.5 (C), 153.4 (C), 143.9 (q, *J* = 31.0 Hz, C-CF$_3$), 137.6 (C), 133.4 (C), 132.7 (C), 128.4 (CH), 128.38 (CH), 128.36 (C), 128.2 (CH), 128.0 (CH), 127.8 (CH), 127.3 (CH), 127.0 (CH), 126.5 (CH), 126.1 (CH), 126.0 (q, *J* = 5.2 Hz, CH), 125.8 (CH), 122.6 (q, *J* = 275.5 Hz, CF$_3$), 122.4 (CH); ^{19}F NMR (376 MHz, CDCl$_3$) δ -66.9 (s); HRMS (ESI) m/z: [M + H]$^+$ Calcd for $C_{21}H_{13}F_3NOS$ 384.0664; Found 384.0662.

(*E*)-1-(Benzo[d]thiazol-2-yl)-4,4,4-trifluoro-3-(furan-2-yl)but-2-en-1-one (**6h**):

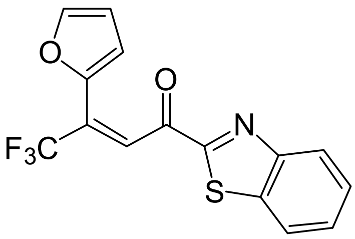

Yield: 60%, 483 mg (2.5 mmol scale Wittig reaction); yellow solid; ^{1}H NMR (400 MHz, $CDCl_3$) δ 8.18 (dm, *J* = 8.2 Hz, 1H), 7.98 (dm, *J* = 8.0 Hz, 1H), 7.60 – 7.51 (m, 3H), 7.44 (d, *J* = 1.8 Hz, 1H), 7.20 (dm, *J* = 3.6 Hz, 1H), 6.49 (dd, *J* = 3.6, 1.8 Hz, 1H); ^{13}C NMR (100 MHz, $CDCl_3$) δ 183.7 (C=O), 166.5 (C), 153.5 (C), 145.2 (CH), 145.1 (C), 137.4 (C), 129.7 (q, *J* = 31.6 Hz, C-CF_3), 128.0 (CH), 127.2 (CH), 125.8 (CH), 122.4 (q, *J* = 5.4 Hz, CH), 122.3 (CH), 122.3 (q, *J* = 275.5 Hz, CF_3), 116.7 (q, *J* = 1.4 Hz, CH), 112.1 (CH); ^{19}F NMR (376 MHz, $CDCl_3$) δ -64.5 (s); HRMS (ESI) m/z: $[M + H]^+$ Calcd for $C_{15}H_9F_3NO_2S$ 324.0301; Found 324.0298.

(*Z*)-1-(Benzo[d]thiazol-2-yl)-4,4,4-trifluoro-3-(thiophen-2-yl)but-2-en-1-one (**6i**):

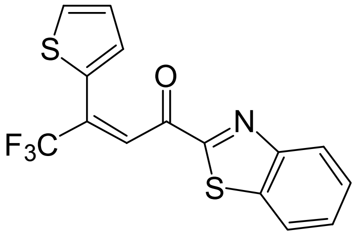

Yield: 53%, 560 mg (3.1 mmol scale Wittig reaction); yellow solid; ^{1}H NMR (400 MHz, $CDCl_3$) δ 8.21 (ddd, *J* = 8.3, 1.4, 0.7 Hz, 1H), 7.98 – 7.94 (m, 2H), 7.61 – 7.51 (m, 2H), 7.49 (dd, *J* = 5.1, 1.2 Hz, 1H), 7.45 (dm, *J* = 3.7 Hz, 1H), 7.07 (dd, *J* = 5.1, 3.8 Hz, 1H); ^{13}C NMR (100 MHz, $CDCl_3$) δ 182.8 (C=O), 166.5 (C), 153.4 (C), 137.6 (C), 136.0 (q, *J* = 31.4 Hz, C-CF_3), 132.0 (CH), 130.3 (C), 129.9 (CH), 128.2 (CH), 127.3 (CH), 127.2 (CH), 125.8 (CH), 125.0 (q, *J* = 5.3 Hz, CH), 122.4 (CH), 122.3 (q, *J* = 276.2 Hz, CF_3); ^{19}F NMR (376 MHz, $CDCl_3$) δ -66.1 (s); HRMS (ESI) m/z: $[M + H]^+$ Calcd for $C_{15}H_9F_3NOS_2$ 340.0072; Found 340.0071.

(*E*)-1-(Benzo[d]thiazol-2-yl)-3-(trifluoromethyl)pent-2-en-1-one (**6j**):

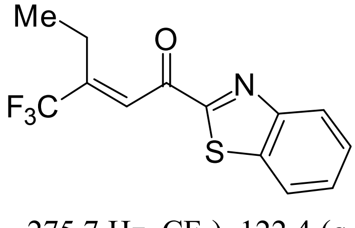

Yield: 45%, 385 mg (3.0 mmol scale Wittig reaction); white solid; ^{1}H NMR (400 MHz, $CDCl_3$) δ 8.20 (dm, *J* = 8.1 Hz, 1H), 7.97 (dm, *J* = 8.1 Hz, 1H), 7.93 (q, *J* = 1.3 Hz, 1H), 7.61 – 7.51 (m, 2H), 2.81 (q, *J* = 7.5 Hz, 2H), 1.27 (t, *J* = 7.5 Hz, 3H); ^{13}C NMR (100 MHz, $CDCl_3$) δ 183.3 (C=O), 167.1 (C), 153.4 (C), 149.7 (q, *J* = 28.9 Hz, C-CF_3), 137.6 (C), 128.1 (CH), 127.2 (CH), 125.8 (CH), 123.6 (q, *J* = 275.7 Hz, CF_3), 122.4 (q, *J* = 5.8 Hz, CH), 122.4 (CH), 21.0 (CH_2), 13.2 (CH_3); ^{19}F NMR (376 MHz, $CDCl_3$) δ -69.1 (s); HRMS (ESI) m/z: $[M + H]^+$ Calcd for $C_{13}H_{11}F_3NOS$ 286.0508; Found *m/z* 286.0506.

(*E*)-1-(Benzo[d]thiazol-2-yl)-5-phenyl-3-(trifluoromethyl)pent-2-en-1-one (**6k**):

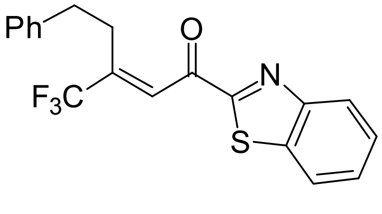

Yield: 52%, 490 mg (2.6 mmol scale Wittig reaction); light yellow solid; ^{1}H NMR (400 MHz, $CDCl_3$) δ 8.20 (ddd, *J* = 8.2, 1.4, 0.7 Hz, 1H), 8.00 (q, *J* = 1.4 Hz, 1H), 7.96 (ddd, *J* = 7.7, 1.5, 0.7 Hz, 1H), 7.61 – 7.50 (m, 2H), 7.37 – 7.25 (m, 4H), 7.19 – 7.13 (m, 1H), 3.09 – 3.01 (m, 2H), 2.95 – 2.87 (m, 2H); ^{13}C NMR (100 MHz, $CDCl_3$) δ 183.2 (C=O), 166.9 (C), 153.4 (C), 147.1 (q, *J* = 29.4 Hz, C-CF_3), 140.6 (C), 137.6 (C), 128.6 (2 x CH), 128.5 (2 x CH), 128.1 (CH), 127.2 (CH), 126.3 (CH), 125.8 (CH), 123.5 (q, *J* = 275.6 Hz, CF_3), 123.4 (q, *J* = 5.8 Hz, CH), 122.4 (CH), 34.9 (CH_2), 29.9 (CH_2); ^{19}F NMR (376 MHz, $CDCl_3$) δ -69.0 (s); HRMS (ESI) m/z: $[M + H]^+$ Calcd for $C_{19}H_{15}F_3NOS$ 362.0821; Found 362.0818.

(*E*)-4-Chloro-4,4-difluoro-3-phenyl-1-(thiazol-2-yl)but-2-en-1-one (**8a**):

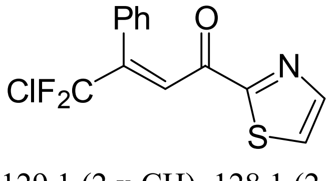

Yield: 82%, 1.23 g (5 mmol scale Wittig reaction); yellow solid; ^{1}H NMR (400 MHz, $CDCl_3$) δ 8.05 (d, *J* = 3.0 Hz, 1H), 7.90 (t, *J* = 1.2 Hz, 1H), 7.70 (d, *J* = 3.0 Hz, 1H), 7.47 – 7.38 (m, 3H), 7.36 – 7.32 (m, 2H); ^{13}C NMR (100 MHz, $CDCl_3$) δ 181.2 (C=O), 167.3 (C), 148.1 (t, *J* = 24.5 Hz, C-CF_2Cl), 145.0 (CH), 131.4 (C), 129.2 (CH), 129.1 (2 x CH), 128.1 (2 x CH), 127.4 (CH), 125.2 (t, *J* = 292.7 Hz, CF_2Cl), 123.9 (t, *J* = 6.3 Hz, CH); ^{19}F NMR (376 MHz, $CDCl_3$) δ -55.1 (s); HRMS (ESI) m/z: $[M + H]^+$ Calcd for $C_{13}H_9ClF_2NOS$ 300.0056; Found 300.0056.

(*E*)-4,4,5,5,5-Pentafluoro-3-phenyl-1-(thiazol-2-yl)pent-2-en-1-one (**8b**):

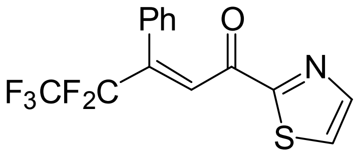

Yield: 85%, 850 mg (3 mmol scale Wittig reaction); yellow solid; ^{1}H NMR (400 MHz, $CDCl_3$) δ 8.05 (d, *J* = 3.0 Hz, 1H), 7.94 (t, *J* = 1.3 Hz, 1H), 7.70 (d, *J* = 3.0 Hz, 1H), 7.44 – 7.35 (m, 3H), 7.29 – 7.25 (m, 2H); ^{13}C NMR (100 MHz, $CDCl_3$) δ 181.0 (C=O), 167.1 (C), 145.1 (CH), 142.9 (t, *J* = 22.2 Hz, C-CF_2), 131.1 (C), 129.3 (CH), 129.0 (2 × CH), 128.1 (2 × CH), 127.4 (CH), 124 – 108 (m, CF_2CF_3); ^{19}F NMR (376 MHz, $CDCl_3$) δ -81.7 (t, *J* = 2.2 Hz, 3F), -114.8 (s, 2F); HRMS (ESI) m/z: $[M + H]^+$ Calcd for $C_{14}H_9F_5NOS$ 334.0319; Found 334.0319.

Analytical data of products of malonate addition to enones under high pressure: 3a-3l, 5a-5k, 5m-5u, 7a-7k, 9a and 9b

Diethyl 2-(1,1,1-trifluoro-4-oxo-2,4-diphenylbutan-2-yl)malonate (**3a**):

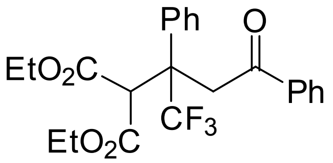

9% NMR yeld at 10 kbar for 3 days; reaction with β-trifluoromethylchalcone (**2a**) c=1.0 mol/L, 5 mol% of **1a**, 1.5 equiv of diethyl malonate in touene. Product was not isolated.
selected characteristic signals for **3a** from the reaction mixture: ^{1}H NMR (400 MHz, $CDCl_3$) δ 4.71 (dq, *J* = 18.7, 1.5 Hz, 1H), 4.50 (1H, s), 4.44 (d, *J* = 18.7 Hz, 1H), 0.92 (t, *J* = 7.1 Hz, 3H); ^{19}F NMR (376 MHz, $CDCl_3$) δ -63.3.

(*R*)-Diethyl 2-(1,1,1-trifluoro-4-(furan-2-yl)-4-oxo-2-phenylbutan-2-yl)malonate (**3b**):

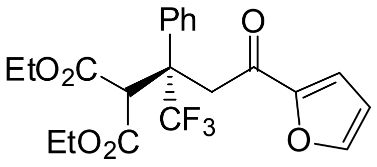

Product **3b** was prepared from enone **2b** according to general high-pressure procedure. A 1.2 mL Teflon ampoule was charged with 25.0 mg of catalyst (1*R*,2*R*)-**1a** (0.06 mmol, 5 mol%), 321 mg of enone **2b** (1.21 mmol), 289 mg of diethyl malonate (1.80 mmol, 1.5 equiv) and filled up with toluene (*ca.* 0.8 mL). The reaction was carried out under pressure of 10–9.5 kbar at 20–25°C for 72 h. Flash column chromatographyon a silica gel using hexane fraction from petroleum/DCM as an eluent afforded 164 mg (0.38 mmol; 32% yield, 37% conversion) of (*R*)-**3b** with 95.3% ee.
Light yellow oil; 95.3% ee, $[\alpha]_D^{22}$= –28,2 (c 1.04, $CHCl_3$); ^{1}H NMR (400 MHz, $CDCl_3$) δ 7.60 (dd, *J* = 1.7, 0.7 Hz, 1H), 7.55 – 7.50 (m, 2H), 7.36 – 7.29 (m, 3H), 7.25 (dd, *J* = 3.6, 0.7 Hz, 1H), 6.54 (dd, *J* = 3.6, 1.7 Hz, 1H), 4.52 (s, 1H), 4.45 (dq, *J* = 18.1, 1.1 Hz, 1H), 4.29 (d, *J* = 18.0 Hz, 1H), 4.31 – 4.19 (m, 2H), 3.91 (qd, *J* = 7.1, 1.2 Hz, 2H), 1.29 (t, *J* = 7.1 Hz, 3H), 0.95 (t, *J* = 7.1 Hz, 3H); ^{13}C NMR (100 MHz, $CDCl_3$) δ 184.5 (C=O), 166.5 (C=O), 166.1 (C=O), 152.6 (C), 146.5 (CH), 134.8 (C), 128.1 (CH), 128.0 (2 ×CH), 127.8 (q, *J* = 1.6 Hz, 2 ×CH), 126.6 (q, *J* = 286.8 Hz, CF_3), 117.5 (CH), 112.3 (CH), 62.0 (CH_2), 61.7 (CH_2), 55.8 (q, *J* = 1.8 Hz, CH), 53.7 (q, *J* = 25.0 Hz, <u>C</u>-CF_3), 36.8 (q, *J* = 1.5 Hz, CH_2), 13.8 (CH_3), 13.4 (CH_3); ^{19}F NMR (376 MHz, $CDCl_3$) δ -64.0; HRMS (ESI) m/z: $[M + H]^+$ Calcd for $C_{21}H_{22}F_3O_6$ 427.1363; Found 427.1369; Enantiomeric excess was determined by HPLC analysis using a Chiralpak® IA column (Hexane/*i*-PrOH: 98/2, flow rate 1.0 mL/min, λ = 219 nm): *ent*-(*R*)-major t_r = 18.3 min and *ent*-(*S*)-minor t_r = 19.8 min.

(*R*)-Diethyl 2-(1,1,1-trifluoro-4-oxo-2-phenyl-4-(thiophen-2-yl)butan-2-yl)malonate (**3c**):

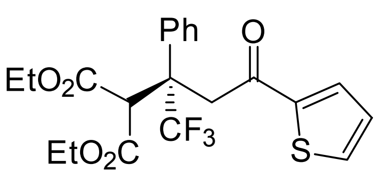

Product **3c** was prepared from enone **2c** according to general high-pressure procedure. A 1.0 mL Teflon ampoule was charged with 20.8 mg of catalyst (1*R*,2*R*)-**1a** (0.05 mmol, 5 mol%), 280 mg of enone **2c** (0.99 mmol), 245 mg of diethyl malonate (1.53 mmol, 1.5 equiv) and filled up with toluene (*ca.* 0.6 mL). The reaction was carried out under pressure of 10–9.5 kbar at 20–25°C for 72 h. Flash column chromatographyon a silica gel using hexane fraction from petroleum/DCM as an eluent afforded 172 mg (0.39 mmol; 39% yield) of (*R*)-**3c** with 99.5% ee.
Colorless oil; 99.5% ee, $[\alpha]_D^{22}$= –37.4 (c 1.01, $CHCl_3$); ^{1}H NMR (400 MHz, $CDCl_3$) δ 7.85 (dd, *J* = 3.8, 1.1 Hz, 1H), 7.65 (dd, *J* = 5.0, 1.1 Hz, 1H), 7.54 – 7.50 (m, 2H), 7.36 – 7.28 (m, 3H), 7.15 (dd, *J* = 5.0, 3.8 Hz, 1H), 4.54 (dq, *J* = 17.9, 1.4 Hz, 1H), 4.50 (s, 1H), 4.39 (d, *J* = 17.9 Hz, 1H), 4.32 – 4.18 (m, 2H), 3.96 – 3.85 (m, 2H), 1.30 (t, *J* = 7.1 Hz, 3H), 0.94 (t, *J* = 7.1 Hz, 3H); ^{13}C NMR (100 MHz, $CDCl_3$) δ 188.5 (C=O), 166.5 (C=O), 166.1 (C=O), 144.6 (C), 134.8 (C), 134.1 (CH), 132.2 (CH), 128.15 (CH), 128.12 (CH), 128.09 (2 × CH), 127.8 (q, *J* = 1.6 Hz, 2 × CH),

126.6 (q, J = 287.1 Hz, CF_3), 62.0 (CH_2), 61.7 (CH_2), 56.0 (q, J = 2.0 Hz, CH), 53.9 (q, J = 24.9 Hz, C-CF_3), 37.6 (q, J = 1.7 Hz, CH_2), 13.8 (CH_3), 13.5 (CH_3); ^{19}F NMR (376 MHz, $CDCl_3$) δ -63.6; HRMS (ESI) m/z: $[M + H]^+$ Calcd for $C_{21}H_{22}F_3O_5S$ 443.1135; Found 443.1141; Enantiomeric excess was determined by HPLC analysis using a Chiralpak® IA column (Hexane/*i*-PrOH: 98/2, flow rate 1.0 mL/min, λ = 219 nm): *ent*-(*R*)-major t_r = 15.7 min and *ent*-(*S*)-minor t_r = 17.0 min.

(*R*)-Diethyl 2-(1,1,1-trifluoro-4-oxo-2-phenyl-4-(pyridin-2-yl)butan-2-yl)malonate (**3d**):

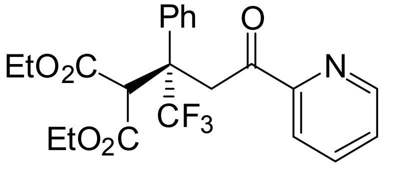

Product **3d** was prepared from enone **2d** according to general high-pressure procedure. A 1.0 mL Teflon ampoule was charged with 20.7 mg of catalyst (1*R*,2*R*)-**1a** (5 mol%), 281 mg of enone **2d** (1.01 mmol), 240 mg of diethyl malonate (1.5 mmol, 1.5 equiv) and filled up with toluene (*ca.* 0.6 mL). The reaction was carried out under pressure of 10–9.5 kbar at 20–25°C for 72 h. Flash column chromatographyon a silica gel using hexane fraction from petroleum/DCM as an eluent afforded 348 mg (0.80 mmol; 79% yield) of (*R*)-**3d** with 95.2% ee.

Pale yellow viscous oil; 95.2% ee, $[\alpha]_D^{25}$= –44.8 (c 1.18, $CHCl_3$); ^{1}H NMR (400 MHz, $CDCl_3$) δ 8.73 (ddd, J = 4.7, 1.7, 0.9 Hz, 1H), 7.99 (dt, J = 7.9, 1.1 Hz, 1H), 7.82 (td, J = 7.7, 1.7 Hz, 1H), 7.53 – 7.48 (m, 2H), 7.48 (ddd, J = 7.6, 4.7, 1.2 Hz, 1H), 7.35 – 7.26 (m, 3H), 4.99 (dq, J = 19.2, 1.2 Hz, 1H), 4.64 (d, J = 19.2 Hz, 1H), 4.59 (s, 1H), 4.25 (dq, J = 7.1, 2.2 Hz, 2H), 3.94 (qd, J = 7.1, 0.6 Hz, 2H), 1.29 (t, J = 7.1 Hz, 3H), 0.97 (t, J = 7.1 Hz, 3H); ^{13}C NMR (100 MHz, $CDCl_3$) δ 197.3 (C=O), 166.6 (C=O), 166.3 (C=O), 153.3 (C), 149.0 (CH), 136.8 (CH), 135.2 (C), 127.97 (2 × CH), 127.95 (2 ×CH + CH), 127.2 (CH), 126.8 (q, J = 287.0 Hz, CF_3), 121.8 (CH), 61.9 (CH_2), 61.6 (CH_2), 55.6 (q, J = 1.9 Hz, CH), 53.7 (q, J = 24.9 Hz, C-CF3), 36.6 (q, J = 1.9 Hz, CH_2), 13.8 (CH_3), 13.5 (CH_3); ^{19}F NMR (376 MHz, $CDCl_3$) δ -64.2; HRMS (ESI) m/z: $[M + H]^+$ Calcd for $C_{22}H_{23}F_3NO_5$ 438.1523; Found 438.1524; Enantiomeric excess was determined by HPLC analysis using a Chiralpak® IA column (Hexane/*i*-PrOH: 98/2, flow rate 1.0 mL/min, λ = 219 nm): *ent*-(*S*)-minor t_r = 20.8 min and *ent*-(*R*)-major t_r = 22.2 min.

(*R*)-Diethyl 2-(1,1,1-trifluoro-4-oxo-2-phenyl-4-(pyridin-4-yl)butan-2-yl)malonate (**3e**):

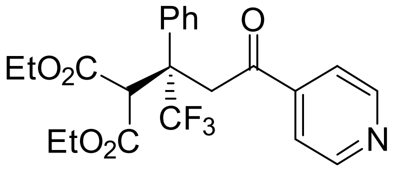

Product **3e** was prepared from enone **2e** according to general high-pressure procedure. A 0.7 mL Teflon ampoule was charged with 15.7 mg of catalyst (1*R*,2*R*)-**1a** (0.038 mmol, 5 mol%), 195 mg of enone **2e** (0.70 mmol), 170 mg of diethyl malonate (1.06 mmol, 1.5 equiv) and filled up with toluene (*ca.* 0.4 mL). The reaction was carried out under pressure of 10–9.5 kbar at 20–25°C for 72 h. Flash column chromatography on a silica gel using hexane fraction from petroleum/DCM as an eluent afforded 138 mg (0.32 mmol; 45% yield) of (*R*)-**3e** with 96% ee.

Colorless oil; 96% ee; ^{1}H NMR (400 MHz, $CDCl_3$) δ 8.84 (dm, J = 6.1 Hz, 2H), 7.81 (dm, J = 6.1 Hz, 2H), 7.45 – 7.38 (m, 2H), 7.37 – 7.30 (m, 3H), 4.71 (dq, J = 18.8, 1,5 Hz, 1H), 4.47 (d, J = 18.8 Hz, 1H), 4.44 (s, 1H), 4.34 – 4.20 (m, 2H), 3.95 – 3.83 (m, 2H), 1.31 (t, J = 7.1 Hz, 3H), 0.93 (t, J = 7.1 Hz, 3H); ^{13}C NMR (100 MHz, $CDCl_3$) δ 194.9 (C=O), 166.4 (C=O), 166.1 (C=O), 150.9 (2 × CH), 143.0 (C), 134.4 (C), 128.3 (CH), 128.2 (2 × CH), 127.6 (q, J = 1.5 Hz, 2 × CH), 126.6 (q, J = 287.1 Hz, CF_3), 121.1 (2 × CH), 62.2 (CH_2), 61.8 (CH_2), 56.0 (q, J = 2.0 Hz, CH), 53.8 (q, J = 25.0 Hz, C-CF_3), 37.1 (q, J = 1.7 Hz, CH_2), 13.8 (CH_3), 13.5 (CH_3); ^{19}F NMR (376 MHz, $CDCl_3$) δ -63.4; HRMS (ESI) m/z: $[M + H]^+$ Calcd for $C_{22}H_{23}F_3NO_5$ 438.1523; Found 438.1526; Enantiomeric excess was determined by HPLC analysis using a Chiralpak® IC column (Hexane/*i*-PrOH: 85/15, flow rate 1.0 mL/min, λ = 219 nm): *ent*-(*S*)-minor t_r = 25.7 min and *ent*-(*R*)-major t_r = 29.6 min.

(*R*)-Diethyl 2-(1,1,1-trifluoro-4-oxo-2-phenyl-4-(pyridin-3-yl)butan-2-yl)malonate (3f):

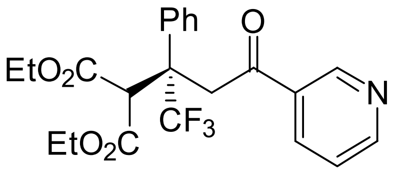

Product **3f** was prepared from enone **2f** according to general high-pressure procedure. A 1.0 mL Teflon ampoule was charged with 22.0 mg of catalyst (1*R*,2*R*)-**1a** (0.053 mmol, 5 mol%), 283 mg of enone **2f** (1.02 mmol), 245 mg of diethyl malonate (1.5 mmol, 1.5 equiv) and filled up with toluene (*ca.* 0.6 mL). The reaction was carried out under pressure of 10–9.5 kbar at 20–25°C for 72 h. Flash column chromatography on a silica gel using hexane fraction from petroleum/DCM as an eluent afforded 168 mg (0.38 mmol; 38% yield) of (*R*)-**3f** with 94% ee.

Colorless oil (solidified upon standing); 94% ee; ^{1}H NMR (400 MHz, $CDCl_3$) δ 9.27 (d, *J* = 2.3 Hz, 1H), 8.80 (dd, *J* = 4.8, 1.7 Hz, 1H), 8.28 (dm, *J* = 8.0 Hz, 1H), 7.48 – 7.41 (m, 3H), 7.37 – 7.30 (m, 3H), 4.70 (dq, *J* = 18.6, 1.4 Hz, 1H), 4.48 (d, *J* = 18.6 Hz, 1H), 4.47 (s, 1H), 4.33 – 4.20 (m, 2H), 3.95 – 3.84 (m, 2H) 1.30 (t, *J* = 7.1 Hz, 3H), 0.93 (t, *J* = 7.1 Hz, 3H); ^{13}C NMR (100 MHz, $CDCl_3$) δ 194.4 (C=O), 166.4 (C=O), 166.0 (C=O), 153.6 (CH), 149.5 (CH), 135.4 (CH), 134.5 (C), 132.4 (C), 128.2 (CH), 128.1 (2 × CH), 127.6 (q, *J* = 1.6 Hz, 2 × CH), 126.6 (q, *J* = 287.0 Hz, CF_3), 123.5 (CH), 62.1 (CH_2), 61.8 (CH_2), 56.0 (q, *J* = 2.1 Hz, CH), 53.8 (q, *J* = 24.9 Hz, C-CF_3), 37.2 (q, *J* = 1.7 Hz, CH_2), 13.8 (CH_3), 13.4 (CH_3); ^{19}F NMR (376 MHz, $CDCl_3$) δ -63.3; HRMS (ESI) m/z: $[M + H]^+$ Calcd for $C_{22}H_{23}F_3NO_5$ 438.1523; Found 438.1524; Enantiomeric excess was determined by HPLC analysis using a Chiralpak® IC column (Hexane/*i*-PrOH: 85/15, flow rate 1.0 mL/min, λ = 219 nm): *ent*-(*R*)-major t_r = 21.2 min and *ent*-(*S*)-minor t_r = 26.3 min.

(*R*)-Diethyl 2-(1,1,1-trifluoro-4-(1-methyl-1H-imidazol-2-yl)-4-oxo-2-phenylbutan-2-yl)malonate (3g):

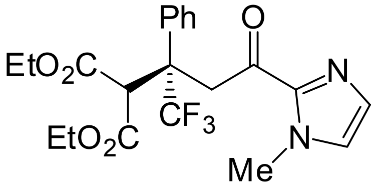

Product **3g** was prepared from enone **2g** according to general high-pressure procedure. A 1.0 mL Teflon ampoule was charged with 20.6 mg of catalyst (1*R*,2*R*)-**1a** (0.05 mmol, 5 mol%), 278 mg of enone **2g** (0.99 mmol), 243 mg of diethyl malonate (1.52 mmol, 1.5 equiv) and filled up with toluene (*ca.* 0.6 mL). The reaction was carried out under pressure of 10–9.5 kbar at 20–25°C for 72 h. Flash column chromatography on a silica gel using hexane fraction from petroleum/DCM as an eluent afforded 349 mg (0.79 mmol; 80% yield) of (*R*)-**3g** with 74.6% ee (51% yield for the reaction performed under similar conditions for 20h).

Colorless viscous oil (solidified upon standing); 74.6% ee, $[\alpha]_D^{25}$= +35.3 (c 1.04, $CHCl_3$); ^{1}H NMR (400 MHz, $CDCl_3$) δ 7.56 – 7.52 (m, 2H), 7.34 – 7.24 (m, 3H), 7.16 (d, *J* = 0.9 Hz, 1H), 7.03 (d, *J* = 0.5 Hz, 1H), 4.84 (dq, *J* = 18.5, 1.0 Hz, 1H), 4.57 (s, 1H), 4.54 (d, *J* = 18.5 Hz, 1H), 4.28 – 4.20 (m, 2H), 3.98 – 3.91 (m, 2H), 3.91 (s, 3H), 1.28 (t, *J* = 7.1 Hz, 3H), 0.98 (t, *J* = 7.1 Hz, 3H); ^{13}C NMR (100 MHz, $CDCl_3$) δ 188.1 (C=O), 166.4 (C=O), 166.1 (C=O), 142.9 (C), 135.1 (C), 129.1 (CH), 127.9 (2 × CH, 2 × CH, CH), 127.2 (CH), 126.7 (q, *J* = 286.9 Hz, CF_3), 61.8 (CH_2), 61.5 (CH_2), 55.5 (q, *J* = 1.8 Hz, CH), 53.6 (q, *J* = 24.8 Hz, C-CF_3), 37.5 (q, *J* = 1.6 Hz, CH_2), 36.1 (CH_3), 13.8 (CH_3), 13.5 (CH_3); ^{19}F NMR (376 MHz, $CDCl_3$) δ -64.3; HRMS (ESI) m/z: $[M + H]^+$ Calcd for $C_{21}H_{24}F_3N_2O_5$ 441.1632; Found 441.1632; Enantiomeric excess was determined by HPLC analysis using a Chiralpak® IC column (Hexane/*i*-PrOH: 95/5, flow rate 1.0 mL/min, λ = 219 nm): *ent*-(*R*)-major t_r = 11.2 min and *ent*-(*S*)-minor t_r = 14.5 min.

(*R*)-Diethyl 2-(1,1,1-trifluoro-4-oxo-2-phenyl-4-(thiazol-2-yl)butan-2-yl)malonate (3h):

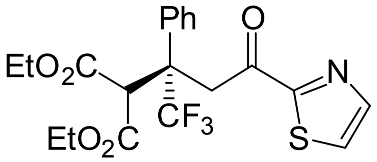

Product **3h** was prepared from enone **2h** according to general high-pressure procedure.

(Table 2, entry 4): A 2.0 mL Teflon ampoule was charged with 45.3 mg of catalyst (1*R*,2*R*)-**1b** (0.1 mmol, 5 mol%), 570 mg of enone **2h** (2.01 mmol), 485 mg of diethyl malonate (3.03 mmol, 1.5 equiv) and filled up with toluene (*ca.* 1.2 mL). The reaction was carried out under pressure of 9.5–9.0 kbar at 20–25°C for 20 h. Flash column chromatography on a silica gel using hexane fraction from petroleum/DCM as an eluent afforded 802 mg (1.81 mmol; 90% yield) of (*R*)-**3h** with 93% ee (86% yield and 75% ee with Takemoto catalyst (**1a**); see Scheme 2).

Scale-up synthesis: A 5 mL Teflon ampoule was charged with catalyst (1*R*,2*R*)-**1b** (45.5 mg, 2 mol%) and enone **2h** (1.45 g, 5.12 mmol), followed by diethyl malonate addition (1.21 g, 7.55 mmol, 1.5 equiv). Then, the Teflon ampoule was filled up with toluene, closed and placed in a high-pressure chamber with inert liquid (hexane). The pressure was slowly increased to 9.5-9 kbar at ambient temperature by hexane compression, and the reaction mixture was kept under these conditions for 20 h. After decompression, the reaction mixture was filtered through a short silica gel column with DCM. The solvent was evaporated, and the residue was crystallized from ethanol to give 1.65 g of (*R*)-**3h** (73% yield, 98% ee) as white crystals.

3h: Initially colorless viscous oil (solidified upon standing); white crystals (crystallized from EtOH); mp. 79.5-80 °C; 93-94% ee, 98% ee after crystallization, $[\alpha]_D^{22}$= –46.7 (c 1.10, $CHCl_3$); ^{1}H NMR (400 MHz, $CDCl_3$) δ 8.05 (d, *J* = 3.0 Hz, 1H), 7.69 (d, *J* = 3.0 Hz, 1H), 7.54 – 7.48 (m, 2H), 7.36 – 7.28 (m, 3H), 4.91 (dq, *J* = 18.8, 1.0 Hz, 1H), 4.61 (d, *J* = 18.8 Hz, 1H), 4.54 (s, 1H), 4.31 – 4.20 (m, 2H), 3.99 – 3.88 (m, 2H), 1.29 (t, *J* = 7.1 Hz, 3H), 0.97 (t, *J* = 7.1 Hz, 3H); ^{13}C NMR (100 MHz, $CDCl_3$) δ 189.3 (C=O), 167.0 (C), 166.4 (C=O), 166.1 (C=O), 144.8 (CH), 134.8 (C), 128.2 (CH), 128.1 (2 × CH), 127.8 (q, *J* = 1.7 Hz, 2 × CH), 126.7 (q, *J* = 286.8 Hz, CF_3), 126.6 (CH), 62.0 (CH_2), 61.7 (CH_2), 55.7 (q, *J* = 1.8 Hz, CH), 53.7 (q, *J* = 25.3 Hz, <u>C</u>-CF_3), 37.2 (q, *J* = 1.9 Hz, CH_2), 13.8 (CH_3), 13.5 (CH_3); ^{19}F NMR (376 MHz, $CDCl_3$) δ -64.2; HRMS (ESI) m/z: $[M + H]^+$ Calcd for $C_{20}H_{21}F_3NO_5S$ 444.1087; Found 444.1085; Enantiomeric excess was determined by HPLC analysis using a Chiralpak® IC column (Hexane/*i*-PrOH: 90/10, flow rate 1.0 mL/min, λ = 219 nm): *ent*-(*S*)-minor t_r = 9.6 min and *ent*-(*R*)-major t_r = 14.2 min.

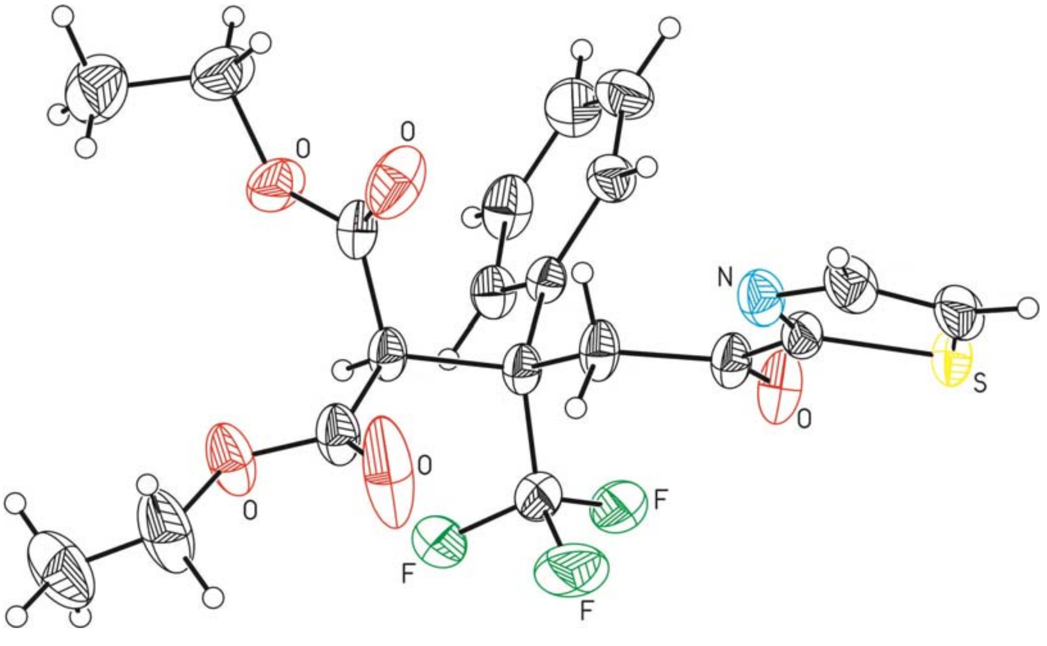

ORTEP of compound (***R***)-**3h** (CCDC Deposition Number 2033588)

(*R*)-Diethyl 2-(4-(4,5-dimethylthiazol-2-yl)-1,1,1-trifluoro-4-oxo-2-phenylbutan-2-yl)malonate (3i):

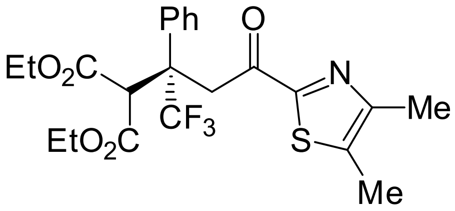

Product **3i** was prepared from enone **2i** according to general high-pressure procedure. A 2.0 mL Teflon ampoule was charged with 20.8 mg of catalyst (1*R*,2*R*)-**1a** (0.05 mmol, 5 mol%), 315 mg of enone **2i** (1.01 mmol), 244 mg of diethyl malonate (1.5 mmol, 1.5 equiv) and filled up with toluene (*ca.* 1.5 mL). The reaction was carried out under pressure of 10–9.5 kbar at 20–25°C for 20 h. Flash column chromatography on a silica gel using hexane fraction from petroleum/DCM as an eluent afforded 356 mg of **3i** (0.75 mmol; 75% yield) with 95% ee.

Light yellow oil; 95.1% ee, $[\alpha]_D^{22}$= –16.0 (c 1.03, $CHCl_3$); ^{1}H NMR (400 MHz, $CDCl_3$) δ 7.57 – 7.48 (m, 2H), 7.37 – 7.25 (m, 3H), 4.76 (d, *J* = 18.5 Hz, 1H), 4.58 (s, 1H), 4.48 (d, *J* = 18.5 Hz, 1H), 4.25 (q, *J* = 7.0 Hz, 2H), 3.94 (q, *J* = 7.1 Hz, 2H), 2.43 (s, 3H), 2.41 (s, 3H), 1.29 (t, *J* = 7.1 Hz, 3H), 0.98 (t, *J* = 7.1 Hz, 3H); ^{13}C NMR (100 MHz, $CDCl_3$) δ 189.1 (C=O), 166.5 (C=O), 166.2 (C=O), 161.6 (C), 151.5 (C), 136.4 (C), 135.0 (C), 128.01 (CH), 127.99 (2 × CH), 127.9 (q, *J* = 1.5 Hz, 2 × CH), 126.7 (q, *J* = 286.8 Hz, CF_3), 61.9 (CH_2), 61.6 (CH_2), 55.5 (q, *J* = 1.3 Hz, CH), 53.7 (q, *J* = 25.0 Hz, <u>C</u>-CF_3), 36.9 (q, *J* = 1.7 Hz, CH_2), 14.9 (CH_3), 13.8 (CH_3), 13.5 (CH_3), 12.1 (CH_3); ^{19}F NMR (376 MHz, $CDCl_3$) δ -64.3; HRMS (ESI) m/z: $[M + H]^+$ Calcd for $C_{22}H_{25}F_3NO_5S$ 472.1400; Found 472.1402; Enantiomeric excess was determined by HPLC analysis using a Chiralpak® IA column (Hexane/*i*-PrOH: 98/2, flow rate 1.0 mL/min, λ = 219 nm): *ent*-(*R*)-major t_r = 17.8 min and *ent*-(*S*)-minor t_r = 19.8 min.

(*R*)-Diethyl 2-(4-(benzo[d]thiazol-2-yl)-1,1,1-trifluoro-4-oxo-2-phenylbutan-2-yl)malonate (3j):

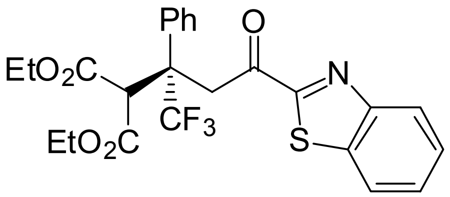

Product **3j** was prepared from enone **2j** according to general high-pressure procedure. A 0.9 mL Teflon ampoule was charged with 9.05 mg of catalyst (1*R*,2*R*)-**1a** (0.022 mmol, 5 mol%), 150 mg of enone **2j** (0.45 mmol), 106 mg of diethyl malonate (0.662 mmol, 1.5 equiv) and filled up with toluene (*ca.* 0.6 mL). The reaction was carried out under pressure of 10–9.5 kbar at 20–25°C for 20 h. Flash column chromatographyon a silica gel using hexane fraction from petroleum/DCM as an eluent afforded 189 mg (0.38 mmol; 85% yield, 98% conversion) of (*R*)-**3j** with 91% ee).

Colorless viscous oil (solidified upon standing); 91% ee, $[\alpha]_D^{22}$= –23.3 (c 1.03, $CHCl_3$); ^{1}H NMR (400 MHz, $CDCl_3$) δ 8.24 (d, *J* = 8.3 Hz, 1H), 7.97 (d, *J* = 7.8 Hz, 1H), 7.62 – 7.49 (m, 4H), 7.39 – 7.28 (m, 3H), 5.03 (d, *J* = 18.9 Hz, 1H), 4.72 (d, *J* = 18.9 Hz, 1H), 4.58 (s, 1H), 4.34 – 4.22 (m, 2H), 4.01 – 3.90 (m, 2H), 1.32 (t, *J* = 7.1 Hz, 3H), 0.99 (t, *J* = 7.1 Hz, 3H); ^{13}C NMR (100 MHz, $CDCl_3$) δ 190.8 (C=O), 166.4 (C=O), 166.2 (C), 166.1 (C=O), 153.4 (C), 137.5 (C), 134.7 (C), 128.2 (CH), 128.1 (2 × CH), 127.8 (q, *J* = 1.5 Hz, 2 × CH), 127.7 (CH), 126.9 (CH), 126.6 (q, *J* = 287.0 Hz, CF_3), 125.7 (CH), 122.3 (CH), 62.0 (CH_2), 61.7 (CH_2), 55.7 (q, *J* = 1.5 Hz, CH), 53.8 (q, *J* = 25.1 Hz, <u>C</u>-CF_3), 37.4 (q, *J* = 1.6 Hz, CH_2), 13.8 (CH_3), 13.5 (CH_3); ^{19}F NMR (376 MHz, $CDCl_3$) δ -64.1; HRMS (ESI) m/z: $[M + H]^+$ Calcd for $C_{24}H_{23}F_3NO_5S$ 494.1244; Found 494.1246; Enantiomeric excess was determined by HPLC analysis using a Chiralpak® IC column (Hexane/*i*-PrOH: 95/5, flow rate 1.0 mL/min, λ = 219 nm): with catalyst (1*R*,2*R*)-**1a** *ent*-(*S*)-minor t_r = 8.1 min and *ent*-(*R*)-major t_r = 12.9 min.

(*R*)-Diethyl 2-(4-(benzo[d]oxazol-2-yl)-1,1,1-trifluoro-4-oxo-2-phenylbutan-2-yl)malonate (3k):

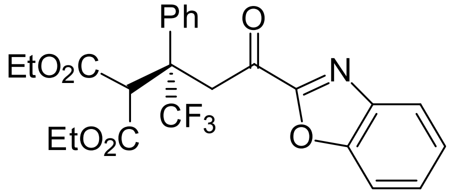

Product **3k** was prepared from enone **2k** according to general high-pressure procedure. A 1.2 mL Teflon ampoule was charged with 14.2 mg of catalyst (1*R*,2*R*)-**1a** (0.034 mmol, 5 mol%), 223 mg of enone **2k** (0.70 mmol), 169 mg of diethyl malonate (1.056 mmol, 1.5 equiv) and filled up with toluene (*ca.* 0.8 mL). The reaction was carried out under pressure of 10–9.5 kbar at 20–25°C for 20 h. Flash column chromatography on a silica gel using hexane fraction from petroleum/DCM as an eluent afforded 275 mg of (*R*)-**7b** (0.58 mmol, 82% yield, 98% conversion) with 91% ee.

Colorless viscous oil (solidified upon standing); 91.1% ee, $[\alpha]_D^{22}= -19.9$ (c 0.99, $CHCl_3$); ^1H NMR (400 MHz, $CDCl_3$) δ 7.93 (dm, *J* = 7.9 Hz, 1H), 7.65 (dm, *J* = 8.2 Hz, 1H), 7.57 – 7.44 (m, 4H), 7.37 – 7.29 (m, 3H), 4.95 (d, *J* = 18.9 Hz, 1H), 4.67 (d, *J* = 18.9 Hz, 1H), 4.55 (s, 1H), 4.33 – 4.21 (m, 2H), 3.95 (q, *J* = 7.1 Hz, 2H), 1.31 (t, *J* = 7.1 Hz, 3H), 0.98 (t, *J* = 7.1 Hz, 3H); ^{13}C NMR (100 MHz, $CDCl_3$) δ 185.2 (C=O), 166.4 (C=O), 166.0 (C=O), 157.1 (C), 150.9 (C), 140.5 (C), 134.4 (C), 128.6 (CH), 128.3 (CH), 128.2 (2 × CH), 127.8 (q, *J* = 1.6 Hz, 2 × CH), 126.6 (q, *J* = 286.4 Hz, CF_3), 125.8 (CH), 122.5 (CH), 111.9 (CH), 62.1 (CH_2), 61.8 (CH_2), 55.4 (q, *J* = 1.6 Hz, CH), 53.7 (q, *J* = 25.2 Hz, <u>C</u>-CF_3), 38.2 (q, *J* = 1.5 Hz, CH_2), 13.9 (CH_3), 13.5 (CH_3); ^{19}F NMR (376 MHz, $CDCl_3$) δ -64.5; HRMS (ESI) m/z: $[M + H]^+$ Calcd for $C_{24}H_{23}F_3NO_6$ 478.1472; Found 478.1479; Enantiomeric excess was determined by HPLC analysis using a Chiralpak® IA column (Hexane/*i*-PrOH: 93/7, flow rate 1.0 mL/min, λ = 219 nm): *ent*-(*R*)-major t_r = 12.8 min and *ent*-(*S*)-minor t_r = 13.9 min.

(*R*)-Dimethyl 2-(1,1,1-trifluoro-4-oxo-2-phenyl-4-(thiazol-2-yl)butan-2-yl)malonate (3l):

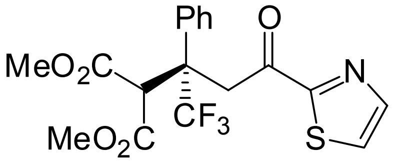

Product **3l** was prepared from enone **2h** according to general high-pressure procedure (Table 2, entry 5).

A 1.2 mL Teflon ampoule was charged with 27.3 mg of catalyst (1*R*,2*R*)-**1b** (0.06 mmol, 5 mol%), 341 mg of enone **2h** (1.20 mmol), 240 mg of dimethyl malonate (1.81 mmol, 1.5 equiv) and filled up with toluene (*ca.* 0.7 mL). The reaction was carried out under pressure of 9.5–9.0 kbar at 20–25°C for 20 h. Flash column chromatography on a silica gel using hexane fraction from petroleum/DCM as an eluent afforded 401 mg (0.96 mmol; 80% yield, 86% NMR yield) of (*R*)-**3l** with 87% ee.

colorless viscous oil (solidified upon long standing); 80% yield, 87.0% ee, $[\alpha]_D^{25}= -46.5$ (c 1.20, $CHCl_3$) $[\alpha]_D^{22}= -46.5$ (c 1.2, $CHCl_3$); ^1H NMR (400 MHz, $CDCl_3$) δ 8.05 (d, *J* = 3.0 Hz, 1H), 7.69 (d, *J* = 3.1 Hz, 1H), 7.54 – 7.47 (m, 2H), 7.37 – 7.28 (m, 3H), 4.89 (dq, *J* = 18.7, 1.0 Hz, 1H), 4.60 (s, 1H), 4.58 (d, *J* = 18.8 Hz, 1H), 3.79 (s, 3H), 3.48 (s, 3H); ^{13}C NMR (100 MHz, $CDCl_3$) δ 189.1 (C=O), 166.7 (C=O + C), 166.3 (C=O), 144.8 (CH), 134.4 (C), 128.2 (CH), 128.1 (2 × CH), 127.6 (q, *J* = 1.6 Hz, 2 ×CH), 126.7 (CH), 126.5 (q, *J* = 286.7 Hz, CF_3), 54.9 (q, *J* = 1.8 Hz, CH), 53.6 (q, *J* = 24.9 Hz, <u>C</u>-CF_3), 52.9 (CH_3), 52.6 (CH_3), 37.0 (q, *J* = 1.8 Hz, CH_2); ^{19}F NMR (376 MHz, $CDCl_3$) δ -64.8; HRMS (ESI) m/z: $[M + H]^+$ Calcd for $C_{18}H_{17}F_3NO_5S$ 416.0774; Found 416.0772; Enantiomeric excess was determined by HPLC analysis using a Chiralpak® IA column (Hexane/*i*-PrOH: 95/5, flow rate 1.0 mL/min, λ = 219 nm): *ent*-(*S*)-minor t_r = 13.7 min and *ent*-(*R*)-major t_r = 16.4 min.

(*R*)-Diethyl 2-(1,1,1-trifluoro-2-(4-fluorophenyl)-4-oxo-4-(thiazol-2-yl)butan-2-yl)malonate (5a):

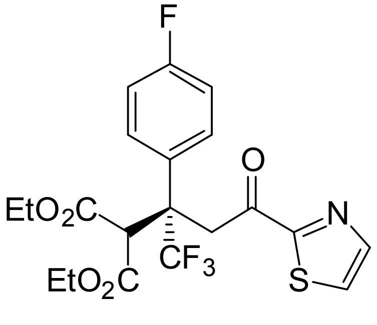

Product **5a** was prepared from enone **4a** according to general high-pressure procedure. A 1.2 mL Teflon ampoule was charged with 28.3 mg of catalyst (1*R*,2*R*)-**1b** (0.062 mmol, 5 mol%), 366.5 mg of enone **4a** (1.22 mmol), 292.0 mg of diethyl malonate (1.8 mmol, 1.5 equiv) and filled up with toluene (*ca.* 0.8 mL). The reaction was carried out under pressure of 10–9.5 kbar at 20–25°C for 20 h. Flash column chromatography on a silica gel using hexane fraction from petroleum/DCM as an eluent afforded 521 mg of (*R*)-**5a** (93% yield, 98% conversion) with 95% ee.

White solid; 95.0% ee, $[\alpha]_D^{22}$= –43.5 (c 1.09, $CHCl_3$); ^{1}H NMR (400 MHz, $CDCl_3$) δ 8.05 (d, *J* = 3.0 Hz, 1H), 7.70 (d, *J* = 3.0 Hz, 1H), 7.53 (dd, *J* = 8.9, 5.0 Hz, 2H), 7.06 – 6.99 (m, 2H), 4.87 (dq, *J* = 18.7, 1.0 Hz, 1H), 4.59 (d, *J* = 18.7 Hz, 1H), 4.52 (s, 1H), 4.31 – 4.19 (m, 2H), 4.01 – 3.94 (m, 2H), 1.29 (t, *J* = 7.1 Hz, 3H), 1.02 (t, *J* = 7.1 Hz, 3H); ^{13}C NMR (100 MHz, $CDCl_3$) δ 189.2 (C=O), 166.8 (C), 166.2 (C=O), 165.9 (C=O), 162.3 (d, *J* = 248.5 Hz, CF), 144.9 (CH), 130.5 (d, *J* = 2.7 Hz, C), 129.9 (dq, *J* = 8.2, 1.7 Hz, 2 × CH), 126.8 (CH), 126.5 (q, *J* = 287.0 Hz, CF_3), 115.0 (d, *J* = 21.4 Hz, 2 × CH), 62.1 (CH_2), 61.8 (CH_2), 55.4 (q, *J* = 0.8 Hz, CH), 53.2 (q, *J* = 25.1 Hz, C-CF_3), 37.2 (q, *J* = 1.6 Hz, CH_2), 13.8 (CH_3), 13.6 (CH_3); ^{19}F NMR (376 MHz, $CDCl_3$) δ -65.0 (s, 3F), -113.9 – -114.0 (m, 1F); HRMS (ESI) m/z: $[M + H]^+$ Calcd for $C_{20}H_{20}F_4NO_5S$ 462.0993; Found 462.0987; Enantiomeric excess was determined by HPLC analysis using a Chiralpak® IC column (Hexane/*i*-PrOH: 90/10, flow rate 1.0 mL/min, λ = 219 nm): *ent*-(*S*)-minor t_r = 7.2 min and *ent*-(*R*)-major t_r = 9.2 min.

(*R*)-Diethyl 2-(2-(4-chlorophenyl)-1,1,1-trifluoro-4-oxo-4-(thiazol-2-yl)butan-2-yl)malonate (5b):

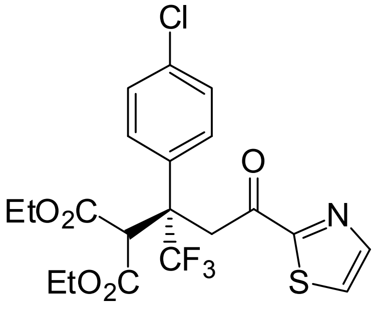

Product **5b** was prepared from enone **4b** according to general high-pressure procedure. A 1.2 mL Teflon ampoule was charged with 28.1 mg of catalyst (1*R*,2*R*)-**1b** (0.062 mmol, 5 mol%), 383 mg of enone **4b** (1.21 mmol), 295 mg of diethyl malonate (1.8 mmol, 1.5 equiv) and filled up with toluene (*ca.* 0.8 mL). The reaction was carried out under pressure of 10–9.5 kbar at 20–25°C for 20 h. Flash column chromatography on a silica gel using hexane fraction from petroleum/DCM as an eluent afforded 510 mg of (*R*)-**5b** (88% yield, 99% conversion) with 89% ee.

Colorless viscous oil; 89.2% ee, $[\alpha]_D^{25}$= –45.8 (c 1.30, $CHCl_3$); ^{1}H NMR (400 MHz, $CDCl_3$) δ 8.04 (d, *J* = 3.0 Hz, 1H), 7.70 (d, *J* = 3.0 Hz, 1H), 7.48 (d, *J* = 8.7 Hz, 2H), 7.31 (dm, *J* = 9.0 Hz, 2H), 4.86 (dq, *J* = 18.7, 0.9 Hz, 1H), 4.58 (d, *J* = 18.7 Hz, 1H), 4.52 (s, 1H), 4.31 – 4.20 (m, 2H), 4.04 – 3.93 (m, 2H), 1.29 (t, *J* = 7.1 Hz, 3H), 1.03 (t, *J* = 7.1 Hz, 3H); ^{13}C NMR (100 MHz, $CDCl_3$) δ 189.1 (C=O), 166.8 (C), 166.2 (C=O), 165.9 (C=O), 144.9 (CH), 134.3 (C), 133.3 (C), 129.5 (q, *J* = 1.7 Hz, 2 × CH), 128.2 (2 × CH), 126.8 (CH), 126.4 (q, *J* = 287.1 Hz, CF_3), 62.1 (CH_2), 61.9 (CH_2), 55.2 (q, *J* = 1.5 Hz, CH), 53.3 (q, *J* = 25.1 Hz, C-CF_3), 37.2 (q, *J* = 1.6 Hz, CH_2), 13.8 (CH_3), 13.6 (CH_3); ^{19}F NMR (376 MHz, $CDCl_3$) δ -64.8; HRMS (ESI) m/z: $[M + H]^+$ Calcd for $C_{20}H_{20}ClF_3NO_5S$ 478.0697; Found 478.0692; Enantiomeric excess was determined by HPLC analysis using a Chiralpak® IC column (Hexane/*i*-PrOH: 90/10, flow rate 1.0 mL/min, λ = 219 nm): *ent*-(*S*)-minor t_r = 6.7 min and *ent*-(*R*)-major t_r = 8.7 min.

(*R*)-Diethyl 2-(1,1,1-trifluoro-4-oxo-4-(thiazol-2-yl)-2-(*p*-tolyl)butan-2-yl)malonate (5c):

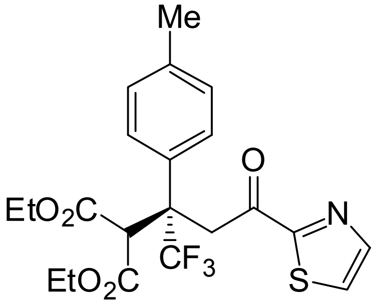

Product **5c** was prepared from enone **4c** according to general high-pressure procedure. A 1.2 mL Teflon ampoule was charged with 27.3 mg of catalyst (1*R*,2*R*)-**1a** (0.06 mmol, 5 mol%), 360 mg of enone **4c** (1.21 mmol), 297 mg of diethyl malonate (1.85 mmol, 1.5 equiv) and filled up with toluene (*ca.* 0.8 mL). The reaction was carried out under pressure of 10–9.5 kbar at 20–25°C for 20h. Flash column chromatography on a silica gel using hexane fraction from petroleum/DCM as an eluent afforded 519 mg of (*R*)-**5c** (94% yield, 98% conversion) with 91% ee.

Colorless viscous oil (solidified upon long standing); 91.1% ee, $[\alpha]_D^{25}$= –41.5 (c 1.17, $CHCl_3$); ^{1}H NMR (400 MHz, $CDCl_3$) δ 8.04 (d, *J* = 3.1 Hz, 1H), 7.68 (d, *J* = 3.1 Hz, 1H), 7.39 (d, *J* = 8.2 Hz, 2H), 7.13 (d, *J* = 8.1 Hz, 2H), 4.89 (dq, *J* = 18.7, 1.1 Hz, 1H), 4.58 (d, *J* = 18.7 Hz, 1H), 4.54 (s, 1H), 4.31 – 4.20 (m, 2H), 4.01 – 3.91 (m, 2H), 2.31 (s, 3H), 1.29 (t, *J* = 7.1 Hz, 3H), 1.00 (t, *J* = 7.1 Hz, 3H); ^{13}C NMR (100 MHz, $CDCl_3$) δ 189.3 (C=O), 167.0 (C), 166.4 (C=O), 166.1 (C), 144.8 (CH), 137.9 (C), 131.6 (C), 128.8 (2 × CH), 127.7 (q, *J* = 1.5 Hz, 2 × CH), 126.7 (q, *J* = 286.8 Hz, CF_3), 126.6 (CH), 61.9 (CH_2), 61.7 (CH_2), 55.5 (q, *J* = 1.6 Hz, CH), 53.4 (q, *J* = 24.9 Hz, C-CF_3), 37.2 (q, *J* = 1.7 Hz, CH_2), 20.9 (CH_3), 13.8 (CH_3), 13.5 (CH_3); ^{19}F NMR (376 MHz, $CDCl_3$) δ -64.5 (s); HRMS (ESI) m/z: $[M + H]^+$ Calcd for $C_{21}H_{23}F_3NO_5S$ 458.1244; Found 458.1237; Enantiomeric excess was determined by HPLC analysis using a Chiralpak® IC column (Hexane/*i*-PrOH: 90/10, flow rate 1.0 mL/min, λ = 219 nm): *ent*-(*S*)-minor t_r = 10.8 min and *ent*-(*R*)-major t_r = 14.0 min.

(*R*)-Diethyl 2-(1,1,1-trifluoro-4-oxo-4-(thiazol-2-yl)-2-(4-(trifluoromethyl)phenyl)butan-2-yl)malonate (5d):

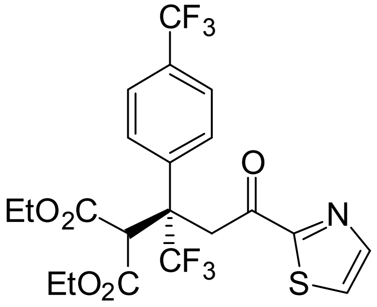

Product **5d** was prepared from enone **4d** according to general high-pressure procedure. A 1.2 mL Teflon ampoule was charged with 27.4 mg of catalyst (1*R*,2*R*)-**1b** (0.060 mmol, 6 mol%), 332 mg of enone **4d** (0.95 mmol), 231 mg of diethyl malonate (1.5 mmol, 1.5 equiv) and filled up with toluene (*ca.* 0.8 mL). The reaction was carried out under pressure of 10–9.5 kbar at 20–25°C for 20 h. Flash column chromatography on a silica gel using hexane fraction from petroleum/DCM as an eluent afforded 432 mg of (*R*)-**5d** (89% yield, 97% conversion) with 89% ee.

Colorless viscous oil (solidified upon long standing); 88.7% ee; $[\alpha]_D^{25}$= –40.1 (c 1.03, $CHCl_3$); ^{1}H NMR (400 MHz, $CDCl_3$) δ 8.06 (d, *J* = 3.0 Hz, 1H), 7.72 (d, *J* = 3.0 Hz, 1H), 7.68 (d, *J* = 8.5 Hz, 2H), 7.61 (d, *J* = 8.6 Hz, 2H), 4.90 (d, *J* = 18.8 Hz, 1H), 4.64 (d, *J* = 18.8 Hz, 1H), 4.55 (s, 1H), 4.32 – 4.21 (m, 2H), 3.97 (q, *J* = 7.1 Hz, 2H), 1.30 (t, *J* = 7.1 Hz, 3H), 0.98 (t, *J* = 7.1 Hz, 3H); ^{13}C NMR (100 MHz, $CDCl_3$) δ 189.0 (C=O), 166.6 (C), 166.1 (C=O), 165.8 (C=O), 144.9 (CH), 138.8 (C), 130.3 (q, *J* = 32.7 Hz, C-CF_3), 128.6 (q, *J* = 1.7 Hz, 2 × CH), 126.9 (CH), 126.3 (q, *J* = 287 Hz, CF_3), 125.0 (q, *J* = 3.7 Hz, 2 × CH), 123.8 (q, *J* = 272.2 Hz, CF_3), 62.2 (CH_2), 61.9 (CH_2), 55.1 (q, *J* = 1.6 Hz, CH), 53.7 (q, *J* = 25.1 Hz, C-CF_3), 37.2 (q, *J* = 1.6 Hz, CH_2), 13.8 (CH_3), 13.4 (CH_3); ^{19}F NMR (376 MHz, $CDCl_3$) δ -62.8 (s, 3F), -64.6 (s, 3F); HRMS (ESI) m/z: $[M + H]^+$ Calcd for $C_{21}H_{20}F_6NO_5S$ 512.0961; Found 512.0959; Enantiomeric excess was determined by HPLC analysis using a Chiralpak® IC column (Hexane/*i*-PrOH: 98/2, flow rate 1.0 mL/min, λ = 219 nm): *ent*-(*S*)-minor t_r = 8.4 min and *ent*-(*R*)-major t_r = 11.0 min.

(*R*)-Diethyl 2-(1,1,1-trifluoro-2-(4-methoxyphenyl)-4-oxo-4-(thiazol-2-yl)butan-2-yl)malonate (5e):

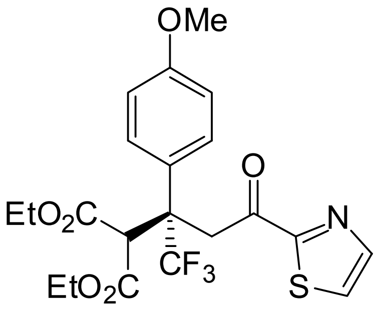

Product **5e** was prepared from enone **4e** according to general high-pressure procedure. A 0.9 mL Teflon ampoule was charged with 13.7 mg of catalyst (1*R*,2*R*)-**1b** (0.03 mmol, 5 mol%), 190 mg of enone **4e** (0.61 mmol), 150 mg of diethyl malonate (0.94 mmol, 1.5 equiv) and filled up with toluene (*ca.* 0.6 mL). The reaction was carried out under pressure of 10–9.5 kbar at 20–25°C for 20 h. Flash column chromatography on a silica gel using hexane fraction from petroleum/DCM as an eluent afforded 260 mg of (*R*)-**5e** (91% yield, 98% conversion) with 92% ee.

Colorless viscous oil; 92.0% ee, $[\alpha]_D^{22}$= –43.9 (c 1.4, $CHCl_3$); ^{1}H NMR (400 MHz, $CDCl_3$) δ 8.04 (d, *J* = 3.0 Hz, 1H), 7.68 (d, *J* = 3.0 Hz, 1H), 7.44 (d, *J* = 8.9 Hz, 2H), 6.85 (dm, *J* = 9.1 Hz, 2H), 4.87 (dq, *J* = 18.6, 0.9 Hz, 1H), 4.57 (d, *J* = 18.6 Hz, 1H), 4.53 (s, 1H), 4.31 – 4.20 (m, 2H), 4.03 – 3.92 (m, 2H), 3.78 (s, 3H), 1.29 (t, *J* = 7.1 Hz, 3H), 1.02 (t, *J* = 7.1 Hz, 3H); ^{13}C NMR (100 MHz, $CDCl_3$) δ 189.4 (C=O), 167.0 (C), 166.4 (C=O), 166.1 (C=O), 159.1 (C), 144.8 (CH), 129.1 (q, *J* = 1.6 Hz, 2 × CH), 126.7 (q, *J* = 286.7 Hz, CF_3), 126.6 (CH), 126.5 (C), 113.4 (2 × CH), 61.9 (CH_2), 61.7 (CH_2), 55.5 (q, *J* = 1.6 Hz, CH), 55.1 (CH_3), 53.1 (q, *J* = 24.9 Hz, C-CF_3), 37.2 (q, *J* = 1.6 Hz, CH_2), 13.8 (CH_3), 13.6 (CH_3); ^{19}F NMR (376 MHz, $CDCl_3$) δ -64.9; HRMS (ESI) m/z: $[M + H]^+$ Calcd for $C_{21}H_{23}F_3NO_6S$ 474.1193; Found 474.1199; Enantiomeric excess was determined by HPLC analysis using a Chiralpak® IC column (Hexane/*i*-PrOH: 90/10, flow rate 1.0 mL/min, λ = 219 nm): *ent*-(*S*)-minor t_r = 16.8 min and *ent*-(*R*)-major t_r= 20.9 min.

(*R*)-Diethyl 2-(2-(3-chlorophenyl)-1,1,1-trifluoro-4-oxo-4-(thiazol-2-yl)butan-2-yl)malonate (5f):

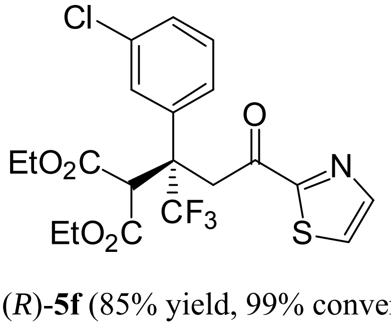

Product **5f** was prepared from enone **4f** according to general high-pressure procedure. A 1.2 mL Teflon ampoule was charged with 28.1 mg of catalyst (1*R*,2*R*)-**1b** (0.062 mmol, 5 mol%), 384 mg of enone **4f** (1.21 mmol), 287.3 mg of diethyl malonate (1.8 mmol, 1.5 equiv) and filled up with toluene (*ca.* 0.8 mL). The reaction was carried out under pressure of 10–9.5 kbar at 20–25°C for 20 h. Flash column chromatography on a silica gel using hexane fraction from petroleum/DCM as an eluent afforded 490 mg of (*R*)-**5f** (85% yield, 99% conversion) with 90% ee.

Colorless viscous oil (solidified upon long standing); 90.2% ee, $[\alpha]_D^{25}$= –49.4 (c 1.26, $CHCl_3$); ^{1}H NMR (400 MHz, $CDCl_3$) δ 8.05 (d, *J* = 3.0 Hz, 1H), 7.71 (d, *J* = 3.0 Hz, 1H), 7.56 (bs, 1H), 7.42 (d, *J* = 7.6 Hz, 1H), 7.31 (dt, *J* = 8.0, 1.6 Hz, 1H), 7.27 (dd, *J* = 8.2, 7.4 Hz, 1H), 4.85 (dq, *J* = 18.7 Hz, 1H), 4.59 (d, *J* = 18.7 Hz, 1H), 4.53 (s, 1H), 4.32 – 4.20 (m, 2H), 3.99 (q, *J* = 7.1 Hz, 2H), 1.29 (t, *J* = 7.1 Hz, 3H), 1.02 (t, *J* = 7.1 Hz, 3H); ^{13}C NMR (100 MHz, $CDCl_3$) δ 189.0 (C=O), 166.7 (C), 166.1 (C=O), 165.8 (C=O), 144.9 (CH), 136.8 (C), 134.1 (C), 129.2 (CH), 128.37 (CH), 128.35 (q, CH), 126.8 (CH), 126.3 (q, *J* = 286.8 Hz, CF_3), 126.2 (q, *J* = 1.6 Hz, CH), 62.1 (CH_2), 61.9 (CH_2), 55.1 (q, *J* = 1.6 Hz, CH), 53.5 (q, *J* = 25.2 Hz, C-CF_3), 37.2 (q, *J* = 1.7 Hz, CH_2), 13.8 (CH_3), 13.5 (CH_3); ^{19}F NMR (376 MHz, $CDCl_3$) δ -64.7; HRMS (ESI) m/z: $[M + H]^+$ Calcd for $C_{20}H_{20}ClF_3NO_5S$ 478.0697; Found 478.0692; Enantiomeric excess was determined by HPLC analysis using a Chiralpak® IC column (Hexane/*i*-PrOH: 90/10, flow rate 1.0 mL/min, λ = 219 nm): *ent*-(*S*)-minor t_r = 7.3 min and *ent*-(*R*)-major t_r = 9.2 min.

(*R*)-Diethyl 2-(1,1,1-trifluoro-4-oxo-4-(thiazol-2-yl)-2-(*m*-tolyl)butan-2-yl)malonate (5g):

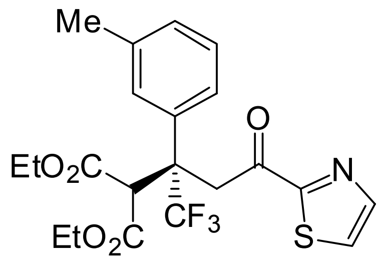

Product **5g** was prepared from enone **4g** according to general high-pressure procedure. A 1.2 mL Teflon ampoule was charged with 27.9 mg of catalyst (1*R*,2*R*)-**1b** (0.061 mmol, 5 mol%), 350 mg of enone **4g** (1.18 mmol), 290 mg of diethyl malonate (1.81 mmol, 1.5 equiv) and filled up with toluene (*ca.* 0.8 mL). The reaction was carried out under pressure of 10–9.5 kbar at 20–25°C for 20 h. Flash column chromatography on a silica gel using hexane fraction from petroleum/DCM as an eluent afforded 481 mg of (*R*)-**5g** (89% yield, 94% conversion) with 93% ee.

White solid; 92.7% ee, $[\alpha]_D^{24}$= –43.8 (c 1.28, $CHCl_3$); ^{1}H NMR (400 MHz, $CDCl_3$) δ 8.04 (d, *J* = 3.0 Hz, 1H), 7.68 (d, *J* = 3.0 Hz, 1H), 7.32 (bs, 1H), 7.29 (bd, *J* = 8.0 Hz, 1H), 7.20 (t, *J* = 7.7 Hz, 1H), 7.11 (bd, *J* = 7.4 Hz, 1H), 4.90 (dq, *J* = 18.7, 1.2 Hz, 1H), 4.59 (d, *J* = 18.7 Hz, 1H), 4.54 (s, 1H), 4.31 – 4.20 (m, 2H), 3.95 (q, *J* = 7.1 Hz, 2H), 2.32 (s, 3H), 1.29 (t, *J* = 7.1 Hz, 3H), 0.98 (t, *J* = 7.1 Hz, 3H); ^{13}C NMR (100 MHz, $CDCl_3$) δ 189.3 (C=O), 167.0 (C), 166.4 (C=O), 166.1 (C=O), 144.8 (CH), 137.6 (C), 134.7 (C), 128.9 (CH), 128.4 (q, *J* = 1.6 Hz, CH), 127.9 (CH), 126.6 (q, *J* = 286.7 Hz, CF_3), 126.58 (CH), 124.9 (q, *J* = 1.5 Hz, CH), 62.0 (CH_2), 61.6 (CH_2), 55.7 (q, *J* = 1.8 Hz, CH), 53.6 (q, *J* = 24.8 Hz, C-CF_3), 37.2 (q, *J* = 1.7 Hz, CH_2), 21.6 (CH_3), 13.8 (CH_3), 13.5 (CH_3); ^{19}F NMR (376 MHz, $CDCl_3$) δ -64.0 (s); HRMS (ESI) m/z: $[M + H]^+$ Calcd for $C_{21}H_{23}F_3NO_5S$ 458.1244; Found 458.1237; Enantiomeric excess was determined by HPLC analysis using a Chiralpak® IC column (Hexane/*i*-PrOH: 90/10, flow rate 1.0 mL/min, λ = 219 nm): *ent*-(*S*)-minor t_r = 9.2 min and *ent*-(*R*)-major t_r = 14.5 min

(*R*)-Diethyl 2-(1,1,1-trifluoro-2-(3-methoxyphenyl)-4-oxo-4-(thiazol-2-yl)butan-2-yl)malonate (5h):

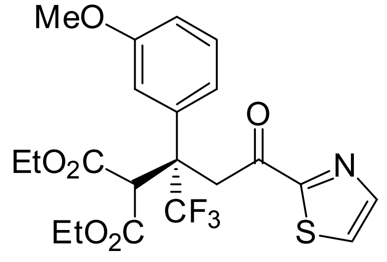

Product **5h** was prepared from enone **4h** according to general high-pressure procedure. A 1.2 mL Teflon ampoule was charged with 22.3 mg of catalyst (1*R*,2*R*)-**1b** (0.049 mmol, 5 mol%), 315 mg of enone **4h** (1.00 mmol), 238 mg of diethyl malonate (1.5 mmol, 1.5 equiv) and filled up with toluene (*ca.* 0.8 mL). The reaction was carried out under pressure of 10–9.5 kbar at 20–25°C for 20 h. Flash column chromatography on a silica gel using hexane fraction from petroleum/DCM as an eluent afforded 390 mg of (*R*)-**5h** (82% yield, 94% conversion) with 93% ee.

White solid; 93.0% ee, $[\alpha]_D^{24}$= –41.3 (c 1.62, $CHCl_3$); ^{1}H NMR (400 MHz, $CDCl_3$) δ 8.04 (d, *J* = 3.1 Hz, 1H), 7.69 (d, *J* = 3.1 Hz, 1H), 7.24 (t, *J* = 8.1 Hz, 1H), 7.11 (bs, 1H), 7.09 (d, *J* = 8.0 Hz, 1H), 6.84 (ddd, *J* = 8.2, 2.4, 0.6 Hz, 1H), 4.90 (dq, *J* = 18.7, 1.0 Hz, 1H), 4.57 (d, *J* = 18.6 Hz, 1H), 4.55 (s, 1H), 4.31 – 4.20 (m, 2H), 3.97 (q, *J* = 7.1 Hz, 2H), 3.74 (s, 3H), 1.29 (t, *J* = 7.1 Hz, 3H), 1.01 (t, *J* = 7.1 Hz, 3H); ^{13}C NMR (100 MHz, $CDCl_3$) δ 189.2 (C=O), 167.0 (C), 166.3 (C=O), 166.0 (C=O), 159.2 (C), 144.8 (CH), 136.3 (C), 129.0 (CH), 126.7 (CH), 126.5 (q, *J* = 286.9 Hz, CF_3), 120.1 (q, *J* = 1.6 Hz, CH), 114.5 (q, *J* = 1.6 Hz, CH), 113.1 (CH), 62.0 (CH_2), 61.7 (CH_2), 55.6 (q, *J* = 1.7 Hz, CH), 55.1 (CH_3), 53.7 (q, *J* = 25.2 Hz, C-CF_3), 37.3 (q, *J* = 1.6 Hz, CH_2), 13.8 (CH_3), 13.5 (CH_3); ^{19}F NMR (376 MHz, $CDCl_3$) δ -64.1; HRMS (ESI) m/z: $[M + H]^+$ Calcd for $C_{21}H_{23}F_3NO_6S$ 474.1193; Found 474.1186; Enantiomeric excess was determined by HPLC analysis using a Chiralpak® IC column (Hexane/*i*-PrOH: 90/10, flow rate 1.0 mL/min, λ = 219 nm): *ent*-(*R*)-major t_r = 17.2 min and *ent*-(*S*)-minor t_r = 19.6 min.

(*R*)-Diethyl 2-(2-(3,5-dichlorophenyl)-1,1,1-trifluoro-4-oxo-4-(thiazol-2-yl)butan-2-yl)malonate (5i):

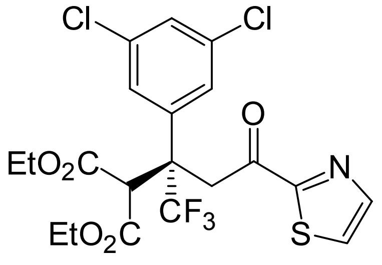

Product **5i** was prepared from enone **4i** according to general high-pressure procedure. A 0.9 mL Teflon ampoule was charged with 20.6 mg of catalyst (1*R*,2*R*)-**1b** (0.045 mmol, 5 mol%), 334 mg of enone **4i** (0.95 mmol), 236 mg of diethyl malonate (1.5 mmol, 1.5 equiv) and filled up with toluene (*ca.* 0.6 mL). The reaction was carried out under pressure of 10–9.5 kbar at 20–25°C for 20 h. Flash column chromatography on a silica gel using hexane fraction from petroleum/DCM as an eluent afforded 410 mg of (*R*)-**5i** (84% yield, 99% conversion) with 92% ee.

Colorless viscous oil; 91.9% ee, $[\alpha]_D^{22}$= –42.7 (c 1.12, $CHCl_3$); ^{1}H NMR (400 MHz, $CDCl_3$) δ 8.06 (d, *J* = 3.0 Hz, 1H), 7.72 (d, *J* = 3.0 Hz, 1H), 7.47 (d, *J* = 1.1 Hz, 2H), 7.34 (t, *J* = 1.7 Hz, 1H), 4.78 (dq, *J* = 18.5, 0.8 Hz, 1H), 4.55 (d, *J* = 18.5 Hz, 1H), 4.51 (s, 1H), 4.32 – 4.20 (m, 2H), 4.04 (q, *J* = 7.1 Hz, 2H), 1.30 (t, *J* = 7.1 Hz, 3H), 1.07 (t, *J* = 7.1 Hz, 3H); ^{13}C NMR (100 MHz, $CDCl_3$) δ 188.7 (C=O), 166.4 (C), 165.9 (C=O), 165.6 (C=O), 144.9 (CH), 138.2 (C), 134.6 (2 × C), 128.4 (CH), 127.0 (CH), 126.9 (q, *J* = 1.7 Hz, 2 × CH), 126.0 (q, *J* = 286.8 Hz, CF_3), 62.2 (CH_2), 62.0 (CH_2), 54.7 (CH), 53.4 (q, *J* = 24.6 Hz, <u>C</u>-CF_3), 37.1 (CH_2), 13.7 (CH_3), 13.5 (CH_3); ^{19}F NMR (376 MHz, $CDCl_3$) δ -65.1 (s); HRMS (ESI) m/z: $[M + H]^+$ Calcd for $C_{20}H_{19}Cl_2F_3NO_5S$ 512.0308; Found 512.0304; Enantiomeric excess was determined by HPLC analysis using a Chiralpak® IC column (Hexane/*i*-PrOH: 90/10, flow rate 1.0 mL/min, λ = 219 nm): *ent*-(*S*)-minor t_r = 7.0 min and *ent*-(*R*)-major t_r = 9.0 min.

(*R*)-Diethyl 2-(2-(3,5-dimethylphenyl)-1,1,1-trifluoro-4-oxo-4-(thiazol-2-yl)butan-2-yl)malonate (5j):

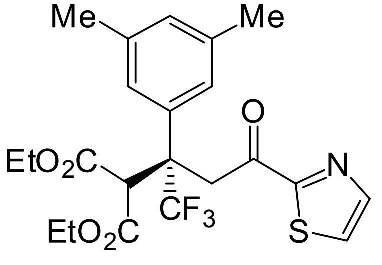

Product **5j** was prepared from enone **4j** according to general high-pressure procedure. A 0.9 mL Teflon ampoule was charged with 22 mg of catalyst (1*R*,2*R*)-**1b** (0.049 mmol, 5 mol%), 300 mg of enone **4j** (0.96 mmol), 234 mg of diethyl malonate (1.46 mmol, 1.5 equiv) and filled up with toluene (*ca.* 0.6 mL). The reaction was carried out under pressure of 10–9.5 kbar at 20–25°C for 20 h. Flash column chromatography on a silica gel using hexane fraction from petroleum/DCM as an eluent afforded 406 mg of (*R*)-**5j** (90% yield, 96% conversion) with 85% ee.

White solid; 84.5% ee, $[\alpha]_D^{24}$= –36.6 (c 1.38, $CHCl_3$); ^{1}H NMR (400 MHz, $CDCl_3$) δ 8.04 (d, *J* = 3.0 Hz, 1H), 7.68 (d, *J* = 3.0 Hz, 1H), 7.10 (bs, 2H), 6.92 (bs, 1H), 4.87 (dq, *J* = 18.6, 1.1 Hz, 1H), 4.57 (d, *J* = 18.6 Hz, 1H), 4.54 (s, 1H), 4.31 – 4.19 (m, 2H), 3.95 (q, *J* = 7.1 Hz, 2H), 2.27 (s, 6H), 1.29 (t, *J* = 7.1 Hz, 3H), 0.99 (t, *J* = 7.1 Hz, 3H); ^{13}C NMR (100 MHz, $CDCl_3$) δ 189.3 (C=O), 167.2 (C), 166.4 (C=O), 166.1 (C=O), 144.7 (CH), 137.4 (2 × C), 134.6 (C), 129.8 (CH), 126.7 (q, *J* = 287.8 Hz, CF_3), 126.5 (CH), 125.5 (q, *J* = 1.5 Hz, 2 × CH), 61.9 (CH_2), 61.6 (CH_2), 55.7 (q, *J* = 1.7 Hz, CH), 53.6 (q, *J* = 24.8 Hz, <u>C</u>-CF_3), 37.3 (q, *J* = 1.8 Hz, CH_2), 21.5 (2 × CH_3), 13.8 (CH_3), 13.5 (CH_3); ^{19}F NMR (376 MHz, $CDCl_3$) δ -63.8 (s); HRMS (ESI) m/z: $[M + H]^+$ Calcd for $C_{22}H_{25}F_3NO_5S$ 472.1400; Found 472.1396; Enantiomeric excess was determined by HPLC analysis using a Chiralpak® IC column (Hexane/*i*-PrOH: 90/10, flow rate 1.0 mL/min, λ = 219 nm): *ent*-(*S*)-minor t_r = 7.9 min and *ent*-(*R*)-major t_r = 12.1 min.

(*R*)-Diethyl 2-(1,1,1-trifluoro-2-(naphthalen-2-yl)-4-oxo-4-(thiazol-2-yl)butan-2-yl)malonate (5k): Product **5k** was prepared from enone **4k** according to general high-pressure procedure. A 2.0 mL Teflon ampoule was charged with 45.0 mg of catalyst (1*R*,2*R*)-**1b** (0.099 mmol, 10 mol%), 335 mg of enone **4k** (1.00 mmol), 245.4 mg of diethyl malonate (1.5 mmol, 1.5 equiv) and filled up with toluene (*ca.* 1.5 mL). The reaction was carried out under pressure of 10–9.5 kbar at 20–25°C for 48 h. Flash column chromatography on a silica gel using hexane fraction from petroleum/DCM as an eluent afforded 395 mg of (*R*)-**5k** (80% yield, 90% conversion) with 93% ee.

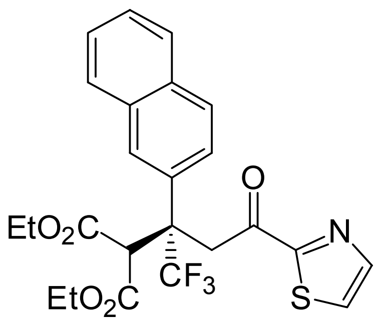

White solid; 93.0% ee, $[\alpha]_D^{25}$= –46.6 (c 1.76, $CHCl_3$); ^{1}H NMR (400 MHz, $CDCl_3$) δ 8.06 (d, *J* = 3.1 Hz, 1H), 7.99 (d, *J* = 1.4 Hz, 1H), 7.83 – 7.73 (m, 3H), 7.68 (d, *J* = 3.0 Hz, 1H), 7.65 (dd, *J* = 8.9, 1.0 Hz, 1H), 7.50 – 7.42 (m, 2H), 5.02 (dq, *J* = 18.7, 0.8 Hz, 1H), 4.71 (d, *J* = ~19 Hz, 1H), 4.68 (s, 1H), 4.33 – 4.22 (m, 2H), 3.94 – 3.81 (m, 2H), 1.30 (t, *J* = 7.1 Hz, 3H), 0.83 (t, *J* = 7.1 Hz, 3H); ^{13}C NMR (100 MHz, $CDCl_3$) δ 189.2 (C=O), 167.0 (C), 166.4 (C=O), 166.0 (C=O), 144.8 (CH), 132.7 (C), 132.6 (C), 132.1 (C), 128.5 (CH), 127.6 (2 × CH), 127.2 (CH), 126.7 (q, *J* = 287 Hz, CF_3), 126.7 (2 × CH), 126.2 (CH), 125.3 (q, *J* = 1.5 Hz, CH), 62.0 (CH_2), 61.7 (CH_2), 55.5 (q, *J* = 1.6 Hz, CH), 53.9 (q, *J* = 24.9 Hz, C-CF_3), 37.3 (d, *J* = 1.7 Hz, CH_2), 13.8 (CH_3), 13.4 (CH_3); ^{19}F NMR (376 MHz, $CDCl_3$) δ -64.1 (s); HRMS (ESI) m/z: $[M + H]^+$ Calcd for $C_{24}H_{23}F_3NO_5S$ 494.1244; Found 494.1245; Enantiomeric excess was determined by HPLC analysis using a Chiralpak® IC column (Hexane/*i*-PrOH: 90/10, flow rate 1.0 mL/min, λ = 219 nm): *ent*-(*S*)-minor t_r = 10.6 min and *ent*-(*R*)-major t_r = 13.9 min.

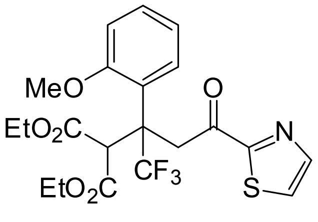

Diethyl 2-(1,1,1-trifluoro-2-(2-methoxyphenyl)-4-oxo-4-(thiazol-2-yl)butan-2-yl)malonate (5l):

the product **5l** is not formed

(*R*)-Diethyl 2-(1,1,1-trifluoro-2-(furan-2-yl)-4-oxo-4-(thiazol-2-yl)butan-2-yl)malonate (5m): Product **5m** was prepared from enone **4m** according to general high-pressure procedure. A 1.2 mL Teflon ampoule was charged with 28.3 mg of catalyst (1*R*,2*R*)-**1b** (0.062 mmol, 5 mol%), 349 mg of enone **4m** (1.28 mmol), 287 mg of diethyl malonate (1.8 mmol, 1.4 equiv) and filled up with toluene (*ca.* 0.8 mL). The reaction was carried out under pressure of 10–9.5 kbar at 20–25°C for 20 h. Flash column chromatography on a silica gel using hexane fraction from petroleum/DCM as an eluent afforded 517 mg of (*R*)-**5m** (93% yield, 99% conversion) with 88% ee.

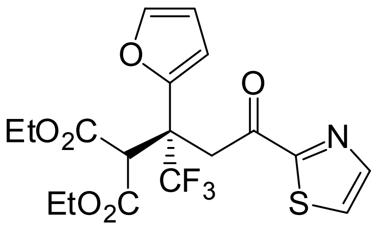

Yellowish oil; 88.0% ee, $[\alpha]_D^{22}$= –22.3 (c 1.55, $CHCl_3$); ^{1}H NMR (400 MHz, $CDCl_3$) δ 8.02 (d, *J* = 3.0 Hz, 1H), 7.69 (d, *J* = 3.0 Hz, 1H), 7.37 (dd, *J* = 1.9, 0.8 Hz, 1H), 6.51 (d, *J* = 3.4 Hz, 1H), 6.37 (dd, *J* = 3.4, 1.8 Hz, 1H), 4.64 (s, 1H), 4.55 (d, *J* = 17.8 Hz, 1H), 4.42 (d, *J* = 17.8 Hz, 1H), 4.23 (q, *J* = 7.1 Hz, 2H), 4.12 (q, *J* = 7.2 Hz, 2H), 1.26 (t, *J* = 7.1 Hz, 3H), 1.16 (t, *J* = 7.1 Hz, 3H); ^{13}C NMR (100 MHz, $CDCl_3$) δ 188.8 (C=O), 166.8 (C), 166.1 (C=O), 165.9 (C=O), 147.8 (C), 144.8 (CH), 142.2 (CH), 126.7 (CH), 125.5 (q, *J* = 286.2 Hz, CF_3), 110.6 (CH), 110.1 (q, *J* = 1.4 Hz, CH), 61.9 (CH_2), 61.8 (CH_2), 52.7 (CH), 50.3 (q, *J* = 26.7 Hz, C-CF_3), 36.9 (q, *J* = 1.3 Hz, CH_2), 13.8 (CH_3), 13.7 (CH_3); ^{19}F NMR (376 MHz, $CDCl_3$) δ -69.0 (s); HRMS (ESI) m/z: $[M + H]^+$ Calcd for $C_{18}H_{19}F_3NO_6S$ 434.0880; Found 434.0877; Enantiomeric excess was determined by HPLC analysis using a Chiralpak® IC column (Hexane/*i*-PrOH: 90/10, flow rate 1.0 mL/min, λ = 219 nm): *ent*-(*S*)-minor t_r = 12.7 min and *ent*-(*R*)-major t_r = 15.2 min.

(*S*)-Diethyl 2-(1,1,1-trifluoro-4-oxo-4-(thiazol-2-yl)-2-(thiophen-2-yl)butan-2-yl)malonate (5n):

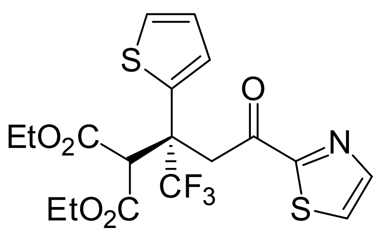

Product **5n** was prepared from enone **4n** according to general high-pressure procedure. A 1.2 mL Teflon ampoule was charged with 27.0 mg of catalyst (1*R*,2*R*)-**1b** (0.060 mmol, 5 mol%), 371 mg of enone **4n** (1.28 mmol), 295 mg of diethyl malonate (1.8 mmol, 1.5 equiv) and filled up with toluene (*ca.* 0.8 mL). The reaction was carried out under pressure of 10–9.5 kbar at 20–25°C for 20 h. Flash column chromatography on a silica gel using hexane fraction from petroleum/DCM as an eluent afforded 512 mg of (*R*)-**5n** (89% yield, 99% conversion) with 88 % ee.

Colorless viscous oil (to light yellow upon standing); 87.5% ee, $[\alpha]_D^{25}$= –46.3 (c 1.30, $CHCl_3$); ^1H NMR (400 MHz, $CDCl_3$) δ 8.03 (d, *J* = 3.0 Hz, 1H), 7.70 (d, *J* = 3.0 Hz, 1H), 7.31 (dd, *J* = 5.1, 1.2 Hz, 1H), 7.31 – 7.28 (m, 1H), 6.97 (dd, *J* = 5.1, 3.8 Hz, 1H), 4.77 (d, *J* = 17.6 Hz, 1H), 4.71 (s, 1H), 4.52 (d, *J* = 17.6 Hz, 1H), 4.28 – 4.19 (m, 2H), 4.07 (q, *J* = 7.2 Hz, 2H), 1.27 (t, *J* = 7.1 Hz, 3H), 1.12 (t, *J* = 7.1 Hz, 3H); ^{13}C NMR (100 MHz, $CDCl_3$) δ 189.1 (C=O), 166.8 (C), 166.3 (C=O), 166.0 (C=O), 144.9 (CH), 137.8 (C), 128.3 (q, *J* = 1.4 Hz, CH), 126.9 (CH), 126.3 (CH), 126.0 (q, *J* = 286.6 Hz, CF_3), 125.7 (CH), 62.0 (CH_2), 61.8 (CH_2), 55.1 (CH), 51.8 (q, *J* = 26.3 Hz, <u>C</u>-CF_3), 39.5 (q, *J* = 1.4 Hz, CH_2), 13.8 (CH_3), 13.6 (CH_3); ^{19}F NMR (376 MHz, $CDCl_3$) δ -68.2 (s); HRMS (ESI) m/z: $[M + H]^+$ Calcd for $C_{18}H_{19}F_3NO_5S_2$ 450.0651; Found 450.0651; Enantiomeric excess was determined by HPLC analysis using a Chiralpak® IC column (Hexane/*i*-PrOH: 90/10, flow rate 1.0 mL/min, λ = 219 nm): *ent*-(*R*)-minor t_r = 10.1 min and *ent*-(*S*)-major t_r = 14.6 min.

(*R*)-Diethyl 2-(1,1,1-trifluoro-4-oxo-4-(thiazol-2-yl)-2-(thiophen-3-yl)butan-2-yl)malonate (5o):

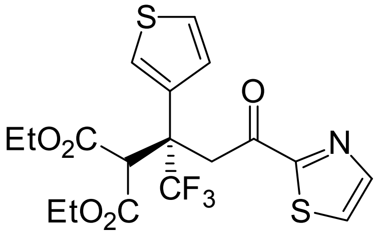

Product **5o** was prepared from enone **4o** according to general high-pressure procedure. A 1.2 mL Teflon ampoule was charged with 28.1 mg of catalyst **1b** (0.062 mmol, 5 mol%), 355 mg of enone **4o** (1.23 mmol), 302 mg of diethyl malonate (1.9 mmol, 1.5 equiv) and filled up with toluene (*ca.* 0.8 mL). The reaction was carried out under pressure of 10–9.5 kbar at 20–25°C for 20 h. Flash column chromatography on a silica gel using hexane fraction from petroleum/DCM as an eluent afforded 451 mg of (*R*)-**5o** (82% yield, 99% conversion) with 87% ee.

Pale yellow viscous oil; 87.2% ee, $[\alpha]_D^{25}$= –41.7 (c 1.06, $CHCl_3$); ^1H NMR (400 MHz, $CDCl_3$) δ 8.02 (d, *J* = 3.0 Hz, 1H), 7.69 (d, *J* = 3.0 Hz, 1H), 7.48 (t, *J* = 2.0 Hz, 1H), 7.28 – 7.25 (m, 2H), 4.72 (d, *J* = 17.8 Hz, 1H), 4.64 (s, 1H), 4.47 (d, *J* = 17.8 Hz, 1H), 4.28 – 4.17 (m, 2H), 4.04 (q, *J* = 7.1 Hz, 2H), 1.27 (t, *J* = 7.1 Hz, 3H), 1.09 (t, *J* = 7.1 Hz, 3H); ^{13}C NMR (100 MHz, $CDCl_3$) δ 189.5 (C=O), 167.0 (C), 166.4 (C=O), 166.2 (C=O), 144.8 (CH), 135.2 (C), 127.7 (q, *J* = 1.8 Hz, CH), 126.8 (CH), 126.4 (q, *J* = 286.2 Hz, CF_3), 124.7 (CH), 124.6 (q, *J* = 1.9 Hz, CH), 61.9 (CH_2), 61.7 (CH_2), 54.6 (q, *J* = 0.9 Hz, CH), 51.4 (q, *J* = 25.6 Hz, <u>C</u>-CF_3), 38.5 (q, *J* = 1.6 Hz, CH_2), 13.8 (CH_3), 13.6 (CH_3); ^{19}F NMR (376 MHz, $CDCl_3$) δ -67.6 (s); HRMS (ESI) m/z: $[M + H]^+$ Calcd for $C_{18}H_{19}F_3NO_5S_2$ 450.0651; Found 450.0651; Enantiomeric excess was determined by HPLC analysis using a Chiralpak® IC column (Hexane/*i*-PrOH: 90/10, flow rate 1.0 mL/min, λ = 219 nm): *ent*-(*S*)-minor t_r = 8.4 min and *ent*-(*R*)-major t_r = 13.2 min.

(*R*)-Diethyl 2-(1,1,1-trifluoro-4-oxo-2-(pyridin-3-yl)-4-(thiazol-2-yl)butan-2-yl)malonate (5p):

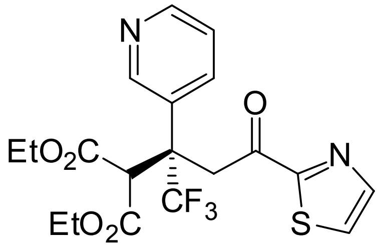

Product **5p** was prepared from enone **4p** according to general high-pressure procedure. A 0.7 mL Teflon ampoule was charged with 15.7 mg of catalyst (1*R*,2*R*)-**1b** (0.035 mmol, 5 mol%), 183 mg of enone **4p** (0.64 mmol), 231 mg of diethyl malonate (1.5 mmol, 1.5 equiv) and filled up with toluene (*ca.* 0.4 mL). The reaction was carried out under pressure of 10–9.5 kbar at 20–25°C for 20 h. Flash column chromatography on a silica gel using hexane fraction from petroleum/AcOEt (9:1→1:4) as an eluent afforded 242 mg of (*R*)-**5p** (85% yield, 97% conversion) with 89% ee.

Yellow viscous oil; 89.2% ee, $[\alpha]_D^{22}$= –44.7 (c 1.15, $CHCl_3$); ^{1}H NMR (400 MHz, $CDCl_3$) δ 8.79 (d, *J* = 2.4 Hz, 1H), 8.57 (dd, *J* = 4.7, 1.4 Hz, 1H), 8.05 (d, *J* = 3.0 Hz, 1H), 7.91 (bd, *J* = 8.3 Hz, 1H), 7.73 (d, *J* = 3.0 Hz, 1H), 7.30 (ddd, *J* = 8.3, 4.7, 0.8 Hz 1H), 4.89 (d, *J* = 18.7 Hz, 1H), 4.60 (d, *J* = 18.7 Hz, 1H), 4.56 (s, 1H), 4.31 – 4.23 (m, 2H), 4.00 (q, *J* = 7.1 Hz, 2H), 1.30 (t, *J* = 7.1 Hz, 3H), 1.02 (t, *J* = 7.1 Hz, 3H); ^{13}C NMR (100 MHz, $CDCl_3$) δ 188.8 (C=O), 166.4 (C), 166.0 (C=O), 165.6 (C=O), 149.0 (2 × CH), 144.8 (CH), 135.9 (CH), 130.5 (C), 127.0 (CH), 126.2 (q, *J* = 287.3 Hz, CF_3), 122.6 (CH), 62.1 (CH_2), 61.9 (CH_2), 54.4 (CH), 52.3 (q, *J* = 25.3 Hz, C-CF_3), 36.5 (CH_2), 13.7 (CH_3), 13.4 (CH_3); ^{19}F NMR (376 MHz, $CDCl_3$) δ -65.7 (s); HRMS (ESI) m/z: $[M + H]^+$ Calcd for $C_{19}H_{20}F_3N_2O_5S$ 445.1040; Found 445.1032; Enantiomeric excess was determined by HPLC analysis using a Chiralpak® IC column (Hexane/*i*-PrOH: 80/20, flow rate 1.0 mL/min, λ = 219 nm): *ent*-(*R*)-major t_r = 16.2 min and *ent*-(*S*)-minor t_r = 21.4 min.

(*R*)-Diethyl 2-(1-oxo-1-(thiazol-2-yl)-3-(trifluoromethyl)pentan-3-yl)malonate (5q):

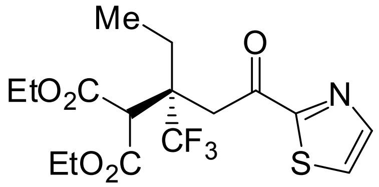

Product **5q** was prepared from enone **4q** according to general high-pressure procedure. A 0.9 mL Teflon ampoule was charged with 22.9 mg of catalyst (1*R*,2*R*)-**1b** (0.032 mmol, 5 mol%), 238 mg of enone **4q** (1.01 mmol), 240 mg of diethyl malonate (1.50 mmol, 1.5 equiv) and filled up with toluene (*ca.* 0.6 mL). The reaction was carried out under pressure of 10–9.5 kbar at 20–25°C for 20 h. Flash column chromatography on a silica gel using hexane fraction from petroleum/DCM as an eluent afforded 348 mg of (*R*)-**5q** (87% yield, 99% conversion) with 94% ee.

Colorless oil; 93.9% ee, $[\alpha]_D^{22}$= –17.7 (c 1.17, $CHCl_3$); ^{1}H NMR (400 MHz, $CDCl_3$) δ 8.02 (d, *J* = 3.0 Hz, 1H), 7.70 (d, *J* = 3.0 Hz, 1H), 4.42 (s, 1H), 4.25 – 4.14 (m, 5H (contains d at 4.17, *J* = 17 Hz, 1H)), 3.78 (d, *J* = 17.2 Hz, 1H), 2.29 (dqq, *J* = 15.2, 7.5, 1.1 Hz, 1H), 2.15 (dq, *J* = 15.0, 7.5 Hz, 1H), 1.26 (t, *J* = 7.1 Hz, 3H), 1.25 (t, *J* = 7.1 Hz, 3H), 1.06 (tq, *J* = 7.5, 1.1 Hz, 3H); ^{13}C NMR (100 MHz, $CDCl_3$) δ 190.7 (C=O), 167.2 (C), 167.03 (C=O), 166.97 (C=O), 144.8 (CH), 127.4 (q, *J* = 286.6 Hz, CF_3), 126.8 (CH), 61.71 (CH_2), 61.68 (CH_2), 52.0 (q, *J* = 1.4 Hz, CH), 48.8 (q, *J* = 23.5 Hz, C-CF_3), 36.3 (q, *J* = 2.0 Hz, CH_2), 24.8 (q, *J* = 1.6 Hz, CH_2), 13.8 (2 × CH_3), 8.7 (CH_3); ^{19}F NMR (376 MHz, $CDCl_3$) δ -68.6 (s); HRMS (ESI) m/z: $[M + H]^+$ Calcd for $C_{16}H_{21}F_3NO_5S$ 396.1087; Found 396.1081; Enantiomeric excess was determined by HPLC analysis using a Chiralpak® ID column (Hexane/*i*-PrOH: 98/2, flow rate 1.0 mL/min, λ = 219 nm): *ent*-(*S*)-minor t_r = 9.1 min and *ent*-(*R*)-major t_r = 10.5 min.

(*R*)-Diethyl 2-(1-oxo-1-(thiazol-2-yl)-3-(trifluoromethyl)heptan-3-yl)malonate (5r):

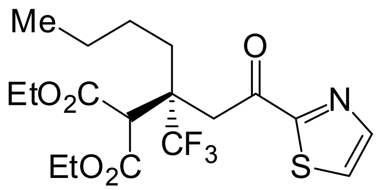

Product **5r** was prepared from enone **4r** according to general high-pressure procedure. A 0.9 mL Teflon ampoule was charged with 18.6 mg of catalyst (1*R*,2*R*)-**1b** (0.041 mmol, 5 mol%), 210 mg of enone **4r** (0.80 mmol), 192 mg of diethyl malonate (1.19 mmol, 1.5 equiv) and filled up with toluene (*ca.* 0.6 mL). The reaction was carried out under pressure of 10–9.5 kbar at 20–25°C for 20 h. Flash column chromatography on a silica gel using hexane fraction from petroleum/DCM as an eluent afforded 297 mg of (*R*)-**5r** (88% yield, 99% conversion) with 88% ee.

Colorless oil; 87.6% ee, $[\alpha]_D^{22}= -14.4$ (c 1.17, $CHCl_3$); ^{1}H NMR (400 MHz, $CDCl_3$) δ 8.02 (d, *J* = 3.0 Hz, 1H), 7.70 (d, *J* = 3.0 Hz, 1H), 4.42 (s, 1H), 4.26 – 4.12 (m, 5H), 3.80 (d, *J* = 17.2 Hz, 1H), 2.26 – 2.14 (m, 1H), 2.04 (ddd, *J* = 14.8, 11.9, 4.8 Hz, 1H), 1.53 – 1.29 (m, 4H), 1.26 (t, *J* = 7.1 Hz, 3H), 1.25 (t, *J* = 7.1 Hz, 3H), 0.92 (t, *J* = 7.2 Hz, 3H); ^{13}C NMR (100 MHz, $CDCl_3$) δ 190.6 (C=O), 167.1 (C), 167.0 (C=O), 166.9 (C=O), 144.8 (CH), 127.3 (q, *J* = 286.5 Hz, CF_3), 126.8 (CH), 61.7 (CH_2), 61.6 (CH_2), 52.1 (q, *J* = 1.4 Hz, CH), 48.7 (q, *J* = 23.7 Hz, C-CF_3), 36.7 (q, *J* = 1.9 Hz, CH_2), 31.6 (q, *J* = 1.4 Hz, CH_2), 26.1 (q, *J* = 1.1 Hz, CH_2), 23.2 (CH_2), 13.82 (CH_3), 13.80 (CH_3), 13.78 (CH_3); ^{19}F NMR (376 MHz, $CDCl_3$) δ -68.8 (s); HRMS (ESI) m/z: $[M + H]^+$ Calcd for $C_{18}H_{25}F_3NO_5S$ 424.1400; Found 424.1404; Enantiomeric excess was determined by HPLC analysis using a Chiralpak® ID column (Hexane/*i*-PrOH: 98/2, flow rate 1.0 mL/min, λ = 219 nm): *ent*-(*S*)-minor t_r = 8.8 min and *ent*-(*R*)-major t_r = 9.8 min.

(*R*)-Diethyl 2-(1-oxo-5-phenyl-1-(thiazol-2-yl)-3-(trifluoromethyl)pentan-3-yl)malonate (5s):

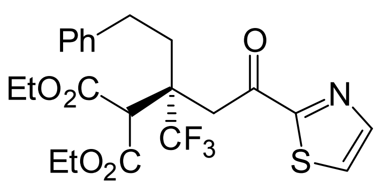

Product **5s** was prepared from enone **4s** according to general high-pressure procedure. A 1.2 mL Teflon ampoule was charged with 27.0 mg of catalyst (1*R*,2*R*)-**1b** (0.060 mmol, 5 mol%), 370 mg of enone **4s** (1.19 mmol), 283 mg of diethyl malonate (1.77 mmol, 1.5 equiv) and filled up with toluene (*ca.* 0.8 mL). The reaction was carried out under pressure of 10–9.5 kbar at 20–25°C for 20 h. Flash column chromatography on a silica gel using hexane fraction from petroleum/DCM as an eluent afforded 449 mg of (*R*)-**5s** (80% yield, 99% conversion) with 76% ee (81% ee in another small-scale sample).

Colorless oil; 75.7-81% ee, $[\alpha]_D^{26}= -22.7$ (c 0.95, $CHCl_3$); ^{1}H NMR (400 MHz, $CDCl_3$) δ 8.02 (d, *J* = 3.0 Hz, 1H), 7.70 (d, *J* = 3.0 Hz, 1H), 7.31 – 7.25 (m, 2H), 7.23 – 7.15 (m, 3H), 4.48 (s, 1H), 4.26 (d, *J* = 17.2 Hz, 1H), 4.25 – 4.16 (m, 4H), 3.93 (d, *J* = 17.2 Hz, 1H), 2.88 – 2.71 (m, 2H), 2.57 – 2.46 (m, 1H), 2.39 – 2.29 (m, 1H), 1.26 (t, *J* = 7.1 Hz, 3H), 1.25 (t, *J* = 7.1 Hz, 3H); ^{13}C NMR (100 MHz, $CDCl_3$) δ 190.4 (C=O), 167.0 (C), 166.9 (C=O), 166.8 (C=O), 144.8 (CH), 141.2 (C), 128.44 (2 × CH), 128.39 (2 × CH), 127.3 (q, *J* = 286.6 Hz, CF_3), 126.9 (CH), 126.1 (CH), 61.79 (CH_2), 61.78 (CH_2), 52.0 (q, *J* = 1.0 Hz, CH), 48.8 (q, *J* = 23.6 Hz, C-CF_3), 36.7 (q, *J* = 1.6 Hz, CH_2), 33.9 (CH_2), 30.5 (q, *J* = 1.2 Hz, CH_2), 13.83 (CH_3), 13.80 (CH_3); ^{19}F NMR (376 MHz, $CDCl_3$) δ -69.0 (s); HRMS (ESI) m/z: $[M + H]^+$ Calcd for $C_{22}H_{25}F_3NO_5S$ 472.1400; Found 472.1398; Enantiomeric excess was determined by HPLC analysis using a Chiralpak® ID column (Hexane/*i*-PrOH: 98/2, flow rate 1.0 mL/min, λ = 219 nm): *ent*-(*S*)-minor t_r = 11.2 min and *ent*-(*R*)-major t_r = 12.2 min.

(*S*)-Triethyl 2-(2-oxo-2-(thiazol-2-yl)ethyl)-2-(trifluoromethyl)propane-1,1,3-tricarboxylate (5t):

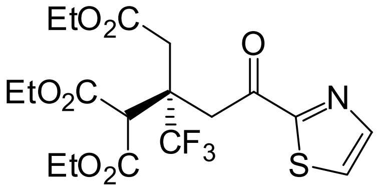

Product **5t** was prepared from enone **4t** according to general high-pressure procedure. A 1.2 mL Teflon ampoule was charged with 24.6 mg of catalyst (1*R*,2*R*)-**1b** (0.054 mmol, 5 mol%), 292 mg of enone **4t** (1.00 mmol), 234 mg of diethyl malonate (1.5 mmol, 1.5 equiv) and filled up with toluene (*ca.* 0.8 mL). The reaction was carried out under pressure of 10–9.5 kbar at 20–25°C for 20 h. Flash column chromatography on a silica gel using hexane fraction from petroleum/DCM as an eluent afforded 406 mg of (*S*)-**5t** (90% yield, 99% conversion, 86% ee).

Pale yellow oil; 85.9% ee, $[\alpha]_D^{22}$= +11.6 (c 1.05, $CHCl_3$); ^1H NMR (400 MHz, $CDCl_3$) δ 8.02 (d, *J* = 3.0 Hz, 1H), 7.69 (d, *J* = 3.0 Hz, 1H), 4.38 (s, 1H), 4.28 – 4.16 (m, 6H), 4.11 (q, *J* = 7.2 Hz, 2H), 3.42 (d, *J* = 15.8 Hz, 1H), 3.25 (d, *J* = 15.8 Hz, 1H), 1.26 (t, *J* = 7.1 Hz, 3H), 1.25 (t, *J* = 7.1 Hz, 3H), 1.22 (t, *J* = 7.1 Hz, 3H); ^{13}C NMR (100 MHz, $CDCl_3$) δ 190.1 (C=O), 169.7 (C=O), 166.8 (C), 166.39 (C=O), 166.37 (C=O), 144.9 (CH), 126.6 (CH), 126.6 (q, *J* = 286.2 Hz, CF_3), 61.99 (CH_2), 61.95 (CH_2), 60.9 (CH_2), 52.4 (q, *J* = 1.2 Hz, CH), 47.6 (q, *J* = 25.0 Hz, C-CF_3), 36.6 (q, *J* = 1.6 Hz, CH_2), 34.6 (q, *J* = 1.7 Hz, CH_2), 13.9 (CH_3), 13.79 (CH_3), 13.76 (CH_3); ^{19}F NMR (376 MHz, $CDCl_3$) δ -70.9 (s); HRMS (ESI) m/z: $[M + H]^+$ Calcd for $C_{18}H_{23}F_3NO_7S$ 454.1142; Found 454.1133; Enantiomeric excess was determined by HPLC analysis using a Chiralpak® IC column (Hexane/*i*-PrOH: 95/5, flow rate 1.0 mL/min, λ = 219 nm): *ent*-(*R*)-minor t_r = 20.9 min and *ent*-(*S*)-major t_r = 25.2 min.

(*R*)-Diethyl 2-(2-((1,3-dioxolan-2-yl)methyl)-1,1,1-trifluoro-4-oxo-4-(thiazol-2-yl)butan-2-yl)malonate (5u):

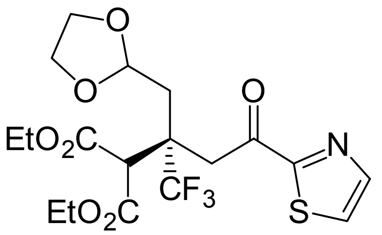

Product **5u** was prepared from enone **4u** according to general high-pressure procedure. A 0.9 mL Teflon ampoule was charged with 21.2 mg of catalyst (1*R*,2*R*)-**1b** (0.048 mmol, 5 mol%), 264 mg of enone **4u** (0.90 mmol), 220 mg of diethyl malonate (1.4 mmol, 1.5 equiv) and filled up with toluene (*ca.* 0.6 mL). The reaction was carried out under pressure of 10–9.5 kbar at 20–25°C for 20 h. Flash column chromatography on a silica gel using hexane fraction from petroleum/DCM as an eluent afforded 307 mg of (*R*)-**5u** (75% yield, 99% conversion) with 83% ee.

Pale yellow oil; 83.0% ee, $[\alpha]_D^{22}$= –12.6 (c 1.15, $CHCl_3$); ^1H NMR (400 MHz, $CDCl_3$) δ 8.02 (d, *J* = 3.0 Hz, 1H), 7.69 (d, *J* = 3.0 Hz, 1H), 5.20 (t, *J* = 5.0 Hz, 1H), 4.50 (s, 1H), 4.27 (d, *J* = 17.4 Hz, 1H), 4.26 – 4.17 (m, 4H), 4.03 (d, *J* = 17.4 Hz, 1H), 3.94 – 3.76 (m, 4H), 2.63 (dd, *J* = 15.3, 5.3 Hz, 1H), 2.46 (dd, *J* = 15.3, 4.8 Hz, 1H), 1.27 (t, *J* = 7.1 Hz, 3H), 1.25 (t, *J* = 7.1 Hz, 3H); ^{13}C NMR (100 MHz, $CDCl_3$) δ 190.6 (C=O), 167.1 (C), 166.8 (C=O), 166.7 (C=O), 144.8 (CH), 127.0 (q, *J* = 286.2 Hz, CF_3), 126.5 (CH), 101.4 (q, *J* = 2.0 Hz, CH), 64.7 (CH_2), 64.5 (CH_2), 61.8 (CH_2), 61.6 (CH_2), 52.4 (q, *J* = 0.9 Hz, CH), 47.3 (q, *J* = 24.7 Hz, C-CF_3), 36.8 (q, *J* = 1.8 Hz, CH_2), 35.6 (CH_2), 13.81 (CH_3), 13.79 (CH_3); ^{19}F NMR (376 MHz, $CDCl_3$) δ -69.3 (s); HRMS (ESI) m/z: $[M + H]^+$ Calcd for $C_{18}H_{23}F_3NO_7S$ 454.1142; Found 454.1139; Enantiomeric excess was determined by HPLC analysis using a Chiralpak® IA column (Hexane/*i*-PrOH: 95/5, flow rate 1.0 mL/min, λ = 219 nm): *ent*-(*R*)-major t_r = 13.0 min and *ent*-(*S*)-minor t_r = 14.3 min.

(*S*)-Diethyl 2-(4-(benzo[d]thiazol-2-yl)-1,1,1-trifluoro-2-(4-fluorophenyl)-4-oxobutan-2-yl)malonate (**7a**):

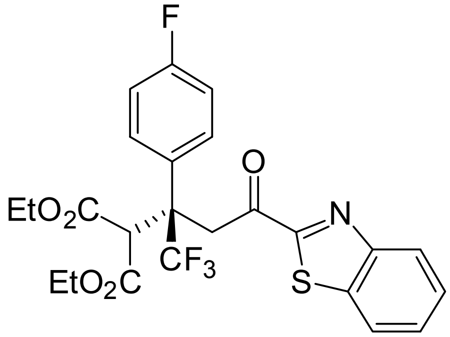

Product **7a** was prepared from enone **6a** according to general high-pressure procedure. A 1.5 mL Teflon ampoule was charged with 12.6 mg of catalyst (1*S*,2*S*)-**1a** (0.030 mmol, 5 mol%), 212 mg of enone **6a** (0.60 mmol), 150 mg of diethyl malonate (0.93 mmol, 1.5 equiv) and filled up with toluene (*ca.* 1.1 mL). The reaction was carried out under pressure of 10–9.5 kbar at 20–25°C for 20 h. Flash column chromatography on a silica gel using hexane fraction from petroleum/DCM as an eluent afforded 257 mg of (*S*)-**7a** (84% yield, 98% conversion, 93% ee).

Colorless viscous oil (solidified upon standing); 93.4% ee, $[\alpha]_D^{22}$= +45.4 (c 1.20, $CHCl_3$); ^{1}H NMR (400 MHz, $CDCl_3$) δ 8.23 (dm, *J* = 8.2 Hz, 1H), 7.98 (dm, *J* = 8.2 Hz, 1H), 7.62 – 7.51 (m, 4H), 7.08 – 7.00 (m, 2H), 4.98 (dq, *J* = 18.7, 0.7 Hz, 1H), 4.69 (d, *J* = 18.7 Hz, 1H), 4.54 (s, 1H), 4.32 – 4.22 (m, 2H), 4.00 (q, *J* = 7.2 Hz, 2H), 1.31 (t, *J* = 7.1 Hz, 3H), 1.04 (t, *J* = 7.1 Hz, 3H); ^{13}C NMR (100 MHz, $CDCl_3$) δ 190.7 (C=O), 166.3 (C=O), 166.04 (C), 165.97 (C=O), 162.3 (d, *J* = 248.6 Hz, CF), 153.4 (C), 137.6 (C), 130.5 (d, *J* = 3.1 Hz, C), 129.9 (dq, *J* = 8.1, 1.6 Hz, 2 × CH), 127.8 (CH), 127.0 (CH), 126.5 (q, *J* = 286.3 Hz, CF_3), 125.7 (CH), 122.4 (CH), 115.0 (d, *J* = 21.4 Hz, 2 × CH), 62.1 (CH_2), 61.9 (CH_2), 55.4 (CH), 53.3 (q, *J* = 25.0 Hz, C-CF_3), 37.5 (q, *J* = 1.7 Hz, CH_2), 13.8 (CH_3), 13.6 (CH_3); ^{19}F NMR (376 MHz,$CDCl_3$) δ -64.9 (s, 3F), -113.7 – -113.8 (m, 1F); HRMS (ESI) m/z: $[M + H]^+$ Calcd for $C_{24}H_{22}F_4NO_5S$ 512.1149; Found 512.1158; Enantiomeric excess was determined by HPLC analysis using a Chiralpak® IA column (Hexane/*i*-PrOH: 95/5, flow rate 1.0 mL/min, λ = 219 nm): with catalyst (1*S*,2*S*)-**1a** *ent*-(*S*)-major t_r = 12.9 min and *ent*-(*R*)-minor t_r = 18.0 min.

(*S*)-Diethyl 2-(4-(benzo[d]thiazol-2-yl)-1,1,1-trifluoro-4-oxo-2-(p-tolyl)butan-2-yl)malonate (**7b**):

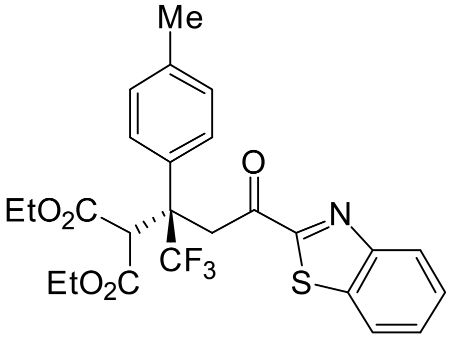

Product **7b** was prepared from enone **6b** according to general high-pressure procedure. A 1.2 mL Teflon ampoule was charged with 12.8 mg of catalyst (1*S*,2*S*)-**1a** (0.031 mmol, 5 mol%), 221 mg of enone **6b** (0.63 mmol), 150 mg of diethyl malonate (1.56 mmol, 1.6 equiv) and filled up with toluene (*ca.* 0.8 mL). The reaction was carried out under pressure of 10–9.5 kbar at 20–25°C for 20 h. Flash column chromatography on a silica gel using hexane fraction from petroleum/DCM as an eluent afforded 264 mg of (*S*)-**7b** (82% yield, 98% conversion) with 90% ee.

Colorless oil; 89.6% ee, $[\alpha]_D^{22}$= +36.4 (c 0.98, $CHCl_3$); ^{1}H NMR (400 MHz, $CDCl_3$) δ 8.23 (dm, *J* = 7.6, 1H), 7.96 (dm, *J* = 7.7, 1H), 7.61 – 7.49 (m, 2H), 7.42 (d, *J* = 8.3 Hz, 2H), 7.14 (d, *J* = 8.2 Hz, 2H), 5.00 (d, *J* = 18.8 Hz, 1H), 4.68 (d, *J* = 18.8 Hz, 1H), 4.57 (s, 1H), 4.33 – 4.21 (m, 2H), 3.98 (q, *J* = 7.1 Hz, 2H), 2.32 (s, 3H), 1.31 (t, *J* = 7.1 Hz, 3H), 1.02 (t, *J* = 7.1 Hz, 3H); ^{13}C NMR (100 MHz, $CDCl_3$) δ 190.8 (C=O), 166.4 (C=O), 166.3 (C), 166.1 (C=O), 153.4 (C), 138.0 (C), 137.5 (C), 131.6 (C), 128.8 (2 × CH), 127.7 (CH + 2 × CH), 126.9 (CH), 126.7 (q, *J* = 287.0 Hz, CF_3), 125.7 (CH), 122.3 (CH), 62.0 (CH_2), 61.7 (CH_2), 55.5 (q, *J* = 1.5 Hz, CH), 53.5 (q, *J* = 24.9 Hz, C-CF_3), 37.4 (q, *J* = 1.5 Hz, CH_2), 20.9 (CH_3), 13.8 (CH_3), 13.5 (CH_3); ^{19}F NMR (376 MHz, $CDCl_3$) δ -64.5 (s); HRMS (ESI) m/z: $[M + H]^+$ Calcd for $C_{25}H_{25}F_3NO_5S$ 508.1400; Found 508.1400; Enantiomeric excess was determined by HPLC analysis using a Chiralpak® IA column (Hexane/*i*-PrOH: 95/5, flow rate 1.0 mL/min, λ = 219 nm): with catalyst (1*S*,2*S*)-**1a** *ent*-(*S*)-major t_r = 12.5 min and *ent*-(*R*)-minor t_r = 14.9 min.

(*S*)-Diethyl 2-(4-(benzo[d]thiazol-2-yl)-1,1,1-trifluoro-4-oxo-2-(4-(trifluoromethyl)-phenyl)butan-2-yl)malonate (7c):

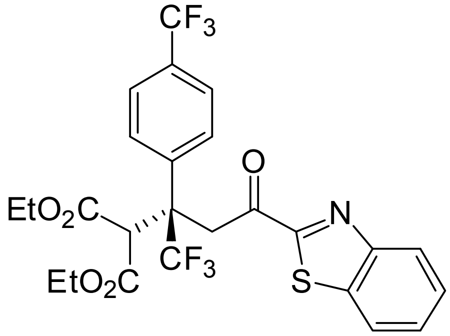

Product **7c** was prepared from enone **6c** according to general high-pressure procedure. A 1.5 mL Teflon ampoule was charged with 9.90 mg of catalyst (1*S*,2*S*)-**1a** (0.024 mmol, 5 mol%), 200 mg of enone **6c** (0.50 mmol), 124 mg of diethyl malonate (0.78 mmol, 1.6 equiv) and filled up with toluene (*ca.* 1.2 mL). The reaction was carried out under pressure of 10–9.5 kbar at 20–25°C for 20 h. Flash column chromatography on a silica gel using hexane fraction from petroleum/DCM as an eluent afforded 245 mg of (*S*)-**7b** (87% yield, 98% conversion, 88% ee).

Colorless oil; 88.2% ee, $[\alpha]_D^{22}$= +36.8 (c 1.15, $CHCl_3$); ^{1}H NMR (400 MHz, $CDCl_3$) δ 8.23 (ddd, *J* = 8.2, 1.3, 0.6 Hz, 1H), 7.97 (ddd, *J* = 7.9, 1.4, 0.7 Hz, 1H), 7.71 (d, *J* = 8.4 Hz, 2H), 7.65 – 7.52 (m, 4H), 5.01 (d, *J* = 18.8 Hz, 1H), 4.74 (d, *J* = 18.8 Hz, 1H), 4.58 (s, 1H), 4.33 – 4.24 (m, 2H), 3.99 (q, *J* = 7.1 Hz, 2H), 1.32 (t, *J* = 7.1 Hz, 3H), 1.00 (t, *J* = 7.1 Hz, 3H); ^{13}C NMR (100 MHz, $CDCl_3$) δ 190.6 (C=O), 166.1 (C=O), 165.8 (2 x C), 153.4 (C), 138.8 (C), 137.5 (C), 130.4 (q, *J* = 32.8 Hz, C-CF_3), 128.6 (q, *J* = 1.6 Hz, 2 x CH), 127.9 (CH), 127.1 (CH), 126.4 (q, *J* = 286 Hz, CF_3), 125.7 (CH), 125.0 (q, *J* = 3.8 Hz, 2 x CH), 123.8 (q, *J* = 272.1 Hz, CF_3), 122.4 (CH), 119.7 (C), 62.3 (CH_2), 62.0 (CH_2), 55.2 (CH), 53.7 (q, *J* = 25.2 Hz, C-CF_3), 37.4 (q, *J* = 1.5 Hz, CH_2), 13.8 (CH_3), 13.5 (CH_3); ^{19}F NMR (376 MHz, $CDCl_3$) δ -62.9 (s, 3F), -64.7 (s, 3F); HRMS (ESI) m/z: $[M + H]^+$ Calcd for $C_{25}H_{22}F_6NO_5S$ 562.1117; Found 562.1121; Enantiomeric excess was determined by HPLC analysis using a Chiralpak® IA column (Hexane/*i*-PrOH: 95/5, flow rate 1.0 mL/min, λ = 219 nm): with catalyst (1*S*,2*S*)-**1a** *ent*-(*S*)-major t_r = 10.4 min and *ent*-(*R*)-minor t_r = 13.4 min.

(*S*)-Diethyl 2-(4-(benzo[d]thiazol-2-yl)-2-(3-chlorophenyl)-1,1,1-trifluoro-4-oxobutan-2-yl)malonate (7d):

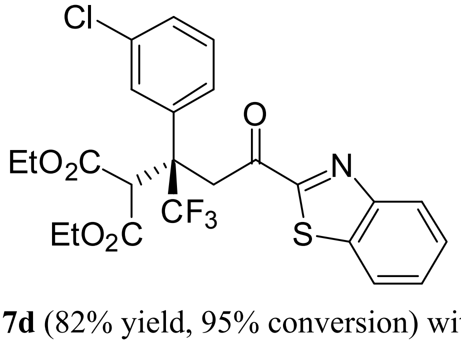

Product **7d** was prepared from enone **6d** according to general high-pressure procedure. A 1.2 mL Teflon ampoule was charged with 10.3 mg of catalyst (1*S*,2*S*)-**1a** (0.025 mmol, 6 mol%), 151 mg of enone **6d** (0.41 mmol), 102 mg of diethyl malonate (0.638 mmol, 1.5 equiv) and filled up with toluene (*ca.* 1.0 mL). The reaction was carried out under pressure of 10–9.5 kbar at 20–25°C for 20 h. Flash column chromatography on a silica gel using hexane fraction from petroleum/DCM as an eluent afforded 177 mg of (*S*)-**7d** (82% yield, 95% conversion) with 95% ee.

Colorless viscous oil; 94.7% ee, $[\alpha]_D^{22}$= +35.7 (c 1.03, $CHCl_3$); ^{1}H NMR (400 MHz, $CDCl_3$) δ 8.23 (ddd, *J* = 8.3, 1.2, 0.6 Hz, 1H), 7.96 (ddd, *J* = 7.8, 1.3, 0.7 Hz, 1H), 7.62 – 7.50 (m, 3H), 7.45 (d, *J* = 7.6 Hz, 1H), 7.34 – 7.25 (m, 2H), 4.96 (d, *J* = 18.7 Hz, 1H), 4.69 (d, *J* = 18.7 Hz, 1H), 4.56 (s, 1H), 4.28 (qd, *J* = 7.1, 1.5 Hz, 2H), 4.01 (q, *J* = 7.1 Hz, 2H), 1.31 (t, *J* = 7.1 Hz, 3H), 1.04 (t, *J* = 7.1 Hz, 3H); ^{13}C NMR (100 MHz, $CDCl_3$) δ 190.5 (C=O), 166.1 (C=O), 165.9 (C), 165.8 (C=O), 153.4 (C), 137.5 (C), 136.8 (C), 134.1 (C), 129.2 (CH), 128.41 (CH), 128.38 (q, *J* = 2.0 Hz, CH), 127.8 (CH), 127.0 (CH), 126.3 (q, *J* = 286.9 Hz, CF_3), 126.2 (q, *J* = 1.4 Hz, CH), 125.7 (CH), 122.3 (CH), 62.2 (CH_2), 61.9 (CH_2), 55.1 (q, *J* = 1.3 Hz, CH), 53.6 (q, *J* = 25.2 Hz, C-CF_3), 37.4 (q, *J* = 1.5 Hz, CH_2), 13.8 (CH_3), 13.5 (CH_3); ^{19}F NMR (376 MHz, $CDCl_3$) δ -64.7 (s); HRMS (ESI) m/z: $[M + H]^+$ Calcd for $C_{24}H_{22}ClF_3NO_5S$ 528.0854; Found 528.0852; Enantiomeric excess was determined by HPLC analysis using a Chiralpak® IC column (Hexane/*i*-PrOH: 90/10, flow rate 1.0 mL/min, λ = 219 nm): with catalyst (1*S*,2*S*)-**1a** *ent*-(*S*)-major t_r = 7.3 min and *ent*-(*R*)-minor t_r = 10.7 min.

(*S*)-Diethyl 2-(4-(benzo[d]thiazol-2-yl)-1,1,1-trifluoro-2-(3-methoxyphenyl)-4-oxobutan-2-yl)malonate (**7e**):

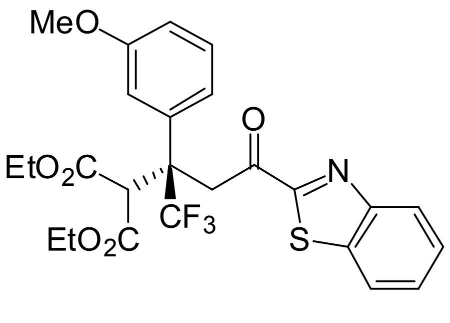

Product **7e** was prepared from enone **6e** according to general high-pressure procedure. A 2.0 mL Teflon ampoule was charged with 14.1 mg of catalyst (1*S*,2*S*)-**1a** (0.034 mmol, 5 mol%), 257 mg of enone **6e** (0.71 mmol), 169 mg of diethyl malonate (1.05 mmol, 1.5 equiv) and filled up with toluene (*ca.* 1.6 mL). The reaction was carried out under pressure of 10–9.5 kbar at 20–25°C for 20 h. Flash column chromatography on a silica gel using hexane fraction from petroleum/DCM as an eluent afforded 322 mg of (*S*)-**7e** (87% yield, 96% conversion, 93% ee).

Colorless viscous oil; 92.6% ee, $[\alpha]_D^{22}$= +25.5 (c 0.90, $CHCl_3$); ^1H NMR (400 MHz, $CDCl_3$) δ 8.23 (ddd, *J* = 8.3, 1.3, 0.7 Hz, 1H), 7.97 (ddd, *J* = 7.8, 1.4, 0.7 Hz, 1H), 7.61 – 7.51 (m, 2H), 7.25 (t, *J* = 8.1 Hz, 1H), 7.16 – 7.08 (m, 2H), 6.85 (ddd, *J* = 8.2, 2.4, 0.7 Hz, 1H), 5.00 (dq, *J* = 18.7, 1.0 Hz, 1H), 4.67 (d, *J* = 18.7 Hz, 1H), 4.58 (s, 1H), 4.28 (qd, *J* = 7.1, 1.9 Hz, 2H), 3.99 (q, *J* = 7.1 Hz, 2H), 3.75 (s, 3H), 1.31 (t, *J* = 7.1 Hz, 3H), 1.03 (t, *J* = 7.1 Hz, 3H); ^{13}C NMR (100 MHz, $CDCl_3$) δ 190.8 (C=O), 166.4 (C=O), 166.2 (C), 166.0 (C=O), 159.3 (C), 153.4 (C), 137.5 (C), 136.3 (C), 129.1 (CH), 127.7 (CH), 126.9 (CH), 126.6 (q, *J* = 287.0 Hz, CF_3), 125.7 (CH), 122.3 (CH), 120.1 (q, *J* = 1.6 Hz, CH), 114.6 (q, *J* = 1.6 Hz, CH), 113.2 (CH), 62.1 (CH_2), 61.8 (CH_2), 55.6 (q, *J* = 1.4 Hz, CH), 55.2 (CH_3), 53.8 (q, *J* = 24.7 Hz, <u>C</u>-CF_3), 37.6 (CH_2), 13.8 (CH_3), 13.6 (CH_3); ^{19}F NMR (376 MHz, $CDCl_3$) δ -64.1 (s); HRMS (ESI) m/z: $[M + H]^+$ Calcd for $C_{25}H_{25}F_3NO_6S$ 524.1349; Found 524.1350; Enantiomeric excess was determined by HPLC analysis using a Chiralpak® IA column (Hexane/*i*-PrOH: 95/5, flow rate 1.0 mL/min, λ = 219 nm): with catalyst (1*S*,2*S*)-**1a** *ent*-(*R*)-minor t_r = 12.8 min and *ent*-(*S*)-major t_r = 15.0 min.

(*S*)-Diethyl 2-(4-(benzo[d]thiazol-2-yl)-2-(3,5-dimethylphenyl)-1,1,1-trifluoro-4-oxobutan-2-yl)malonate (**7f**):

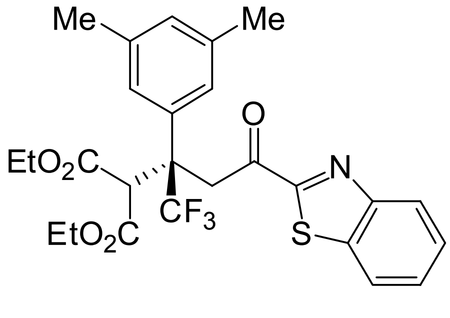

Product **7f** was prepared from enone **6f** according to general high-pressure procedure. A 0.7 mL Teflon ampoule was charged with 12.2 mg of catalyst (1*S*,2*S*)-**1a** (0.030 mmol, 8 mol%), 126 mg of enone **6f** (0.35 mmol), 83 mg of diethyl malonate (0.52 mmol, 1.5 equiv) and filled up with toluene (*ca.* 0.5 mL). The reaction was carried out under pressure of 10–9.5 kbar at 20–25°C for 20 h. Flash column chromatography on a silica gel using hexane fraction from petroleum/DCM as an eluent afforded 143 mg of (*S*)-**7f** (78% yield, 91% conversion, 93% ee).

Colorless oil; 92.7% ee, $[\alpha]_D^{22}$= +28.2 (c 0.75, $CHCl_3$); ^1H NMR (400 MHz, $CDCl_3$) δ 8.23 (d, *J* = 8.0 Hz, 1H), 7.96 (d, *J* = 7.9 Hz, 1H), 7.61 – 7.49 (m, 2H), 7.13 (s, 2H), 6.93 (s, 1H), 4.99 (d, *J* = 18.7 Hz, 1H), 4.68 (d, *J* = 18.7 Hz, 1H), 4.57 (s, 1H), 4.36 – 4.20 (m, 2H), 3.97 (q, *J* = 7.1 Hz, 2H), 2.27 (s, 6H), 1.31 (t, *J* = 7.1 Hz, 3H), 1.01 (t, *J* = 7.1 Hz, 3H); ^{13}C NMR (100 MHz, $CDCl_3$) δ 190.8 (C=O), 166.4 (C=O), 166.3 (C), 166.1 (C=O), 153.5 (C), 137.5 (C), 137.4 (2 x C), 134.6 (C), 129.8 (CH), 127.7 (CH), 126.9 (CH), 126.7 (q, *J* = 286.9 Hz, CF_3), 125.7 (CH), 125.5 (q, *J* = 1.2 Hz, 2 x CH), 122.3 (CH), 62.0 (CH_2), 61.6 (CH_2), 55.7 (q, *J* = 1.4 Hz, CH), 53.7 (q, *J* = 25.0 Hz, <u>C</u>-CF_3), 37.5 (q, *J* = 1.5 Hz, CH_2), 21.5 (2 × CH_3), 13.9 (CH_3), 13.5 (CH_3); ^{19}F NMR (376 MHz,$CDCl_3$) δ -63.8 (s); HRMS (ESI) m/z: $[M + H]^+$ Calcd for $C_{26}H_{27}F_3NO_5S$ 522.1557; Found 522.1559; Enantiomeric excess was determined by HPLC analysis using a Chiralpak® IC column (Hexane/*i*-PrOH: 90/10, flow rate 1.0 mL/min, λ = 219 nm): with catalyst (1*S*,2*S*)-**1a** *ent*-(*S*)-major t_r = 6.5 min and *ent*-(*R*)-minor t_r = 14.3.

(*S*)-Diethyl 2-(4-(benzo[d]thiazol-2-yl)-1,1,1-trifluoro-2-(naphthalen-2-yl)-4-oxobutan-2-yl)malonate (7g):

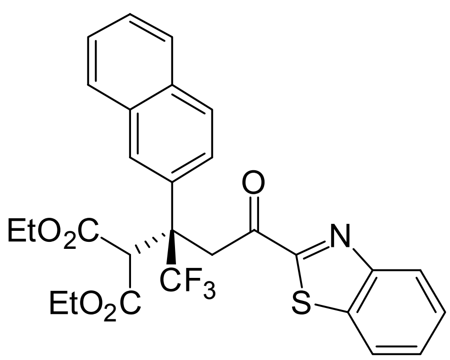

Product **7g** was prepared from enone **6g** according to general high-pressure procedure. A 1.5 mL Teflon ampoule was charged with 13.4 mg of catalyst (1*S*,2*S*)-**1a** (0.032 mmol, 6 mol%), 211 mg of enone **6g** (0.55 mmol), 142 mg of diethyl malonate (0.89 mmol, 1.6 equiv) and filled up with toluene (*ca.* 1.2 mL). The reaction was carried out under pressure of 10–9.5 kbar at 20–25°C for 20 h. Flash column chromatography on a silica gel using hexane fraction from petroleum/DCM as an eluent afforded 243 mg of (*S*)-**7g** (81% yield, 97% conversion) with 91% ee.

Colorless viscous oil (solidified upon standing); 90.8% ee, $[\alpha]_D^{22}$= +87.7 (c 1.18, $CHCl_3$); ^1H NMR (400 MHz, $CDCl_3$) δ 8.25 (ddd, *J* = 8.3, 1.2, 0.6 Hz, 1H), 8.02 (bs, 1H), 7.96 (ddd, *J* = 7.9, 1.3, 0.6 Hz, 1H), 7.84 – 7.74 (m, 3H), 7.67 (d, *J* = 8.9 Hz, 1H), 7.61 – 7.42 (m, 4H), 5.13 (d, *J* = 18.8 Hz, 1H), 4.81 (d, *J* = 18.8 Hz, 1H), 4.71 (s, 1H), 4.36 – 4.23 (m, 2H), 3.96 – 3.83 (m, 2H), 1.32 (t, *J* = 7.1 Hz, 3H), 0.85 (t, *J* = 7.1 Hz, 3H); ^{13}C NMR (100 MHz, $CDCl_3$) δ 190.8 (C=O), 166.4 (C=O), 166.2 (C), 166.0 (C=O), 153.5 (C), 137.6 (C), 132.7 (C), 132.6 (C), 132.1 (C), 129.6 (q, *J* = 286.8 Hz, CF_3), 128.5 (CH), 127.8 (CH), 127.64 (CH), 127.61 (q, *J* = 1.3 Hz, CH), 127.2 (CH), 127.0 (CH), 126.7 (q, *J* = 287 Hz, CF_3), 126.7 (CH), 126.2 (CH), 125.7 (CH), 125.3 (q, *J* = 1.7 Hz, CH), 122.4 (CH), 62.1 (CH_2), 61.8 (CH_2), 55.5 (q, *J* = 1.6 Hz, CH), 53.9 (q, *J* = 25.0 Hz, C-CF_3), 37.5 (q, *J* = 1.3 Hz, CH_2), 13.9 (CH_3), 13.4 (CH_3); ^{19}F NMR (376 MHz, $CDCl_3$) δ -64.1; HRMS (ESI) m/z: $[M + H]^+$ Calcd for $C_{28}H_{25}F_3NO_5S$ 544.1400; Found 544.1400; Enantiomeric excess was determined by HPLC analysis using a Chiralpak® IA column (Hexane/*i*-PrOH: 95/5, flow rate 1.0 mL/min, λ = 219 nm): with catalyst (1*S*,2*S*)-**1a** *ent*-(*S*)-major t_r = 15.5 min and *ent*-(*R*)-minor t_r = 16.9 min.

(*S*)-Diethyl 2-(4-(benzo[d]thiazol-2-yl)-1,1,1-trifluoro-2-(furan-2-yl)-4-oxobutan-2-yl)malonate (7h):

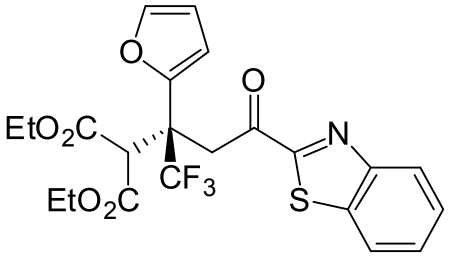

Product **7h** was prepared from enone **6h** according to general high-pressure procedure. A 1.5 mL Teflon ampoule was charged with 16.4 mg of catalyst (1*S*,2*S*)-**1a** (0.040 mmol, 5 mol%), 257 mg of enone **6h** (0.80 mmol), 196 mg of diethyl malonate (1.23 mmol, 1.5 equiv) and filled up with toluene (*ca.* 1.1 mL). The reaction was carried out under pressure of 10–9.5 kbar at 20–25°C for 20 h. Flash column chromatography on a silica gel using hexane fraction from petroleum/DCM as an eluent afforded 317 mg of (*S*)-**7h** (82% yield, 99% conversion) with 82% ee.

Yellow oil; 81.9% ee, $[\alpha]_D^{22}$= +22.7 (c 1.05, $CHCl_3$); ^1H NMR (400 MHz, $CDCl_3$) δ 8.20 (dm, *J* = 8.0 Hz, 1H), 7.96 (dm, *J* = 7.8 Hz, 1H), 7.61 – 7.50 (m, 2H), 7.38 (dd, *J* = 1.8, 0.8 Hz, 1H), 6.53 (d, *J* = 3.4 Hz, 1H), 6.38 (dd, *J* = 3.4, 1.8 Hz, 1H), 4.66 (d, *J* = 17.9 Hz, 1H), 4.65 (s, 1H), 4.54 (d, *J* = 17.9 Hz, 1H), 4.24 (q, *J* = 7.1 Hz, 2H), 4.14 (q, *J* = 7.1 Hz, 2H), 1.28 (t, *J* = 7.1 Hz, 3H), 1.17 (t, *J* = 7.1 Hz, 3H); ^{13}C NMR (100 MHz, $CDCl_3$) δ 190.3 (C=O), 166.15 (C=O), 166.05 (C), 165.9 (C=O), 153.4 (C), 147.7 (C), 142.3 (CH), 137.5 (C), 127.8 (CH), 127.0 (CH), 125.7 (CH), 125.5 (q, *J* = 286.2 Hz, CF_3), 122.3 (CH), 110.7 (CH), 110.1 (q, *J* = 1.4 Hz, CH), 62.0 (CH_2), 61.9 (CH_2), 52.8 (CH), 50.3 (q, *J* = 26.5 Hz, C-CF_3), 37.1 (q, *J* = 1.3 Hz, CH_2), 13.8 (CH_3), 13.7 (CH_3); ^{19}F NMR (376 MHz, $CDCl_3$) δ -69.0; HRMS (ESI) m/z: $[M + H]^+$ Calcd for $C_{22}H_{21}F_3NO_6S$ 484.1036; Found 484.1039; Enantiomeric excess was determined by HPLC analysis using a Chiralpak® IC column (Hexane/*i*-PrOH: 90/10, flow rate 1.0 mL/min, λ = 219 nm): with catalyst (1*S*,2*S*)-**1a** *ent*-(*S*)-major t_r = 14.3 min and *ent*-(*R*)-minor t_r = 18.0 min.

(*R*)-Diethyl 2-(4-(benzo[d]thiazol-2-yl)-1,1,1-trifluoro-4-oxo-2-(thiophen-2-yl)butan-2-yl)malonate (**7i**):

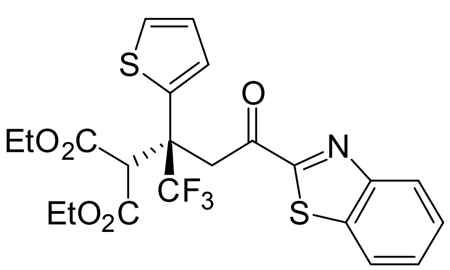

Product **7i** was prepared from enone **6i** according to general high-pressure procedure. A 1.2 mL Teflon ampoule was charged with 16.8 mg of catalyst (1*S*,2*S*)-**1a** (0.041 mmol, 6 mol%), 245 mg of enone **6i** (0.72 mmol), 174 mg of diethyl malonate (1.09 mmol, 1.5 equiv) and filled up with toluene (*ca.* 0.8 mL). The reaction was carried out under pressure of 10–9.5 kbar at 20–25°C for 20 h. Flash column chromatography on a silica gel using hexane fraction from petroleum/DCM as an eluent afforded 312 mg of (*R*)-**7i** (87% yield, 99% conversion) with 83% ee.

Pale yellow oil (solidified upon standing); 82.6% ee, $[\alpha]_D^{22}$= +46.9 (c 1.15, $CHCl_3$); ^1H NMR (400 MHz, $CDCl_3$) δ 8.21 (dm, *J* = 8.0 Hz, 1H), 7.97 (dm, *J* = 8.0 Hz, 1H), 7.62 – 7.50 (m, 2H), 7.32 (d, *J* = 4.7 Hz, 2H), 7.02 – 6.95 (m, 1H), 4.89 (d, *J* = 17.7 Hz, 1H), 4.73 (s, 1H), 4.62 (d, *J* = 17.7 Hz, 1H), 4.25 (q, *J* = 7.1 Hz, 2H), 4.09 (q, *J* = 7.1 Hz, 2H), 1.29 (t, *J* = 7.1 Hz, 3H), 1.13 (t, *J* = 7.1 Hz, 3H); ^{13}C NMR (100 MHz, $CDCl_3$) δ 190.7 (C=O), 166.3 (C=O), 166.04 (C), 166.03 (C=O), 153.4 (C), 137.8 (C), 137.6 (C), 128.3 (q, *J* = 1.5 Hz, CH), 127.9 (CH), 127.0 (CH), 126.3 (CH), 126.0 (q, *J* = 286.4 Hz, CF_3), 125.82 (CH), 125.76 (CH), 122.3 (CH), 62.0 (CH_2), 61.8 (CH_2), 55.1 (CH), 51.9 (q, *J* = 26.3 Hz, C-CF_3), 39.7 (CH_2), 13.8 (CH_3), 13.7 (CH_3); ^{19}F NMR (376 MHz, $CDCl_3$) δ -68.1 (s); HRMS (ESI) m/z: $[M + H]^+$ Calcd for $C_{22}H_{21}F_3NO_5S_2$ 500.0808; Found 500.0810; Enantiomeric excess was determined by HPLC analysis using a Chiralpak® IA column (Hexane/*i*-PrOH: 90/10, flow rate 1.0 mL/min, λ = 219 nm): with catalyst (1*S*,2*S*)-**1a** *ent*-(*R*)-major t_r = 7.0 min and *ent*-(*S*)-minor t_r = 7.7 min.

(*S*)-Diethyl 2-(1-(benzo[d]thiazol-2-yl)-1-oxo-3-(trifluoromethyl)pentan-3-yl)malonate (**7j**):

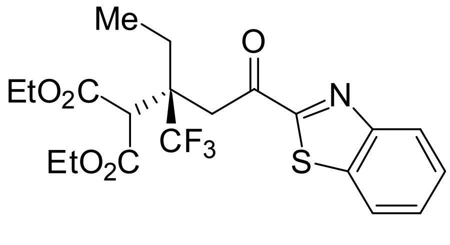

Product **7j** was prepared from enone **6j** according to general high-pressure procedure. A 1.2 mL Teflon ampoule was charged with 14.0 mg of catalyst (1*S*,2*S*)-**1a** (0.034 mmol, 5 mol%), 202 mg of enone **6j** (0.71 mmol), 175 mg of diethyl malonate (1.09 mmol, 1.5 equiv) and filled up with toluene (*ca.* 0.8 mL). The reaction was carried out under pressure of 10–9.5 kbar at 20–25°C for 20 h. Flash column chromatography on a silica gel using hexane fraction from petroleum/DCM as an eluent afforded 253 mg of (*S*)-**7j** (80% yield, 99% conversion) with 97% ee.

Colorless oil; 96.9% ee, $[\alpha]_D^{22}$= –7.2 (c 1.20, $CHCl_3$); ^1H NMR (400 MHz, $CDCl_3$) δ 8.20 (dm, *J* = 8.0 Hz, 1H), 7.97 (dm, *J* = 8.0 Hz, 1H), 7.61 – 7.49 (m, 2H), 4.45 (s, 1H), 4.28 (d, *J* = 17.4 Hz, 1H), 4.25 – 4.15 (m, 4H), 3.90 (d, *J* = 17.4 Hz, 1H), 2.38 – 2.26 (m, 1H), 2.19 (dq, *J* = 14.9, 7.5 Hz, 1H), 1.27 (t, *J* = 7.1 Hz, 3H), 1.25 (t, *J* = 7.1 Hz, 3H), 1.09 (t, *J* = 7.5 Hz, 3H); ^{13}C NMR (100 MHz, $CDCl_3$) δ 192.2 (C=O), 167.0 (C=O), 166.9 (C=O), 166.3 (C), 153.4 (C), 137.5 (C), 127.8 (CH), 127.4 (q, *J* = 286.6 Hz, CF_3), 127.0 (CH), 125.7 (CH), 122.3 (CH), 61.74 (CH_2), 61.69 (CH_2), 52.0 (q, *J* = 1.3 Hz, CH), 48.9 (q, *J* = 23.6 Hz, C-CF_3), 36.5 (q, *J* = 1.9 Hz, CH_2), 24.8 (q, *J* = 1.6 Hz, CH_2), 13.82 (CH_3), 13.80 (CH_3), 8.8 (CH_3); ^{19}F NMR (376 MHz, $CDCl_3$) δ -68.6 (s); HRMS (ESI) m/z: $[M + H]^+$ Calcd for $C_{20}H_{23}F_3NO_5S$ 446.1244; Found 446.1244; Enantiomeric excess was determined by HPLC analysis using a Chiralpak® IA column (Hexane/*i*-PrOH: 98/2, flow rate 1.0 mL/min, λ = 219 nm): with catalyst (1*S*,2*S*)-**1a** *ent*-(*S*)-major t_r = 7.9 min and *ent*-(*R*)-minor t_r = 8.6 min.

(*S*)-Diethyl 2-(1-(benzo[d]thiazol-2-yl)-1-oxo-5-phenyl-3-(trifluoromethyl)pentan-3-yl)malonate (7k):

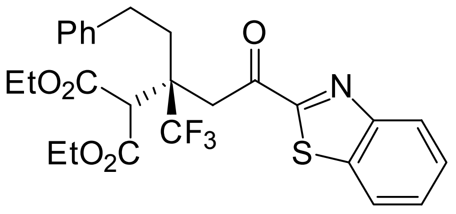

Product **7k** was prepared from enone **6k** according to general high-pressure procedure. A 1.2 mL Teflon ampoule was charged with 12.4 mg of catalyst (1*S*,2*S*)-**1a** (0.030 mmol, 5 mol%), 207 mg of enone **6k** (0.57 mmol), 143 mg of diethyl malonate (0.89 mmol, 1.6 equiv) and filled up with toluene (*ca.* 0.9 mL). The reaction was carried out under pressure of 10–9.5 kbar at 20–25°C for 20 h. Flash column chromatography on a silica gel using hexane fraction from petroleum/DCM as an eluent afforded 251 mg of (*S*)-**7k** (84% yield, 99% conversion) with 95% ee.

Colorless oil; 94.6% ee, $[\alpha]_D^{22}$= +35.5 (c 1.03, $CHCl_3$); ^{1}H NMR (400 MHz, $CDCl_3$) δ 8.21 (dm, *J* = 8.0 Hz, 1H), 7.97 (dm, *J* = 8.0 Hz, 1H), 7.61 – 7.51 (m, 2H), 7.32 – 7.16 (m, 5H), 4.50 (s, 1H), 4.36 (d, *J* = 17.4 Hz, 1H), 4.27 – 4.16 (m, 4H), 4.04 (d, *J* = 17.4 Hz, 1H), 2.92 – 2.73 (m, 2H), 2.61 – 2.49 (m, 1H), 2.43 – 2.31 (m, 1H), 1.27 (t, *J* = 7.0 Hz, 3H), 1.25 (t, *J* = 7.1 Hz, 3H); ^{13}C NMR (100 MHz, $CDCl_3$) δ 192.0 (C=O), 166.9 (C=O), 166.8 (C=O), 166.3 (C), 153.4 (C), 141.2 (C), 137.6 (C), 128.5 (2 x CH), 128.4 (2 x CH), 127.9 (CH), 127.3 (q, *J* = 286.5 Hz, CF_3), 127.0 (CH), 126.1 (CH), 125.7 (CH), 122.4 (CH), 61.9 (CH_2), 61.8 (CH_2), 52.1 (q, *J* = 0.9 Hz, CH), 48.9 (q, *J* = 23.7 Hz, <u>C</u>-CF_3), 36.9 (CH_2), 33.9 (CH_2), 30.6 (CH_2), 13.9 (CH_3), 13.8 (CH_3); ^{19}F NMR (376 MHz, $CDCl_3$) δ -69.0 (s); HRMS (ESI) m/z: $[M + H]^+$ Calcd for $C_{26}H_{27}F_3NO_5S$ 522.1557; Found 522.1560; Enantiomeric excess was determined by HPLC analysis using a Chiralpak® IA column (Hexane/*i*-PrOH: 95/5, flow rate 1.0 mL/min, λ = 219 nm): with catalyst (1*S*,2*S*)-**1a** *ent*-(*S*)-major t_r = 8.8 min and *ent*-(*R*)-minor t_r = 13.6 min.

(*R*)-Diethyl 2-(1-chloro-1,1-difluoro-4-oxo-2-phenyl-4-(thiazol-2-yl)butan-2-yl)malonate (9a):

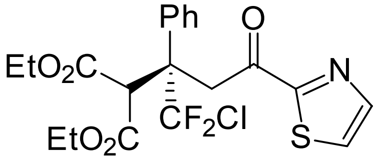

Product **9a** was prepared from enone **8a** according to general high-pressure procedure. A 1.0 mL Teflon ampoule was charged with 23.0 mg of catalyst (1*R*,2*R*)-**1b** (0.051 mmol, 5 mol%), 299 mg of enone **8a** (1.00 mmol), 234 mg of diethyl malonate (1.46 mmol, 1.5 equiv) and filled up with toluene (*ca.*0.5 mL). The reaction was carried out under pressure of 10–9.5 kbar at 20–25°C for 20 h. Flash column chromatography on a silica gel using hexane fraction from petroleum/DCM as an eluent afforded 263 mg of (*R*)-**9a** (57% yield) with 92% ee.

Colorless viscous oil (solidified upon standing); 91.6% ee, $[\alpha]_D^{26}$= –40.1 (c 0.61, $CHCl_3$); ^{1}H NMR (400 MHz, $CDCl_3$) δ 8.05 (d, *J* = 3.0 Hz, 1H), 7.69 (d, *J* = 3.0 Hz, 1H), 7.59 – 7.53 (m, 2H), 7.36 – 7.30 (m, 3H), 4.94 (d, *J* = 18.6 Hz, 1H), 4.69 (s, 1H), 4.65 (d, *J* = 18.6 Hz, 1H), 4.29 – 4.19 (m, 2H), 4.04 – 3.91 (m, 2H), 1.28 (t, *J* = 7.1 Hz, 3H), 0.97 (t, *J* = 7.1 Hz, 3H); ^{13}C NMR (100 MHz, $CDCl_3$) δ 189.2 (C=O), 167.0 (C), 166.7 (C=O), 166.0 (C=O), 144.7 (CH), 134.9 (C), 132.1 (t, *J* = 302.5 Hz, CF_2Cl), 128.7 (t, *J* = 2.0 Hz, 2 x CH), 128.2 (CH), 127.6 (2 x CH), 126.7 (CH), 61.9 (CH_2), 61.7 (CH_2), 58.2 (t, *J* = 19.9 Hz, <u>C</u>-CF_2Cl), 54.1 (CH), 37.4 (CH_2), 13.8 (CH_3), 13.4 (CH_3); ^{19}F NMR (376 MHz, $CDCl_3$) δ -51.0 (d, *J* = 165.3 Hz, 1F), -52.4 (d, *J* = 165.3 Hz, 1F); HRMS (ESI) m/z: $[M + H]^+$ Calcd for $C_{20}H_{21}ClF_2NO_5S$ 460.0792; Found 460.0786; Enantiomeric excess was determined by HPLC analysis using a Chiralpak® IC column (Hexane/*i*-PrOH: 90/10, flow rate 1.0 mL/min, λ = 219 nm): with catalyst (1*R*,2*R*)-**1b** *ent*-(*S*)-minor t_r = 11.2 min and *ent*-(*R*)-major t_r = 15.4 min.

Diethyl 2-(1,1,1,2,2-pentafluoro-5-oxo-3-phenyl-5-(thiazol-2-yl)pentan-3-yl)malonate (9b):

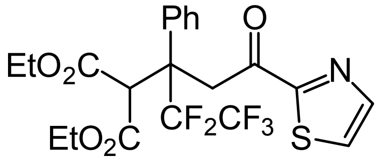

Product **9b** was prepared from enone **8b** according to general high-pressure procedure. A 0.5 mL Teflon ampoule was charged with 21.1 mg of catalyst **1b** (0.046 mmol, 10 mol%), 158 mg of enone **8b** (0.47 mmol), 107 mg of diethyl malonate (0.669 mmol, 1.5 equiv) and filled up with toluene (*ca.*0.3 mL). The reaction was carried out under pressure of 10–9.5 kbar at 20–25°C for 72 h. Flash column chromatography on a silica gel using hexane fraction from petroleum/DCM as an eluent afforded 69 mg of **9b** (30% yield; ee was not determined).

Colorless viscous oil, ^{1}H NMR (400 MHz, $CDCl_3$) δ 8.07 (d, *J* = 3.0 Hz, 1H), 7.70 (d, *J* = 3.0 Hz, 1H), 7.52 – 7.45 (m, 2H), 7.35 – 7.28 (m, 3H), 4.97 (d, *J* = 18.8 Hz, 1H), 4.71 (s, 1H), 4.51 (d, *J* = 19.1 Hz, 1H), 4.28 – 4.17 (m, 2H), 4.05 – 3.91 (m, 2H), 1.26 (t, *J* = 7.1 Hz, 3H), 0.93 (t, *J* = 7.2 Hz, 3H); ^{13}C NMR (100 MHz, $CDCl_3$) δ 188.5 (C=O), 167.0 (C), 166.8 (C=O), 165.9 (C=O), 144.8 (CH), 132.6 (*J* = 4.1 Hz, C), 128.8 (d, *J* = 2.6 Hz, 2 x CH), 128.4 (CH), 127.6 (2 × CH), 126.5 (CH), 122 – 113 (m, CF_2CF_3), 61.8 (CH_2), 61.6 (CH_2), 52.8 (t, *J* = 17.3 Hz, C-CF_2), 52.3 (d, *J* = 6.0 Hz, CH), 35.7 (m, CH_2), 13.8 (CH_3), 13.4 (CH_3); ^{19}F NMR (376 MHz, $CDCl_3$) δ -76.15 (s, 3F), -110.0 (d, *J* = 273.4 Hz, 1F), -113.9 (d, *J* = 273.4 Hz, 1F); HRMS (ESI) m/z: $[M + H]^+$ Calcd for $C_{21}H_{21}F_5NO_5S$ 494.1055; Found 494.1058.

Analytical data of compounds 10a-10g obtained from adduct 3h

(*R*)-5-Oxo-3-phenyl-5-(thiazol-2-yl)-3-(trifluoromethyl)pentanoic acid (10a):

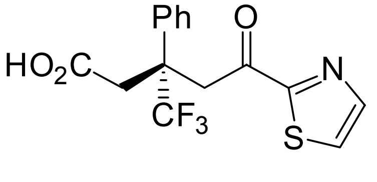

Adduct (*R*)-**3h** (289 mg, 0.65 mmol, 98% ee) and 10 mL of 6M HCl (aq) were placed in a sealed tube and heated using a thermoblock at 105°C for 24h with stirring. After cooling the reaction mixture to room temperature, product was extracted with chloroform and concentrated under reduced pressure. The residue was purified by column chromatography on a silica gel with DCM/MeOH (→95:5) to give title compound **10a** (206 mg, 92% yield) as a colorless oil.

$[\alpha]_D^{22}$= –47.1 (c=1.0, $CHCl_3$); ^{1}H NMR (400 MHz, $CDCl_3$) δ 10.4 (bs, 1H), 8.02 (d, *J* = 3.0 Hz, 1H), 7.67 (d, *J* = 3.0 Hz, 1H), 7.53 – 7.44 (m, 2H), 7.40 – 7.29 (m, 3H), 4.56 (d, *J* = 18.1 Hz, 1H), 4.25 (d, *J* = 18.0 Hz, 1H), 3.62 (s, 2H); ^{13}C NMR (100 MHz, $CDCl_3$) δ 189.8 (C=O), 175.6 (CO_2H), 166.9 (C), 144.7 (CH), 136.2 (C), 128.6 (2 × CH), 128.4 (CH), 126.9 (2 × CH), 126.8 (CH), 126.7 (q, *J* = 284.2 Hz, CF_3), 48.3 (q, *J* = 25.1 Hz, C-CF_3), 37.2 (CH_2), 34.6 (CH_2); ^{19}F NMR (376 MHz, $CDCl_3$) δ -73.7 (s); HRMS (ESI) m/z: $[M + H]^+$ Calcd for $C_{15}H_{13}F_3NO_3S$ 344.0563; Found 344.0564.

(*R*)-Ethyl 5-oxo-3-phenyl-5-(thiazol-2-yl)-3-(trifluoromethyl)pentanoate (10b):

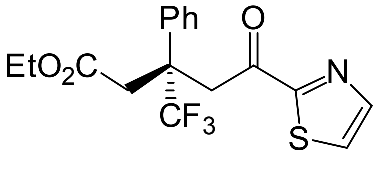

To 170 mg of carboxylic acid **10a** (0.5 mmol) in 10 mL of EtOH were added 15 drops of concentrated H_2SO_4 and the solution was refluxed using heating mantle for 12 hours. The solvent was evaporated under reduced pressure, and the crude product was diluted with toluene and purified by column chromatography on a silica gel (hexane fraction from petroleum/AcOEt, 5%→20% of AcOEt) to give 158 mg of title compound **10b** (85%) as a white solid with 97% ee.

$[\alpha]_D^{26}$= –47.1 (c=1.08, $CHCl_3$); ^{1}H NMR (400 MHz, $CDCl_3$) δ 8.03 (d, *J* = 3.0 Hz, 1H), 7.67 (d, *J* = 3.0 Hz, 1H), 7.53 – 7.45 (m, 2H), 7.41 – 7.29 (m, 3H), 4.59 (d, *J* = 18.3 Hz, 1H), 4.28 (d, *J* = 18.3 Hz, 1H), 4.15 – 4.03 (m, 2H), 3.59, 3.54 (ABq, *J* = 15.5 Hz, 2H), 1.18 (t, *J* = 7.1 Hz, 3H); ^{13}C NMR (100 MHz, $CDCl_3$) δ 189.6 (C=O), 170.1 (CO_2), 167.0 (C), 144.7 (CH), 136.5 (q, *J* = 1.0 Hz, C), 128.5 (2 × CH), 128.2 (CH), 127.0 (2 × CH), 126.8 (q, *J* = 284.2 Hz, CF_3), 126.6 (CH), 60.8 (CH_2), 48.3 (q, *J* = 24.8 Hz, C-CF_3), 37.6 (q, *J* = 1.6 Hz, CH_2), 34.9 (q, *J* = 1.8 Hz, CH_2), 13.9 (CH_3); ^{19}F NMR (376 MHz,$CDCl_3$) δ -73.7 (s); HRMS (ESI) m/z: $[M + H]^+$ Calcd for $C_{17}H_{17}F_3NO_3S$ 372.0876; Found 372.0876; Enantiomeric excess was determined by HPLC analysis using a Chiralpak® IB column (Hexane/i-PrOH 90/10, flow rate 1.0mL/min, λ = 219 nm): ent-(R)-major t_r = 6.4 min and ent-(S)-minor t_r = 7.3 min.

(*S*)-4-Phenyl-6-(thiazol-2-yl)-4-(trifluoromethyl)-3,4-dihydro-2H-pyran-2-one (10c):

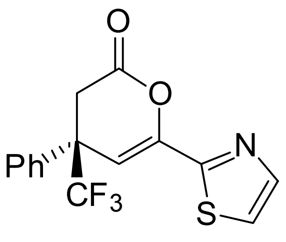

To 345 mg of carboxylic acid (*R*)-**10a** (1 mmol) in dry CH_2Cl_2 (20 mL) were added oxalyl chloride (0.26 mL, 3 mmol) and DMF (3 drops) at 0 °C under argon atmosphere. After 40 min of stirring, the mixture was warmed to rt and stirred for another 12 hours. Then, the solution was concentrated in vacuo and the residue was purified by silica-gel column chromatography (hexane fraction from petroleum/AcOEt, 5%→20% of AcOEt) to give 320 mg of the product **10c** (98% yield) as a pale yellow oil with 99% ee.

$[\alpha]_D^{22}$= –14.3 (c=0.98, $CHCl_3$); ^{1}H NMR (400 MHz, $CDCl_3$) δ 7.93 (d, *J* = 3.2 Hz, 1H), 7.54 – 7.49 (m, 2H), 7.47 (d, *J* = 3.2 Hz, 1H), 7.44 – 7.35 (m, 3H), 6.91 (d, *J* = 1.1 Hz, 1H), 3.46 (dd, *J* = 15.9, 1.1 Hz, 1H), 3.33 (d, *J* = 15.9 Hz, 1H); ^{13}C NMR (100 MHz, $CDCl_3$) δ 163.6 (C=O), 159.3 (C), 147.1 (C), 144.3 (CH), 134.3 (C), 129.3 (CH), 129.1 (2 × CH), 127.4 (q, *J* = 1.1 Hz, 2 × CH), 125.8 (q, *J* = 283.1 Hz, CF_3), 121.1 (CH), 101.6 (q, *J* = 2.3 Hz, CH), 48.2 (q, *J* = 27.8 Hz, C-CF_3), 34.9 (q, *J* = 2.2 Hz, CH_2); ^{19}F NMR (376 MHz, $CDCl_3$) δ -74.4 (s); HRMS (ESI) m/z: $[M + H]^+$ Calcd for $C_{15}H_{11}F_3NO_2S$ 326.0457; Found 326.0458; Enantiomeric excess was determined by HPLC analysis using a Chiralpak® IC column (Hexane/i-PrOH 80/20, flow rate 1.0mL/min, λ = 219 nm): ent-(S)-major t_r =5.9 min and ent-(R)-minor t_r =6.7 min.

(2*S*)-Diethyl 2-phenyl-3-(thiazole-2-carbonyl)-2-(trifluoromethyl)cyclopropane-1,1-dicarboxylate (*cis/trans* mixture, **10d**): [39]

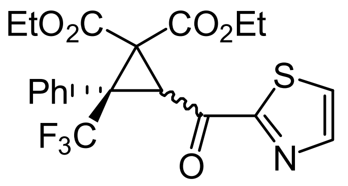

To 100 mg of compound (*R*)-**3h** (0.226 mmol) in anhydrous toluene (10mL) was added 90 mg iodine (0.354mmol) and then 177 mg of DBU (1.16 mmol) was added dropwise at room temperature. After stirring at room temperature for 30 minutes, the reaction mixture was quenched by aqueous $Na_2S_2O_3$ and extracted with ethyl acetate. The combinate extracts were dried over anhydrous $MgSO_4$ and evaporated to dryness. The crude product was purified by silica-gel column chromatography (hexane fraction from petroleum/AcOEt, 5%→20% of AcOEt) to give 90 mg of mixture of diastereoisomers (*cis/trans*) **10d** (90% yield, 7:3 dr) as a colorless oil. (*cis/trans* diastereomers could not be separated).

^{1}H NMR (400 MHz, $CDCl_3$) δ 8.14 (d, *J* = 3.0 Hz, 1H, major), 8.10 (d, *J* = 3.0 Hz, 1H, minor), 7.76 (d, *J* = 3.0 Hz, 1H, major), 7.73 (d, *J* = 3.0 Hz, 1H, minor), 7.50 – 7.24 (m, Ph, major + minor), 4.49 (s, 1H, major), 4.43 (s, 1H, minor), 4.46 – 4.30 (m, OCH_2, major + minor), 4.02 – 3.89 (m, OCH_2, major + minor), 1.36 (t, *J* = 7.1 Hz, CH_3, minor), 1.35 (t, *J* = 7.1 Hz, CH_3, major), 1.06 (t, *J* = 7.1 Hz, CH_3, major), 0.98 (t, *J* = 7.1 Hz, CH_3, minor); ^{13}C NMR (100 MHz, $CDCl_3$) δ 184.3 (C=O, minor), 183.1 (C=O, major), 166.4 (C, minor), 166.0 (C, major), 164.6 (C, major), 164.4 (C, minor), 163.34 (C, minor), 163.31 (C, major), 145.3 (CH, minor), 145.2 (CH, major), 133.0 (CH, major), 132.5 (C, major), 130.5 (bs, 2 × CH, major), 129.2 (2 × CH, minor), 129.0 (CH, minor), 128.4 (bs, 2 × CH, major), 127.3 (2 × CH, minor), 127.1 (CH, minor), 126.9 (CH, major), 126.5 (C, minor), 123.8 (q, *J* = 277.9 Hz, CF_3, minor), 123.4 (q, *J* = 278.4 Hz, CF_3, major), 62.8 (CH_2, minor), 62.7 (CH_2, major), 62.2 (CH_2, major), 62.1 (CH_2, minor), 46.0 (q, *J* = 32.7 Hz, C-CF_3, minor), 44.3 (q, *J* = 34.3 Hz, C-CF_3, major), 44.1 (C, minor), 43.5 (C, major), 37.2 (CH, major), 33.5 (q, *J* = 2.4 Hz, CH, minor), 13.7 (CH_3, major + minor), 13.6 (CH_3, major), 13.4 (CH_3, minor); ^{19}F NMR (376 MHz, $CDCl_3$) δ -59.4 (s, major), -67.5 (s, minor); HRMS (ESI) m/z: $[M + H]^+$ Calcd for $C_{20}H_{19}F_3NO_5S$ 442.0931; Found 442.0928; Enantiomeric excess was determinated by HPLC analysis using a Chiralpak® ID column (Hexane/i-PrOH 90/10, flow rate 1.0mL/min, λ = 219 nm): ent-(R)-minor t_r = 10.08 min, 15.29 min and ent-(S)-major t_r = 9.07 min, 13.57 min.

(*S*)-4-Phenyl-6-(thiazol-2-yl)-4-(trifluoromethyl)-3,4-dihydropyridin-2(1H)-one (10e):

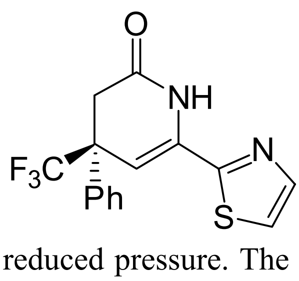

Adduct (*R*)-**3h** (360 mg, 0.81 mmol, 98% ee) and NH_4OAc (1.52 g, 19.7 mmol) in 9 mL of AcOH/toluene (1:1 mixture) was heated in a sealed tube using a thermoblock at 105 °C for 36 h. Then, the reaction mixture was cooled, $CHCl_3$ was added and resulting solution transformed to the flask. Solvent was removed under reduced pressure, and the residue was treated with saturated $NaHCO_3$ and extracted with AcOEt. The organic layer was dried over Na_2CO_3, and the solvent was removed under reduced pressure. The crude product was purified by silica-gel column chromatography (hexane fraction from petroleum/AcOEt, 5%→20% of AcOEt) to give 220 mg of the **10e** (84% yield) as a colorless oil with 98.7% ee.

$[\alpha]_D^{22}$= -149.2 (c=1.02, $CHCl_3$); ^{1}H NMR (400 MHz, $CDCl_3$) δ 8.28 (bs, 1H, NH), 7.79 (d, *J* = 3.2 Hz, 1H), 7.54 – 7.48 (m, 2H), 7.42 – 7.33 (m, 3H), 7.39 (d, *J* = ~3.1 Hz, 1H), 6.21 (t, *J* = 1.4 Hz, 1H), 3.31 (d, *J* = 16.2 Hz, 1H), 3.18 (d, *J* = 16.2 Hz, 1H); ^{13}C NMR (100 MHz, CDC_3) δ 166.5 (C=O), 160.5 (C), 143.0 (CH), 135.3 (C), 133.4 (C), 128.9 (CH), 128.8 (2 × CH), 127.5 (q, *J* = 1.1 Hz, 2 × CH), 126.3 (q, *J* = 282.8 Hz, CF_3), 120.3 (CH), 103.4 (q, *J* = 2.5 Hz, CH), 49.1 (q, *J* = 27.0 Hz, C-CF_3), 36.5 (q, *J* = 2.0 Hz, CH_2); ^{19}F NMR (376 MHz, $CDCl_3$) δ -74.1 (s);

[39] Miao, C.-B.; Zhang, M.; Tian, Z.-Y.; Xi, H.-T.; Sun, X.-Q.; Yang, H.-T. *J. Org. Chem.* **2011**, *76*, 9809 – 9816.

HRMS (ESI) m/z: $[M + H]^+$ Calcd for $C_{15}H_{12}F_3N_2OS$ 325.0617; Found 325.0619; Enantiomeric excess was determined by HPLC analysis using a Chiralpak® IC column (Hexane/i-PrOH 80/20, flow rate 1.0mL/min, λ = 219 nm): ent-(S)-major t_r = 13.0 min and ent-(R)-minor t_r = 16.4 min.

(*S*)-6-Oxo-4-phenyl-4-(trifluoromethyl)-1,4,5,6-tetrahydropyridine-2-carbaldehyde (10f): [40]

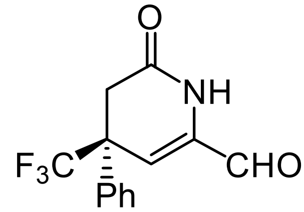

98.7% ee; ^{1}H NMR (400 MHz, $CDCl_3$) δ 9.42 (s, 1H), 7.74 (bs, 1H), 7.52 – 7.33 (m, 5H), 6.47 (s, 1H), 3.32 (d, *J* = 16.4 Hz, 1H), 3.14 (d, *J* = 16.4 Hz, 1H); ^{13}C NMR (100 MHz, $CDCl_3$) δ 184.3 (CHO), 165.8 (C=O), 138.4 (C), 133.9 (C), 129.3 (CH), 129.1 (2 × CH), 127.4 (q, *J* = 0.9 Hz, 2 × CH), 125.9 (q, *J* = 282.7 Hz, CF_3), 120.3 (q, *J* = 2.3 Hz, =CH), 49.5 (q, *J* = 27.5 Hz, C-CF_3), 36.1 (q, *J* = 2.0 Hz, CH_2); ^{19}F NMR (376 MHz, $CDCl_3$) δ -73.9 (s); HRMS (ESI) m/z: $[M + H]^+$ Calcd for $C_{13}H_{12}F_3NO_2$ 270.0736; Found 270.0737; Enantiomeric excess was determined by HPLC analysis using a Chiralpak® IC column (Hexane/i-PrOH 70/30, flow rate 1.0 mL/min, λ = 219 nm): ent-(S)-major t_r =15.6 min and ent-(R)-minor t_r =20.5 min.

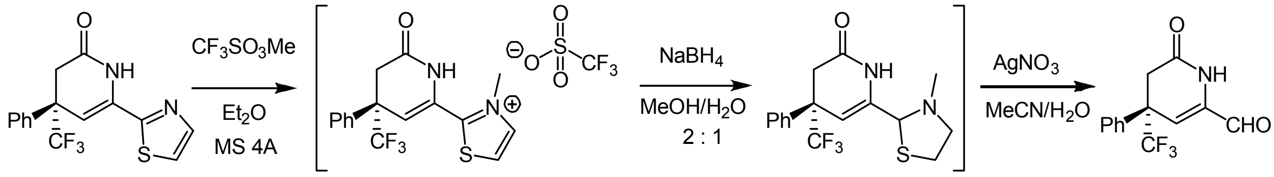

To stirred solution of compound (*S*)-**10e** (181 mg, 0.56 mmol) in Et_2O (3.5 mL) were added molecular sieves (4Å, 350mg). Then 220 μL of methyl triflate (2 mmol) was added dropwise. After stirring at room temperature for 24 hours, the reaction mixture was filtred through Cellite and evaporated. The obtained *N*-methyl thiazolium salt was used without purification in the next step.

^{1}H NMR (400 MHz, $(CD_3)_2SO$) δ 10.20 (s, 1H), 8.58 (d, *J* = 3.8 Hz, 1H), 8.45 (d, *J* = 3.8 Hz, 1H), 7.68 – 7.60 (m, 2H), 7.52 – 7.41 (m, 3H), 6.58 (s, 1H), 4.12 (s, 3H), 3.42, 3.37 (ABq, *J* = 16.2 Hz, 2H); ^{13}C NMR (100 MHz, $(CD_3)_2SO$) δ 166.9 (C=O), 162.0 (C), 139.4 (CH), 134.7 (C), 129.2 (CH), 128.9 (2 × CH), 127.9 (2 × CH), 126.9 (C), 126.3 (q, *J* = 283.0 Hz, CF_3), 126.2 (CH), 120.7 (q, *J* = 322.3 Hz, $CF_3SO_3^-$), 112.8 (CH), 48.8 (q, *J* = 26.4 Hz, C-CF_3), 40.7 (CH_3), 34.8 (CH_2); ^{19}F NMR (376 MHz, $(CD_3)_2SO$) δ -73.4 (s), -77.8 (s).

The *N*-methyl thiazolium salt was dissolved in 54 mL of H_2O:MeOH (1:2) and 390 mg of $NaBH_4$ (10.3 mmol) was added in portions. After stirring at room temperature for 30 min, acetone (12 mL) was added and the solution was concentrated in vacuo and extracted with AcOEt. The combinate extracts were dried over anhydrous $MgSO_4$ and evaporated to dryness. The residue was dissolved in acetonitrile (18 mL), and the solution of $AgNO_3$ (196 mg, 1.15 mmol) in water (1.8 mL) was added with vigorous stirring. After stirring for 20 min. the acetonitrile was evaporated in vacuo and the residue was extracted with DCM. The organic phase was dried over $MgSO_4$, the solvent was evaporated, and the residue was purified by silica-gel column chromatography (hexane fraction from petroleum/AcOEt, 5%→20% of AcOEt) to give the product **10f** (83 mg, 55% yield) as a white solid.

[40] (a) Dondoni, A.; Giovannini, P. P.; Perrone, D. *J. Org. Chem.* **2002**, *67*, 7203–7214. (b) Dondoni, A.; Perrone D. *Org. Synth.* **2000**, *77*, 78.

(*S*,*E*)-Ethyl 3-(6-oxo-4-phenyl-4-(trifluoromethyl)-1,4,5,6-tetrahydropyridin-2-yl)acrylate (10g):

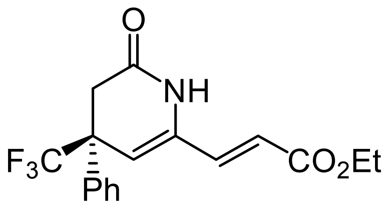

A solution of aldehyde **10f** (34 mg, 0.126 mmol) and ethyl (triphenylphosphoranylidene)acetate (57 mg, 0.164 mmol) in $CHCl_3$ (4mL) was heated using a thermoblock at 50 °C in a sealed test tube for 12 h. Then, the mixture was cooled, $CHCl_3$ was removed under reduced pressure, and the residue was purified by silica-gel column chromatography (hexane fraction from petroleum/AcOEt 8:2) to give the product **10g** (31 mg, 72% yield) as a colorless oil with 98.8% ee.

^{1}H NMR (400 MHz, $CDCl_3$) δ 8.73 (bs, 1H), 7.48 – 7.40 (m, 2H), 7.38 – 7.31 (m, 3H), 7.20 (d, *J* = 16.1 Hz, 1H), 6.22 (d, *J* = 16.1 Hz, 1H), 5.93 (s, 1H), 4.26 (q, *J* = 7.1 Hz, 2H), 3.25 (d, *J* = 16.2 Hz, 1H), 3.09 (d, *J* = 16.2 Hz, 1H), 1.33 (t, *J* = 7.1 Hz, 3H); ^{13}C NMR (100 MHz, $CDCl_3$) δ 168.7 (C=O), 165.9 (C=O), 136.7 (=CH), 135.8 (C), 134.9 (C), 128.9 (CH), 128.8 (2 × CH), 127.6 (2 × CH), 126.2 (q, *J* = 283.2 Hz, CF_3), 119.5 (=CH), 112.4 (q, *J* = 2.0 Hz, =CH), 61.0 (CH_2), 48.9 (q, *J* = 27.0 Hz, <u>C</u>-CF_3), 36.1 (q, *J* = 2.0 Hz, CH_2), 14.2 (CH_3); ^{19}F NMR (376 MHz, $CDCl_3$) δ -74.3 (s, 93% of *E*-isomer) and -74.27 (s, 7% of *Z*-isomer); HRMS (ESI) m/z: $[M + H]^+$ Calcd for $C_{17}H_{17}F_3NO_3$ 340.1155; Found 340.1154; Enantiomeric excess was determinated by HPLC analysis using a Chiralpak® IC column (Hexane/i-PrOH 90/10, flow rate 1.0mL/min, λ = 219 nm): ent-(R)-minor t_r = 20.6 min and ent-(S)-major t_r = 23.1 min.

HPLC chromatograms: 3b-3l, 5a-5k, 5m-5u, 7a-7k, 9a, 10b-10g

3b

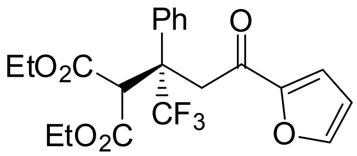

CHIRALPAK® IA
Hexane/*i*-PrOH: 98/2; flow rate 1.0 mL/min
219 nm

RACEMATE

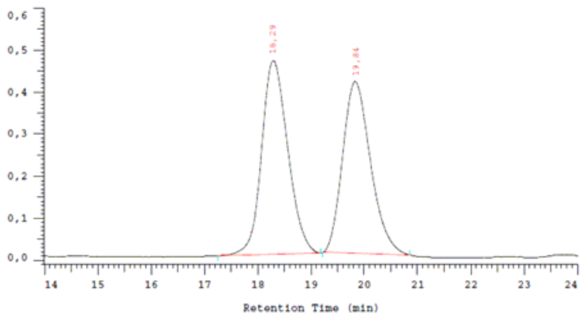

RT	Area	Conc 1
18,29	7843614	51,215
19,84	7471462	48,785
	15315076	100,000

95.3% ee

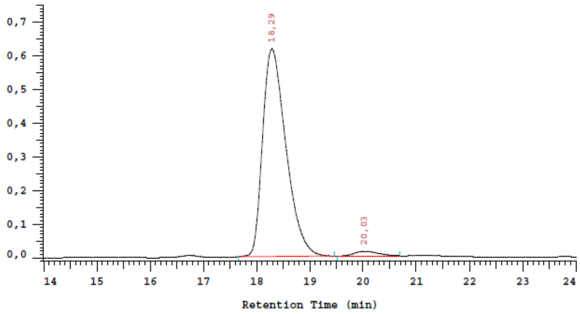

RT	Area	Conc 1
18,29	9262177	97,630
20,03	224872	2,370
	9487049	100,000

3c

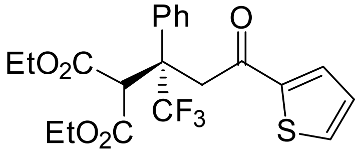

CHIRALPAK® IA
Hexane/*i*-PrOH: 98/2; flow rate 1.0 mL/min
219 nm

RACEMATE

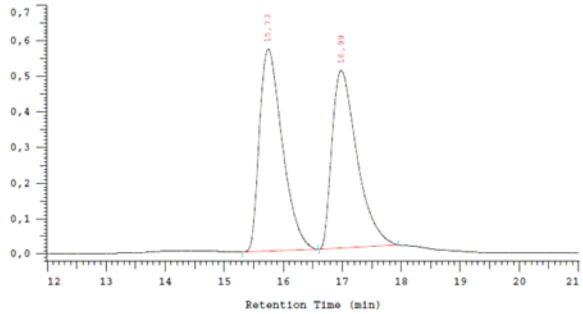

RT	Area	Conc 1
15,73	7639660	50,897
16,99	7370281	49,103
	15009941	100,000

99.5% ee

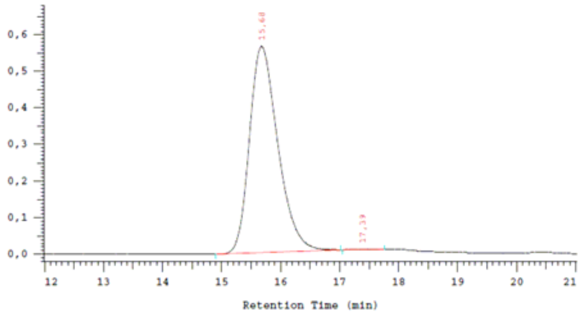

RT	Area	Conc 1
15,68	9394192	99,757
17,39	22915	0,243
	9417107	100,000

3d

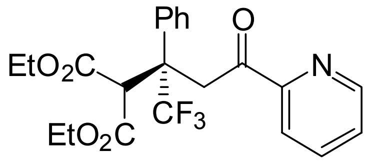

CHIRALPAK® IA
Hexane/*i*-PrOH: 98/2; flow rate 1.0 mL/min
219 nm

RACEMATE

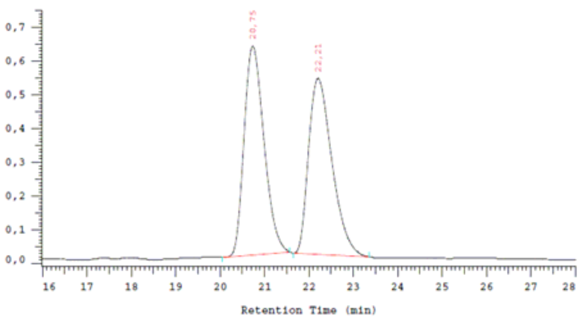

95.2% ee

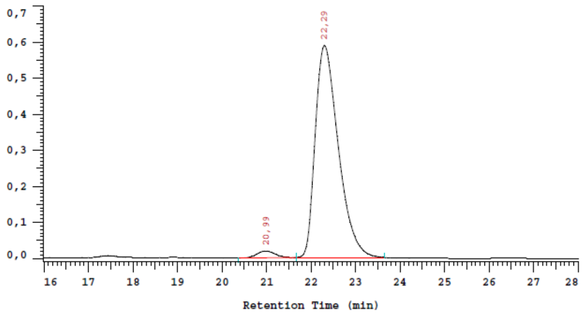

RT	Area	Conc 1
20,75	9634356	50,248
22,21	9539183	49,752
	19173539	100,000

RT	Area	Conc 1
20,99	258542	2,392
22,29	10550840	97,608
	10809382	100,000

3e

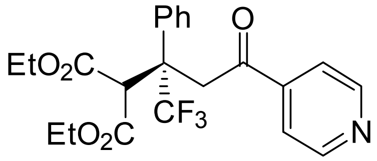

CHIRALPAK® IC
Hexane/*i*-PrOH: 85/15; flow rate 1.0 mL/min
219 nm

RACEMATE

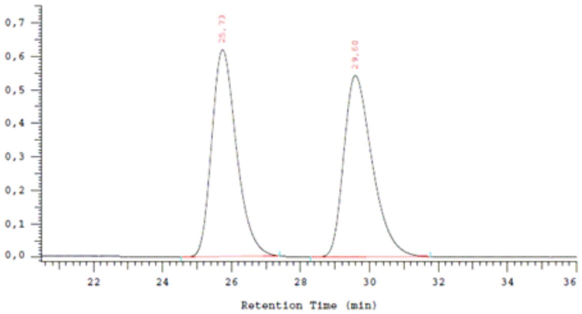

96.0% ee

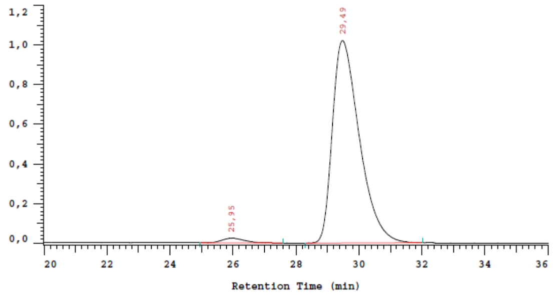

RT	Area	Conc 1
25,73	15428600	49,771
29,60	15570403	50,229
	30999003	100,000

RT	Area	Conc 1
25,95	614698	1,978
29,49	30469353	98,022
	31084051	100,000

3f

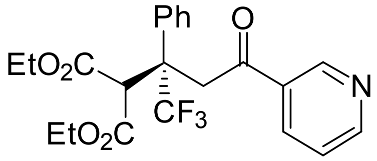

CHIRALPAK® IC
Hexane/*i*-PrOH: 85/15; flow rate 1.0 mL/min
219 nm

RACEMATE

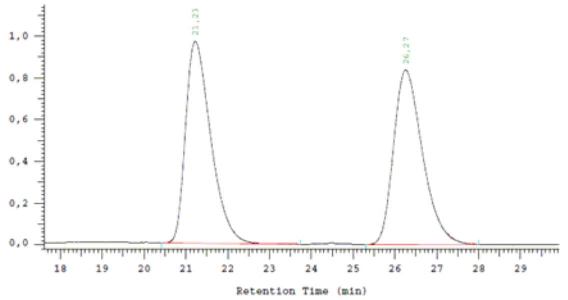

RT	Area	Conc 1
21,23	20193686	50,020
26,27	20177414	49,980
	40371100	100,000

94.1% ee

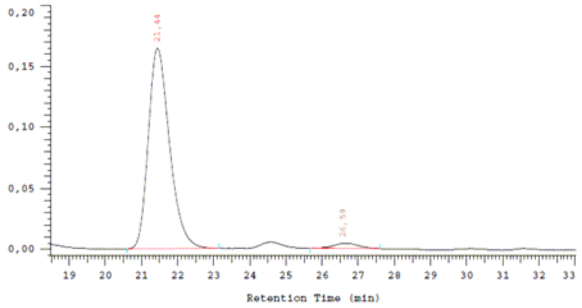

RT	Area	Conc 1
21,44	3347141	97,046
26,59	101867	2,954
	3449008	100,000

3g

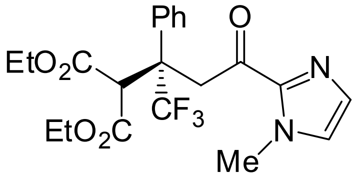

CHIRALPAK® IC
Hexane/*i*-PrOH: 95/5; flow rate 1.0 mL/min
219 nm

RACEMATE

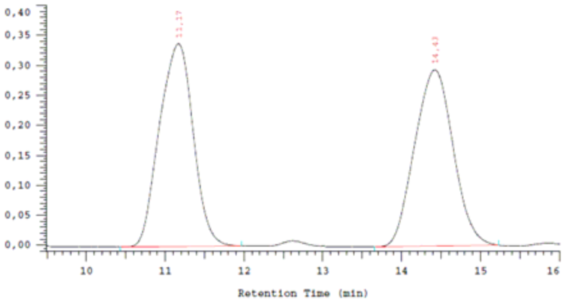

RT	Area	Conc 1
11,17	5047938	50,306
14,43	4986544	49,694
	10034482	100,000

74.6% ee

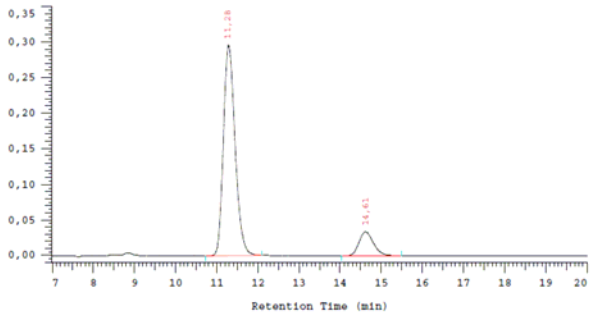

RT	Area	Conc 1
11,28	2914744	87,303
14,61	423915	12,697
	3338659	100,000

3h

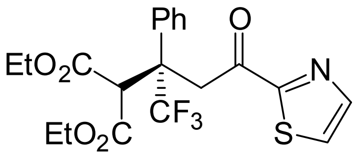

CHIRALPAK® IC
Hexane/*i*-PrOH: 90/10; flow rate 1.0 mL/min
219 nm

RACEMATE

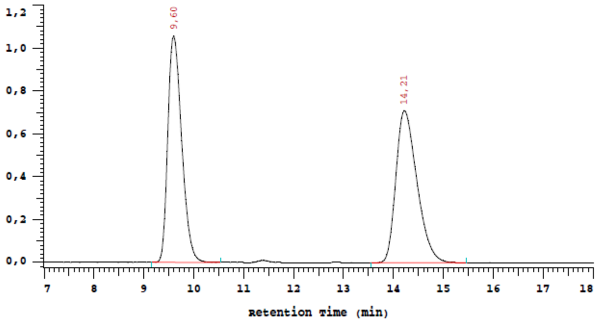

RT	Area	Conc 1
9,60	10227788	49,621
14,21	10383896	50,379
	20611684	100,000

92.0% ee

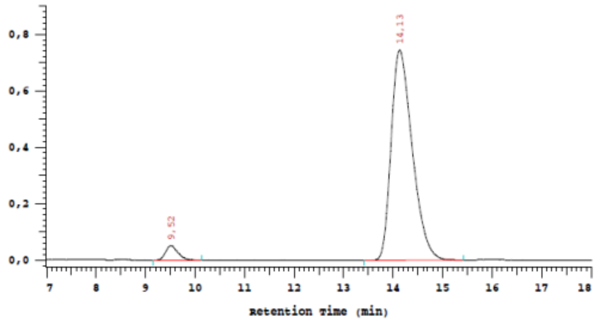

RT	Area	Conc 1
9,52	457092	3,981
14,13	11023811	96,019
	11480903	100,000

After single crystallization (from EtOH) ee >98%

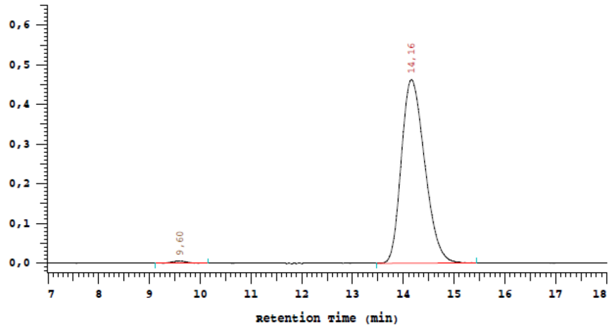

RT	Area	Conc 1
9,60	61572	0,826
14,16	7391726	99,174
	7453298	100,000

3i

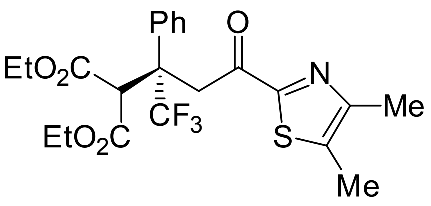

CHIRALPAK® IA
Hexane/*i*-PrOH: 98/2; flow rate 1.0 mL/min
219 nm

RACEMATE

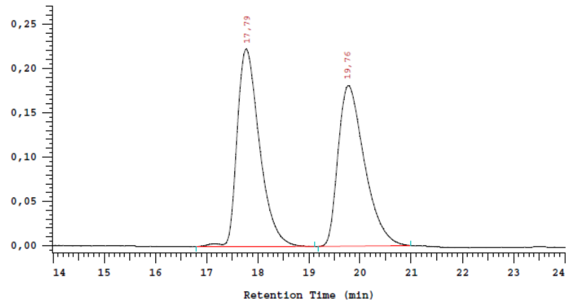

95.1% ee

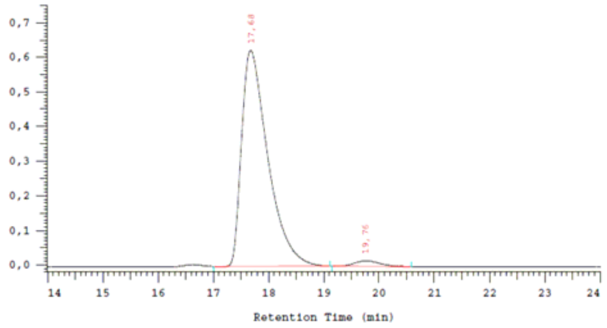

RT	Area	Conc 1
17,79	3389802	51,069
19,76	3247925	48,931
	6637727	100,000

RT	Area	Conc 1
17,68	10325414	97,568
19,76	257393	2,432
	10582807	100,000

3j

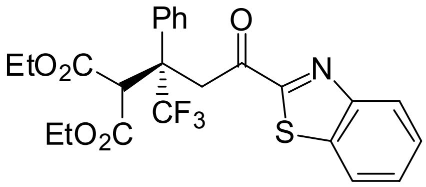

CHIRALPAK® IC
Hexane/*i*-PrOH: 95/5; flow rate 1.0 mL/min
219 nm

RACEMATE

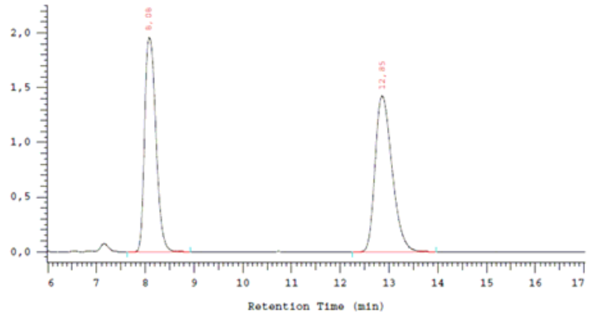

91.0% ee

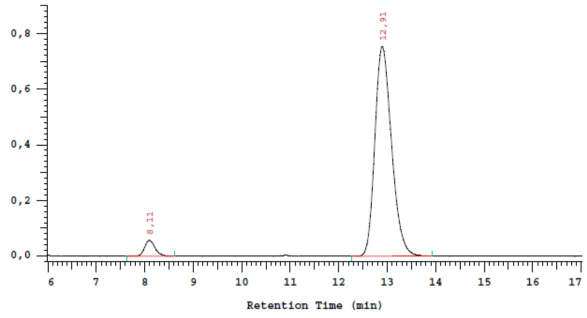

RT	Area	Conc 1
8,08	15710644	47,710
12,85	17218825	52,290
	32929469	100,000

RT	Area	Conc 1
8,11	423217	4,496
12,91	8989168	95,504
	9412385	100,000

3k

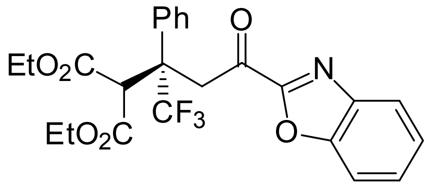

CHIRALPAK® IA
Hexane/*i*-PrOH: 93/7; flow rate 1.0 mL/min
219 nm

RACEMATE

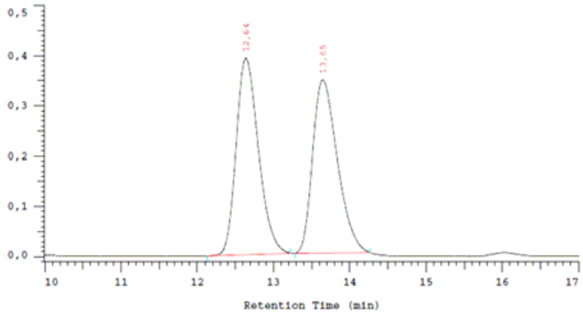

91.1% ee

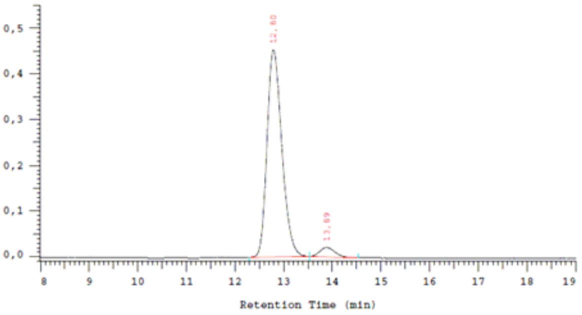

RT	Area	Conc 1
12,64	3977968	50,667
13,65	3873178	49,333
	7851146	100,000

RT	Area	Conc 1
12,80	4785253	95,568
13,89	221941	4,432
	5007194	100,000

3l

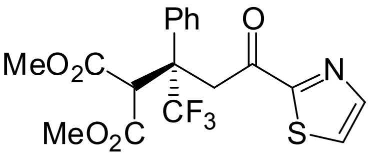

CHIRALPAK® IA
Hexane/*i*-PrOH: 95/5; flow rate 1.0 mL/min
219 nm

RACEMATE

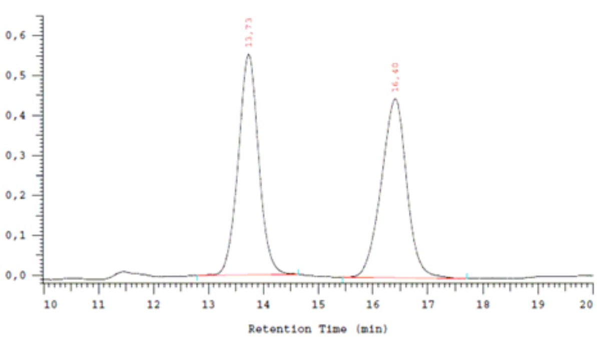

87.0% ee

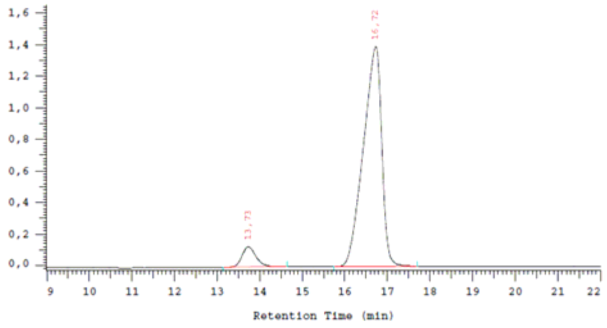

RT	Area	Conc 1
13,73	7149069	49,722
16,40	7229008	50,278
	14378077	100,000

RT	Area	Conc 1
13,73	1454390	6,490
16,72	20955286	93,510
	22409676	100,000

5a

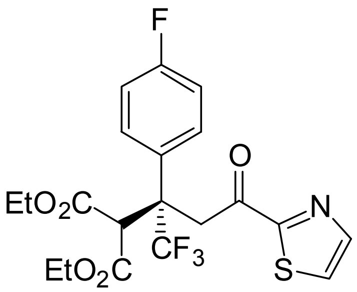

CHIRALPAK® IC
Hexane/*i*-PrOH: 90/10; flow rate 1.0 mL/min
219 nm

RACEMATE

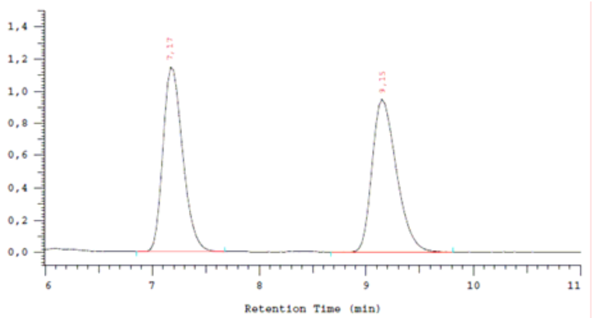

RT	Area	Conc 1
7,17	7333788	49,578
9,15	7458704	50,422
	14792492	100,000

95.0% ee

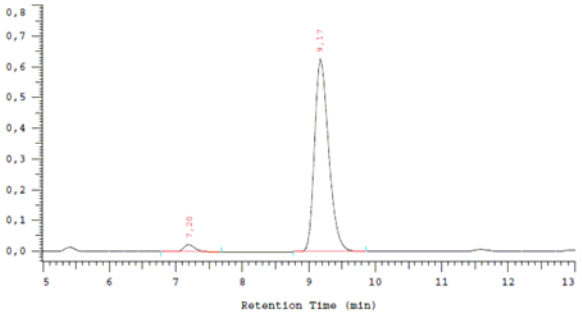

RT	Area	Conc 1
7,20	118622	2,513
9,17	4601060	97,487
	4719682	100,000

5b

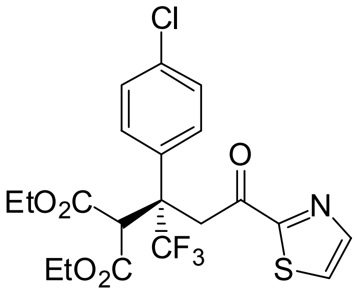

CHIRALPAK® IC
Hexane/*i*-PrOH: 90/10; flow rate 1.0 mL/min
219 nm

RACEMATE

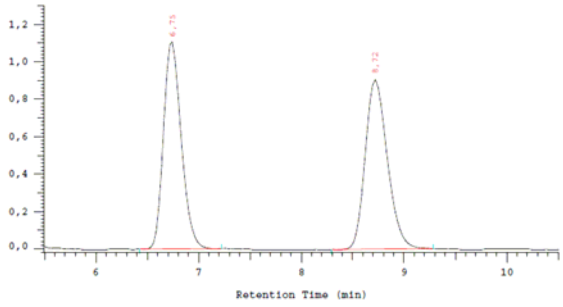

RT	Area	Conc 1
6,75	6665480	49,679
8,72	6751608	50,321
	13417088	100,000

89.2% ee

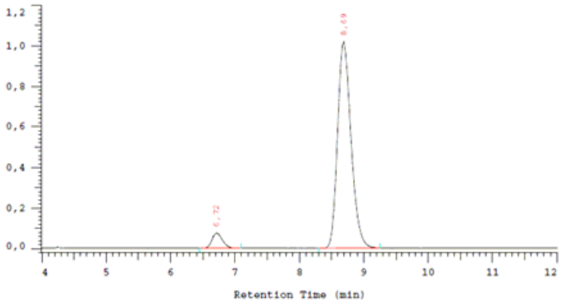

RT	Area	Conc 1
6,72	423398	5,378
8,69	7448795	94,622
	7872193	100,000

5c

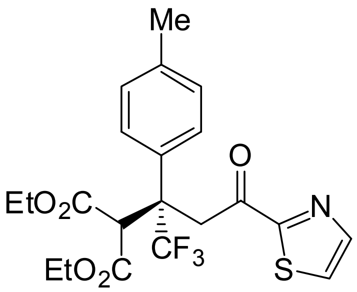

CHIRALPAK® IC
Hexane/*i*-PrOH: 90/10; flow rate 1.0 mL/min
219 nm

RACEMATE

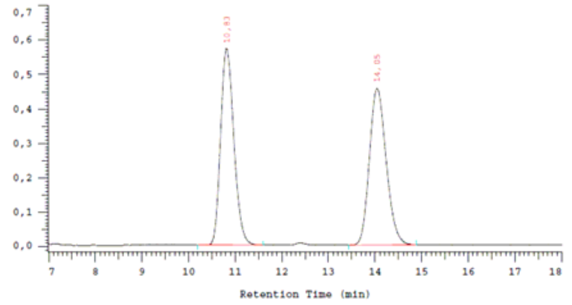

91.1% ee

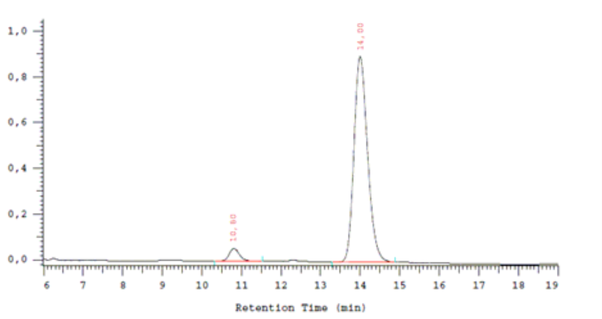

RT	Area	Conc 1
10,83	5635670	49,823
14,05	5675822	50,177
	11311492	100,000

RT	Area	Conc 1
10,80	494267	4,428
14,00	10666911	95,572
	11161178	100,000

5d

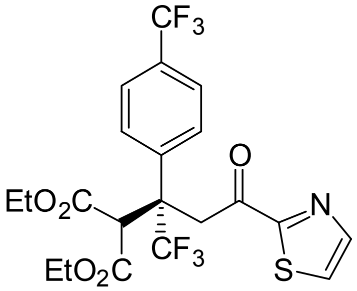

CHIRALPAK® IC
Hexane/*i*-PrOH: 98/2; flow rate 1.0 mL/min
219 nm

RACEMATE

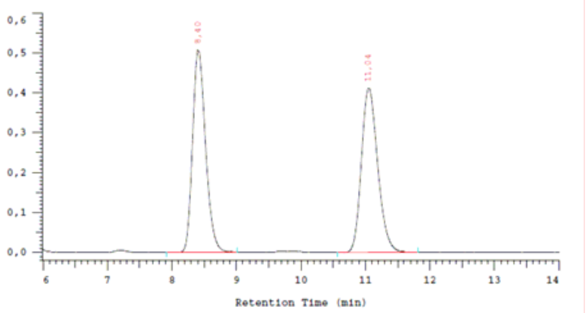

88.7% ee

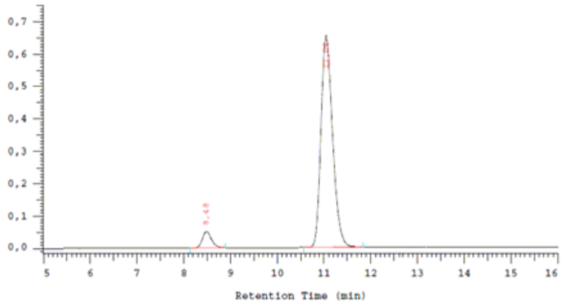

RT	Area	Conc 1
8,40	3521598	49,402
11,04	3606907	50,598
	7128505	100,000

RT	Area	Conc 1
8,48	336029	5,653
11,04	5608124	94,347
	5944153	100,000

5e

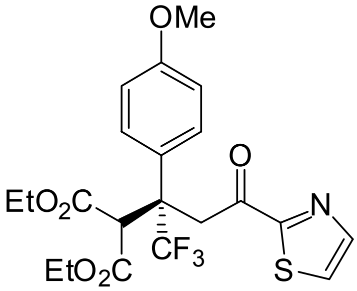

CHIRALPAK® IC
Hexane/*i*-PrOH: 90/10; flow rate 1.0 mL/min
219 nm

RACEMATE

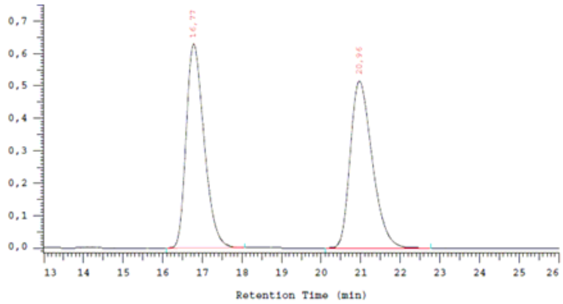

RT	Area	Conc 1
16,77	9866895	49,905
20,96	9904656	50,095
	19771551	100,000

92.0% ee

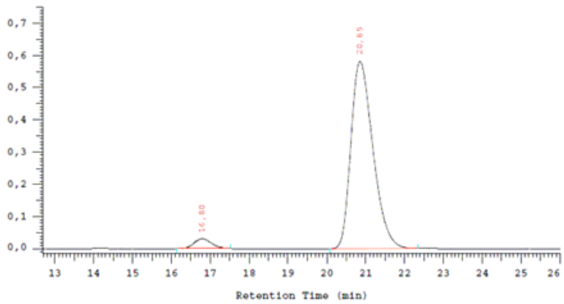

RT	Area	Conc 1
16,80	476984	4,010
20,85	11418537	95,990
	11895521	100,000

5f

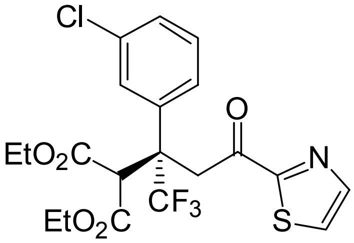

CHIRALPAK® IC
Hexane/*i*-PrOH: 90/10; flow rate 1.0 mL/min
219 nm

RACEMATE

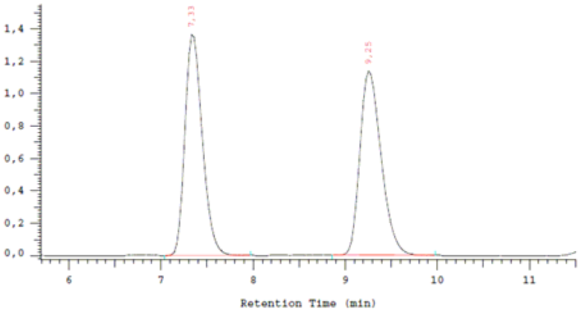

RT	Area	Conc 1
7,33	8947378	49,668
9,25	9066920	50,332
	18014298	100,000

90.2% ee

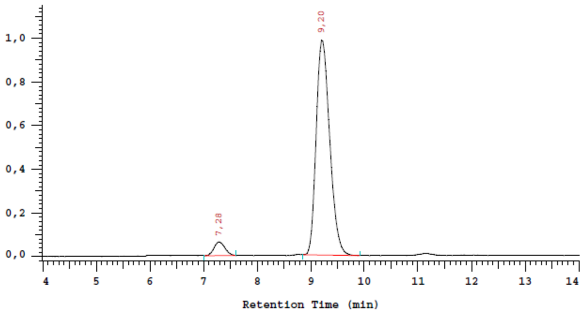

RT	Area	Conc 1
7,28	442892	4,885
9,20	8622938	95,115
	9065830	100,000

5g

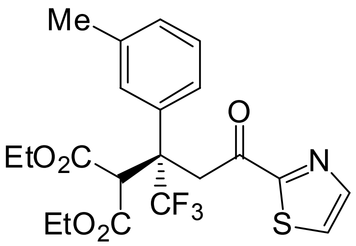

CHIRALPAK® IC
Hexane/*i*-PrOH: 90/10; flow rate 1.0 mL/min
219 nm

RACEMATE

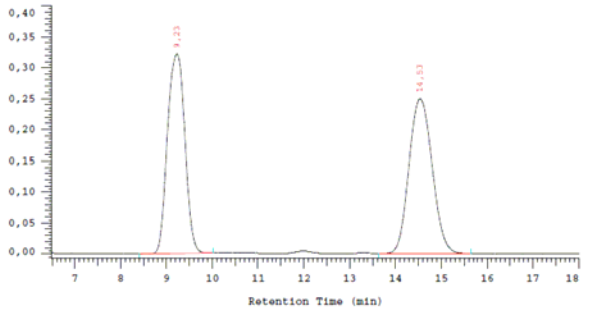

92.7% ee

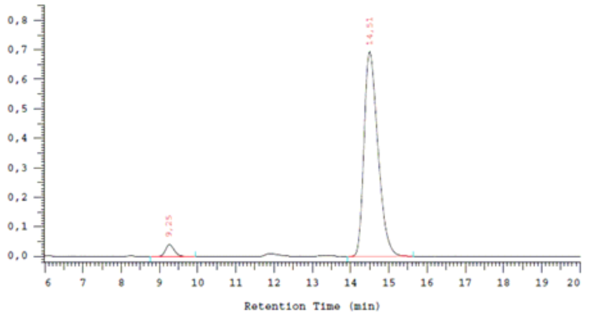

RT	Area	Conc 1
9,23	4257850	49,110
14,53	4412140	50,890
	8669990	100,000

RT	Area	Conc 1
9,25	331767	3,648
14,51	8762168	96,352
	9093935	100,000

5h

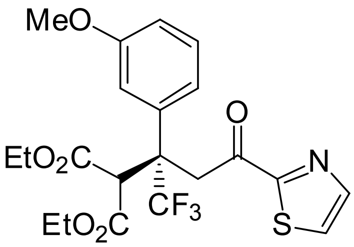

CHIRALPAK® IC
Hexane/*i*-PrOH: 90/10; flow rate 1.0 mL/min
219 nm

RACEMATE

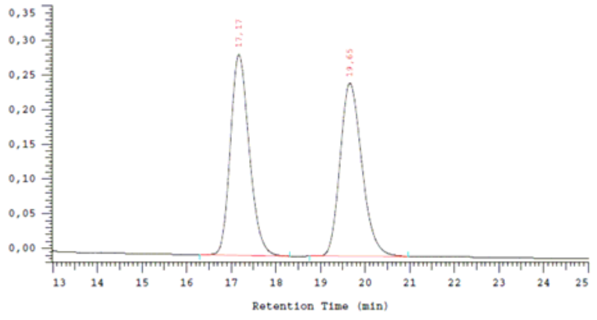

93.0% ee

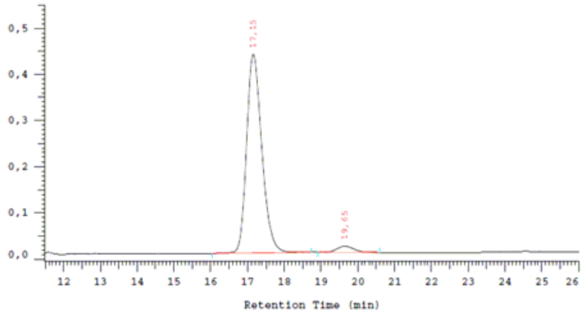

RT	Area	Conc 1
17,17	4259994	50,141
19,65	4236117	49,859
	8496111	100,000

RT	Area	Conc 1
17,15	6186613	96,493
19,65	224827	3,507
	6411440	100,000

5i

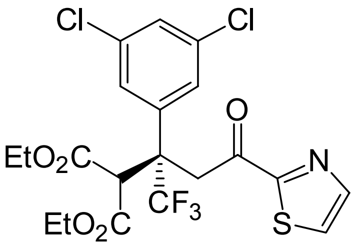

CHIRALPAK® IC
Hexane/*i*-PrOH: 90/10; flow rate 1.0 mL/min
219 nm

RACEMATE

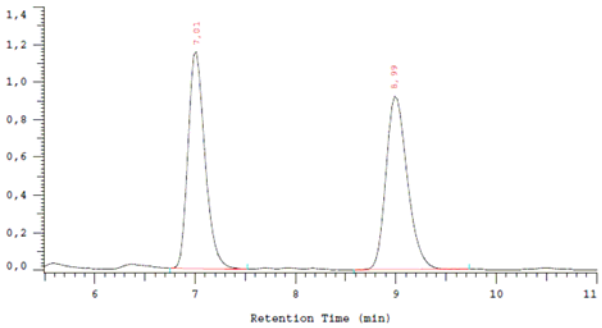

RT	Area	Conc 1
7,01	6748834	49,844
8,99	6791042	50,156
	13539876	100,000

91.9% ee

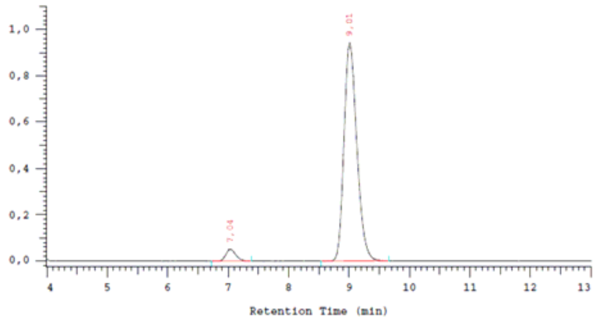

RT	Area	Conc 1
7,04	292994	4,052
9,01	6938339	95,948
	7231333	100,000

5j

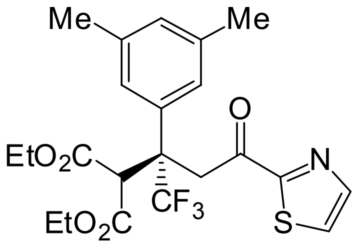

CHIRALPAK® IC
Hexane/*i*-PrOH: 90/10; flow rate 1.0 mL/min
219 nm

RACEMATE

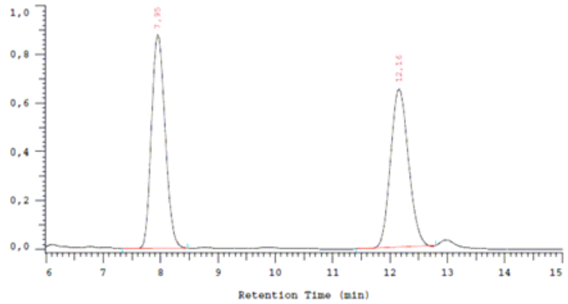

RT	Area	Conc 1
7,95	7232098	50,679
12,16	7038400	49,321
	14270498	100,000

84.5% ee

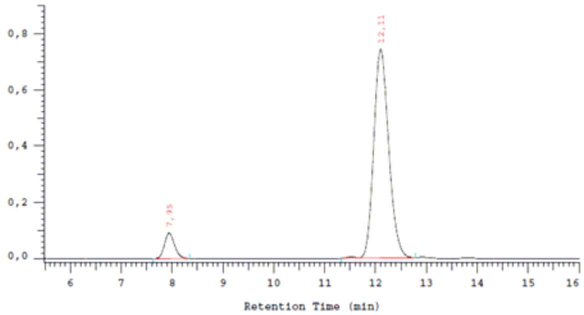

RT	Area	Conc 1
7,95	635057	7,727
12,11	7583443	92,273
	8218500	100,000

5k

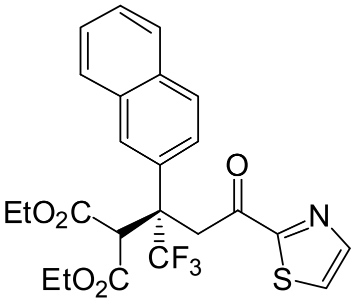

CHIRALPAK® IC
Hexane/*i*-PrOH: 90/10; flow rate 1.0 mL/min
219 nm

RACEMATE

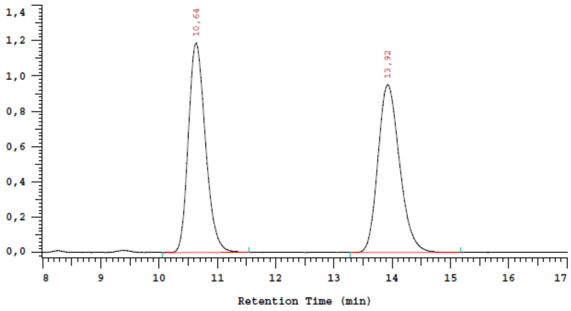

RT	Area	Conc 1
10,64	12033987	49,502
13,92	12275908	50,498
	24309895	100,000

93.0% ee

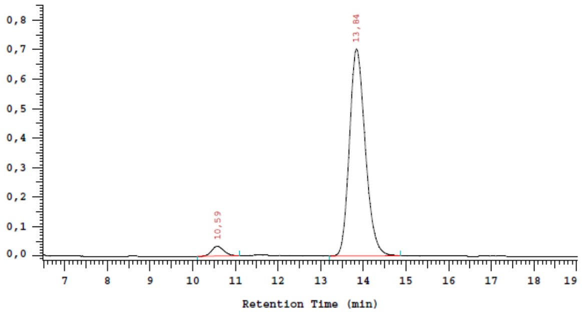

RT	Area	Conc 1
10,59	328684	3,481
13,84	9113683	96,519
	9442367	100,000

5m

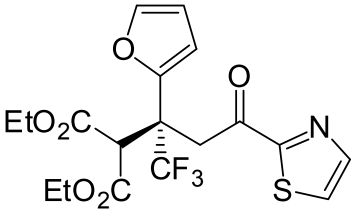

CHIRALPAK® IC
Hexane/*i*-PrOH: 90/10; flow rate 1.0 mL/min
219 nm

RACEMATE

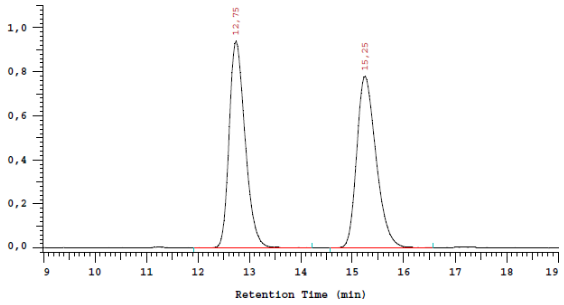

RT	Area	Conc 1
12,75	10091424	49,903
15,25	10130538	50,097
	20221962	100,000

88.0% ee

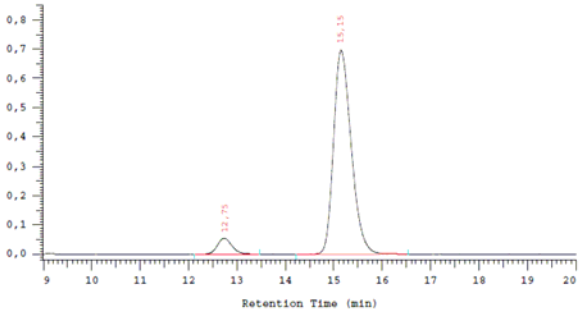

RT	Area	Conc 1
12,75	564722	6,023
15,15	8811361	93,977
	9376083	100,000

5n

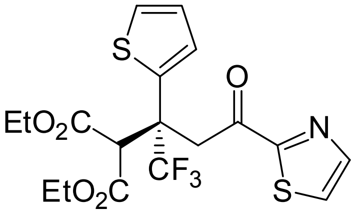

CHIRALPAK® IC
Hexane/*i*-PrOH: 90/10; flow rate 1.0 mL/min
219 nm

RACEMATE

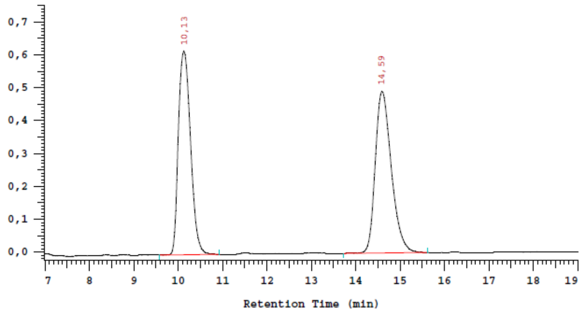

RT	Area	Conc 1
10,13	5889233	48,932
14,59	6146419	51,068
	12035652	100,000

87.5% ee

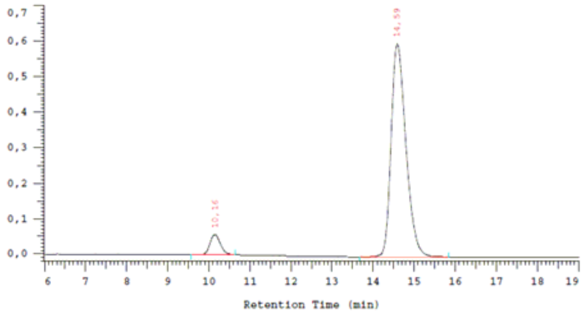

RT	Area	Conc 1
10,16	500563	6,247
14,59	7512484	93,753
	8013047	100,000

5o

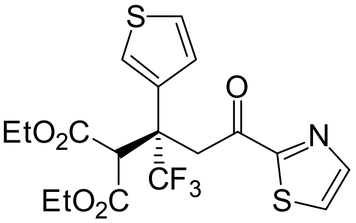

CHIRALPAK® IC
Hexane/*i*-PrOH: 90/10; flow rate 1.0 mL/min
219 nm

RACEMATE

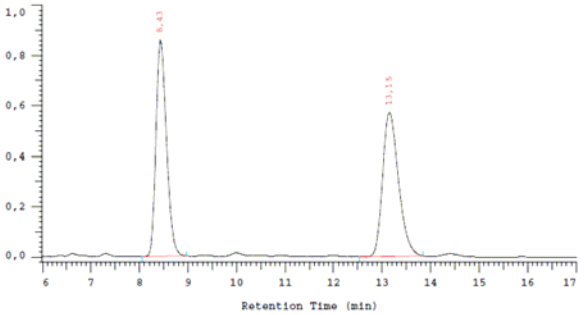

RT	Area	Conc 1
8,43	6433968	49,835
13,15	6476656	50,165
	12910624	100,000

87.2% ee

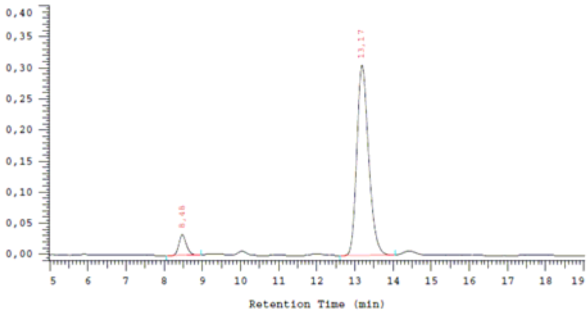

RT	Area	Conc 1
8,48	226293	6,396
13,17	3311770	93,604
	3538063	100,000

5p

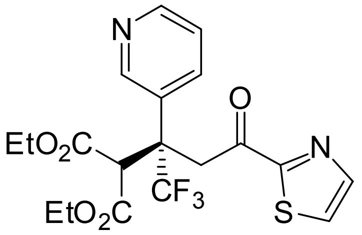

CHIRALPAK® IC
Hexane/*i*-PrOH: 80/20; flow rate 1.0 mL/min
219 nm

RACEMATE

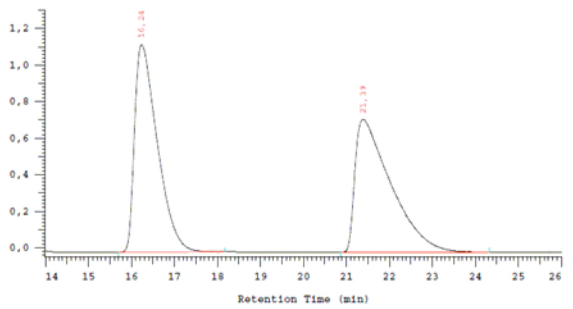

89.2% ee

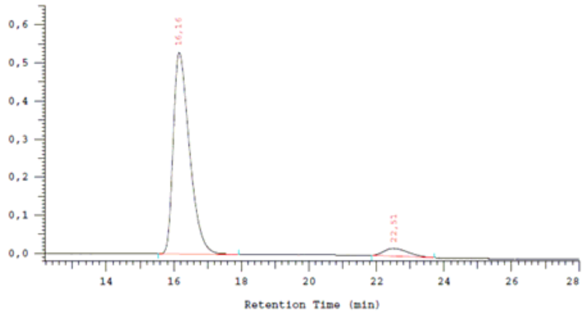

RT	Area	Conc 1
16,24	20834704	49,798
21,39	21004030	50,202
	41838734	100,000

RT	Area	Conc 1
16,16	8905276	94,611
22,51	507263	5,389
	9412539	100,000

5q

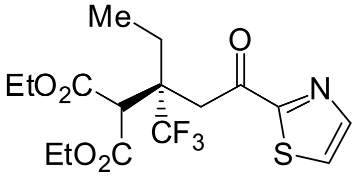

CHIRALPAK® ID
Hexane/*i*-PrOH: 98/2; flow rate 1.0 mL/min
219 nm

RACEMATE

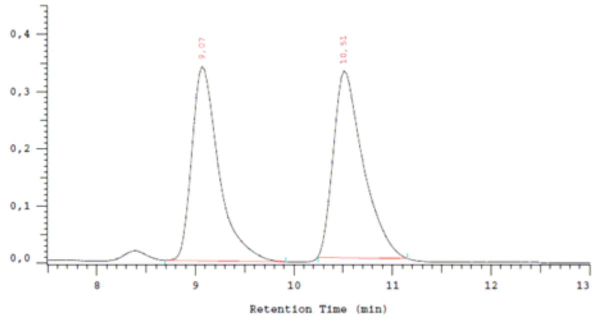

93.9% ee

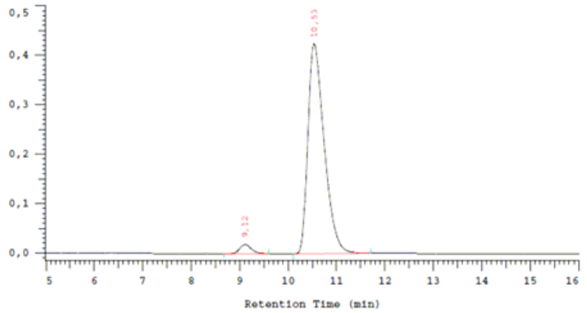

RT	Area	Conc 1
9,07	3219802	48,381
10,51	3435238	51,619
	6655040	100,000

RT	Area	Conc 1
9,12	156498	3,074
10,53	4934983	96,926
	5091481	100,000

5r

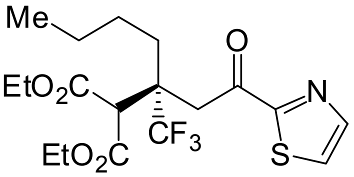

CHIRALPAK® ID
Hexane/*i*-PrOH: 98/2; flow rate 1.0 mL/min
219 nm

RACEMATE

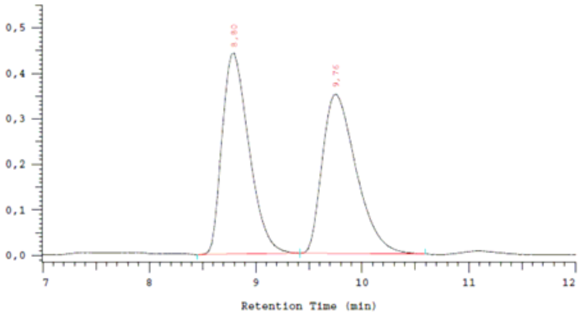

87.6% ee

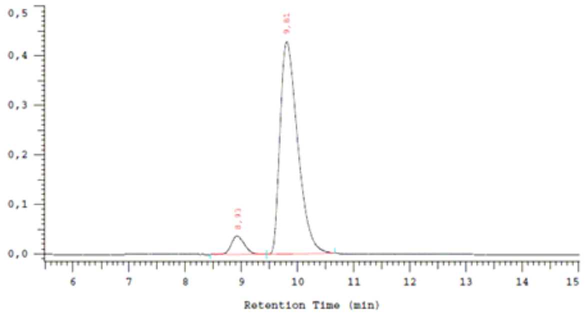

RT	Area	Conc 1
8,80	3879085	50,031
9,76	3874276	49,969
	7753361	100,000

RT	Area	Conc 1
8,93	308680	6,191
9,81	4677215	93,809
	4985895	100,000

5s

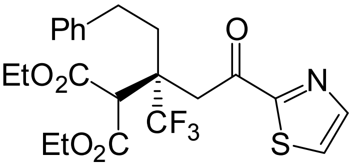

CHIRALPAK® ID
Hexane/*i*-PrOH: 98/2; flow rate 1.0 mL/min
219 nm

RACEMATE

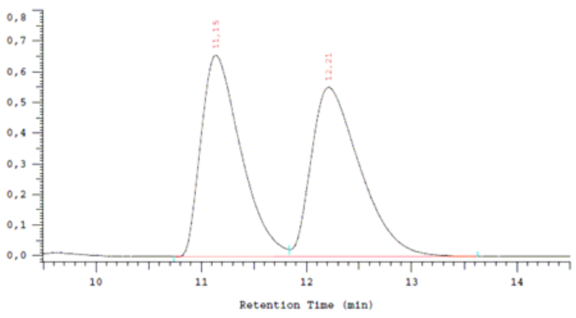

75.7% ee

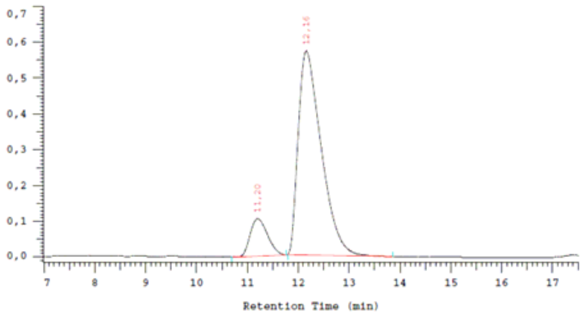

RT	Area	Conc 1
11,15	8645934	49,246
12,21	8910693	50,754
	17556627	100,000

RT	Area	Conc 1
11,20	1237426	12,148
12,16	8949128	87,852
	10186554	100,000

5t

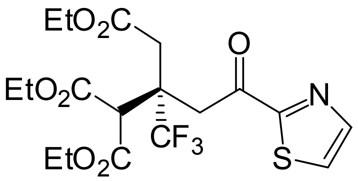

CHIRALPAK® IC
Hexane/*i*-PrOH: 95/5; flow rate 1.0 mL/min
219 nm

RACEMATE

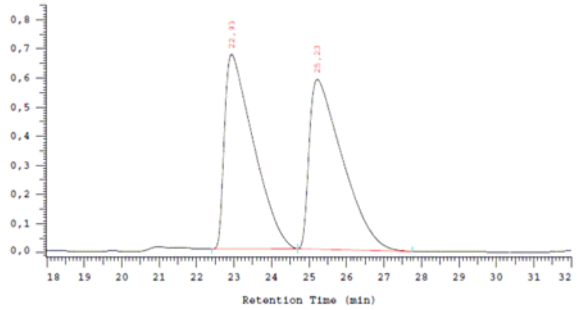

85.9% ee

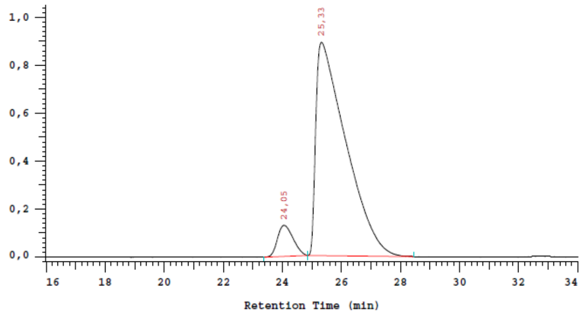

RT	Area	Conc 1
22,93	18033904	50,024
25,23	18016545	49,976
	36050449	100,000

RT	Area	Conc 1
24,05	2380376	7,029
25,33	31485046	92,971
	33865422	100,000

5u

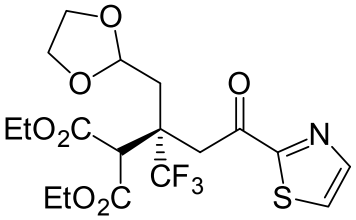

CHIRALPAK® IA
Hexane/*i*-PrOH: 95/5; flow rate 1.0 mL/min
219 nm

RACEMATE

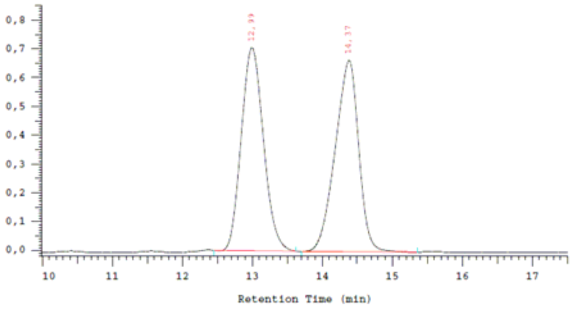

83.0% ee

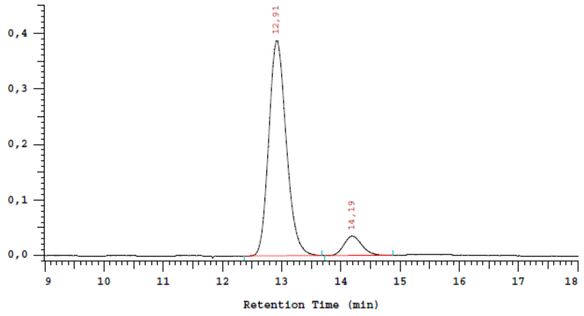

RT	Area	Conc 1
12,99	7741903	49,913
14,37	7768915	50,087
	15510818	100,000

RT	Area	Conc 1
12,91	3969890	91,509
14,19	368340	8,491
	4338230	100,000

7a

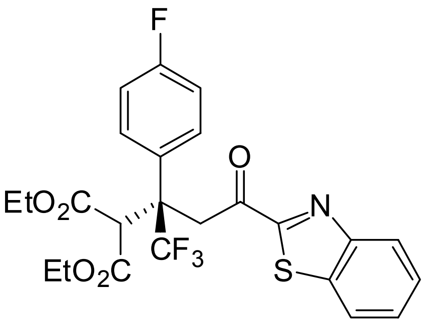

CHIRALPAK® IA
Hexane/*i*-PrOH: 95/5; flow rate 1.0 mL/min
219 nm

RACEMATE

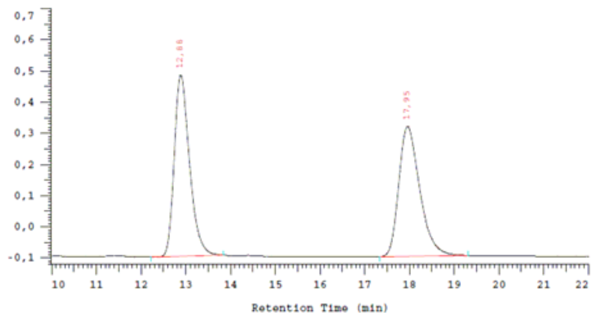

RT	Area	Conc 1
12,88	6926909	50,512
17,95	6786573	49,488
	13713482	100,000

93.4% ee

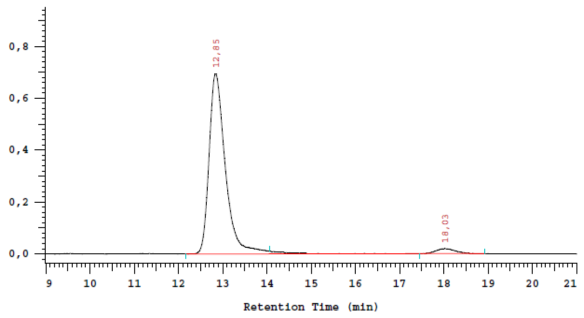

RT	Area	Conc 1
12,85	8854725	96,722
18,03	300094	3,278
	9154819	100,000

7b

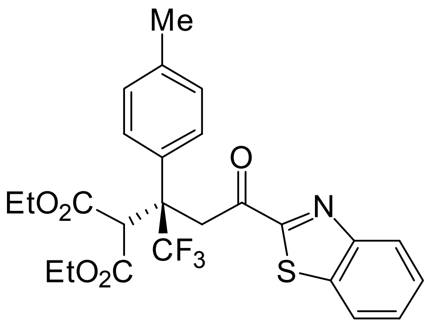

CHIRALPAK® IA
Hexane/*i*-PrOH: 95/5; flow rate 1.0 mL/min
219 nm

RACEMATE

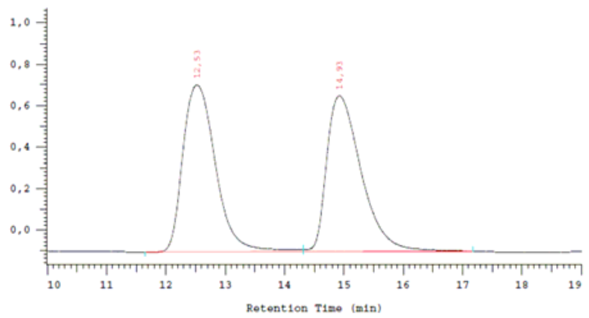

RT	Area	Conc 1
12,53	15501140	50,349
14,93	15286518	49,651
	30787658	100,000

89.6% ee

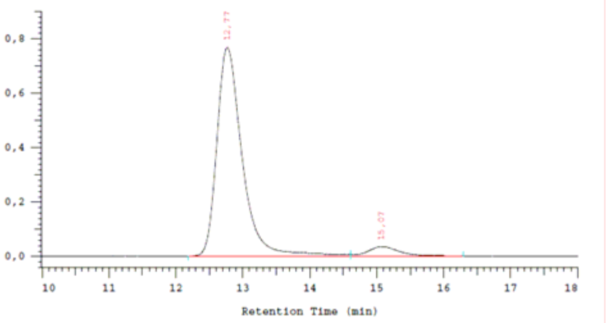

RT	Area	Conc 1
12,77	10029669	94,822
15,07	547729	5,178
	10577398	100,000

7c

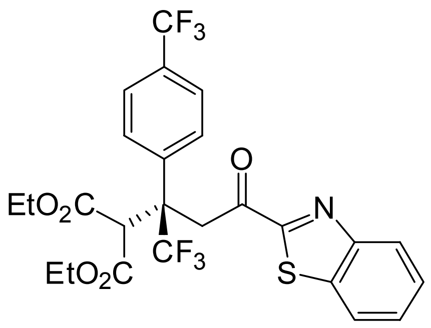

CHIRALPAK® IA
Hexane/*i*-PrOH: 95/5; flow rate 1.0 mL/min
219 nm

RACEMATE

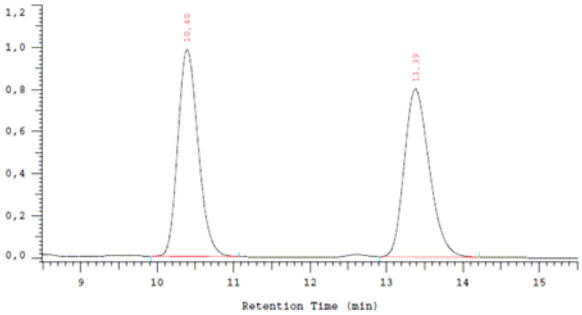

RT	Area	Conc 1
10,40	9052784	49,892
13,39	9091866	50,108
	18144650	100,000

88.2% ee

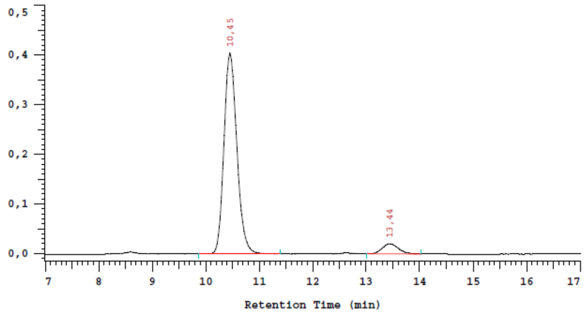

RT	Area	Conc 1
10,45	3393281	94,091
13,44	213114	5,909
	3606395	100,000

7d

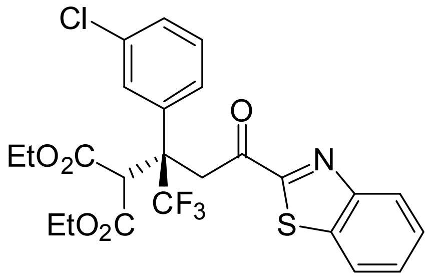

CHIRALPAK® IC
Hexane/*i*-PrOH: 90/10; flow rate 1.0 mL/min
219 nm

RACEMATE

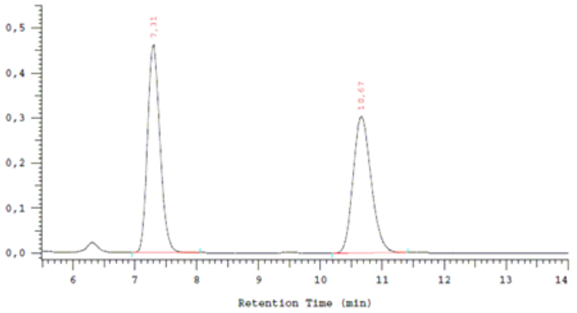

RT	Area	Conc 1
7,31	3223175	51,136
10,67	3079907	48,864
	6303082	100,000

94.7% ee

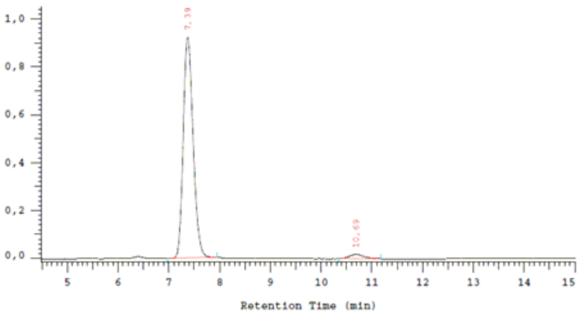

RT	Area	Conc 1
7,39	6201013	97,362
10,69	168047	2,638
	6369060	100,000

7e

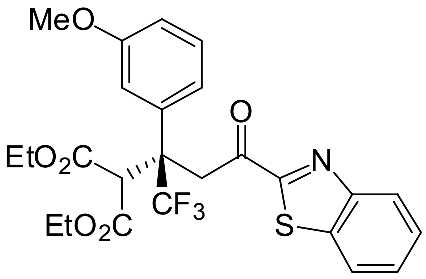

CHIRALPAK® IA
Hexane/*i*-PrOH: 95/5; flow rate 1.0 mL/min
219 nm

RACEMATE

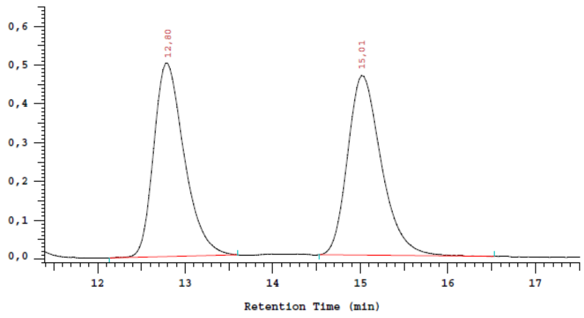

92.6% ee

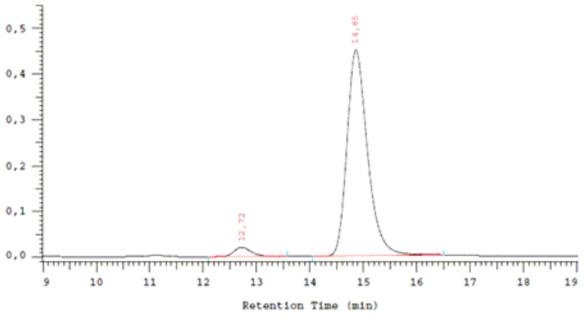

RT	Area	Conc 1
12,80	6048387	49,320
15,01	6215114	50,680
	12263501	100,000

RT	Area	Conc 1
12,72	231894	3,699
14,85	6037732	96,301
	6269626	100,000

7f

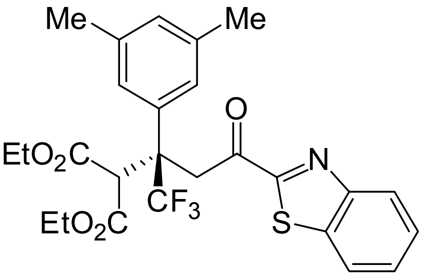

CHIRALPAK® IC
Hexane/*i*-PrOH: 90/10; flow rate 1.0 mL/min
219 nm

RACEMATE

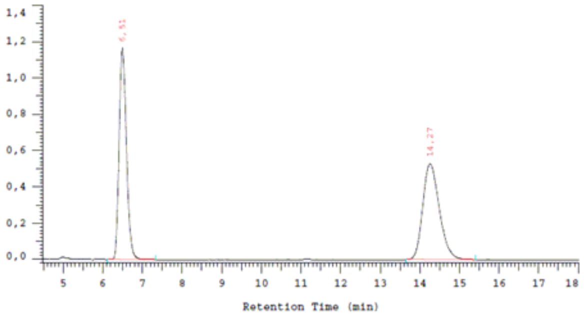

92.7% ee

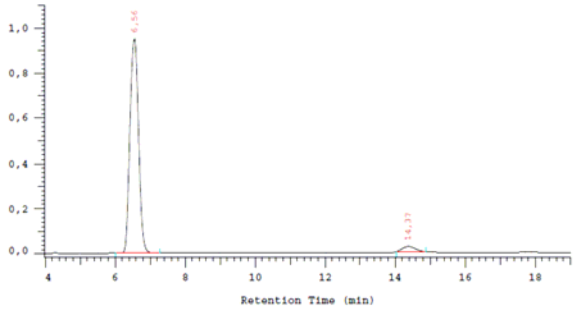

RT	Area	Conc 1
6,51	7465646	49,385
14,27	7651611	50,615
	15117257	100,000

RT	Area	Conc 1
6,56	8099768	96,346
14,37	307196	3,654
	8406964	100,000

7g

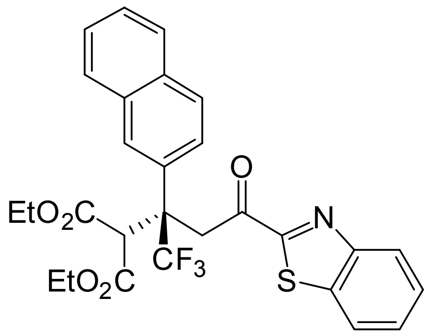

CHIRALPAK® IA
Hexane/*i*-PrOH: 95/5; flow rate 1.0 mL/min
219 nm

RACEMATE

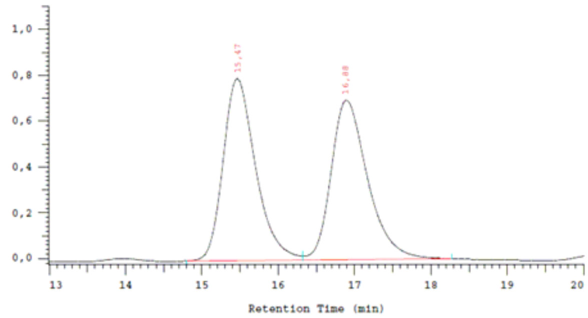

RT	Area	Conc 1
15,47	11739893	50,343
16,88	11579780	49,657
	23319673	100,000

90.8% ee

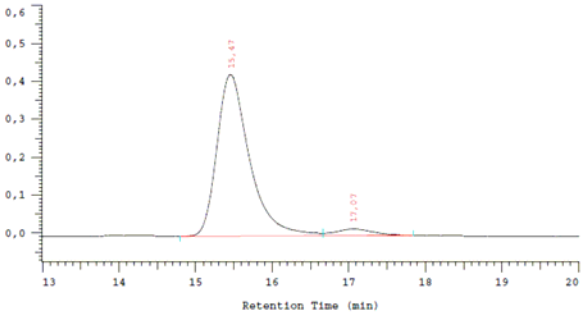

RT	Area	Conc 1
15,47	6305465	95,382
17,07	305262	4,618
	6610727	100,000

7h

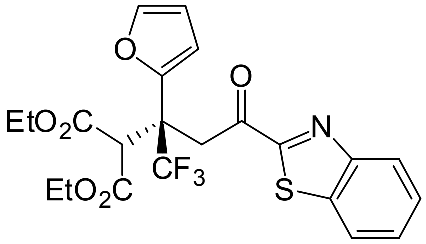

CHIRALPAK® IC
Hexane/*i*-PrOH: 90/10; flow rate 1.0 mL/min
219 nm

RACEMATE

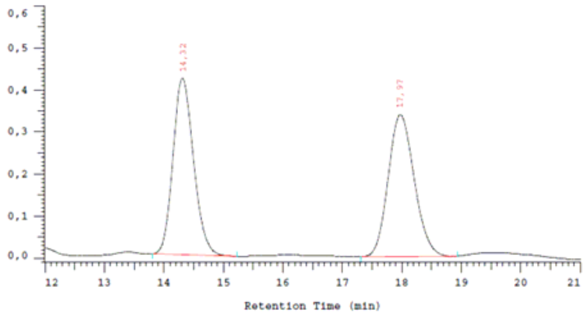

RT	Area	Conc 1
14,32	5083521	49,694
17,97	5146116	50,306
	10229637	100,000

81.9% ee

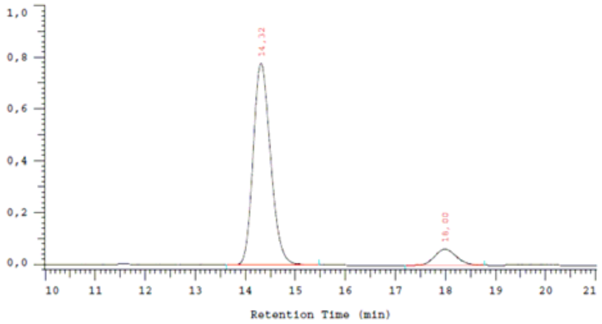

RT	Area	Conc 1
14,32	9371544	90,934
18,00	934364	9,066
	10305908	100,000

7i

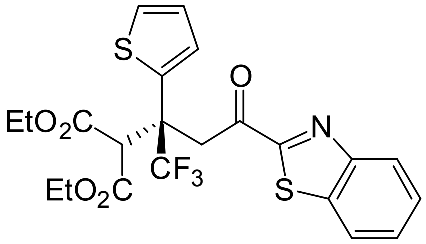

CHIRALPAK® IA
Hexane/*i*-PrOH: 90/10; flow rate 1.0 mL/min
219 nm

RACEMATE

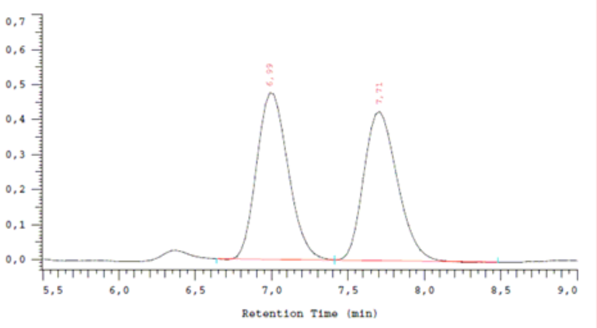

RT	Area	Conc 1
6,99	3404392	51,180
7,71	3247451	48,820
	6651843	100,000

82.6% ee

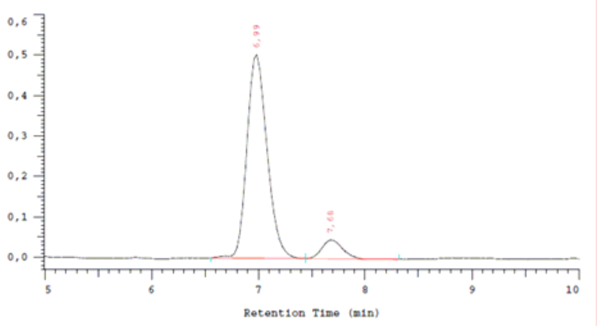

RT	Area	Conc 1
6,99	3292836	91,294
7,68	314003	8,706
	3606839	100,000

7j

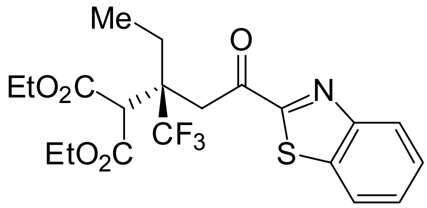

CHIRALPAK® IA
Hexane/*i*-PrOH: 98/2; flow rate 1.0 mL/min
219 nm

RACEMATE

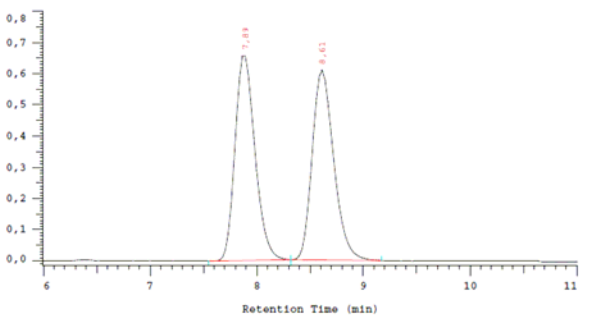

RT	Area	Conc 1
7,89	4274529	50,080
8,61	4260816	49,920
	8535345	100,000

96.9% ee

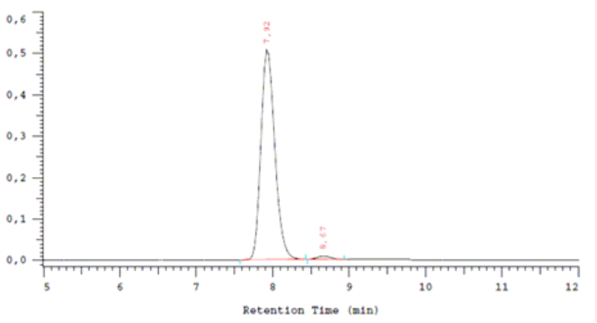

RT	Area	Conc 1
7,92	3185866	98,457
8,67	49932	1,543
	3235798	100,000

7k

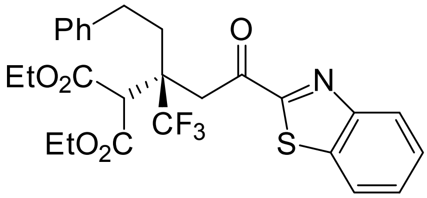

CHIRALPAK® IA
Hexane/*i*-PrOH: 95/5; flow rate 1.0 mL/min
219 nm

RACEMATE

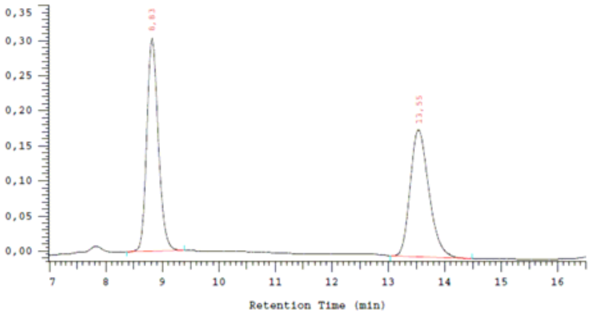

RT	Area	Conc 1
8,83	2136386	50,535
13,55	2091139	49,465
	4227525	100,000

94.6% ee

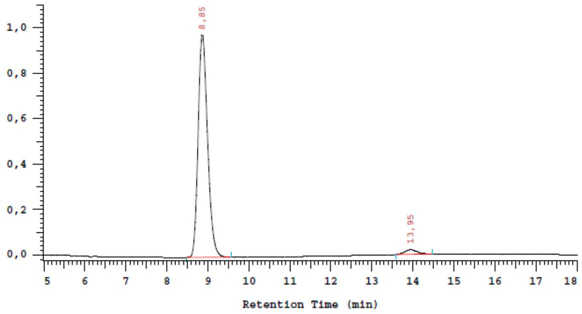

RT	Area	Conc 1
8,85	7816816	97,282
13,95	218384	2,718
	8035200	100,000

9a

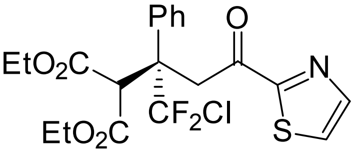

CHIRALPAK® IC
Hexane/*i*-PrOH: 90/10; flow rate 1.0 mL/min
219 nm

RACEMATE

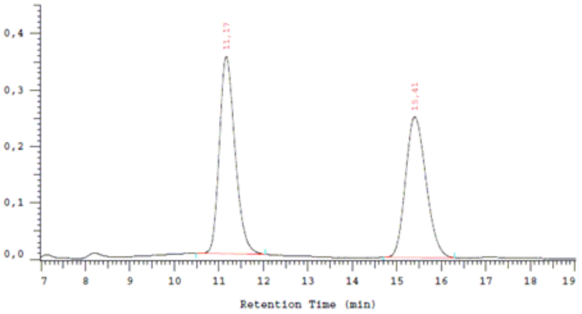

RT	Area	Conc 1
11,17	4287580	51,649
15,41	4013841	48,351
	8301421	100,000

91.6% ee

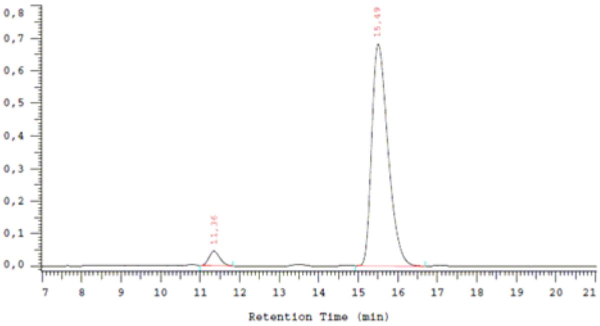

RT	Area	Conc 1
11,36	426136	4,204
15,49	9710897	95,796
	10137033	100,000

10b

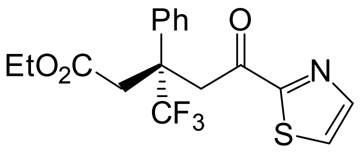

CHIRALPAK® IB
Hexane/*i*-PrOH: 90/10; flow rate 1.0 mL/min
219 nm

RACEMATE

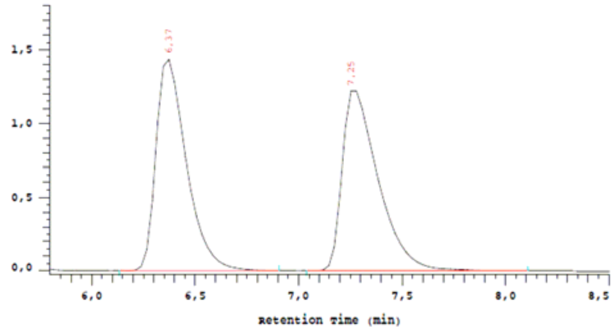

RT	Area	Conc 1
6,37	7355016	49,787
7,25	7418091	50,213
	14773107	100,000

97.1% ee

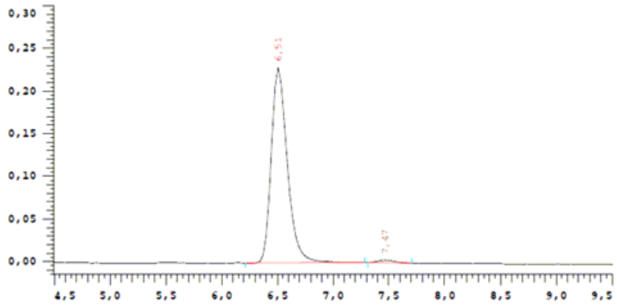

RT	Area	Conc 1
6,51	1143810	98,551
7,47	16811	1,449
	1160621	100,000

10c

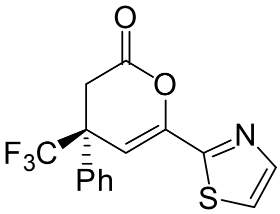

CHIRALPAK® IC
Hexane/*i*-PrOH: 80/20; flow rate 1.0 mL/min
219 nm

RACEMATE

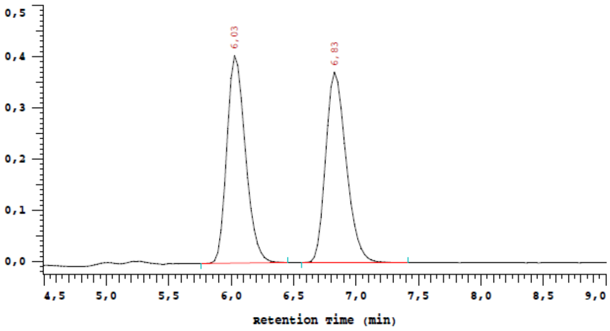

RT	Area	Conc 1
6,03	2105587	49,896
6,83	2114355	50,104
	4219942	100,000

99.4% ee

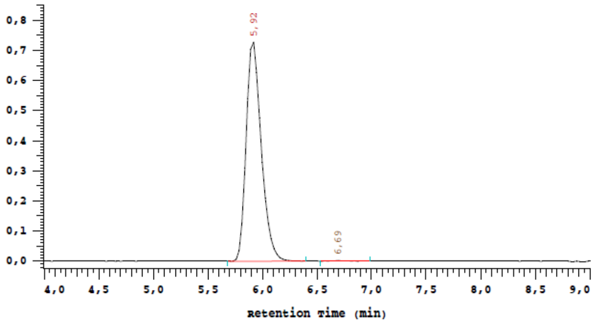

RT	Area	Conc 1
5,92	3512381	99,704
6,69	10415	0,296
	3522796	100,000

10d

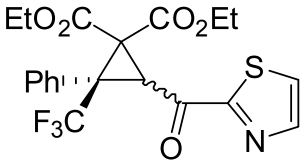

CHIRALPAK® ID
Hexane/*i*-PrOH: 90/10; flow rate 1.0 mL/min
219 nm

RACEMATE

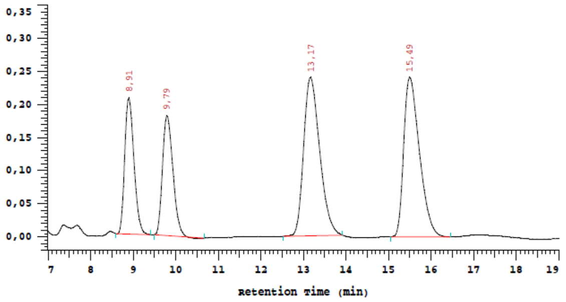

RT	Area	Conc 1
8,91	1547711	16,615
9,79	1538620	16,517
13,17	3130125	33,602
15,49	3098876	33,266
	9315332	100,000

98.7% ee

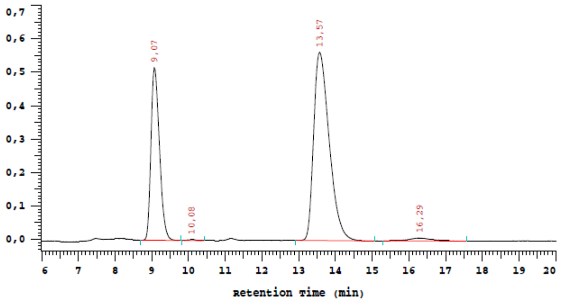

RT	Area	Conc 1
9,07	4118763	32,706
10,08	23612	0,187
13,57	8253643	65,539
16,29	197410	1,568
	12593428	100,000

10e

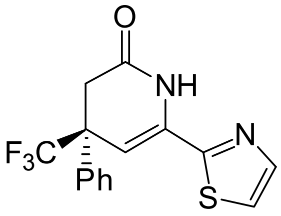

CHIRALPAK® IC
Hexane/*i*-PrOH: 80/20; flow rate 1.0 mL/min
219 nm

RACEMATE

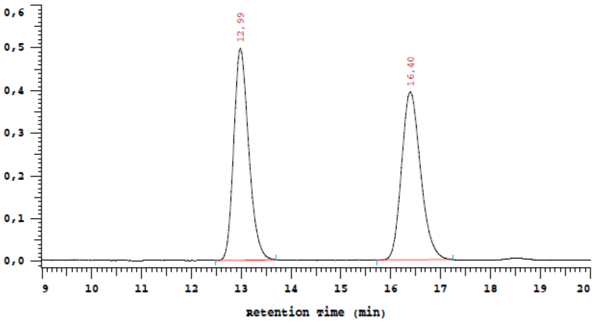

RT	Area	Conc 1
12,99	5215171	50,061
16,40	5202555	49,939
	10417726	100,000

98.7% ee

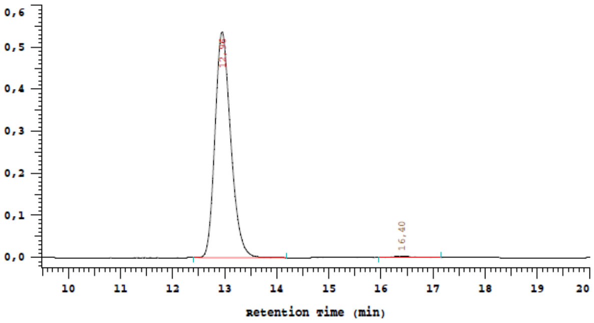

RT	Area	Conc 1
12,96	5636288	99,362
16,40	36208	0,638
	5672496	100,000

10f

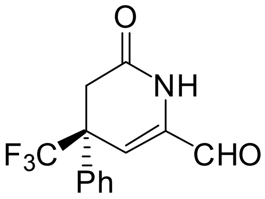

CHIRALPAK® IC
Hexane/*i*-PrOH: 70/30; flow rate 1.0 mL/min
219 nm

RACEMATE

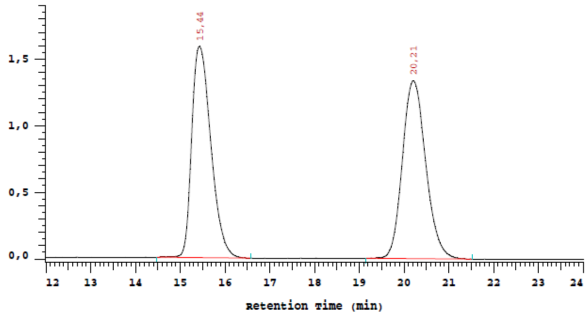

RT	Area	Conc 1
15,44	23485225	49,182
20,21	24265974	50,818
	47751199	100,000

98.7% ee

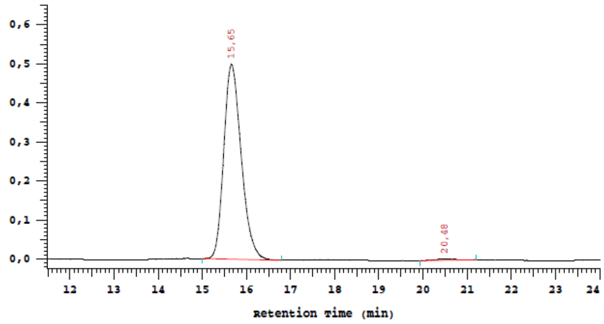

RT	Area	Conc 1
15,65	6866966	99,337
20,48	45824	0,663
	6912790	100,000

10g

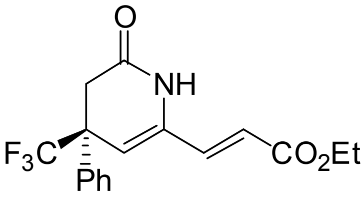

CHIRALPAK® IC
Hexane/*i*-PrOH: 90/10; flow rate 1.0 mL/min
219 nm

RACEMATE

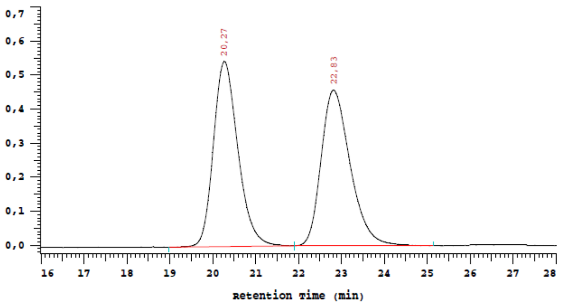

RT	Area	Conc 1
20,27	10828668	50,053
22,83	10805808	49,947
	21634476	100,000

98.8% ee

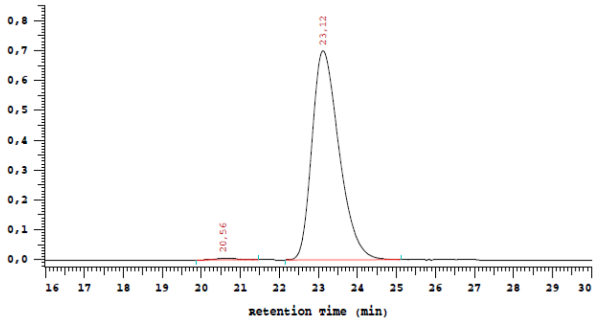

RT	Area	Conc 1
20,56	99700	0,589
23,12	16837600	99,411
	16937300	100,000

Crystallographic data: *(E)*-2h and *(R)*-3h

X-ray crystal structure of compound (*E*)-2h (CCDC 2033577)

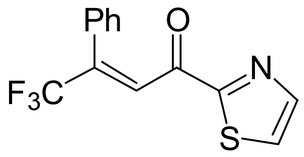

Good-quality single-crystal of compoundwas selected for the X-ray diffraction experiment at T= 100(2) K. The crystal was mounted with paratone-N oil to the MiTeGenmicromount. Diffraction data were collected on the Agilent Technologies SuperNova Dual Source with the MoKα radiation (λ = 0.71073 Å). The lattice parameters were obtained by least-squares fit to the optimized setting angles of the reflections collected by using the CrysAlis CCD software.[41] Data were reduced using the CrysAlis RED program.[41] The multi-scan Empirical absorption correction using spherical harmonics, implemented in SCALE3 ABSPACK scaling algorithm was applied.[41] The structural determination procedure was carried out using the SHELX package.[42] The structures were solved with direct methods, and then successive least-squares refinements were carried out based on full-matrix least-squares on F^2 using the SHELXMP program.[42] All the H atoms were positioned geometrically with the C–H bond length equal to 0.93Å for the aromatic and methylidenehydrogen atoms,and constrained to ride on their parent atoms with Uiso(H) = $1.2U_{eq}$(C). The figures for this report were prepared using ORTEP-3[43] and Mercury programs.[44] Investigated compound crystallizes in the monoclinic $P2_1/n$ space group with one molecule in the asymmetric unit of the crystal lattice. The crystallographic data are summarized in the Table S2. Selected bond lengths, valence and torsion angles are gathered in Tables S3-S5.

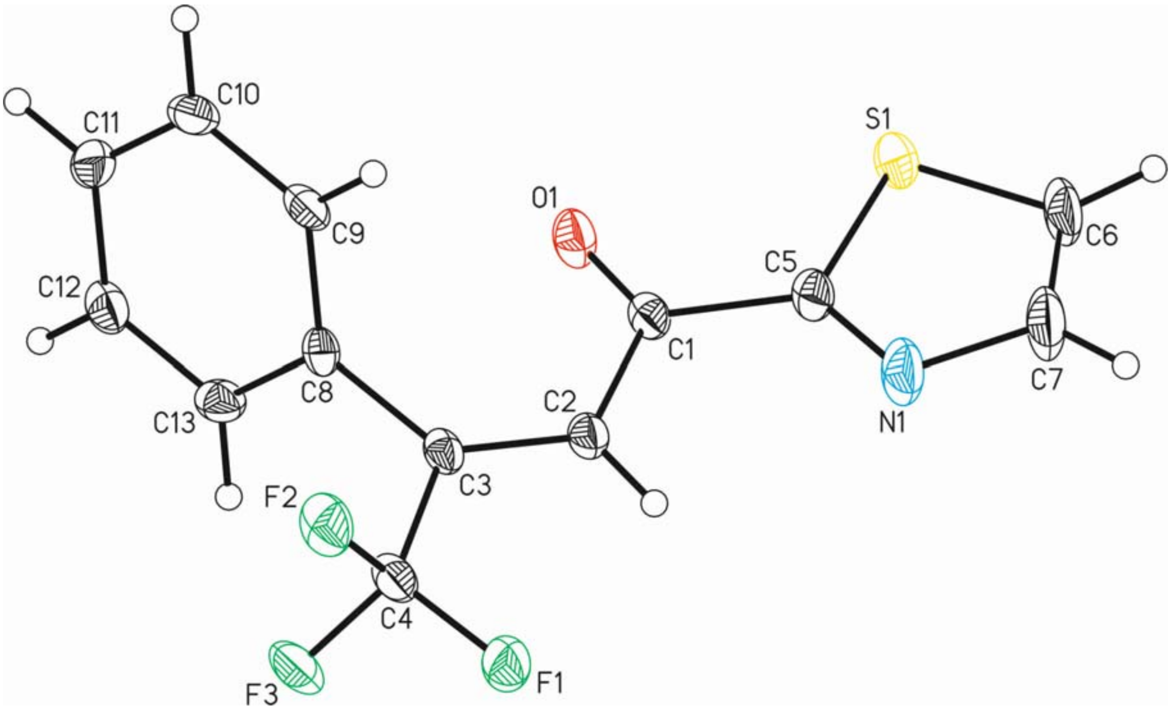

Molecular structure of *(E)*-**2h** showing the atom labeling scheme. Displacement ellipsoids are drawn at the 50% probability level, and H atoms are shown as small spheres of arbitrary radius.

[41] *CrysAlis CCD and CrysAlis RE*D; Oxford Diffraction Ltd: Yarnton, 2008.

[42] Sheldrick, G. M. *ActaCrystallogr., Sect. A: Found. Crystallogr*. 2008, **64**, 112–122.

[43] Farrugia, L. J. *J. Appl. Cryst.* 1997, **30**, 565–572.

[44] Macrae, C. F., Edgington, P. R., McCabe, P., Pidcock, E., Shields, G. P., Taylor, R., Towler, M. and van de Streek, J. *J. Appl. Cryst.* 2006, **39**, 453–457.

Crystallographic data (excluding structural factors) for compound **2h** also has been deposited at the Cambridge Crystallographic Data Centre under the deposition number **CCDC 2033577**.

Table S2. Crystal data and structure refinement for investigated compound.

Identification code	(*E*)-**2h**
Empirical formula	$C_{13}H_8F_3NOS$
Formula weight	283.26
Temperature/K	100(2)
Crystal system	monoclinic
Space group	$P2_1/n$
a/Å	5.7664(3)
b/Å	8.5449(3)
c/Å	26.018(2)
α/°	90.00
β/°	96.338(6)
γ/°	90.00
Volume/Å^3	1274.16(13)
Z	4
ρ_{calc}g/cm^3	1.477
μ/mm^{-1}	0.280
F(000)	576.0
Crystal size/mm	0.25 × 0.23 × 0.10
Radiation	MoKα (λ = 0.71073)
2Θ range for data collection/°	5.02 to 52.74
Index ranges	-7 ≤ h ≤ 7, -10 ≤ k ≤ 10, -32 ≤ l ≤ 32
Reflections collected	15480
Independent reflections	2600 [R_{int} = 0.0493, R_{sigma} = 0.0352]
Data/restraints/parameters	2600/0/172
Goodness-of-fit on F^2	1.364
Final R indexes [I>=2σ (I)]	R_1 = 0.0809, wR_2 = 0.1630
Final R indexes [all data]	R_1 = 0.0835, wR_2 = 0.1640
Largest diff. peak/hole / e Å^{-3}	0.40/-0.36

2h was crystallized from AcOEt

Table S3. The bond lengths for investigated compound.

Atom	Atom	Length/Å	Atom	Atom	Length/Å
C1	C2	1.490(6)	C5	S1	1.728(4)
C1	C5	1.490(6)	C6	C7	1.355(7)
C1	O1	1.217(5)	C6	S1	1.708(5)
C2	C3	1.339(6)	C7	N1	1.374(6)
C3	C4	1.512(6)	C8	C9	1.400(6)
C3	C8	1.496(6)	C8	C13	1.390(6)
C4	F1	1.338(5)	C9	C10	1.391(6)
C4	F2	1.353(5)	C10	C11	1.390(6)
C4	F3	1.345(5)	C11	C12	1.392(6)
C5	N1	1.307(6)	C12	C13	1.391(6)

Table S4. The valence angles for investigated compound.

Atom	Atom	Atom	Angle/°	Atom	Atom	Atom	Angle/°
C5	C1	C2	115.5(4)	N1	C5	C1	124.8(4)
O1	C1	C2	124.4(4)	N1	C5	S1	115.6(3)
O1	C1	C5	120.1(4)	C7	C6	S1	111.1(4)
C3	C2	C1	123.5(4)	C6	C7	N1	115.1(4)
C2	C3	C4	118.7(4)	C9	C8	C3	120.0(4)
C2	C3	C8	126.4(4)	C13	C8	C3	120.7(4)
C8	C3	C4	114.9(3)	C13	C8	C9	119.3(4)
F1	C4	C3	113.9(3)	C10	C9	C8	120.0(4)
F1	C4	F2	106.4(3)	C11	C10	C9	120.5(4)
F1	C4	F3	107.2(3)	C10	C11	C12	119.4(4)
F2	C4	C3	110.6(3)	C13	C12	C11	120.3(4)
F3	C4	C3	111.9(3)	C8	C13	C12	120.5(4)
F3	C4	F2	106.5(3)	C5	N1	C7	109.9(4)
C1	C5	S1	119.6(3)	C6	S1	C5	88.3(2)

Table S5. The torsion angles for investigated compound.

A	B	C	D	Angle/°	A	B	C	D	Angle/°
C1	C2	C3	C4	179.4(4)	C7	C6	S1	C5	0.0(4)
C1	C2	C3	C8	-2.3(7)	C8	C3	C4	F1	-178.8(3)
C1	C5	N1	C7	-179.8(4)	C8	C3	C4	F2	-59.1(4)
C1	C5	S1	C6	179.8(4)	C8	C3	C4	F3	59.4(5)
C2	C1	C5	N1	-0.7(6)	C8	C9	C10	C11	0.5(6)
C2	C1	C5	S1	179.5(3)	C9	C8	C13	C12	0.4(6)
C2	C3	C4	F1	-0.3(5)	C9	C10	C11	C12	-0.3(6)
C2	C3	C4	F2	119.4(4)	C10	C11	C12	C13	0.1(6)
C2	C3	C4	F3	-122.1(4)	C11	C12	C13	C8	-0.1(6)
C2	C3	C8	C9	-71.0(6)	C13	C8	C9	C10	-0.6(6)
C2	C3	C8	C13	109.2(5)	N1	C5	S1	C6	0.0(4)
C3	C8	C9	C10	179.6(4)	O1	C1	C2	C3	-13.7(7)
C3	C8	C13	C12	-179.8(4)	O1	C1	C5	N1	179.5(4)
C4	C3	C8	C9	107.4(4)	O1	C1	C5	S1	-0.3(6)
C4	C3	C8	C13	-72.5(5)	S1	C5	N1	C7	0.0(5)
C5	C1	C2	C3	166.5(4)	S1	C6	C7	N1	0.0(6)
C6	C7	N1	C5	0.0(7)					

X-ray crystal structure of compound (*R*)-3h (CCDC 2033588)

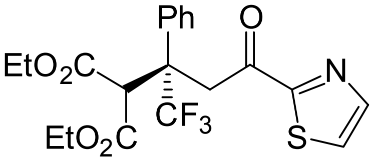

The X-ray diffraction data for enantiomer of **3h** was collected using SuperNova Dual Source diffractometer using CuKα radiation in 295(2) K. Data reduction and analysis were carried out with the Crysalis Pro software.[45] Using Olex2[46] package, the structures were solved with the ShelXS[47] structure solution program using direct methods and refined with the ShelXL2[48] refinement package using the least squares minimisation. The refinement was based on F2 for all reflections except those with negative intensities. Weighted R factors (wR) and all goodness of-fit (GooF) values were based on F2. Conventional R factors were based on the amplitudes, with F set to zero for negative F2. The F02 > 2σ(F02) criterion was applied only for R factors calculation and was not relevant to the choice of reflections for the refinement. The R factors based on F2 are for all structures about twice as large as those based on F.

Crystal data, data collection and refinement details are presented in Table S6. Fractional atomic coordinates and equivalent isotropic displacement parameters, anisotropic displacement parameters, bond lengths, value of angles and hydrogen atom coordinates are presented in Tables S7-11, respectively. Single-crystal X-Ray diffraction analysis proved that investigated compound crystallizes in orthorhombic $P2_12_12_1$ space groupwith one molecule of compound in the assymetric unit of the crystal lattice. The compound containsone asymmetric carbon atom (C3), which absolute configuration is R.

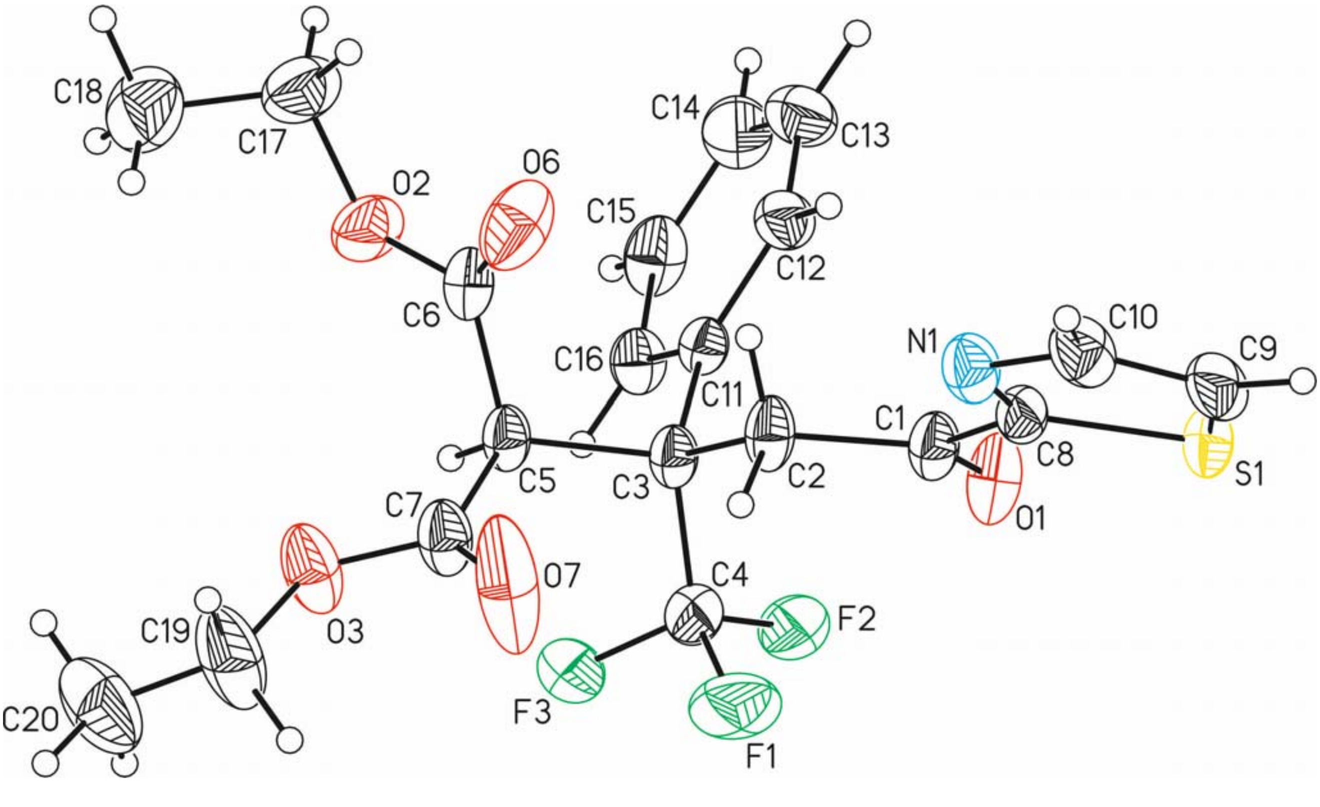

Crystallographic data (excluding structural factors) for compound (–)-(*R*)-**3h** also has been deposited at the Cambridge Crystallographic Data Centre under the deposition number **CCDC 2033588**.

[45] Agilent, CrysAlis PRO, Agilent Technologies, Yarnton, England, 2012, www.rigaku.com/en/products/smc/crysalis

[46] O.V. Dolomanov, L.J. Bourhis, R.J. Gildea, J.A.K. Howard, H. Puschmann, J. Appl. Cryst. 42 (2009) 339-341.

[47] G.M. Sheldrick, *ActaCryst.*, A64 (2008) 112-122.

[48] G.M. Sheldrick, *ActaCryst.*, C71 (2015) 3-8.

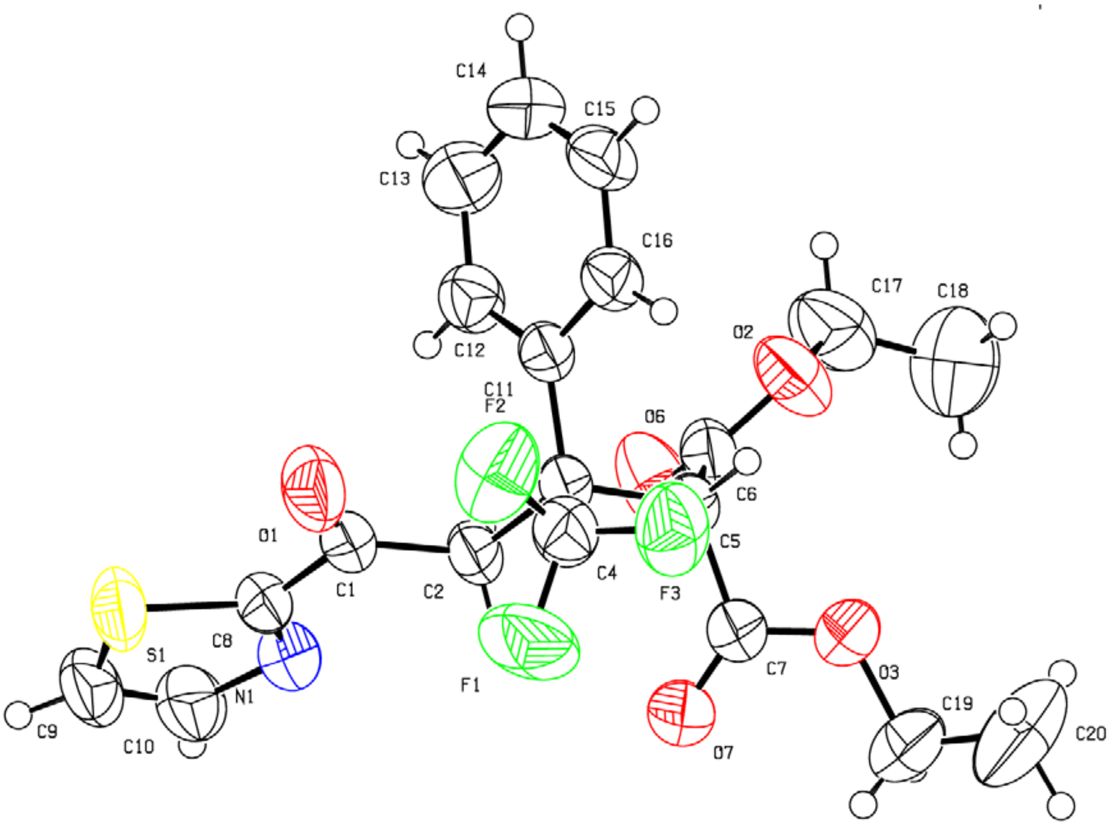

Molecular structure of (–)-(*R*)-**3h** showing atom labelling scheme. Displacement ellipsoids are drawn at the 50% probability level. The H-atoms are drawn as small spheres of arbitrary radius.

Table S6. Crystal data and structure refinement for (*R*)-3h

Empirical formula	$C_{20}H_{20}F_3NO_5S$
Formula weight	443.43
Temperature/K	295(2)
Crystal system	Orthorhombic
Space group	$P2_12_12_1$
a/Å	10.20965(7)
b/Å	13.60847(10)
c/Å	15.68612(10)
α/°	90
β/°	90
γ/°	90
Volume/Å^3	2179.39(3)
Z	4
ρ_{calc}g/cm^3	1.351
μ/mm^{-1}	1.830
F(000)	920.0
Crystalsize/mm^3	0.50 × 0.18 × 0.08
Radiation	CuKα (λ = 1.54184)
2Θ range for data collection/°	8.602 to 154.874
Index ranges	-12 ≤ h ≤ 12, -17 ≤ k ≤ 17, -19 ≤ l ≤ 19
Reflectionscollected	60427
Independent reflections	4617 [R_{int} = 0.0543, R_{sigma} = 0.0188]
Data/restraints/parameters	4617/0/273
Goodness-of-fit on F^2	1.039
Final R indexes [I>=2σ (I)]	R_1 = 0.0591, wR_2 = 0.1531
Final R indexes [all data]	R_1 = 0.0598, wR_2 = 0.1547
Largest diff. peak/hole / e Å^{-3}	0.39/-0.41
Flack parameter	0.013(5)

3h was crystallized from ethanol

Table S7 Fractional Atomic Coordinates (×10^4) and Equivalent Isotropic Displacement Parameters (Å^2×10^3) for (*R*)-3h. U_{eq} is defined as 1/3 of of the trace of the orthogonalised U_{IJ} tensor.

Atom	*x*	*y*	*z*	U(eq)
C3	7449(3)	5040(2)	2298.3(17)	53.0(6)
N1	8168(3)	2906(3)	220.8(19)	71.0(7)
O2	5637(4)	4021(2)	3962.7(16)	87.4(9)
C2	7598(3)	4319(3)	1548(2)	63.5(8)
O1	9775(3)	4900(3)	1205.7(19)	89.8(9)
C8	9011(3)	3595(2)	389.7(18)	58.1(6)
C11	8454(3)	4880(2)	3010.0(16)	49.7(5)
F3	6760(3)	6737.3(19)	2395.5(19)	96.0(8)
C5	6067(3)	4919(2)	2733.9(17)	55.9(6)
C16	8552(3)	5537(2)	3687.6(19)	59.6(7)
F1	7173(4)	6181(3)	1149.5(18)	125.9(13)
O3	3936(3)	5442(3)	2599.2(19)	97.4(10)
C15	9384(4)	5355(4)	4368(2)	78.9(10)
C1	8886(3)	4337(3)	1071.1(19)	62.2(7)
F2	8716(3)	6500.5(19)	2002(2)	103.0(9)
C6	5892(3)	3924(3)	3151(2)	62.3(7)
C7	4862(3)	5057(3)	2164(2)	72.8(9)
O6	5899(4)	3164(3)	2784(2)	107.1(12)
C12	9209(4)	4032(2)	3051(2)	64.2(7)
C14	10121(4)	4502(4)	4386(3)	89.3(12)
C10	8590(5)	2355(3)	-451(3)	82.0(11)
O7	4781(4)	4885(7)	1444(2)	189(4)
C13	10035(5)	3858(4)	3733(3)	88.0(12)
C17	5248(6)	3155(4)	4448(3)	99.0(14)
C4	7530(4)	6111(3)	1961(2)	73.0(9)
C19	2676(5)	5590(7)	2180(4)	128(3)
C9	9744(5)	2632(3)	-793(2)	84.8(12)
C20	1911(5)	6216(5)	2697(6)	136(3)
C18	3918(7)	3281(5)	4775(5)	123(2)
S1	10359.5(10)	3614.8(8)	-266.2(6)	80.1(3)

Table S8 Anisotropic Displacement Parameters (Å^2×10^3) for (*R*)-3h. The Anisotropic displacement factor exponent takes the form: $-2\pi^2[h^2a^{*2}U_{11}+2hka^*b^*U_{12}+\ldots]$.

Atom	U_{11}	U_{22}	U_{33}	U_{23}	U_{13}	U_{12}
C3	45.6(12)	71.0(15)	42.4(12)	-7.9(11)	4.6(10)	1.0(11)
N1	66.9(16)	87.7(18)	58.4(15)	-10.4(14)	4.4(12)	11.1(14)
O2	127(2)	85.0(15)	50.1(11)	2.1(11)	14.7(14)	11.1(17)
C2	49.9(14)	96(2)	45.0(13)	-18.9(14)	6.2(12)	-4.5(14)
O1	65.2(14)	127(2)	77.2(16)	-25.4(16)	21.4(13)	-21.9(16)
C8	56.3(14)	75.7(16)	42.4(12)	4.7(12)	5.9(11)	13.2(13)
C11	47.9(12)	58.3(13)	42.9(12)	-2.4(10)	4.6(10)	-2.2(10)
F3	106.3(19)	78.8(13)	102.9(18)	12.5(13)	24.8(15)	29.4(13)
C5	46.8(13)	79.6(17)	41.3(12)	-10.6(12)	5.8(10)	2.7(12)
C16	57.4(14)	69.6(15)	51.7(14)	-12.5(12)	1.0(12)	-1.8(13)
F1	168(3)	140(3)	69.3(15)	33.6(17)	-12.6(17)	38(2)
O3	60.4(14)	160(3)	71.7(16)	-28.3(18)	-9.9(12)	31.7(17)
C15	71(2)	116(3)	49.7(16)	-16.5(17)	-2.7(14)	-11(2)
C1	50.9(14)	91(2)	44.5(13)	-5.8(13)	5.8(11)	1.0(14)
F2	86.5(16)	81.2(14)	141(2)	39.0(15)	23.3(16)	-4.8(12)
C6	49.4(14)	82.2(18)	55.4(15)	-15.8(14)	9.5(12)	-7.1(13)

C7	52.7(15)	113(3)	52.2(15)	-13.9(16)	-0.5(12)	8.6(17)
O6	144(3)	92.3(19)	85.4(19)	-27.9(16)	40(2)	-36(2)
C12	72.1(19)	61.5(15)	59.1(15)	-2.3(13)	3.3(14)	10.1(14)
C14	77(2)	129(4)	61.1(19)	8(2)	-12.8(17)	14(2)
C10	92(3)	86(2)	68(2)	-18.0(18)	2(2)	21(2)
O7	72.1(19)	431(11)	64.3(17)	-65(4)	-16.2(15)	67(4)
C13	94(3)	91(3)	79(2)	13(2)	-4(2)	29(2)
C17	129(4)	98(3)	70(2)	16(2)	6(3)	4(3)
C4	69.7(19)	88(2)	61.2(18)	14.8(16)	9.4(15)	12.0(17)
C19	63(2)	216(7)	104(4)	-38(4)	-22(2)	41(3)
C9	104(3)	93(2)	58.2(17)	-6.5(17)	17(2)	41(2)
C20	62(2)	127(4)	220(8)	-19(5)	-14(4)	10(3)
C18	110(4)	106(4)	152(6)	7(4)	28(4)	-23(3)
S1	74.2(5)	97.8(6)	68.2(5)	1.5(4)	29.9(4)	16.4(5)

Table S9 Bond Lengths for (*R*)-3h.

Atom	Atom	Length/Å	Atom	Atom	Length/Å
C3	C2	1.539(4)	C5	C7	1.532(4)
C3	C11	1.531(4)	C16	C15	1.386(5)
C3	C5	1.577(4)	F1	C4	1.327(5)
C3	C4	1.553(5)	O3	C7	1.278(4)
N1	C8	1.300(5)	O3	C19	1.459(5)
N1	C10	1.364(5)	C15	C14	1.383(7)
O2	C6	1.306(4)	F2	C4	1.323(5)
O2	C17	1.458(6)	C6	O6	1.184(5)
C2	C1	1.513(4)	C7	O7	1.156(5)
O1	C1	1.206(4)	C12	C13	1.382(6)
C8	C1	1.476(4)	C14	C13	1.351(7)
C8	S1	1.719(3)	C10	C9	1.349(7)
C11	C16	1.392(4)	C17	C18	1.461(9)
C11	C12	1.390(4)	C19	C20	1.412(9)
F3	C4	1.345(5)	C9	S1	1.693(5)
C5	C6	1.514(5)			

Table S10 Bond Angles for (*R*)-3h.

Atom	Atom	Atom	Angle/°	Atom	Atom	Atom	Angle/°
C2	C3	C5	110.7(2)	O1	C1	C8	119.7(3)
C2	C3	C4	109.4(3)	C8	C1	C2	115.0(3)
C11	C3	C2	113.7(2)	O2	C6	C5	110.7(3)
C11	C3	C5	105.6(2)	O6	C6	O2	124.4(4)
C11	C3	C4	110.2(3)	O6	C6	C5	124.7(3)
C4	C3	C5	107.0(2)	O3	C7	C5	109.4(3)
C8	N1	C10	110.2(3)	O7	C7	C5	127.0(3)
C6	O2	C17	118.8(3)	O7	C7	O3	123.5(4)
C1	C2	C3	117.0(3)	C13	C12	C11	121.1(3)
N1	C8	C1	125.7(3)	C13	C14	C15	119.6(4)
N1	C8	S1	114.8(2)	C9	C10	N1	115.4(4)
C1	C8	S1	119.5(3)	C14	C13	C12	121.0(4)
C16	C11	C3	120.9(3)	O2	C17	C18	109.9(5)
C12	C11	C3	121.5(3)	F3	C4	C3	113.1(3)
C12	C11	C16	117.2(3)	F1	C4	C3	112.3(4)
C6	C5	C3	112.7(2)	F1	C4	F3	106.3(3)
C6	C5	C7	105.5(3)	F2	C4	C3	114.1(3)
C7	C5	C3	117.0(2)	F2	C4	F3	104.9(4)
C15	C16	C11	121.1(3)	F2	C4	F1	105.5(4)
C7	O3	C19	117.9(3)	C20	C19	O3	108.2(5)
C14	C15	C16	119.9(4)	C10	C9	S1	110.6(3)
O1	C1	C2	125.3(3)	C9	S1	C8	88.99(19)

Table S11 Hydrogen Atom Coordinates (Å×10⁴) and Isotropic Displacement Parameters (Å²×10³) for (*R*)-3h.

Atom	*x*	*y*	*z*	U(eq)
**H2A**	7463.18	3658.92	1763.03	76
**H2B**	6902.35	4452.46	1142.8	76
**H5**	6012.63	5412.94	3186.96	67
**H16**	8051.56	6107.74	3683.57	71
**H15**	9445.3	5804.65	4812.31	95
**H12**	9156.77	3572.99	2613.22	77
**H14**	10673.28	4373.45	4843.8	107
**H10**	8114.41	1823.58	-659.74	98
**H13**	10539.76	3289.12	3741.64	106
**H17A**	5849.22	3057.7	4919.72	119
**H17B**	5284.99	2578.74	4085.57	119
**H19A**	2805.89	5885.37	1623.13	153
**H19B**	2235.6	4964.99	2103.67	153
**H9**	10146.19	2326.72	-1254.21	102
**H20A**	1792.79	5919.98	3247.11	204
**H20B**	1072.73	6317.11	2434.42	204
**H20C**	2348.3	6836.22	2761.07	204
**H18A**	3897.23	3827.4	5162.08	184
**H18B**	3652.73	2695.21	5068.79	184
**H18C**	3331.24	3403.54	4308.92	184

Copies of NMR spectra (^{1}H NMR + ^{19}F NMR miniature, ^{13}C NMR) – enones 2e-2k, 4a-4u, 6a-6k, 8a and 8b:

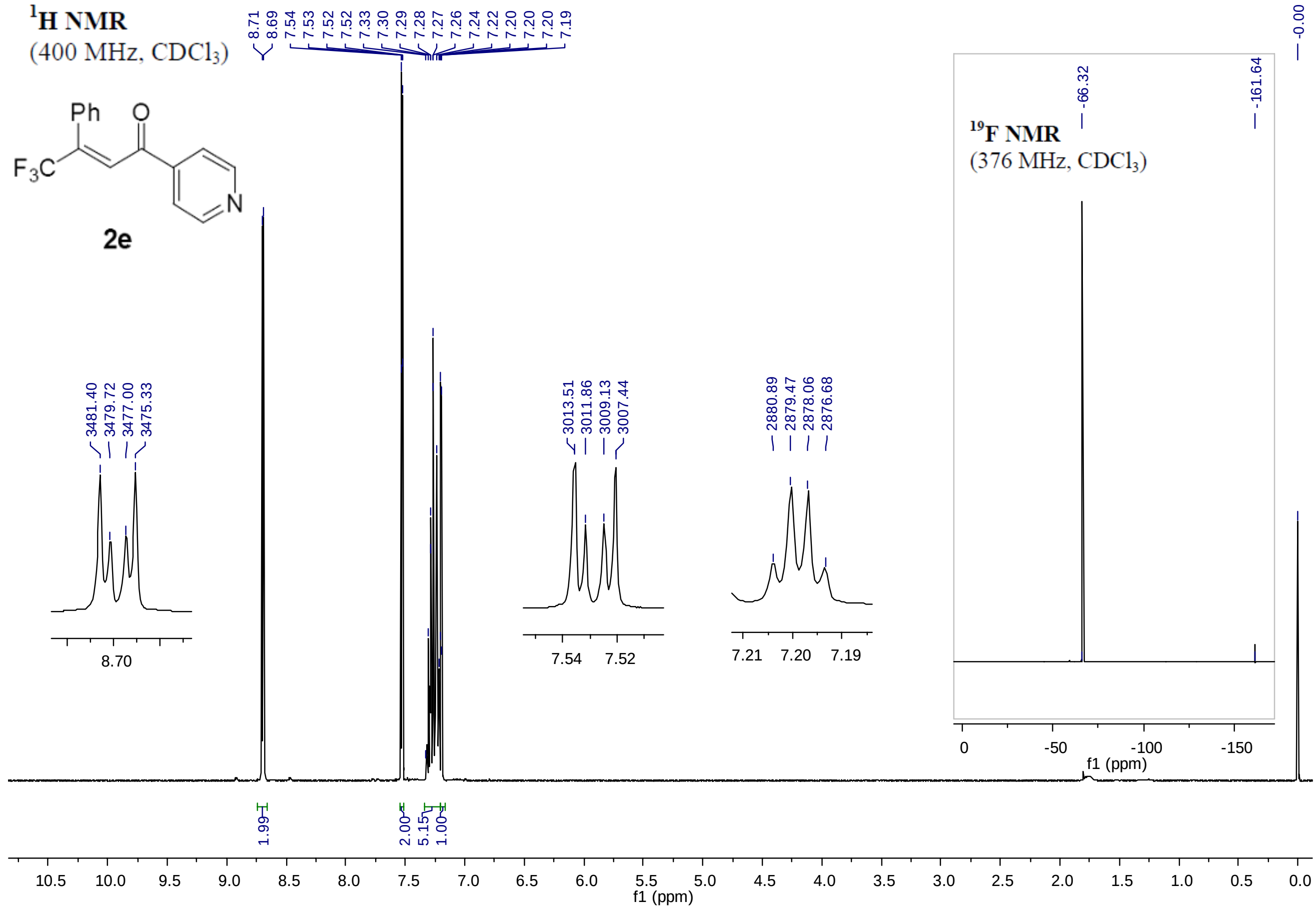

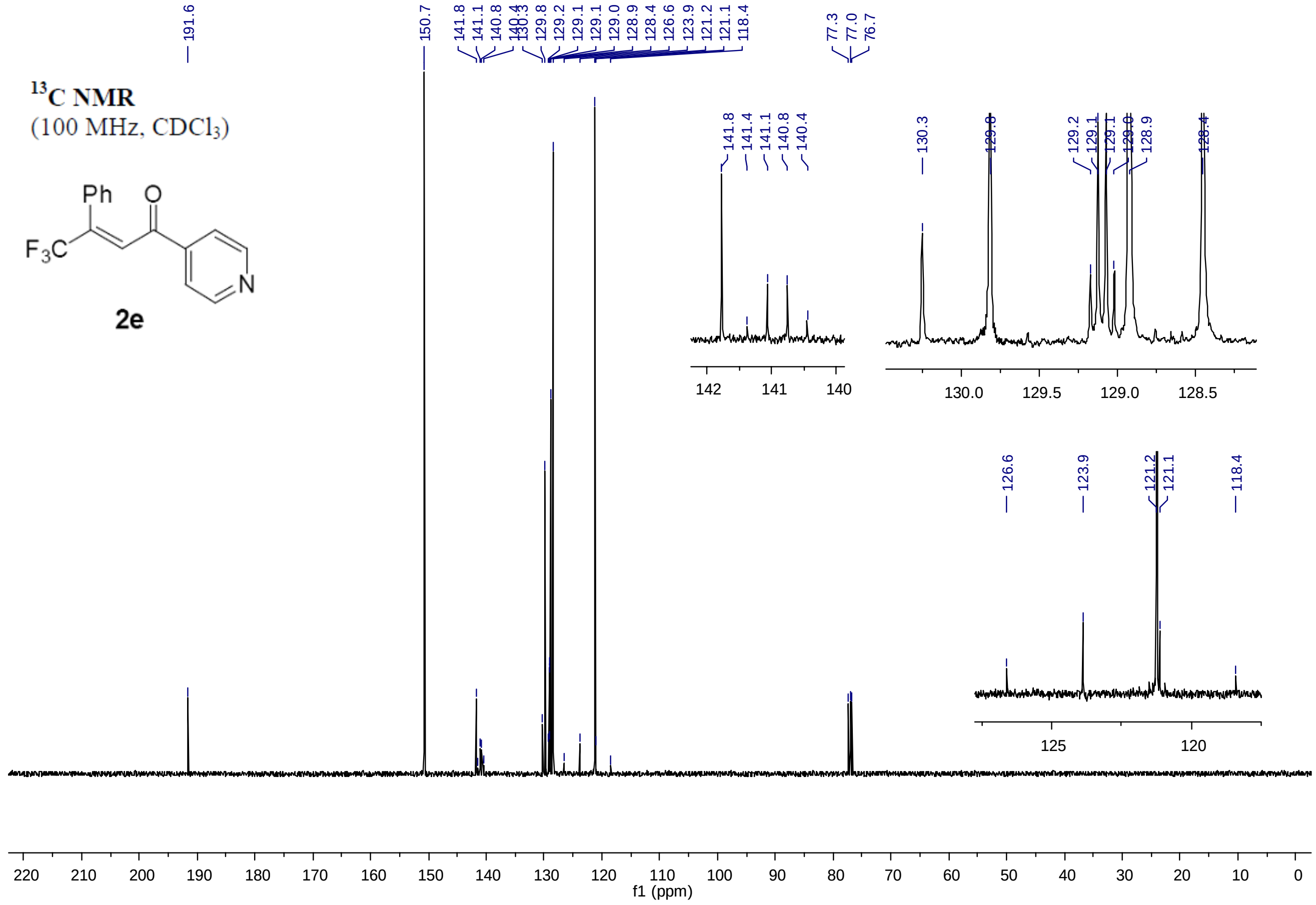

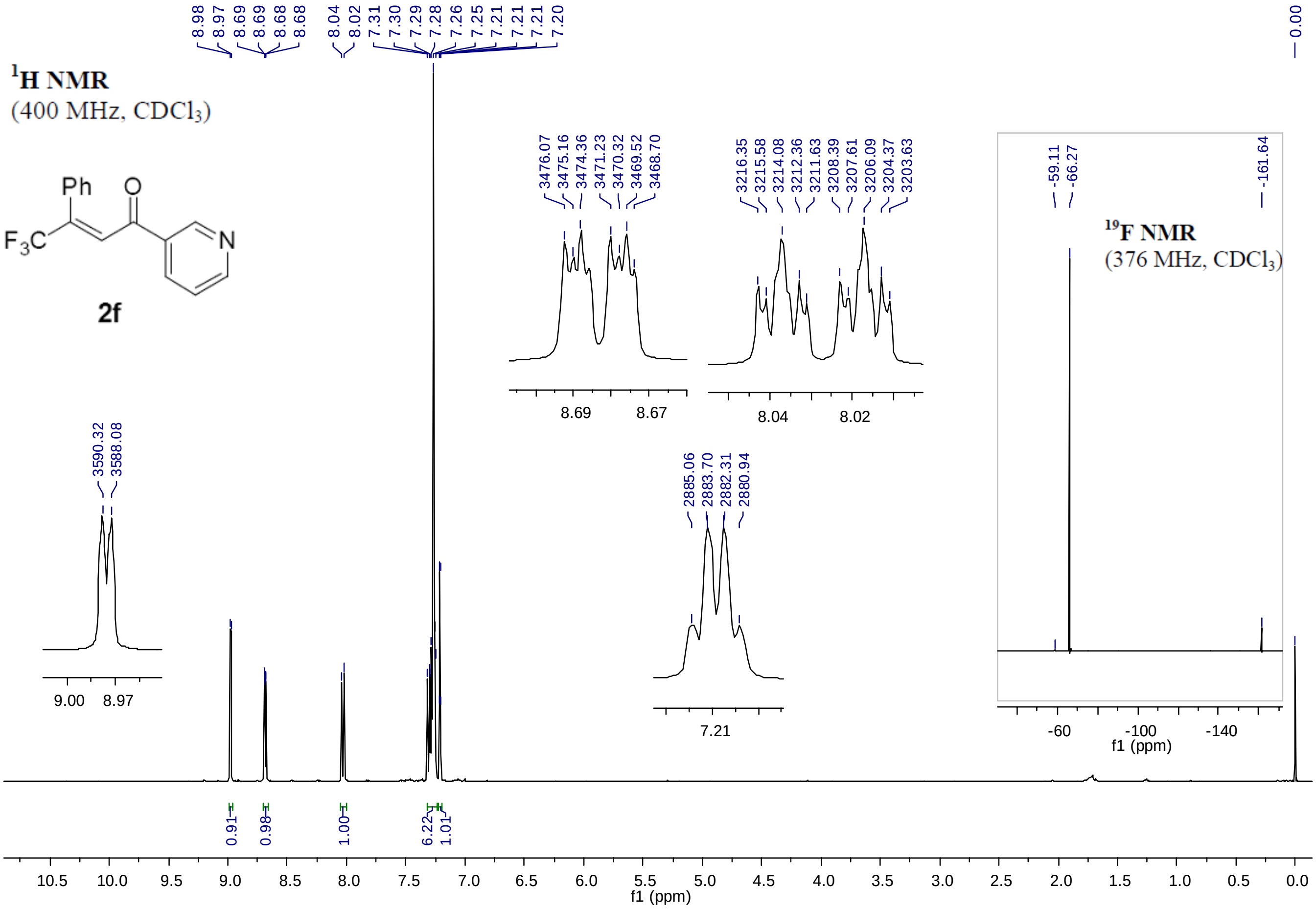

1H NMR
(400 MHz, CDCl3)
2f
19F NMR
(376 MHz, CDCl3)
f1 (ppm)

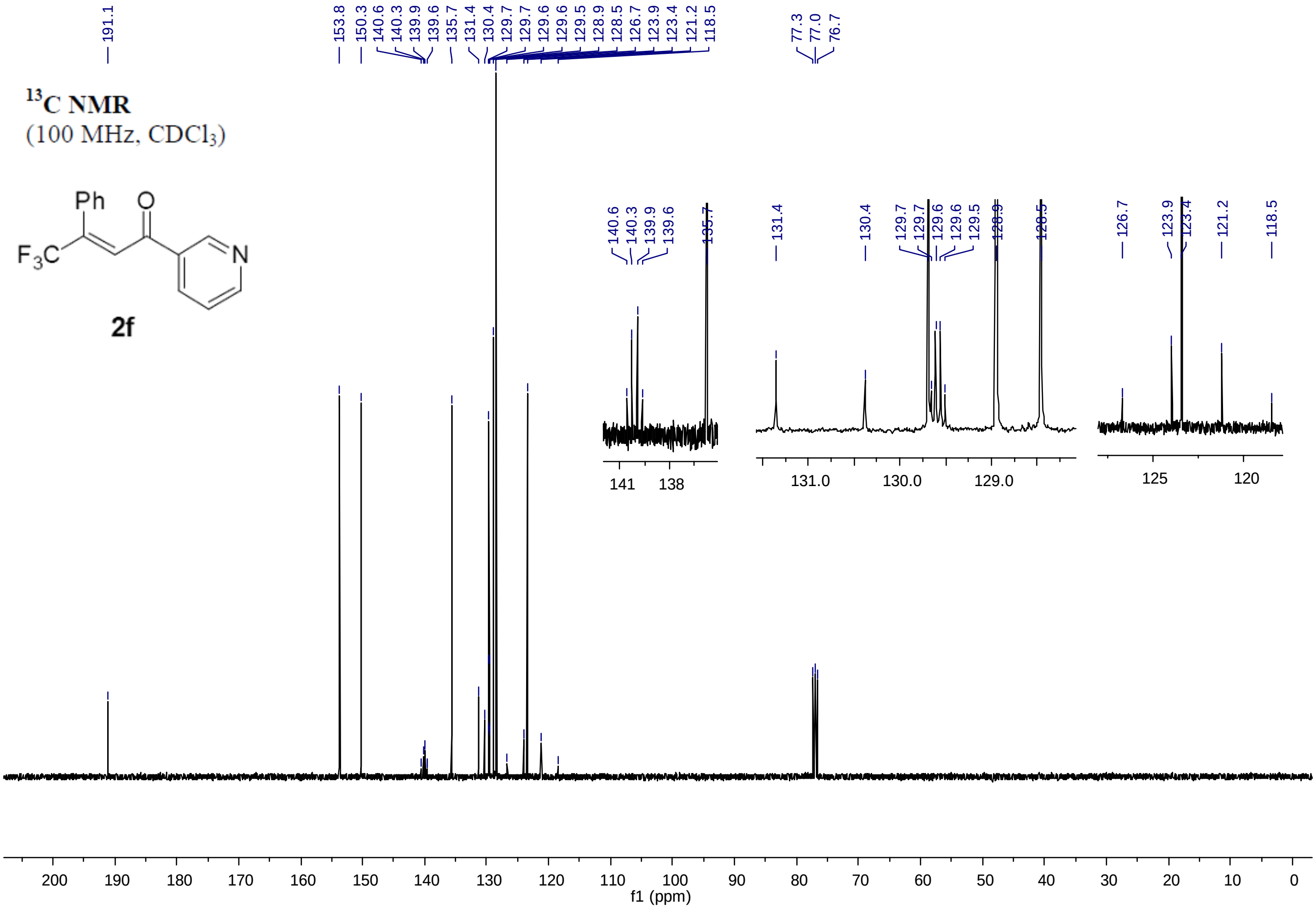

13C NMR
(100 MHz, CDCl3)
2f
f1 (ppm)

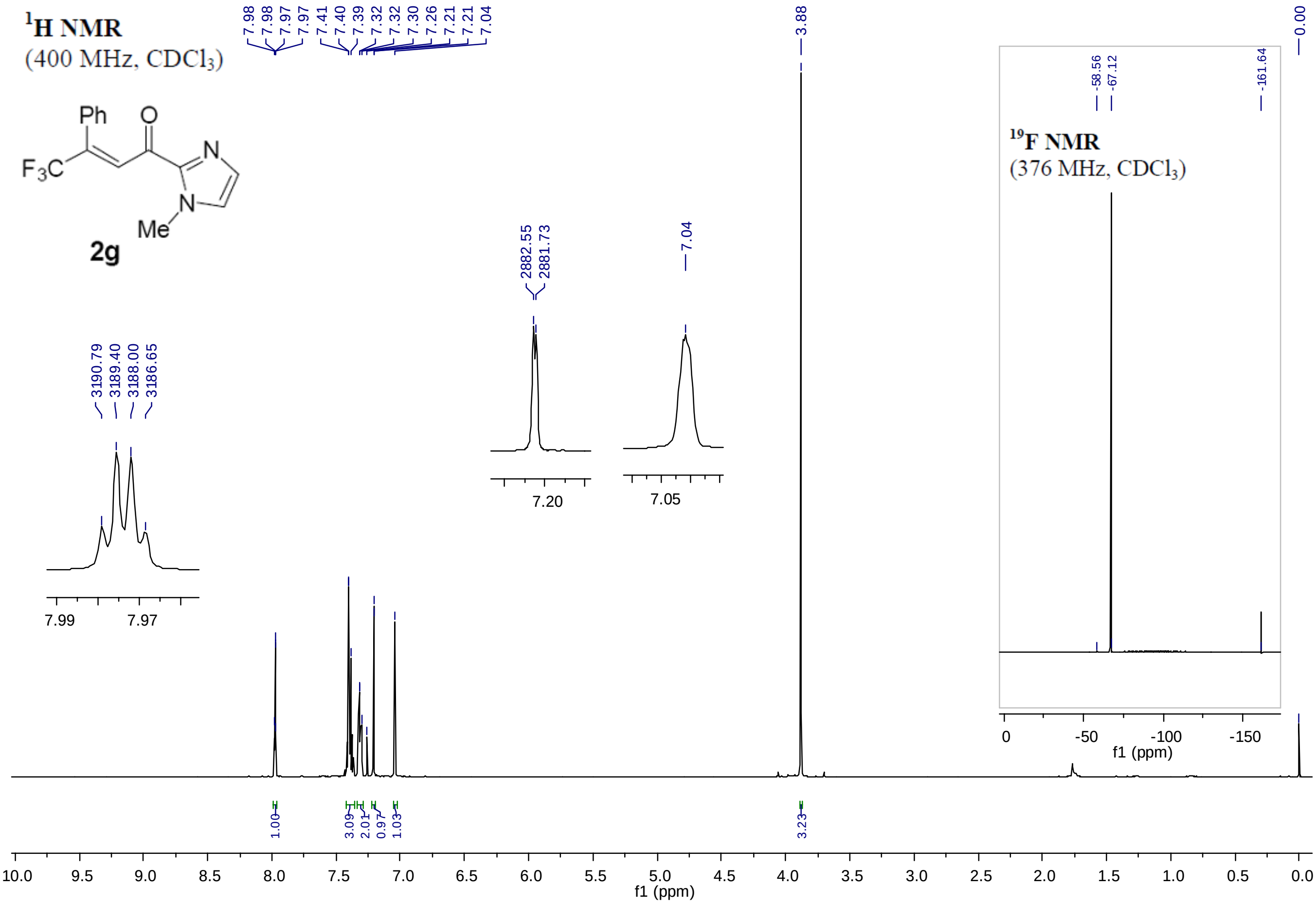

^{1}H NMR
(400 MHz, CDCl$_3$)
^{19}F NMR
(376 MHz, CDCl$_3$)
2g
f1 (ppm)

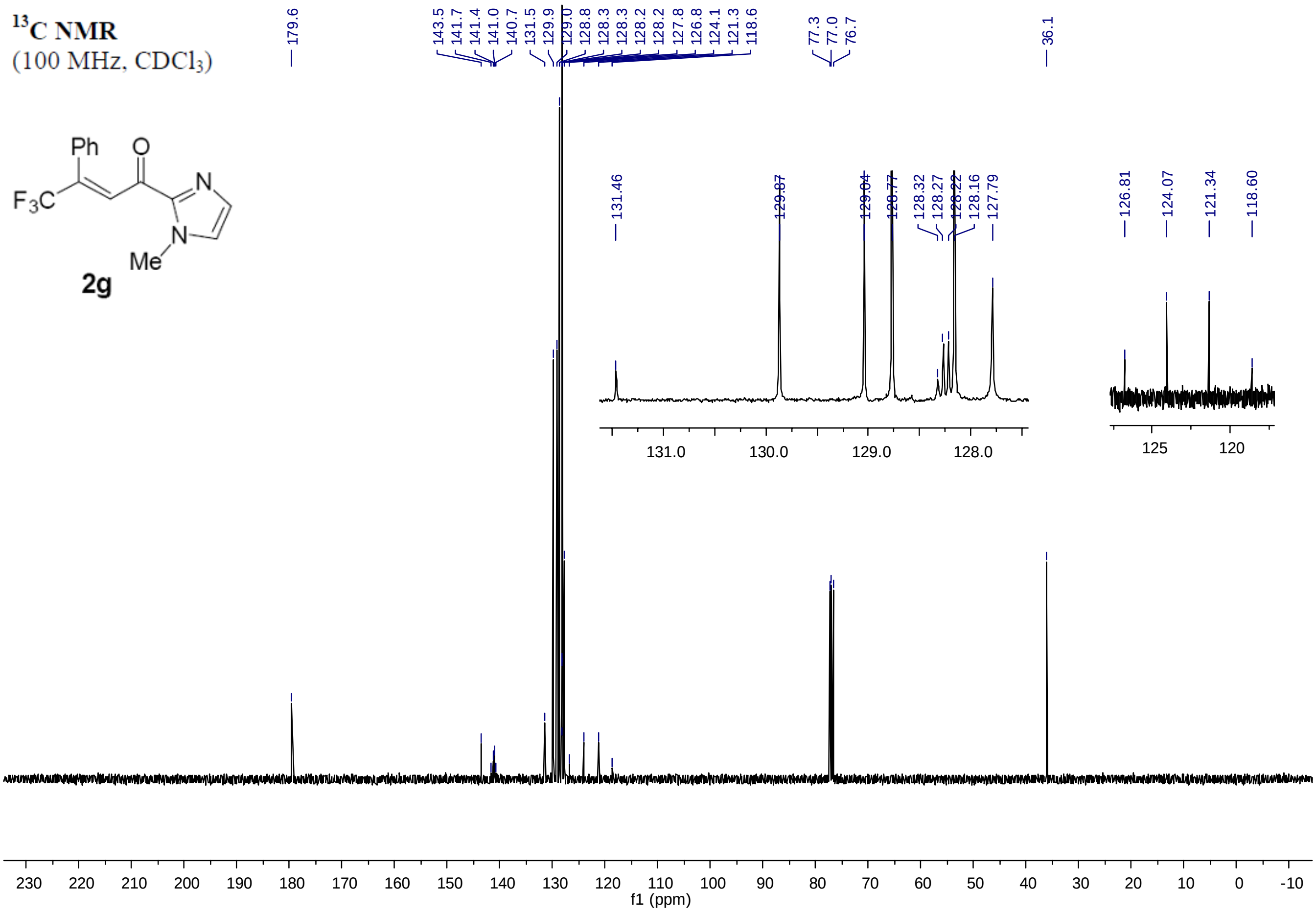

^{13}C NMR
(100 MHz, CDCl$_3$)
2g
f1 (ppm)

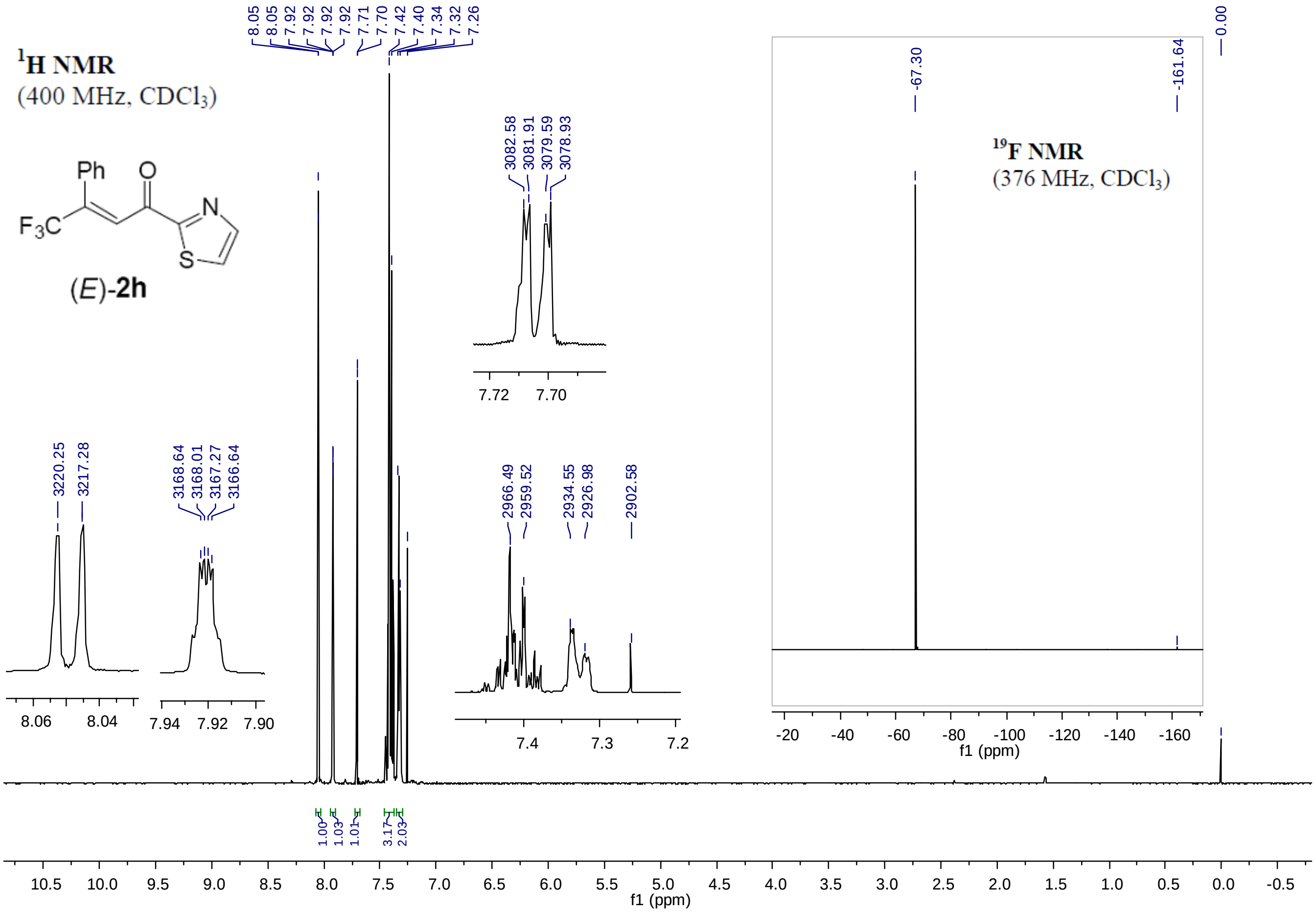
1H NMR
(400 MHz, CDCl3)
Ph
O
F3C
N
S
(E)-2h
8.05 8.05 7.92 7.92 7.92 7.92 7.71 7.70 7.42 7.40 7.34 7.32 7.26
0.00
3220.25 3217.28
3168.64 3168.01 3167.27 3166.64
3082.58 3081.91 3079.59 3078.93
2966.49 2959.52 2934.55 2926.98 2902.58
19F NMR
(376 MHz, CDCl3)
-67.30
-161.64
1.00 1.03 1.01 3.17 2.03
f1 (ppm)

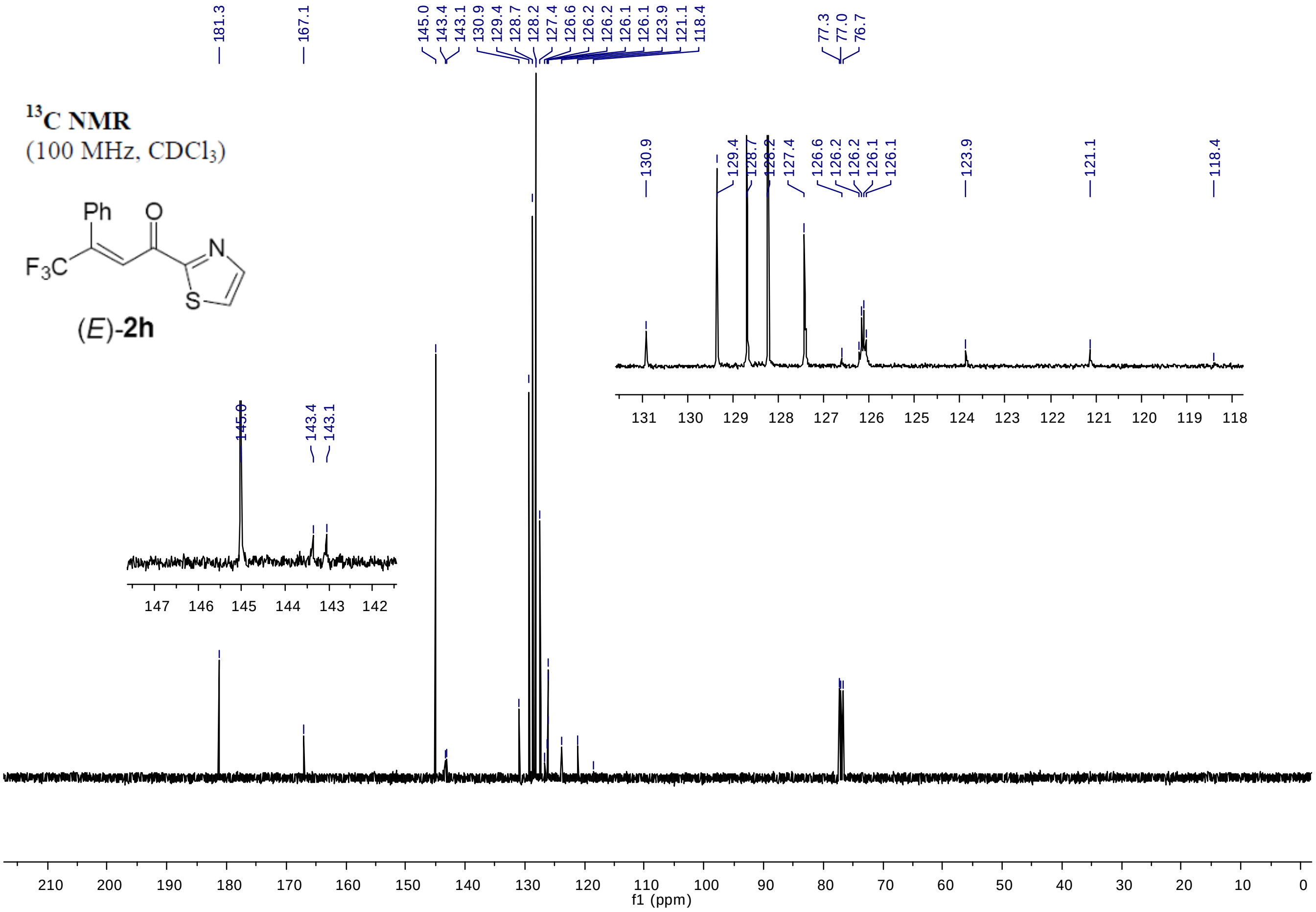
13C NMR
(100 MHz, CDCl3)
Ph
O
F3C
N
S
(E)-2h
181.3
167.1
145.0 143.4 143.1 130.9 129.4 128.7 128.2 127.4 126.6 126.2 126.2 126.1 126.1 123.9 121.1 118.4
77.3 77.0 76.7
f1 (ppm)

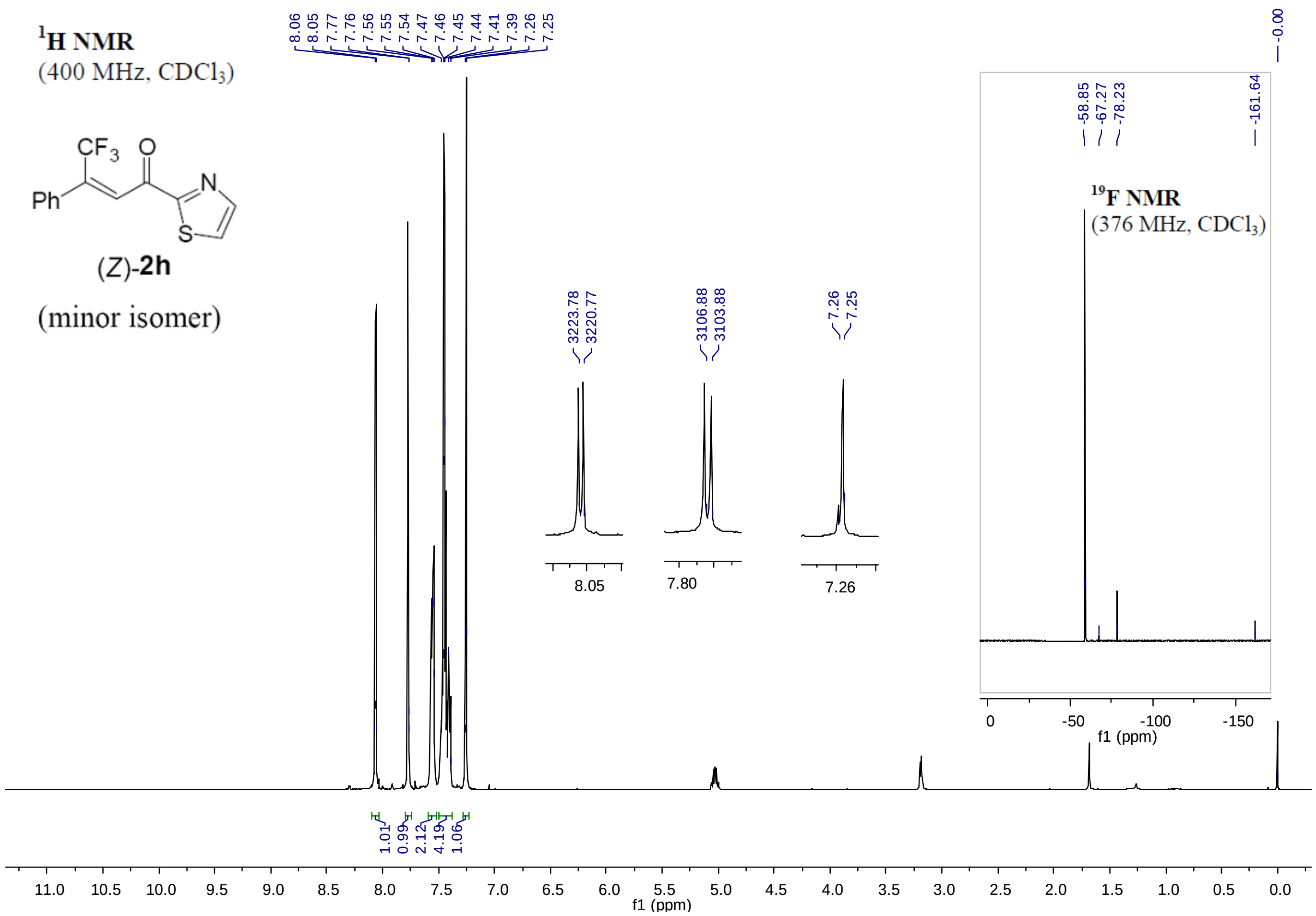

1H NMR
(400 MHz, CDCl3)
CF3 O
Ph
N
S
(Z)-2h
(minor isomer)
8.06 8.05 7.77 7.76 7.56 7.55 7.54 7.47 7.46 7.45 7.44 7.41 7.39 7.26 7.25
-0.00
3223.78 3220.77
3106.88 3103.88
7.26 7.25
8.05
7.80
7.26
-58.85 -67.27 -78.23 -161.64
19F NMR
(376 MHz, CDCl3)
0 -50 -100 -150
f1 (ppm)
1.01 0.99 2.12 4.19 1.06
11.0 10.5 10.0 9.5 9.0 8.5 8.0 7.5 7.0 6.5 6.0 5.5 5.0 4.5 4.0 3.5 3.0 2.5 2.0 1.5 1.0 0.5 0.0
f1 (ppm)

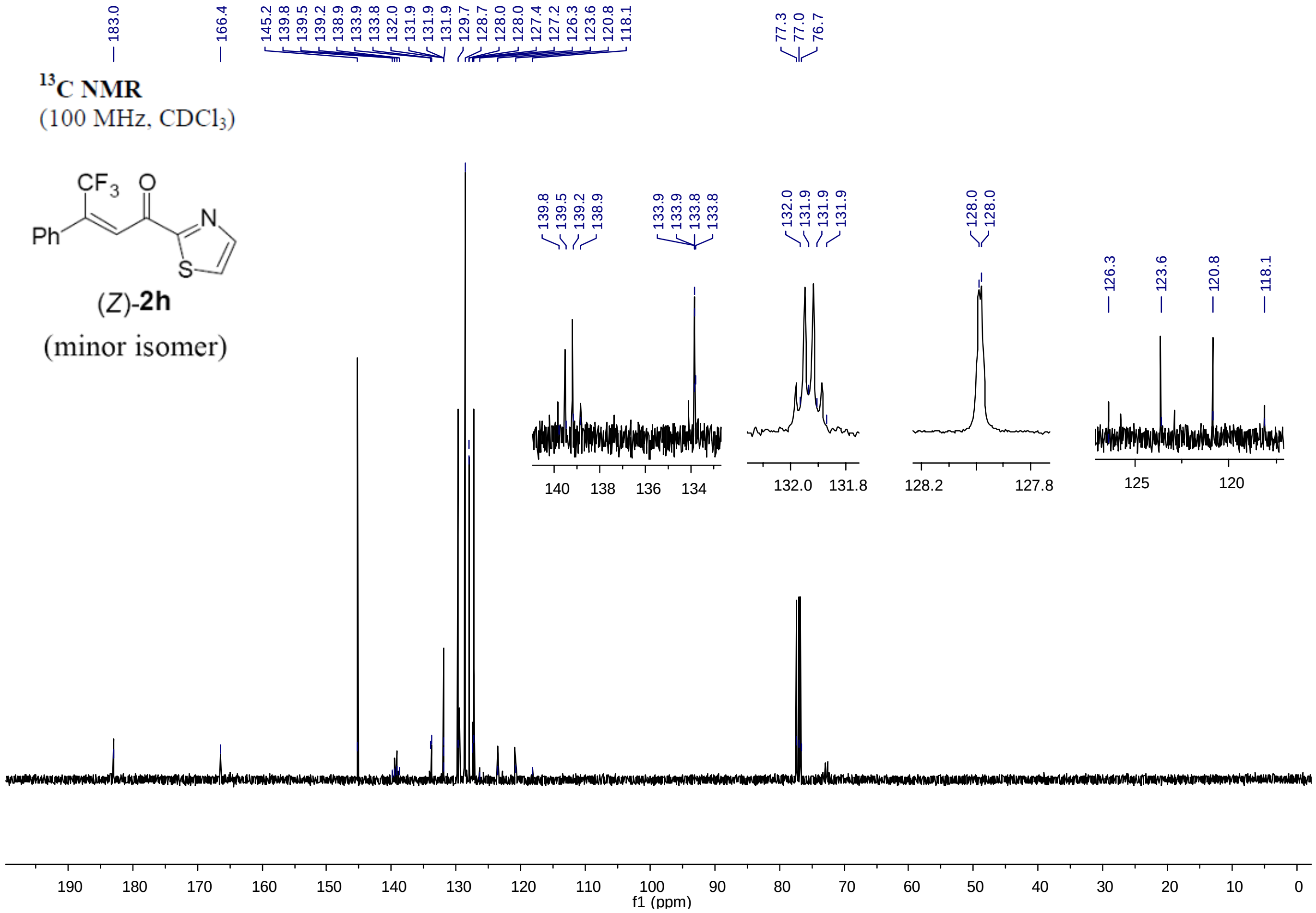

183.0
166.4
145.2 139.8 139.5 139.2 138.9 133.9 133.8 132.0 131.9 131.9 129.7 128.7 128.0 128.0 127.4 127.2 126.3 123.6 120.8 118.1
77.3 77.0 76.7
13C NMR
(100 MHz, CDCl3)
CF3 O
Ph
N
S
(Z)-2h
(minor isomer)
139.8 139.5 139.2 138.9
133.9 133.9 133.8 133.8
132.0 131.9 131.9 131.9
128.0 128.0
126.3
123.6
120.8
118.1
140 138 136 134
132.0 131.8
128.2 127.8
125 120
190 180 170 160 150 140 130 120 110 100 90 80 70 60 50 40 30 20 10 0
f1 (ppm)

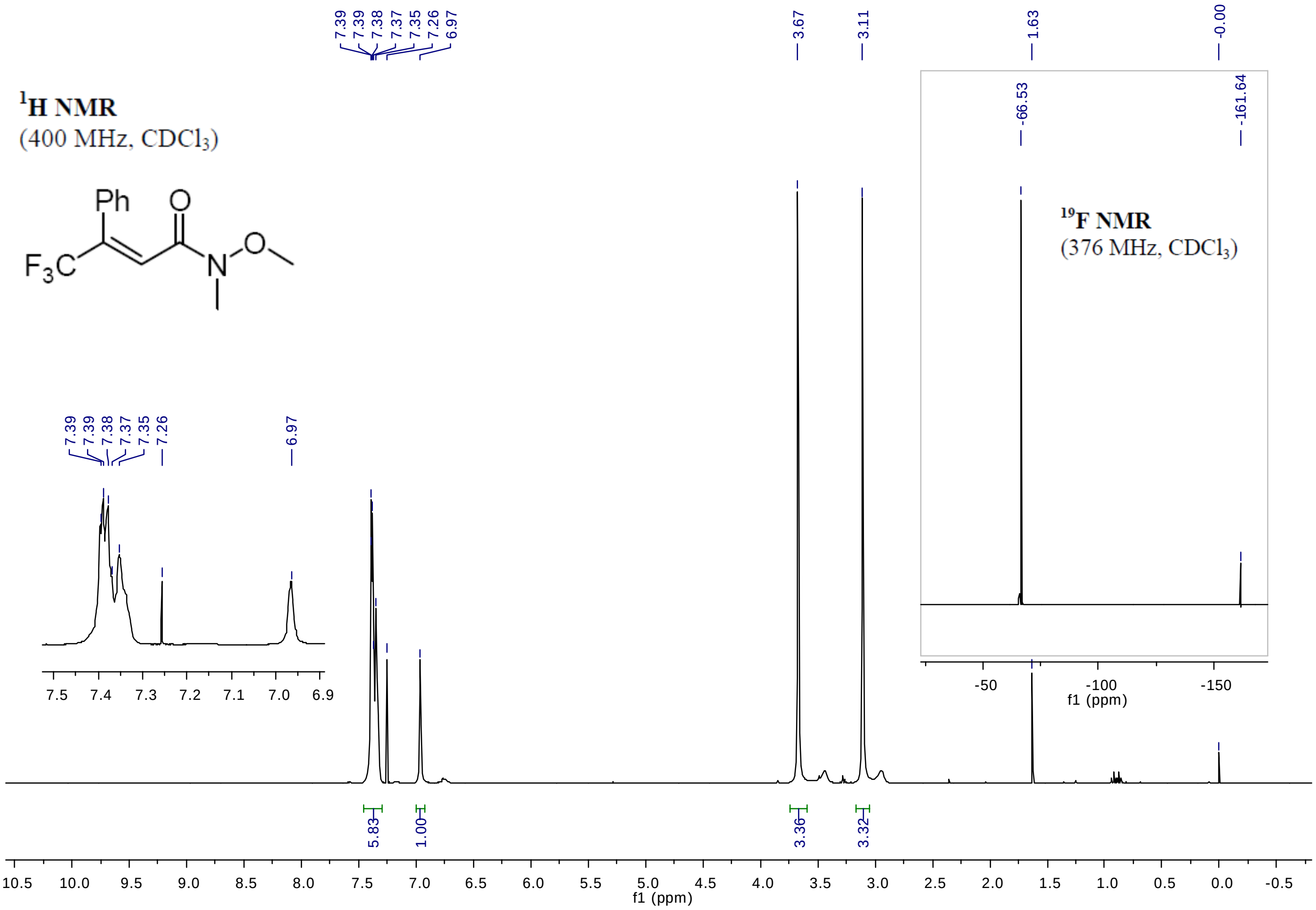
1H NMR
(400 MHz, CDCl3)
Ph
O
F3C
N
O
7.39
7.39
7.38
7.37
7.35
7.26
6.97
3.67
3.11
1.63
-0.00
19F NMR
(376 MHz, CDCl3)
-66.53
-161.64
-50
-100
-150
f1 (ppm)
7.5 7.4 7.3 7.2 7.1 7.0 6.9
5.83
1.00
3.36
3.32
10.5 10.0 9.5 9.0 8.5 8.0 7.5 7.0 6.5 6.0 5.5 5.0 4.5 4.0 3.5 3.0 2.5 2.0 1.5 1.0 0.5 0.0 -0.5
f1 (ppm)

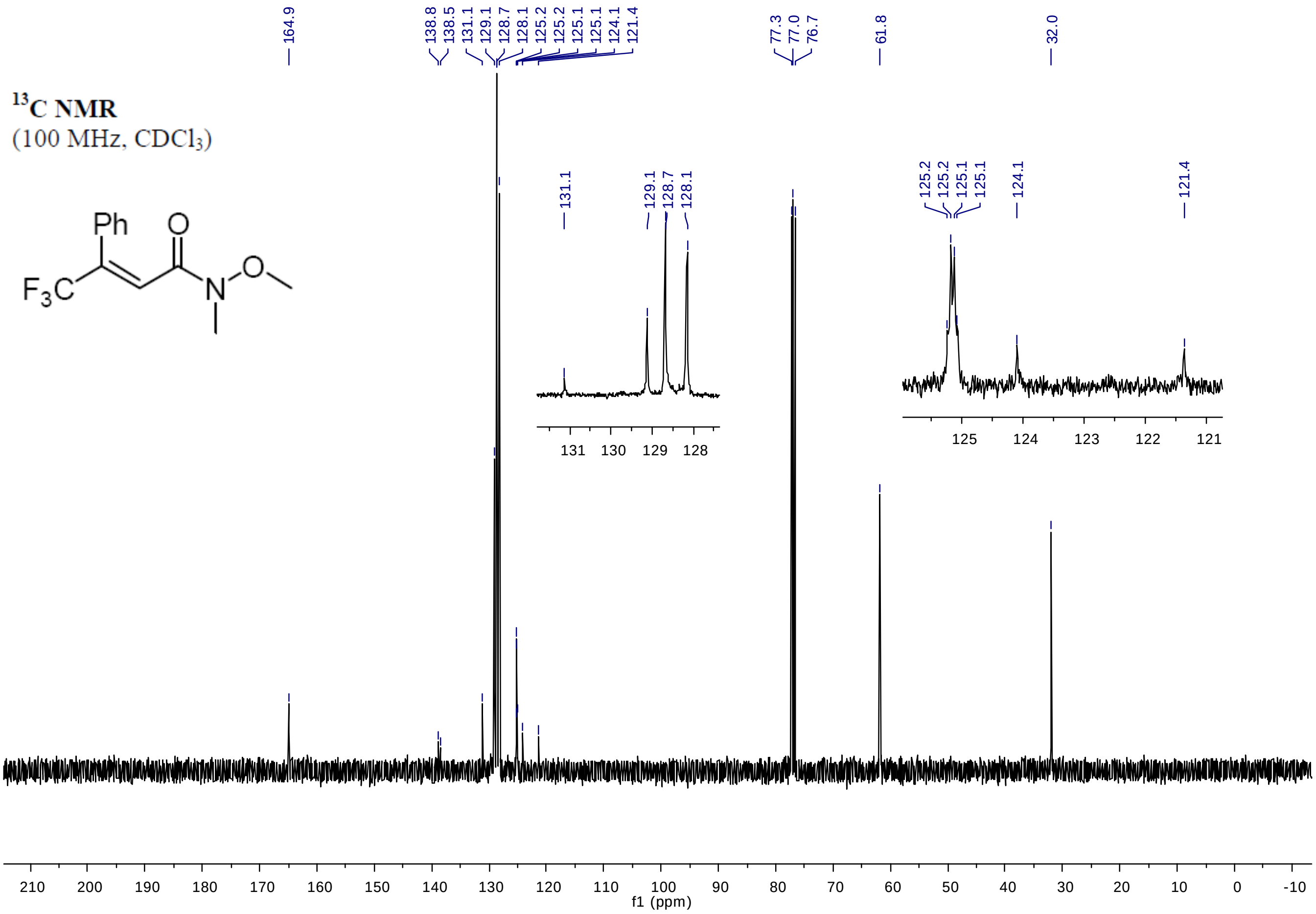
13C NMR
(100 MHz, CDCl3)
Ph
O
F3C
N
O
164.9
138.8
138.5
131.1
129.1
128.7
128.1
125.2
125.2
125.1
125.1
124.1
121.4
77.3
77.0
76.7
61.8
32.0
131 130 129 128
125 124 123 122 121
210 200 190 180 170 160 150 140 130 120 110 100 90 80 70 60 50 40 30 20 10 0 -10
f1 (ppm)

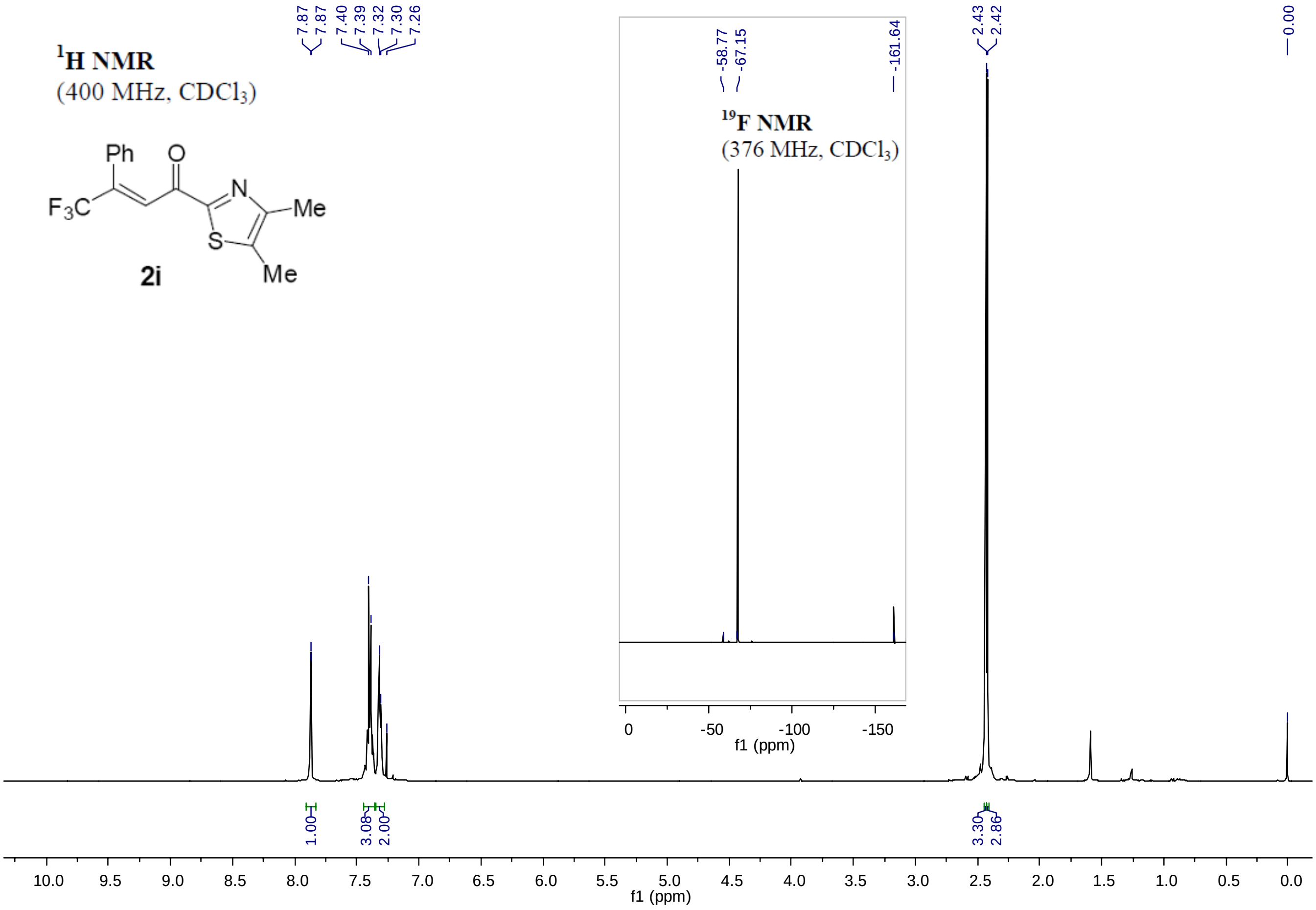
1H NMR
(400 MHz, CDCl3)
Ph
O
F3C
N
Me
S
Me
2i
7.87
7.87
7.40
7.39
7.32
7.30
7.26
-58.77
-67.15
-161.64
19F NMR
(376 MHz, CDCl3)
0
-50
-100
-150
f1 (ppm)
2.43
2.42
0.00
1.00
3.08
2.00
3.30
2.86
10.0
9.5
9.0
8.5
8.0
7.5
7.0
6.5
6.0
5.5
5.0
4.5
4.0
3.5
3.0
2.5
2.0
1.5
1.0
0.5
0.0
f1 (ppm)

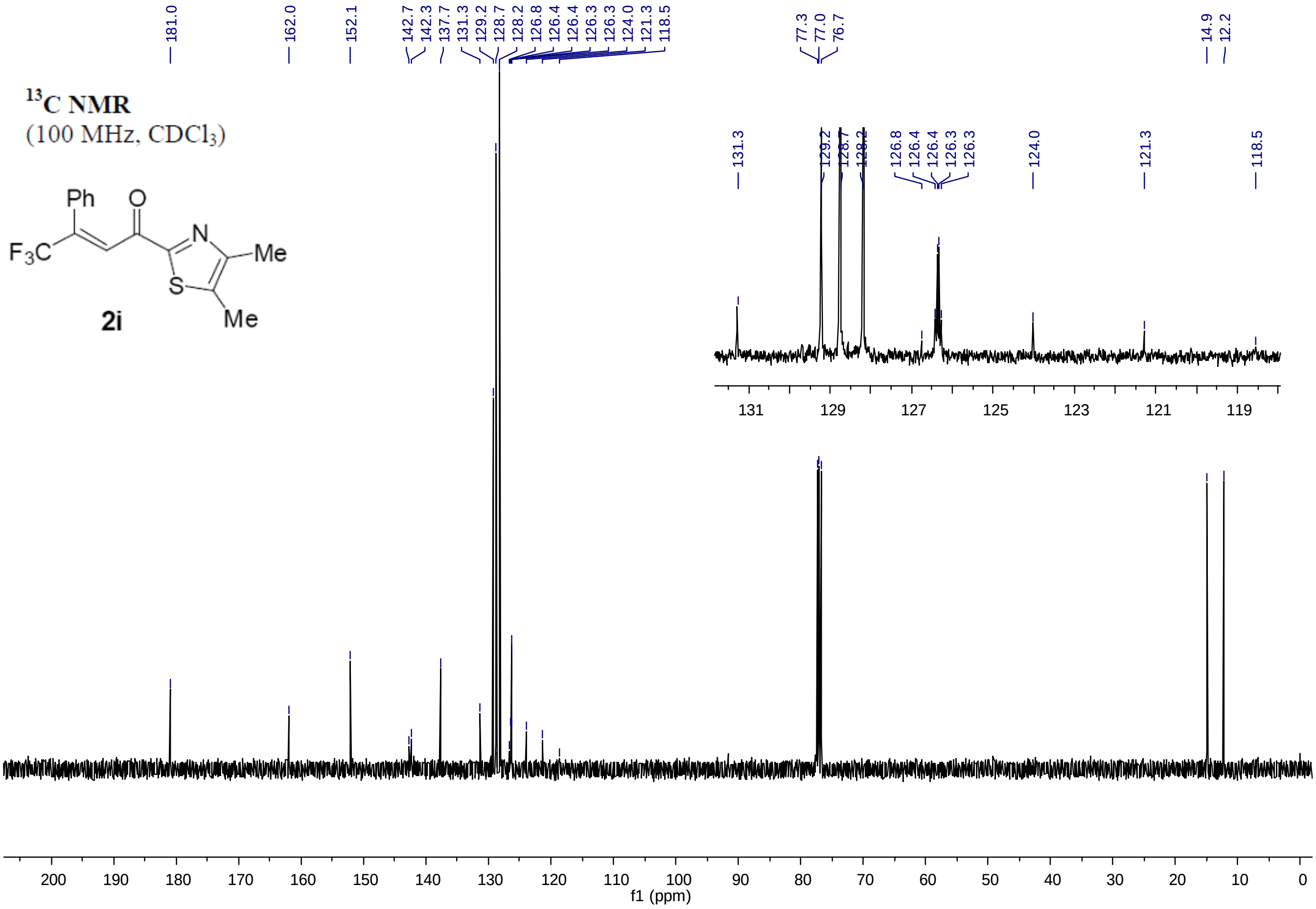
181.0
162.0
152.1
142.7
142.3
137.7
131.3
129.2
128.7
128.2
126.8
126.4
126.4
126.3
126.3
124.0
121.3
118.5
77.3
77.0
76.7
14.9
12.2
13C NMR
(100 MHz, CDCl3)
Ph
O
F3C
N
Me
S
Me
2i
131.3
129.2
128.7
128.2
126.8
126.4
126.4
126.3
126.3
124.0
121.3
118.5
131
129
127
125
123
121
119
200
190
180
170
160
150
140
130
120
110
100
90
80
70
60
50
40
30
20
10
0
f1 (ppm)

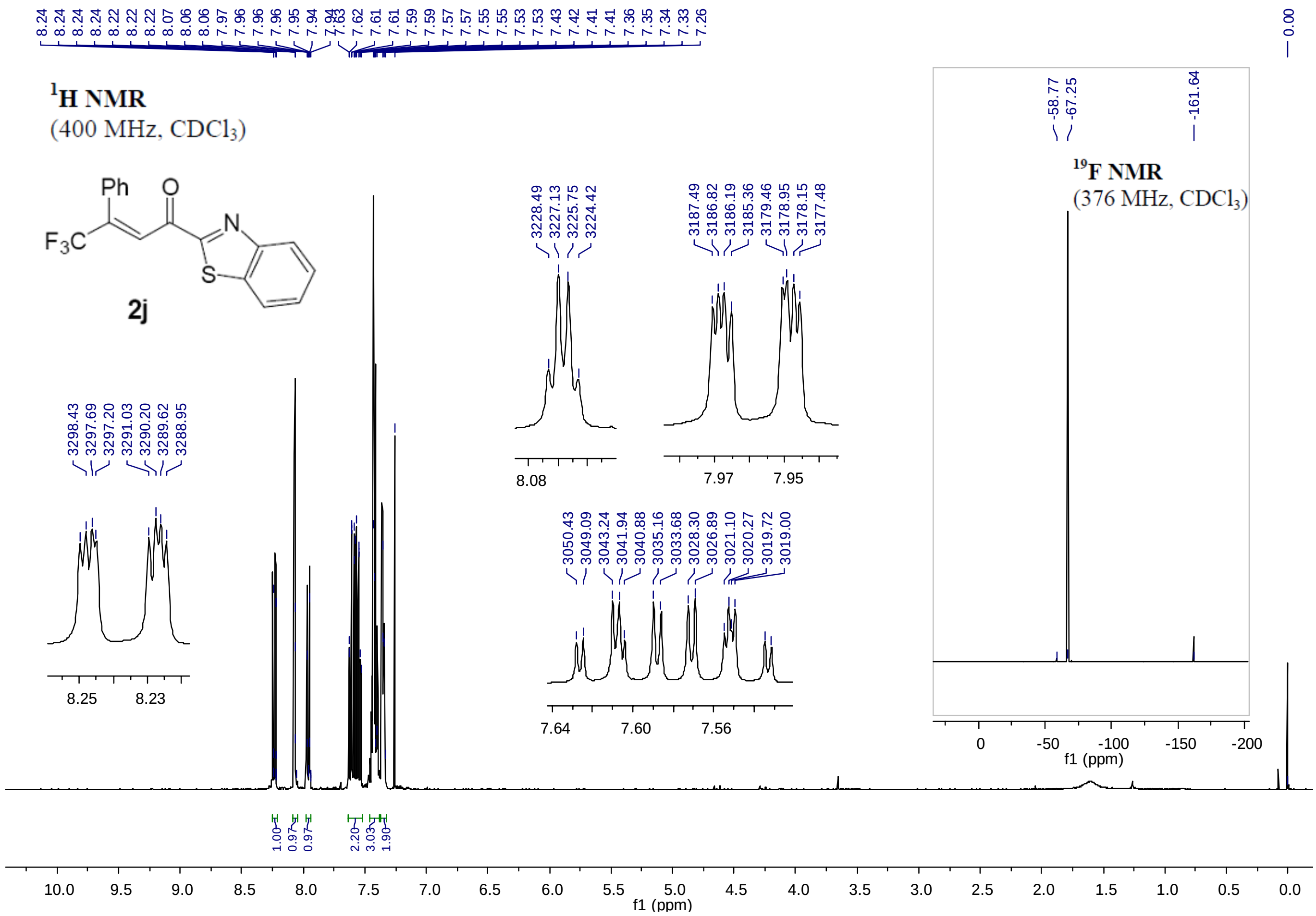

1H NMR
(400 MHz, CDCl3)
19F NMR
(376 MHz, CDCl3)
2j
f1 (ppm)

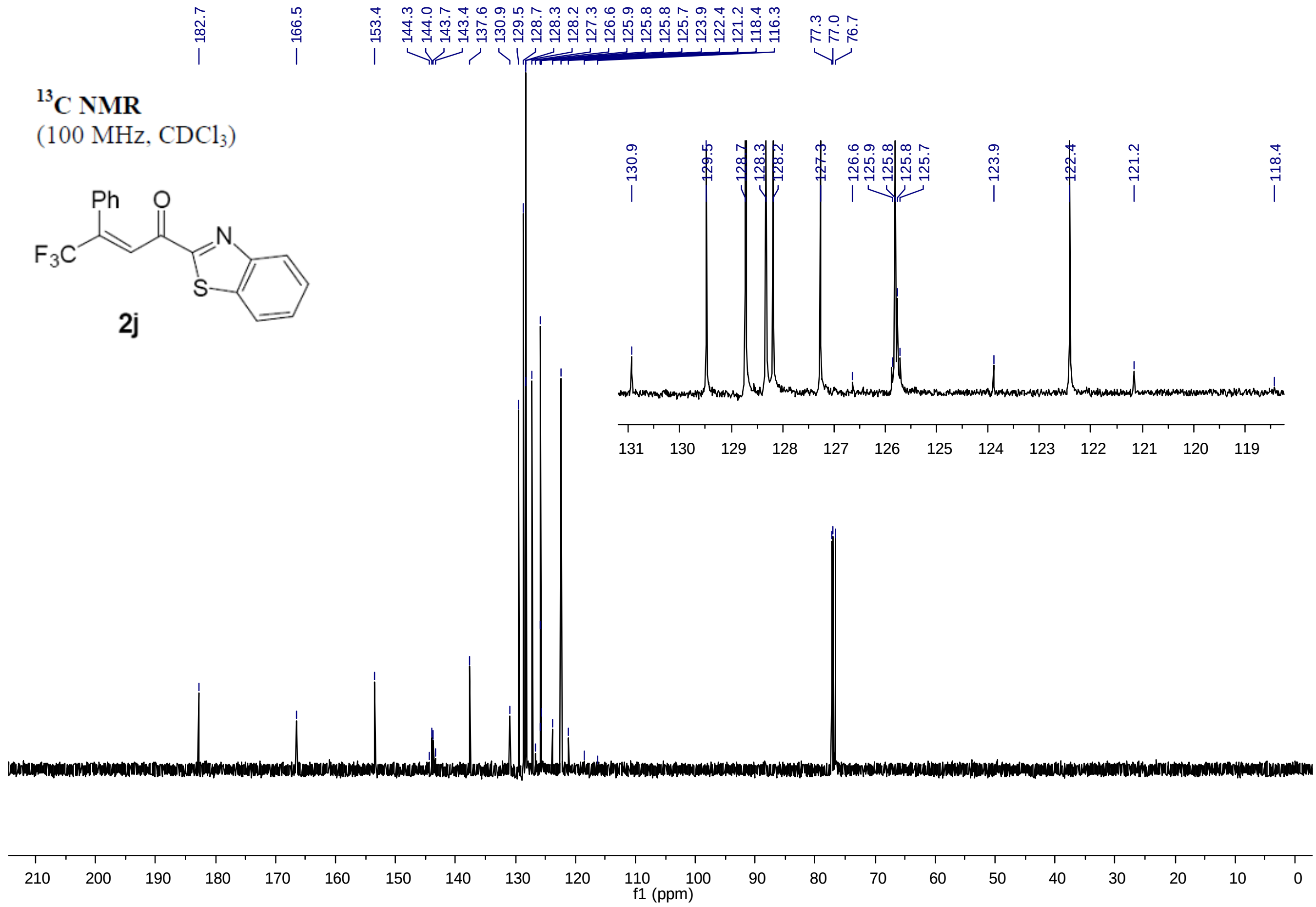

13C NMR
(100 MHz, CDCl3)
2j
f1 (ppm)

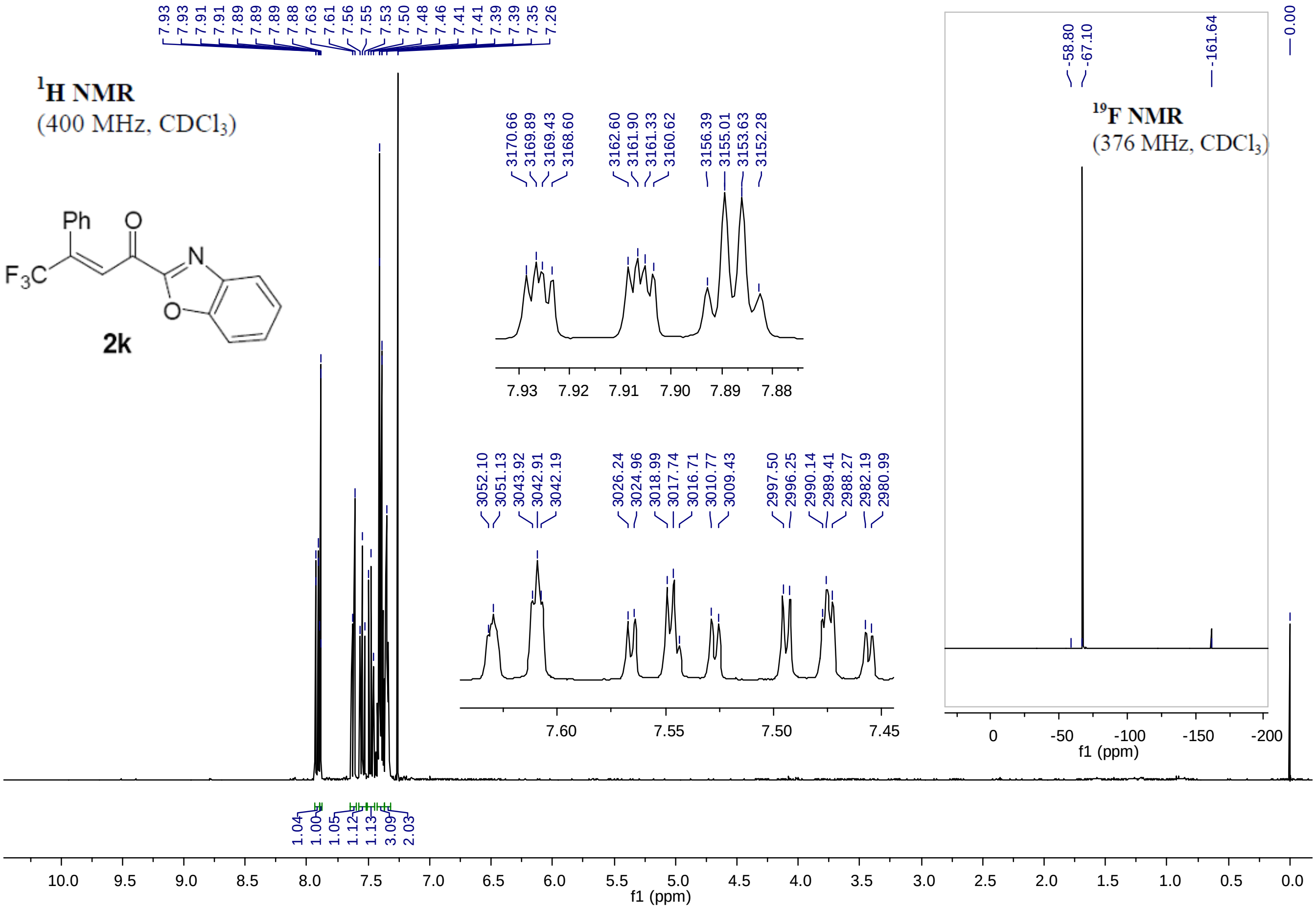
1H NMR
(400 MHz, CDCl3)
Ph
O
F3C
N
O
2k
19F NMR
(376 MHz, CDCl3)
f1 (ppm)

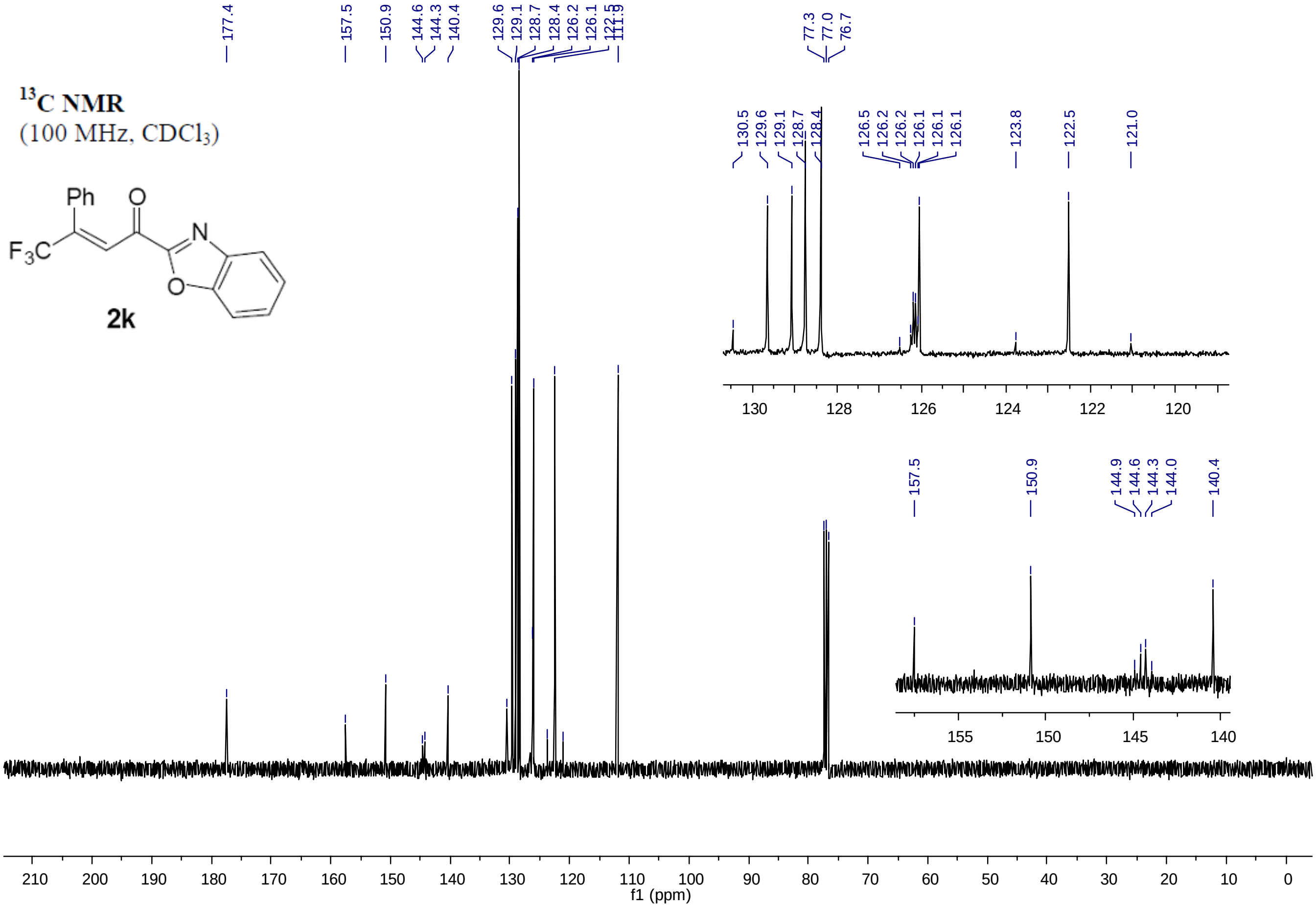
13C NMR
(100 MHz, CDCl3)
Ph
O
F3C
N
O
2k
f1 (ppm)

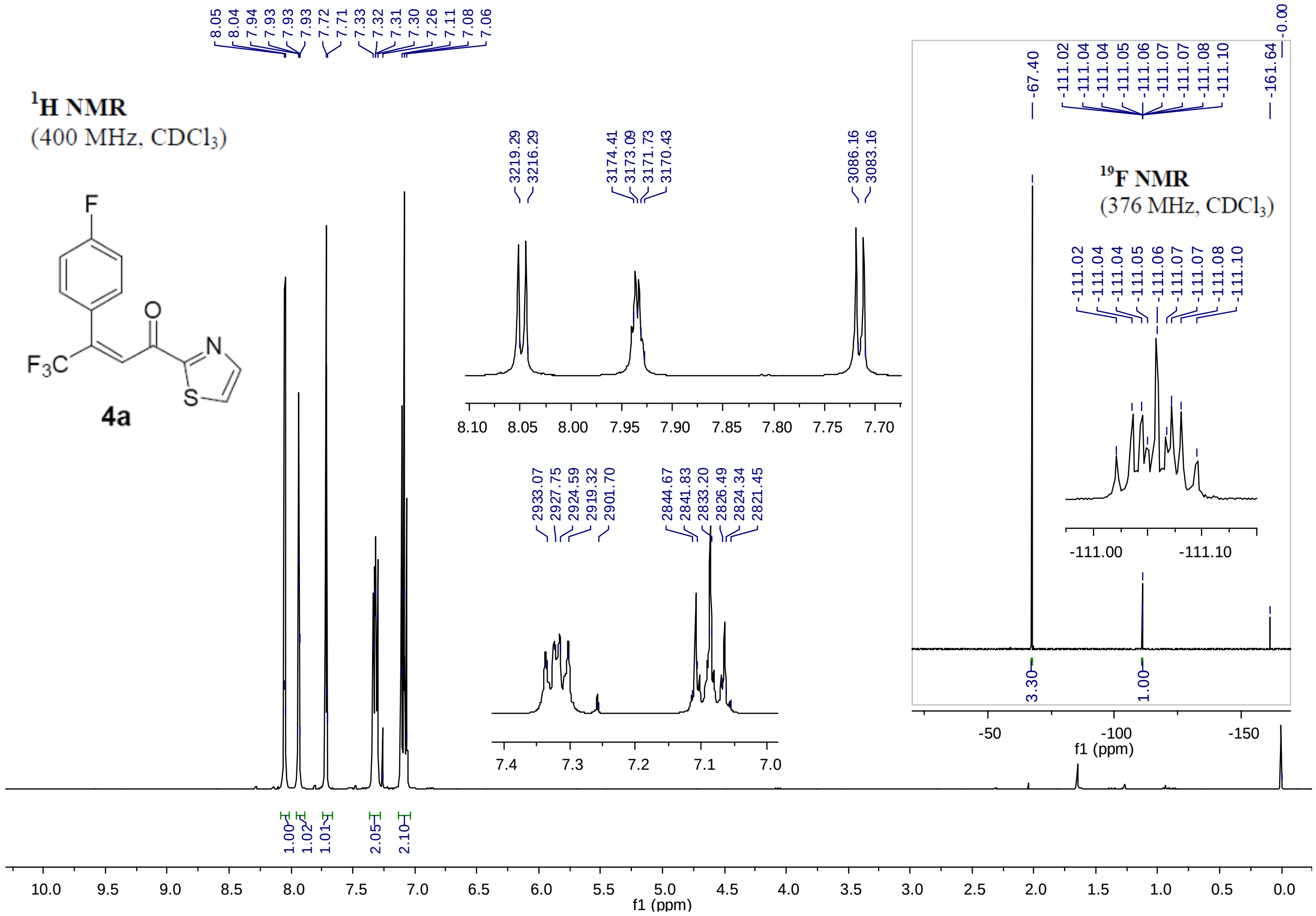

^{1}H NMR
(400 MHz, CDCl$_3$)
^{19}F NMR
(376 MHz, CDCl$_3$)
4a
f1 (ppm)

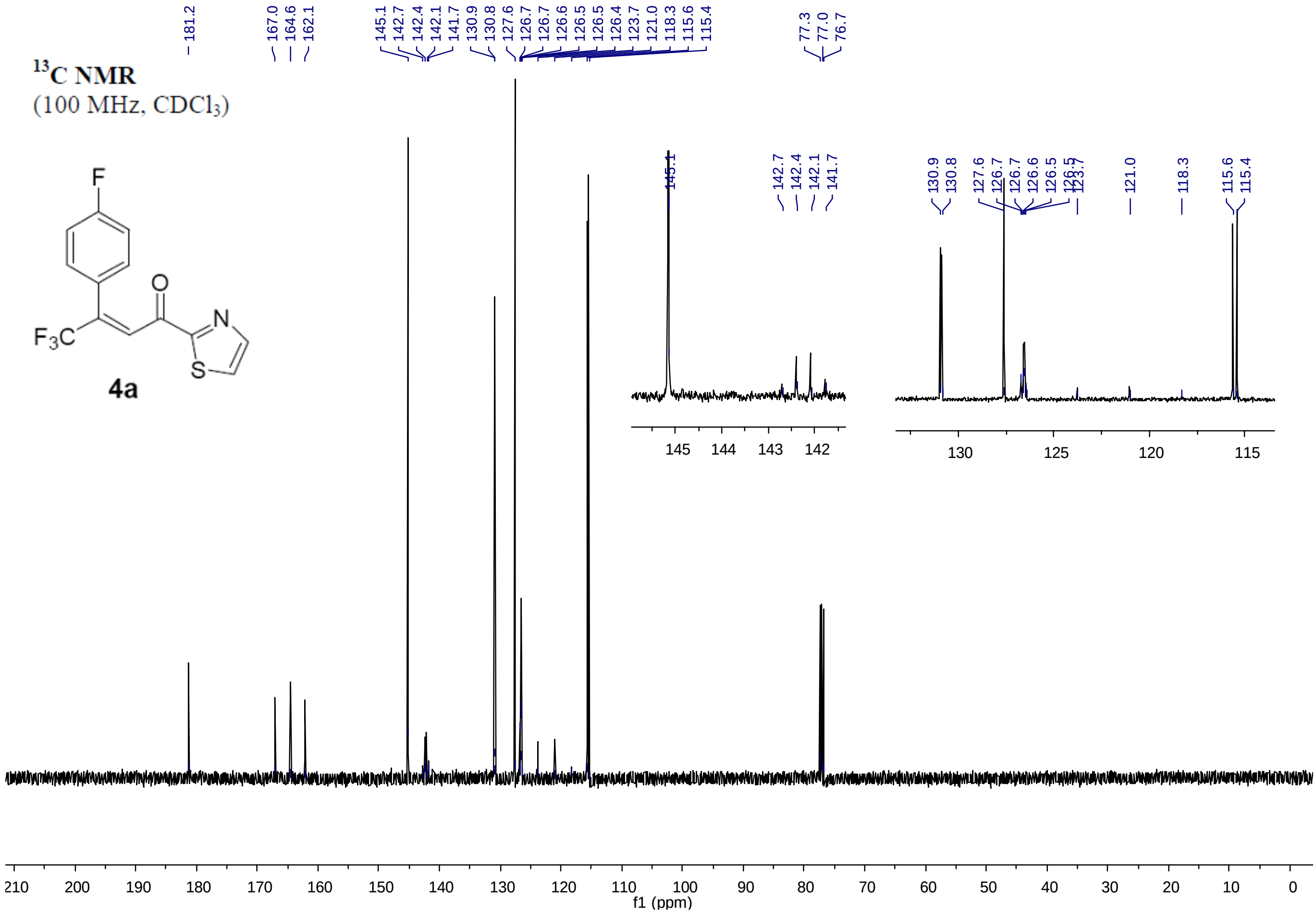

^{13}C NMR
(100 MHz, CDCl$_3$)
4a
f1 (ppm)

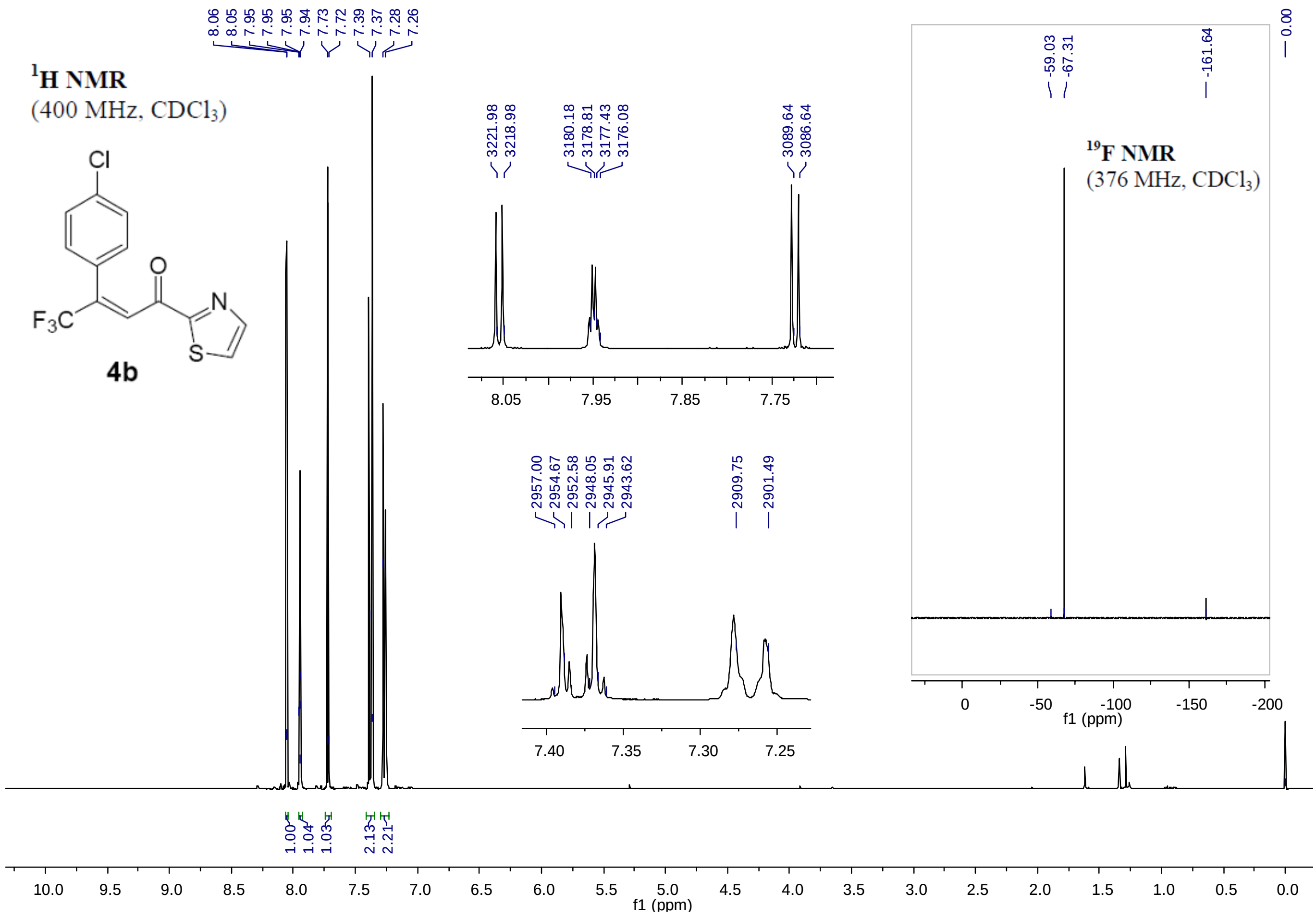

¹H NMR
(400 MHz, CDCl₃)
4b
¹⁹F NMR
(376 MHz, CDCl₃)
f1 (ppm)

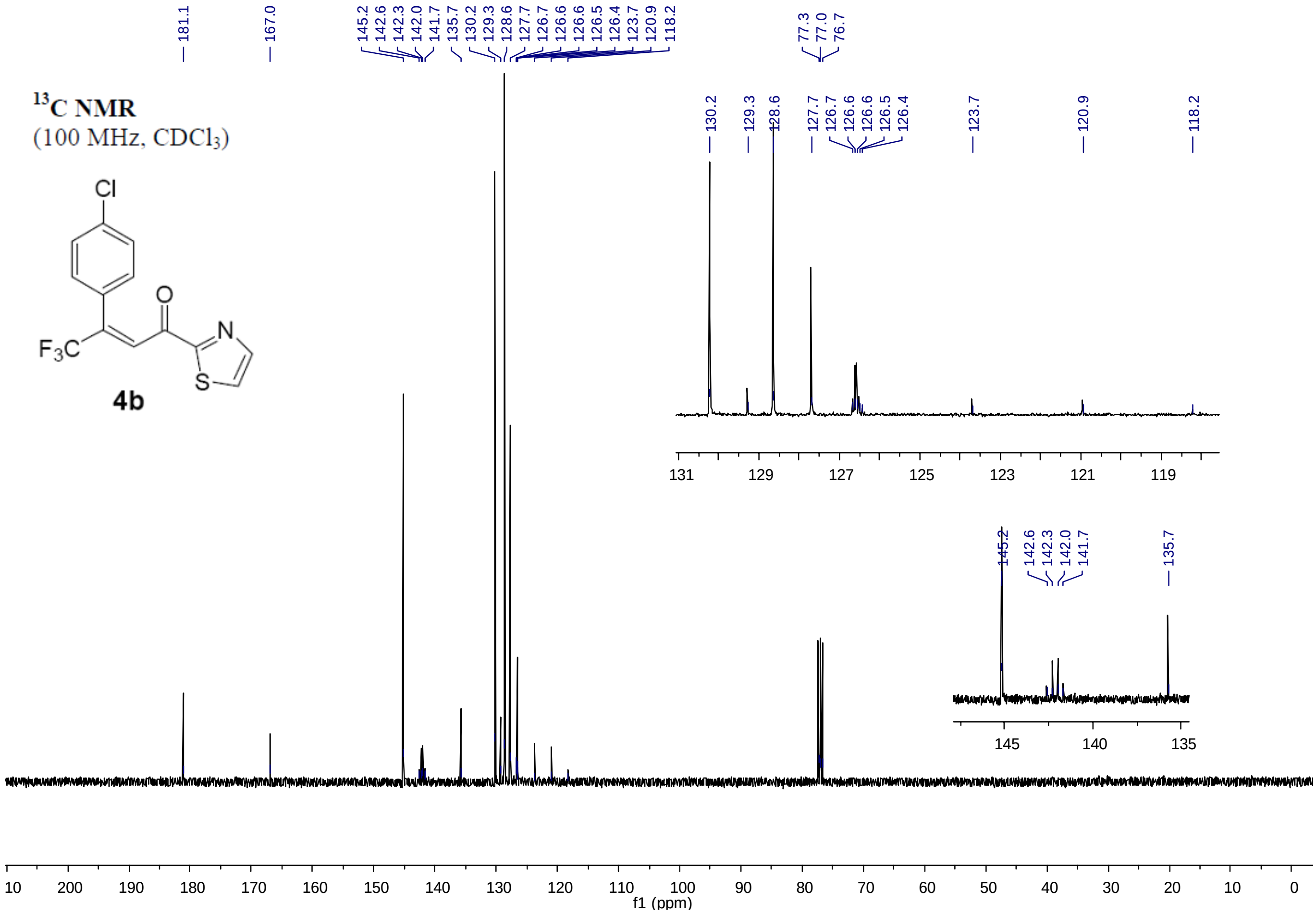

¹³C NMR
(100 MHz, CDCl₃)
4b
f1 (ppm)

^{1}H NMR
(400 MHz, $CDCl_3$)

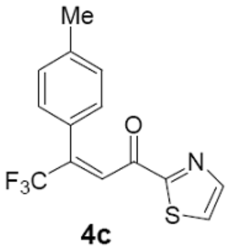

4c

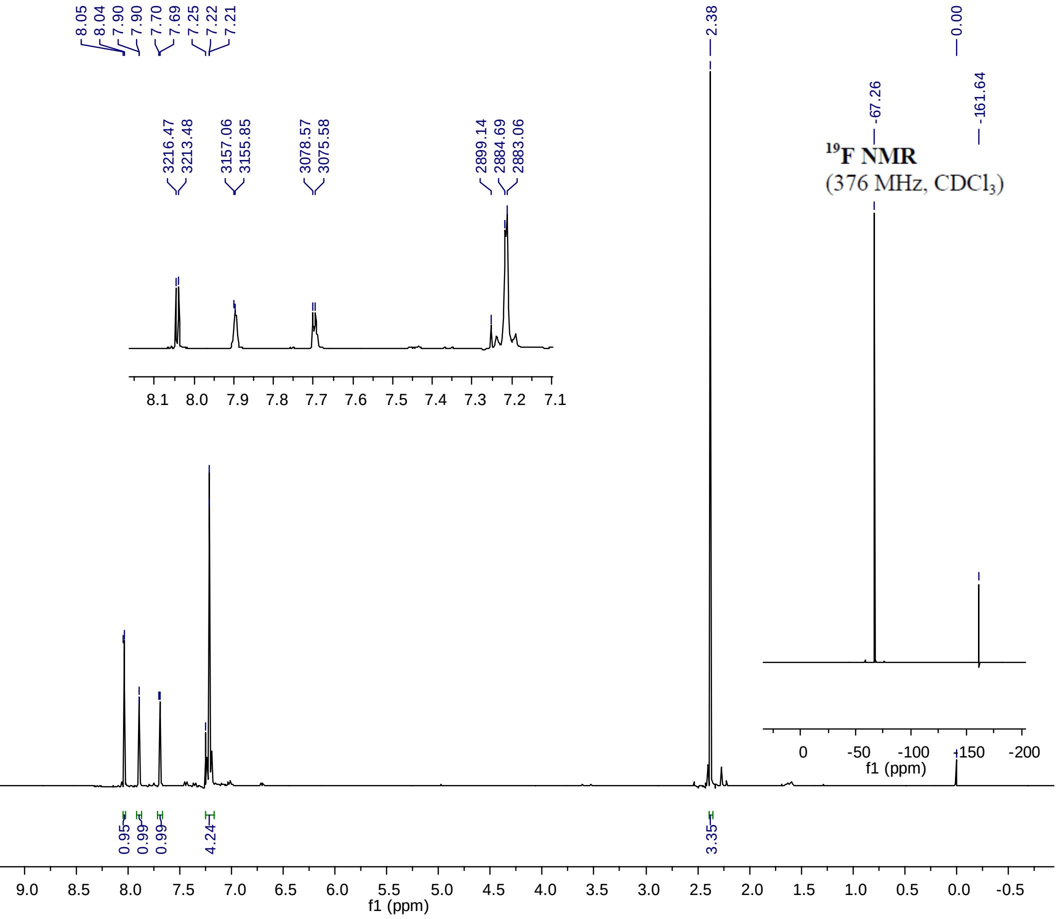

^{13}C NMR
(100 MHz, $CDCl_3$)

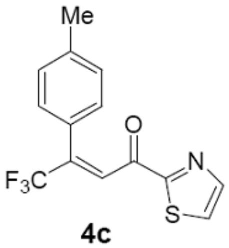

4c

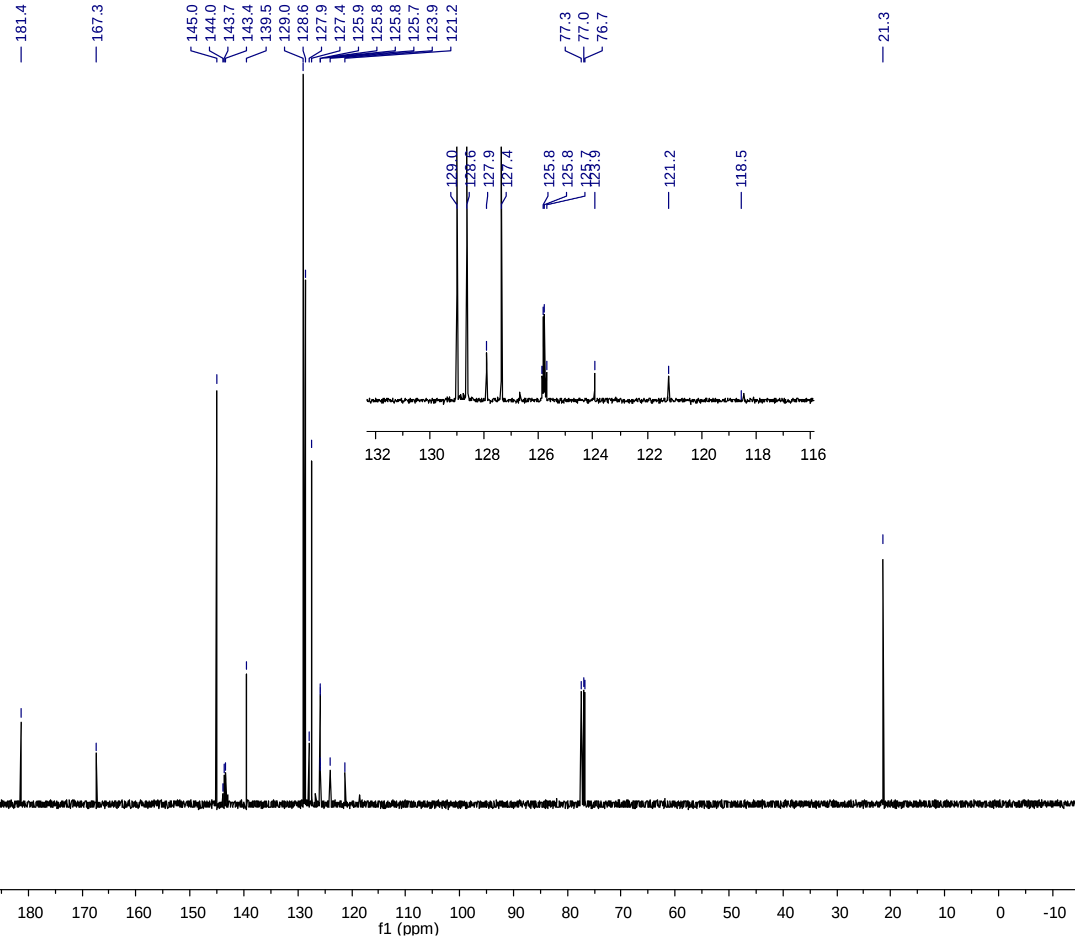

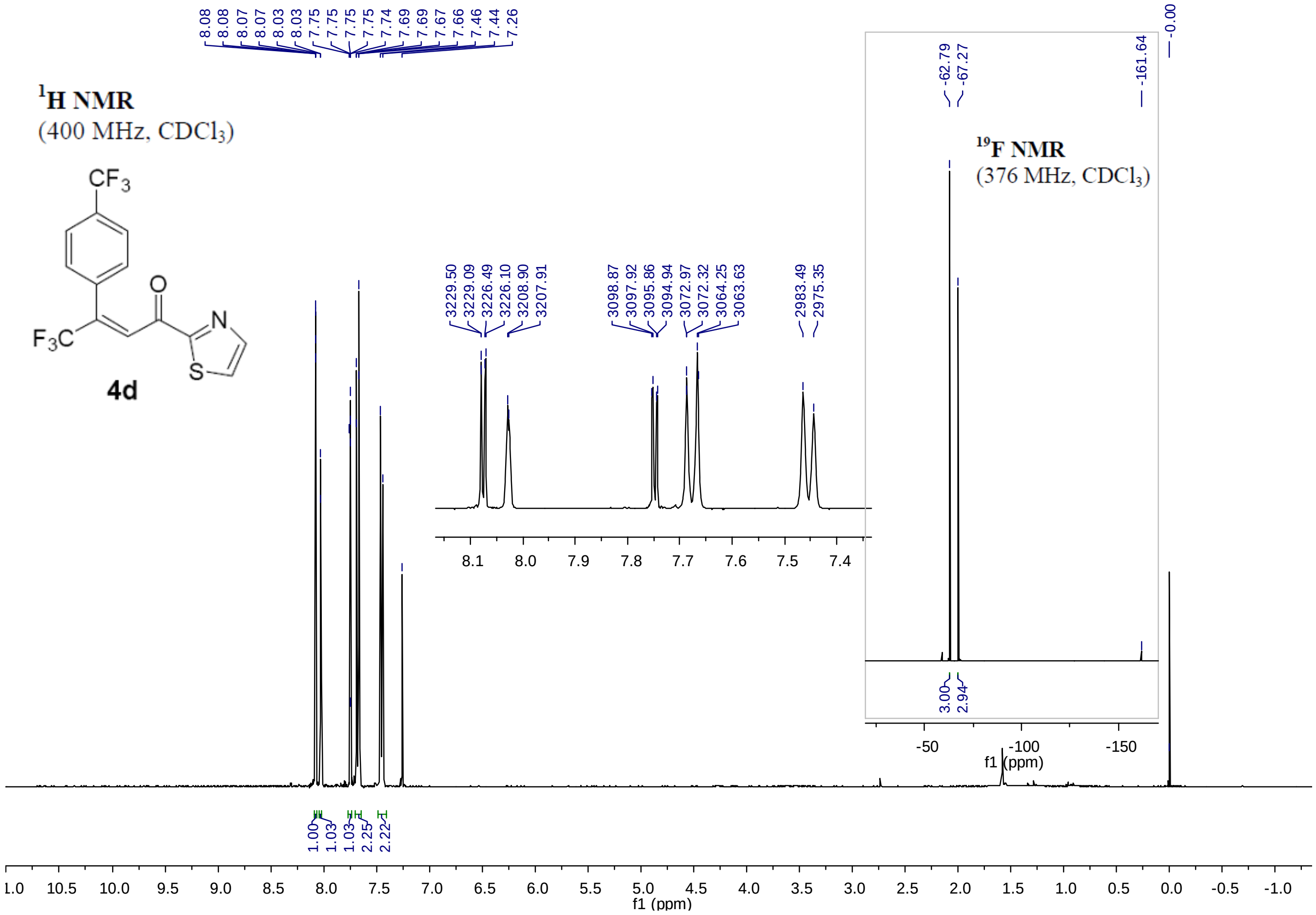

1H NMR
(400 MHz, CDCl3)
4d
19F NMR
(376 MHz, CDCl3)
f1 (ppm)

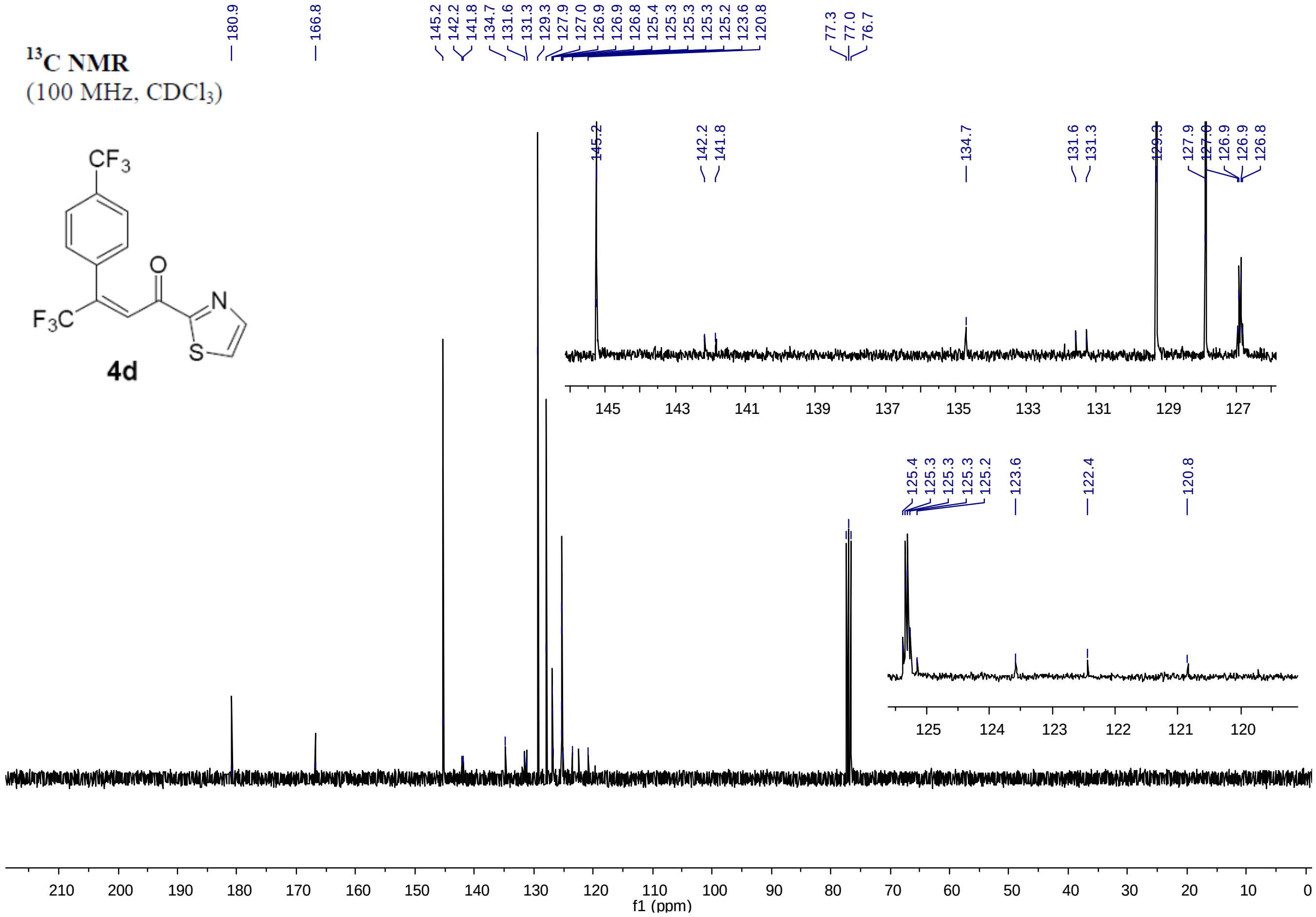

13C NMR
(100 MHz, CDCl3)
4d
f1 (ppm)

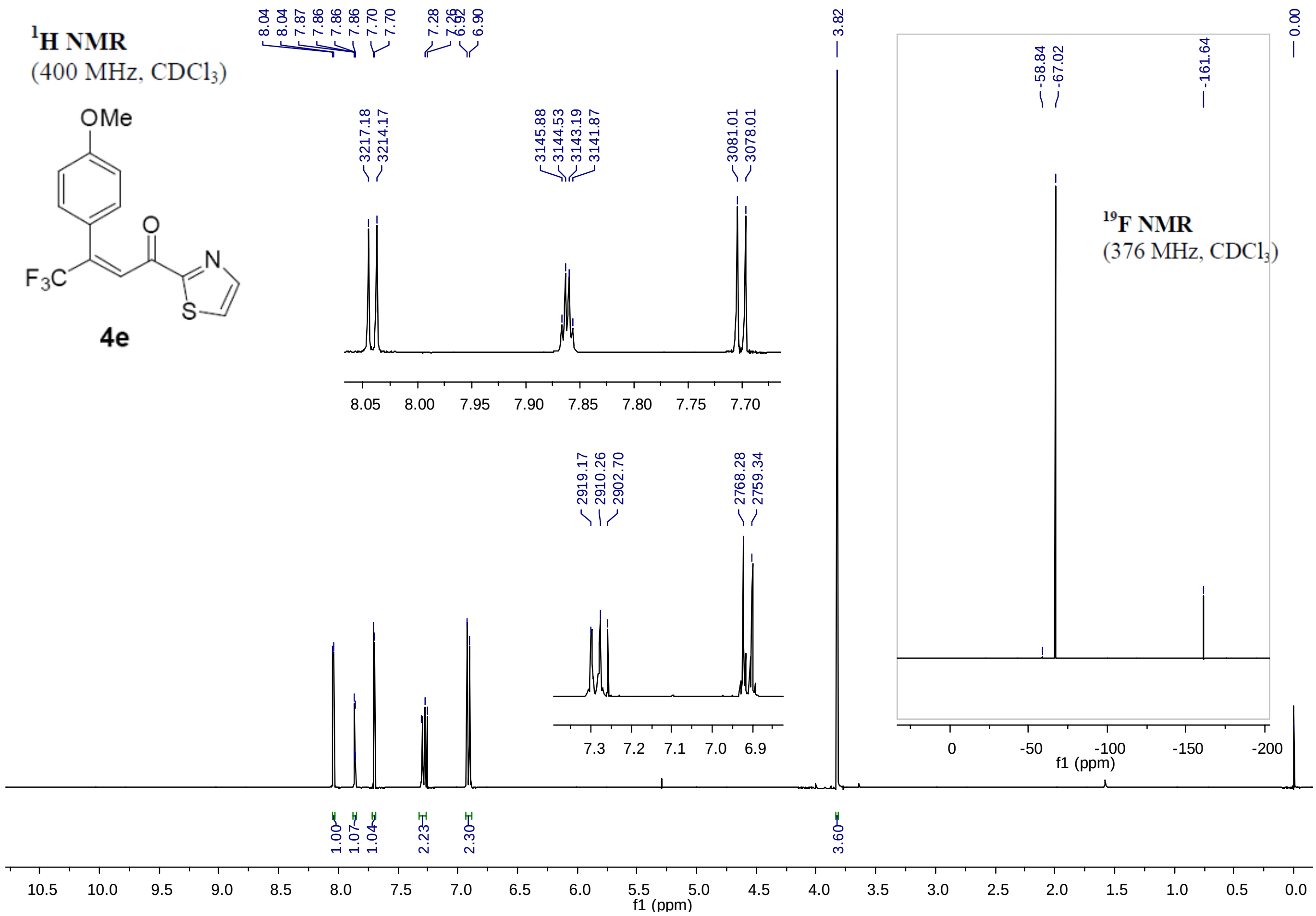
1H NMR
(400 MHz, CDCl3)
OMe
F3C
O
N
S
4e
19F NMR
(376 MHz, CDCl3)
f1 (ppm)

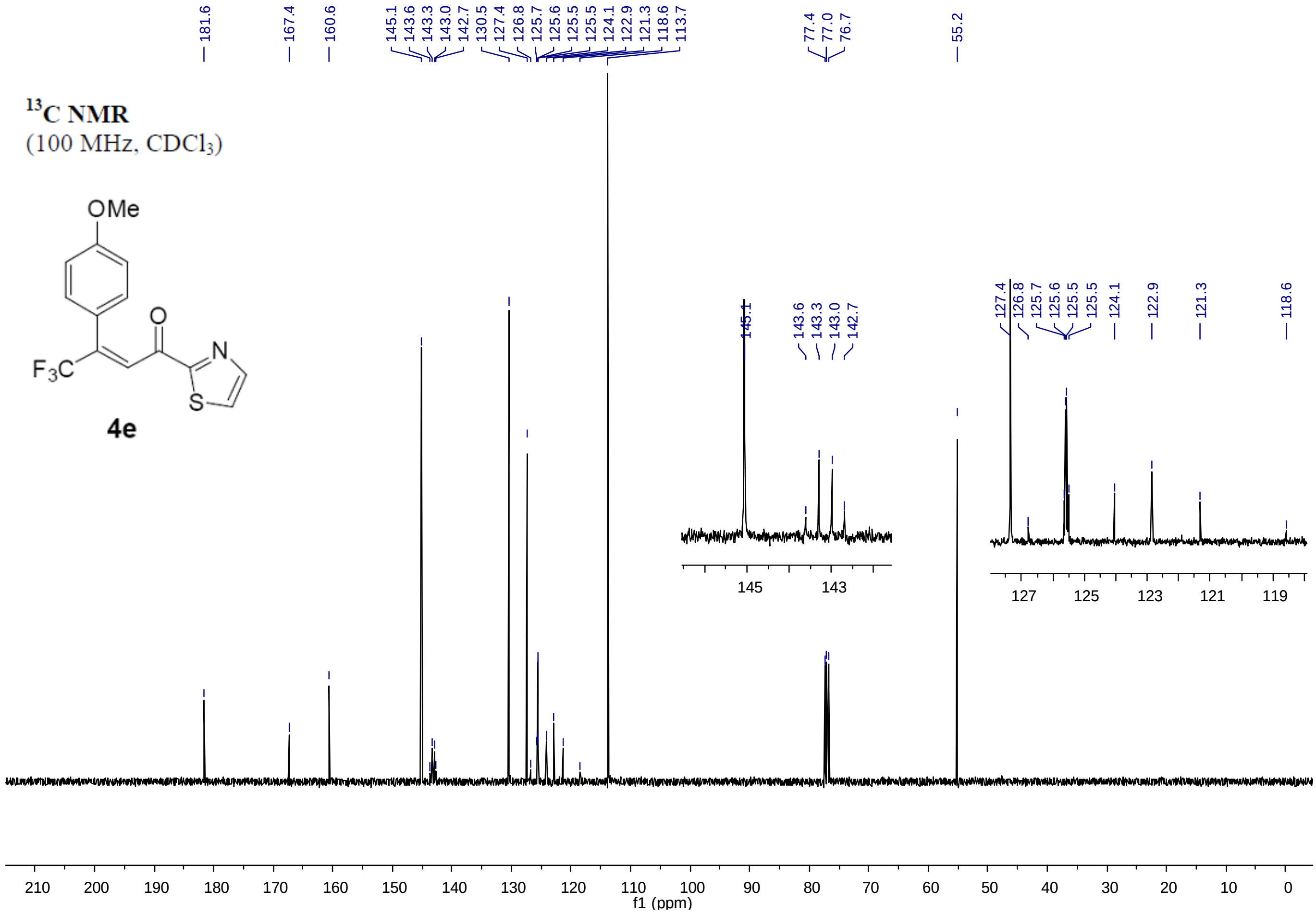
13C NMR
(100 MHz, CDCl3)
OMe
F3C
O
N
S
4e
f1 (ppm)

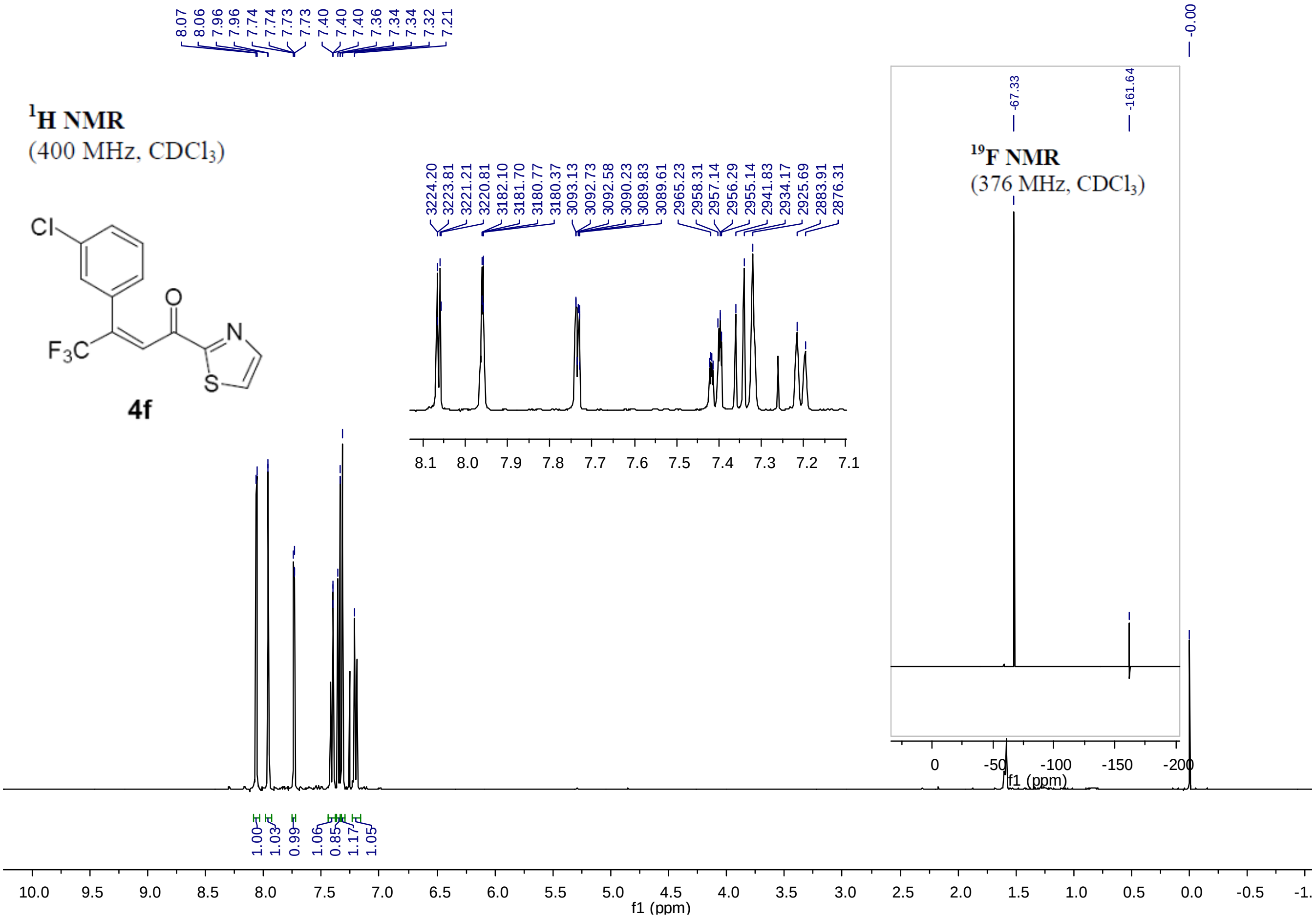

1H NMR
(400 MHz, CDCl3)
4f
19F NMR
(376 MHz, CDCl3)
-67.33
-161.64
f1 (ppm)

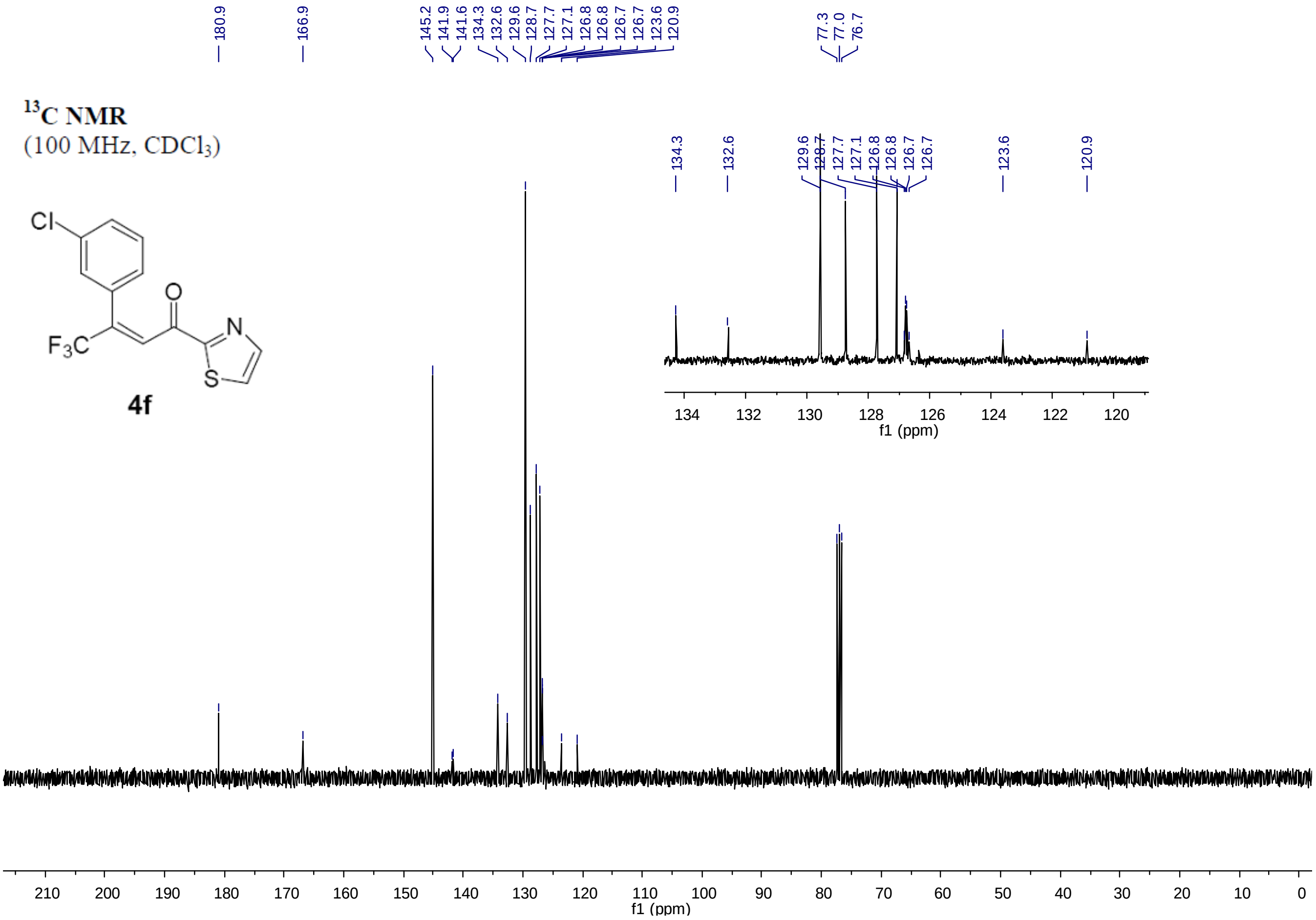

13C NMR
(100 MHz, CDCl3)
4f
f1 (ppm)

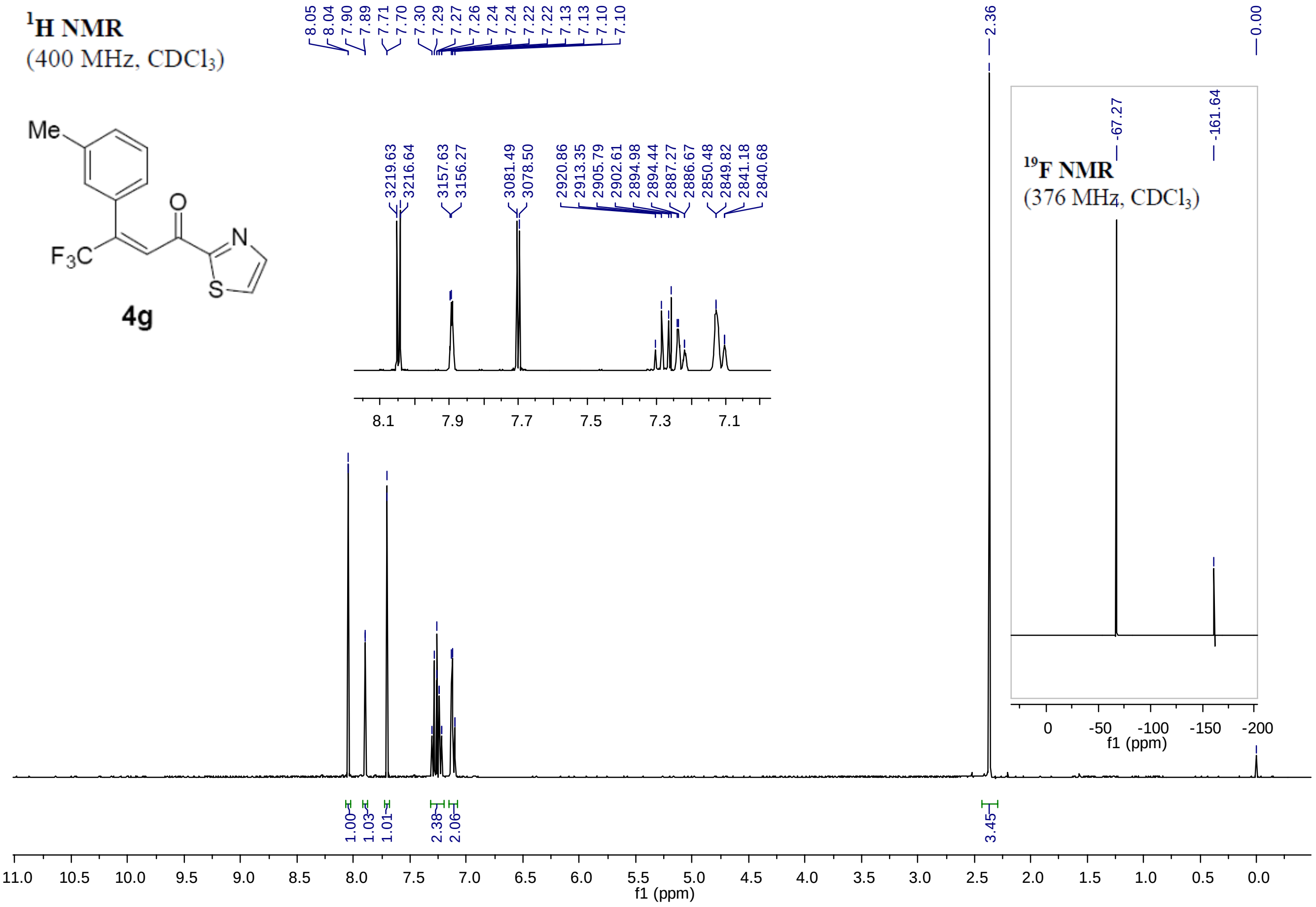

1H NMR
(400 MHz, CDCl3)
Me
F3C
O
N
S
4g
19F NMR
(376 MHz, CDCl3)
f1 (ppm)

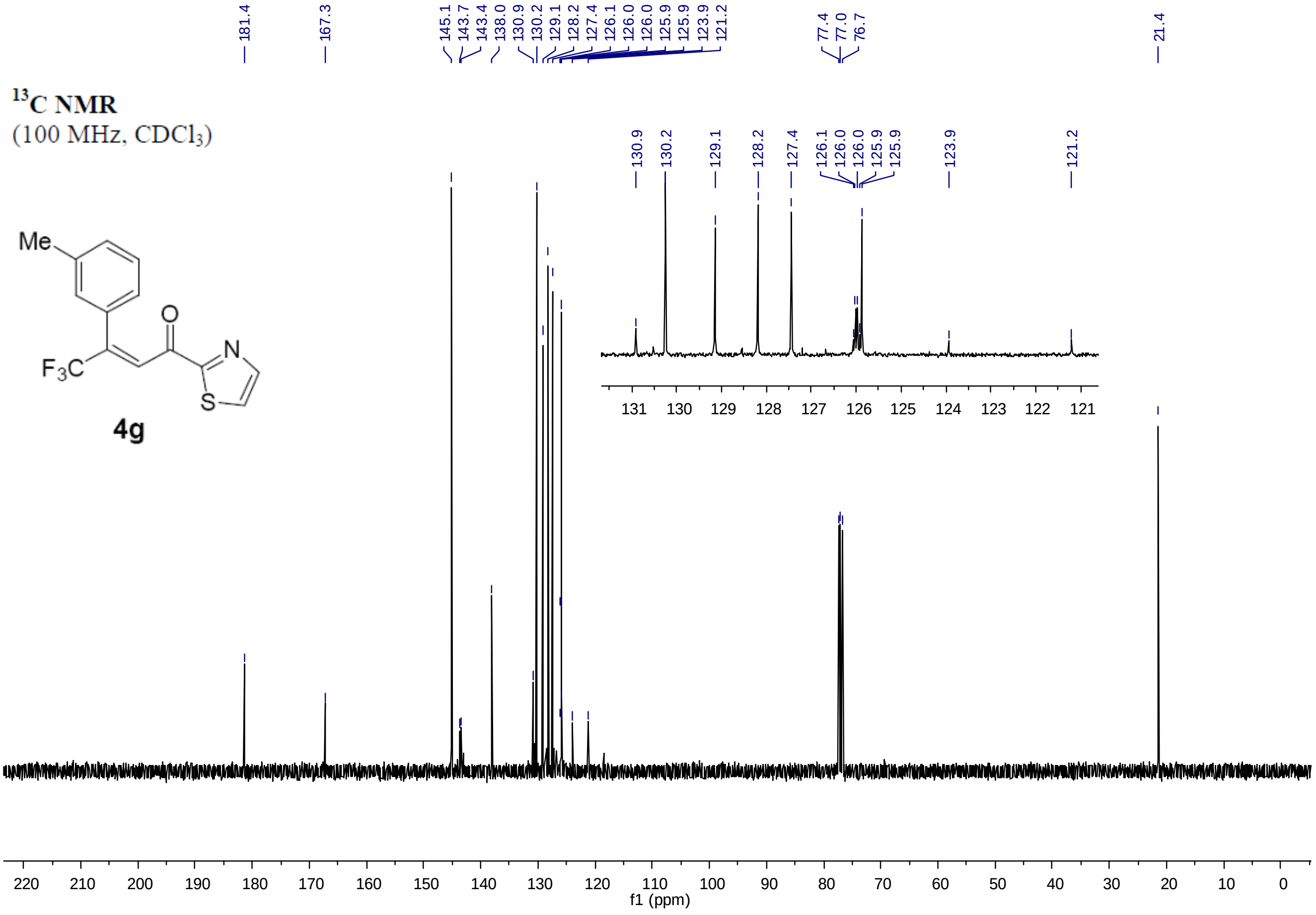

13C NMR
(100 MHz, CDCl3)
Me
F3C
O
N
S
4g
f1 (ppm)

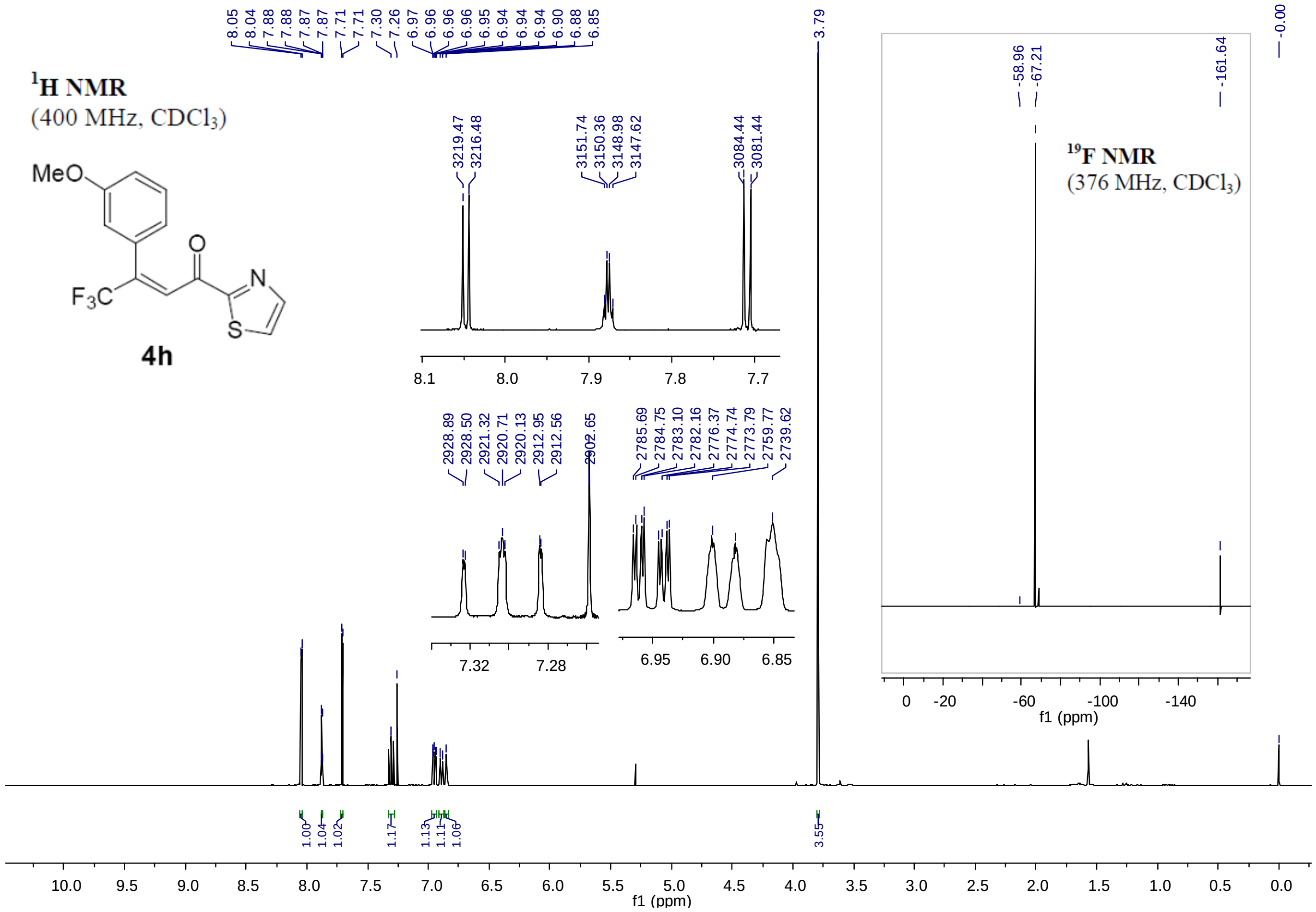

1H NMR
(400 MHz, CDCl3)
MeO
F3C
O
N
S
4h
19F NMR
(376 MHz, CDCl3)
f1 (ppm)

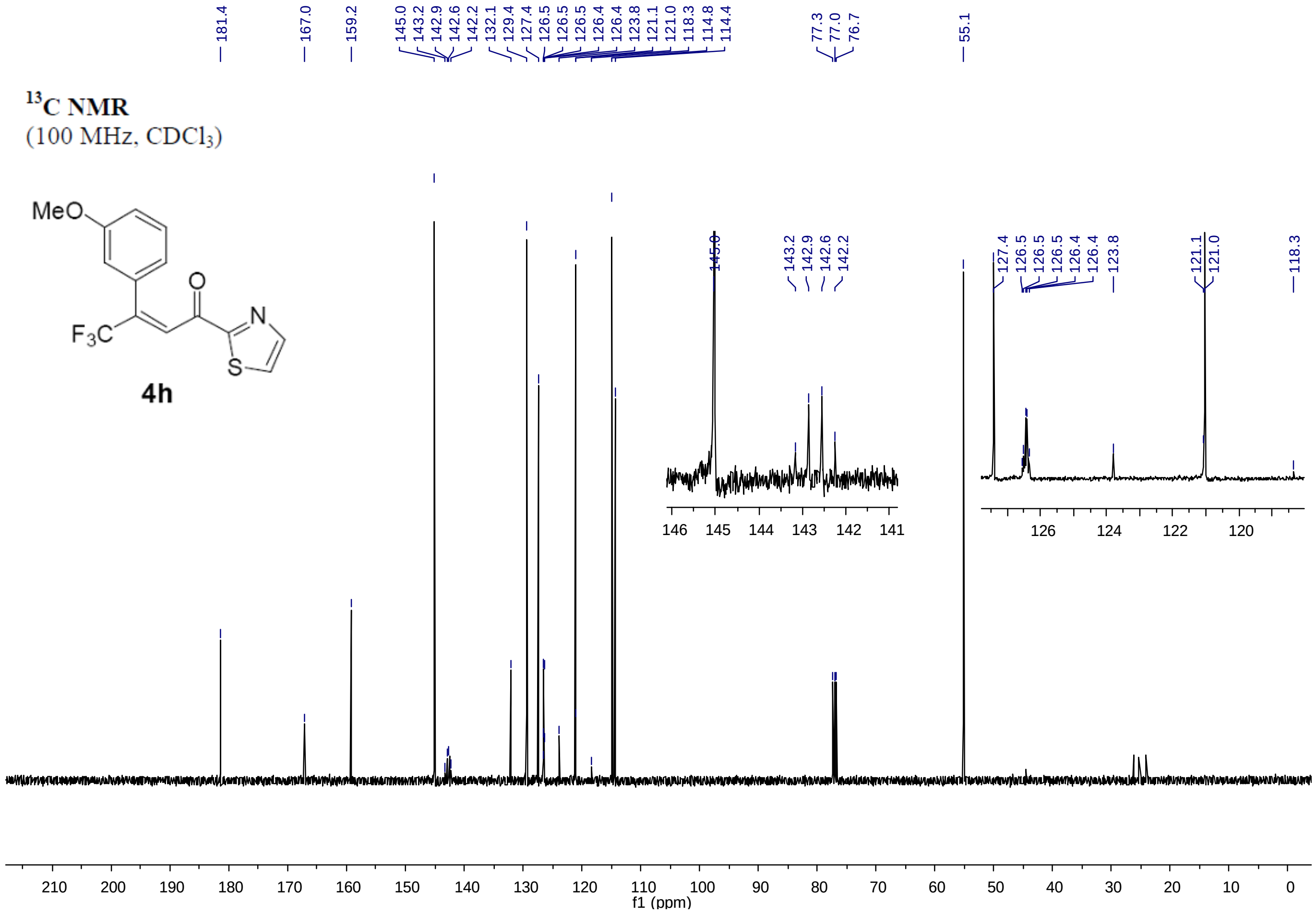

13C NMR
(100 MHz, CDCl3)
MeO
F3C
O
N
S
4h
f1 (ppm)

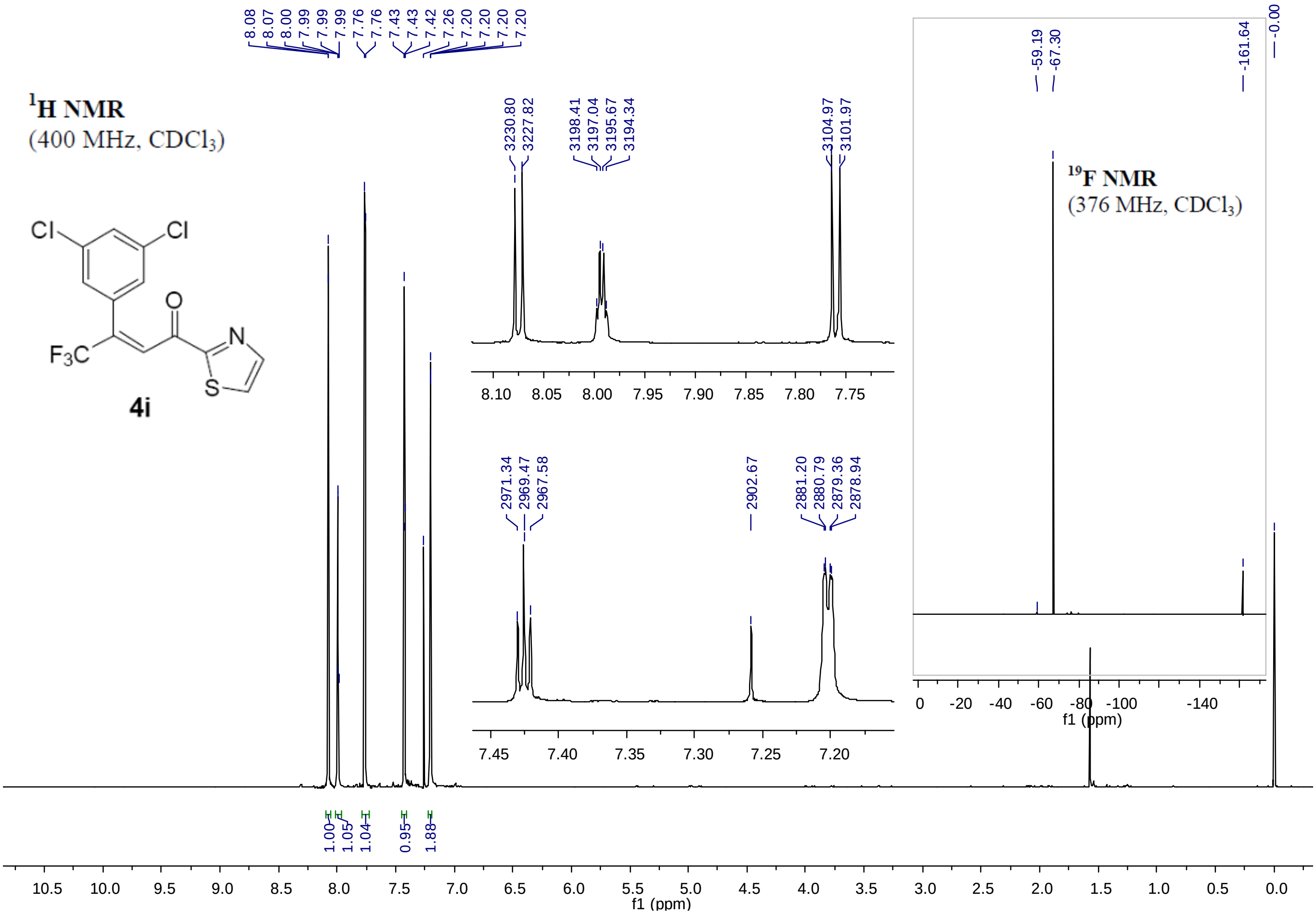
^{1}H NMR
(400 MHz, $CDCl_3$)
4i
^{19}F NMR
(376 MHz, $CDCl_3$)
f1 (ppm)

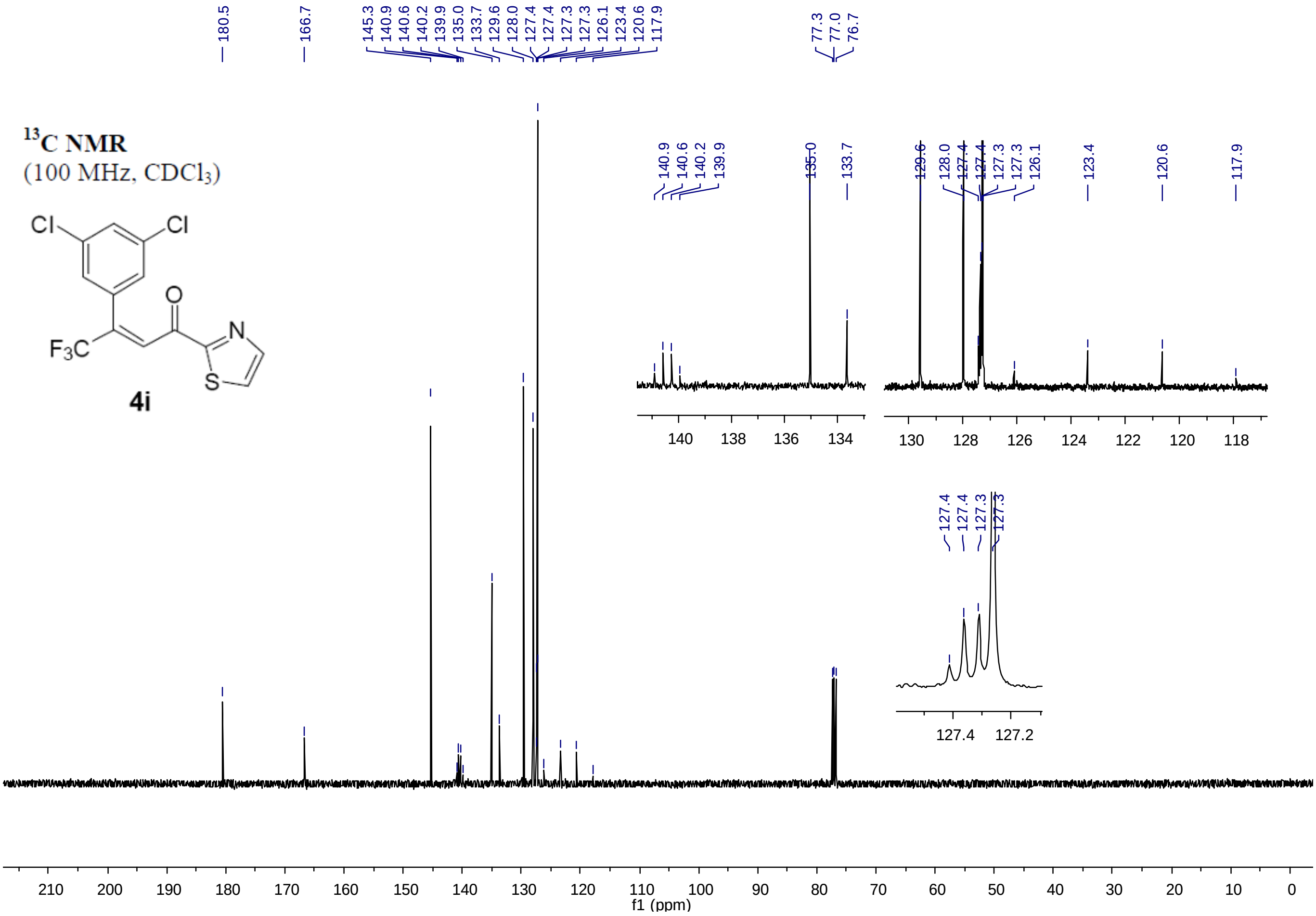
^{13}C NMR
(100 MHz, $CDCl_3$)
4i
f1 (ppm)

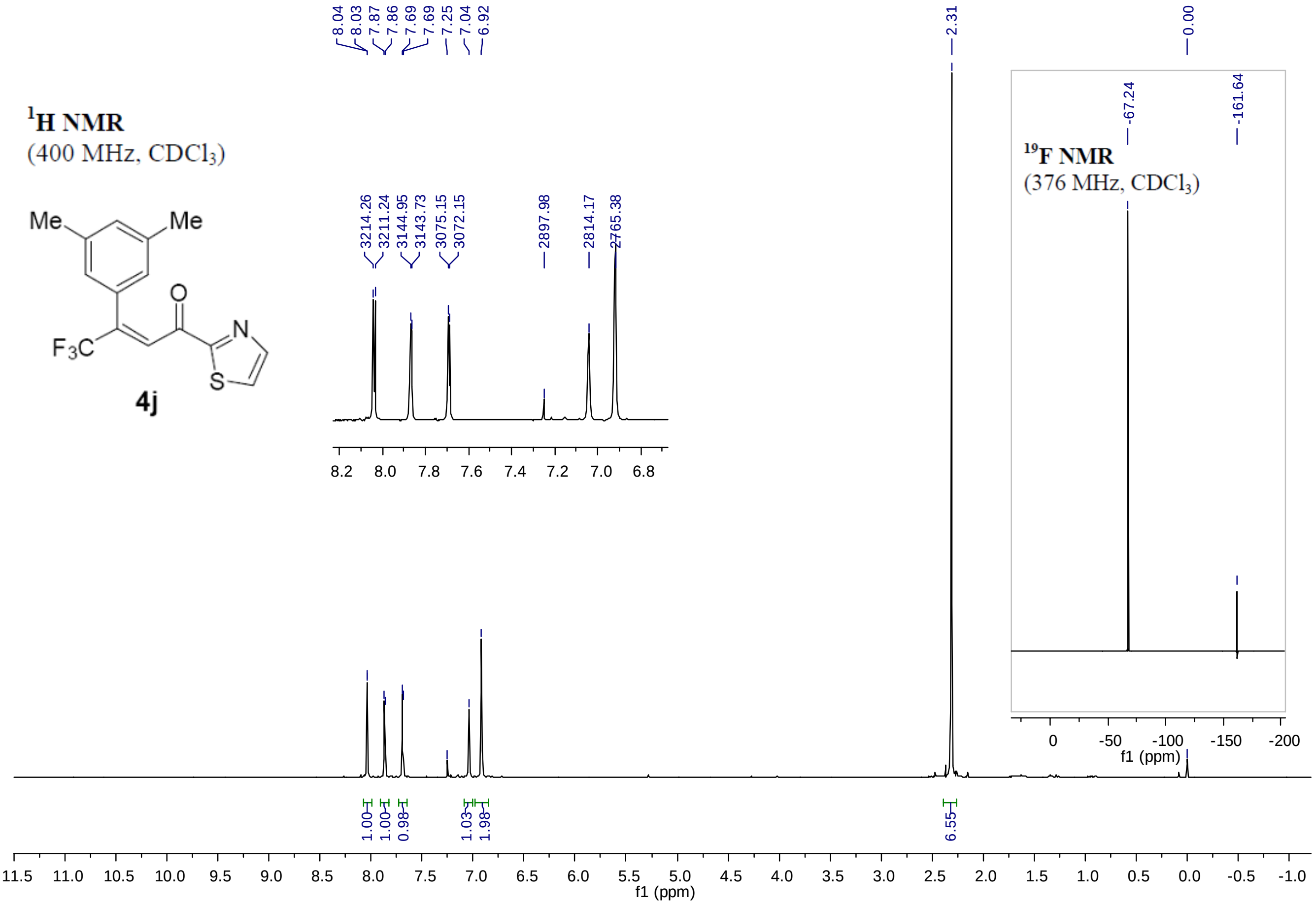
1H NMR
(400 MHz, CDCl3)
Me
Me
O
F3C
N
S
4j
8.04
8.03
7.87
7.86
7.69
7.69
7.25
7.04
6.92
2.31
0.00
3214.26
3211.24
3144.95
3143.73
3075.15
3072.15
2897.98
2814.17
2765.38
19F NMR
(376 MHz, CDCl3)
-67.24
-161.64
1.00
1.00
0.98
1.03
1.98
6.55
f1 (ppm)

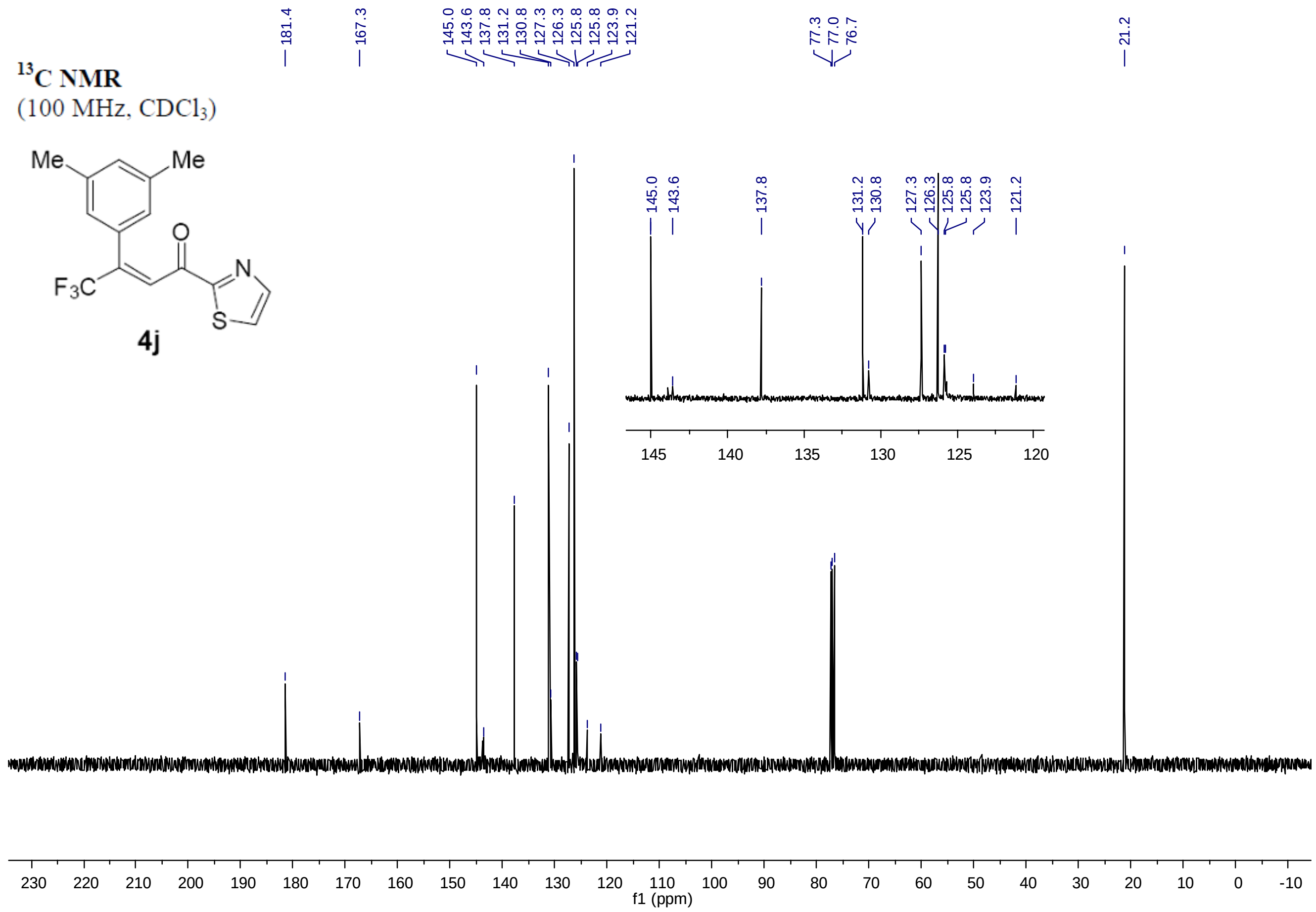
13C NMR
(100 MHz, CDCl3)
Me
Me
O
F3C
N
S
4j
181.4
167.3
145.0
143.6
137.8
131.2
130.8
127.3
126.3
125.8
125.8
123.9
121.2
77.3
77.0
76.7
21.2
f1 (ppm)

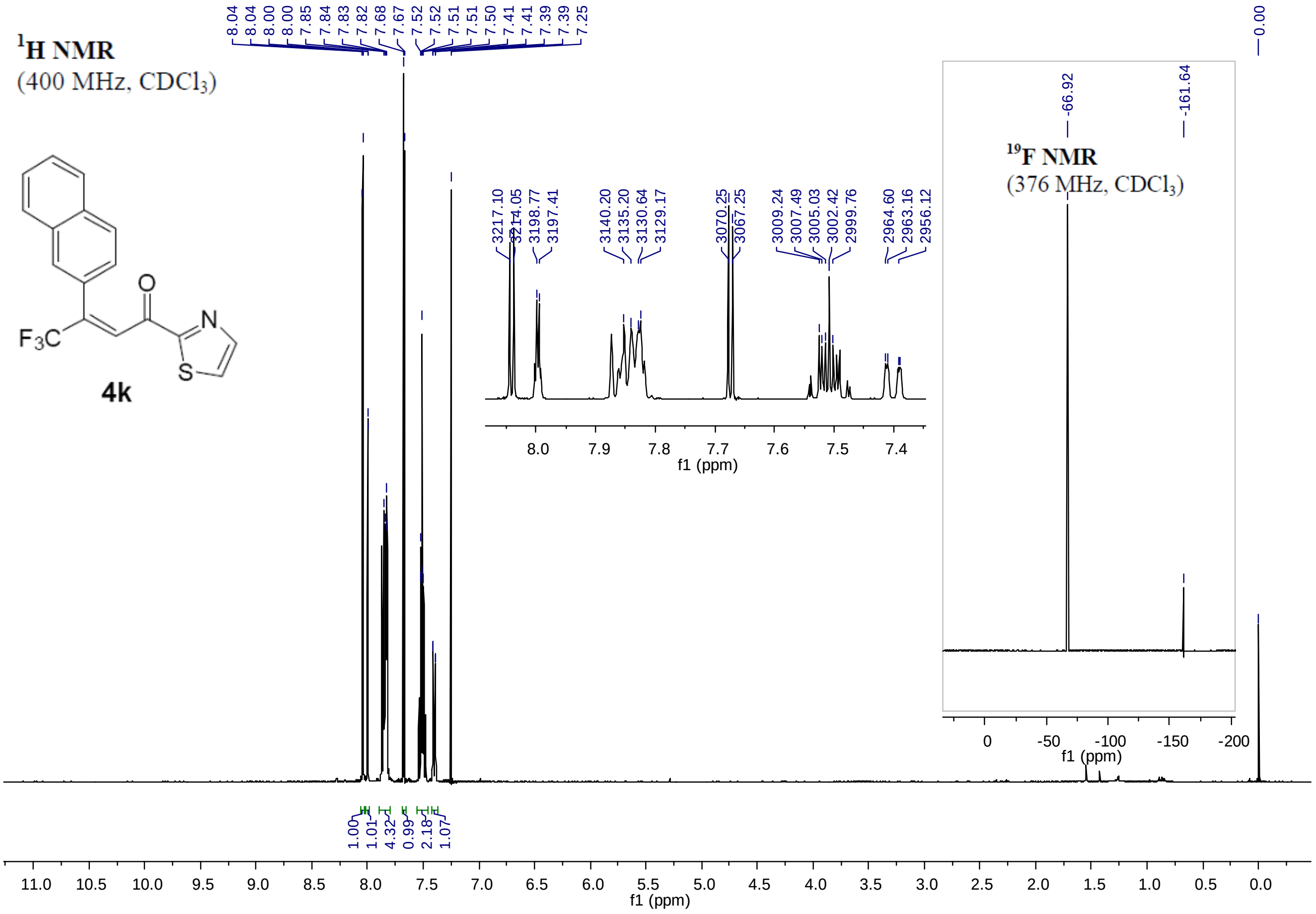
1H NMR
(400 MHz, CDCl3)
F3C
O
N
S
4k
19F NMR
(376 MHz, CDCl3)
-66.92
-161.64
f1 (ppm)
1.00
1.01
4.32
0.99
2.18
1.07

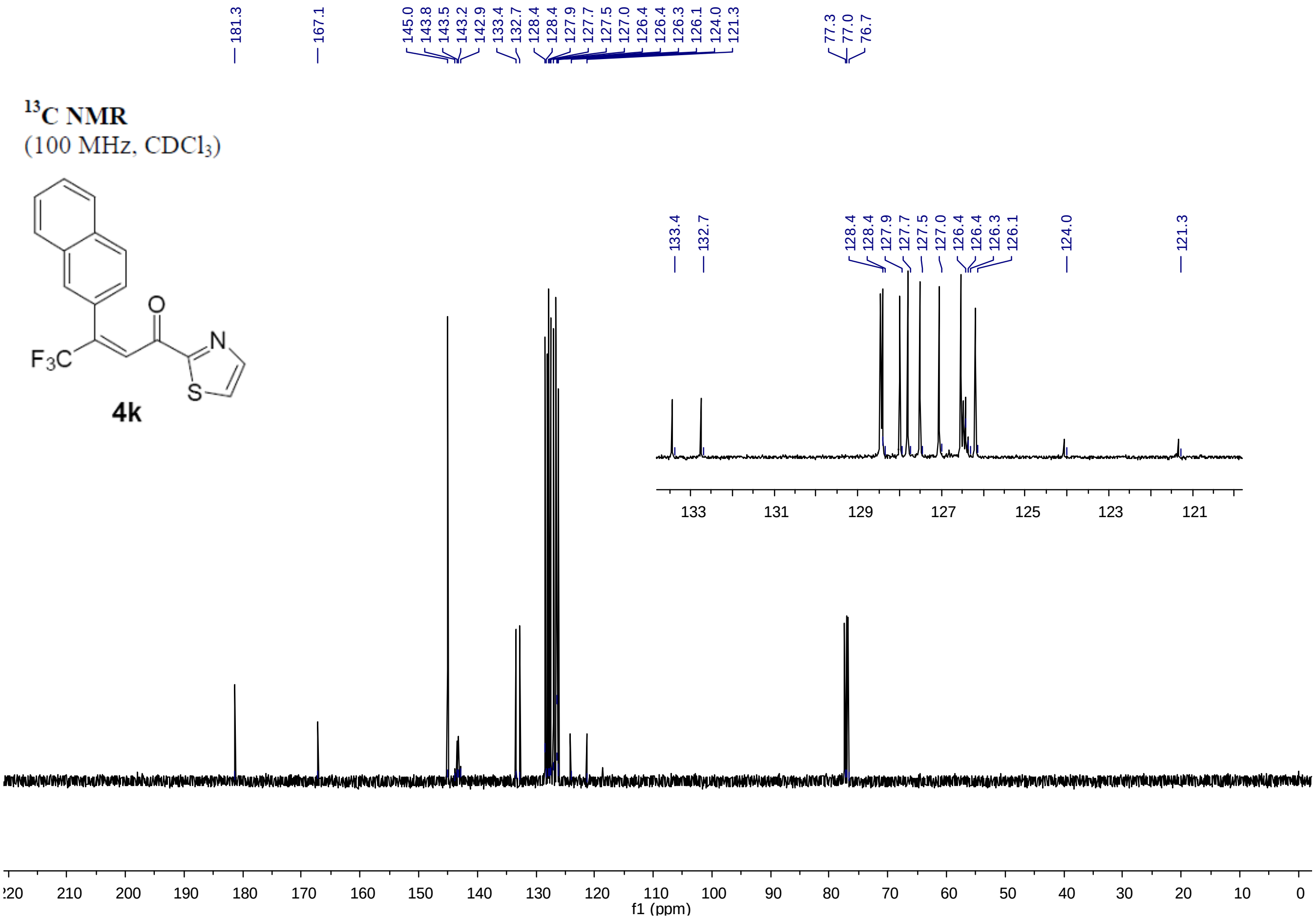
13C NMR
(100 MHz, CDCl3)
F3C
O
N
S
4k
181.3
167.1
145.0
143.8
143.5
143.2
142.9
133.4
132.7
128.4
128.4
127.9
127.7
127.5
127.0
126.4
126.4
126.3
126.1
124.0
121.3
77.3
77.0
76.7
f1 (ppm)

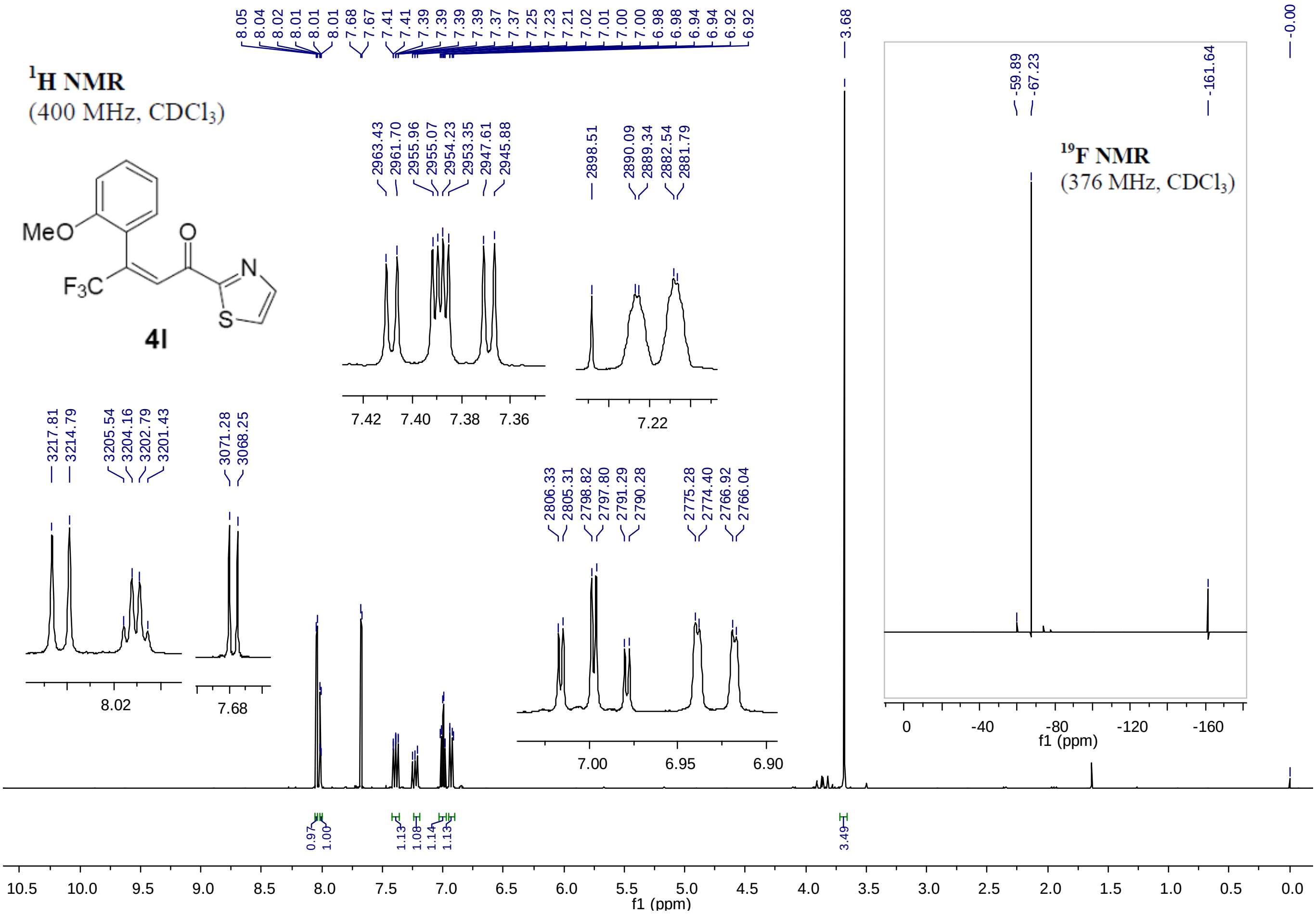

1H NMR
(400 MHz, CDCl3)
MeO
F3C
O
N
S
4l
19F NMR
(376 MHz, CDCl3)
f1 (ppm)

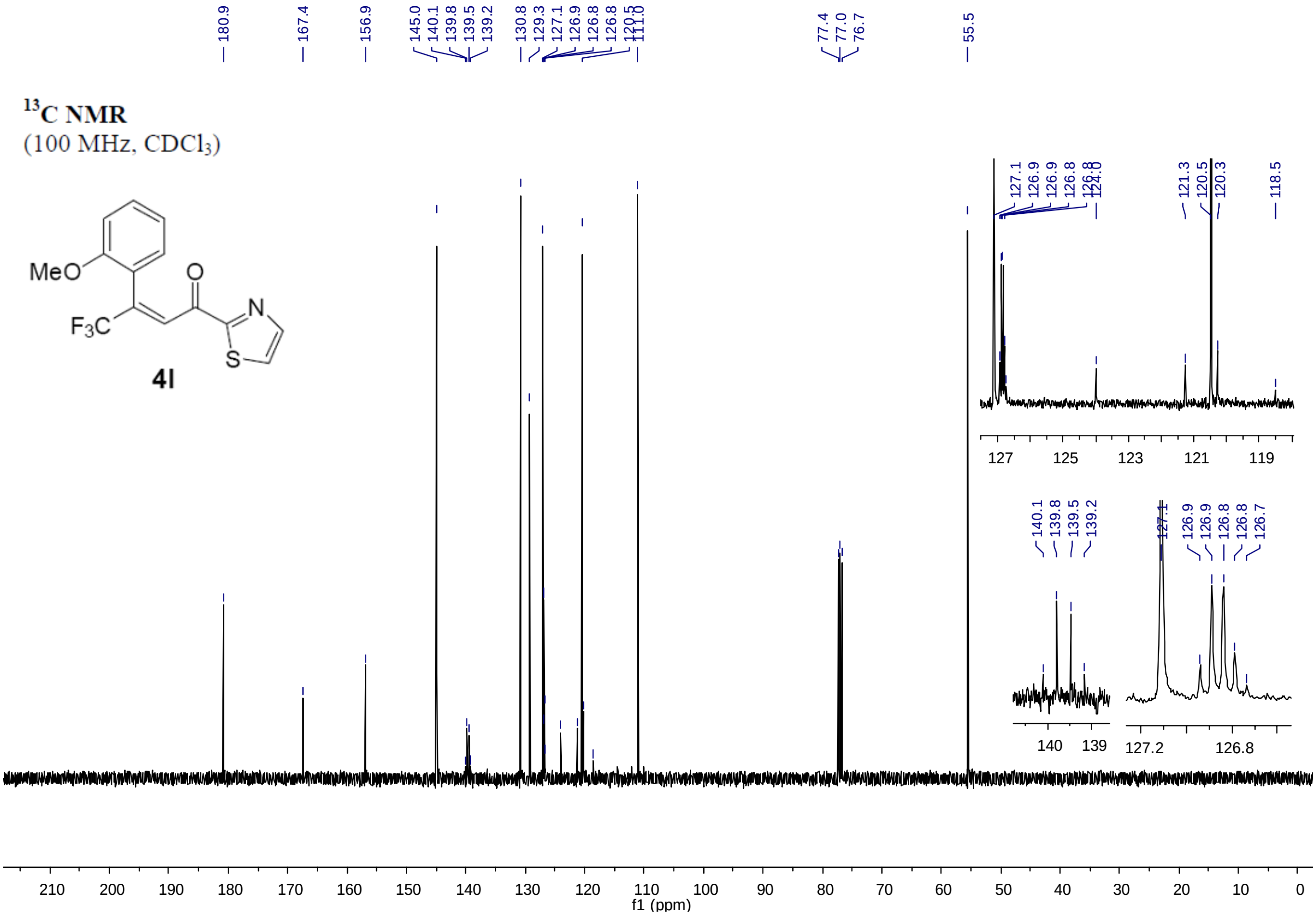

13C NMR
(100 MHz, CDCl3)
MeO
F3C
O
N
S
4l
f1 (ppm)

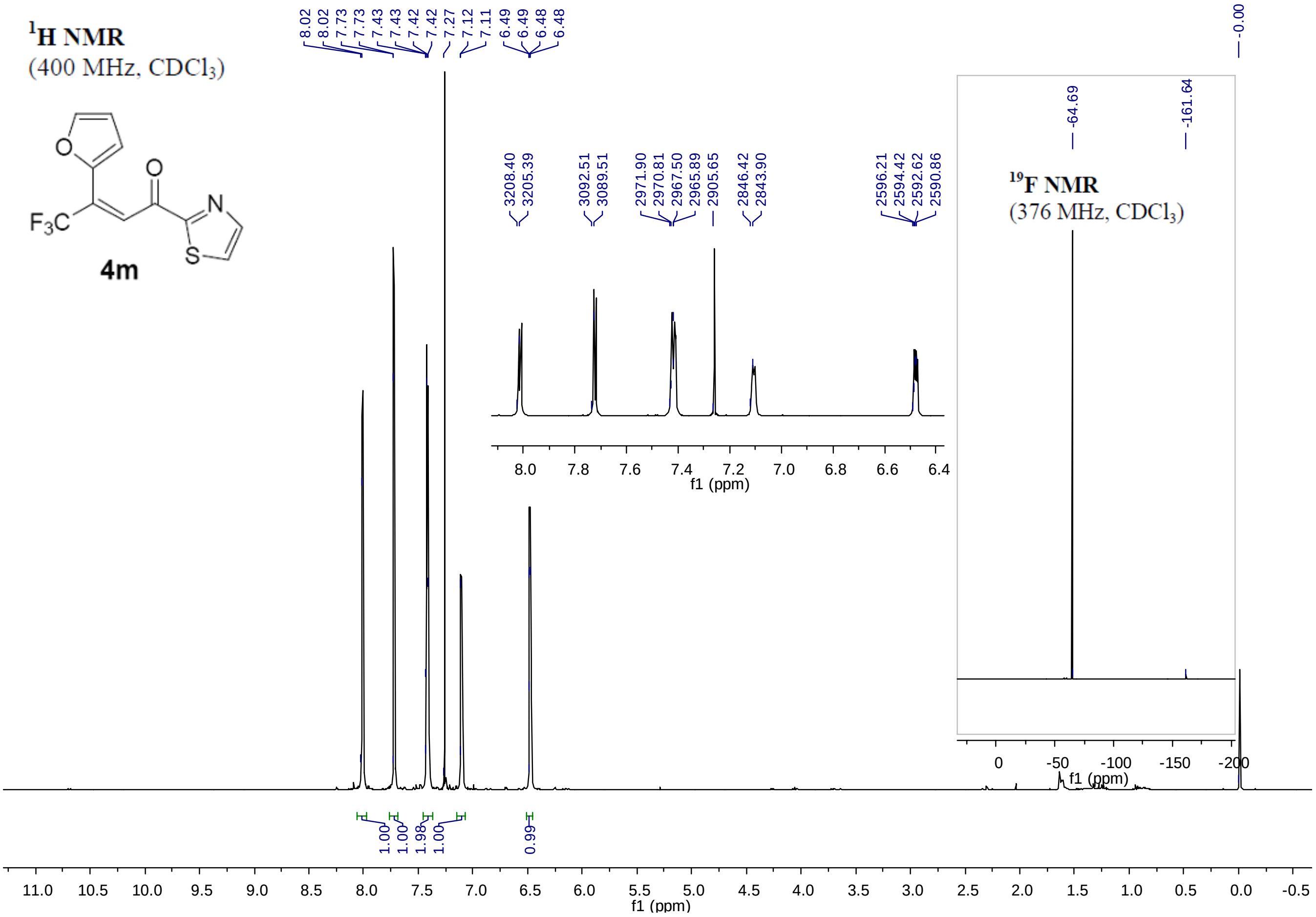

1H NMR
(400 MHz, CDCl3)
4m
19F NMR
(376 MHz, CDCl3)
f1 (ppm)

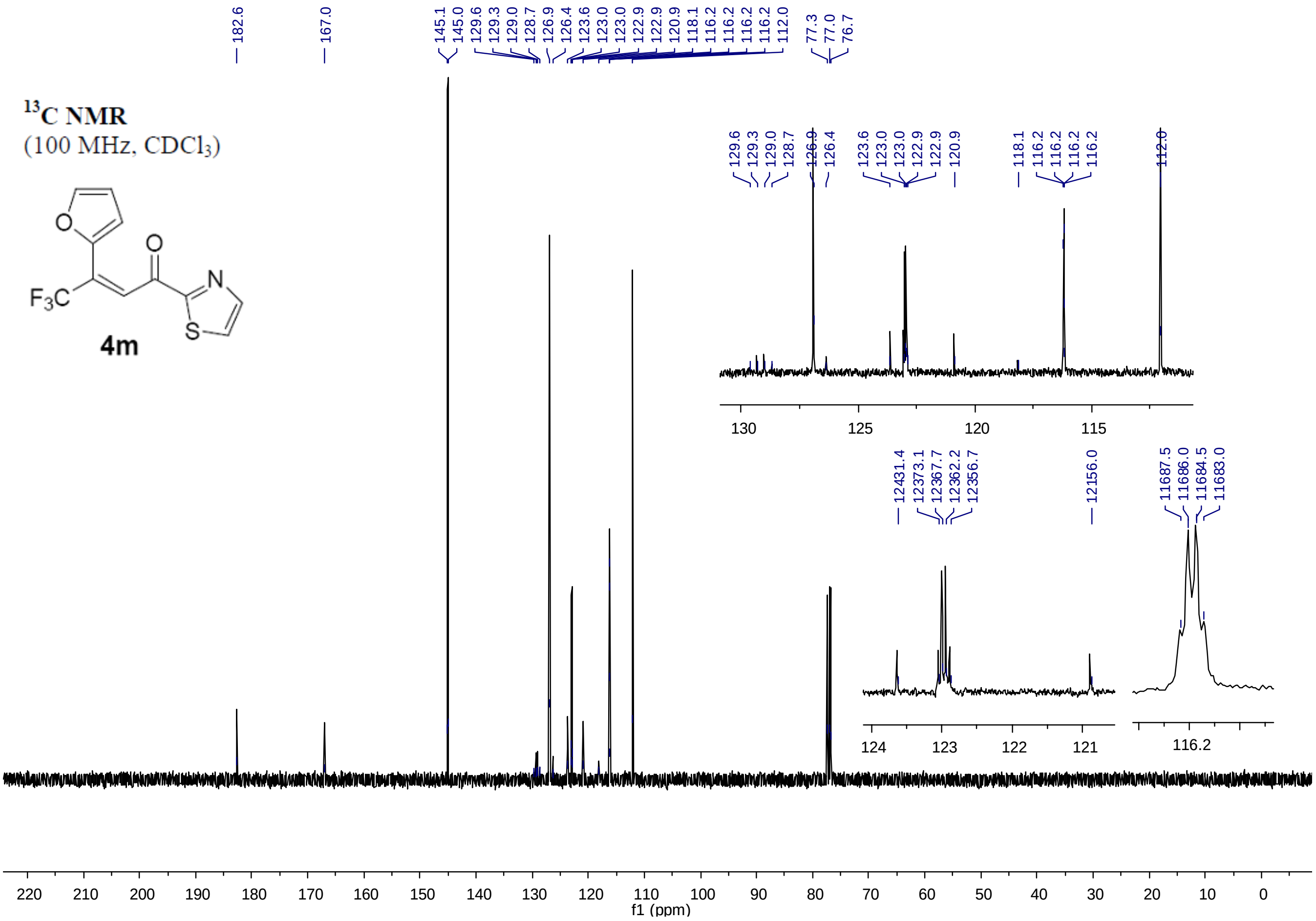

13C NMR
(100 MHz, CDCl3)
4m
f1 (ppm)

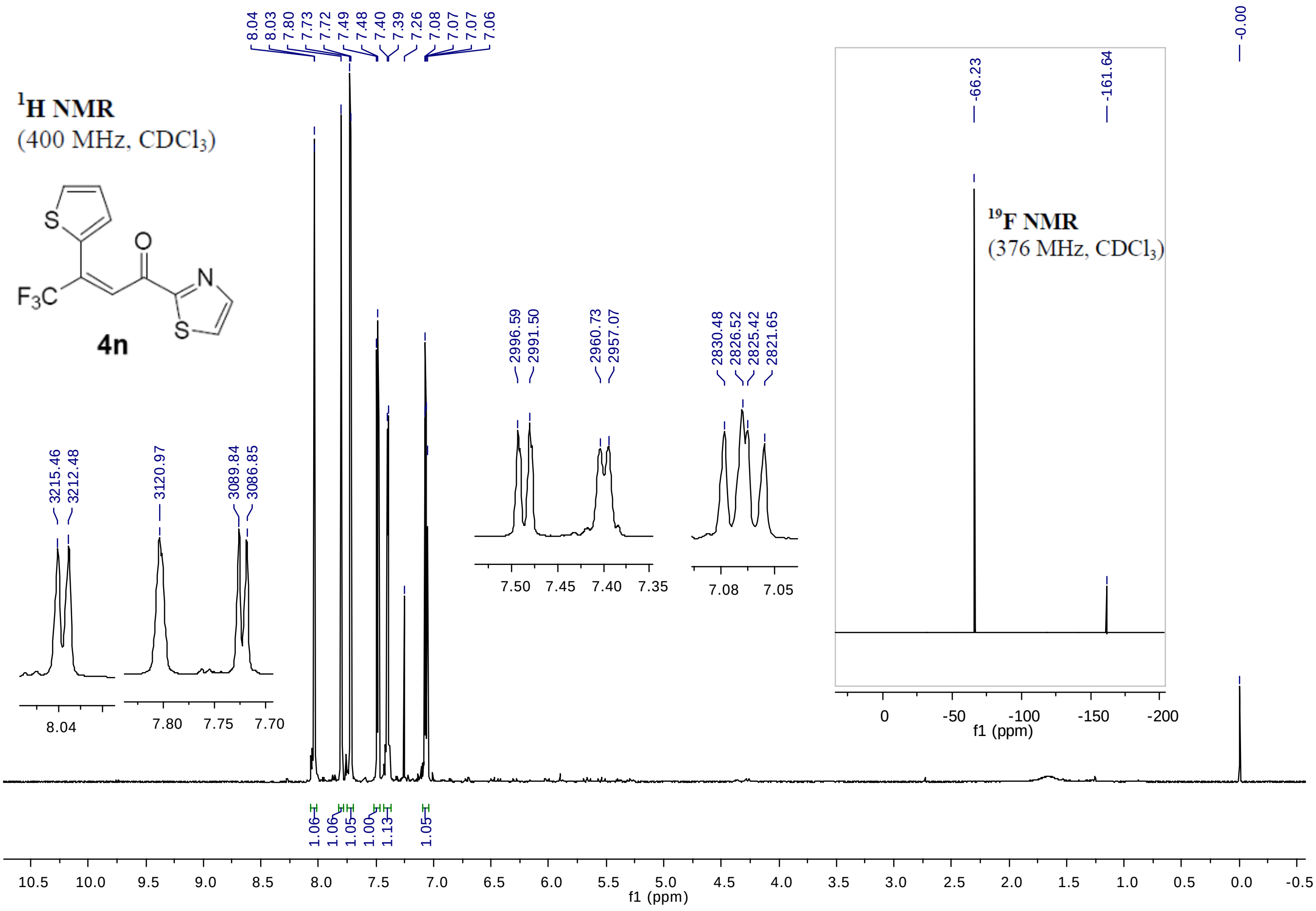
1H NMR
(400 MHz, CDCl3)
F3C
4n
8.04 8.03 7.80 7.73 7.72 7.49 7.48 7.40 7.39 7.26 7.08 7.07 7.07 7.06
3215.46 3212.48
3120.97
3089.84 3086.85
2996.59 2991.50
2960.73 2957.07
2830.48 2826.52 2825.42 2821.65
1.06 1.06 1.05 1.00 1.13 1.05
-0.00
19F NMR
(376 MHz, CDCl3)
-66.23
-161.64
f1 (ppm)

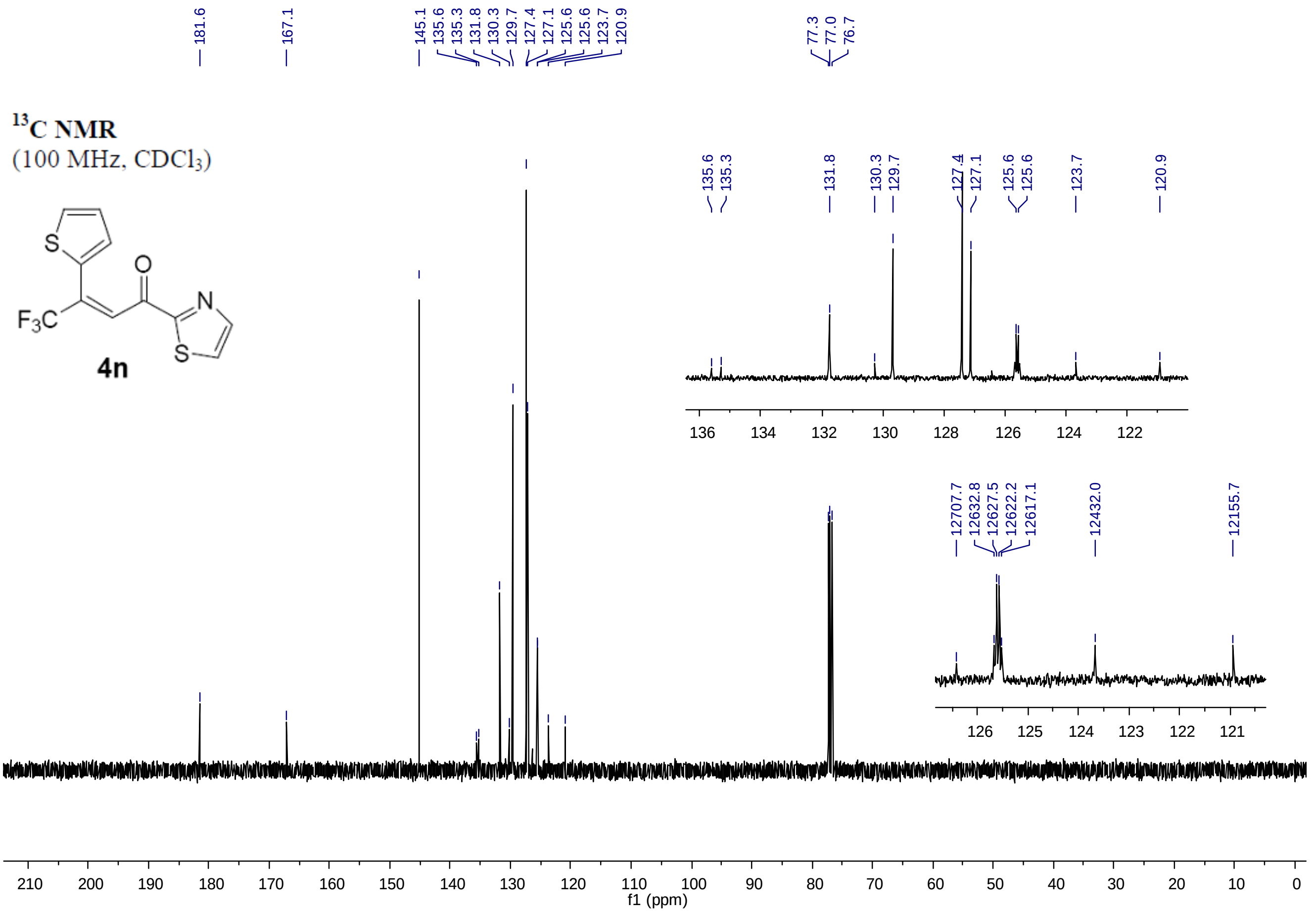
13C NMR
(100 MHz, CDCl3)
F3C
4n
181.6
167.1
145.1 135.6 135.3 131.8 130.3 129.7 127.4 127.1 125.6 125.6 123.7 120.9
77.3 77.0 76.7
12707.7 12632.8 12627.5 12622.2 12617.1
12432.0
12155.7
f1 (ppm)

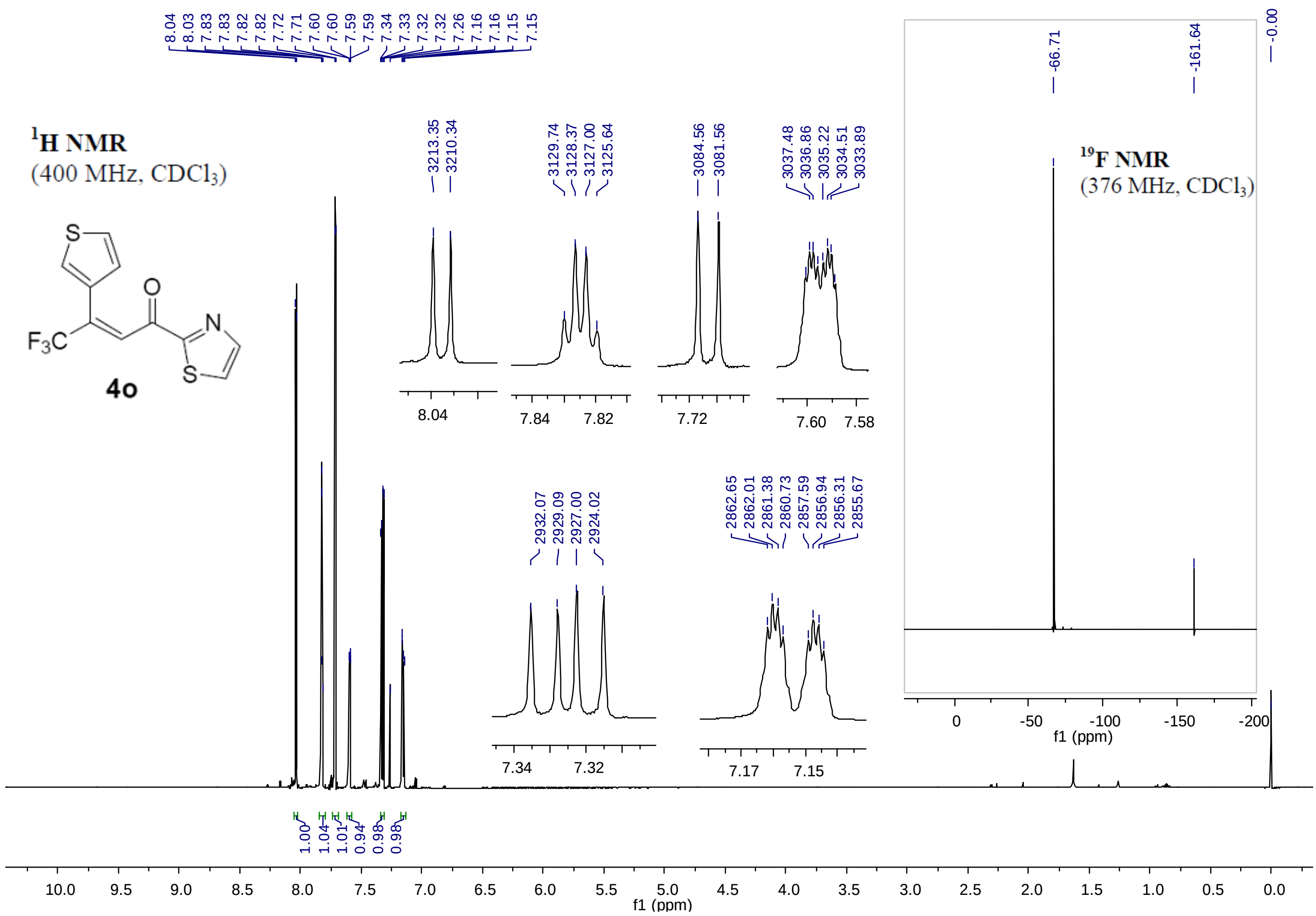

8.04 8.03 7.83 7.83 7.82 7.82 7.72 7.71 7.60 7.60 7.59 7.59 7.34 7.33 7.32 7.32 7.26 7.16 7.16 7.15 7.15
1H NMR
(400 MHz, CDCl3)
F3C
4o
-66.71
-161.64
-0.00
19F NMR
(376 MHz, CDCl3)
1.00 1.04 1.01 0.94 0.98 0.98
f1 (ppm)

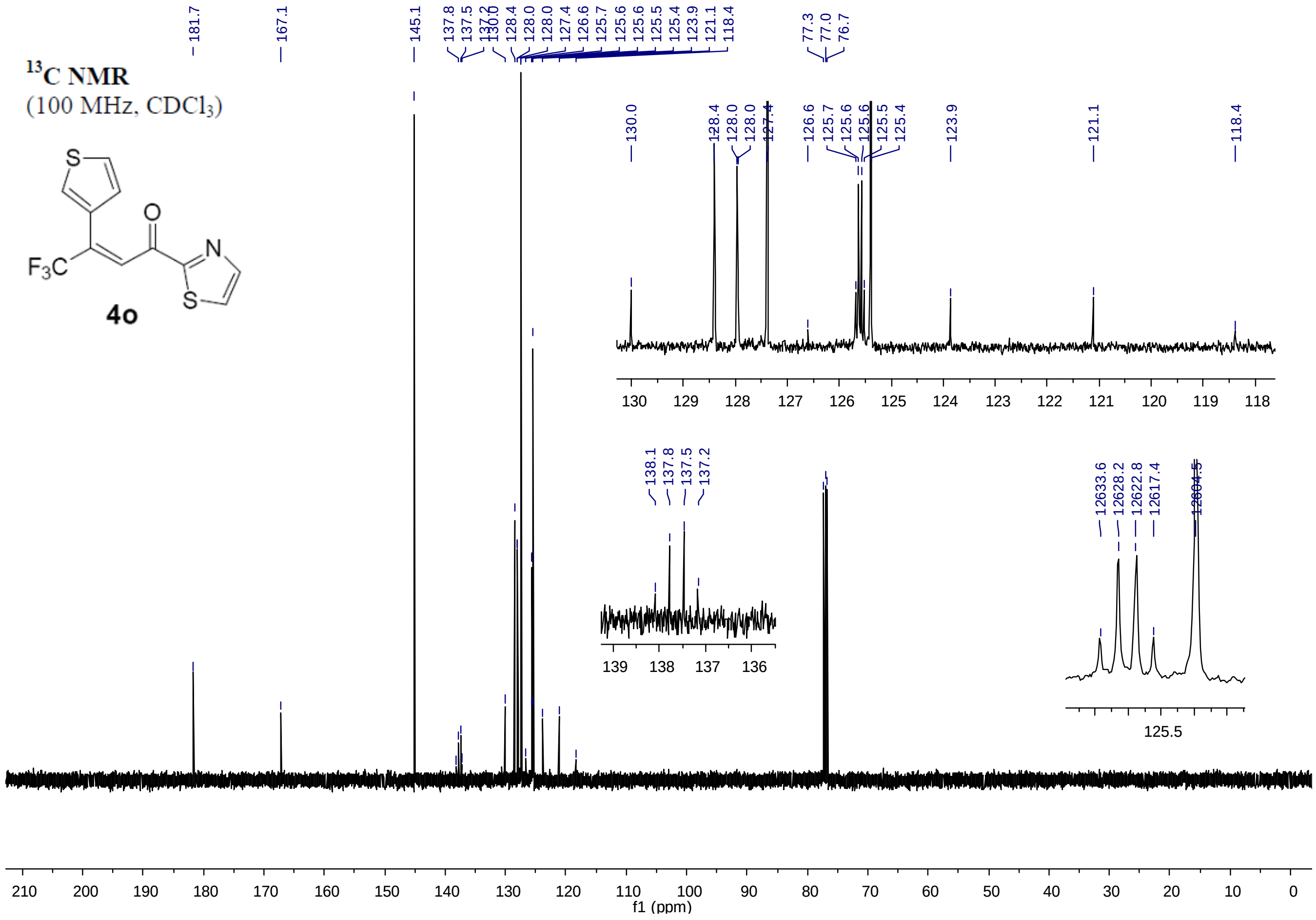

181.7
167.1
145.1
13C NMR
(100 MHz, CDCl3)
F3C
4o
77.3 77.0 76.7
f1 (ppm)

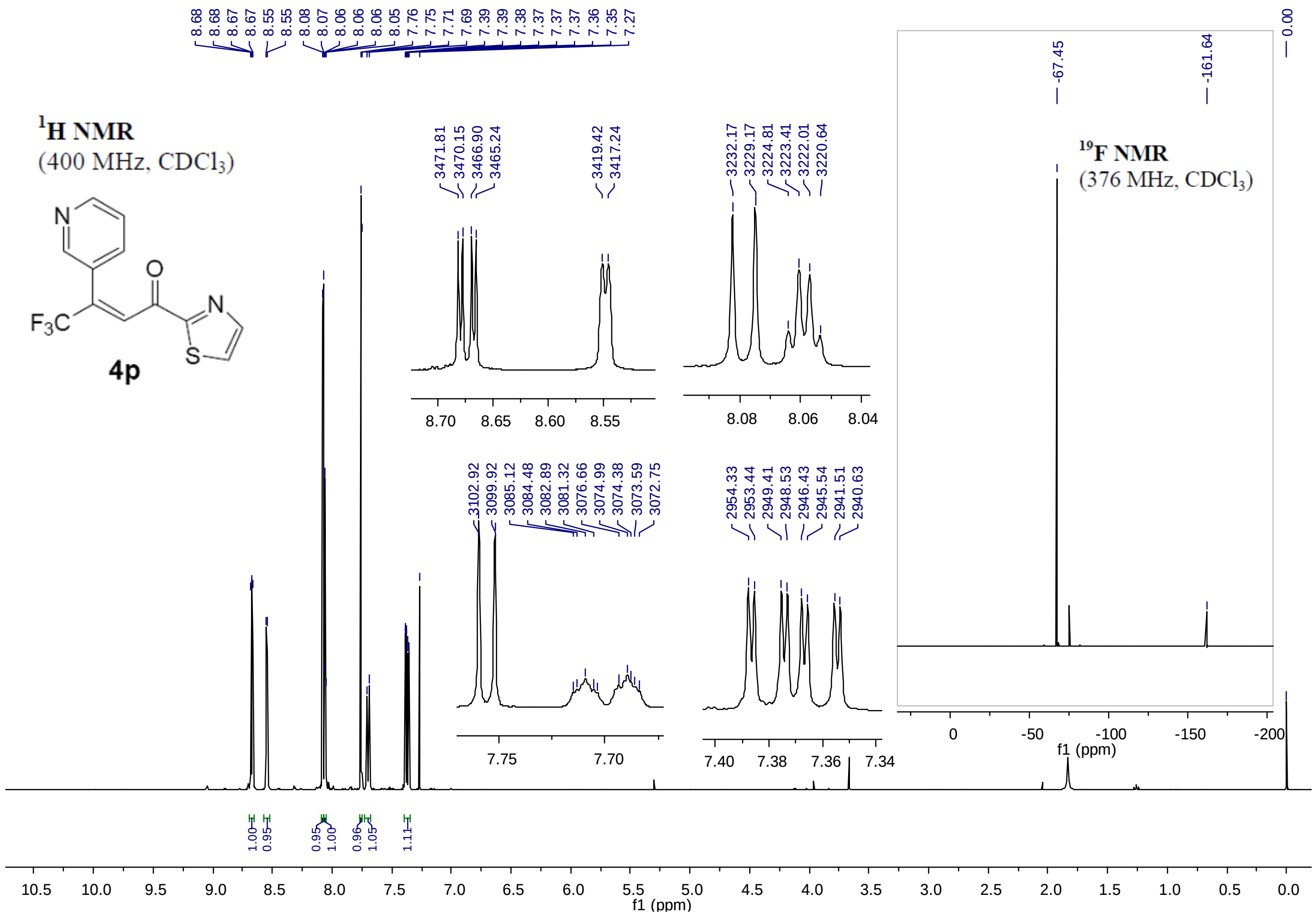

8.68 8.68 8.67 8.67 8.55 8.55 8.08 8.07 8.06 8.06 8.06 8.05 7.76 7.75 7.71 7.69 7.39 7.39 7.38 7.37 7.37 7.37 7.36 7.35 7.27
^{1}H NMR
(400 MHz, CDCl$_3$)
F_3C
4p
3471.81 3470.15 3466.90 3465.24 3419.42 3417.24
3232.17 3229.17 3224.81 3223.41 3222.01 3220.64
3102.92 3099.92 3085.12 3084.48 3082.89 3081.32 3076.66 3074.99 3074.38 3073.59 3072.75
2954.33 2953.44 2949.41 2948.53 2946.43 2945.54 2941.51 2940.63
-67.45
-161.64
^{19}F NMR
(376 MHz, CDCl$_3$)
0 -50 -100 -150 -200
f1 (ppm)
0.00
1.00 0.95 0.95 1.00 0.96 1.05 1.11
10.5 10.0 9.5 9.0 8.5 8.0 7.5 7.0 6.5 6.0 5.5 5.0 4.5 4.0 3.5 3.0 2.5 2.0 1.5 1.0 0.5 0.0
f1 (ppm)

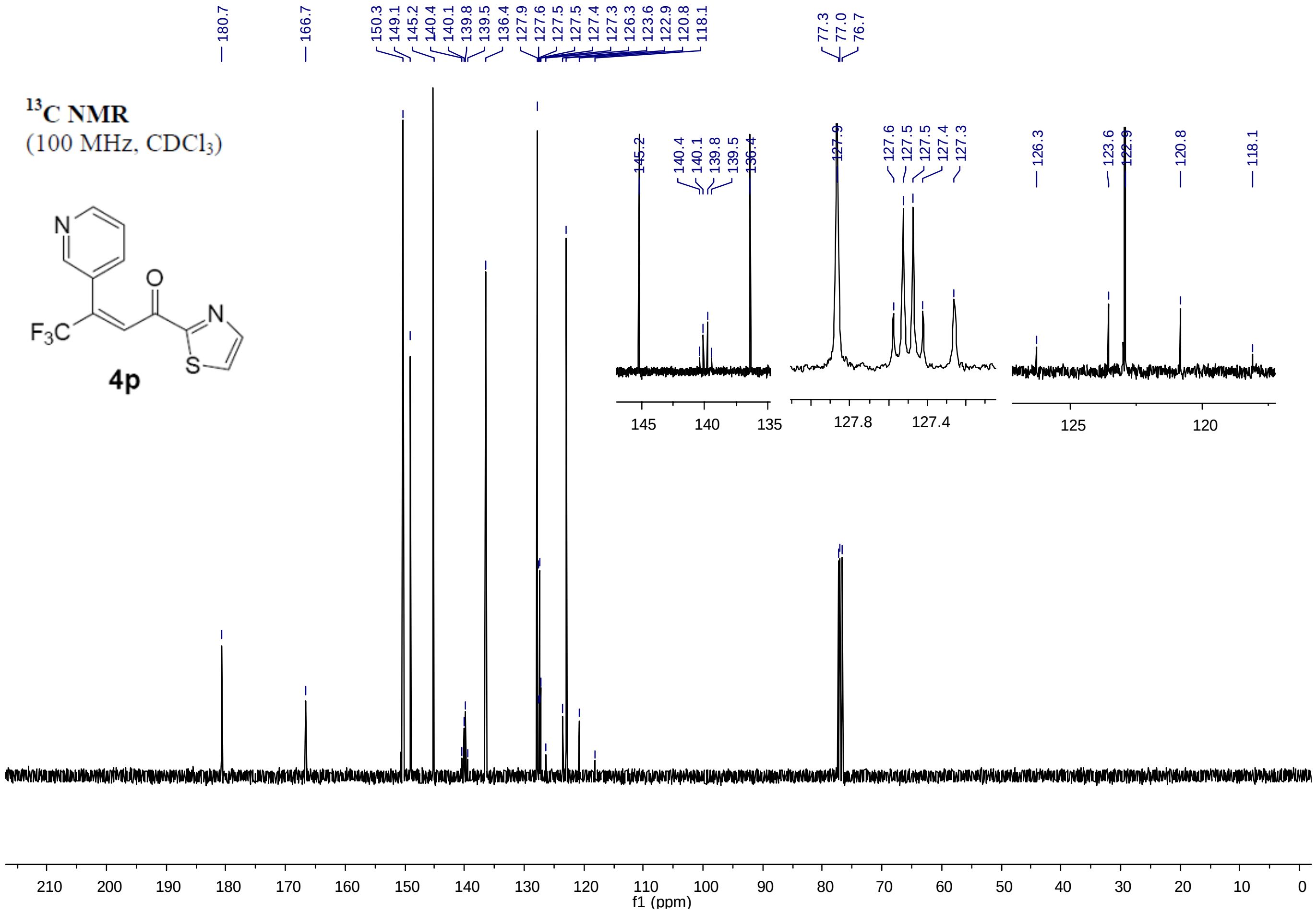

180.7 166.7 150.3 149.1 145.2 140.4 140.1 139.8 139.5 136.4 127.9 127.6 127.5 127.5 127.4 127.3 126.3 123.6 122.9 120.8 118.1 77.3 77.0 76.7
^{13}C NMR
(100 MHz, CDCl$_3$)
F_3C
4p
210 200 190 180 170 160 150 140 130 120 110 100 90 80 70 60 50 40 30 20 10 0
f1 (ppm)

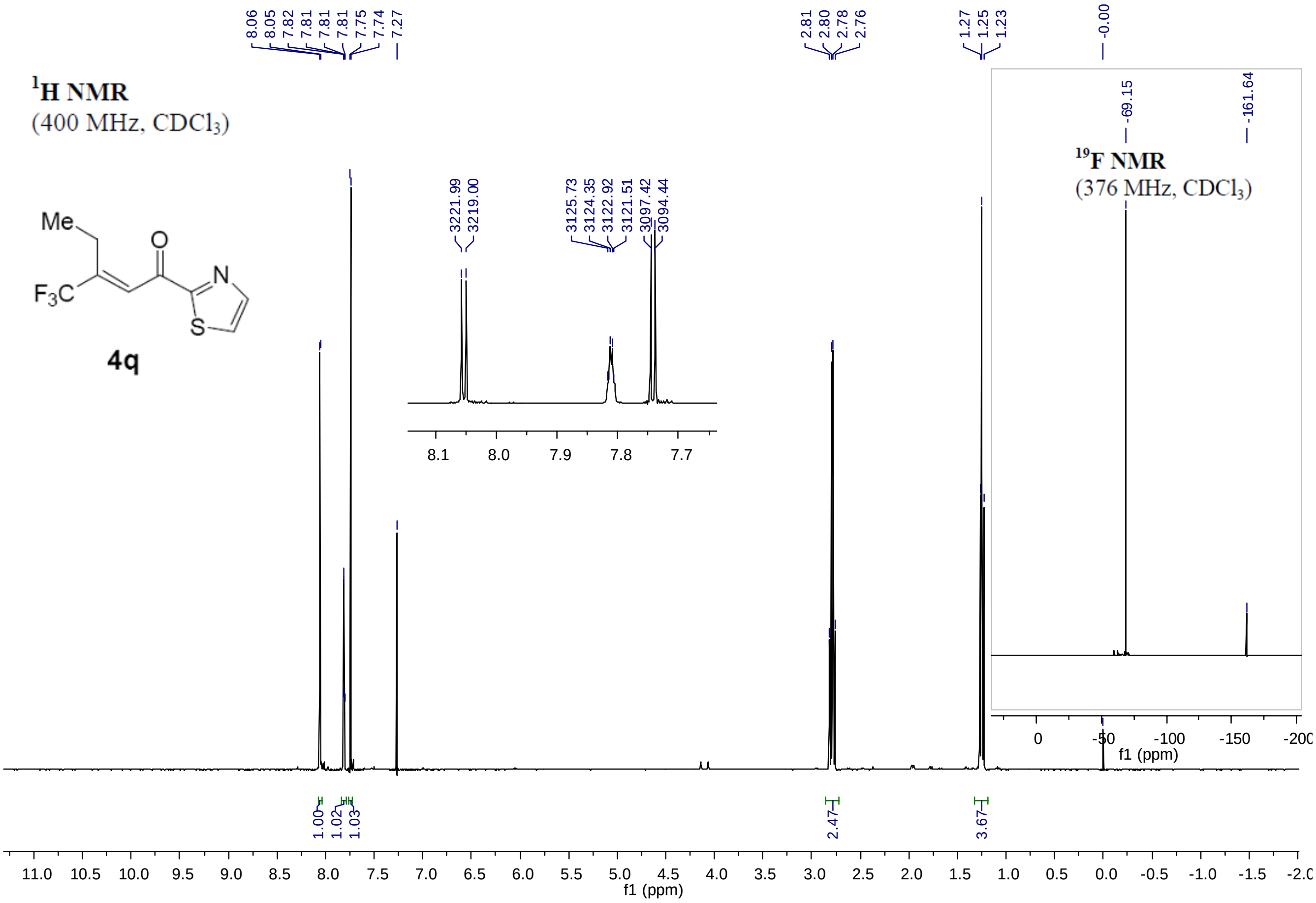

8.06 8.05 7.82 7.81 7.81 7.81 7.75 7.74 7.27
2.81 2.80 2.78 2.76
1.27 1.25 1.23
-0.00
^{1}H NMR
(400 MHz, CDCl$_3$)
Me
O
N
F$_3$C
S
4q
3221.99 3219.00
3125.73 3124.35 3122.92 3121.51 3097.42 3094.44
8.1 8.0 7.9 7.8 7.7
-69.15
-161.64
^{19}F NMR
(376 MHz, CDCl$_3$)
0 -50 -100 -150 -200
f1 (ppm)
1.00 1.02 1.03
2.47
3.67
11.0 10.5 10.0 9.5 9.0 8.5 8.0 7.5 7.0 6.5 6.0 5.5 5.0 4.5 4.0 3.5 3.0 2.5 2.0 1.5 1.0 0.5 0.0 -0.5 -1.0 -1.5 -2.0
f1 (ppm)

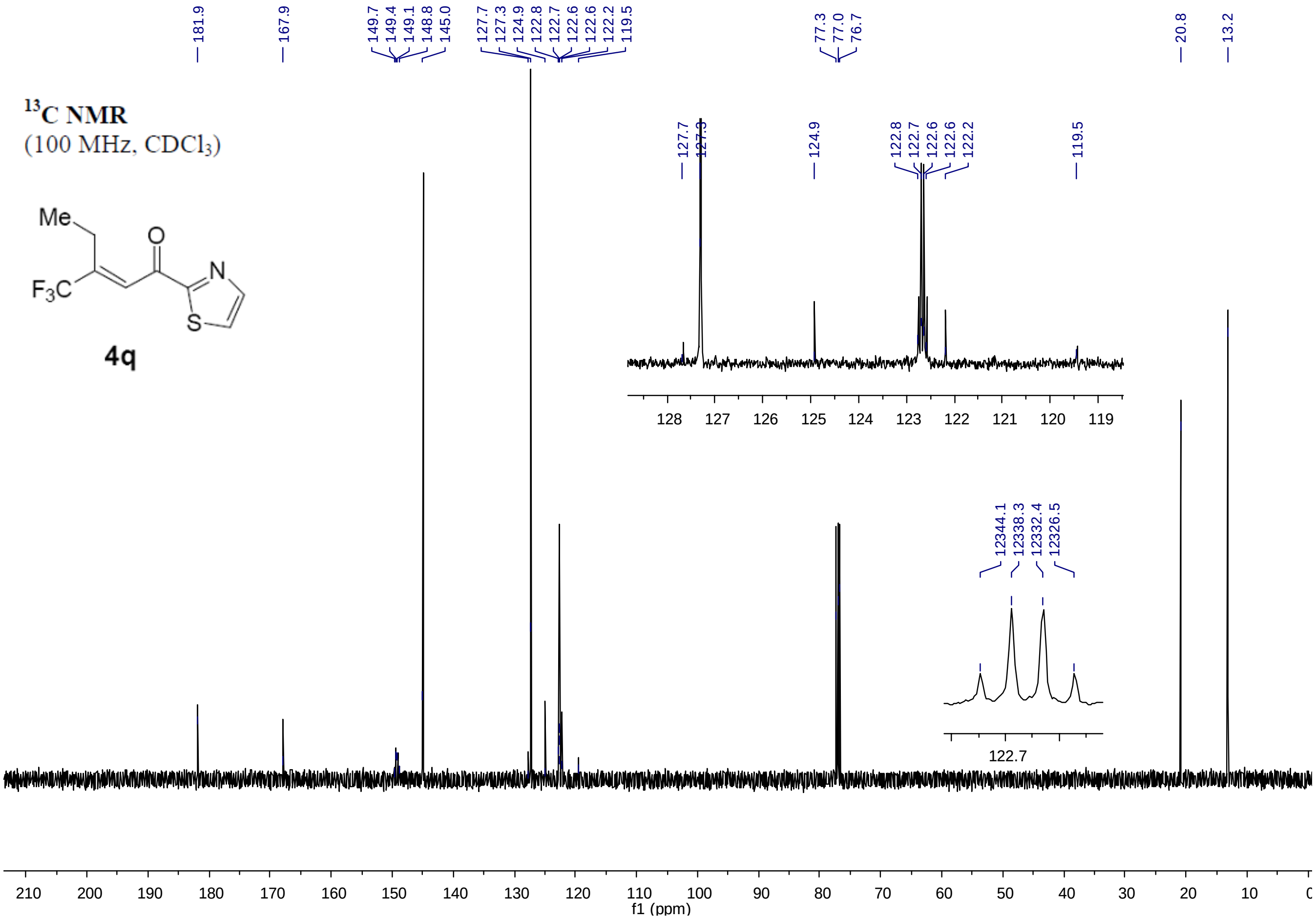

181.9
167.9
149.7 149.4 149.1 148.8 145.0
127.7 127.3 124.9 122.8 122.7 122.6 122.6 122.2 119.5
77.3 77.0 76.7
20.8
13.2
^{13}C NMR
(100 MHz, CDCl$_3$)
Me
O
N
F$_3$C
S
4q
127.7 127.3
124.9
122.8 122.7 122.6 122.6 122.2
119.5
128 127 126 125 124 123 122 121 120 119
12344.1 12338.3 12332.4 12326.5
122.7
210 200 190 180 170 160 150 140 130 120 110 100 90 80 70 60 50 40 30 20 10 0
f1 (ppm)

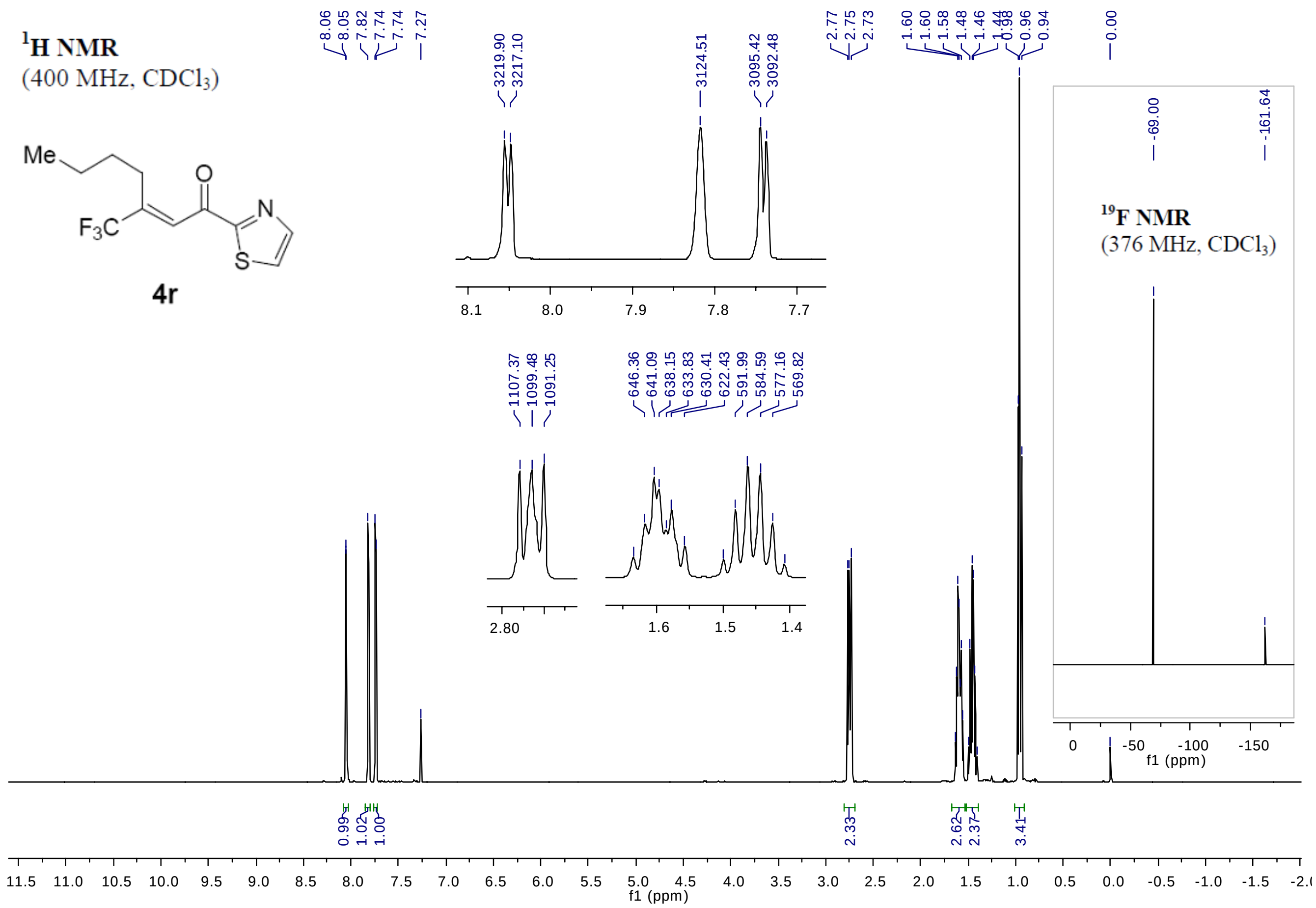
1H NMR
(400 MHz, CDCl3)
Me
F3C
O
N
S
4r
19F NMR
(376 MHz, CDCl3)
-69.00
-161.64
f1 (ppm)

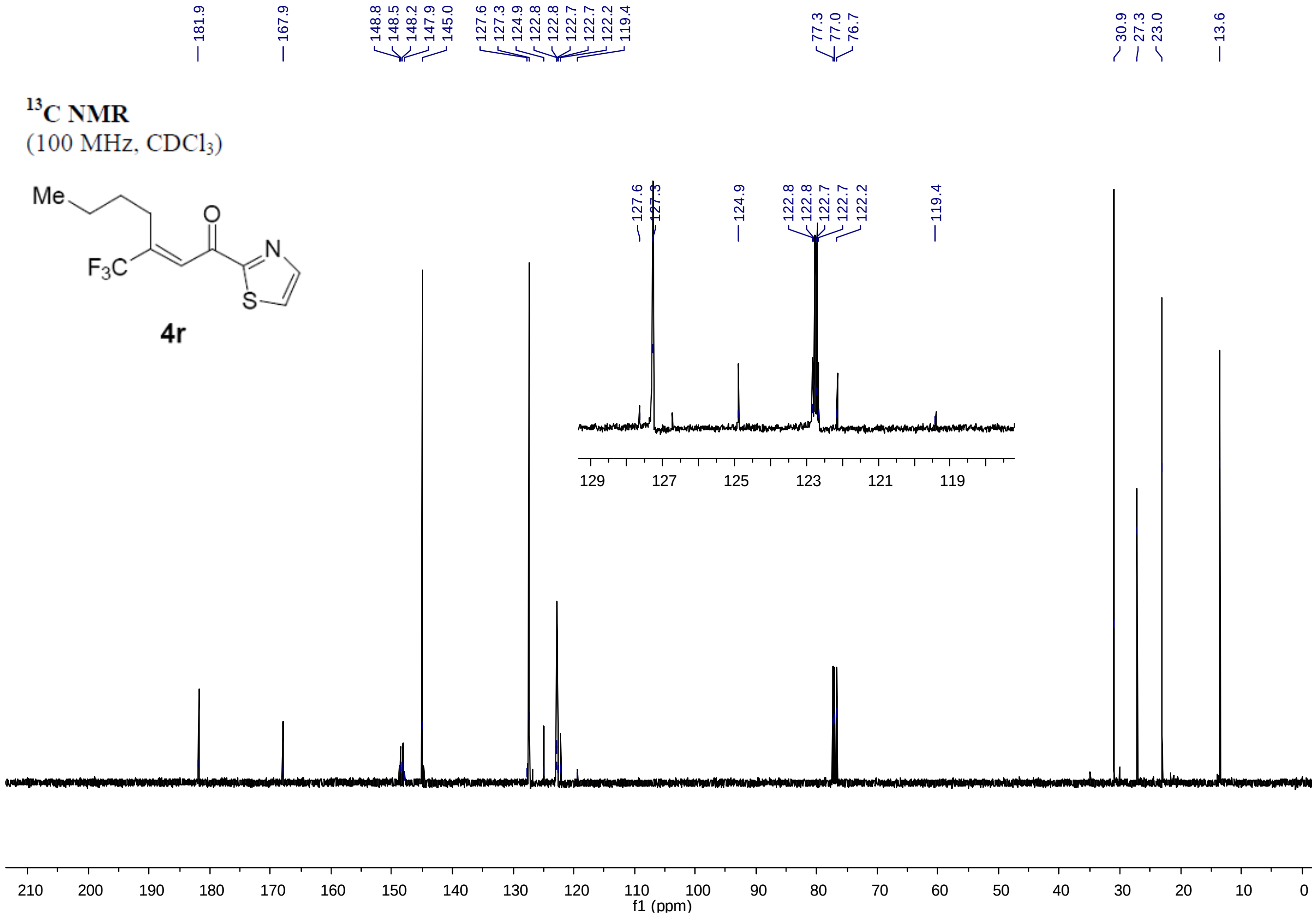
13C NMR
(100 MHz, CDCl3)
Me
F3C
O
N
S
4r
181.9
167.9
148.8
148.5
148.2
147.9
145.0
127.6
127.3
124.9
122.8
122.8
122.7
122.7
122.2
119.4
77.3
77.0
76.7
30.9
27.3
23.0
13.6
f1 (ppm)

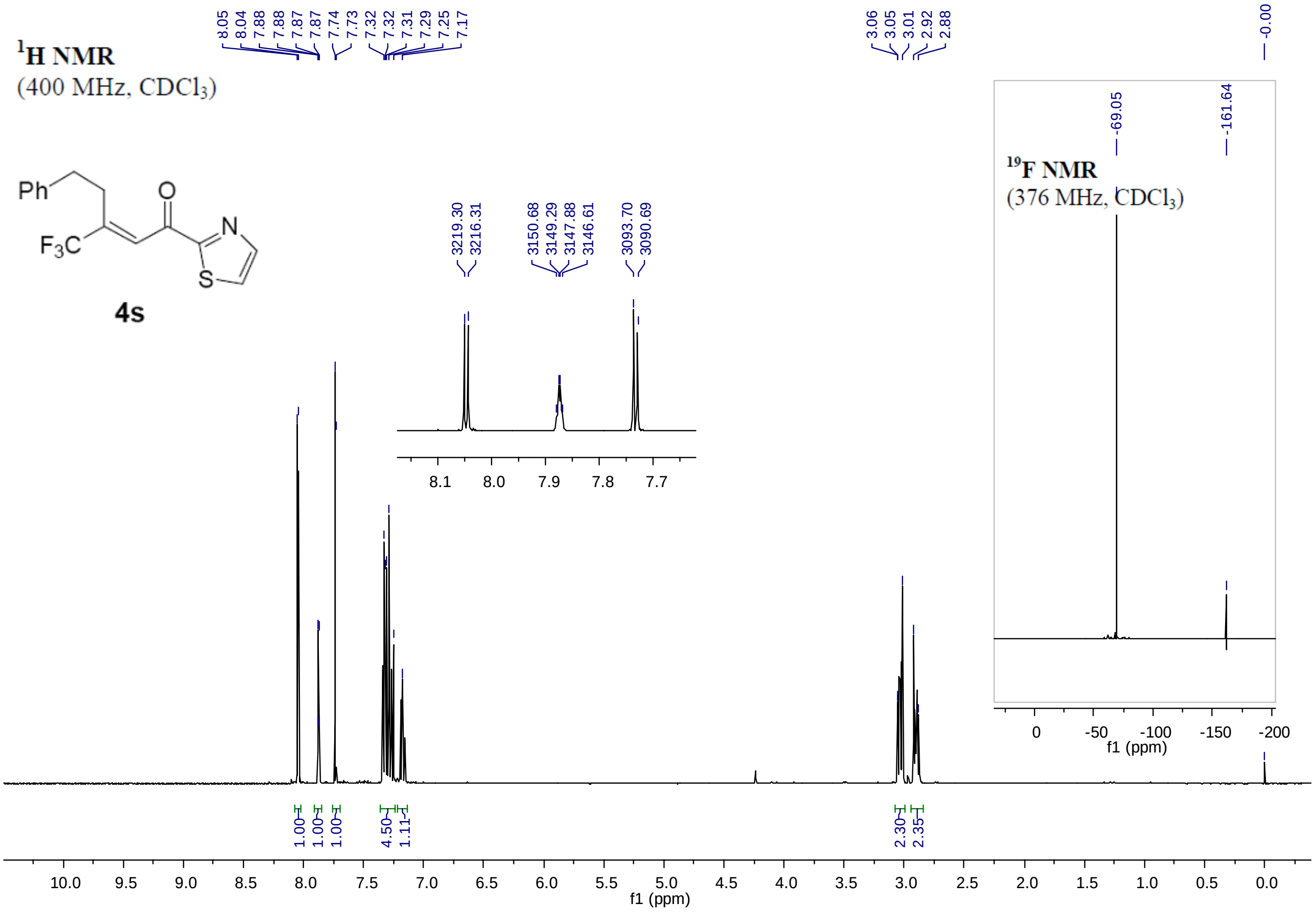

^{1}H NMR
(400 MHz, CDCl$_3$)
Ph
O
N
F$_3$C
S
4s
8.05 8.04 7.88 7.88 7.87 7.87 7.74 7.73 7.32 7.32 7.31 7.29 7.25 7.17
3.06 3.05 3.01 2.92 2.88
-0.00
3219.30 3216.31
3150.68 3149.29 3147.88 3146.61
3093.70 3090.69
^{19}F NMR
(376 MHz, CDCl$_3$)
-69.05
-161.64
1.00 1.00 1.00 4.50 1.11
2.30 2.35
f1 (ppm)

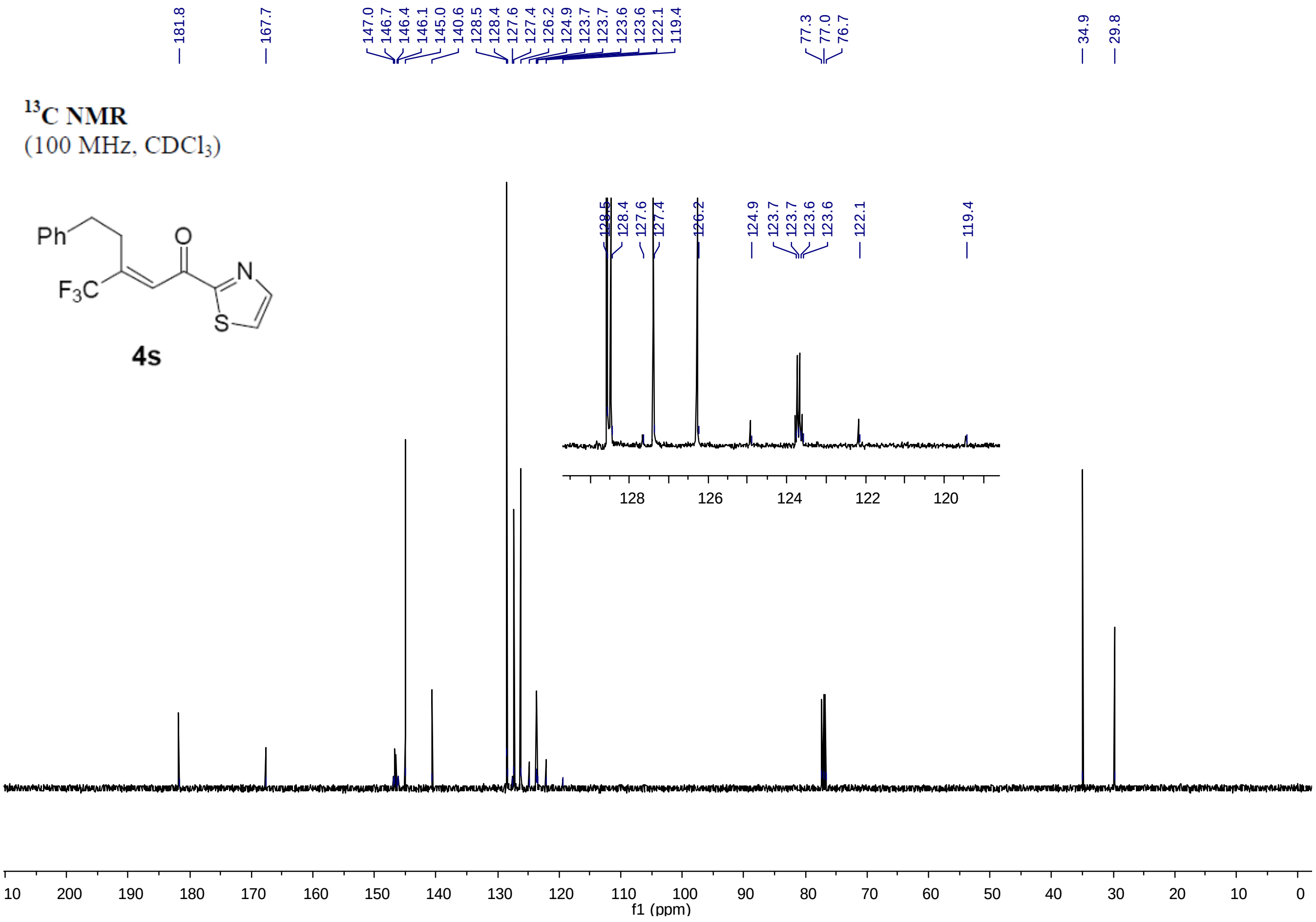

181.8
167.7
147.0 146.7 146.4 146.1 145.0 140.6 128.5 128.4 127.6 127.4 126.2 124.9 123.7 123.7 123.6 123.6 122.1 119.4
77.3 77.0 76.7
34.9 29.8
^{13}C NMR
(100 MHz, CDCl$_3$)
Ph
O
N
F$_3$C
S
4s
f1 (ppm)

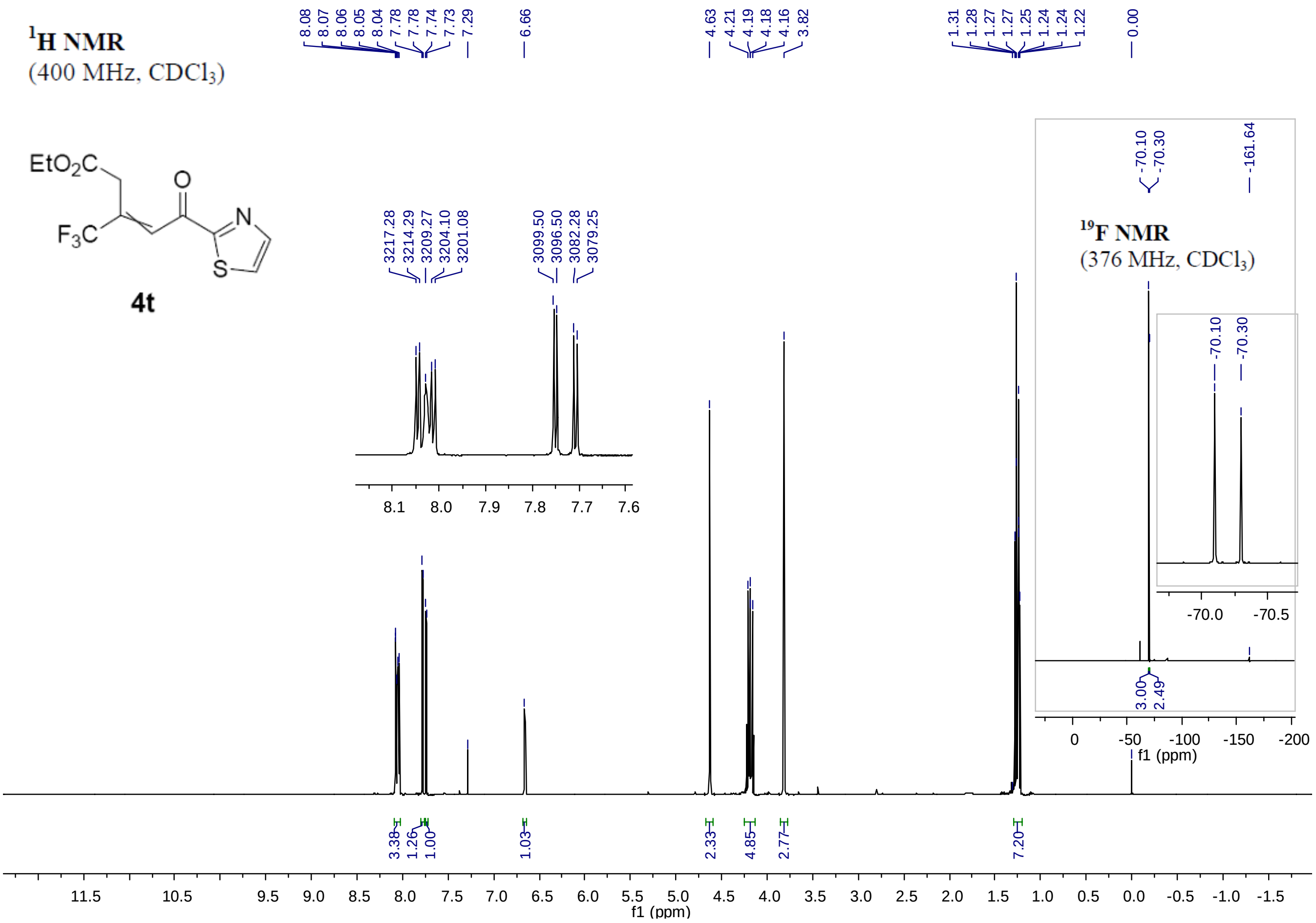
1H NMR
(400 MHz, CDCl3)
4t
19F NMR
(376 MHz, CDCl3)
f1 (ppm)

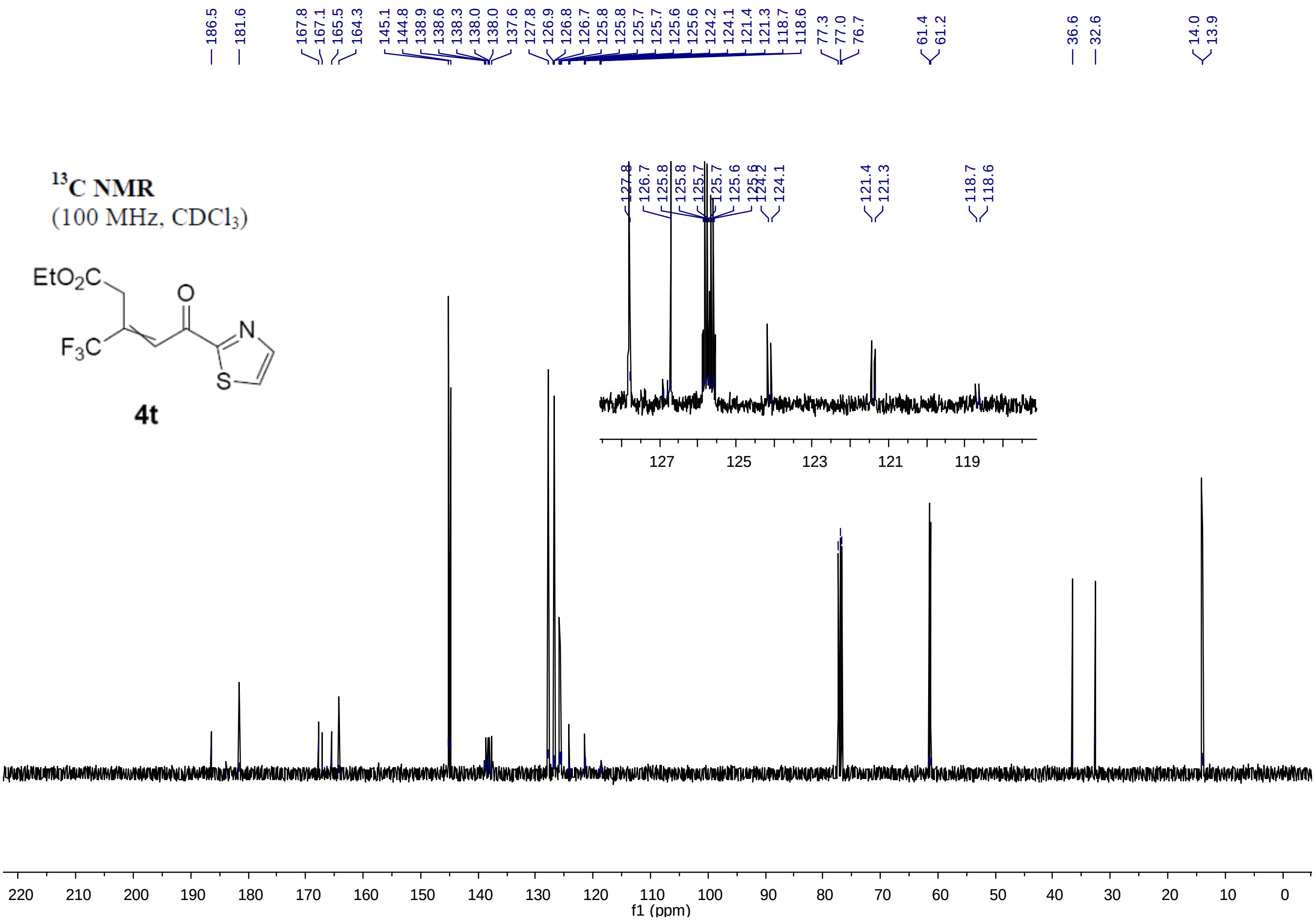
13C NMR
(100 MHz, CDCl3)
4t
f1 (ppm)

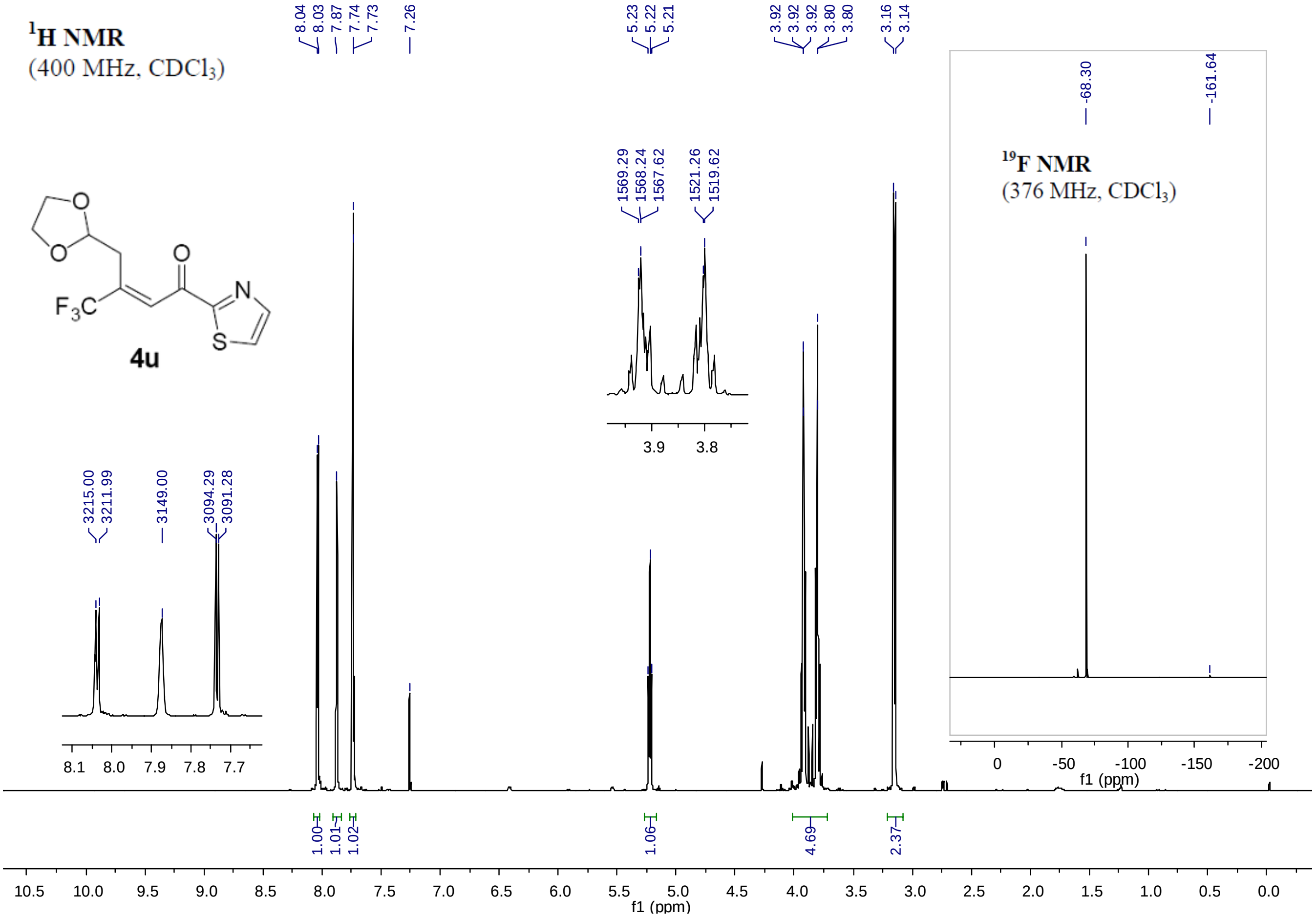

1H NMR
(400 MHz, CDCl3)
4u
19F NMR
(376 MHz, CDCl3)
f1 (ppm)

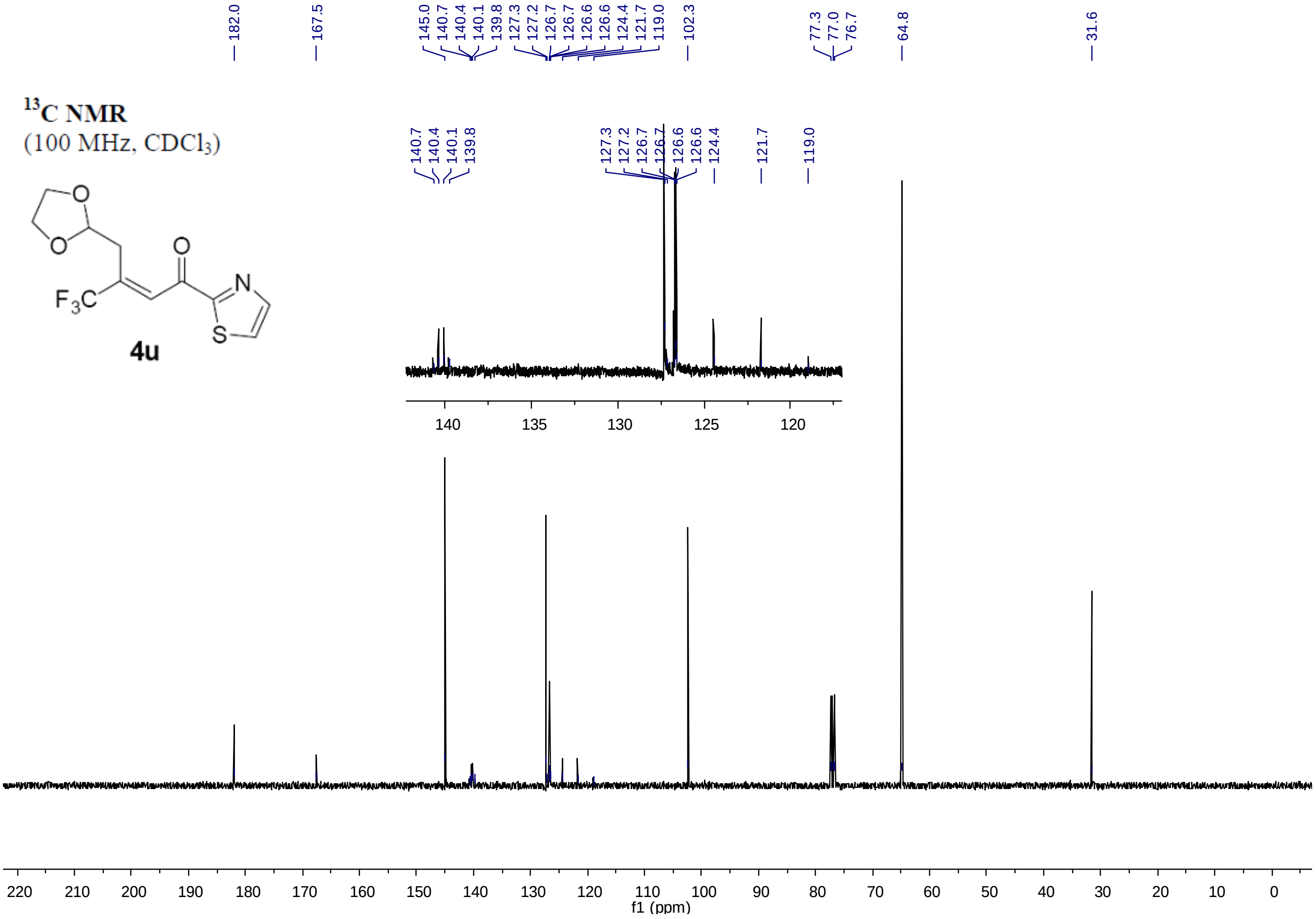

13C NMR
(100 MHz, CDCl3)
4u
f1 (ppm)

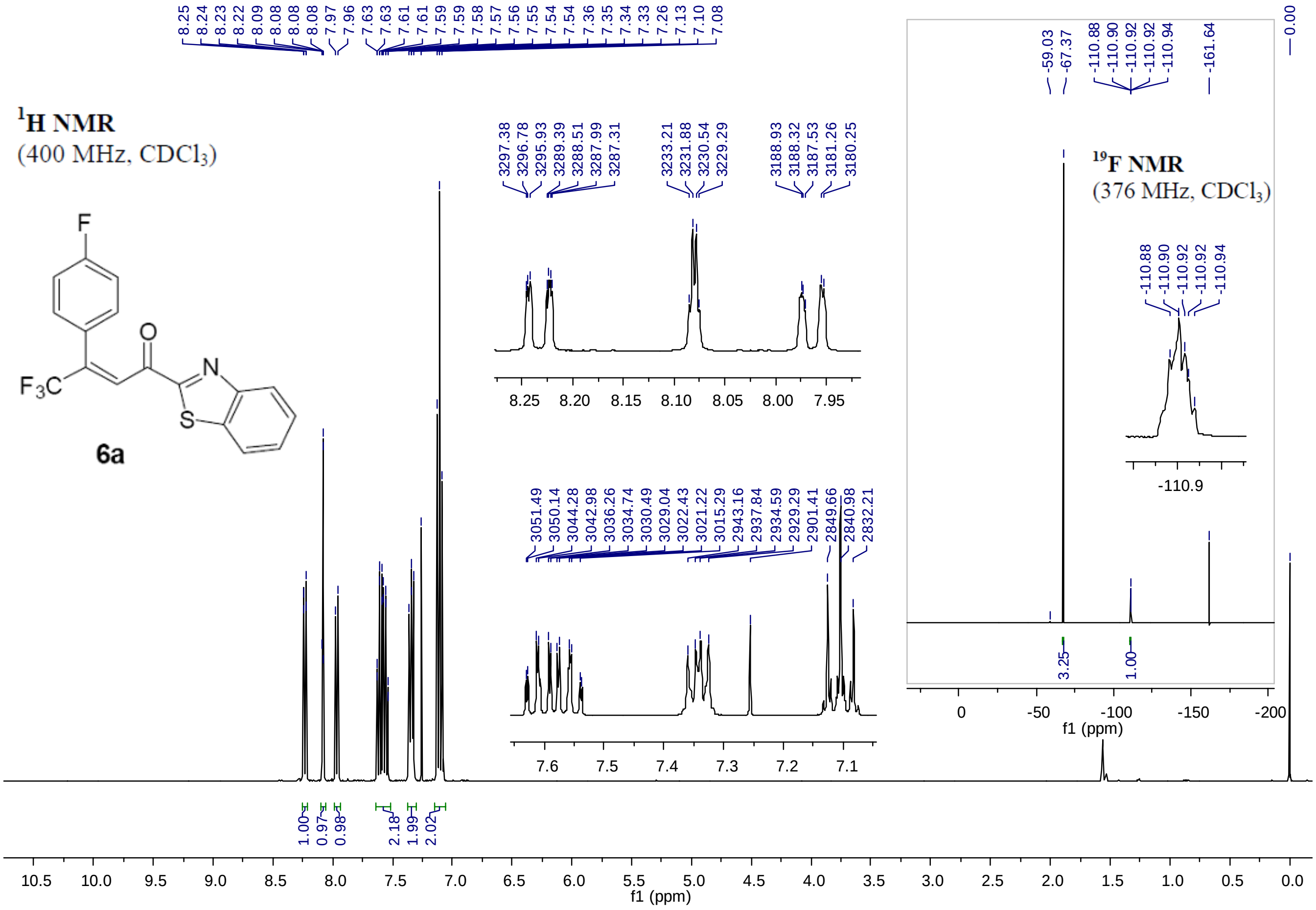

1H NMR
(400 MHz, CDCl3)
6a
F
F3C
O
N
S
8.25 8.24 8.23 8.22 8.09 8.08 8.08 8.08 7.97 7.96 7.63 7.63 7.61 7.61 7.59 7.59 7.58 7.57 7.56 7.55 7.54 7.54 7.36 7.35 7.34 7.33 7.26 7.13 7.10 7.08
3297.38 3296.78 3295.93 3289.39 3288.51 3287.99 3287.31 3233.21 3231.88 3230.54 3229.29 3188.93 3188.32 3187.53 3181.26 3180.25
3051.49 3050.14 3044.28 3042.98 3036.26 3034.74 3030.49 3029.04 3022.43 3021.22 3015.29 2943.16 2937.84 2934.59 2929.29 2901.41 2849.66 2840.98 2832.21
1.00 0.97 0.98 2.18 1.99 2.02
f1 (ppm)
19F NMR
(376 MHz, CDCl3)
-59.03 -67.37 -110.88 -110.90 -110.92 -110.92 -110.94 -161.64 -0.00
3.25 1.00
-110.9

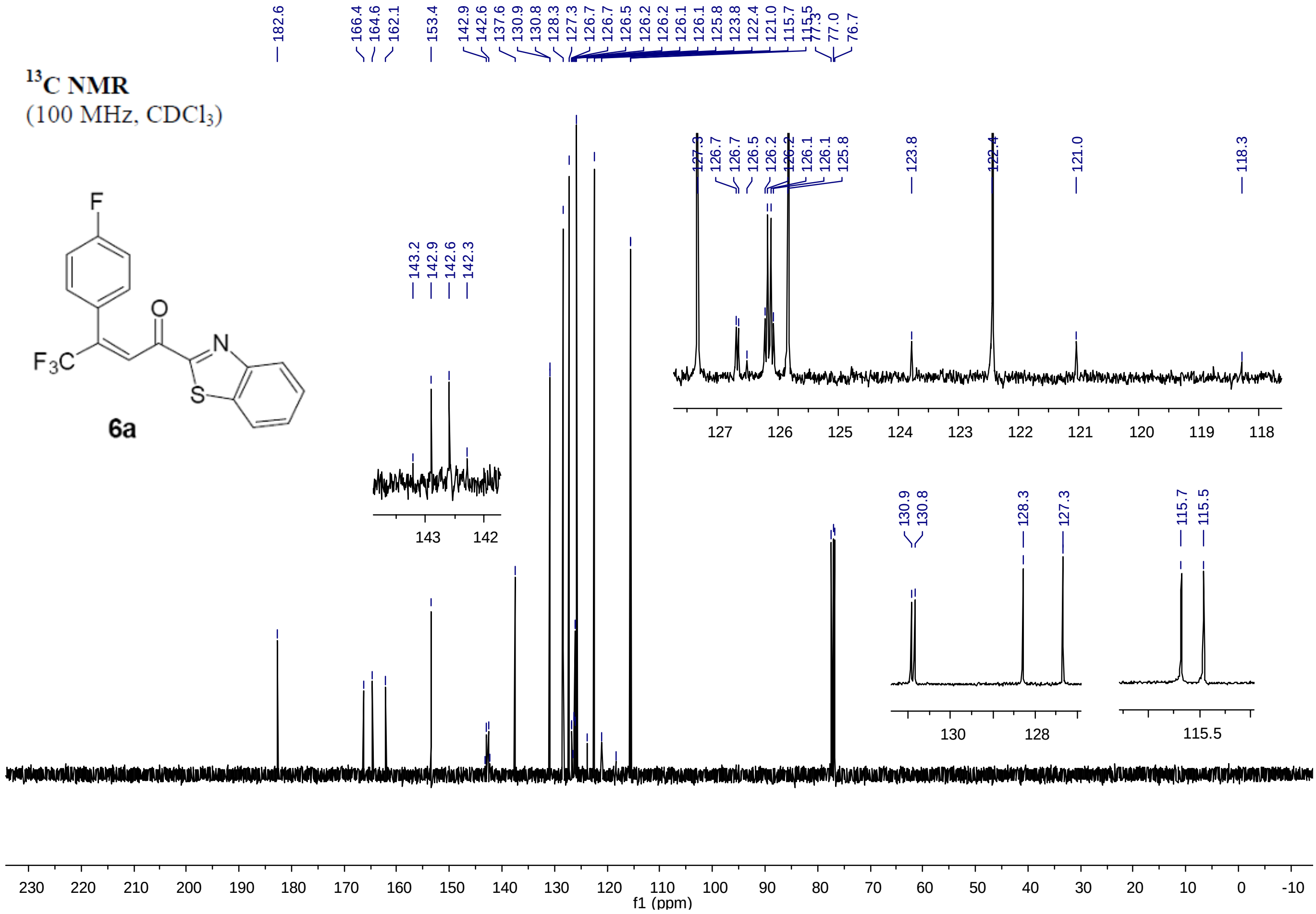

13C NMR
(100 MHz, CDCl3)
6a
F
F3C
O
N
S
182.6 166.4 164.6 162.1 153.4 142.9 142.6 137.6 130.9 130.8 128.3 127.3 126.7 126.7 126.5 126.2 126.2 126.1 126.1 125.8 123.8 122.4 121.0 115.7 115.5 77.3 77.0 76.7
143.2 142.9 142.6 142.3
127.3 126.7 126.7 126.5 126.2 126.2 126.1 126.1 125.8 123.8 122.4 121.0 118.3
130.9 130.8 128.3 127.3 115.7 115.5
f1 (ppm)

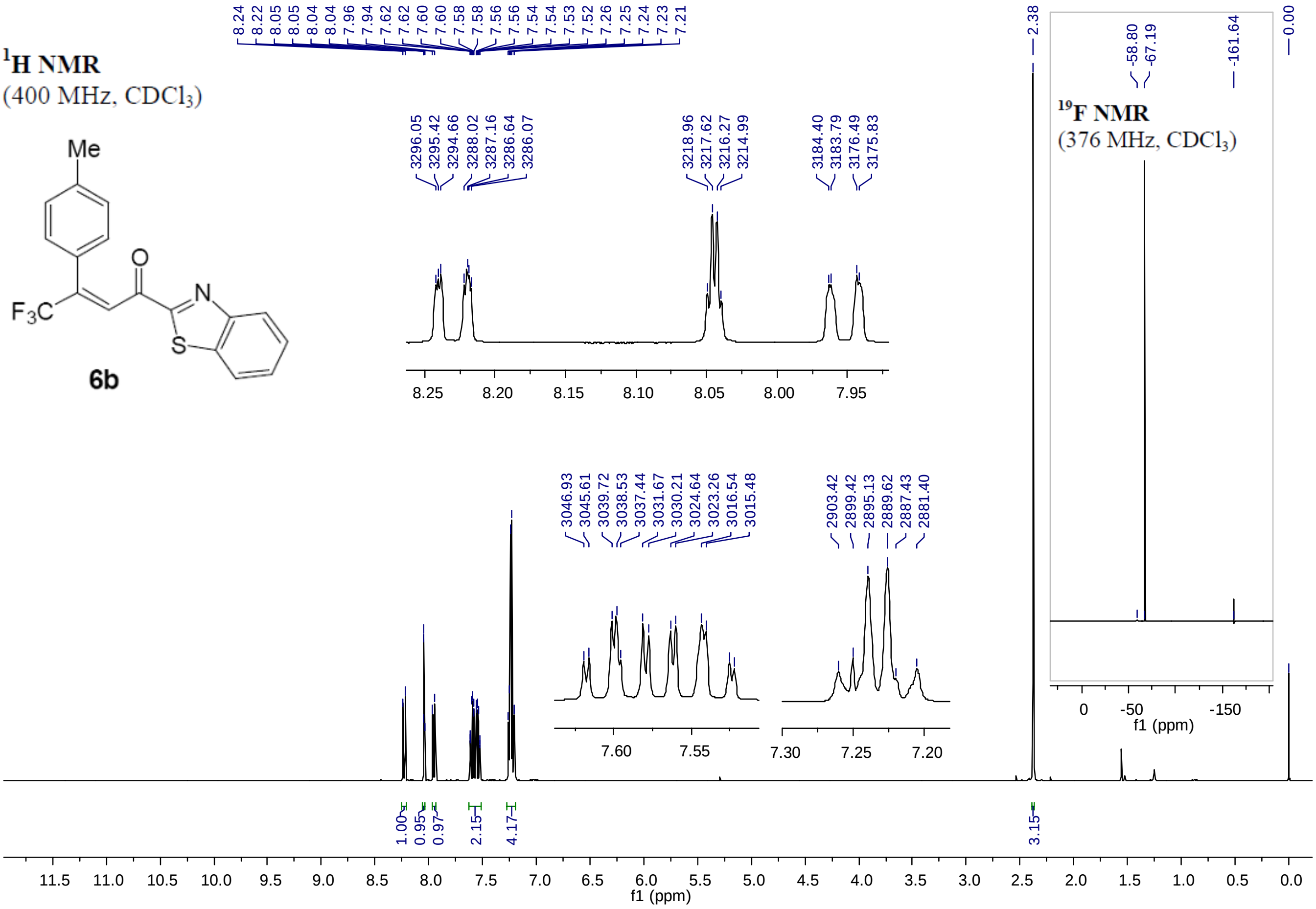
1H NMR
(400 MHz, CDCl3)
Me
O
N
F3C
S
6b
19F NMR
(376 MHz, CDCl3)
-58.80
-67.19
-161.64
f1 (ppm)

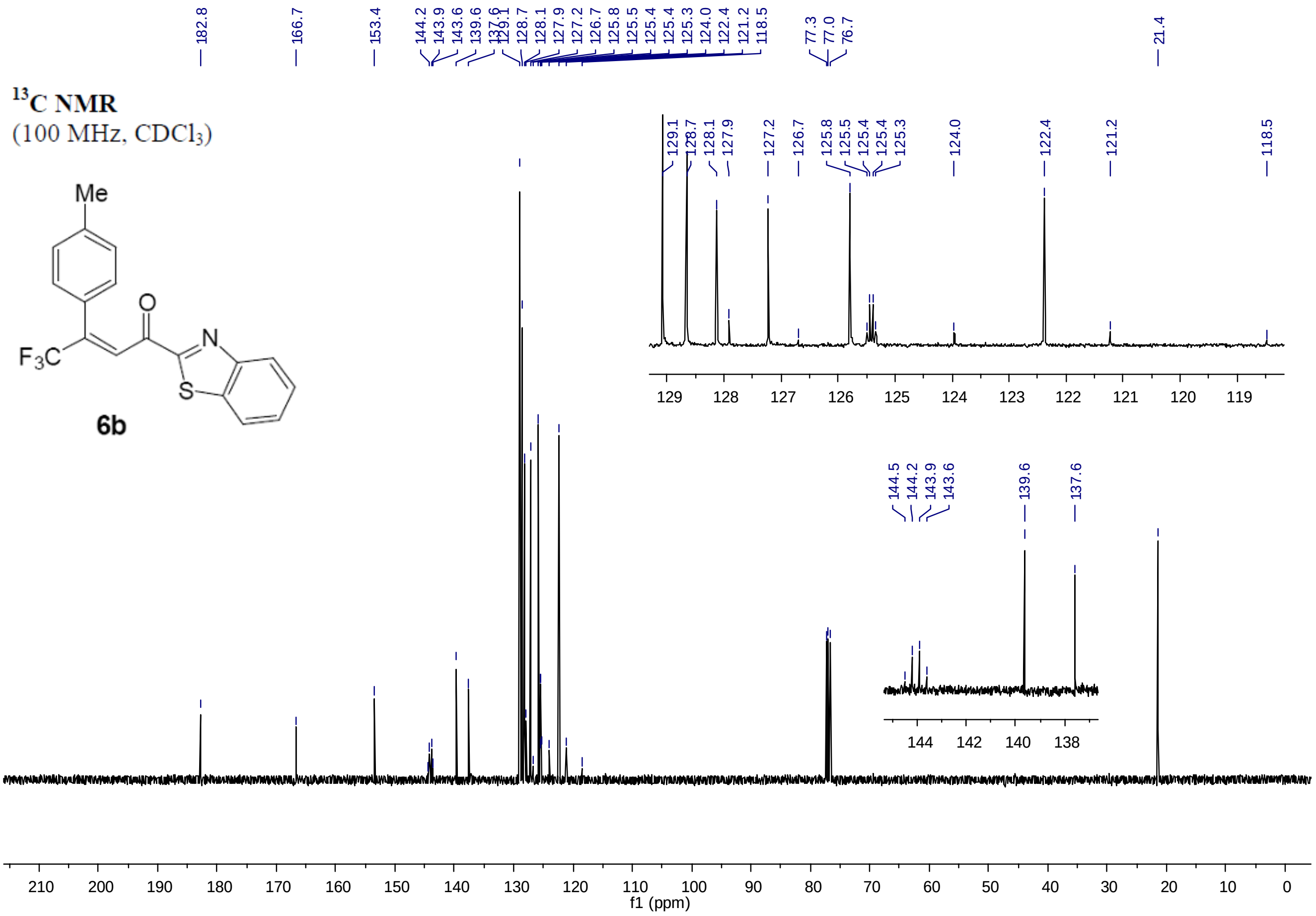
13C NMR
(100 MHz, CDCl3)
Me
O
N
F3C
S
6b
f1 (ppm)

^{1}H NMR
(400 MHz, CDCl$_3$)

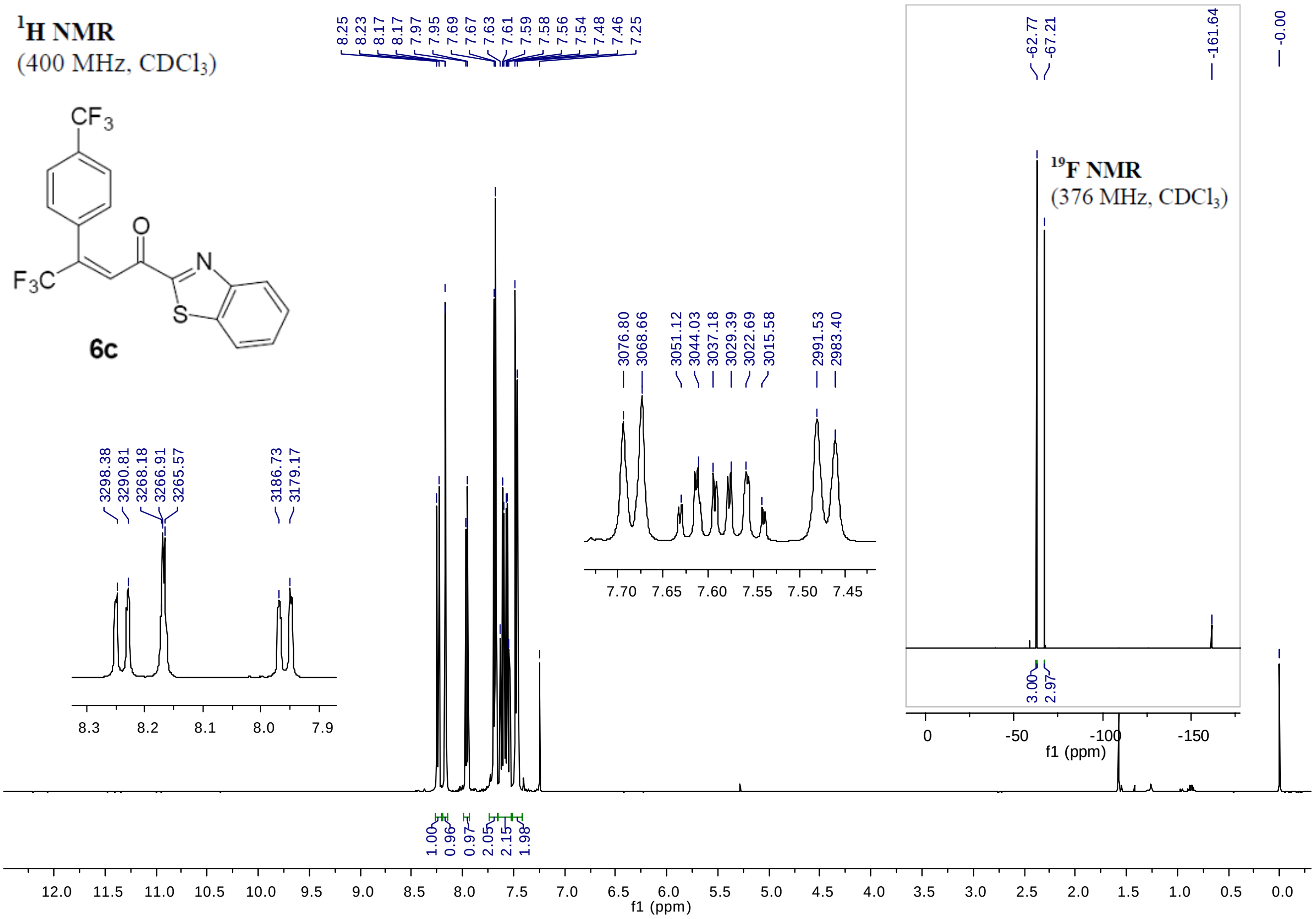

^{13}C NMR
(100 MHz, CDCl$_3$)

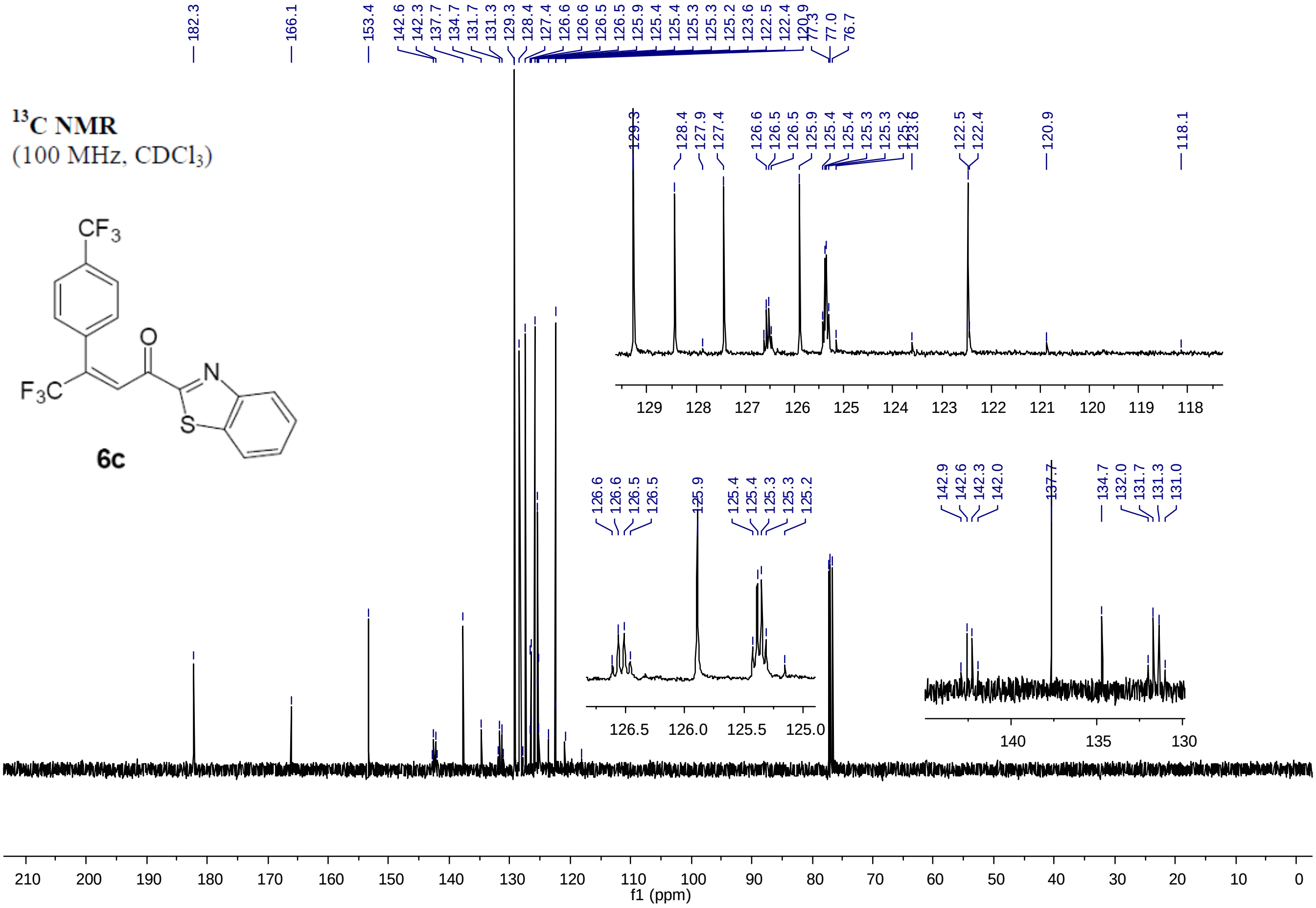

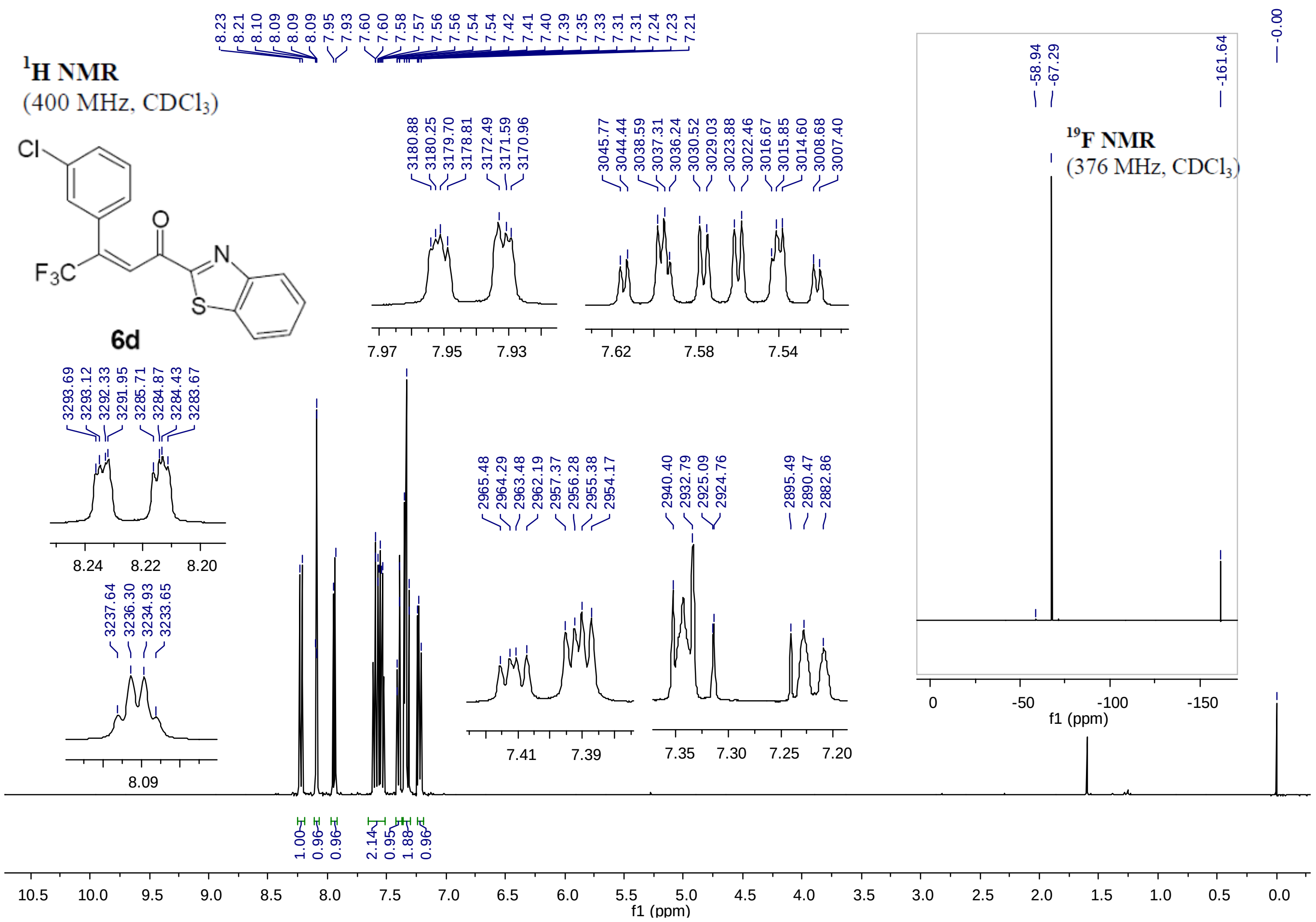

^{1}H NMR
(400 MHz, CDCl$_3$)
6d
^{19}F NMR
(376 MHz, CDCl$_3$)
f1 (ppm)

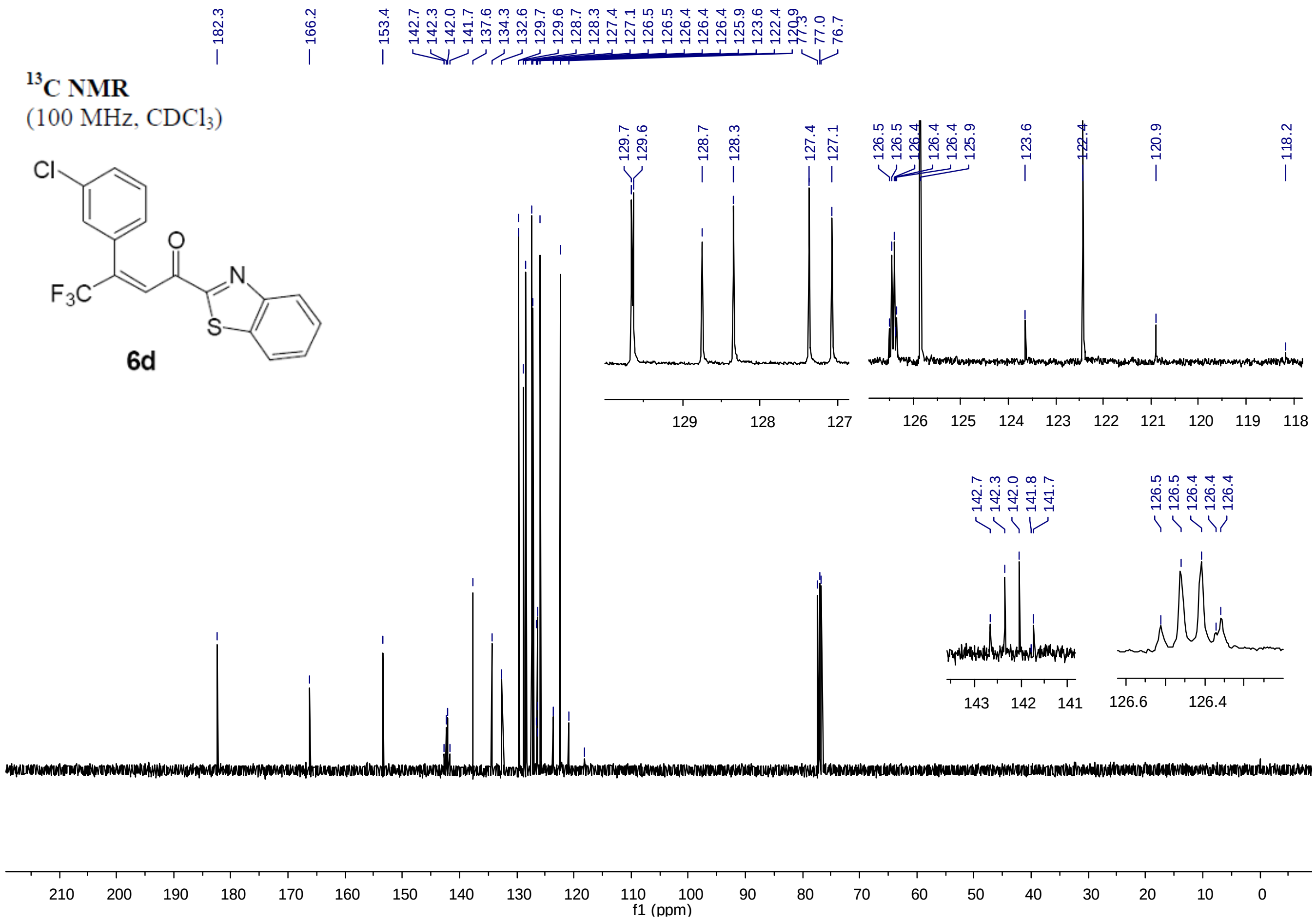

^{13}C NMR
(100 MHz, CDCl$_3$)
6d
f1 (ppm)

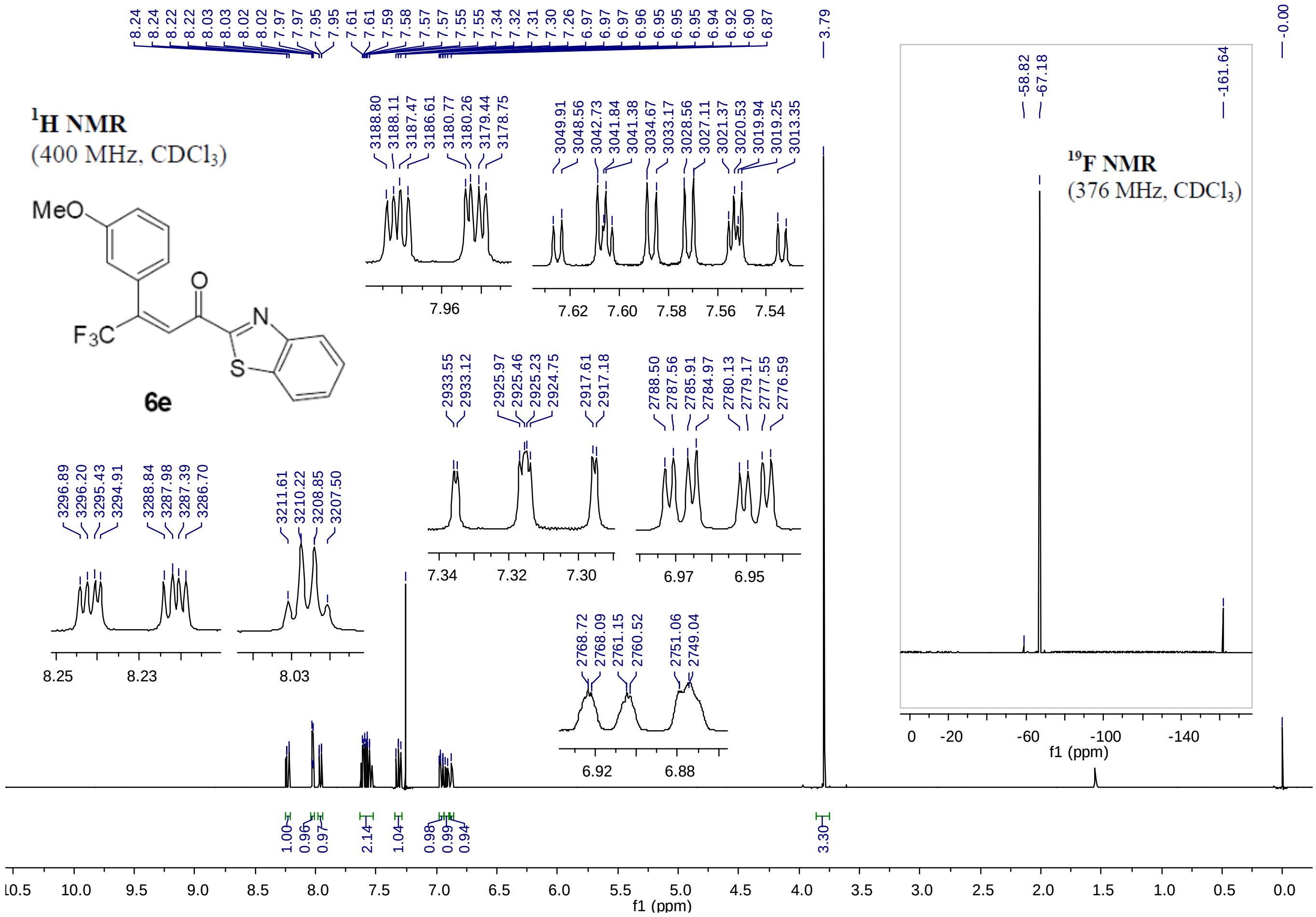

1H NMR
(400 MHz, CDCl3)
MeO
F3C
O
N
S
6e
19F NMR
(376 MHz, CDCl3)
-58.82
-67.18
-161.64
f1 (ppm)

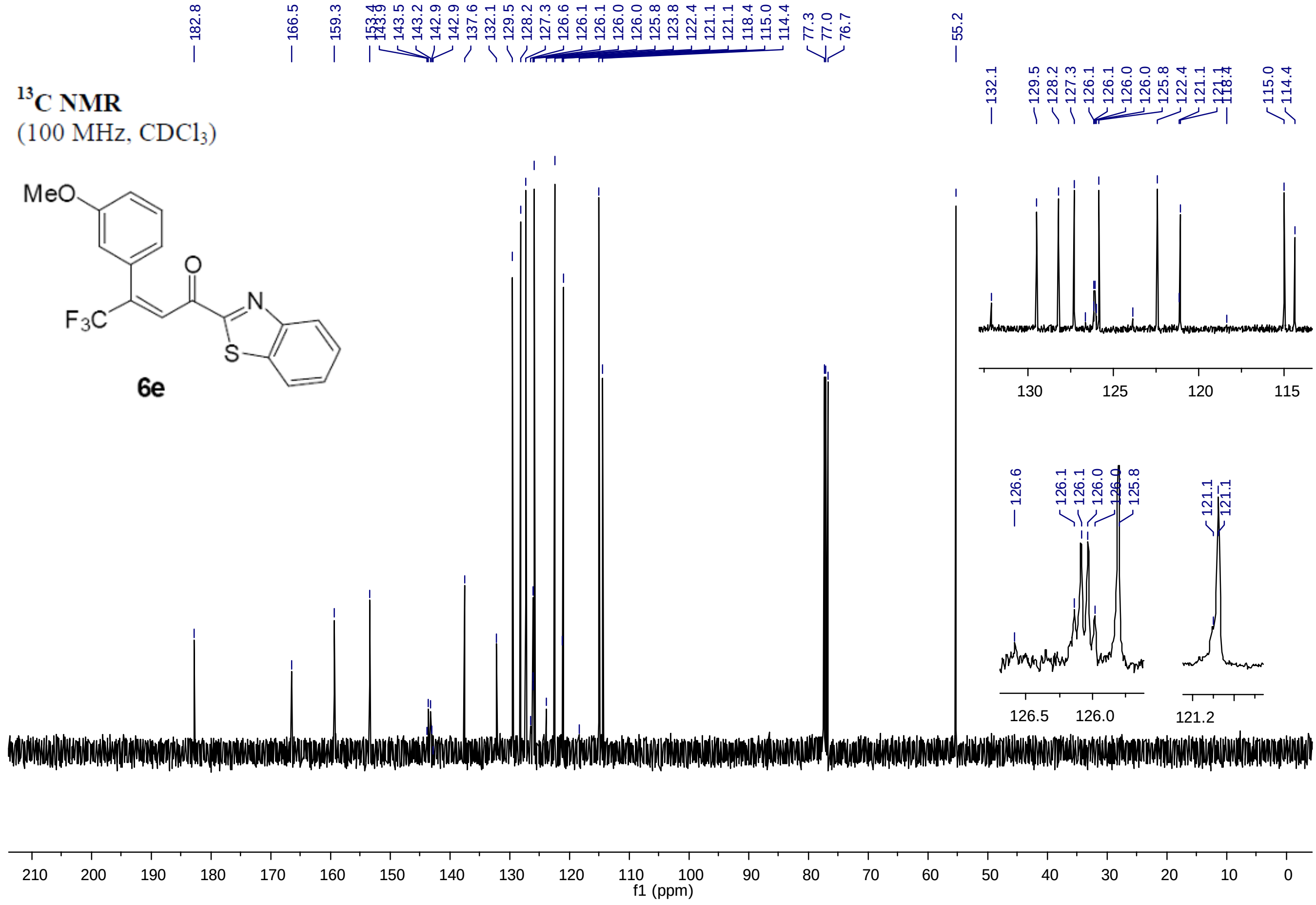

13C NMR
(100 MHz, CDCl3)
MeO
F3C
O
N
S
6e
f1 (ppm)

^{1}H NMR
(400 MHz, $CDCl_3$)

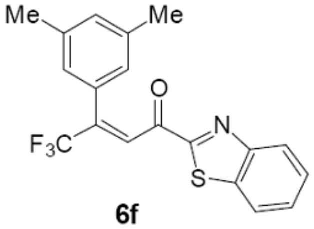

6f

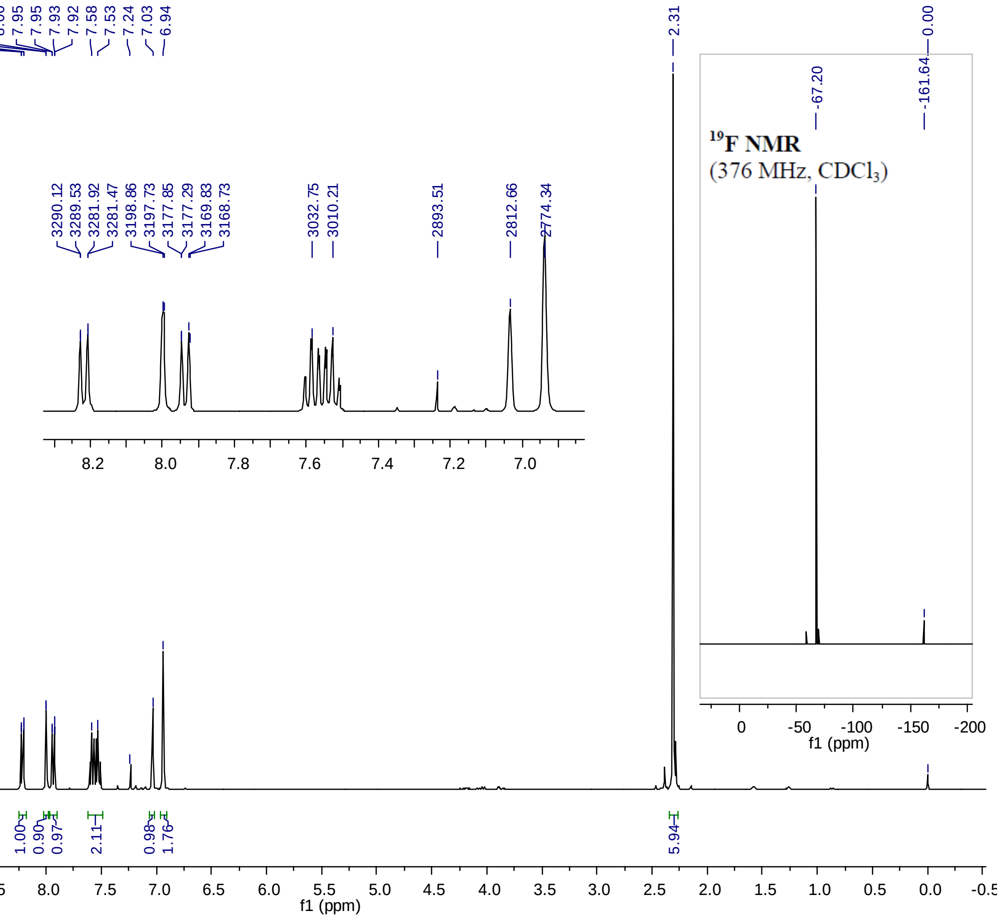

^{13}C NMR
(100 MHz, $CDCl_3$)

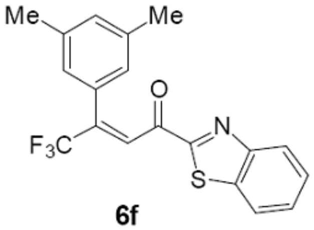

6f

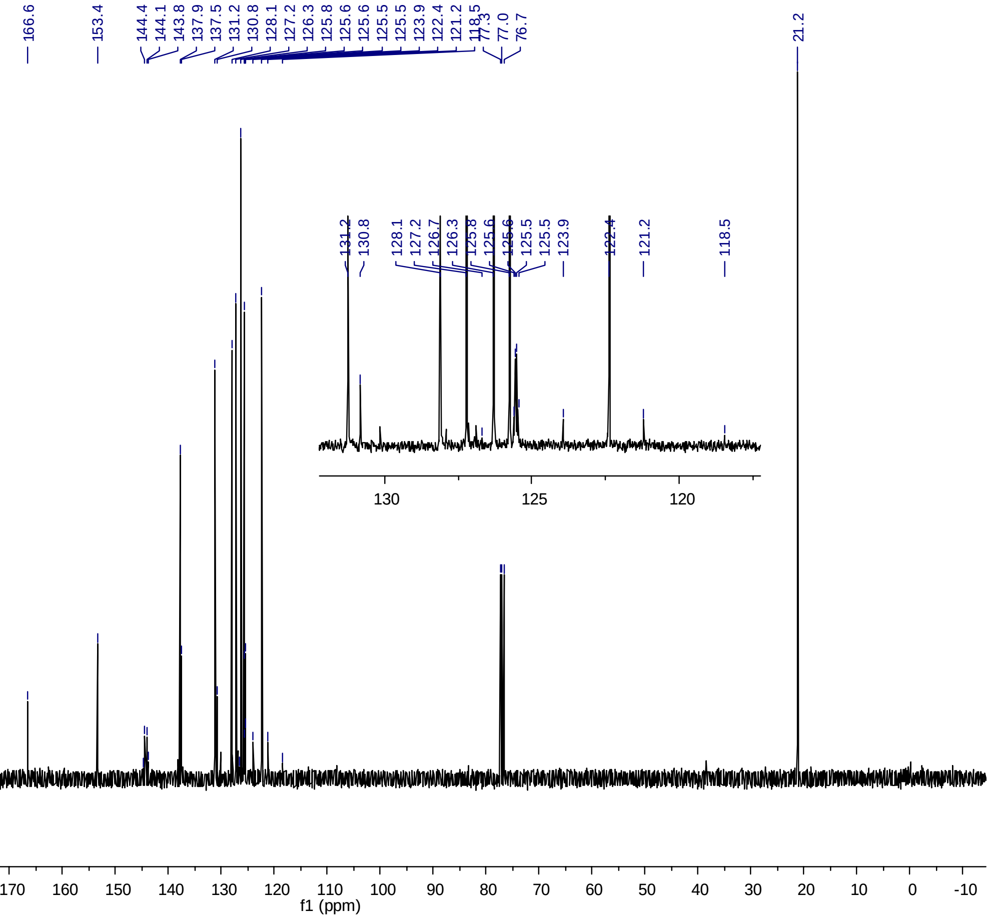

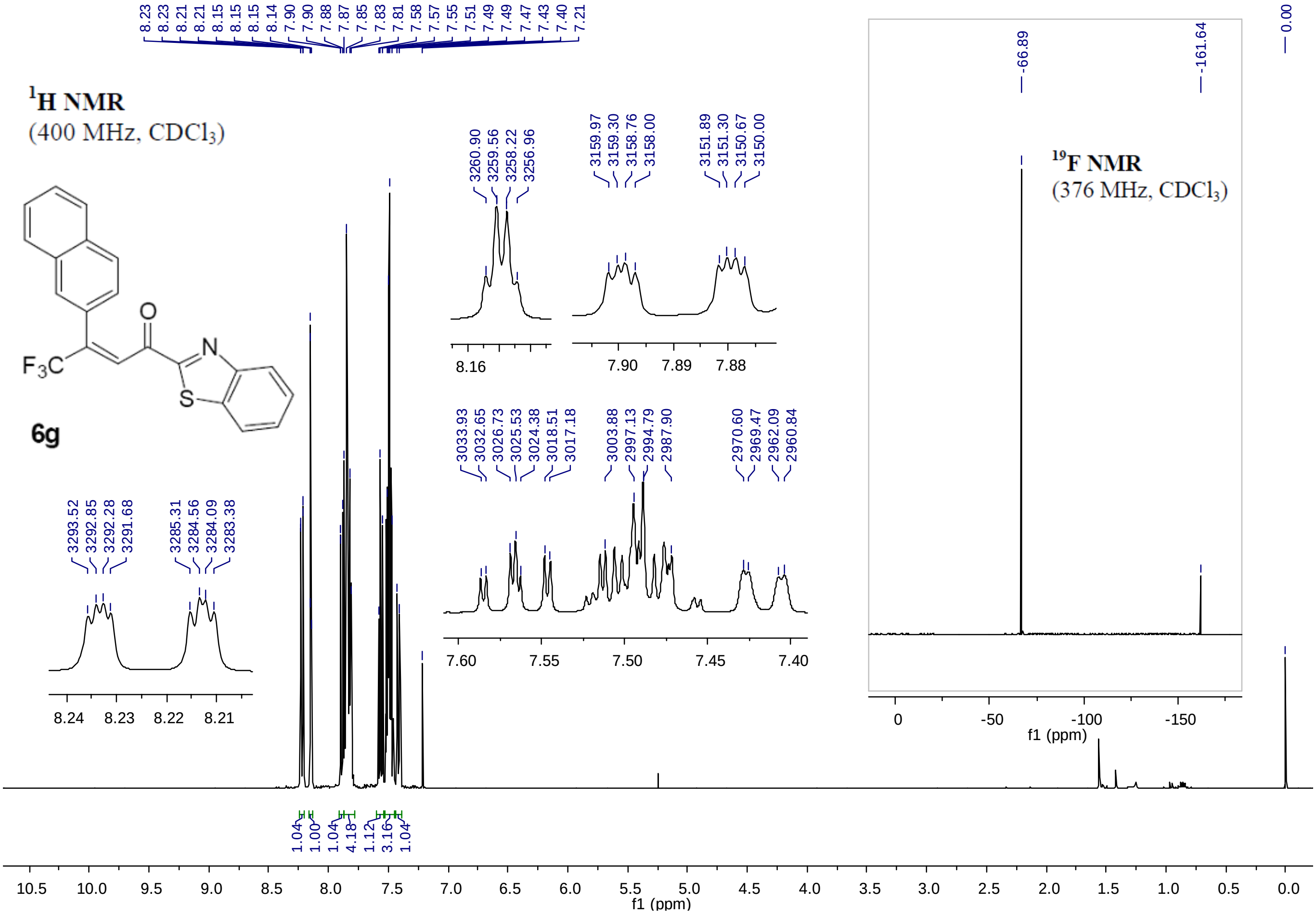

^{1}H NMR
(400 MHz, CDCl$_3$)
6g
^{19}F NMR
(376 MHz, CDCl$_3$)
-66.89
-161.64
0.00
f1 (ppm)

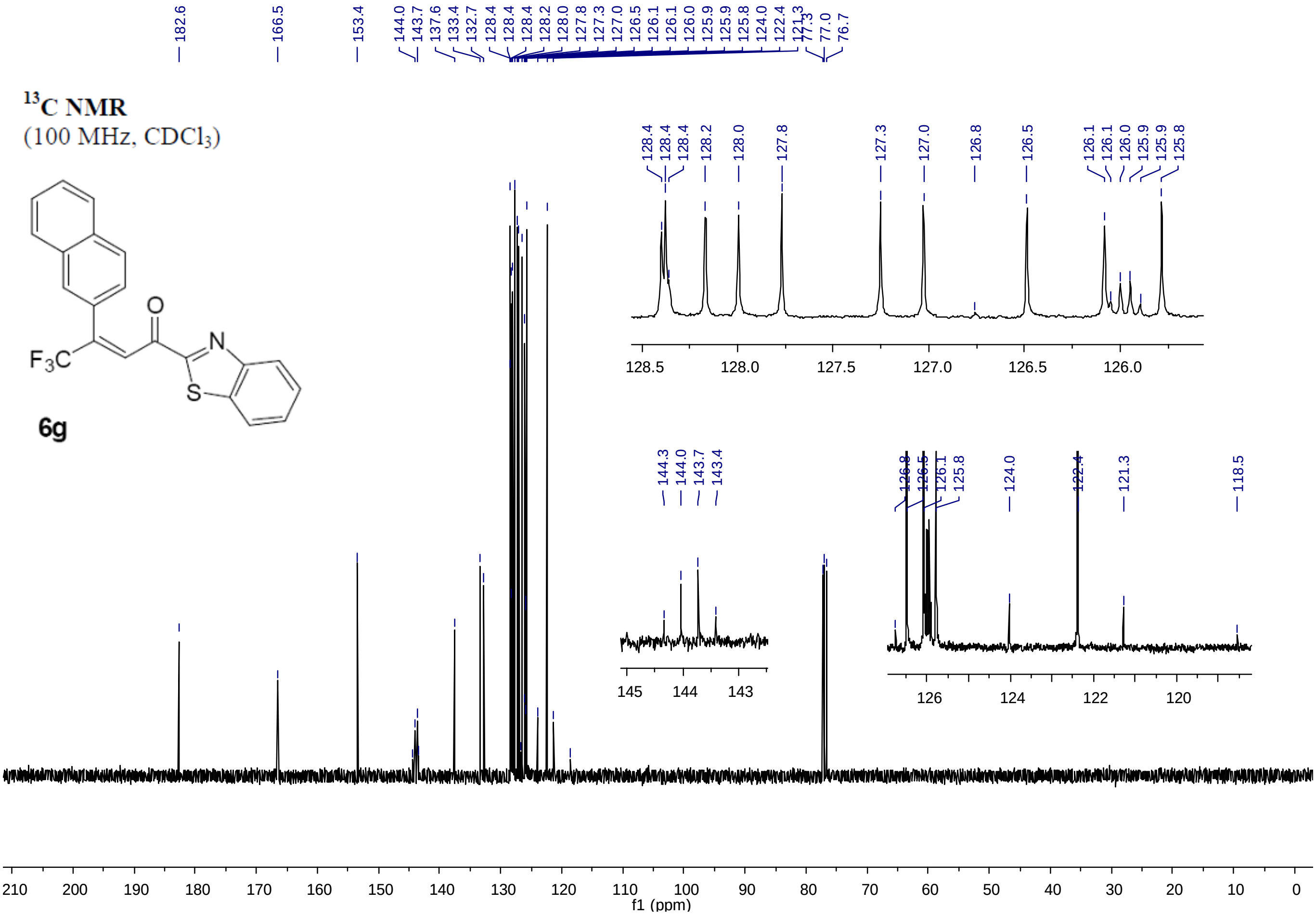

^{13}C NMR
(100 MHz, CDCl$_3$)
6g
182.6
166.5
153.4
77.3
77.0
76.7
f1 (ppm)

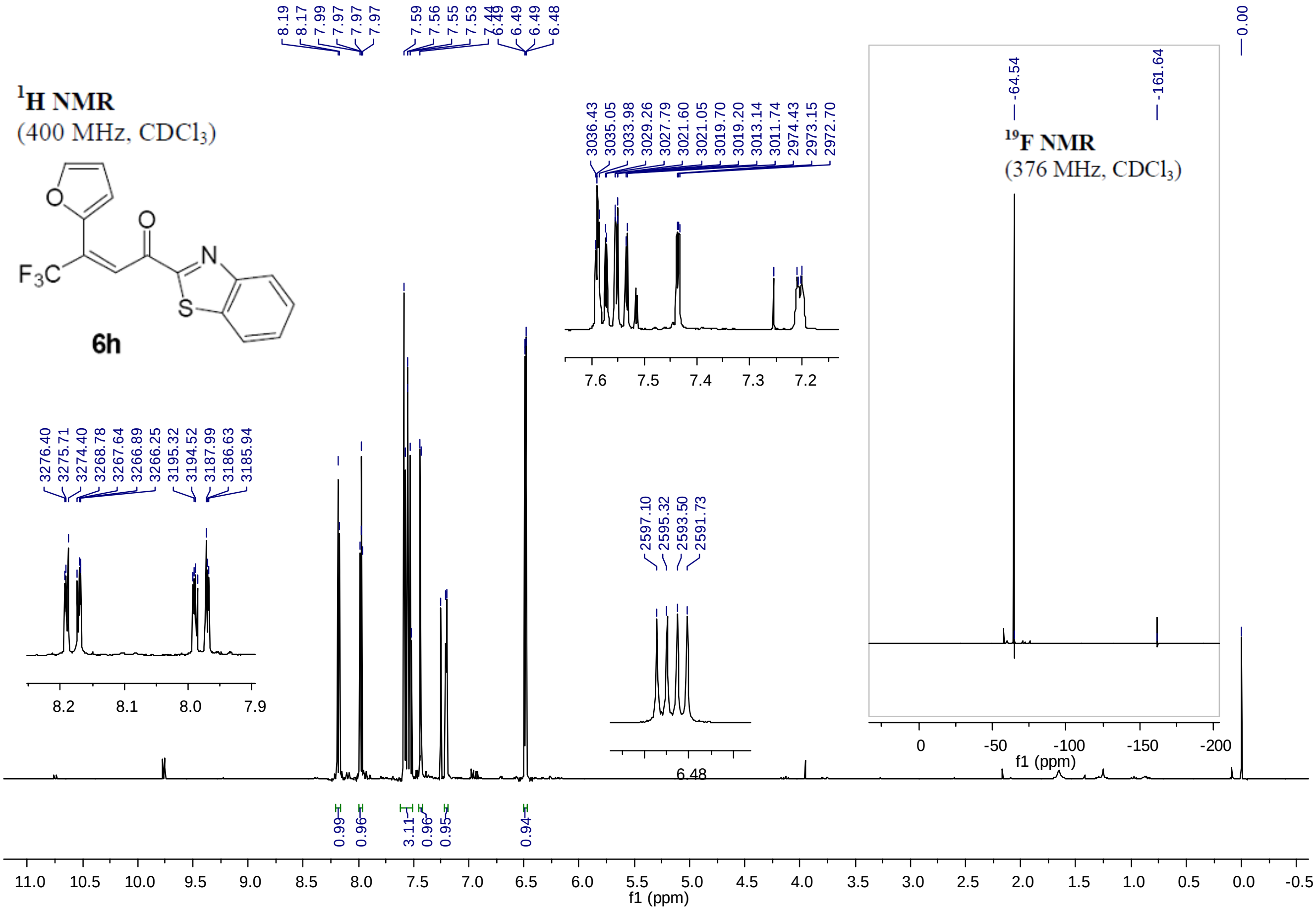

¹H NMR
(400 MHz, CDCl3)
6h
¹⁹F NMR
(376 MHz, CDCl3)
-64.54
-161.64
0.00
f1 (ppm)

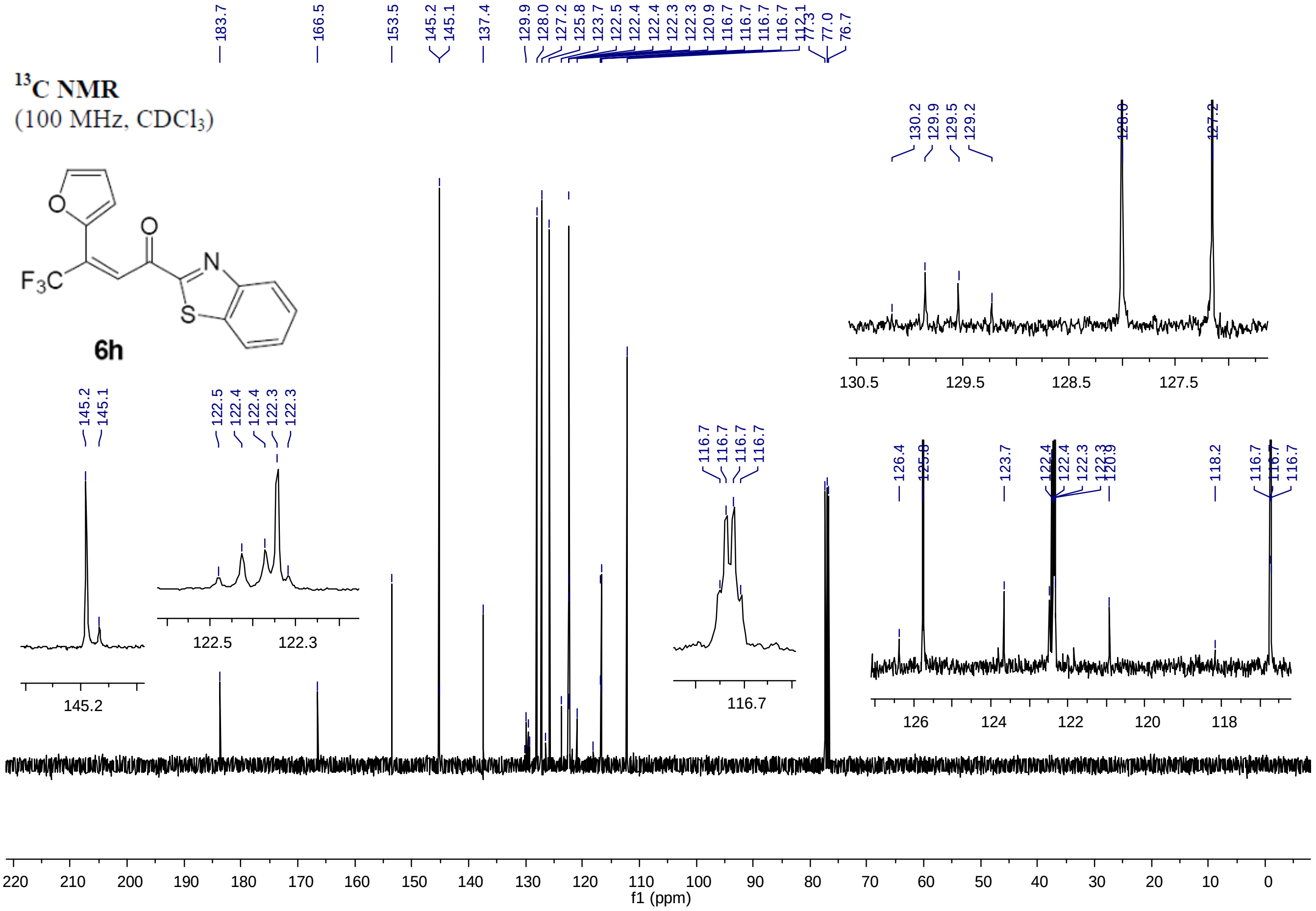

¹³C NMR
(100 MHz, CDCl3)
6h
183.7
166.5
153.5
145.2
145.1
137.4
77.3
77.0
76.7
f1 (ppm)

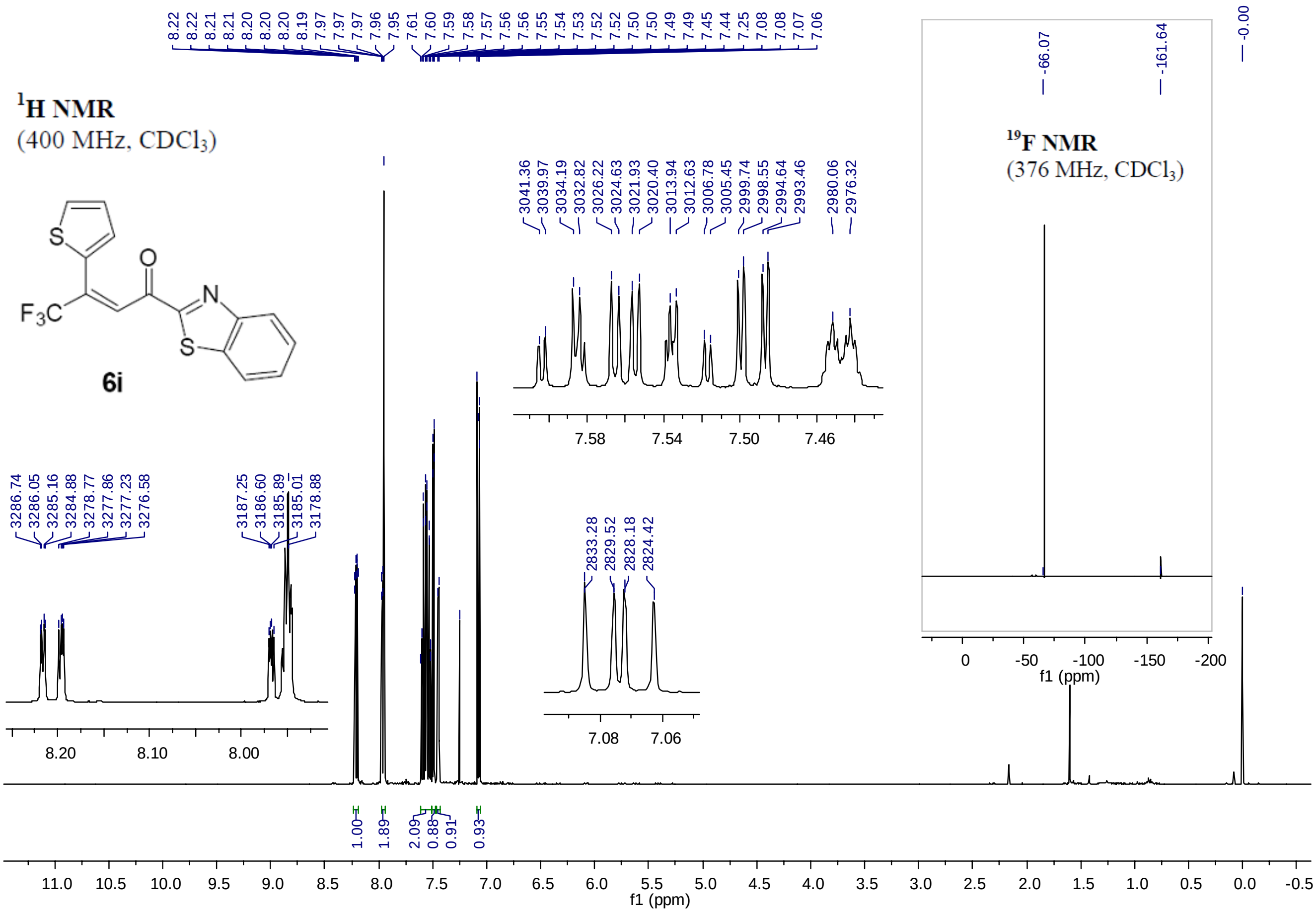

8.22 8.22 8.21 8.21 8.20 8.20 8.20 8.19 7.97 7.97 7.97 7.96 7.95 7.61 7.60 7.59 7.58 7.57 7.56 7.56 7.55 7.54 7.53 7.52 7.52 7.50 7.50 7.49 7.49 7.45 7.44 7.25 7.08 7.08 7.07 7.06
1H NMR
(400 MHz, CDCl3)
6i
3041.36 3039.97 3034.19 3032.82 3026.22 3024.63 3021.93 3020.40 3013.94 3012.63 3006.78 3005.45 2999.74 2998.55 2994.64 2993.46 2980.06 2976.32
7.58 7.54 7.50 7.46
3286.74 3286.05 3285.16 3284.88 3278.77 3277.86 3277.23 3276.58
3187.25 3186.60 3185.89 3185.01 3178.88
8.20 8.10 8.00
2833.28 2829.52 2828.18 2824.42
7.08 7.06
-66.07
-161.64
-0.00
19F NMR
(376 MHz, CDCl3)
0 -50 -100 -150 -200
f1 (ppm)
1.00 1.89 2.09 0.88 0.91 0.93
11.0 10.5 10.0 9.5 9.0 8.5 8.0 7.5 7.0 6.5 6.0 5.5 5.0 4.5 4.0 3.5 3.0 2.5 2.0 1.5 1.0 0.5 0.0 -0.5
f1 (ppm)

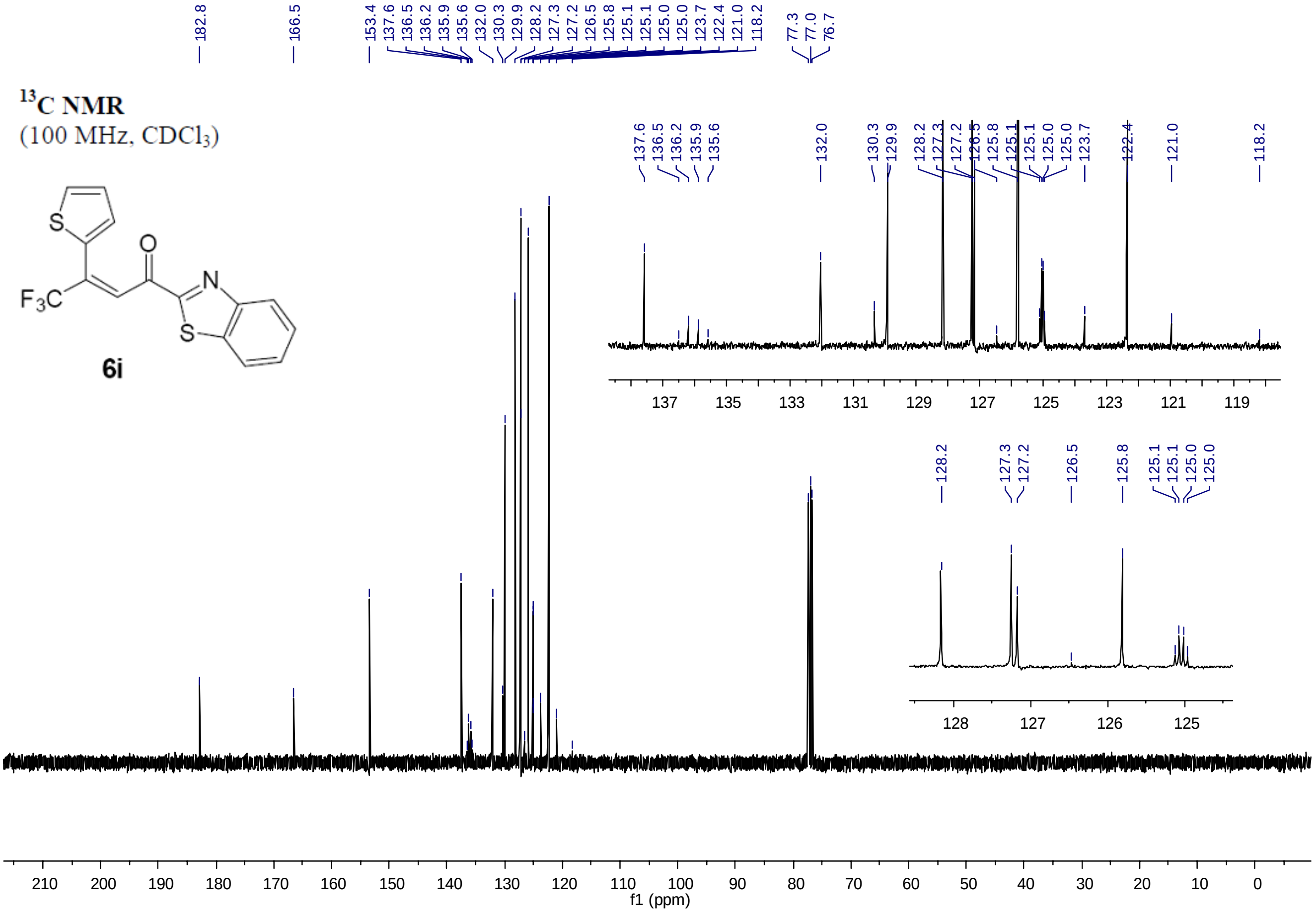

182.8
166.5
153.4 137.6 136.5 136.2 135.9 135.6 132.0 130.3 129.9 128.2 127.3 127.2 126.5 125.8 125.1 125.1 125.0 125.0 123.7 122.4 121.0 118.2
77.3 77.0 76.7
13C NMR
(100 MHz, CDCl3)
6i
137.6 136.5 136.2 135.9 135.6 132.0 130.3 129.9 128.2 127.3 127.2 126.5 125.8 125.1 125.1 125.0 125.0 123.7 122.4 121.0 118.2
137 135 133 131 129 127 125 123 121 119
128.2 127.3 127.2 126.5 125.8 125.1 125.1 125.0 125.0
128 127 126 125
210 200 190 180 170 160 150 140 130 120 110 100 90 80 70 60 50 40 30 20 10 0
f1 (ppm)

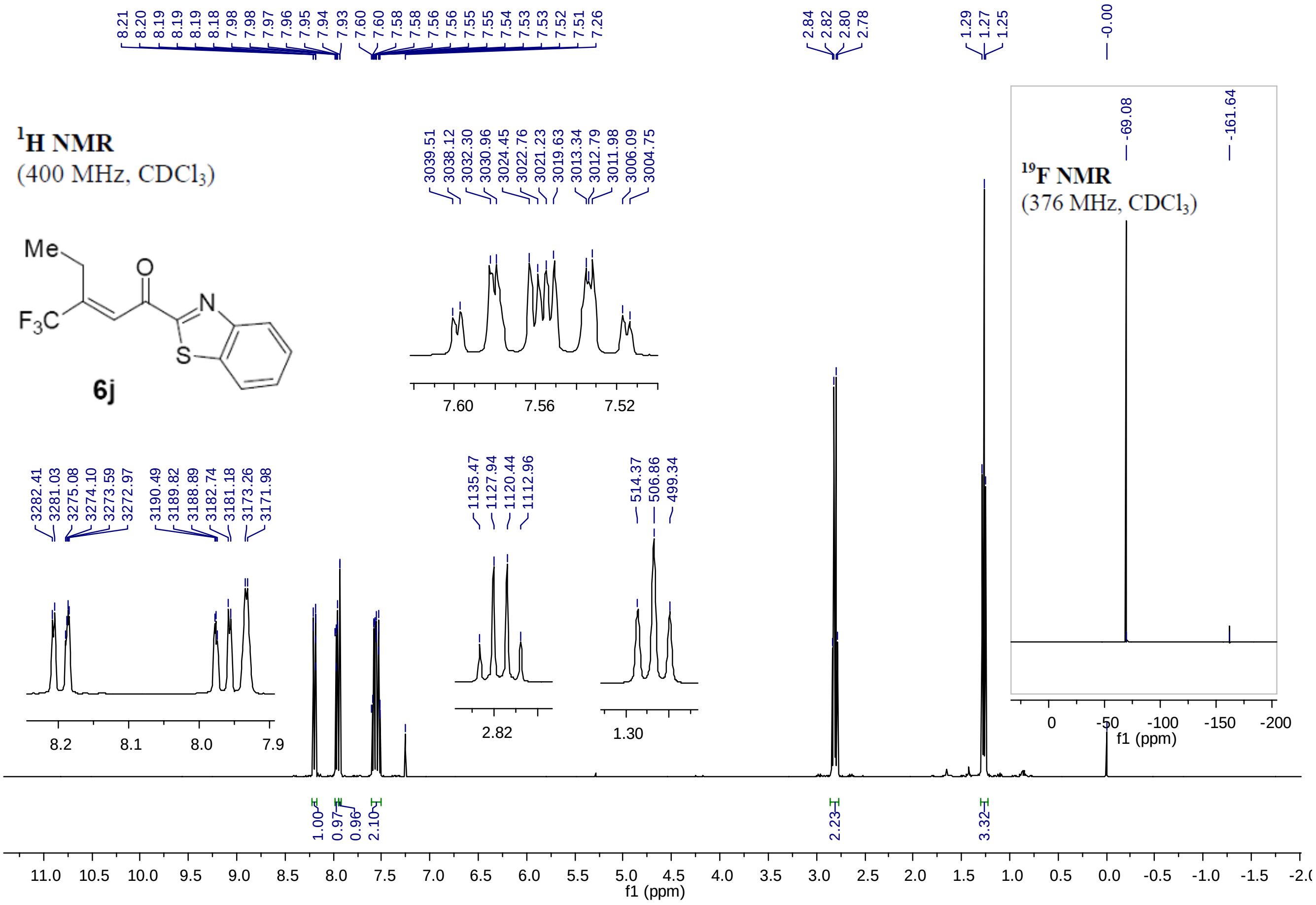

1H NMR
(400 MHz, CDCl3)
Me
F3C
O
N
S
6j
19F NMR
(376 MHz, CDCl3)
-69.08
-161.64
f1 (ppm)

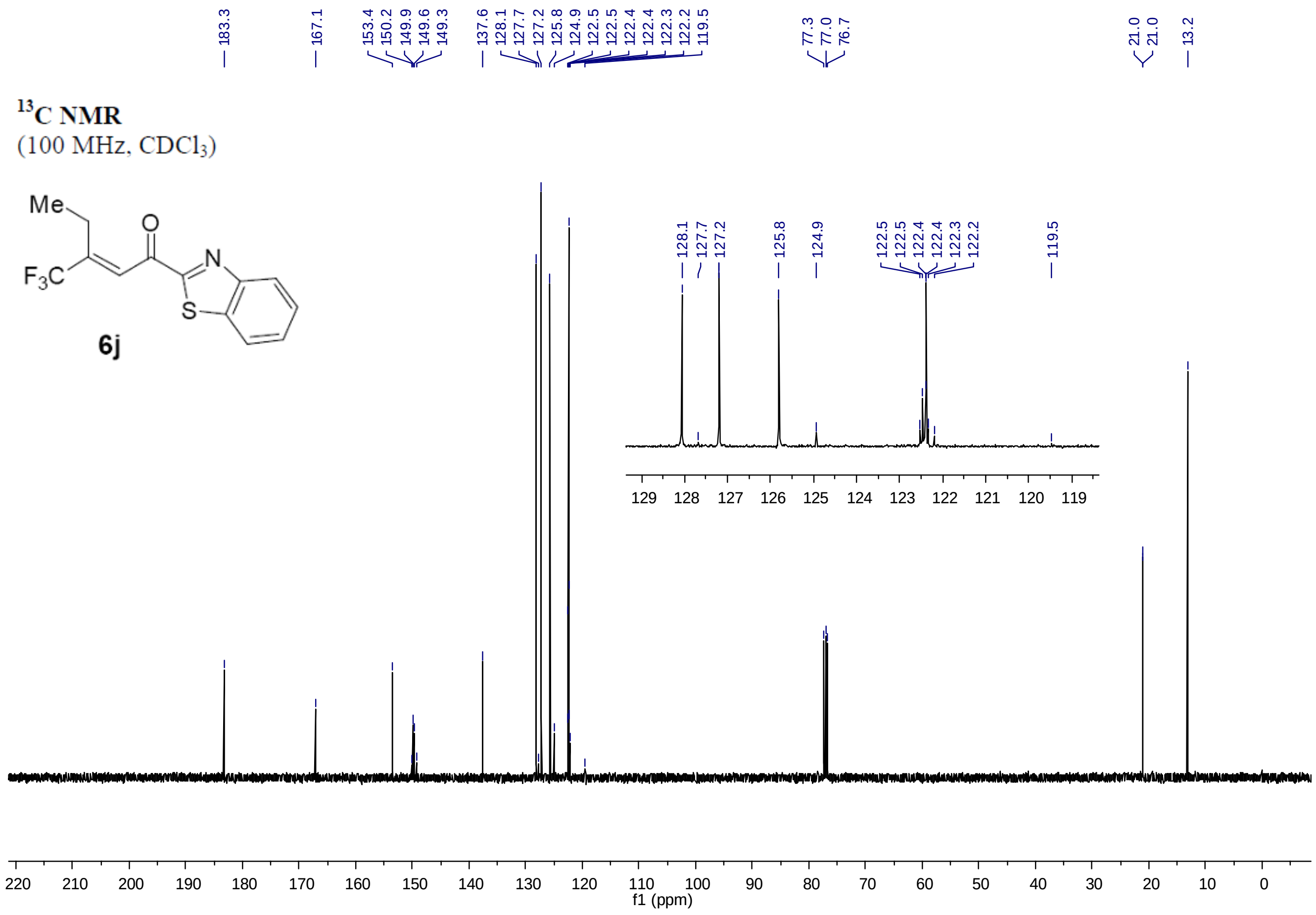

13C NMR
(100 MHz, CDCl3)
Me
F3C
O
N
S
6j
183.3
167.1
153.4
150.2
149.9
149.6
149.3
137.6
128.1
127.7
127.2
125.8
124.9
122.5
122.5
122.4
122.4
122.3
122.2
119.5
77.3
77.0
76.7
21.0
21.0
13.2
f1 (ppm)

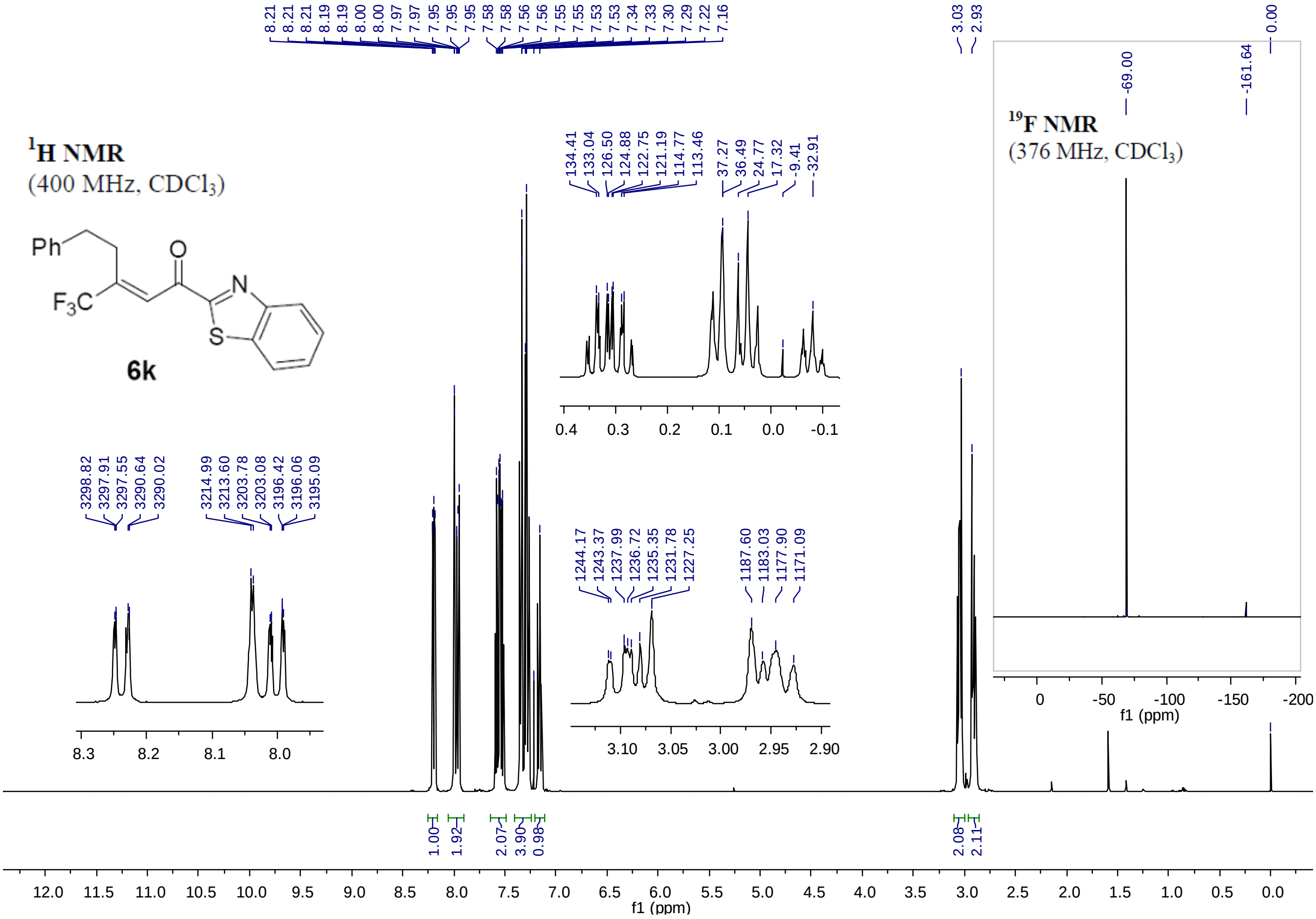
1H NMR
(400 MHz, CDCl3)
6k
19F NMR
(376 MHz, CDCl3)
f1 (ppm)

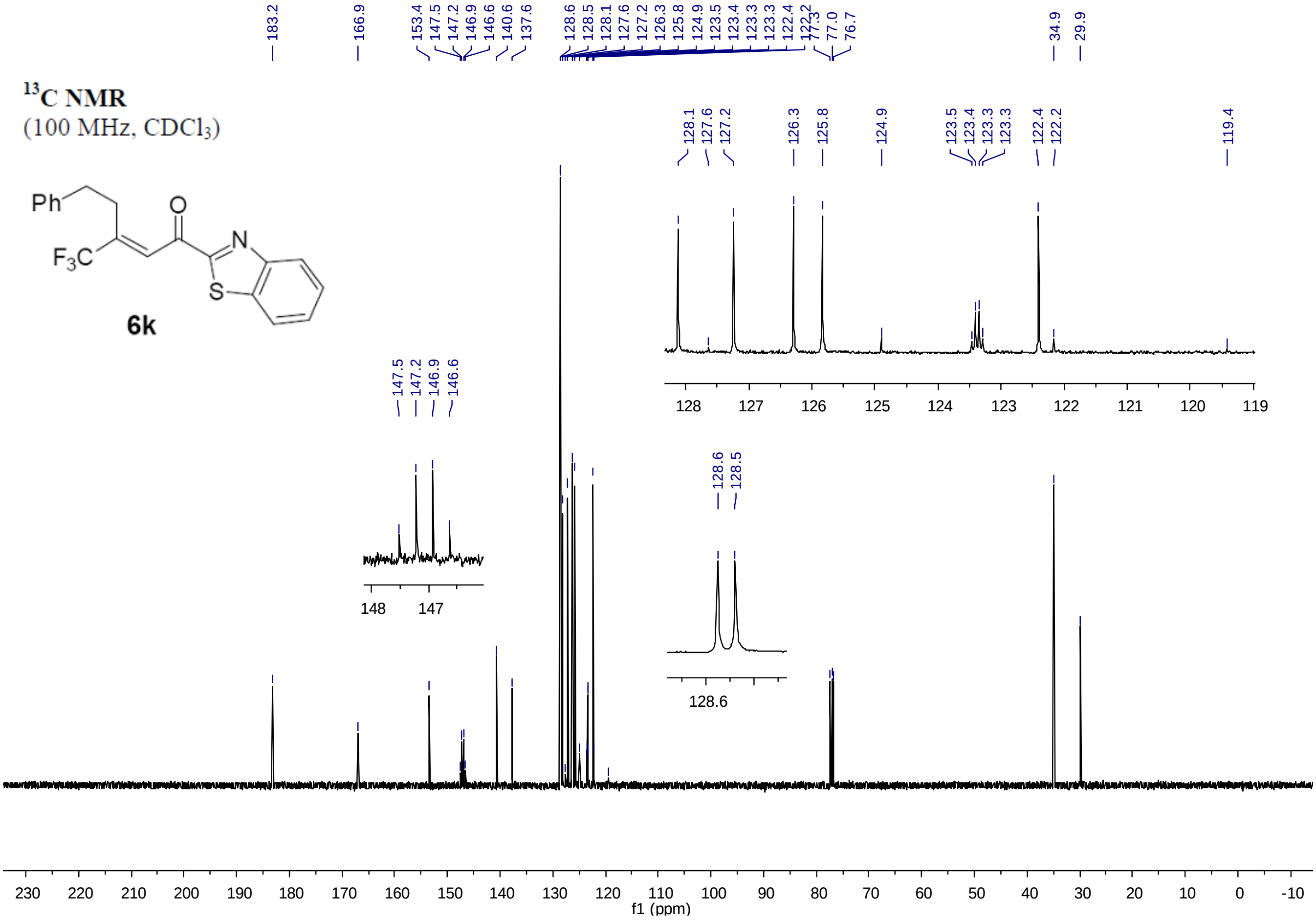
13C NMR
(100 MHz, CDCl3)
6k
f1 (ppm)

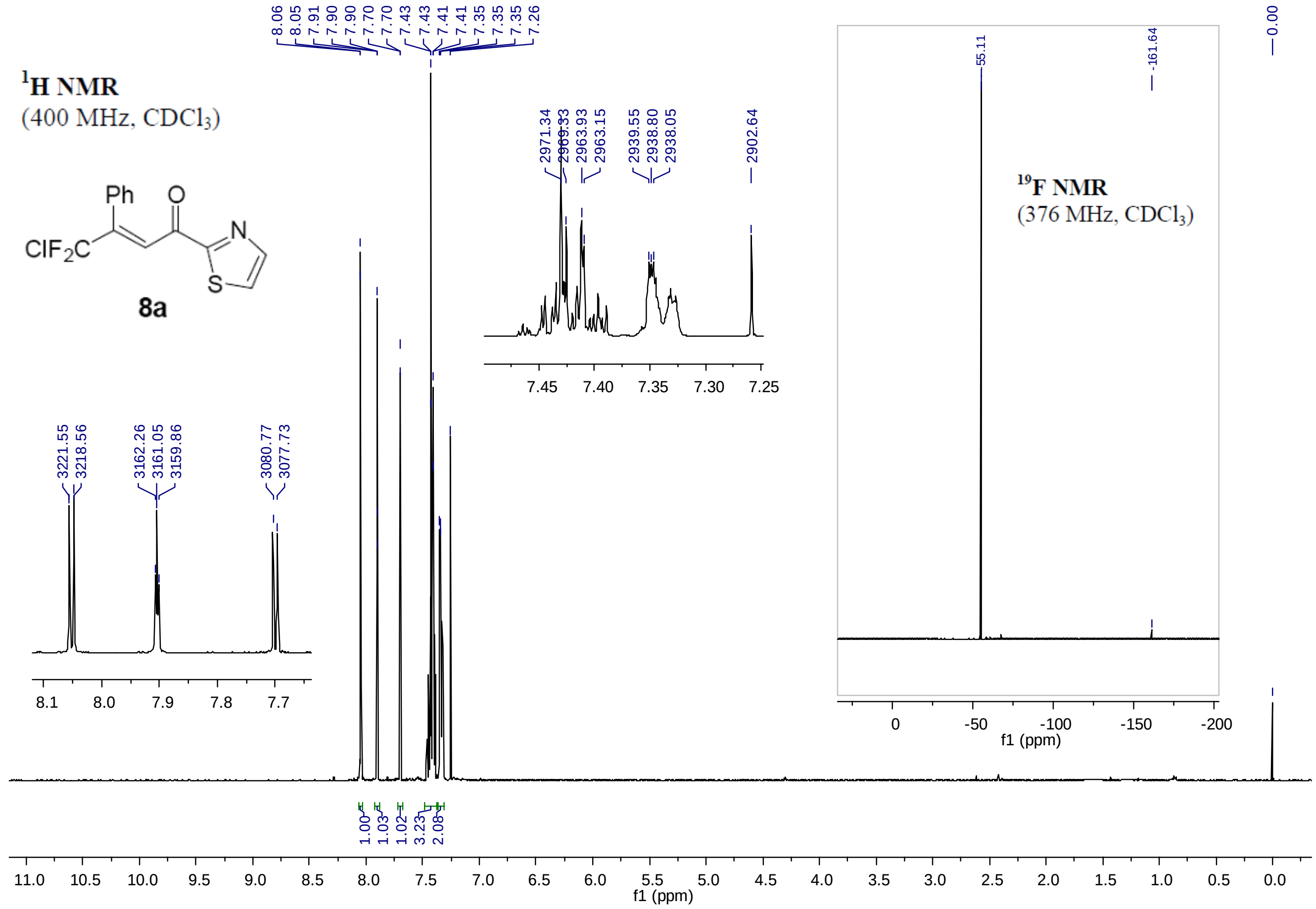
1H NMR
(400 MHz, CDCl3)
Ph
O
N
ClF2C
S
8a
8.06 8.05 7.91 7.90 7.90 7.70 7.70 7.43 7.43 7.41 7.41 7.35 7.35 7.35 7.26
3221.55 3218.56
3162.26 3161.05 3159.86
3080.77 3077.73
2971.34 2969.33 2963.93 2963.15 2939.55 2938.80 2938.05 2902.64
19F NMR
(376 MHz, CDCl3)
-55.11
-161.64
0.00
1.00 1.03 1.02 3.23 2.08
f1 (ppm)

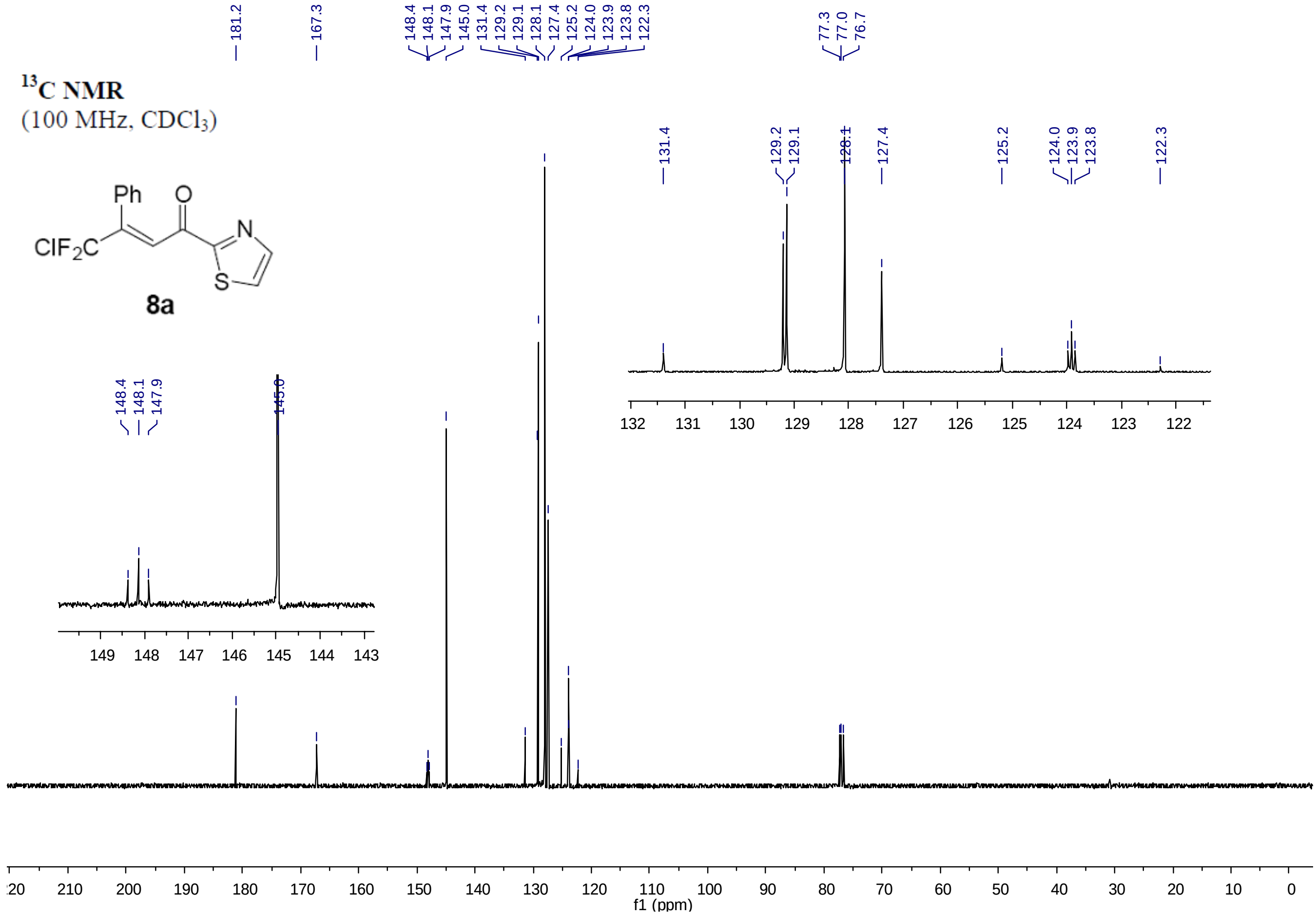
13C NMR
(100 MHz, CDCl3)
Ph
O
N
ClF2C
S
8a
181.2
167.3
148.4 148.1 147.9 145.0 131.4 129.2 129.1 128.1 127.4 125.2 124.0 123.9 123.8 122.3
77.3 77.0 76.7
f1 (ppm)

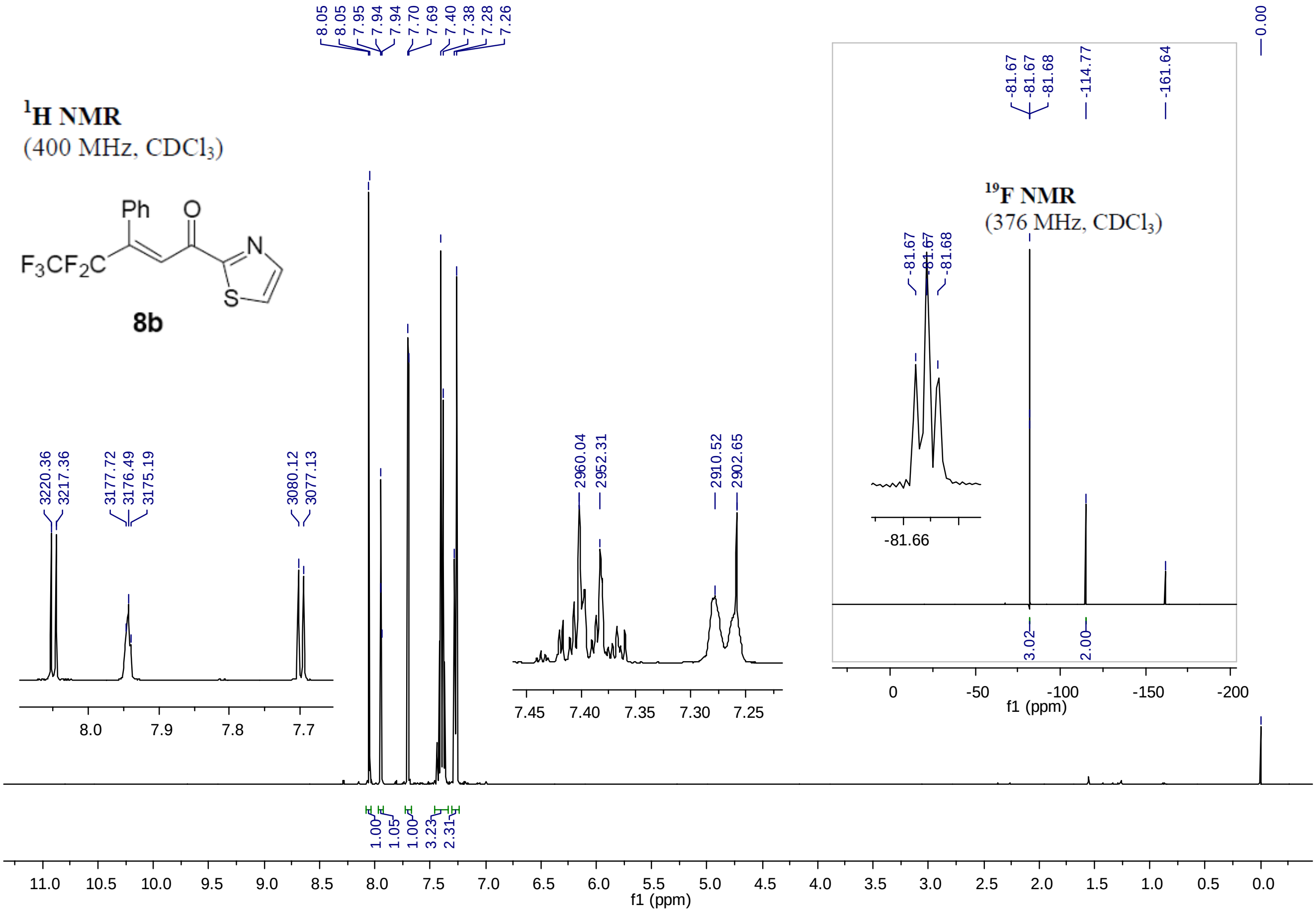

^{1}H NMR
(400 MHz, $CDCl_3$)
^{19}F NMR
(376 MHz, $CDCl_3$)
Ph
O
F_3CF_2C
N
S
8b
f1 (ppm)

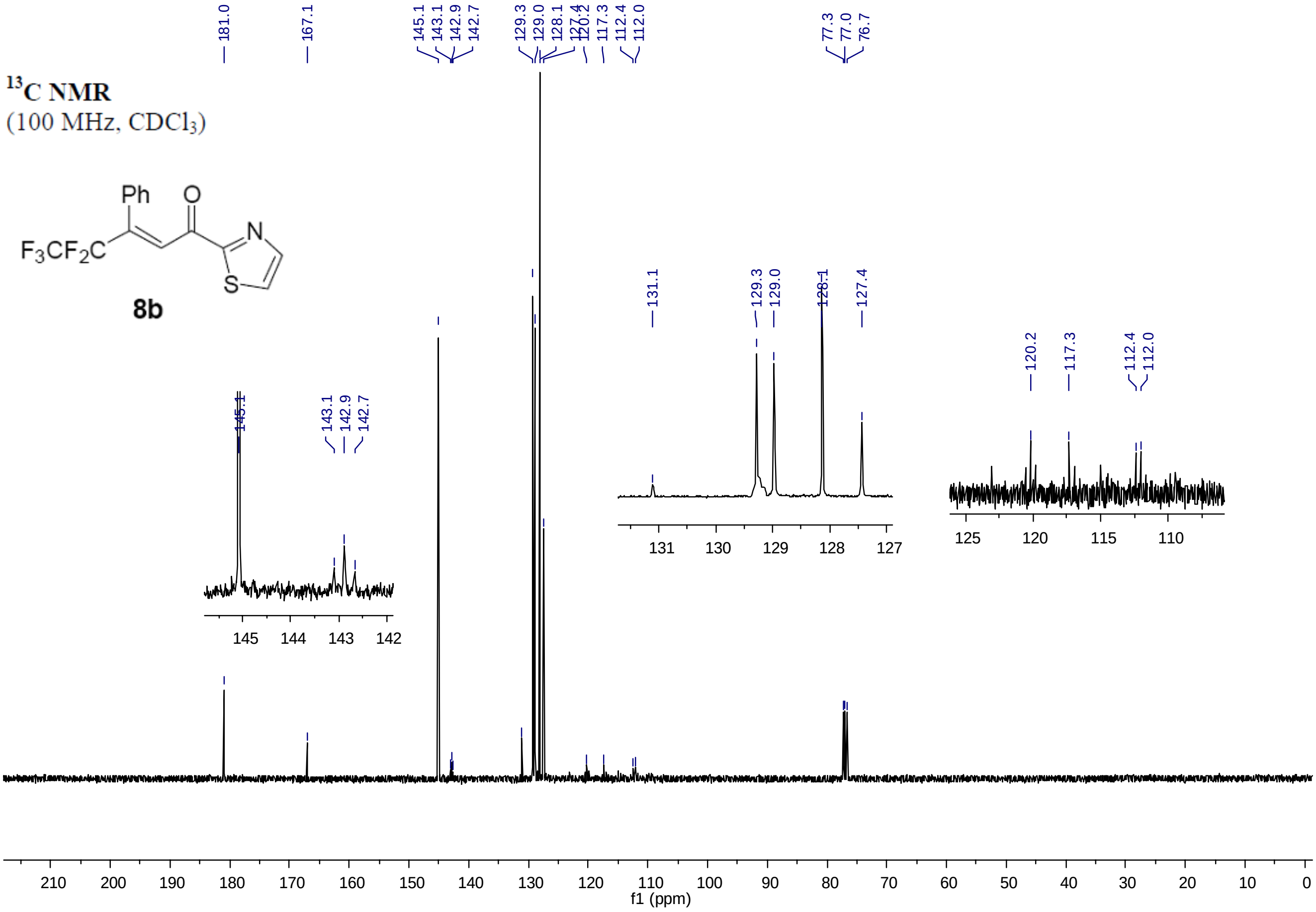

^{13}C NMR
(100 MHz, $CDCl_3$)
Ph
O
F_3CF_2C
N
S
8b
f1 (ppm)

Copies of NMR spectra - products of malonate addition to enones: 3a-3l, 5a-5k, 5m-5u, 7a-7k, 9a, 9b

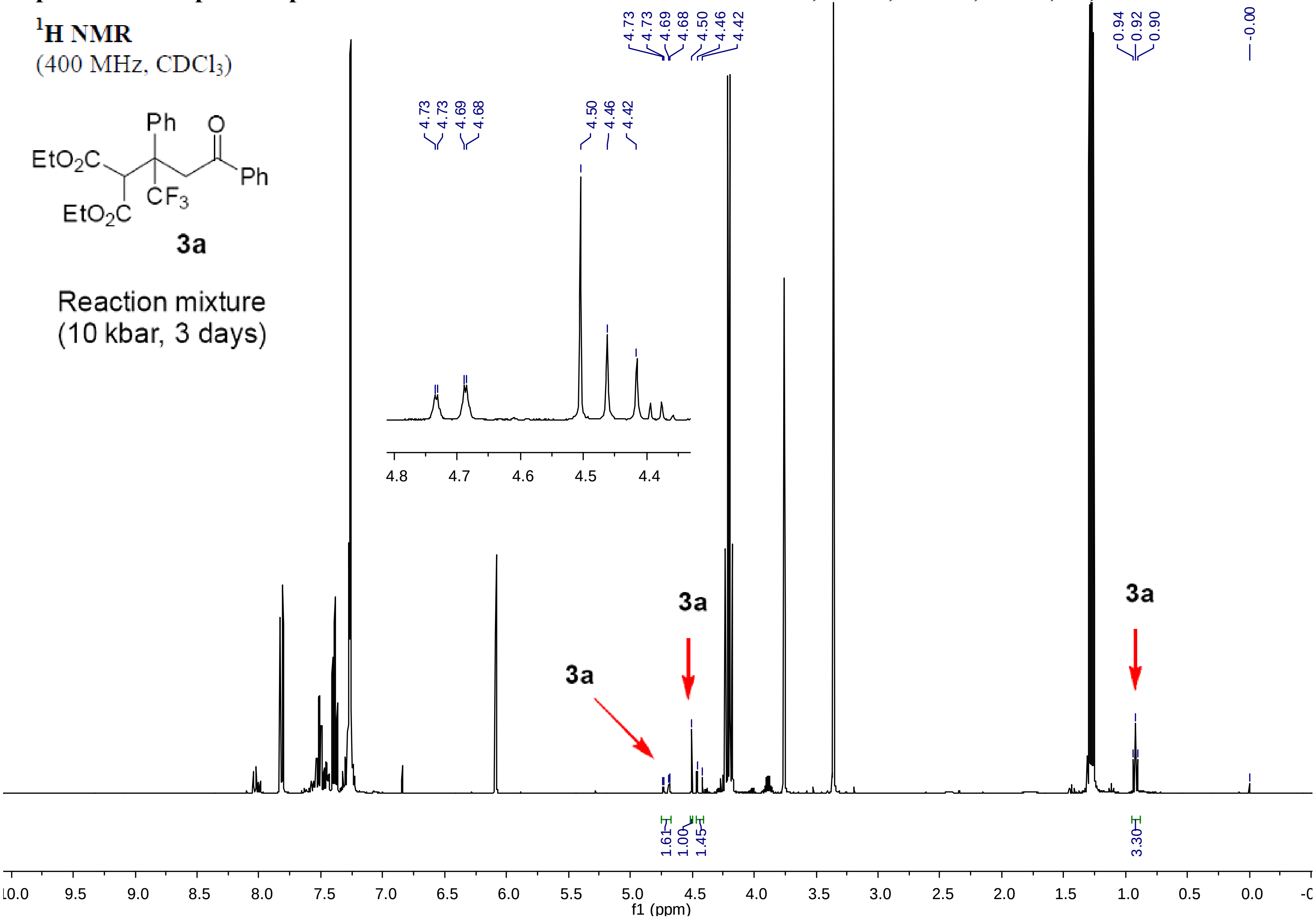

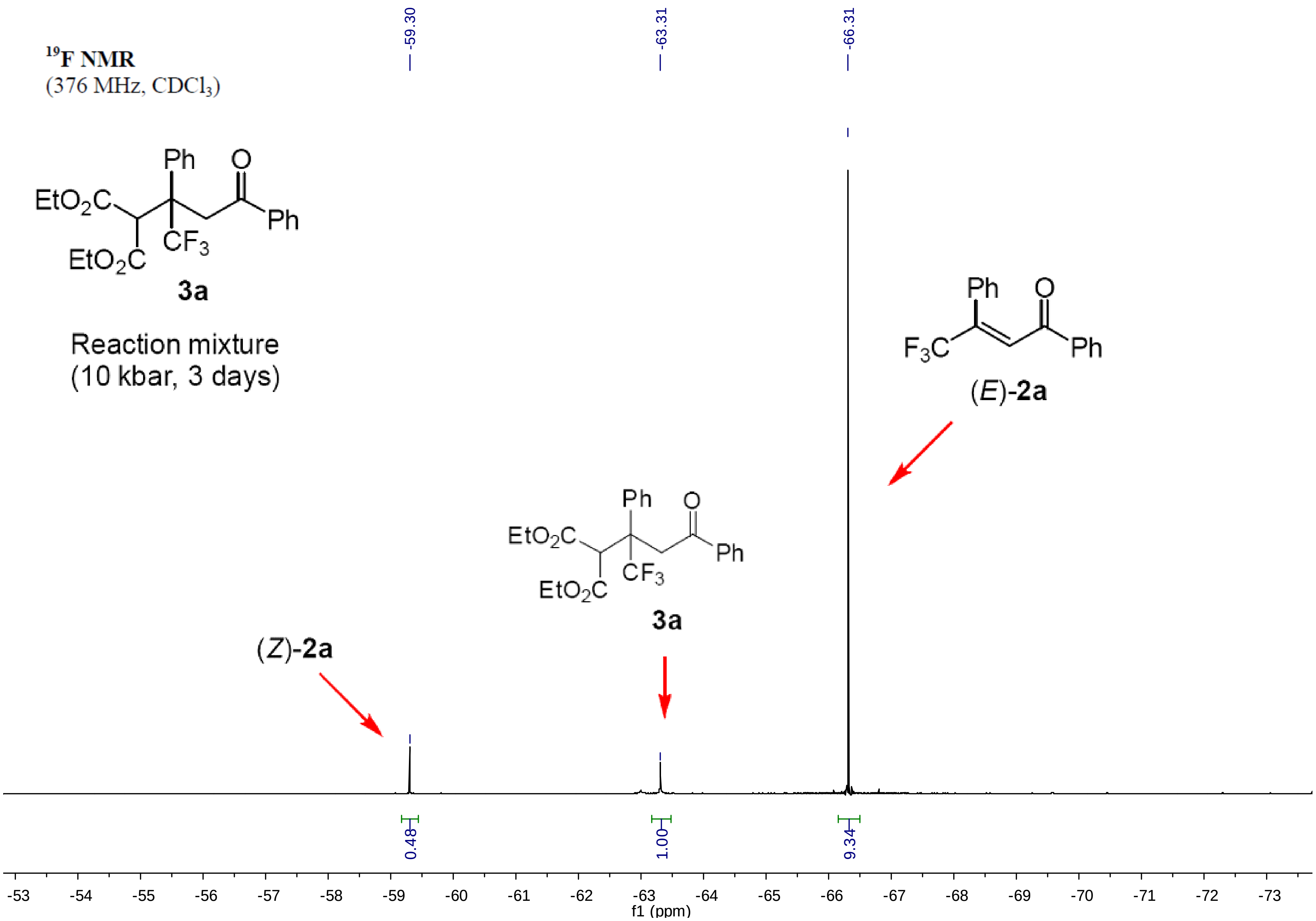

19F NMR
(376 MHz, CDCl3)
-59.30
-63.31
-66.31
3a
Reaction mixture
(10 kbar, 3 days)
(E)-2a
3a
(Z)-2a
0.48
1.00
9.34
-53 -54 -55 -56 -57 -58 -59 -60 -61 -62 -63 -64 -65 -66 -67 -68 -69 -70 -71 -72 -73
f1 (ppm)

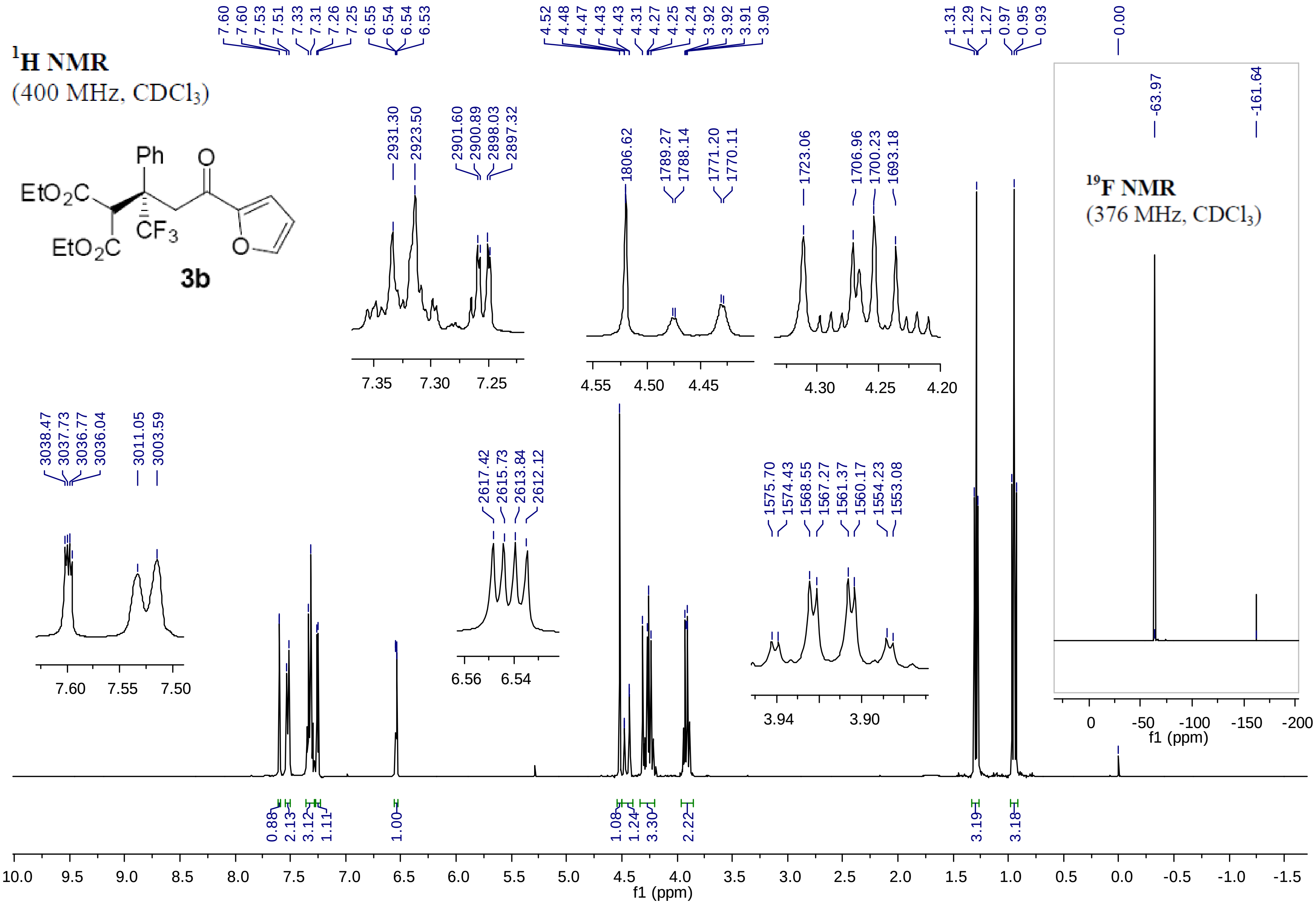
1H NMR
(400 MHz, CDCl3)
Ph
O
EtO2C
CF3
EtO2C
O
3b
19F NMR
(376 MHz, CDCl3)
-63.97
-161.64
f1 (ppm)

^{13}C NMR
(100 MHz, $CDCl_3$)

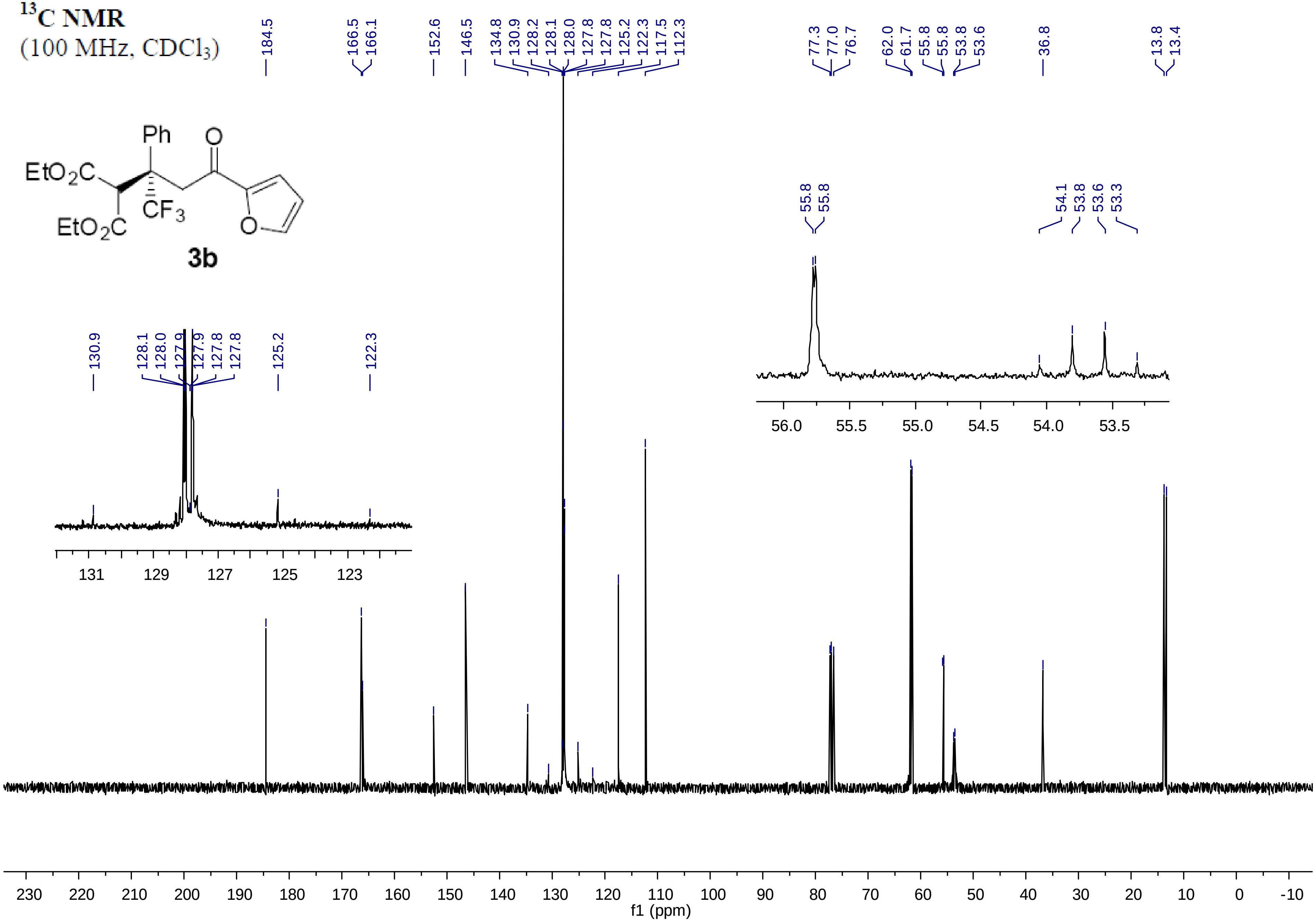

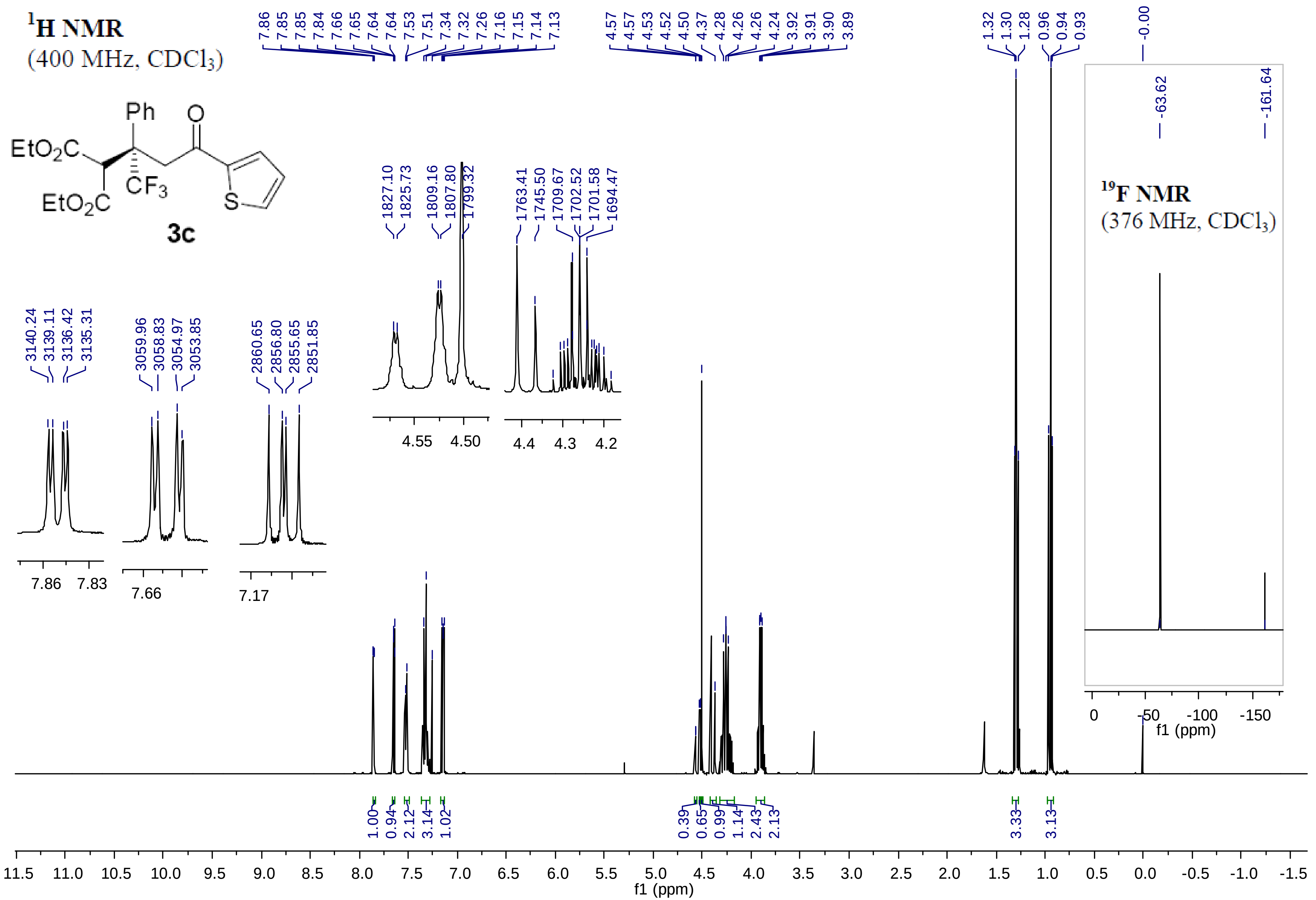

1H NMR
(400 MHz, CDCl3)
EtO2C
EtO2C
Ph
O
CF3
S
3c
19F NMR
(376 MHz, CDCl3)
f1 (ppm)

^{13}C NMR (100 MHz, $CDCl_3$)

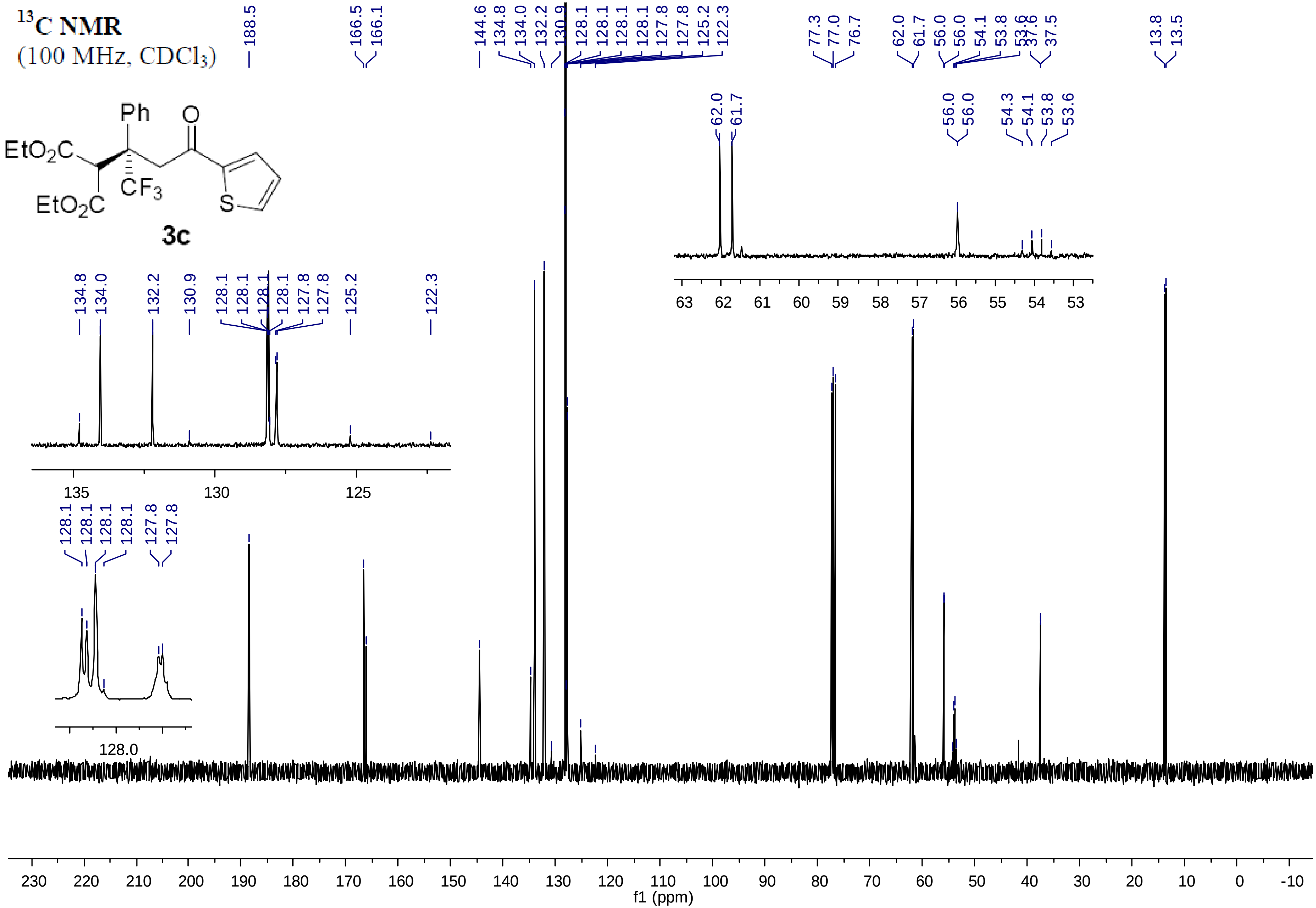

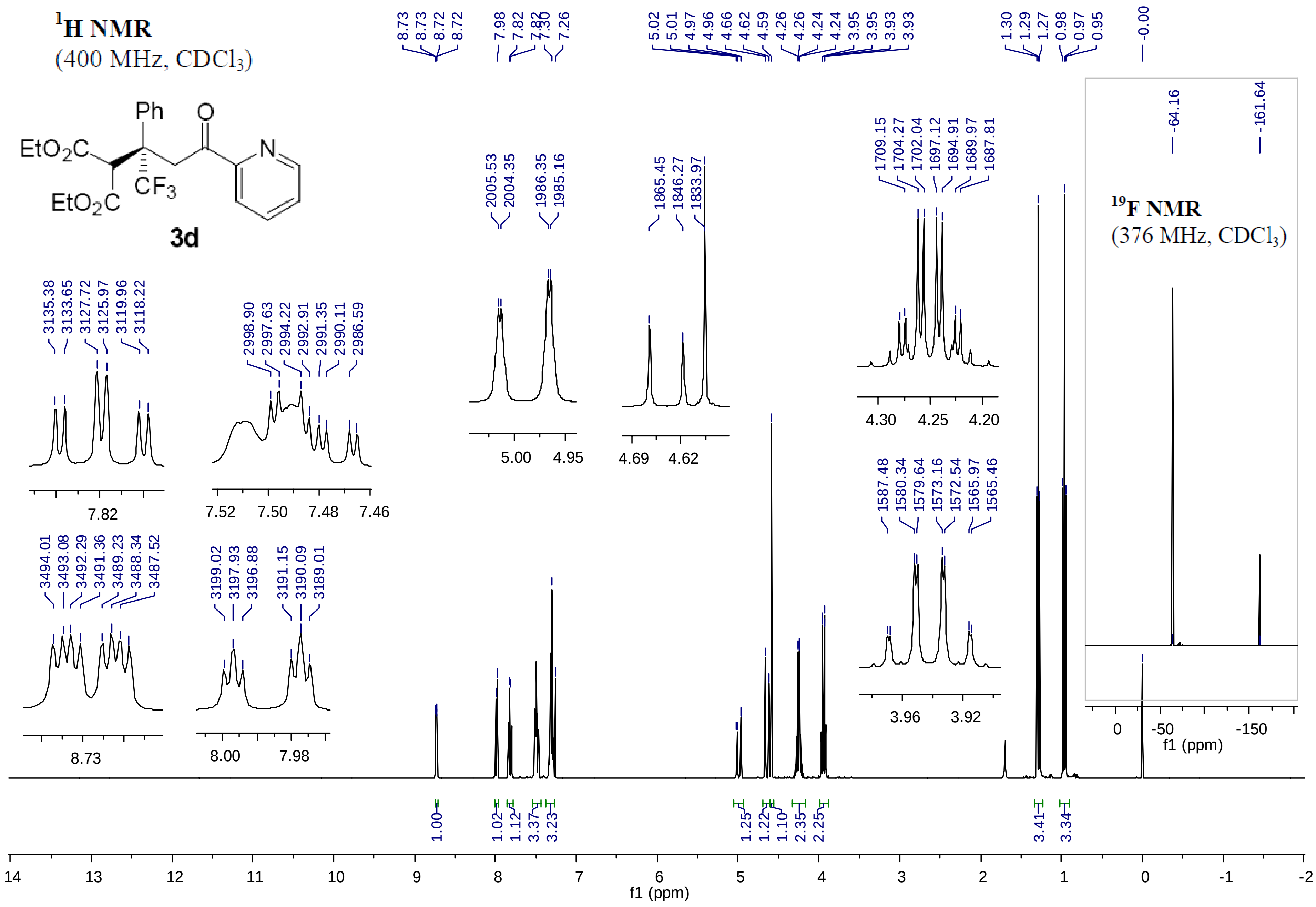

1H NMR
(400 MHz, CDCl3)
3d
19F NMR
(376 MHz, CDCl3)
f1 (ppm)

^{13}C NMR
(100 MHz, $CDCl_3$)

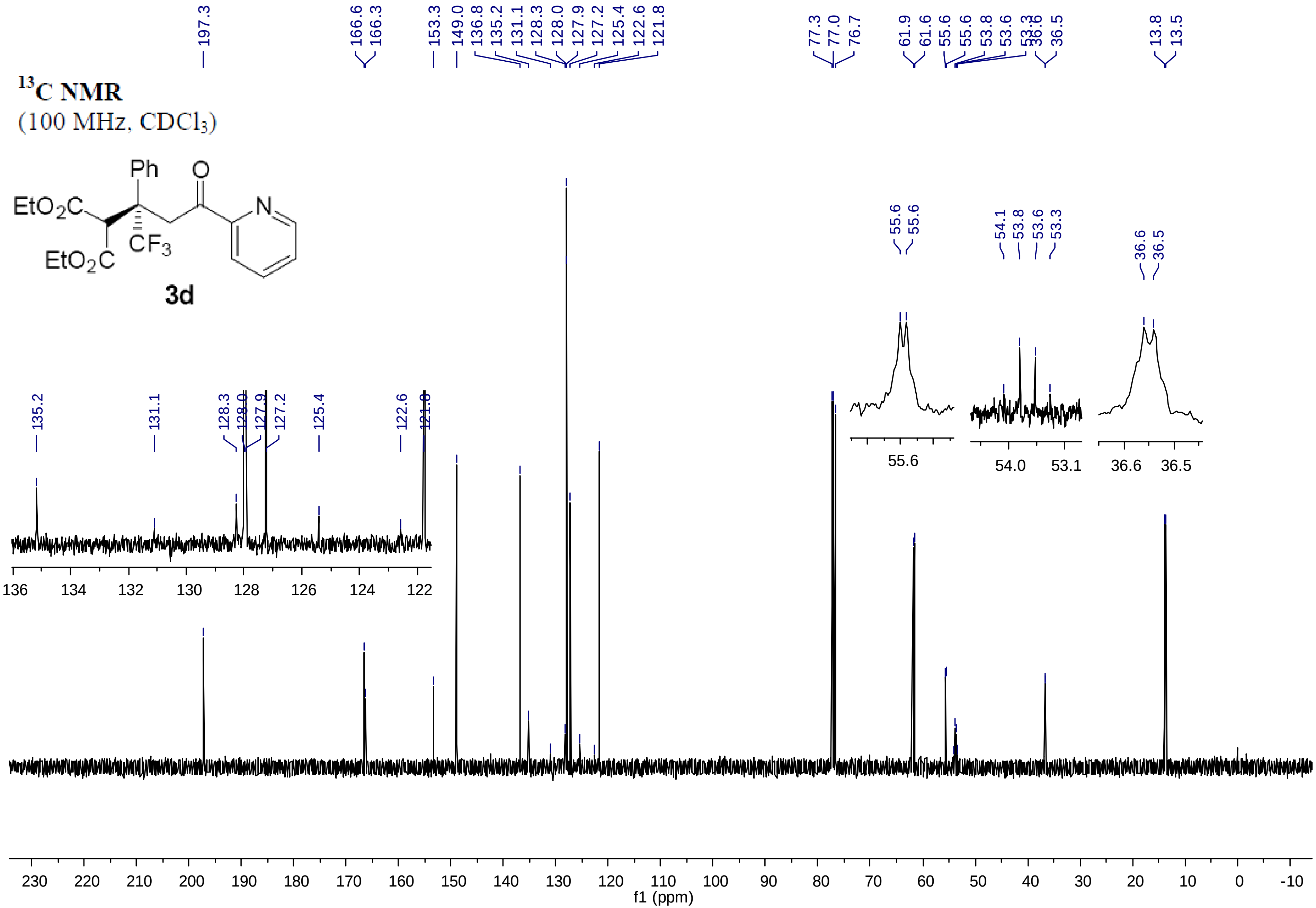

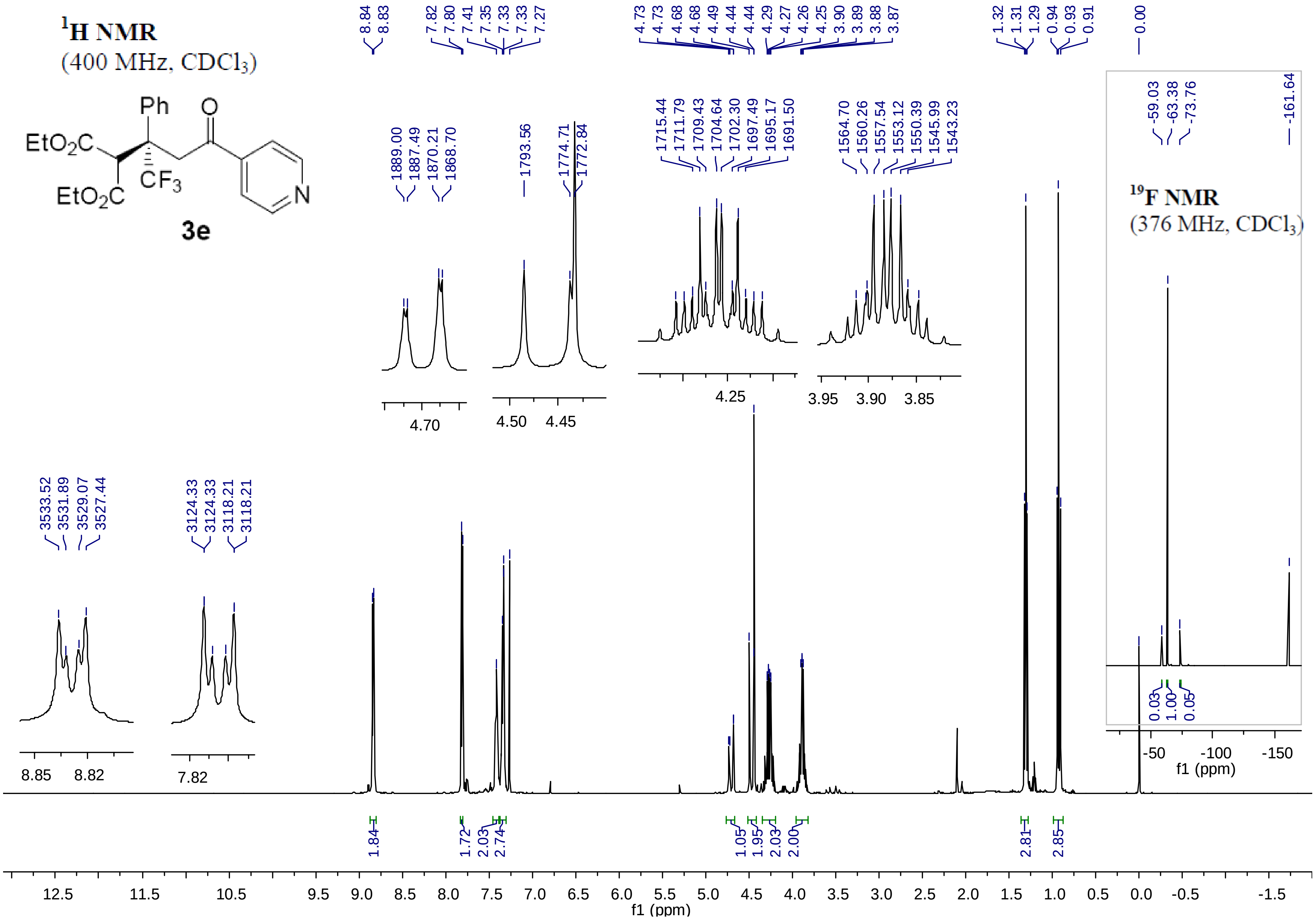

1H NMR
(400 MHz, CDCl3)
3e
19F NMR
(376 MHz, CDCl3)
f1 (ppm)

^{13}C NMR
(100 MHz, CDCl$_3$)

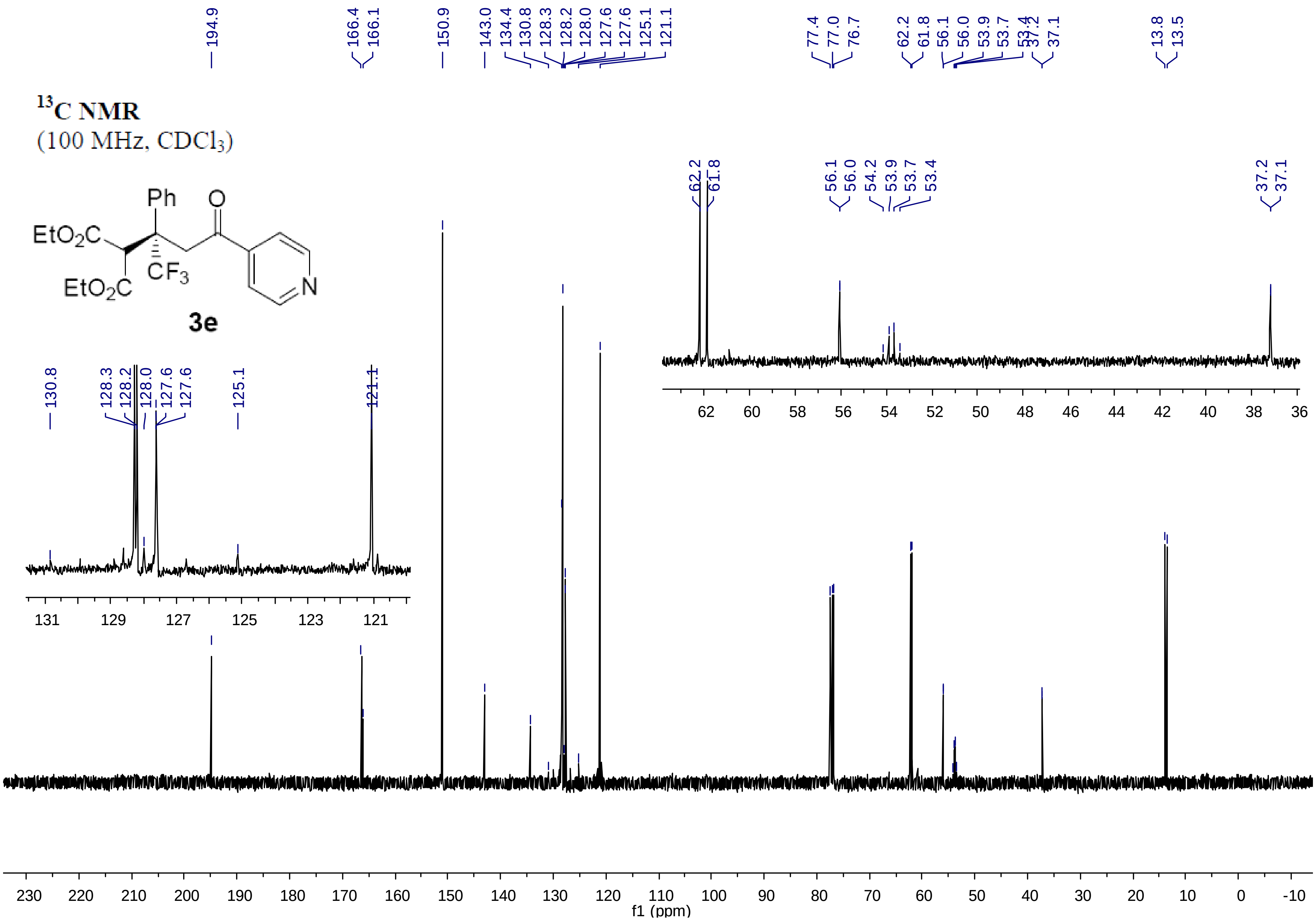

3e

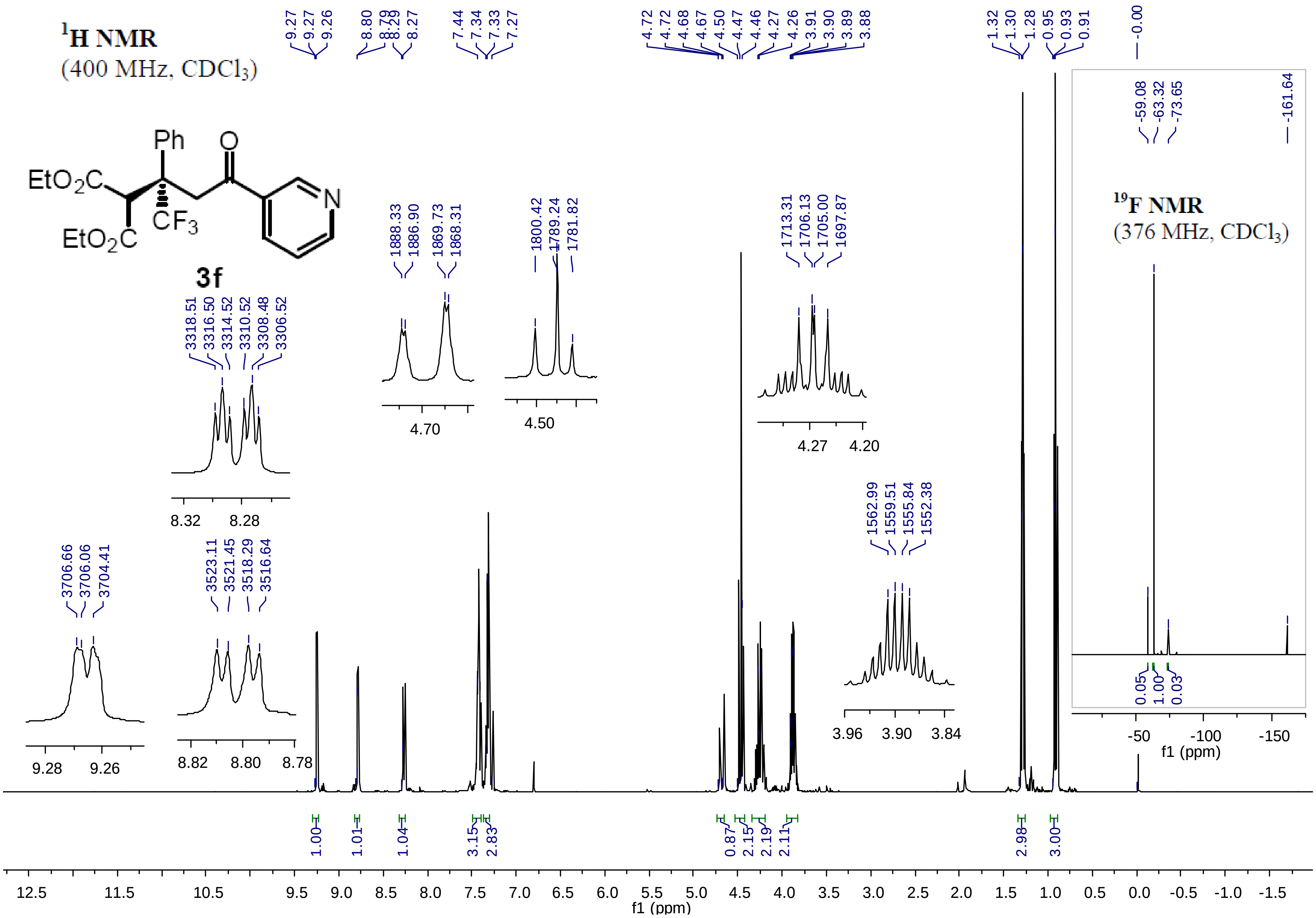
1H NMR
(400 MHz, CDCl3)
3f
19F NMR
(376 MHz, CDCl3)
f1 (ppm)

^{13}C NMR

(100 MHz, $CDCl_3$)

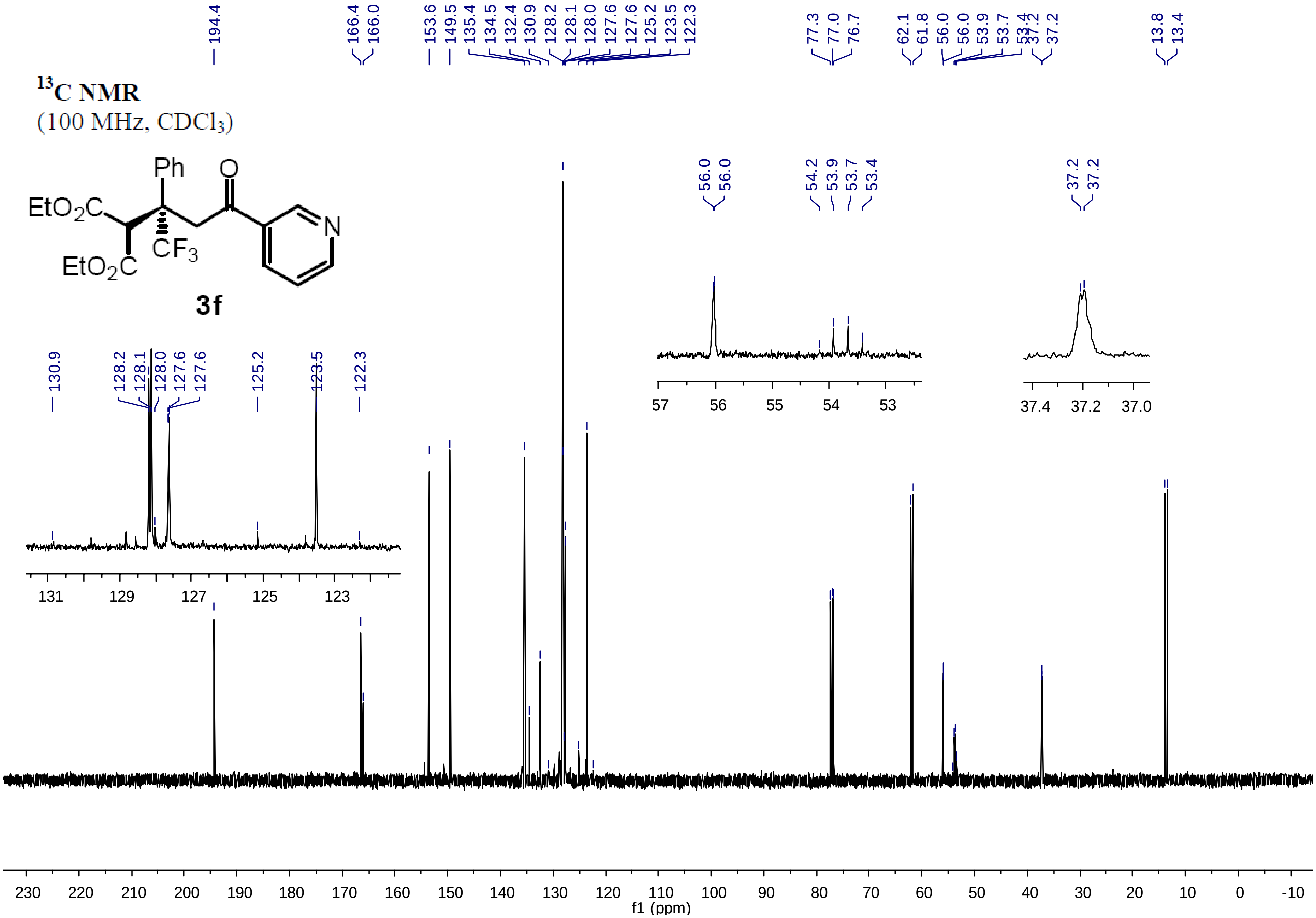

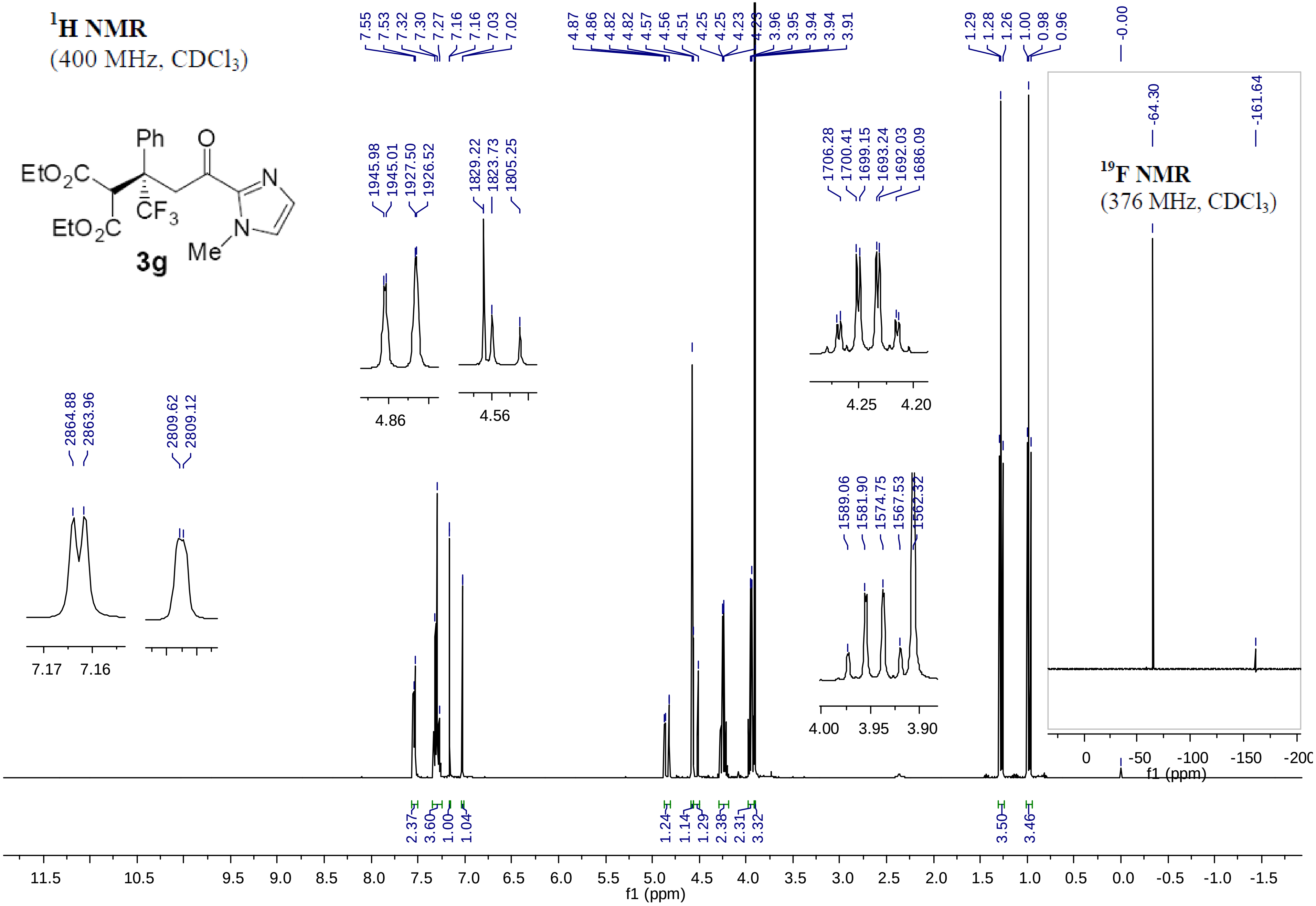

^{1}H NMR
(400 MHz, CDCl$_3$)
3g
^{19}F NMR
(376 MHz, CDCl$_3$)
f1 (ppm)

^{13}C NMR
(100 MHz, $CDCl_3$)

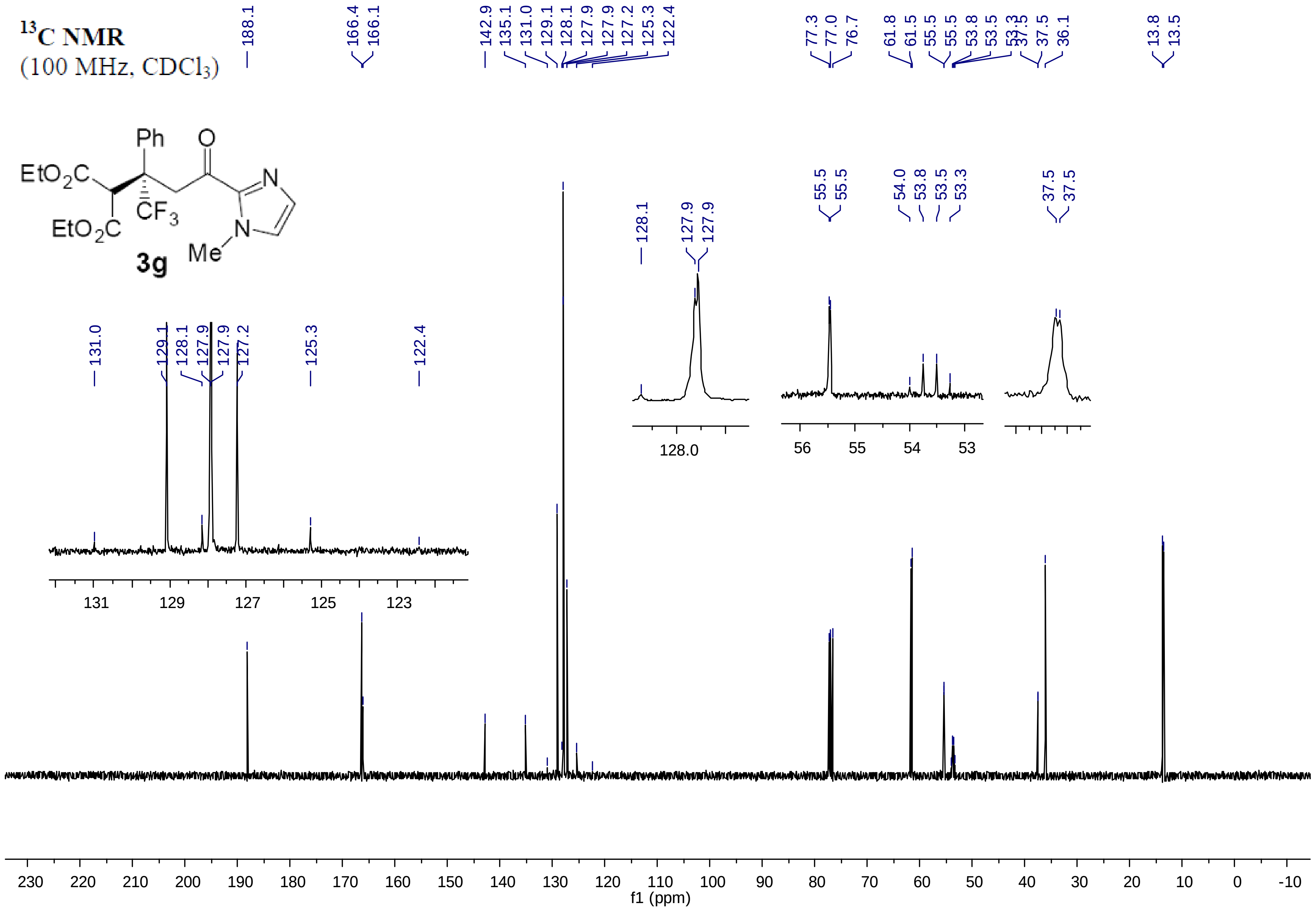

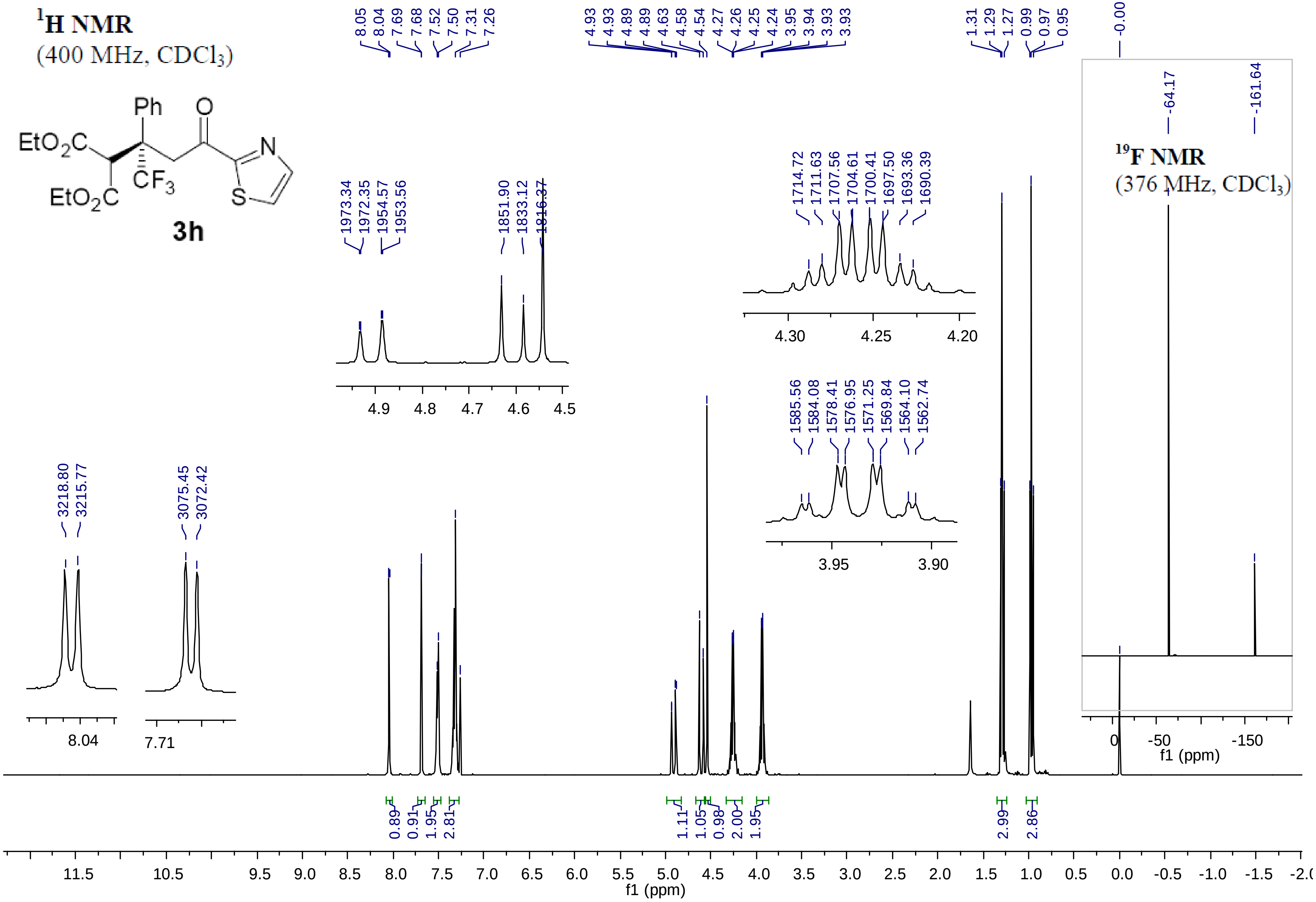

1H NMR
(400 MHz, CDCl3)
EtO2C
EtO2C
Ph
O
CF3
N
S
3h
19F NMR
(376 MHz, CDCl3)
-64.17
-161.64
f1 (ppm)

^{13}C NMR
(100 MHz, $CDCl_3$)

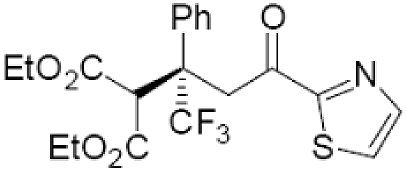

3h

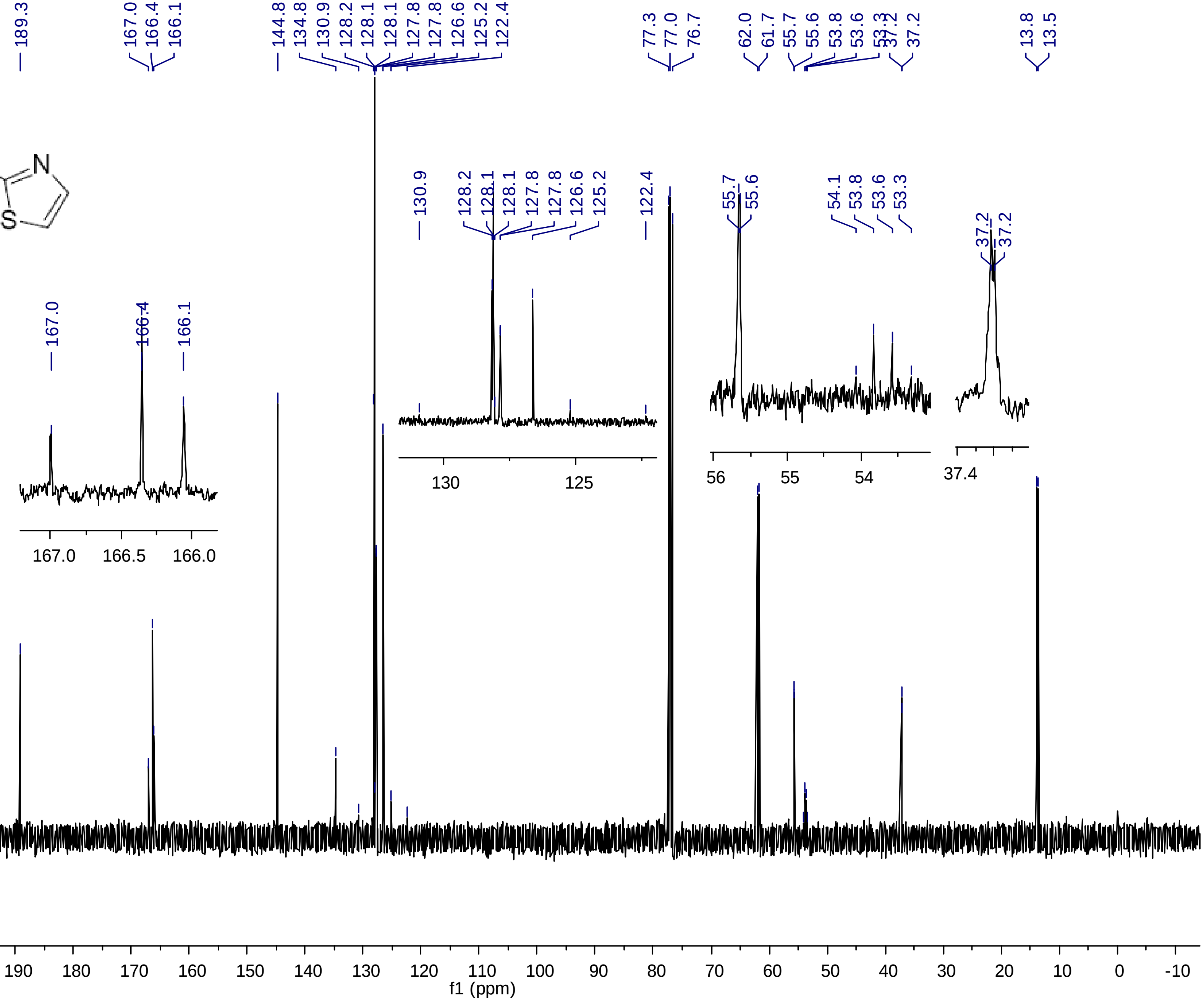

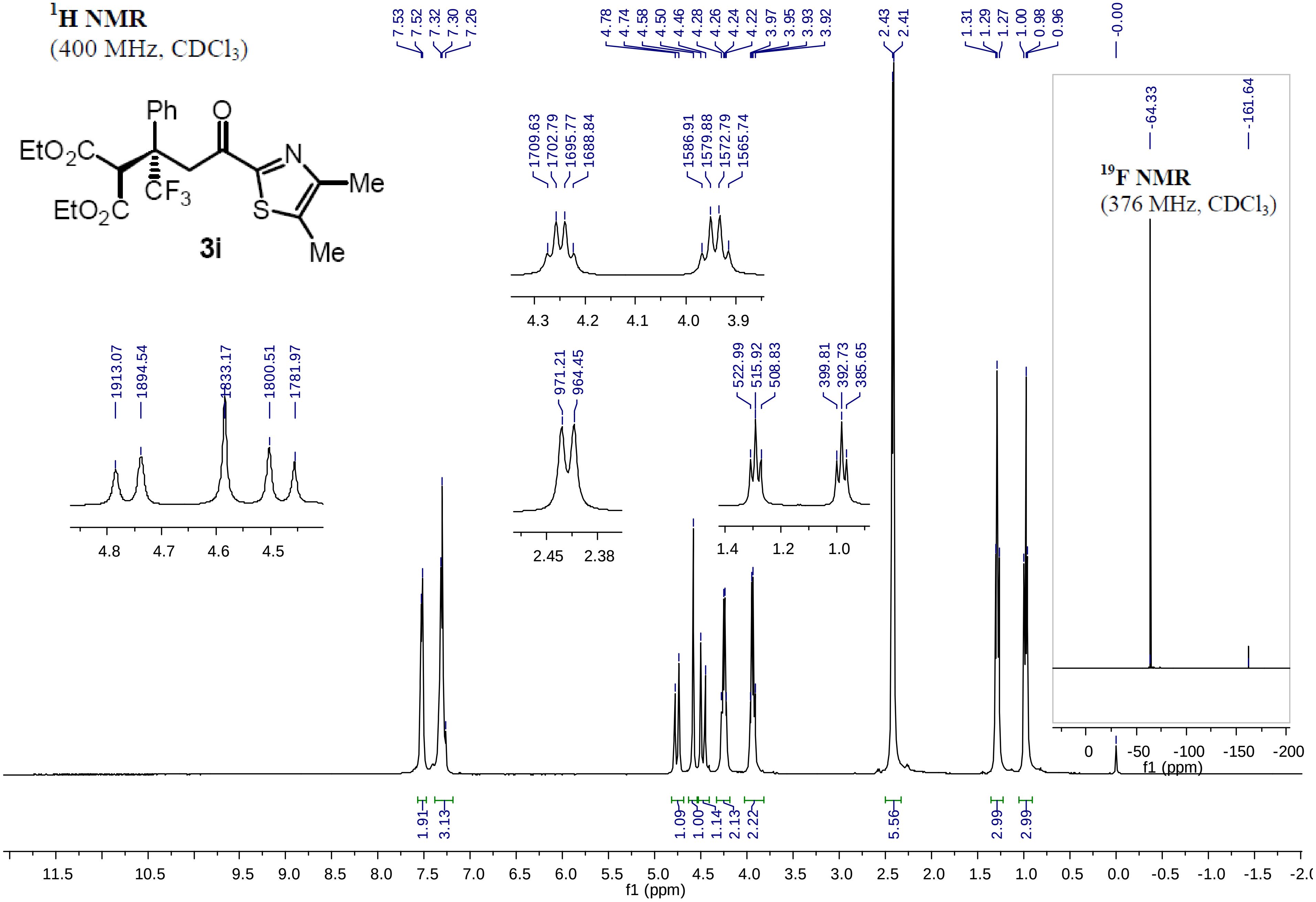

^{1}H NMR
(400 MHz, CDCl$_3$)
3i
^{19}F NMR
(376 MHz, CDCl$_3$)
f1 (ppm)

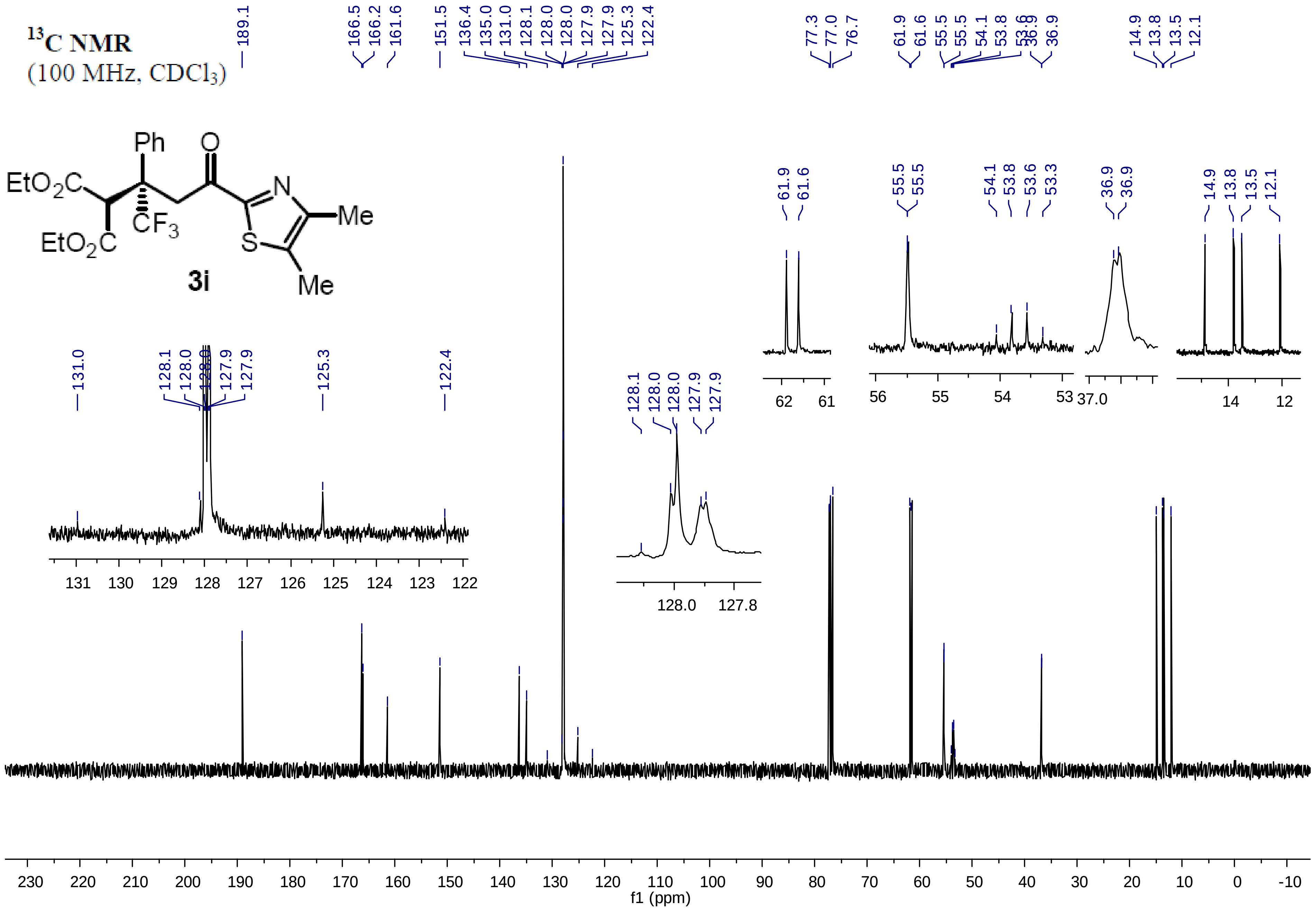
13C NMR
(100 MHz, CDCl3)
3i
EtO2C
EtO2C
Ph
O
CF3
N
S
Me
Me
189.1
166.5
166.2
161.6
151.5
136.4
135.0
131.0
128.1
128.0
128.0
127.9
127.9
125.3
122.4
77.3
77.0
76.7
61.9
61.6
55.5
55.5
54.1
53.8
53.6
53.3
36.9
36.9
14.9
13.8
13.5
12.1
f1 (ppm)

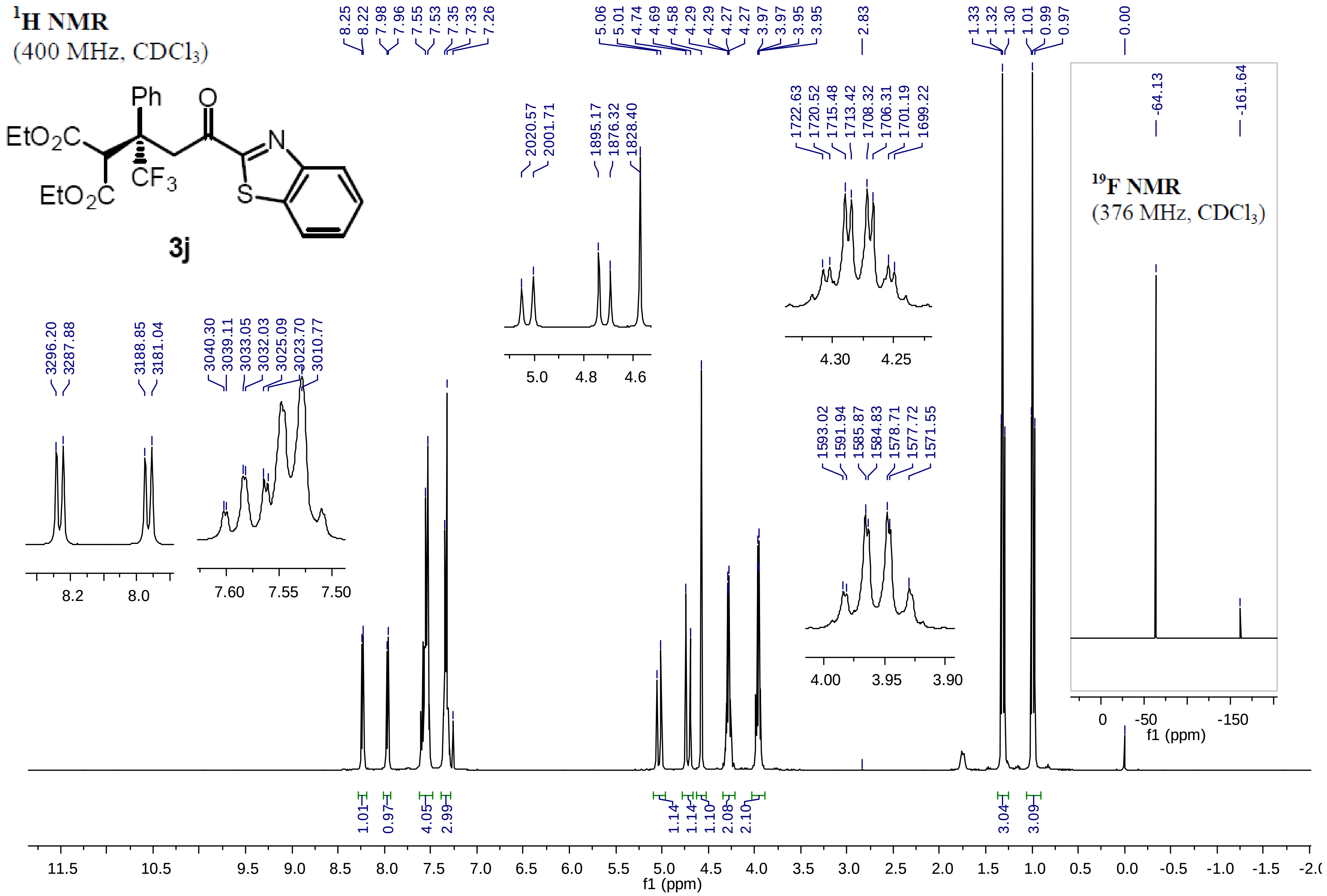
1H NMR
(400 MHz, CDCl3)
3j
19F NMR
(376 MHz, CDCl3)
-64.13
-161.64
f1 (ppm)

^{13}C NMR
(100 MHz, $CDCl_3$)

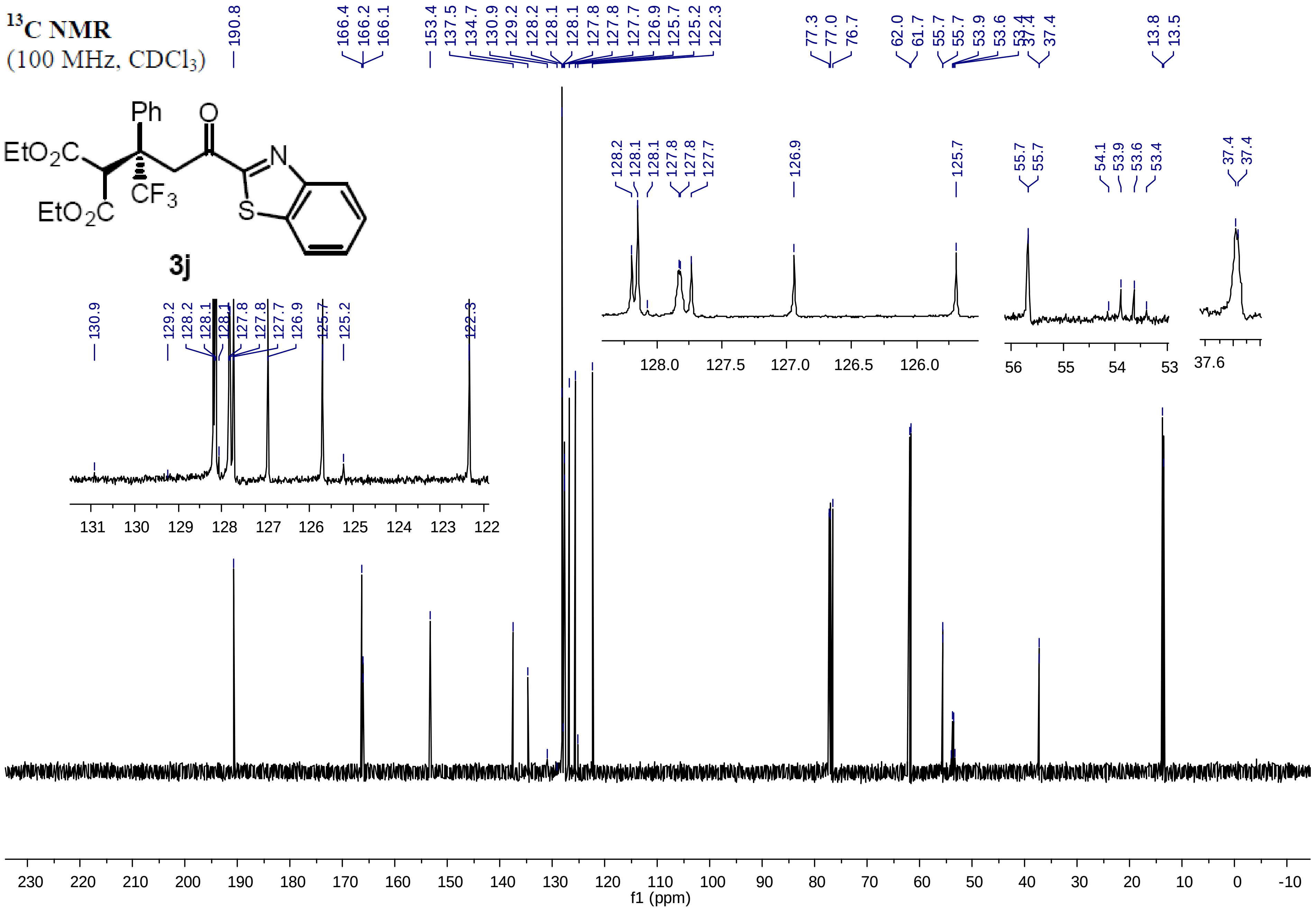

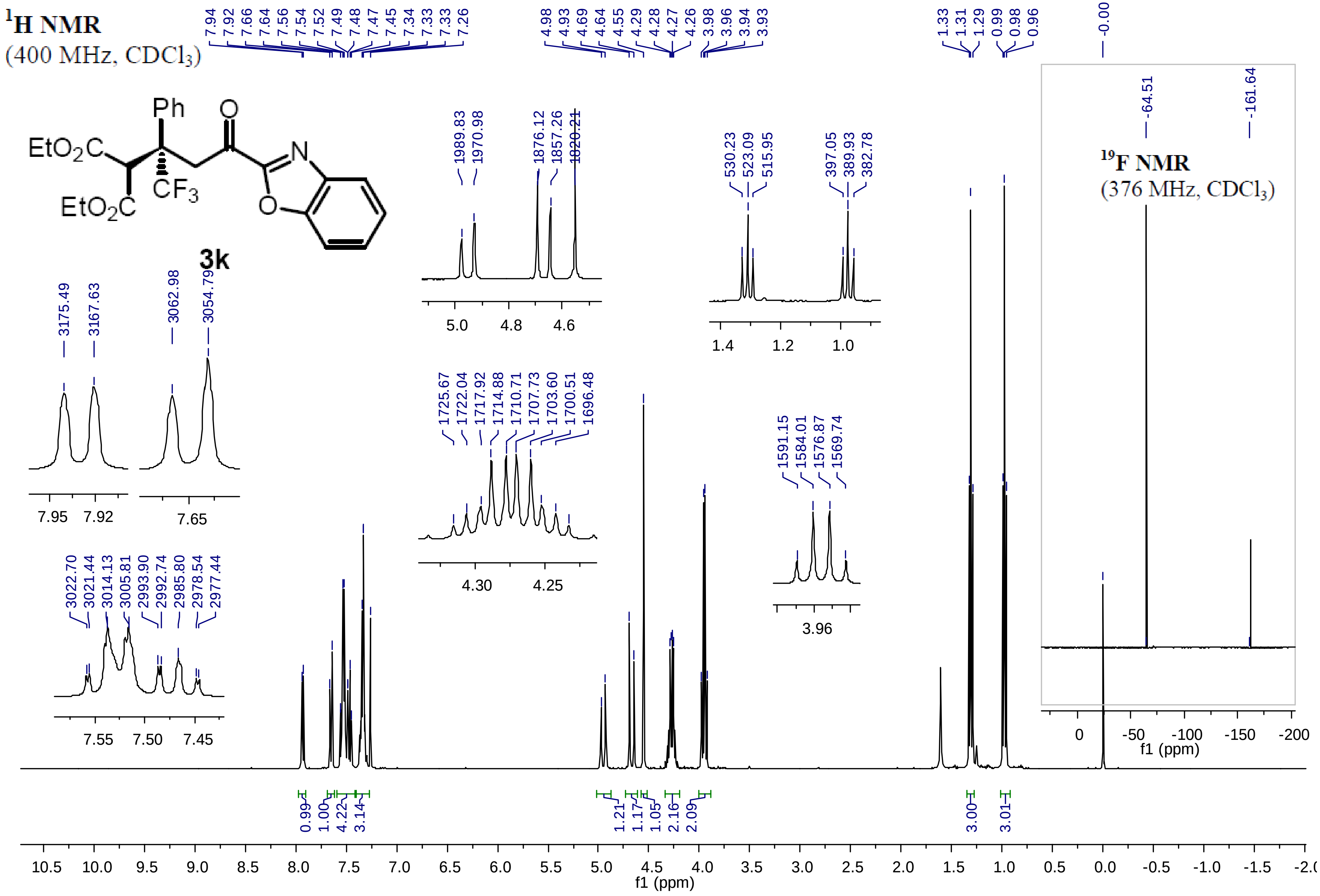

1H NMR
(400 MHz, CDCl3)
19F NMR
(376 MHz, CDCl3)
3k
-64.51
-161.64
f1 (ppm)

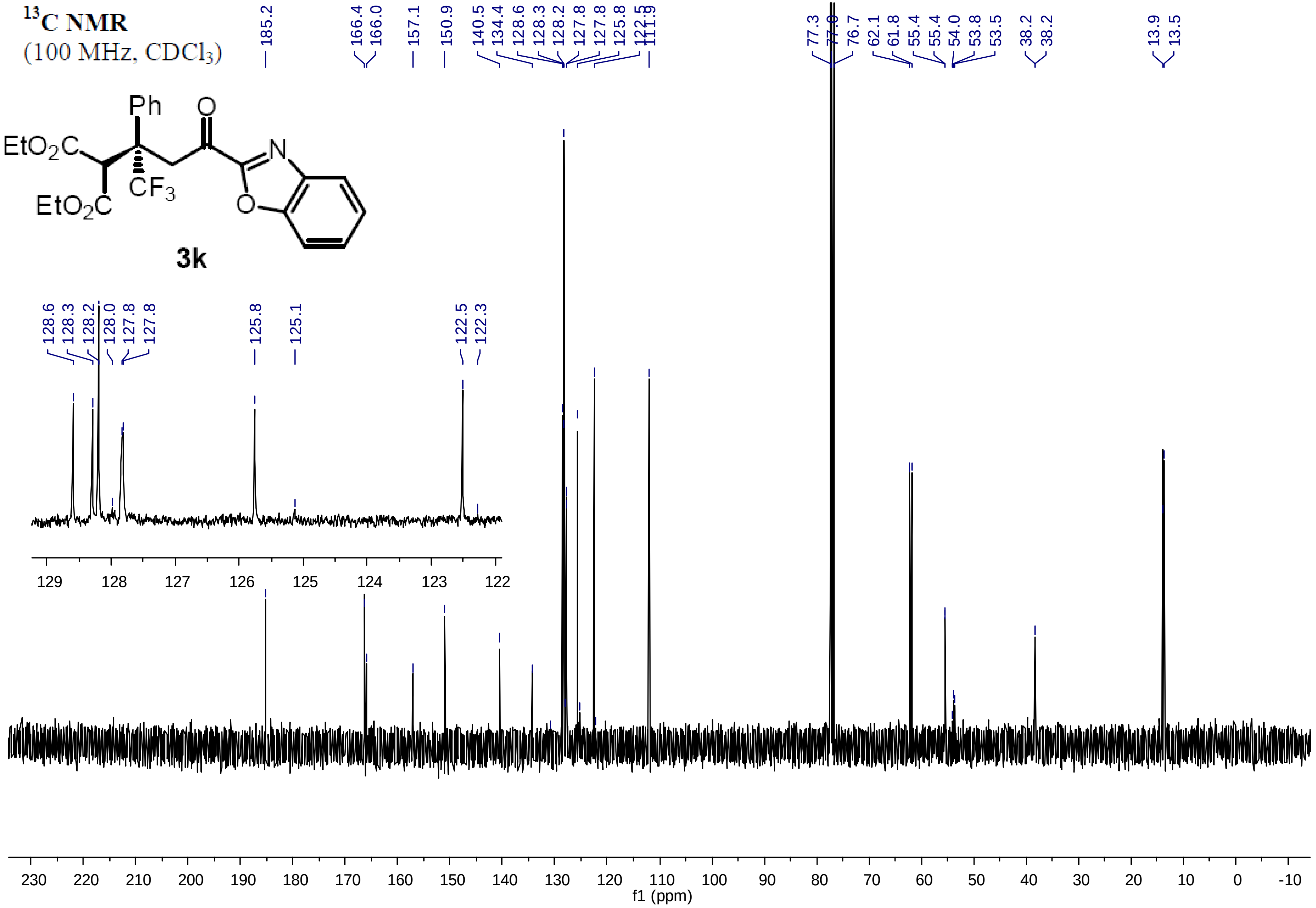

13C NMR
(100 MHz, CDCl3)
3k
185.2
166.4
166.0
157.1
150.9
140.5
134.4
128.6
128.3
128.2
127.8
127.8
125.8
122.5
111.9
77.3
77.0
76.7
62.1
61.8
55.4
55.4
54.0
53.8
53.5
38.2
38.2
13.9
13.5
128.6
128.3
128.2
128.0
127.8
127.8
125.8
125.1
122.5
122.3
f1 (ppm)

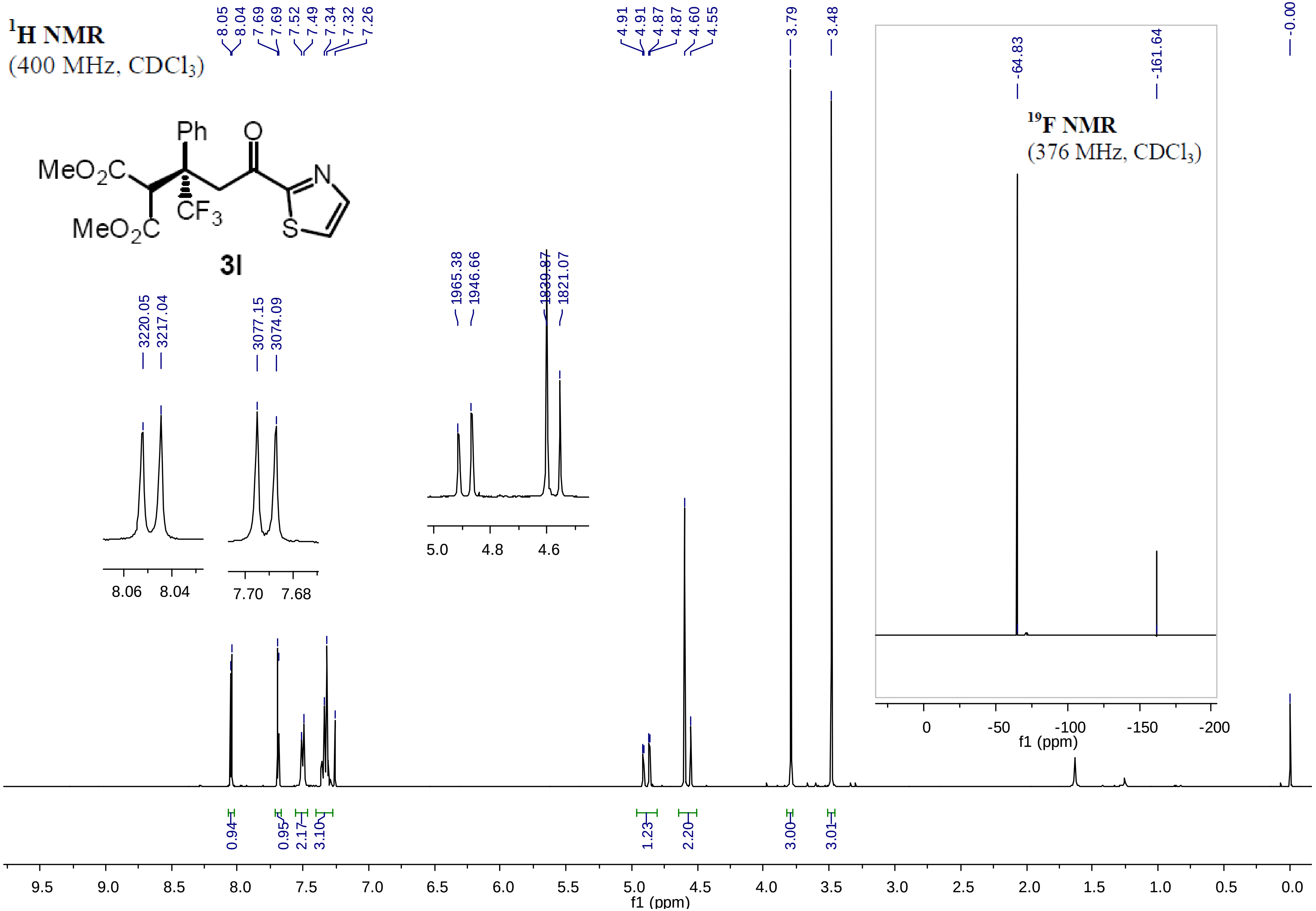

^{1}H NMR
(400 MHz, CDCl$_3$)
3l
^{19}F NMR
(376 MHz, CDCl$_3$)
-64.83
-161.64
8.05
8.04
7.69
7.69
7.52
7.49
7.34
7.32
7.26
4.91
4.91
4.87
4.87
4.60
4.55
3.79
3.48
-0.00
3220.05
3217.04
3077.15
3074.09
1965.38
1946.66
1839.87
1821.07
0.94
0.95
2.17
3.10
1.23
2.20
3.00
3.01
f1 (ppm)

^{13}C NMR
(100 MHz, $CDCl_3$)

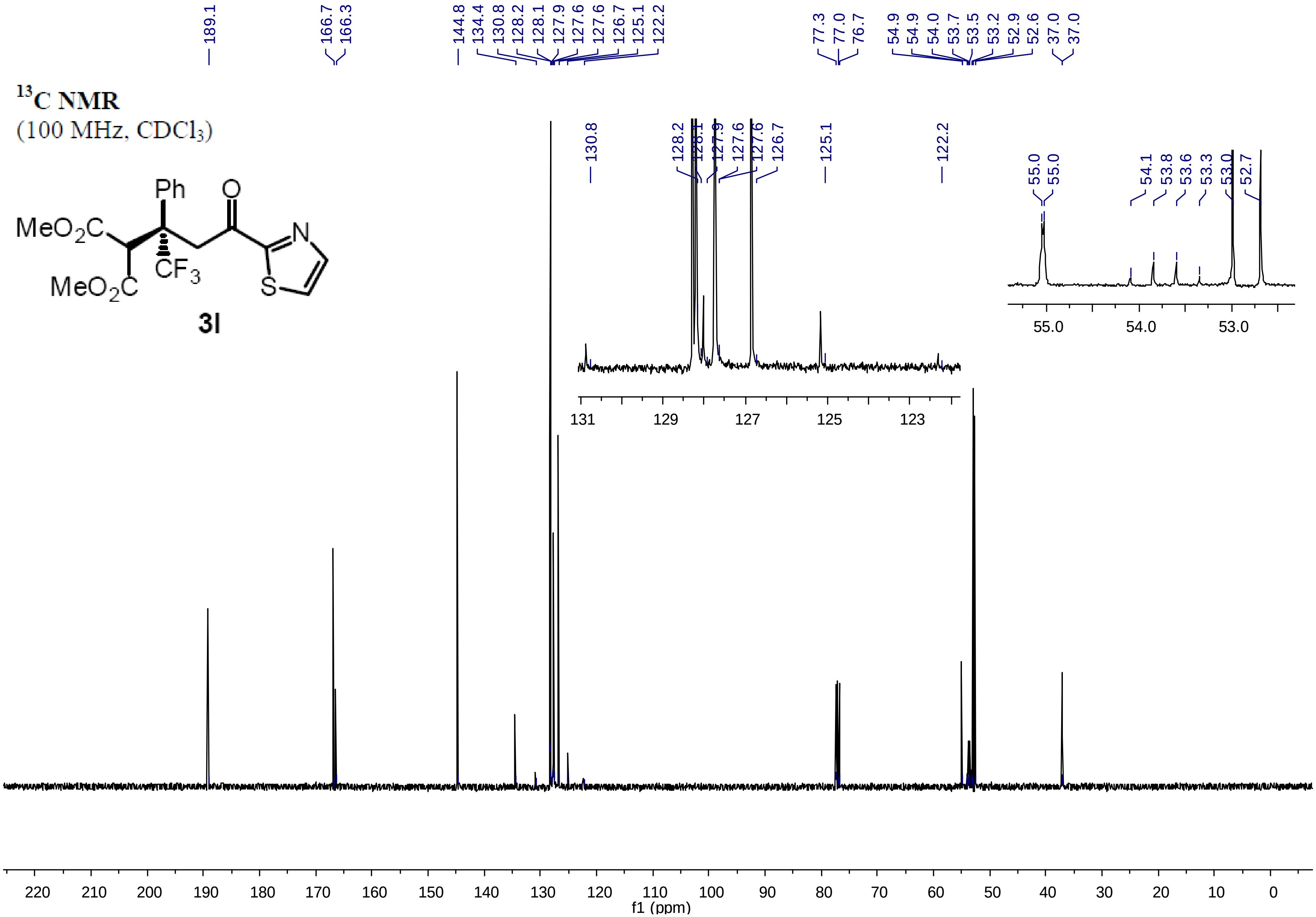

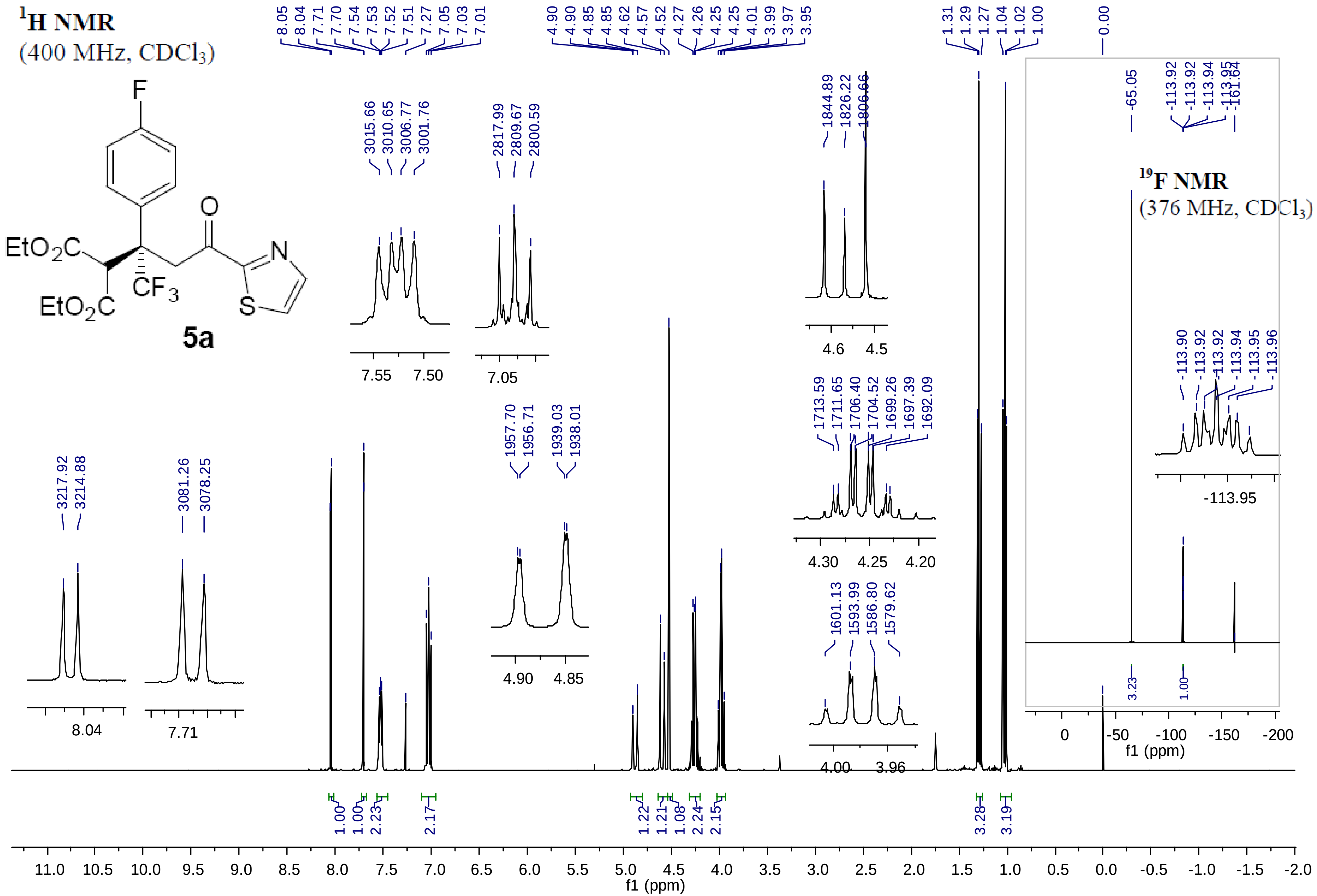

1H NMR
(400 MHz, CDCl3)
19F NMR
(376 MHz, CDCl3)
5a
F
O
EtO2C
EtO2C
CF3
N
S
f1 (ppm)

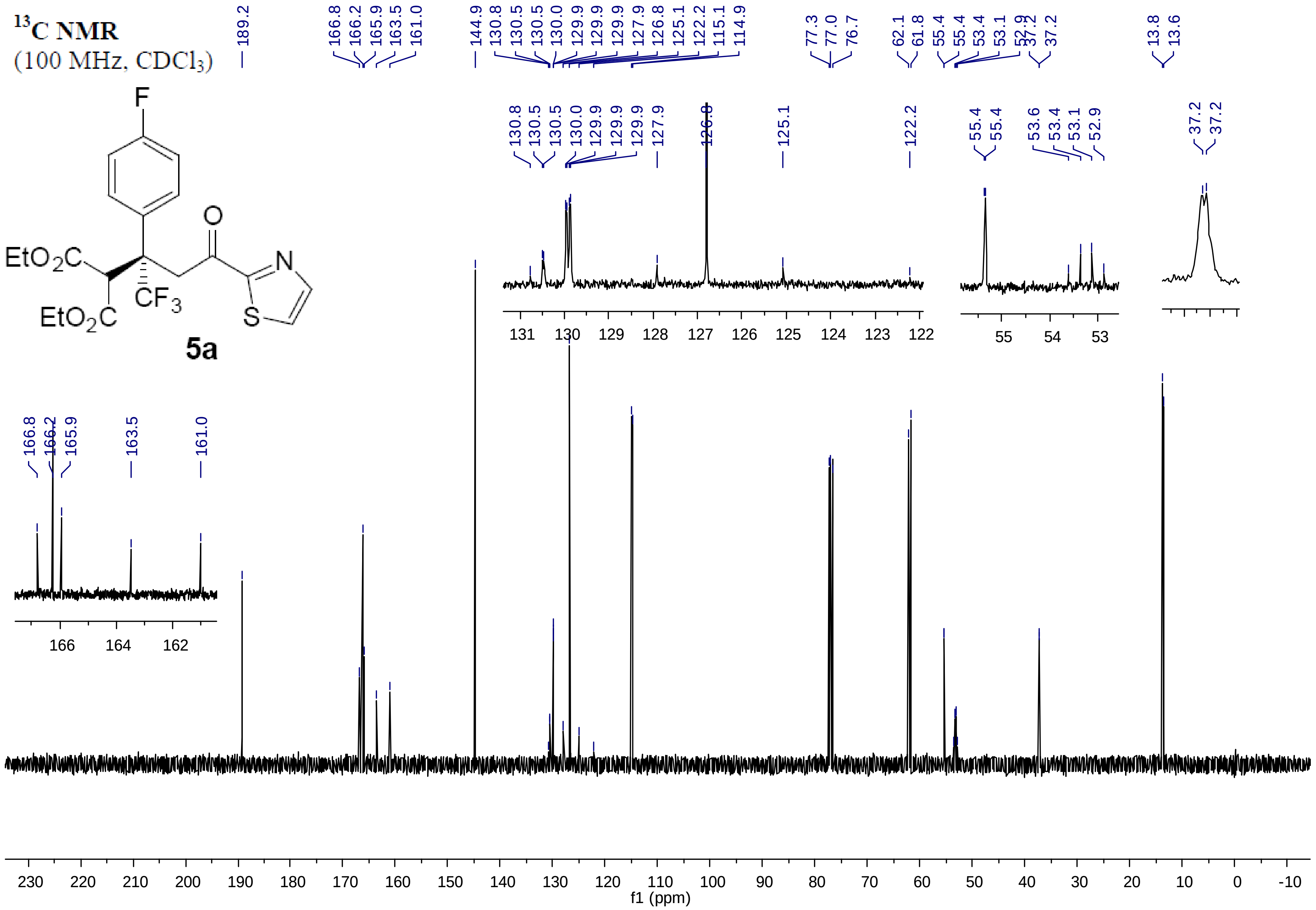

13C NMR
(100 MHz, CDCl3)
5a
EtO2C
EtO2C
CF3
F
O
N
S
189.2
166.8
166.2
165.9
163.5
161.0
144.9
130.8
130.5
130.5
130.0
129.9
129.9
129.9
127.9
126.8
125.1
122.2
115.1
114.9
77.3
77.0
76.7
62.1
61.8
55.4
55.4
53.4
53.1
52.9
37.2
37.2
13.8
13.6
f1 (ppm)

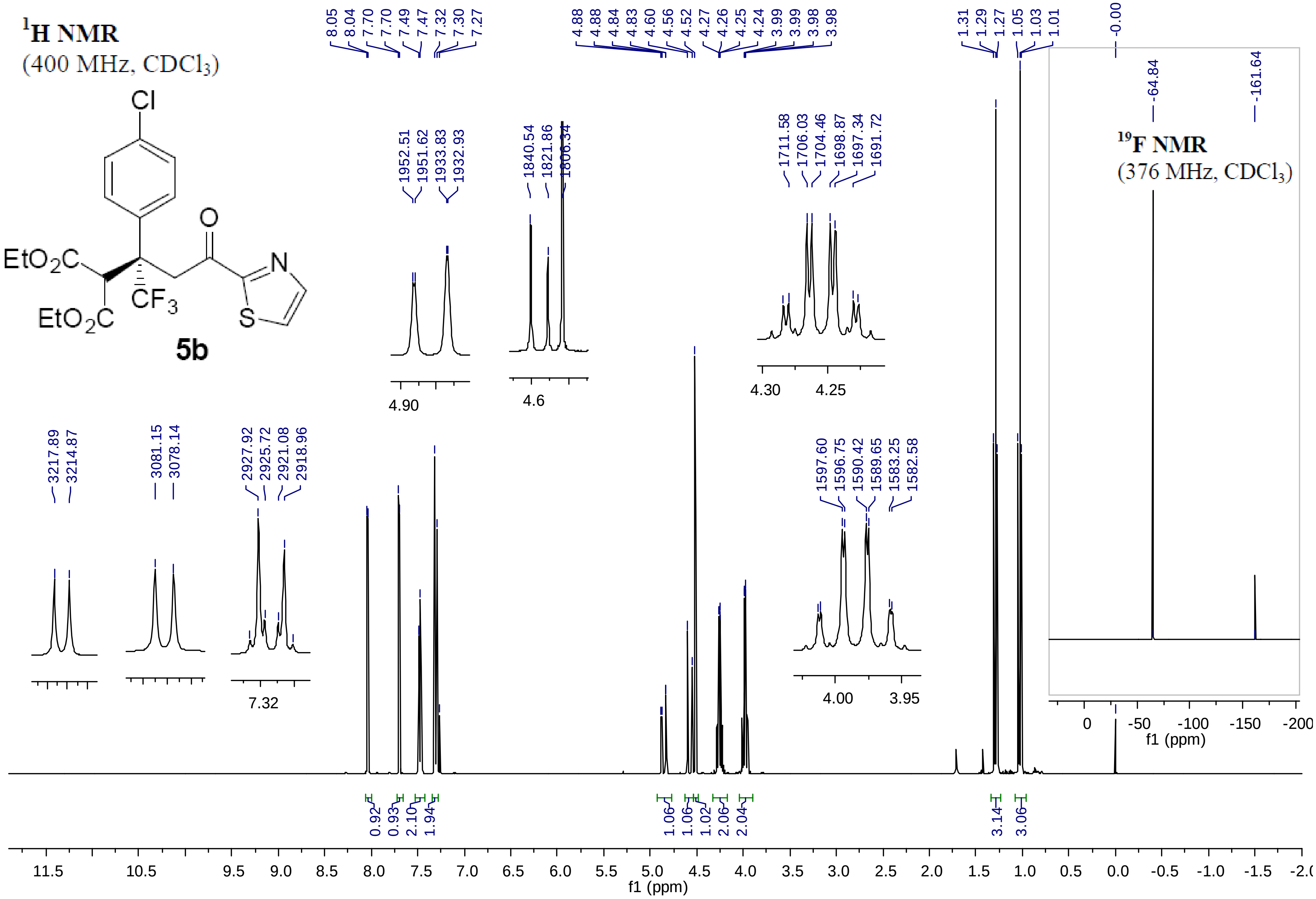

1H NMR
(400 MHz, CDCl3)
5b
19F NMR
(376 MHz, CDCl3)
f1 (ppm)

^{13}C NMR
(100 MHz, $CDCl_3$)

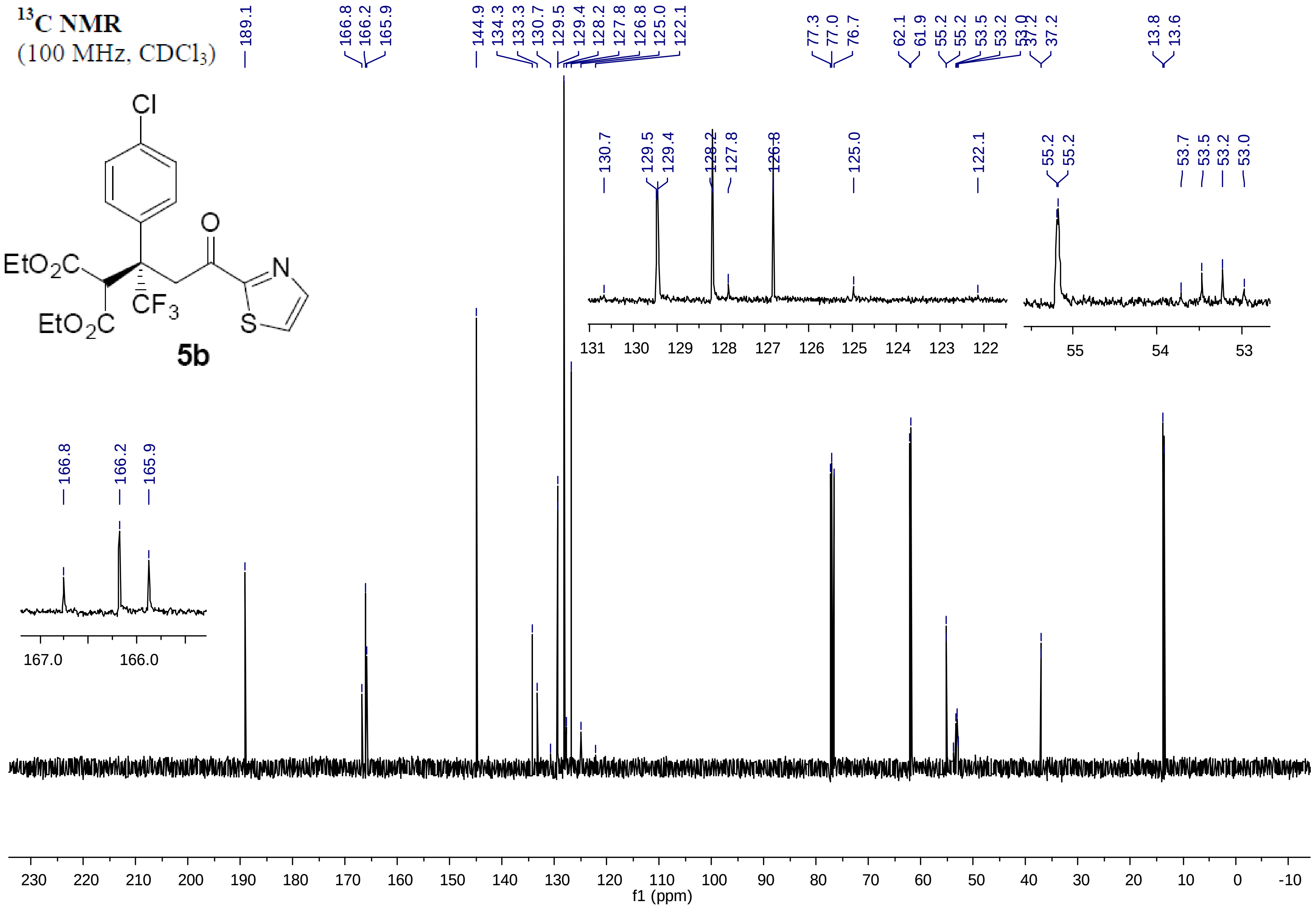

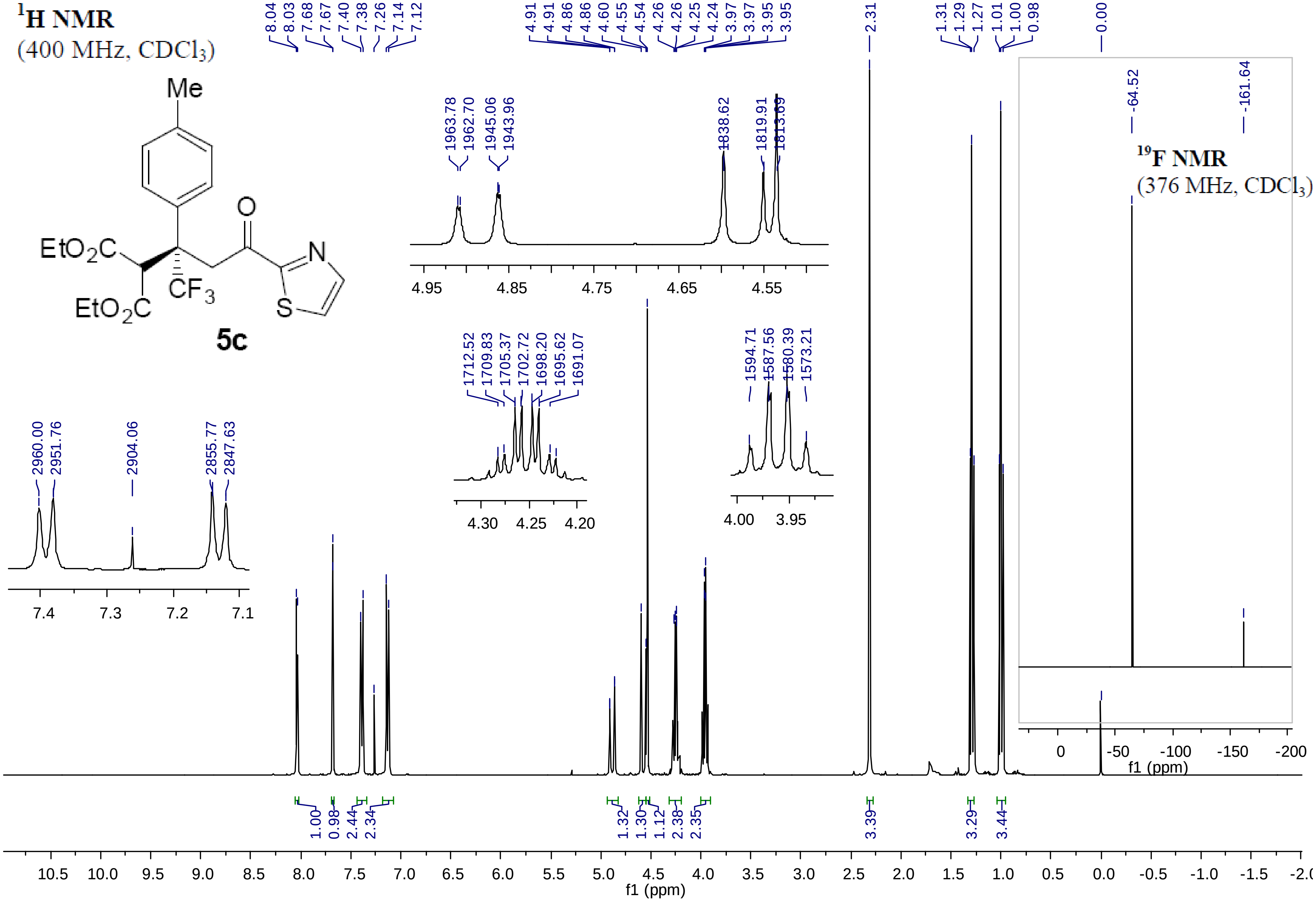

1H NMR
(400 MHz, CDCl3)
Me
EtO2C
EtO2C
CF3
O
N
S
5c
19F NMR
(376 MHz, CDCl3)
-64.52
-161.64
f1 (ppm)

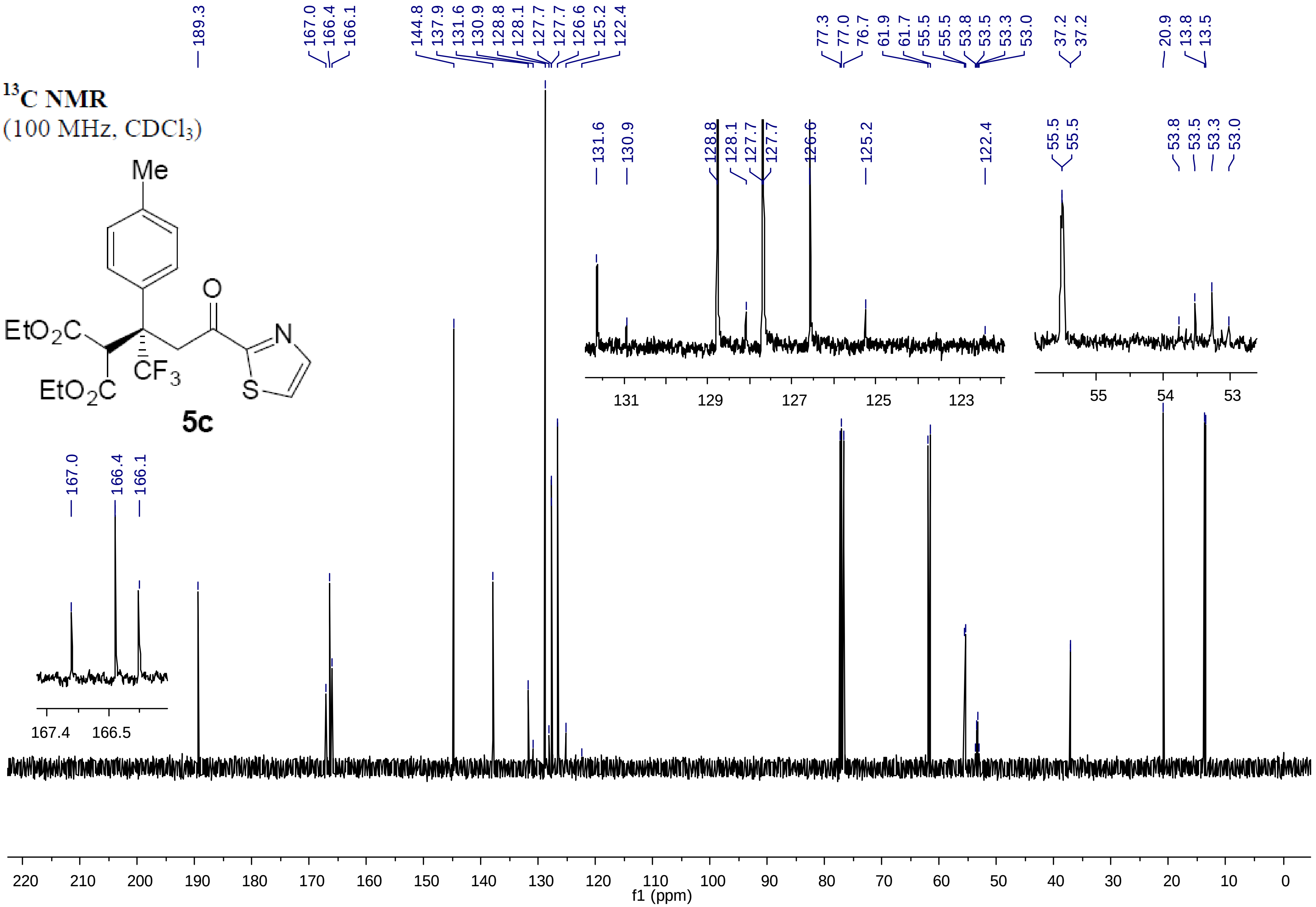

13C NMR
(100 MHz, CDCl3)
189.3
167.0
166.4
166.1
144.8
137.9
131.6
130.9
128.8
128.1
127.7
127.7
126.6
125.2
122.4
77.3
77.0
76.7
61.9
61.7
55.5
55.5
53.8
53.5
53.3
53.0
37.2
37.2
20.9
13.8
13.5
Me
O
EtO2C
N
CF3
EtO2C
S
5c
f1 (ppm)

^{1}H NMR
(400 MHz, $CDCl_3$)

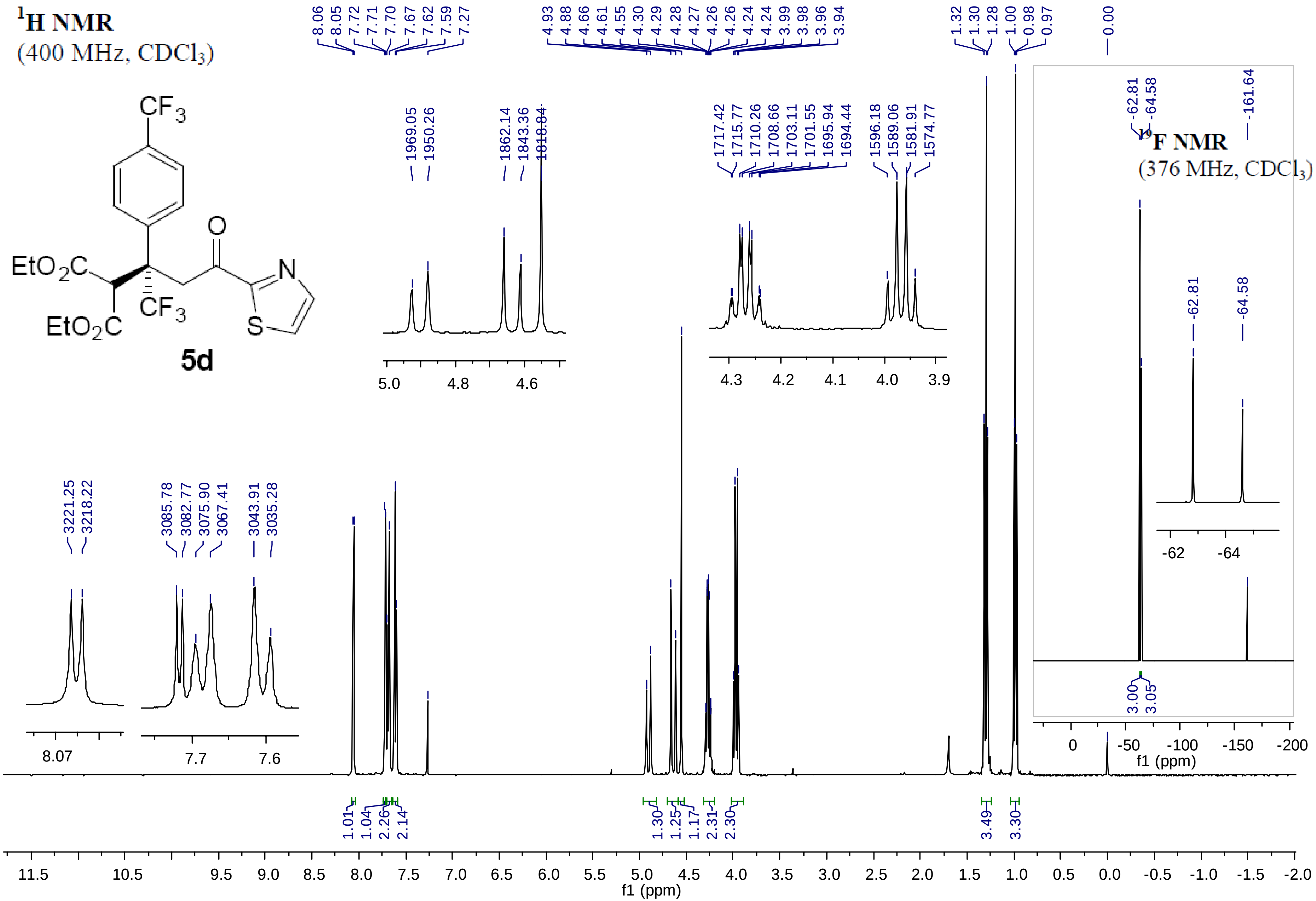

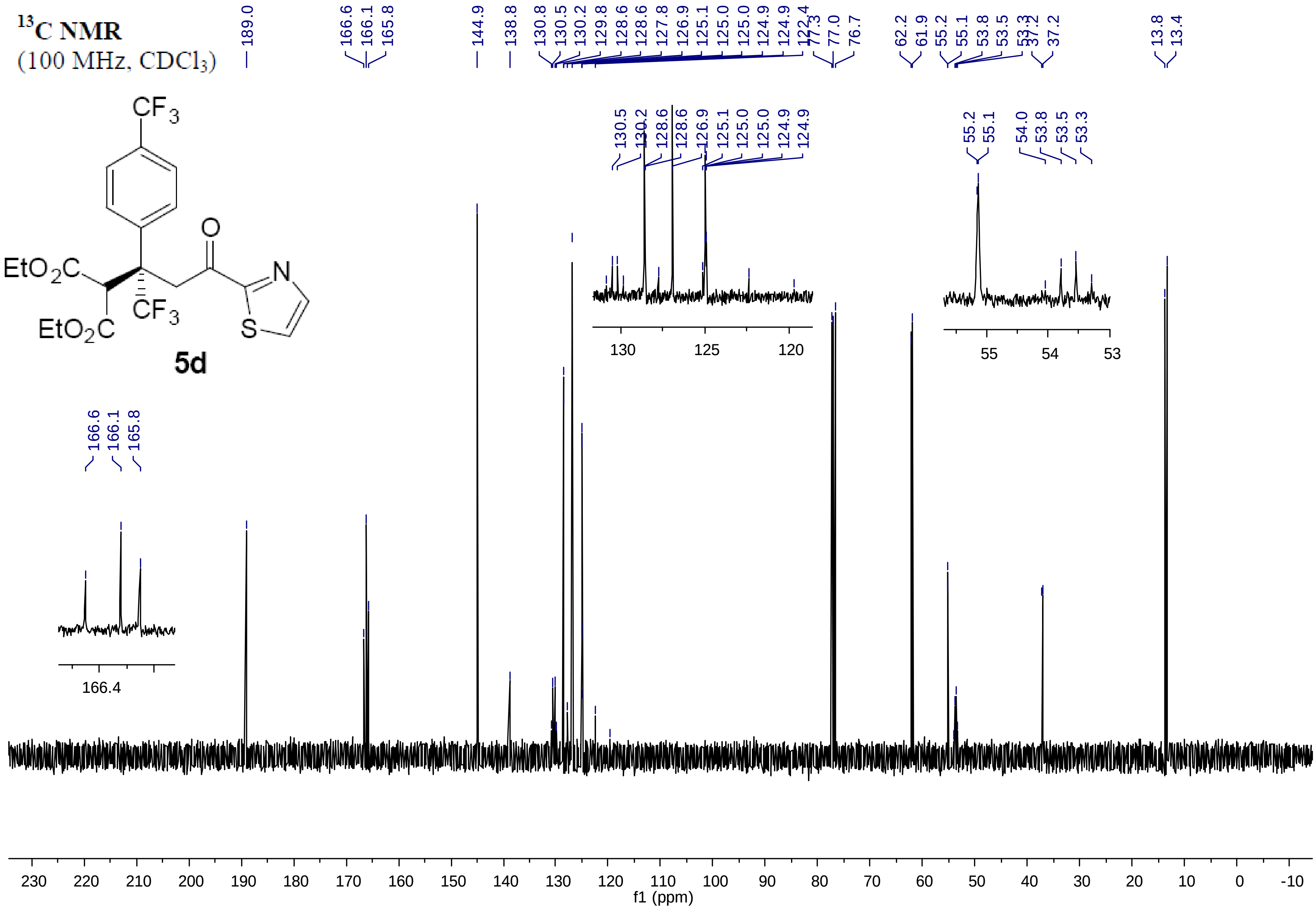

13C NMR
(100 MHz, CDCl3)
5d
189.0
166.6
166.1
165.8
144.9
138.8
130.8
130.5
130.2
129.8
128.6
128.6
127.8
126.9
125.1
125.0
125.0
124.9
124.9
122.4
77.3
77.0
76.7
62.2
61.9
55.2
55.1
53.8
53.5
53.3
37.2
37.2
13.8
13.4
f1 (ppm)

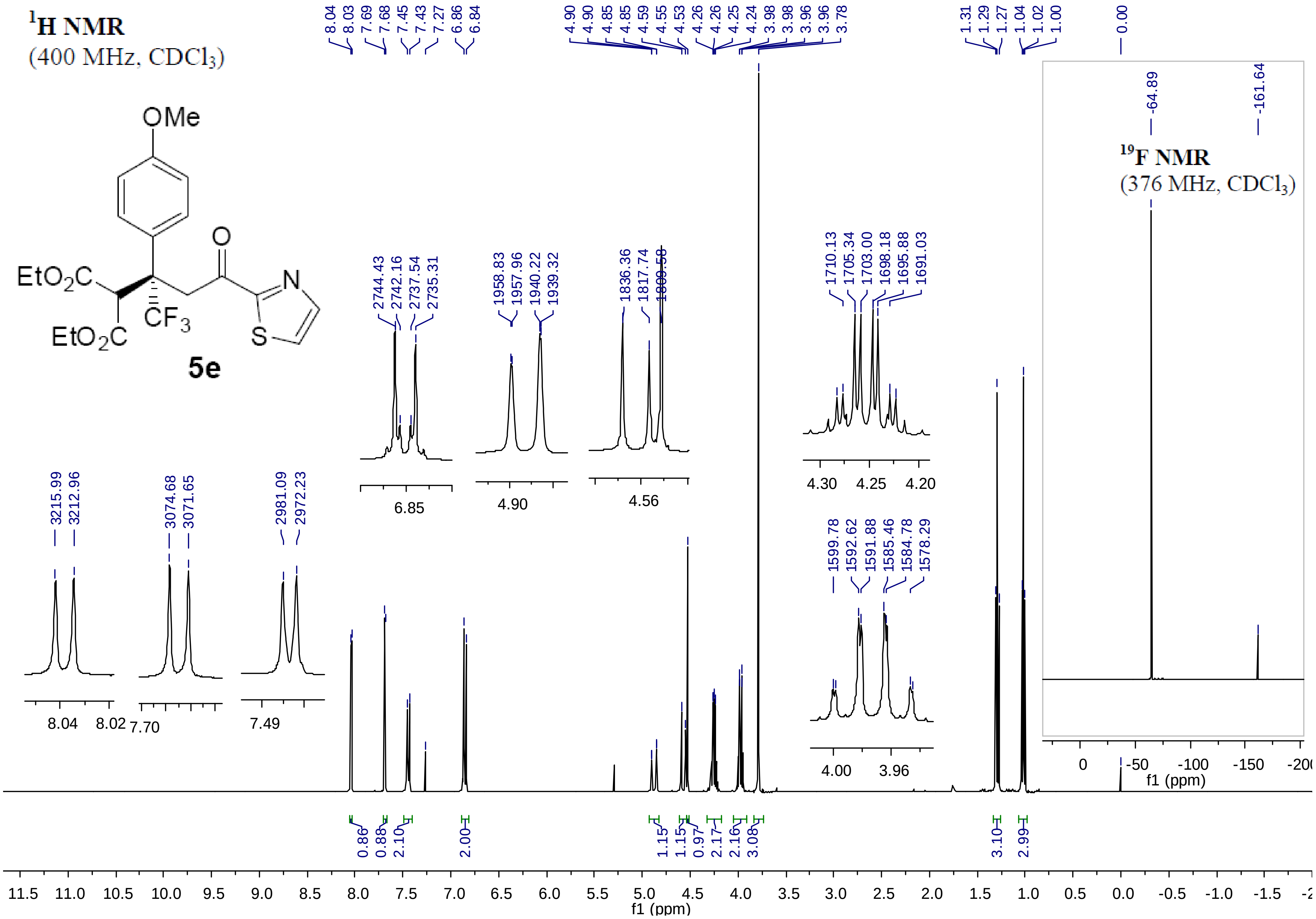

^{1}H NMR
(400 MHz, $CDCl_3$)
OMe
O
EtO_2C
EtO_2C
CF_3
N
S
5e
^{19}F NMR
(376 MHz, $CDCl_3$)
-64.89
-161.64
8.04 8.03 7.69 7.68 7.45 7.43 7.27 6.86 6.84
4.90 4.90 4.85 4.85 4.59 4.55 4.53 4.26 4.26 4.25 4.24 3.98 3.98 3.96 3.96 3.78
1.31 1.29 1.27 1.04 1.02 1.00
0.00
3215.99 3212.96
3074.68 3071.65
2981.09 2972.23
2744.43 2742.16 2737.54 2735.31
1958.83 1957.96 1940.22 1939.32
1836.36 1817.74 1809.58
1710.13 1705.34 1703.00 1698.18 1695.88 1691.03
1599.78 1592.62 1591.88 1585.46 1584.78 1578.29
0.86 0.88 2.10 2.00 1.15 1.15 0.97 2.17 2.16 3.08 3.10 2.99
f1 (ppm)

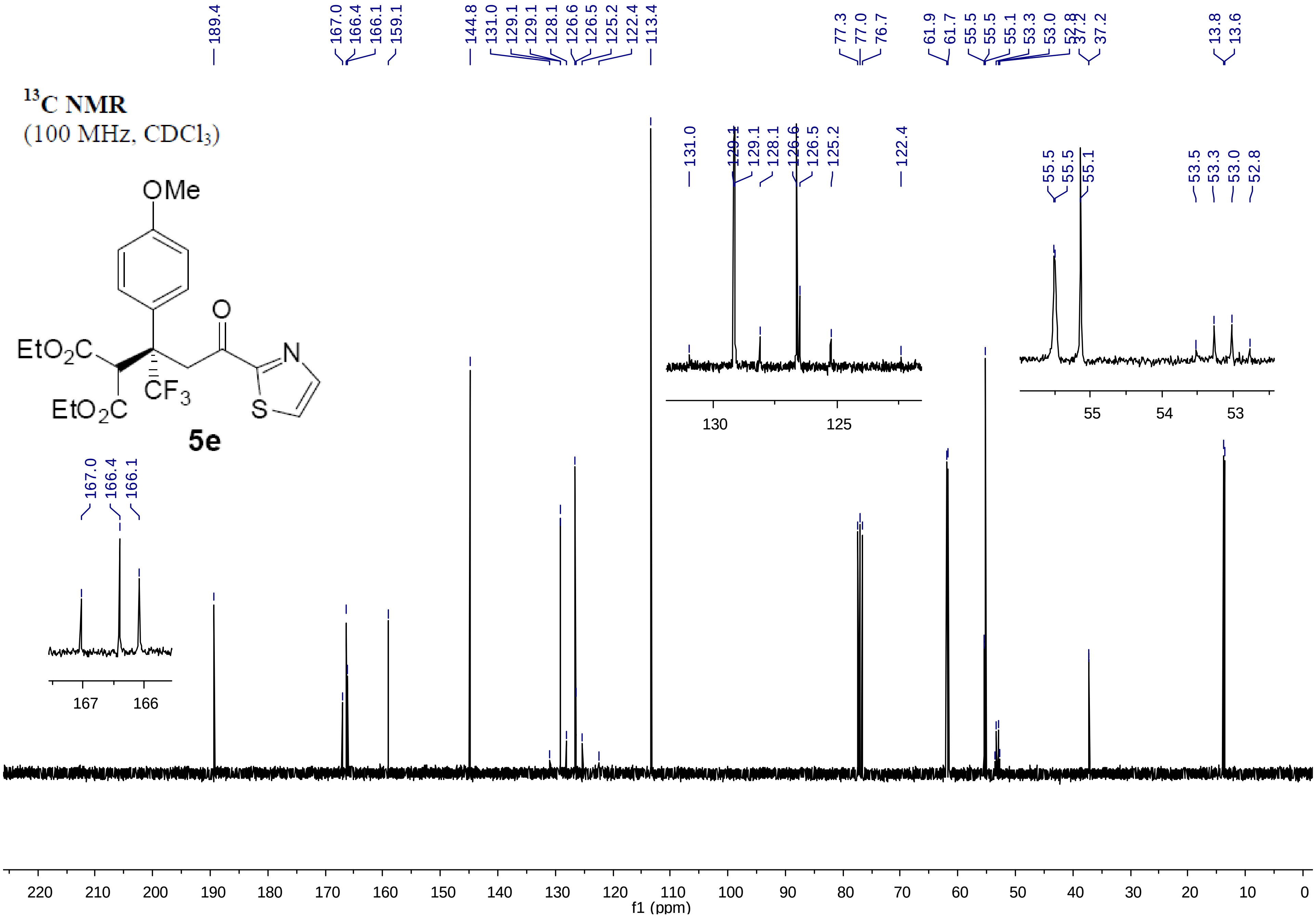

13C NMR
(100 MHz, CDCl3)
OMe
O
EtO2C
N
EtO2C
CF3
S
5e
189.4
167.0
166.4
166.1
159.1
144.8
131.0
129.1
129.1
128.1
126.6
126.5
125.2
122.4
113.4
77.3
77.0
76.7
61.9
61.7
55.5
55.5
55.1
53.3
53.0
52.8
37.2
37.2
13.8
13.6
53.5
167
166
130
125
55
54
53
220
210
200
190
180
170
160
150
140
130
120
110
100
90
80
70
60
50
40
30
20
10
0
f1 (ppm)

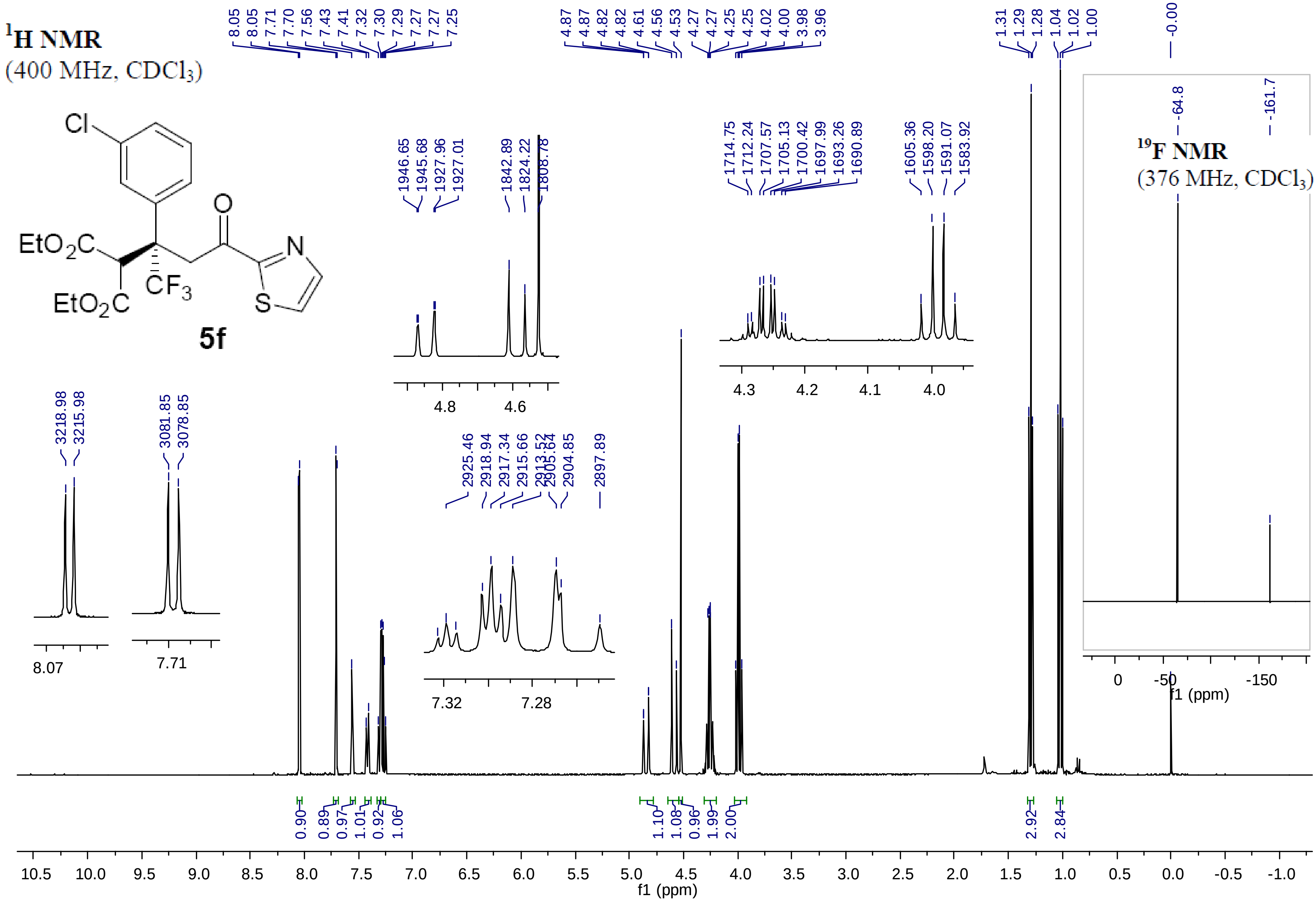

^{1}H NMR
(400 MHz, CDCl3)
Cl
EtO2C
EtO2C
CF3
O
N
S
5f
^{19}F NMR
(376 MHz, CDCl3)
f1 (ppm)

^{13}C NMR

(100 MHz, $CDCl_3$)

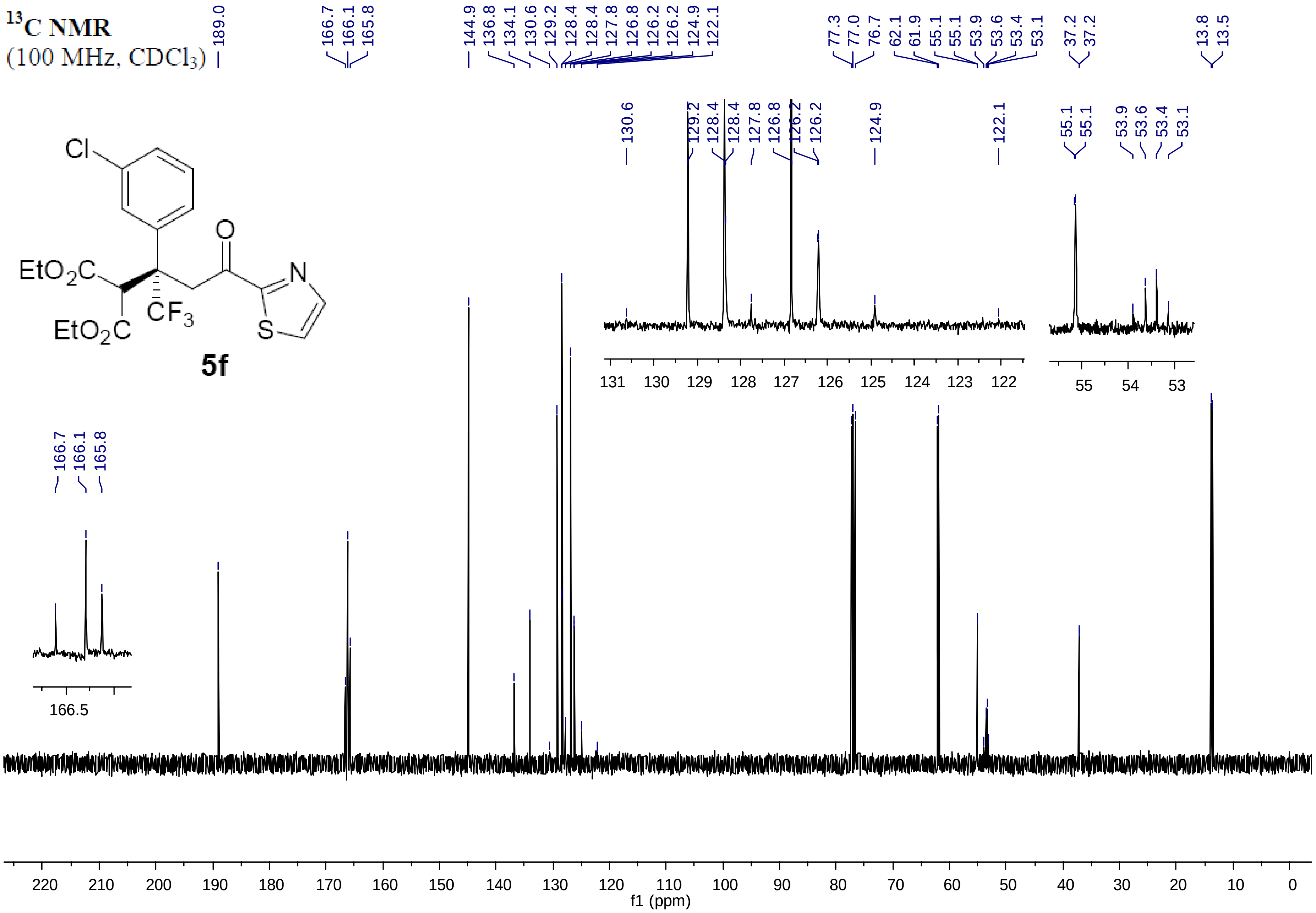

5f

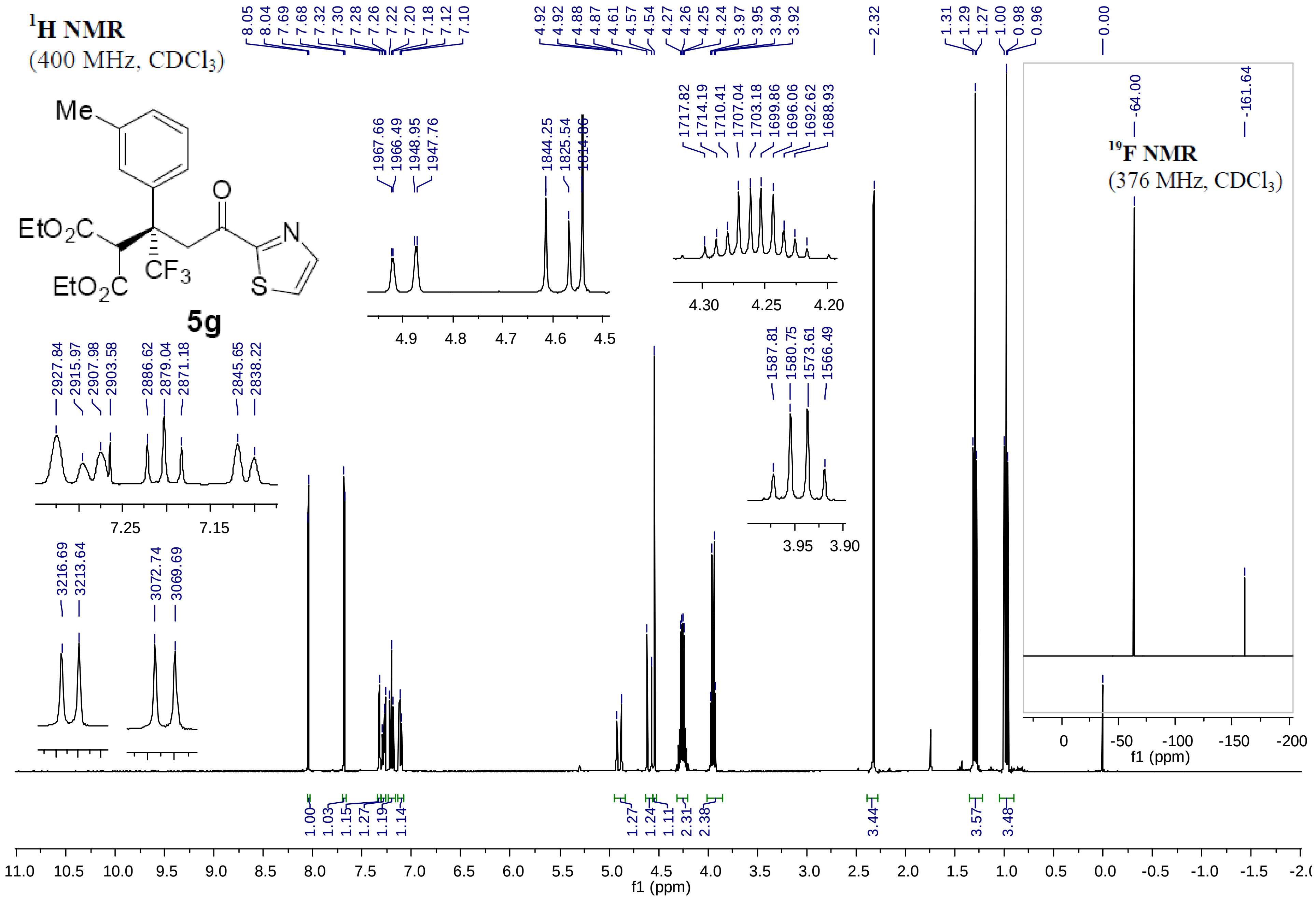

^{1}H NMR
(400 MHz, CDCl3)
^{19}F NMR
(376 MHz, CDCl3)
5g
f1 (ppm)

^{13}C NMR
(100 MHz, $CDCl_3$)

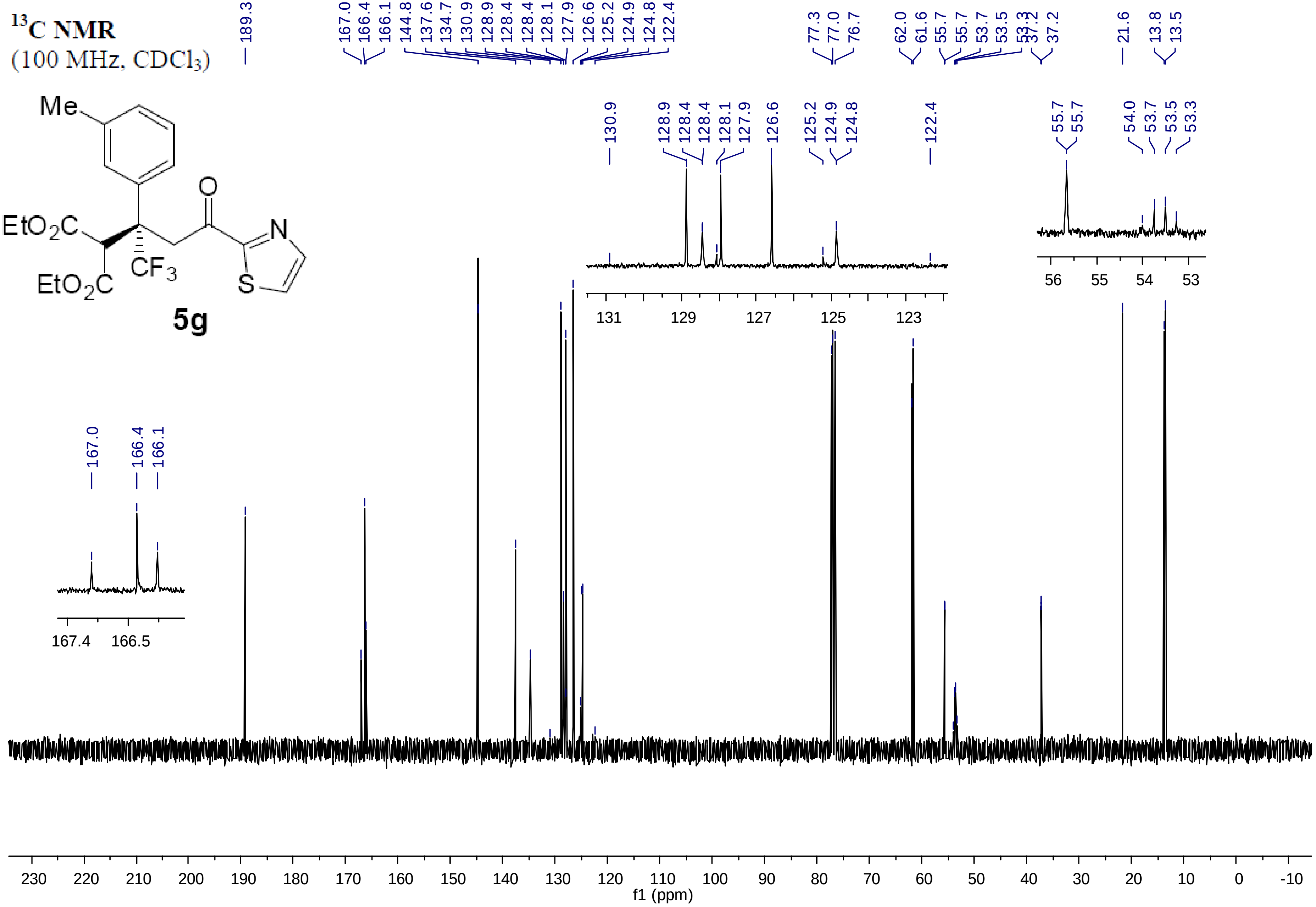

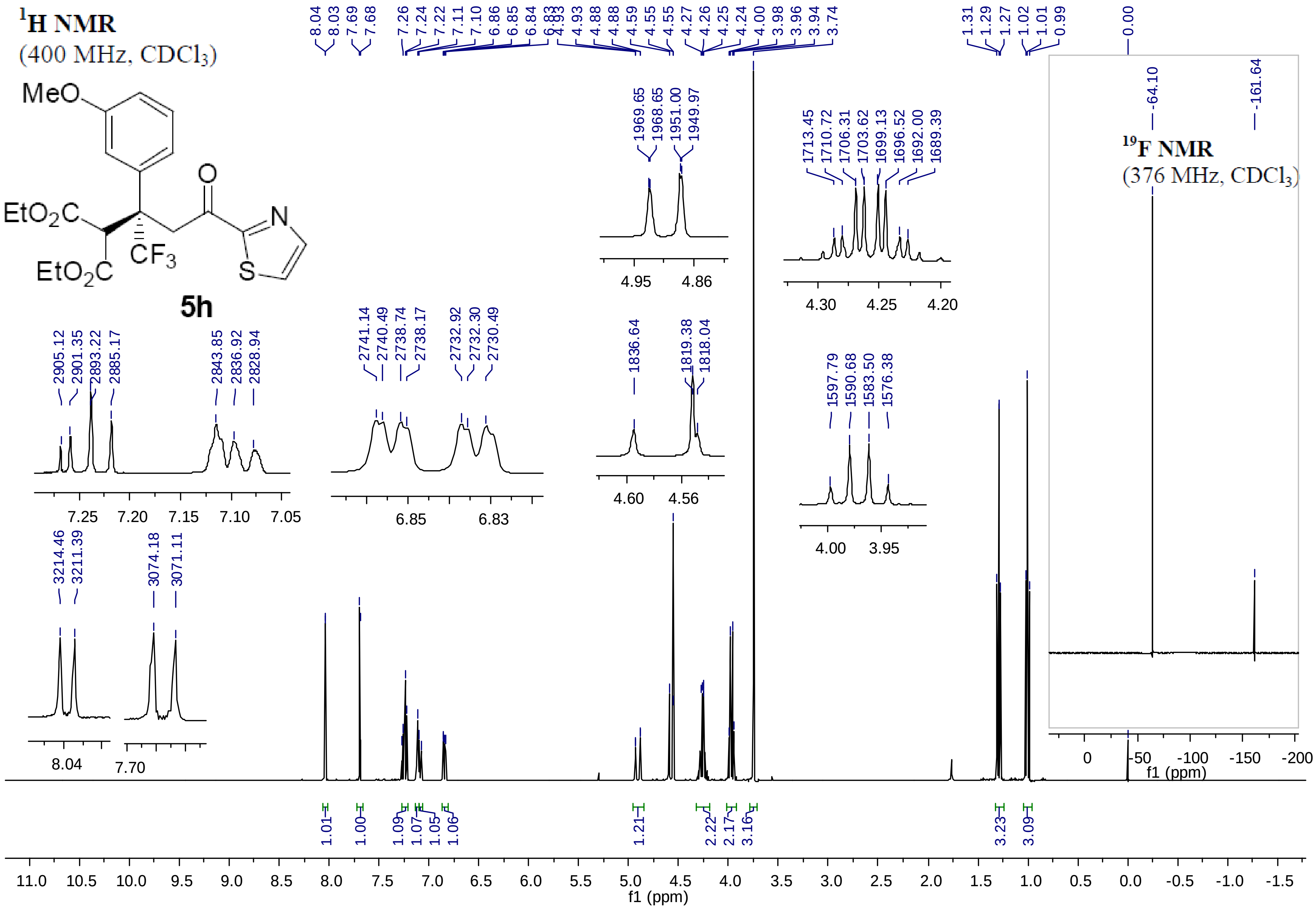

1H NMR
(400 MHz, CDCl3)
MeO
EtO2C
EtO2C
CF3
O
N
S
5h
19F NMR
(376 MHz, CDCl3)
-64.10
-161.64
f1 (ppm)

^{13}C NMR
(100 MHz, $CDCl_3$)

5h

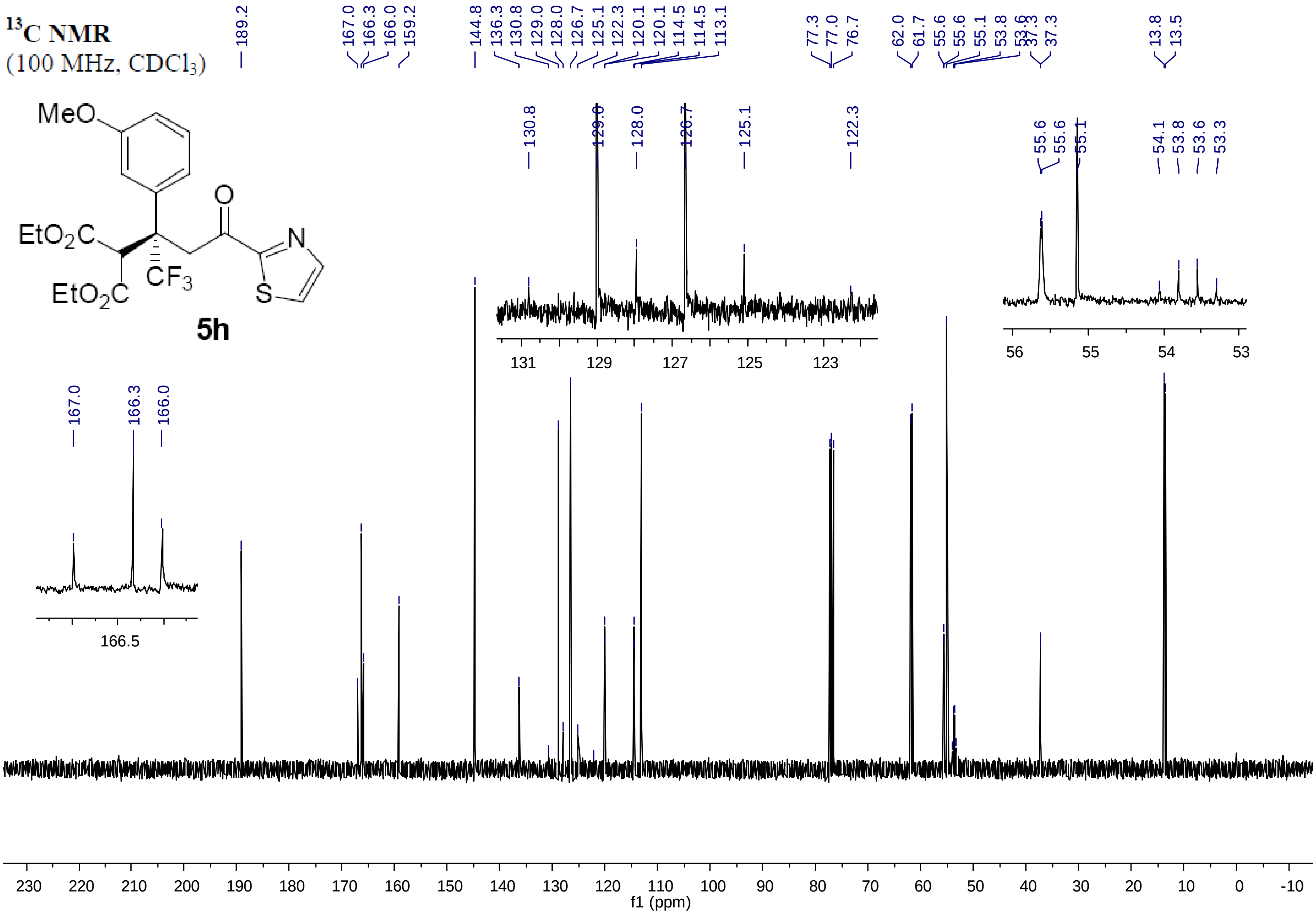

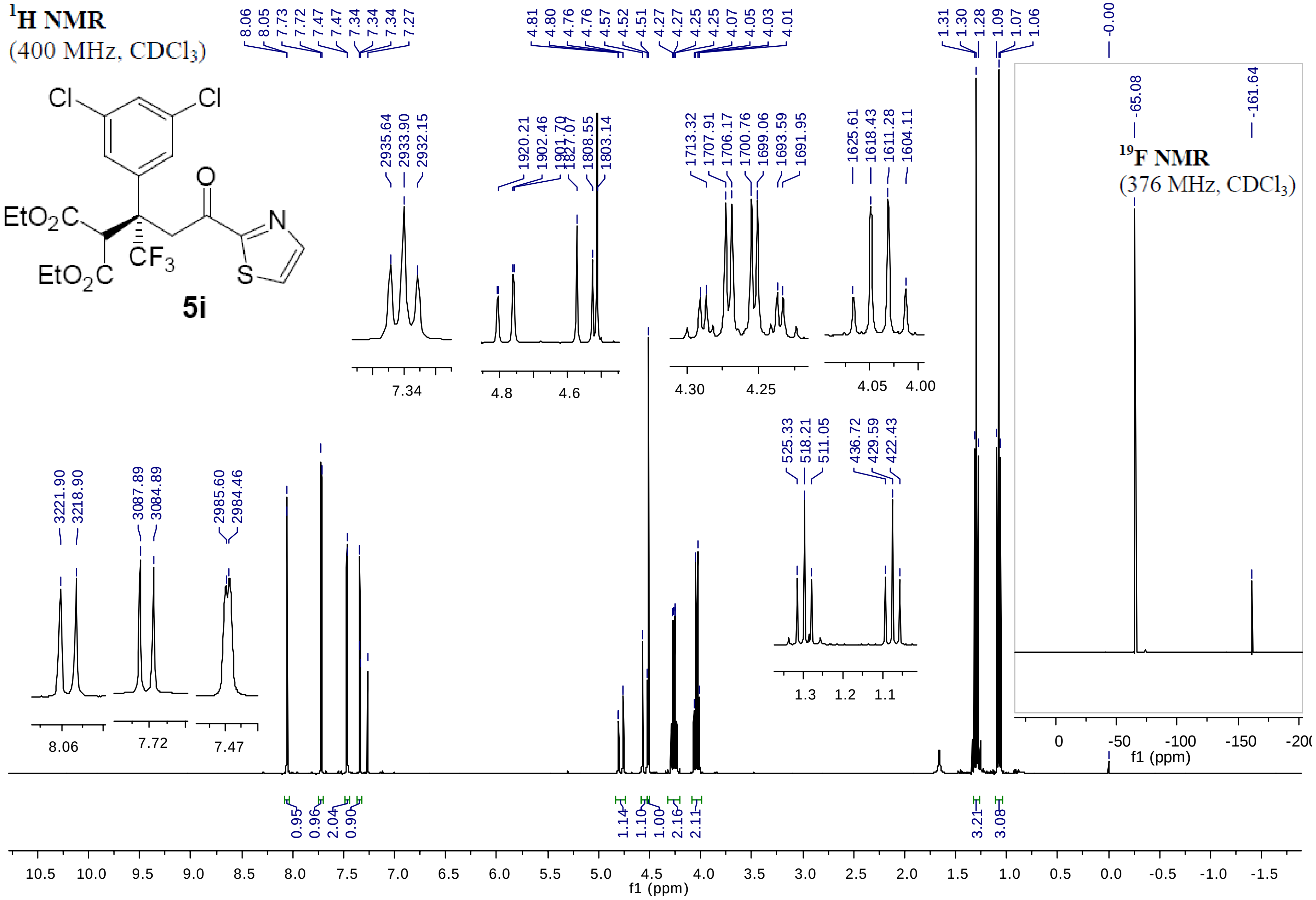

1H NMR
(400 MHz, CDCl3)
5i
19F NMR
(376 MHz, CDCl3)
-65.08
-161.64
f1 (ppm)

^{13}C NMR
(100 MHz, $CDCl_3$)

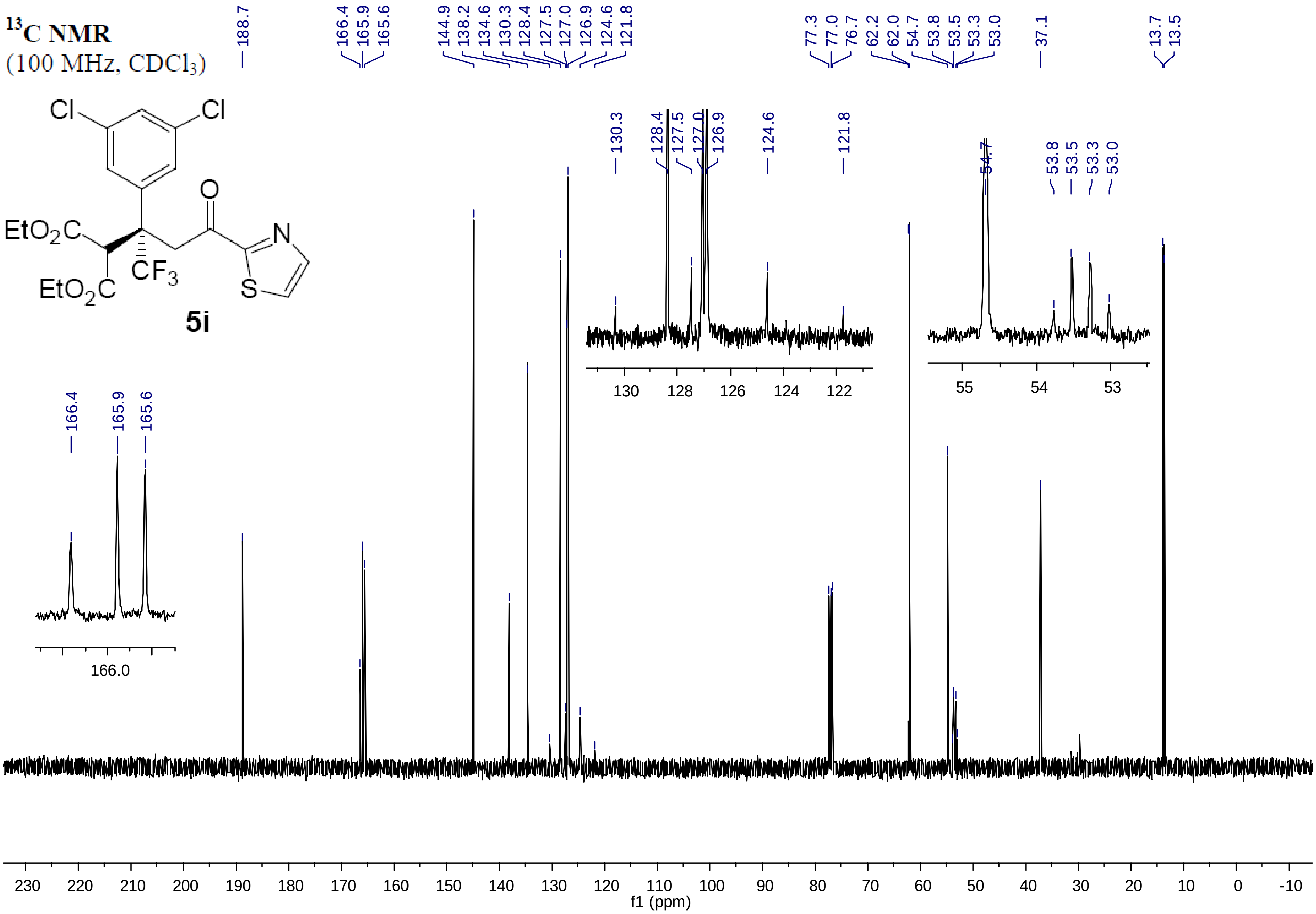

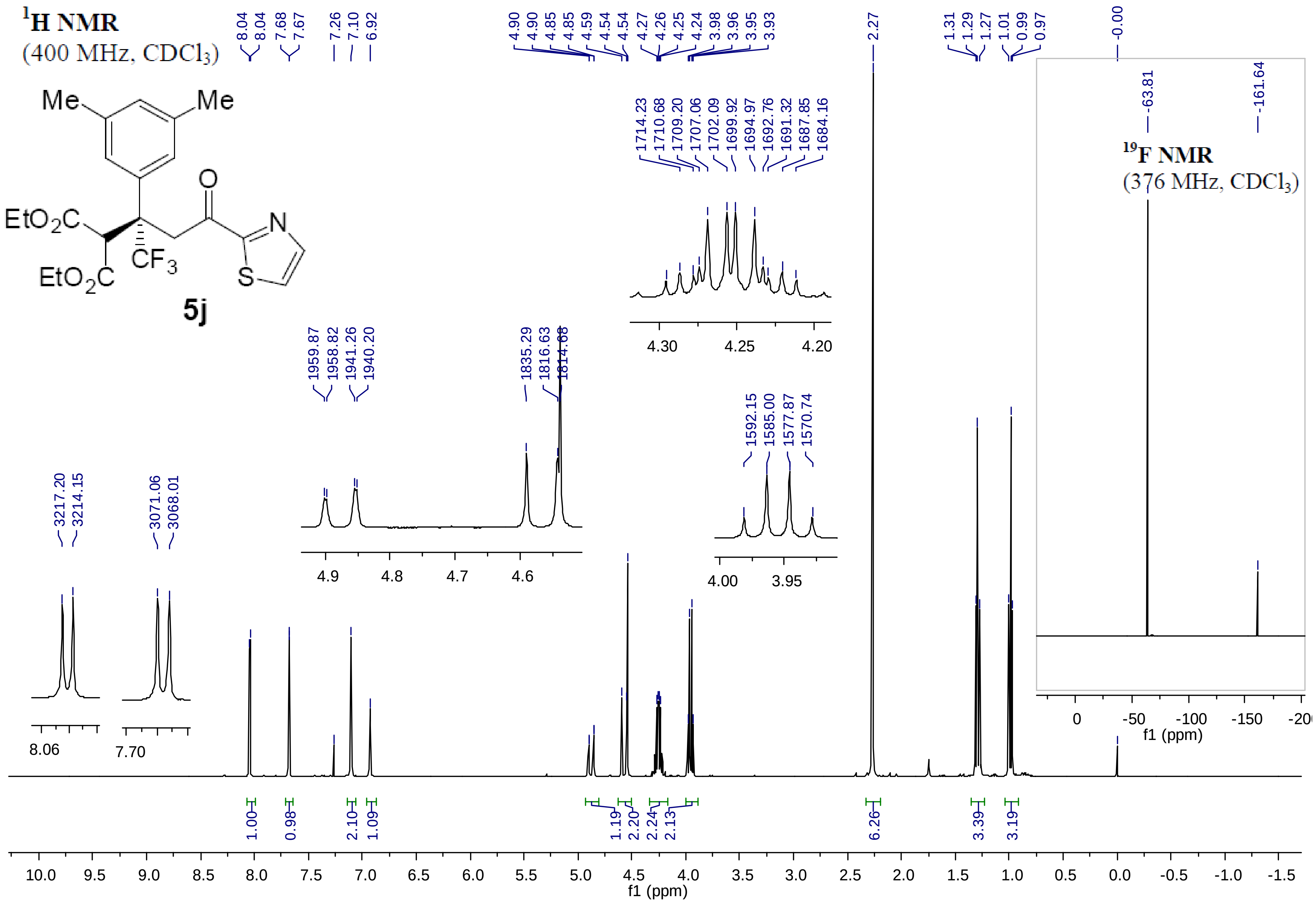

1H NMR
(400 MHz, CDCl3)
5j
19F NMR
(376 MHz, CDCl3)
-63.81
-161.64
f1 (ppm)

^{13}C NMR
(100 MHz, $CDCl_3$)

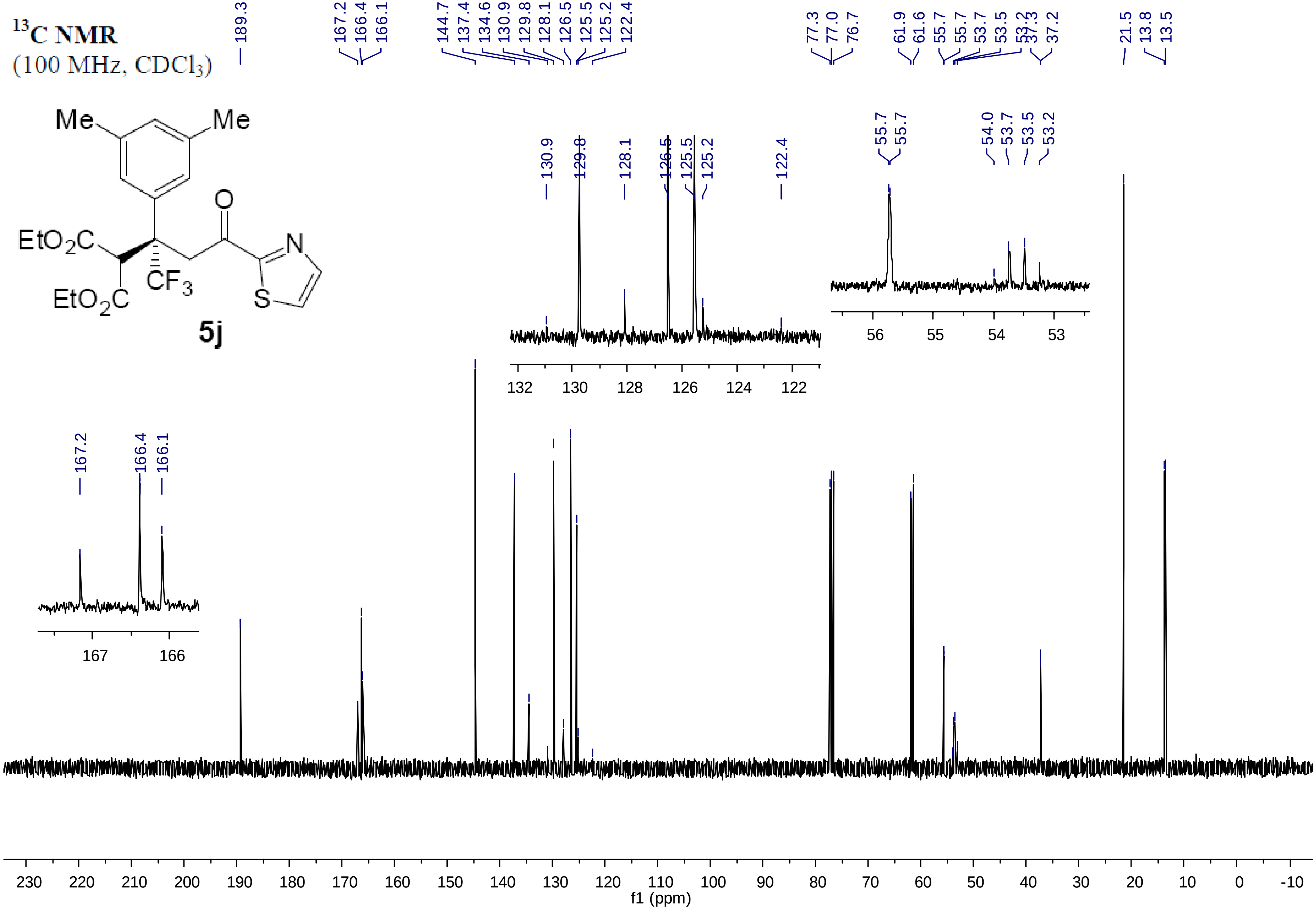

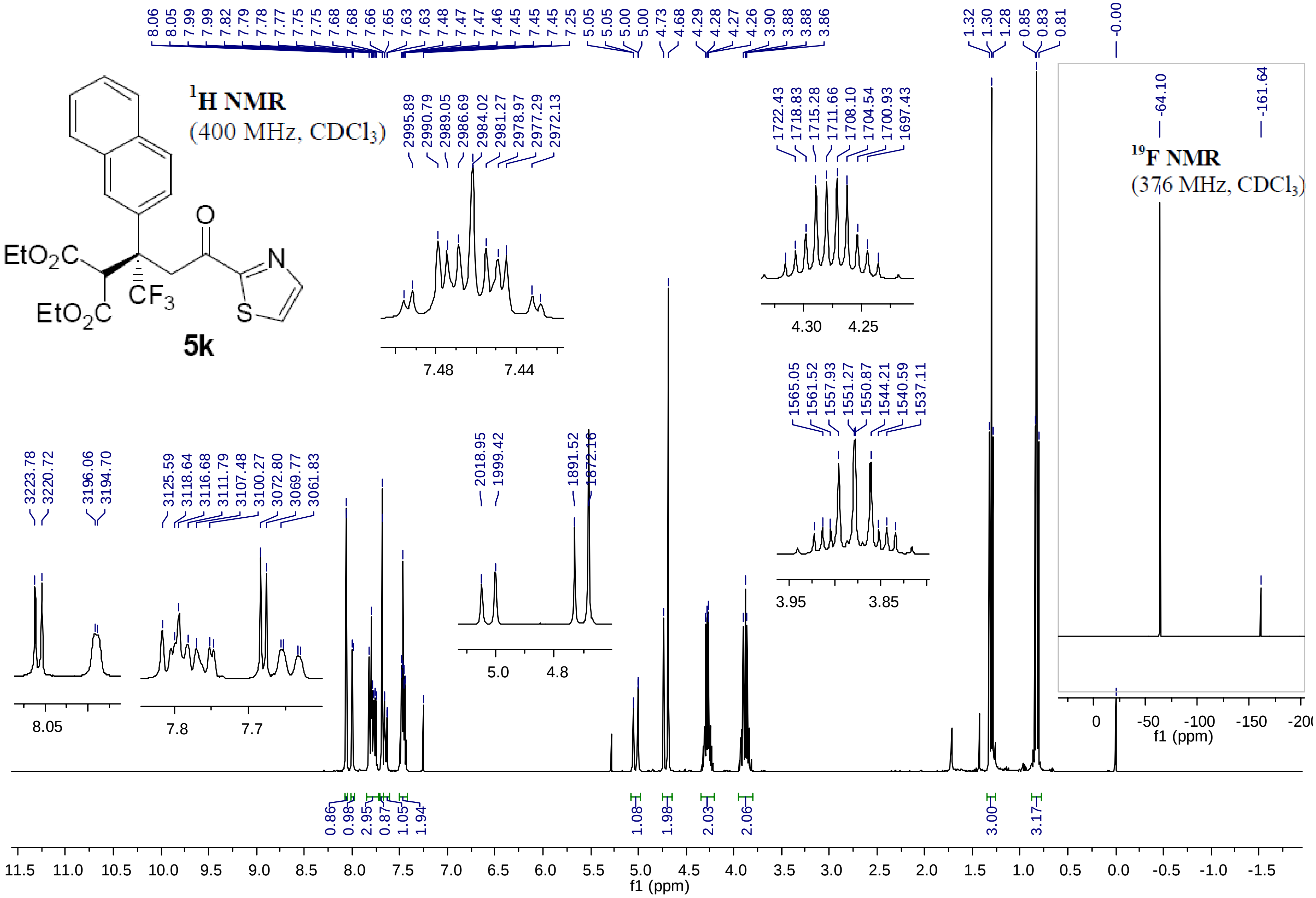

1H NMR
(400 MHz, CDCl3)
19F NMR
(376 MHz, CDCl3)
5k
-64.10
-161.64
f1 (ppm)

^{13}C NMR
(100 MHz, $CDCl_3$)

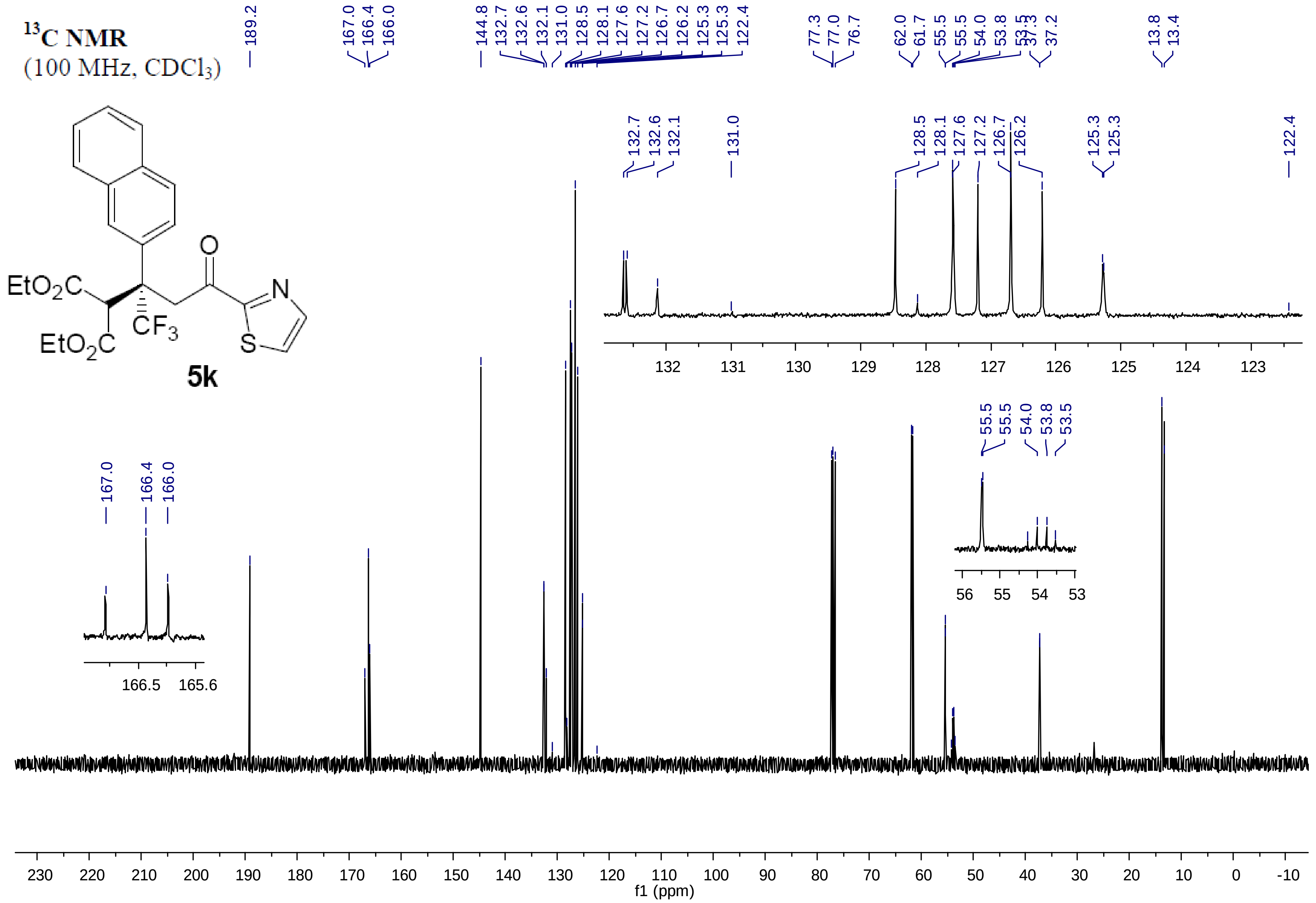

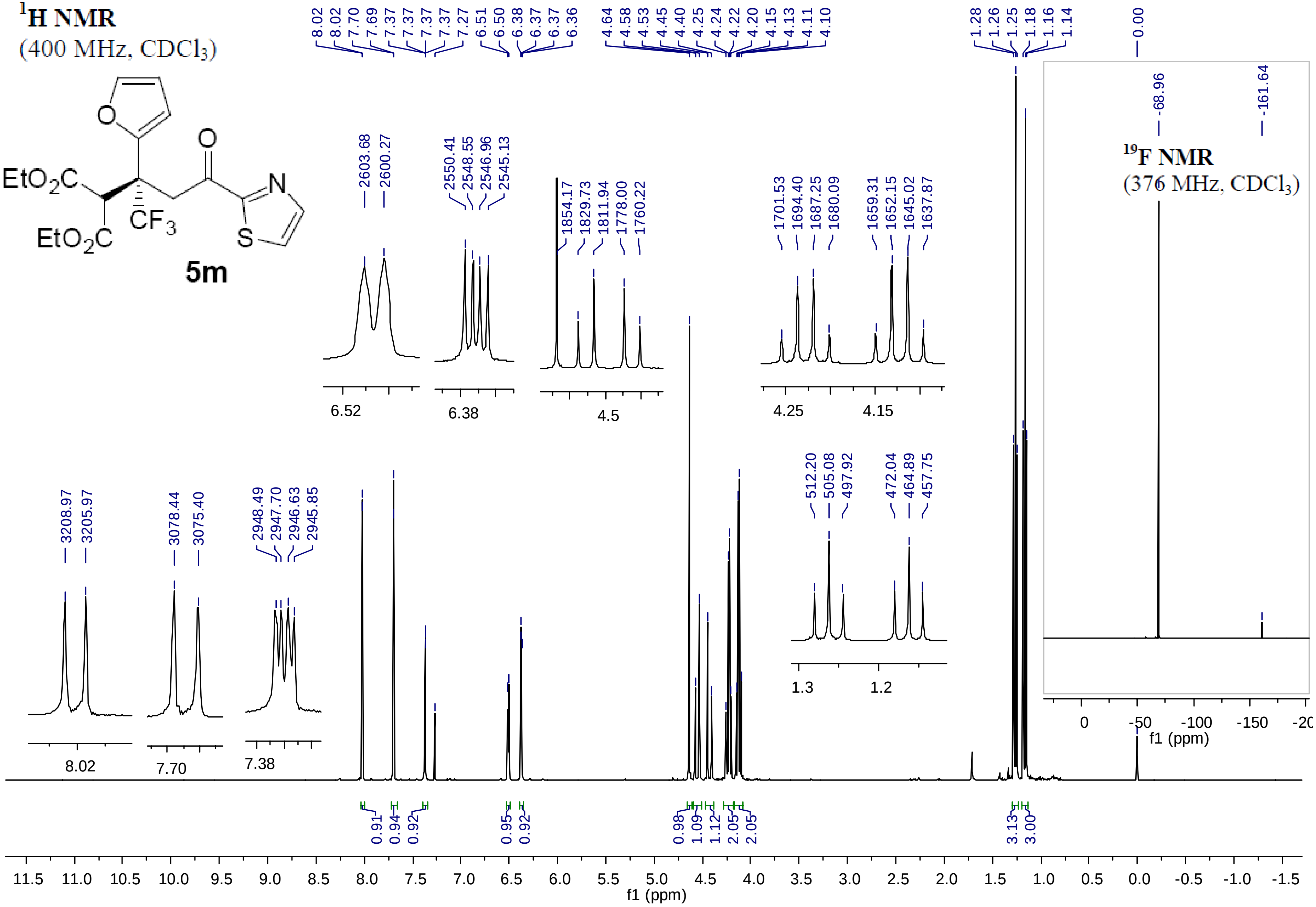

1H NMR
(400 MHz, CDCl3)
5m
19F NMR
(376 MHz, CDCl3)
-68.96
-161.64
f1 (ppm)

^{13}C NMR
(100 MHz, $CDCl_3$)

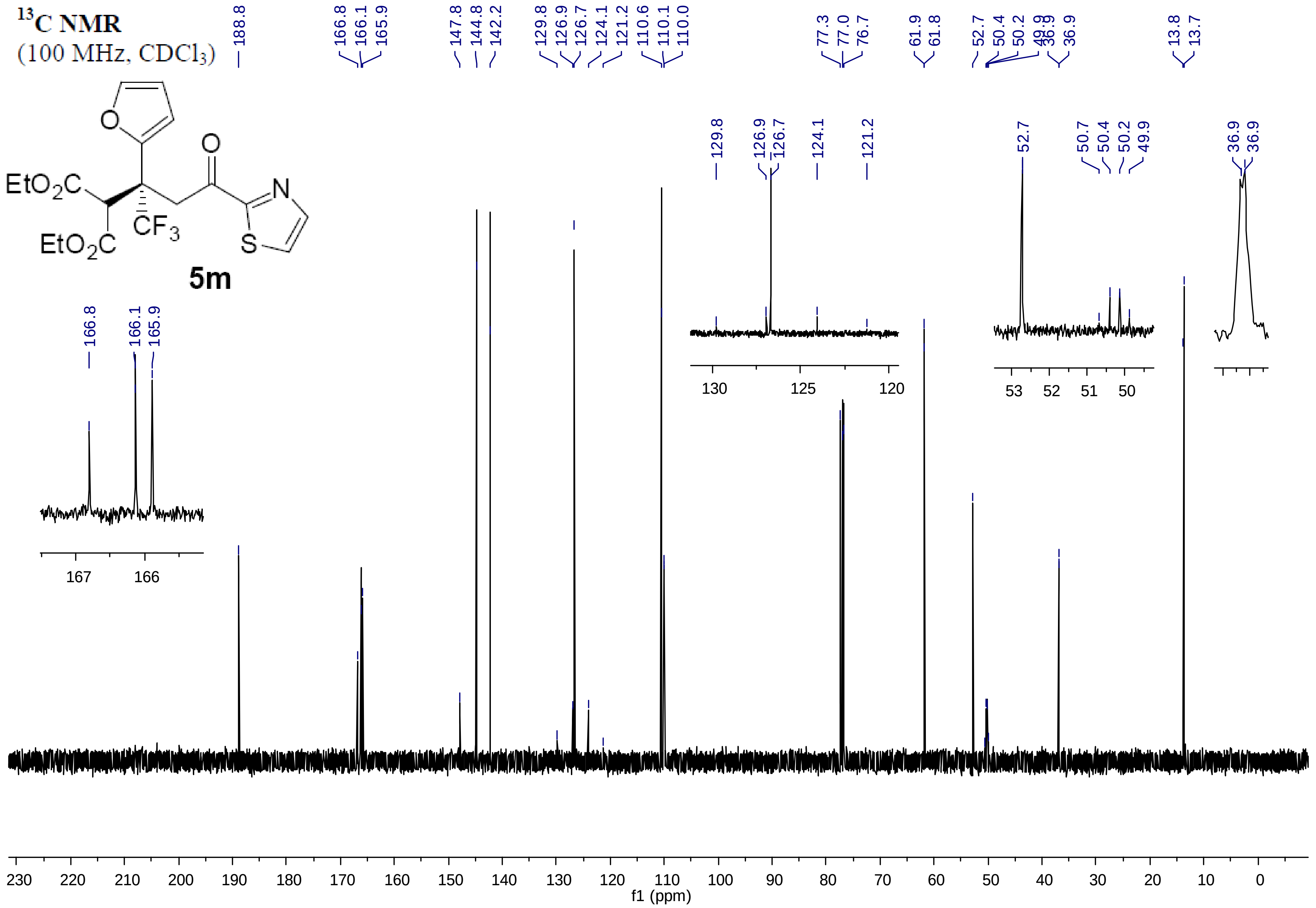

^{1}H NMR
(400 MHz, $CDCl_3$)

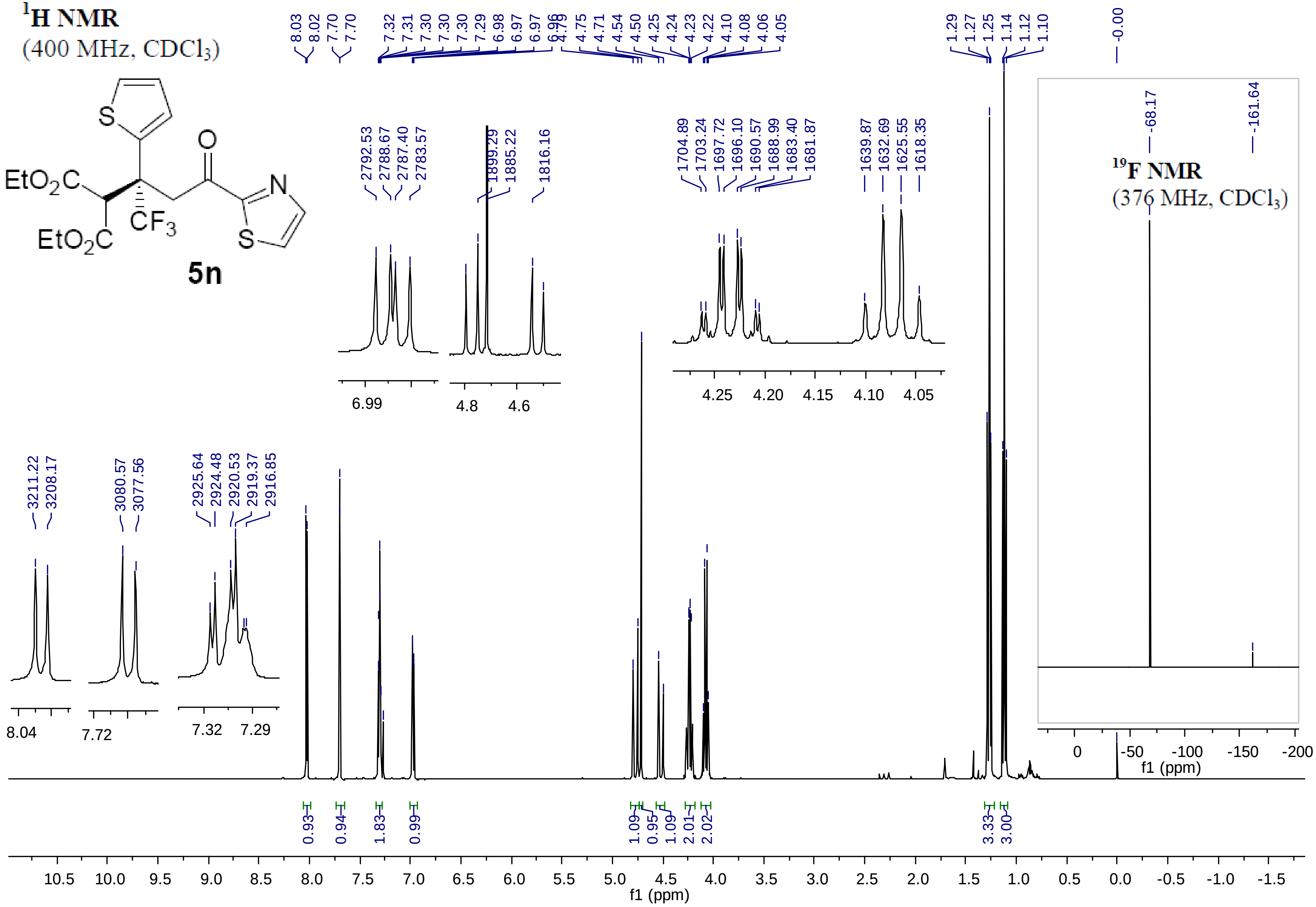

^{13}C NMR
(100 MHz, $CDCl_3$)

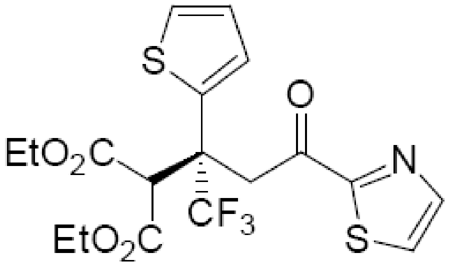

5n

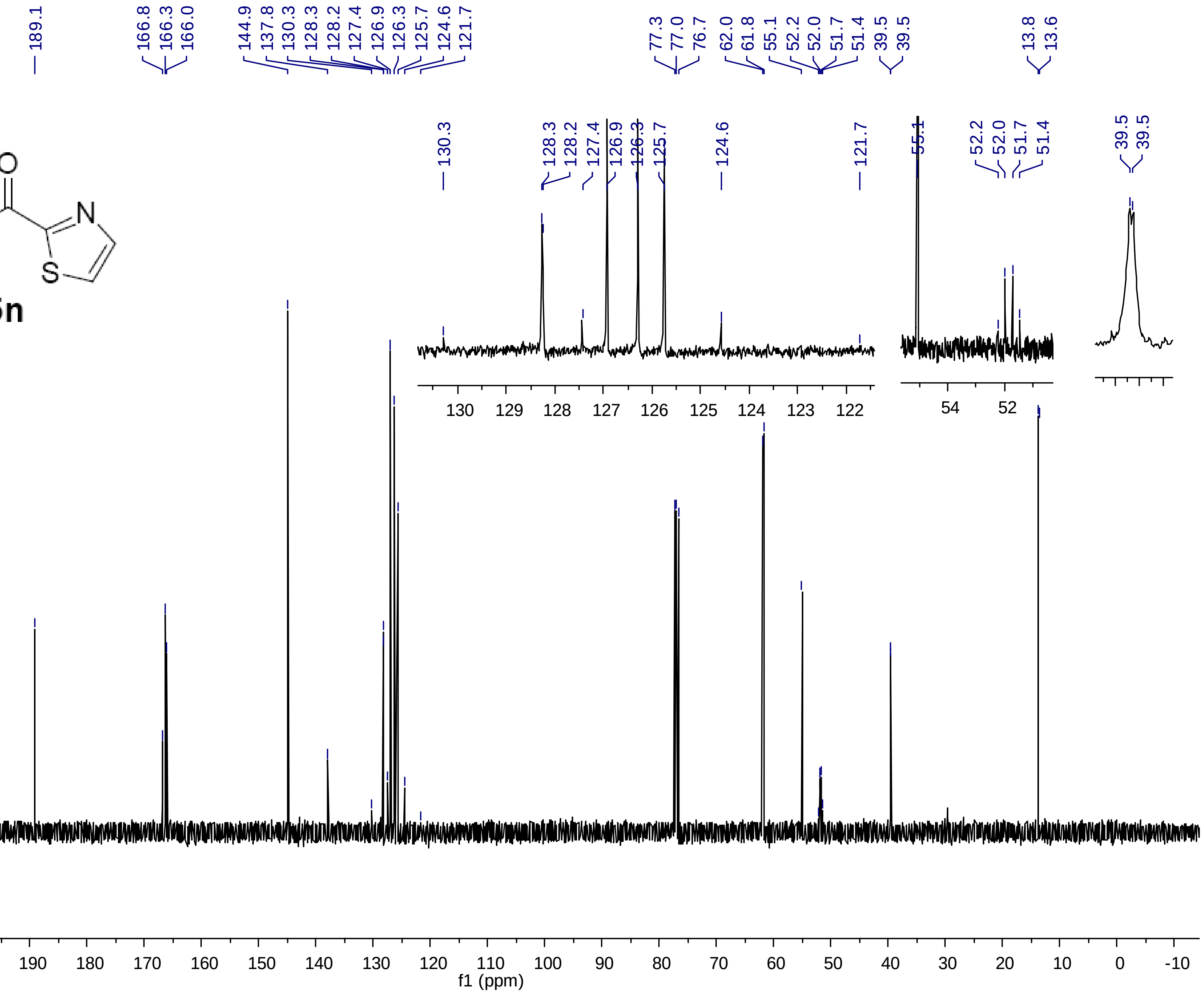

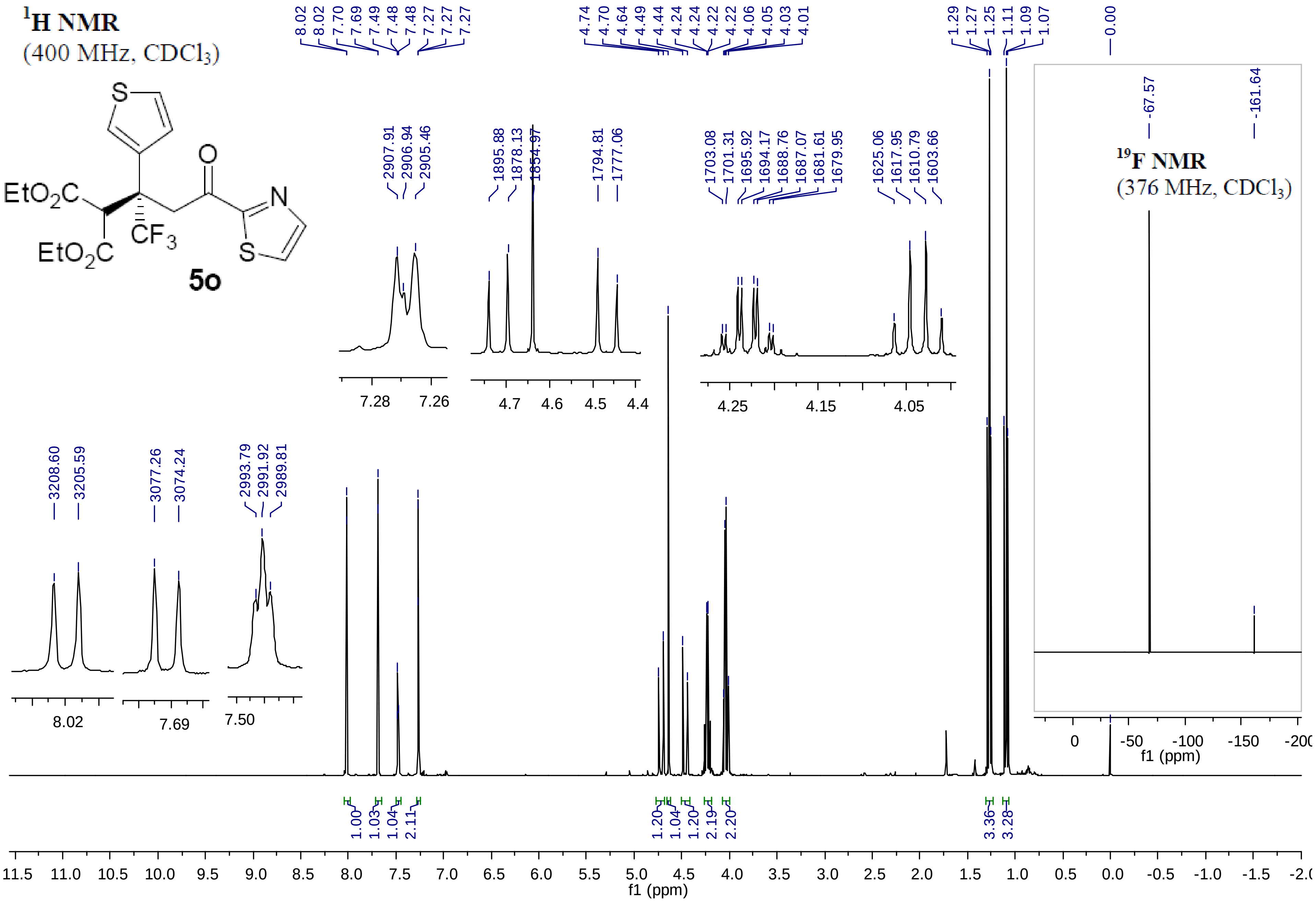

1H NMR
(400 MHz, CDCl3)
EtO2C
EtO2C
CF3
S
O
N
S
5o
19F NMR
(376 MHz, CDCl3)
-67.57
-161.64
f1 (ppm)

^{13}C NMR
(100 MHz, $CDCl_3$)

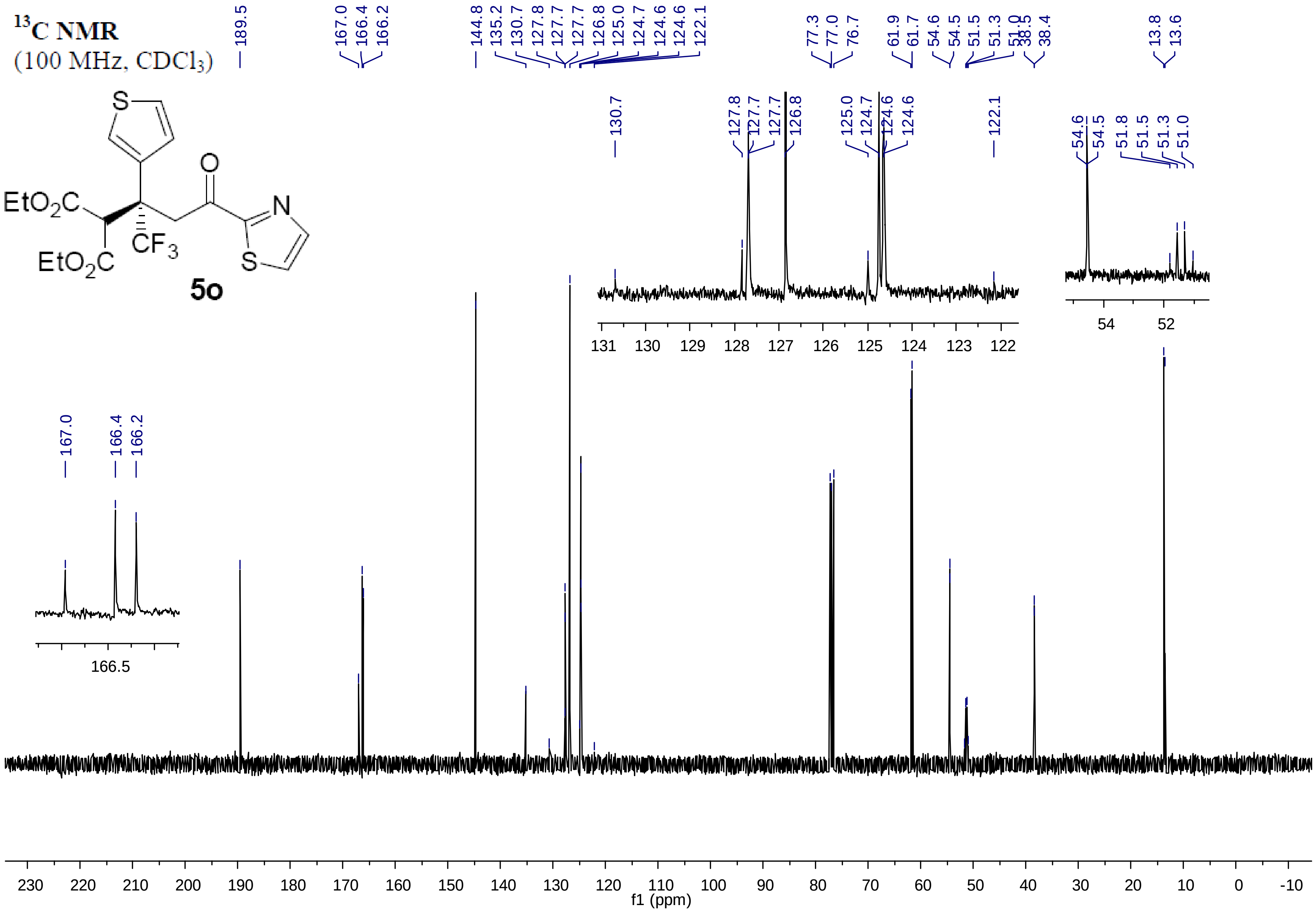

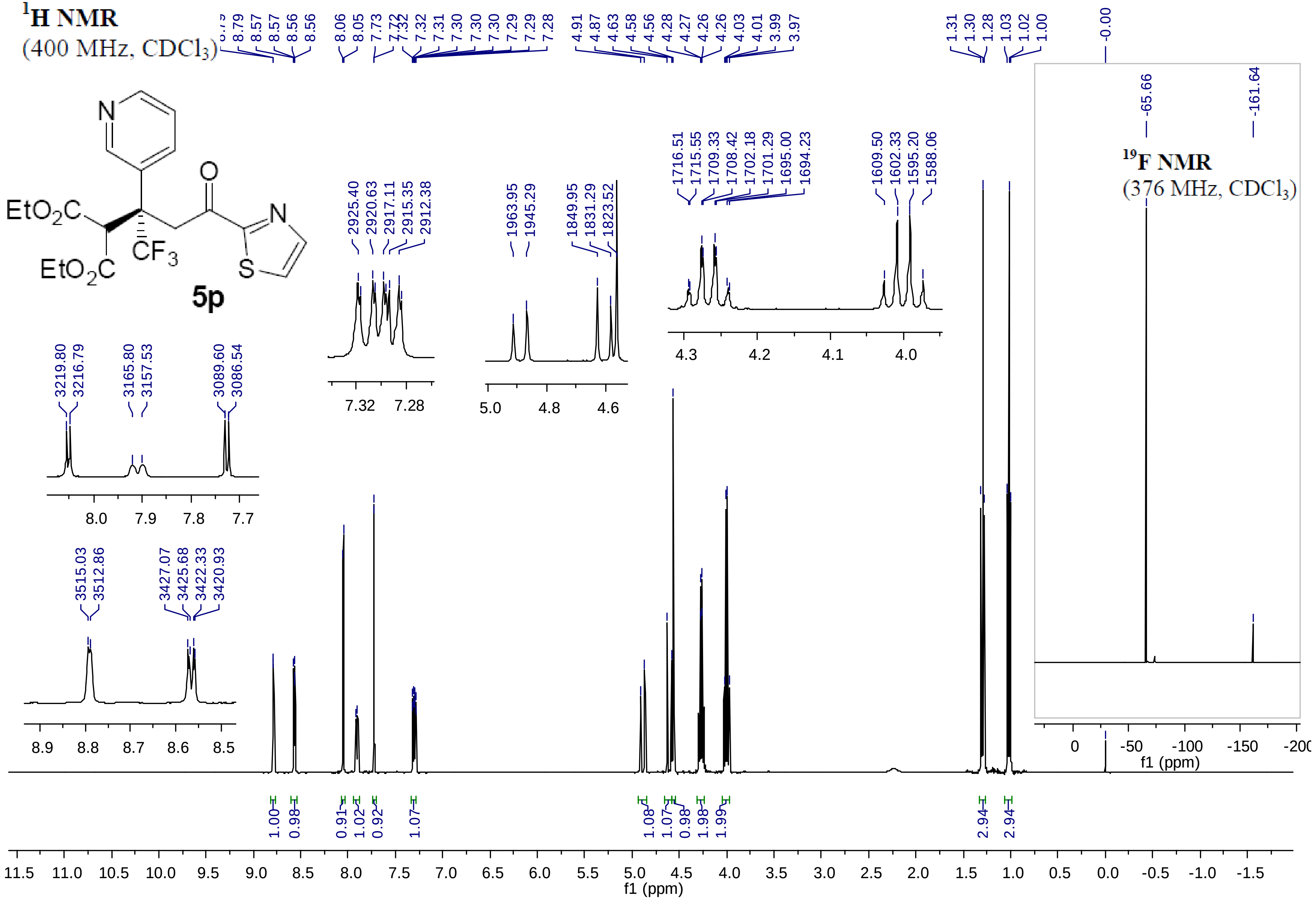

^{1}H NMR
(400 MHz, CDCl$_3$)
^{19}F NMR
(376 MHz, CDCl$_3$)
5p
EtO$_2$C
EtO$_2$C
CF$_3$
f1 (ppm)

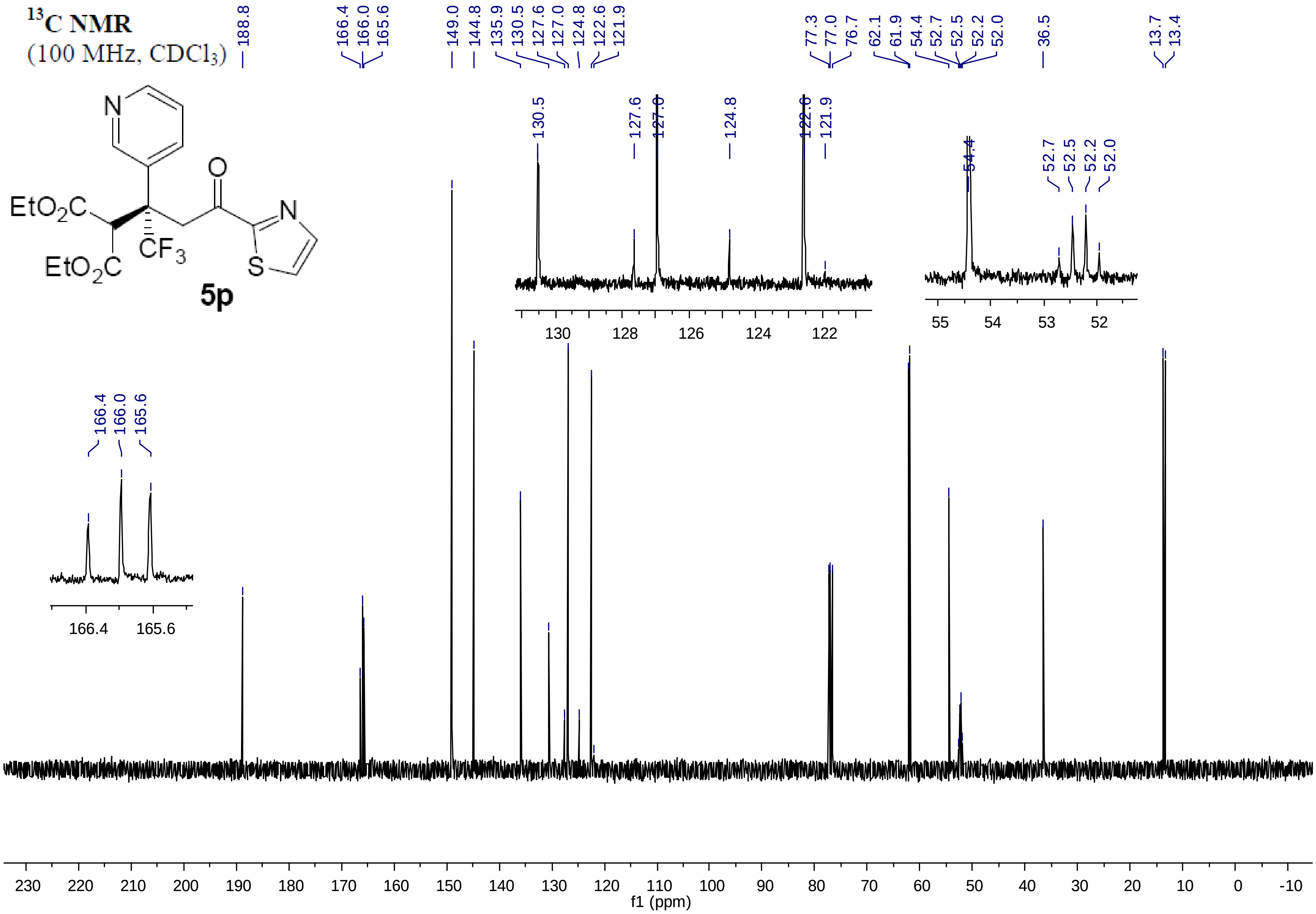

13C NMR
(100 MHz, CDCl3)
188.8
166.4
166.0
165.6
149.0
144.8
135.9
130.5
127.6
127.0
124.8
122.6
121.9
77.3
77.0
76.7
62.1
61.9
54.4
52.7
52.5
52.2
52.0
36.5
13.7
13.4
EtO2C
EtO2C
CF3
N
O
N
S
5p
f1 (ppm)

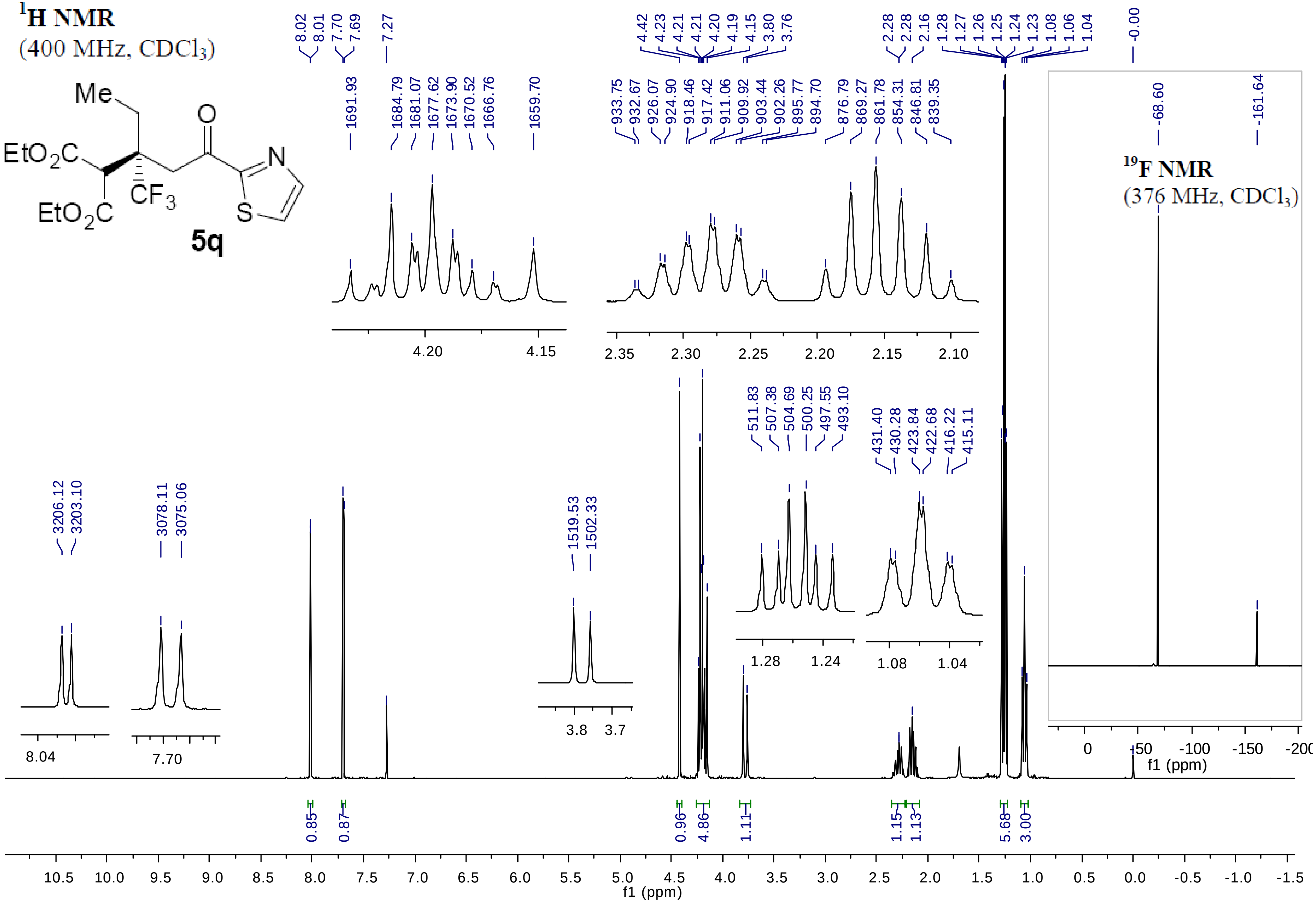

1H NMR
(400 MHz, CDCl3)
5q
19F NMR
(376 MHz, CDCl3)
-68.60
-161.64
f1 (ppm)

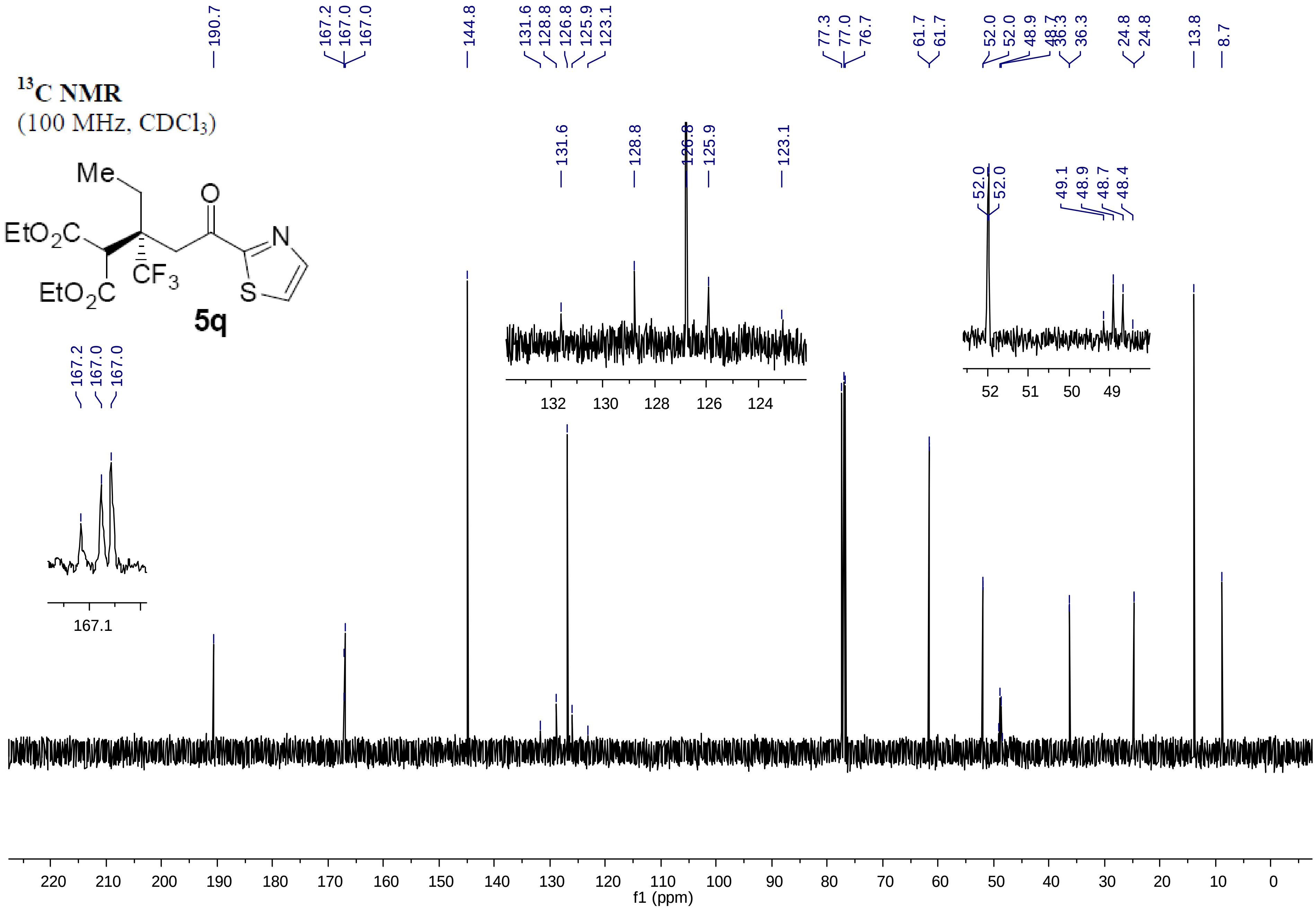

^{13}C NMR
(100 MHz, CDCl$_3$)
5q
190.7
167.2 167.0 167.0
144.8
131.6 128.8 126.8 125.9 123.1
77.3 77.0 76.7
61.7 61.7
52.0 52.0 48.9 48.7 36.3
24.8 24.8
13.8
8.7
49.1 48.9 48.7 48.4
167.1
f1 (ppm)

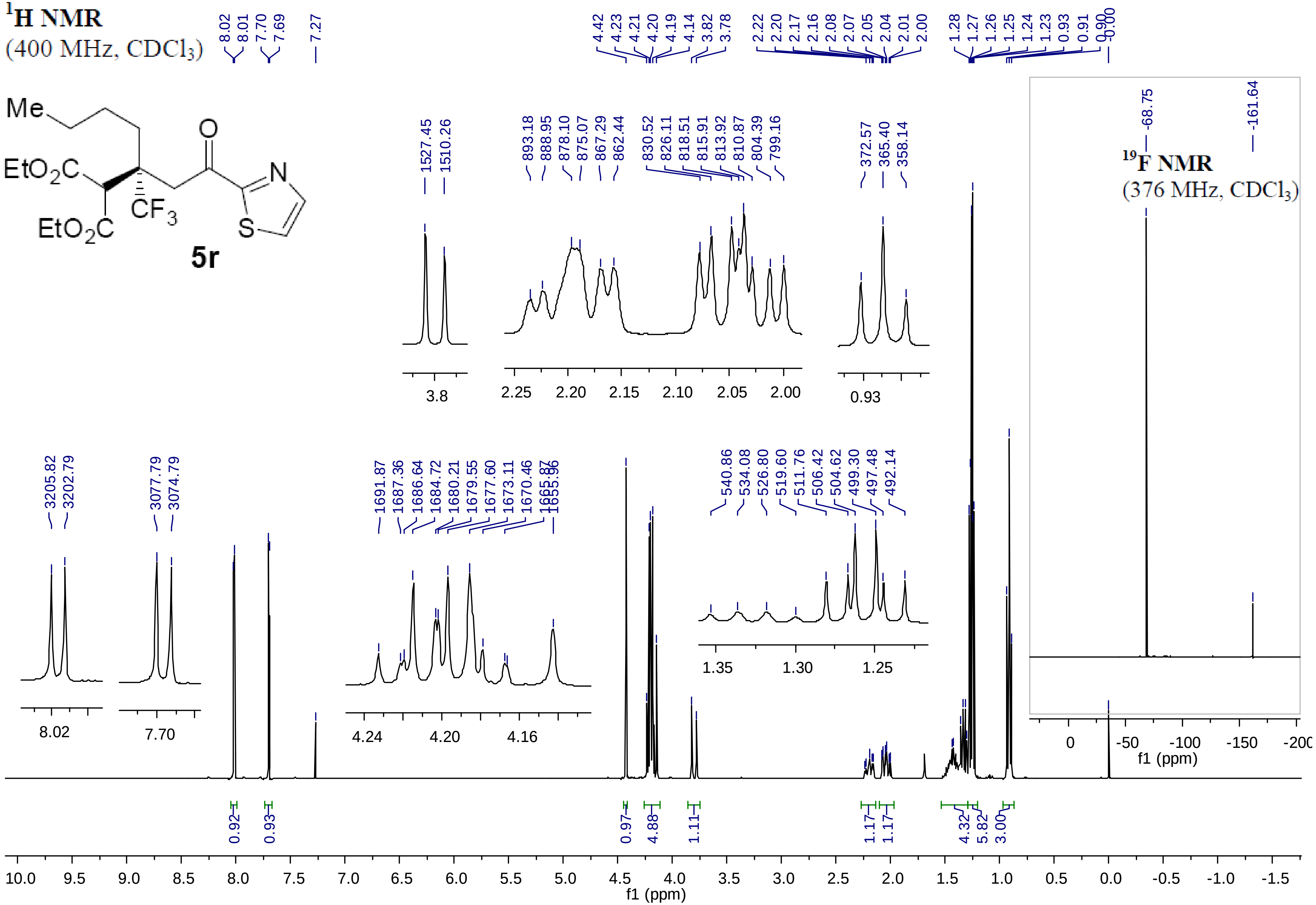

1H NMR
(400 MHz, CDCl3)
8.02 8.01 7.70 7.69 7.27
4.42 4.23 4.21 4.20 4.19 4.14 3.82 3.78
2.22 2.20 2.17 2.16 2.08 2.07 2.05 2.04 2.01 2.00
1.28 1.27 1.26 1.25 1.24 1.23 0.93 0.91 0.90 0.00
Me
EtO2C
EtO2C
CF3
O
N
S
5r
1527.45 1510.26
893.18 888.95 878.10 875.07 867.29 862.44
830.52 826.11 818.51 815.91 813.92 810.87 804.39 799.16
372.57 365.40 358.14
3.8
2.25 2.20 2.15 2.10 2.05 2.00
0.93
19F NMR
(376 MHz, CDCl3)
-68.75
-161.64
0 -50 -100 -150 -200
f1 (ppm)
3205.82 3202.79
3077.79 3074.79
1691.87 1687.36 1686.64 1684.72 1680.21 1679.55 1677.60 1673.11 1670.46 1665.87 1665.96
540.86 534.08 526.80 519.60 511.76 506.42 504.62 499.30 497.48 492.14
8.02
7.70
4.24 4.20 4.16
1.35 1.30 1.25
0.92 0.93 0.97 4.88 1.11 1.17 1.17 4.32 5.82 3.00
10.0 9.5 9.0 8.5 8.0 7.5 7.0 6.5 6.0 5.5 5.0 4.5 4.0 3.5 3.0 2.5 2.0 1.5 1.0 0.5 0.0 -0.5 -1.0 -1.5
f1 (ppm)

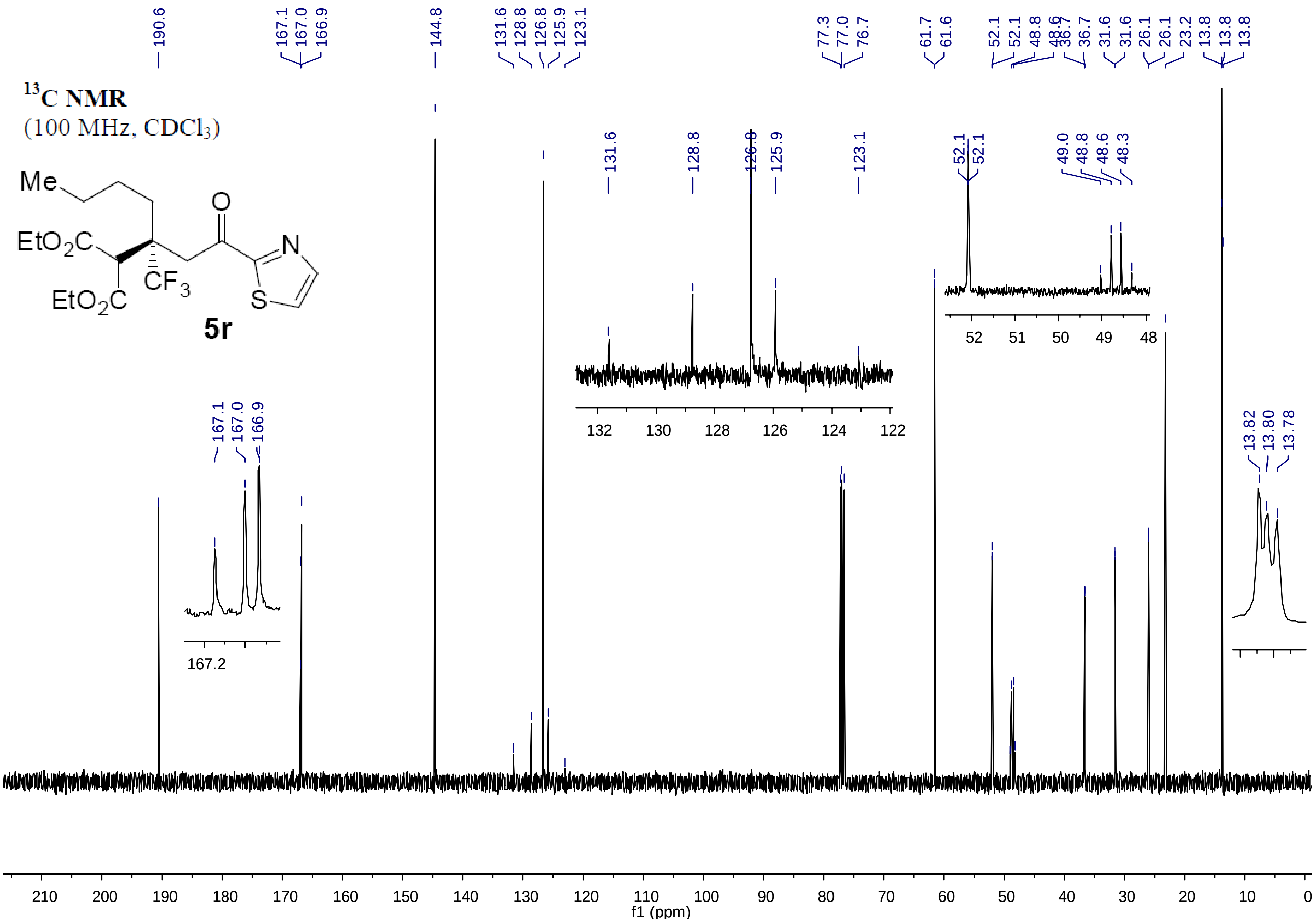

13C NMR
(100 MHz, CDCl3)
Me
EtO2C
EtO2C
O
N
S
CF3
5r
190.6
167.1
167.0
166.9
144.8
131.6
128.8
126.8
125.9
123.1
77.3
77.0
76.7
61.7
61.6
52.1
52.1
48.8
48.6
36.7
31.6
31.6
26.1
26.1
23.2
13.8
13.8
13.8
49.0
48.8
48.6
48.3
13.82
13.80
13.78
167.2
f1 (ppm)

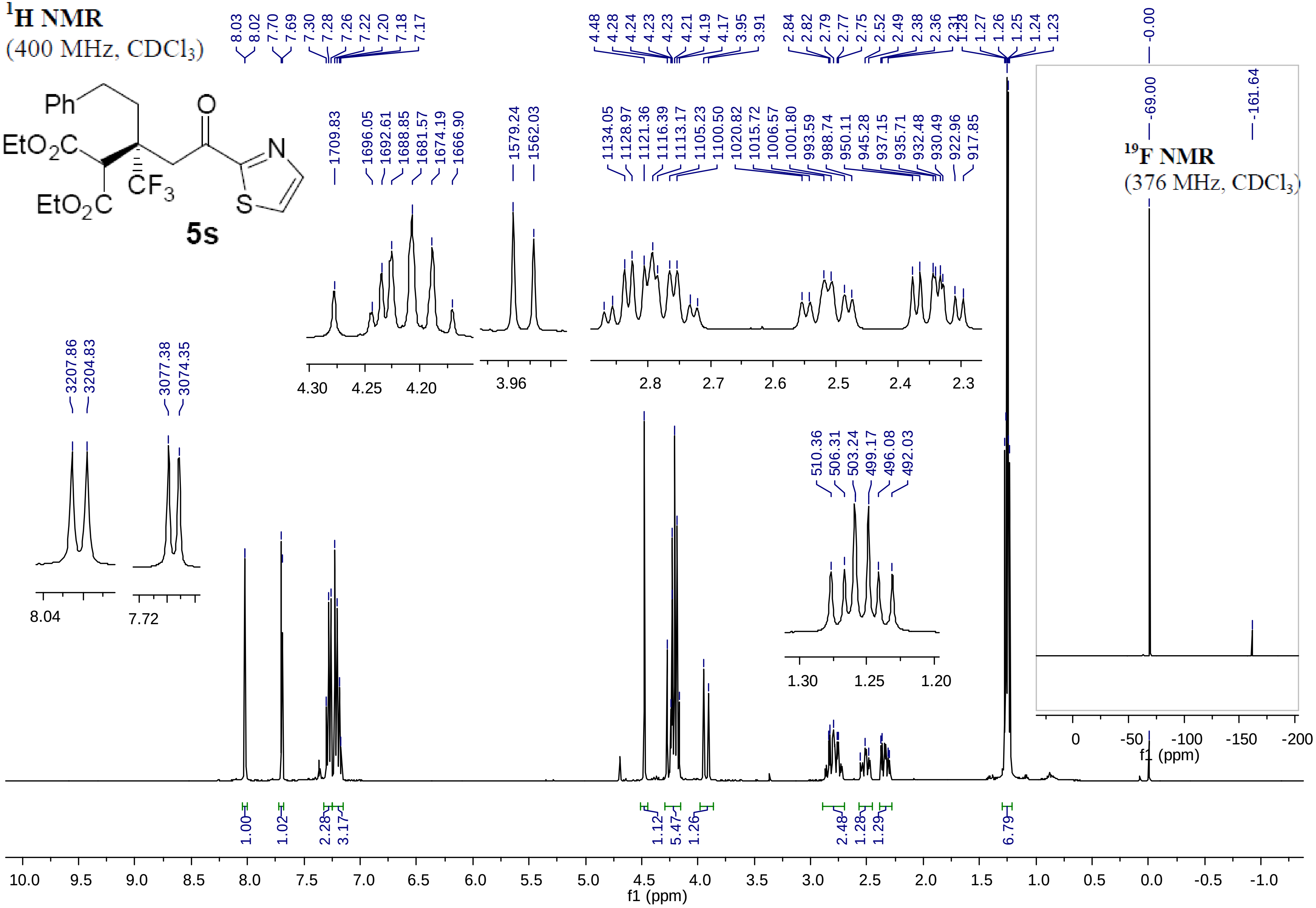

^{1}H NMR
(400 MHz, CDCl$_3$)
^{19}F NMR
(376 MHz, CDCl$_3$)
5s
f1 (ppm)

^{13}C NMR
(100 MHz, $CDCl_3$)

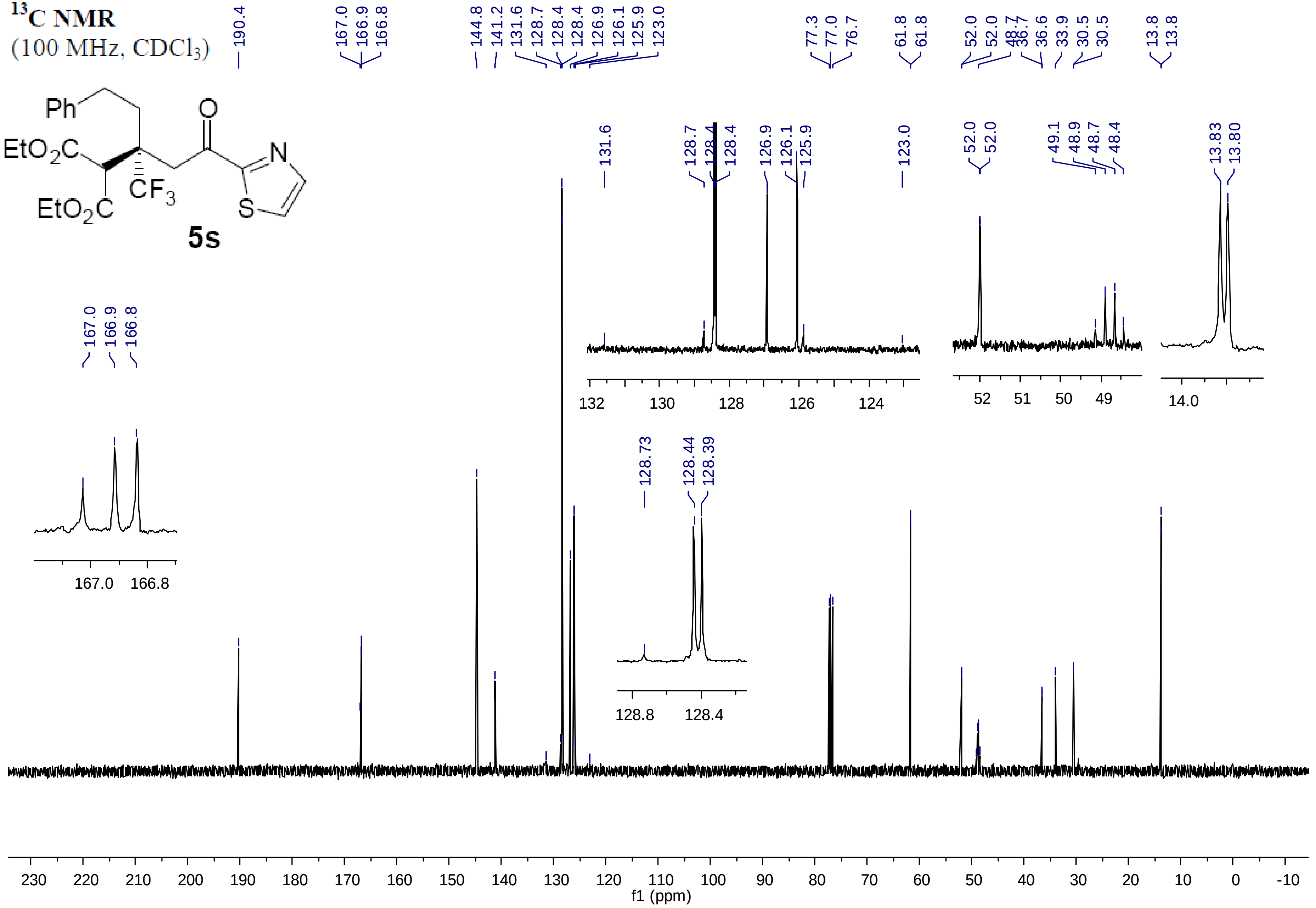

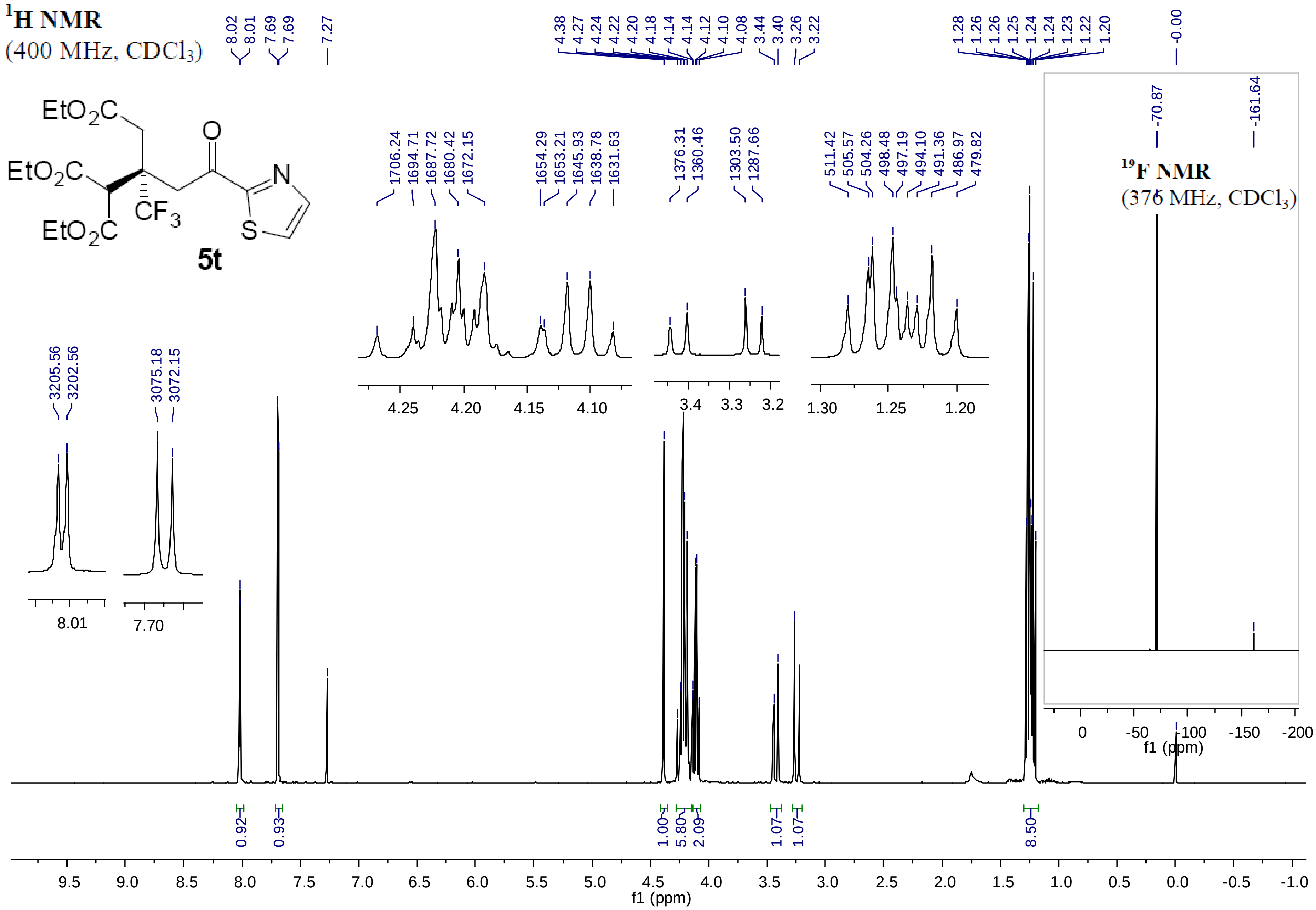

^{1}H NMR
(400 MHz, CDCl$_3$)
5t
^{19}F NMR
(376 MHz, CDCl$_3$)
-70.87
-161.64
f1 (ppm)

^{13}C NMR
(100 MHz, $CDCl_3$)

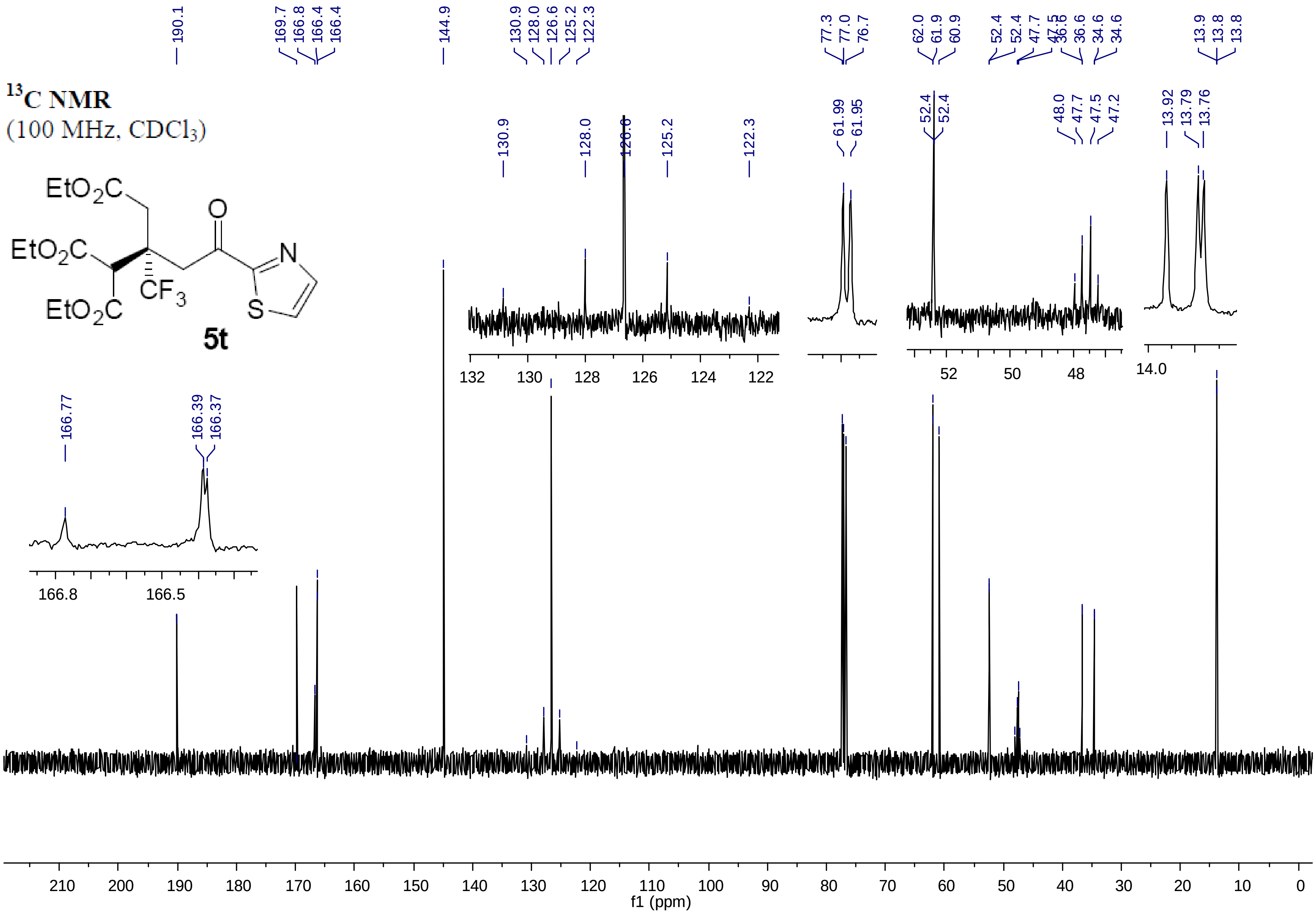

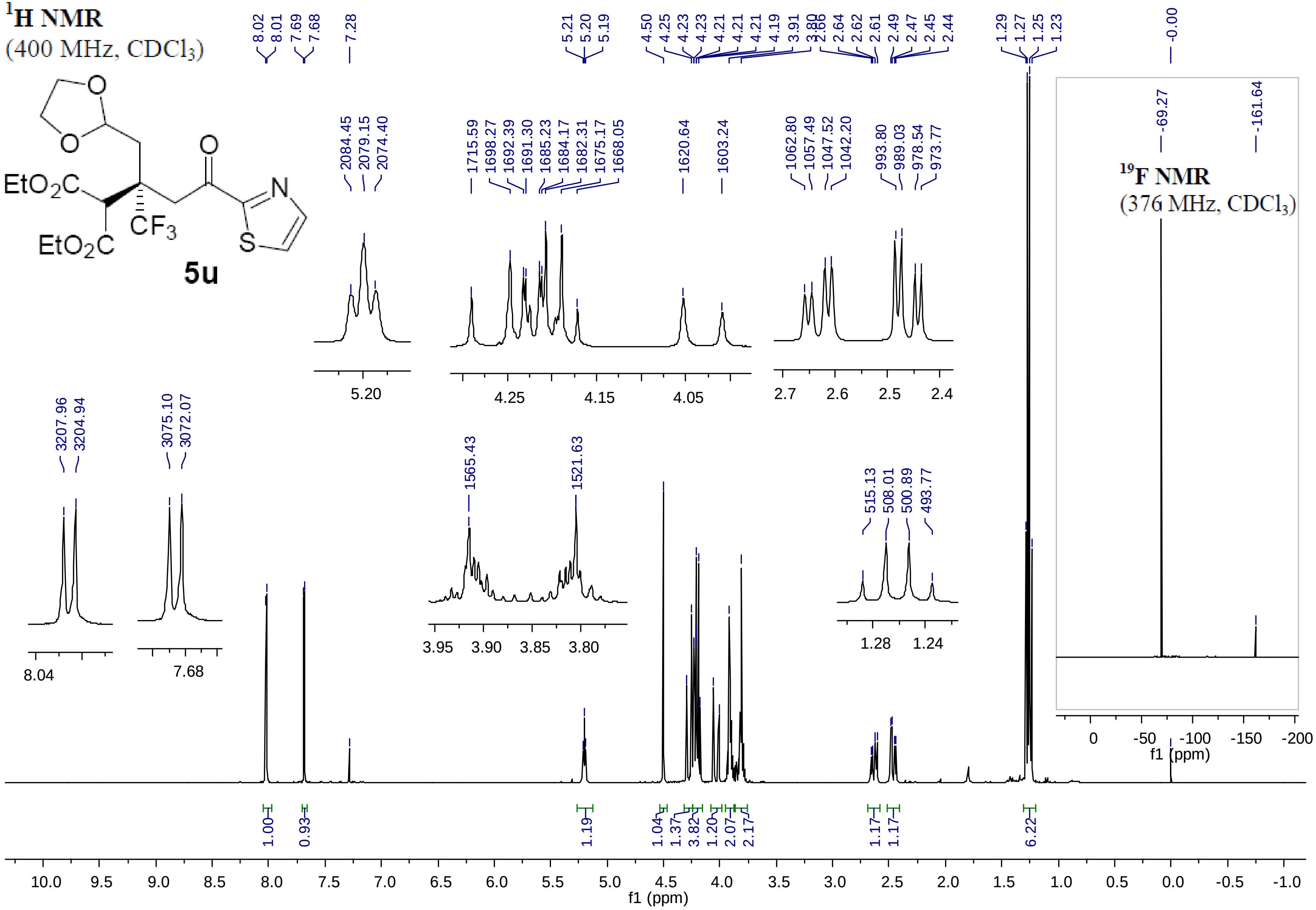

^{1}H NMR
(400 MHz, CDCl$_3$)
5u
^{19}F NMR
(376 MHz, CDCl$_3$)
-69.27
-161.64
f1 (ppm)

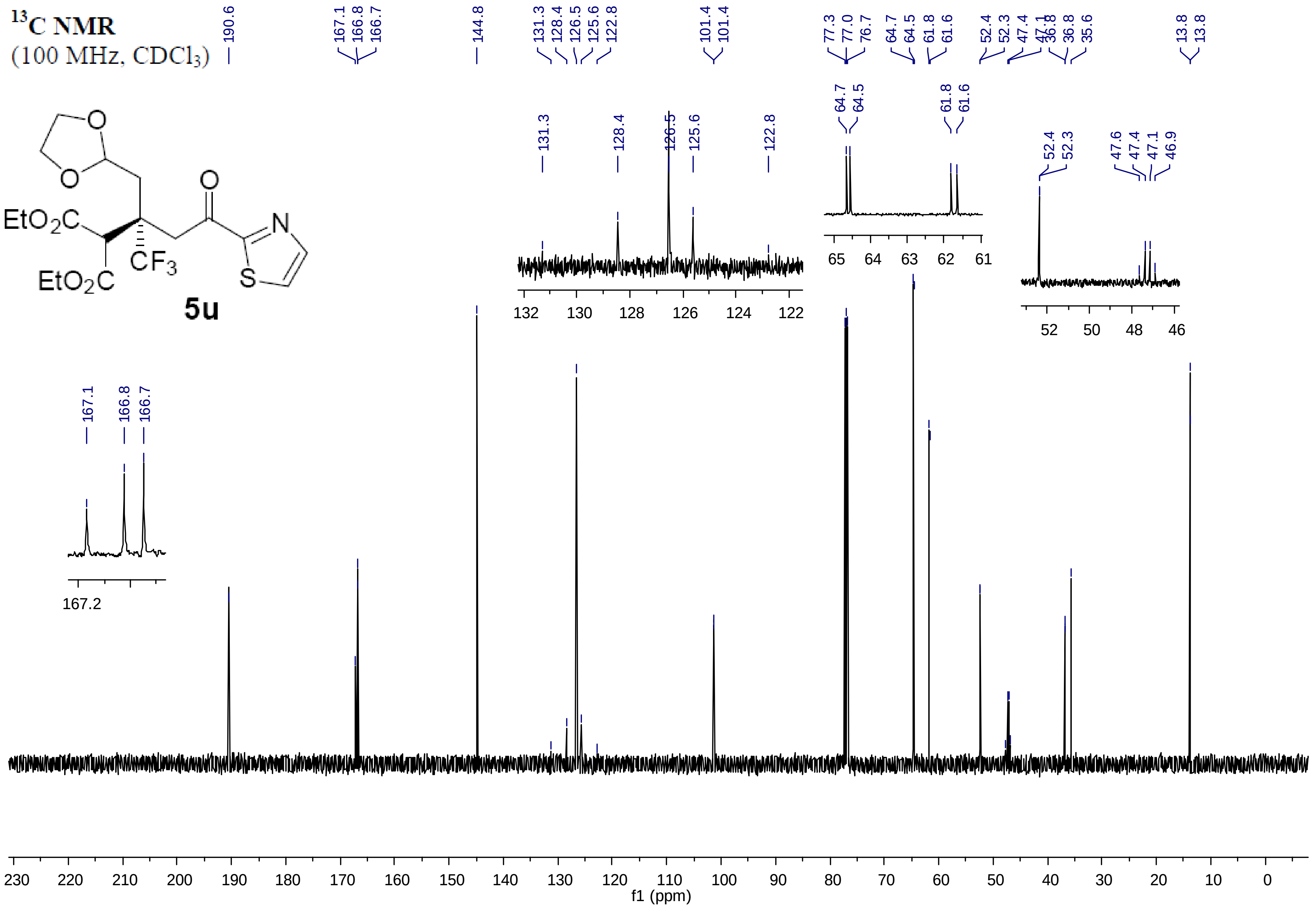

13C NMR
(100 MHz, CDCl3)
5u
190.6
167.1
166.8
166.7
144.8
131.3
128.4
126.5
125.6
122.8
101.4
101.4
77.3
77.0
76.7
64.7
64.5
61.8
61.6
52.4
52.3
47.4
47.1
36.8
36.8
35.6
13.8
13.8
f1 (ppm)

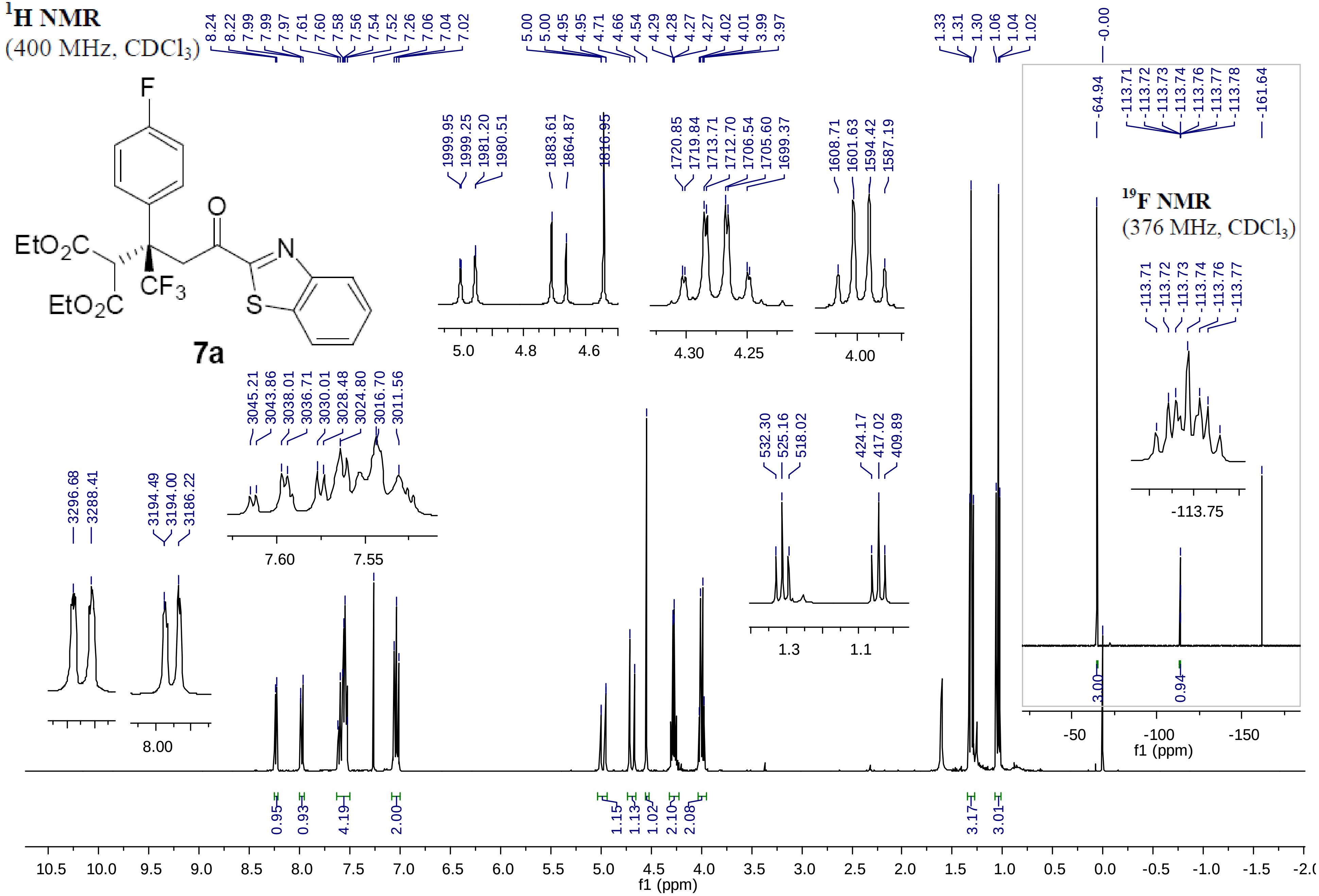

^{1}H NMR
(400 MHz, CDCl$_3$)
8.24 8.22 7.99 7.99 7.97 7.61 7.60 7.58 7.56 7.54 7.52 7.26 7.06 7.04 7.02
5.00 5.00 4.95 4.95 4.71 4.66 4.54 4.29 4.28 4.27 4.27 4.02 4.01 3.99 3.97
1.33 1.31 1.30 1.06 1.04 1.02
-0.00
F
O
EtO$_2$C
CF$_3$
N
S
EtO$_2$C
7a
1999.95 1999.25 1981.20 1980.51 1883.61 1864.87 1816.95
1720.85 1719.84 1713.71 1712.70 1706.54 1705.60 1699.37
1608.71 1601.63 1594.42 1587.19
3045.21 3043.86 3038.01 3036.71 3030.01 3028.48 3024.80 3016.70 3011.56
532.30 525.16 518.02
424.17 417.02 409.89
3296.68 3288.41
3194.49 3194.00 3186.22
^{19}F NMR
(376 MHz, CDCl$_3$)
-64.94 -113.71 -113.72 -113.73 -113.74 -113.76 -113.77 -113.78 -161.64
-113.71 -113.72 -113.73 -113.74 -113.76 -113.77
-113.75
3.00 0.94
-50 -100 -150
f1 (ppm)
0.95 0.93 4.19 2.00 1.15 1.13 1.02 2.10 2.08 3.17 3.01
10.5 10.0 9.5 9.0 8.5 8.0 7.5 7.0 6.5 6.0 5.5 5.0 4.5 4.0 3.5 3.0 2.5 2.0 1.5 1.0 0.5 0.0 -0.5 -1.0 -1.5
f1 (ppm)

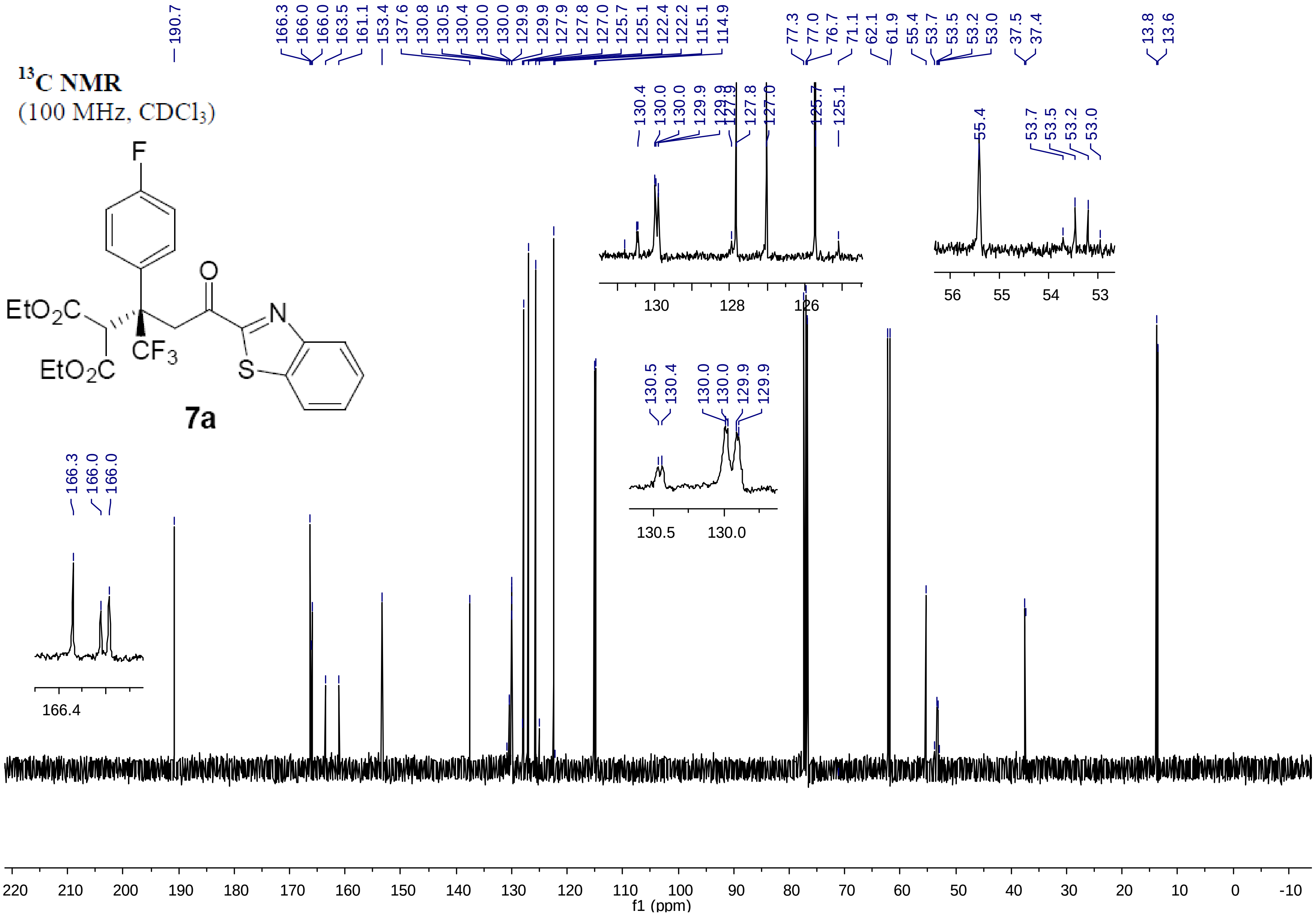

13C NMR
(100 MHz, CDCl3)
7a
190.7
166.3
166.0
166.0
163.5
161.1
153.4
137.6
130.8
130.5
130.4
130.0
130.0
129.9
129.9
127.9
127.8
127.0
125.7
125.1
122.4
122.2
115.1
114.9
77.3
77.0
76.7
71.1
62.1
61.9
55.4
53.7
53.5
53.2
53.0
37.5
37.4
13.8
13.6
f1 (ppm)

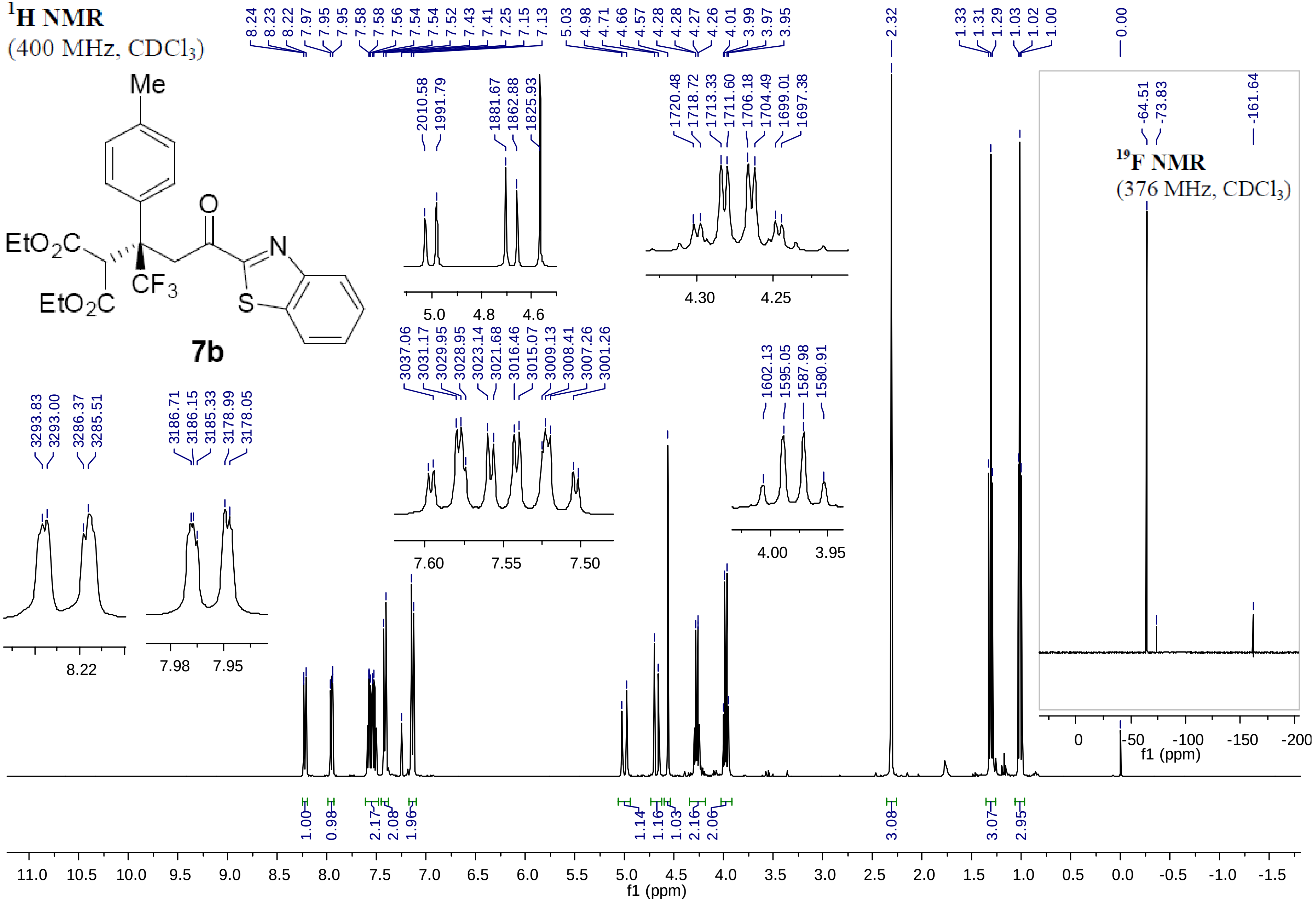

1H NMR
(400 MHz, CDCl3)
7b
8.24 8.23 8.22 7.97 7.95 7.95 7.58 7.58 7.56 7.54 7.54 7.52 7.43 7.41 7.25 7.15 7.13
5.03 4.98 4.71 4.66 4.57 4.28 4.28 4.27 4.26 4.01 3.99 3.97 3.95
2.32
1.33 1.31 1.29 1.03 1.02 1.00
0.00
2010.58 1991.79 1881.67 1862.88 1825.93
1720.48 1718.72 1713.33 1711.60 1706.18 1704.49 1699.01 1697.38
3293.83 3293.00 3286.37 3285.51
3186.71 3186.15 3185.33 3178.99 3178.05
3037.06 3031.17 3029.95 3028.95 3023.14 3021.68 3016.46 3015.07 3009.13 3008.41 3007.26 3001.26
1602.13 1595.05 1587.98 1580.91
19F NMR
(376 MHz, CDCl3)
-64.51 -73.83 -161.64
1.00 0.98 2.17 2.08 1.96 1.14 1.16 1.03 2.16 2.06 3.08 3.07 2.95
f1 (ppm)

^{13}C NMR
(100 MHz, $CDCl_3$)

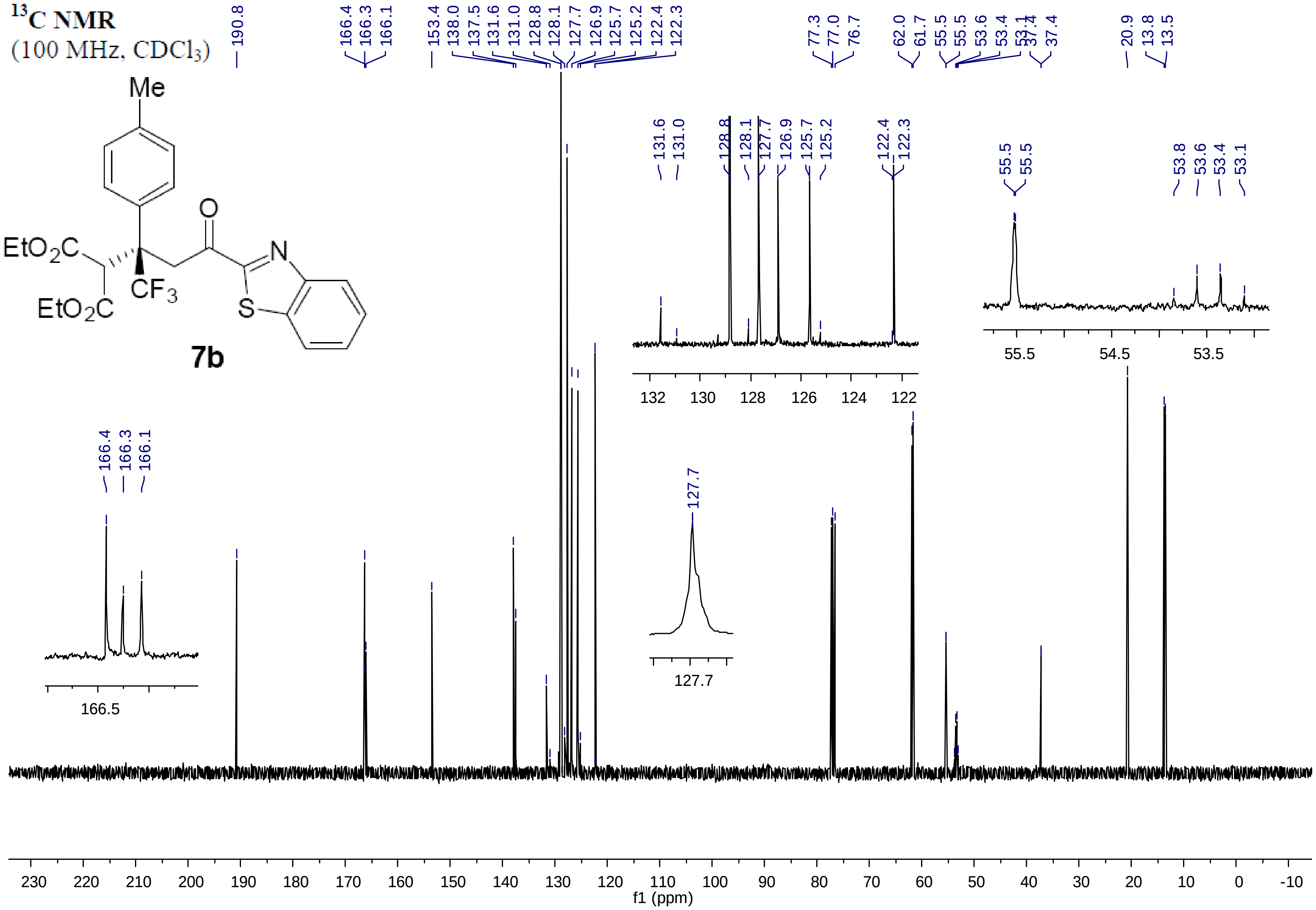

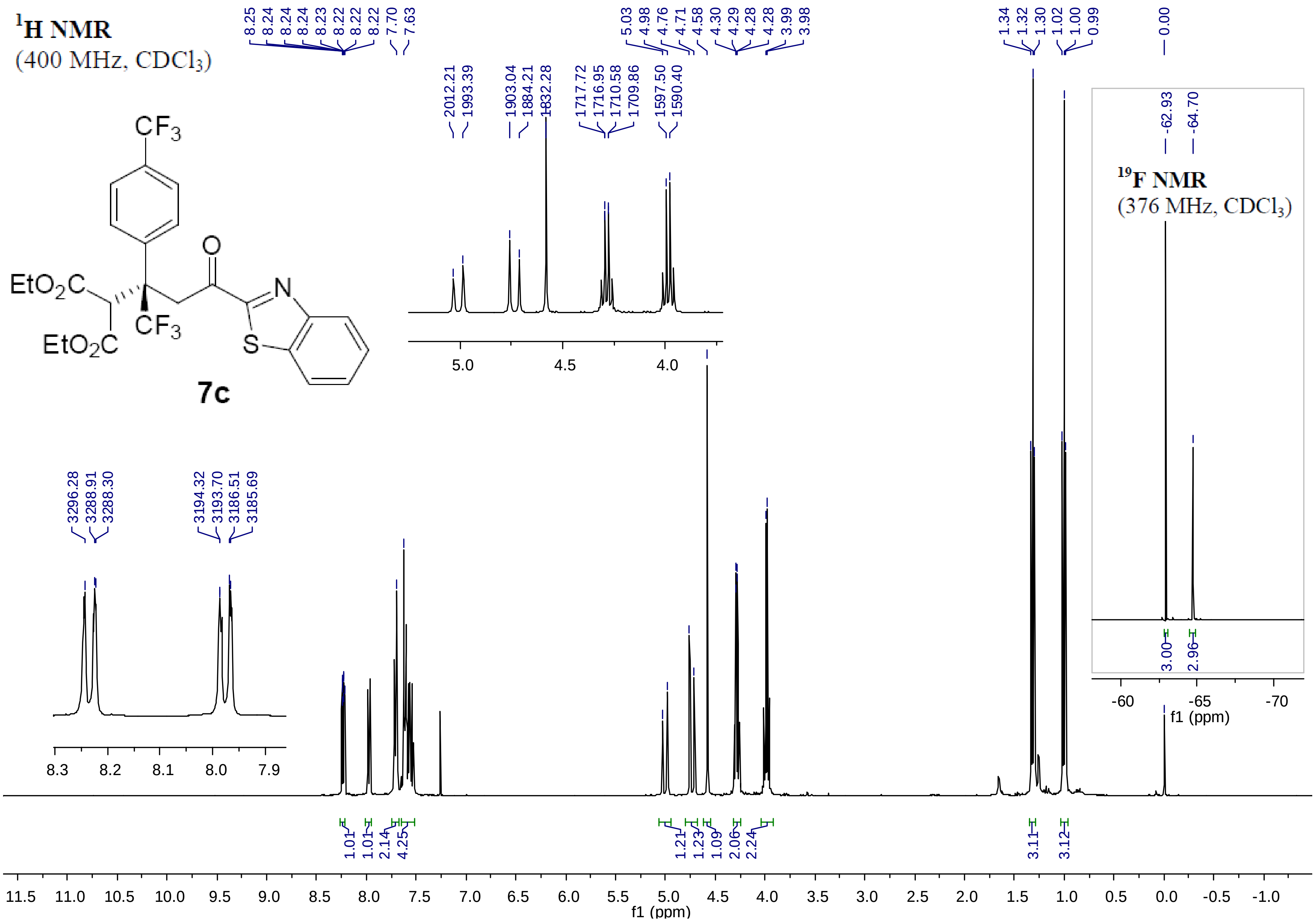

1H NMR
(400 MHz, CDCl3)
CF3
O
EtO2C
N
CF3
S
EtO2C
7c
19F NMR
(376 MHz, CDCl3)
-62.93
-64.70
3.00
2.96
-60
-65
-70
f1 (ppm)
1.01
1.01
2.14
4.25
1.21
1.23
1.09
2.06
2.24
3.11
3.12
f1 (ppm)

^{13}C NMR

(100 MHz, $CDCl_3$)

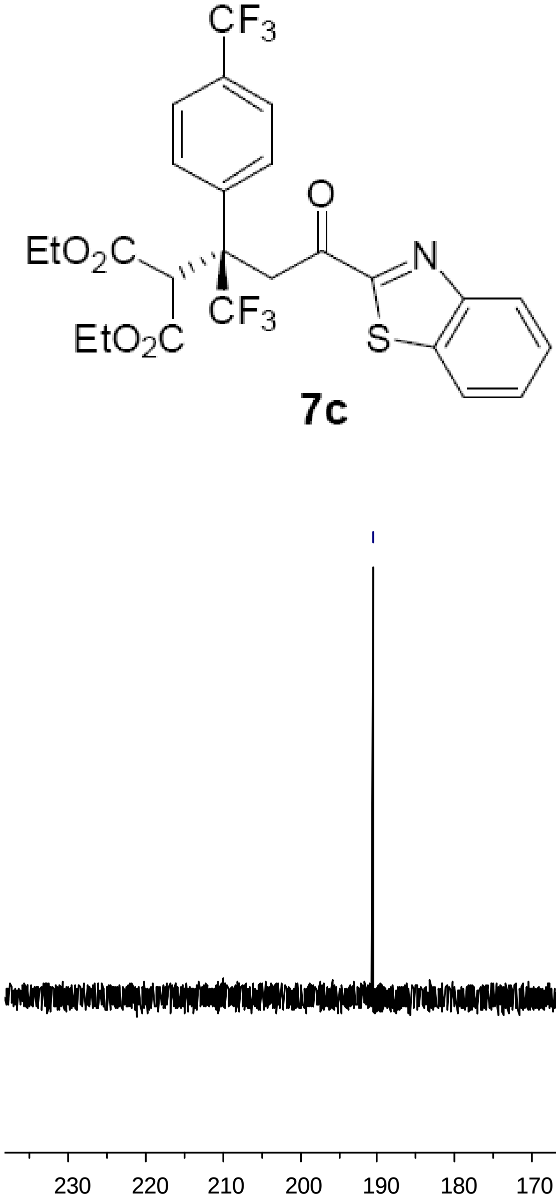

7c

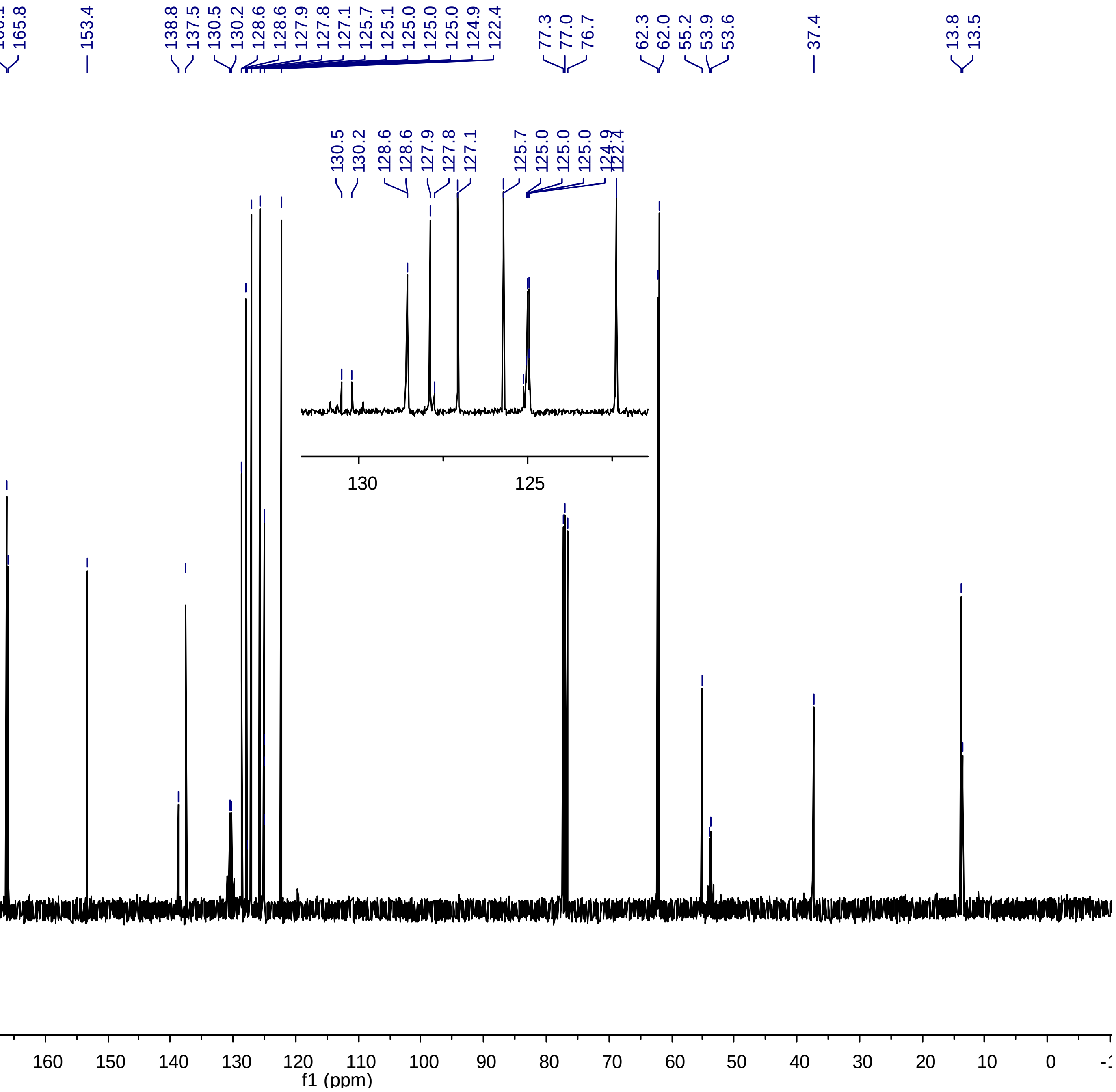

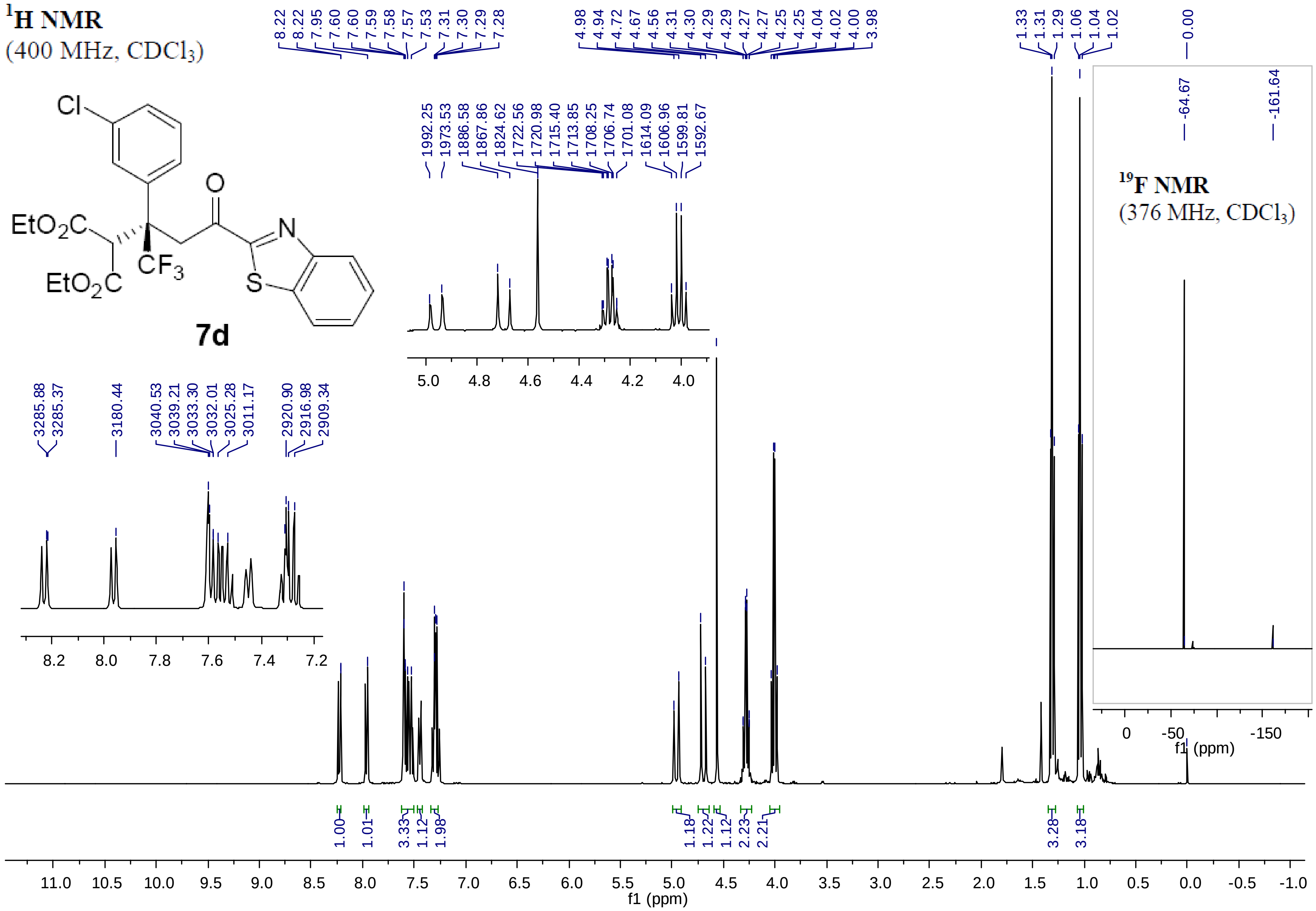

1H NMR
(400 MHz, CDCl3)
7d
19F NMR
(376 MHz, CDCl3)
8.22 8.22 7.95 7.60 7.60 7.59 7.58 7.57 7.53 7.31 7.30 7.29 7.28
4.98 4.94 4.72 4.67 4.56 4.31 4.30 4.29 4.29 4.27 4.27 4.25 4.25 4.04 4.02 4.00 3.98
1.33 1.31 1.29 1.06 1.04 1.02
0.00
-64.67
-161.64
1.00 1.01 3.33 1.12 1.98 1.18 1.22 1.12 2.23 2.21 3.28 3.18
f1 (ppm)

^{13}C NMR

(100 MHz, $CDCl_3$)

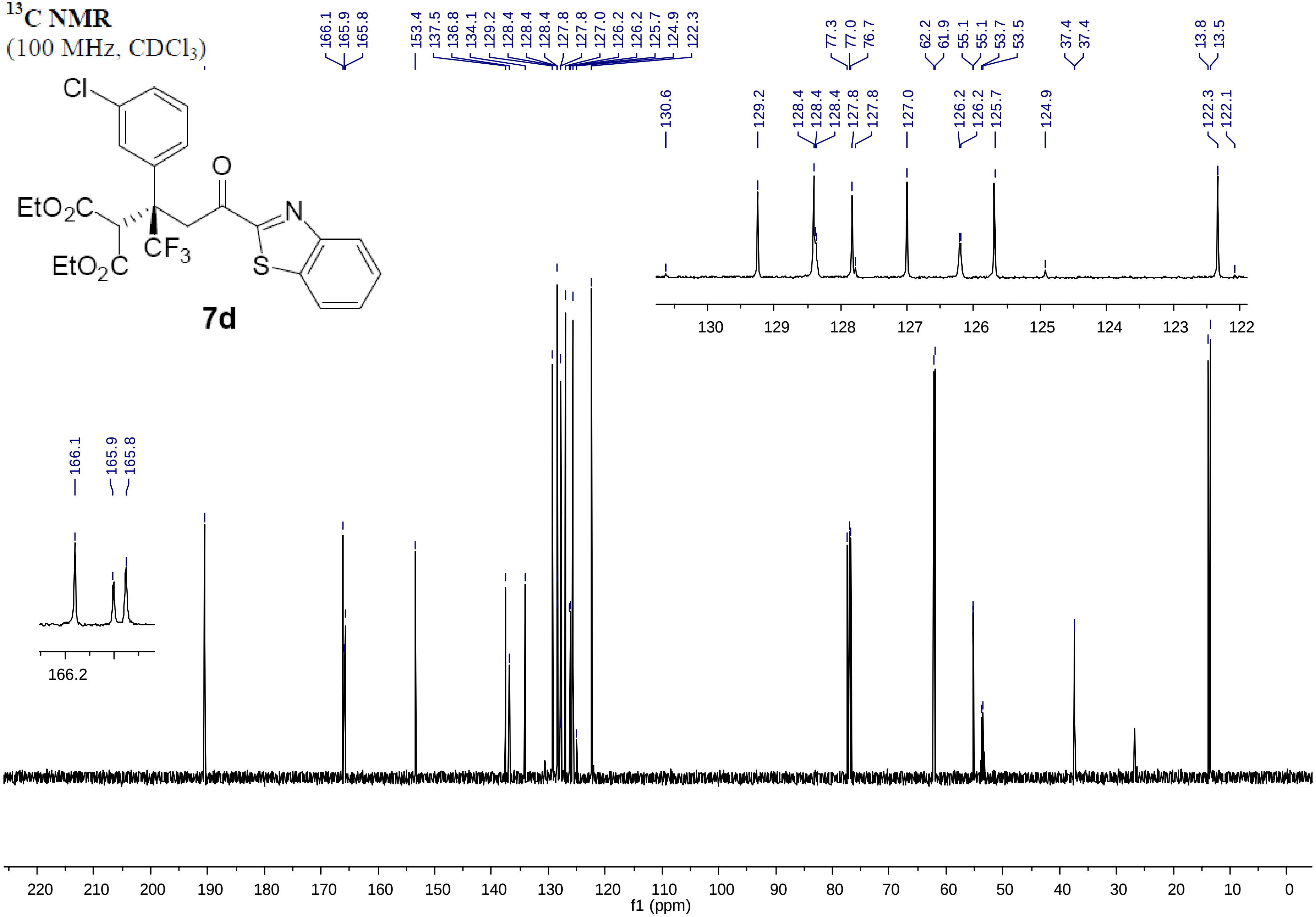

7d

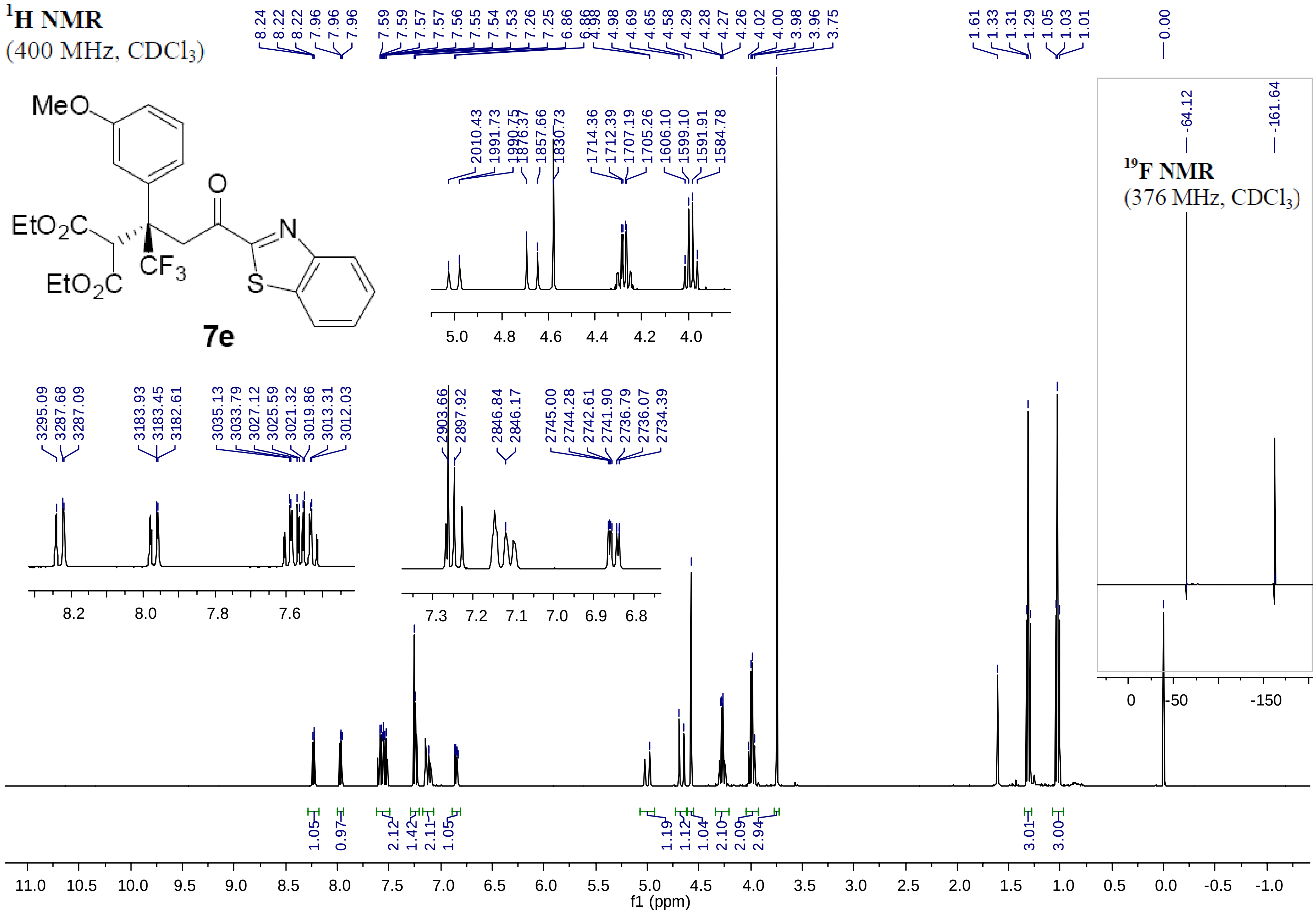

1H NMR
(400 MHz, CDCl3)
7e
19F NMR
(376 MHz, CDCl3)
-64.12
-161.64
f1 (ppm)

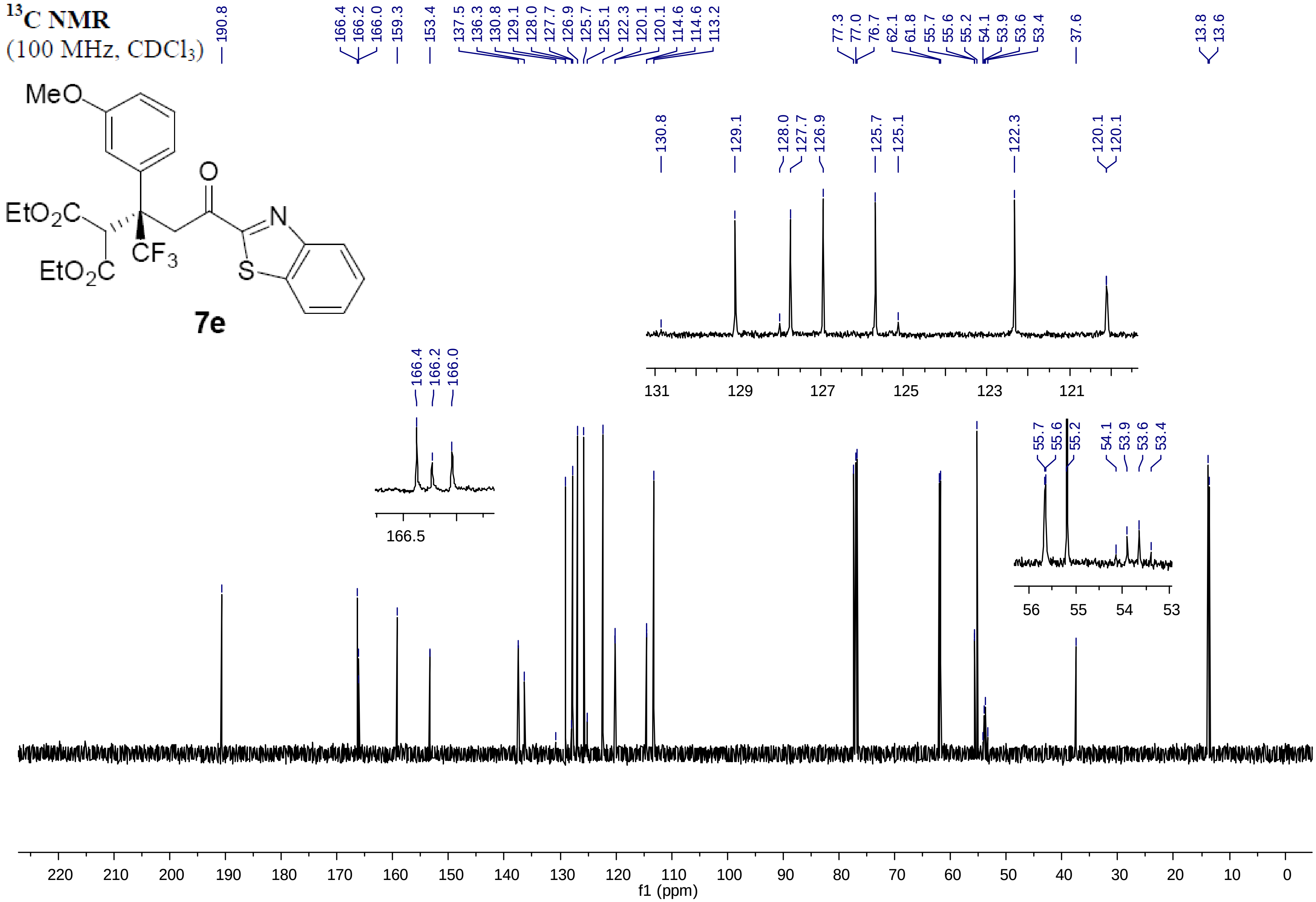

13C NMR
(100 MHz, CDCl3)
190.8
166.4
166.2
166.0
159.3
153.4
137.5
136.3
130.8
129.1
128.0
127.7
126.9
125.7
125.1
122.3
120.1
120.1
114.6
114.6
113.2
77.3
77.0
76.7
62.1
61.8
55.7
55.6
55.2
54.1
53.9
53.6
53.4
37.6
13.8
13.6
MeO
O
EtO2C
EtO2C
CF3
N
S
7e
f1 (ppm)

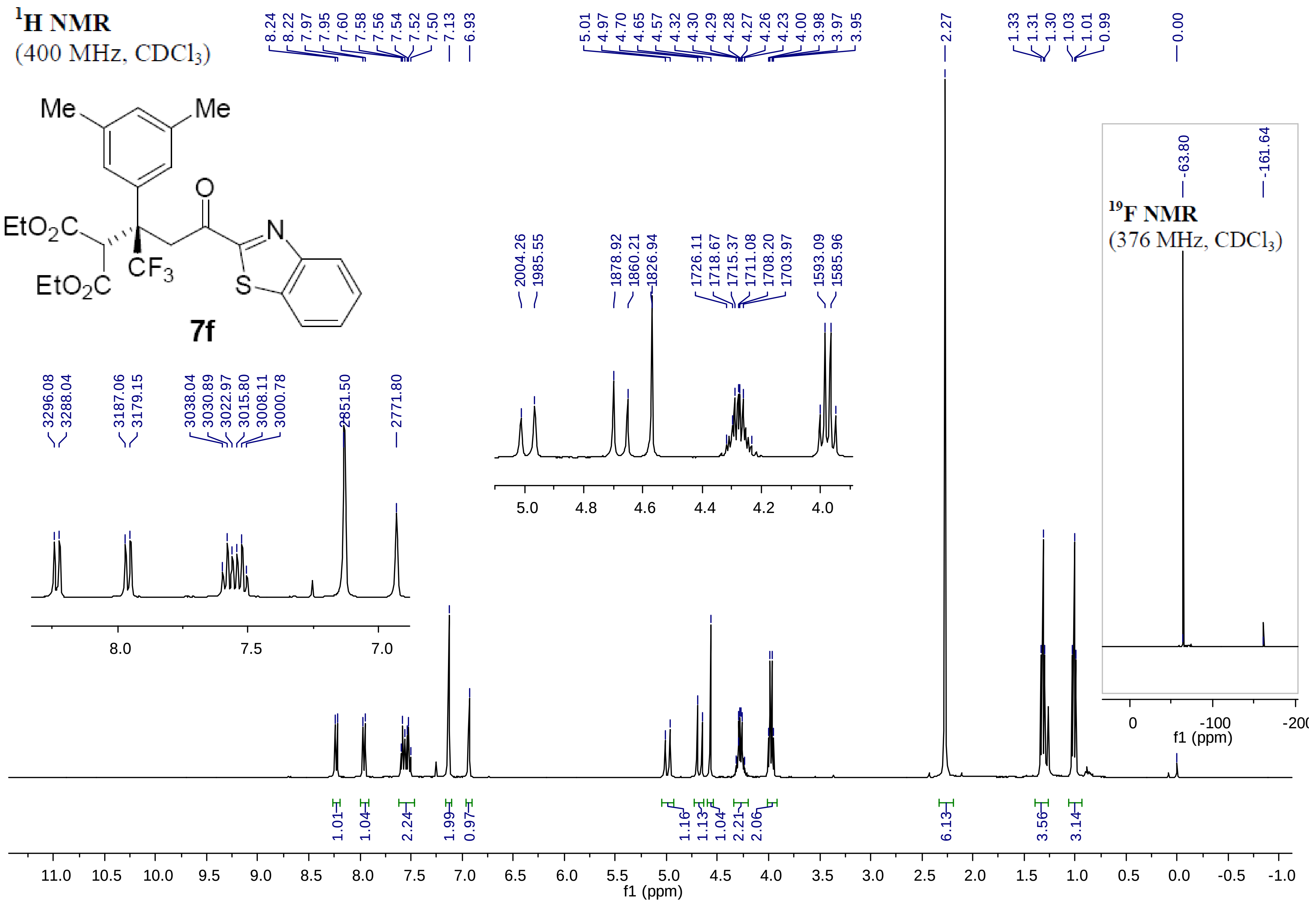

^{1}H NMR
(400 MHz, CDCl$_3$)
7f
^{19}F NMR
(376 MHz, CDCl$_3$)
-63.80
-161.64
f1 (ppm)

^{13}C NMR
(100 MHz, $CDCl_3$)

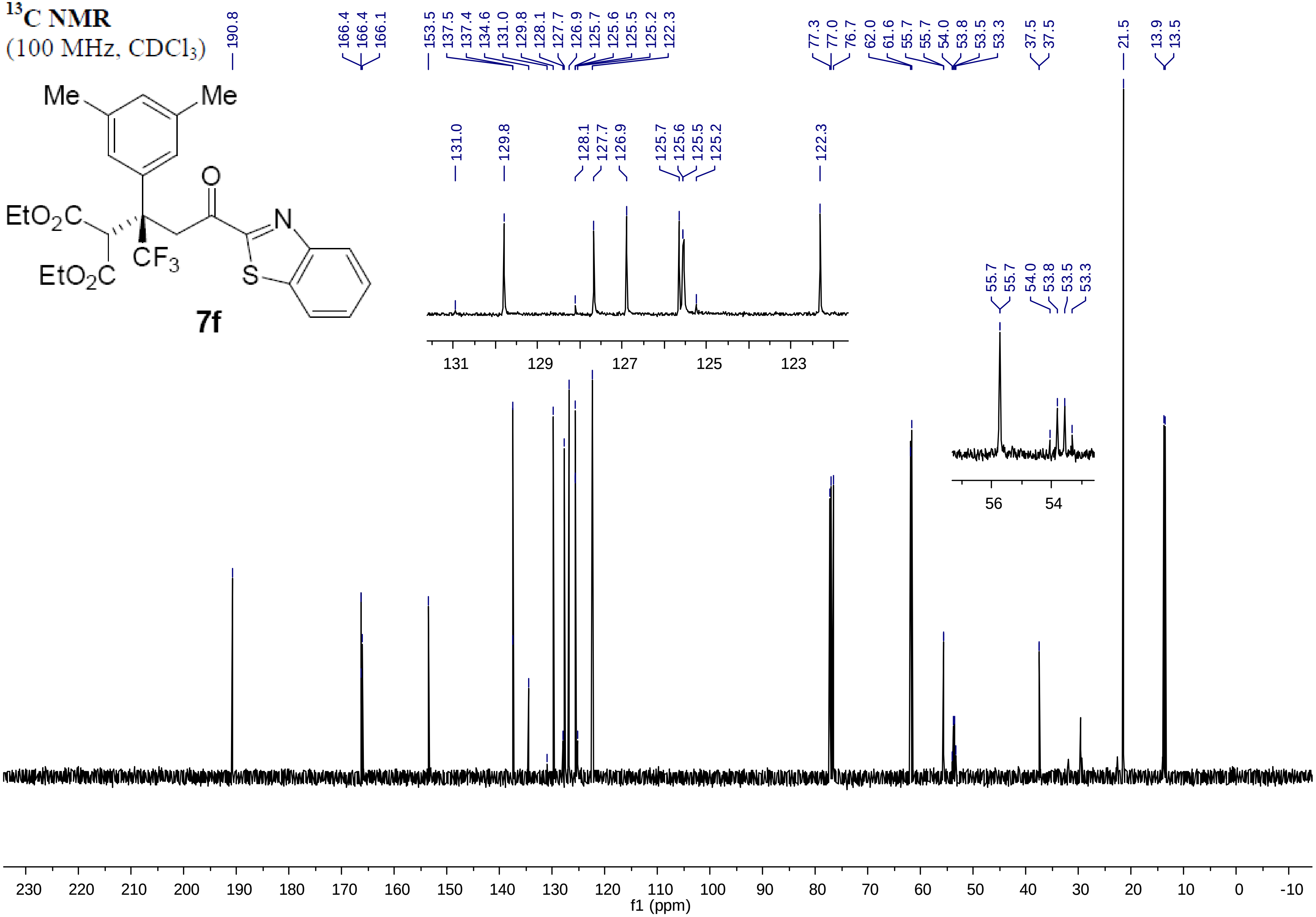

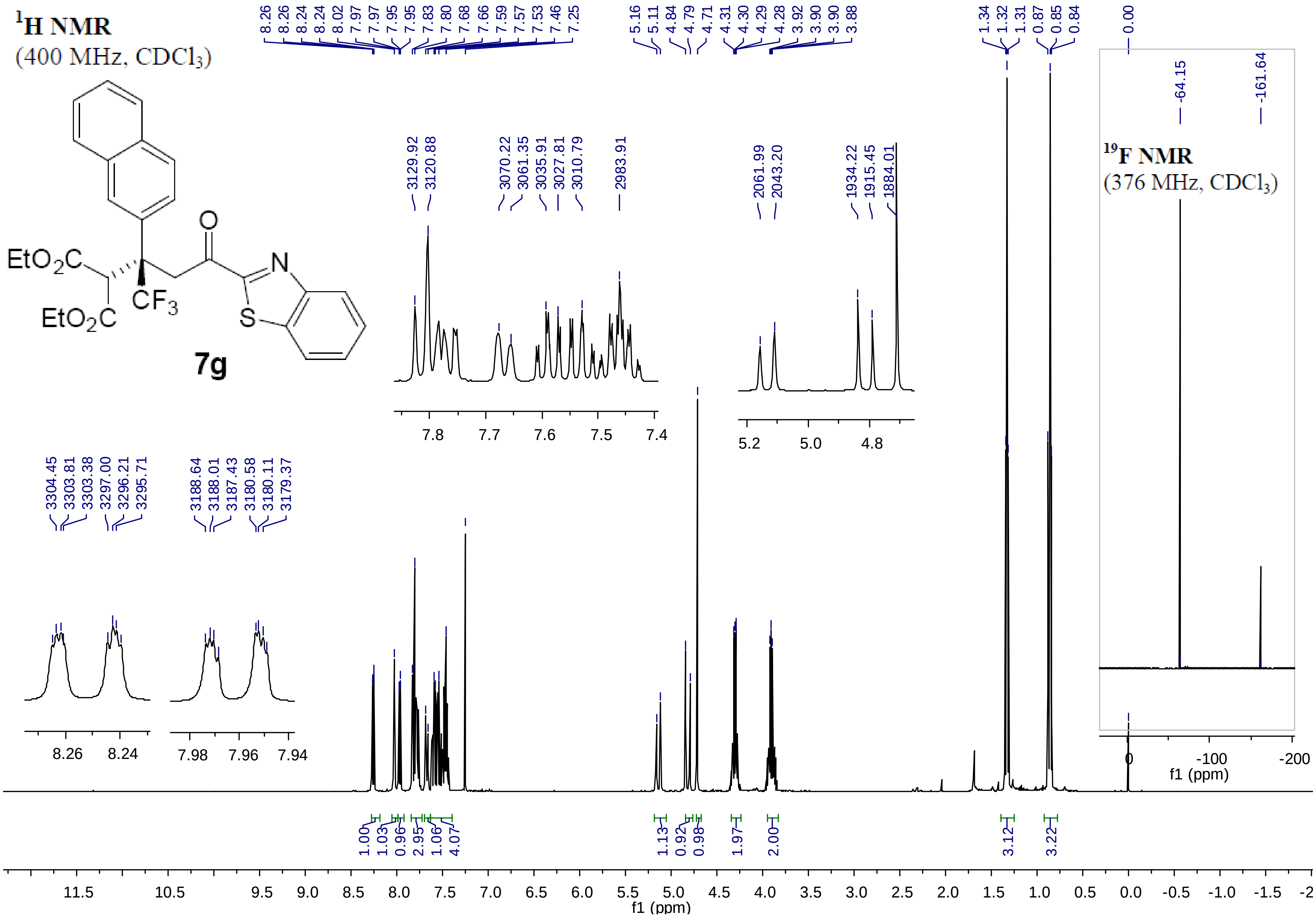
1H NMR
(400 MHz, CDCl3)
EtO2C
EtO2C
CF3
O
N
S
7g
8.26 8.26 8.24 8.24 8.02 7.97 7.97 7.95 7.95 7.83 7.80 7.68 7.66 7.59 7.57 7.53 7.46 7.25
5.16 5.11 4.84 4.79 4.71 4.31 4.30 4.29 4.28 3.92 3.90 3.90 3.88
1.34 1.32 1.31 0.87 0.85 0.84
0.00
3129.92 3120.88 3070.22 3061.35 3035.91 3027.81 3010.79 2983.91
2061.99 2043.20 1934.22 1915.45 1884.01
3304.45 3303.81 3303.38 3297.00 3296.21 3295.71
3188.64 3188.01 3187.43 3180.58 3180.11 3179.37
19F NMR
(376 MHz, CDCl3)
-64.15
-161.64
1.00 1.03 0.96 2.95 1.06 4.07 1.13 0.92 0.98 1.97 2.00 3.12 3.22
f1 (ppm)

^{13}C NMR
(100 MHz, $CDCl_3$)

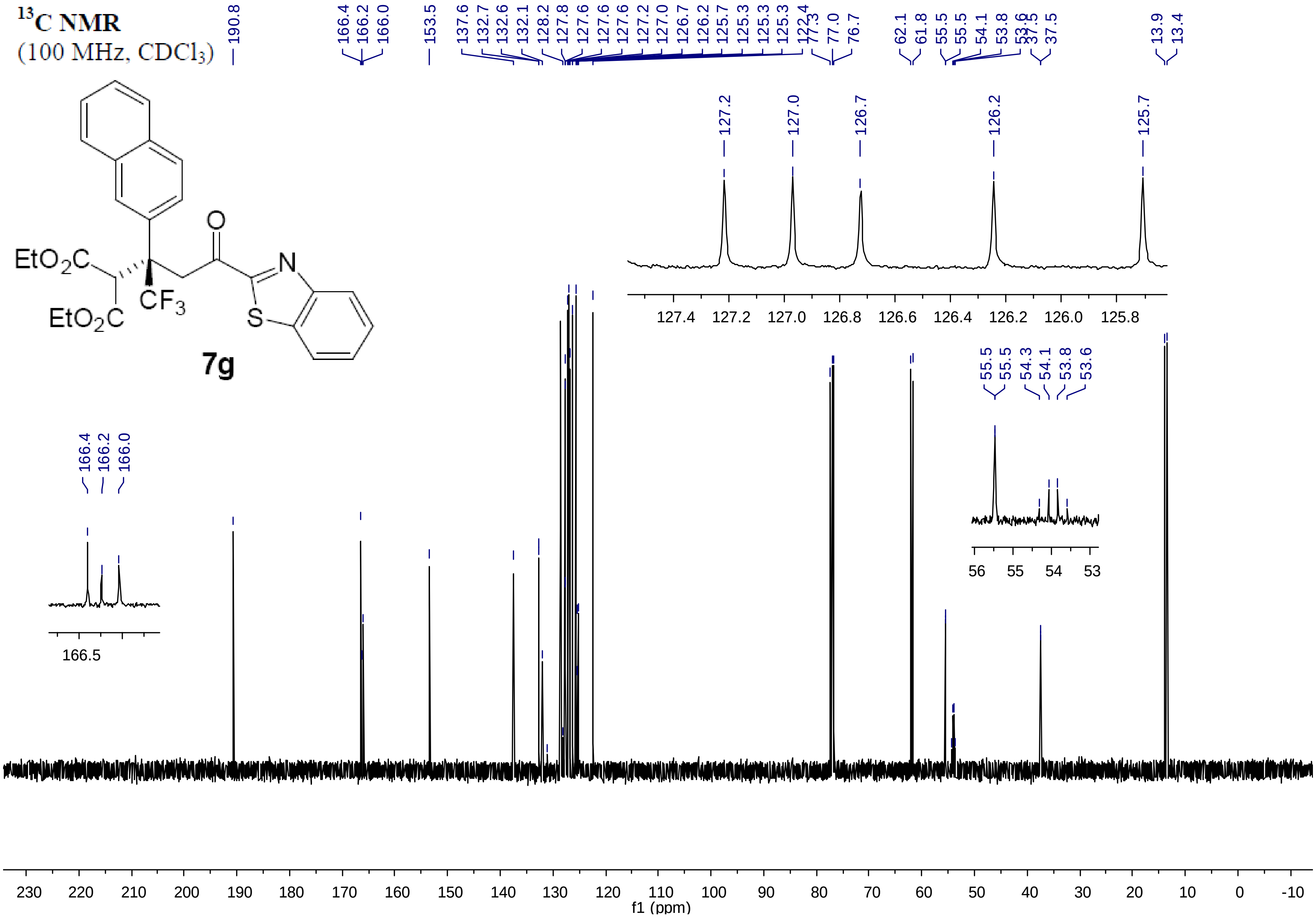

^{1}H NMR
(400 MHz, $CDCl_3$)

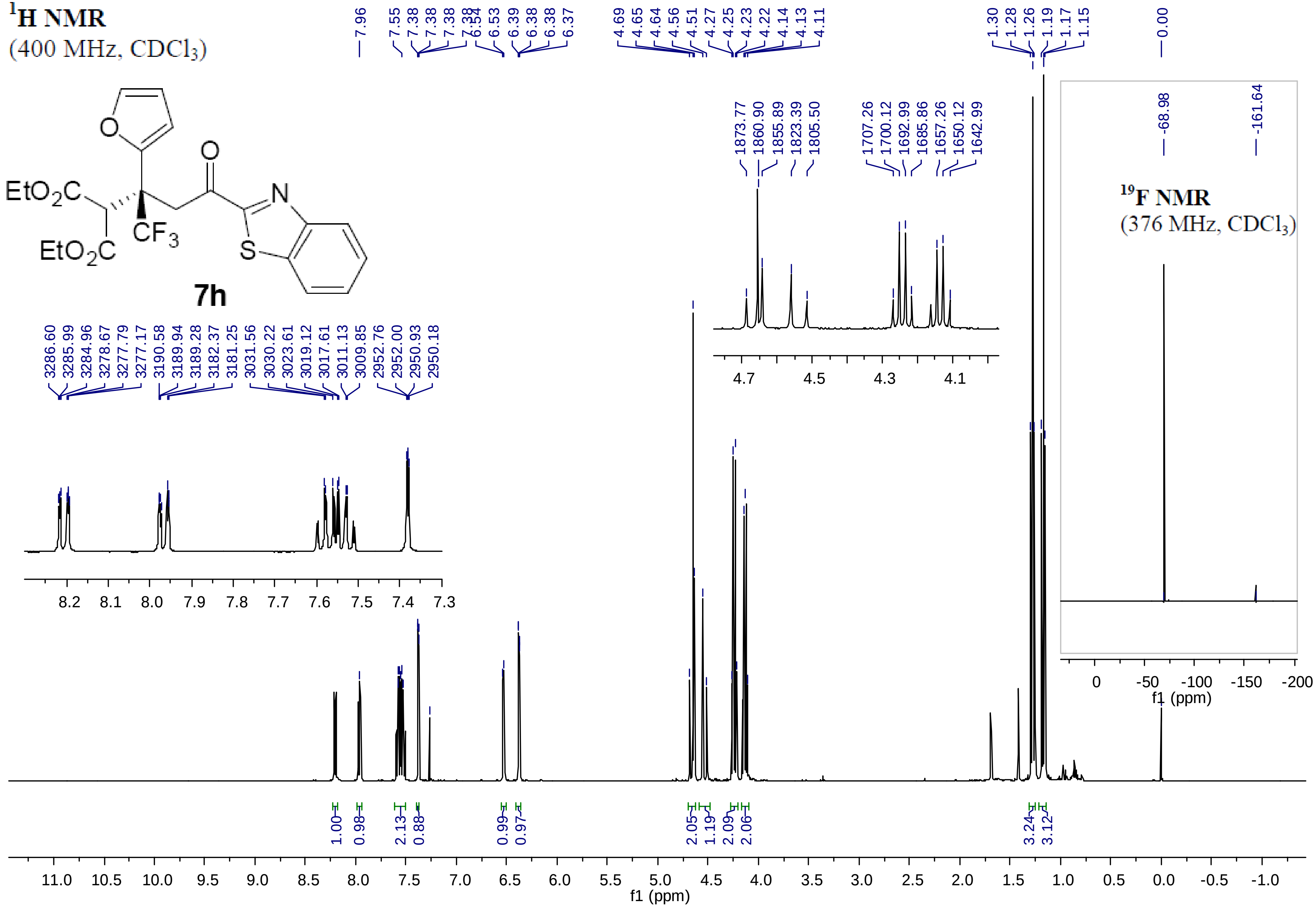

^{13}C NMR

(100 MHz, $CDCl_3$)

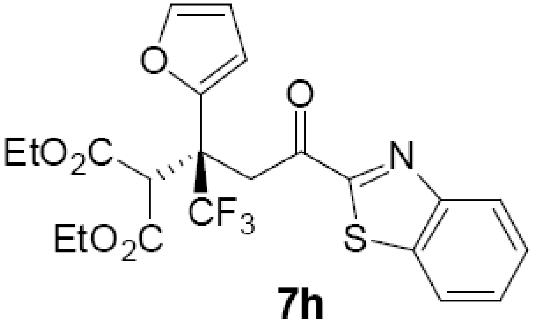

7h

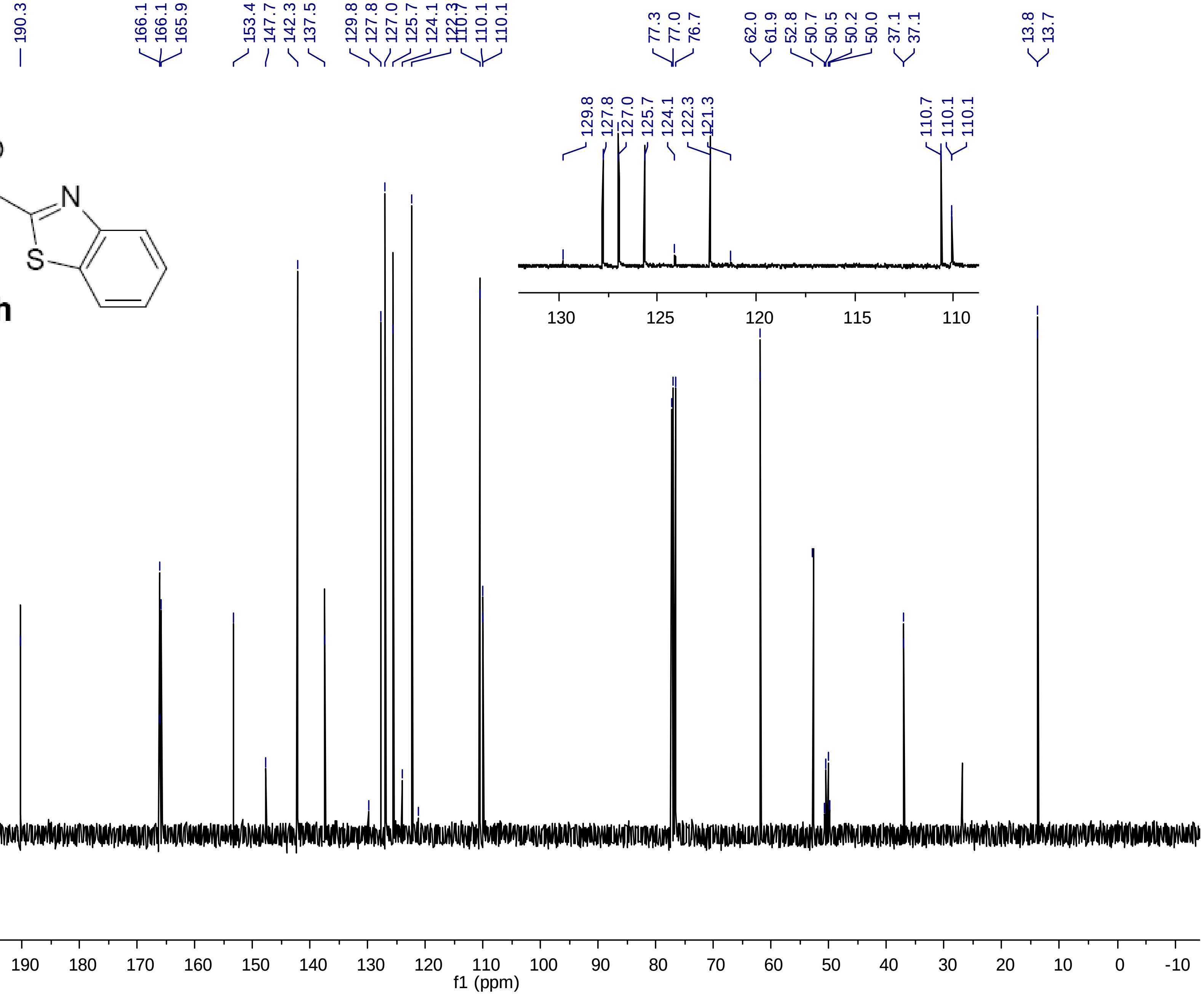

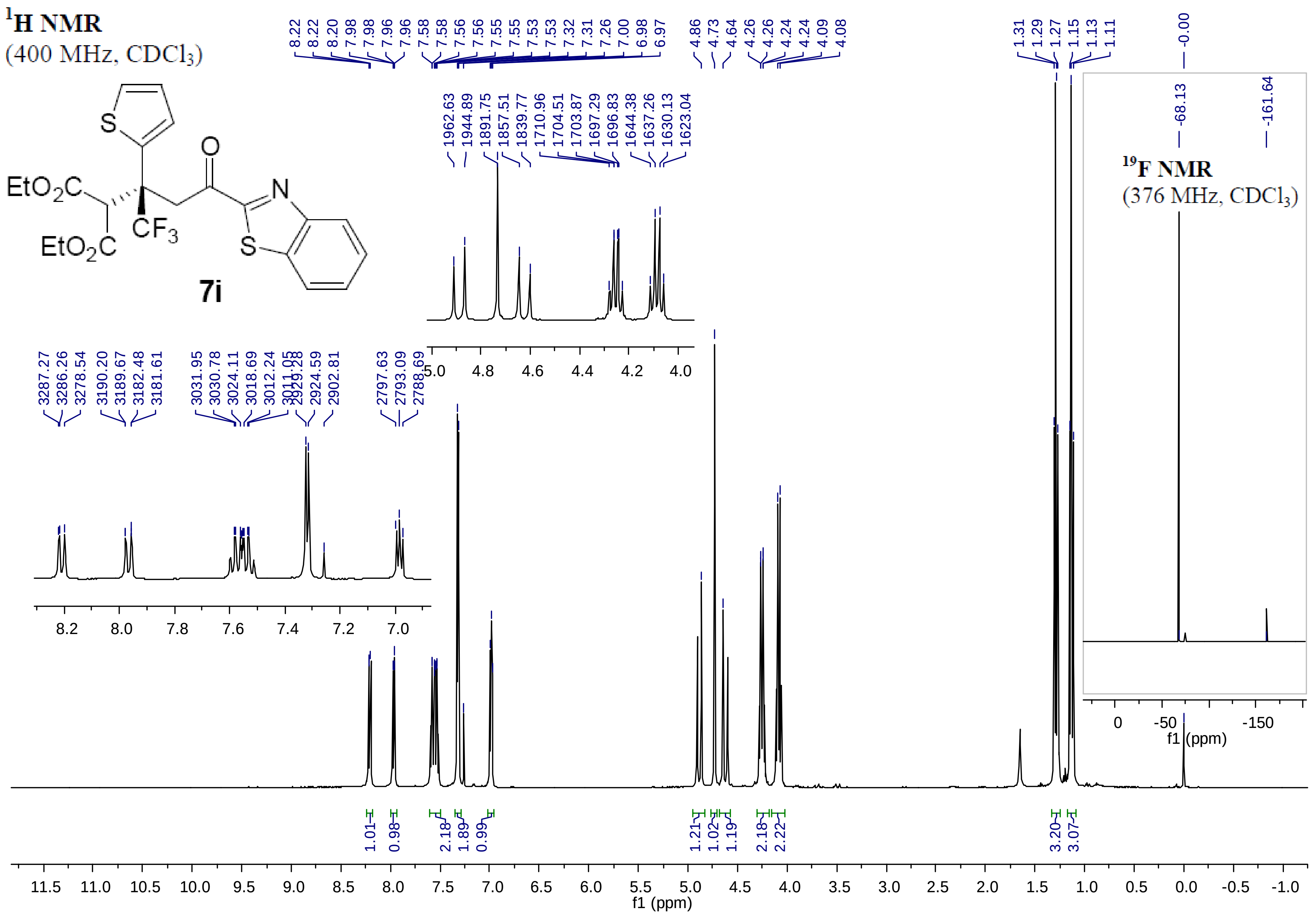

1H NMR
(400 MHz, CDCl3)
EtO2C
EtO2C
CF3
S
O
N
S
7i
19F NMR
(376 MHz, CDCl3)
-68.13
-161.64
f1 (ppm)

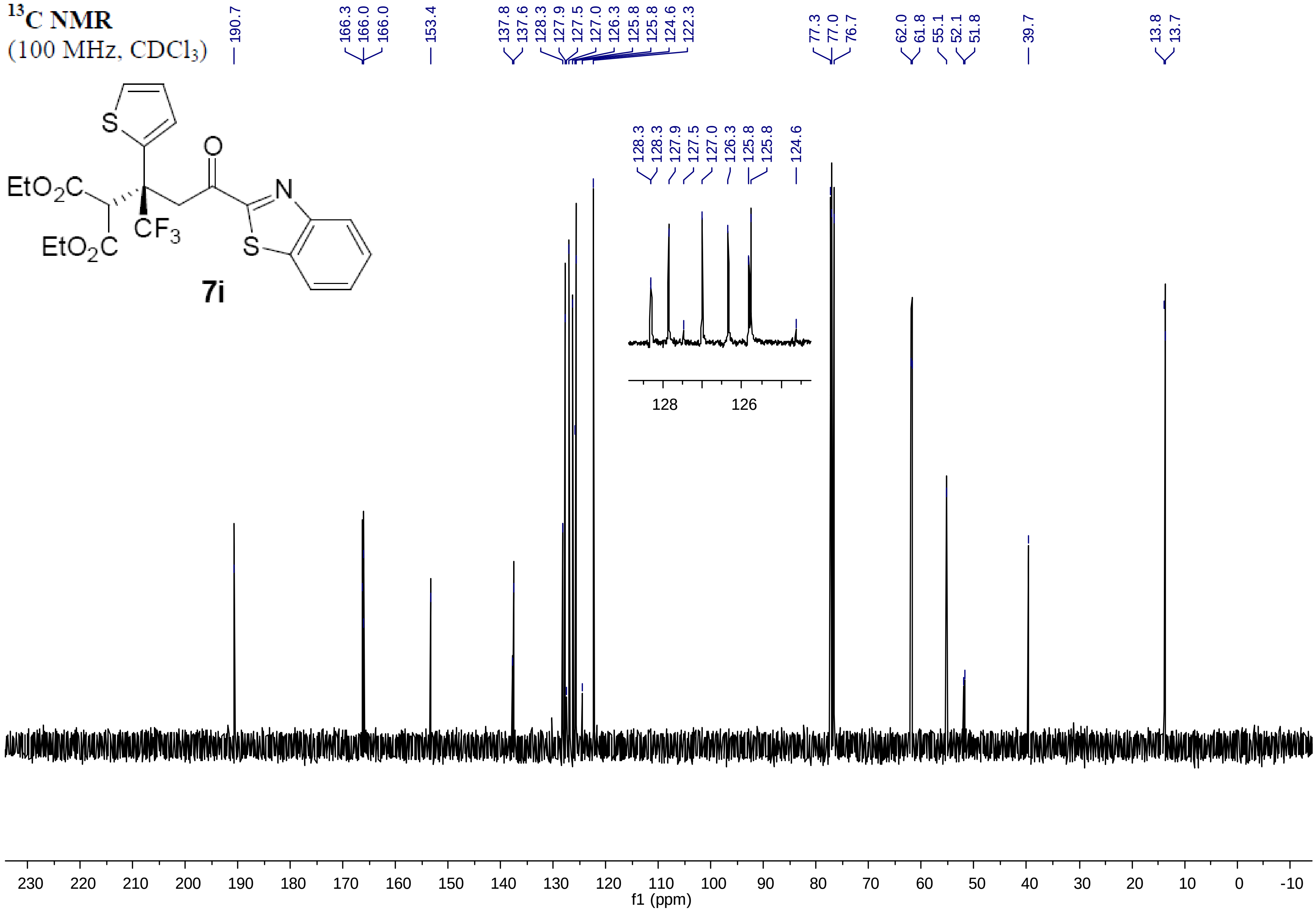
13C NMR
(100 MHz, CDCl3)
190.7
166.3
166.0
166.0
153.4
137.8
137.6
128.3
127.9
127.5
127.0
126.3
125.8
125.8
124.6
122.3
77.3
77.0
76.7
62.0
61.8
55.1
52.1
51.8
39.7
13.8
13.7
7i
f1 (ppm)

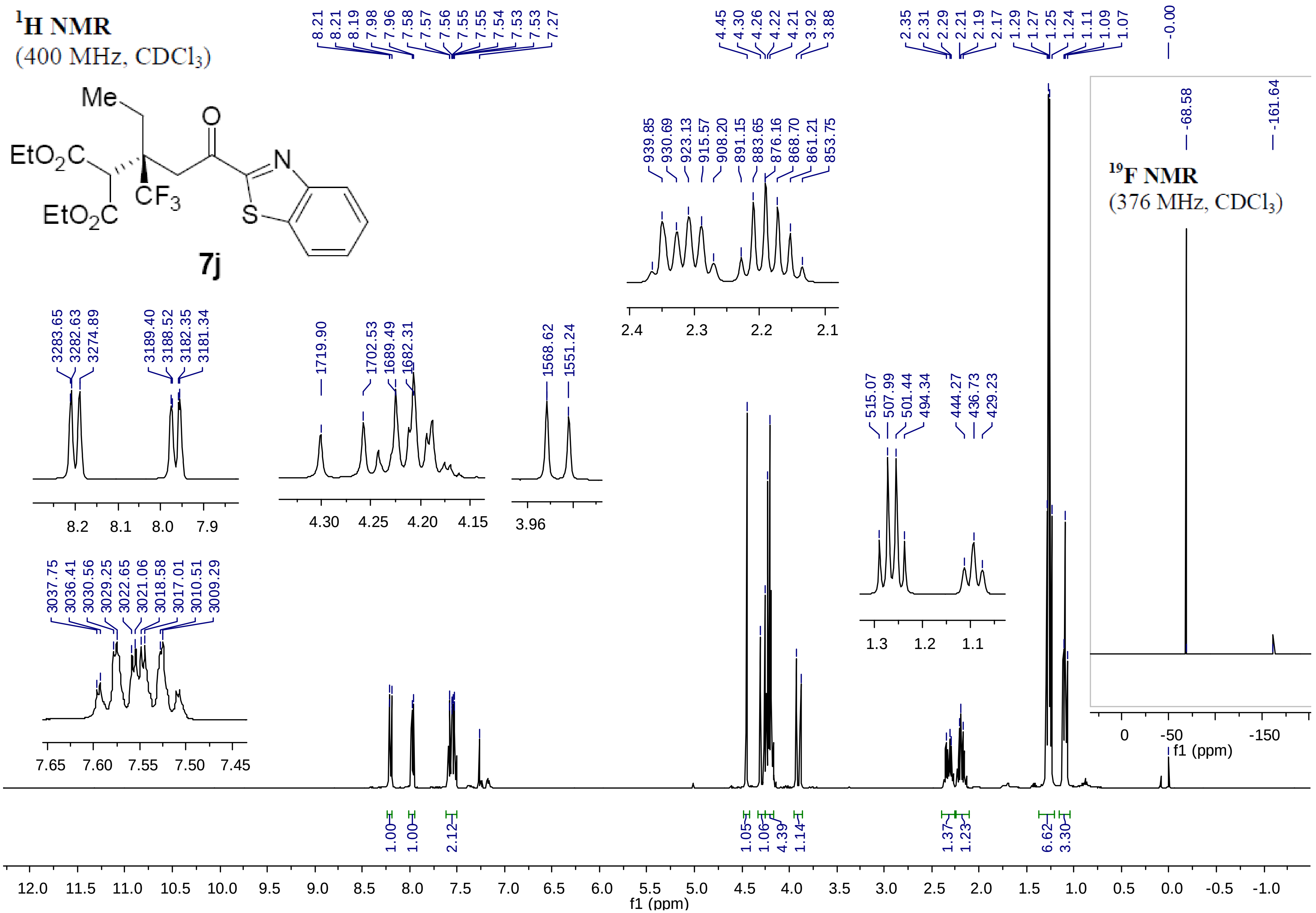

1H NMR
(400 MHz, CDCl3)
Me
EtO2C
EtO2C
CF3
O
N
S
7j
19F NMR
(376 MHz, CDCl3)
-68.58
-161.64
f1 (ppm)

^{13}C NMR
(100 MHz, $CDCl_3$)

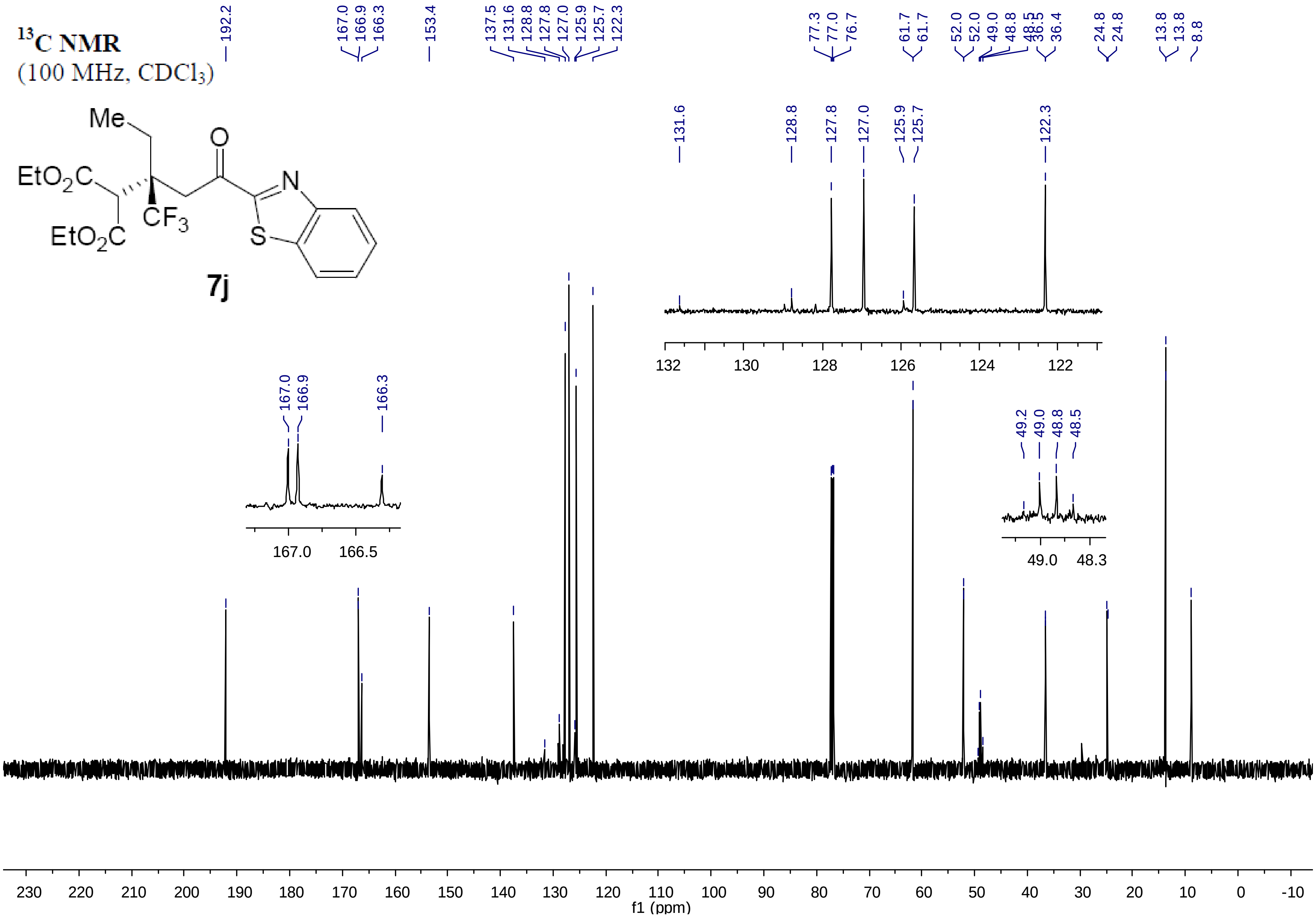

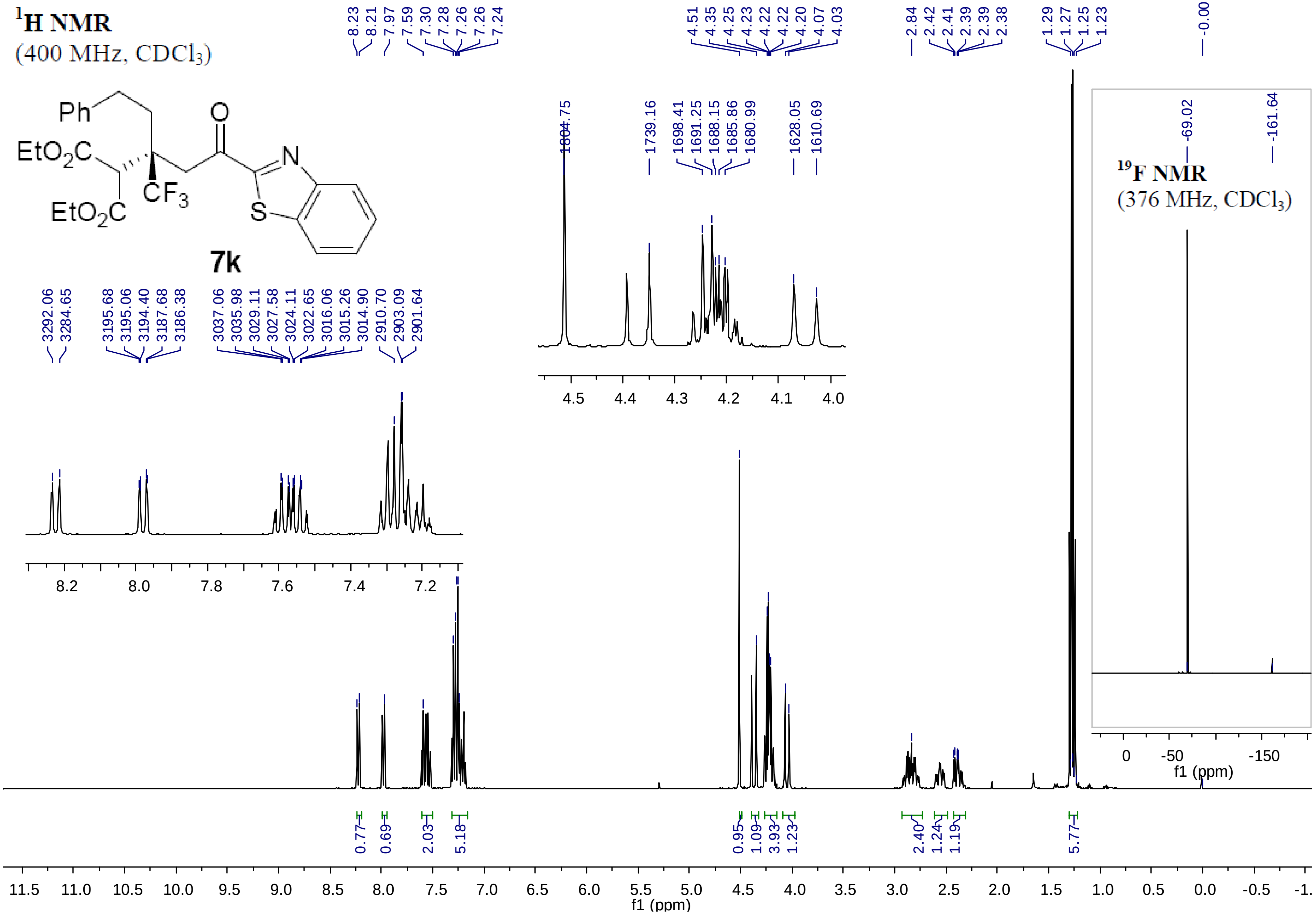

^{1}H NMR
(400 MHz, CDCl$_3$)
Ph
EtO$_2$C
EtO$_2$C
O
N
S
CF$_3$
7k
8.23 8.21 7.97 7.59 7.30 7.28 7.26 7.26 7.24
4.51 4.35 4.25 4.23 4.22 4.22 4.20 4.07 4.03
2.84 2.42 2.41 2.39 2.39 2.38
1.29 1.27 1.25 1.23
-0.00
3292.06 3284.65 3195.68 3195.06 3194.40 3187.68 3186.38 3037.06 3035.98 3029.11 3027.58 3024.11 3022.65 3016.06 3015.26 3014.90 2910.70 2903.09 2901.64
1804.75 1739.16 1698.41 1691.25 1688.15 1685.86 1680.99 1628.05 1610.69
^{19}F NMR
(376 MHz, CDCl$_3$)
-69.02
-161.64
0.77 0.69 2.03 5.18 0.95 1.09 3.93 1.23 2.40 1.24 1.19 5.77
f1 (ppm)

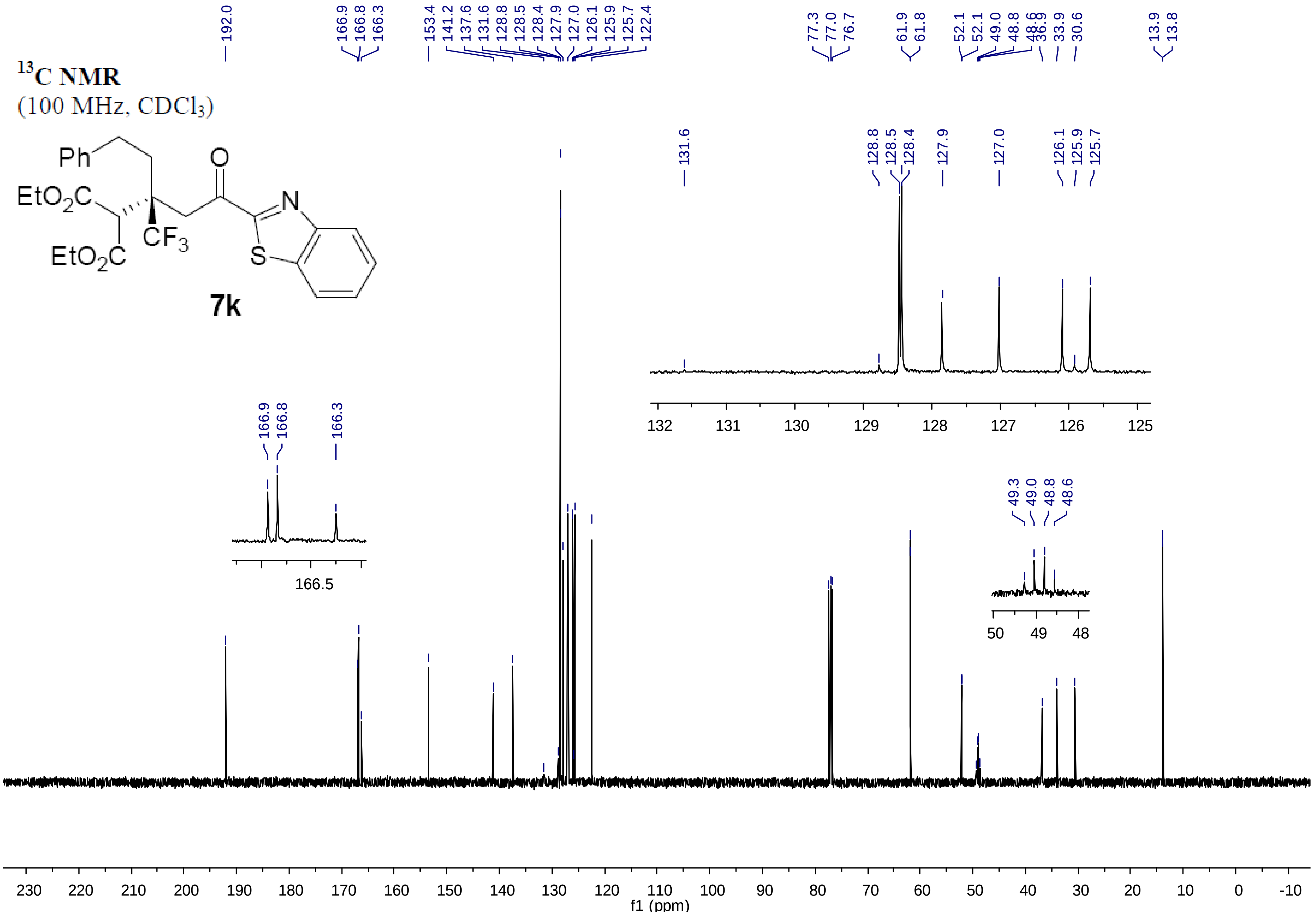

^{13}C NMR
(100 MHz, $CDCl_3$)
Ph
O
EtO_2C
N
CF_3
EtO_2C
S
7k
192.0
166.9
166.8
166.3
153.4
141.2
137.6
131.6
128.8
128.5
128.4
127.9
127.0
126.1
125.9
125.7
122.4
77.3
77.0
76.7
61.9
61.8
52.1
52.1
49.0
48.8
48.6
36.9
33.9
30.6
13.9
13.8
49.3
166.5
132 131 130 129 128 127 126 125
50 49 48
230 220 210 200 190 180 170 160 150 140 130 120 110 100 90 80 70 60 50 40 30 20 10 0 -10
f1 (ppm)

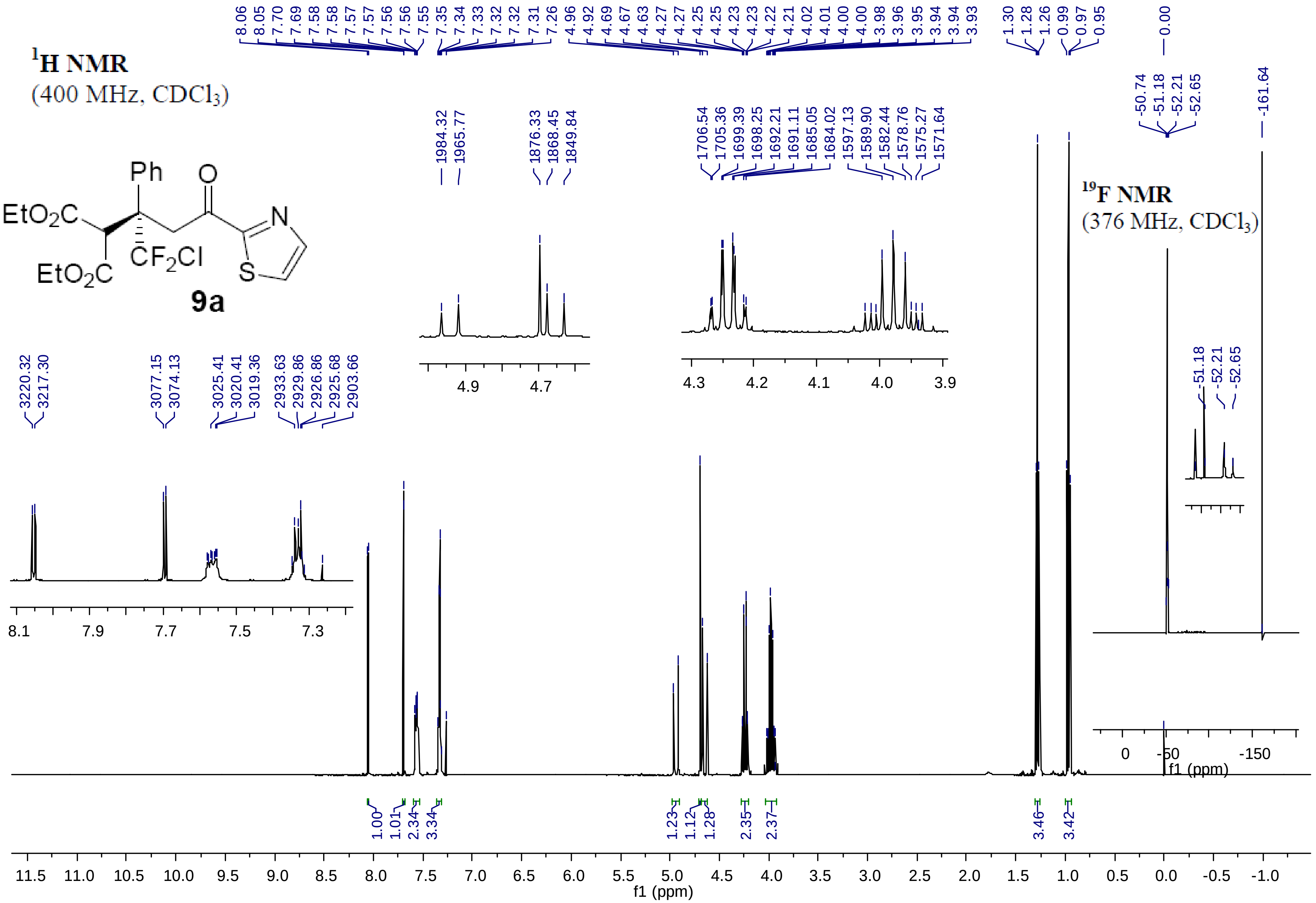
1H NMR
(400 MHz, CDCl3)
Ph
O
EtO2C
N
CF2Cl
S
EtO2C
9a
19F NMR
(376 MHz, CDCl3)
f1 (ppm)

^{13}C NMR
(100 MHz, $CDCl_3$)

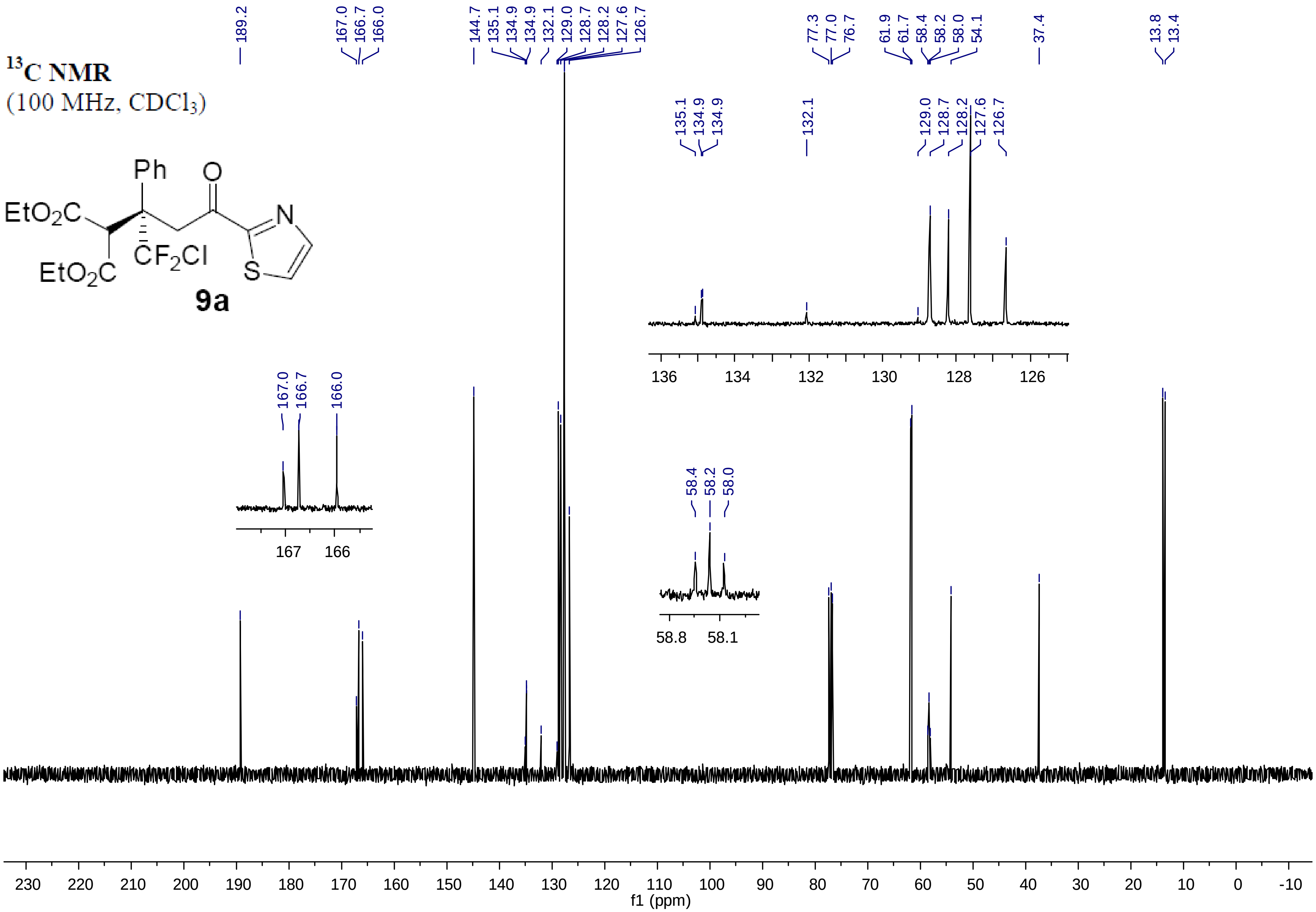

9a

^{1}H NMR
(400 MHz, $CDCl_3$)

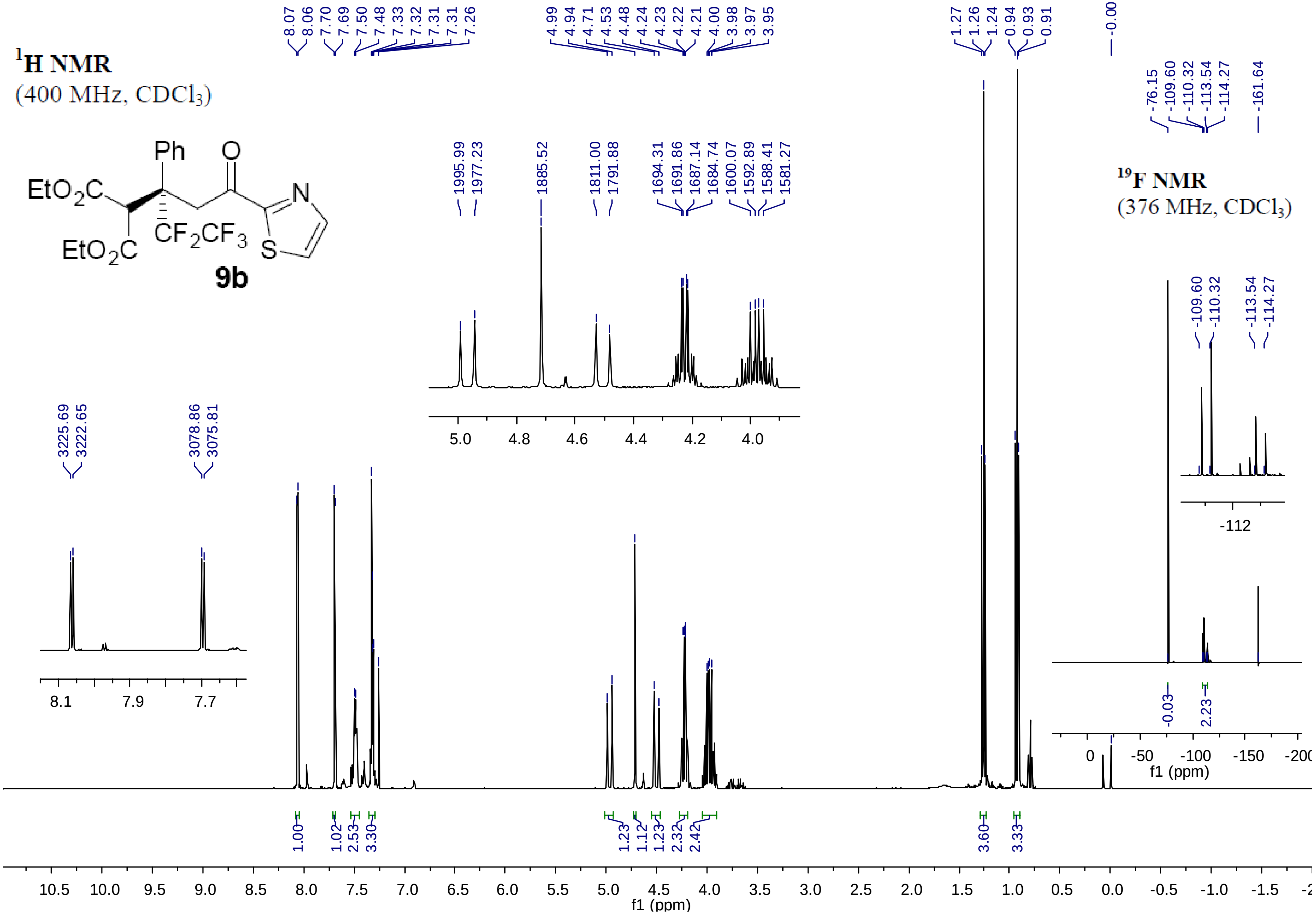

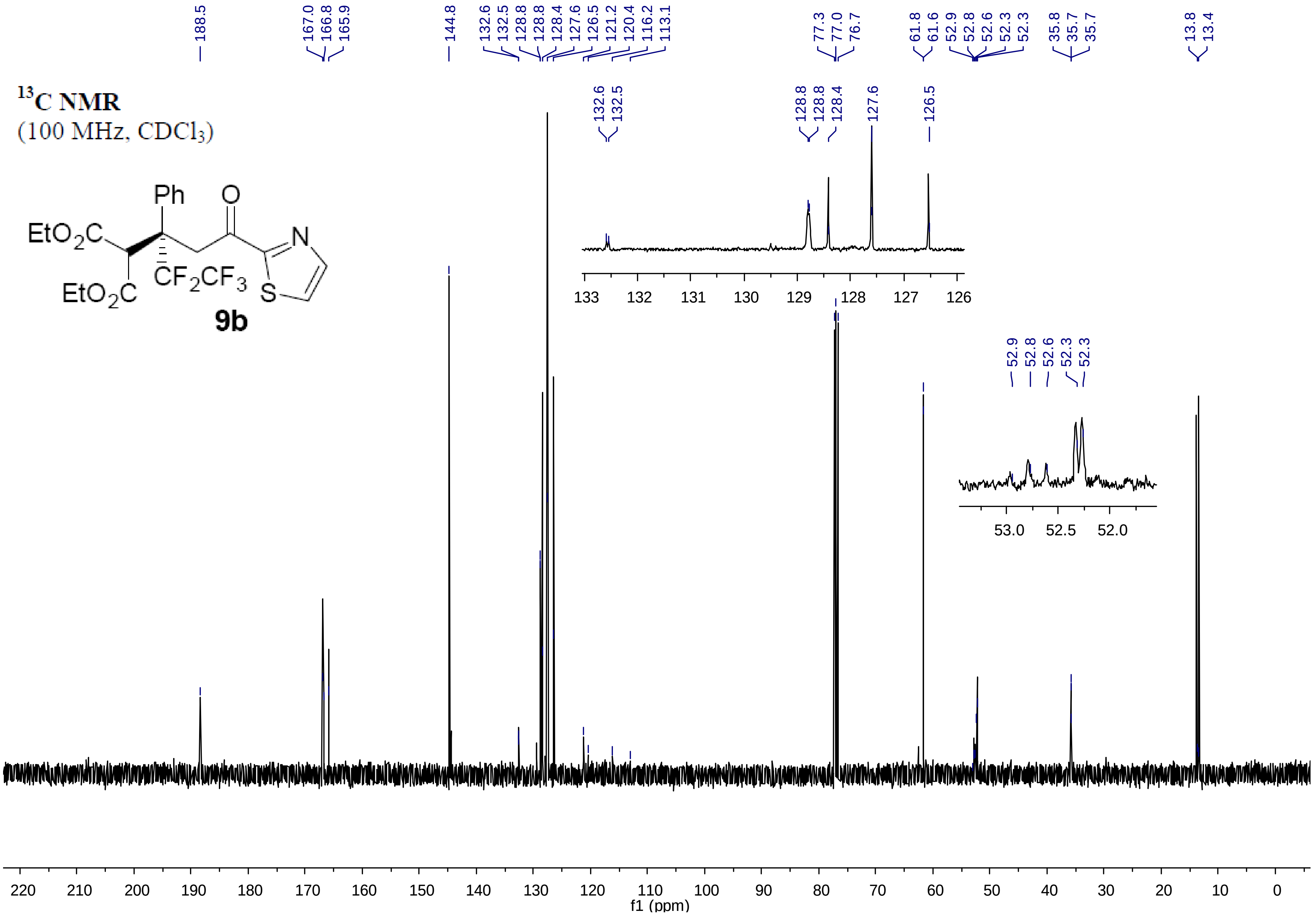

^{13}C NMR
(100 MHz, CDCl$_3$)
9b
188.5
167.0
166.8
165.9
144.8
132.6
132.5
128.8
128.8
128.4
127.6
126.5
121.2
120.4
116.2
113.1
77.3
77.0
76.7
61.8
61.6
52.9
52.8
52.6
52.3
52.3
35.8
35.7
35.7
13.8
13.4
f1 (ppm)

Copies of NMR spectra - compounds 10a-10g, obtained from adduct 3h

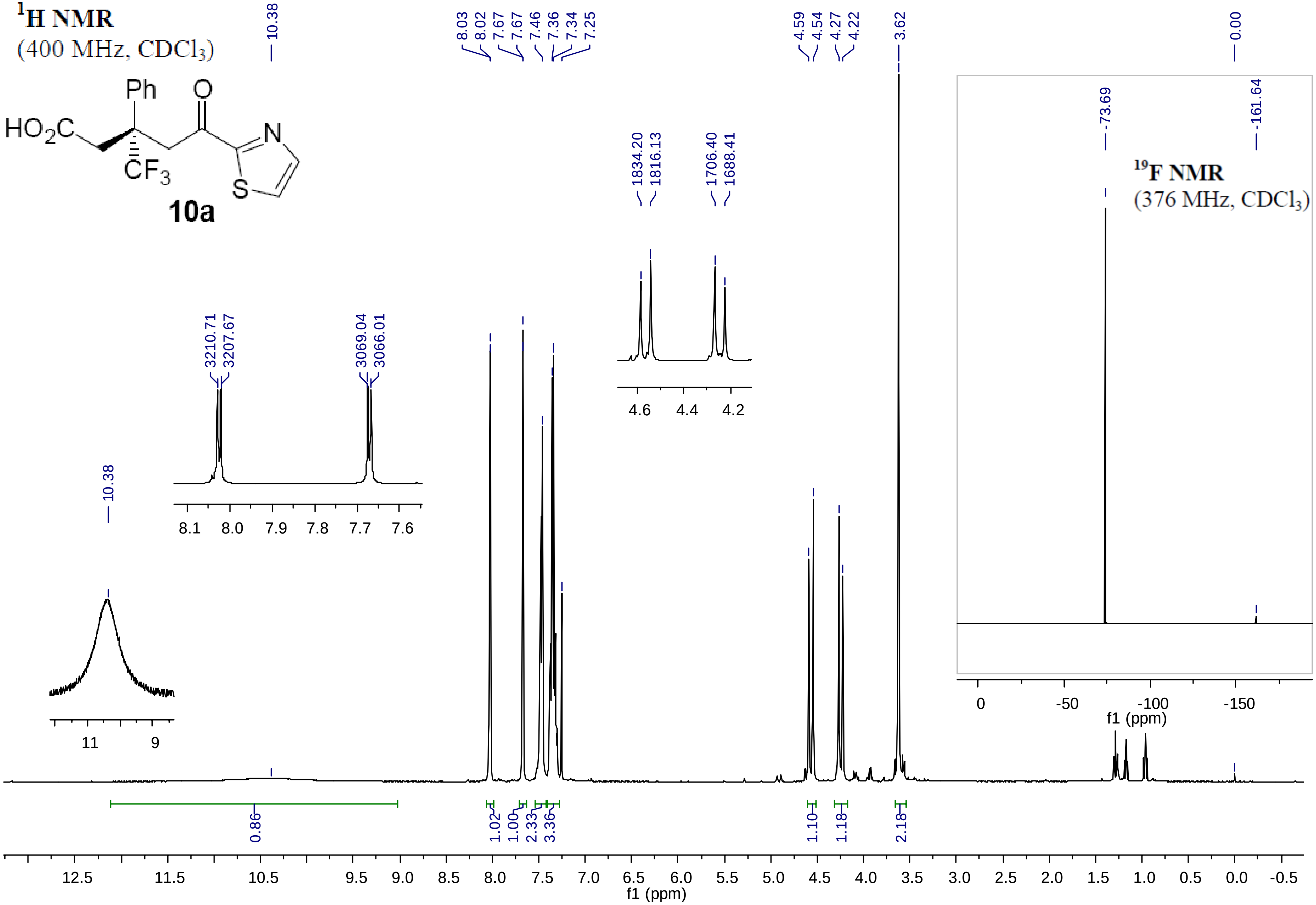

^{13}C NMR
(100 MHz, CDCl$_3$)

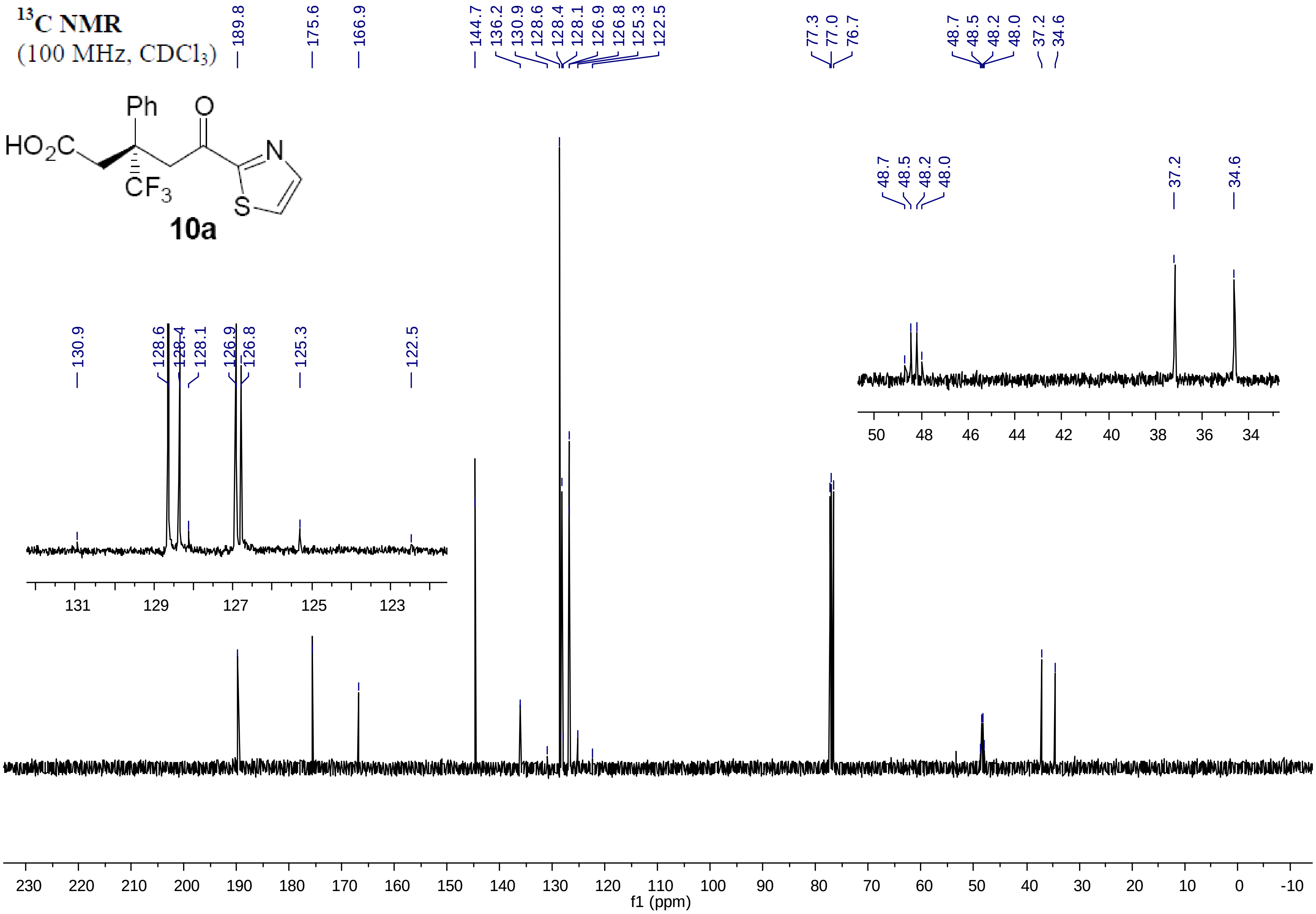

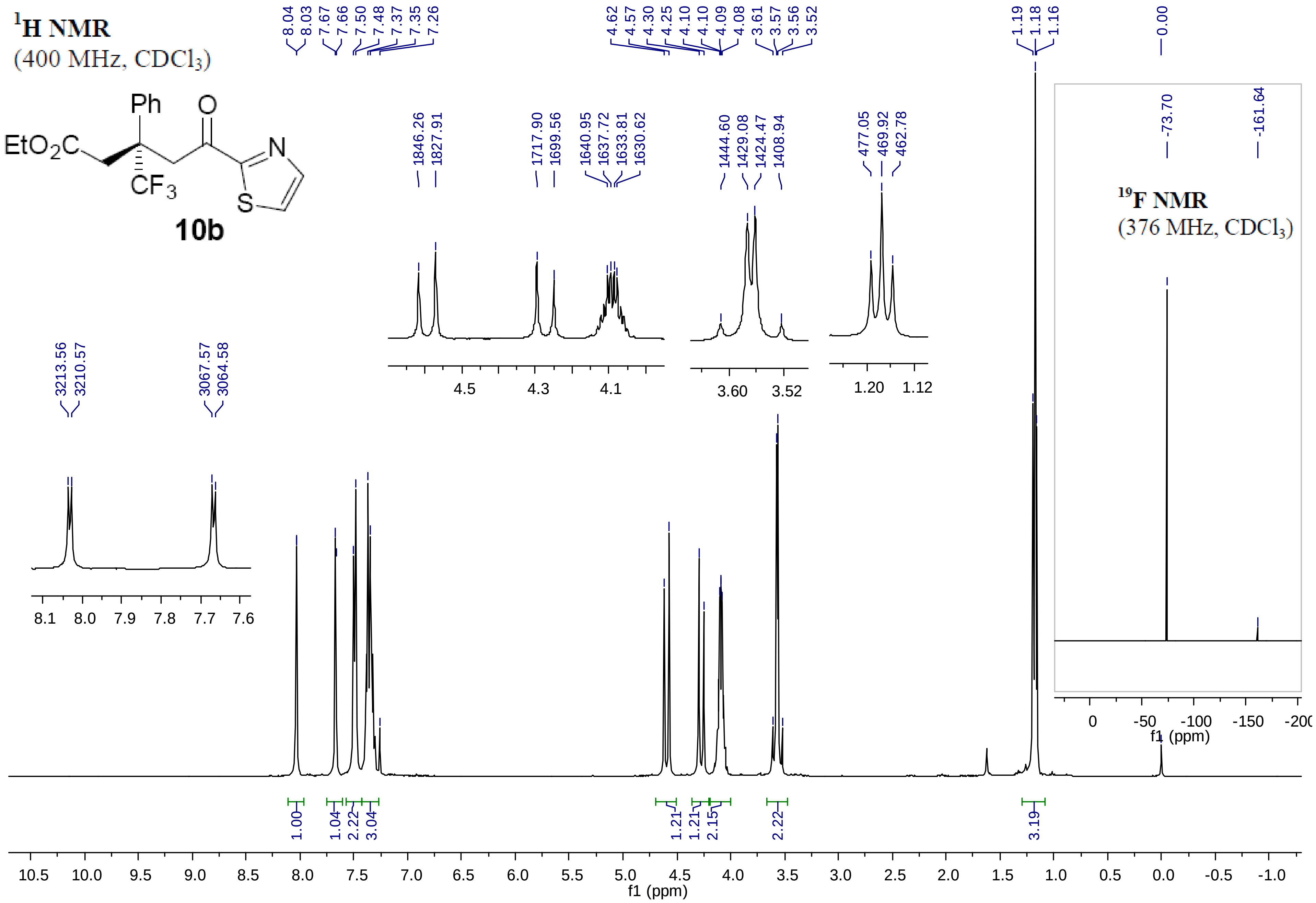

1H NMR
(400 MHz, CDCl3)
10b
19F NMR
(376 MHz, CDCl3)
-73.70
-161.64
f1 (ppm)

^{13}C NMR
(100 MHz, $CDCl_3$)

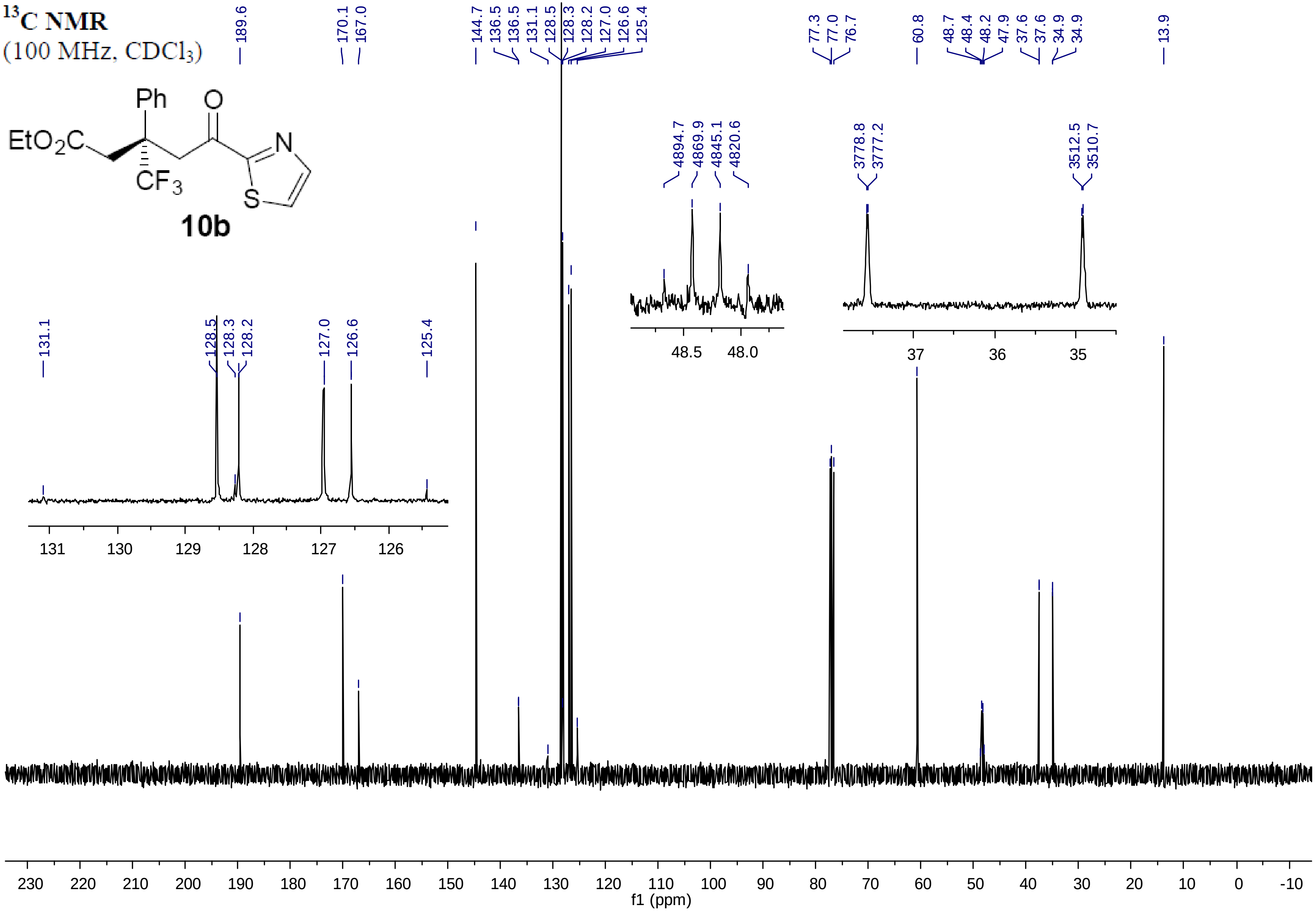

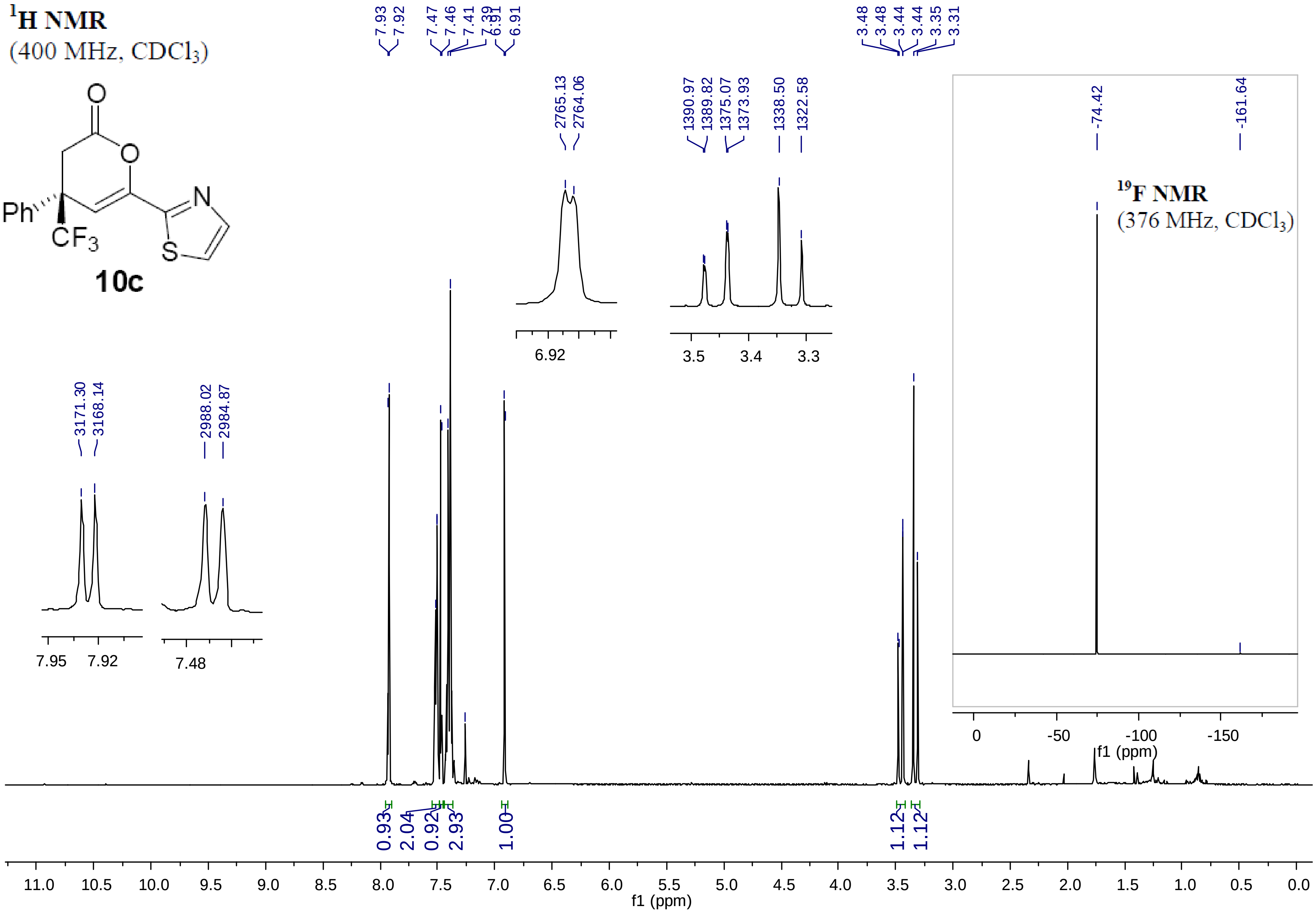
^{1}H NMR
(400 MHz, $CDCl_3$)
Ph
CF_3
O
O
N
S
10c
7.93
7.92
7.47
7.46
7.41
7.39
6.91
6.91
3.48
3.48
3.44
3.44
3.35
3.31
2765.13
2764.06
6.92
1390.97
1389.82
1375.07
1373.93
1338.50
1322.58
3.5
3.4
3.3
3171.30
3168.14
7.95
7.92
2988.02
2984.87
7.48
-74.42
-161.64
^{19}F NMR
(376 MHz, $CDCl_3$)
0
-50
-100
-150
f1 (ppm)
0.93
2.04
0.92
2.93
1.00
1.12
1.12
11.0
10.5
10.0
9.5
9.0
8.5
8.0
7.5
7.0
6.5
6.0
5.5
5.0
4.5
4.0
3.5
3.0
2.5
2.0
1.5
1.0
0.5
0.0
f1 (ppm)

^{13}C NMR
(100 MHz, $CDCl_3$)

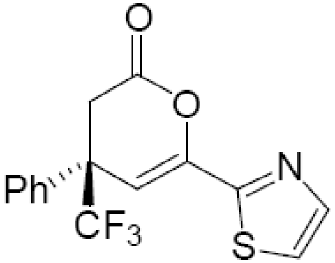

10c

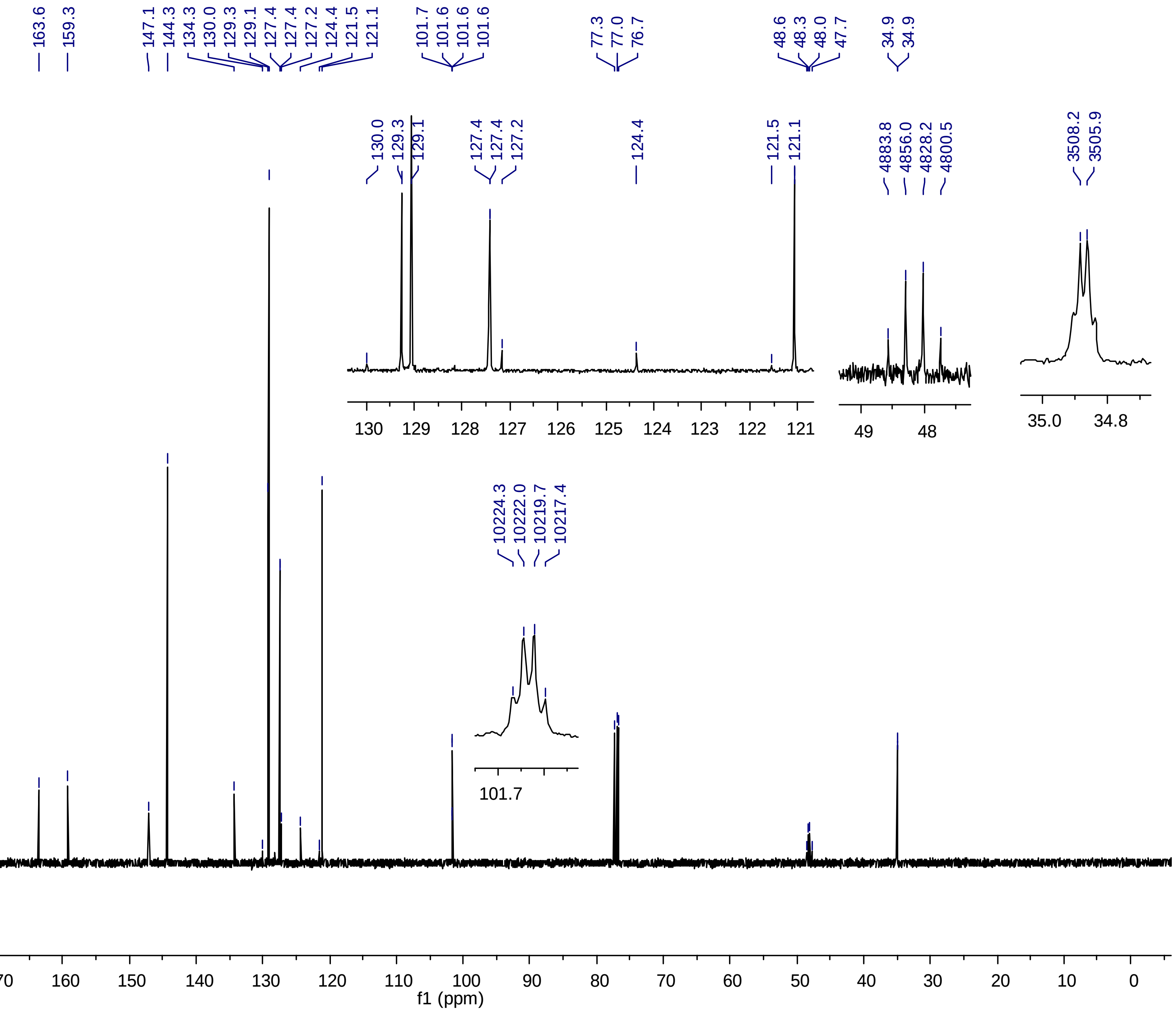

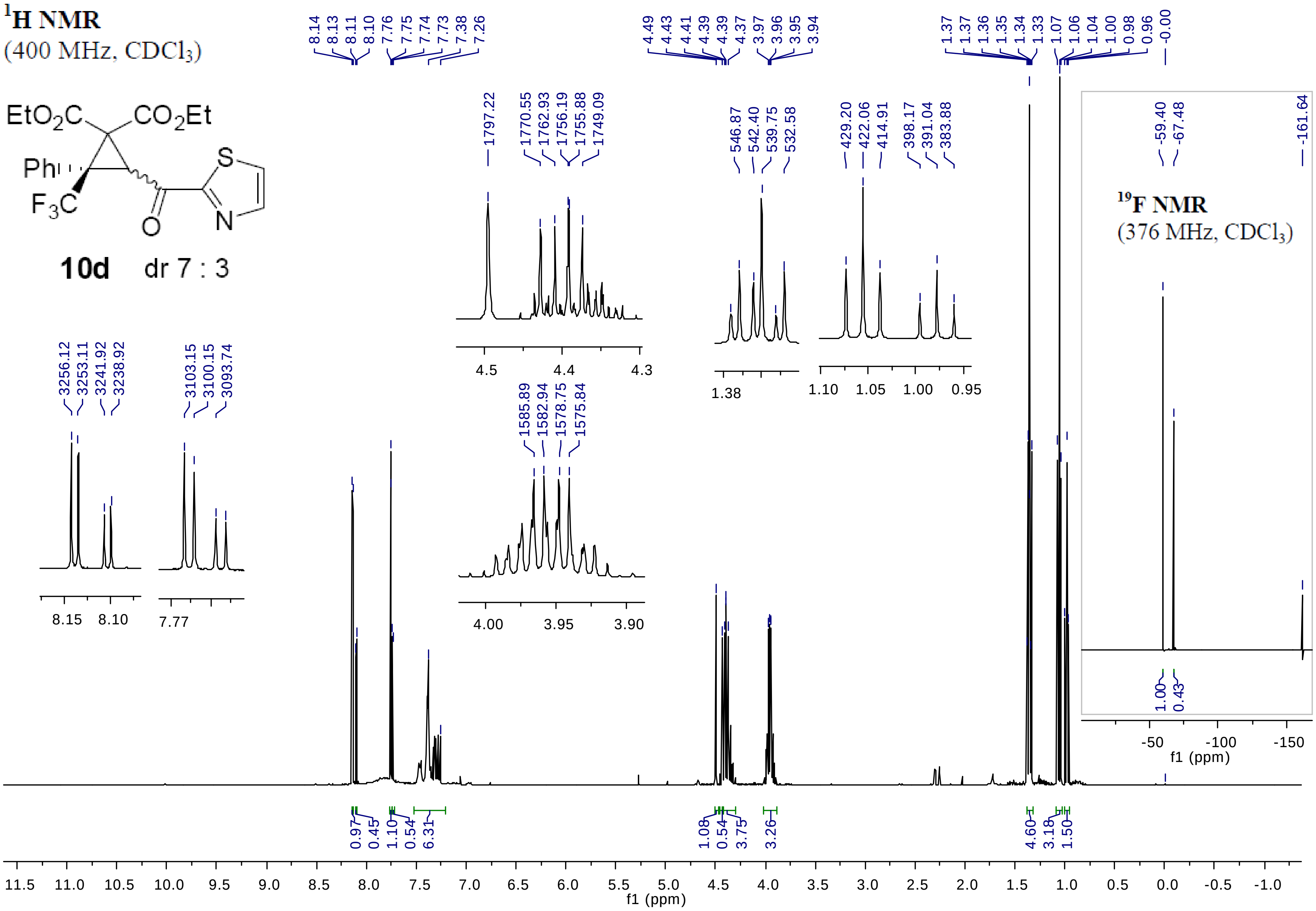

^{1}H NMR
(400 MHz, CDCl$_3$)
EtO$_2$C
CO$_2$Et
Ph
F$_3$C
S
N
O
10d dr 7 : 3
^{19}F NMR
(376 MHz, CDCl$_3$)
-59.40
-67.48
-161.64
1.00
0.43
f1 (ppm)
8.14
8.13
8.11
8.10
7.76
7.75
7.74
7.73
7.38
7.26
4.49
4.43
4.41
4.39
4.39
4.37
3.97
3.96
3.95
3.94
1.37
1.37
1.36
1.35
1.34
1.33
1.07
1.06
1.04
1.00
0.98
0.96
-0.00
0.97
0.45
1.10
0.54
6.31
1.08
0.54
3.75
3.26
4.60
3.18
1.50
f1 (ppm)

^{13}C NMR
(100 MHz, $CDCl_3$)

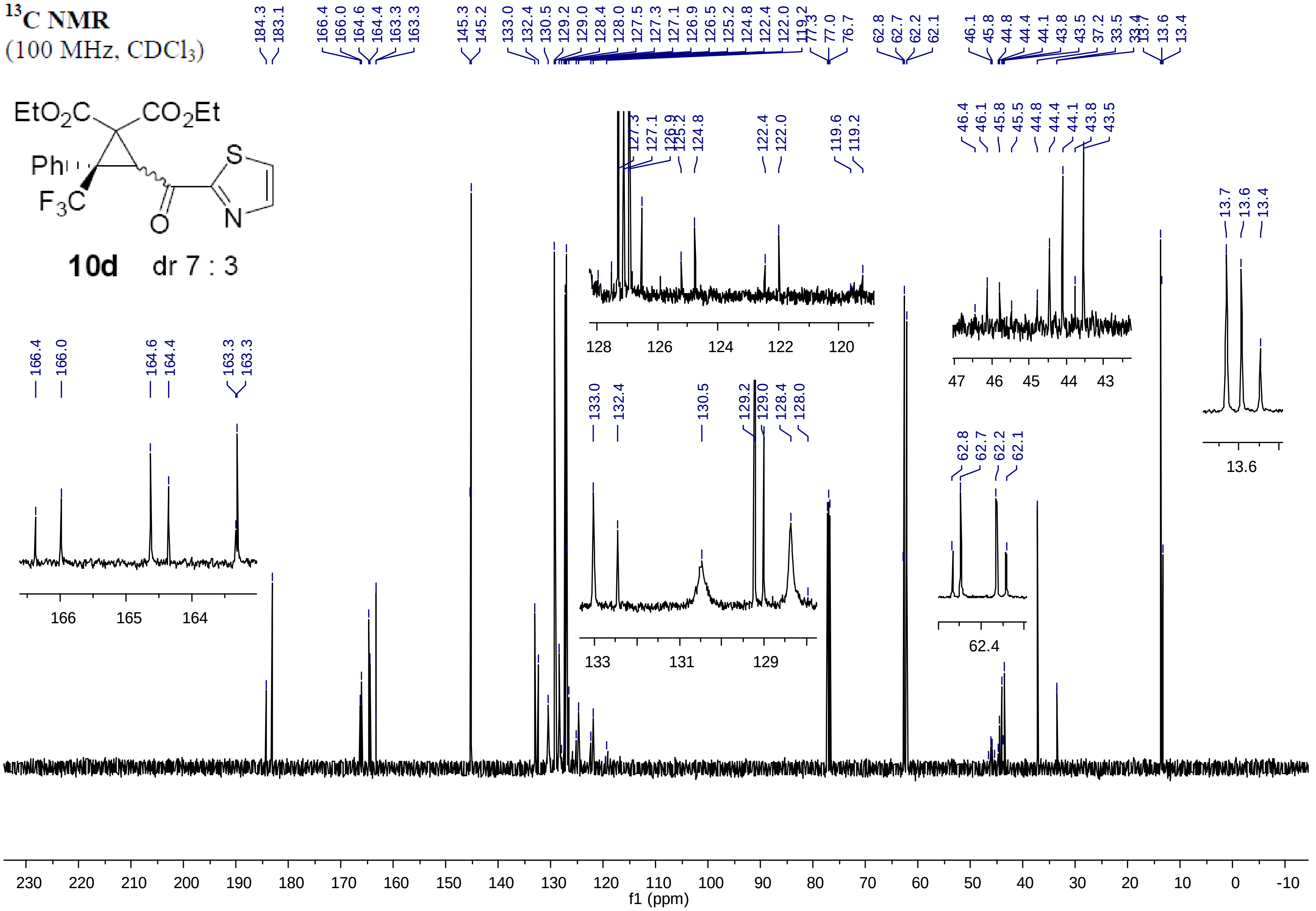

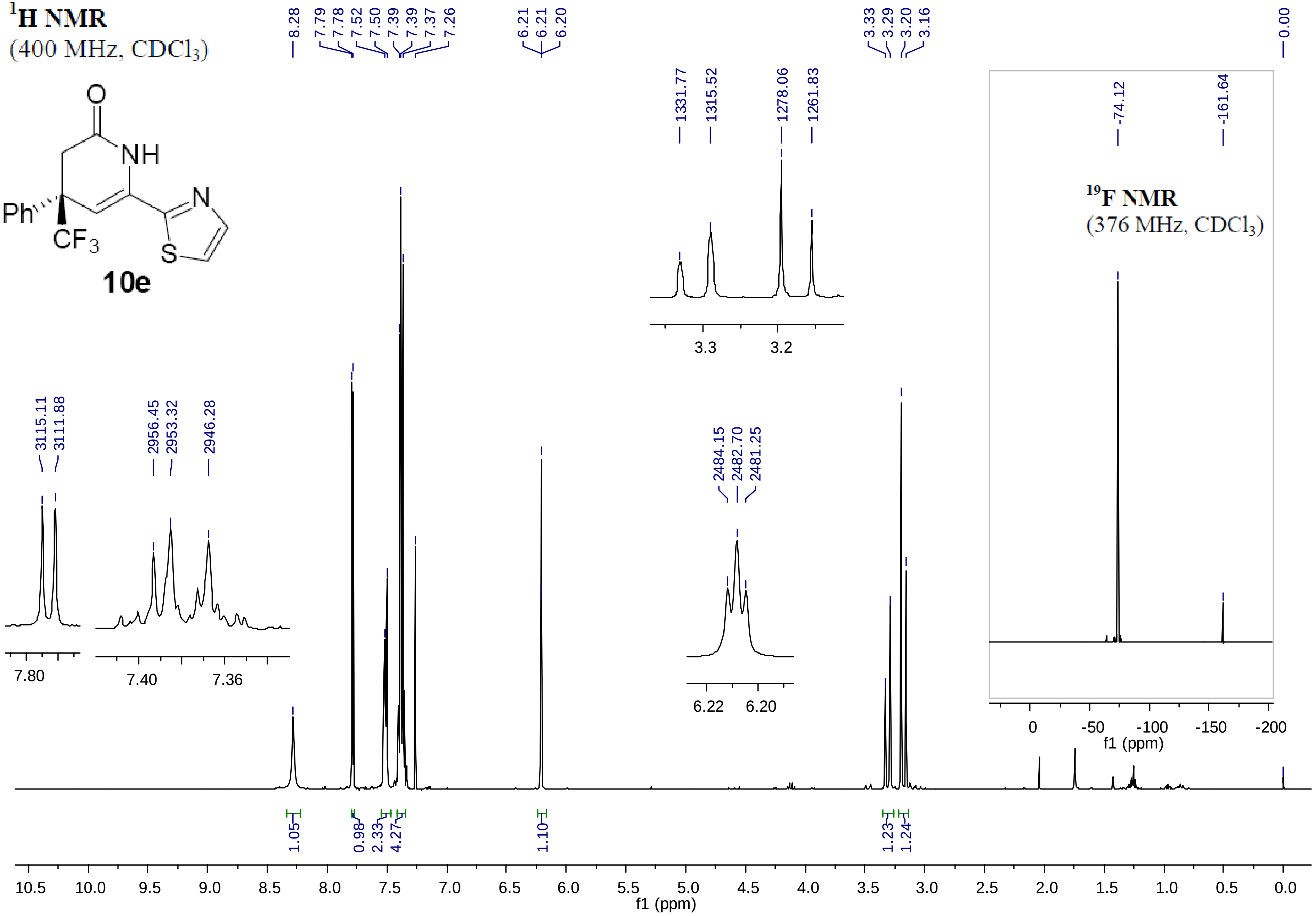

1H NMR
(400 MHz, CDCl3)
10e
19F NMR
(376 MHz, CDCl3)
f1 (ppm)

^{13}C NMR
(100 MHz, $CDCl_3$)

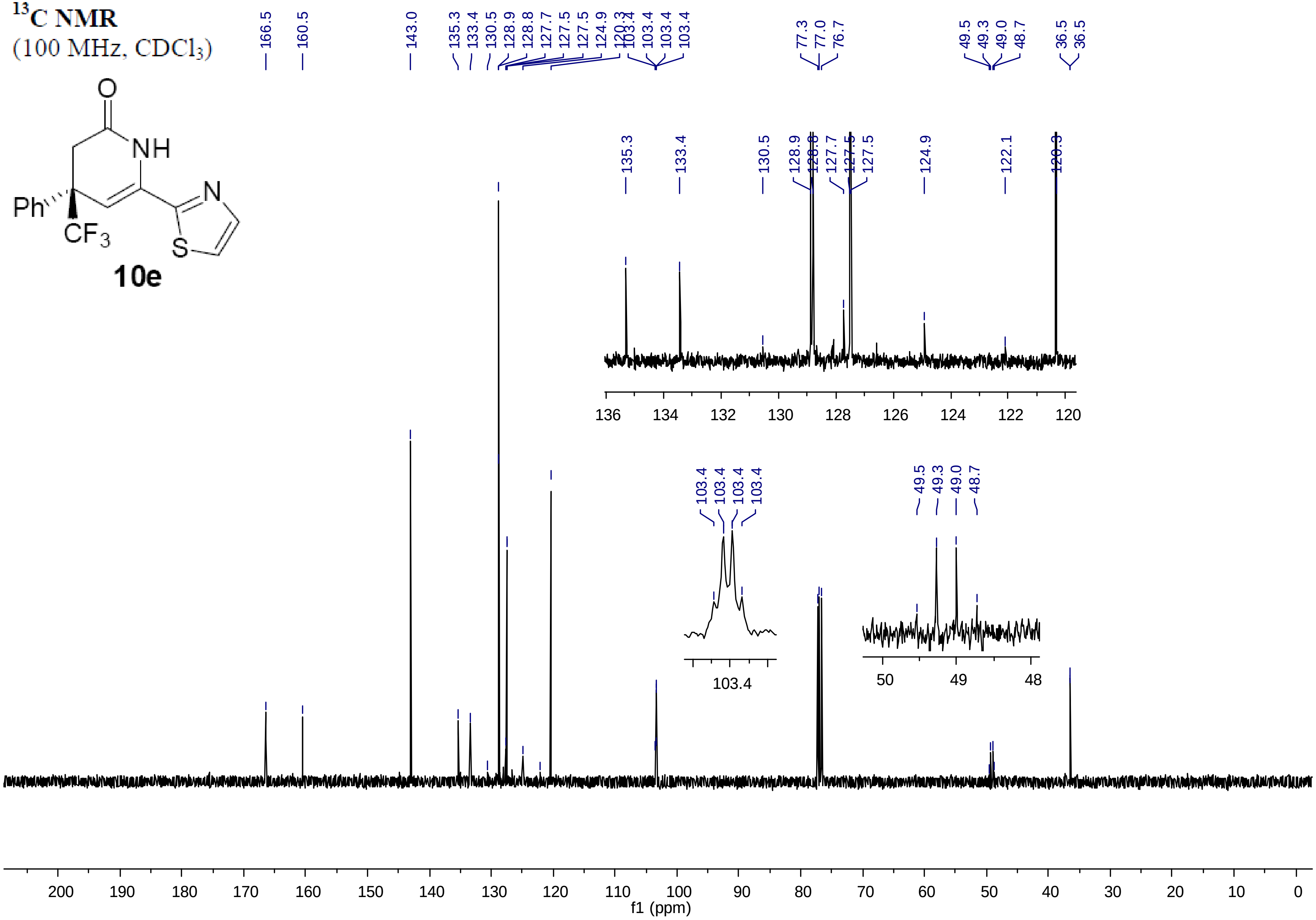

10e

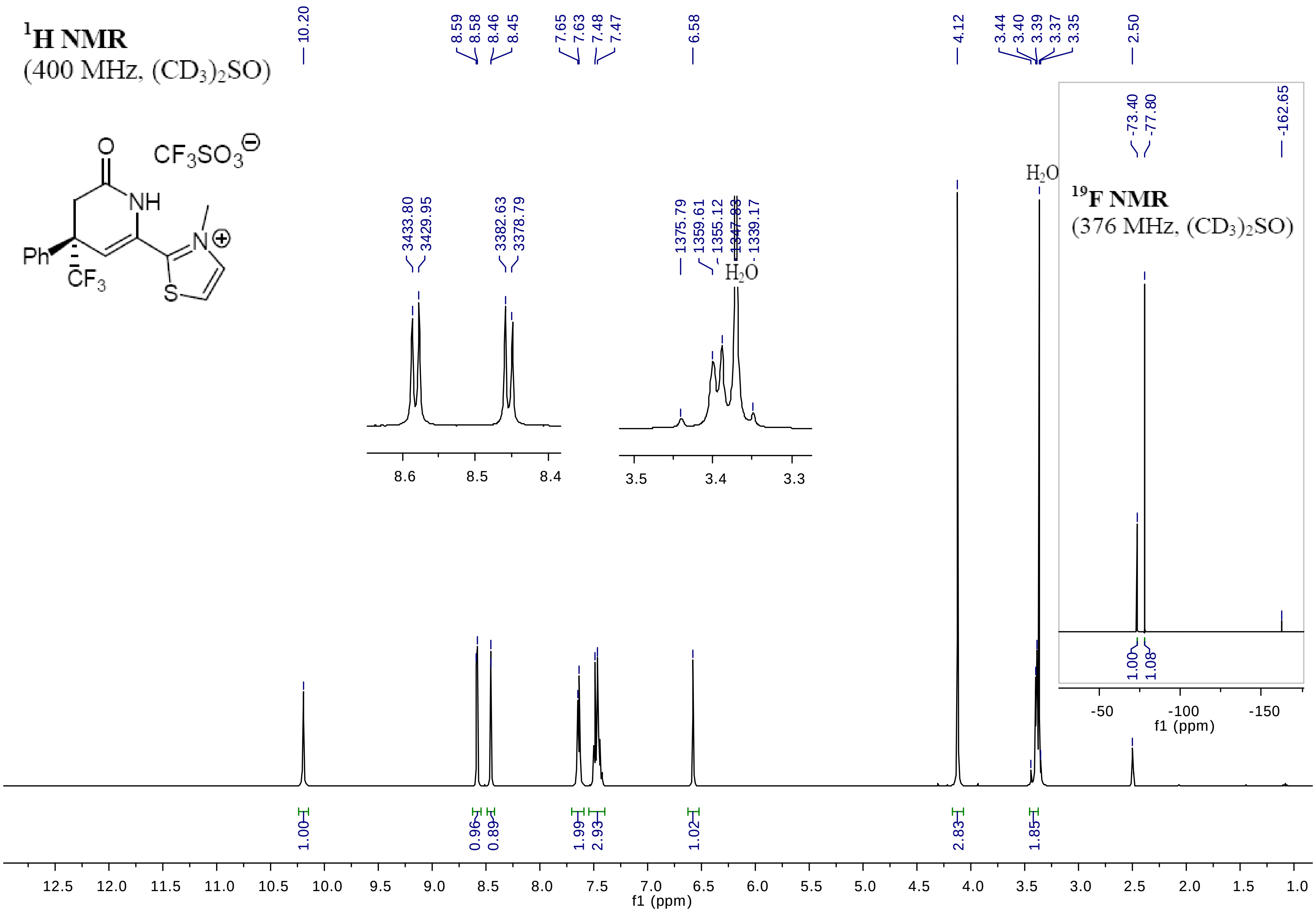

¹H NMR
(400 MHz, (CD₃)₂SO)
O
NH
CF₃SO₃⊖
N⊕
Ph
CF₃
S
10.20
8.59
8.58
8.46
8.45
7.65
7.63
7.48
7.47
6.58
4.12
3.44
3.40
3.39
3.37
3.35
2.50
3433.80
3429.95
3382.63
3378.79
1375.79
1359.61
1355.12
1347.83
1339.17
H₂O
H₂O
8.6
8.5
8.4
3.5
3.4
3.3
¹⁹F NMR
(376 MHz, (CD₃)₂SO)
-73.40
-77.80
-162.65
1.00
1.08
-50
-100
-150
f1 (ppm)
1.00
0.96
0.89
1.99
2.93
1.02
2.83
1.85
12.5
12.0
11.5
11.0
10.5
10.0
9.5
9.0
8.5
8.0
7.5
7.0
6.5
6.0
5.5
5.0
4.5
4.0
3.5
3.0
2.5
2.0
1.5
1.0
f1 (ppm)

^{13}C NMR
(100 MHz, $(CD_3)_2SO$)

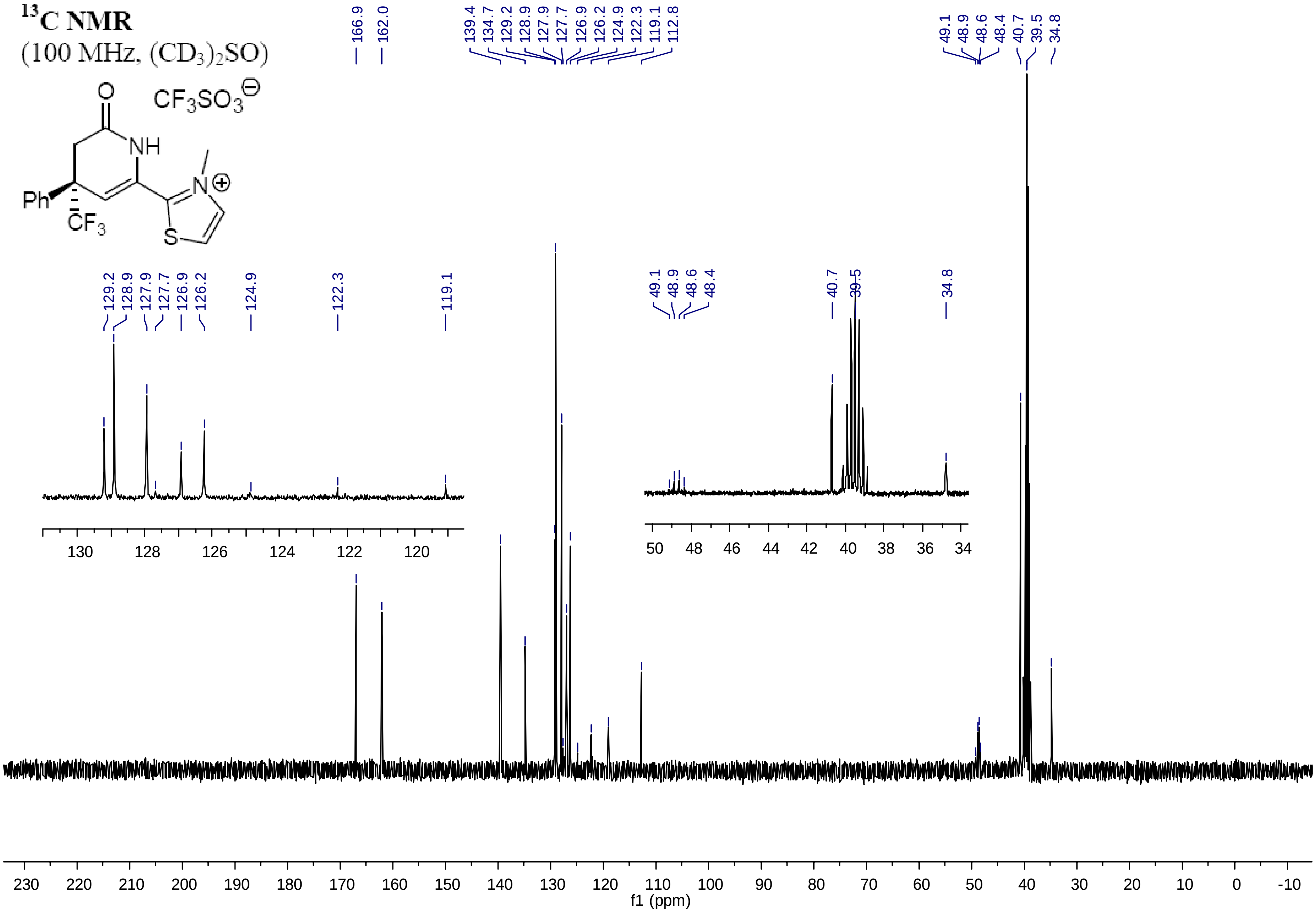

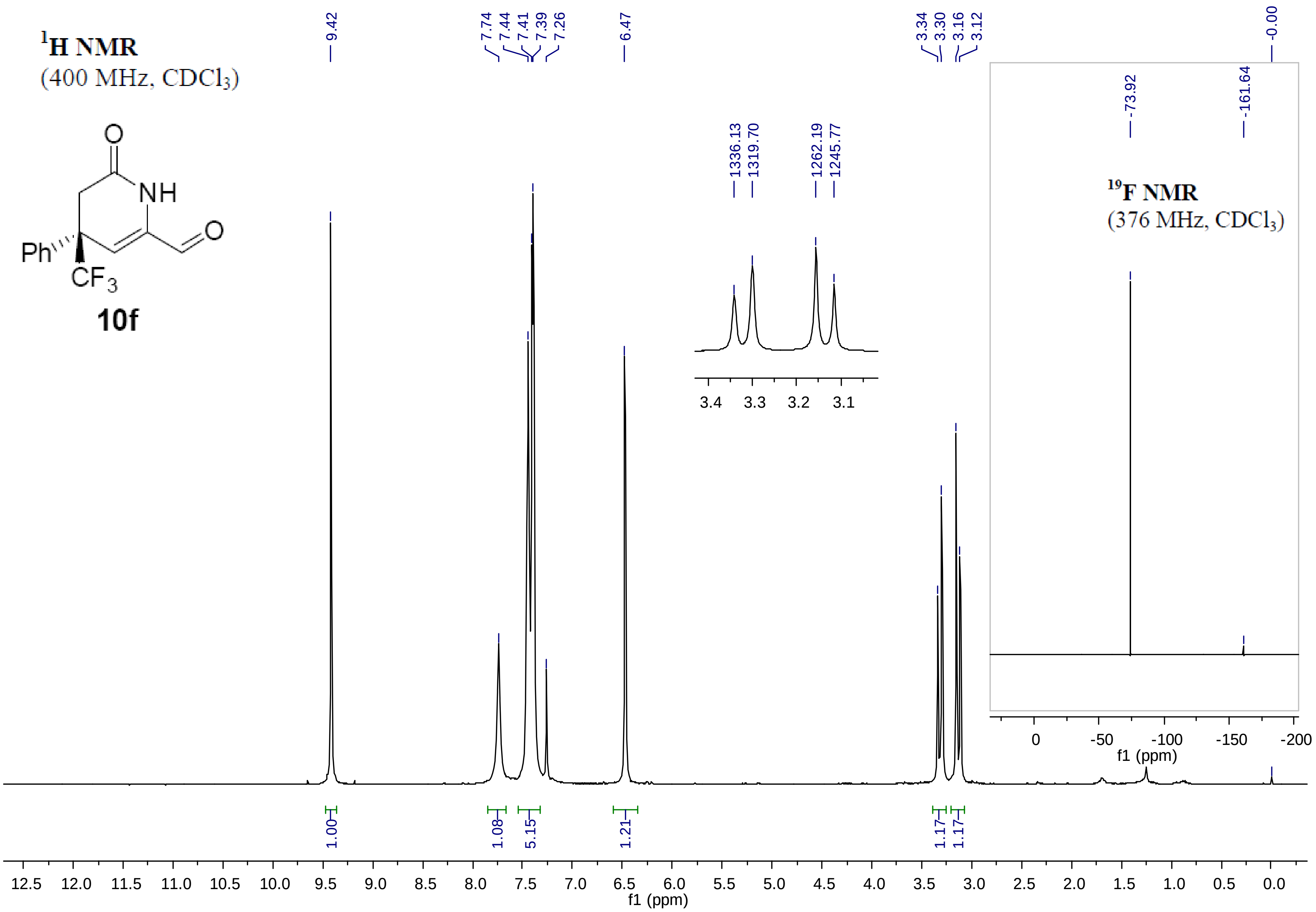

1H NMR
(400 MHz, CDCl3)
NH
O
O
Ph
CF3
10f
9.42
7.74
7.44
7.41
7.39
7.26
6.47
3.34
3.30
3.16
3.12
-0.00
1336.13
1319.70
1262.19
1245.77
3.4
3.3
3.2
3.1
-73.92
-161.64
19F NMR
(376 MHz, CDCl3)
0
-50
-100
-150
-200
f1 (ppm)
1.00
1.08
5.15
1.21
1.17
1.17
12.5
12.0
11.5
11.0
10.5
10.0
9.5
9.0
8.5
8.0
7.5
7.0
6.5
6.0
5.5
5.0
4.5
4.0
3.5
3.0
2.5
2.0
1.5
1.0
0.5
0.0
f1 (ppm)

^{13}C NMR
(100 MHz, $CDCl_3$)

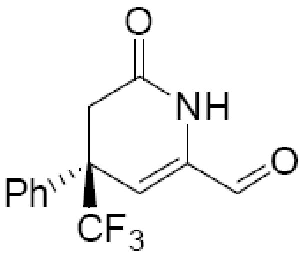

10f

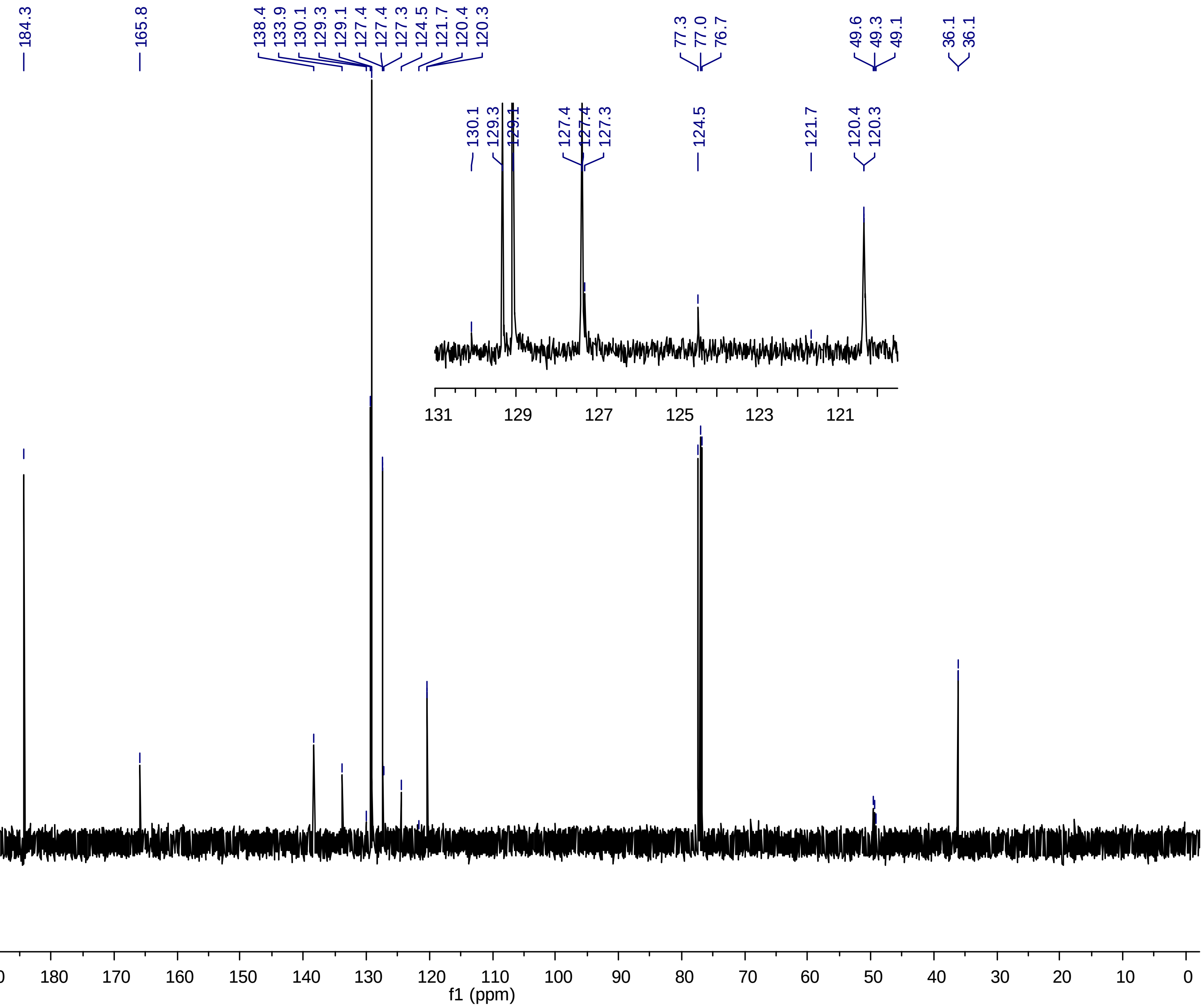

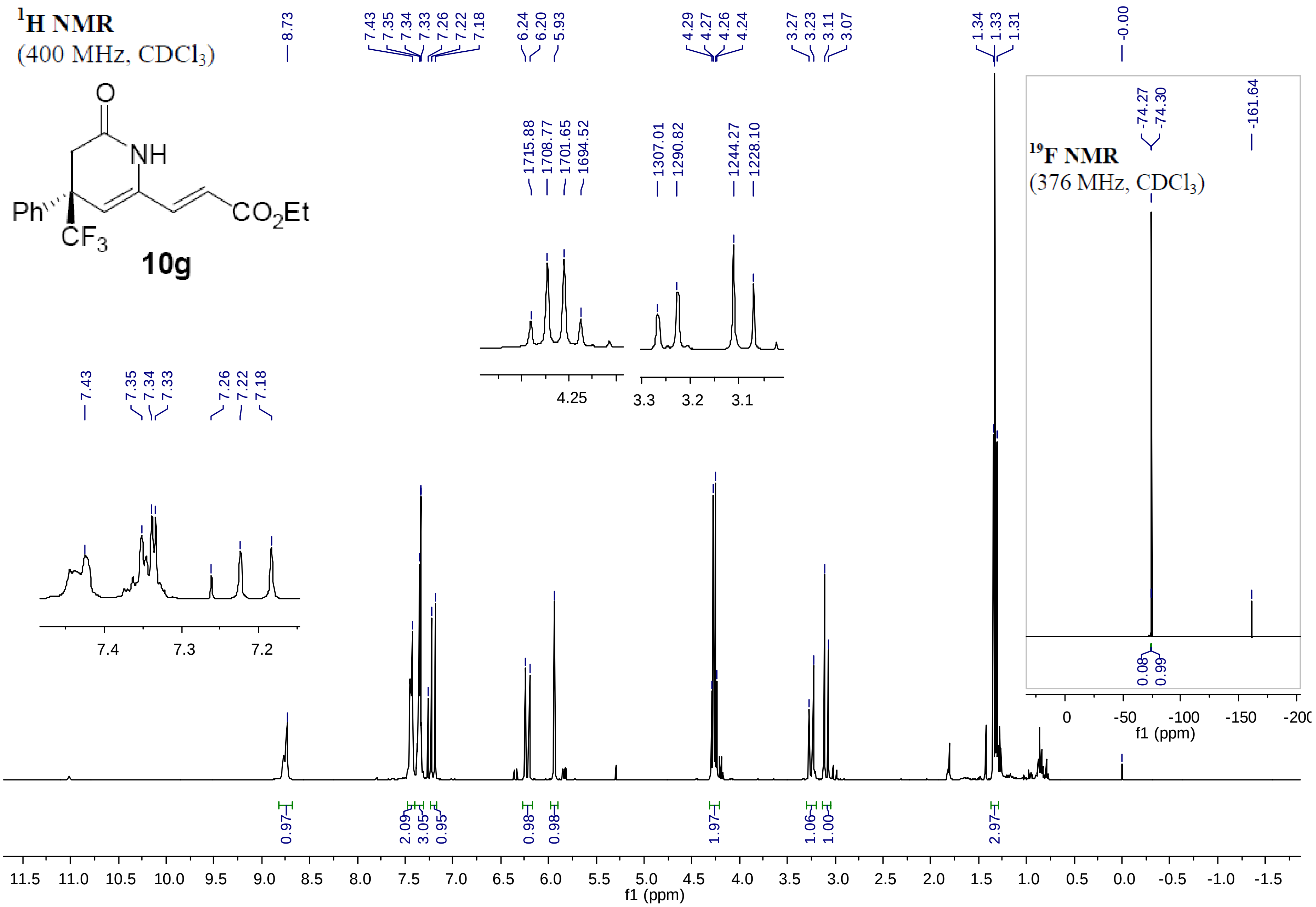
1H NMR
(400 MHz, CDCl3)
10g
19F NMR
(376 MHz, CDCl3)
f1 (ppm)

^{13}C NMR
(100 MHz, $CDCl_3$)

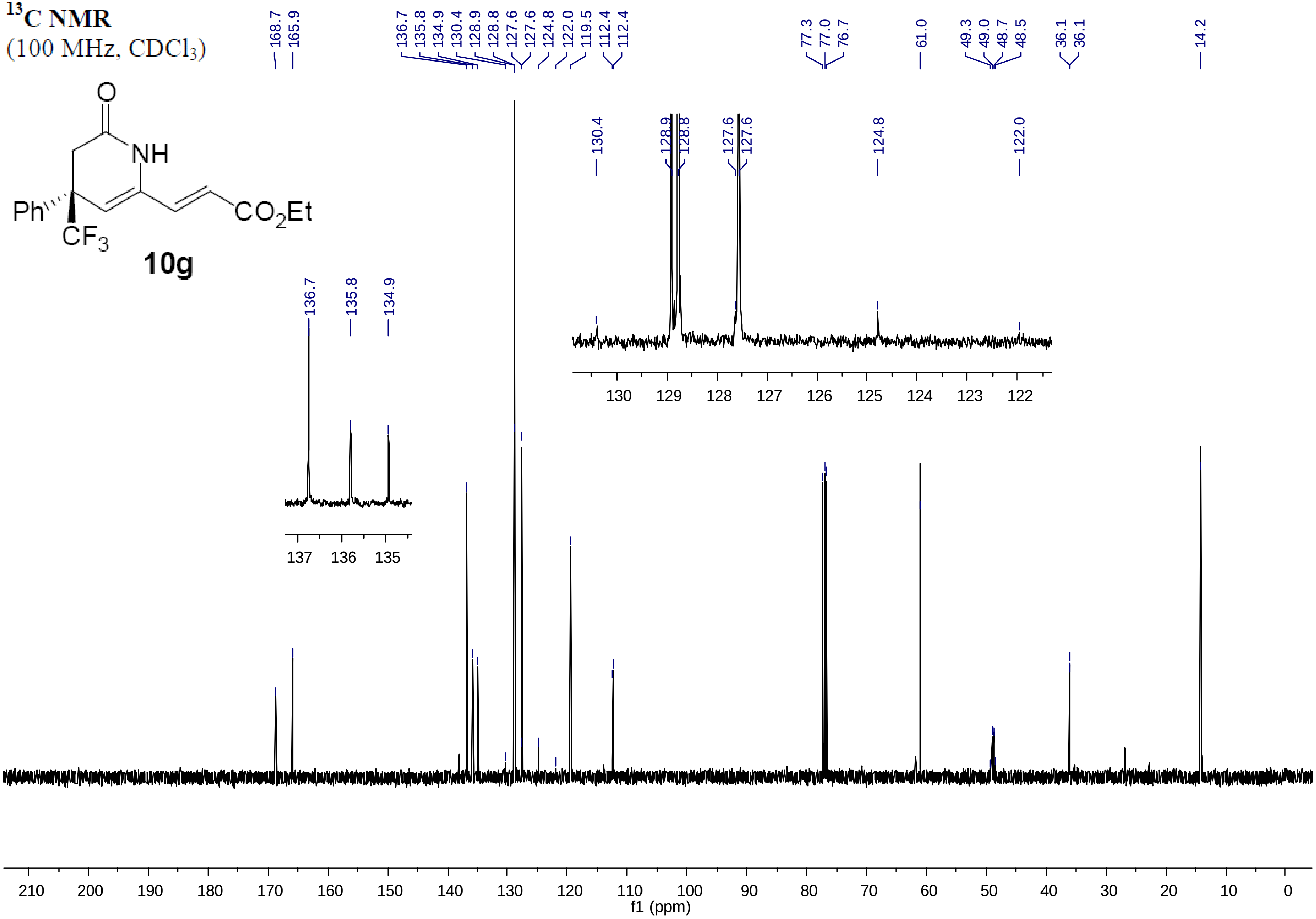

Additional copies of NMR spectra - ^{19}F NMR and DEPT 135

Copies of ^{19}F NMR spectra - enones 2e-2k, 4a-4u, 6a-6k, 8a and 8b

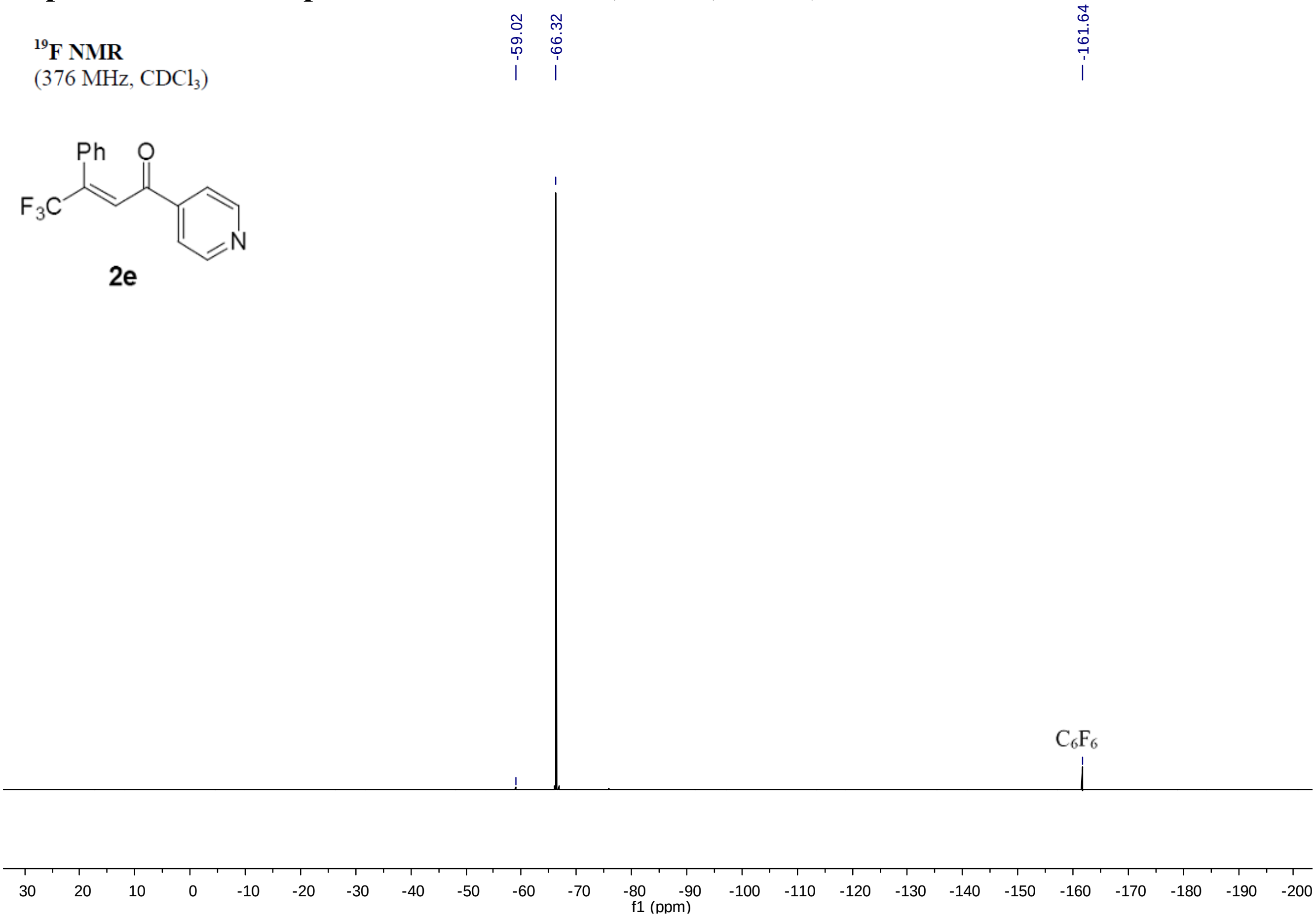

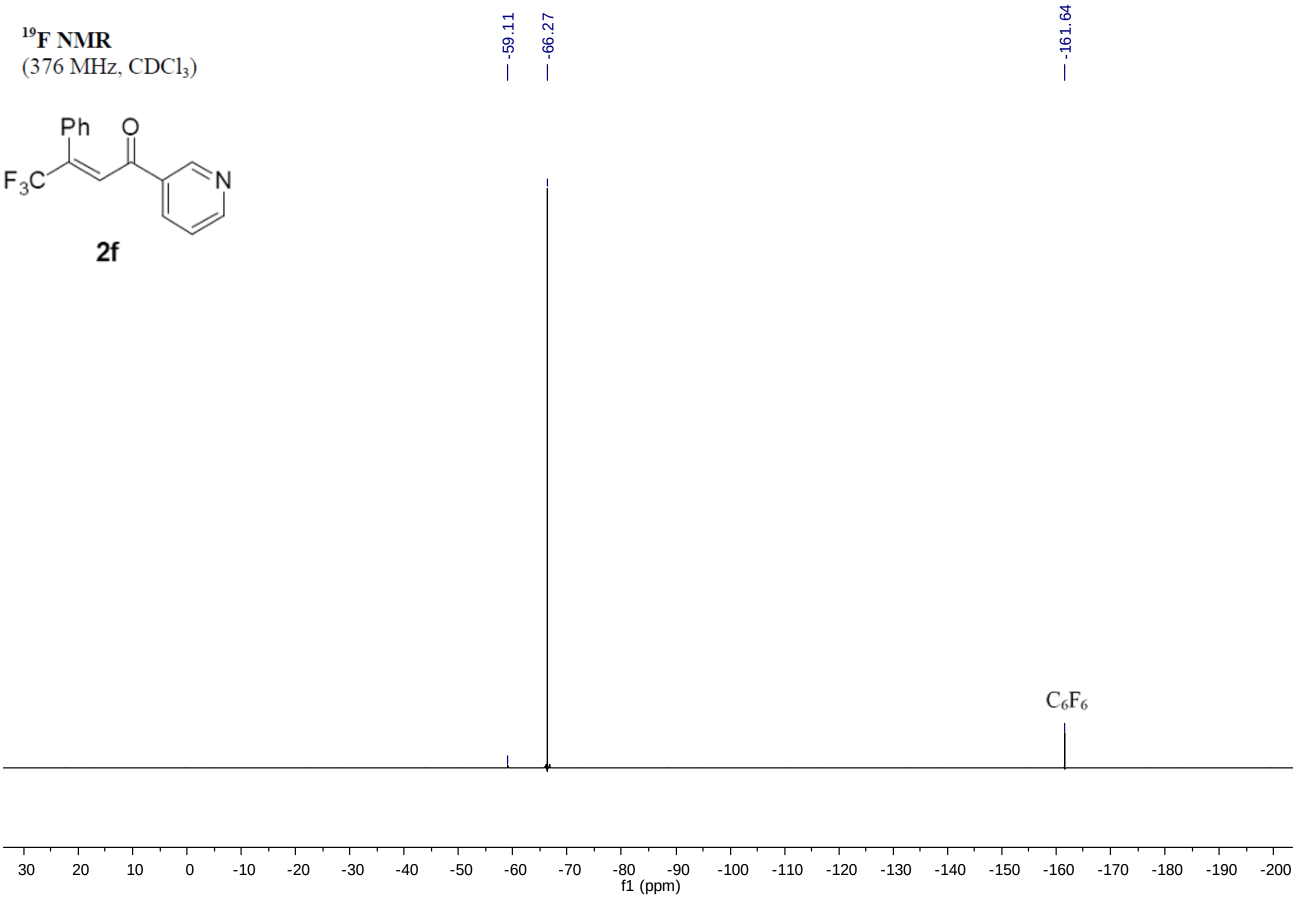

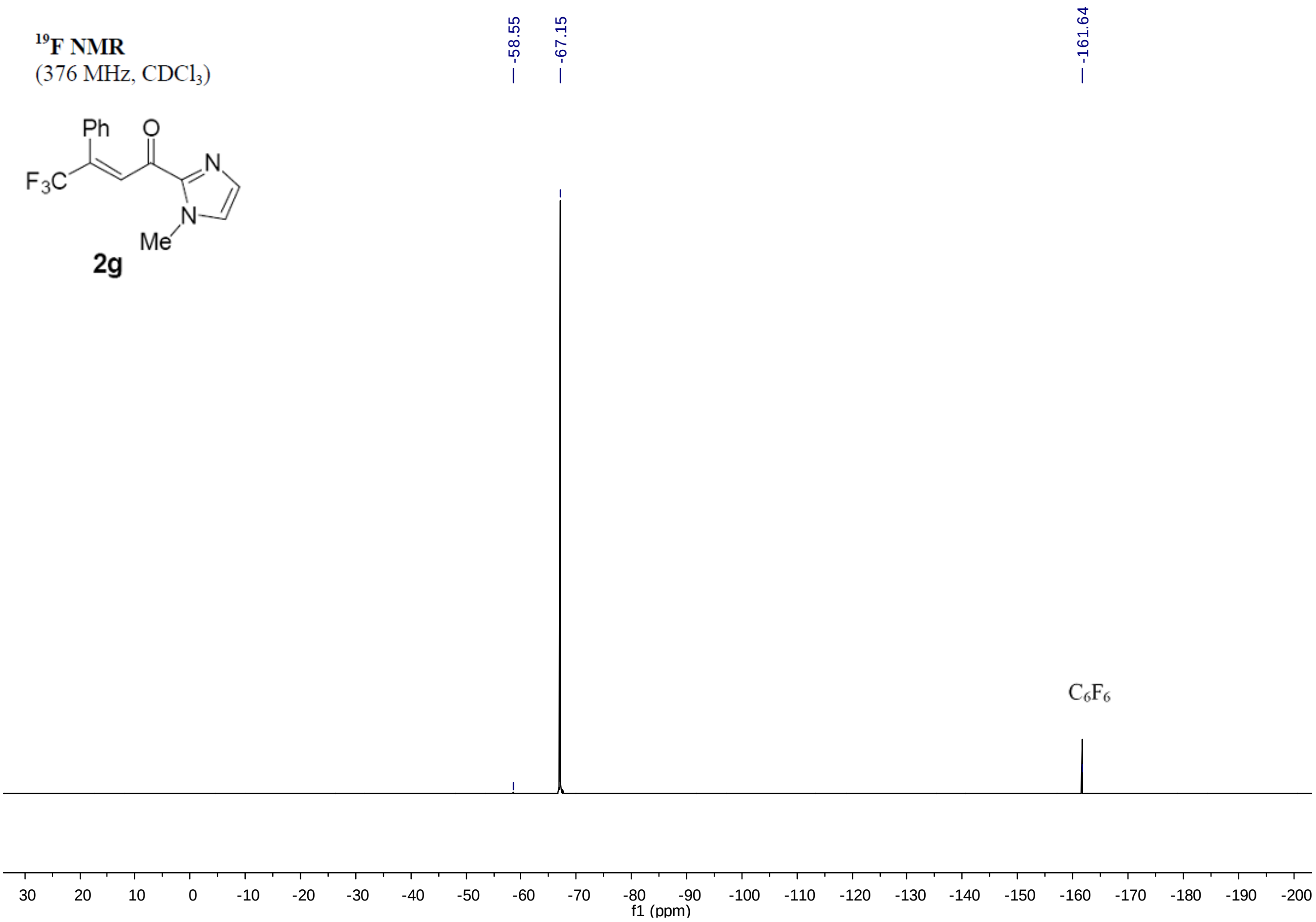

19F NMR
(376 MHz, CDCl3)
-58.55
-67.15
-161.64
Ph
O
F3C
N
N
Me
2g
C6F6
30 20 10 0 -10 -20 -30 -40 -50 -60 -70 -80 -90 -100 -110 -120 -130 -140 -150 -160 -170 -180 -190 -200
f1 (ppm)

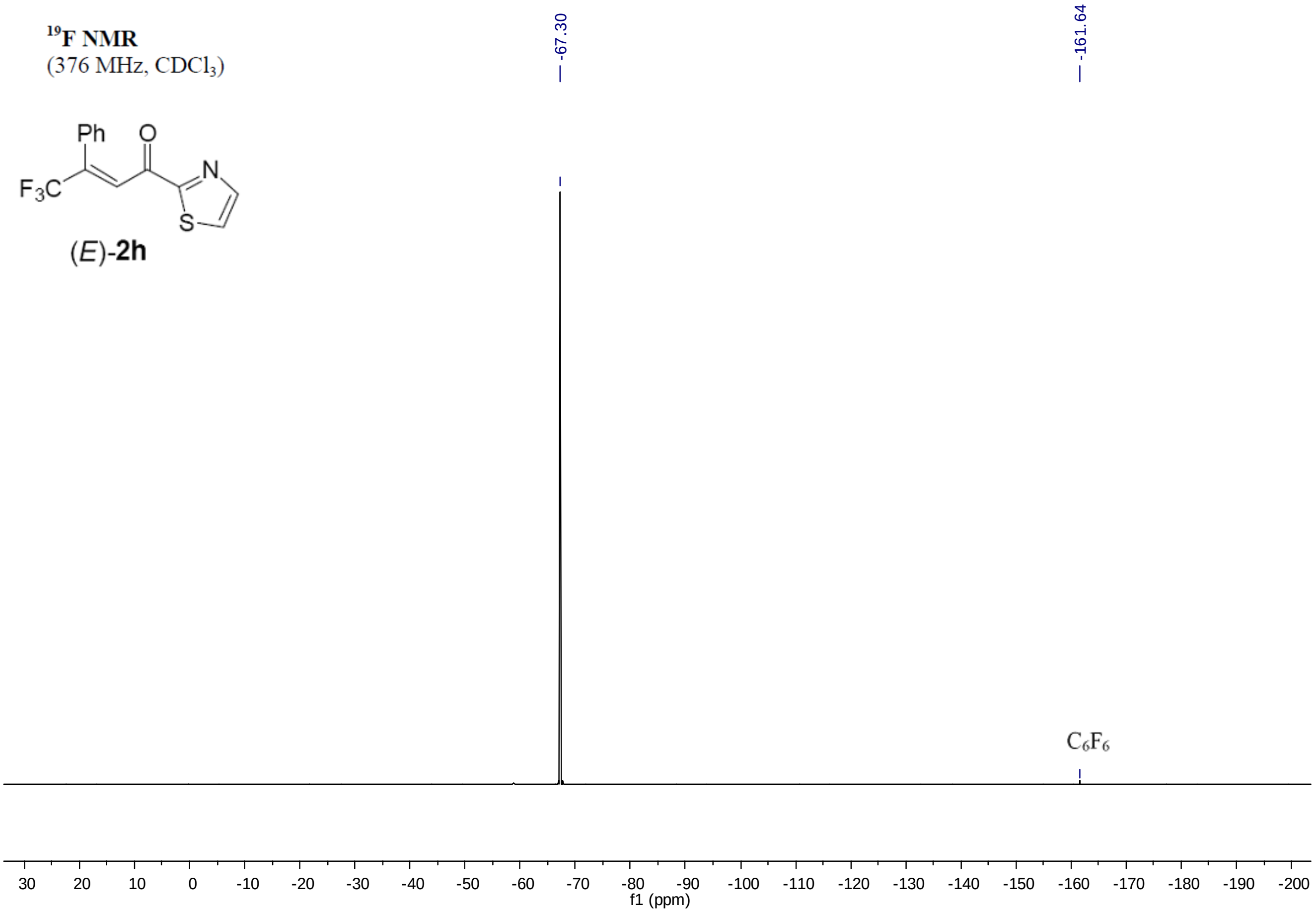

19F NMR
(376 MHz, CDCl3)
-67.30
-161.64
Ph
O
F3C
N
S
(E)-2h
C6F6
30 20 10 0 -10 -20 -30 -40 -50 -60 -70 -80 -90 -100 -110 -120 -130 -140 -150 -160 -170 -180 -190 -200
f1 (ppm)

^{19}F NMR
(376 MHz, $CDCl_3$)

(Z)-**2h**
(minor isomer)

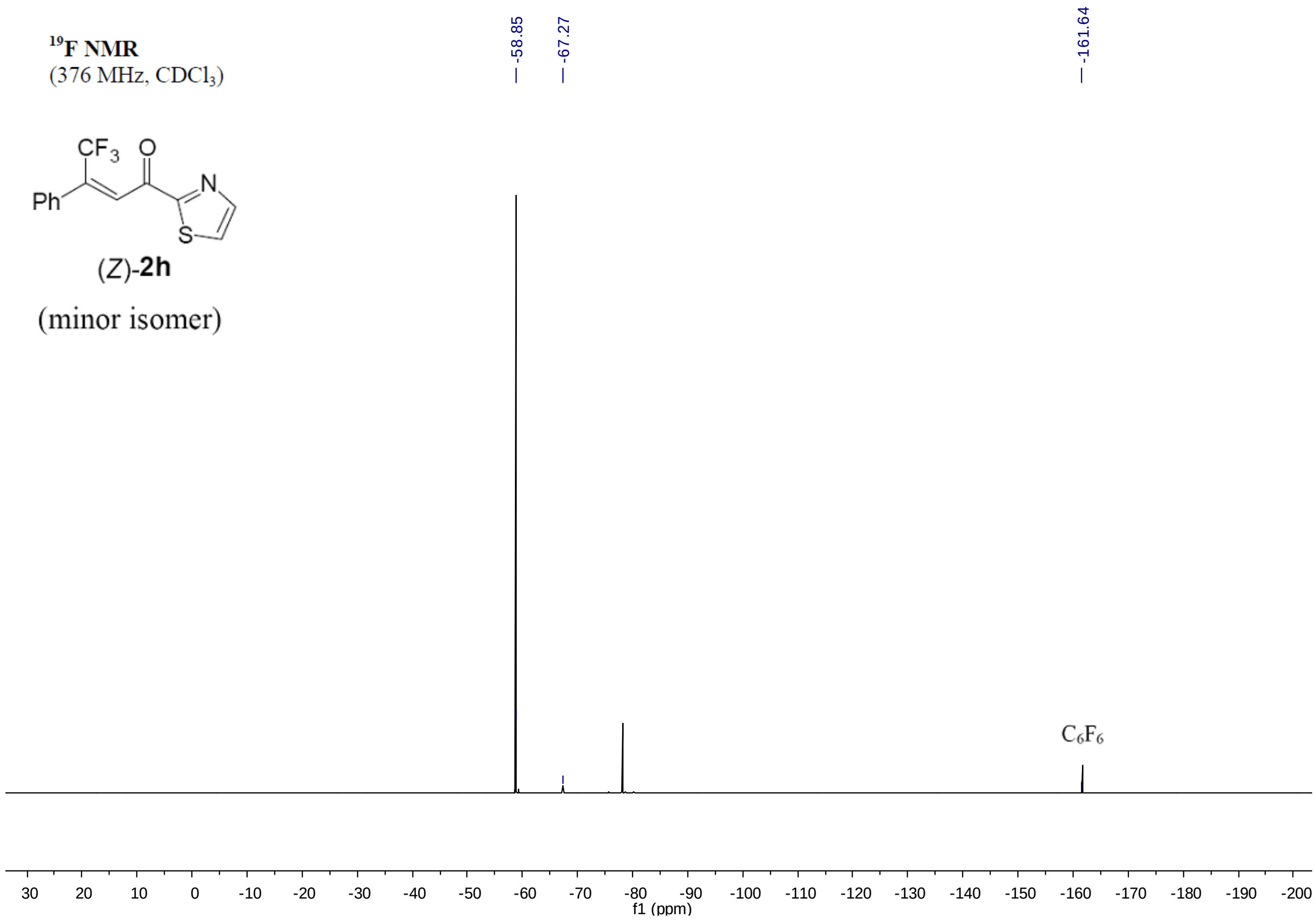

^{19}F NMR
(376 MHz, $CDCl_3$)

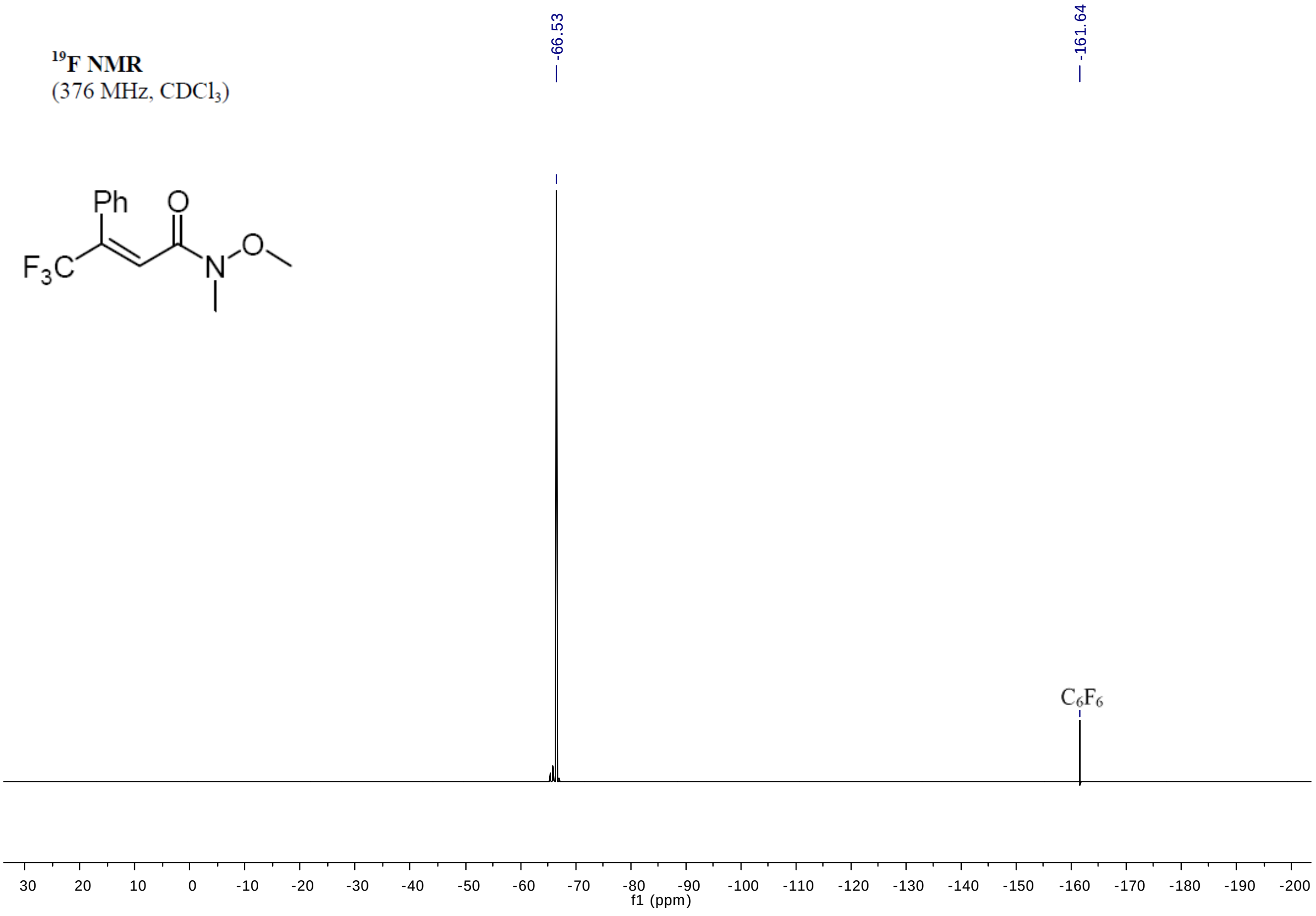

^{19}F NMR
(376 MHz, $CDCl_3$)

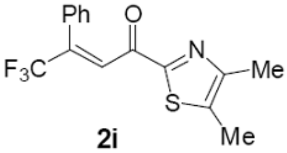

2i

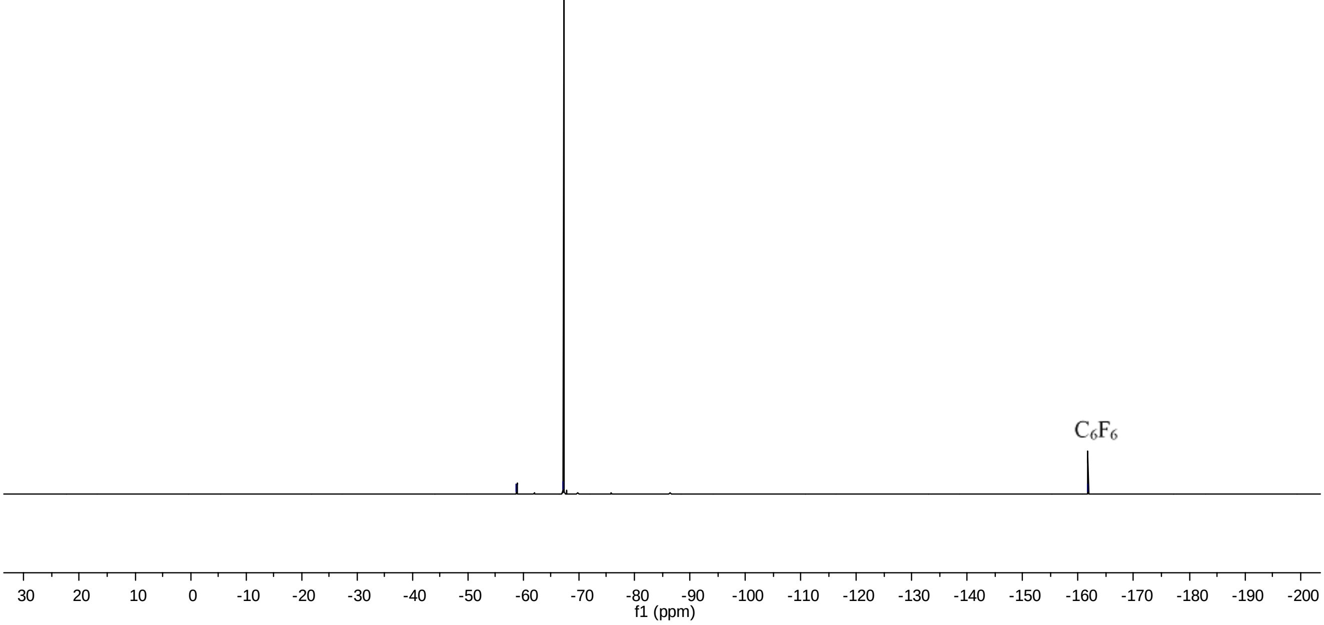

^{19}F NMR
(376 MHz, $CDCl_3$)

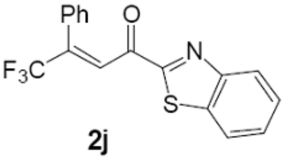

2j

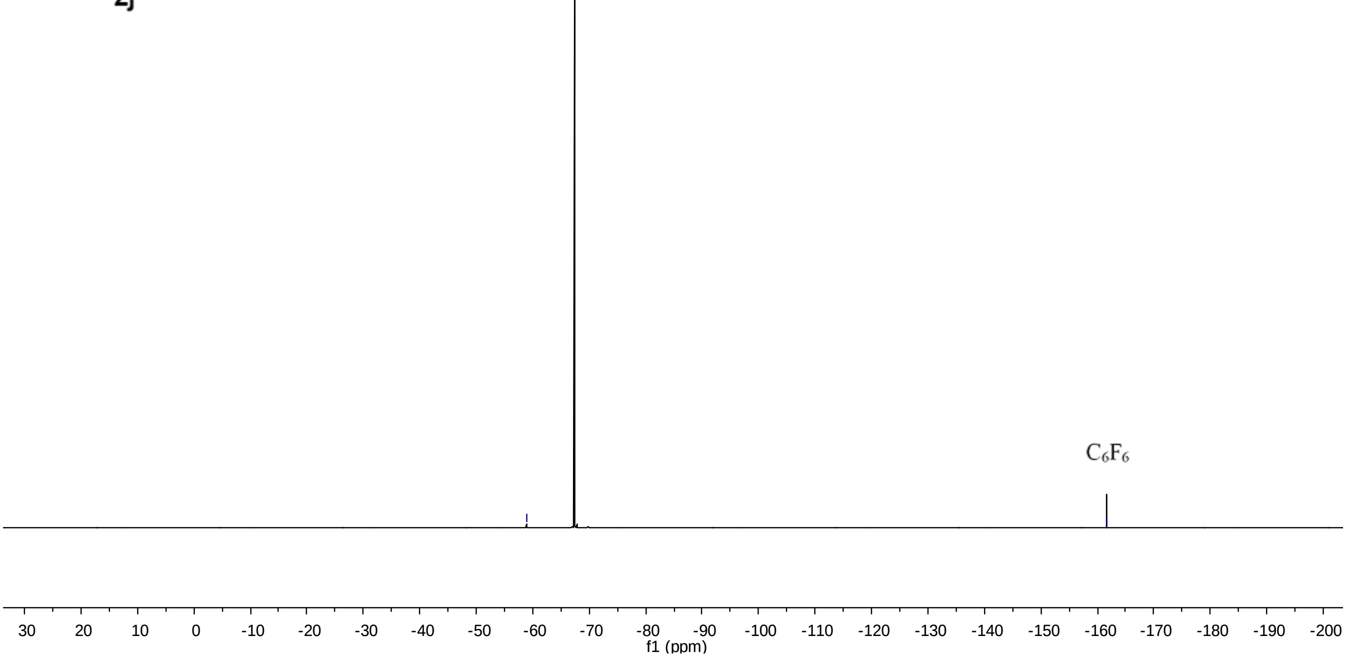

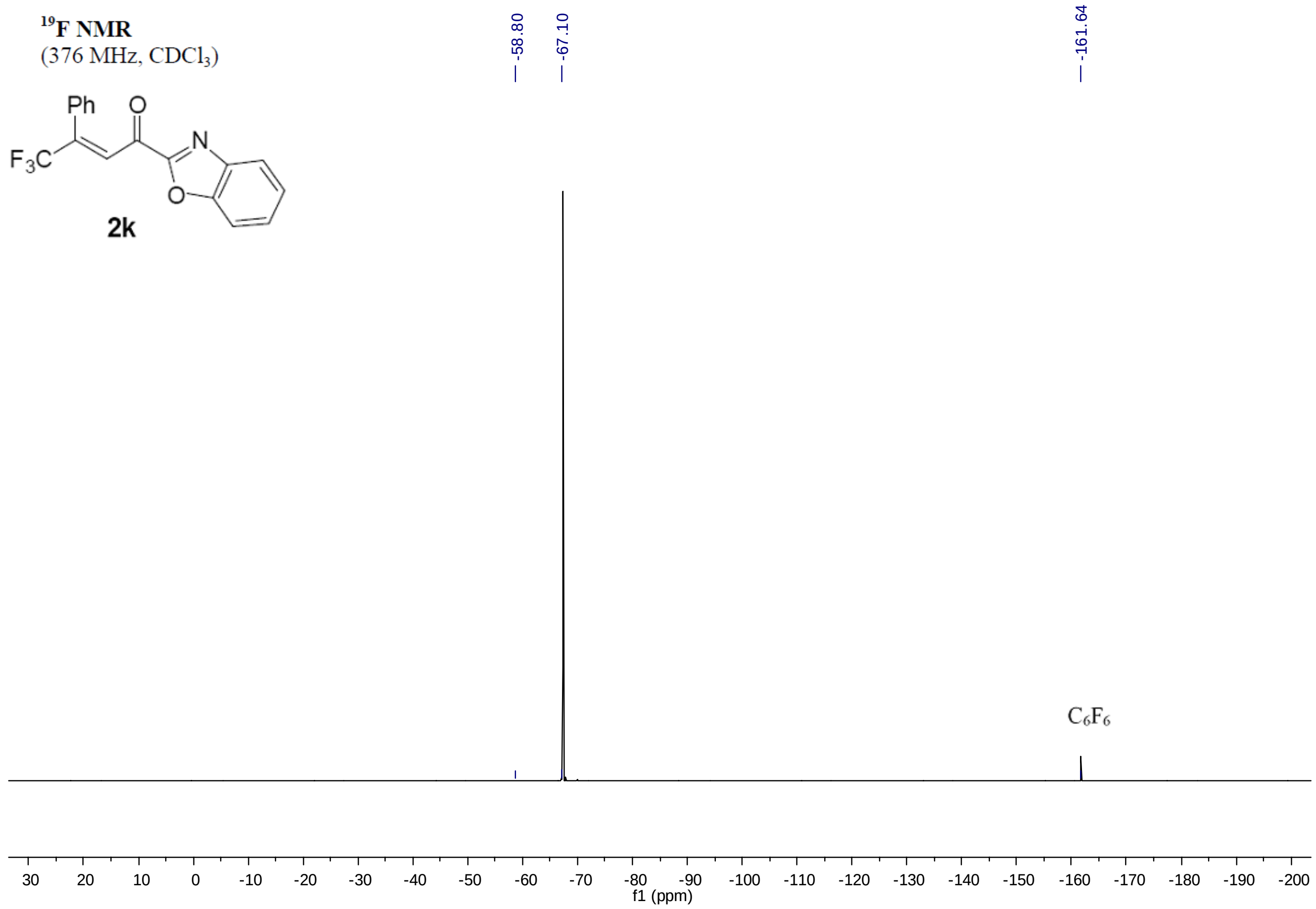

^{19}F NMR
(376 MHz, CDCl$_3$)
2k
-58.80
-67.10
-161.64
C$_6$F$_6$
f1 (ppm)

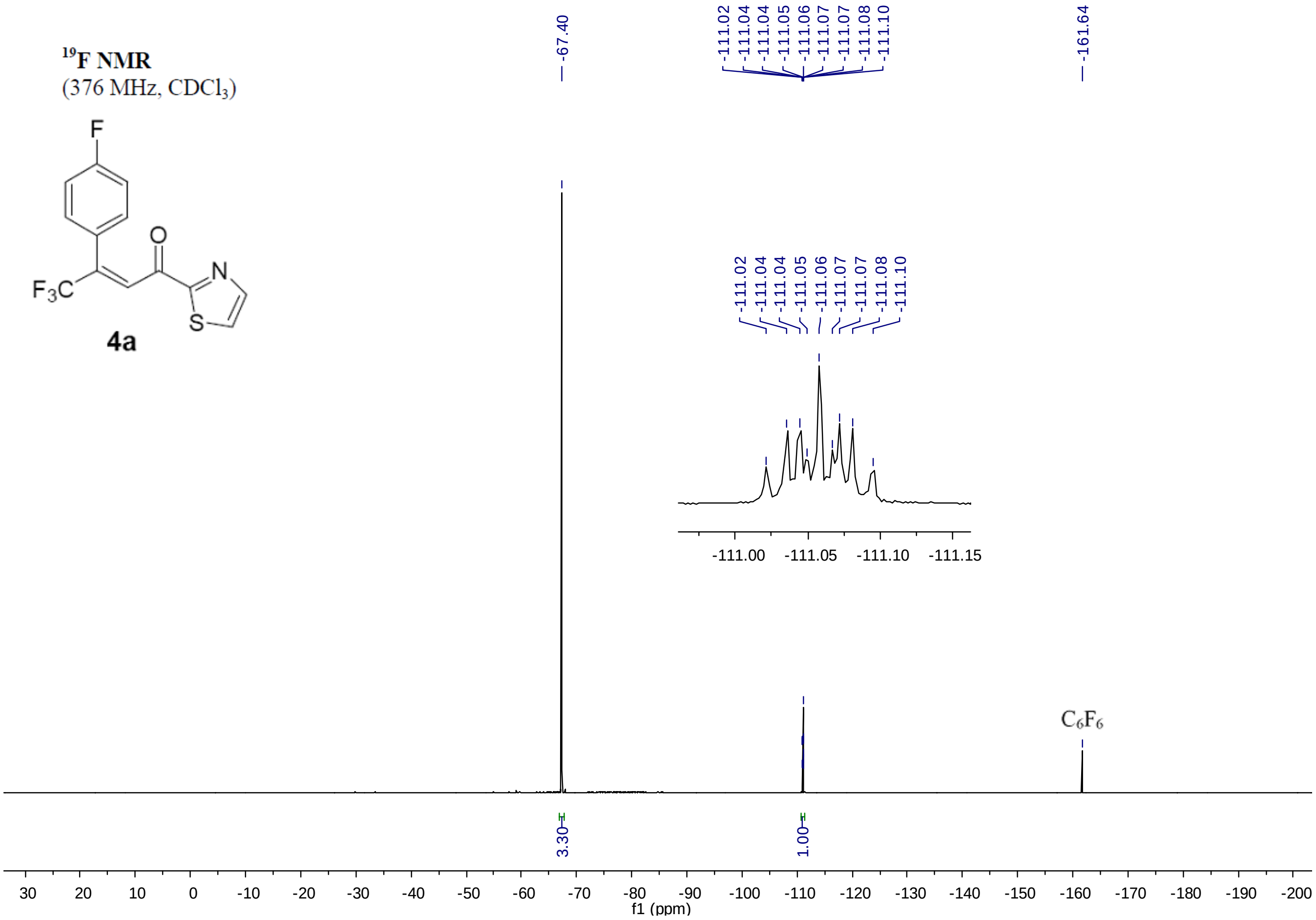

^{19}F NMR
(376 MHz, CDCl$_3$)
4a
-67.40
-111.02
-111.04
-111.04
-111.05
-111.06
-111.07
-111.07
-111.08
-111.10
-161.64
C$_6$F$_6$
3.30
1.00
f1 (ppm)

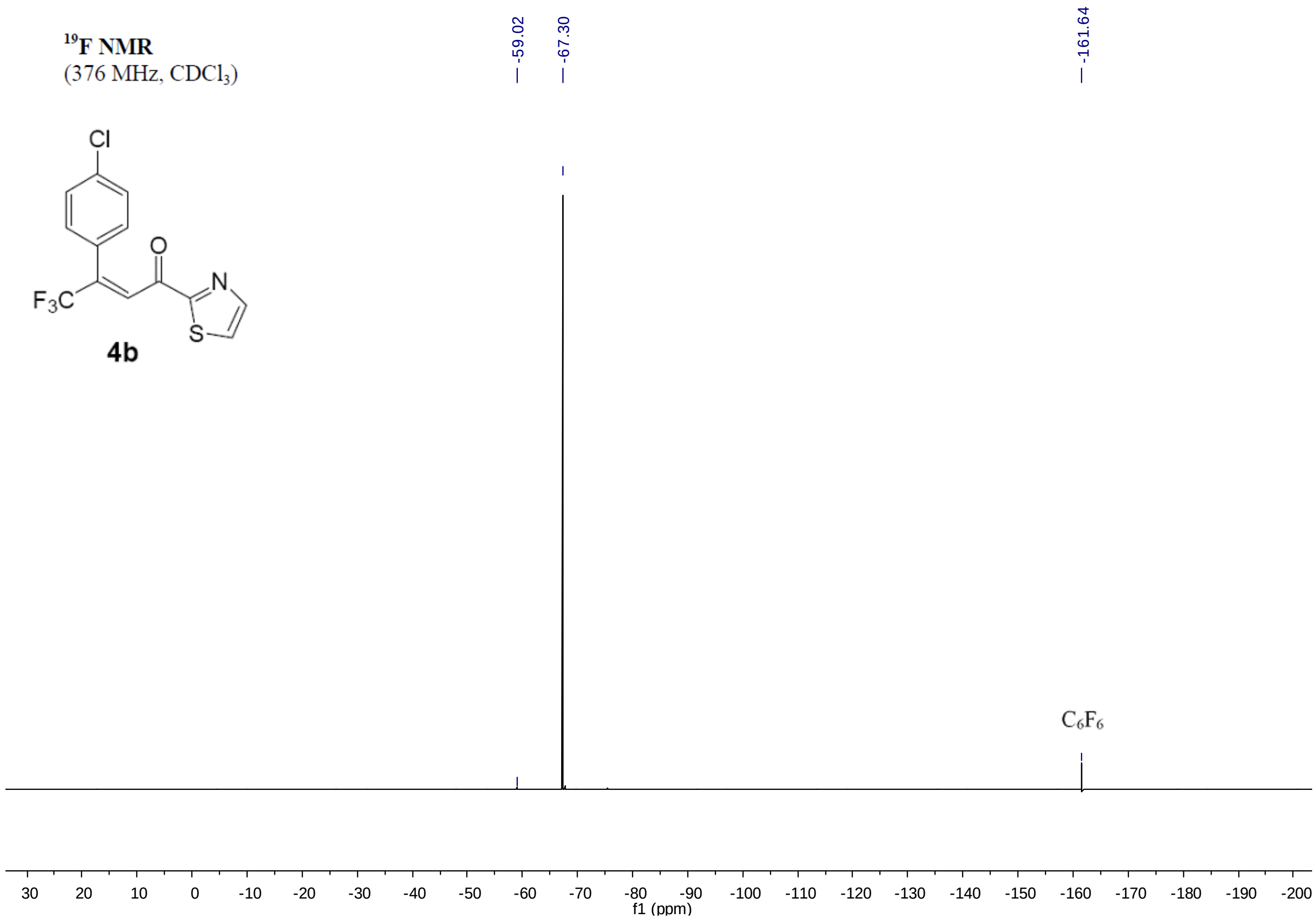

19F NMR
(376 MHz, CDCl3)
-59.02
-67.30
-161.64
Cl
O
N
F3C
S
4b
C6F6
30 20 10 0 -10 -20 -30 -40 -50 -60 -70 -80 -90 -100 -110 -120 -130 -140 -150 -160 -170 -180 -190 -200
f1 (ppm)

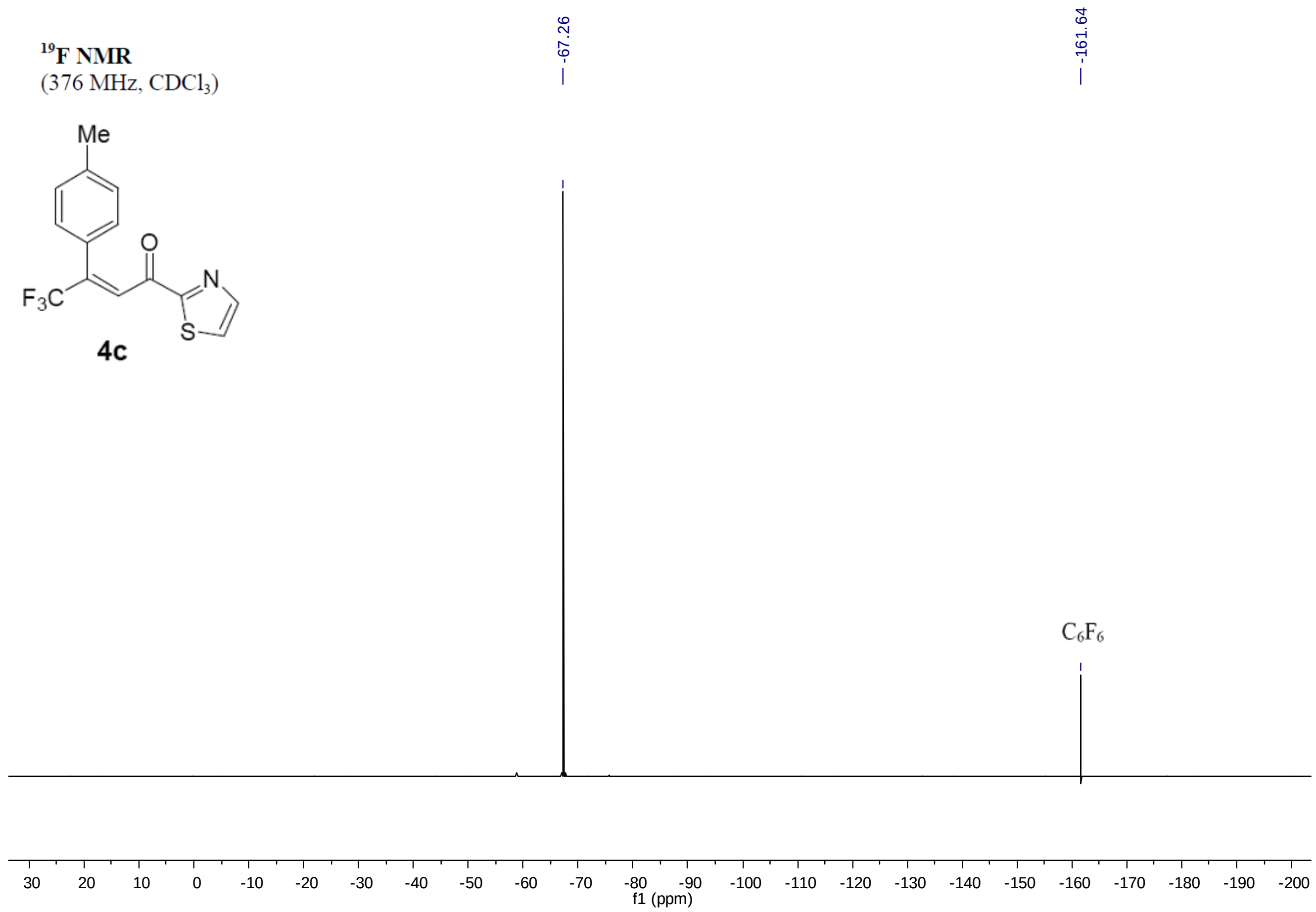

19F NMR
(376 MHz, CDCl3)
-67.26
-161.64
Me
O
N
F3C
S
4c
C6F6
30 20 10 0 -10 -20 -30 -40 -50 -60 -70 -80 -90 -100 -110 -120 -130 -140 -150 -160 -170 -180 -190 -200
f1 (ppm)

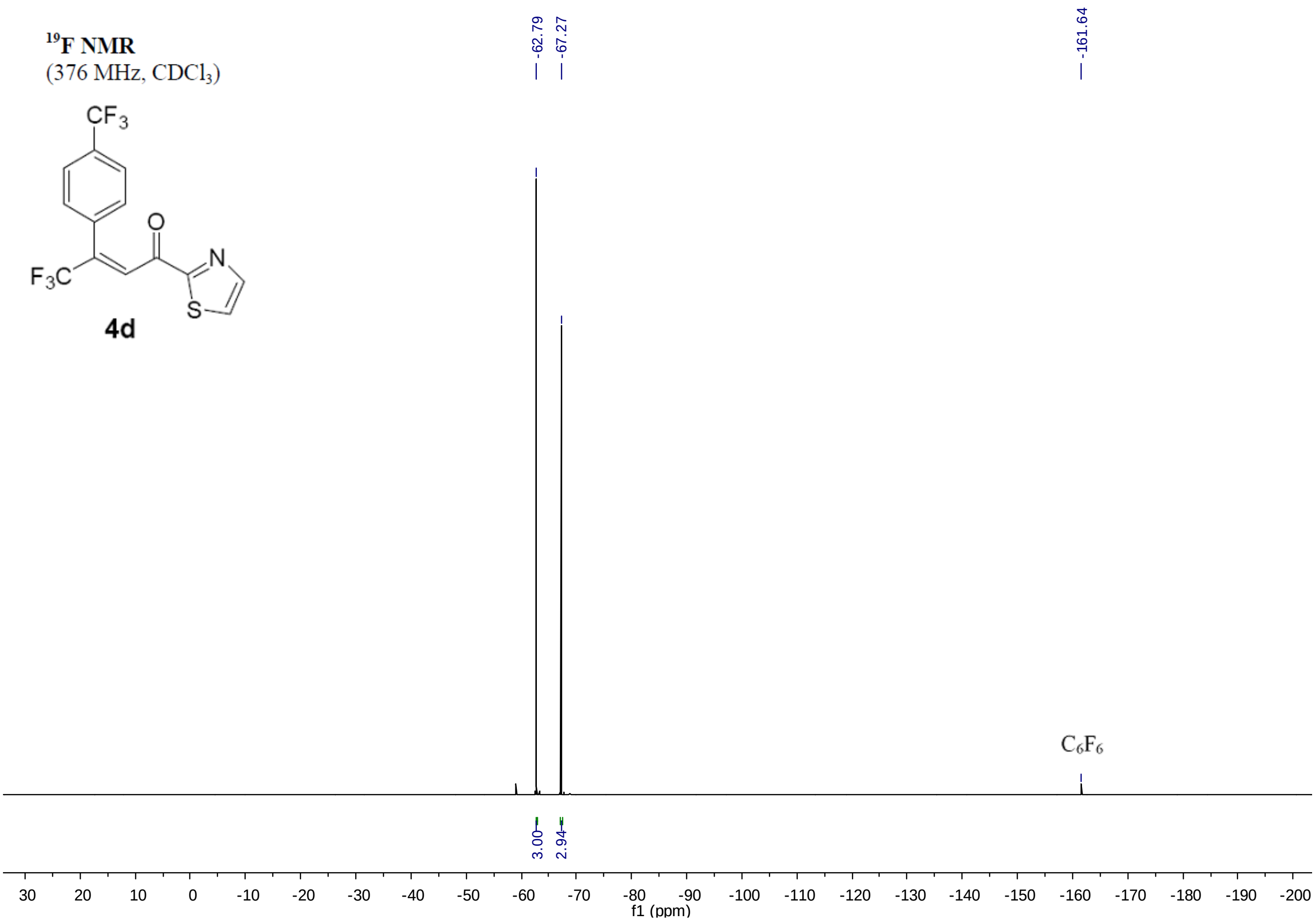
19F NMR
(376 MHz, CDCl3)
CF3
O
N
F3C
S
4d
-62.79
-67.27
-161.64
C6F6
3.00
2.94
30 20 10 0 -10 -20 -30 -40 -50 -60 -70 -80 -90 -100 -110 -120 -130 -140 -150 -160 -170 -180 -190 -200
f1 (ppm)

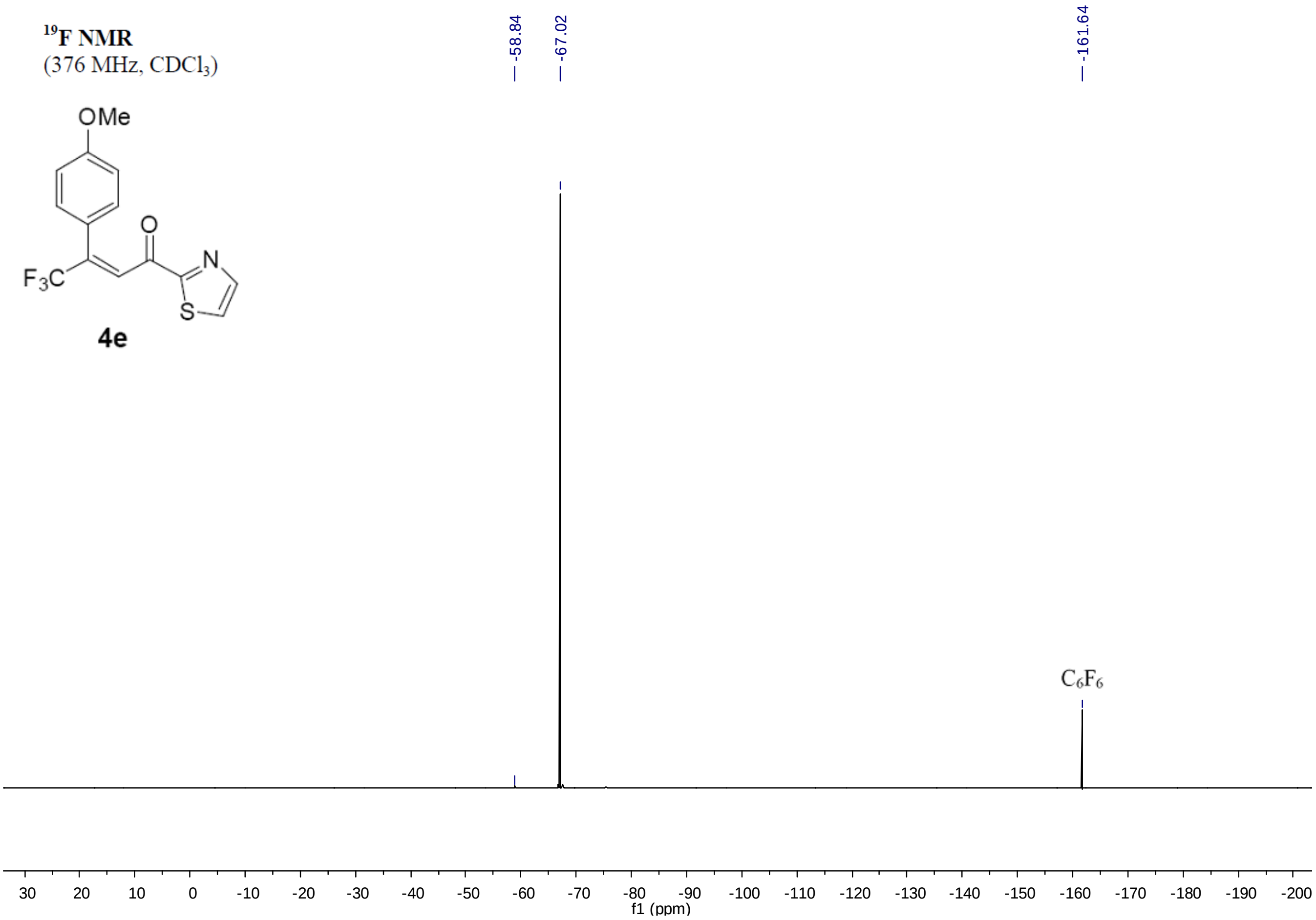
19F NMR
(376 MHz, CDCl3)
OMe
O
N
F3C
S
4e
-58.84
-67.02
-161.64
C6F6
30 20 10 0 -10 -20 -30 -40 -50 -60 -70 -80 -90 -100 -110 -120 -130 -140 -150 -160 -170 -180 -190 -200
f1 (ppm)

^{19}F NMR
(376 MHz, $CDCl_3$)

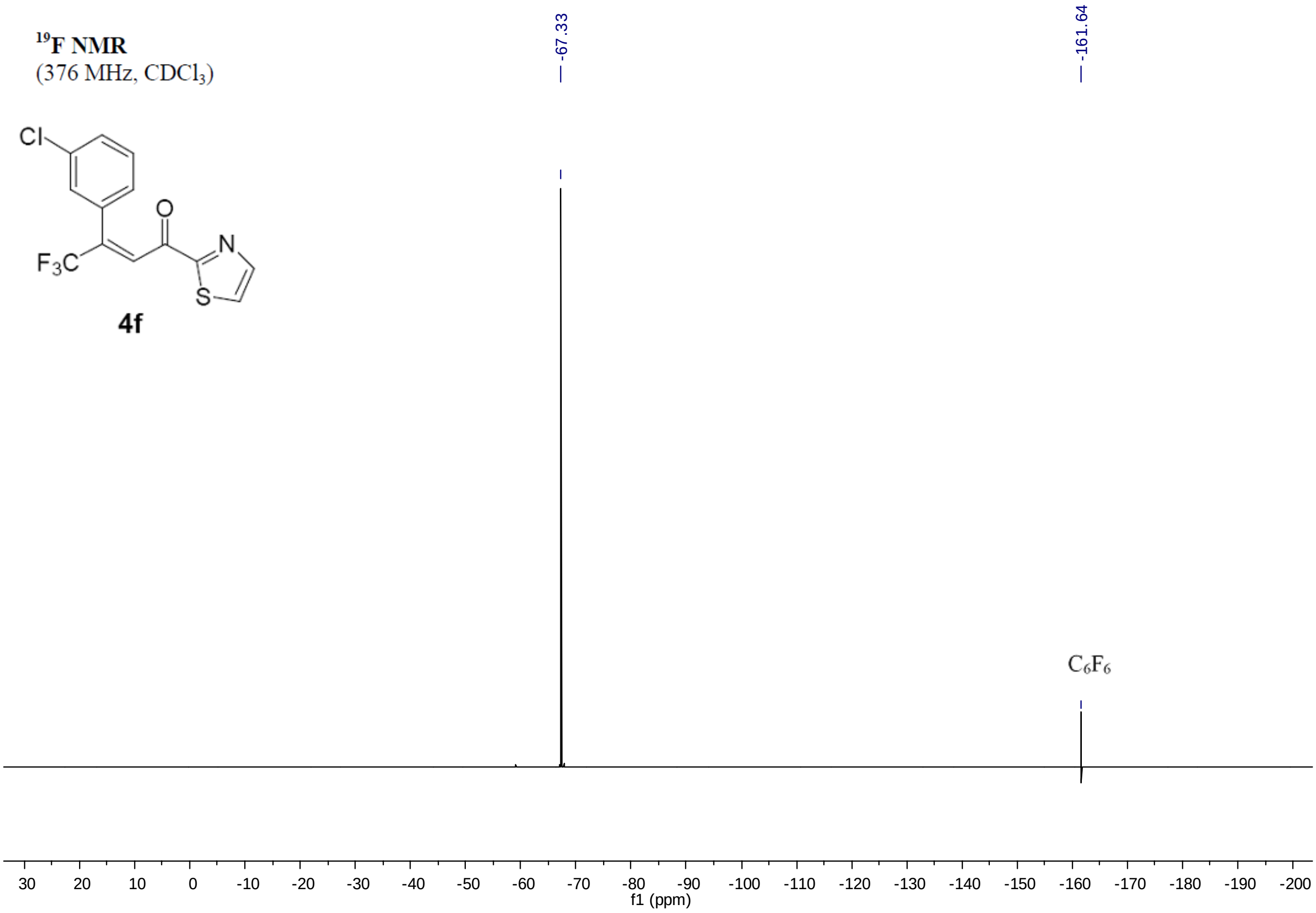

4f

^{19}F NMR
(376 MHz, $CDCl_3$)

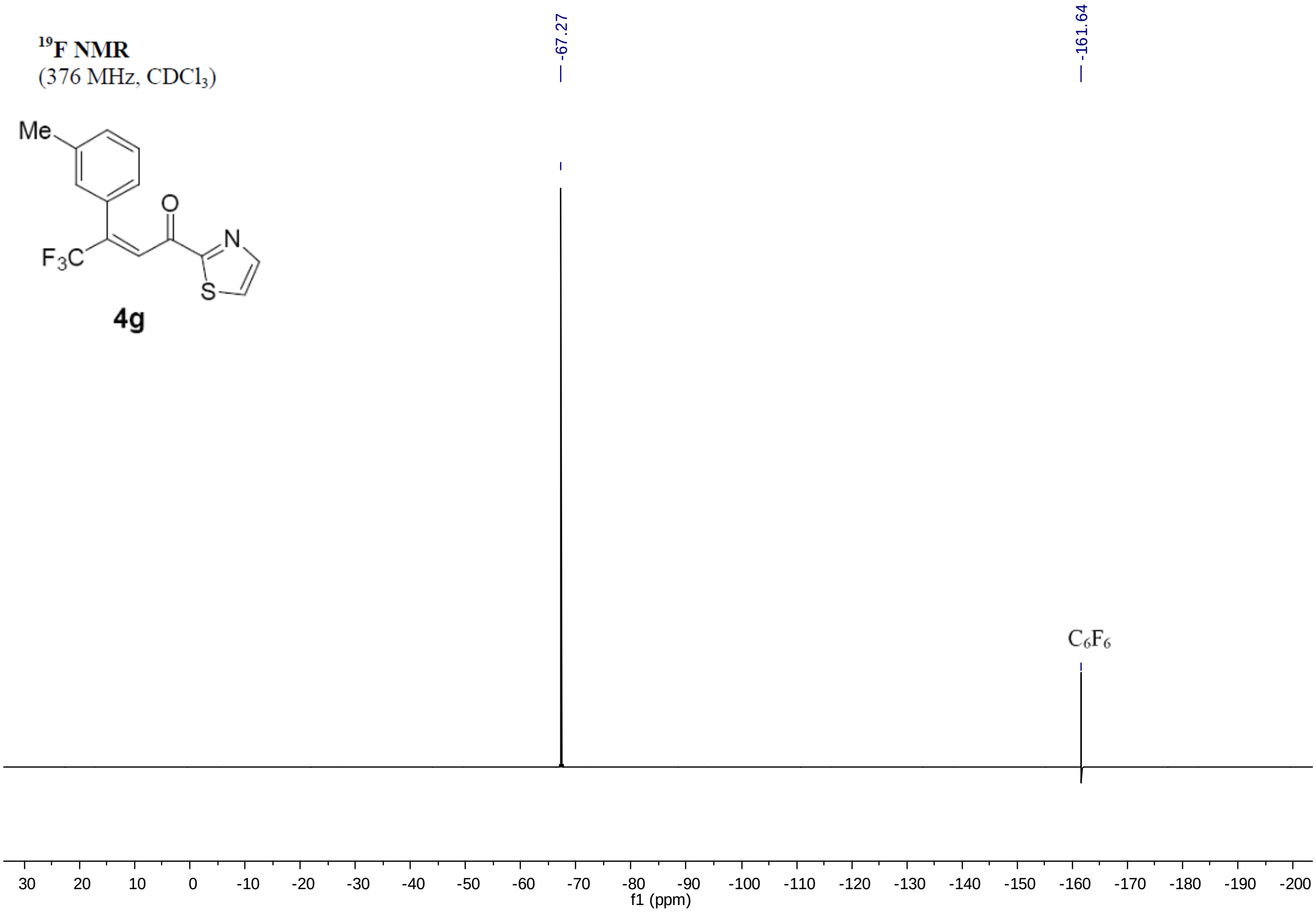

4g

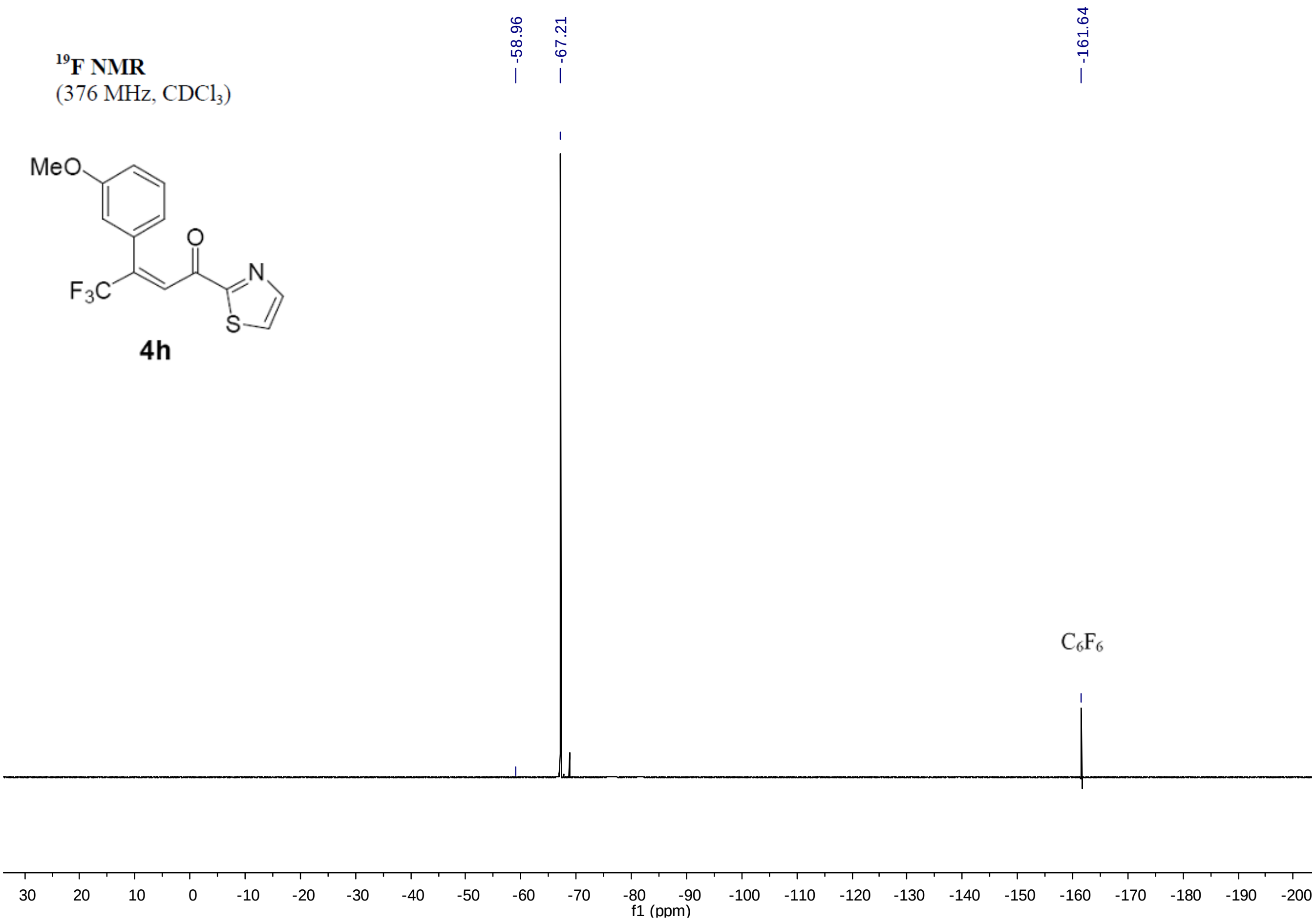
19F NMR
(376 MHz, CDCl3)
MeO
F3C
O
N
S
4h
-58.96
-67.21
-161.64
C6F6
30 20 10 0 -10 -20 -30 -40 -50 -60 -70 -80 -90 -100 -110 -120 -130 -140 -150 -160 -170 -180 -190 -200
f1 (ppm)

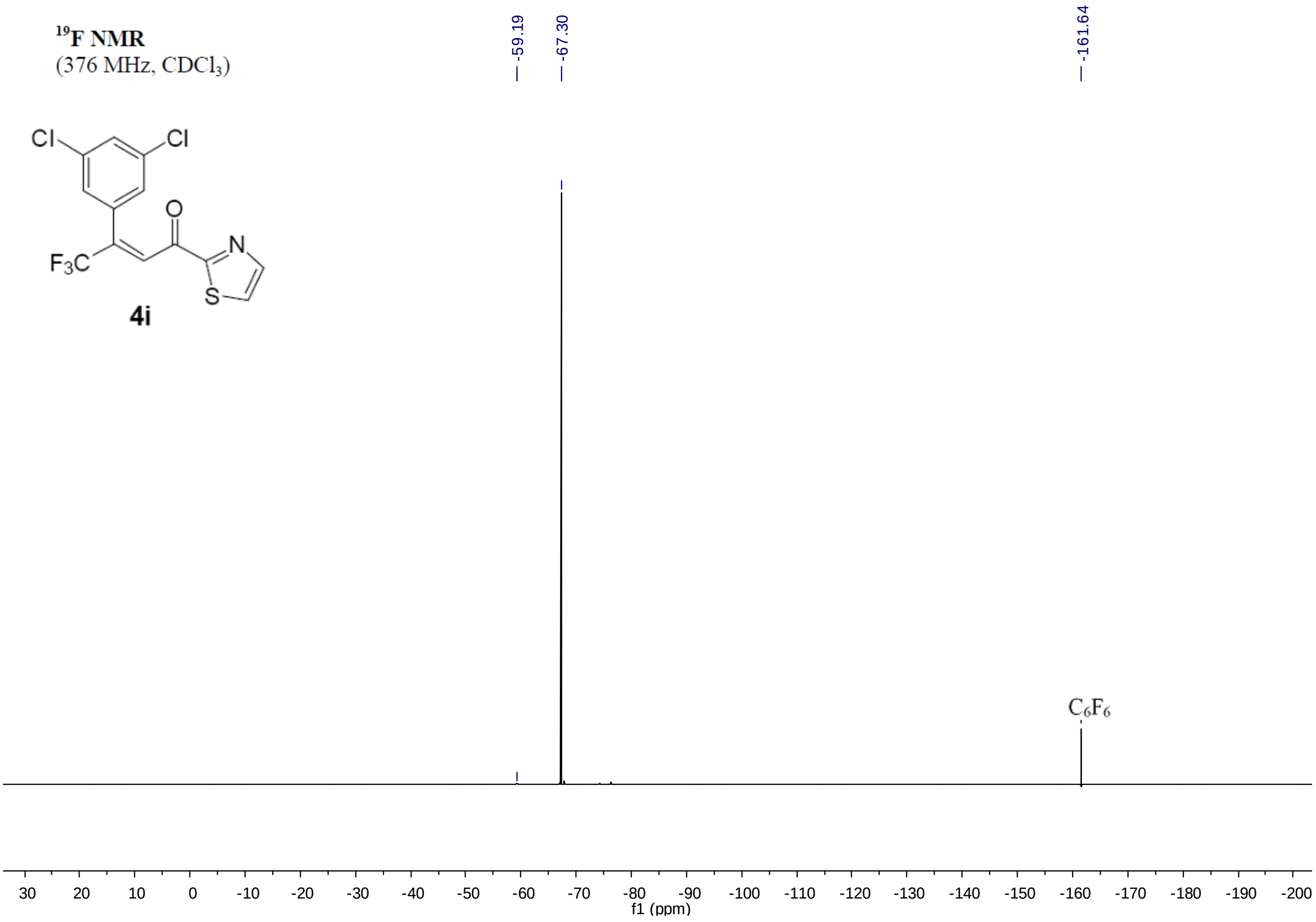
19F NMR
(376 MHz, CDCl3)
Cl
Cl
F3C
O
N
S
4i
-59.19
-67.30
-161.64
C6F6
30 20 10 0 -10 -20 -30 -40 -50 -60 -70 -80 -90 -100 -110 -120 -130 -140 -150 -160 -170 -180 -190 -200
f1 (ppm)

^{19}F NMR
(376 MHz, $CDCl_3$)

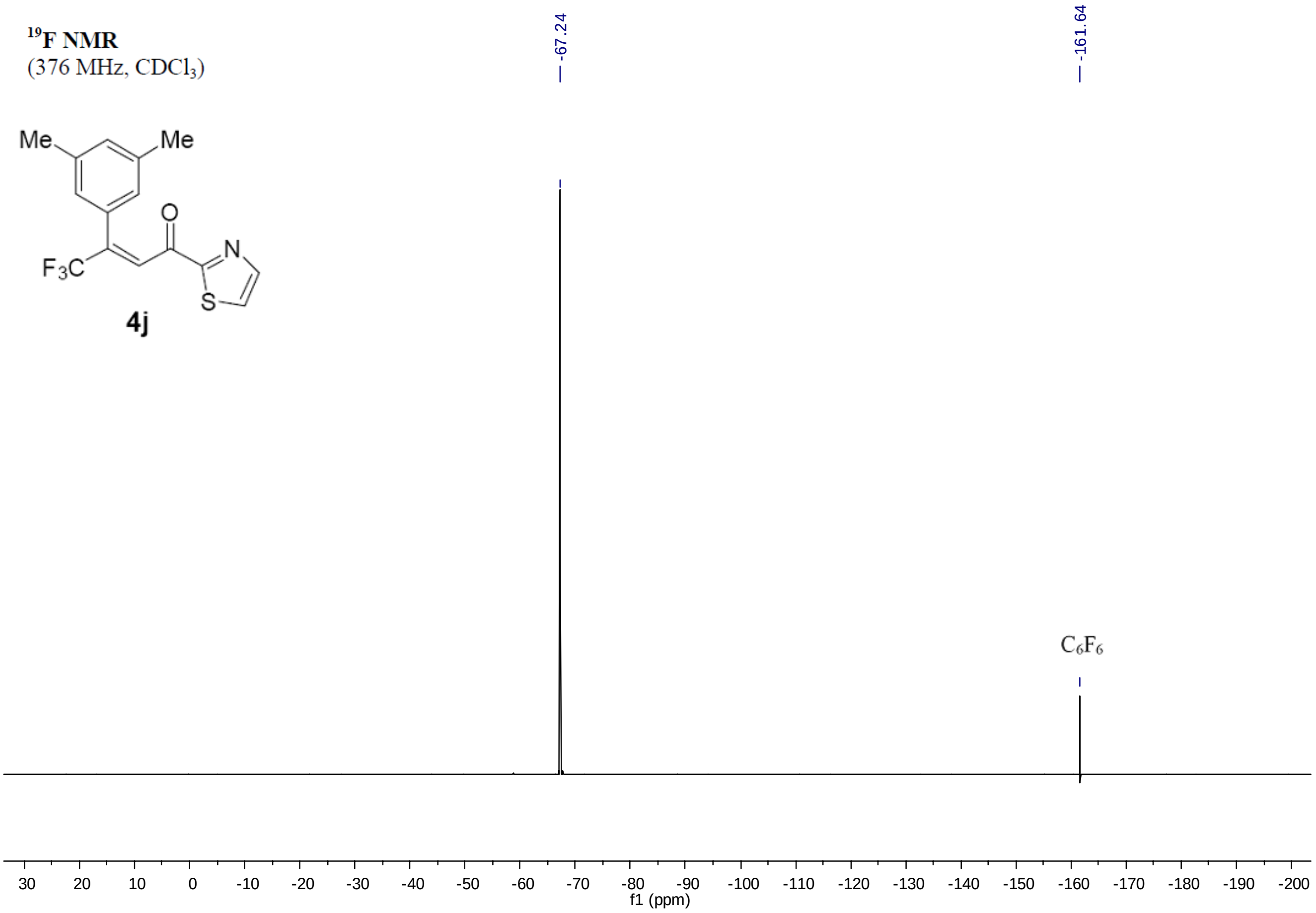

^{19}F NMR
(376 MHz, $CDCl_3$)

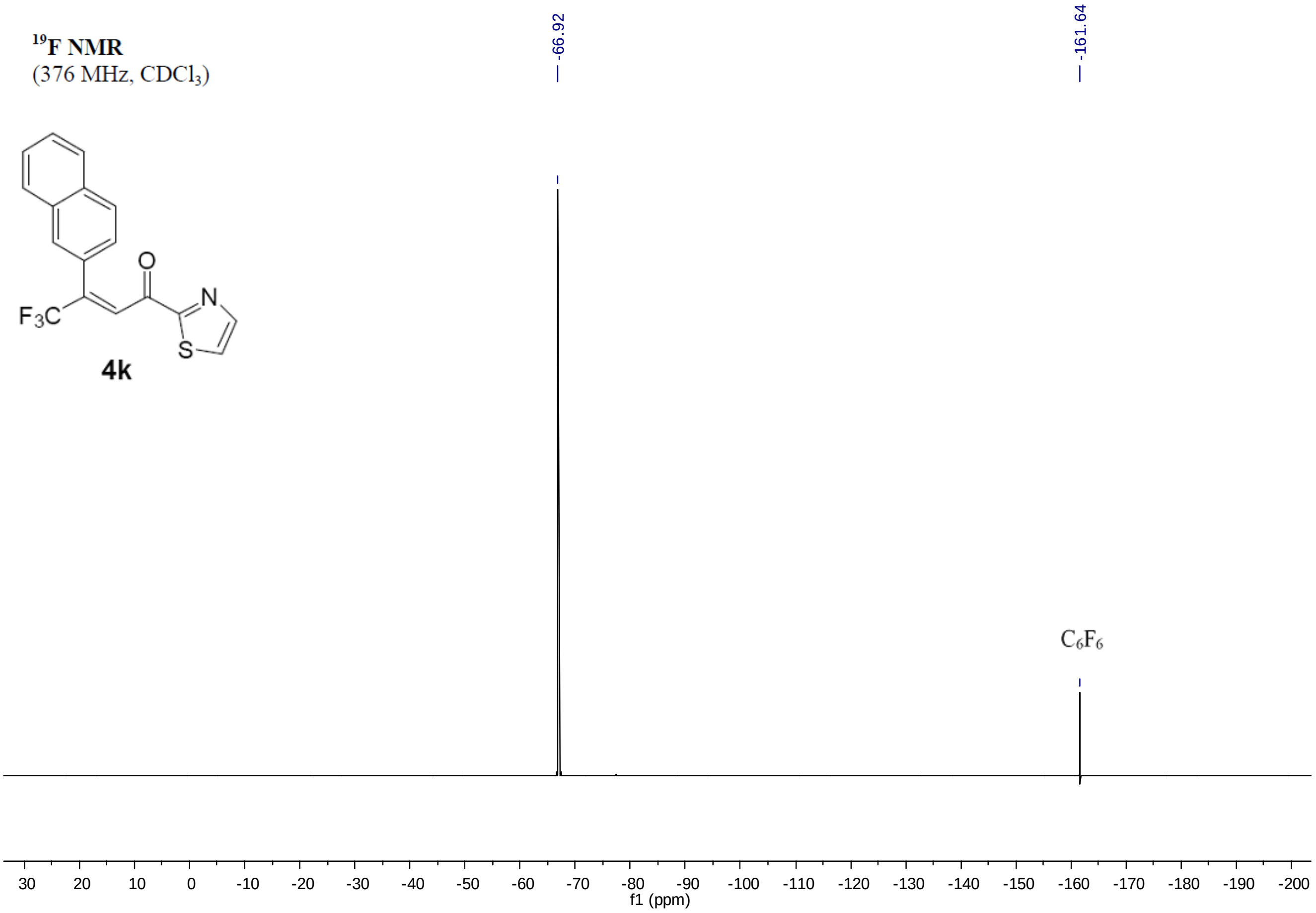

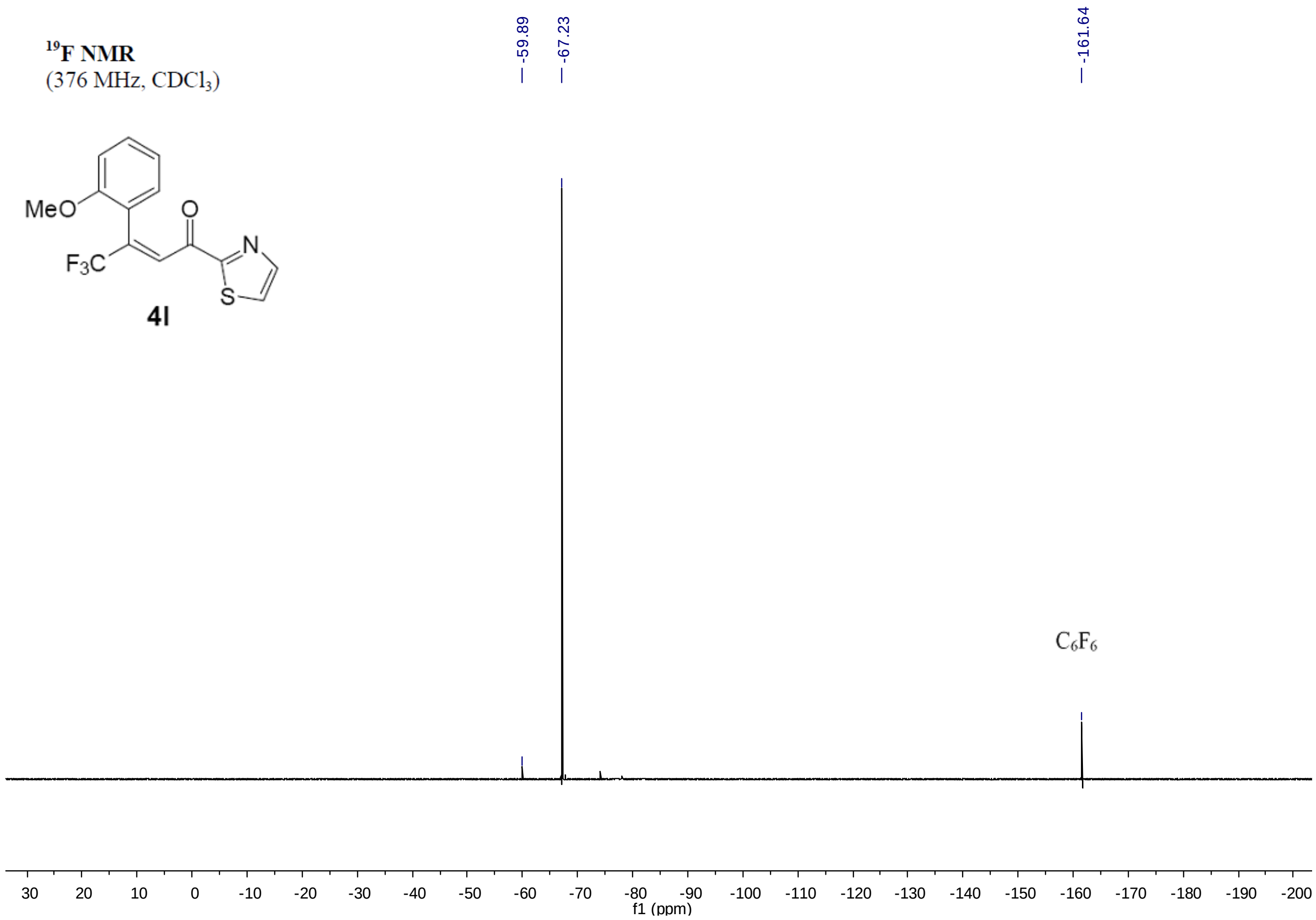
19F NMR
(376 MHz, CDCl3)
-59.89
-67.23
-161.64
MeO
F3C
O
N
S
4l
C6F6
30 20 10 0 -10 -20 -30 -40 -50 -60 -70 -80 -90 -100 -110 -120 -130 -140 -150 -160 -170 -180 -190 -200
f1 (ppm)

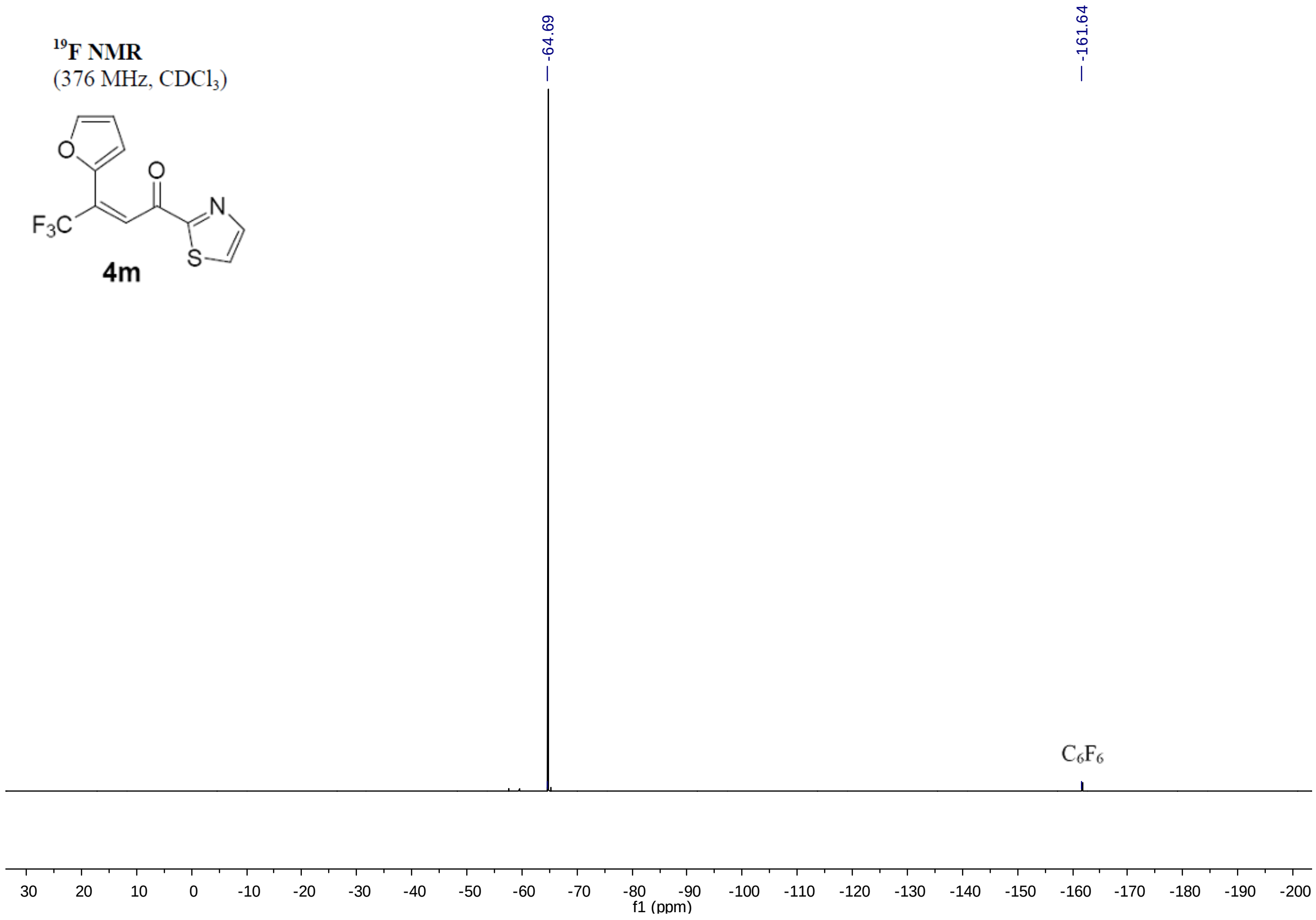
19F NMR
(376 MHz, CDCl3)
-64.69
-161.64
O
F3C
O
N
S
4m
C6F6
30 20 10 0 -10 -20 -30 -40 -50 -60 -70 -80 -90 -100 -110 -120 -130 -140 -150 -160 -170 -180 -190 -200
f1 (ppm)

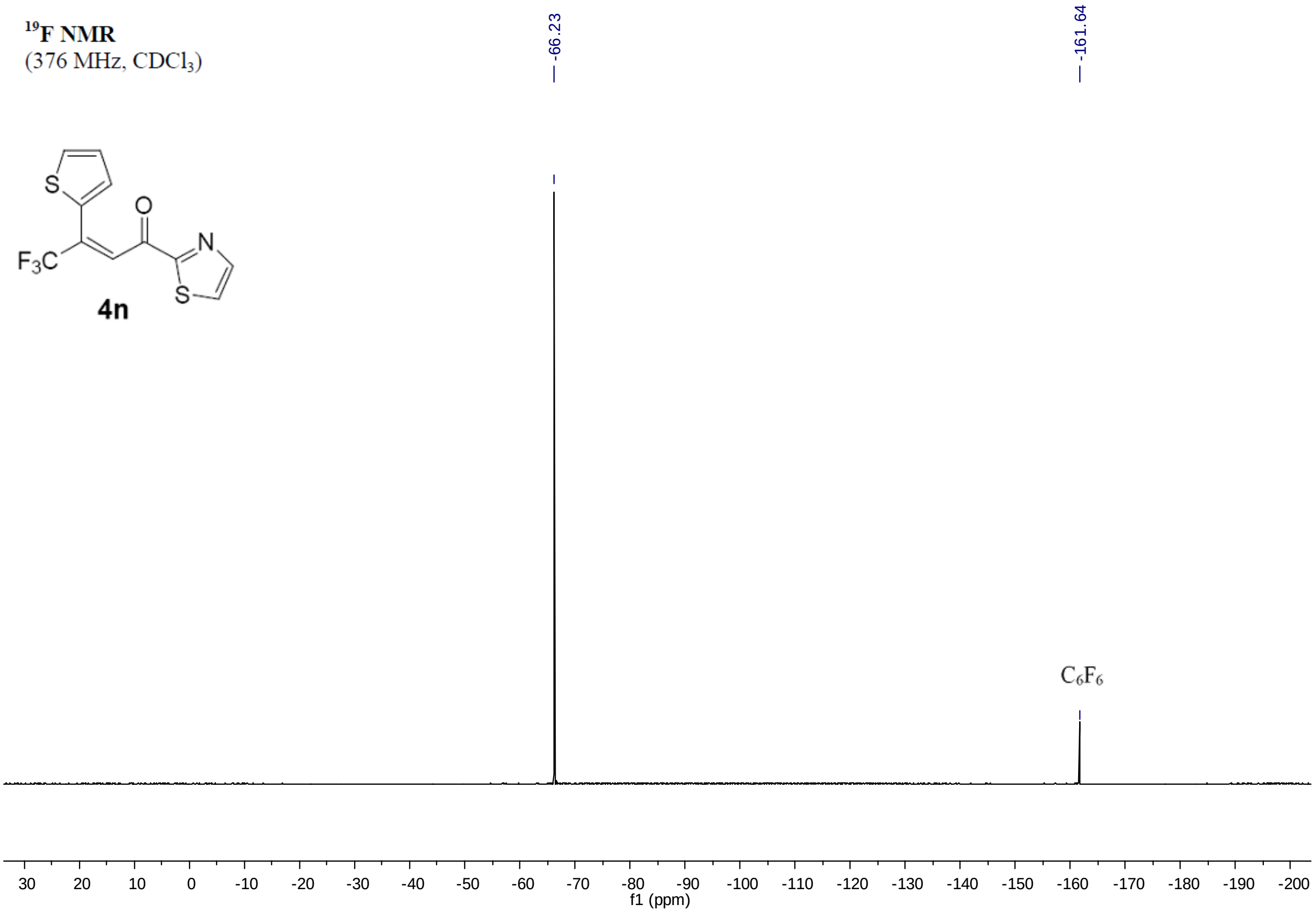

19F NMR
(376 MHz, CDCl3)
-66.23
-161.64
F3C
O
N
S
S
4n
C6F6
30 20 10 0 -10 -20 -30 -40 -50 -60 -70 -80 -90 -100 -110 -120 -130 -140 -150 -160 -170 -180 -190 -200
f1 (ppm)

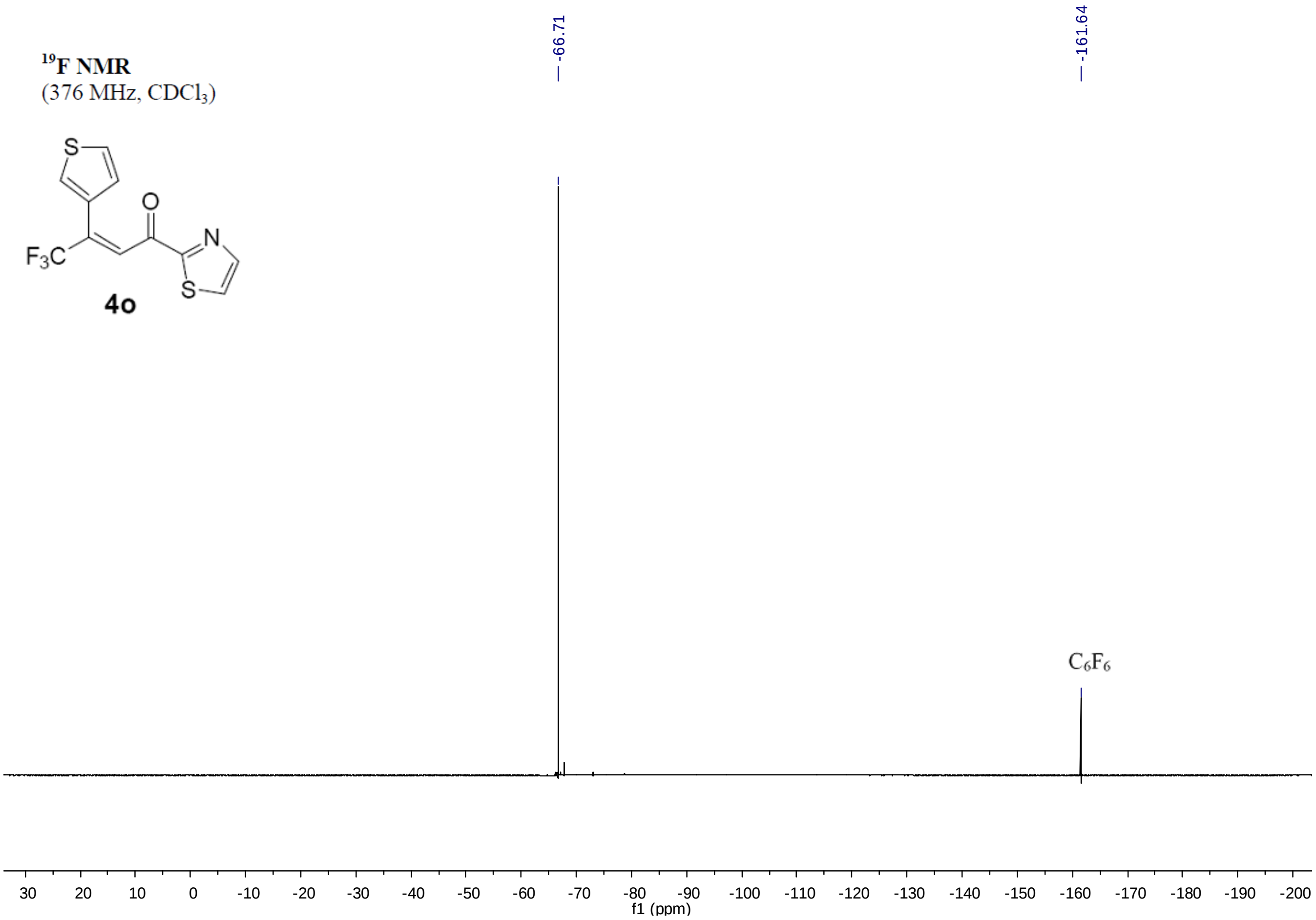

19F NMR
(376 MHz, CDCl3)
-66.71
-161.64
F3C
O
N
S
S
4o
C6F6
30 20 10 0 -10 -20 -30 -40 -50 -60 -70 -80 -90 -100 -110 -120 -130 -140 -150 -160 -170 -180 -190 -200
f1 (ppm)

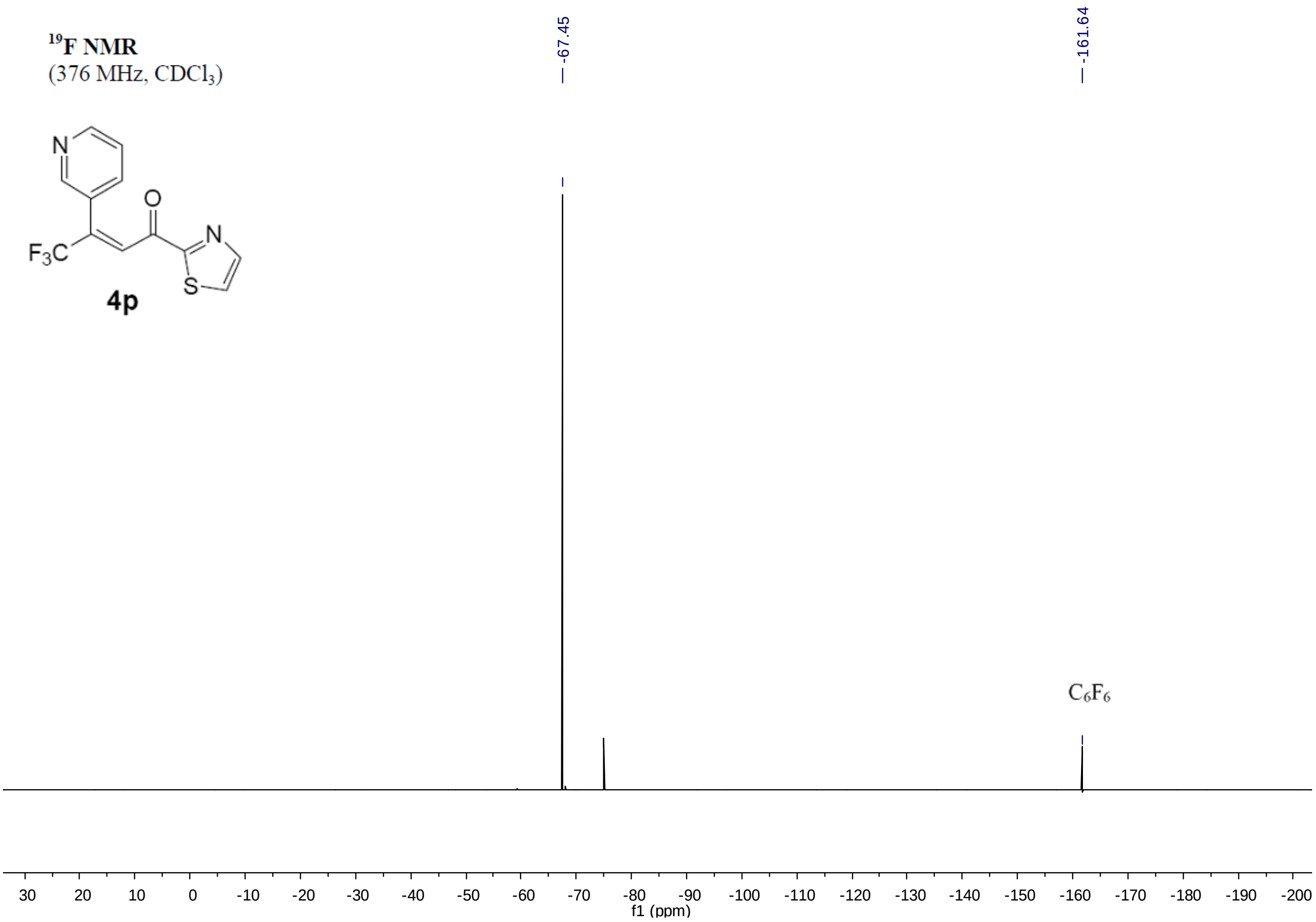
19F NMR
(376 MHz, CDCl3)
4p
-67.45
-161.64
C6F6
f1 (ppm)

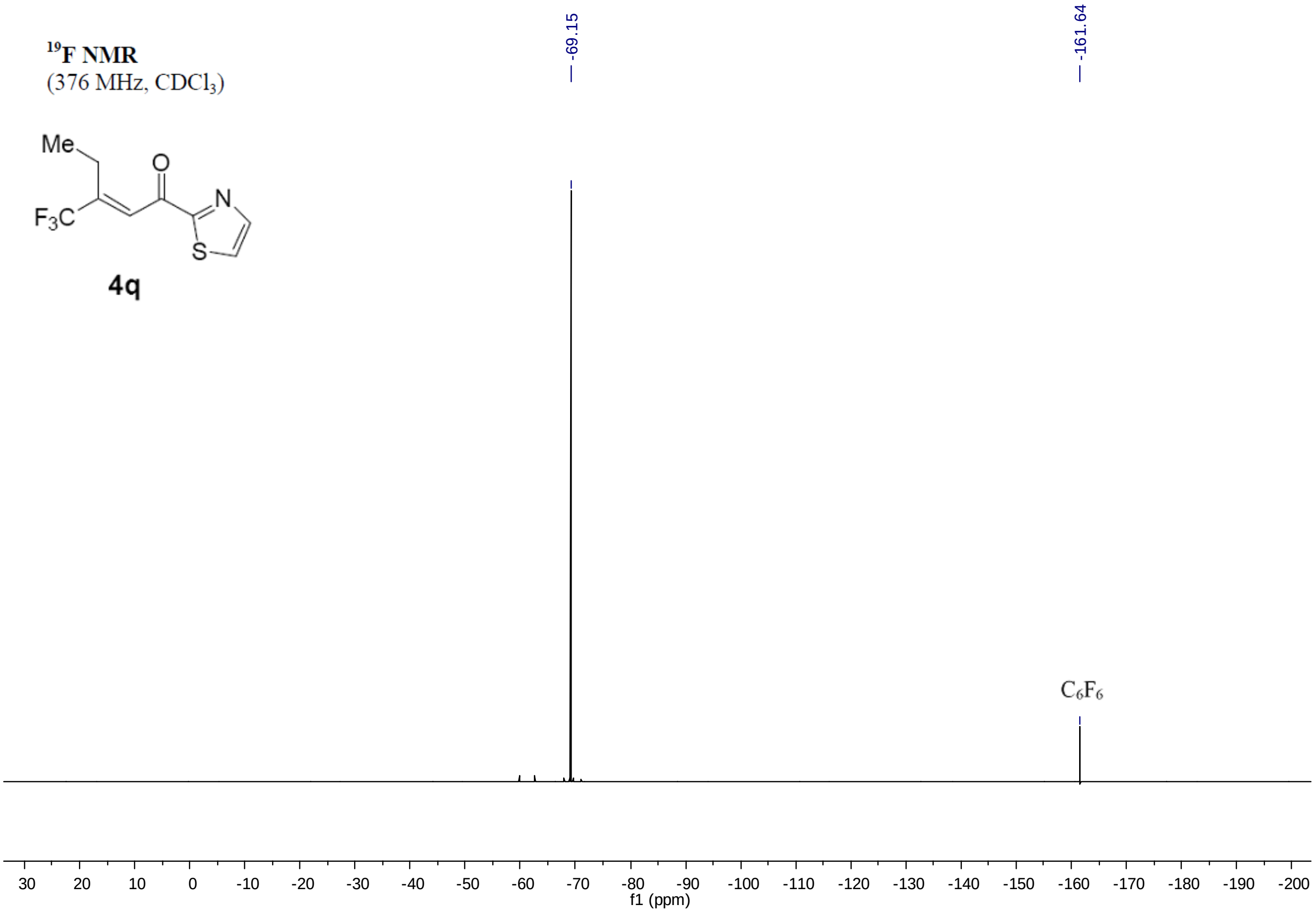
19F NMR
(376 MHz, CDCl3)
Me
4q
-69.15
-161.64
C6F6
f1 (ppm)

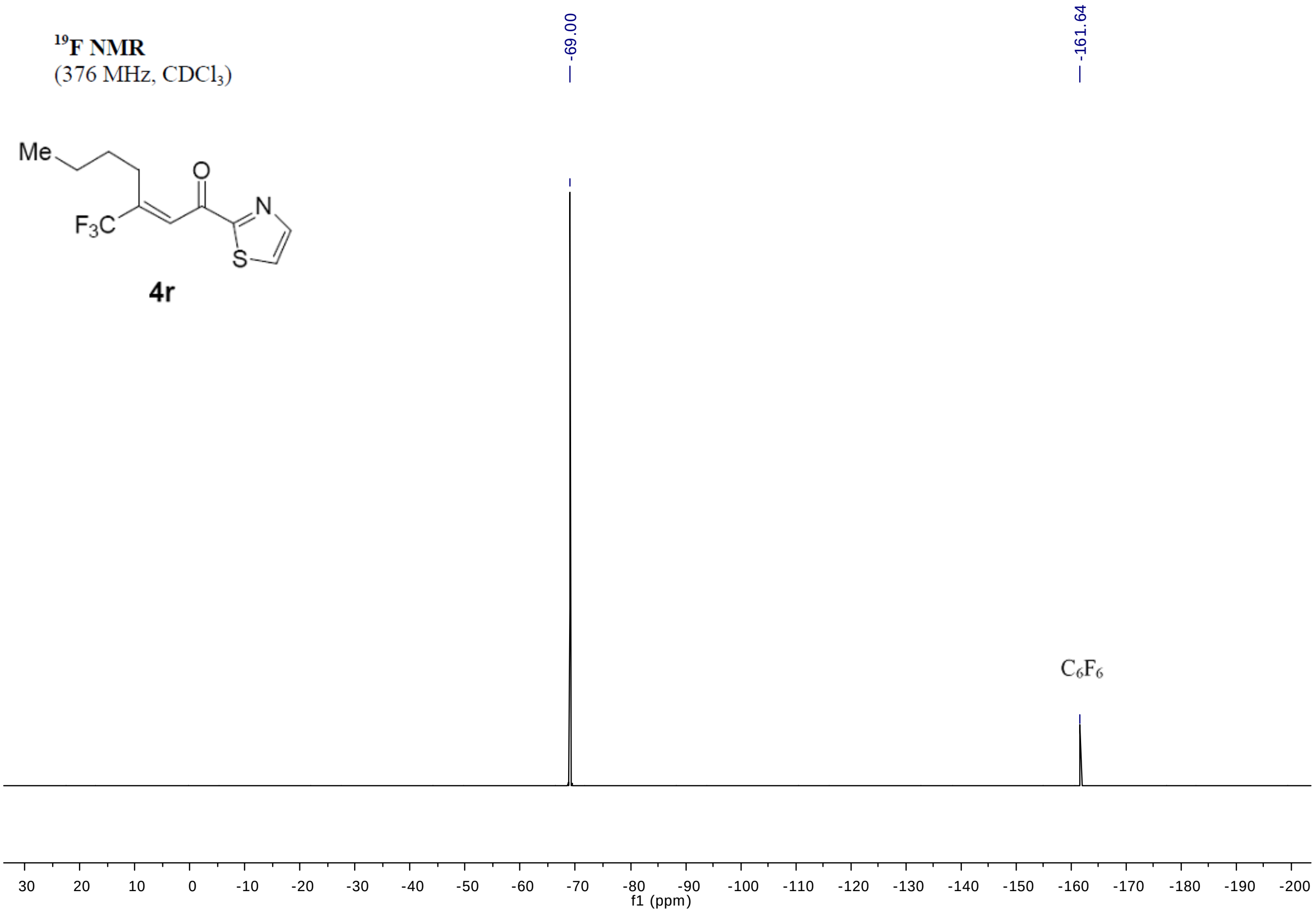
19F NMR
(376 MHz, CDCl3)
-69.00
-161.64
Me
O
N
F3C
S
4r
C6F6
30 20 10 0 -10 -20 -30 -40 -50 -60 -70 -80 -90 -100 -110 -120 -130 -140 -150 -160 -170 -180 -190 -200
f1 (ppm)

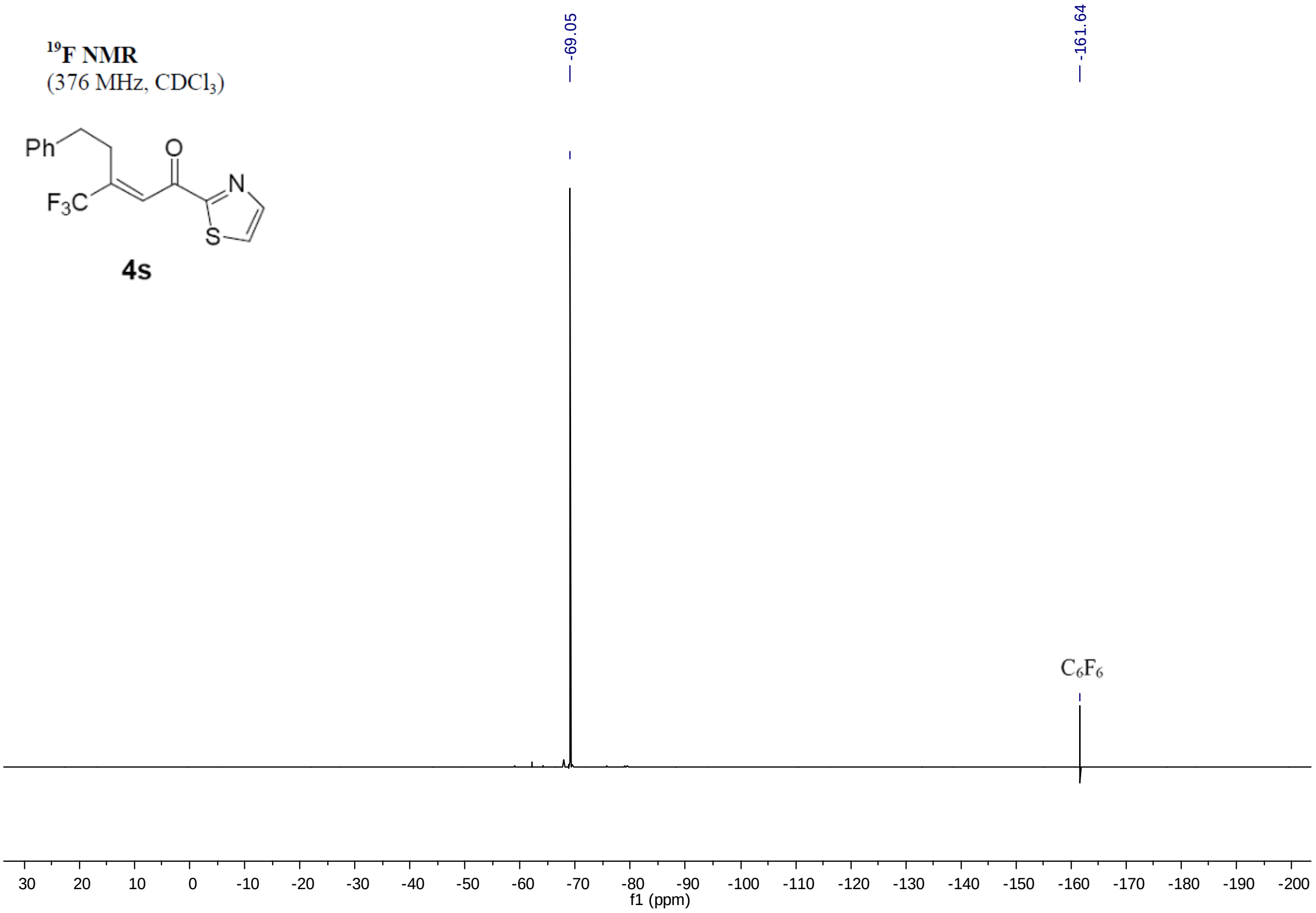
19F NMR
(376 MHz, CDCl3)
-69.05
-161.64
Ph
O
N
F3C
S
4s
C6F6
30 20 10 0 -10 -20 -30 -40 -50 -60 -70 -80 -90 -100 -110 -120 -130 -140 -150 -160 -170 -180 -190 -200
f1 (ppm)

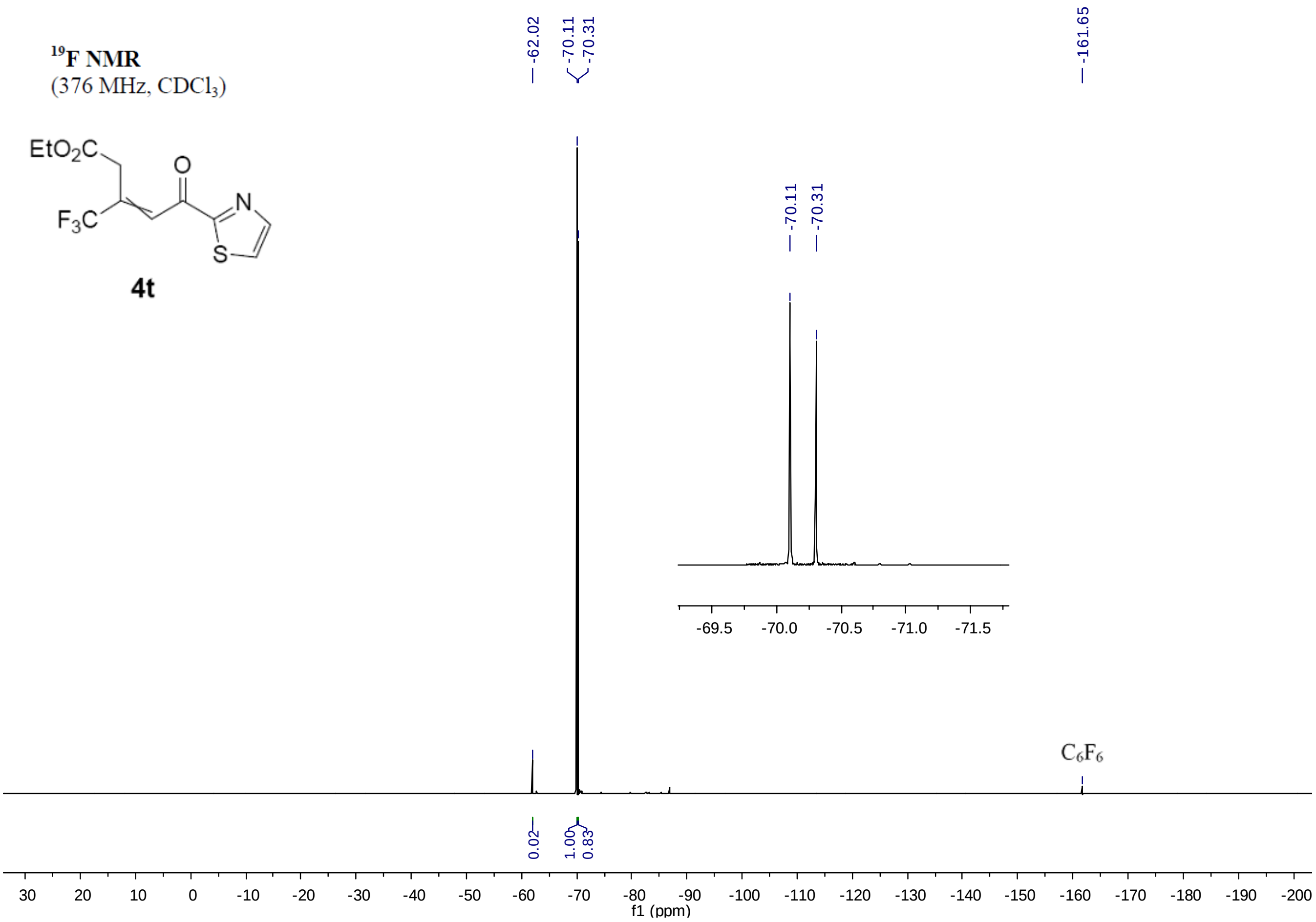
19F NMR
(376 MHz, CDCl3)
EtO2C
O
N
F3C
S
4t
-62.02
-70.11
-70.31
-161.65
-70.11
-70.31
-69.5
-70.0
-70.5
-71.0
-71.5
C6F6
0.02
1.00
0.83
30 20 10 0 -10 -20 -30 -40 -50 -60 -70 -80 -90 -100 -110 -120 -130 -140 -150 -160 -170 -180 -190 -200
f1 (ppm)

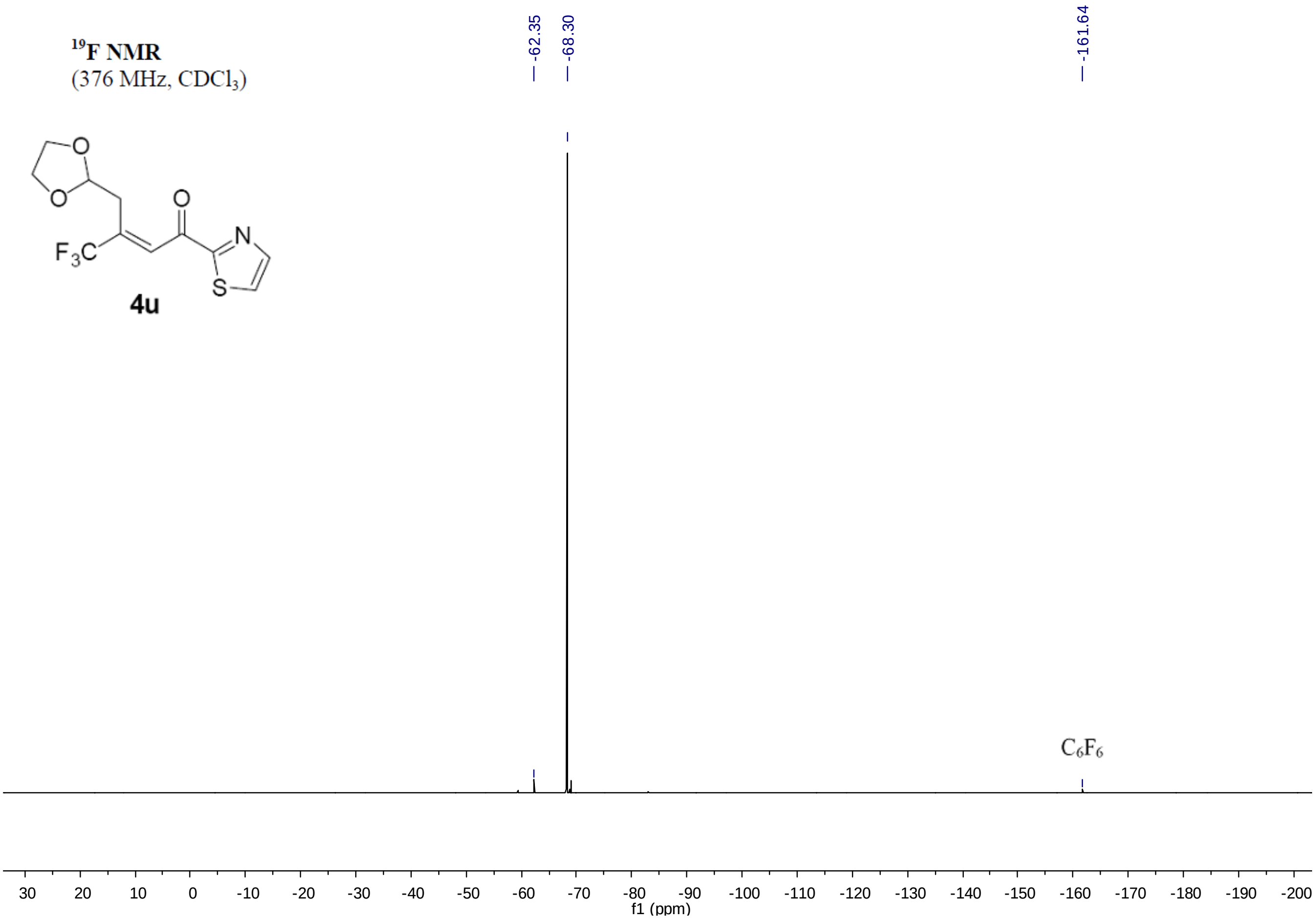
19F NMR
(376 MHz, CDCl3)
O
O
O
N
F3C
S
4u
-62.35
-68.30
-161.64
C6F6
30 20 10 0 -10 -20 -30 -40 -50 -60 -70 -80 -90 -100 -110 -120 -130 -140 -150 -160 -170 -180 -190 -200
f1 (ppm)

^{19}F NMR
(376 MHz, $CDCl_3$)

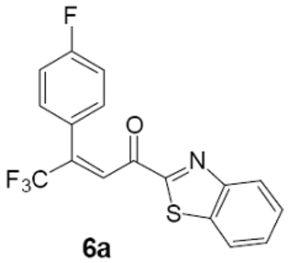

6a

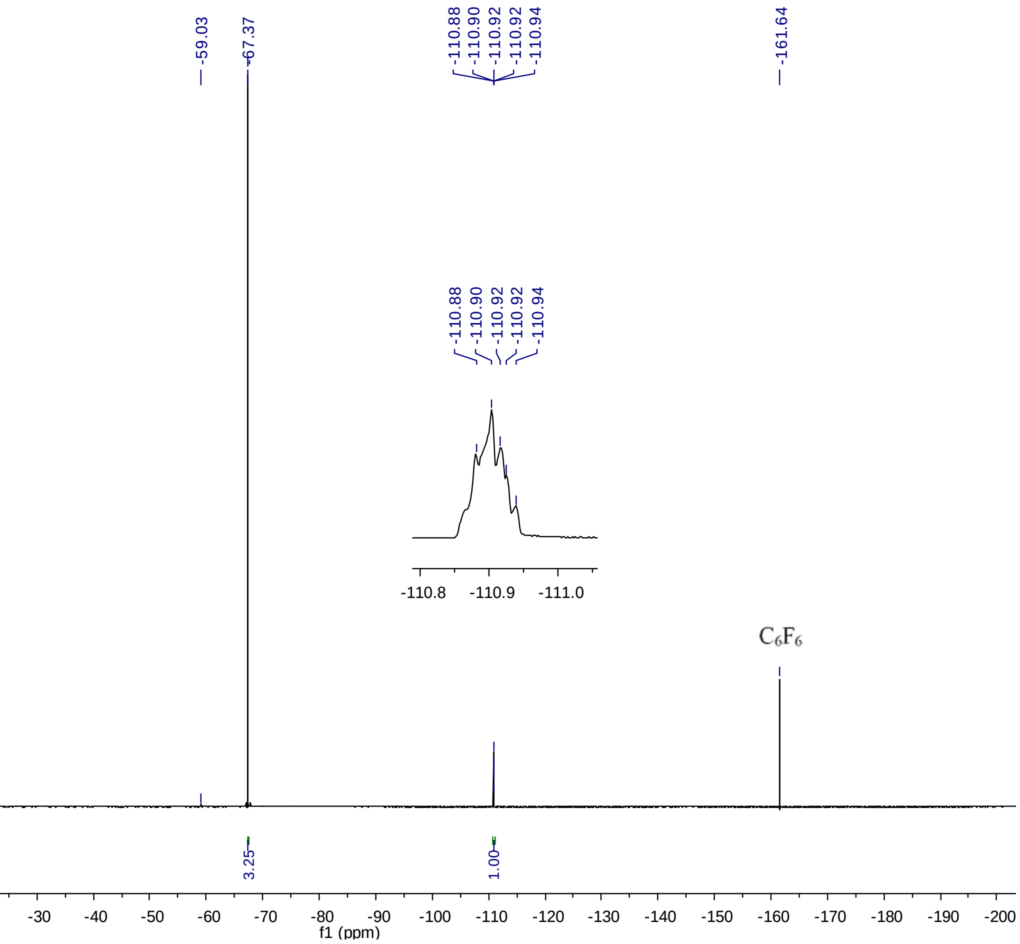

^{19}F NMR
(376 MHz, $CDCl_3$)

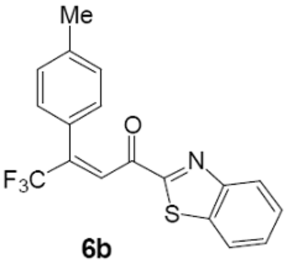

6b

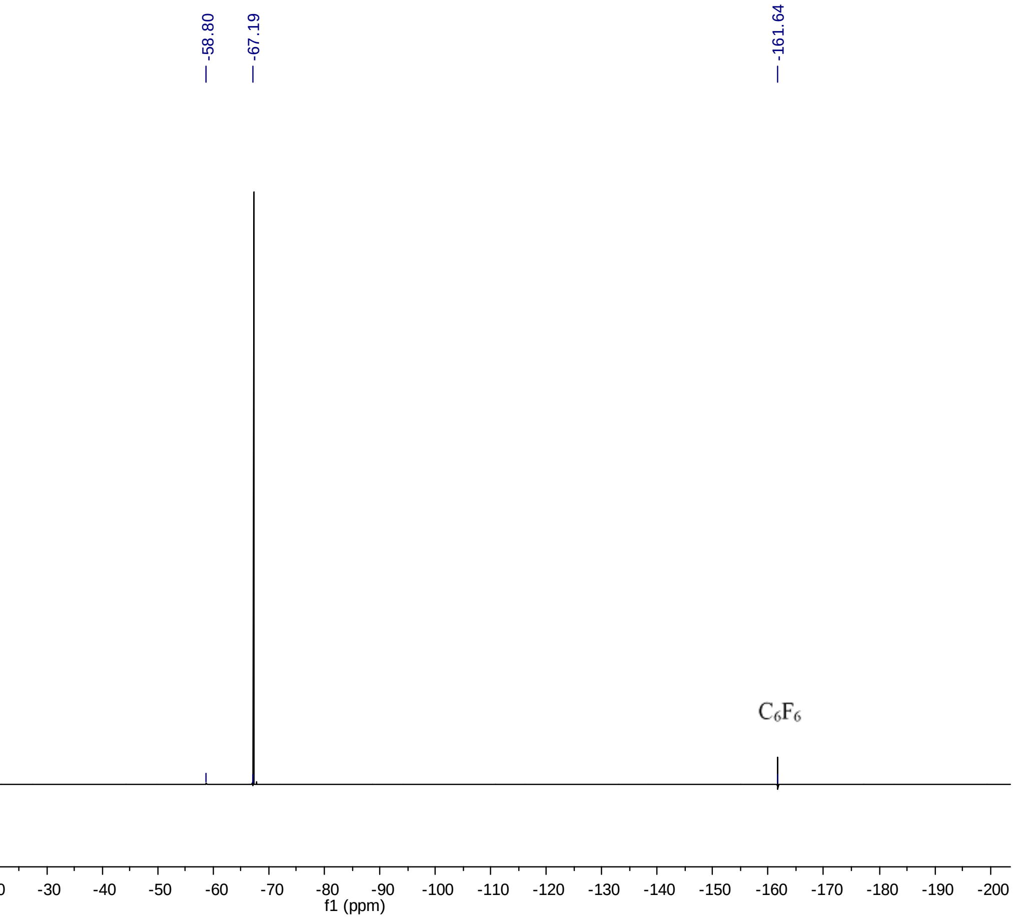

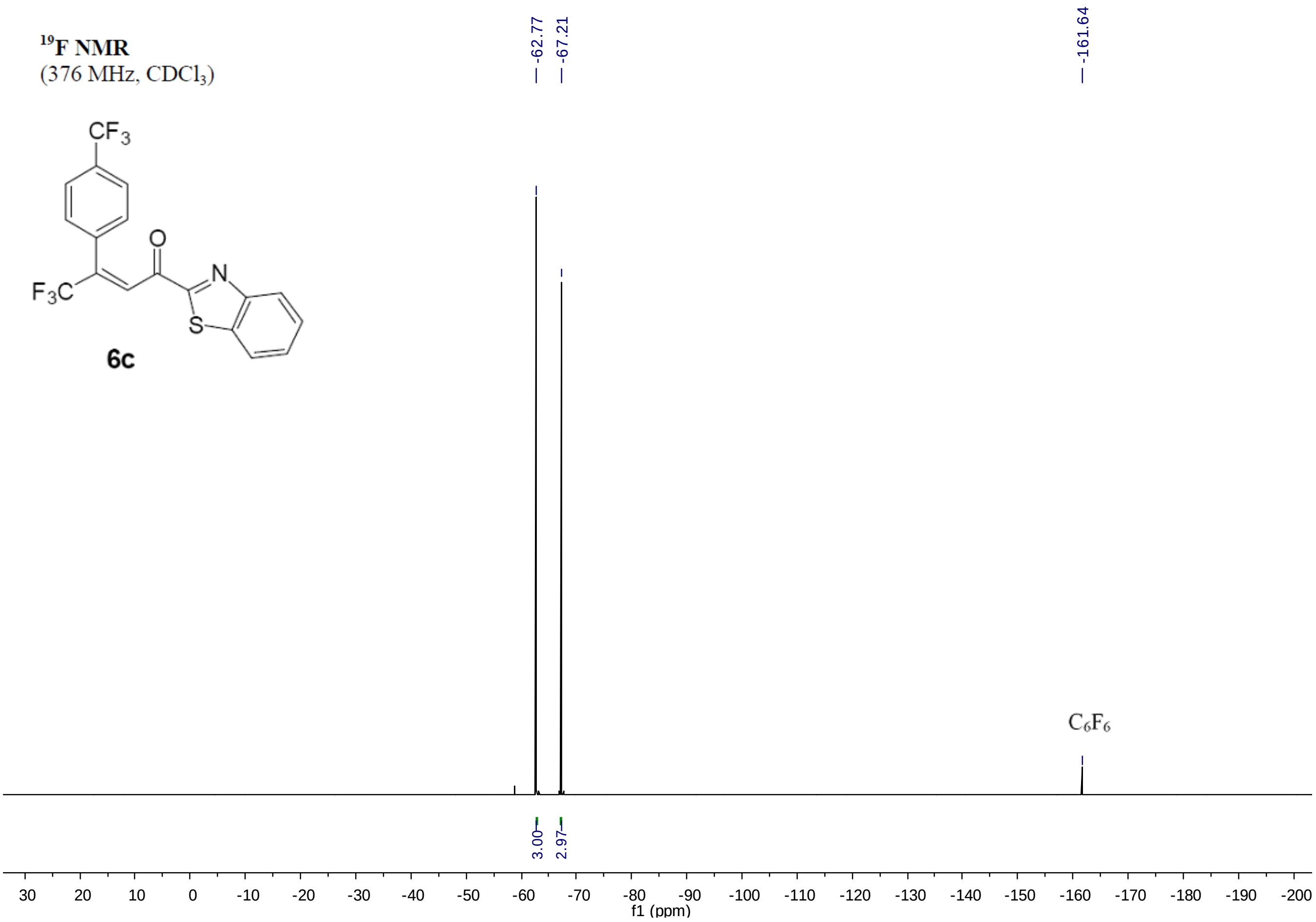
19F NMR
(376 MHz, CDCl3)
6c
-62.77
-67.21
-161.64
C6F6
3.00
2.97
f1 (ppm)

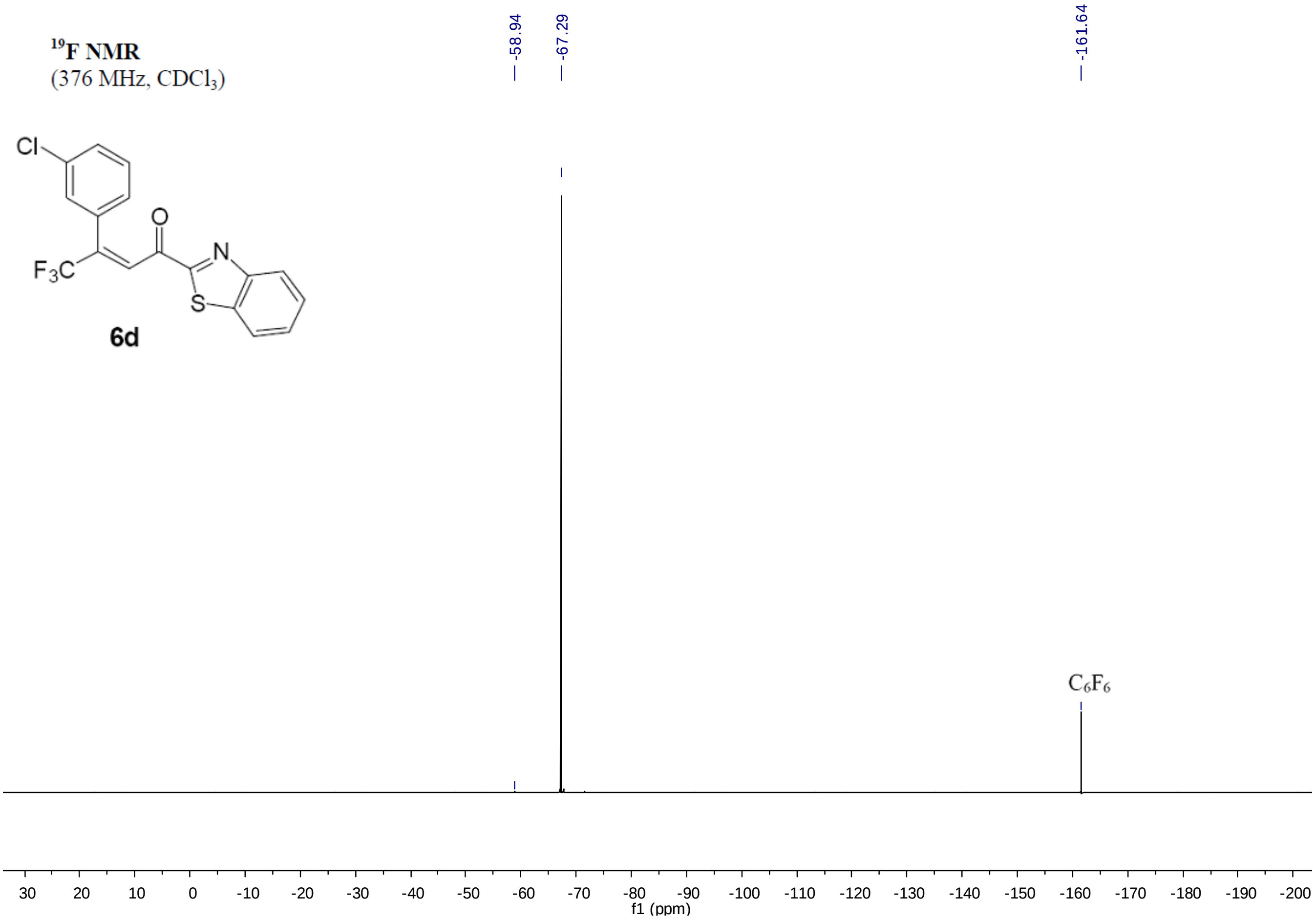
19F NMR
(376 MHz, CDCl3)
6d
-58.94
-67.29
-161.64
C6F6
f1 (ppm)

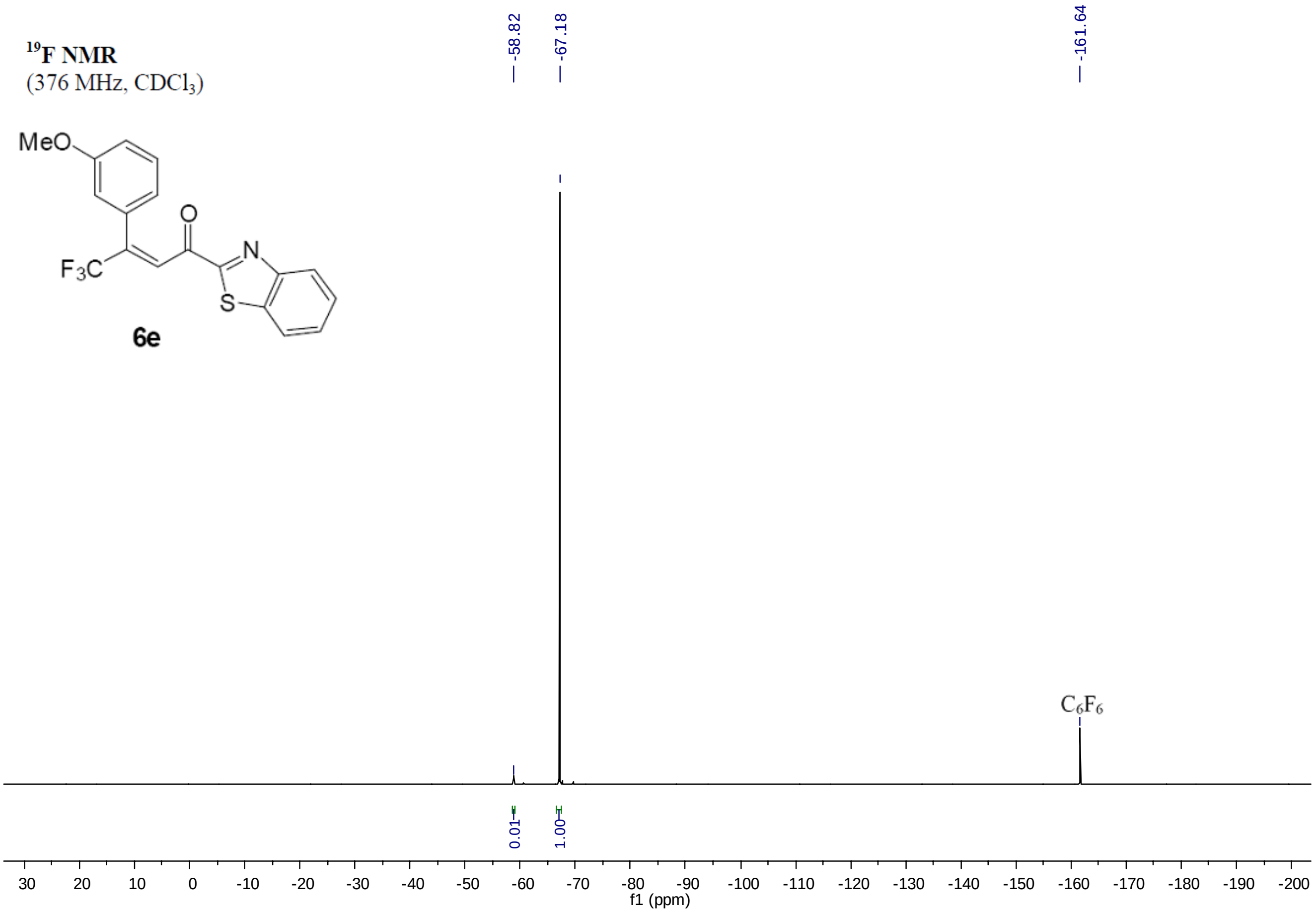

19F NMR
(376 MHz, CDCl3)
MeO
F3C
O
N
S
6e
-58.82
-67.18
-161.64
C6F6
0.01
1.00
30 20 10 0 -10 -20 -30 -40 -50 -60 -70 -80 -90 -100 -110 -120 -130 -140 -150 -160 -170 -180 -190 -200
f1 (ppm)

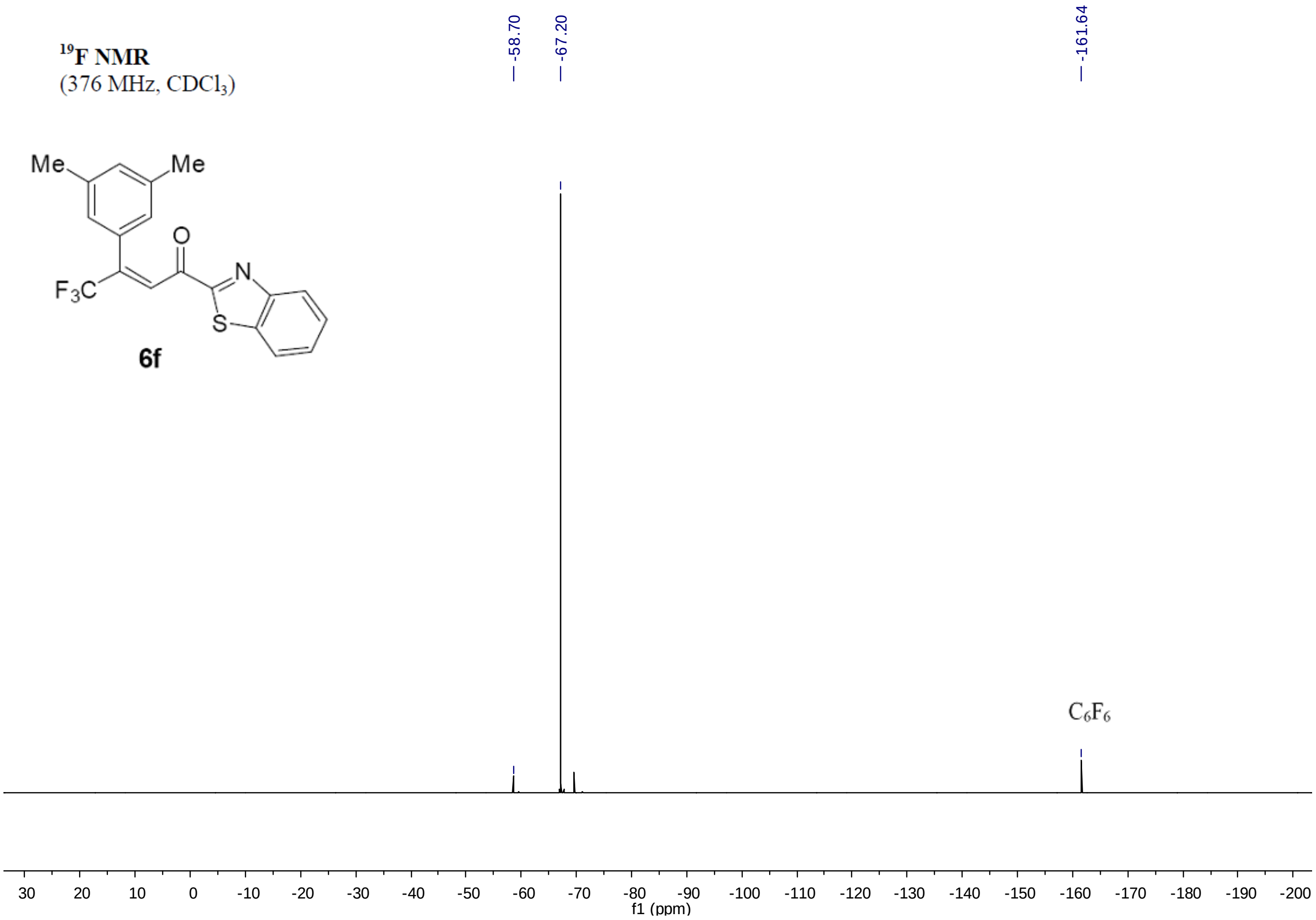

19F NMR
(376 MHz, CDCl3)
Me
Me
F3C
O
N
S
6f
-58.70
-67.20
-161.64
C6F6
30 20 10 0 -10 -20 -30 -40 -50 -60 -70 -80 -90 -100 -110 -120 -130 -140 -150 -160 -170 -180 -190 -200
f1 (ppm)

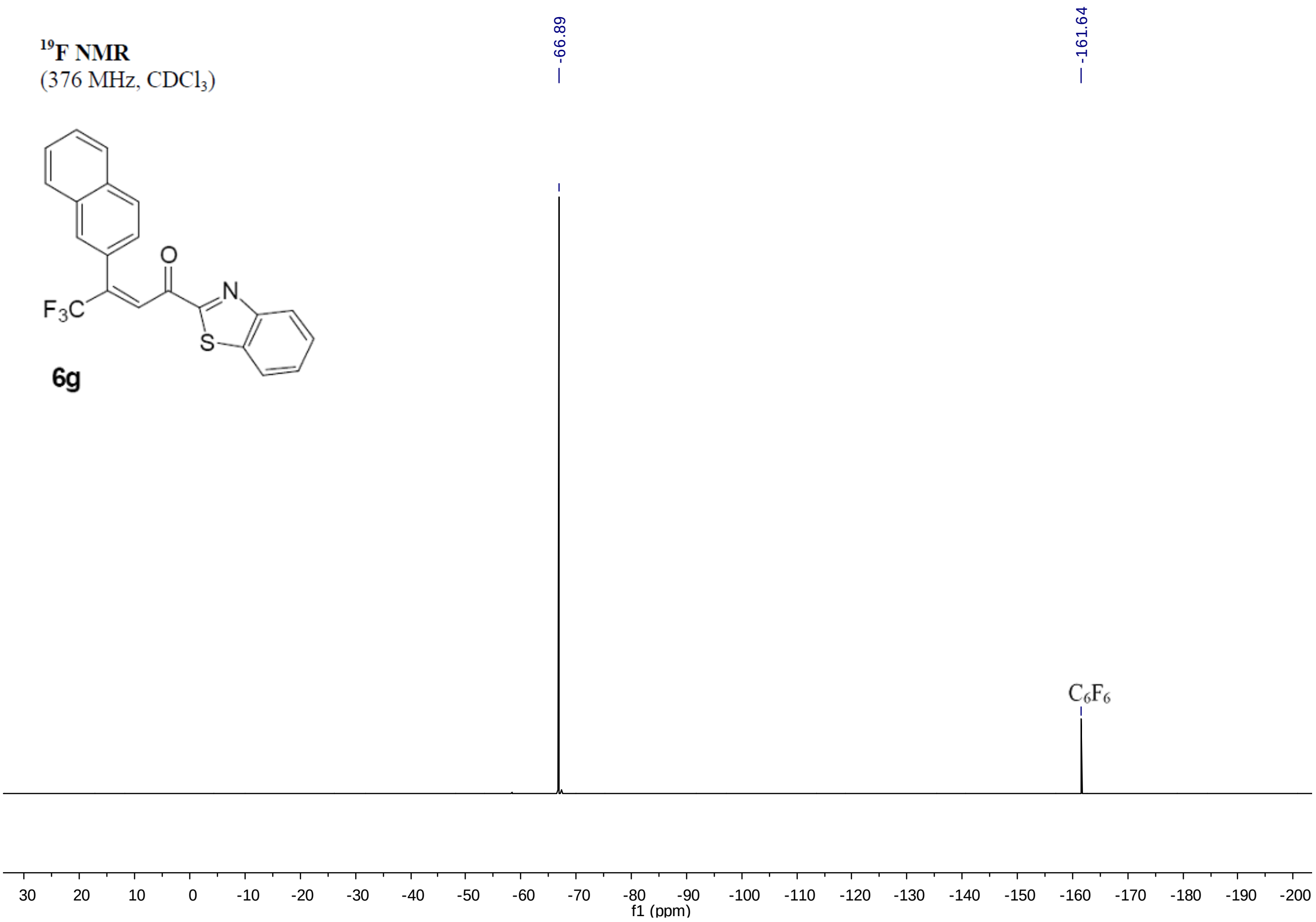
19F NMR
(376 MHz, CDCl3)
6g
-66.89
-161.64
C6F6
30 20 10 0 -10 -20 -30 -40 -50 -60 -70 -80 -90 -100 -110 -120 -130 -140 -150 -160 -170 -180 -190 -200
f1 (ppm)

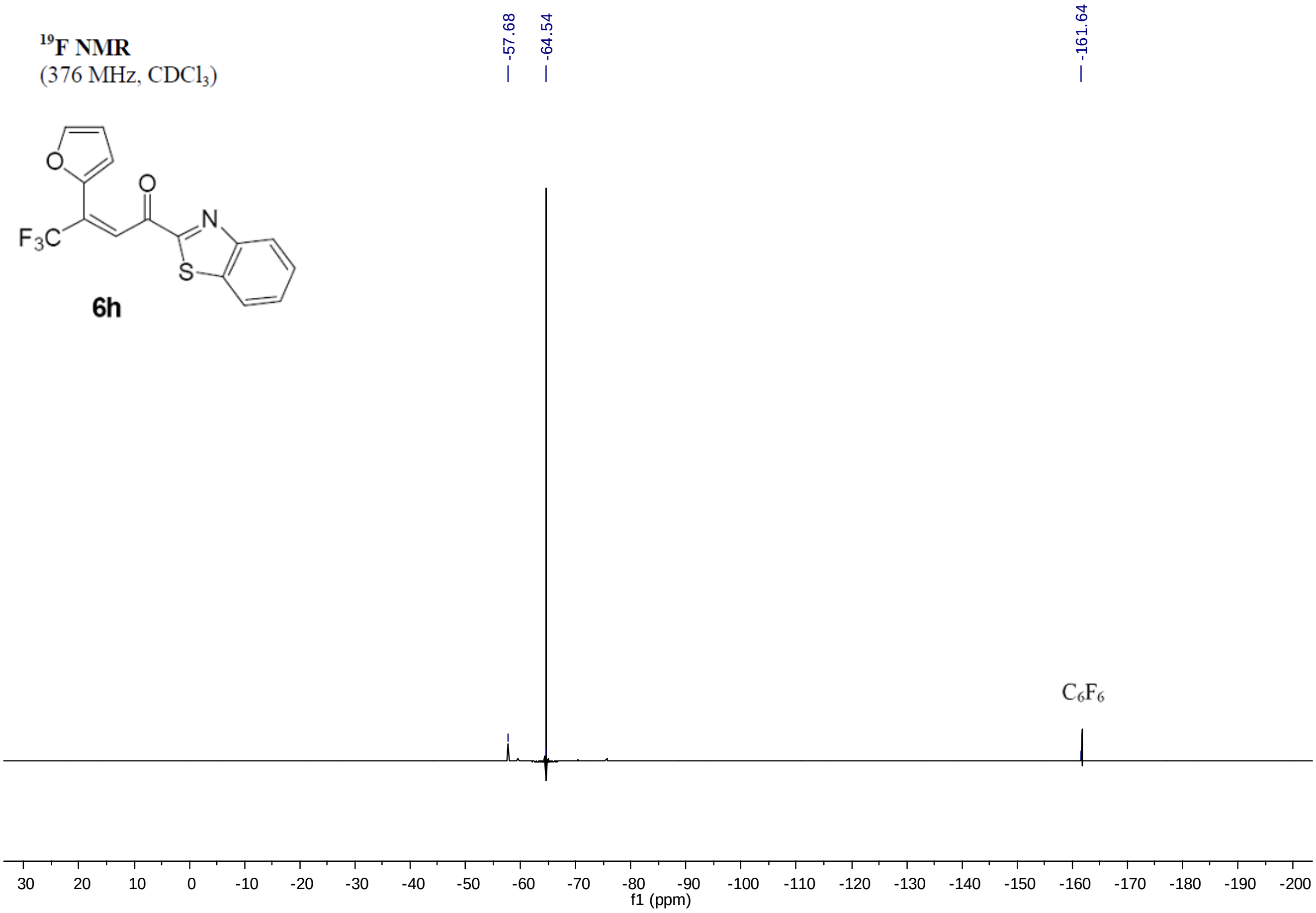
19F NMR
(376 MHz, CDCl3)
6h
-57.68
-64.54
-161.64
C6F6
30 20 10 0 -10 -20 -30 -40 -50 -60 -70 -80 -90 -100 -110 -120 -130 -140 -150 -160 -170 -180 -190 -200
f1 (ppm)

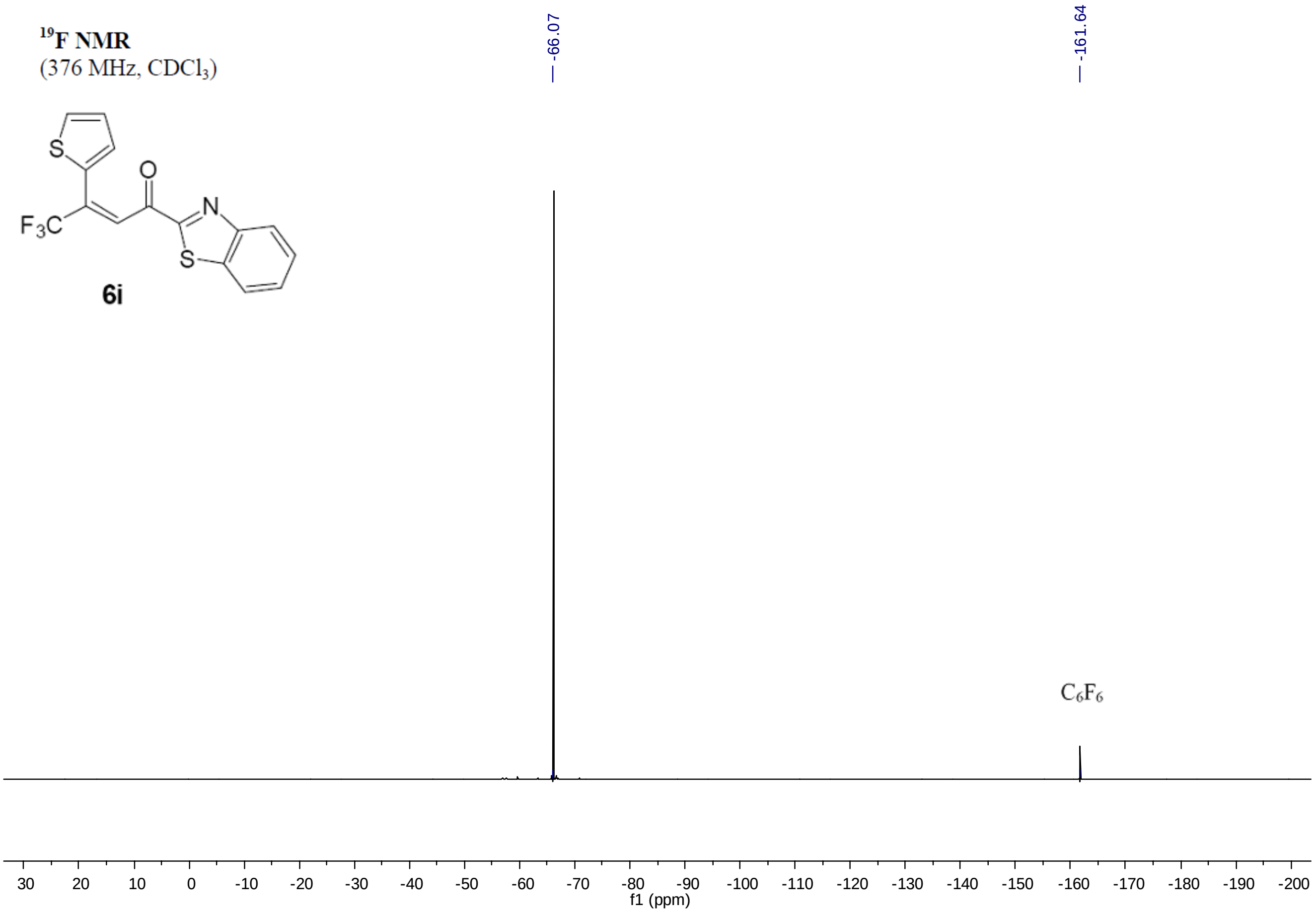
19F NMR
(376 MHz, CDCl3)
6i
-66.07
-161.64
C6F6
f1 (ppm)

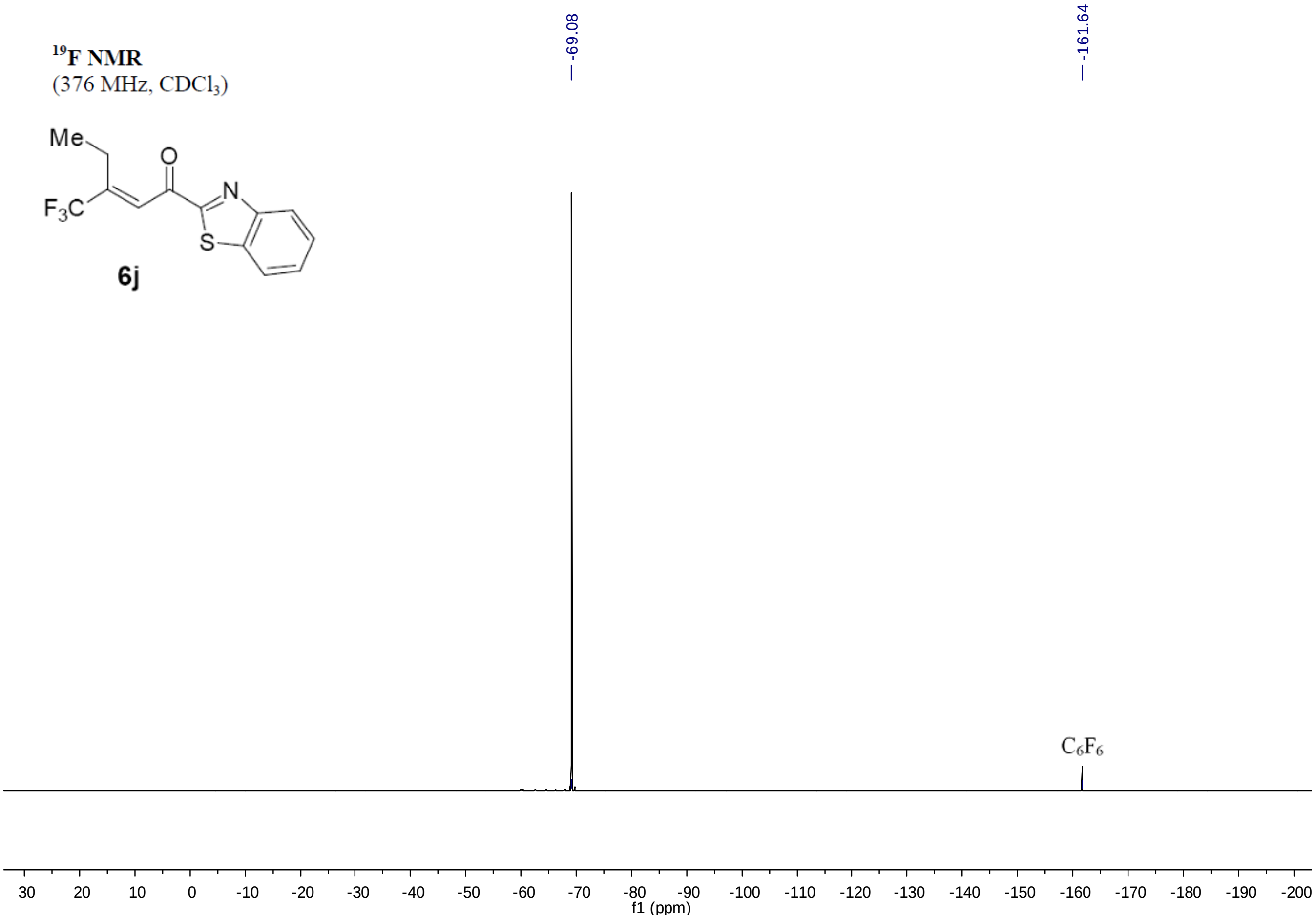
19F NMR
(376 MHz, CDCl3)
6j
-69.08
-161.64
C6F6
f1 (ppm)

^{19}F NMR
(376 MHz, $CDCl_3$)

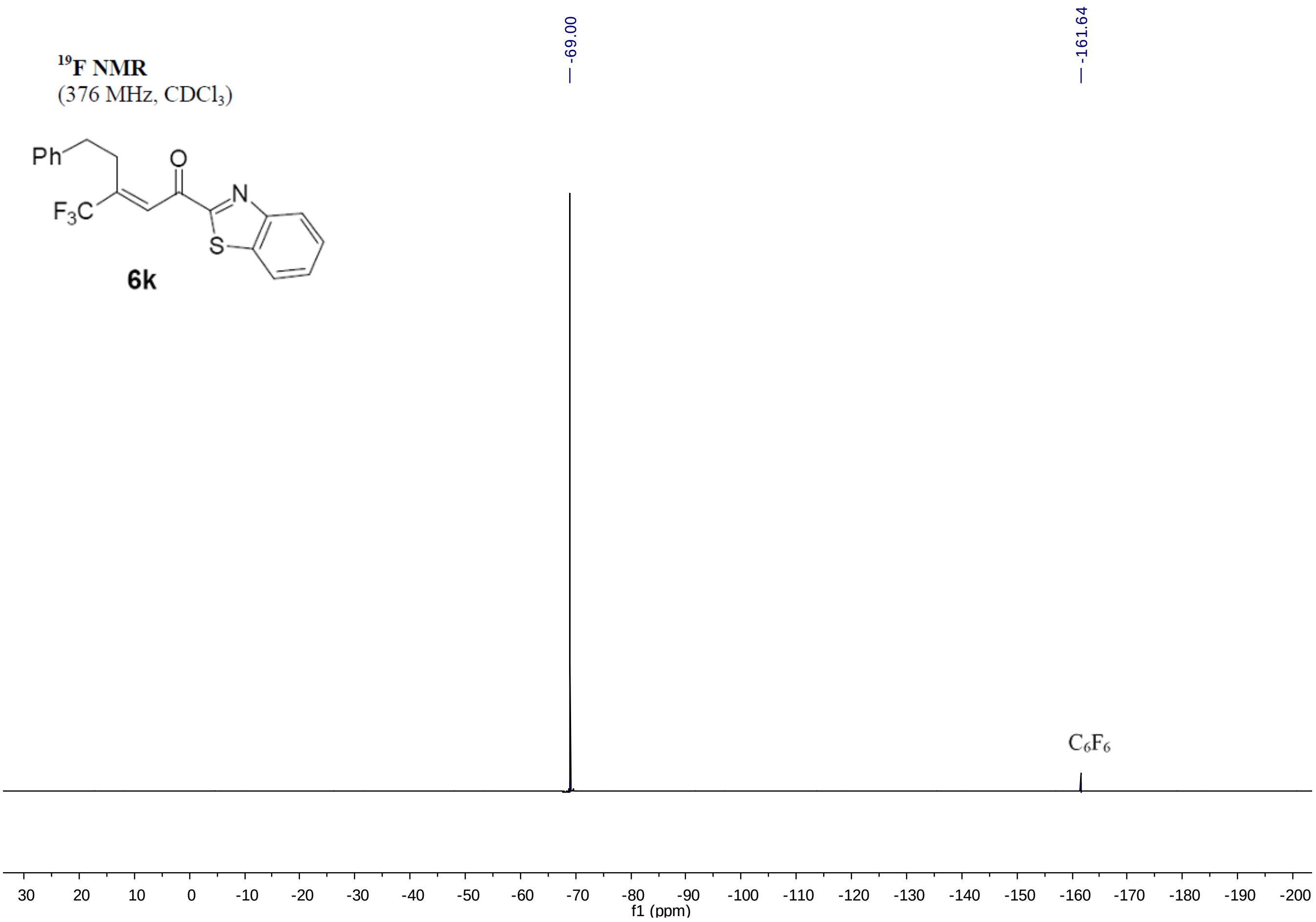

^{19}F NMR
(376 MHz, $CDCl_3$)

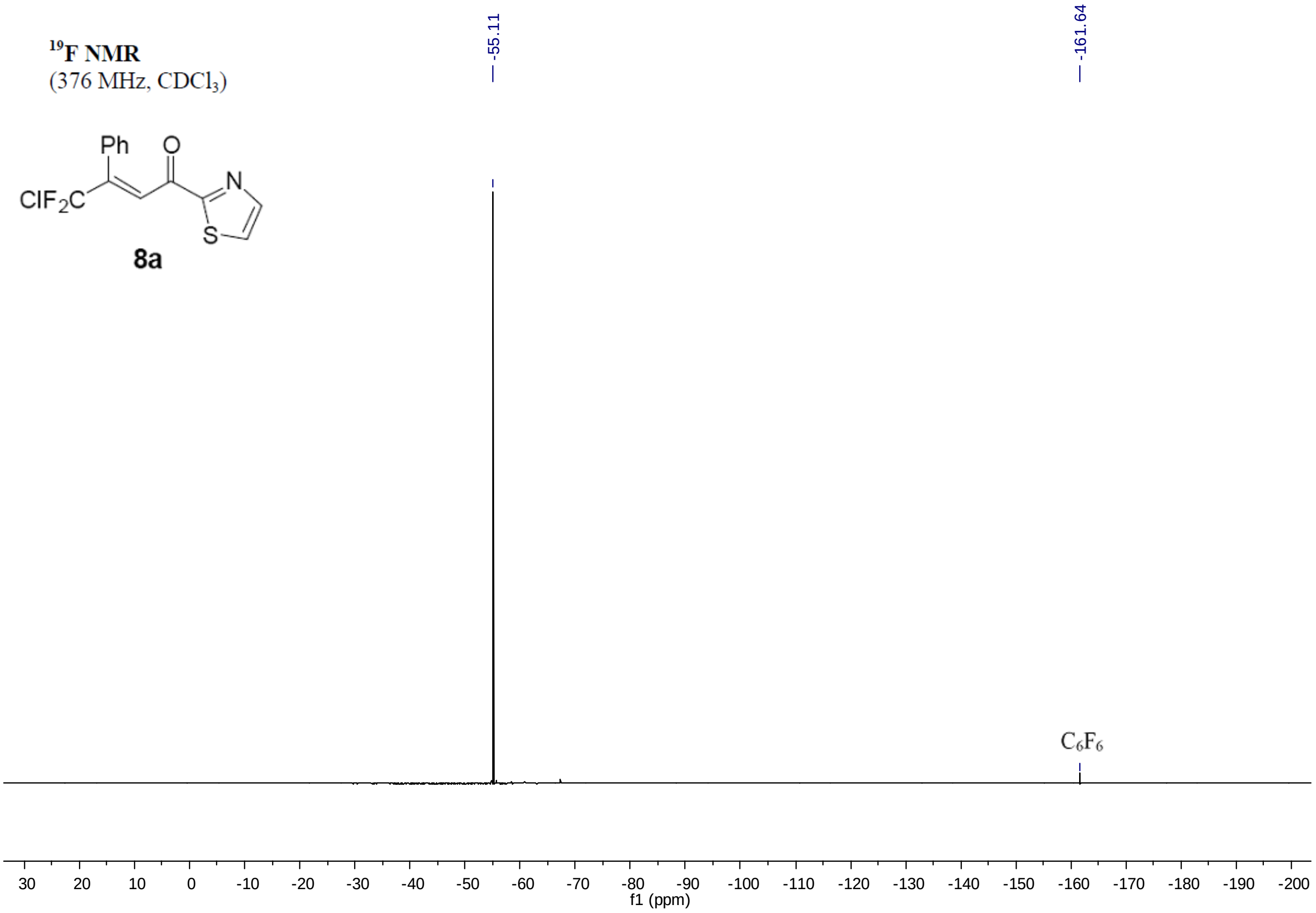

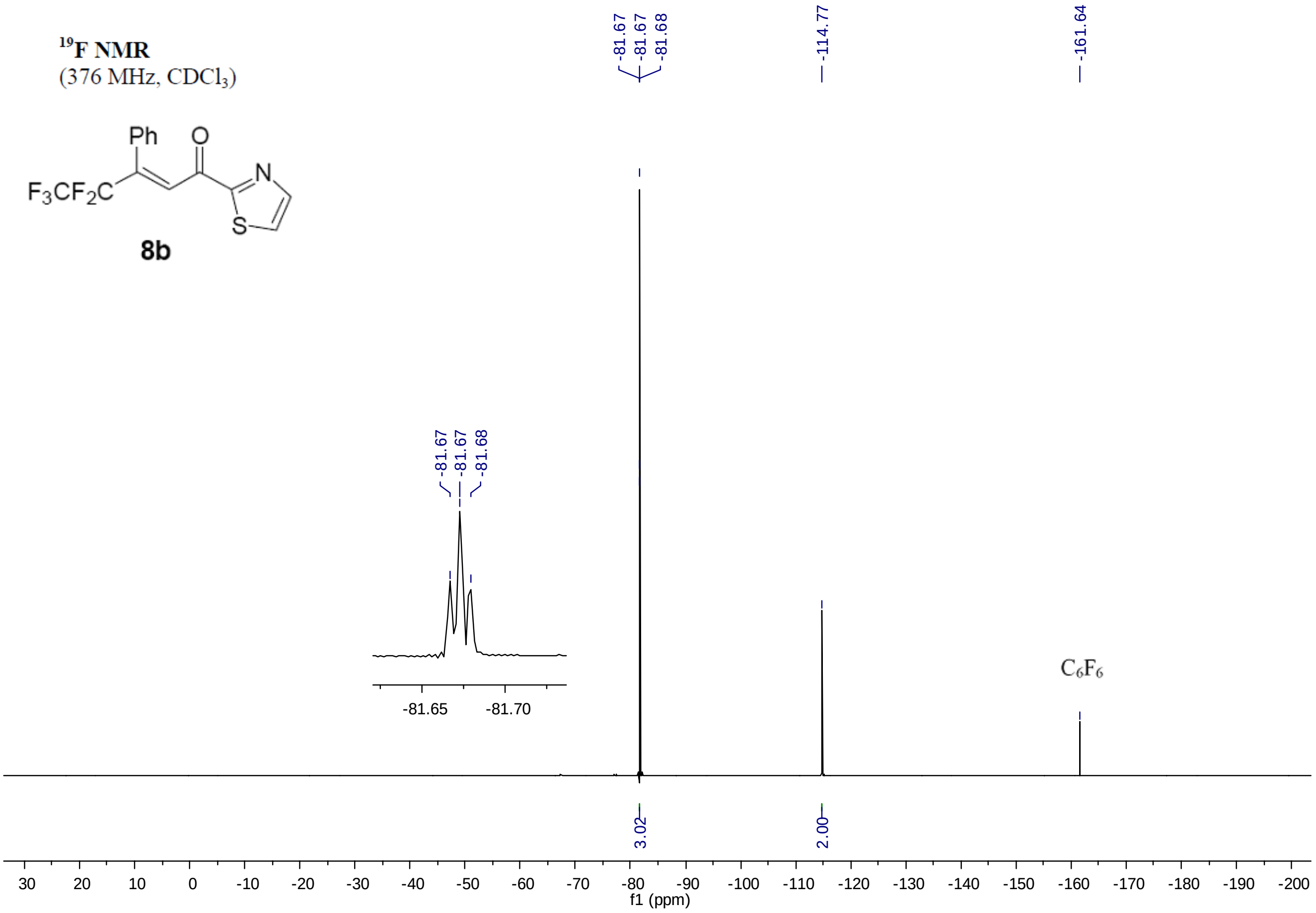

Copies of DEPT 135 spectra – enones 2e-2k, 4a-4u, 6a-6k, 8a and 8b

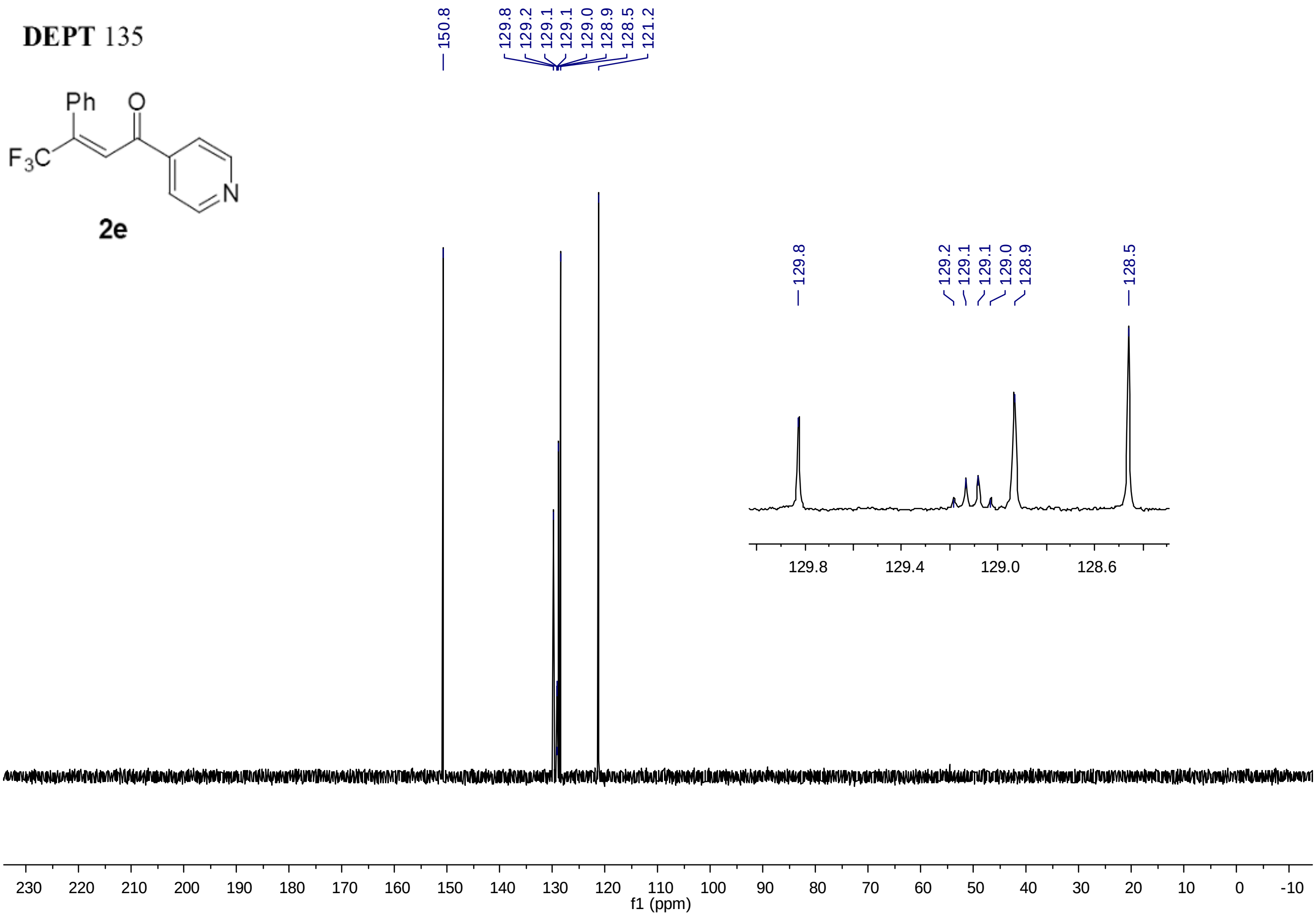

DEPT 135

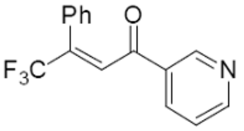

2f

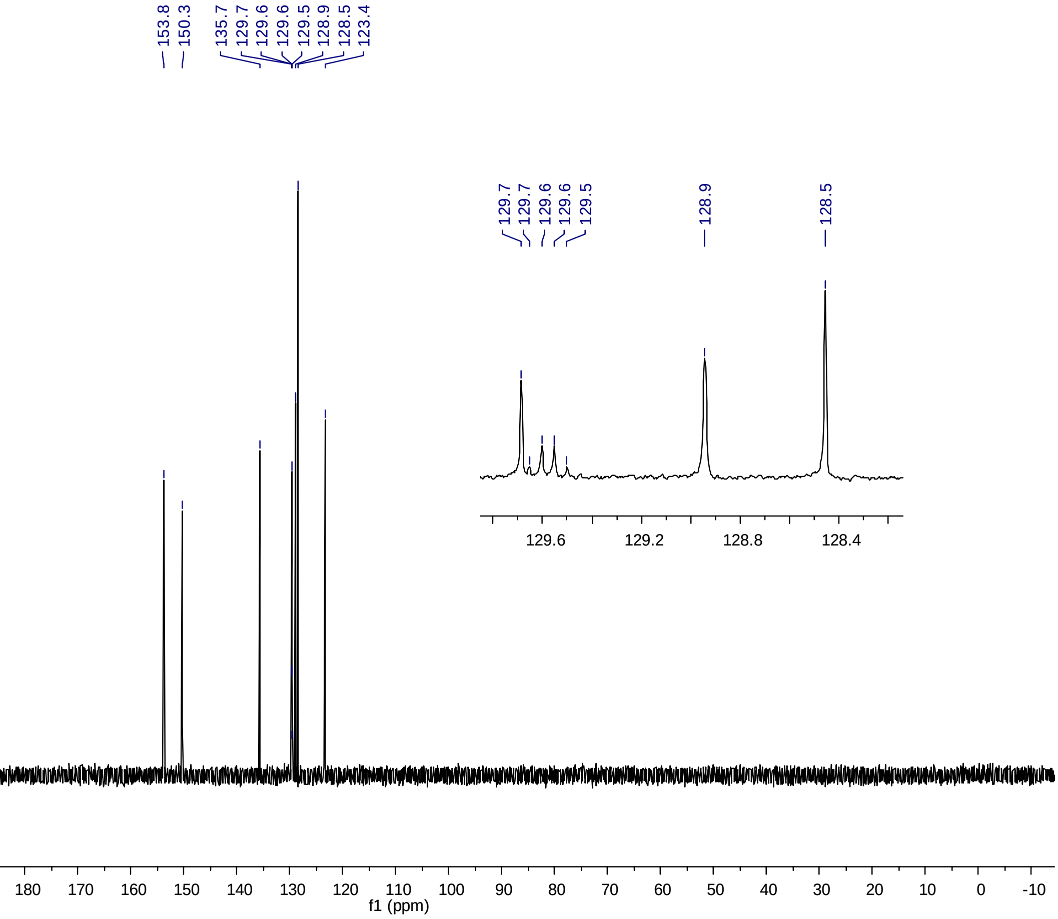

DEPT 135

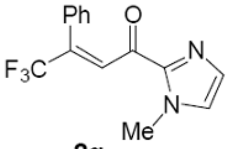

2g

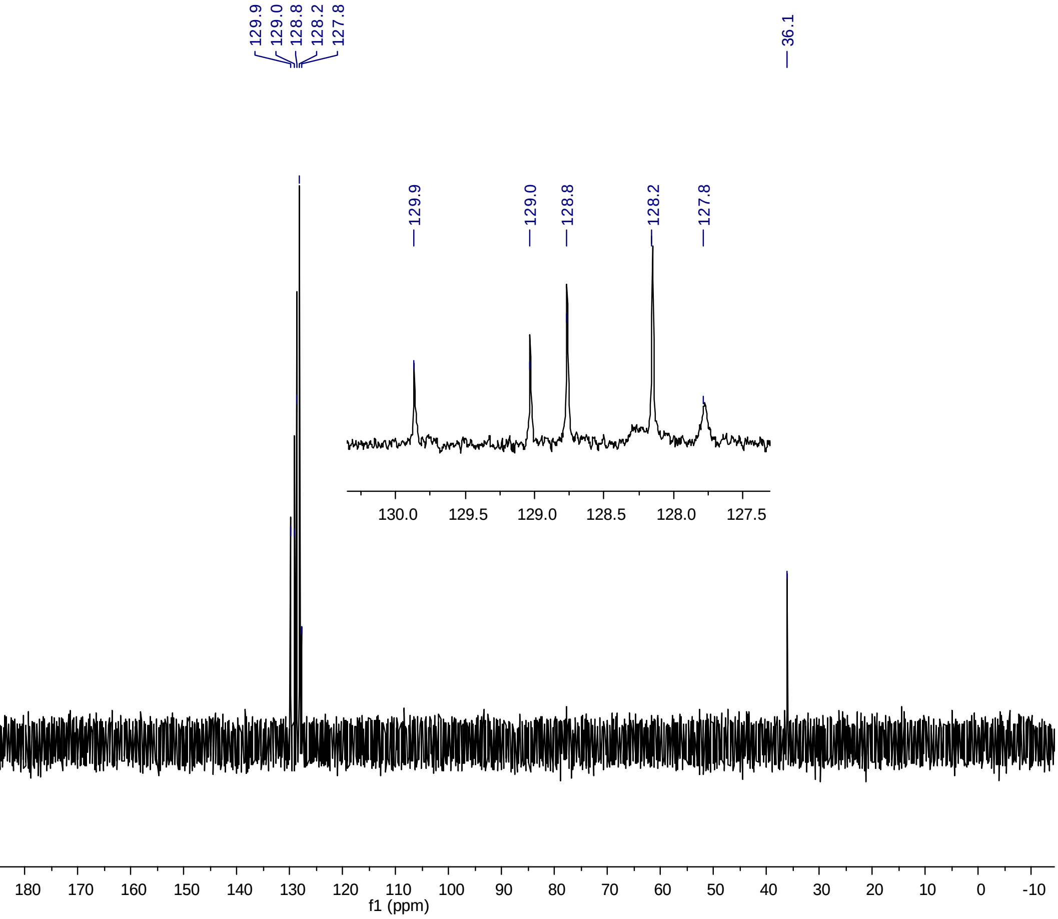

DEPT 135

(*E*)-**2h**

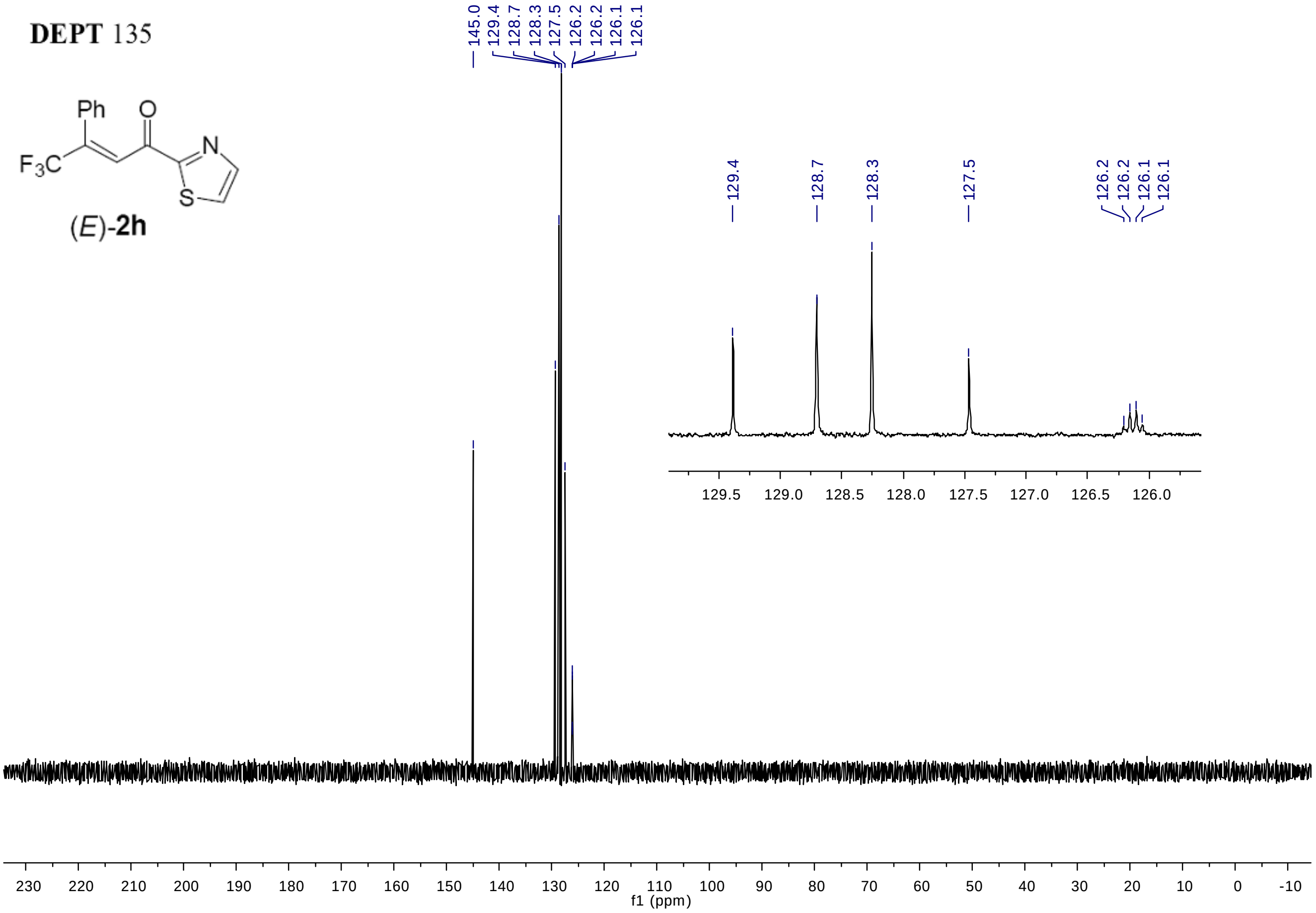

DEPT 135

(*Z*)-**2h**

(minor isomer)

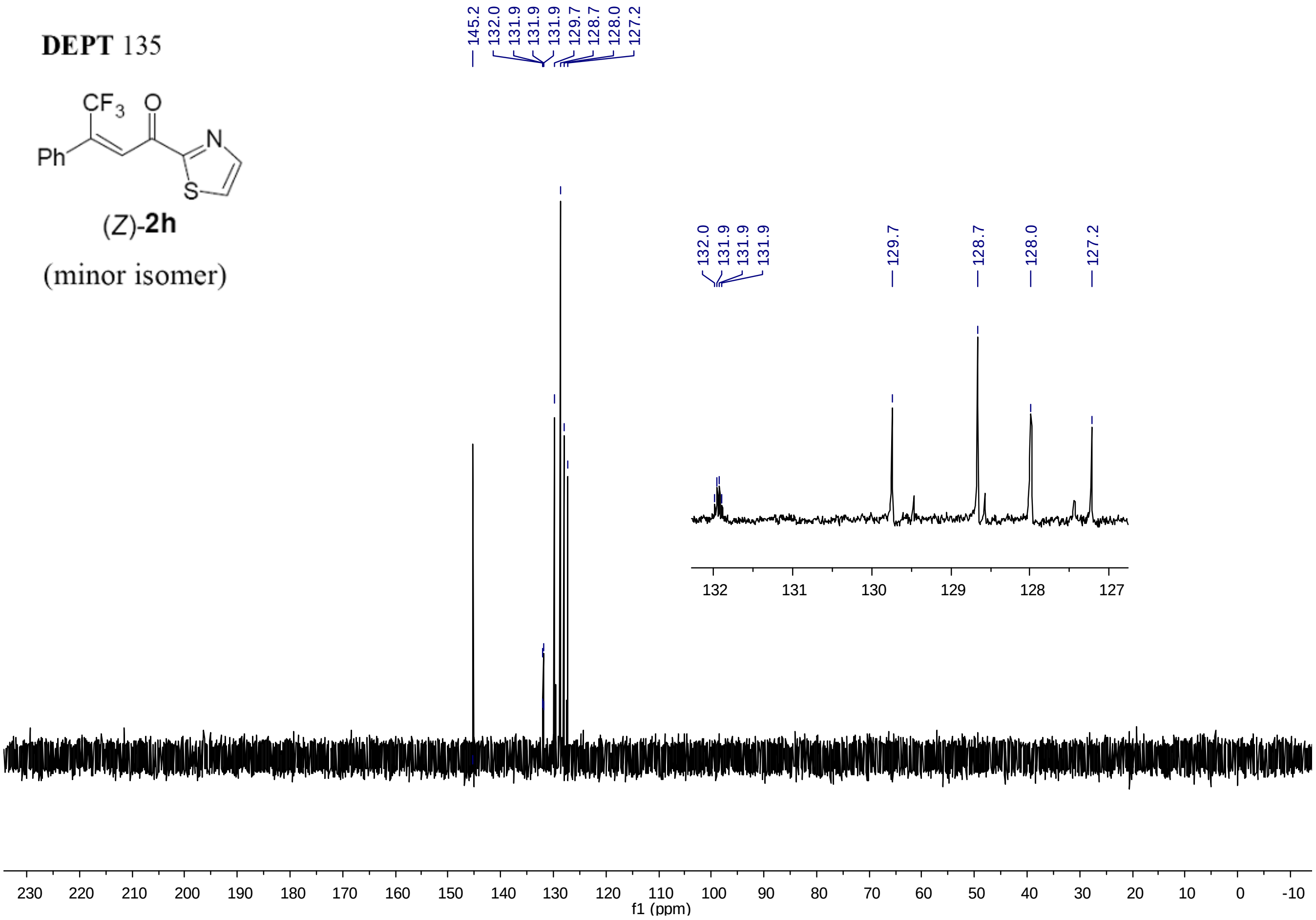

DEPT 135

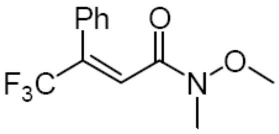

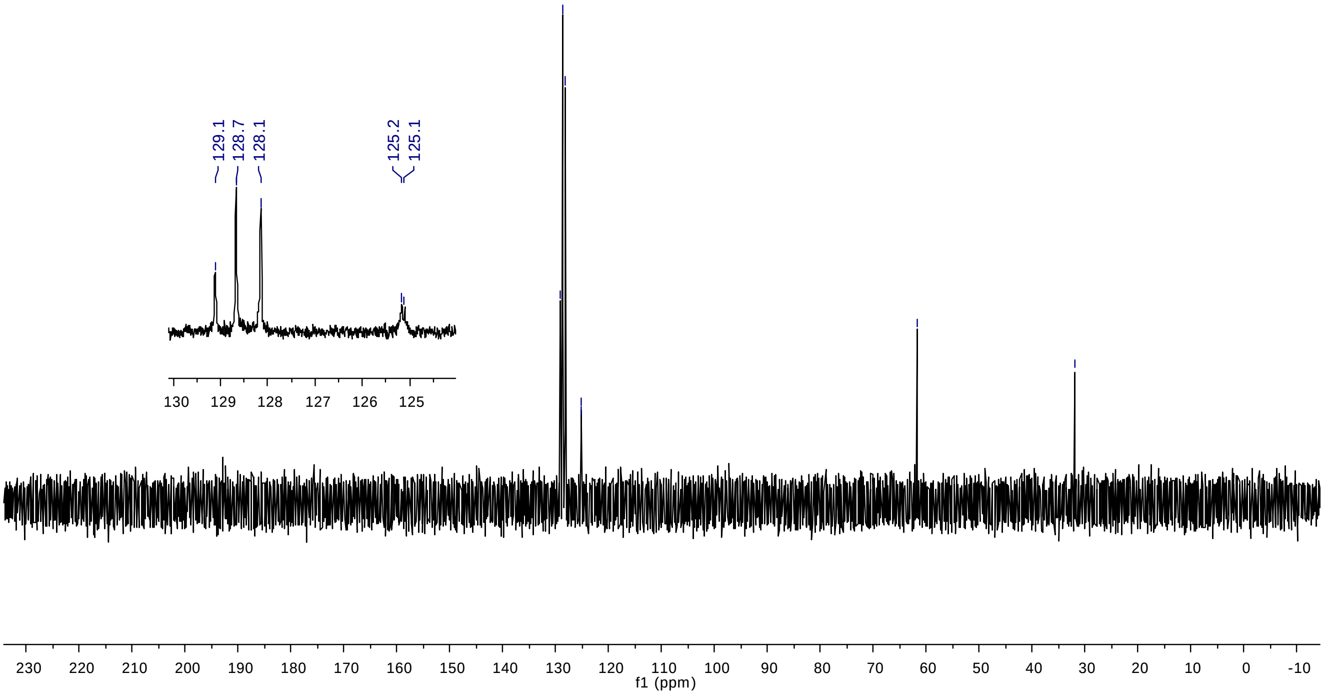

DEPT 135

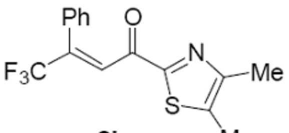

2i

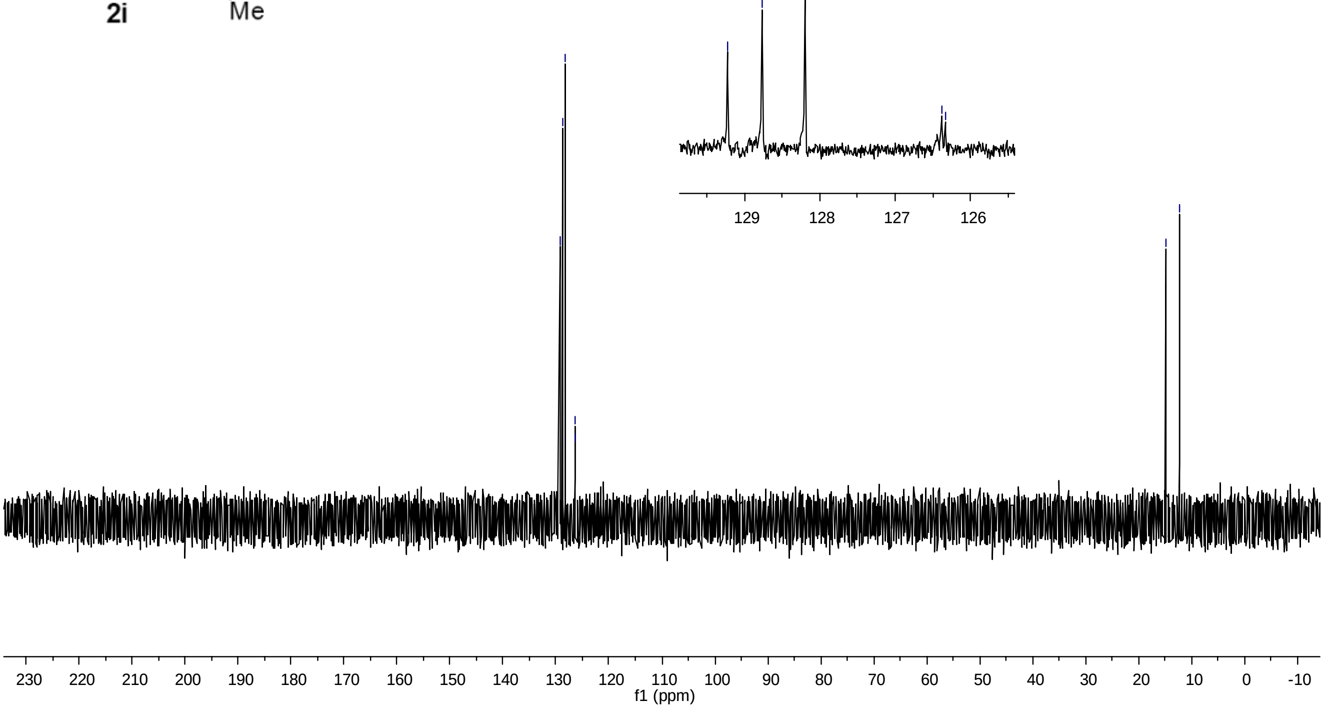

DEPT 135

2j

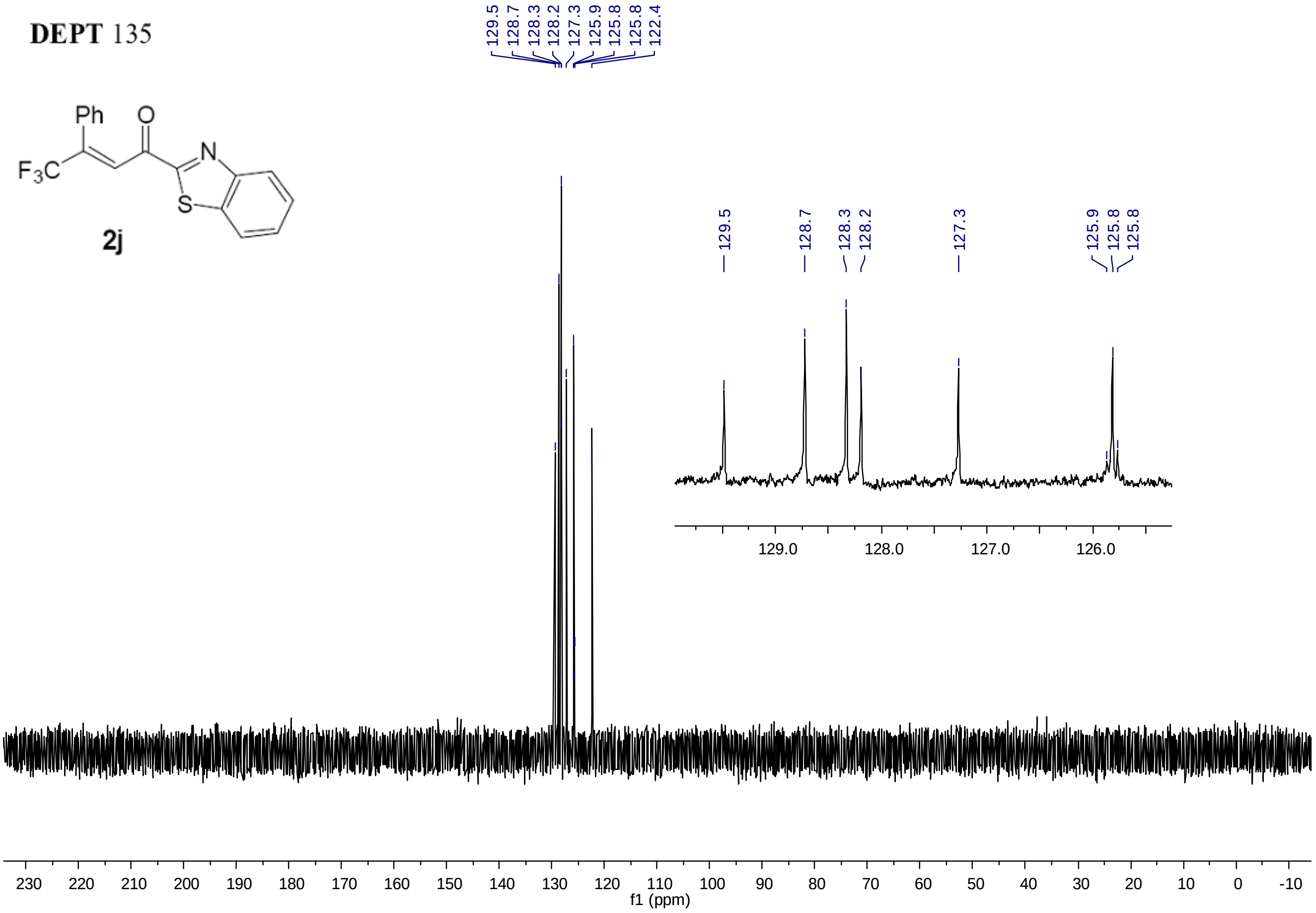

DEPT 135

2k

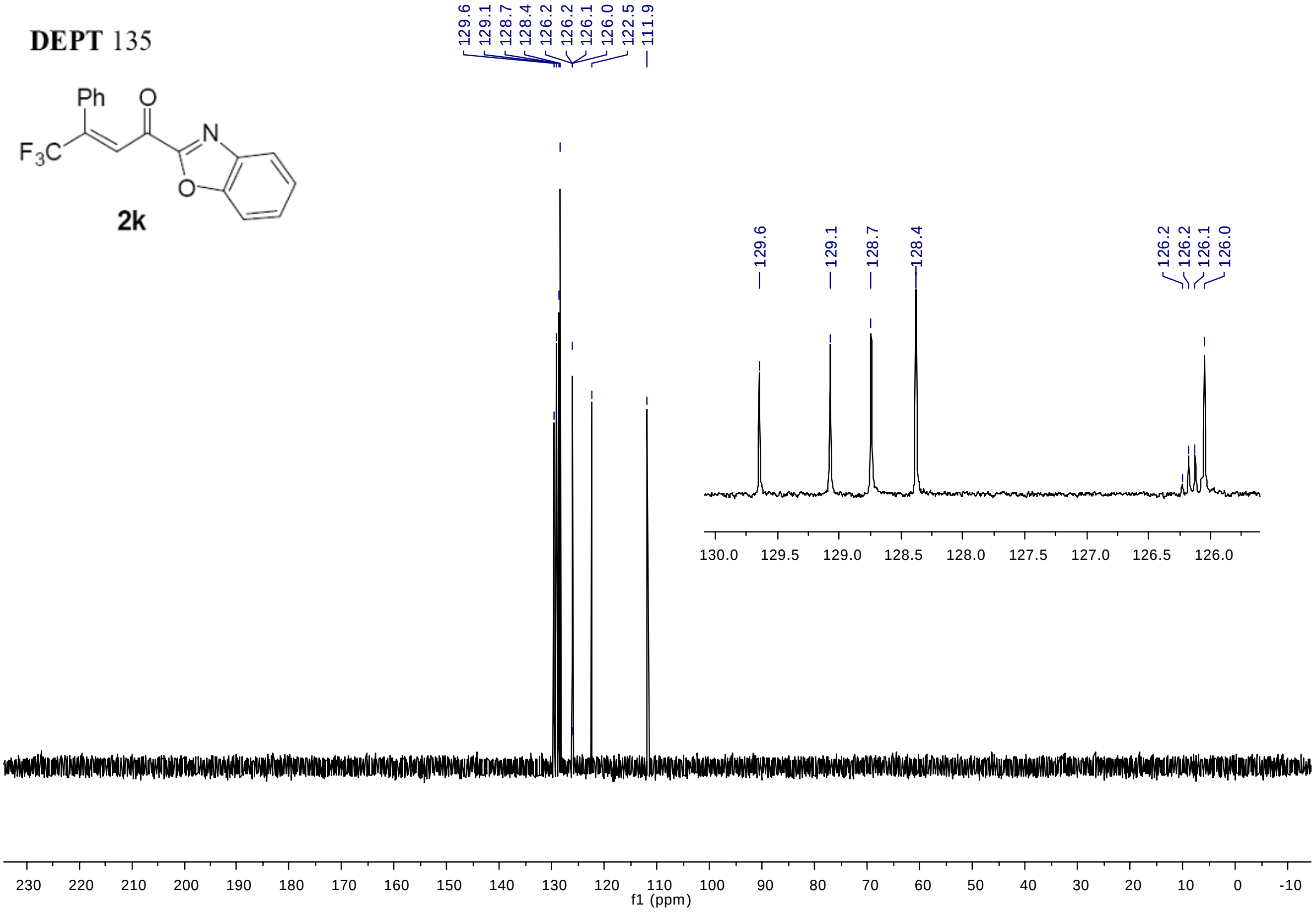

DEPT 135

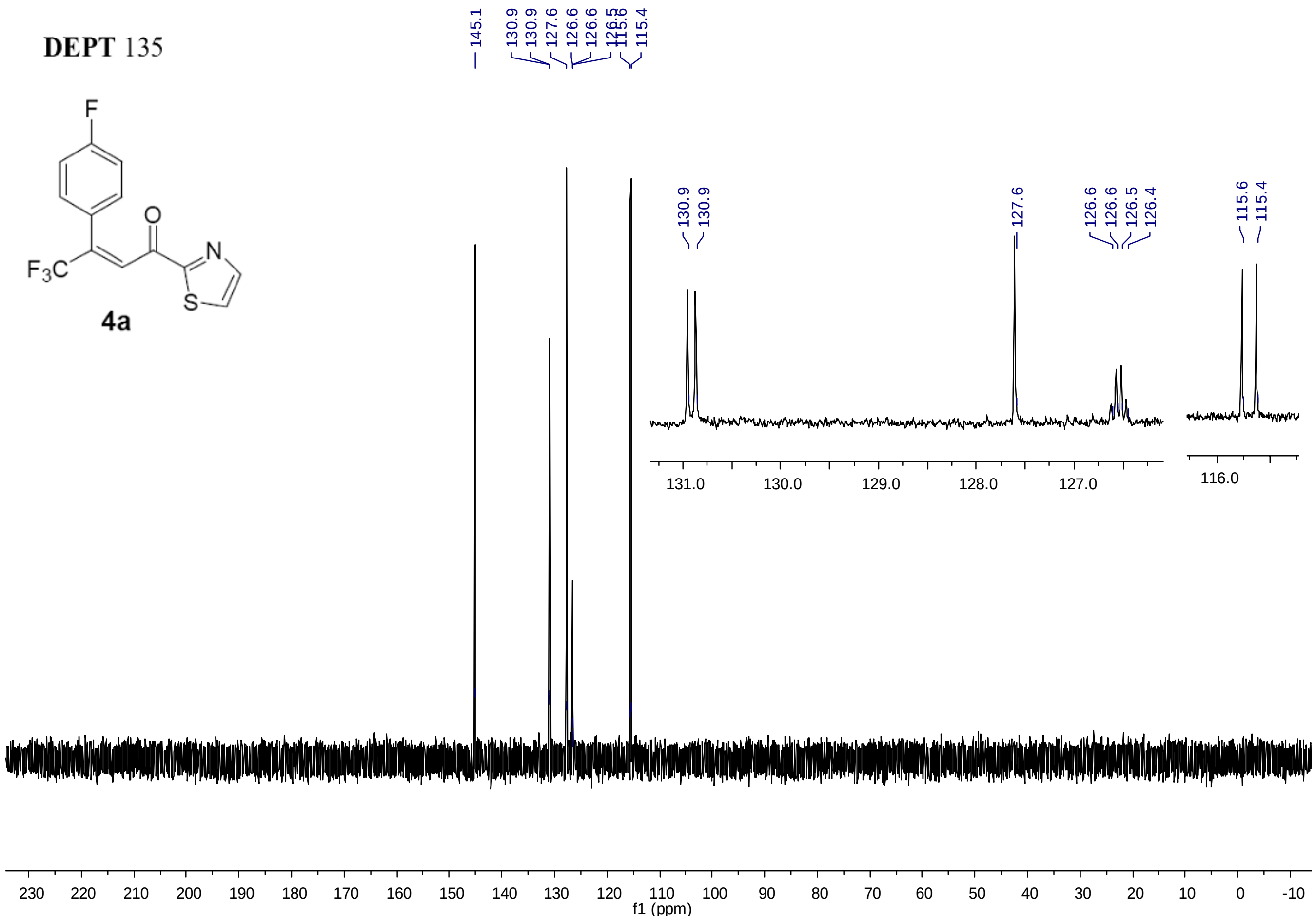

DEPT 135

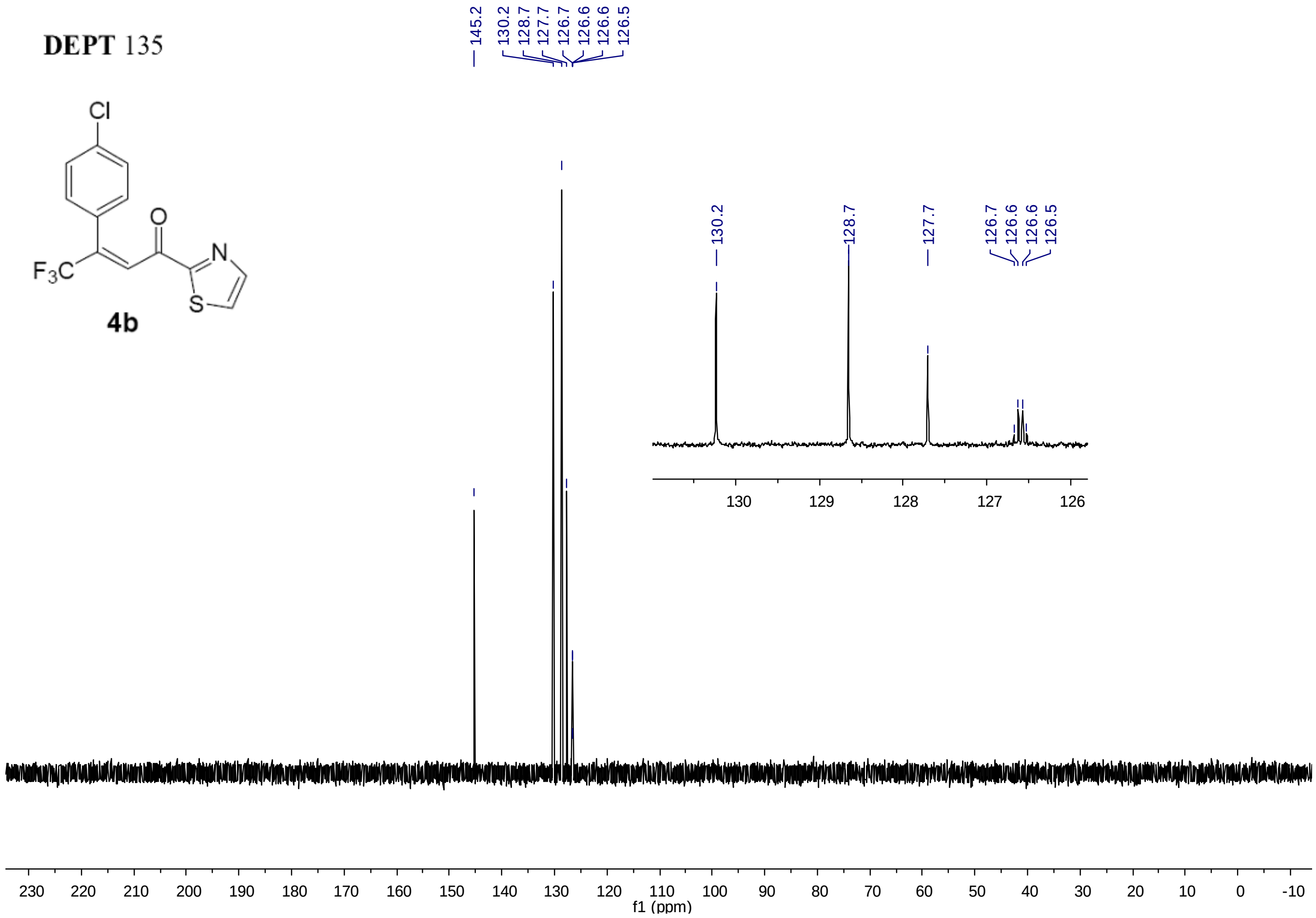

DEPT 135

4c

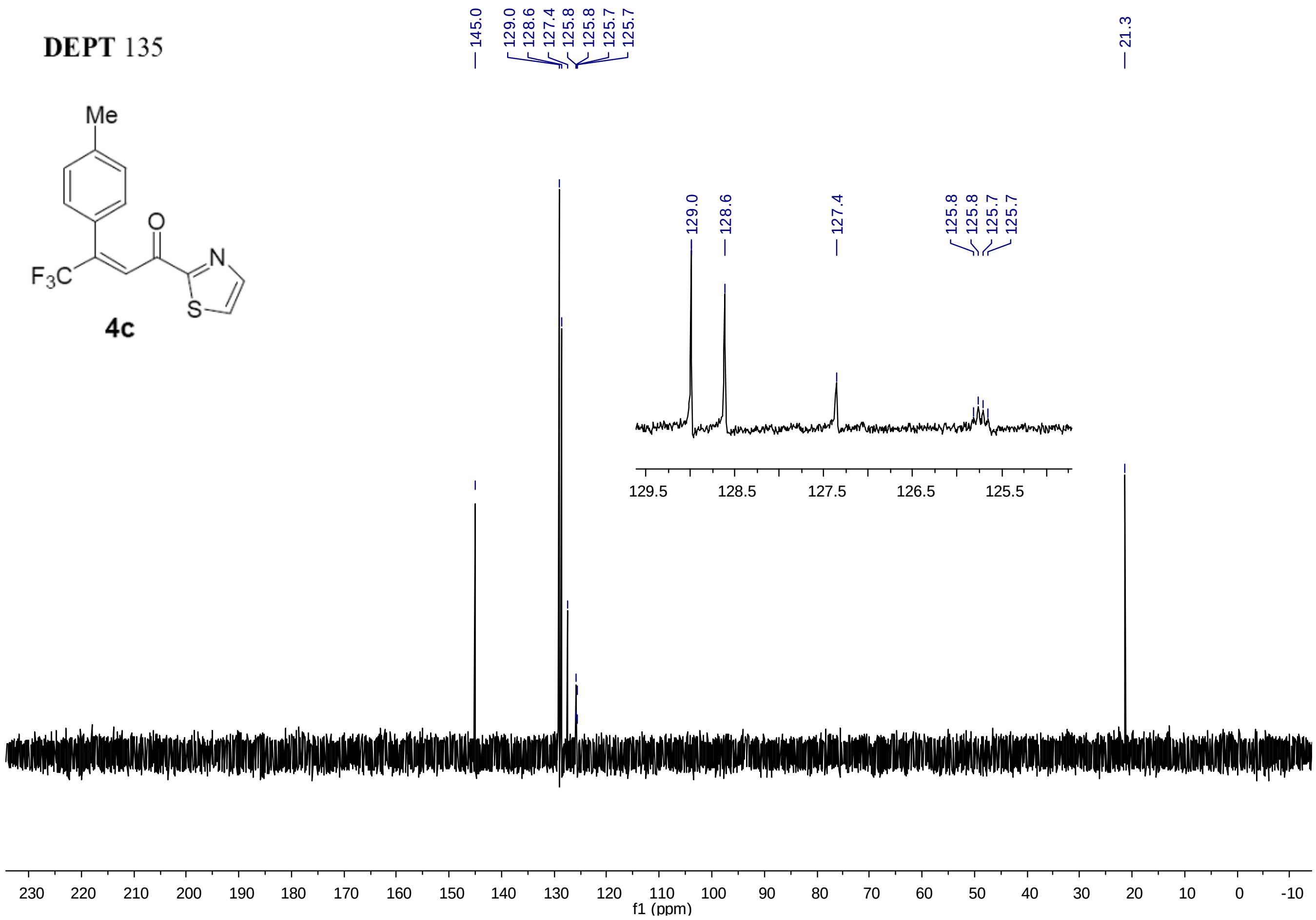

DEPT 135

4d

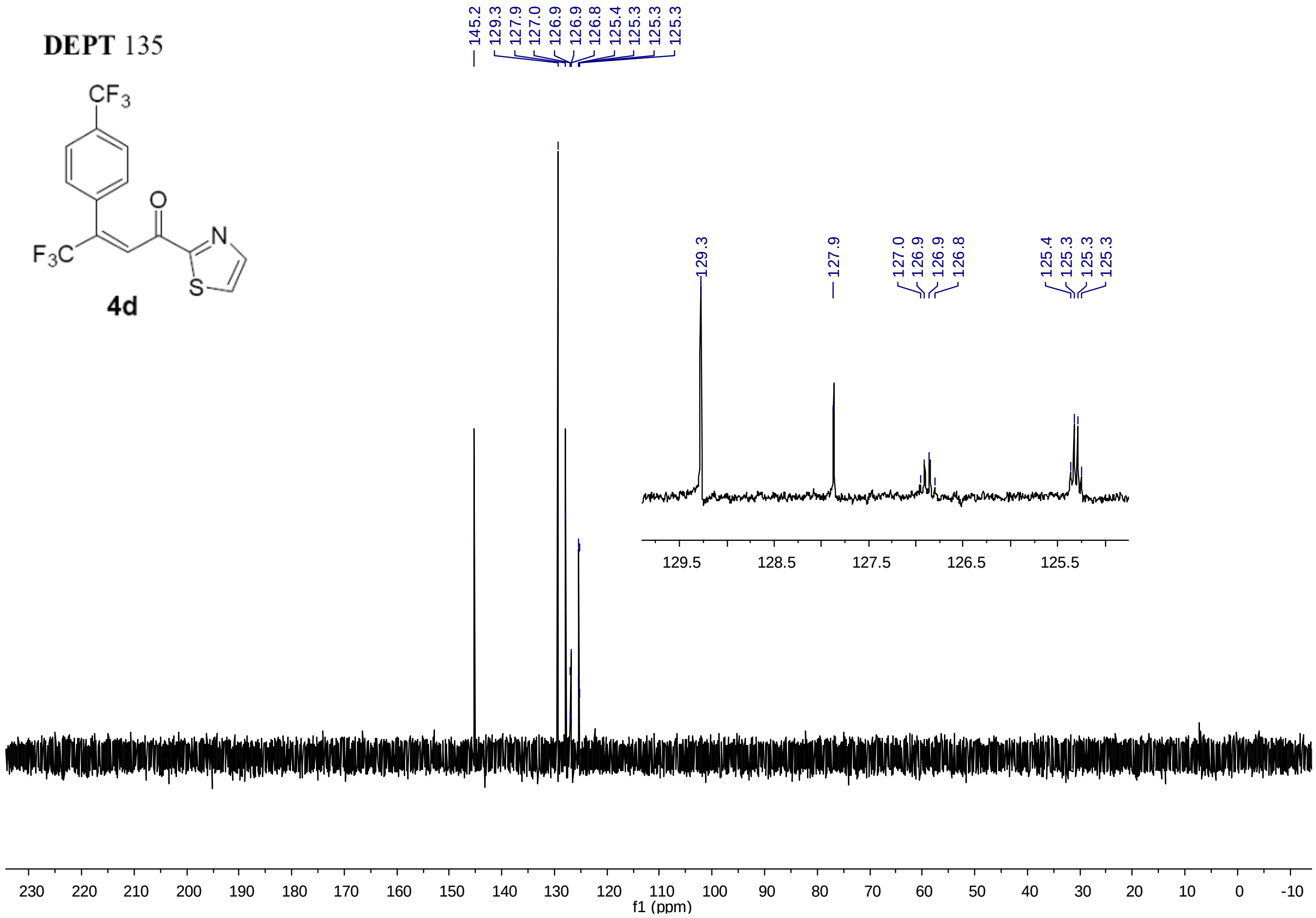

DEPT 135

4e

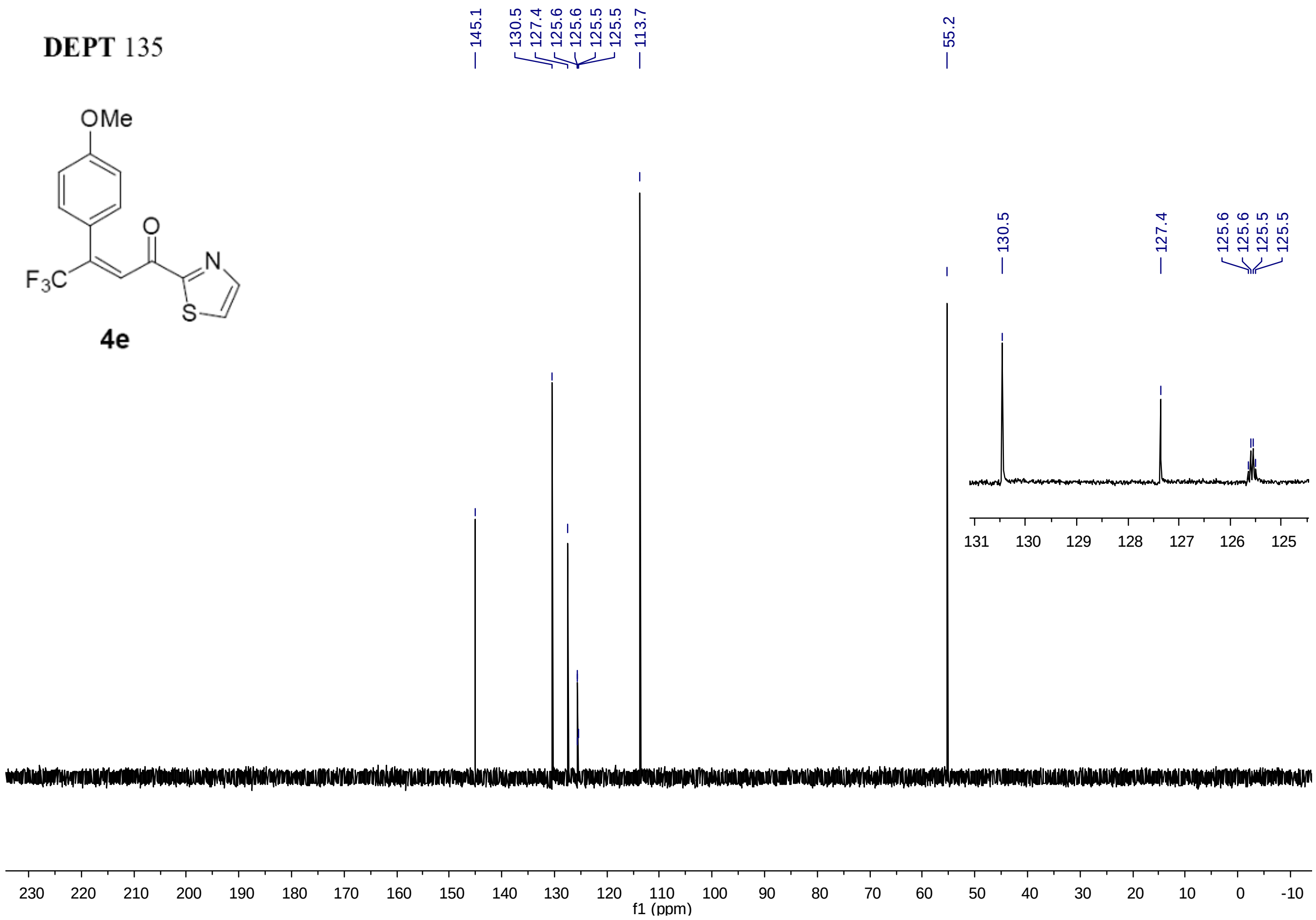

DEPT 135

4f

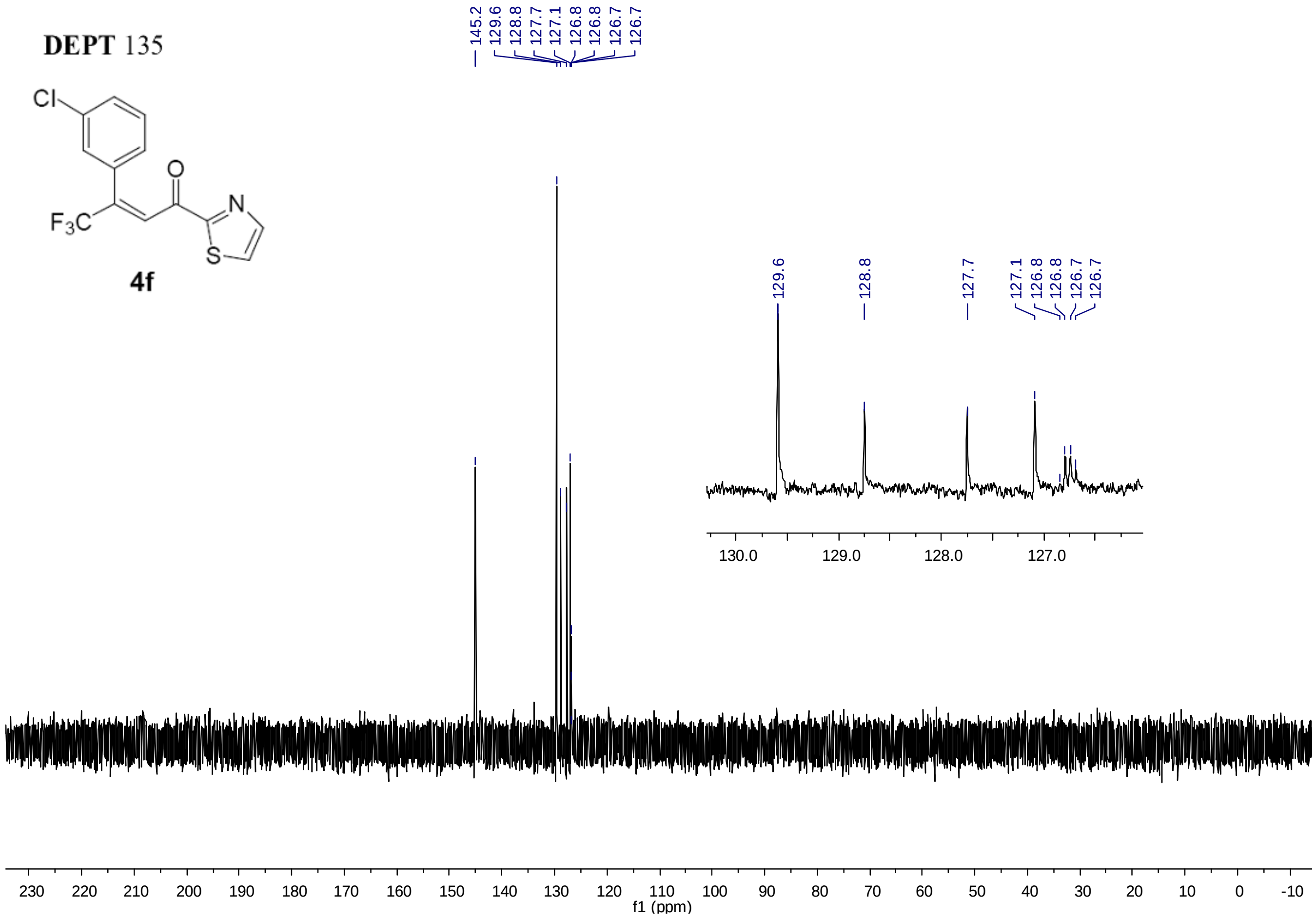

DEPT 135

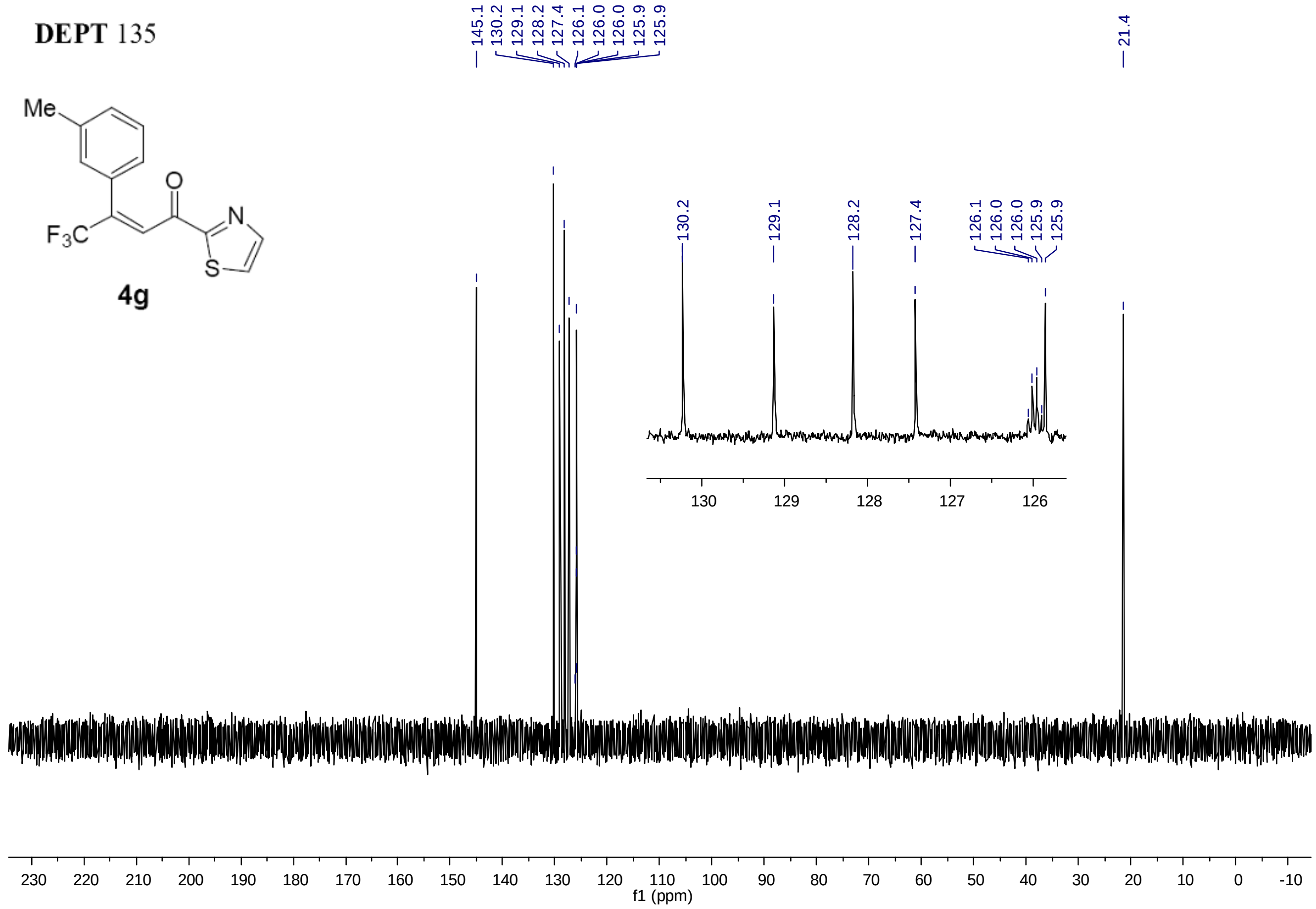

4g

DEPT 135

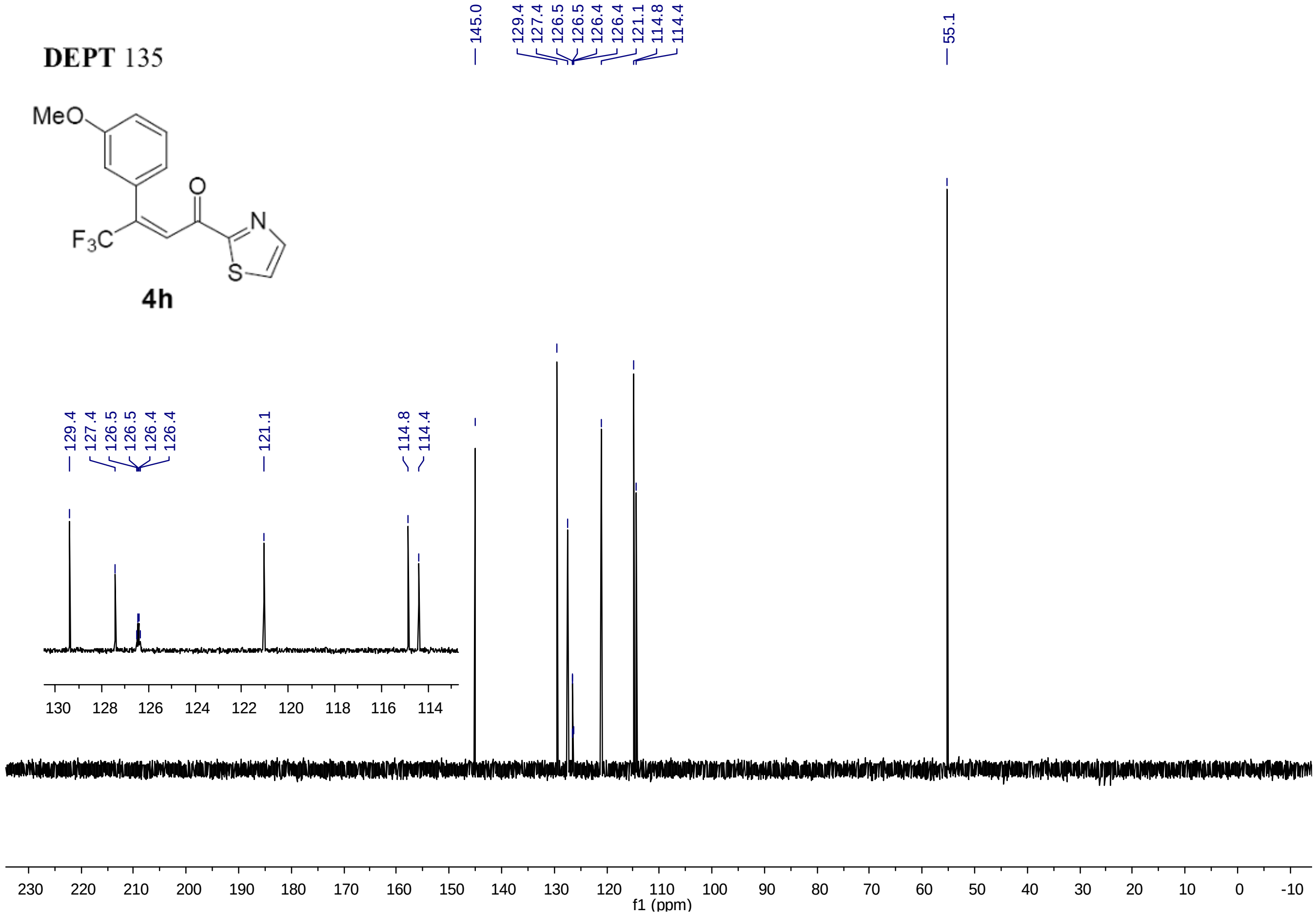

4h

DEPT 135

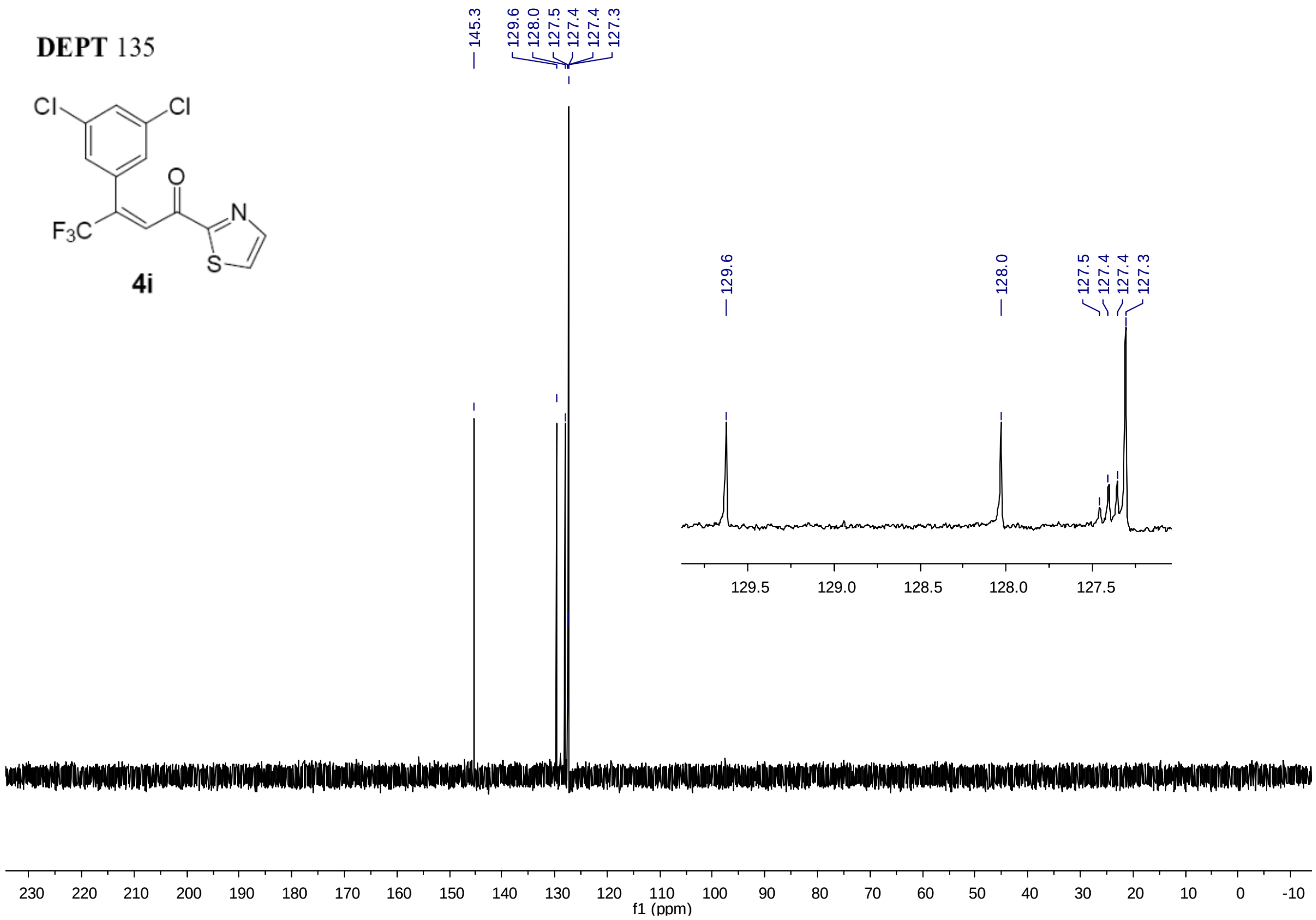

DEPT 135

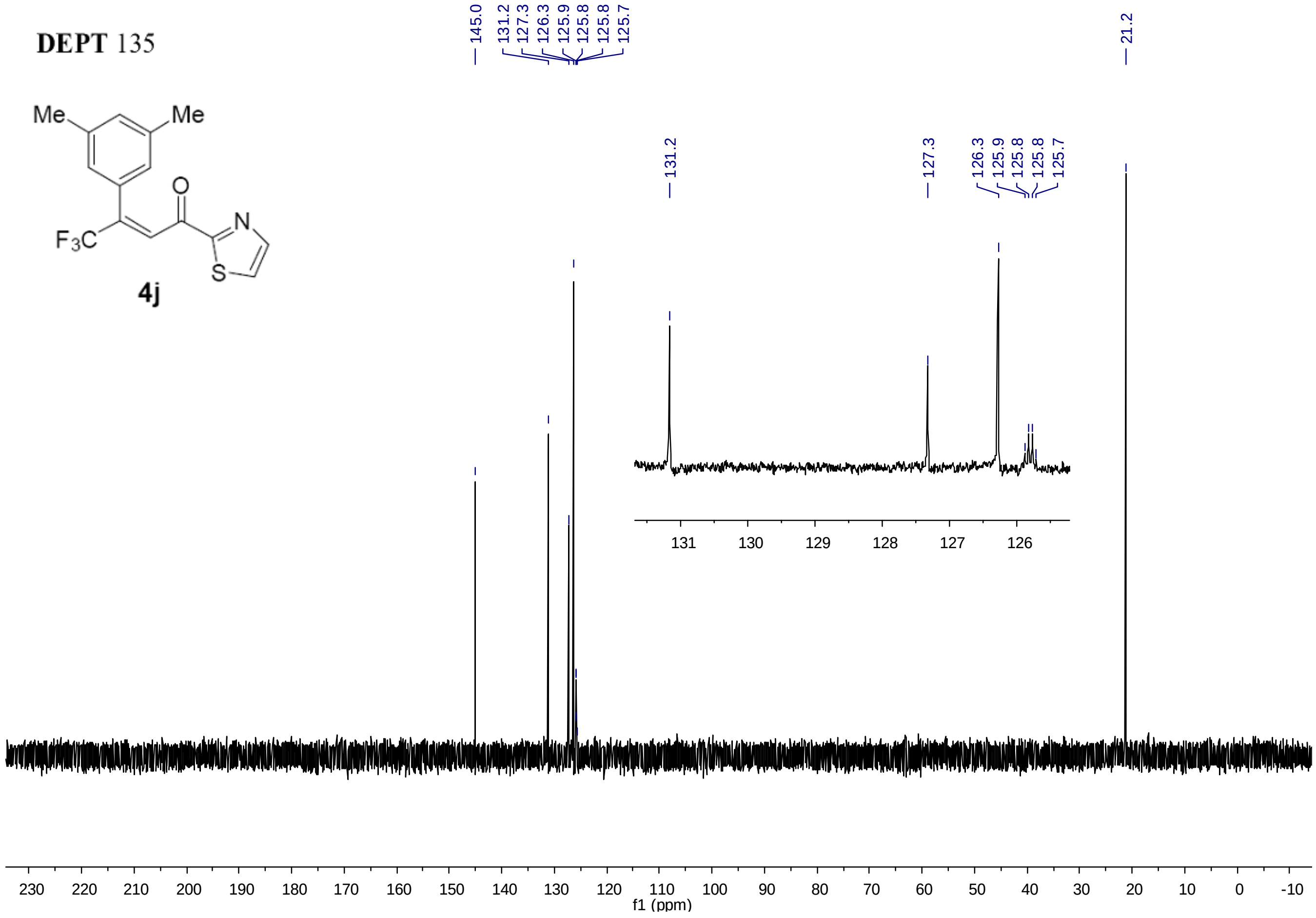

DEPT 135

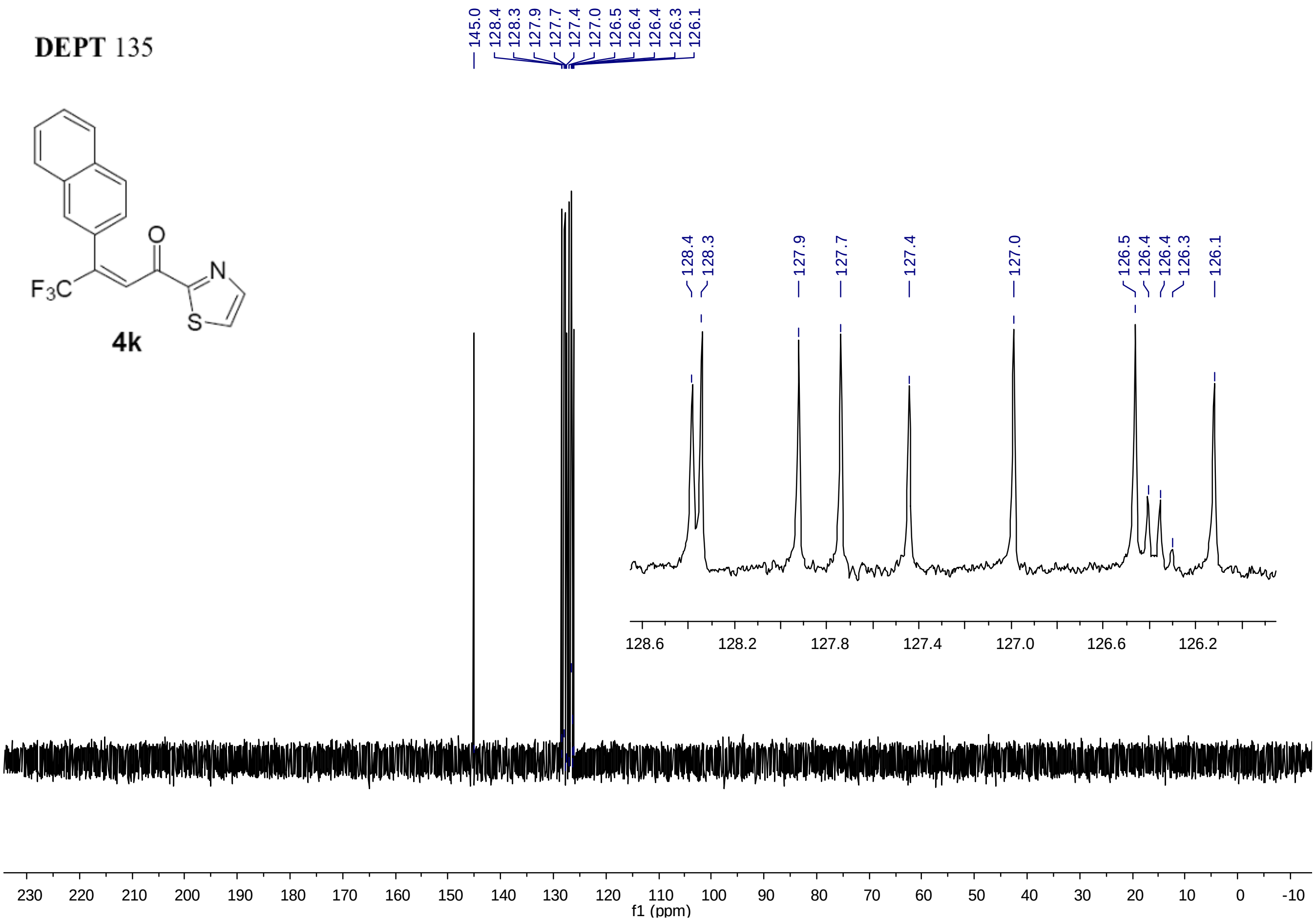

DEPT 135

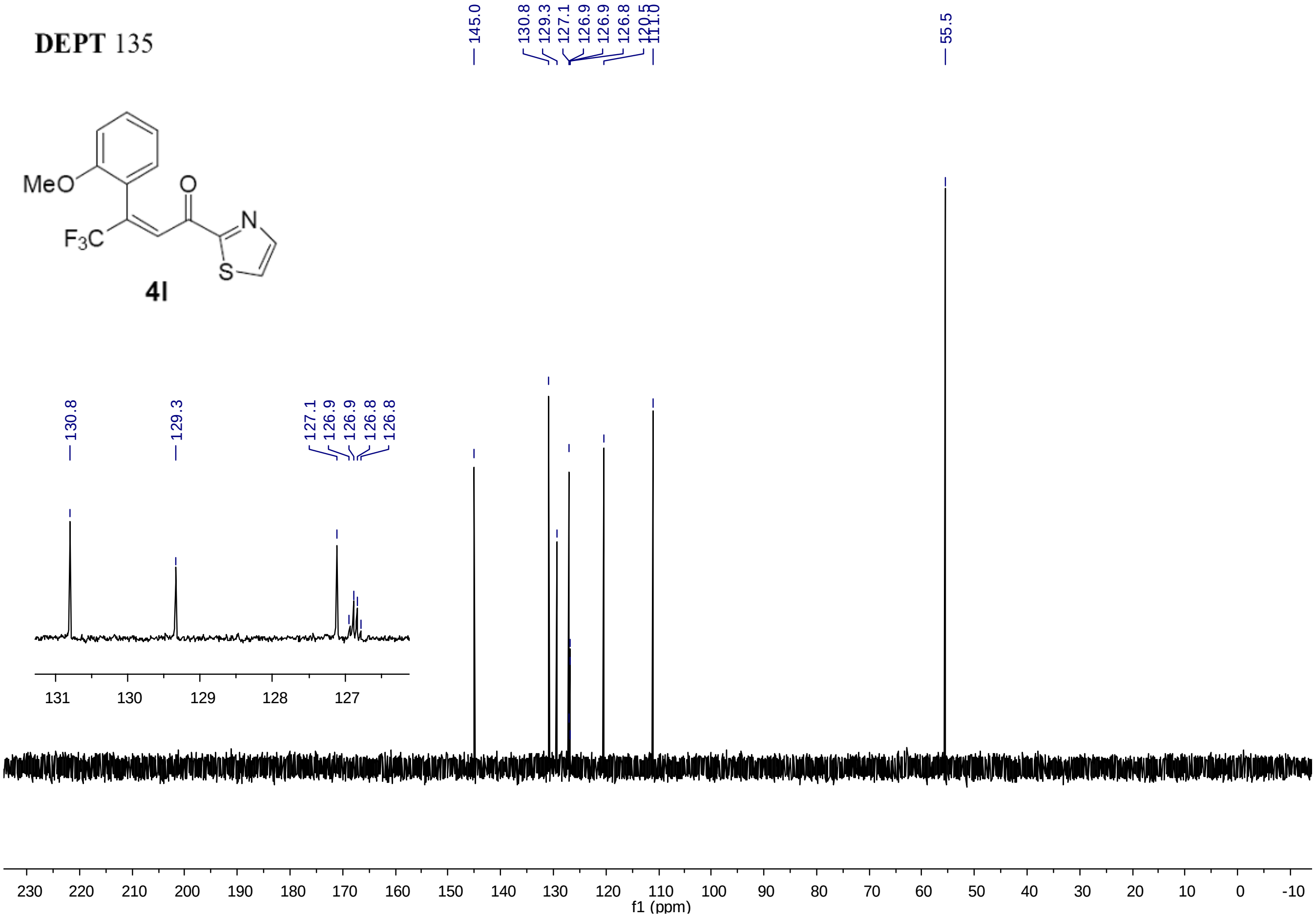

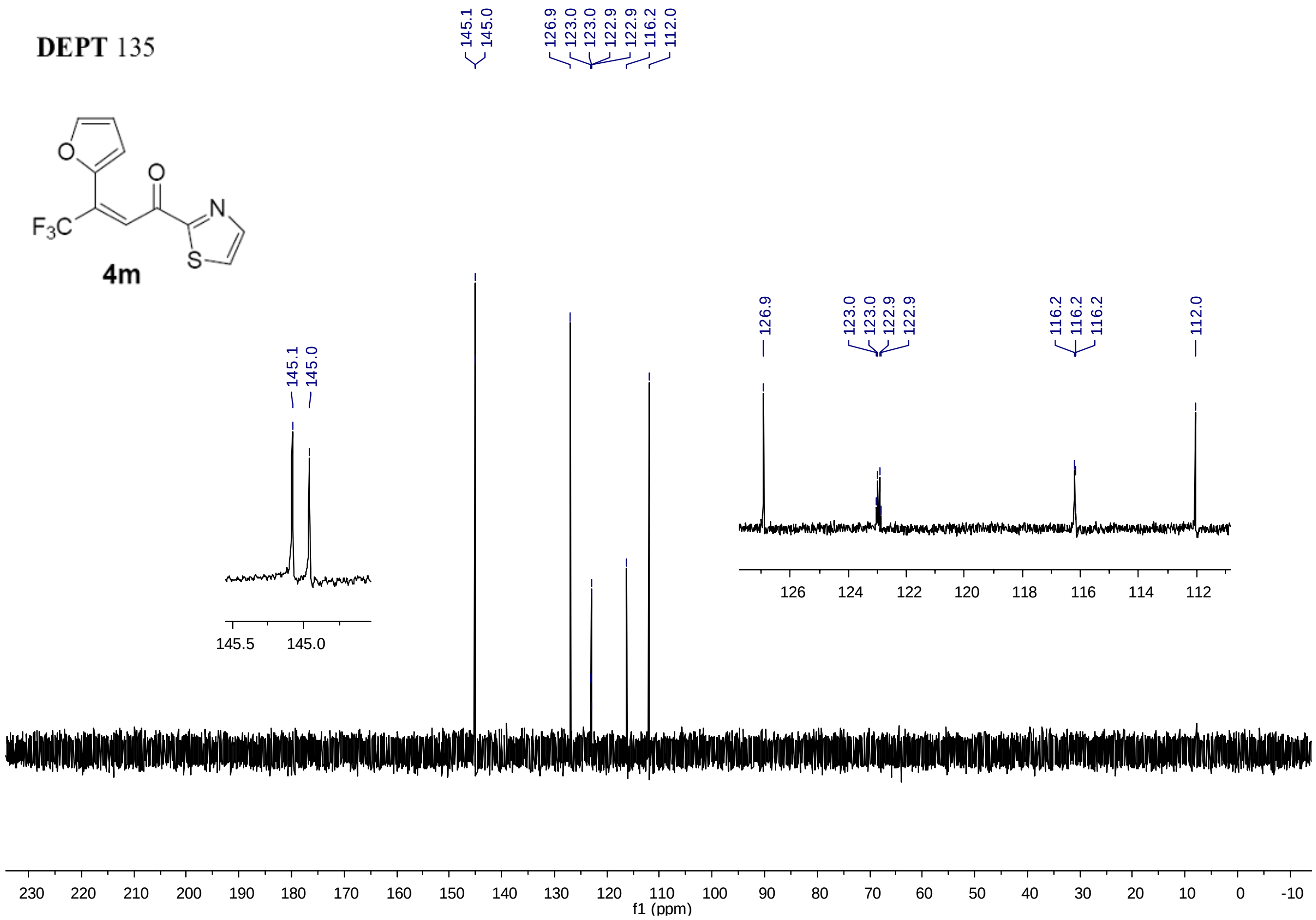
DEPT 135
4m
145.1
145.0
126.9
123.0
123.0
122.9
122.9
116.2
116.2
116.2
112.0
f1 (ppm)

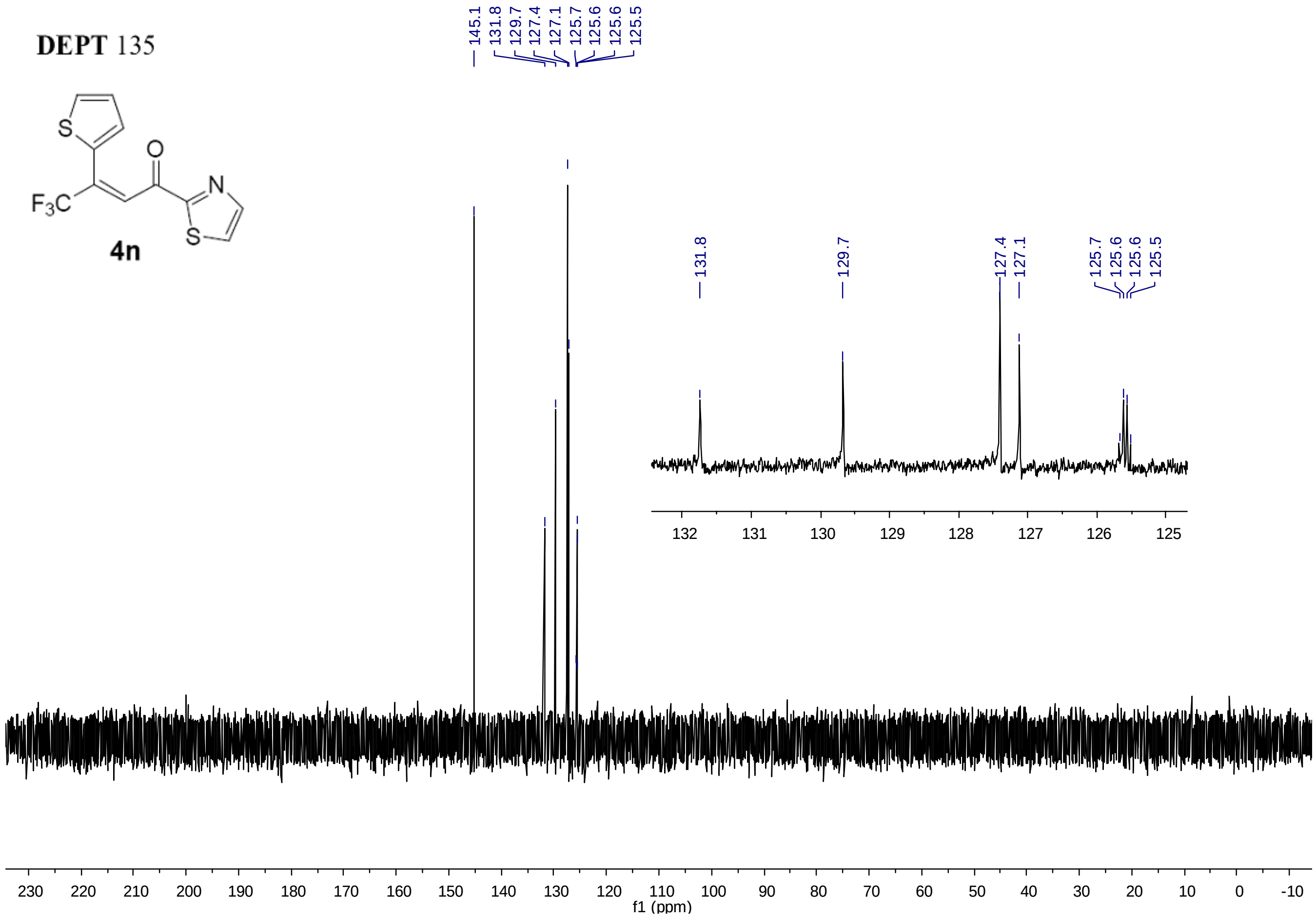
DEPT 135
4n
145.1
131.8
129.7
127.4
127.1
125.7
125.6
125.6
125.5
f1 (ppm)

DEPT 135

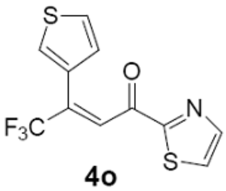

4o

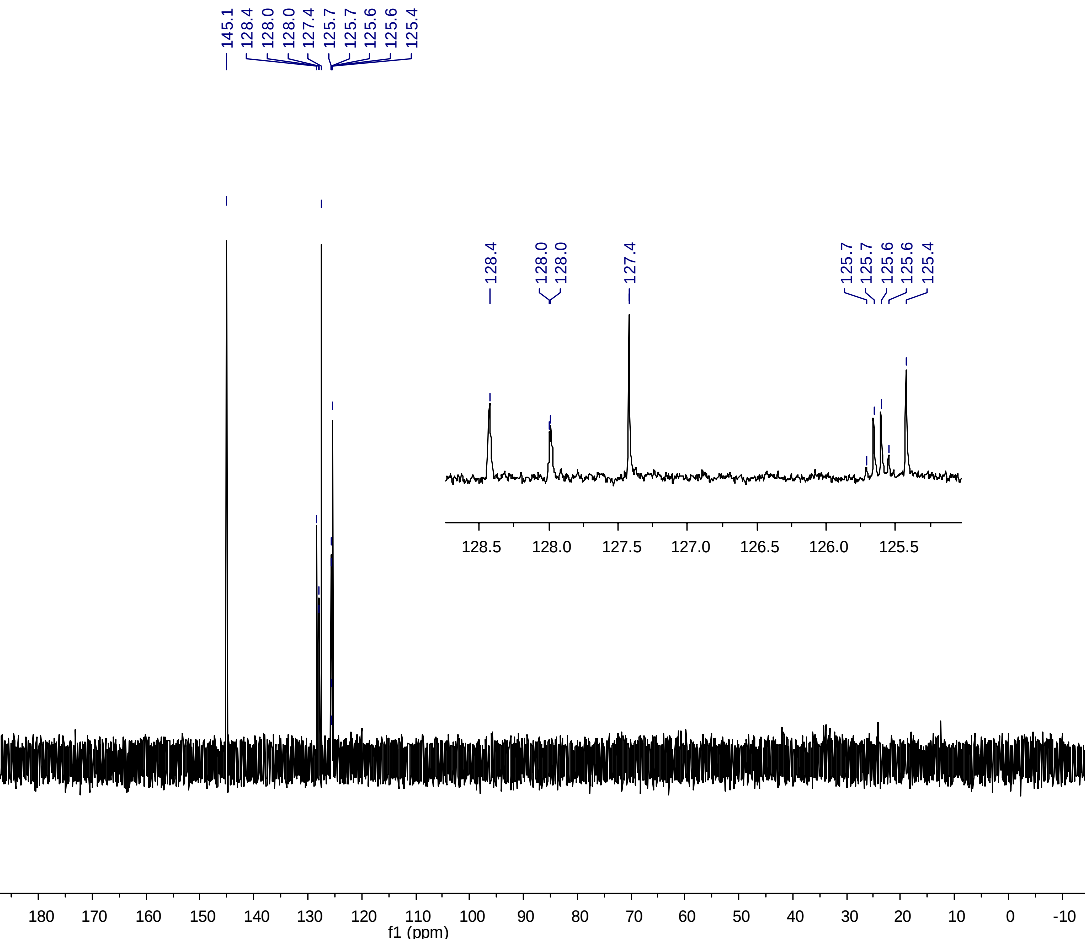

DEPT 135

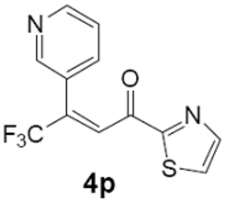

4p

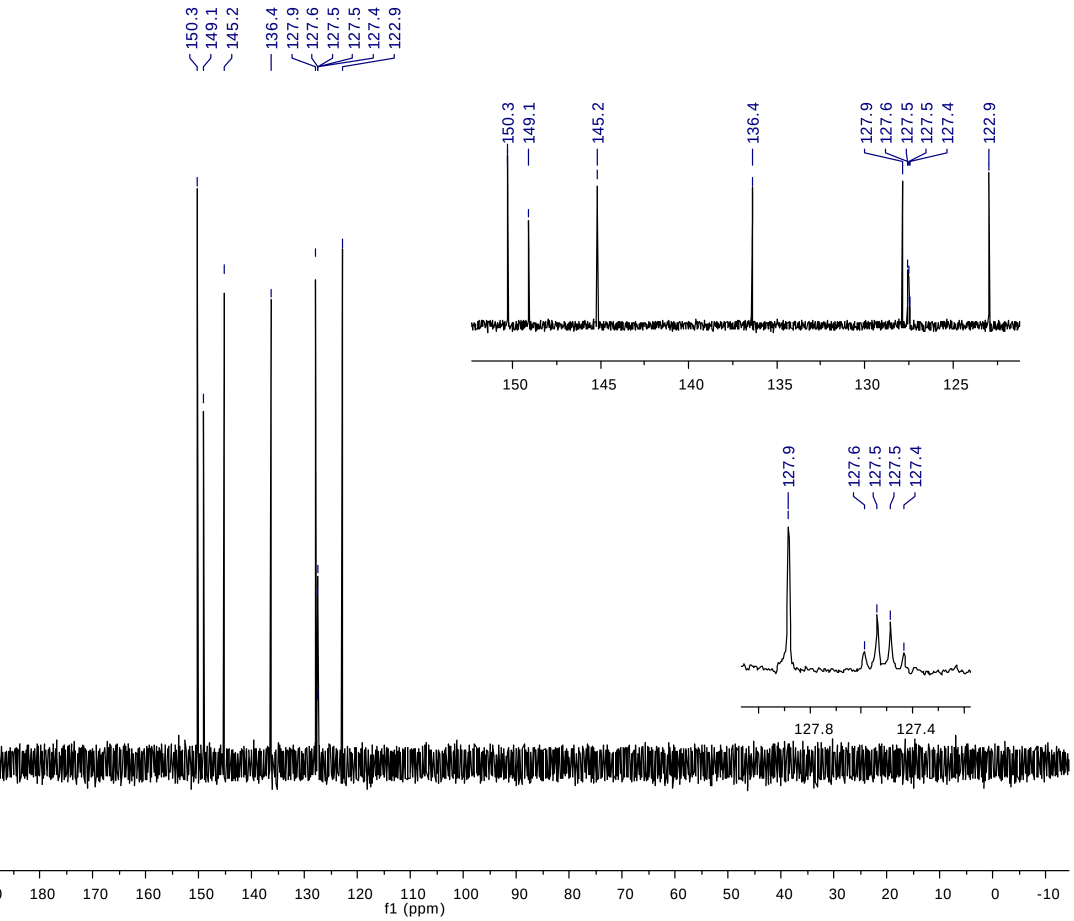

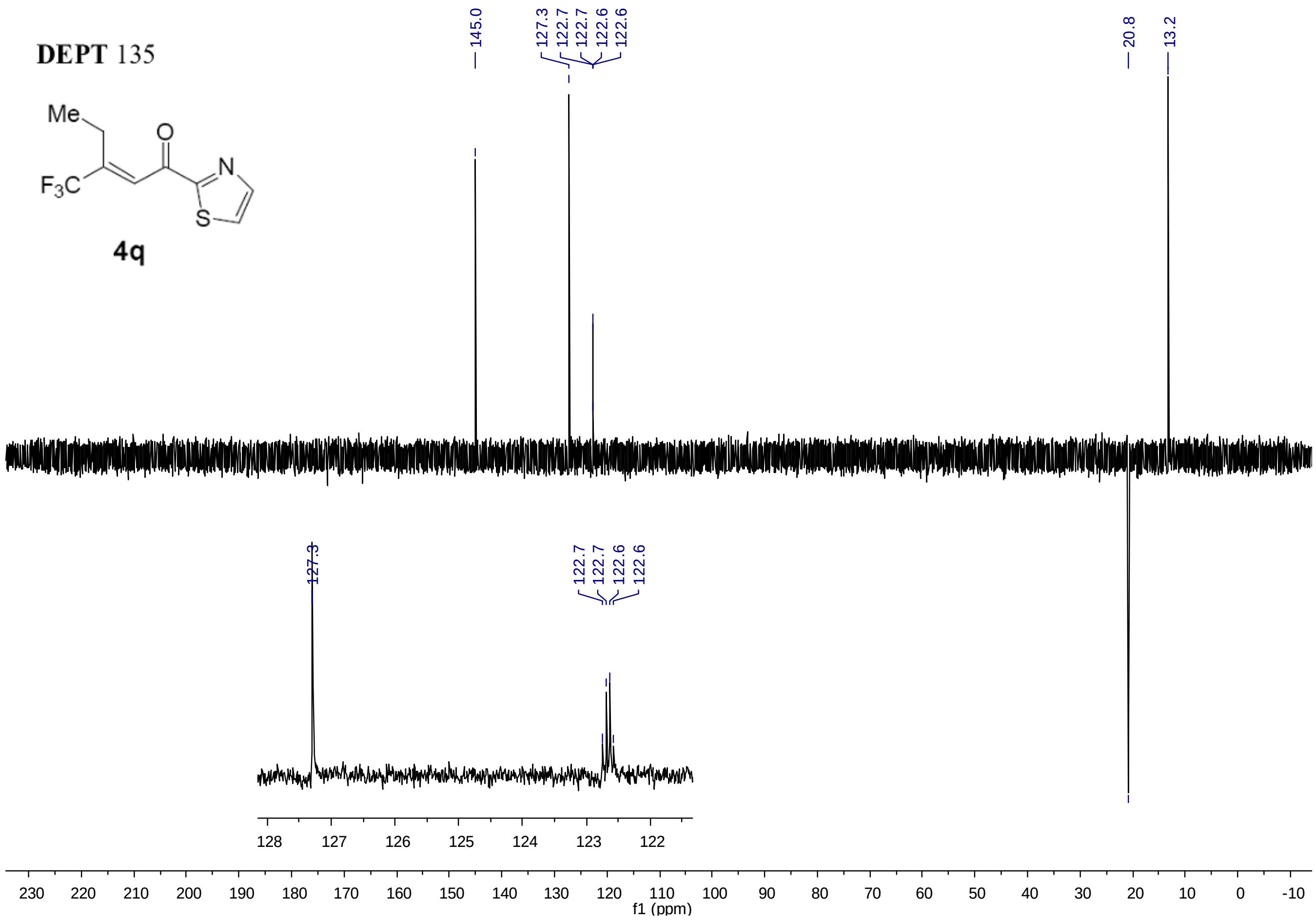
DEPT 135
Me
O
N
F3C
S
4q
145.0
127.3
122.7
122.7
122.6
122.6
20.8
13.2
f1 (ppm)

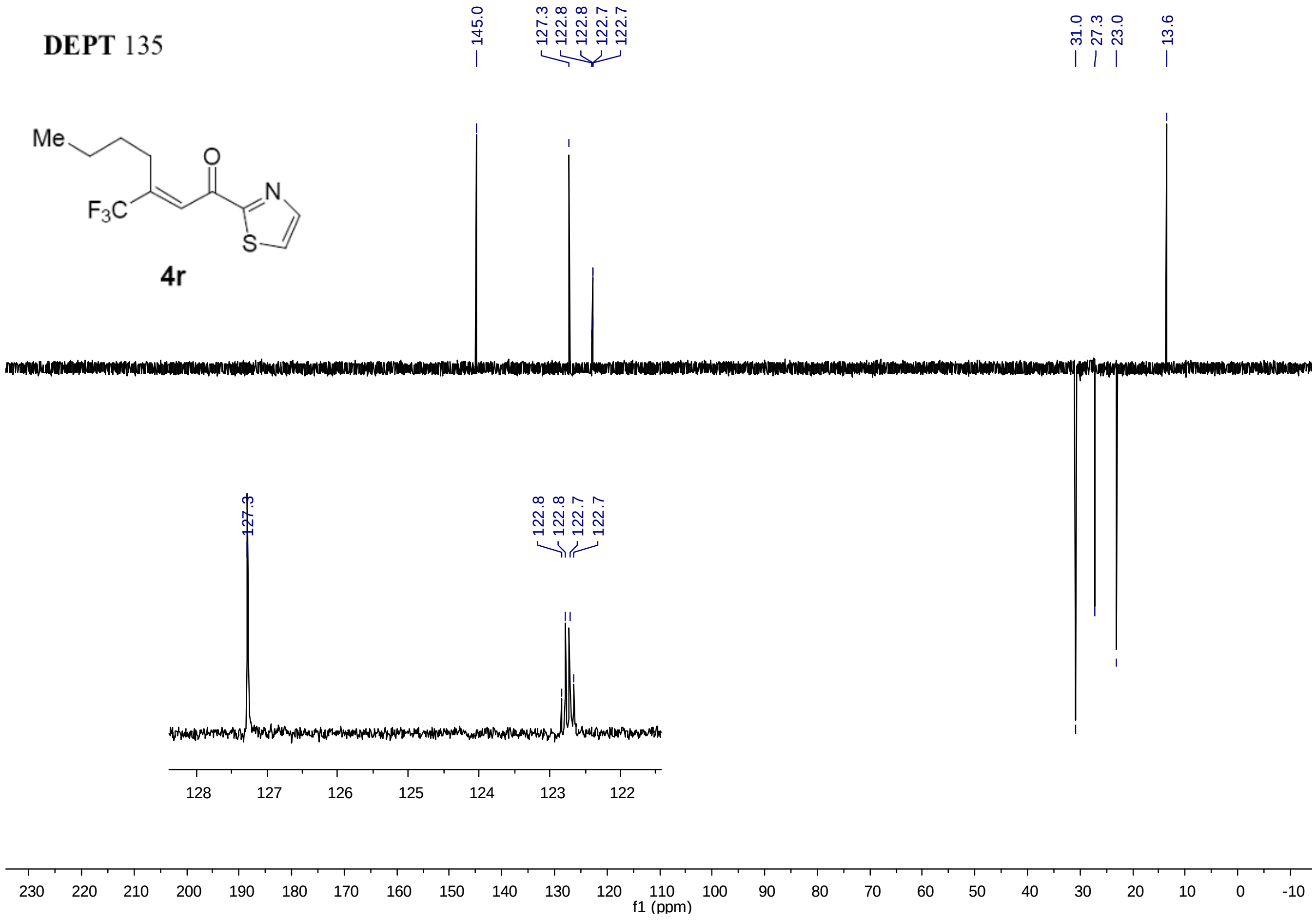
DEPT 135
Me
O
N
F3C
S
4r
145.0
127.3
122.8
122.8
122.7
122.7
31.0
27.3
23.0
13.6
f1 (ppm)

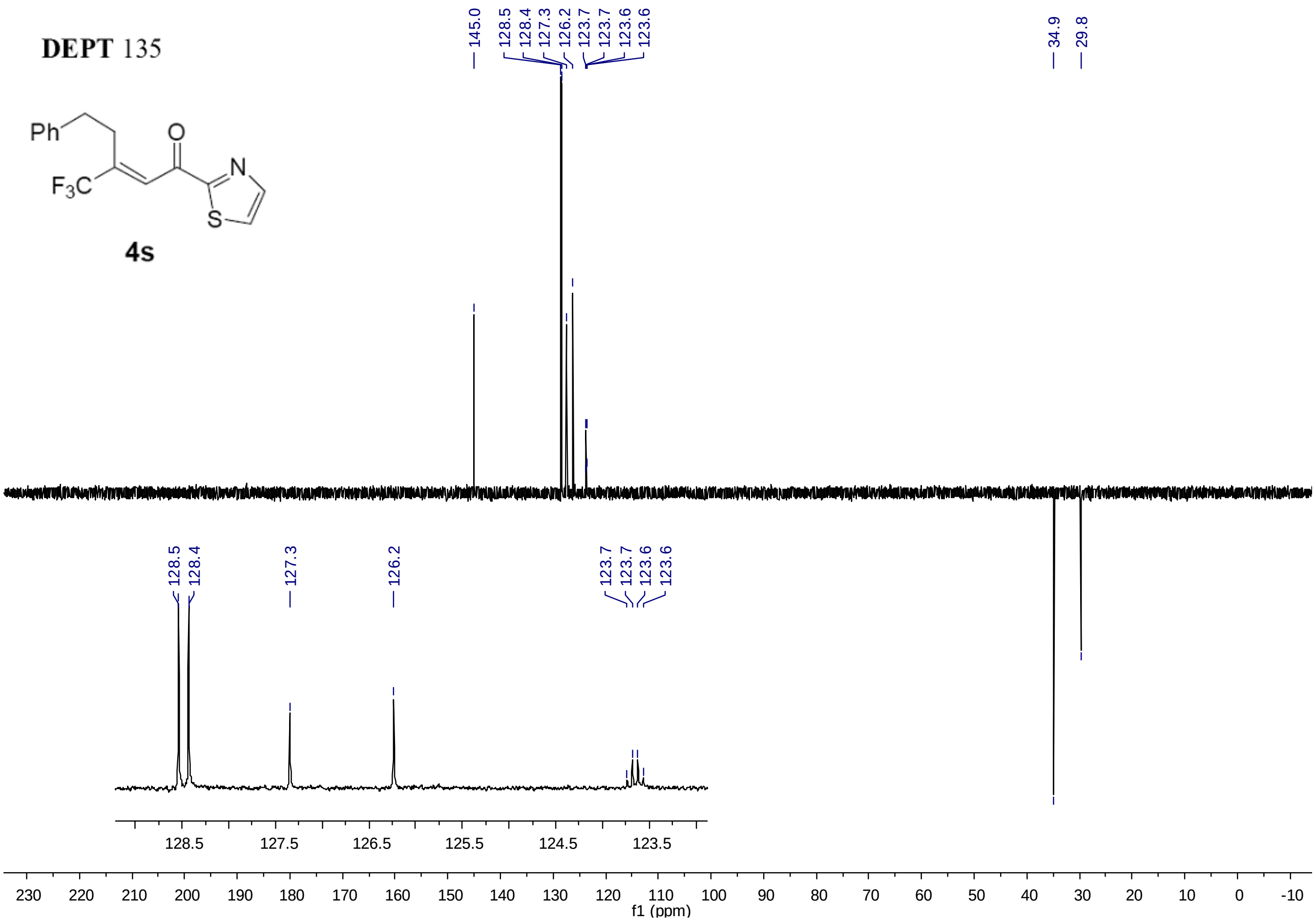
DEPT 135
Ph
O
N
F3C
S
4s
145.0
128.5
128.4
127.3
126.2
123.7
123.7
123.6
123.6
34.9
29.8
128.5
127.5
126.5
125.5
124.5
123.5
230 220 210 200 190 180 170 160 150 140 130 120 110 100 90 80 70 60 50 40 30 20 10 0 -10
f1 (ppm)

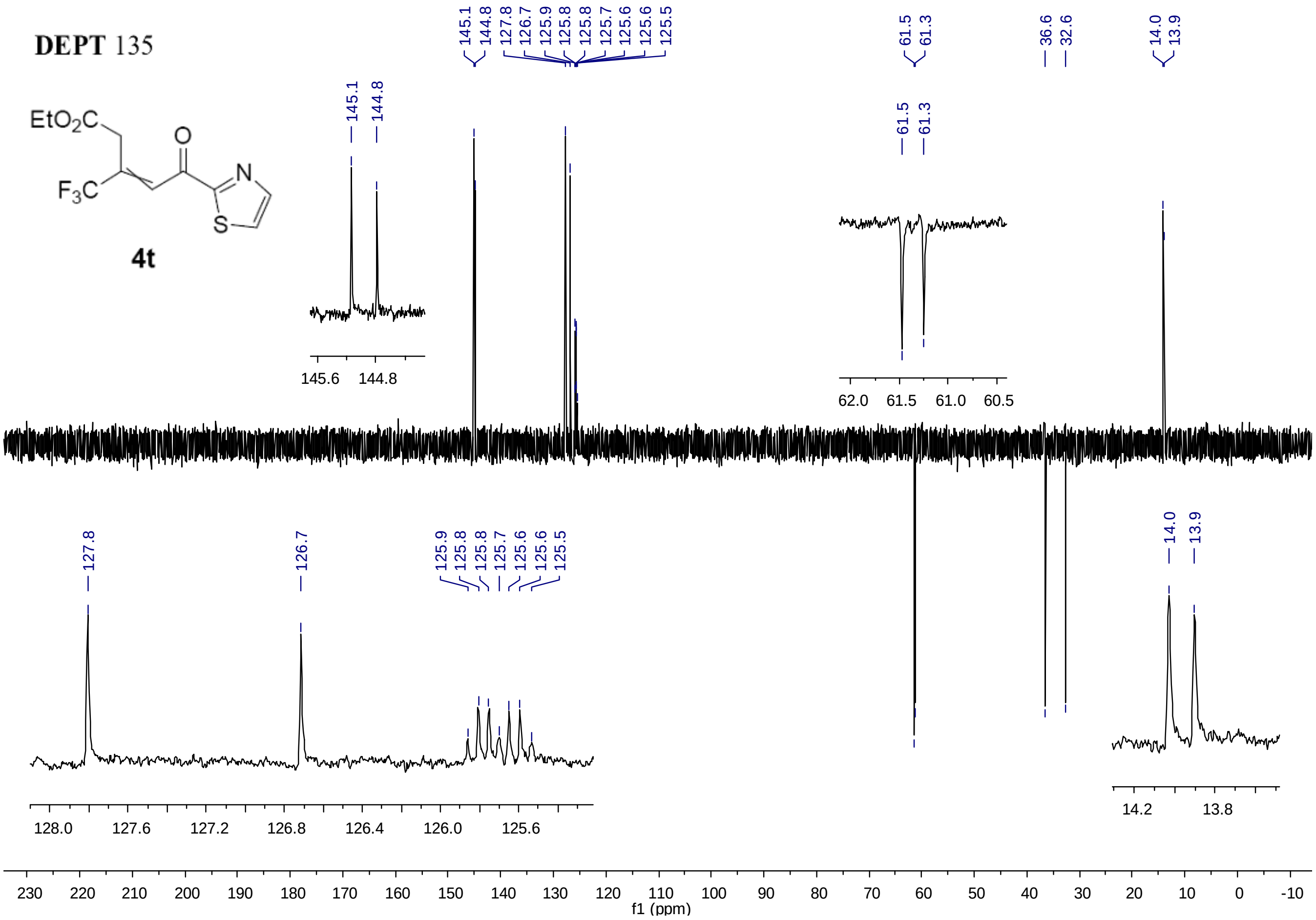
DEPT 135
EtO2C
O
N
F3C
S
4t
145.1
144.8
127.8
126.7
125.9
125.8
125.8
125.7
125.6
125.6
125.5
61.5
61.3
36.6
32.6
14.0
13.9
145.6
144.8
62.0
61.5
61.0
60.5
128.0
127.6
127.2
126.8
126.4
126.0
125.6
14.2
13.8
230 220 210 200 190 180 170 160 150 140 130 120 110 100 90 80 70 60 50 40 30 20 10 0 -10
f1 (ppm)

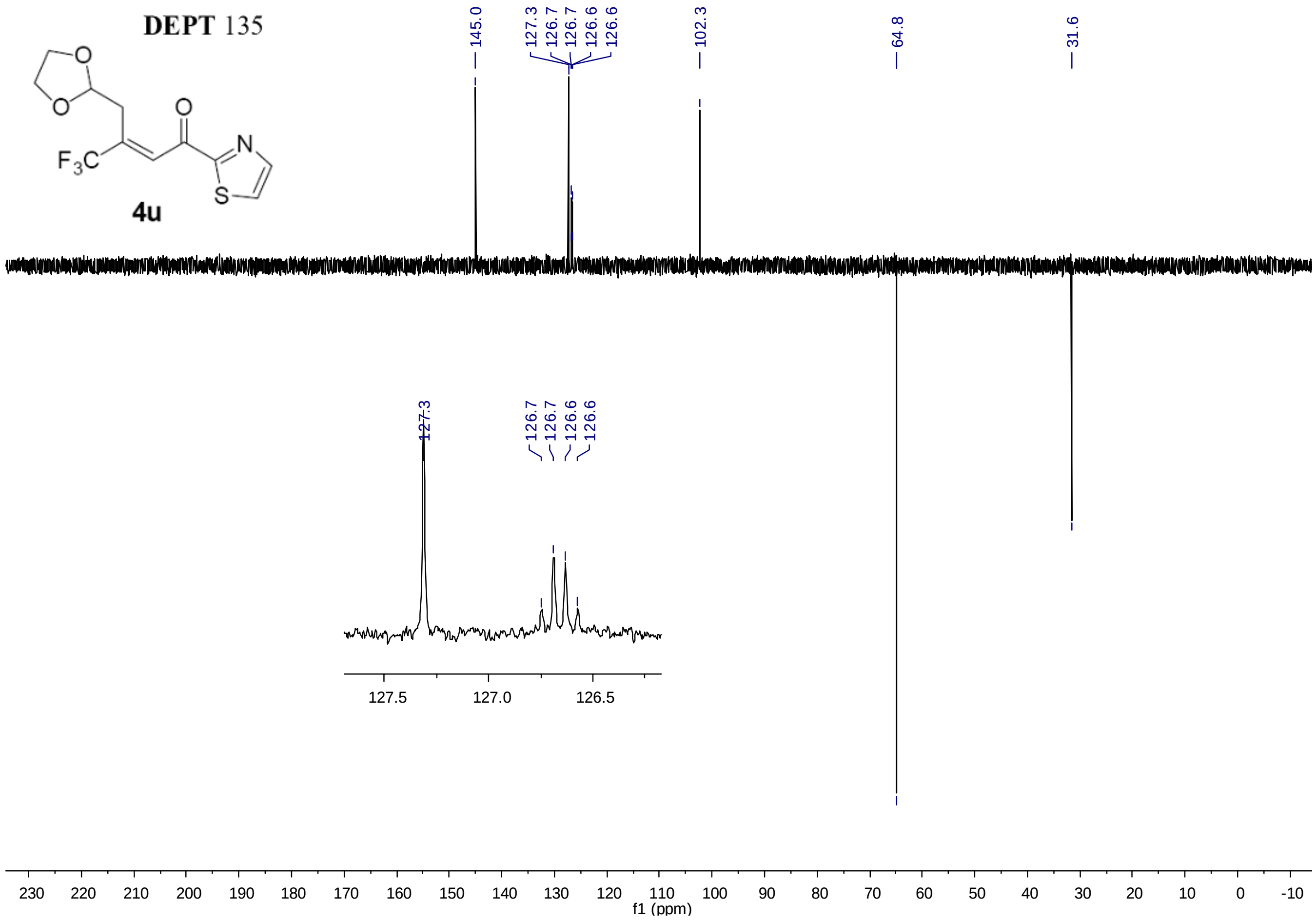
DEPT 135
F3C
O
N
S
4u
145.0
127.3
126.7
126.7
126.6
126.6
102.3
64.8
31.6
127.5
127.0
126.5
230 220 210 200 190 180 170 160 150 140 130 120 110 100 90 80 70 60 50 40 30 20 10 0 -10
f1 (ppm)

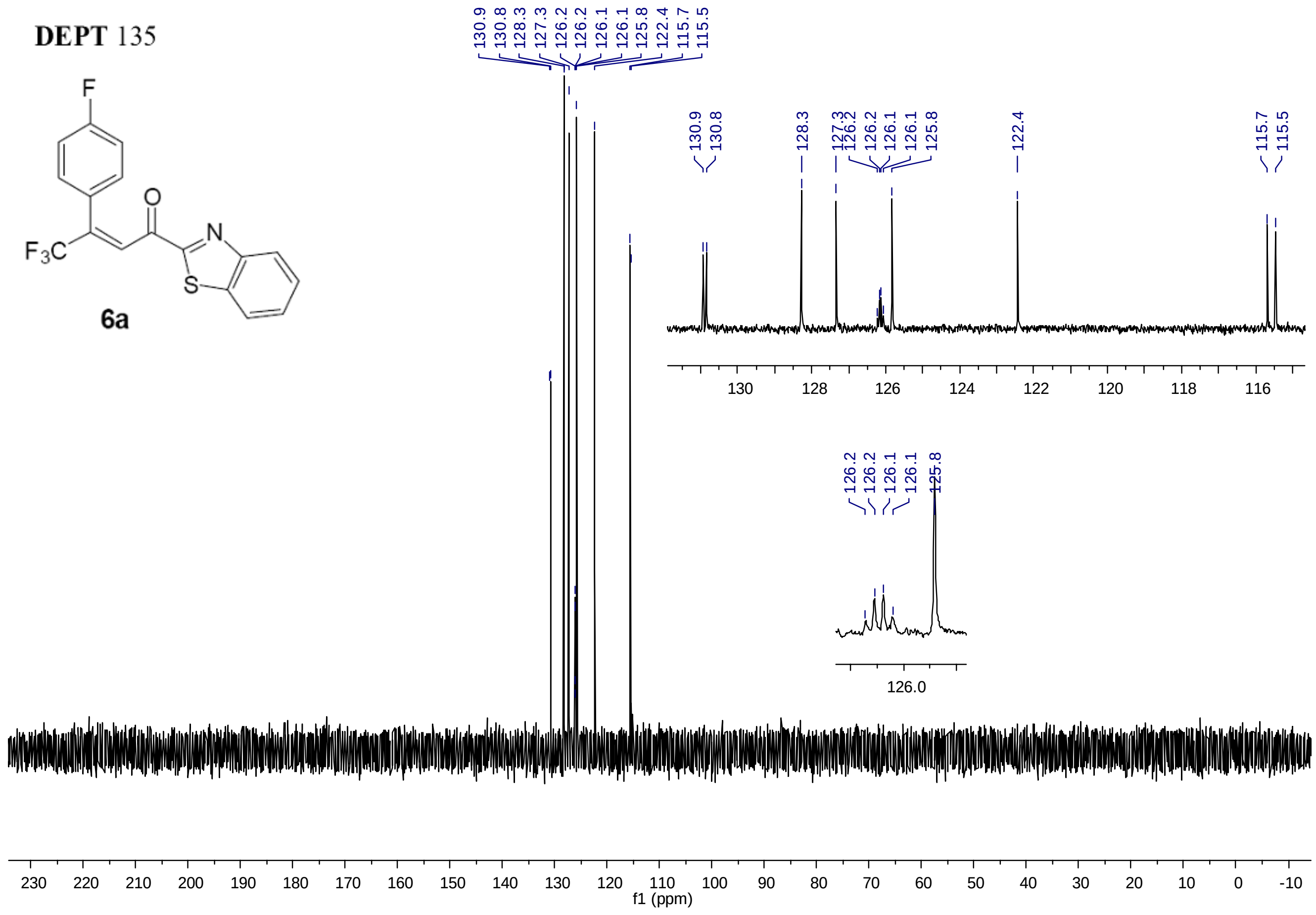
DEPT 135
F
F3C
O
N
S
6a
130.9
130.8
128.3
127.3
126.2
126.2
126.1
126.1
125.8
122.4
115.7
115.5
130 128 126 124 122 120 118 116
126.0
230 220 210 200 190 180 170 160 150 140 130 120 110 100 90 80 70 60 50 40 30 20 10 0 -10
f1 (ppm)

DEPT 135

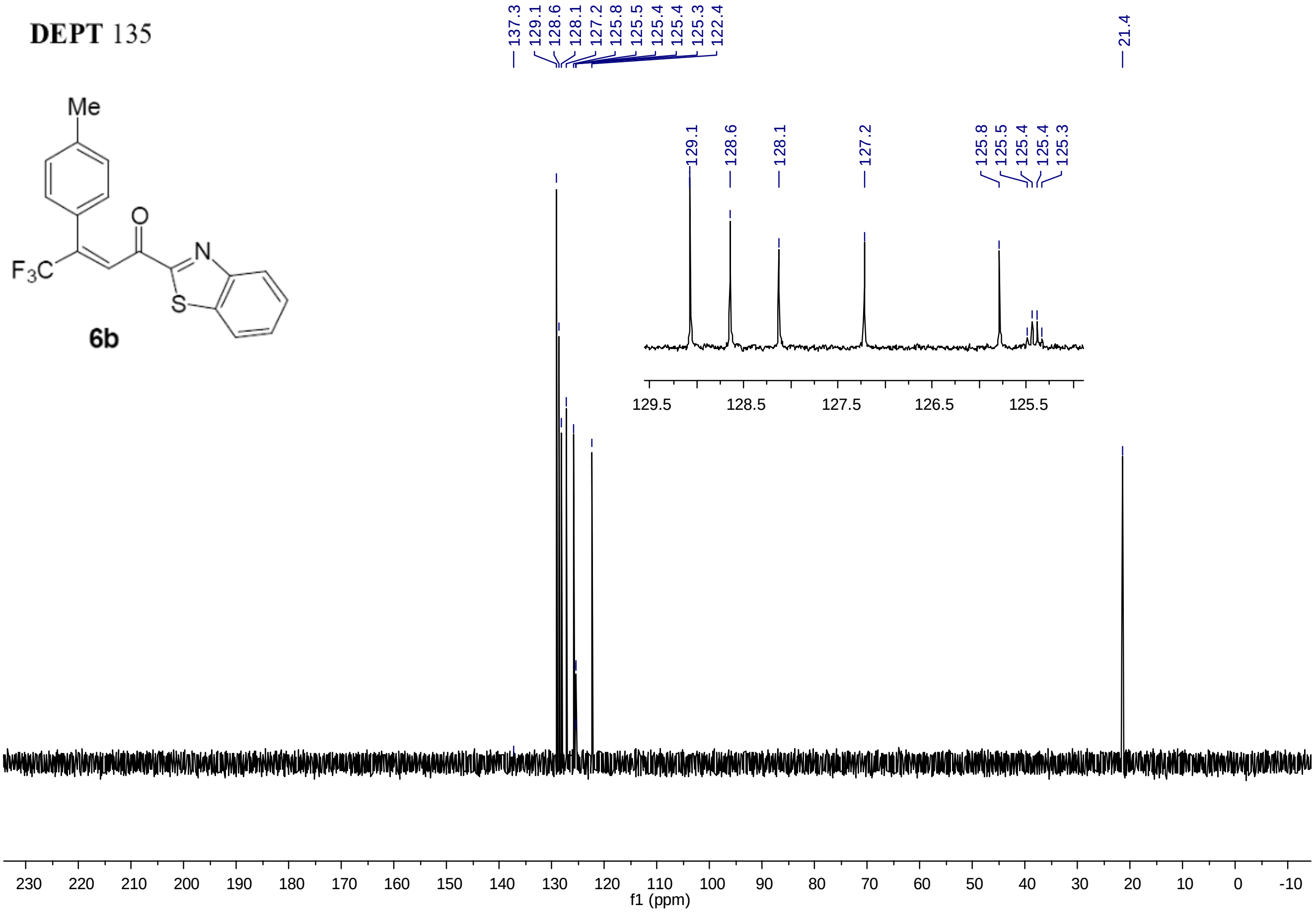

DEPT 135

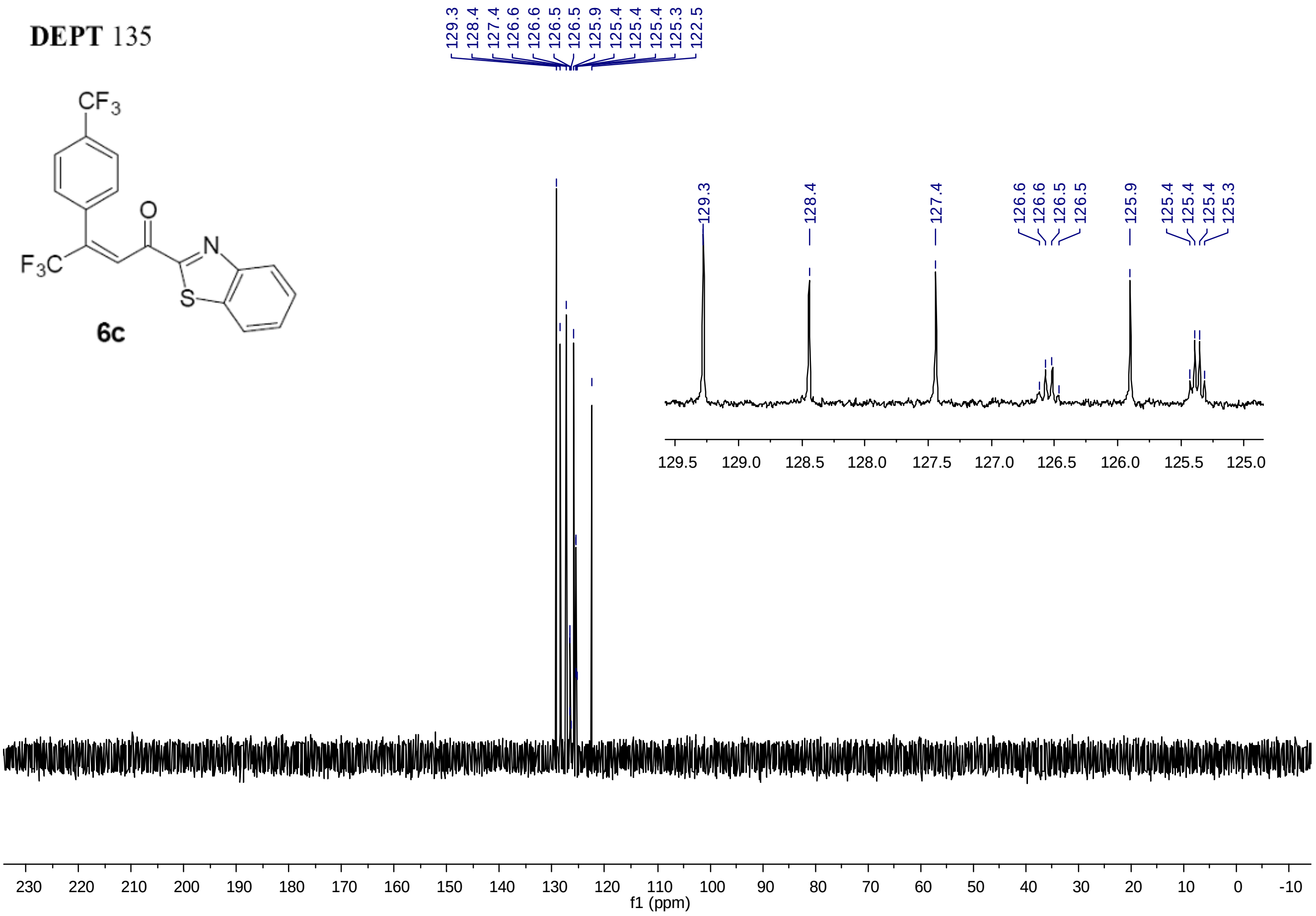

DEPT 135

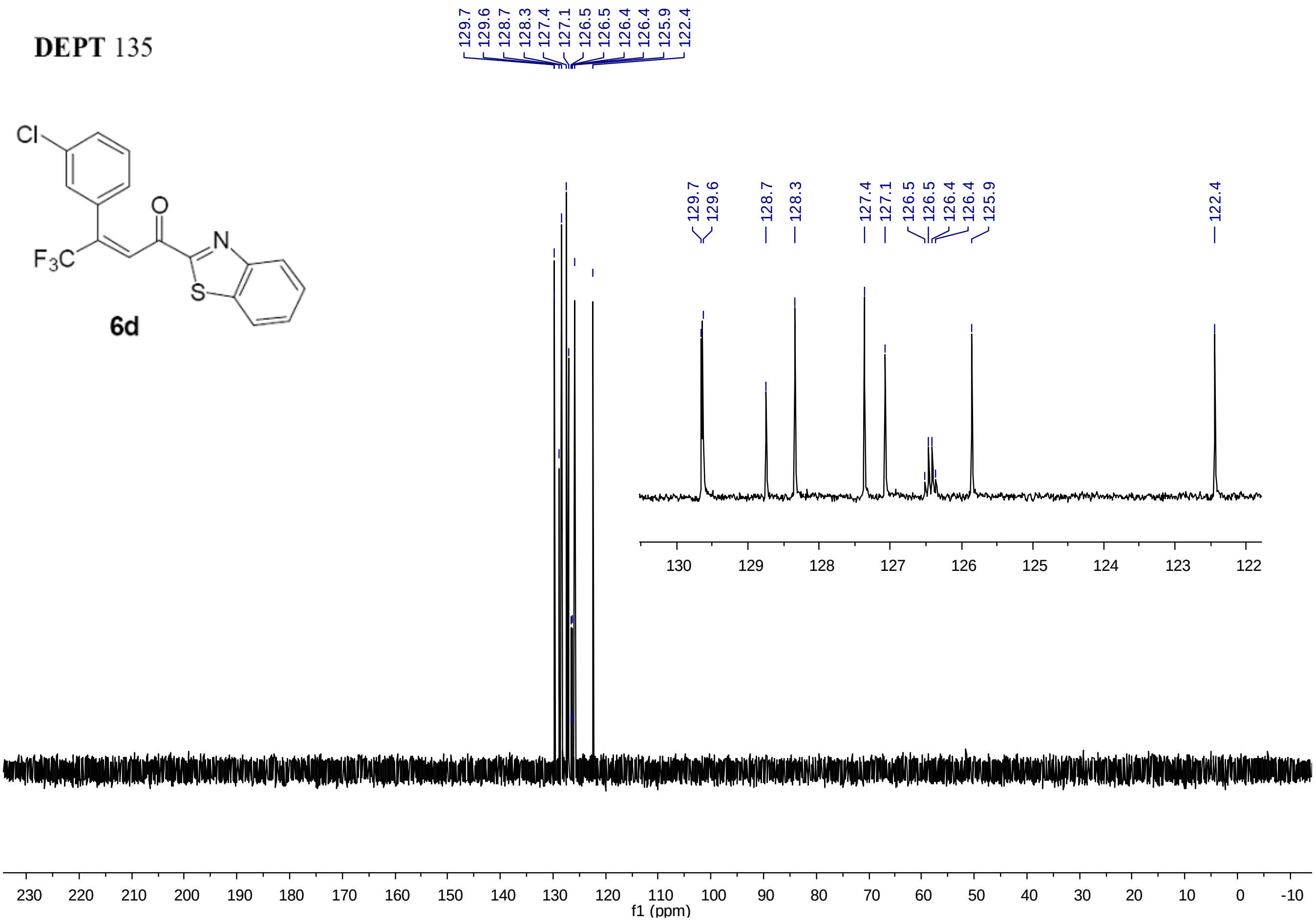

DEPT 135

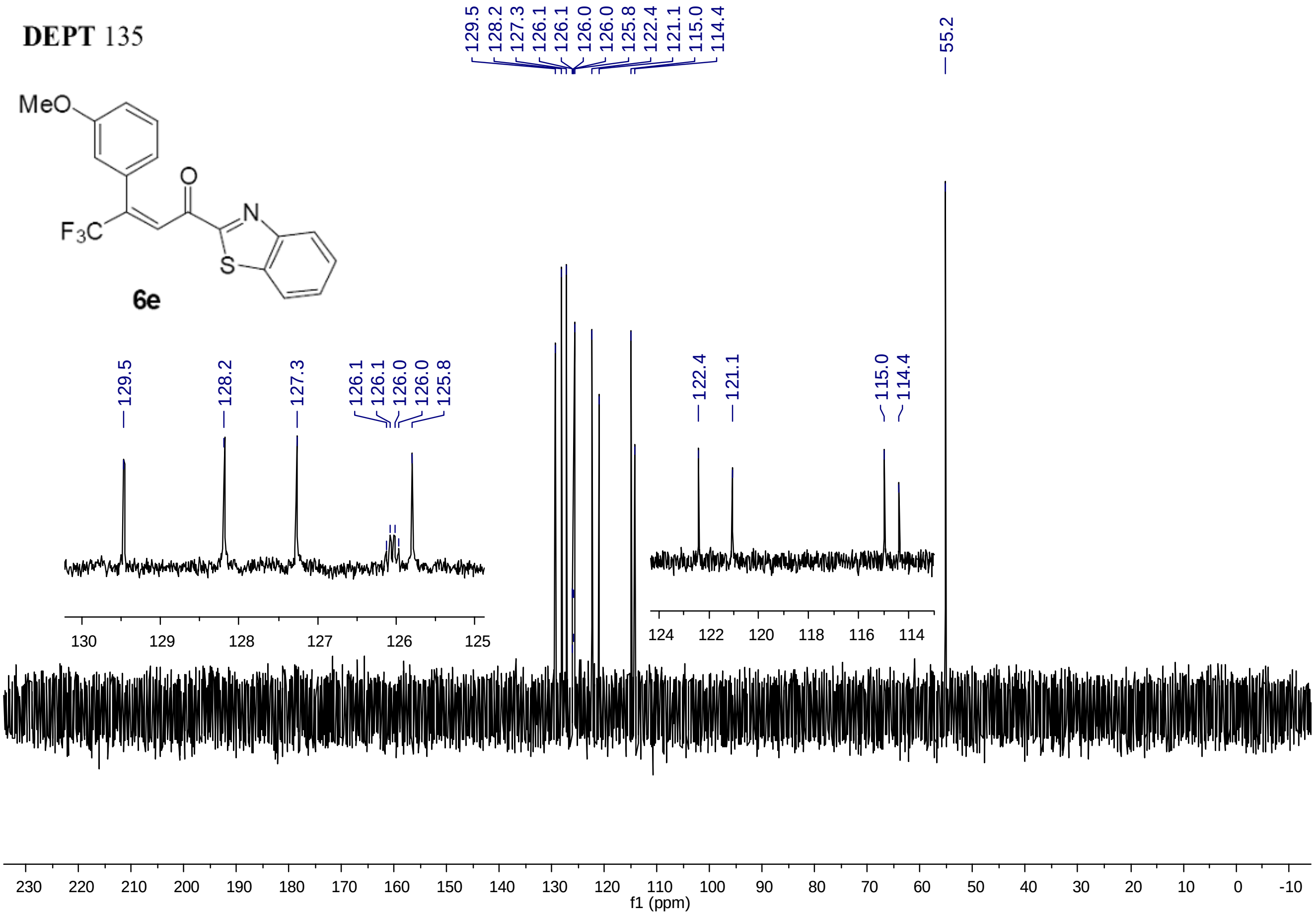

DEPT 135

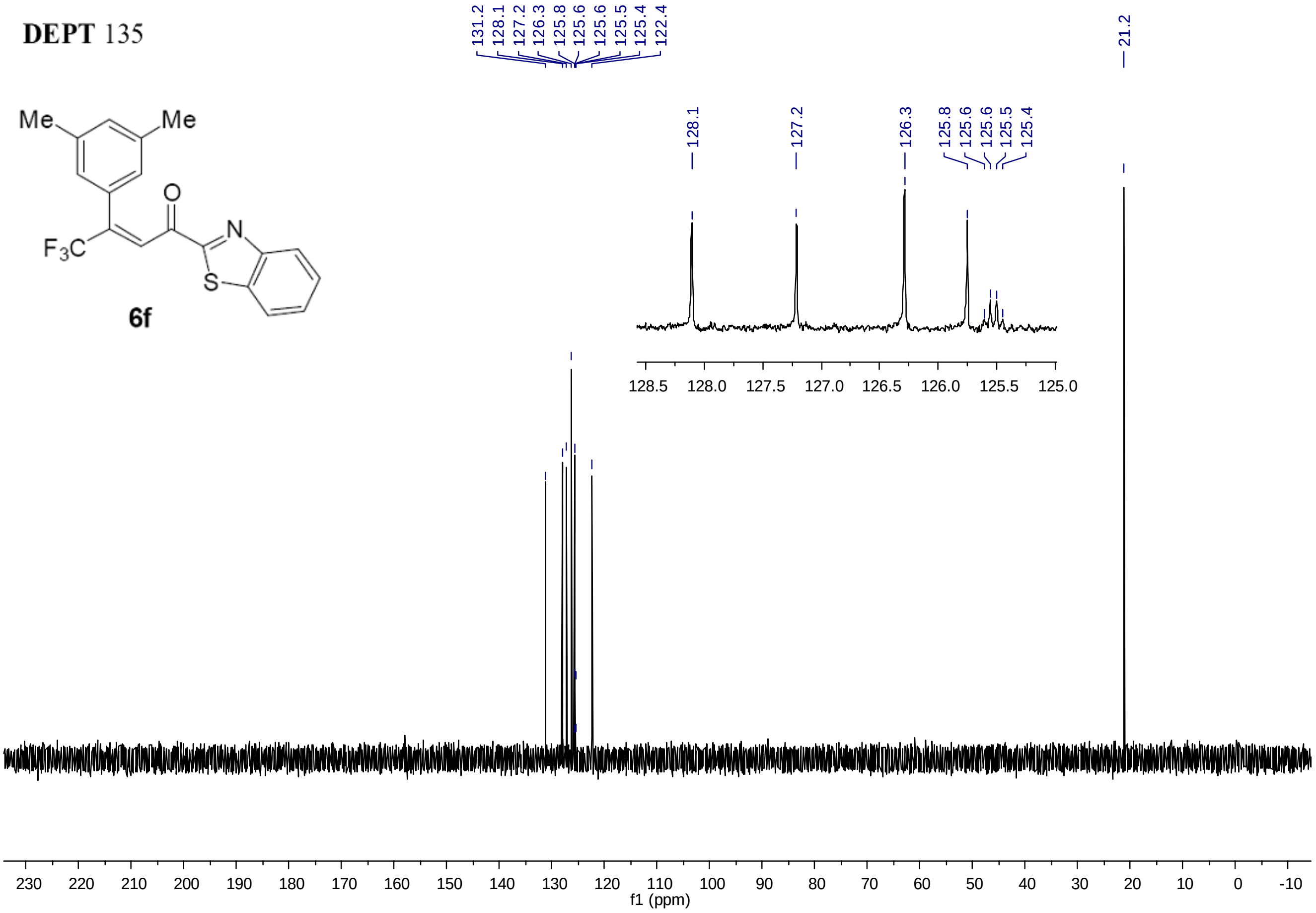

6f

DEPT 135

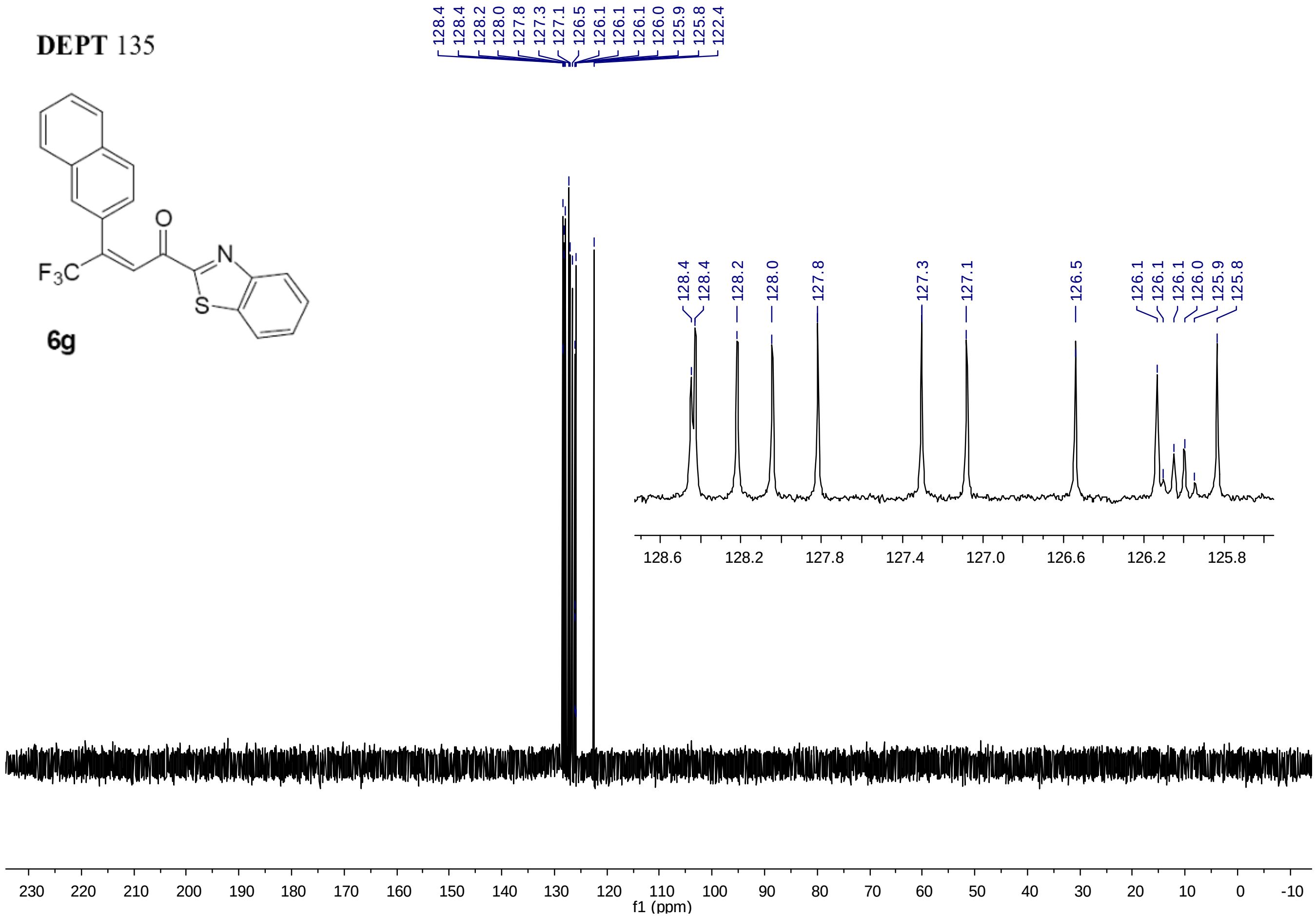

6g

DEPT 135

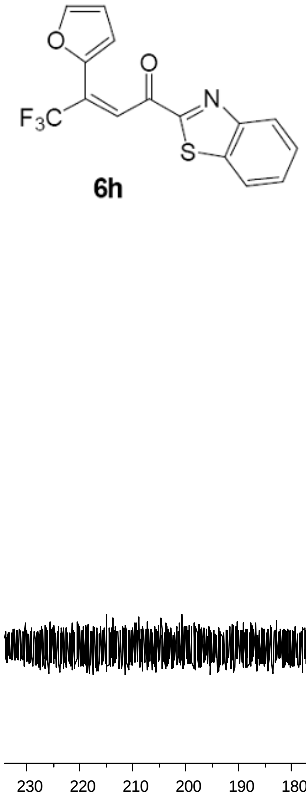

6h

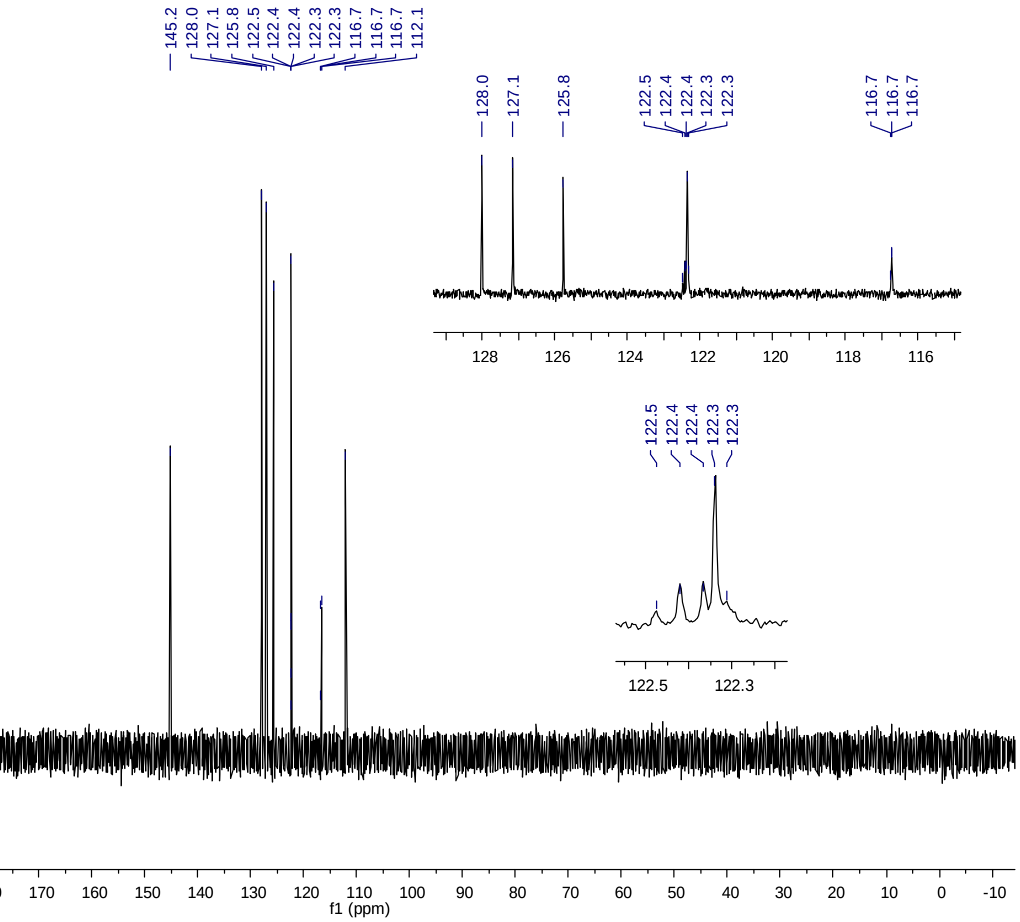

DEPT 135

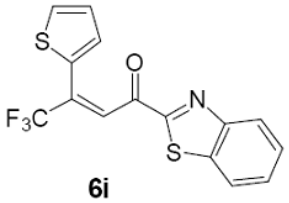

6i

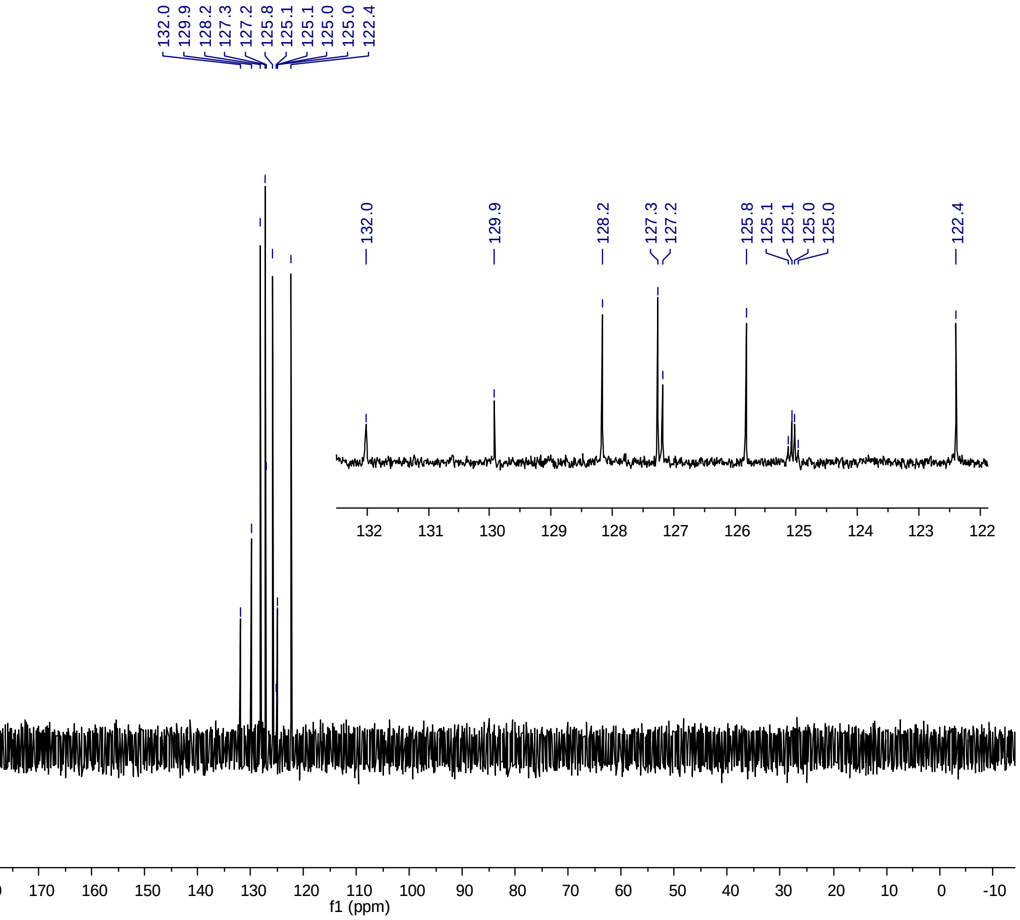

DEPT 135

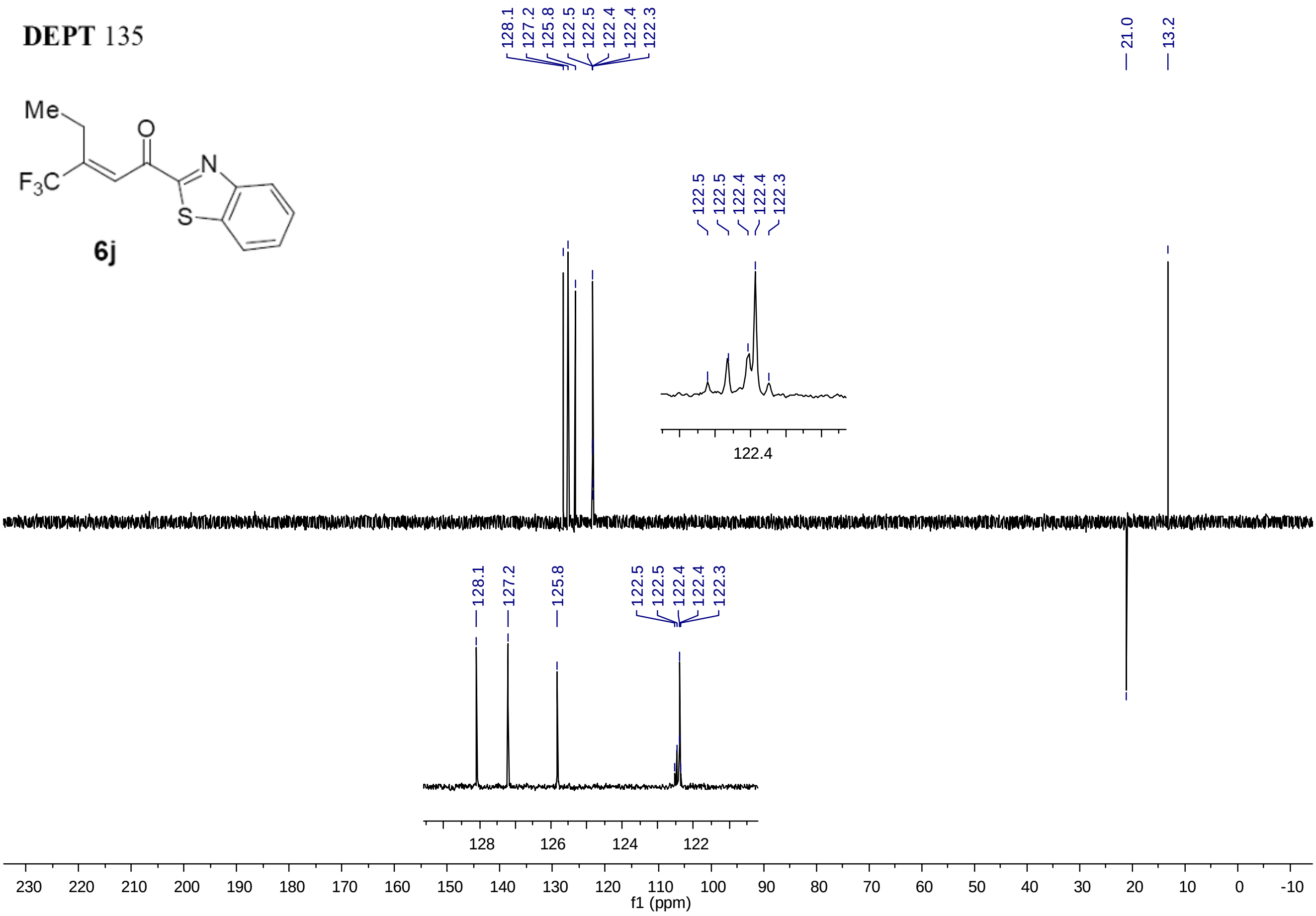

6j

DEPT 135

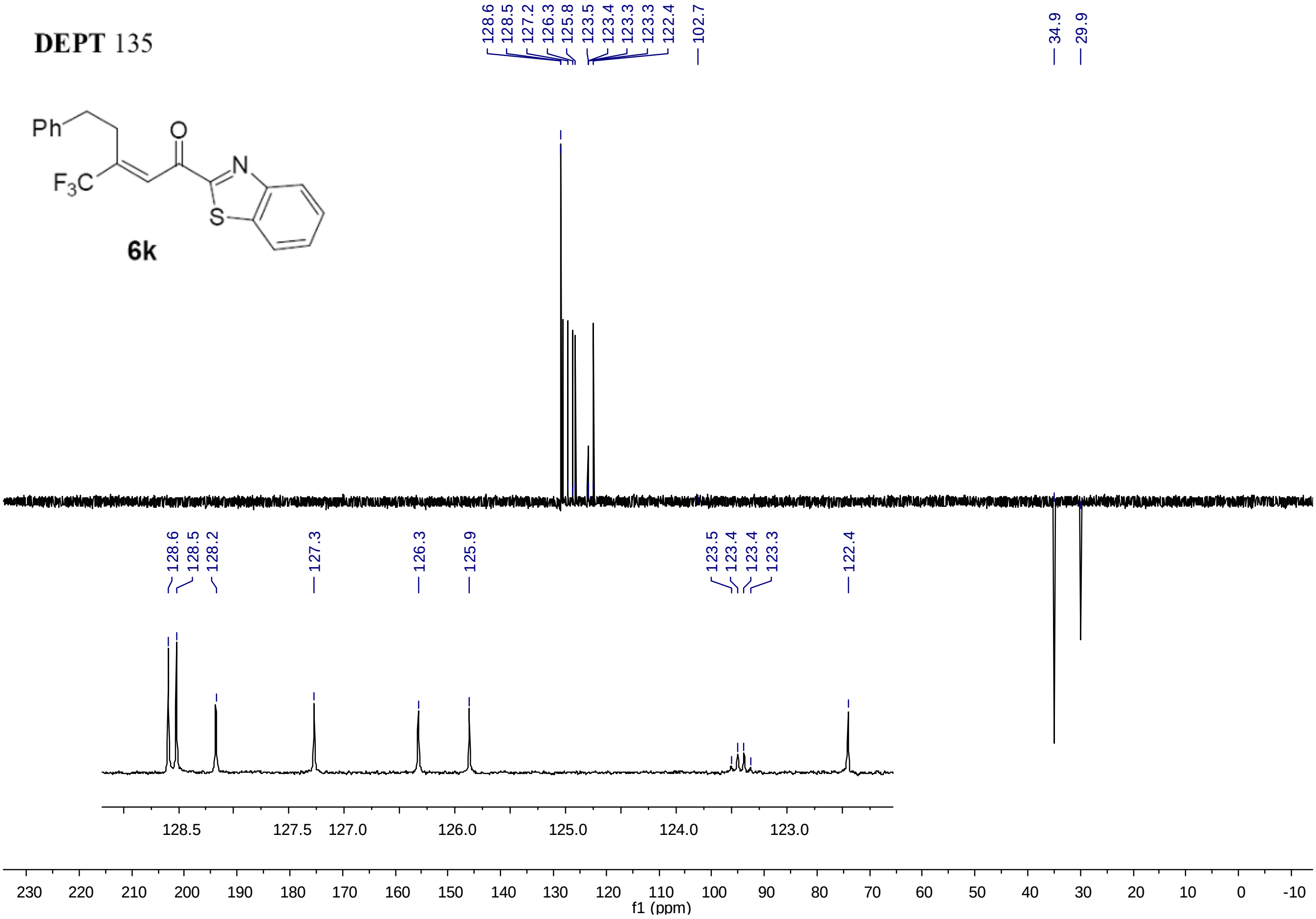

6k

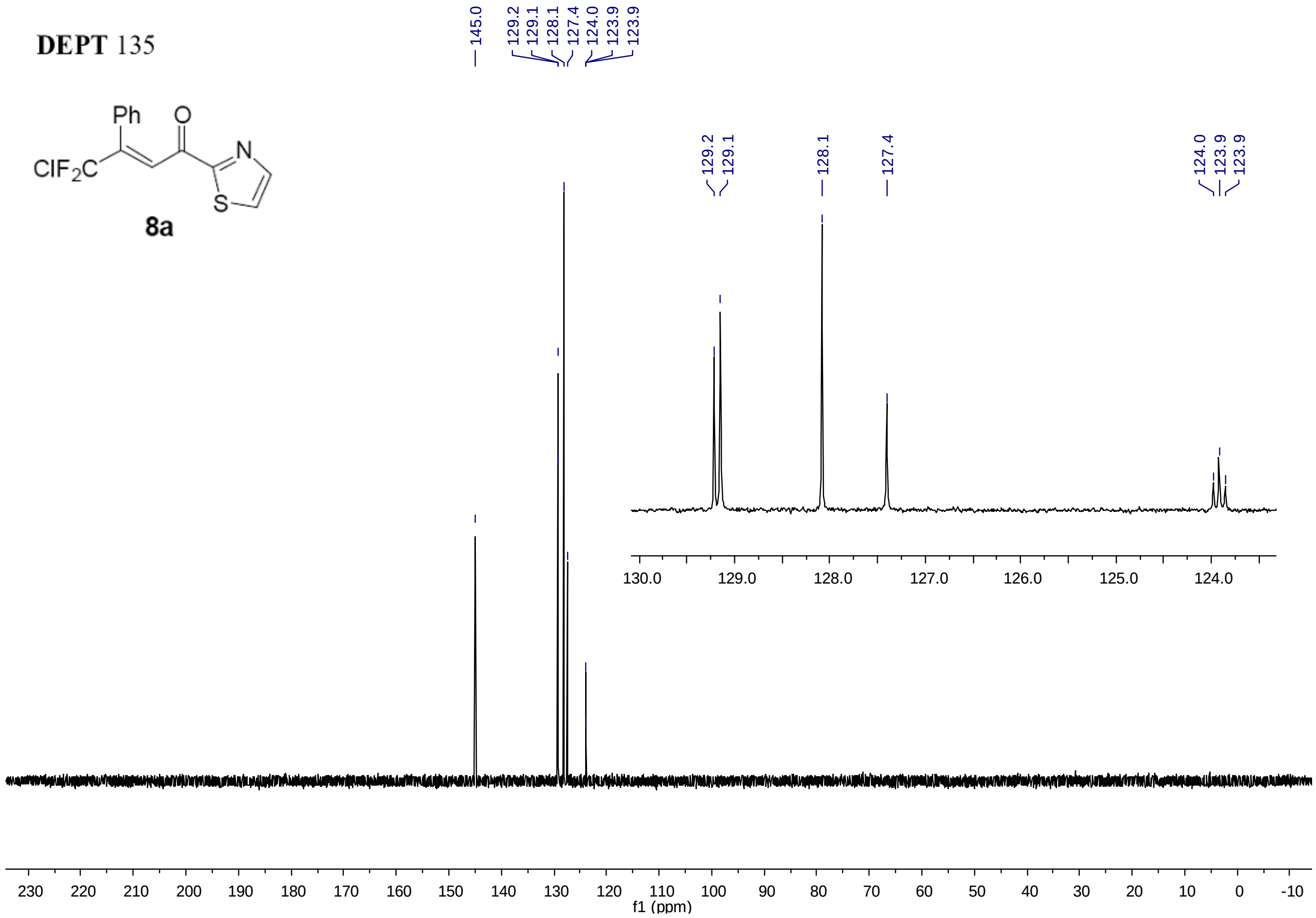
DEPT 135
Ph
O
ClF2C
N
S
8a
145.0
129.2
129.1
128.1
127.4
124.0
123.9
123.9
130.0 129.0 128.0 127.0 126.0 125.0 124.0
230 220 210 200 190 180 170 160 150 140 130 120 110 100 90 80 70 60 50 40 30 20 10 0 -10
f1 (ppm)

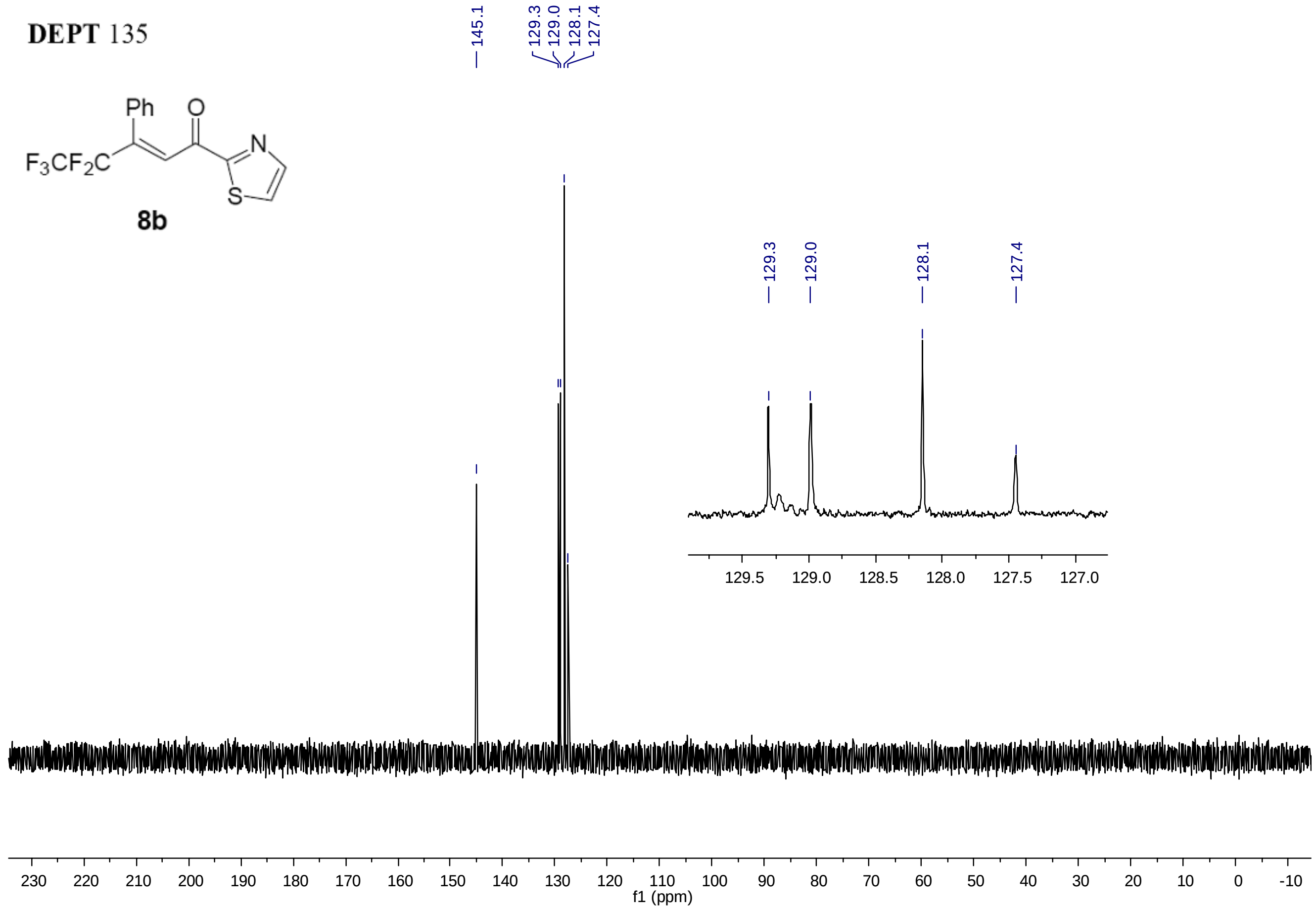
DEPT 135
Ph
O
F3CF2C
N
S
8b
145.1
129.3
129.0
128.1
127.4
129.5 129.0 128.5 128.0 127.5 127.0
230 220 210 200 190 180 170 160 150 140 130 120 110 100 90 80 70 60 50 40 30 20 10 0 -10
f1 (ppm)

Copies of ^{19}F NMR spectra - malonate adducts 3b-3l, 5a-5k, 5m-5u, 7a-7k, 9a, 9b

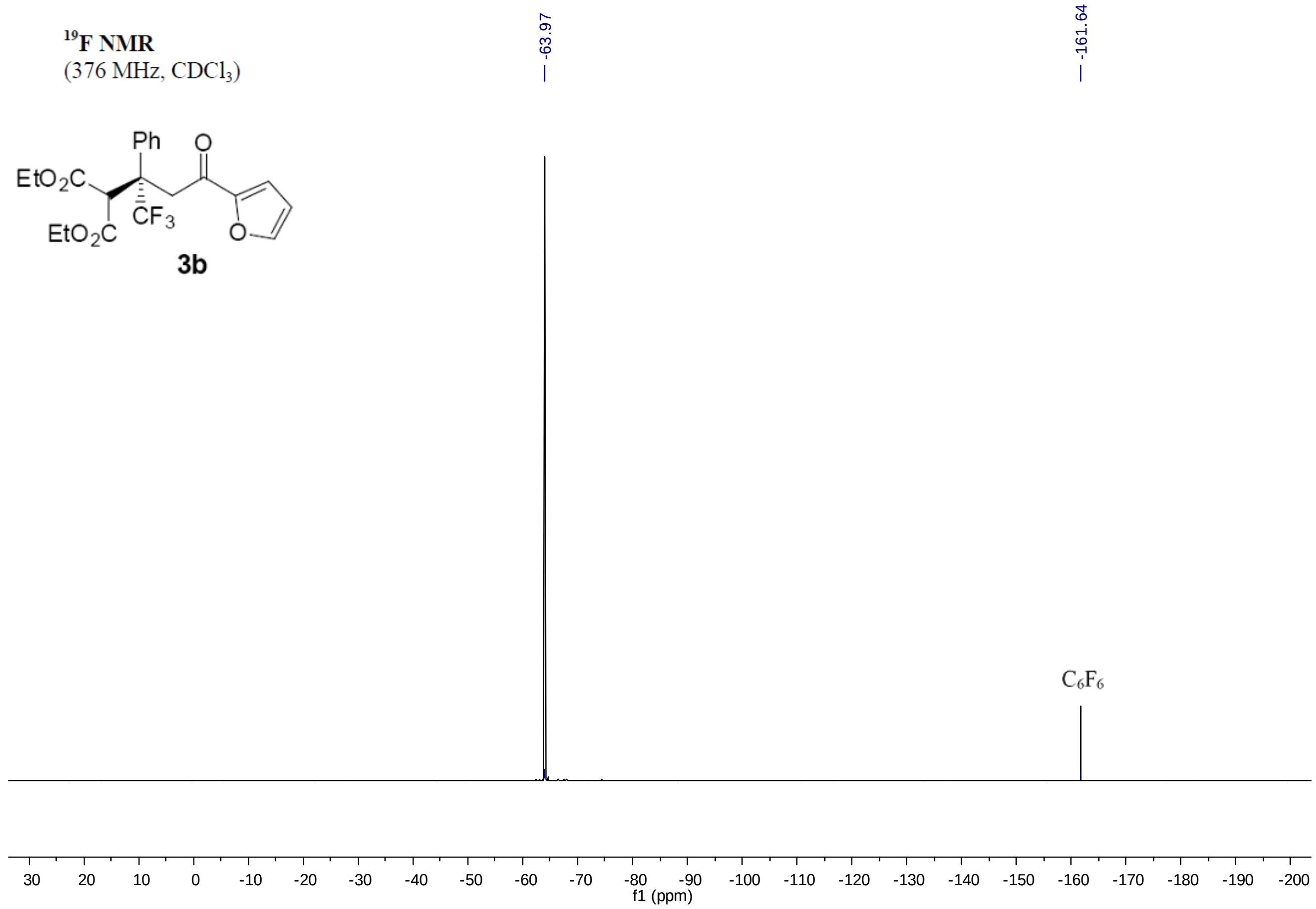

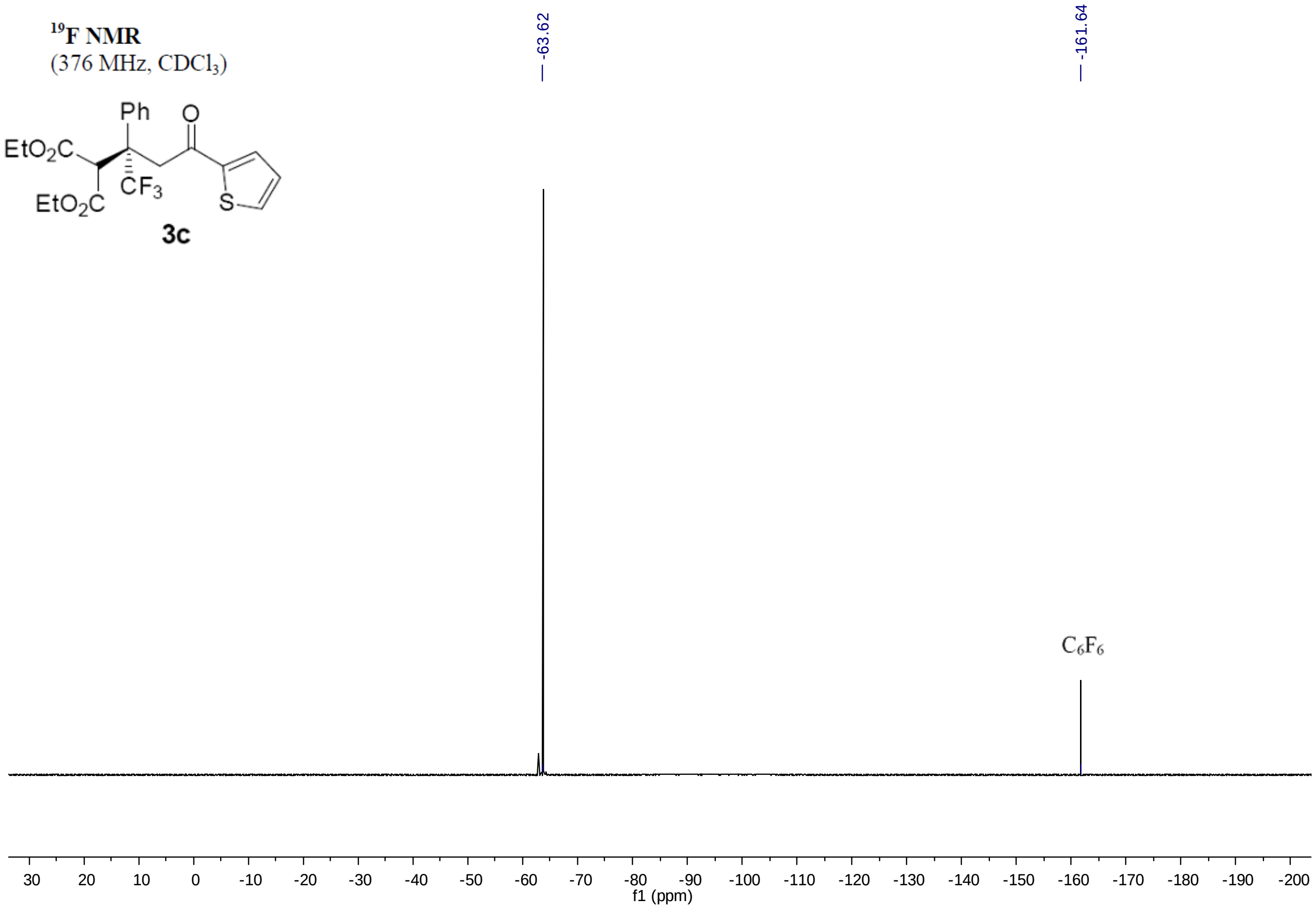

^{19}F NMR
(376 MHz, $CDCl_3$)

3d

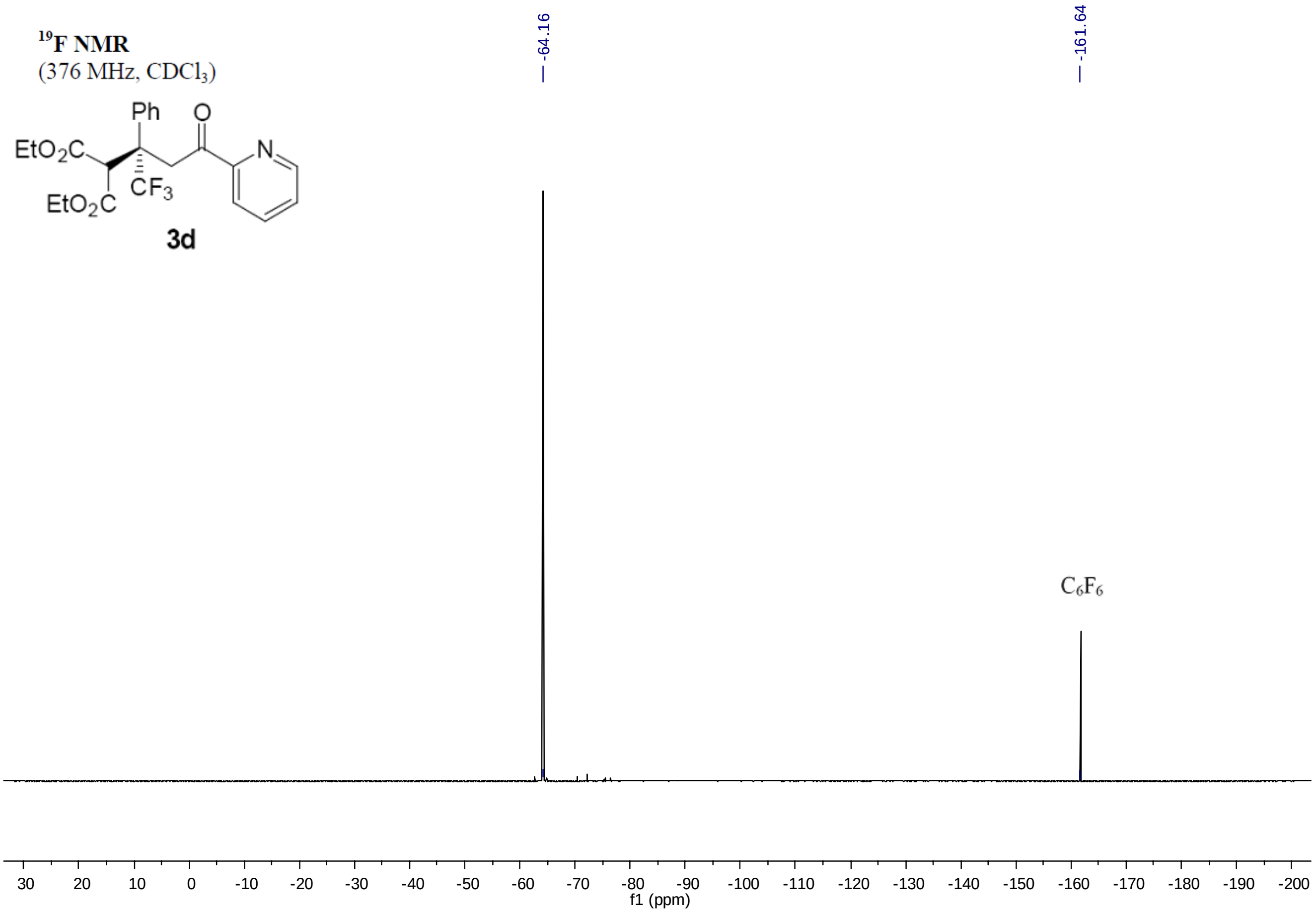

^{19}F NMR
(376 MHz, $CDCl_3$)

3e

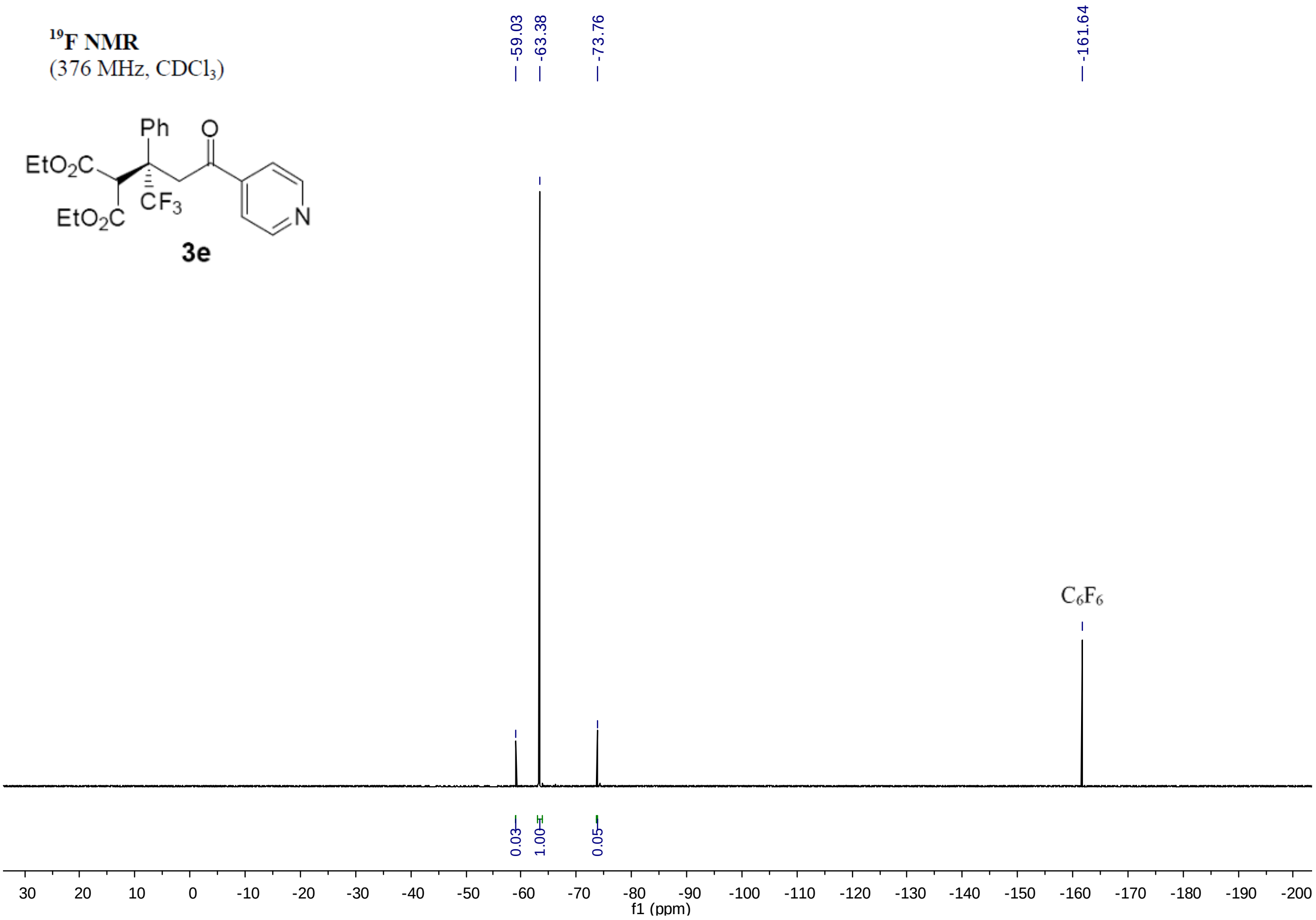

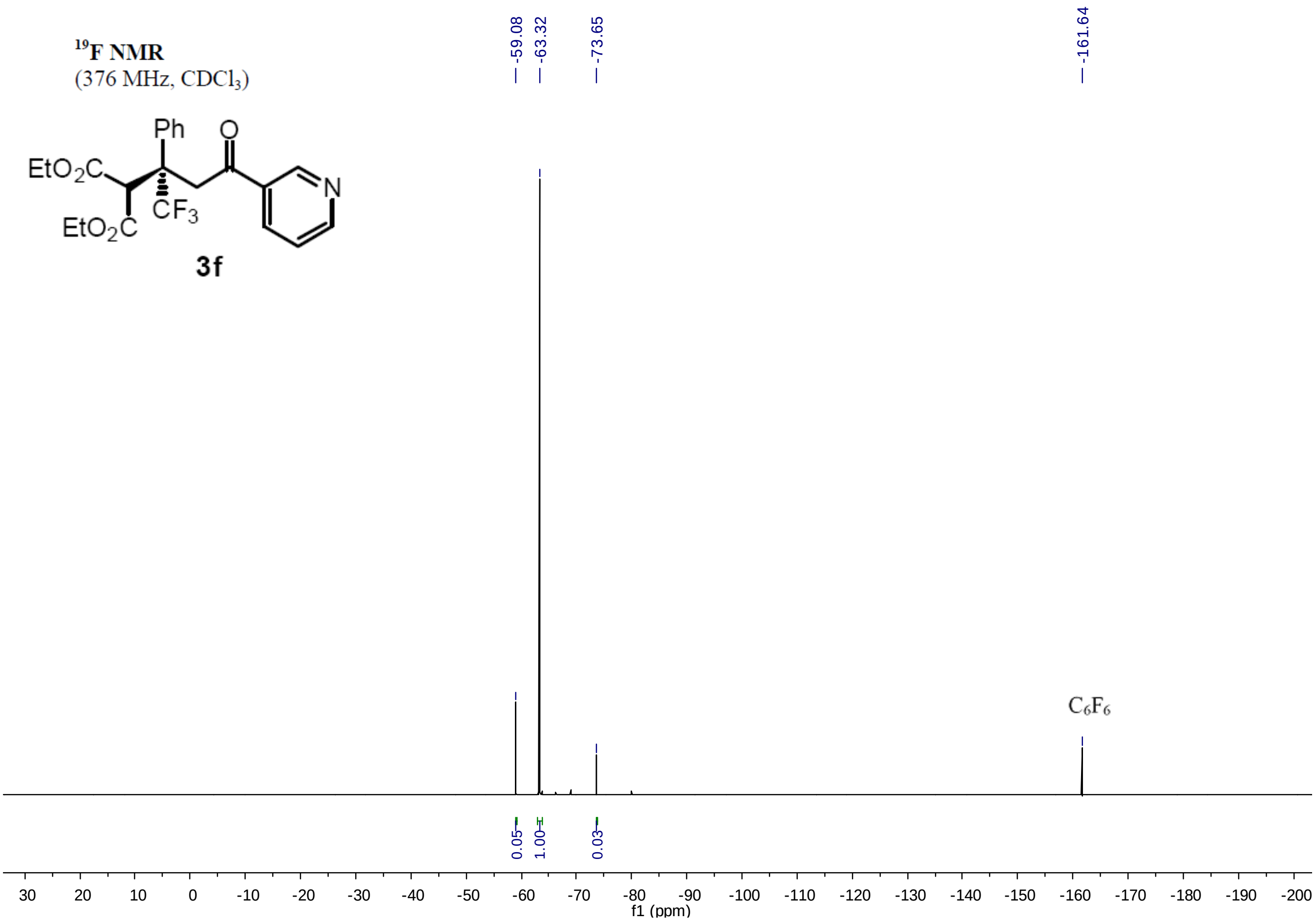

^{19}F NMR
(376 MHz, CDCl$_3$)
Ph
O
EtO$_2$C
N
CF$_3$
EtO$_2$C
3f
-59.08
-63.32
-73.65
-161.64
C$_6$F$_6$
0.05
1.00
0.03
30 20 10 0 -10 -20 -30 -40 -50 -60 -70 -80 -90 -100 -110 -120 -130 -140 -150 -160 -170 -180 -190 -200
f1 (ppm)

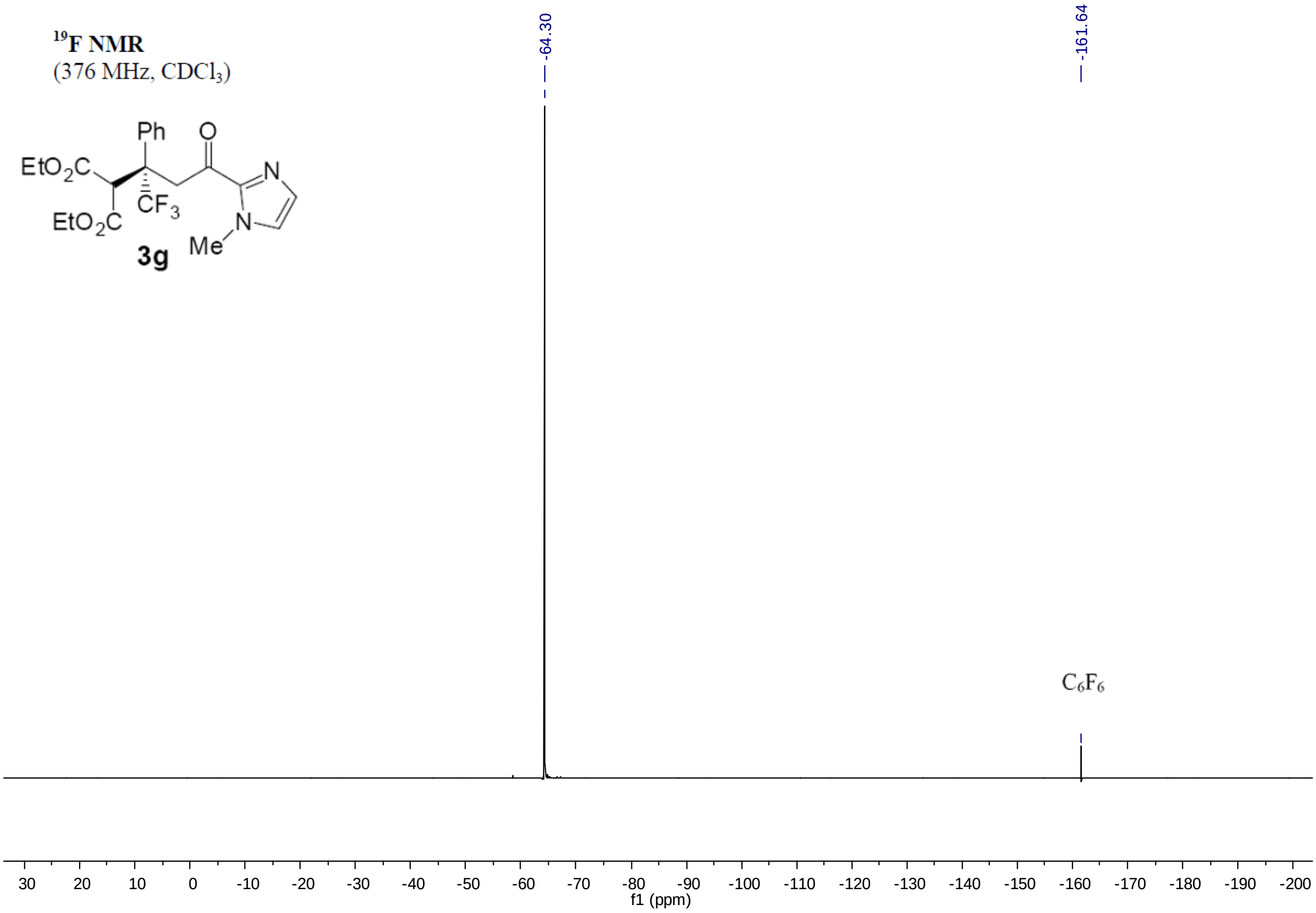

^{19}F NMR
(376 MHz, CDCl$_3$)
Ph
O
EtO$_2$C
N
CF$_3$
EtO$_2$C
N
3g
Me
-64.30
-161.64
C$_6$F$_6$
30 20 10 0 -10 -20 -30 -40 -50 -60 -70 -80 -90 -100 -110 -120 -130 -140 -150 -160 -170 -180 -190 -200
f1 (ppm)

^{19}F NMR
(376 MHz, $CDCl_3$)

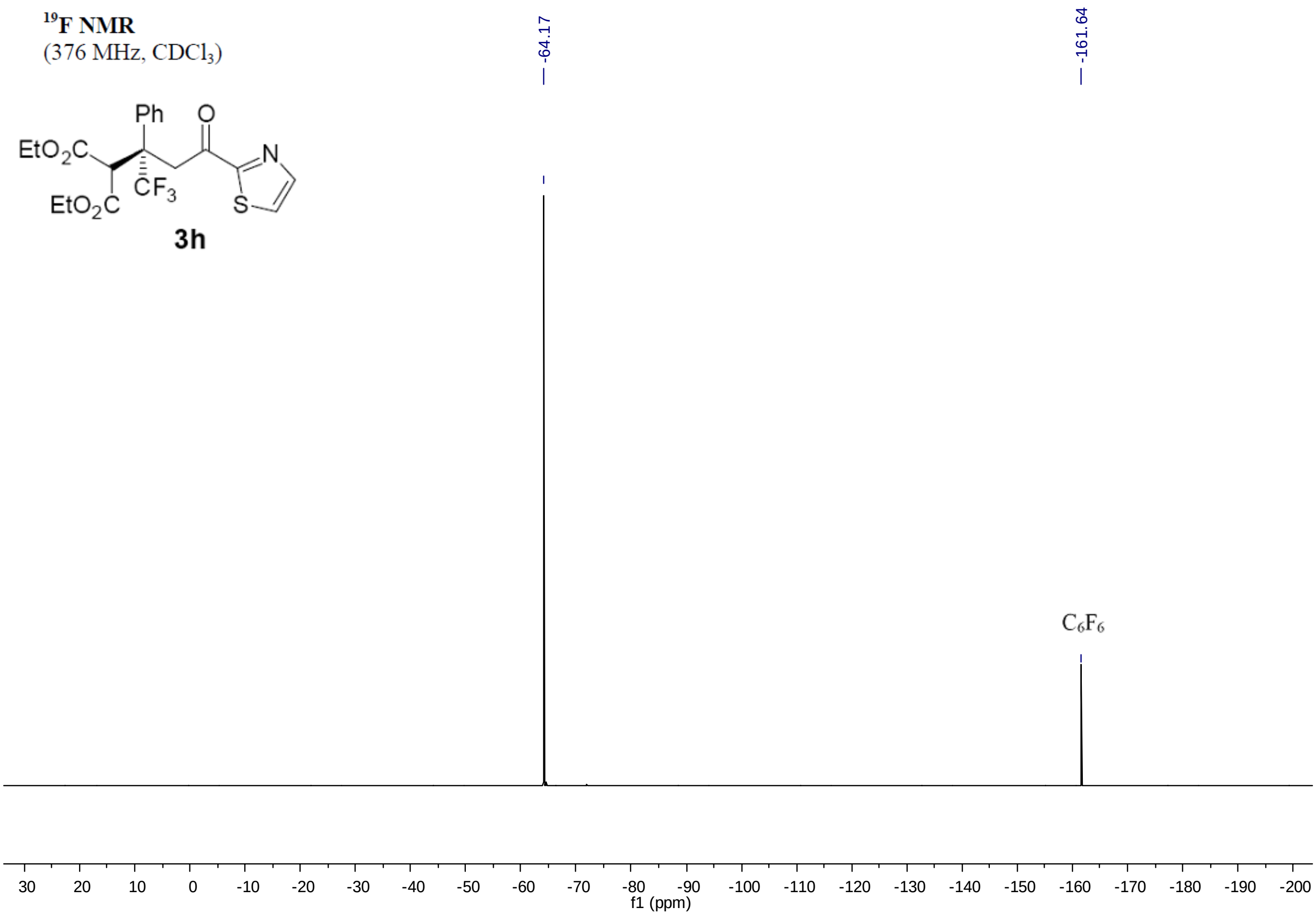

^{19}F NMR
(376 MHz, $CDCl_3$)

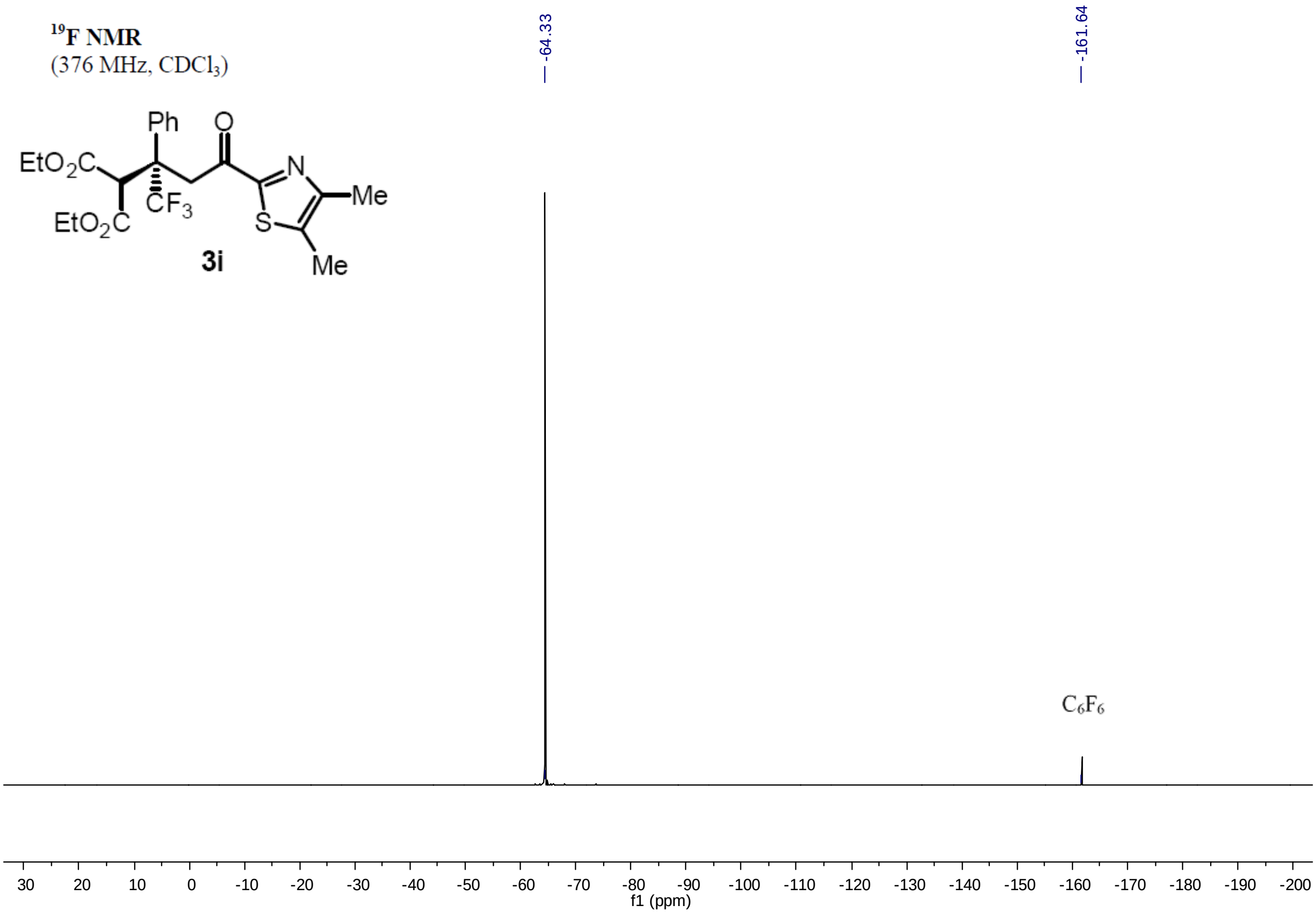

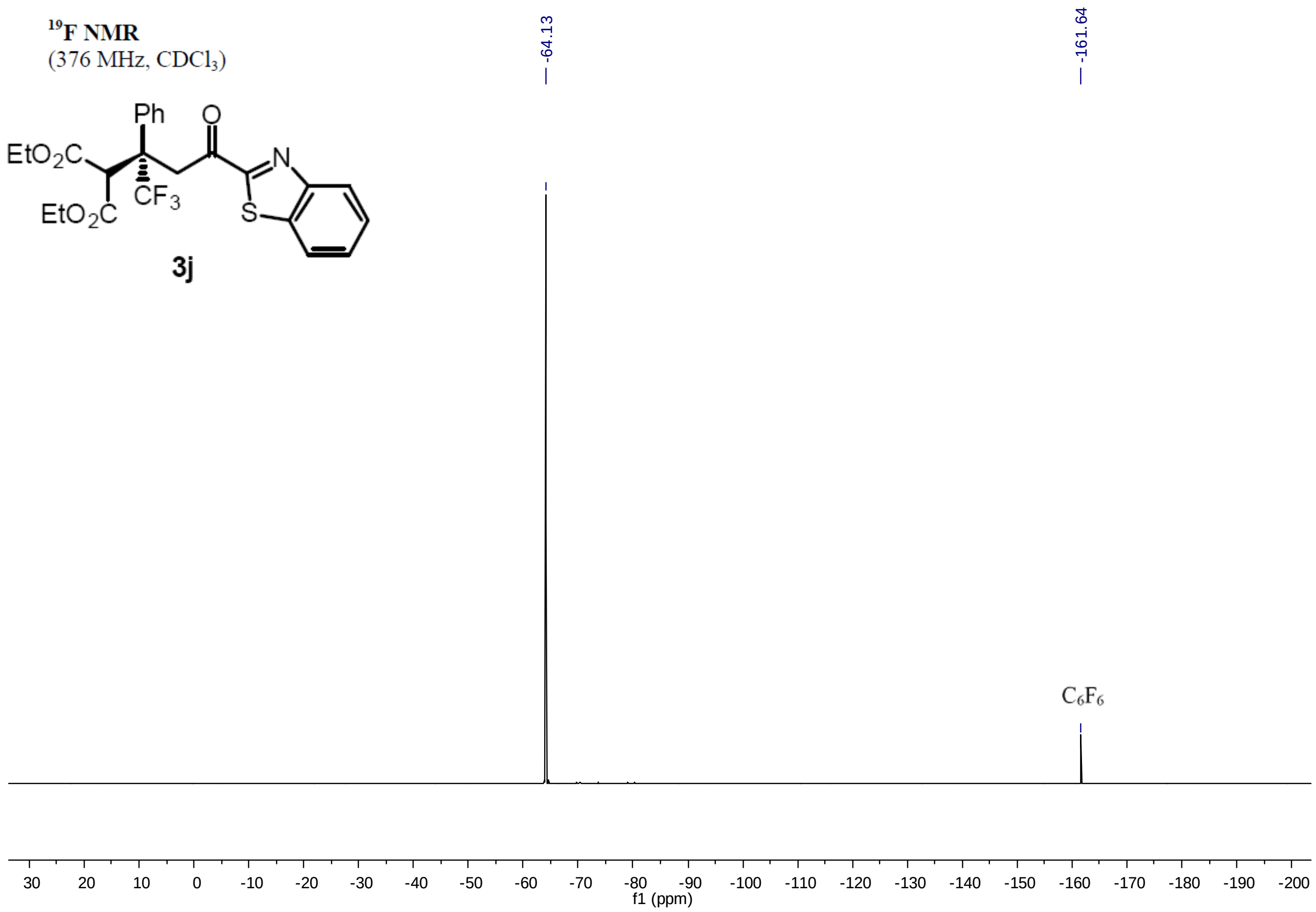

^{19}F NMR
(376 MHz, $CDCl_3$)
-64.13
-161.64
Ph
O
EtO_2C
N
CF_3
EtO_2C
S
3j
C_6F_6
30 20 10 0 -10 -20 -30 -40 -50 -60 -70 -80 -90 -100 -110 -120 -130 -140 -150 -160 -170 -180 -190 -200
f1 (ppm)

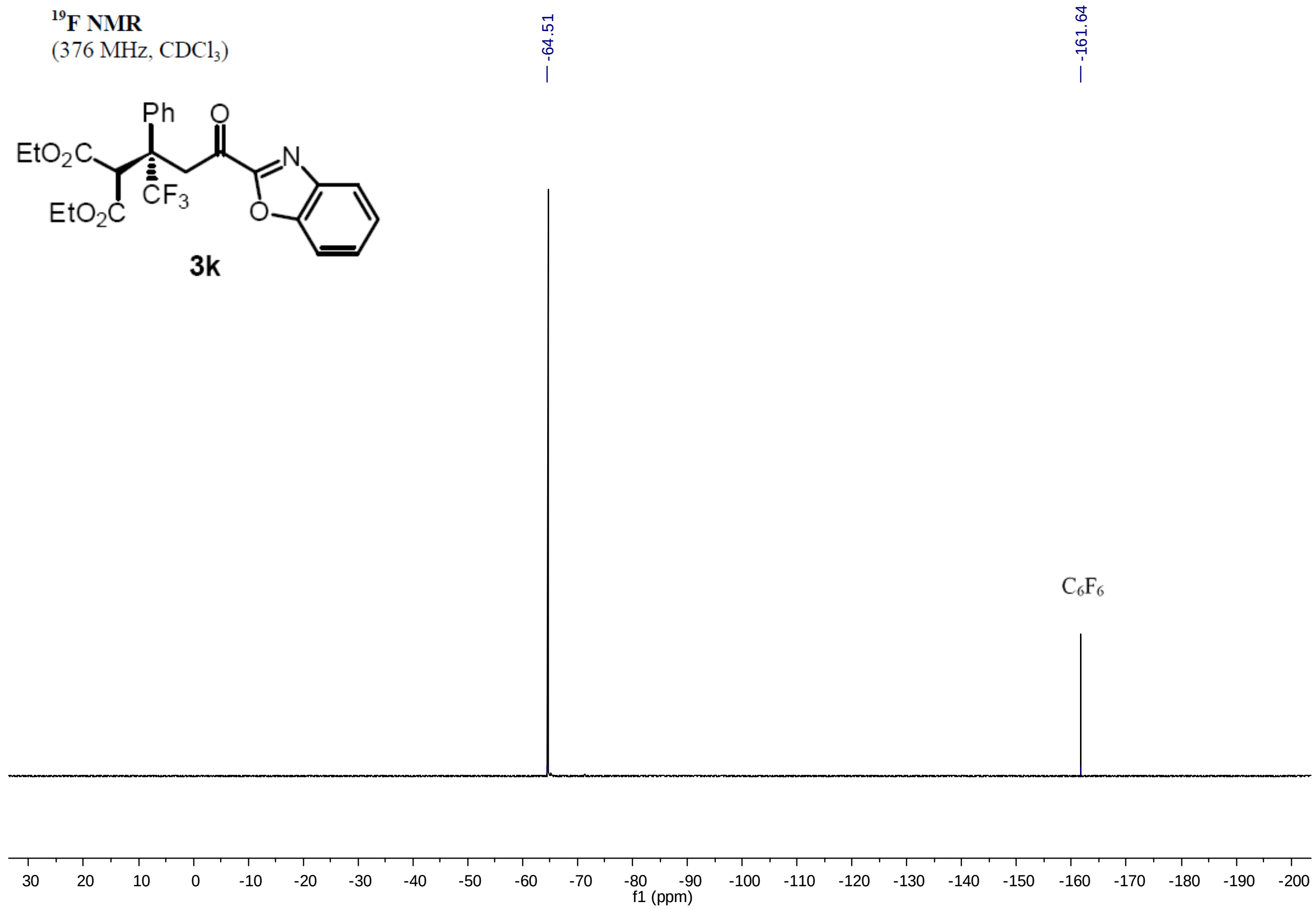

^{19}F NMR
(376 MHz, $CDCl_3$)
-64.51
-161.64
Ph
O
EtO_2C
N
CF_3
EtO_2C
O
3k
C_6F_6
30 20 10 0 -10 -20 -30 -40 -50 -60 -70 -80 -90 -100 -110 -120 -130 -140 -150 -160 -170 -180 -190 -200
f1 (ppm)

^{19}F NMR
(376 MHz, $CDCl_3$)

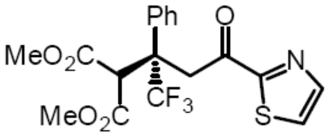

3l

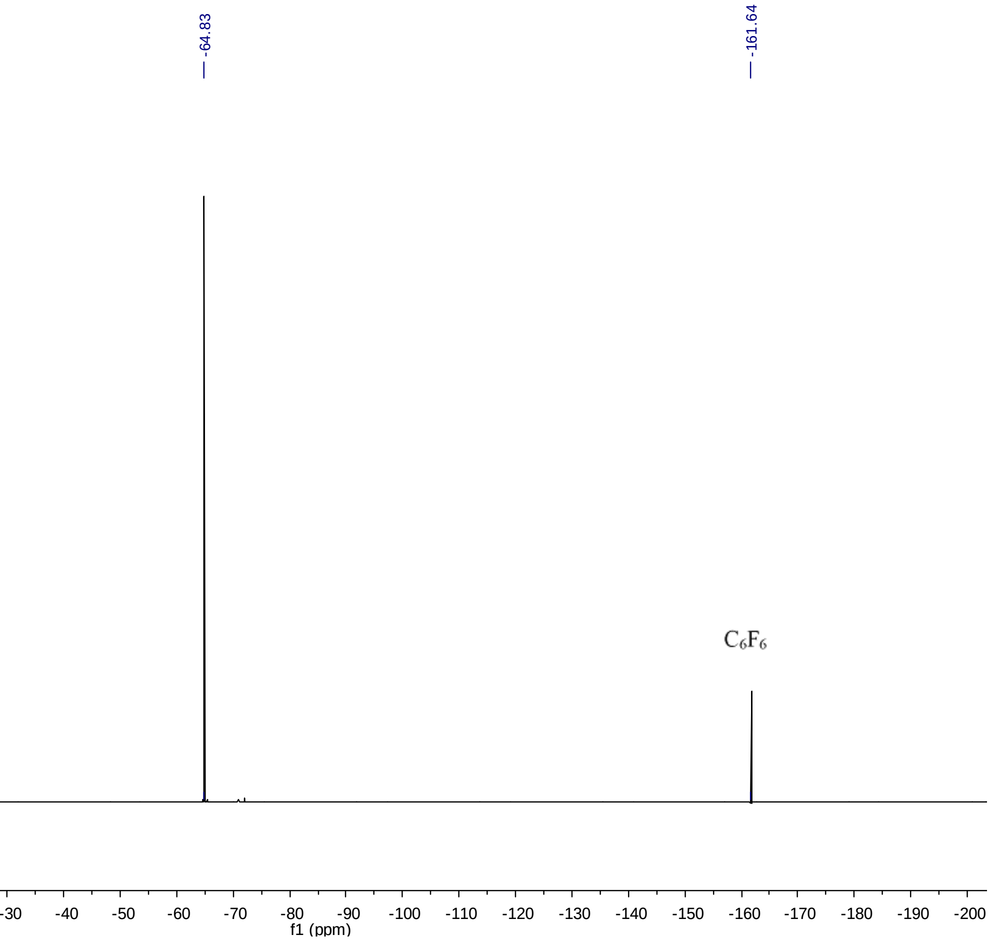

^{19}F NMR
(376 MHz, $CDCl_3$)

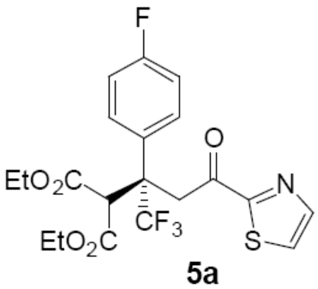

5a

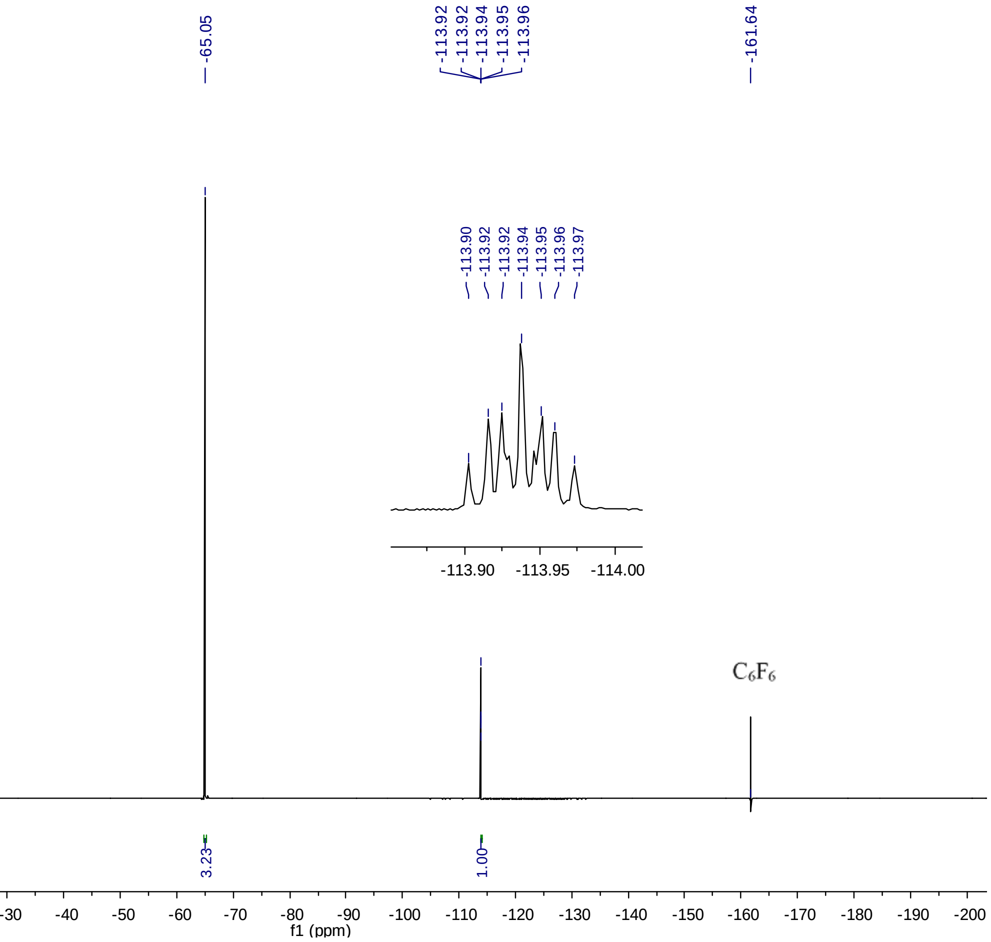

^{19}F NMR
(376 MHz, $CDCl_3$)

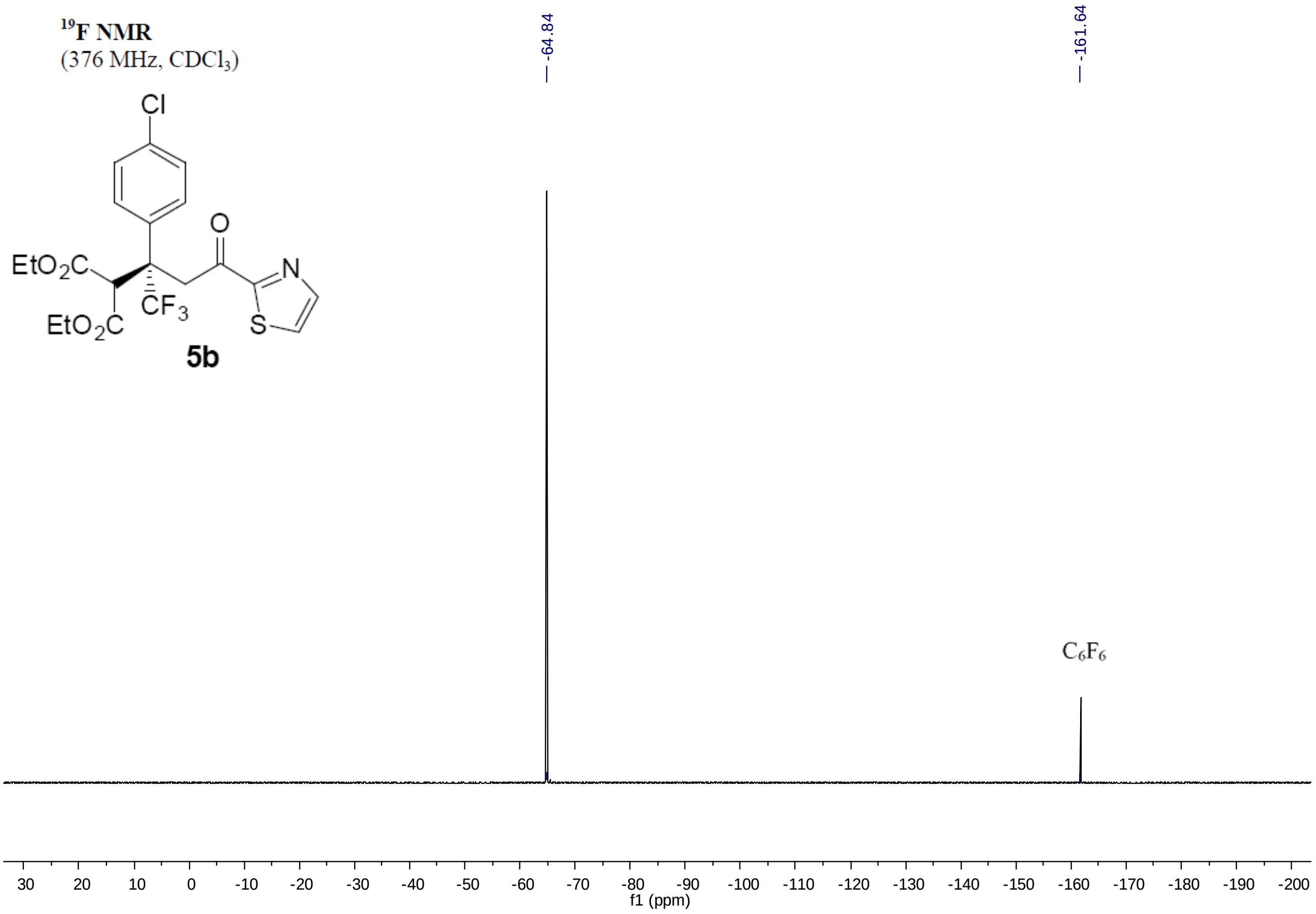

^{19}F NMR
(376 MHz, $CDCl_3$)

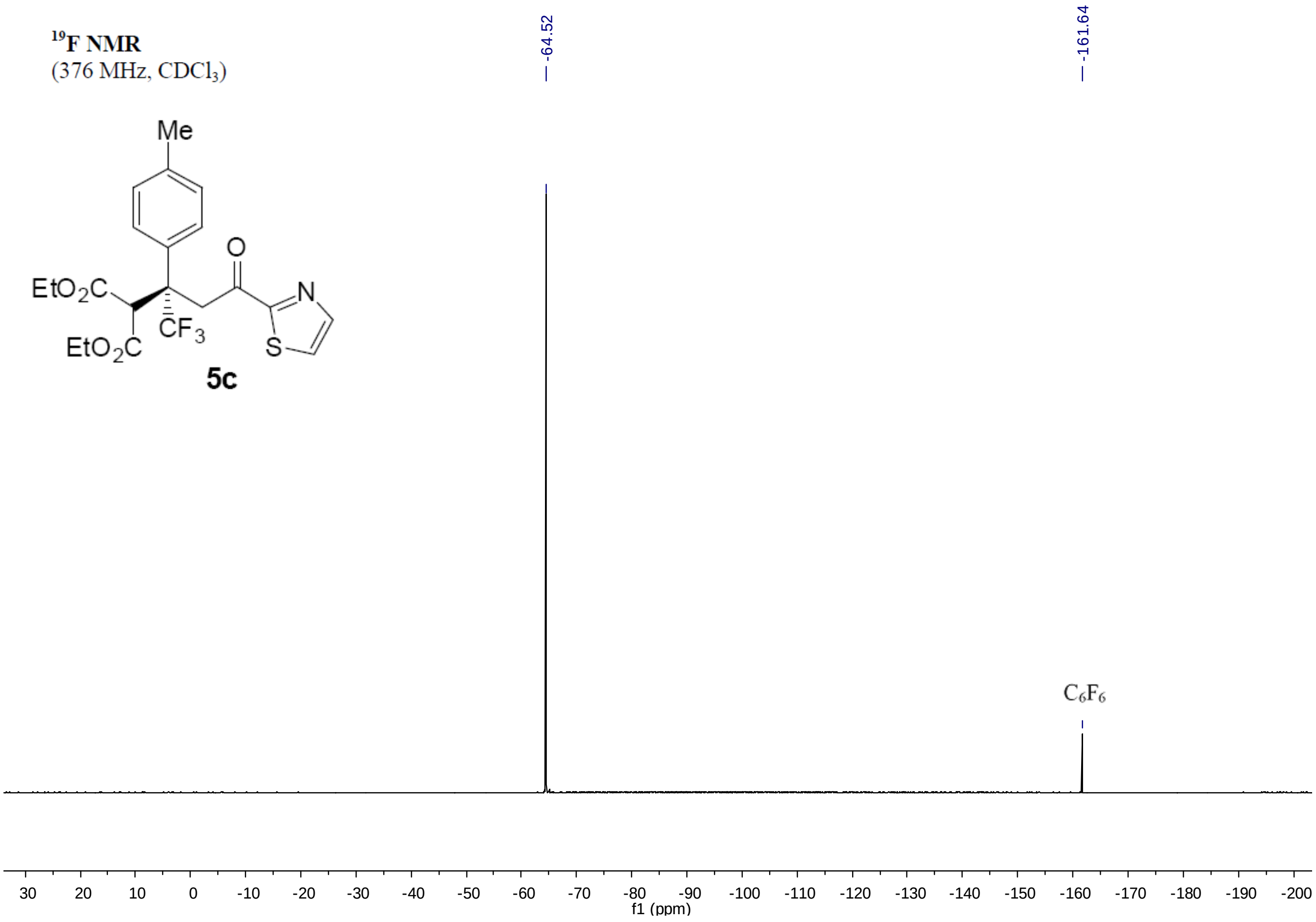

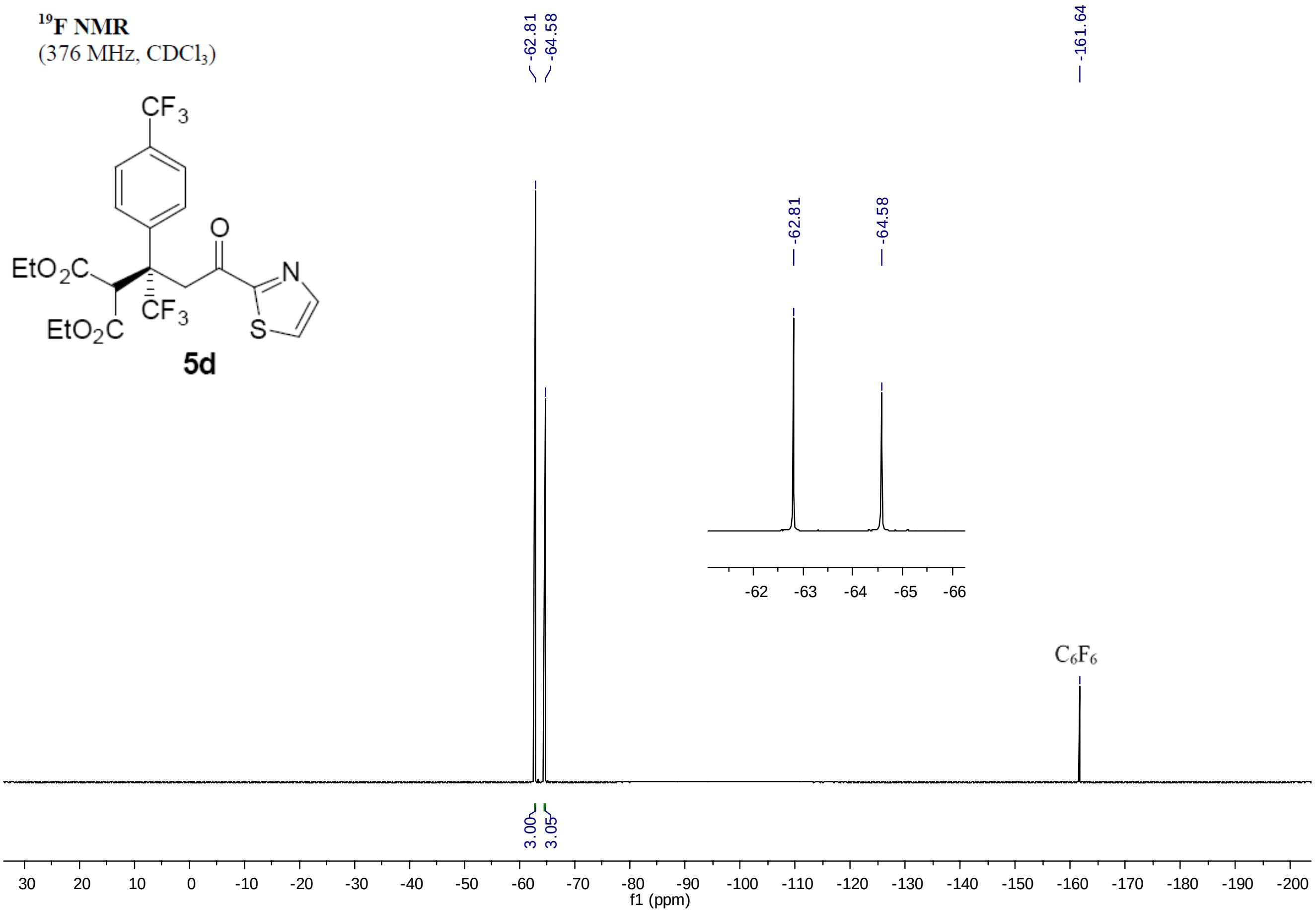
19F NMR
(376 MHz, CDCl3)
5d
-62.81
-64.58
-161.64
C6F6
3.00
3.05
f1 (ppm)

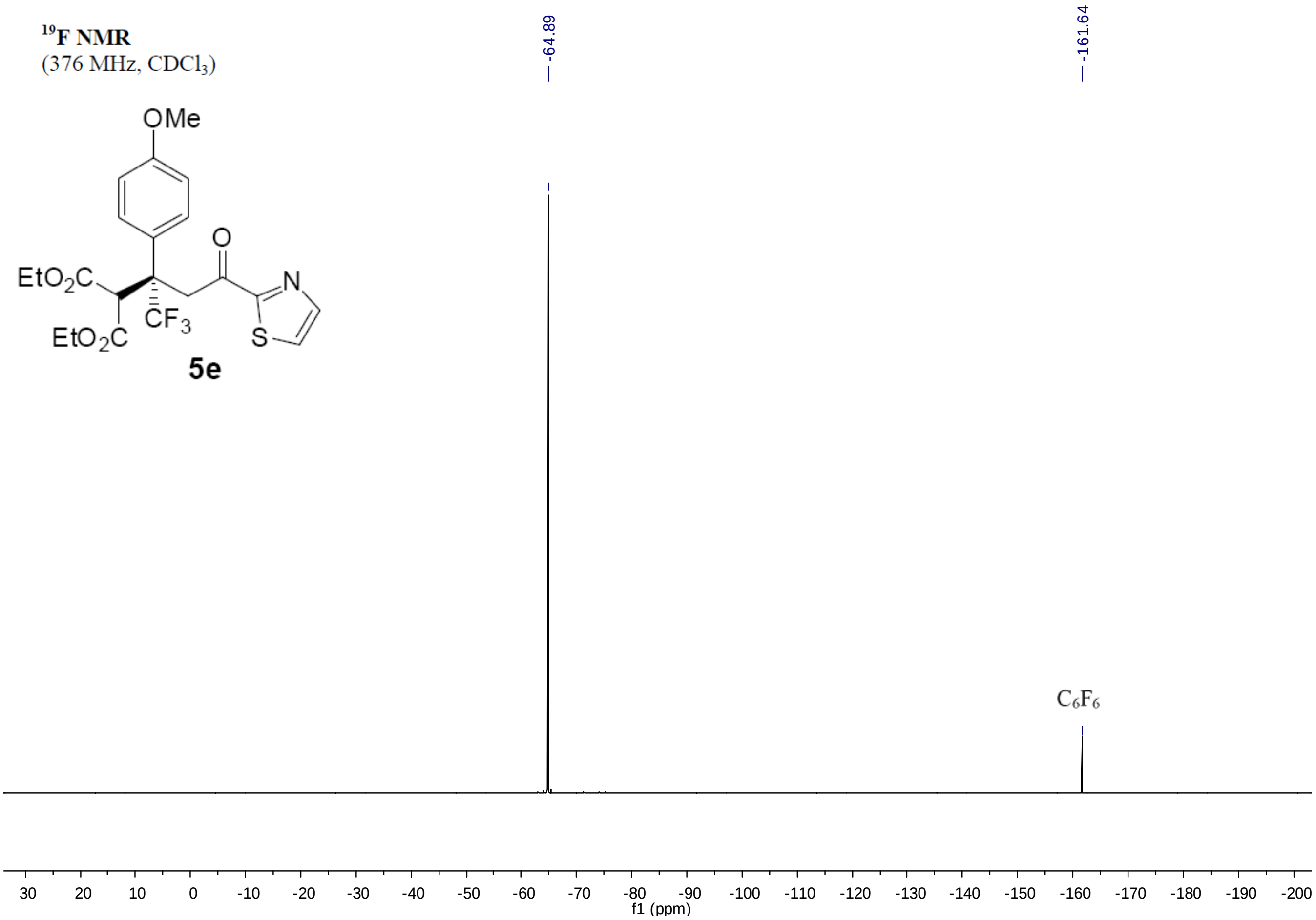
19F NMR
(376 MHz, CDCl3)
5e
-64.89
-161.64
C6F6
f1 (ppm)

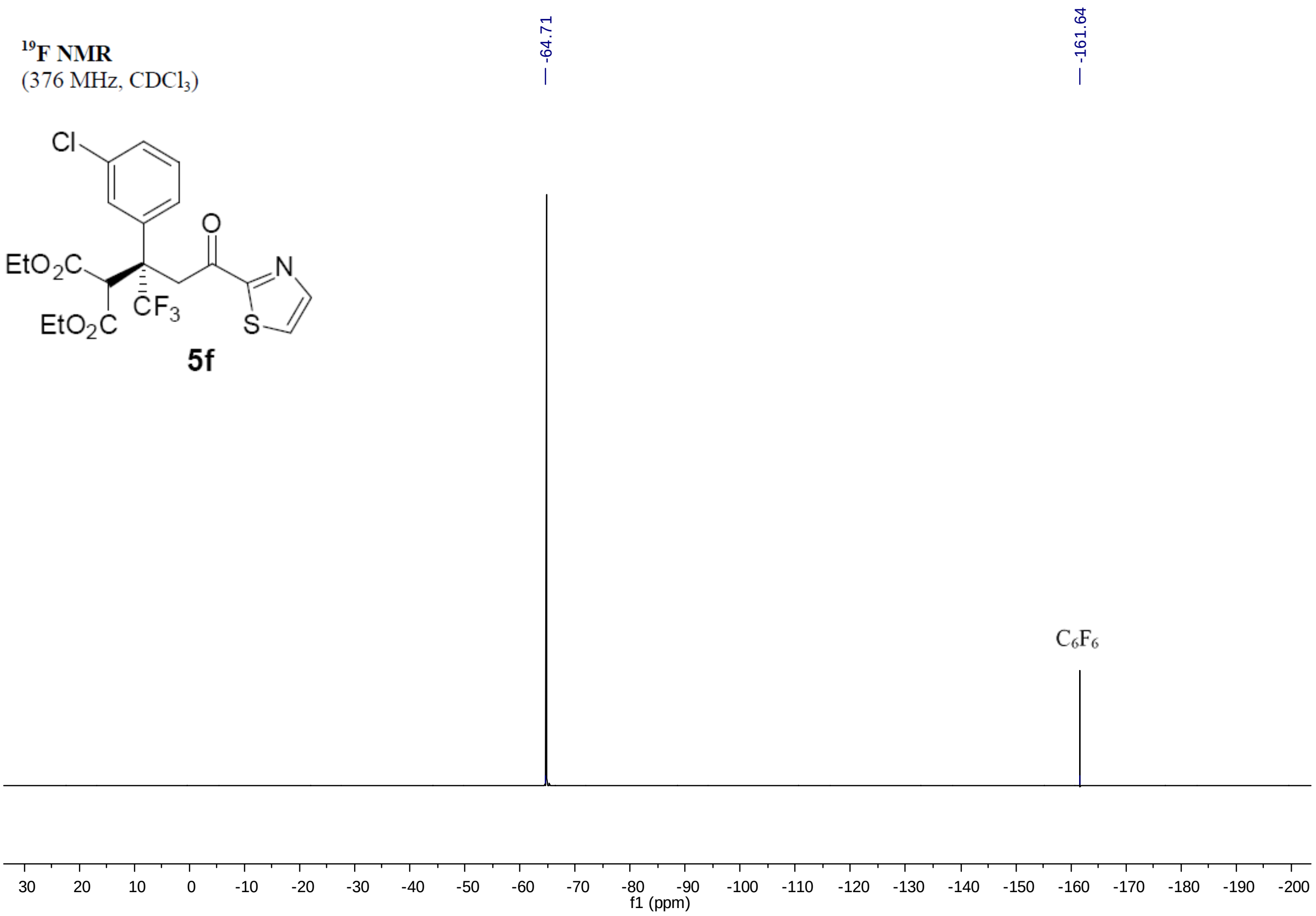

^{19}F NMR
(376 MHz, $CDCl_3$)
-64.71
-161.64
C_6F_6
Cl
O
EtO_2C
N
CF_3
EtO_2C
S
5f
30 20 10 0 -10 -20 -30 -40 -50 -60 -70 -80 -90 -100 -110 -120 -130 -140 -150 -160 -170 -180 -190 -200
f1 (ppm)

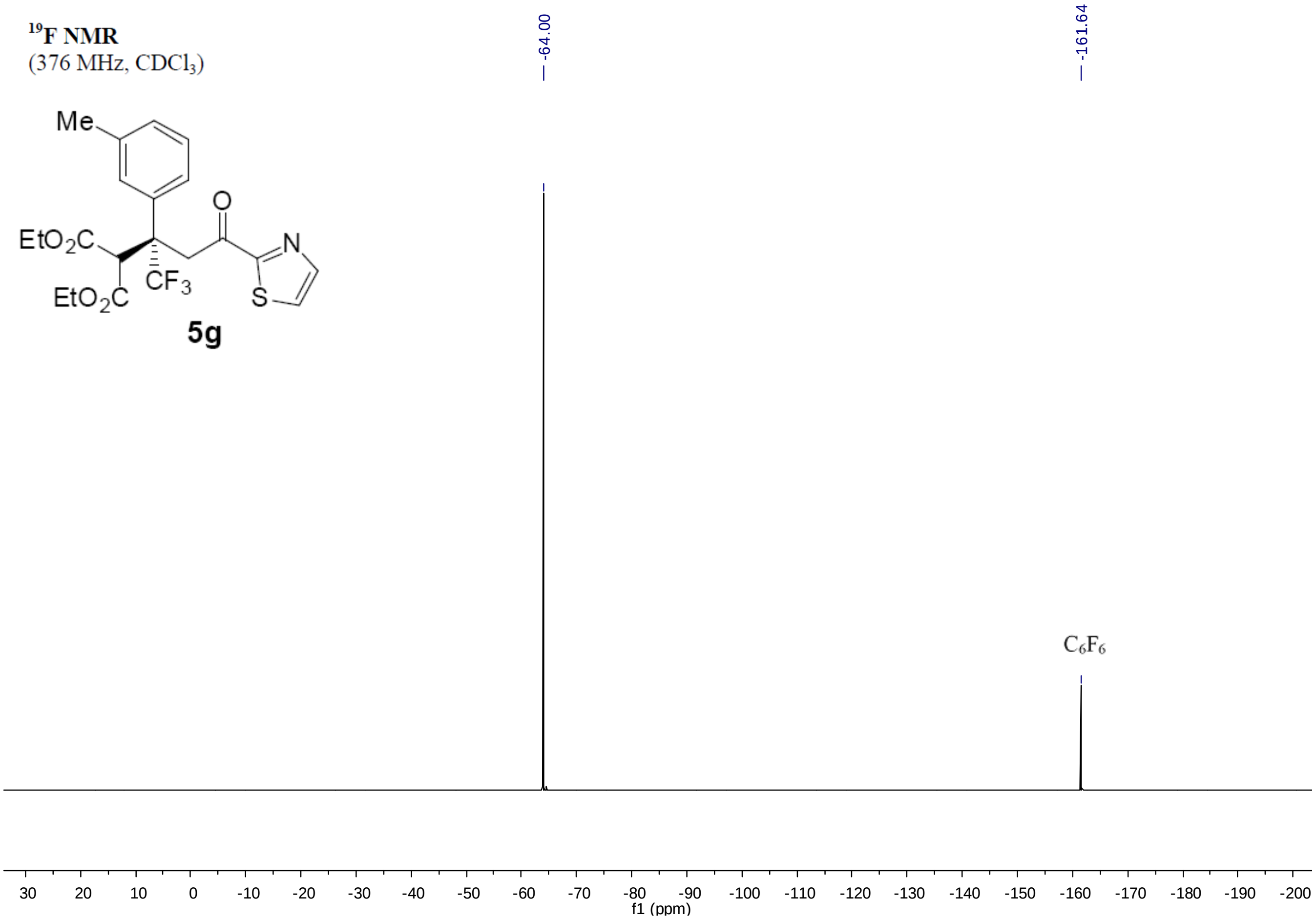

^{19}F NMR
(376 MHz, $CDCl_3$)
-64.00
-161.64
C_6F_6
Me
O
EtO_2C
N
CF_3
EtO_2C
S
5g
30 20 10 0 -10 -20 -30 -40 -50 -60 -70 -80 -90 -100 -110 -120 -130 -140 -150 -160 -170 -180 -190 -200
f1 (ppm)

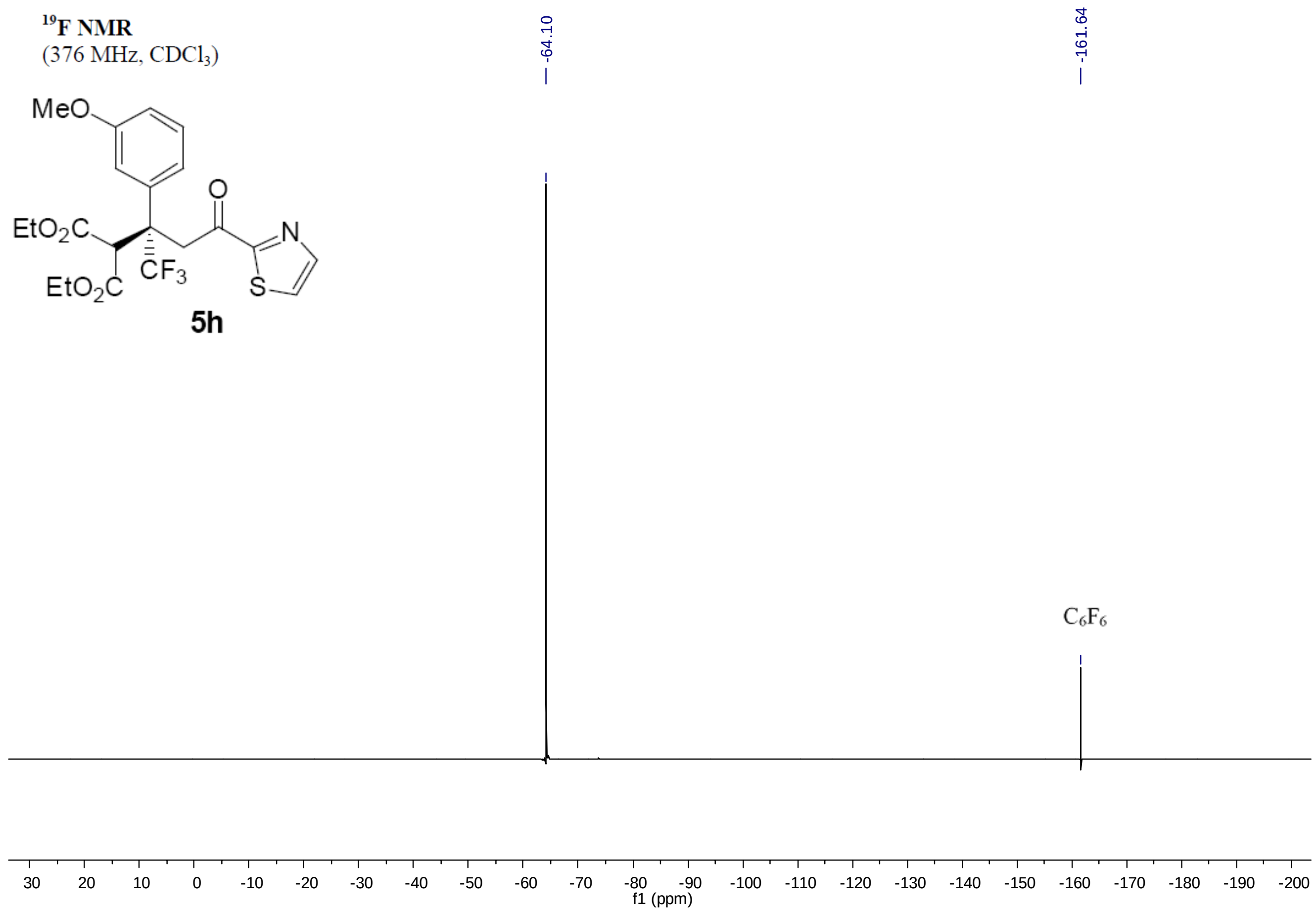
^{19}F NMR
(376 MHz, $CDCl_3$)
MeO
EtO_2C
EtO_2C
CF_3
O
N
S
5h
-64.10
-161.64
C_6F_6
30 20 10 0 -10 -20 -30 -40 -50 -60 -70 -80 -90 -100 -110 -120 -130 -140 -150 -160 -170 -180 -190 -200
f1 (ppm)

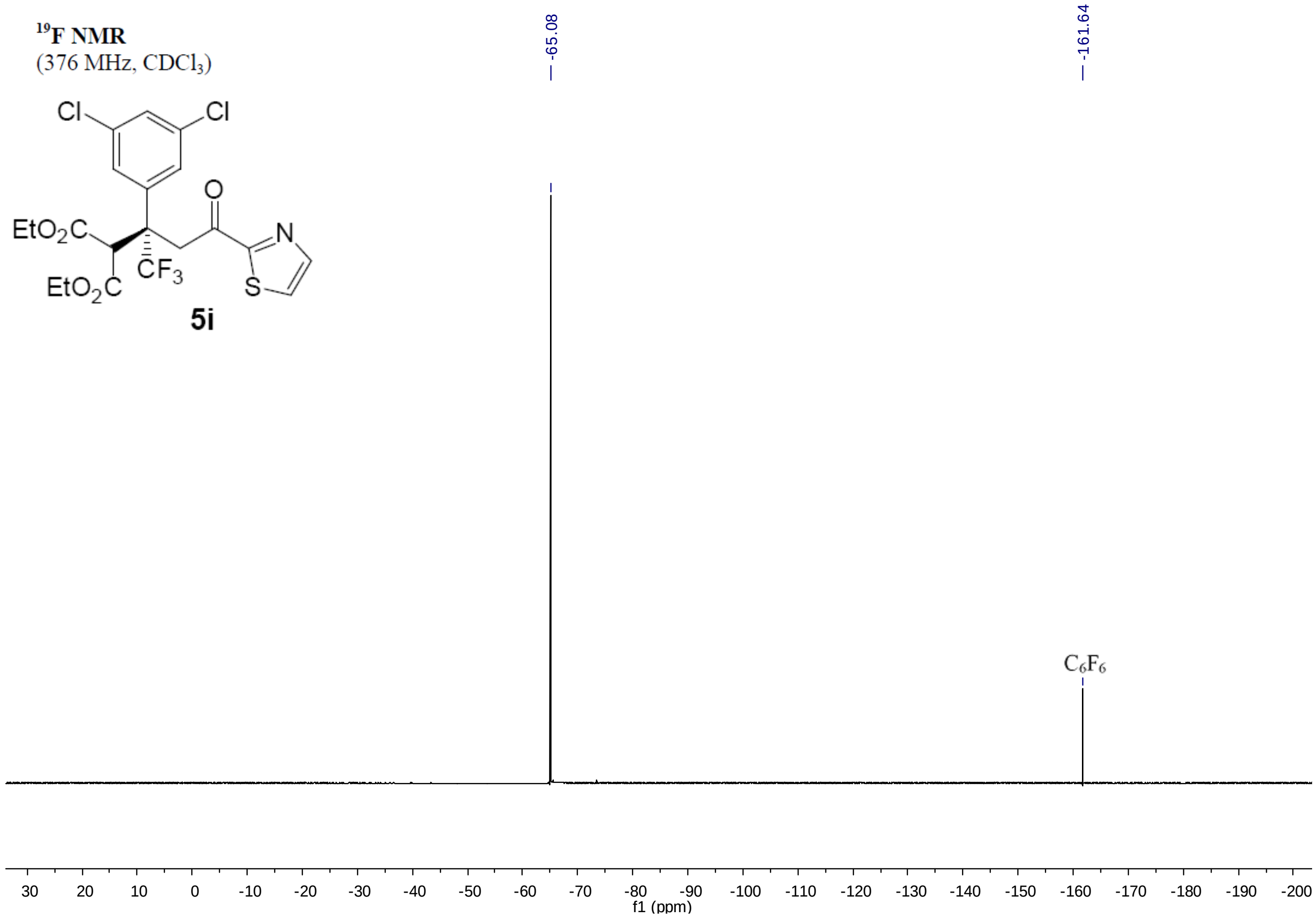
^{19}F NMR
(376 MHz, $CDCl_3$)
Cl
Cl
EtO_2C
EtO_2C
CF_3
O
N
S
5i
-65.08
-161.64
C_6F_6
30 20 10 0 -10 -20 -30 -40 -50 -60 -70 -80 -90 -100 -110 -120 -130 -140 -150 -160 -170 -180 -190 -200
f1 (ppm)

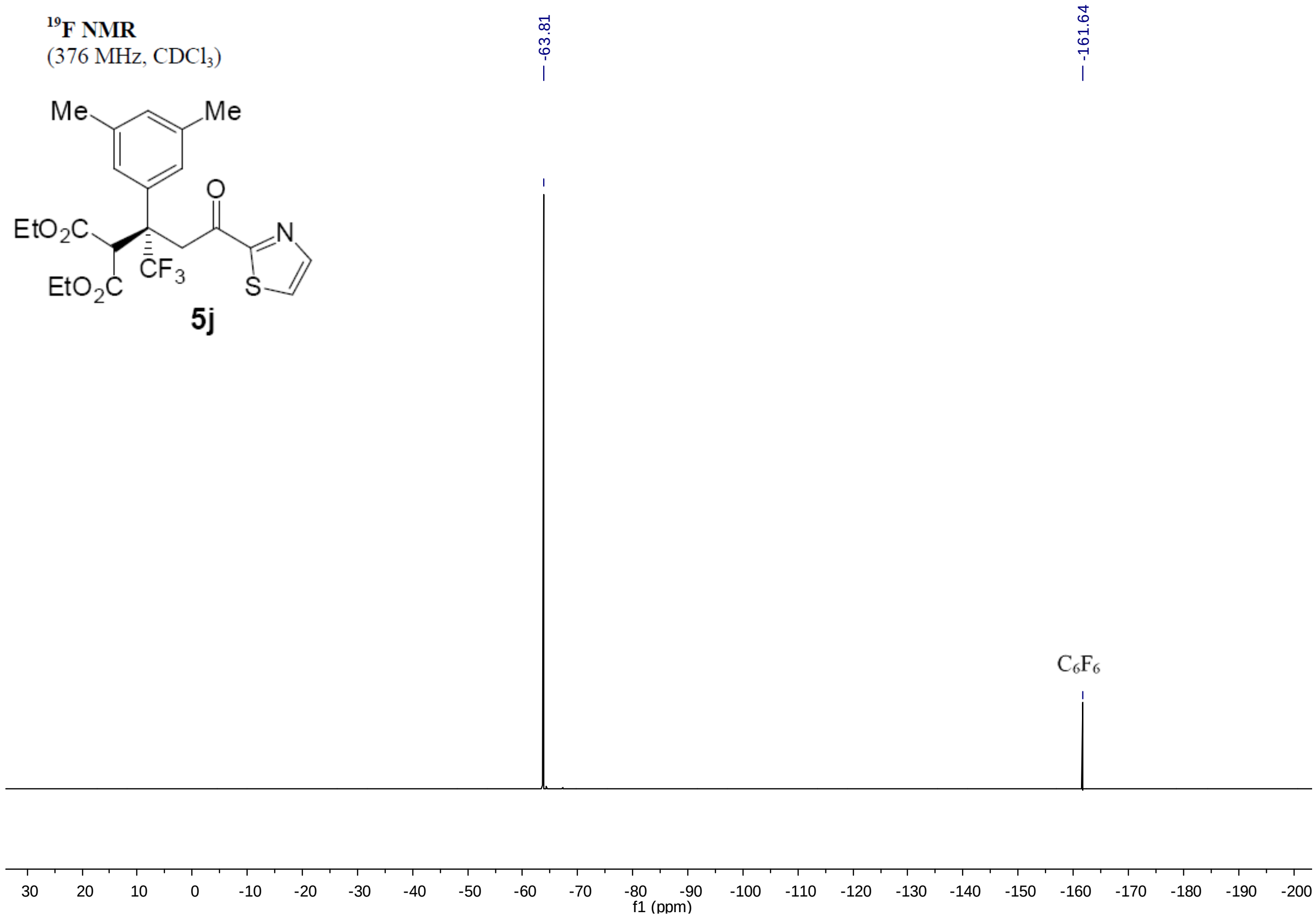

19F NMR
(376 MHz, CDCl3)
-63.81
-161.64
5j
C6F6
f1 (ppm)

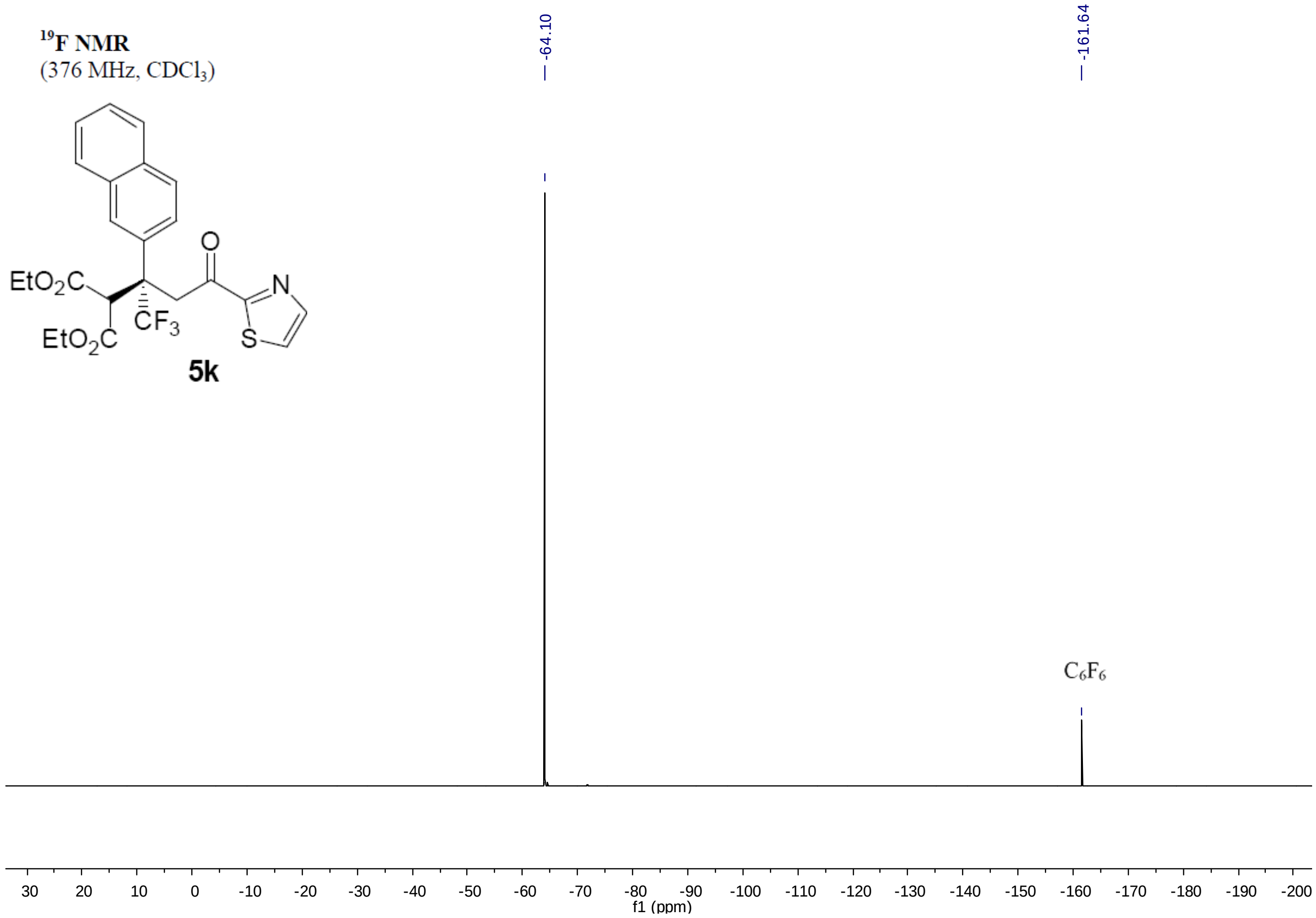

19F NMR
(376 MHz, CDCl3)
-64.10
-161.64
5k
C6F6
f1 (ppm)

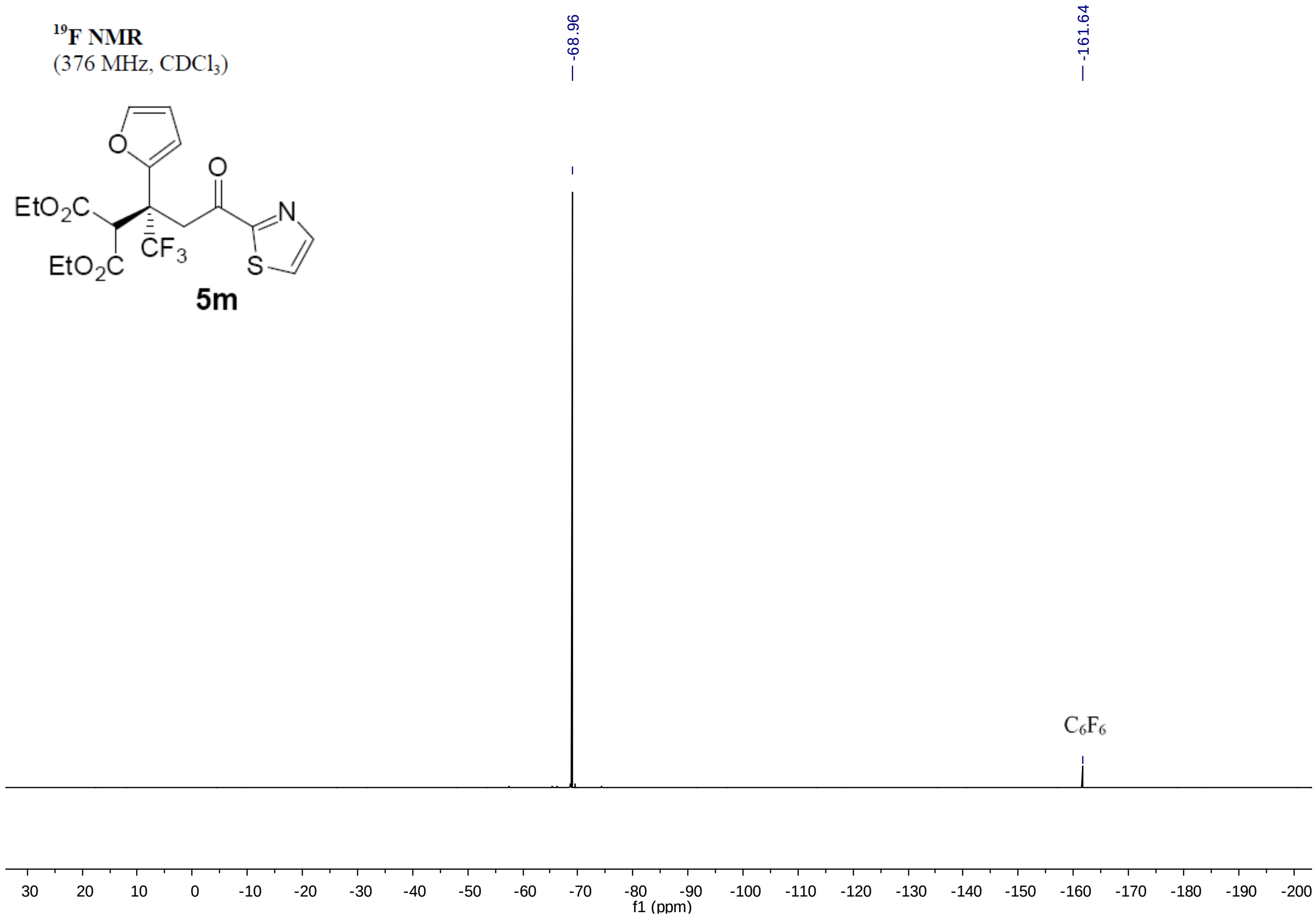
19F NMR
(376 MHz, CDCl3)
5m
-68.96
-161.64
C6F6
f1 (ppm)

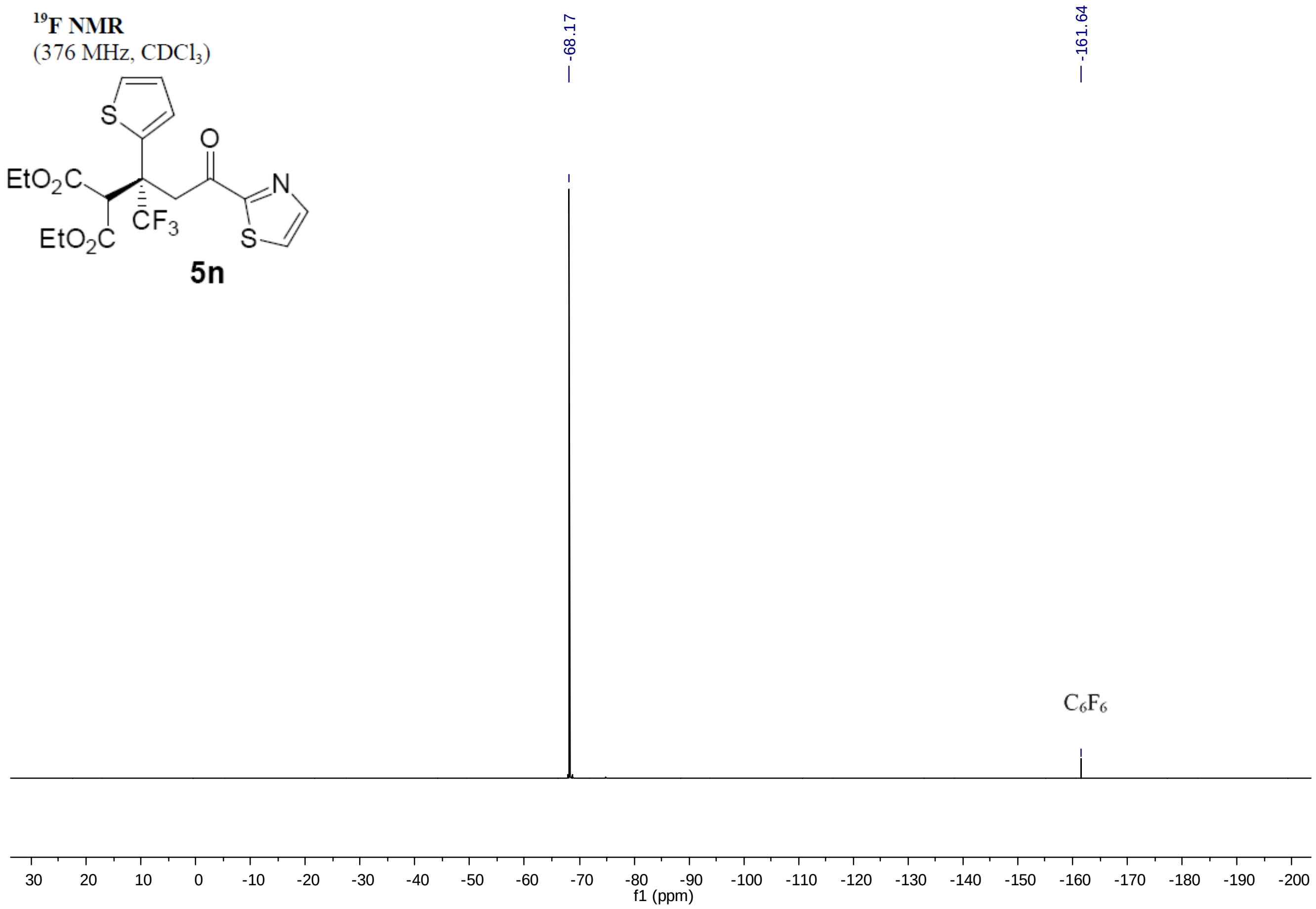
19F NMR
(376 MHz, CDCl3)
5n
-68.17
-161.64
C6F6
f1 (ppm)

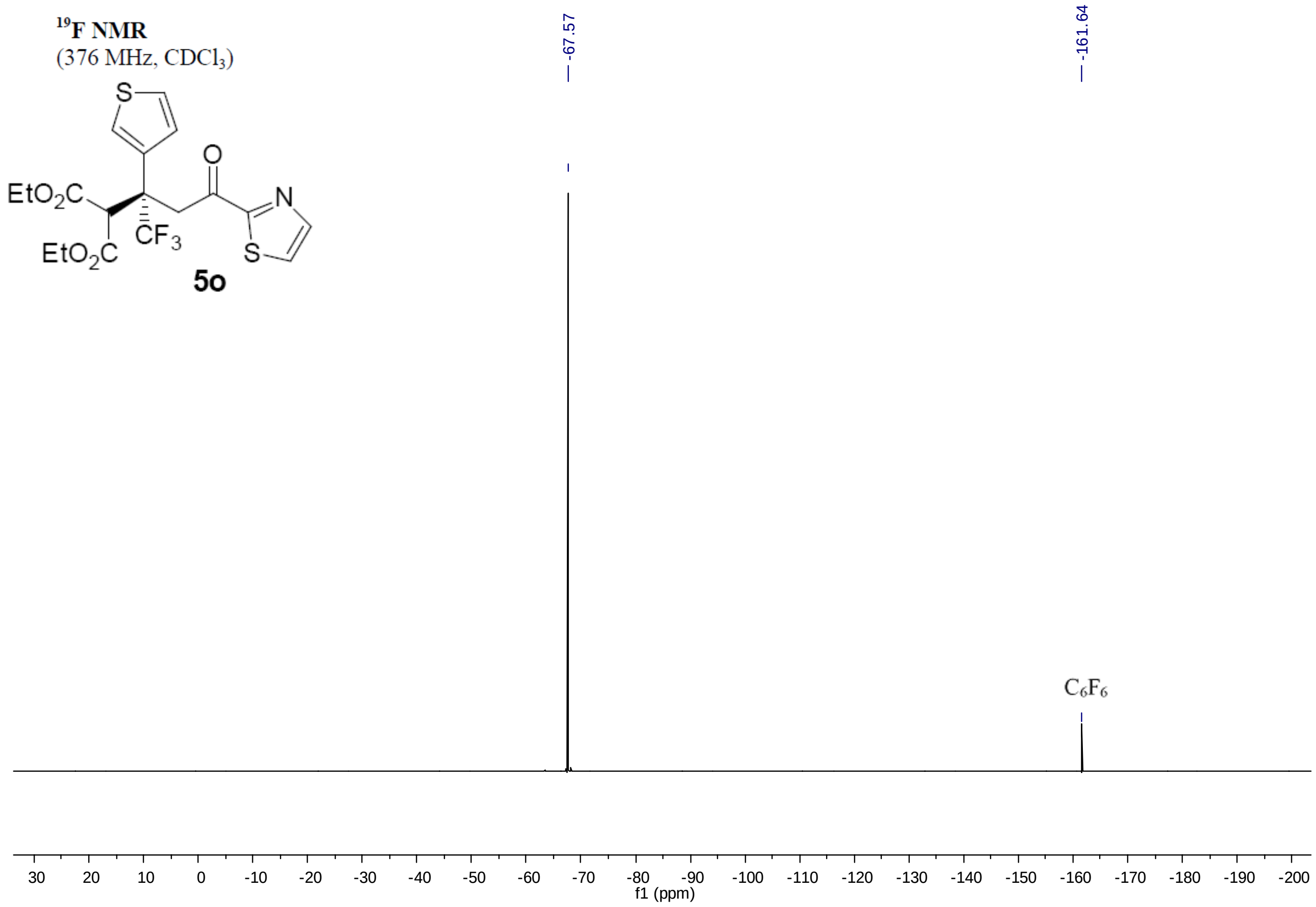

19F NMR
(376 MHz, CDCl3)
5o
-67.57
-161.64
C6F6
f1 (ppm)

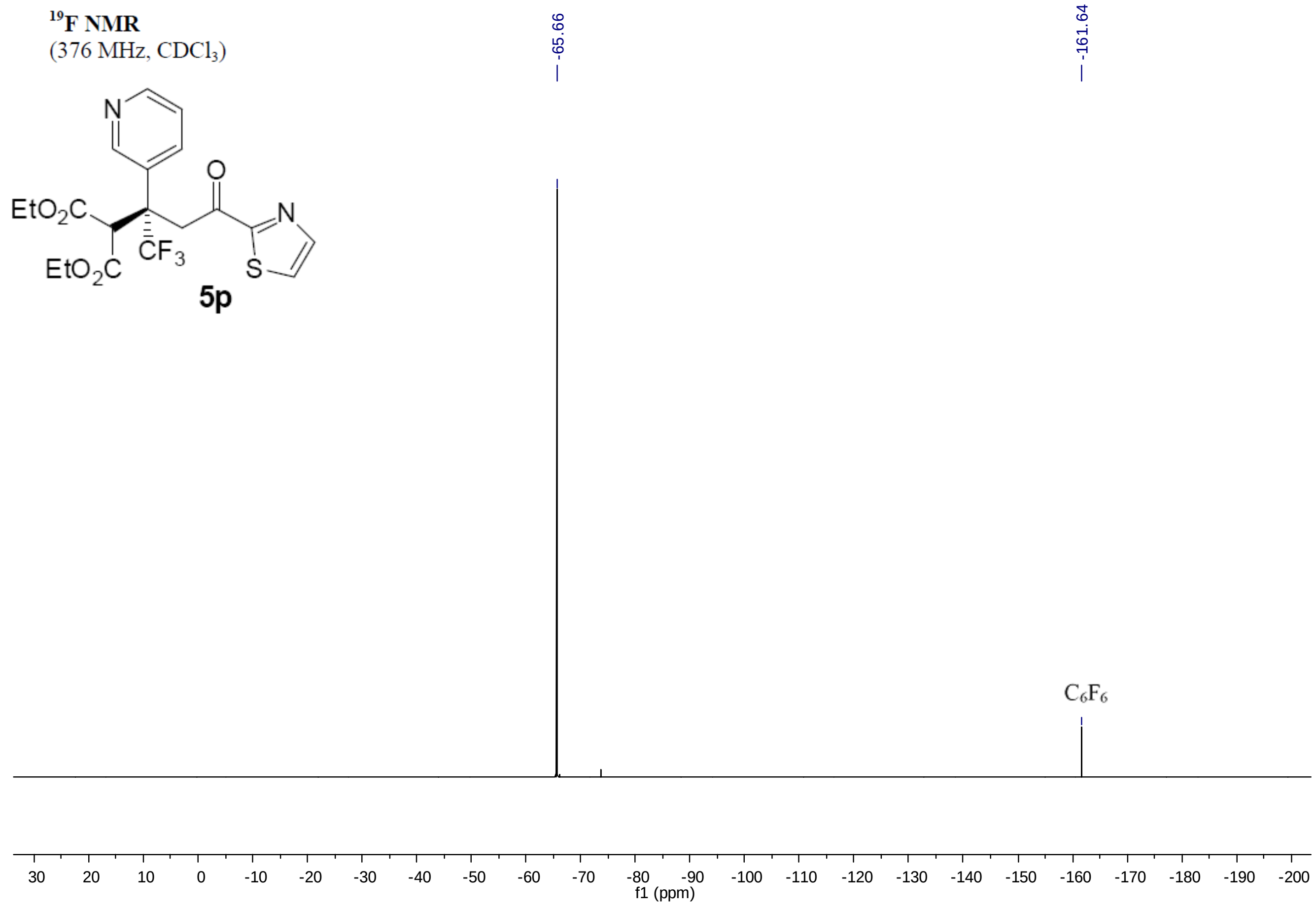

19F NMR
(376 MHz, CDCl3)
5p
-65.66
-161.64
C6F6
f1 (ppm)

^{19}F NMR
(376 MHz, $CDCl_3$)

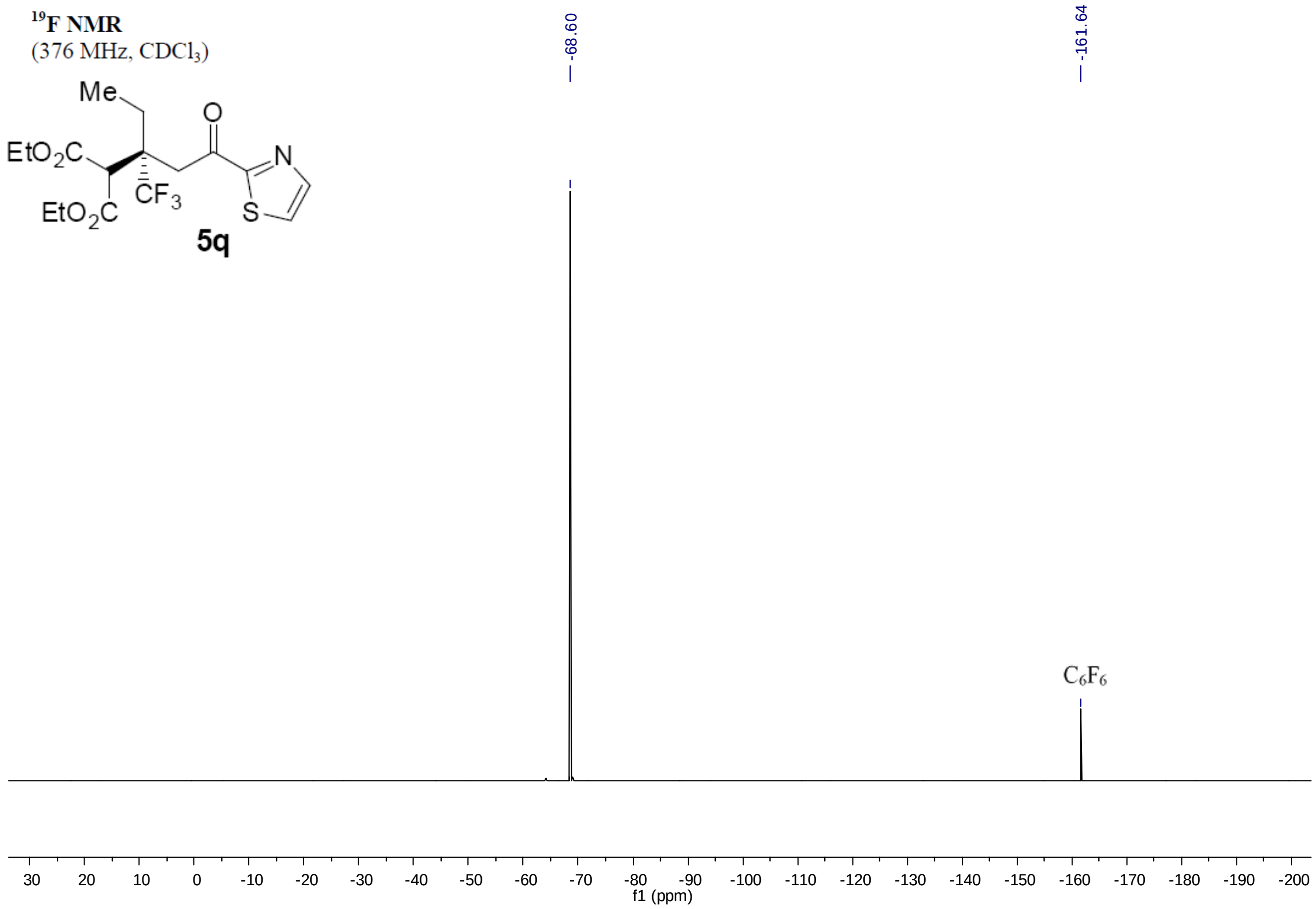

^{19}F NMR
(376 MHz, $CDCl_3$)

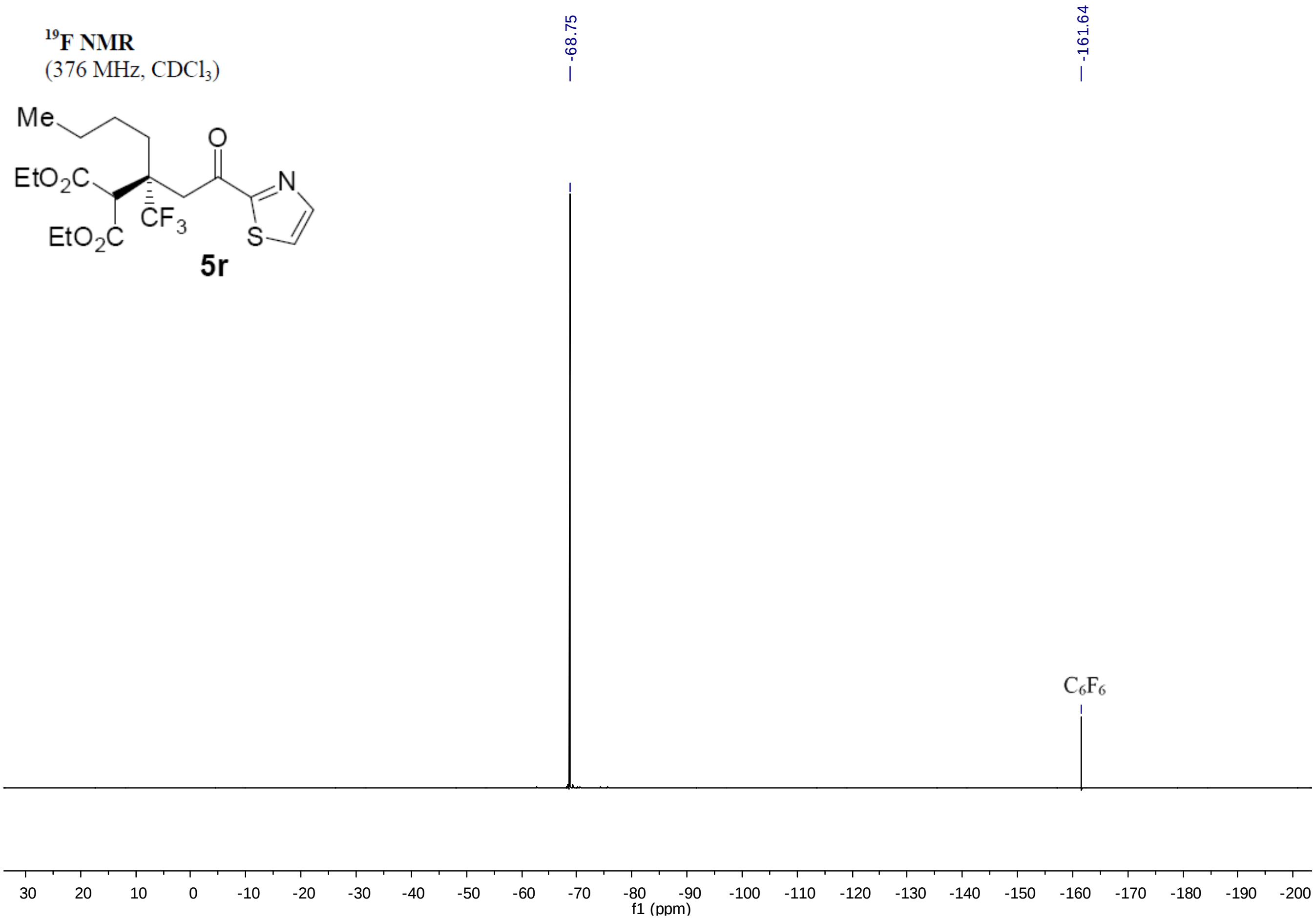

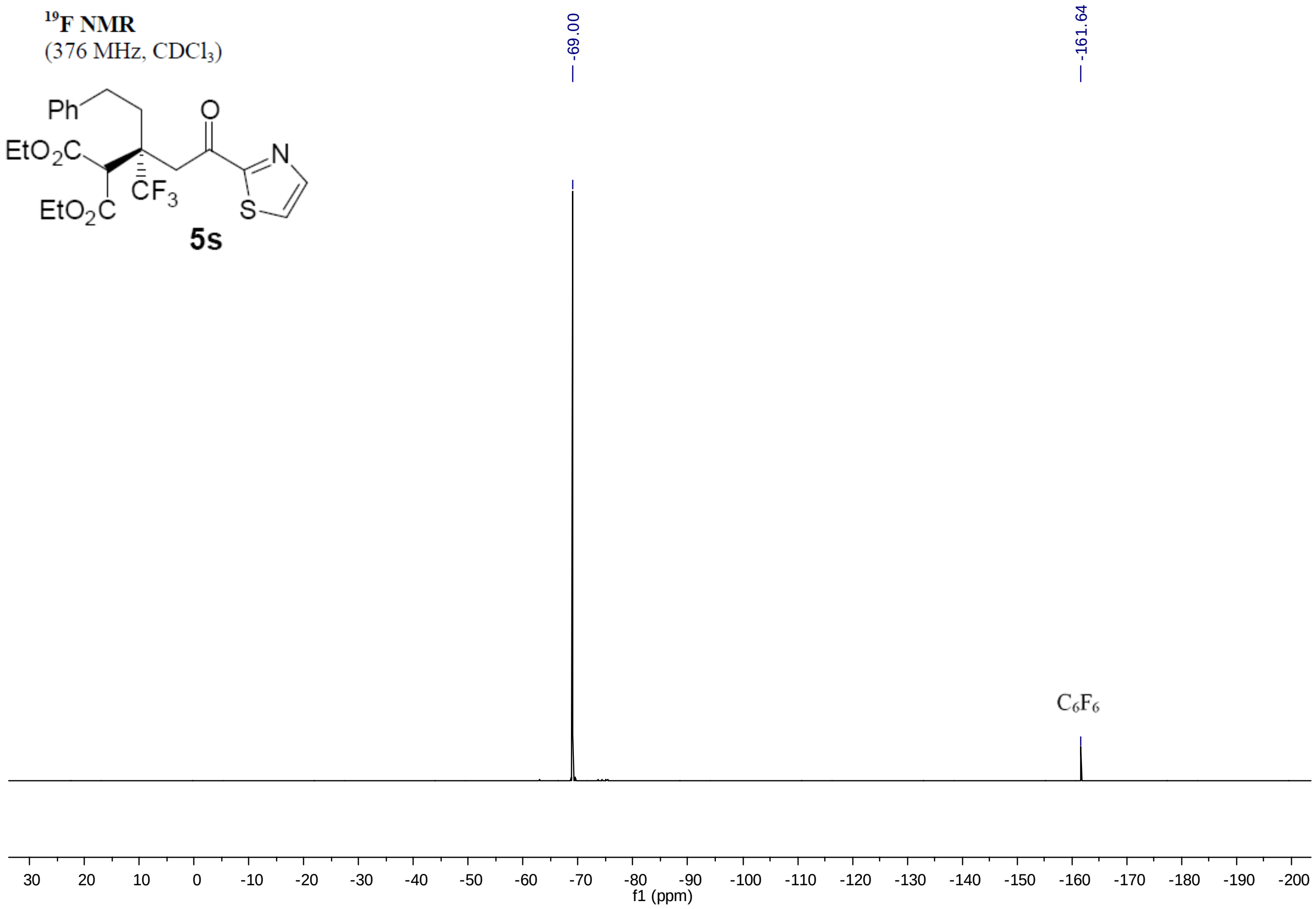
19F NMR
(376 MHz, CDCl3)
Ph
EtO2C
EtO2C
CF3
O
N
S
5s
-69.00
-161.64
C6F6
30 20 10 0 -10 -20 -30 -40 -50 -60 -70 -80 -90 -100 -110 -120 -130 -140 -150 -160 -170 -180 -190 -200
f1 (ppm)

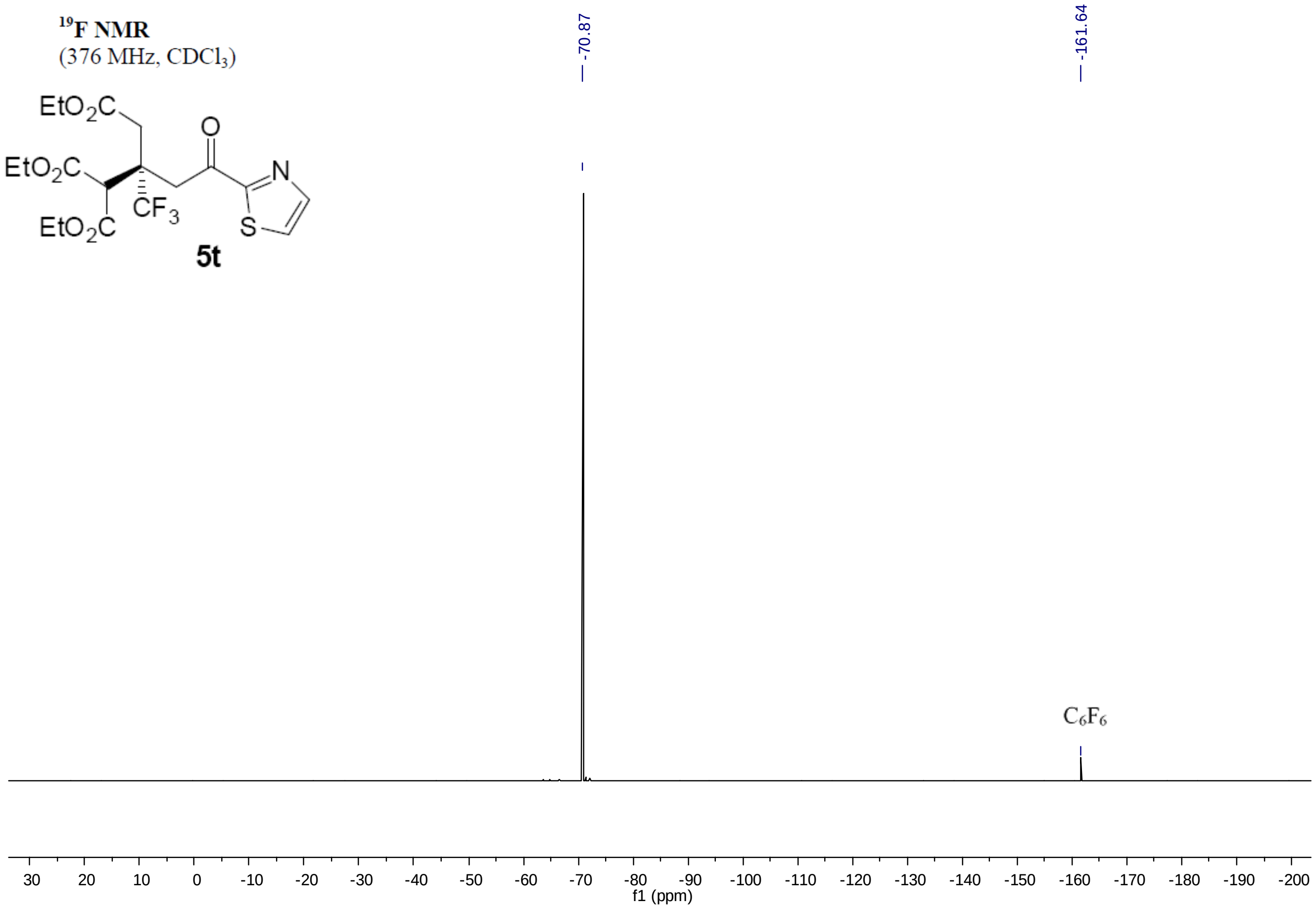
19F NMR
(376 MHz, CDCl3)
EtO2C
EtO2C
EtO2C
CF3
O
N
S
5t
-70.87
-161.64
C6F6
30 20 10 0 -10 -20 -30 -40 -50 -60 -70 -80 -90 -100 -110 -120 -130 -140 -150 -160 -170 -180 -190 -200
f1 (ppm)

^{19}F NMR
(376 MHz, $CDCl_3$)

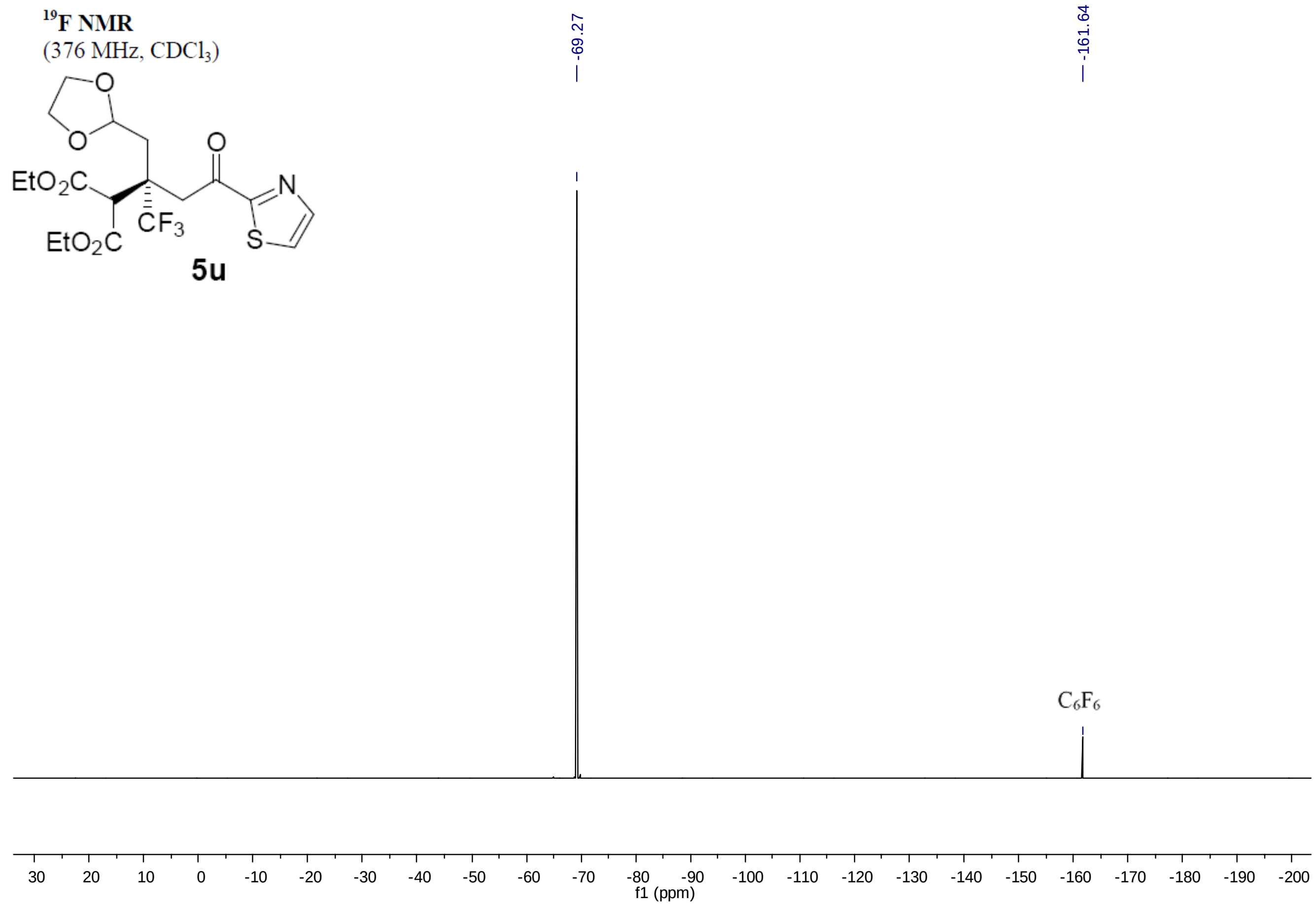

^{19}F NMR
(376 MHz, $CDCl_3$)

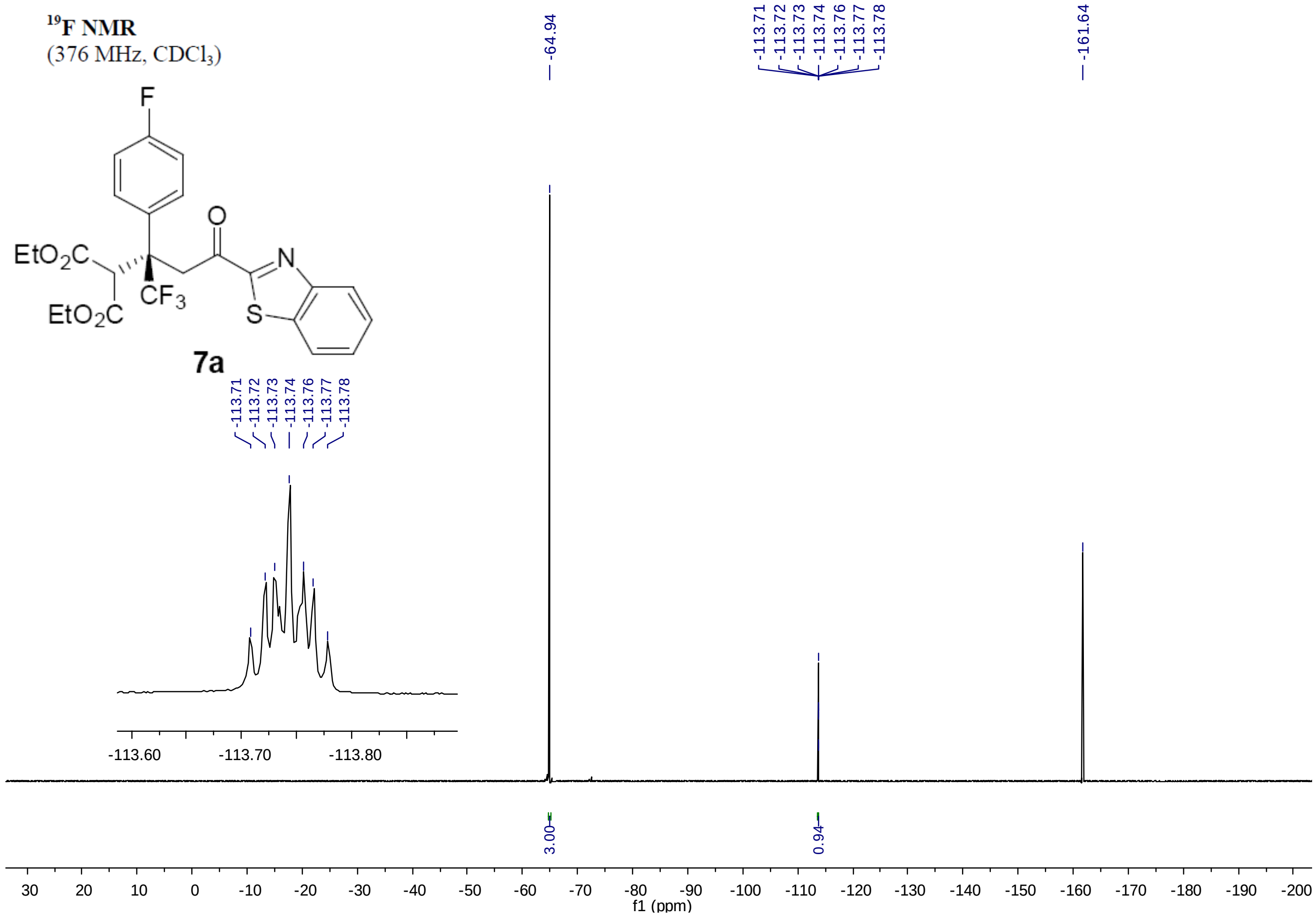

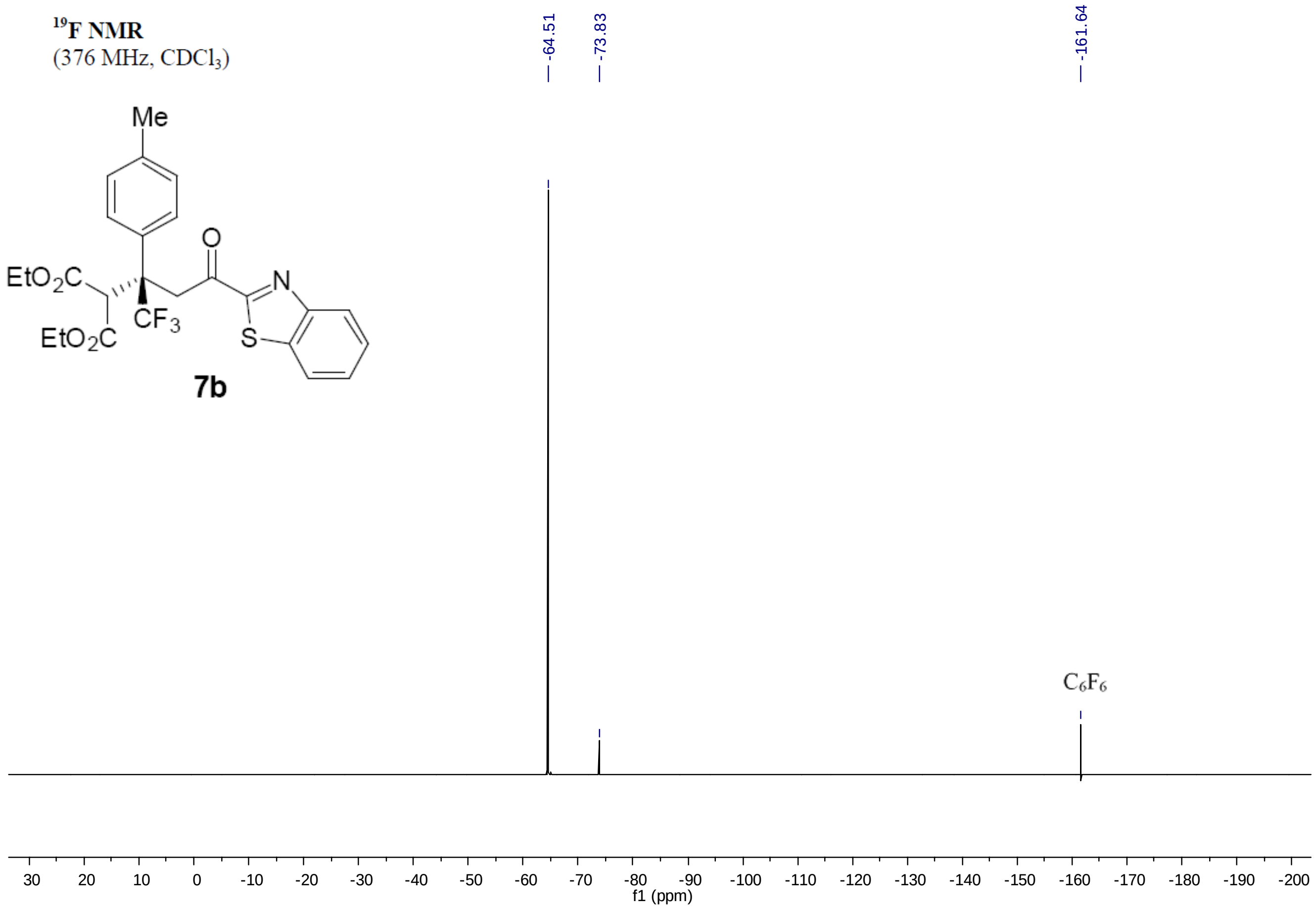
19F NMR
(376 MHz, CDCl3)
-64.51
-73.83
-161.64
Me
O
EtO2C
N
CF3
EtO2C
S
7b
C6F6
30 20 10 0 -10 -20 -30 -40 -50 -60 -70 -80 -90 -100 -110 -120 -130 -140 -150 -160 -170 -180 -190 -200
f1 (ppm)

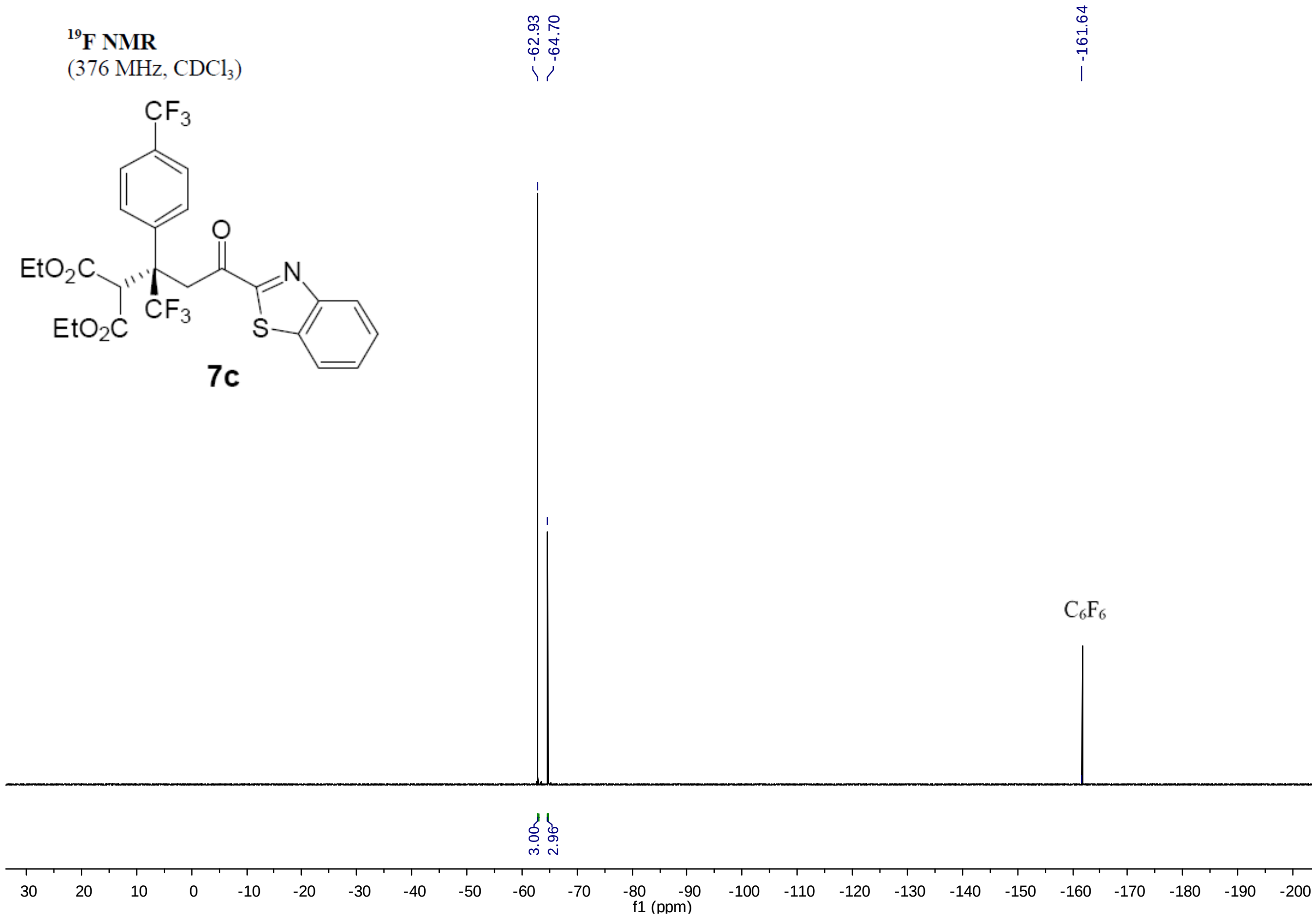
19F NMR
(376 MHz, CDCl3)
-62.93
-64.70
-161.64
CF3
O
EtO2C
N
CF3
EtO2C
S
7c
C6F6
3.00
2.96
30 20 10 0 -10 -20 -30 -40 -50 -60 -70 -80 -90 -100 -110 -120 -130 -140 -150 -160 -170 -180 -190 -200
f1 (ppm)

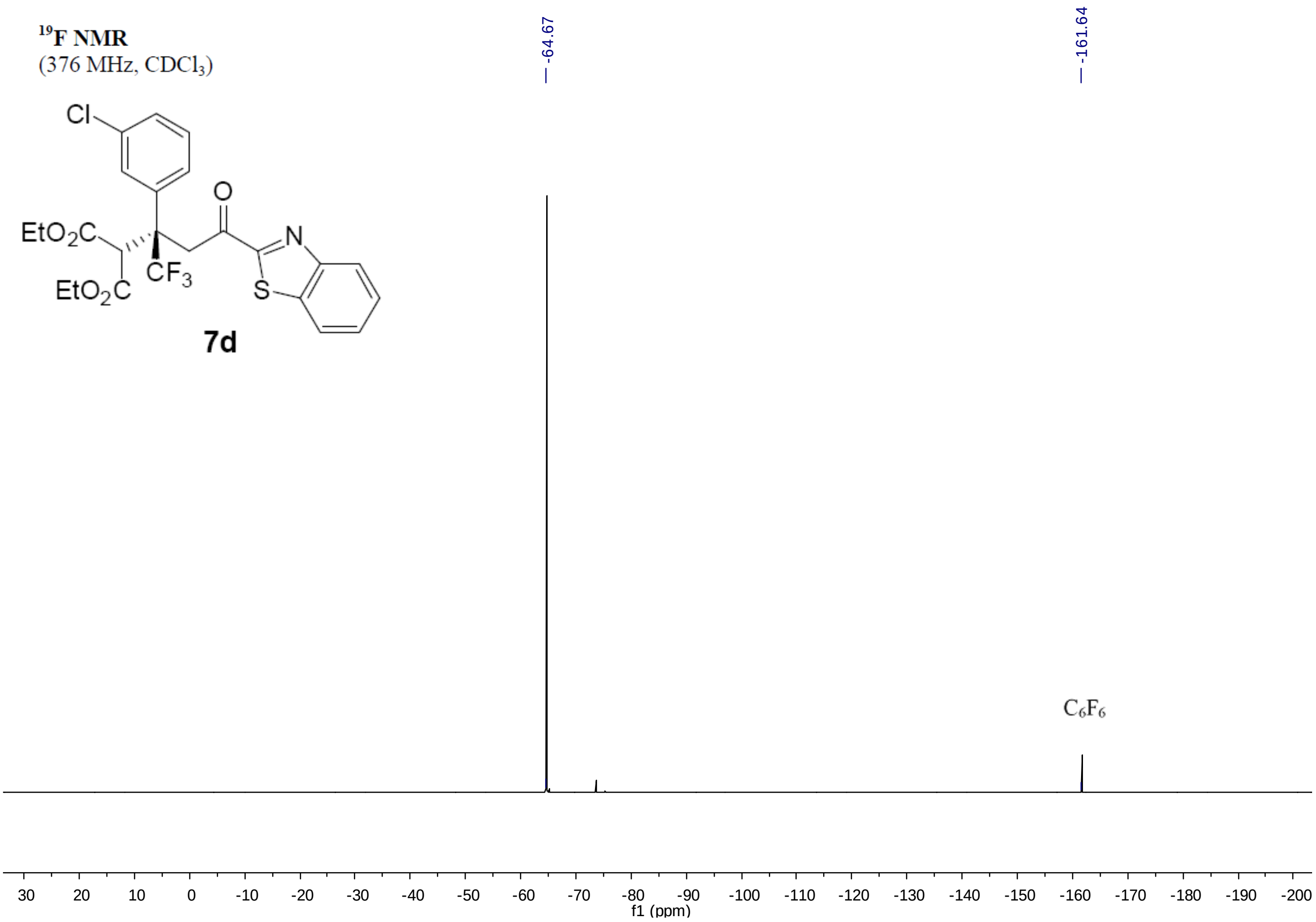
19F NMR
(376 MHz, CDCl3)
-64.67
-161.64
Cl
EtO2C
EtO2C
CF3
O
N
S
7d
C6F6
30 20 10 0 -10 -20 -30 -40 -50 -60 -70 -80 -90 -100 -110 -120 -130 -140 -150 -160 -170 -180 -190 -200
f1 (ppm)

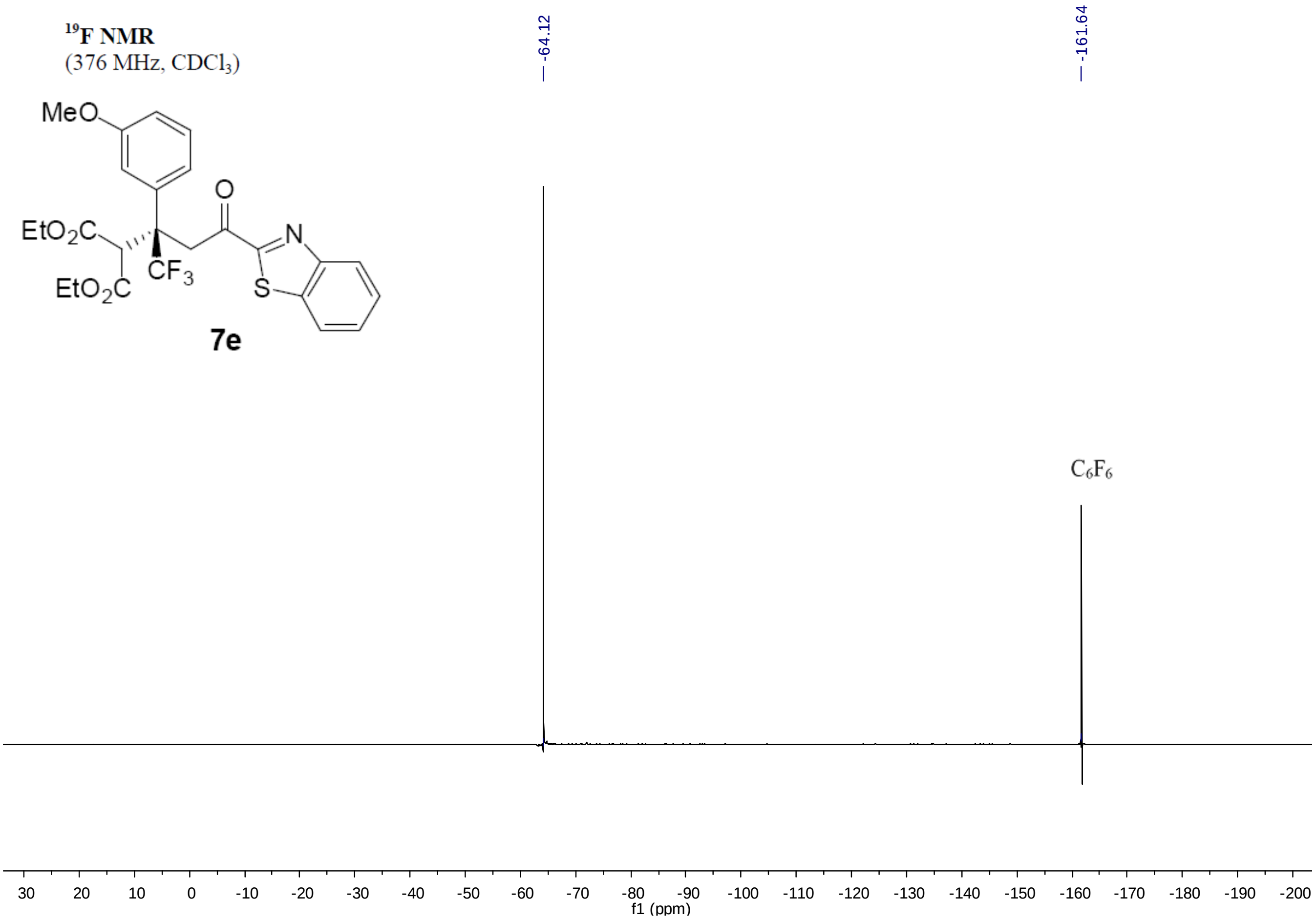
19F NMR
(376 MHz, CDCl3)
-64.12
-161.64
MeO
EtO2C
EtO2C
CF3
O
N
S
7e
C6F6
30 20 10 0 -10 -20 -30 -40 -50 -60 -70 -80 -90 -100 -110 -120 -130 -140 -150 -160 -170 -180 -190 -200
f1 (ppm)

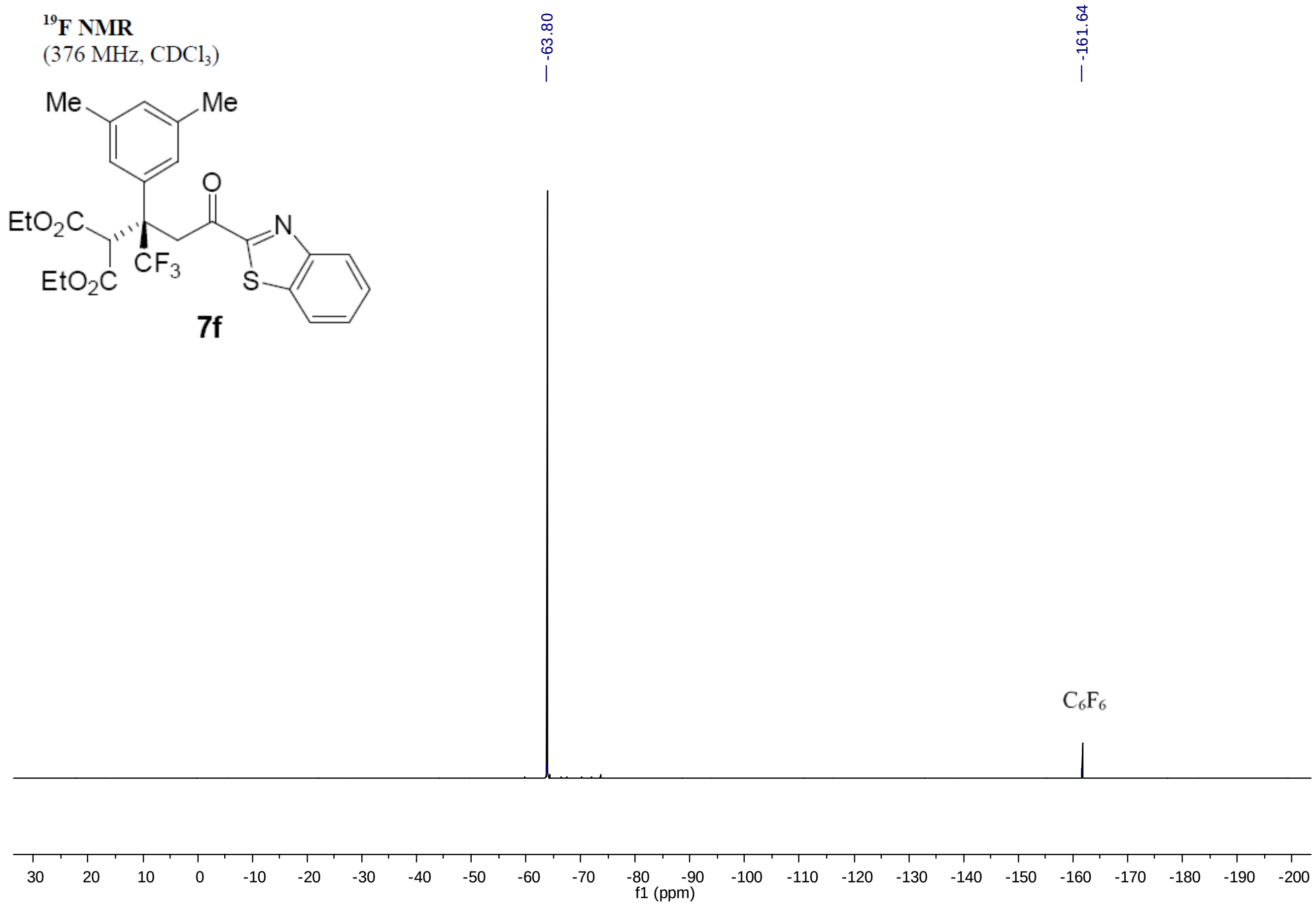
19F NMR
(376 MHz, CDCl3)
-63.80
-161.64
Me
Me
O
EtO2C
N
CF3
S
EtO2C
7f
C6F6
30 20 10 0 -10 -20 -30 -40 -50 -60 -70 -80 -90 -100 -110 -120 -130 -140 -150 -160 -170 -180 -190 -200
f1 (ppm)

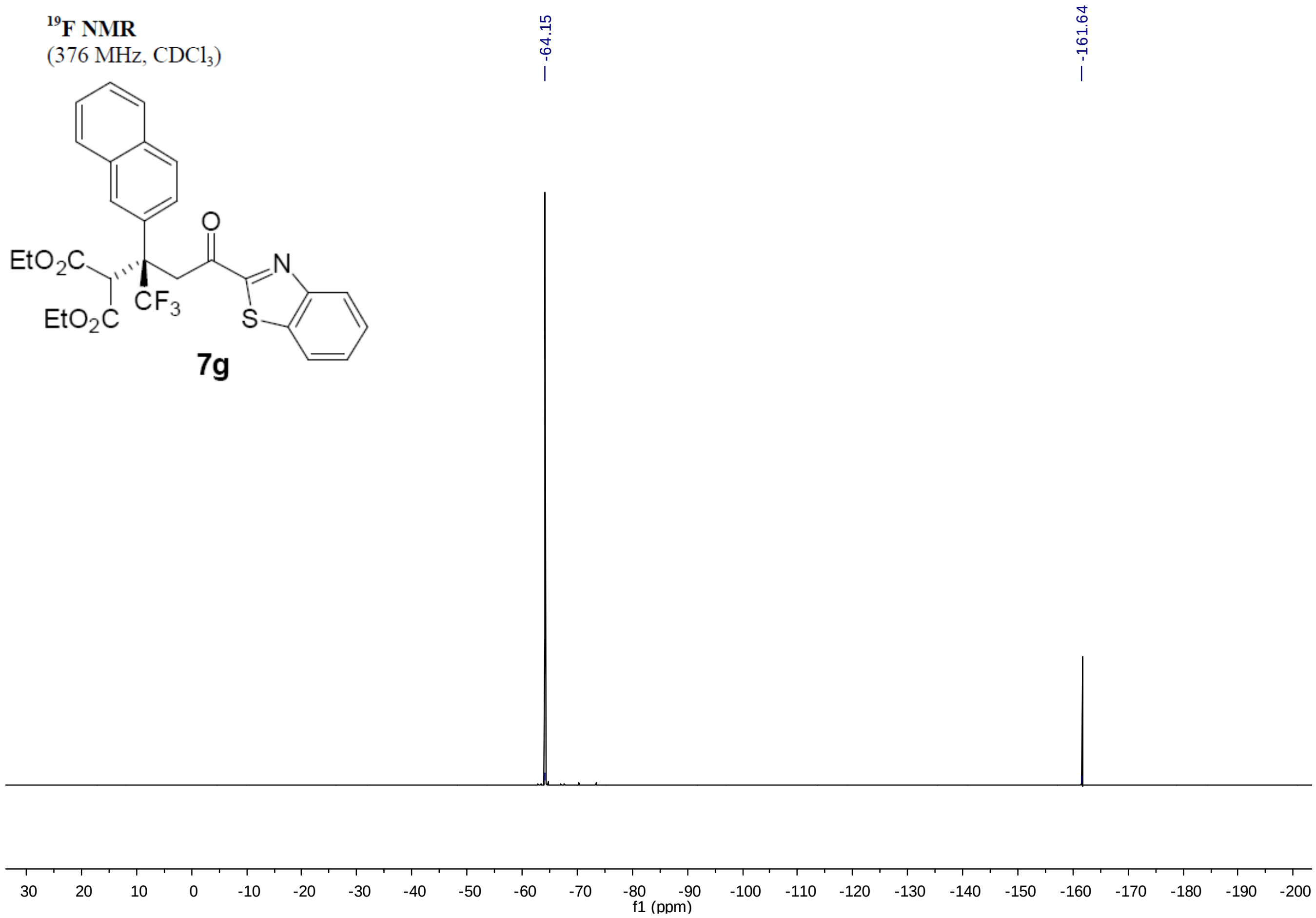
19F NMR
(376 MHz, CDCl3)
-64.15
-161.64
O
EtO2C
N
CF3
S
EtO2C
7g
30 20 10 0 -10 -20 -30 -40 -50 -60 -70 -80 -90 -100 -110 -120 -130 -140 -150 -160 -170 -180 -190 -200
f1 (ppm)

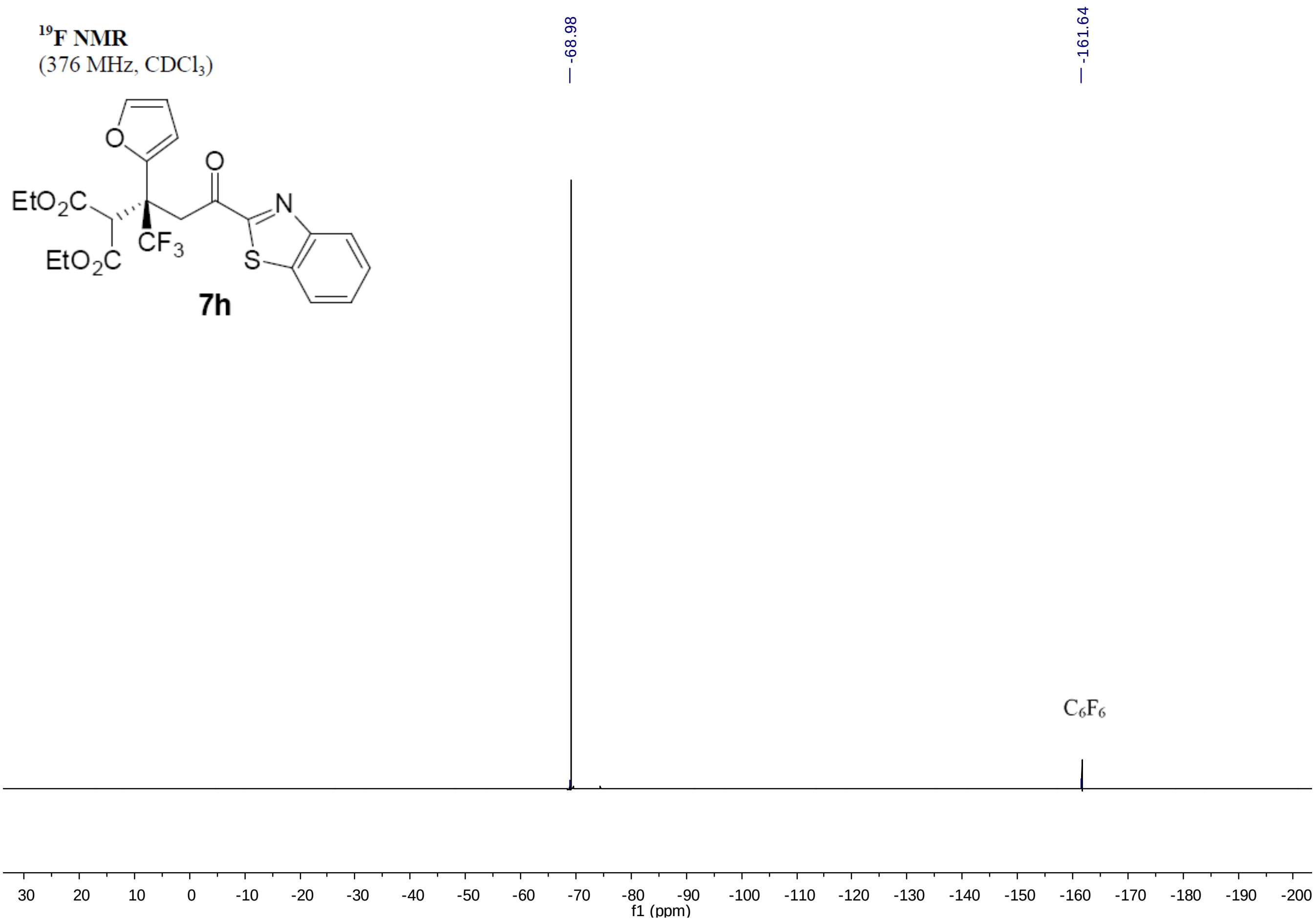

19F NMR
(376 MHz, CDCl3)
EtO2C
EtO2C
CF3
O
O
N
S
7h
-68.98
-161.64
C6F6
30 20 10 0 -10 -20 -30 -40 -50 -60 -70 -80 -90 -100 -110 -120 -130 -140 -150 -160 -170 -180 -190 -200
f1 (ppm)

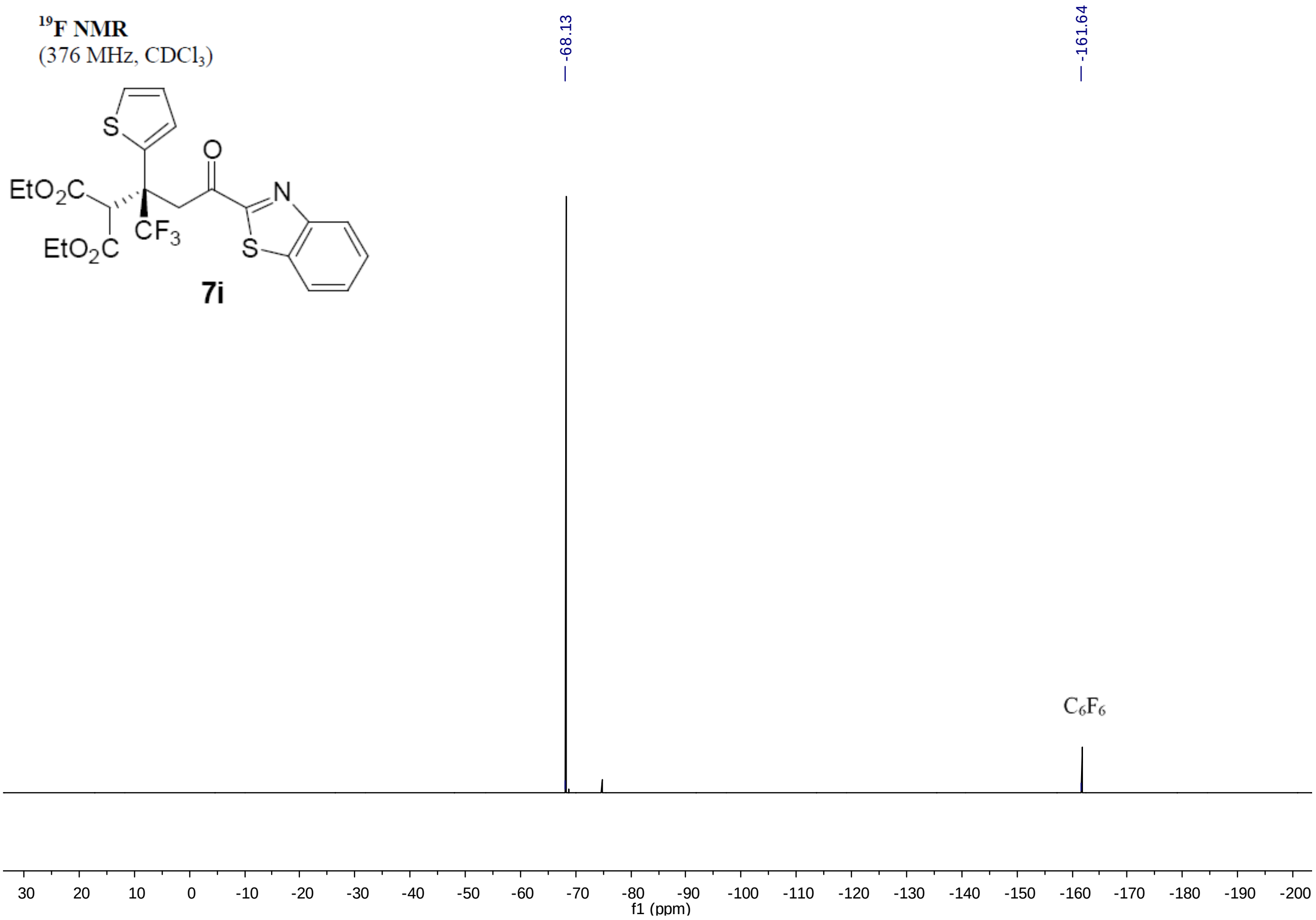

19F NMR
(376 MHz, CDCl3)
EtO2C
EtO2C
CF3
S
O
N
S
7i
-68.13
-161.64
C6F6
30 20 10 0 -10 -20 -30 -40 -50 -60 -70 -80 -90 -100 -110 -120 -130 -140 -150 -160 -170 -180 -190 -200
f1 (ppm)

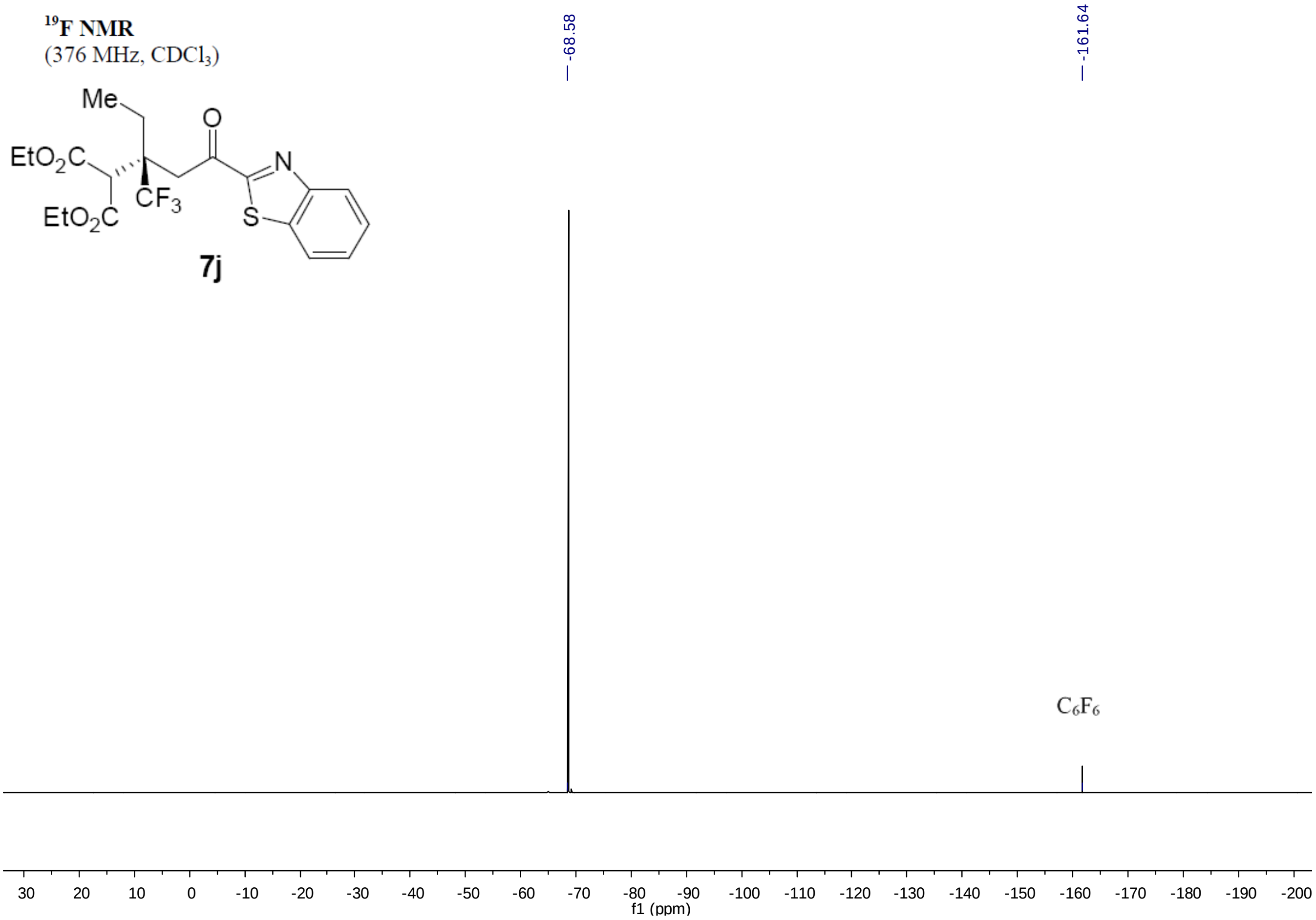
19F NMR
(376 MHz, CDCl3)
Me
EtO2C
EtO2C
CF3
O
N
S
7j
-68.58
-161.64
C6F6
30 20 10 0 -10 -20 -30 -40 -50 -60 -70 -80 -90 -100 -110 -120 -130 -140 -150 -160 -170 -180 -190 -200
f1 (ppm)

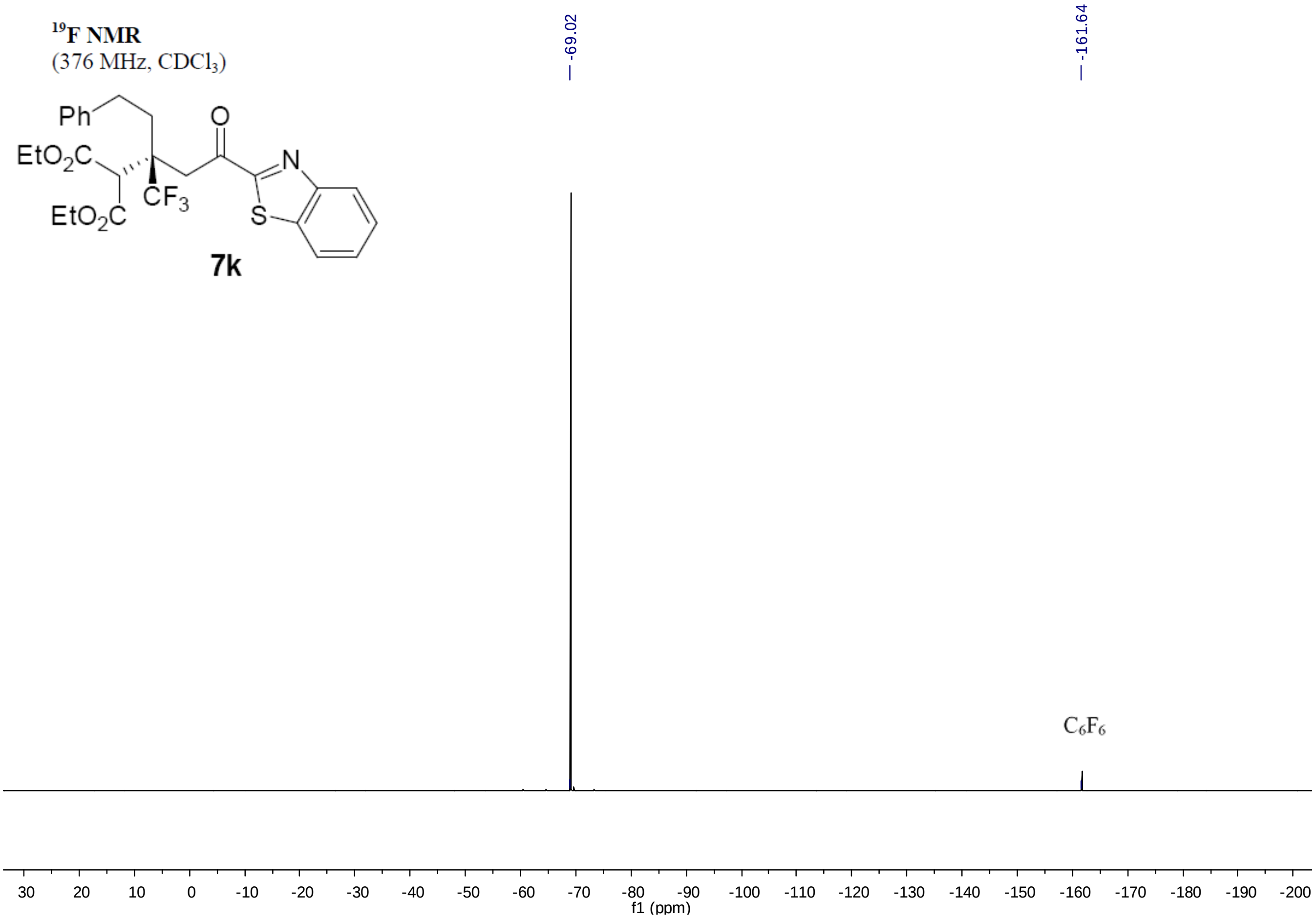
19F NMR
(376 MHz, CDCl3)
Ph
EtO2C
EtO2C
CF3
O
N
S
7k
-69.02
-161.64
C6F6
30 20 10 0 -10 -20 -30 -40 -50 -60 -70 -80 -90 -100 -110 -120 -130 -140 -150 -160 -170 -180 -190 -200
f1 (ppm)

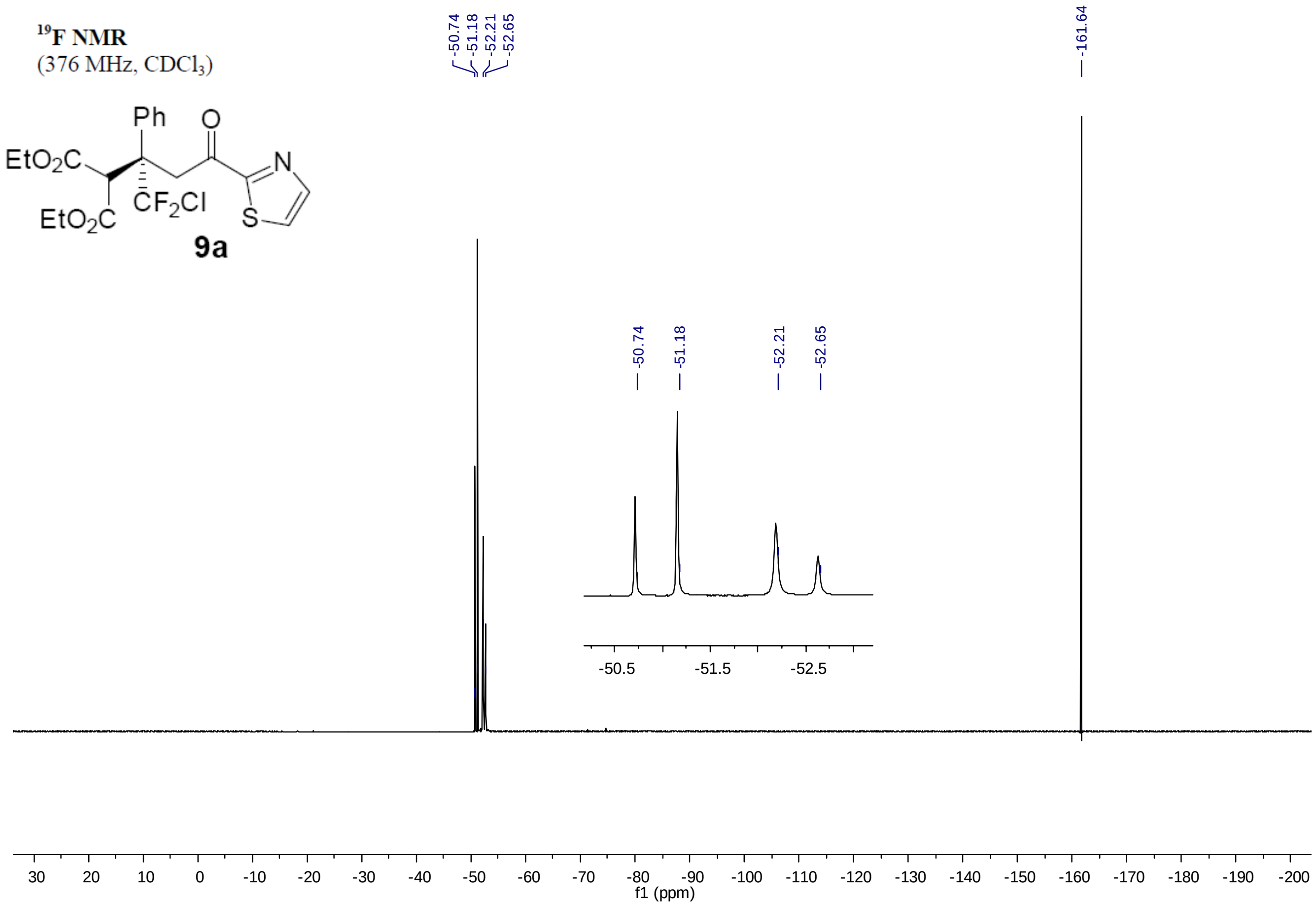
19F NMR
(376 MHz, CDCl3)
Ph
O
EtO2C
N
EtO2C
CF2Cl
S
9a
-50.74
-51.18
-52.21
-52.65
-161.64
-50.5
-51.5
-52.5
f1 (ppm)

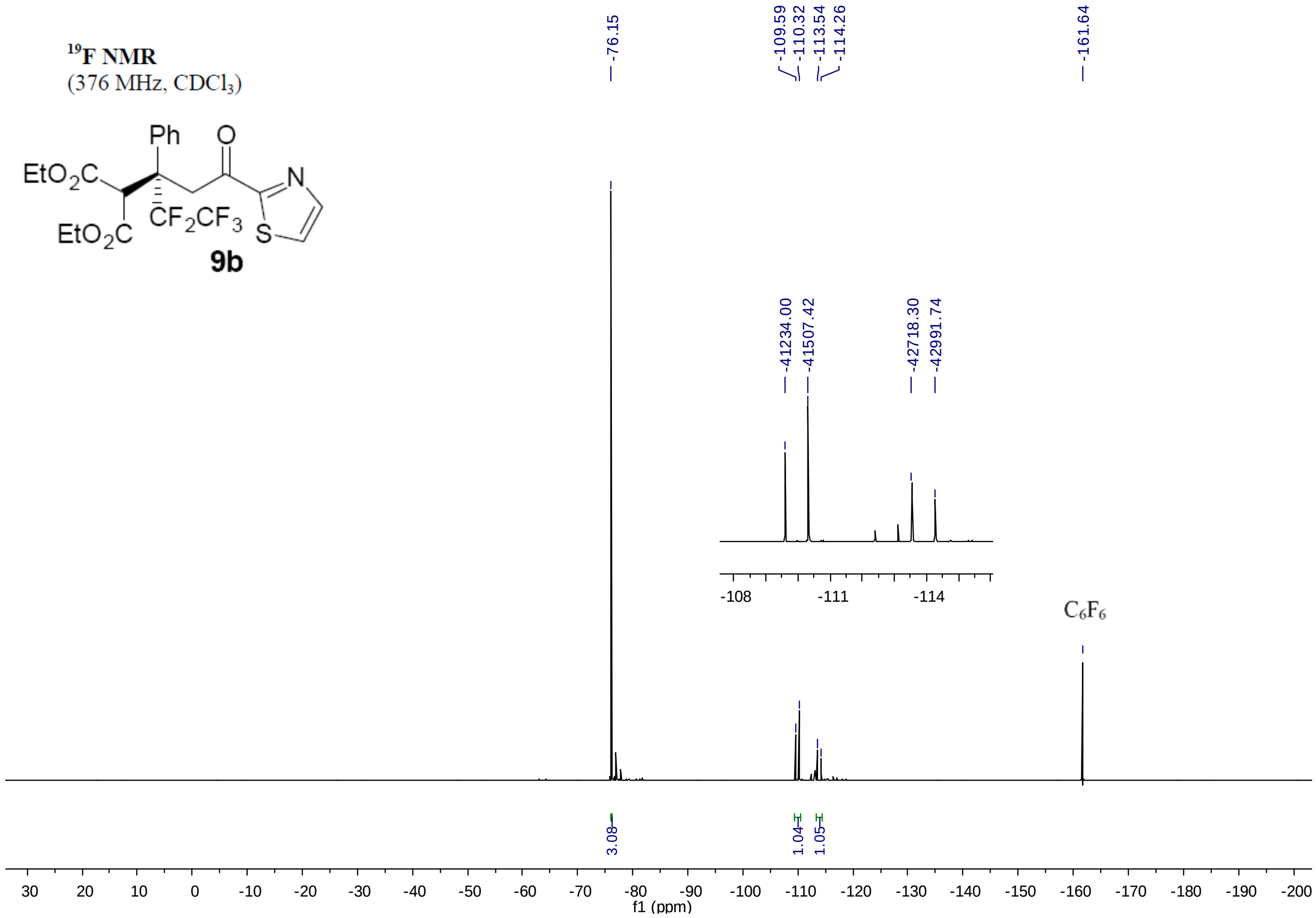
19F NMR
(376 MHz, CDCl3)
Ph
O
EtO2C
N
EtO2C
CF2CF3
S
9b
-76.15
-109.59
-110.32
-113.54
-114.26
-161.64
-41234.00
-41507.42
-42718.30
-42991.74
-108
-111
-114
C6F6
3.08
1.04
1.05
f1 (ppm)

Copies of DEPT 135 spectra - malonate adducts 3b-3l, 5a-5k, 5m-5u, 7a-7k, 9a, 9b

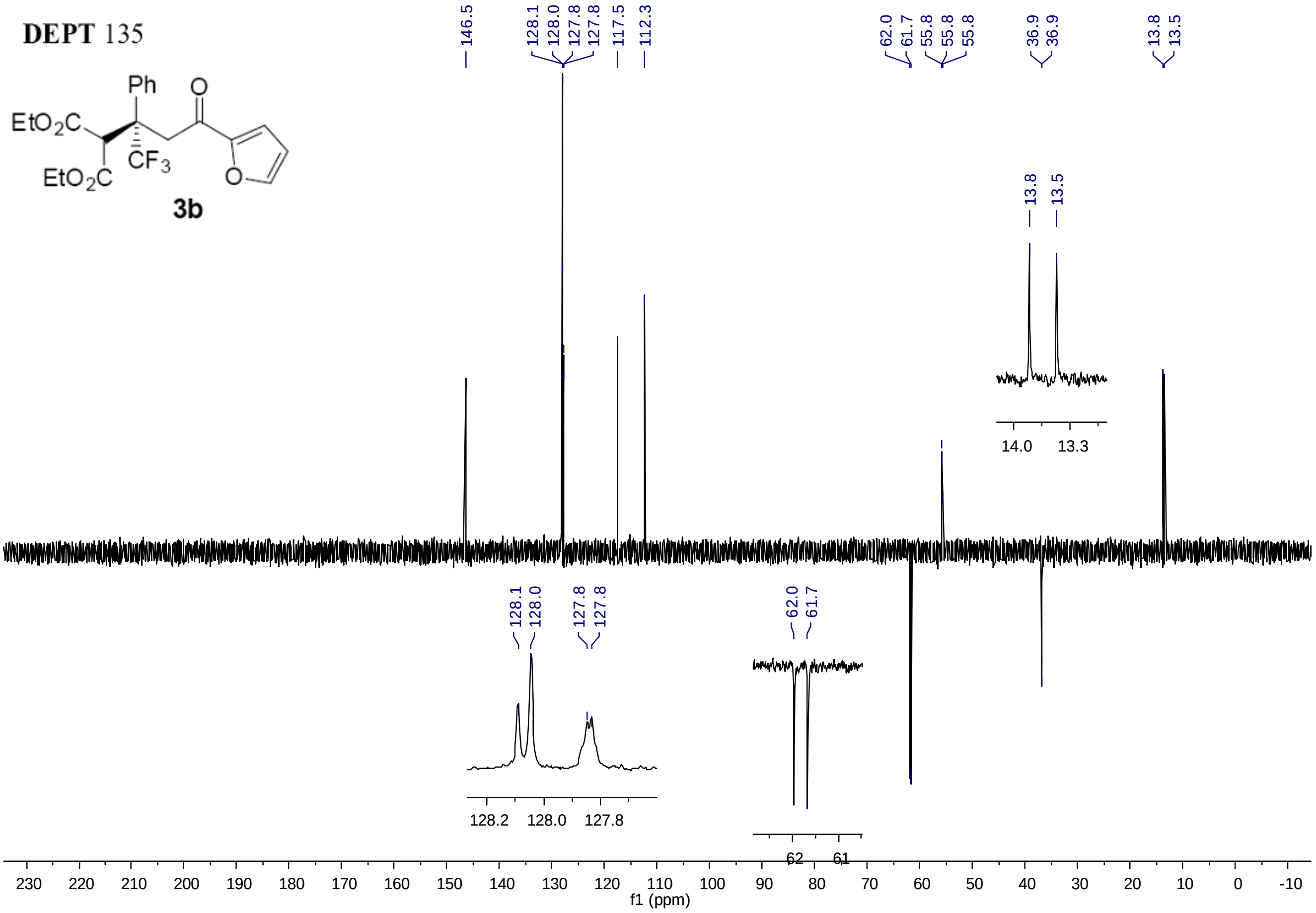

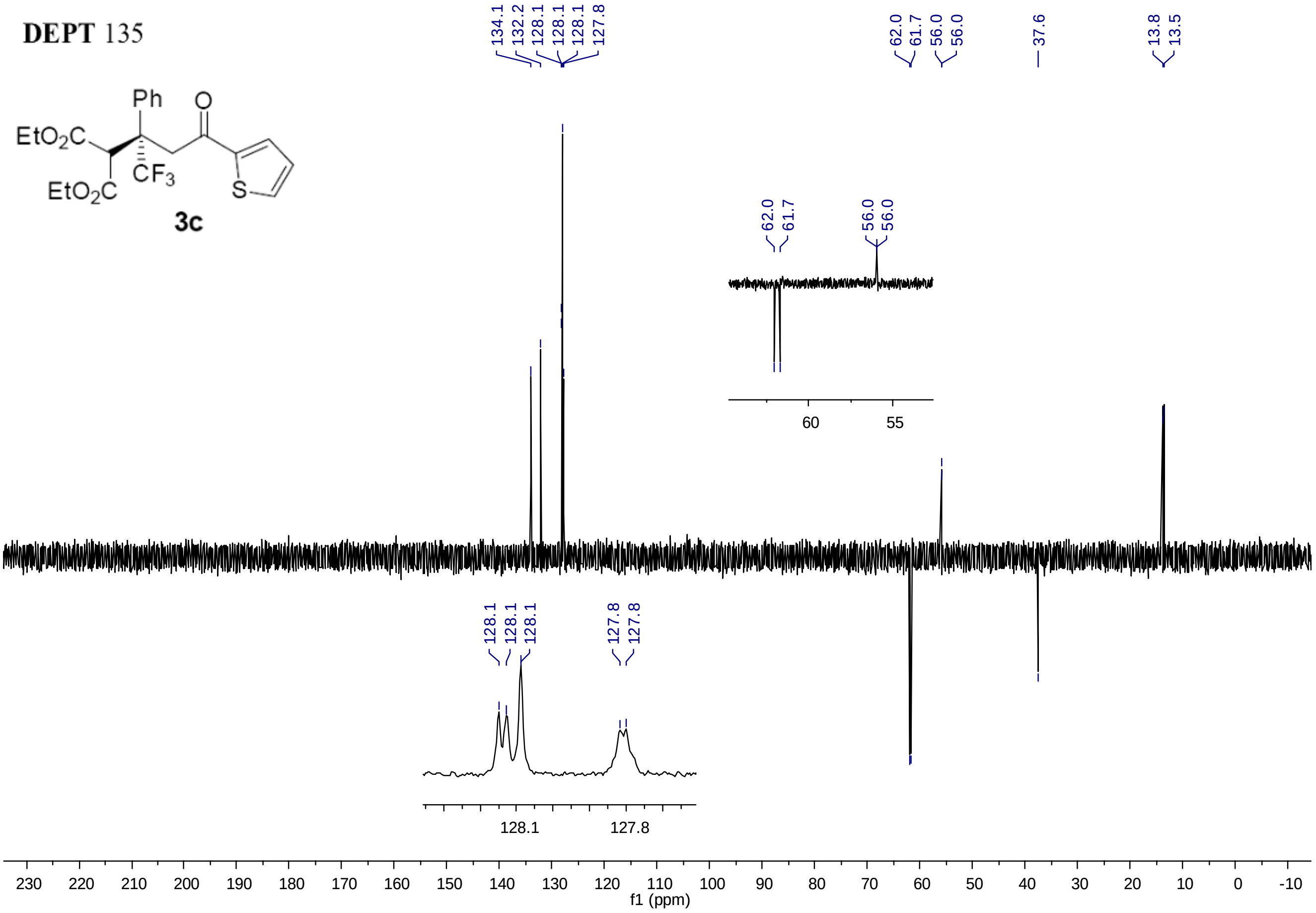

DEPT 135

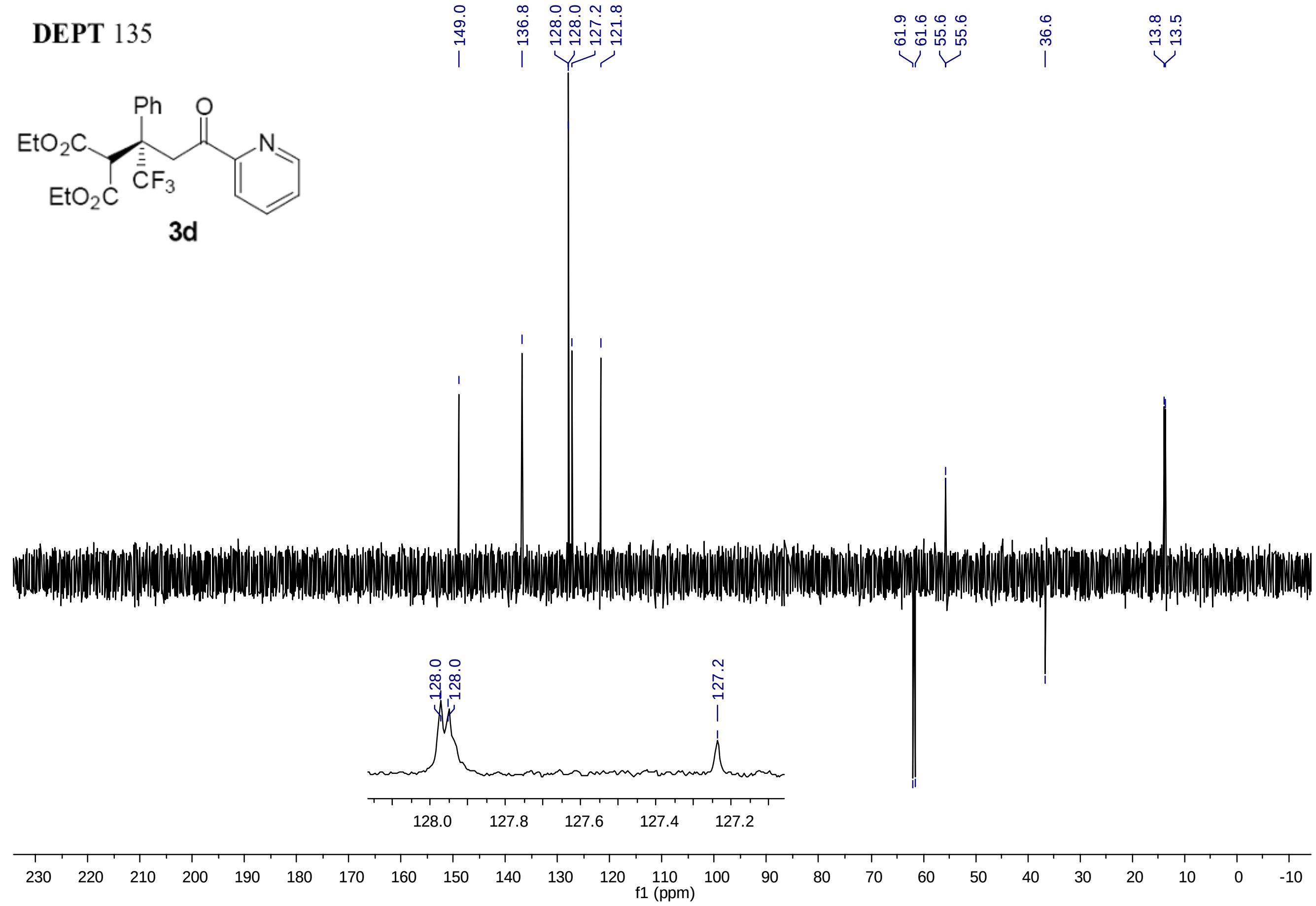

DEPT 135

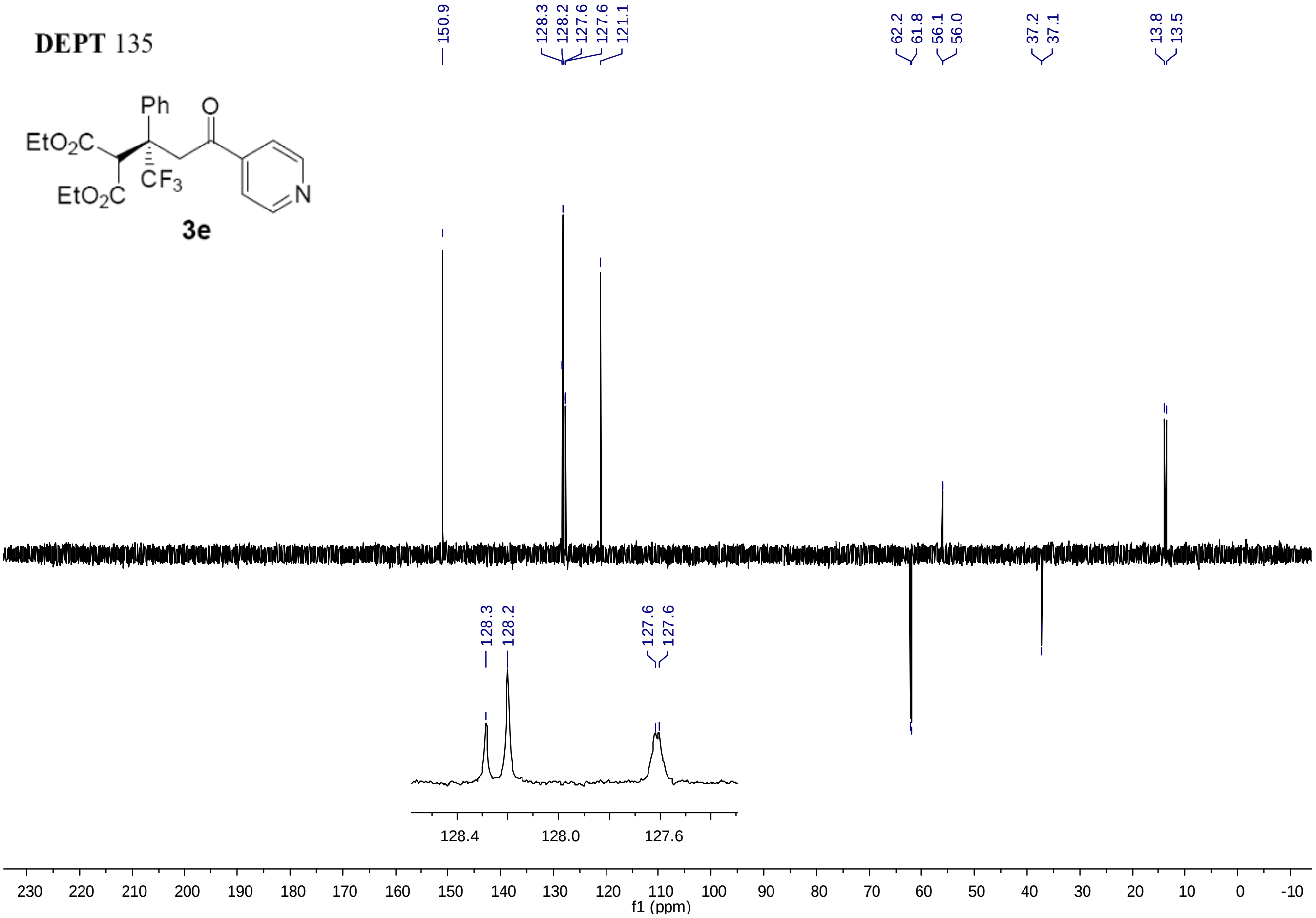

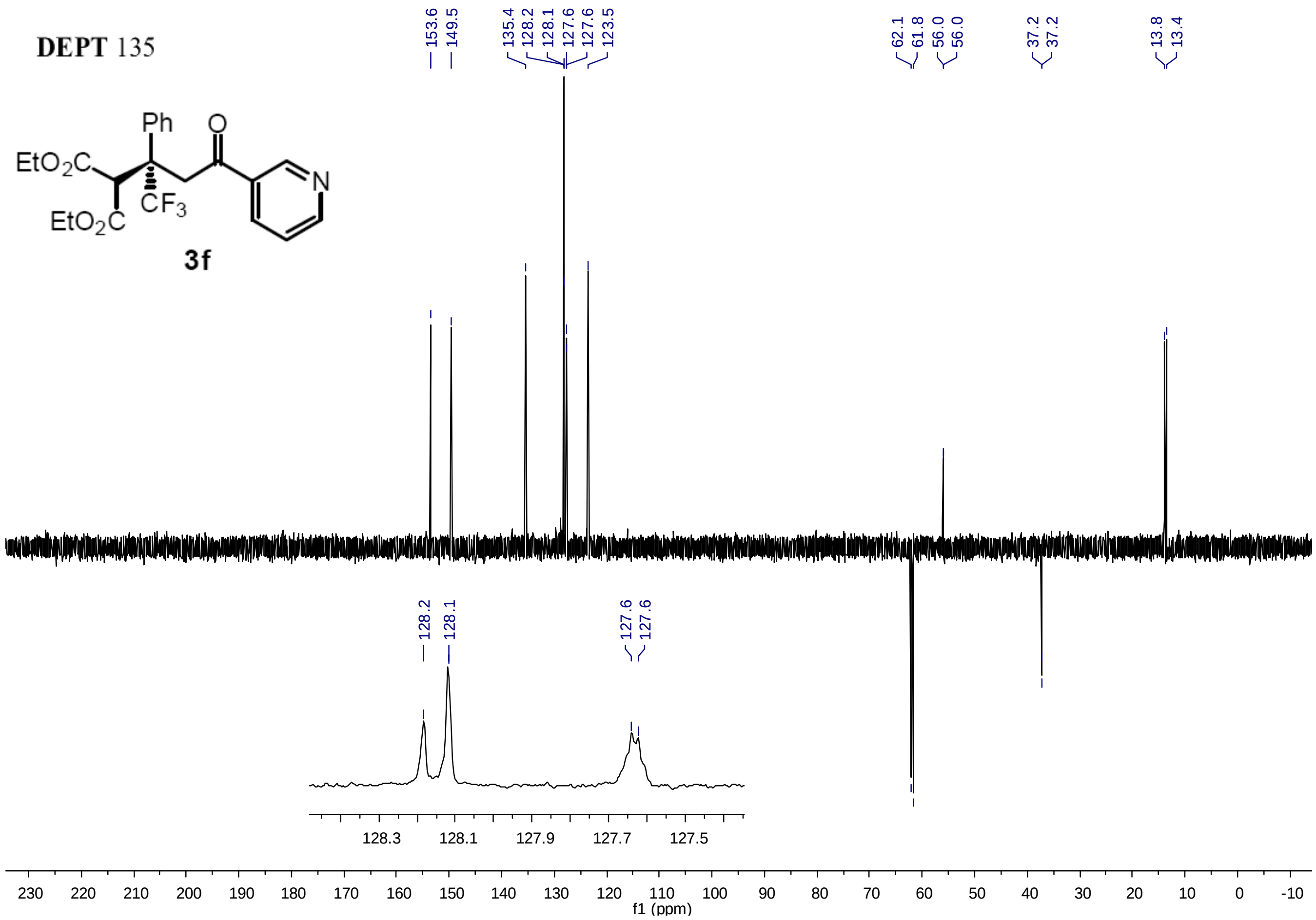
DEPT 135
Ph
O
EtO2C
CF3
EtO2C
N
3f
153.6
149.5
135.4
128.2
128.1
127.6
127.6
123.5
62.1
61.8
56.0
56.0
37.2
37.2
13.8
13.4
128.2
128.1
127.6
127.6
128.3
128.1
127.9
127.7
127.5
230 220 210 200 190 180 170 160 150 140 130 120 110 100 90 80 70 60 50 40 30 20 10 0 -10
f1 (ppm)

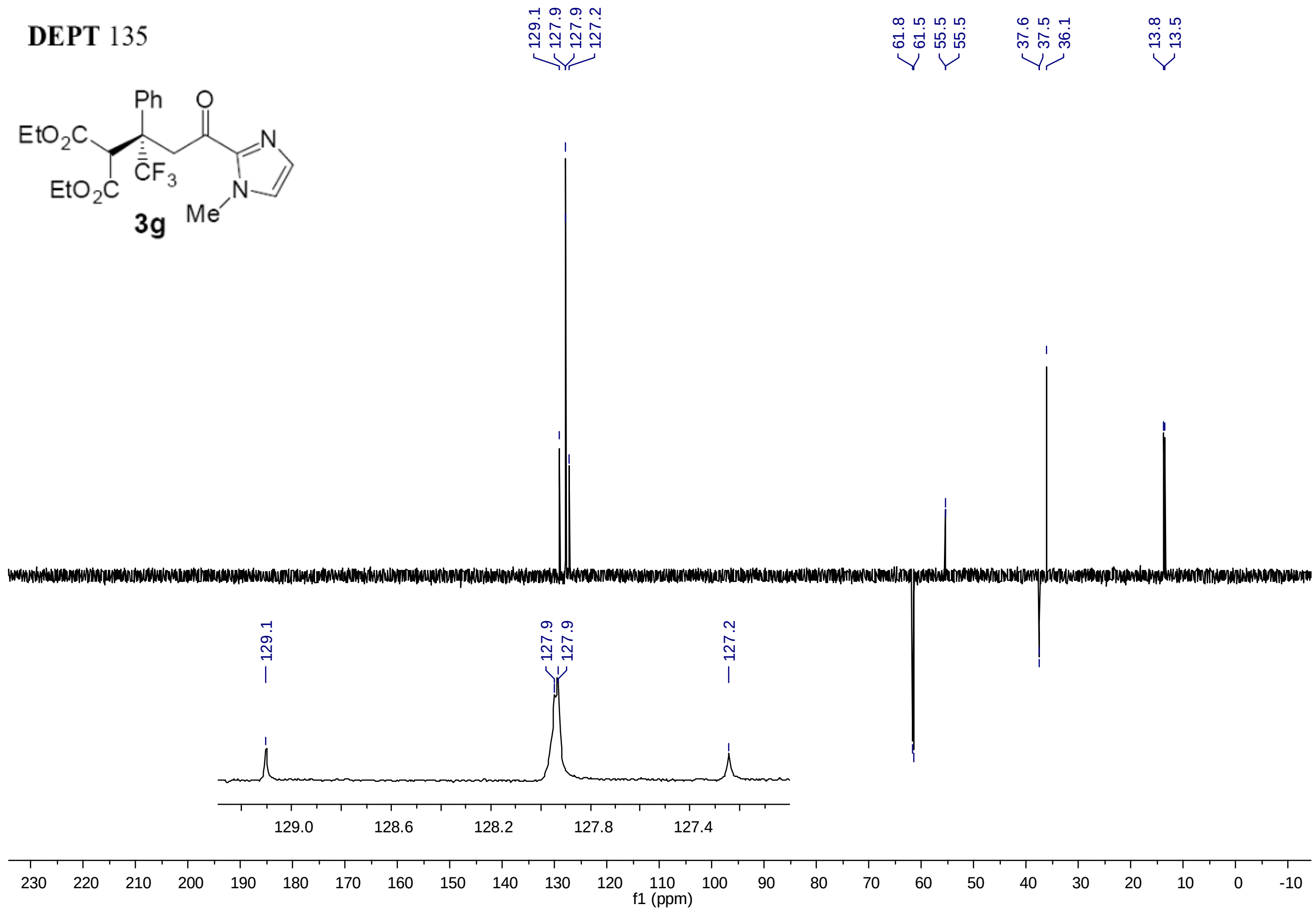
DEPT 135
Ph
O
EtO2C
CF3
EtO2C
N
N
Me
3g
129.1
127.9
127.9
127.2
61.8
61.5
55.5
55.5
37.6
37.5
36.1
13.8
13.5
129.1
127.9
127.9
127.2
129.0
128.6
128.2
127.8
127.4
230 220 210 200 190 180 170 160 150 140 130 120 110 100 90 80 70 60 50 40 30 20 10 0 -10
f1 (ppm)

DEPT 135

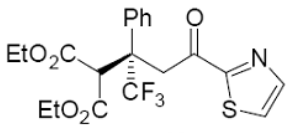

3h

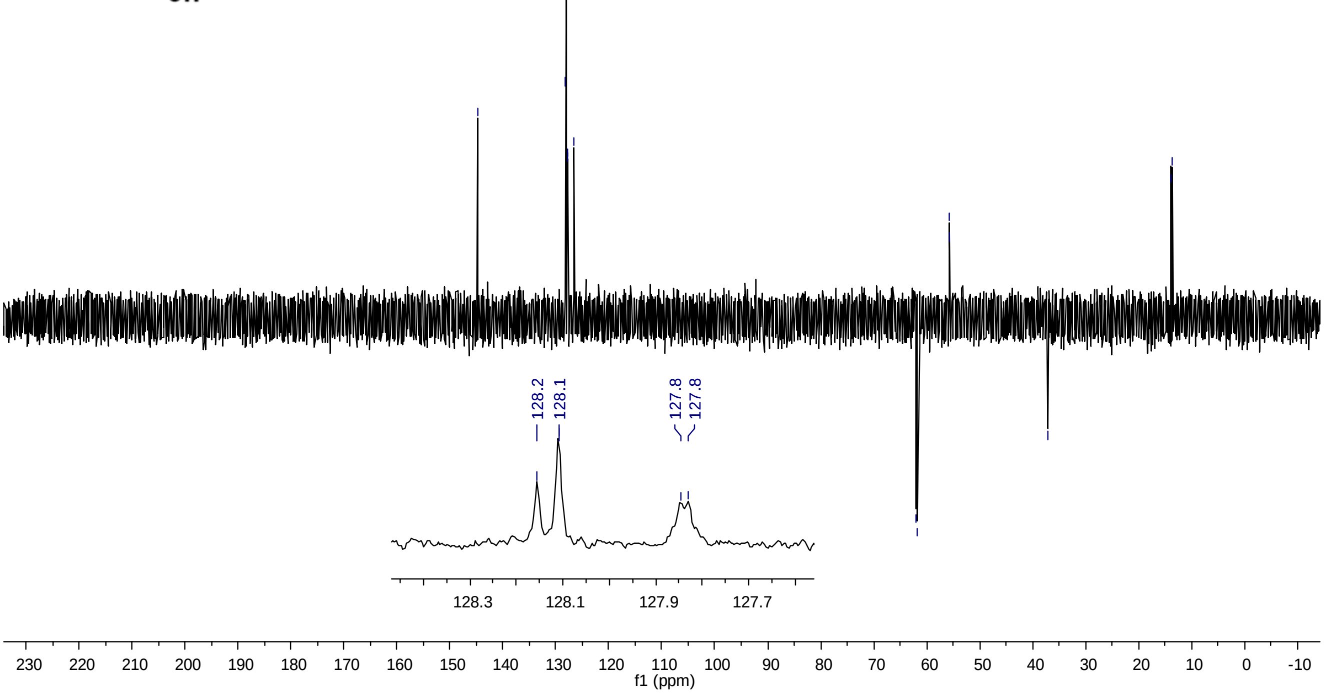

DEPT 135

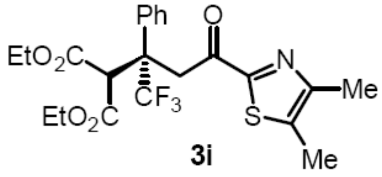

3i

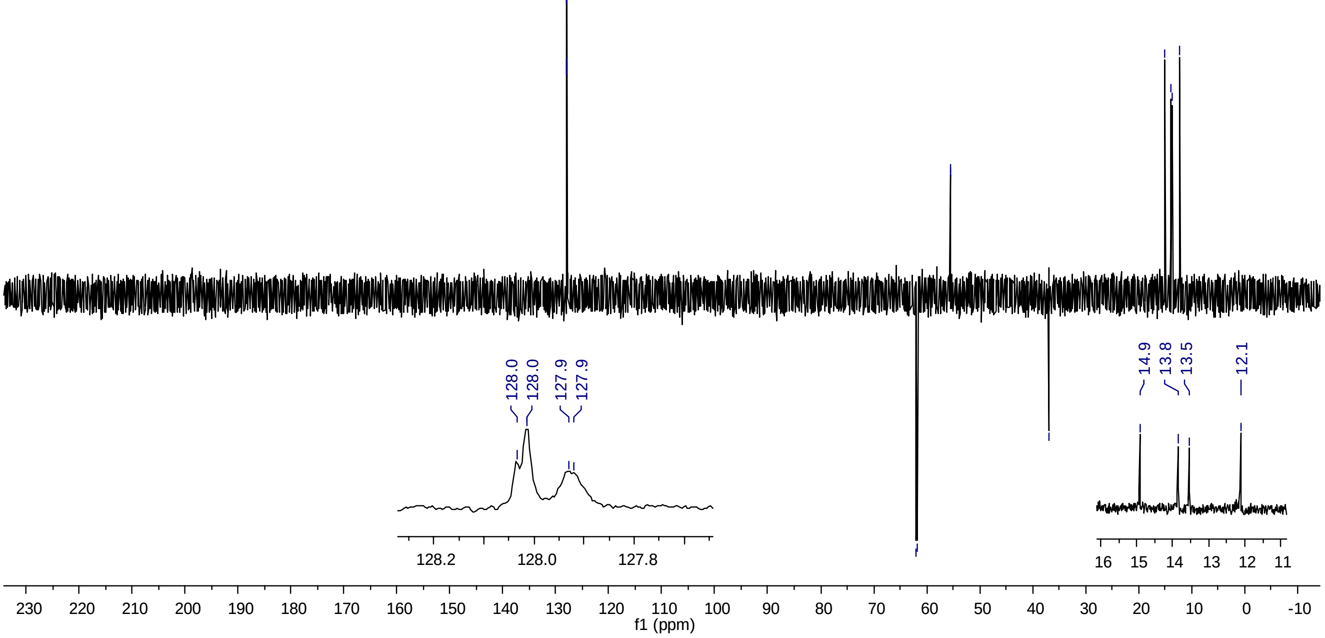

DEPT 135

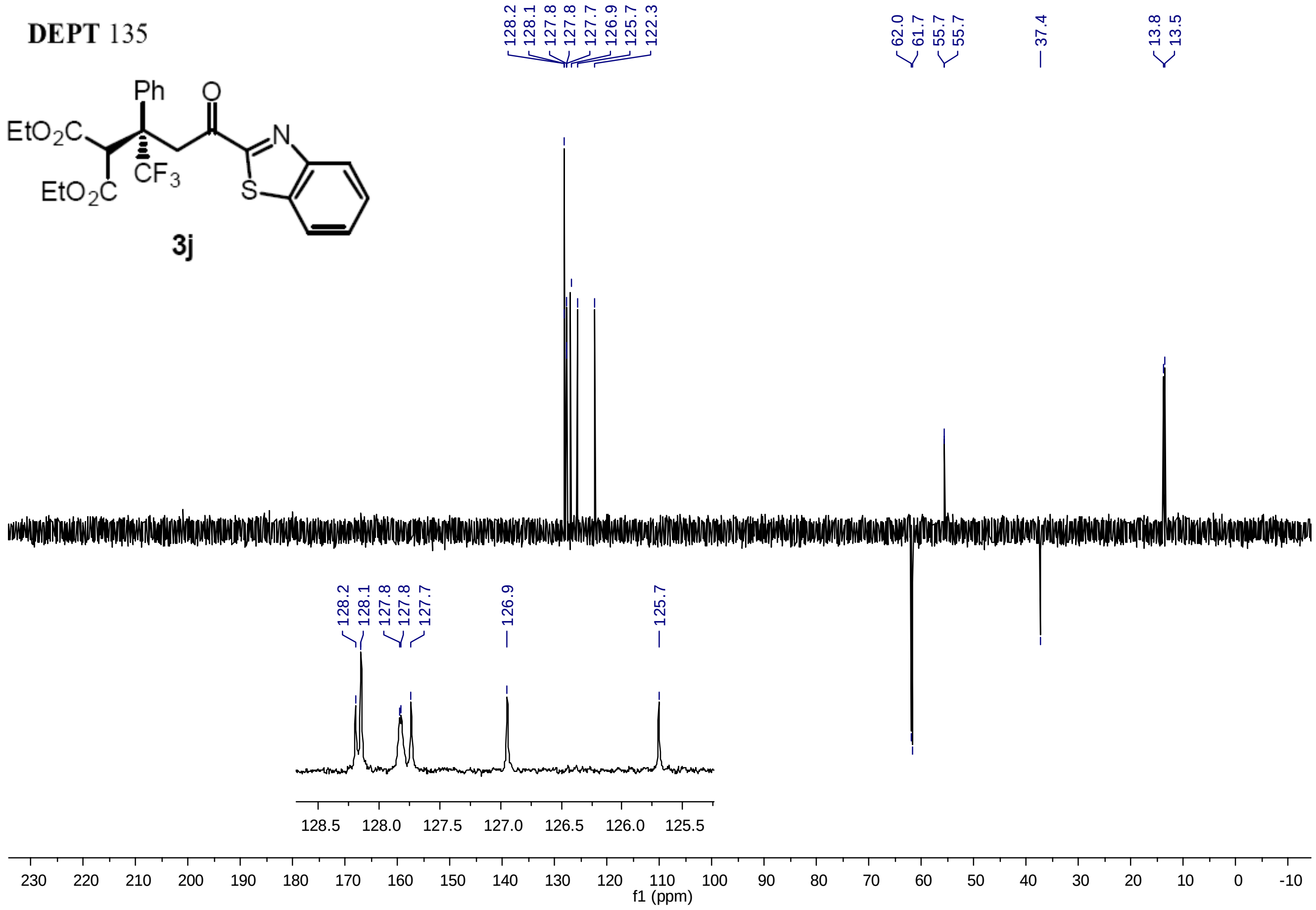

DEPT 135

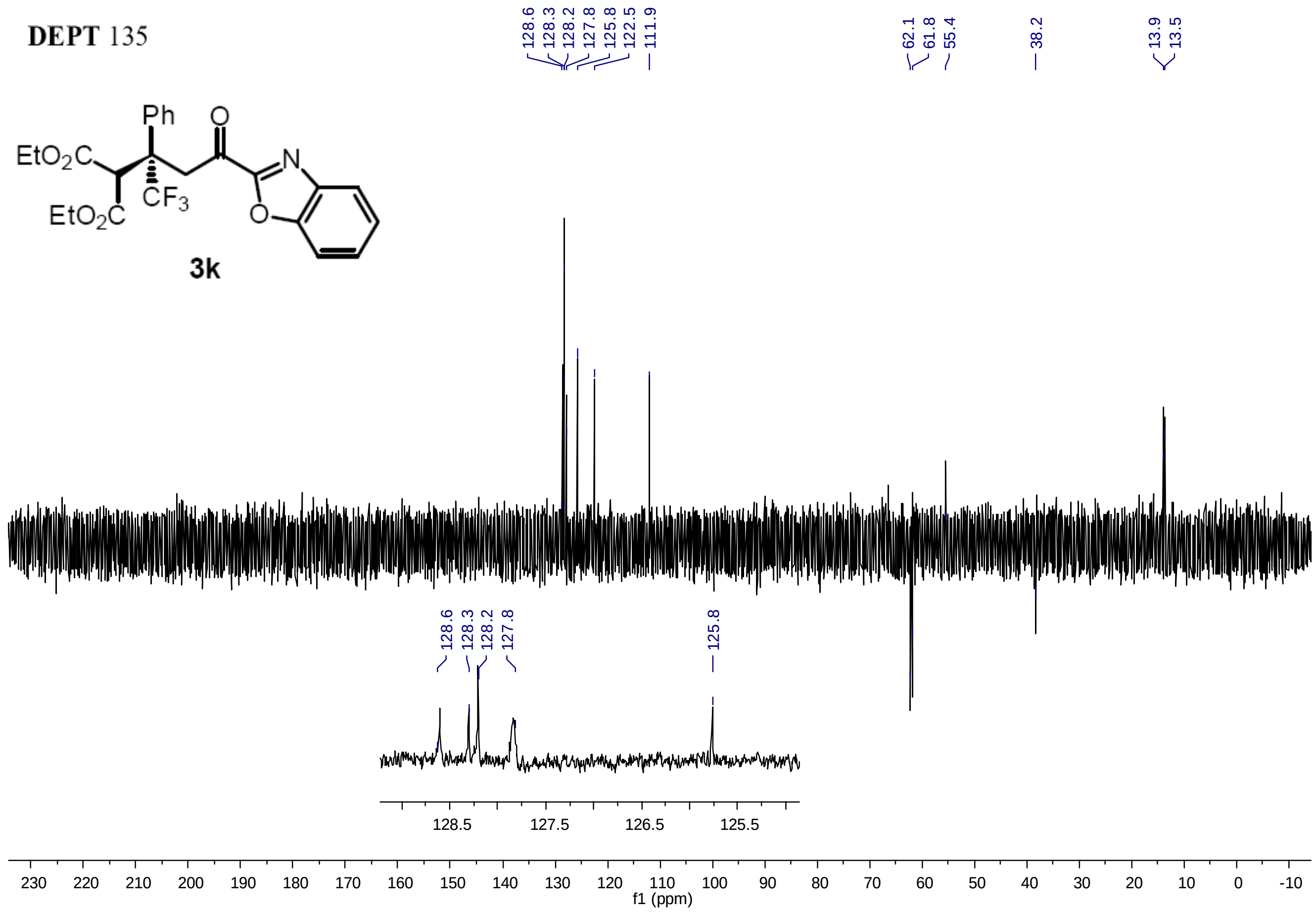

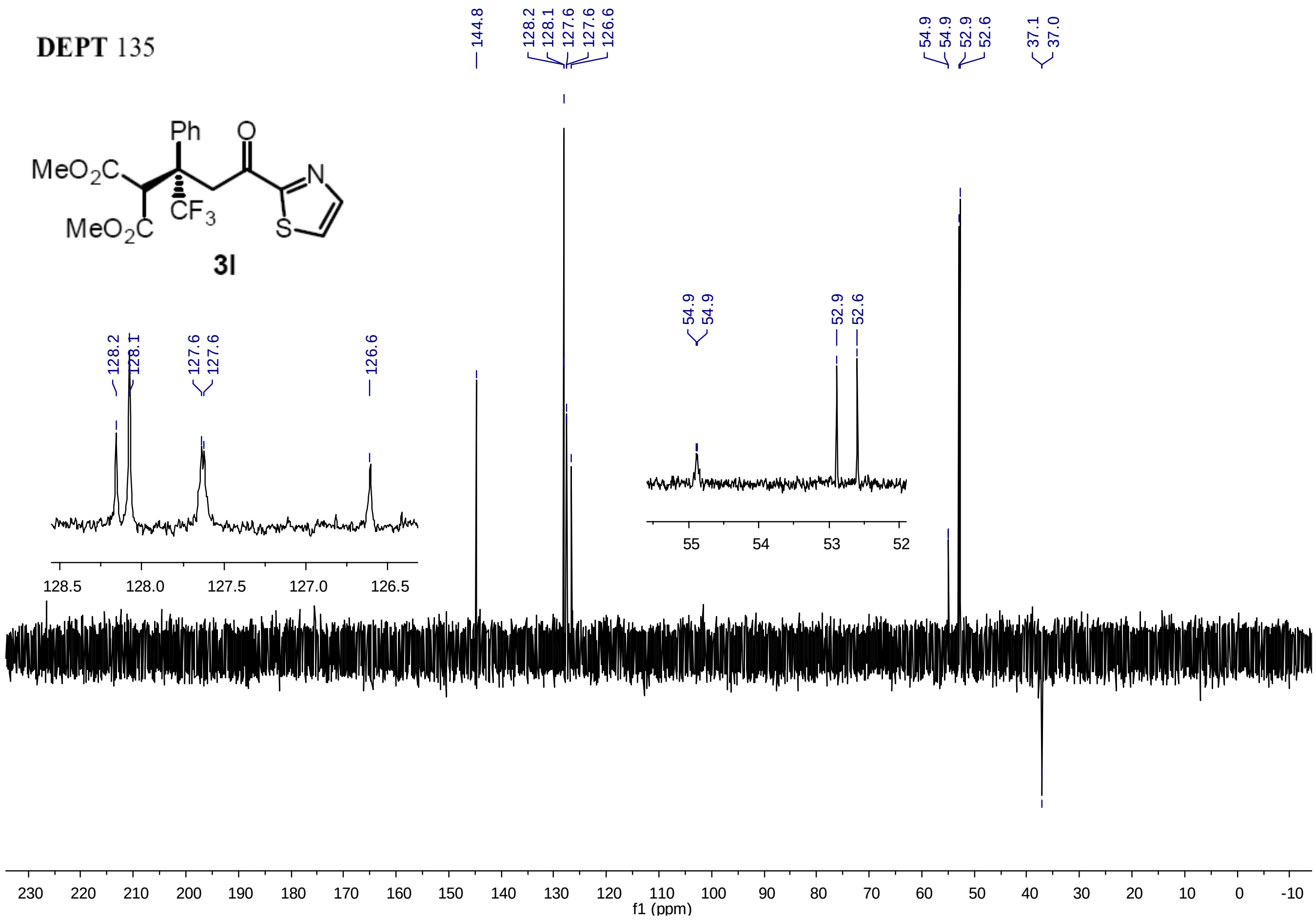
DEPT 135
3l
144.8
128.2
128.1
127.6
127.6
126.6
54.9
54.9
52.9
52.6
37.1
37.0
f1 (ppm)

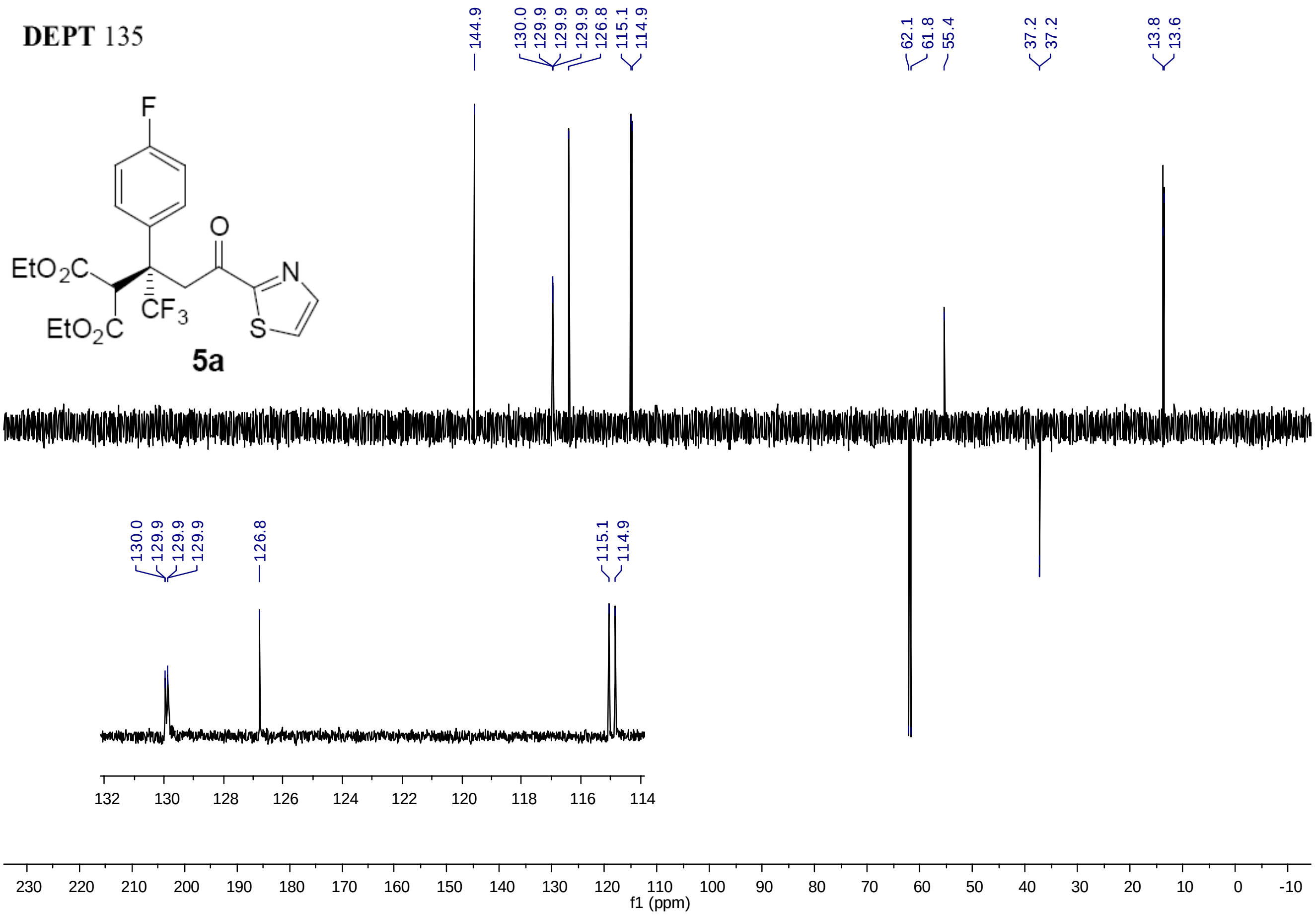
DEPT 135
5a
144.9
130.0
129.9
129.9
129.9
126.8
115.1
114.9
62.1
61.8
55.4
37.2
37.2
13.8
13.6
f1 (ppm)

DEPT 135

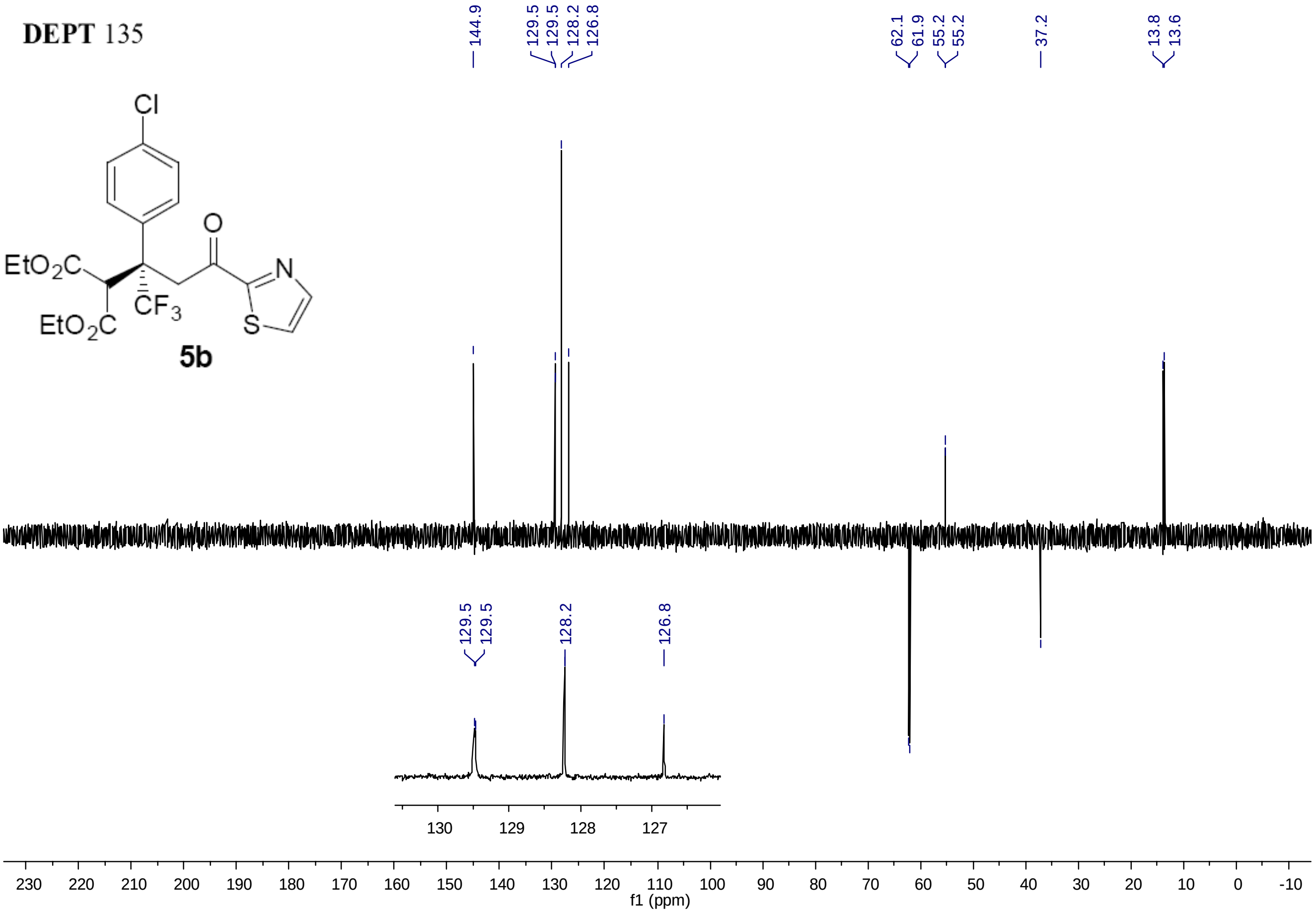

DEPT 135

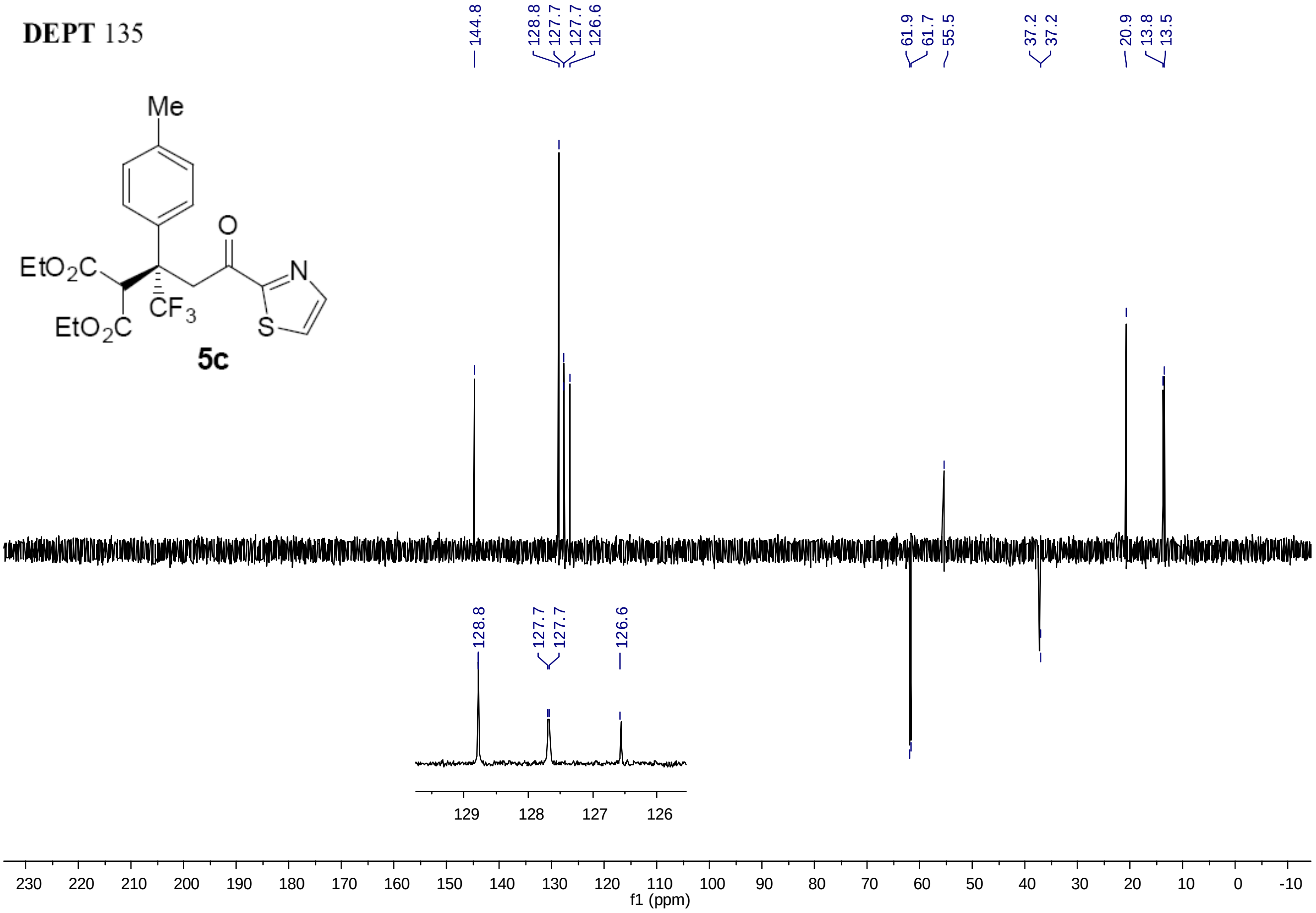

DEPT 135

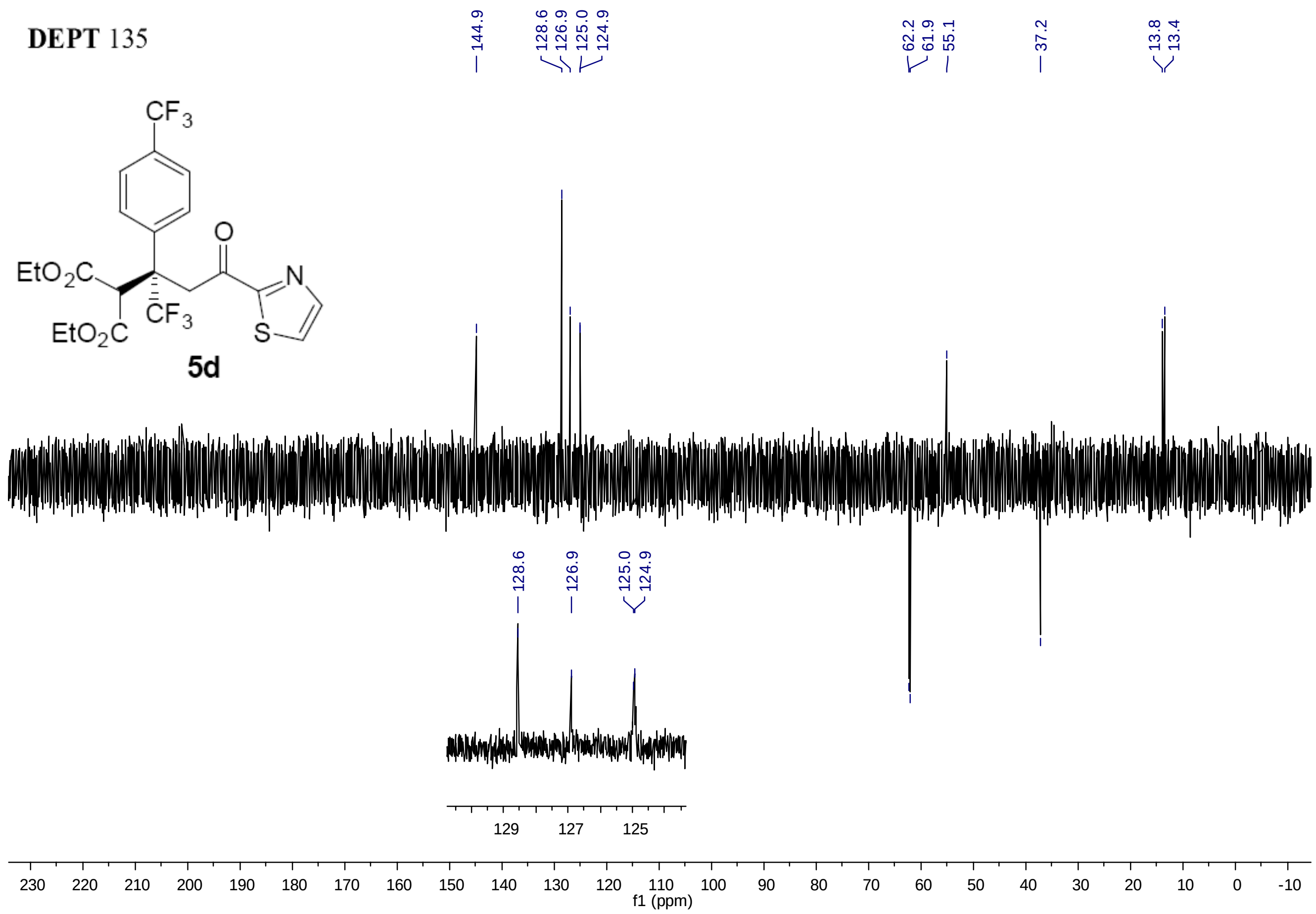

DEPT 135

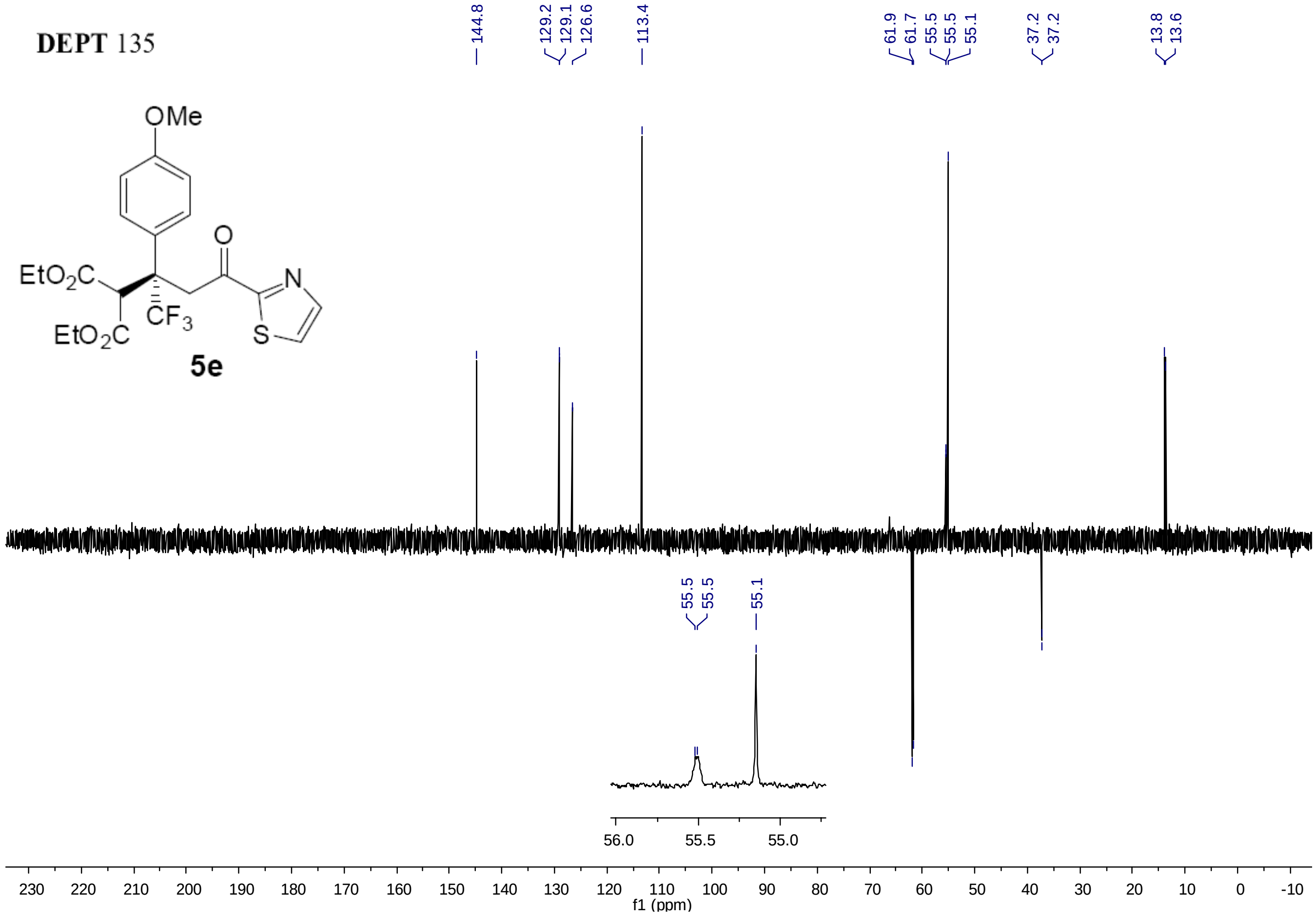

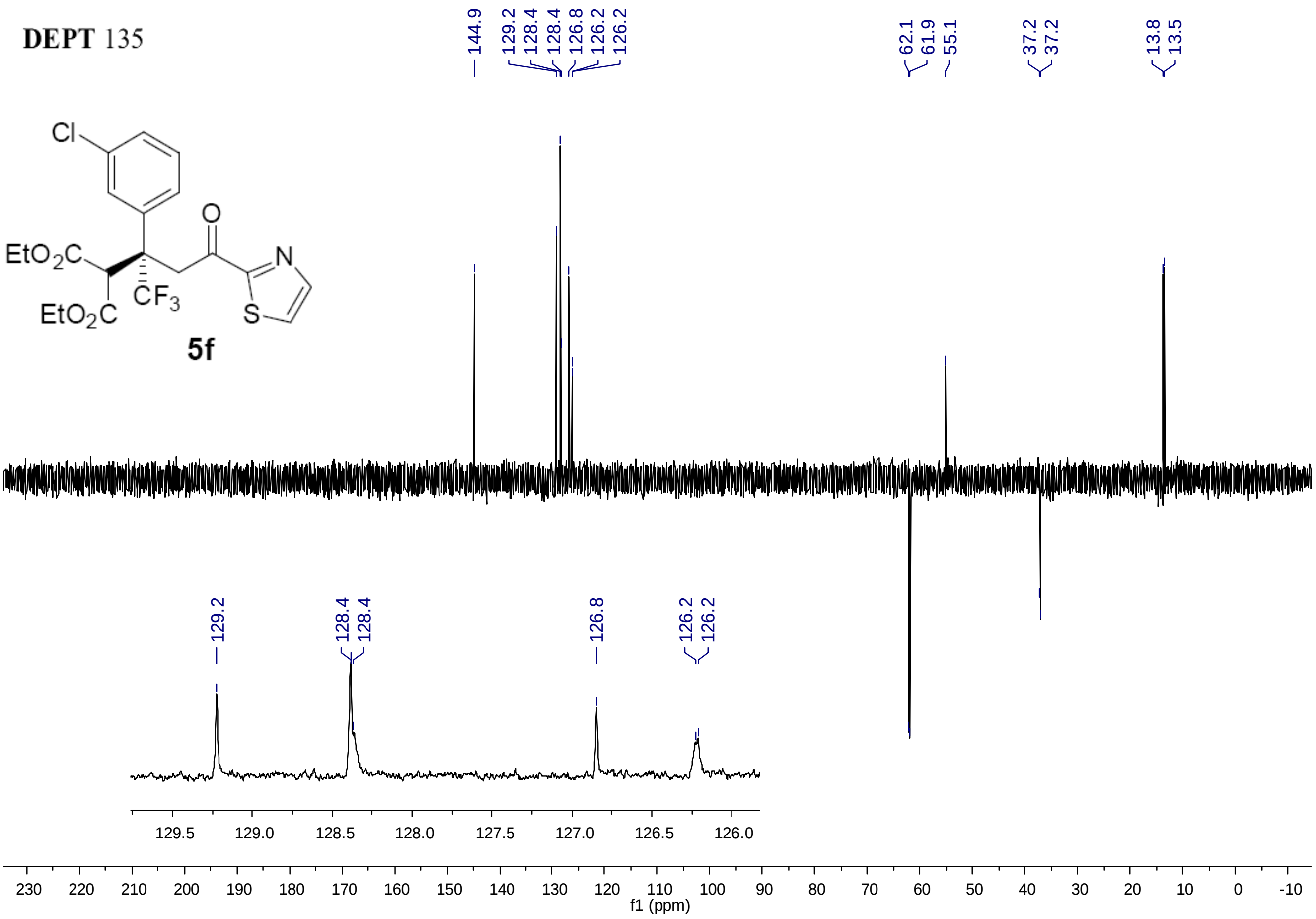
DEPT 135
5f
144.9
129.2
128.4
128.4
126.8
126.2
126.2
62.1
61.9
55.1
37.2
37.2
13.8
13.5
f1 (ppm)

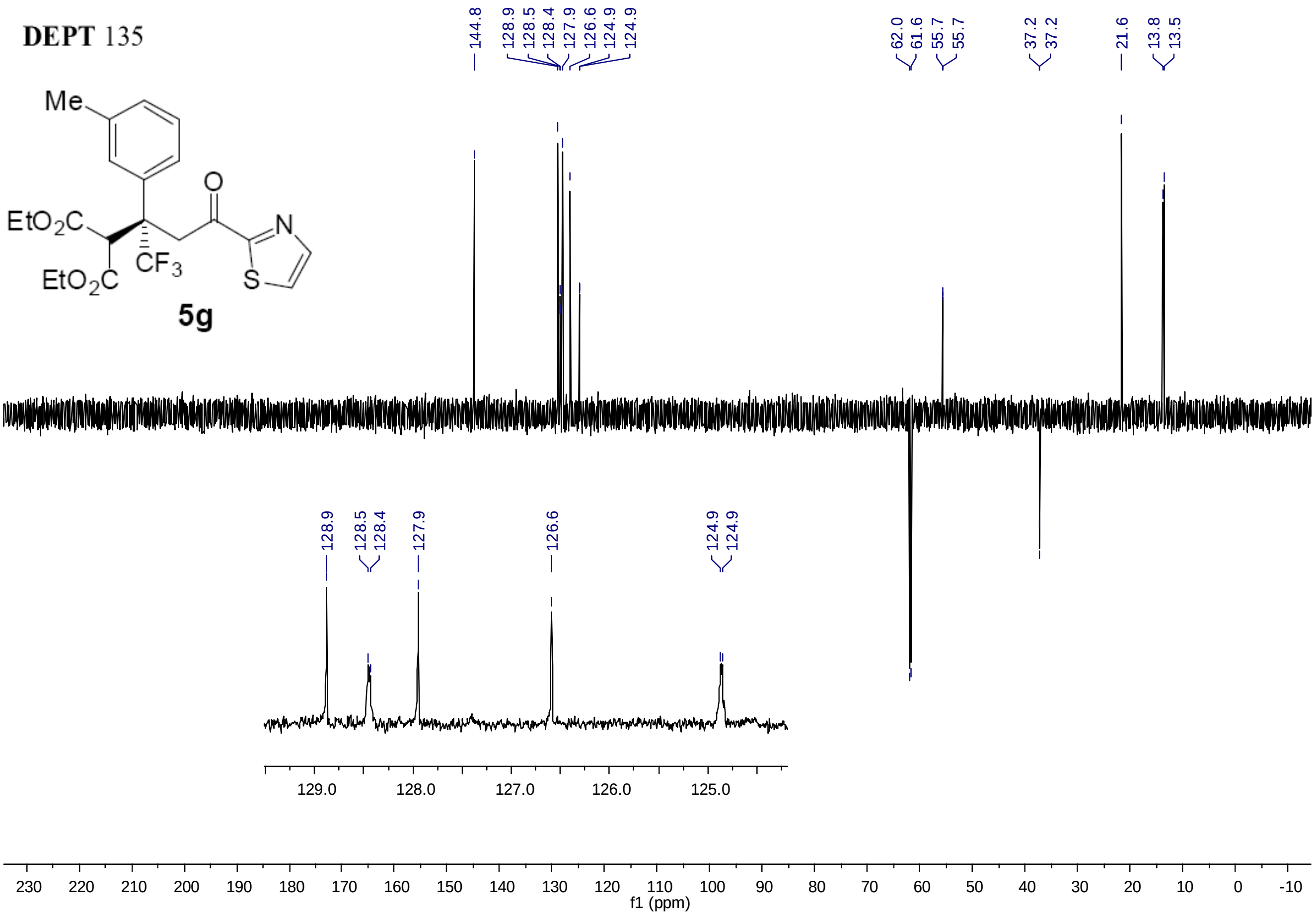
DEPT 135
5g
144.8
128.9
128.5
128.4
127.9
126.6
124.9
124.9
62.0
61.6
55.7
55.7
37.2
37.2
21.6
13.8
13.5
f1 (ppm)

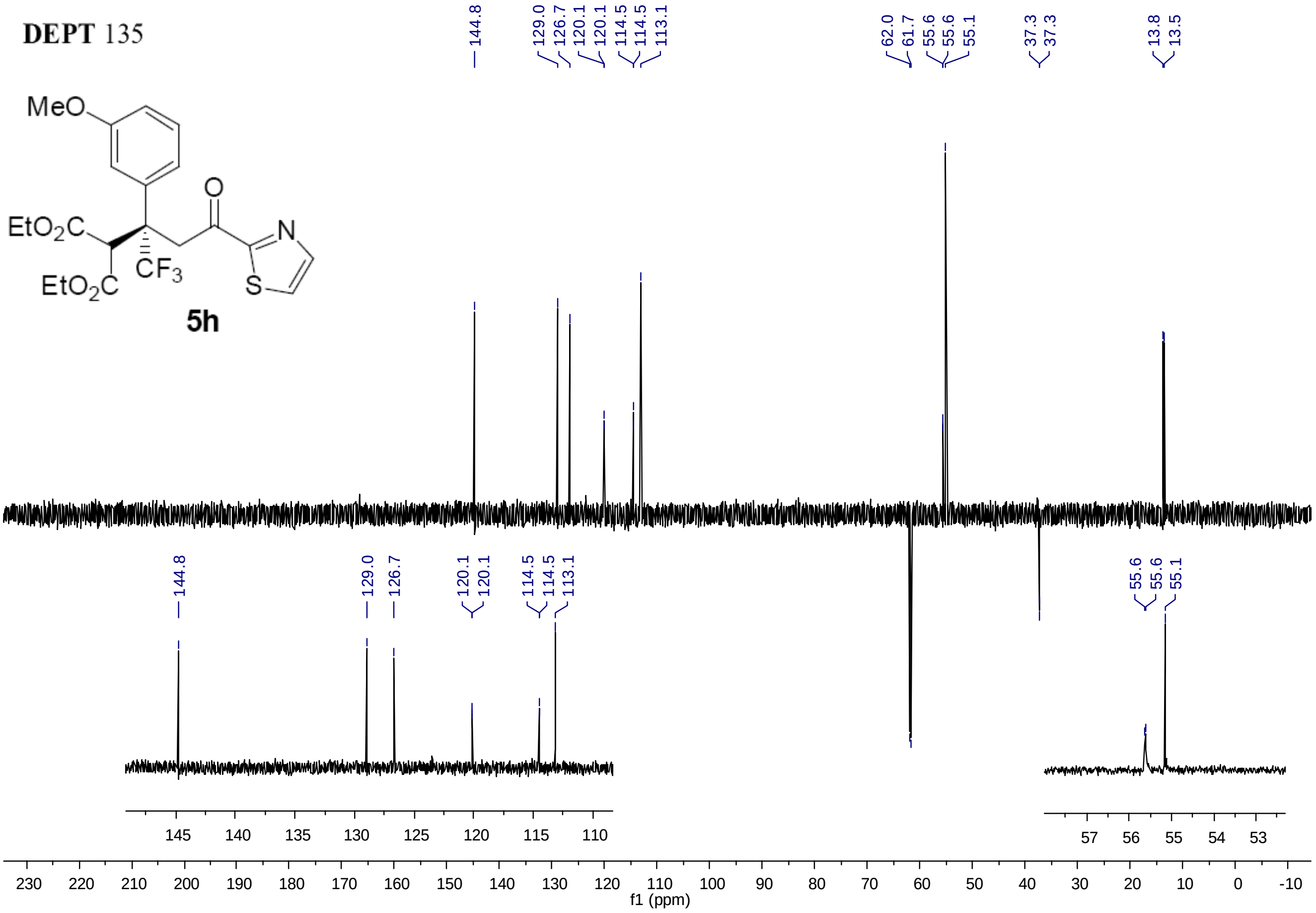
DEPT 135
MeO
EtO2C
EtO2C
CF3
O
N
S
5h
144.8
129.0
126.7
120.1
120.1
114.5
114.5
113.1
62.0
61.7
55.6
55.6
55.1
37.3
37.3
13.8
13.5
f1 (ppm)

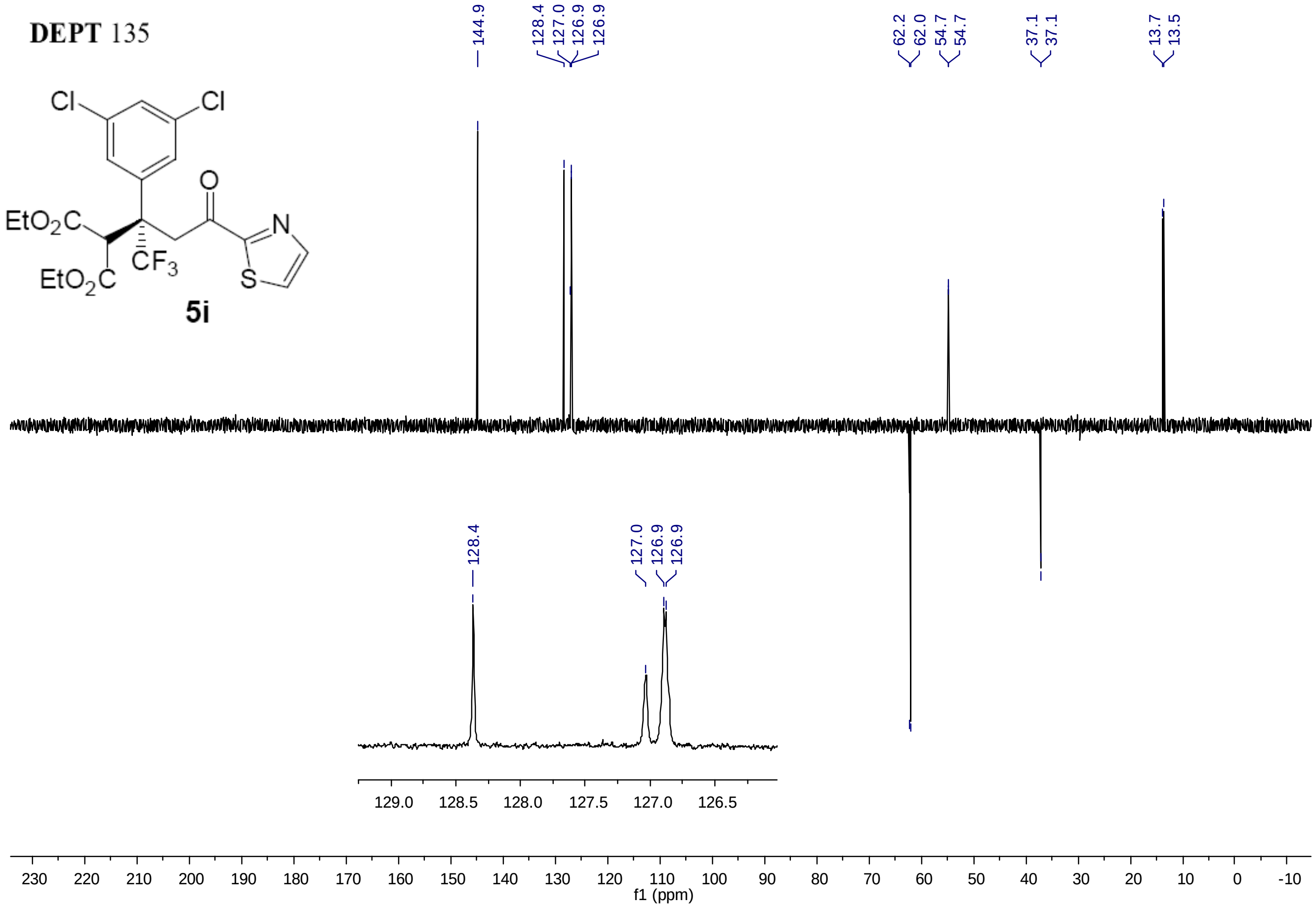
DEPT 135
Cl
Cl
EtO2C
EtO2C
CF3
O
N
S
5i
144.9
128.4
127.0
126.9
126.9
62.2
62.0
54.7
54.7
37.1
37.1
13.7
13.5
f1 (ppm)

DEPT 135

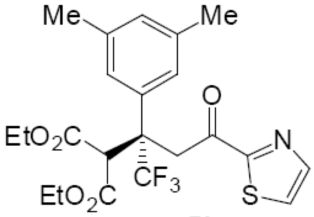

5j

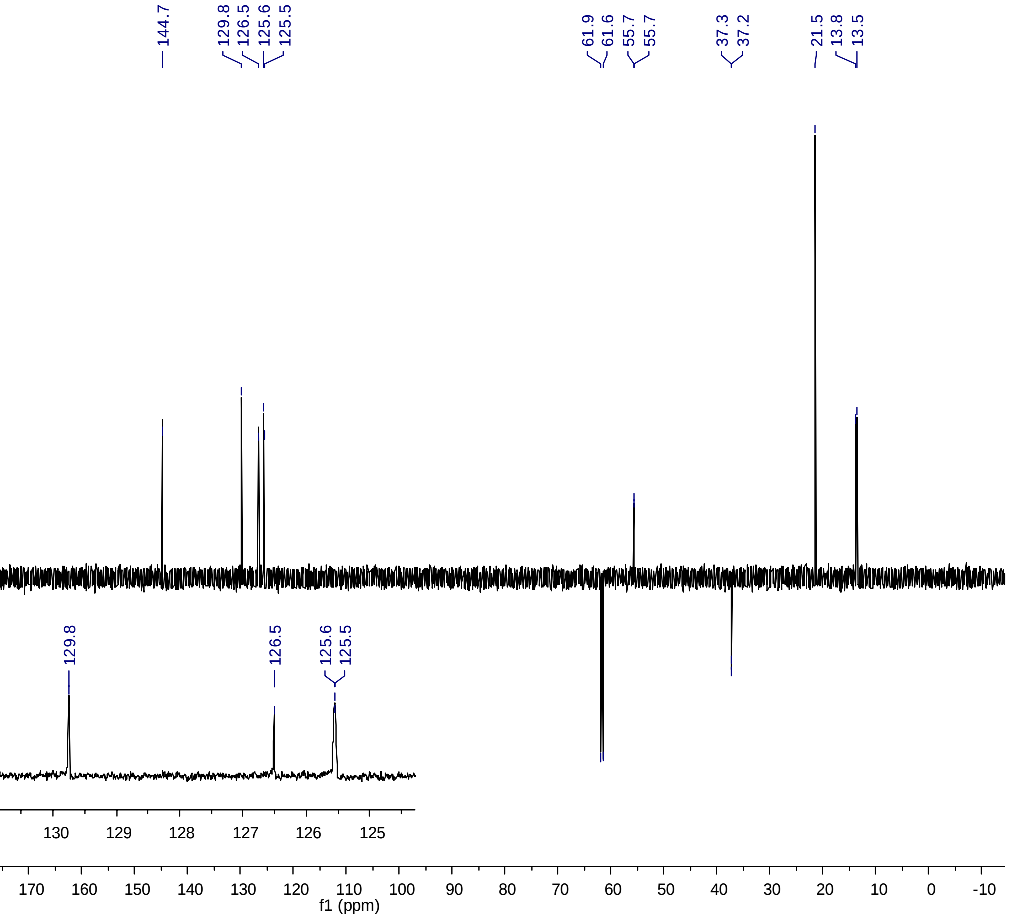

DEPT 135

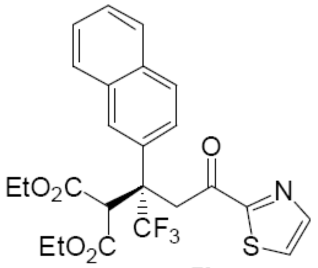

5k

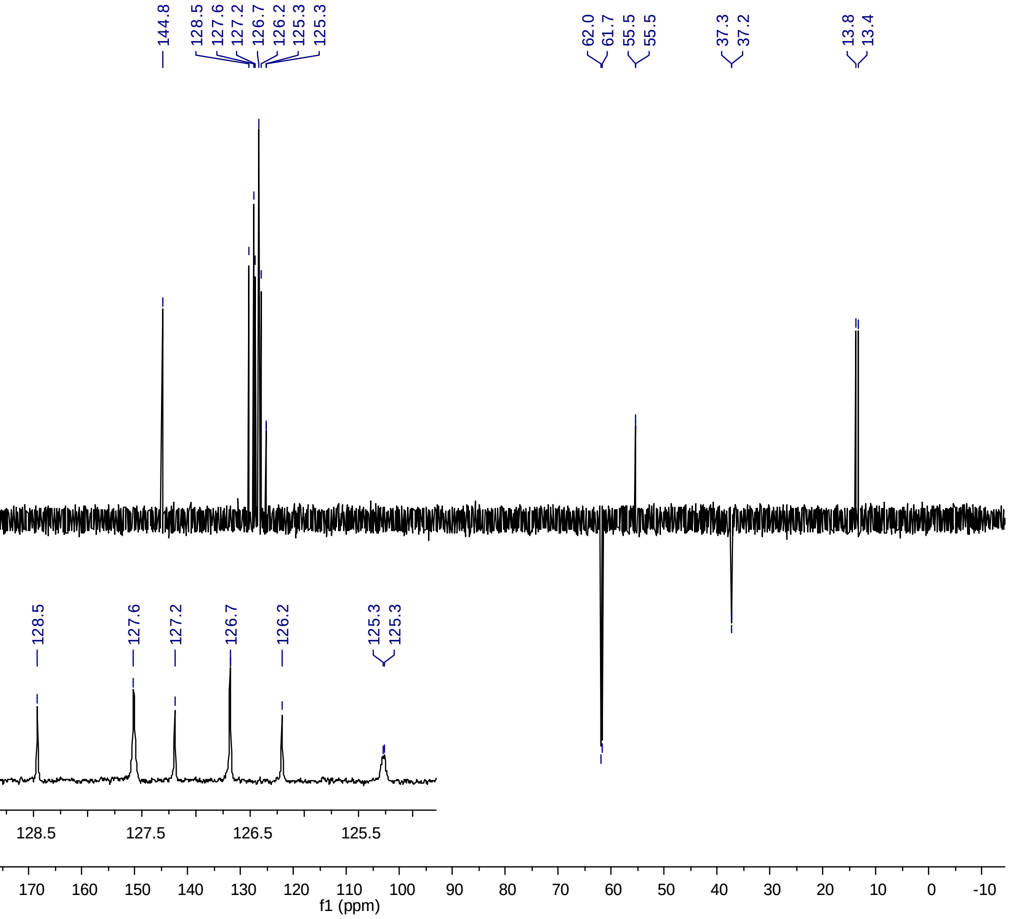

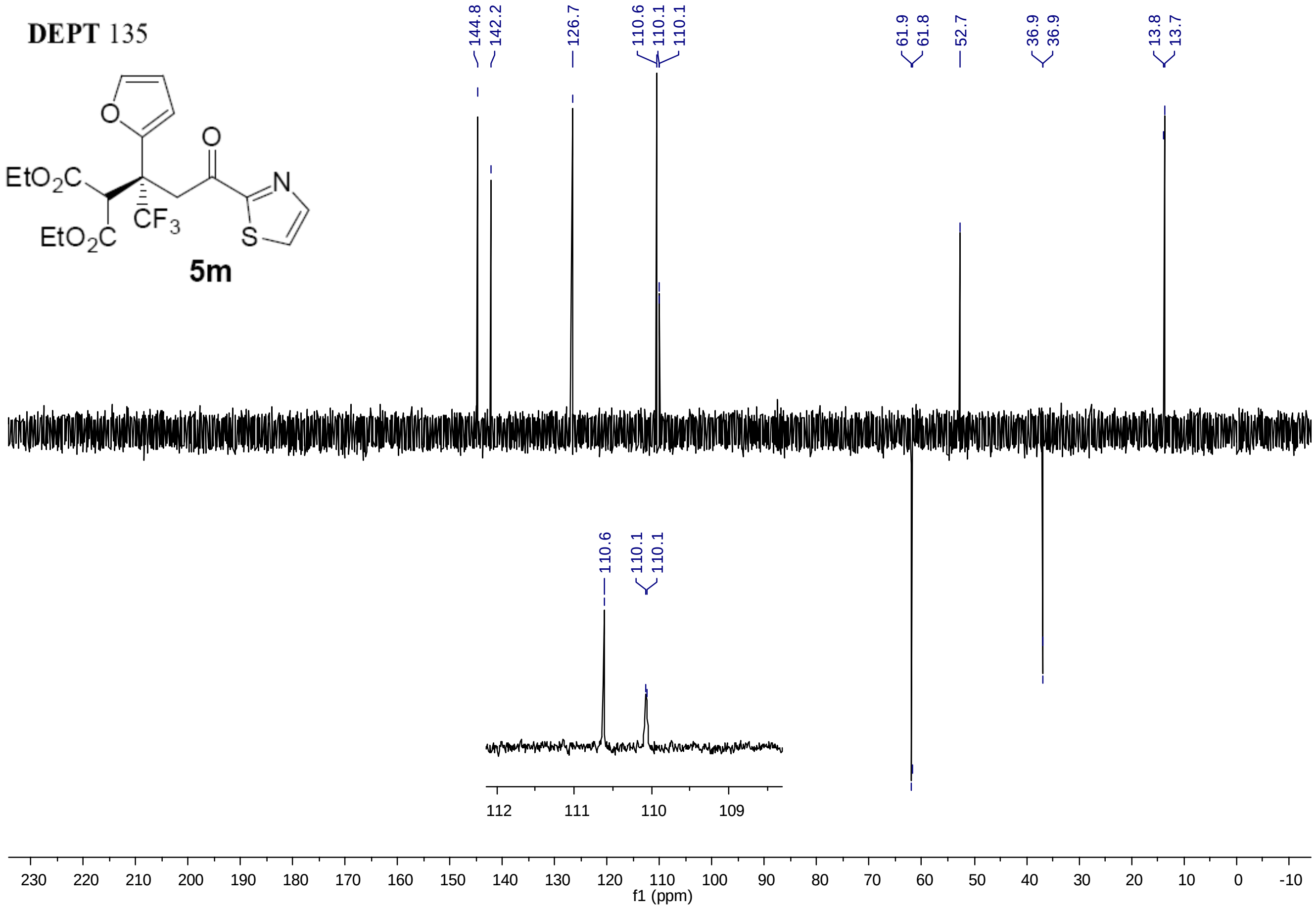
DEPT 135
EtO2C
EtO2C
CF3
O
N
S
5m
144.8
142.2
126.7
110.6
110.1
110.1
61.9
61.8
52.7
36.9
36.9
13.8
13.7
112
111
110
109
230 220 210 200 190 180 170 160 150 140 130 120 110 100 90 80 70 60 50 40 30 20 10 0 -10
f1 (ppm)

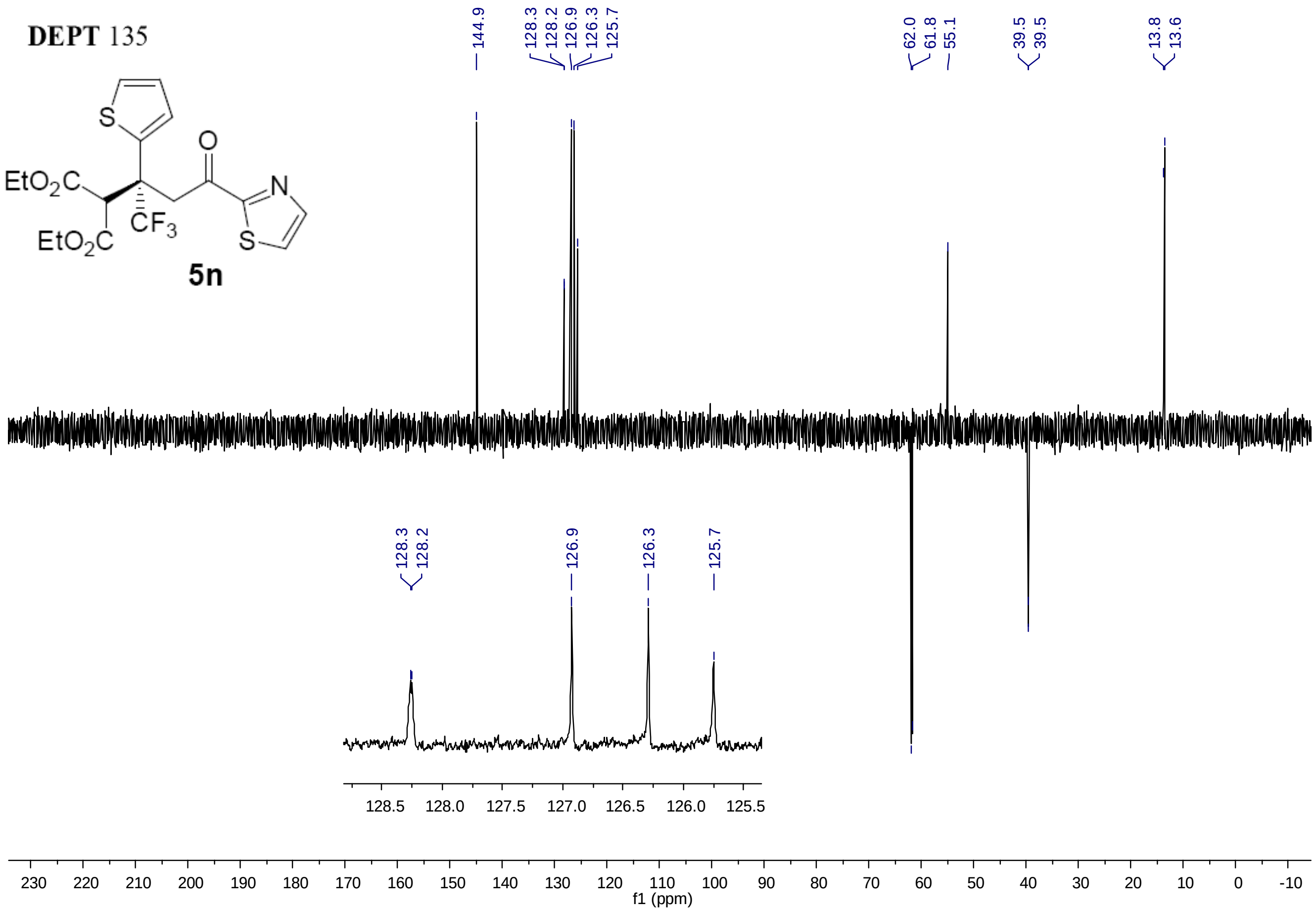
DEPT 135
EtO2C
EtO2C
CF3
O
N
S
5n
144.9
128.3
128.2
126.9
126.3
125.7
62.0
61.8
55.1
39.5
39.5
13.8
13.6
128.5 128.0 127.5 127.0 126.5 126.0 125.5
230 220 210 200 190 180 170 160 150 140 130 120 110 100 90 80 70 60 50 40 30 20 10 0 -10
f1 (ppm)

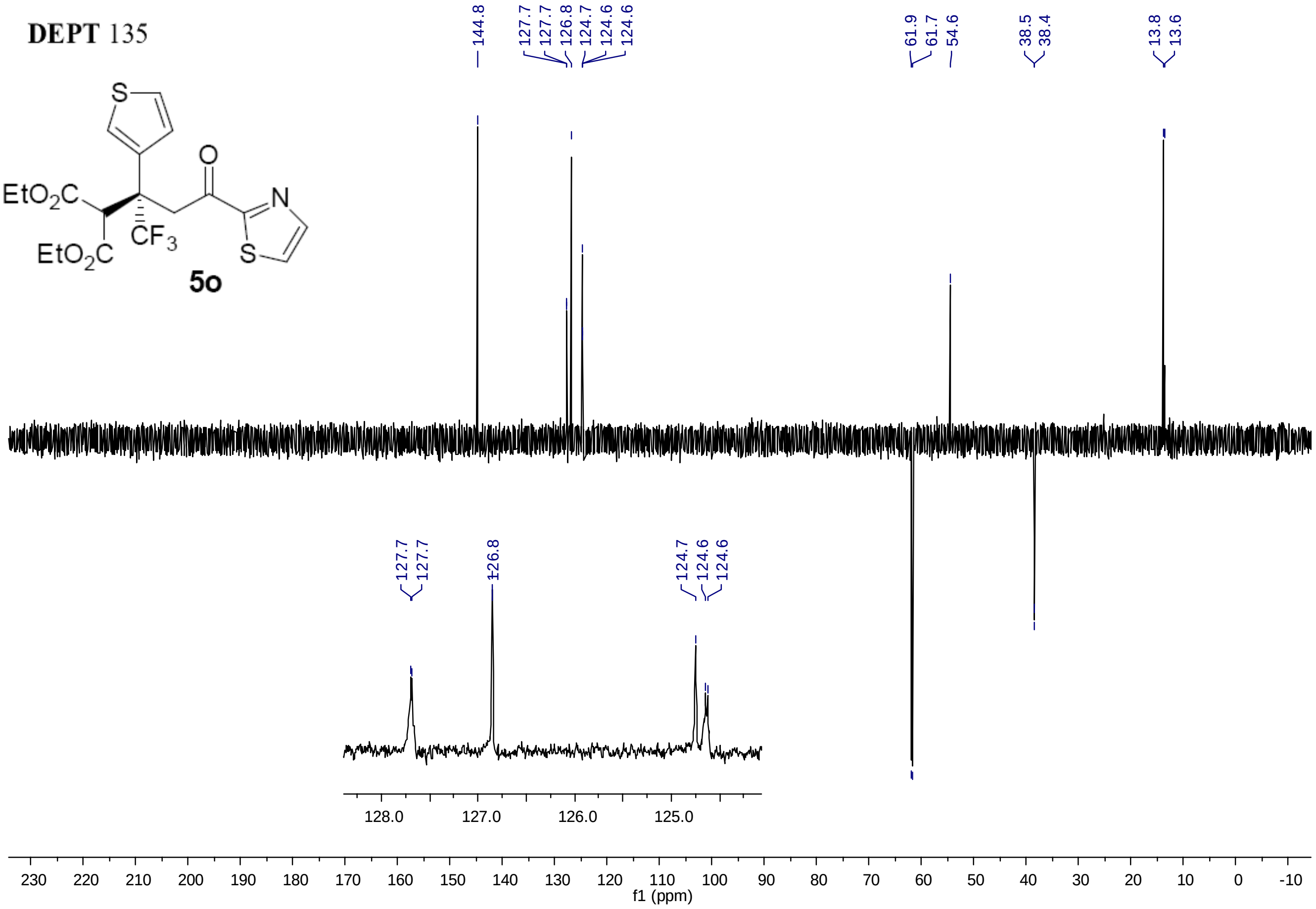
DEPT 135
EtO2C
EtO2C
CF3
O
N
S
S
5o
144.8
127.7
127.7
126.8
124.7
124.6
124.6
61.9
61.7
54.6
38.5
38.4
13.8
13.6
128.0
127.0
126.0
125.0
230 220 210 200 190 180 170 160 150 140 130 120 110 100 90 80 70 60 50 40 30 20 10 0 -10
f1 (ppm)

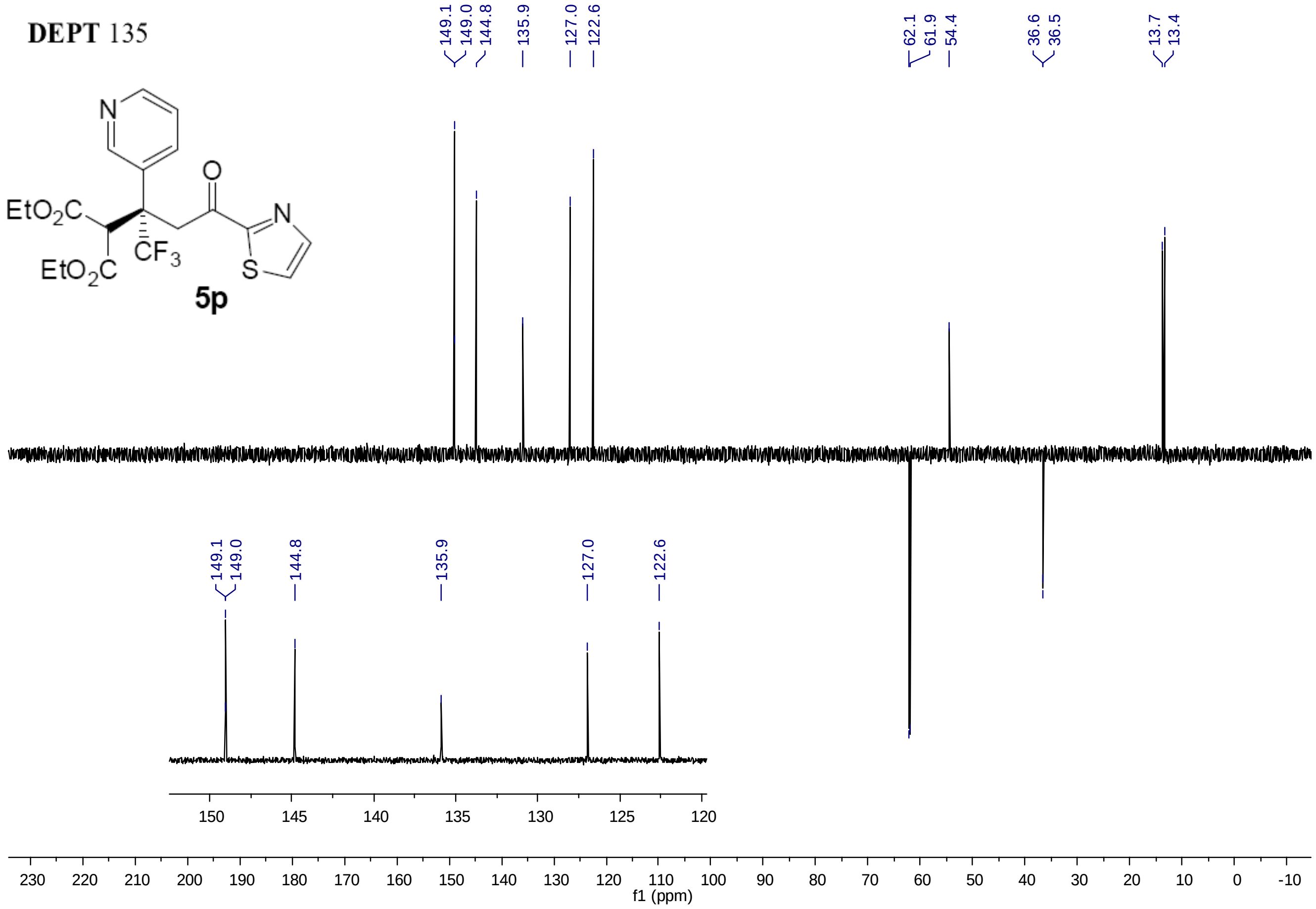
DEPT 135
N
EtO2C
EtO2C
CF3
O
N
S
5p
149.1
149.0
144.8
135.9
127.0
122.6
62.1
61.9
54.4
36.6
36.5
13.7
13.4
150 145 140 135 130 125 120
230 220 210 200 190 180 170 160 150 140 130 120 110 100 90 80 70 60 50 40 30 20 10 0 -10
f1 (ppm)

DEPT 135

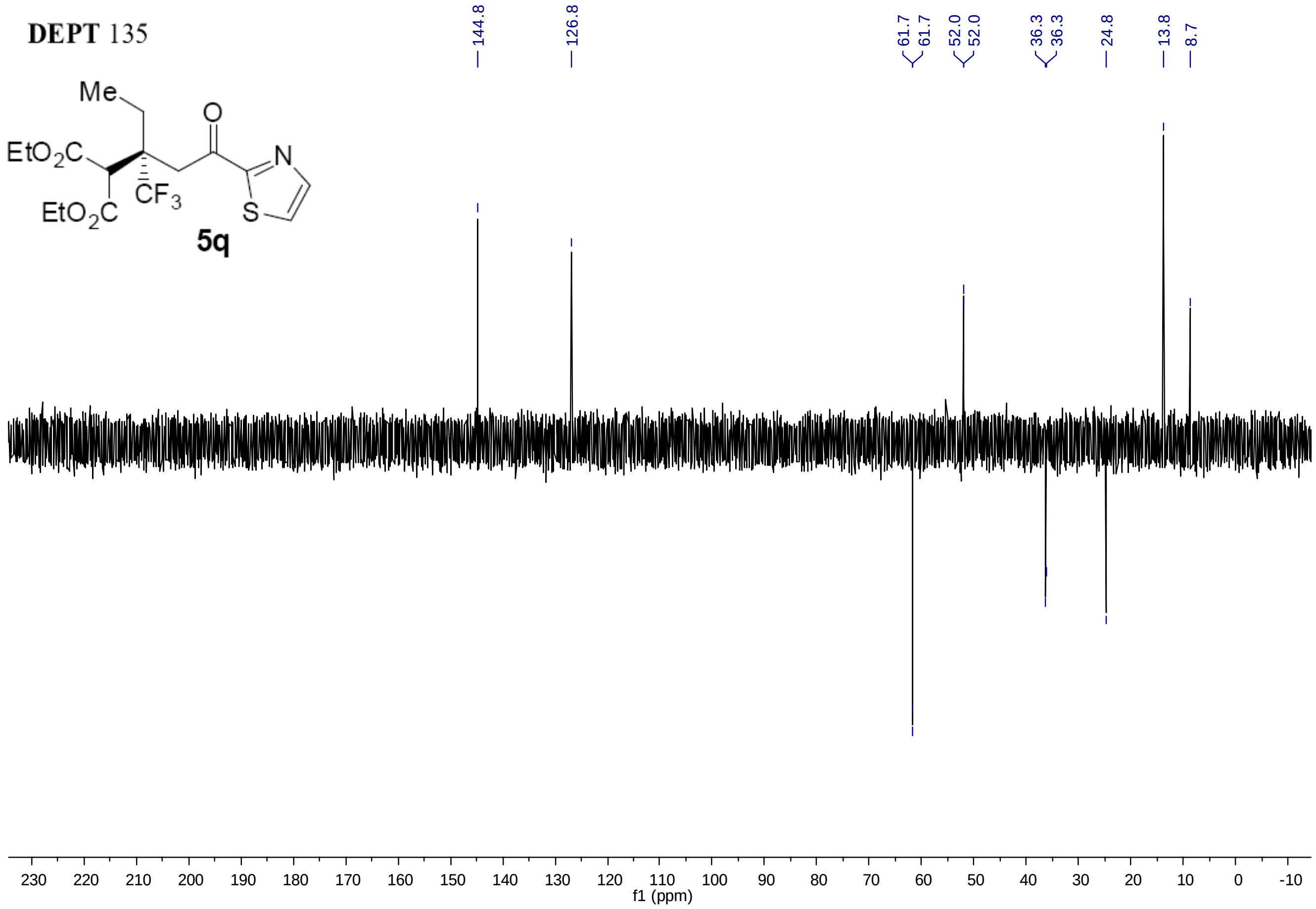

DEPT 135

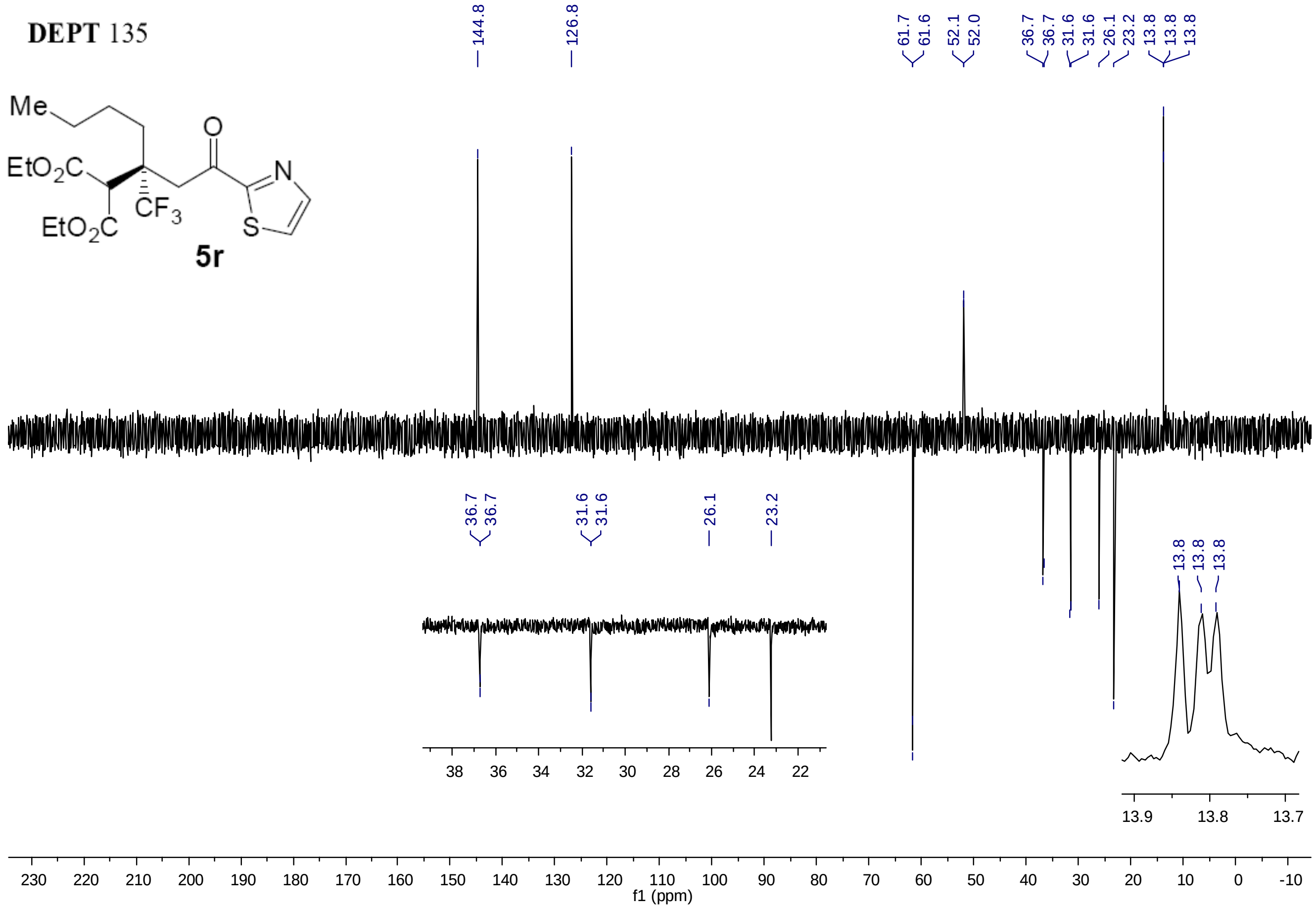

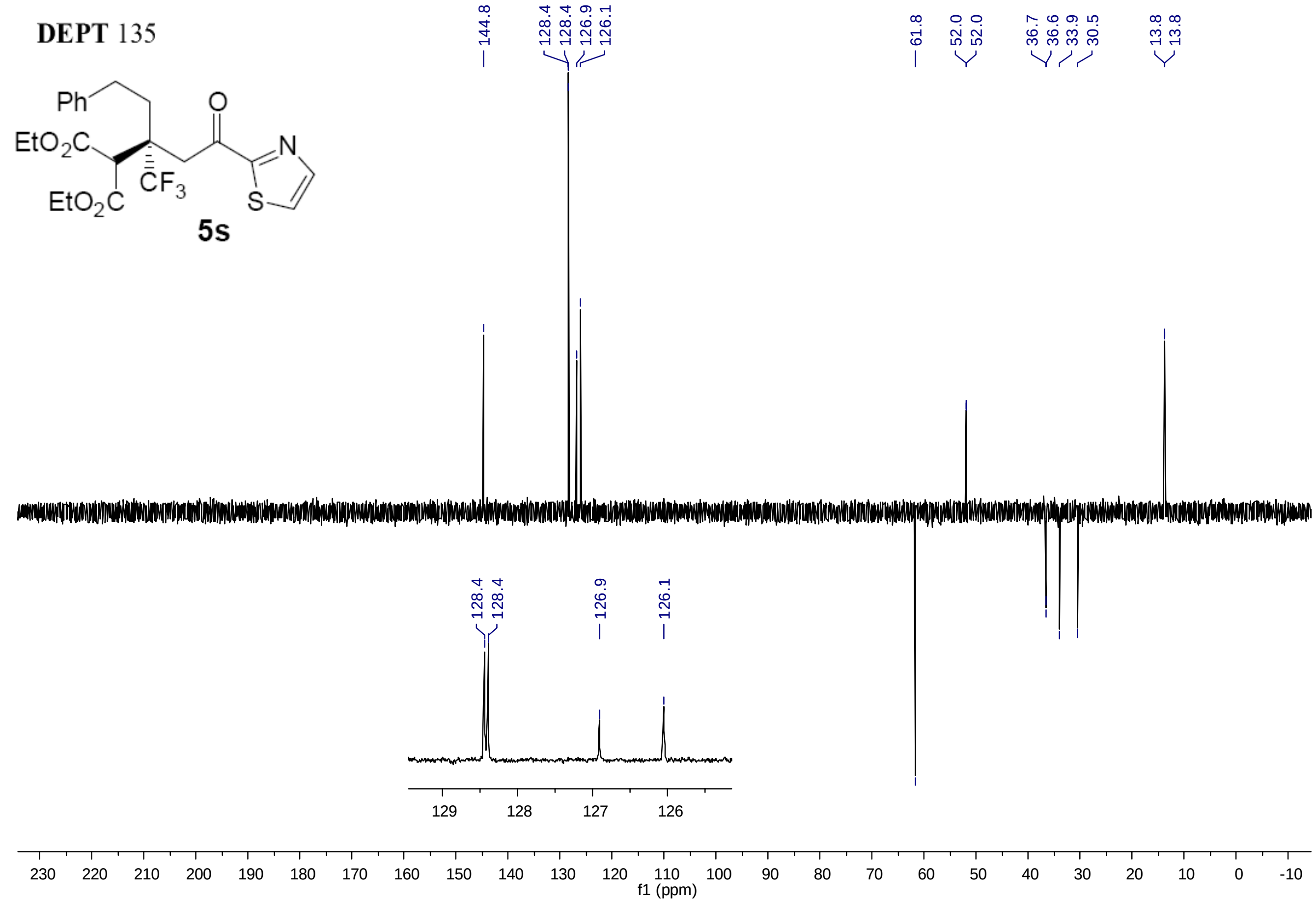
DEPT 135
Ph
EtO2C
EtO2C
CF3
O
N
S
5s
144.8
128.4
128.4
126.9
126.1
61.8
52.0
52.0
36.7
36.6
33.9
30.5
13.8
13.8
128.4
128.4
126.9
126.1
129
128
127
126
230 220 210 200 190 180 170 160 150 140 130 120 110 100 90 80 70 60 50 40 30 20 10 0 -10
f1 (ppm)

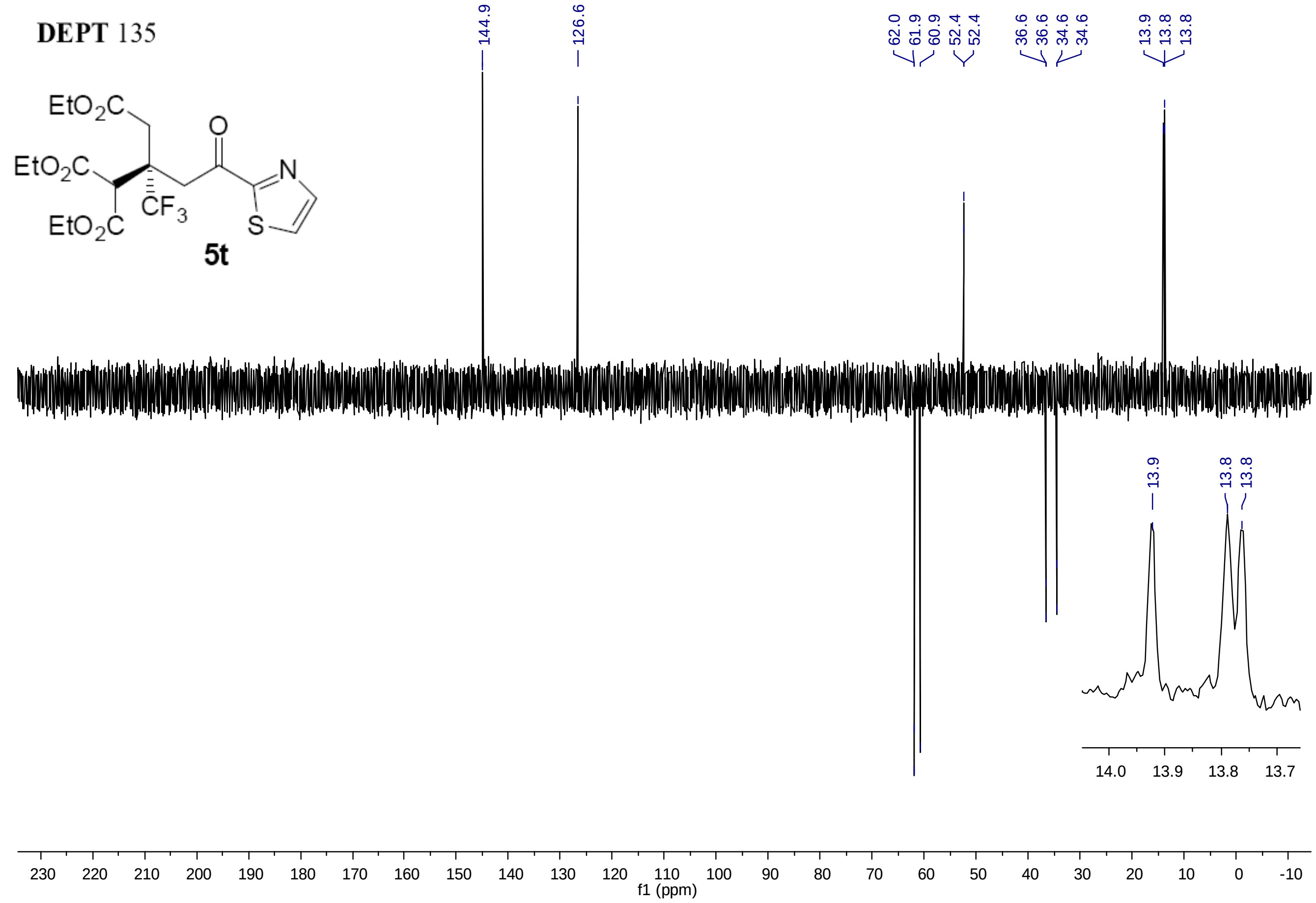
DEPT 135
EtO2C
EtO2C
EtO2C
CF3
O
N
S
5t
144.9
126.6
62.0
61.9
60.9
52.4
52.4
36.6
36.6
34.6
34.6
13.9
13.8
13.8
13.9
13.8
13.8
14.0
13.9
13.8
13.7
230 220 210 200 190 180 170 160 150 140 130 120 110 100 90 80 70 60 50 40 30 20 10 0 -10
f1 (ppm)

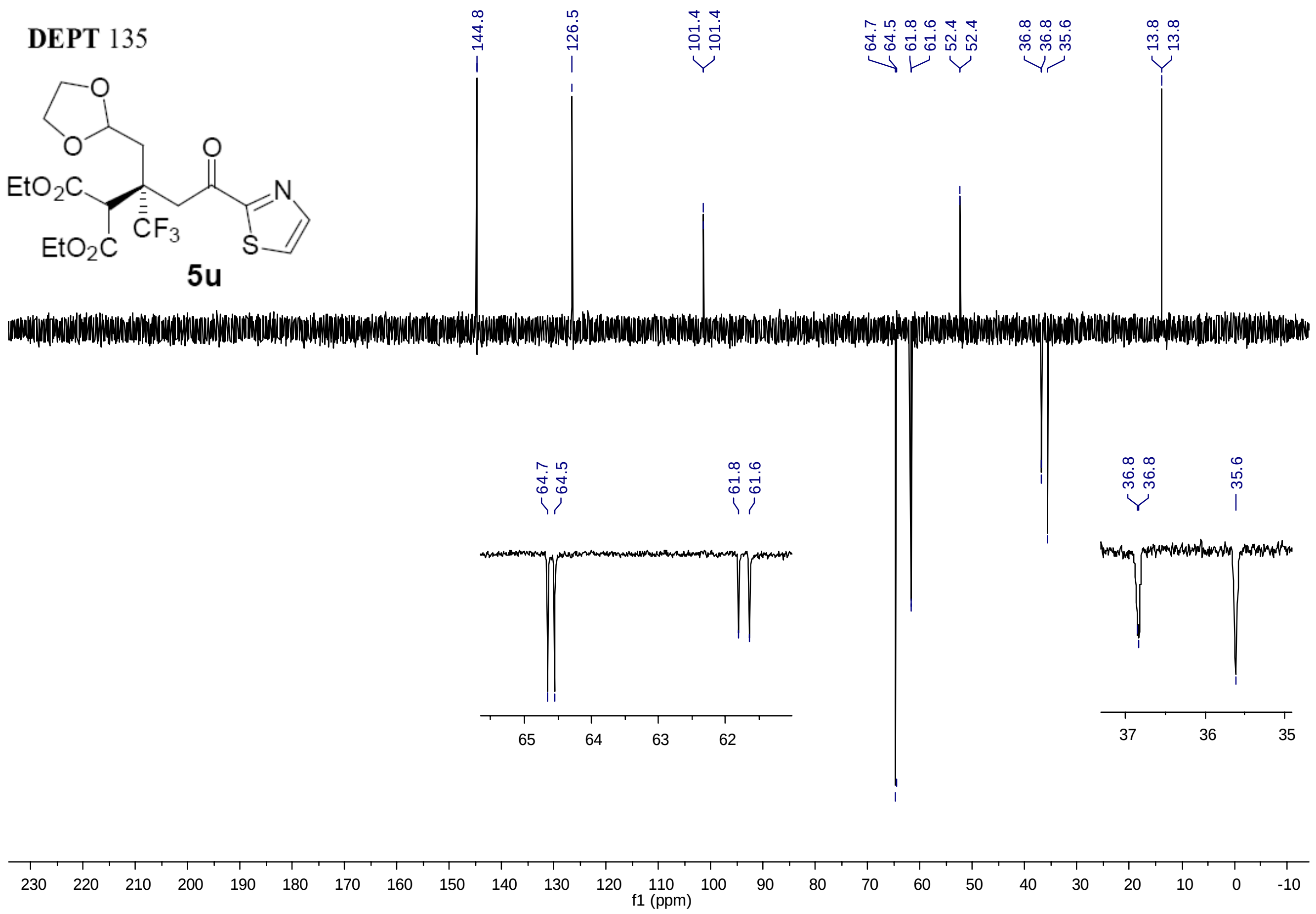
DEPT 135
5u
144.8
126.5
101.4
101.4
64.7
64.5
61.8
61.6
52.4
52.4
36.8
36.8
35.6
13.8
13.8
f1 (ppm)

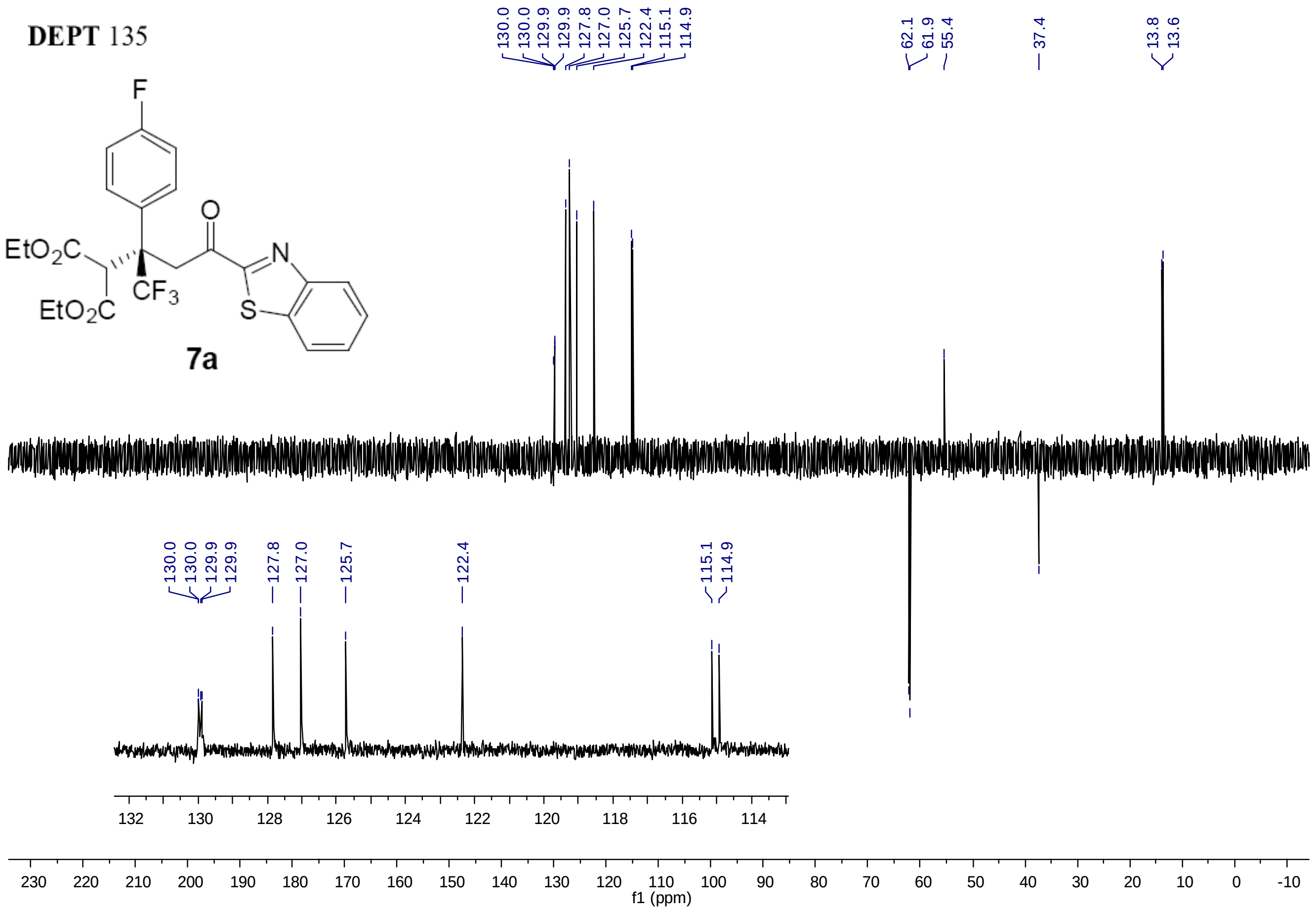
DEPT 135
7a
130.0
130.0
129.9
129.9
127.8
127.0
125.7
122.4
115.1
114.9
62.1
61.9
55.4
37.4
13.8
13.6
f1 (ppm)

DEPT 135

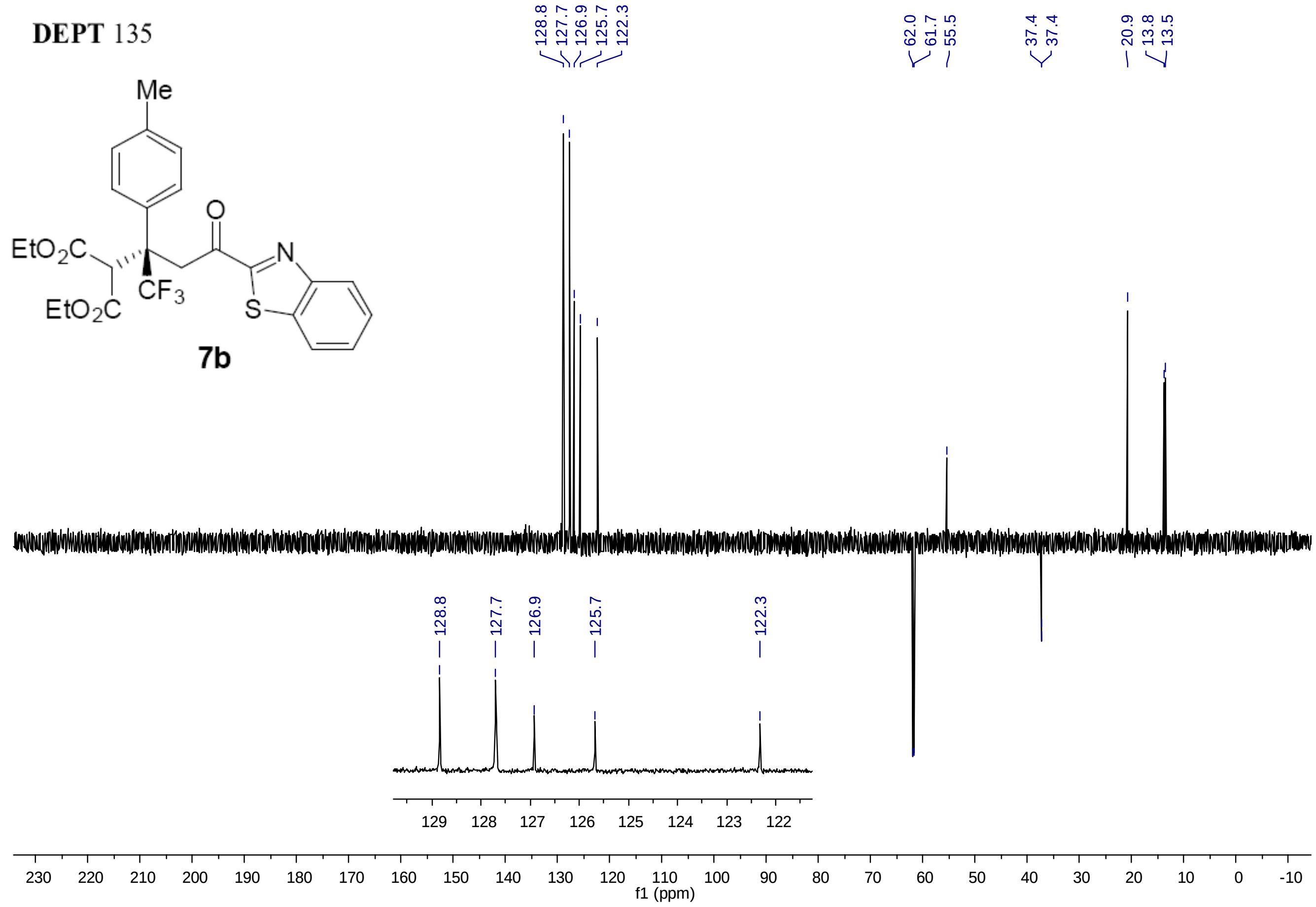

DEPT 135

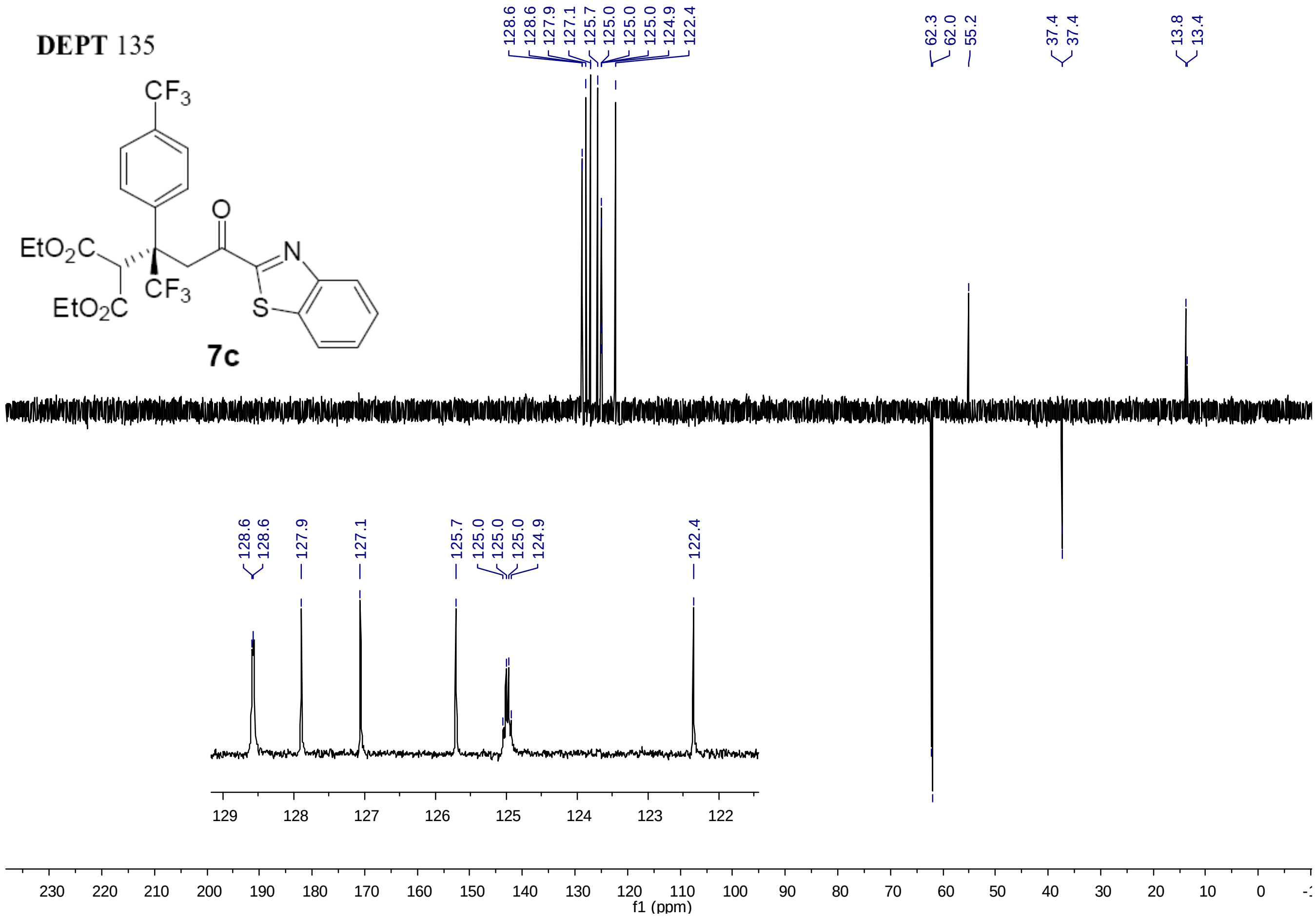

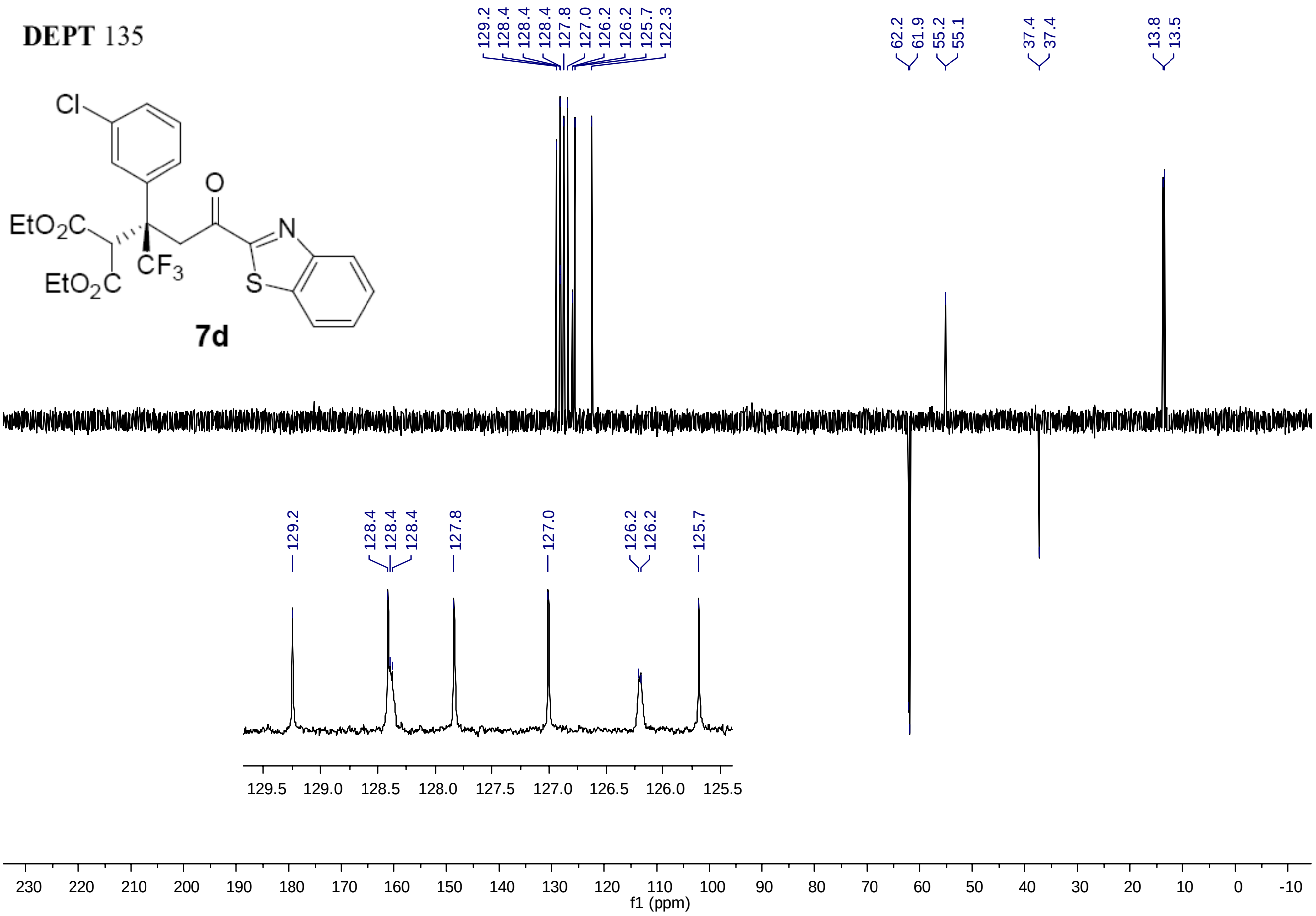
DEPT 135
7d
129.2 128.4 128.4 128.4 127.8 127.0 126.2 126.2 125.7 122.3
62.2 61.9 55.2 55.1
37.4 37.4
13.8 13.5
129.2 128.4 128.4 128.4 127.8 127.0 126.2 126.2 125.7
f1 (ppm)

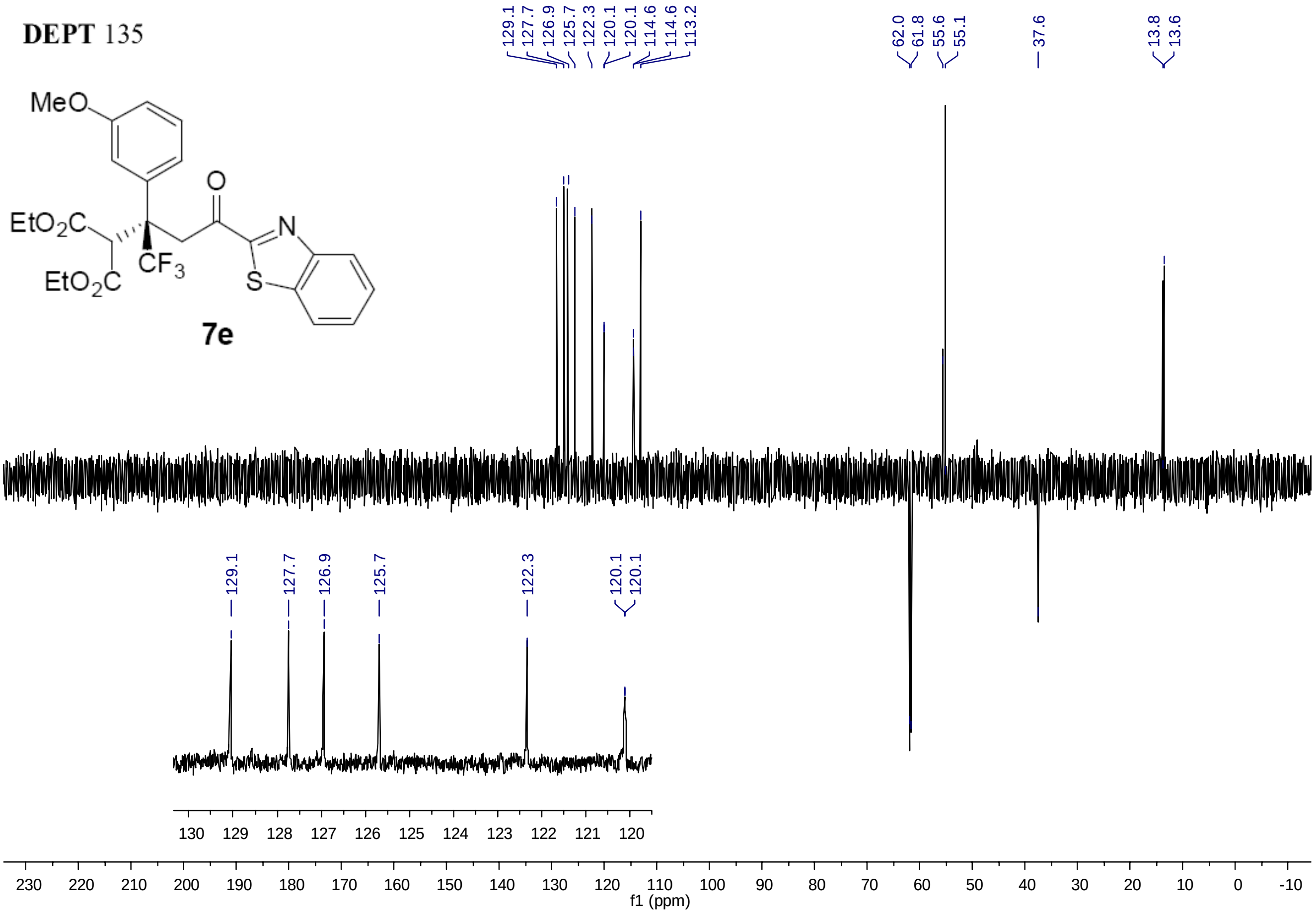
DEPT 135
7e
129.1 127.7 126.9 125.7 122.3 120.1 120.1 114.6 114.6 113.2
62.0 61.8 55.6 55.1
37.6
13.8 13.6
129.1 127.7 126.9 125.7 122.3 120.1 120.1
f1 (ppm)

DEPT 135

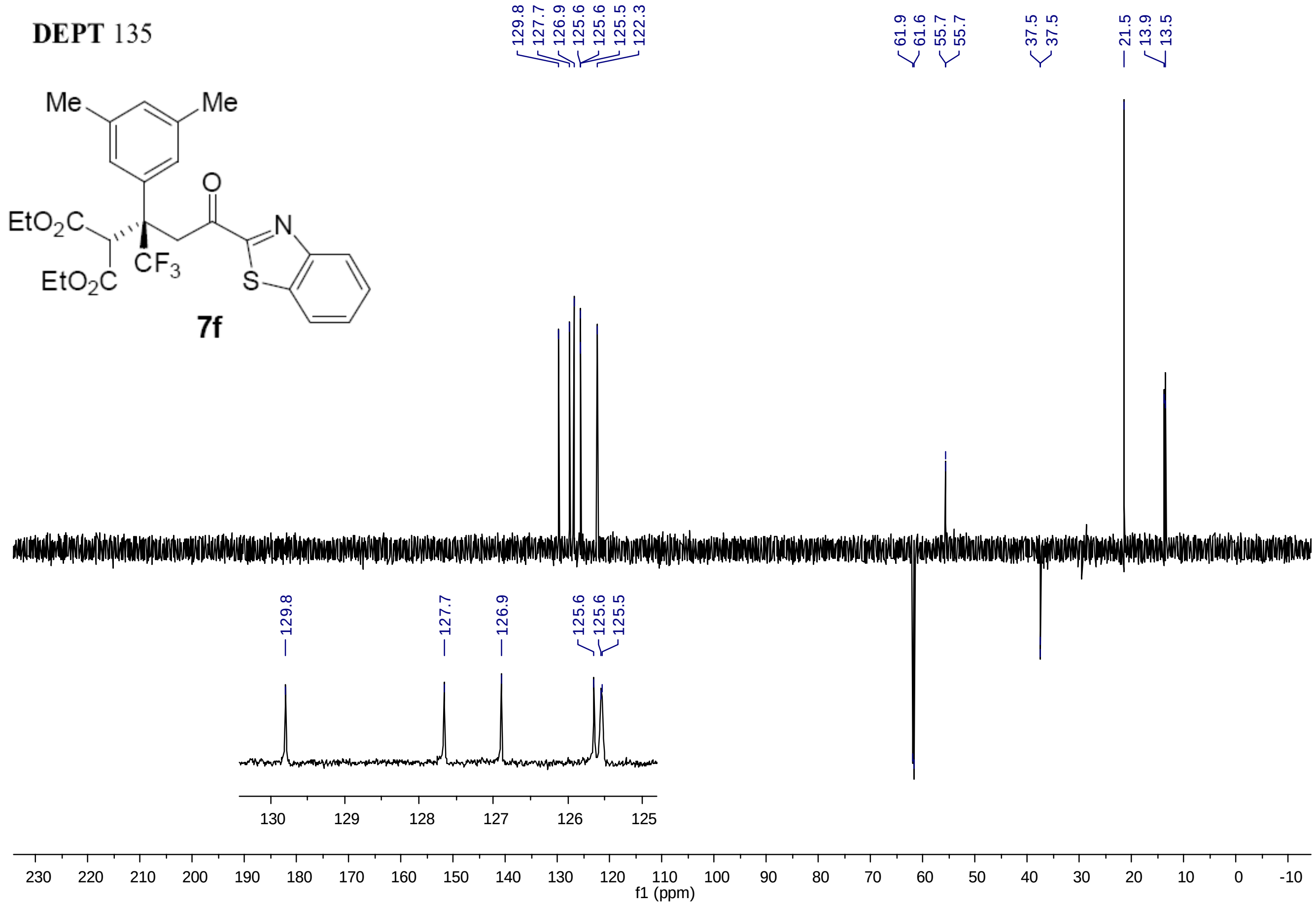

7f

DEPT 135

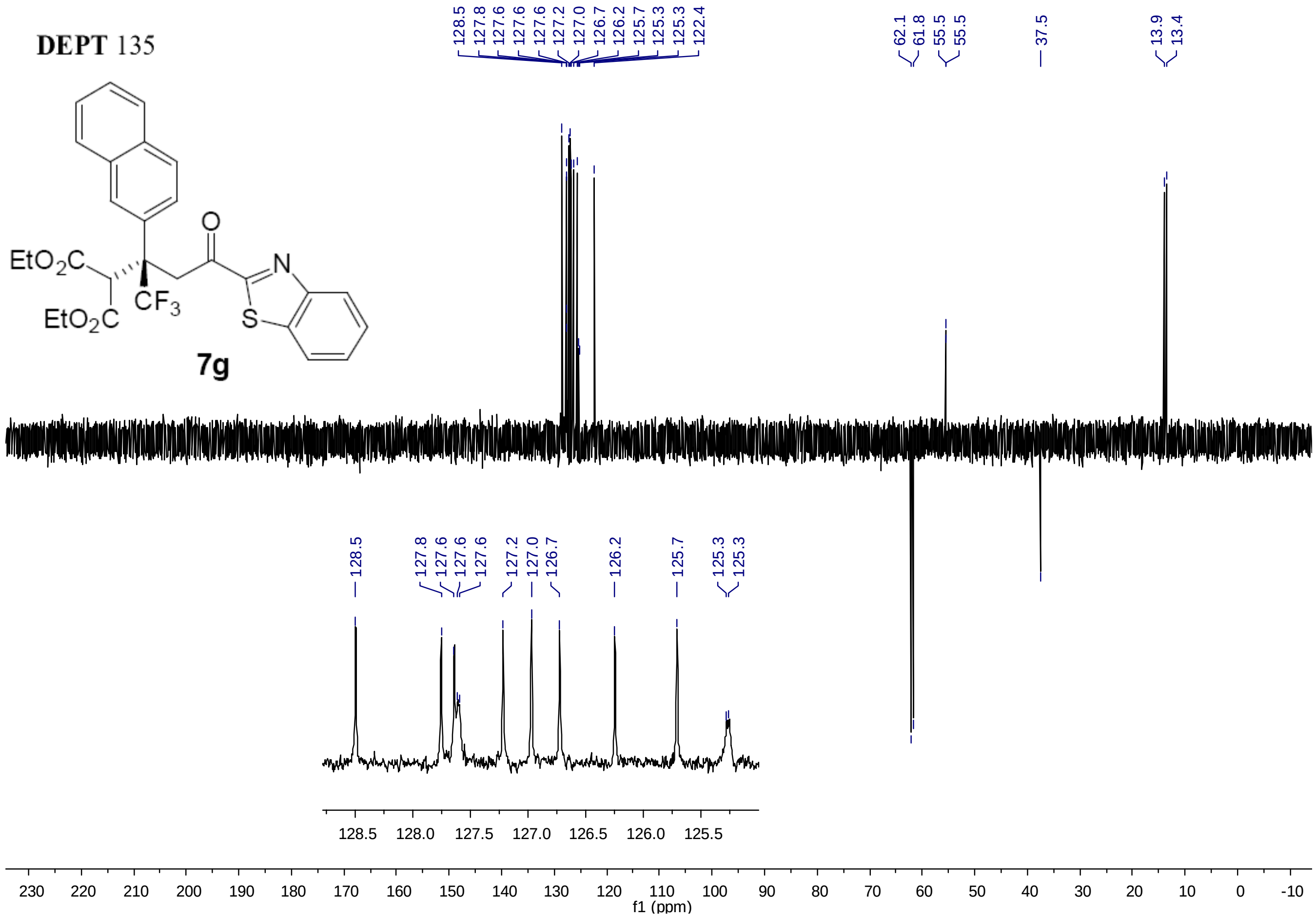

7g

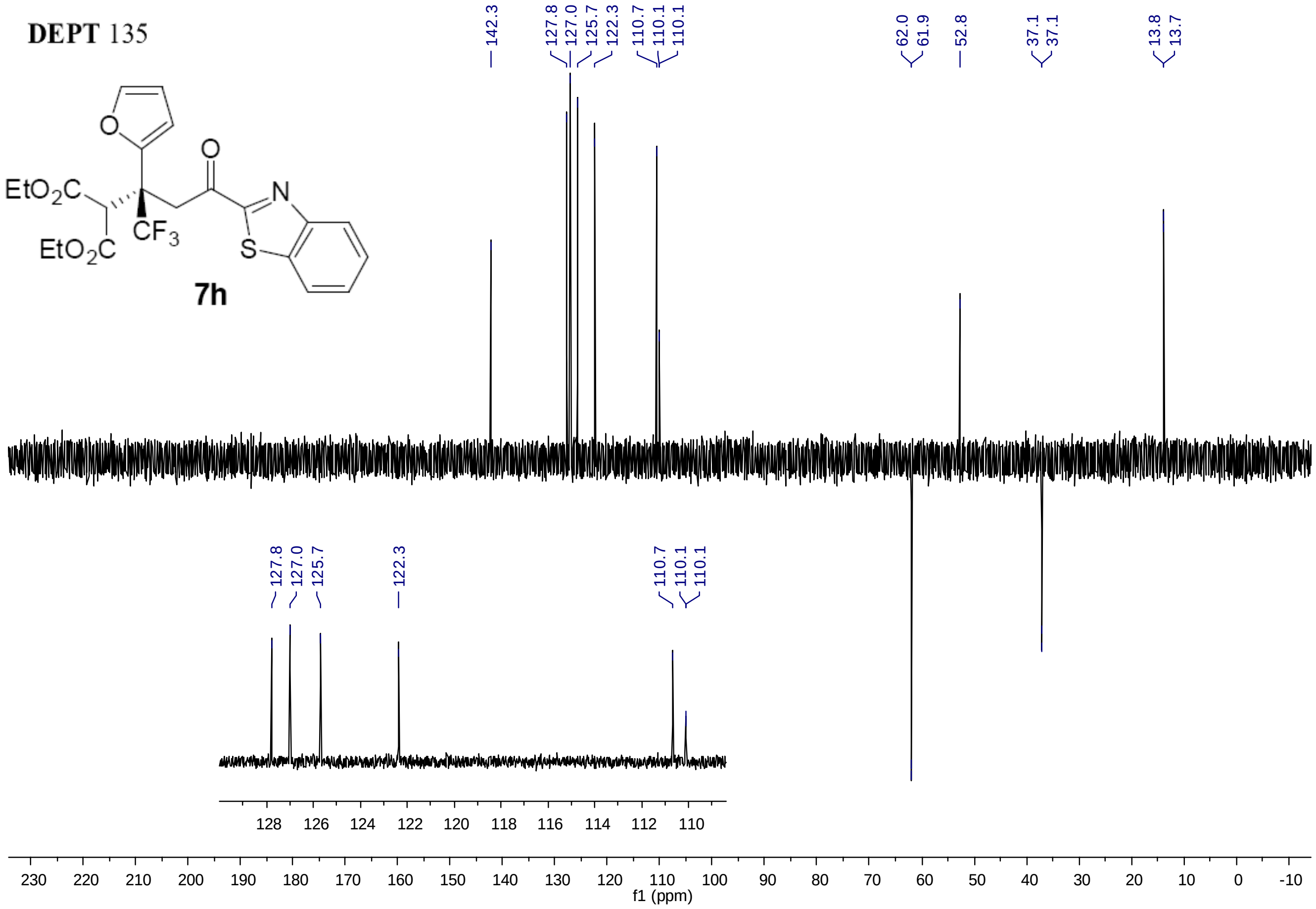
DEPT 135
EtO2C
EtO2C
CF3
O
N
S
7h
142.3
127.8
127.0
125.7
122.3
110.7
110.1
110.1
62.0
61.9
52.8
37.1
37.1
13.8
13.7
f1 (ppm)

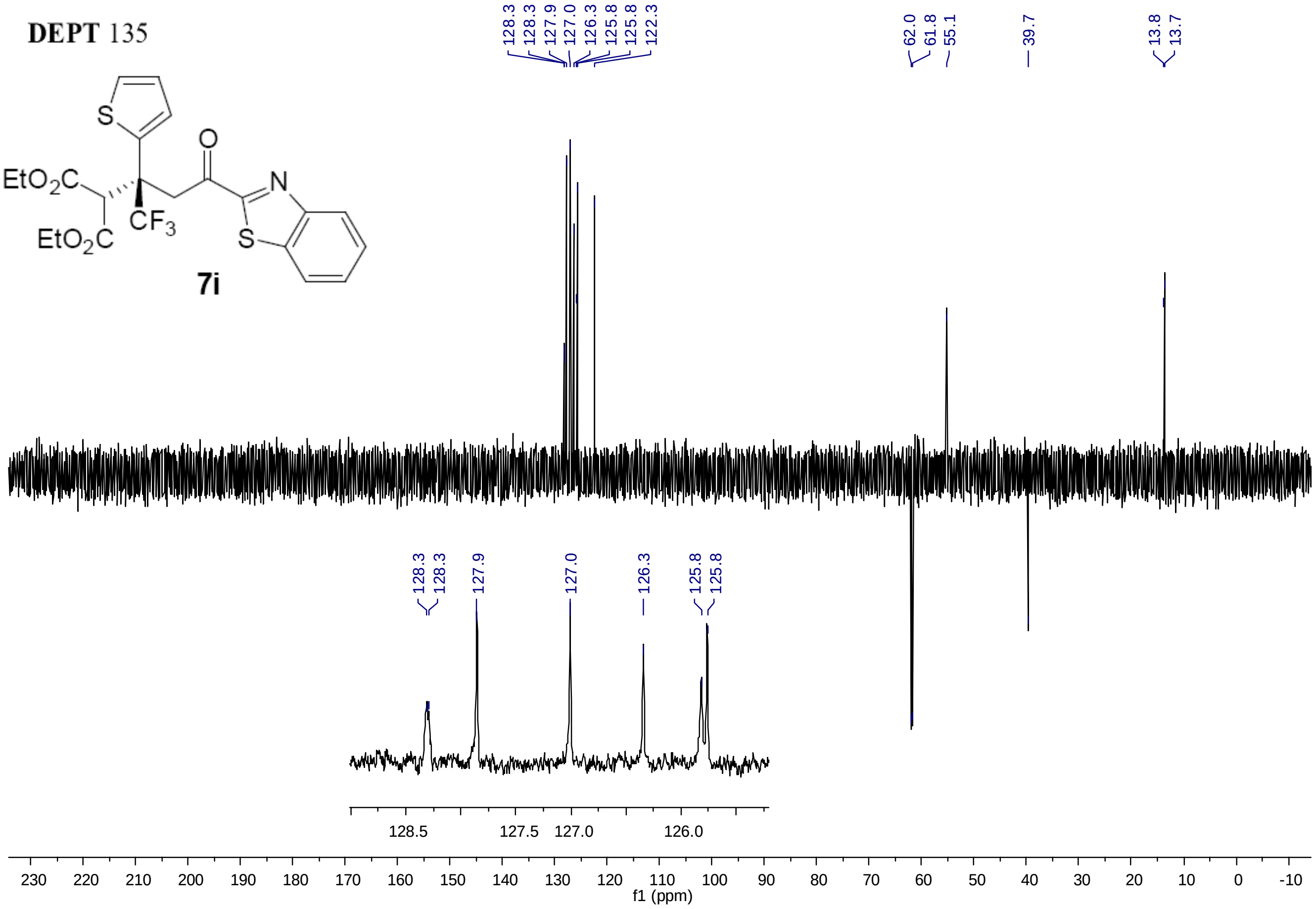
DEPT 135
EtO2C
EtO2C
CF3
O
N
S
S
7i
128.3
128.3
127.9
127.0
126.3
125.8
125.8
122.3
62.0
61.8
55.1
39.7
13.8
13.7
f1 (ppm)

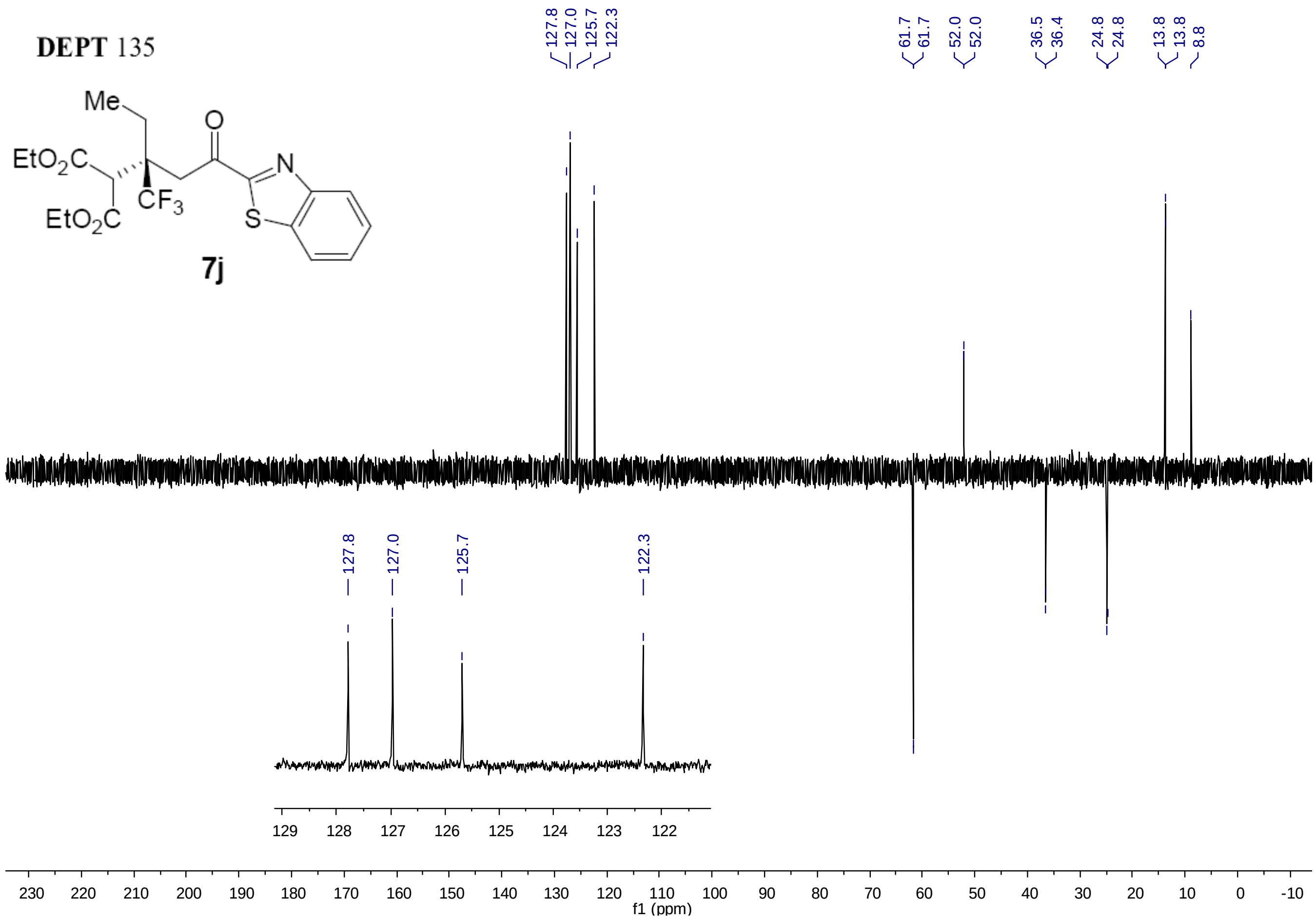
DEPT 135
Me
EtO2C
EtO2C
CF3
O
N
S
7j
127.8
127.0
125.7
122.3
61.7
61.7
52.0
52.0
36.5
36.4
24.8
24.8
13.8
13.8
8.8
f1 (ppm)

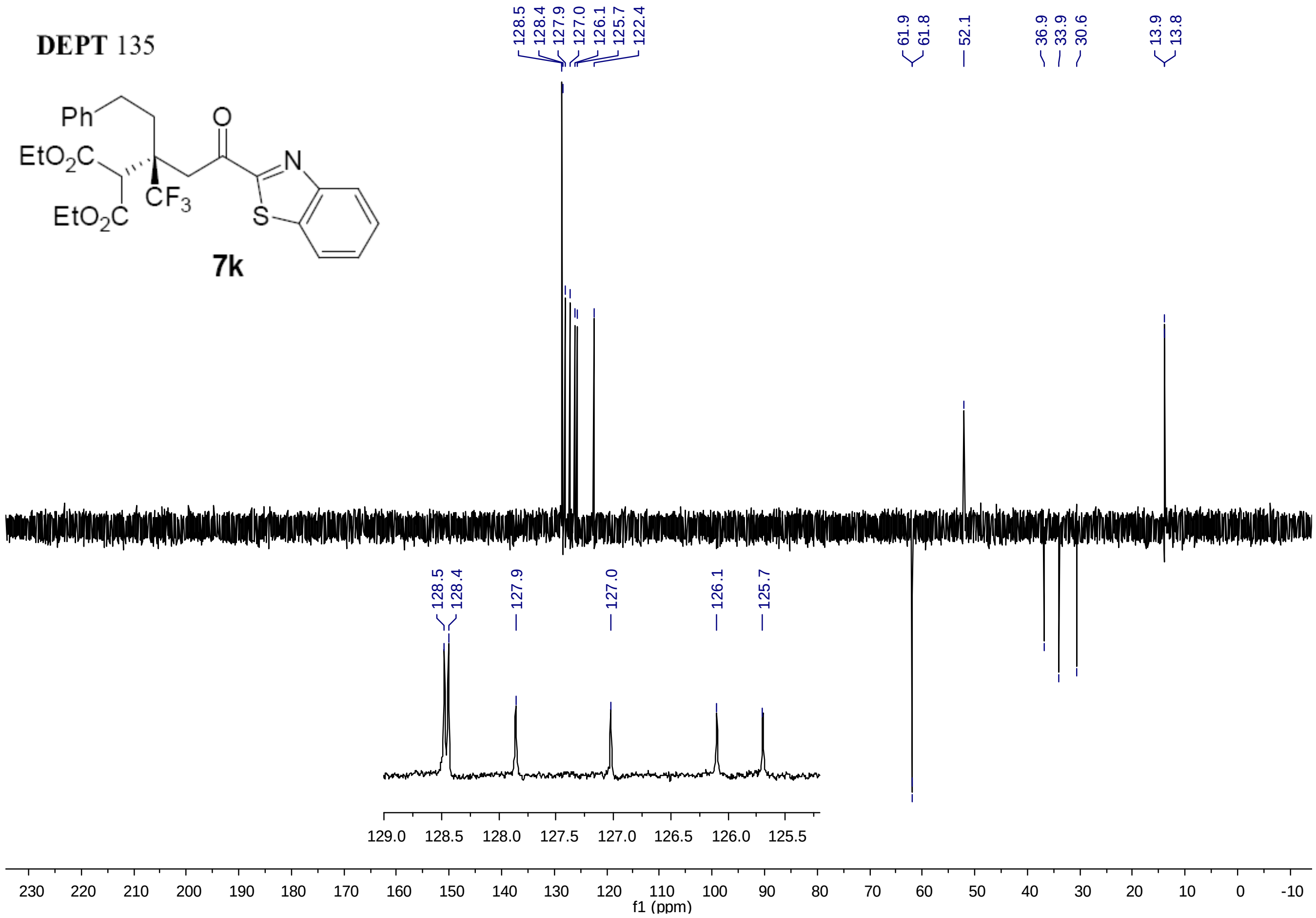
DEPT 135
Ph
EtO2C
EtO2C
CF3
O
N
S
7k
128.5
128.4
127.9
127.0
126.1
125.7
122.4
61.9
61.8
52.1
36.9
33.9
30.6
13.9
13.8
f1 (ppm)

DEPT 135

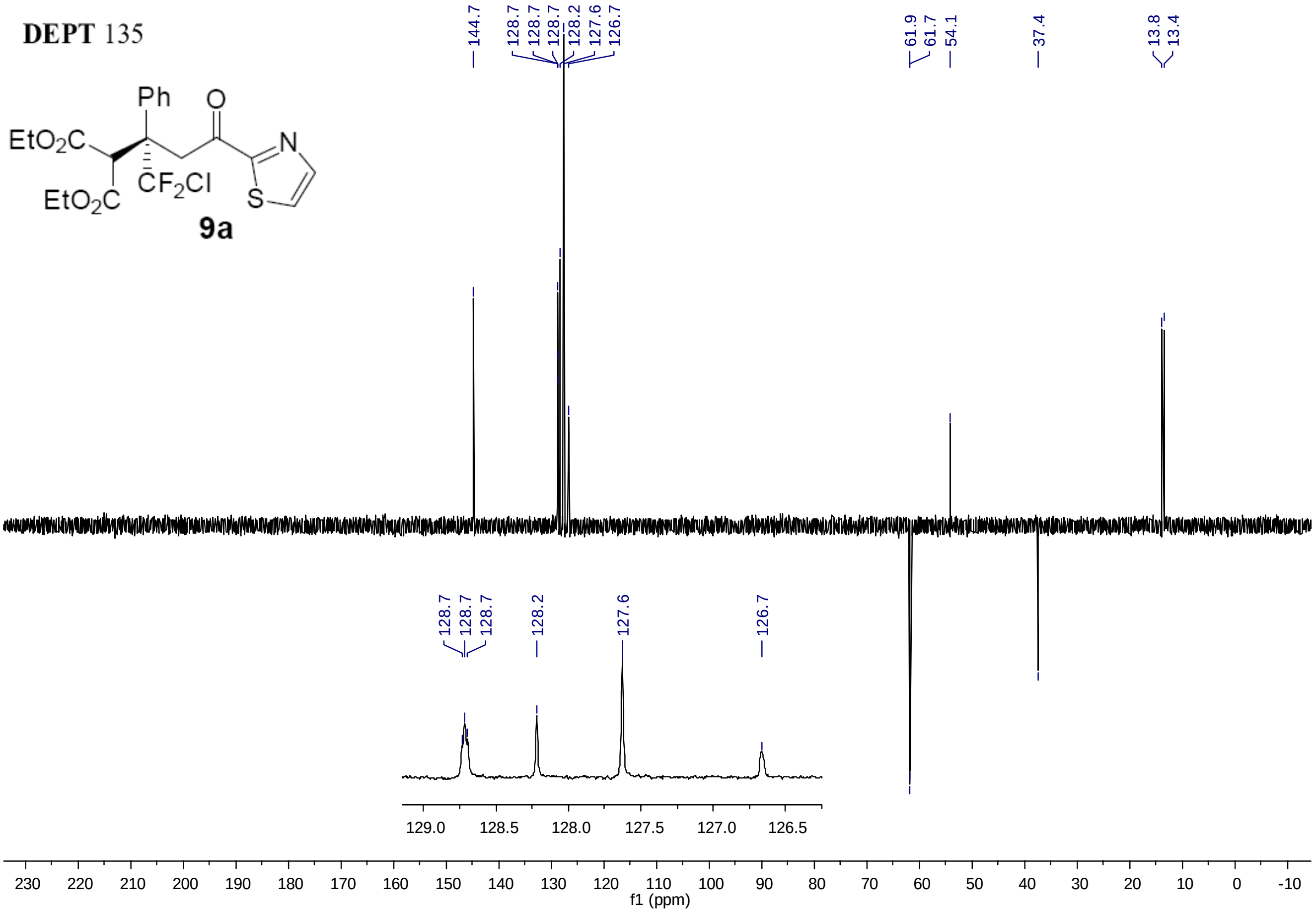

9a

DEPT 135

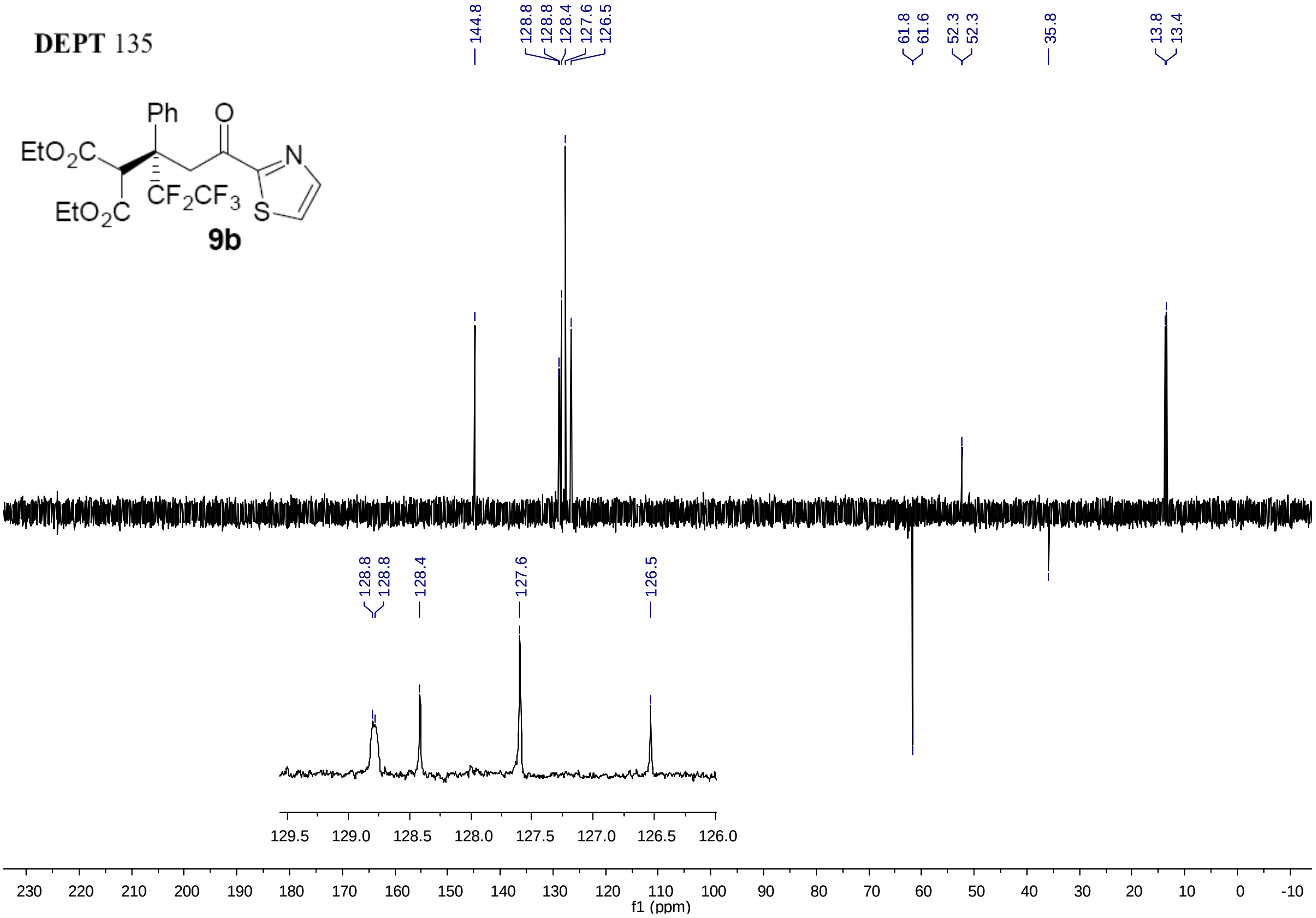

9b

Copies of ^{19}F NMR spectra - compounds 10a-10g, obtained from adduct 3h

^{19}F NMR
(376 MHz, $CDCl_3$)

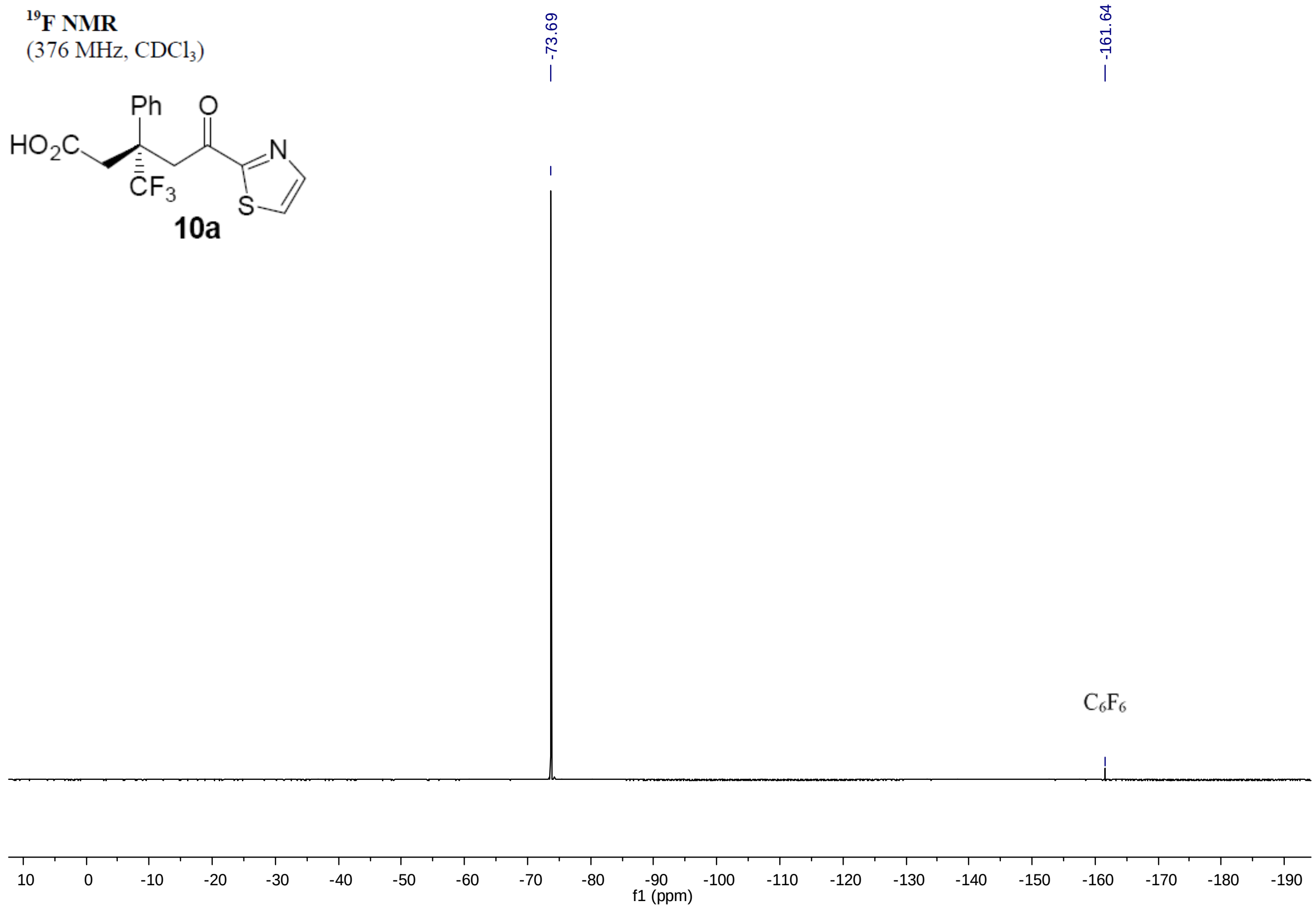

^{19}F NMR
(376 MHz, $CDCl_3$)

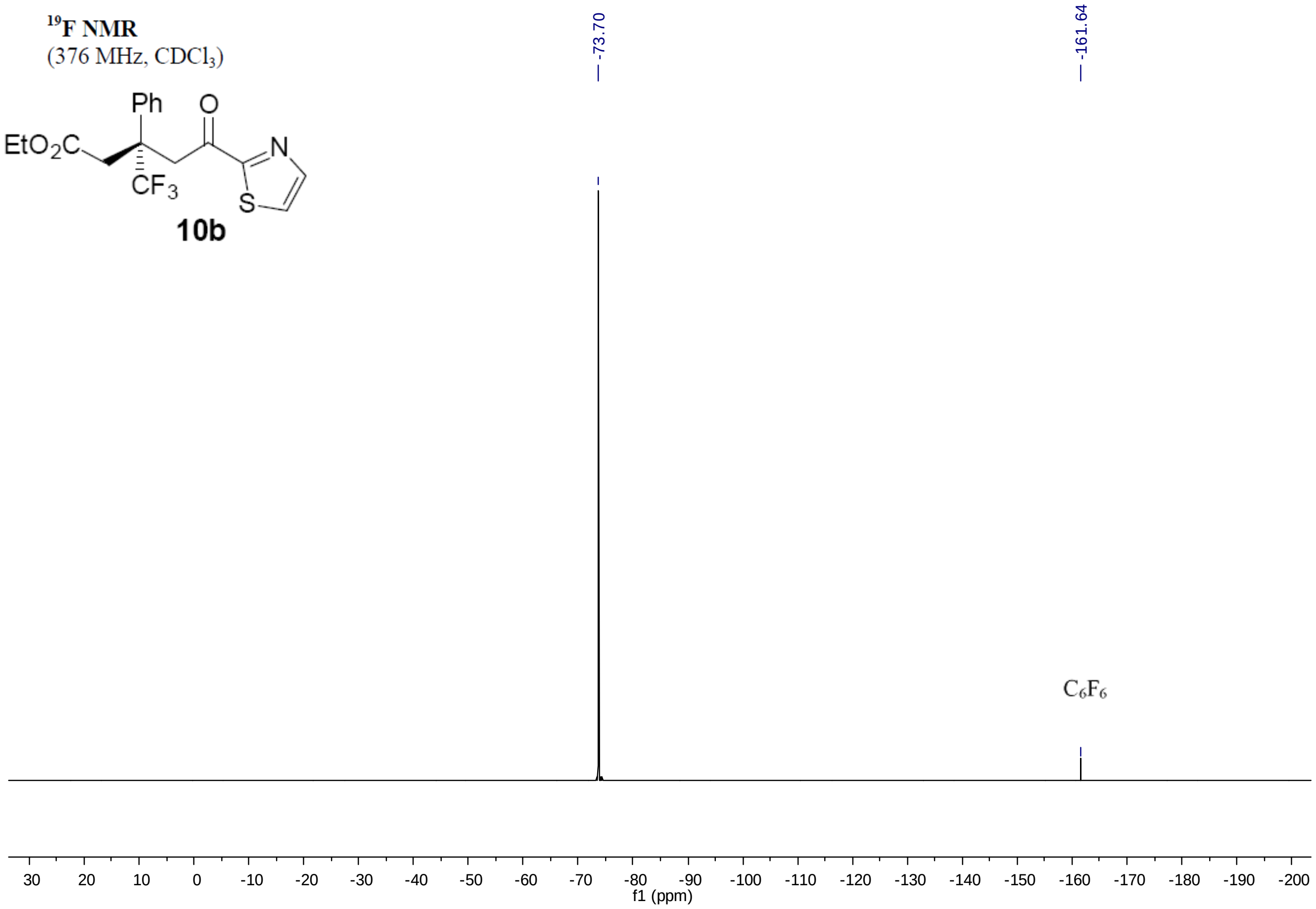

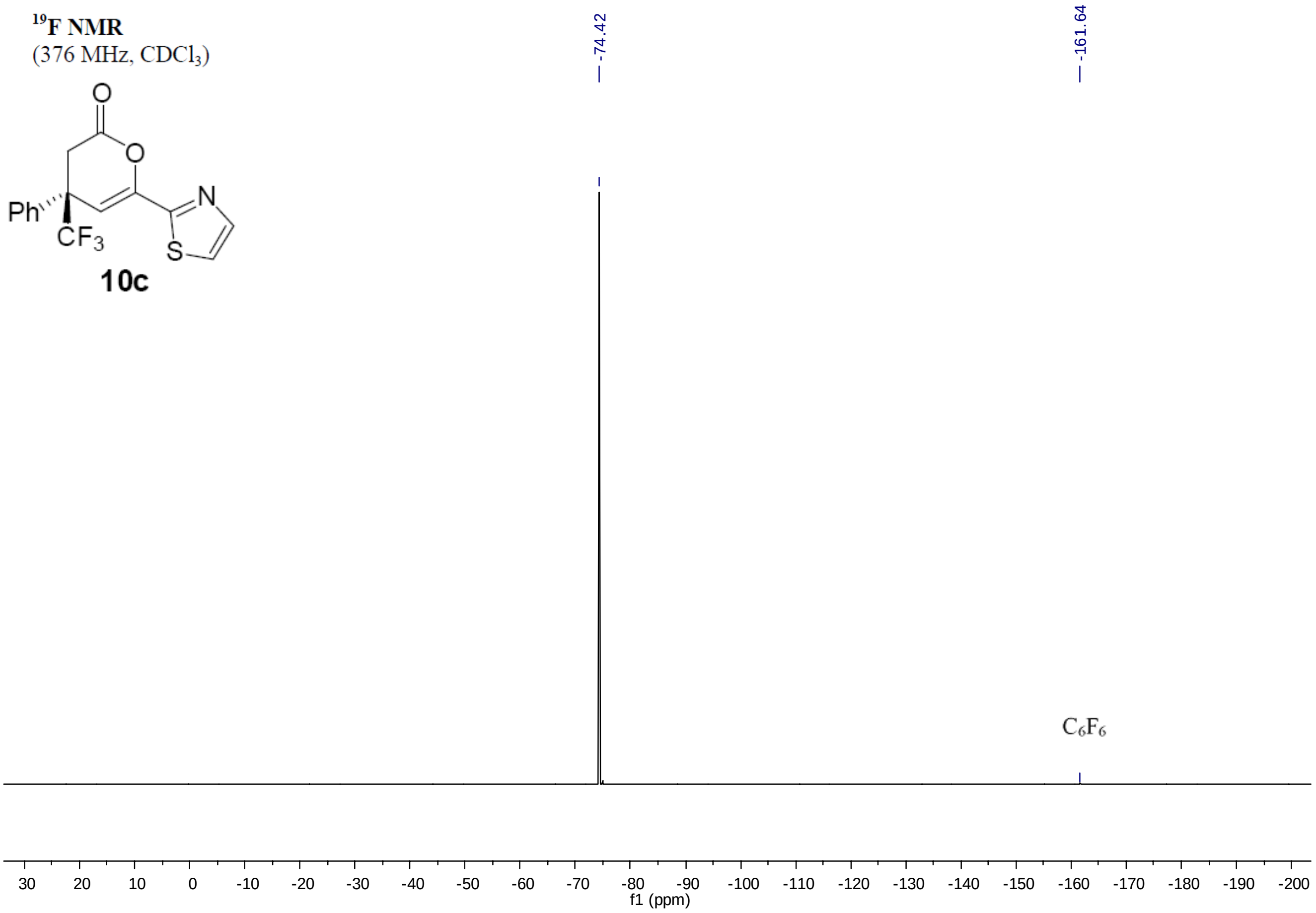

19F NMR
(376 MHz, CDCl3)
-74.42
-161.64
Ph
CF3
10c
C6F6
f1 (ppm)

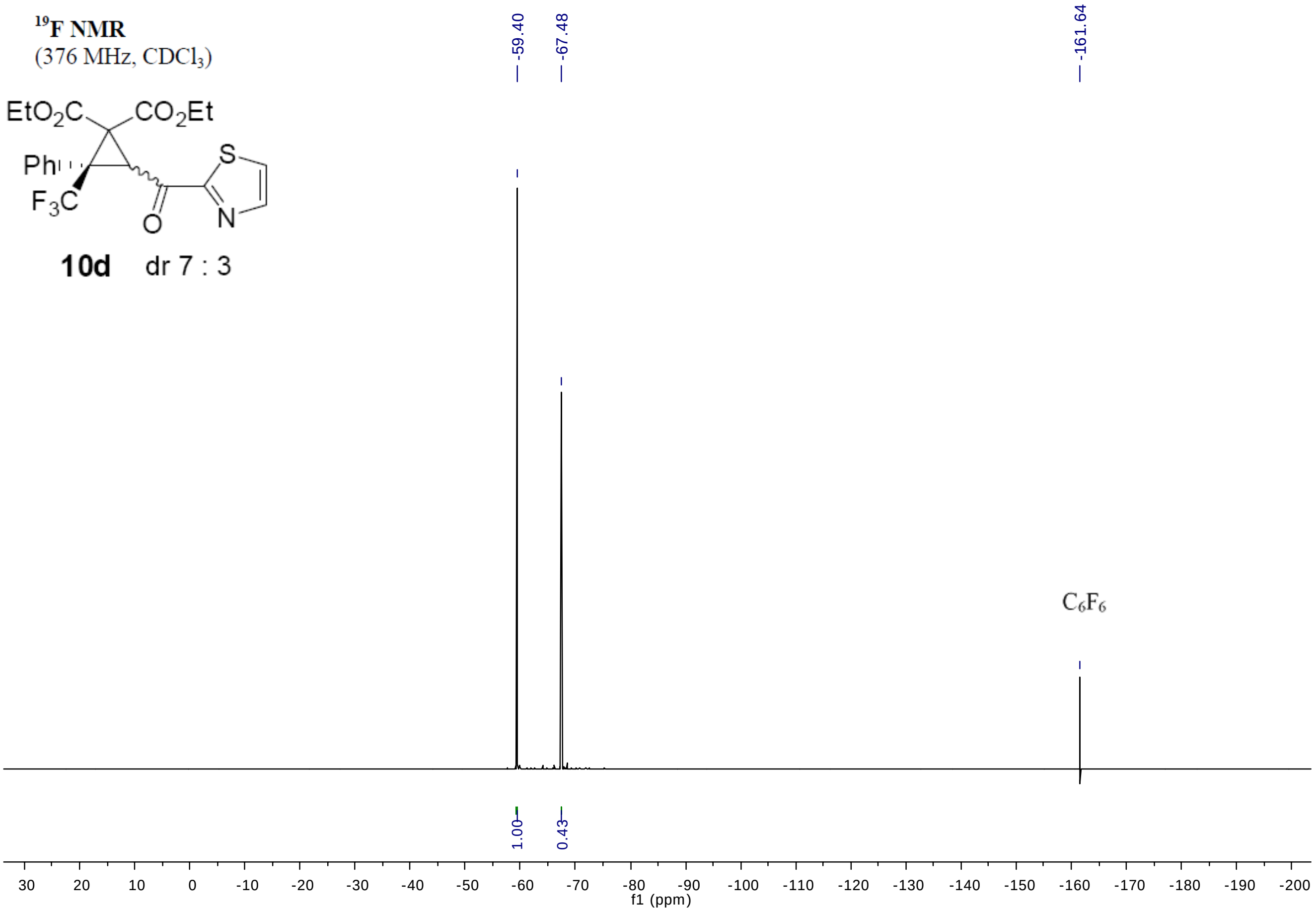

19F NMR
(376 MHz, CDCl3)
-59.40
-67.48
-161.64
EtO2C
CO2Et
Ph
F3C
10d
dr 7 : 3
C6F6
1.00
0.43
f1 (ppm)

^{19}F NMR
(376 MHz, $CDCl_3$)

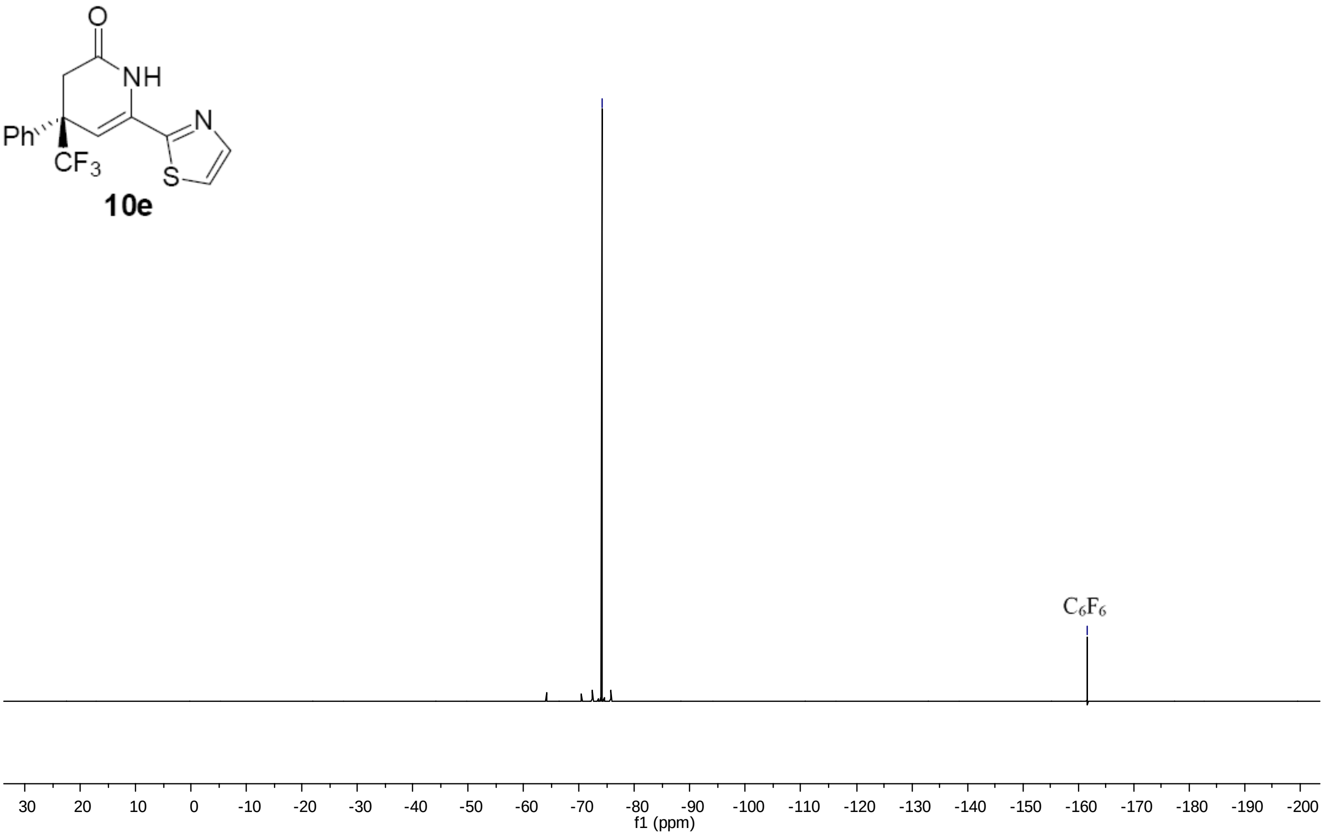

^{19}F NMR
(376 MHz, $(CD_3)_2SO$)

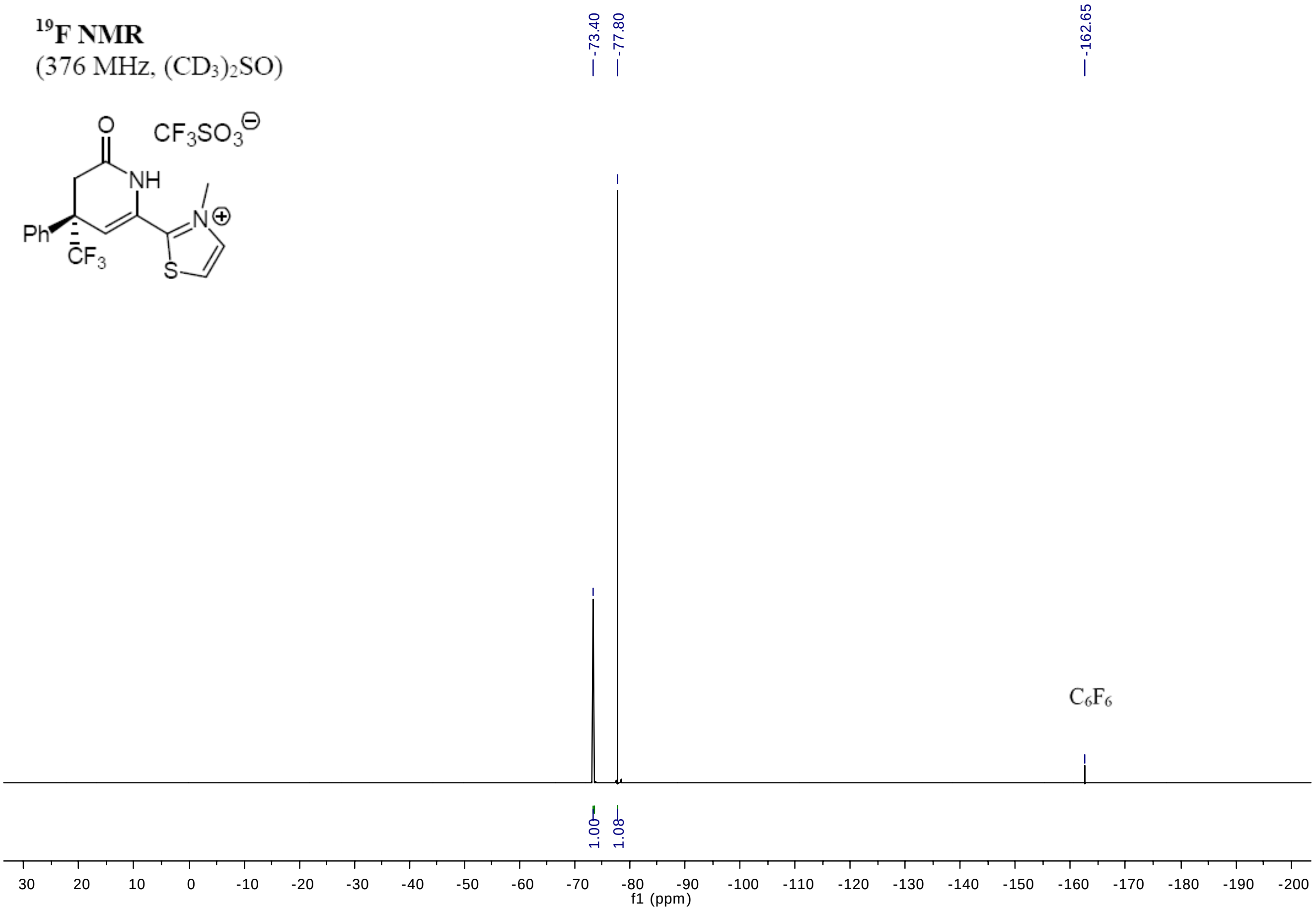

^{19}F NMR
(376 MHz, $CDCl_3$)

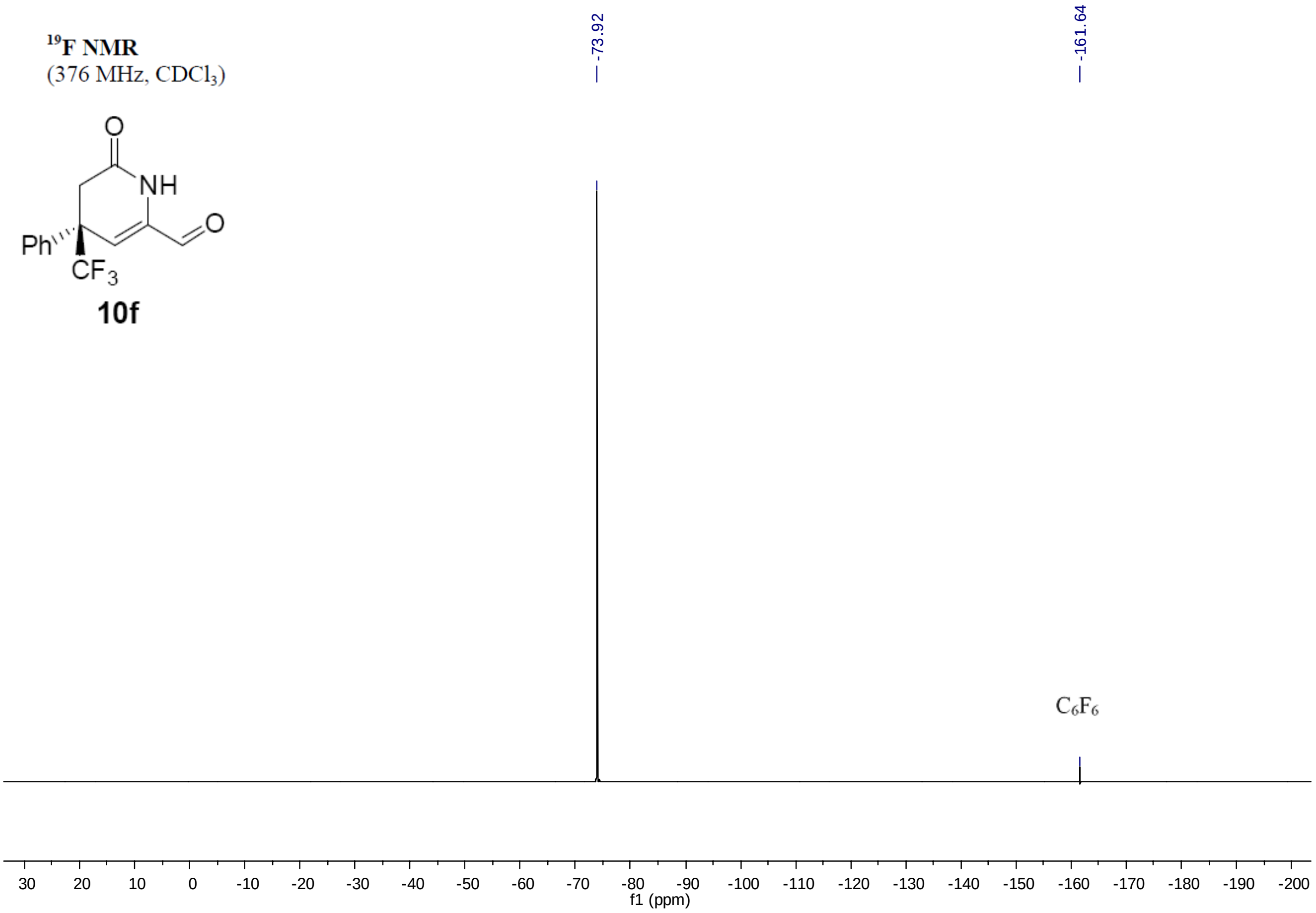

10f

^{19}F NMR
(376 MHz, $CDCl_3$)

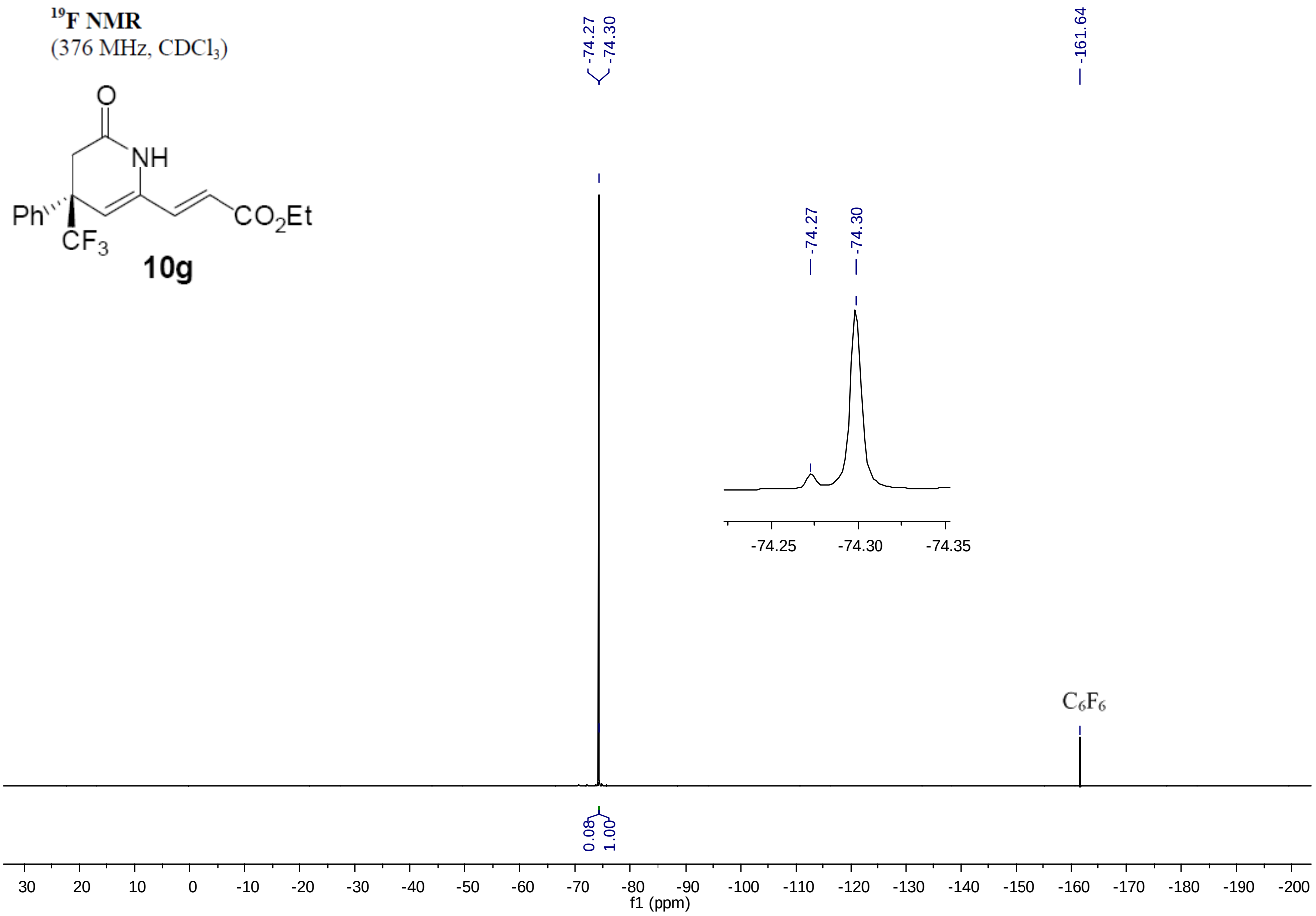

10g

Copies of DEPT 135 spectra - compounds 10a-10g, obtained from adduct 3h

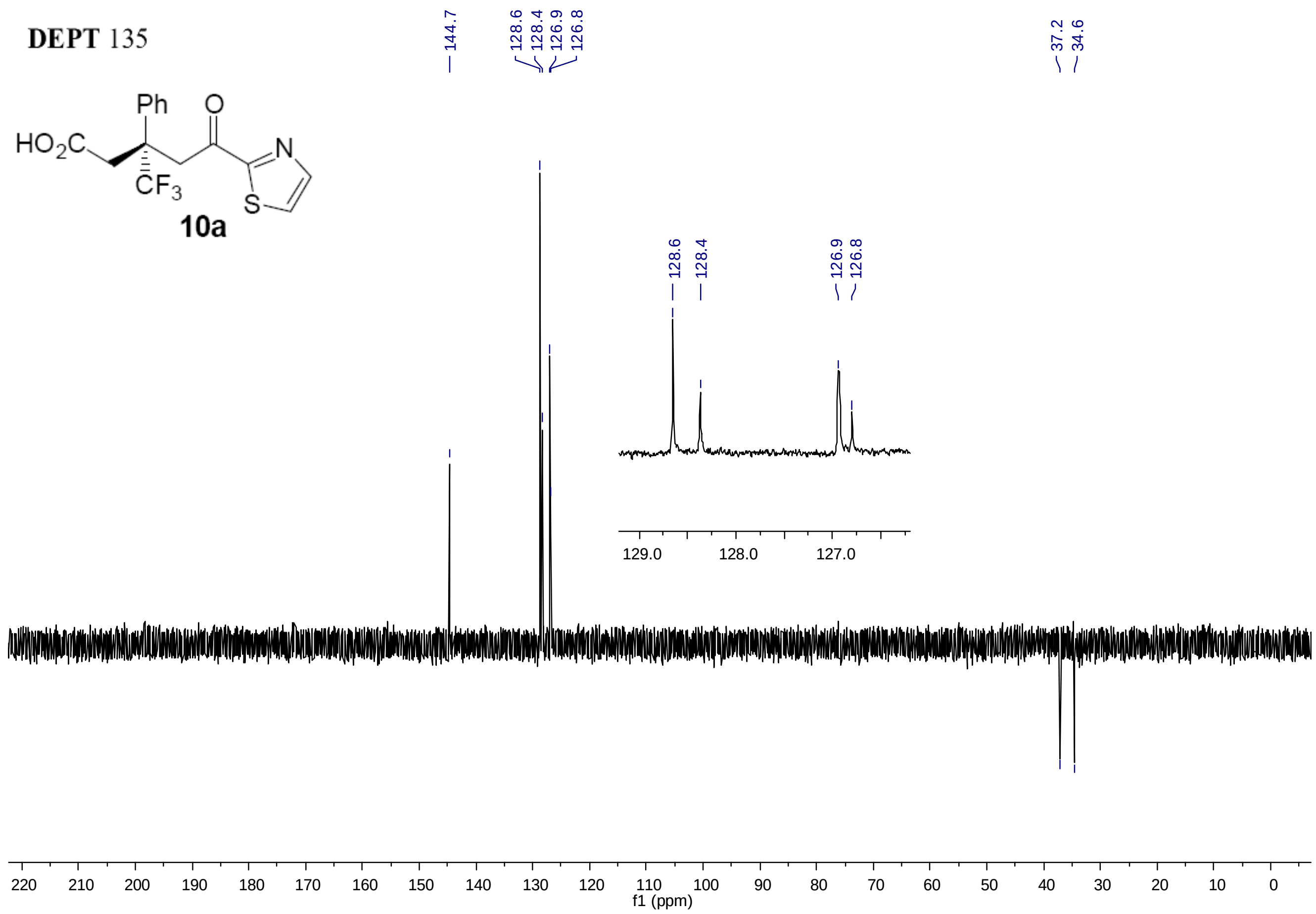

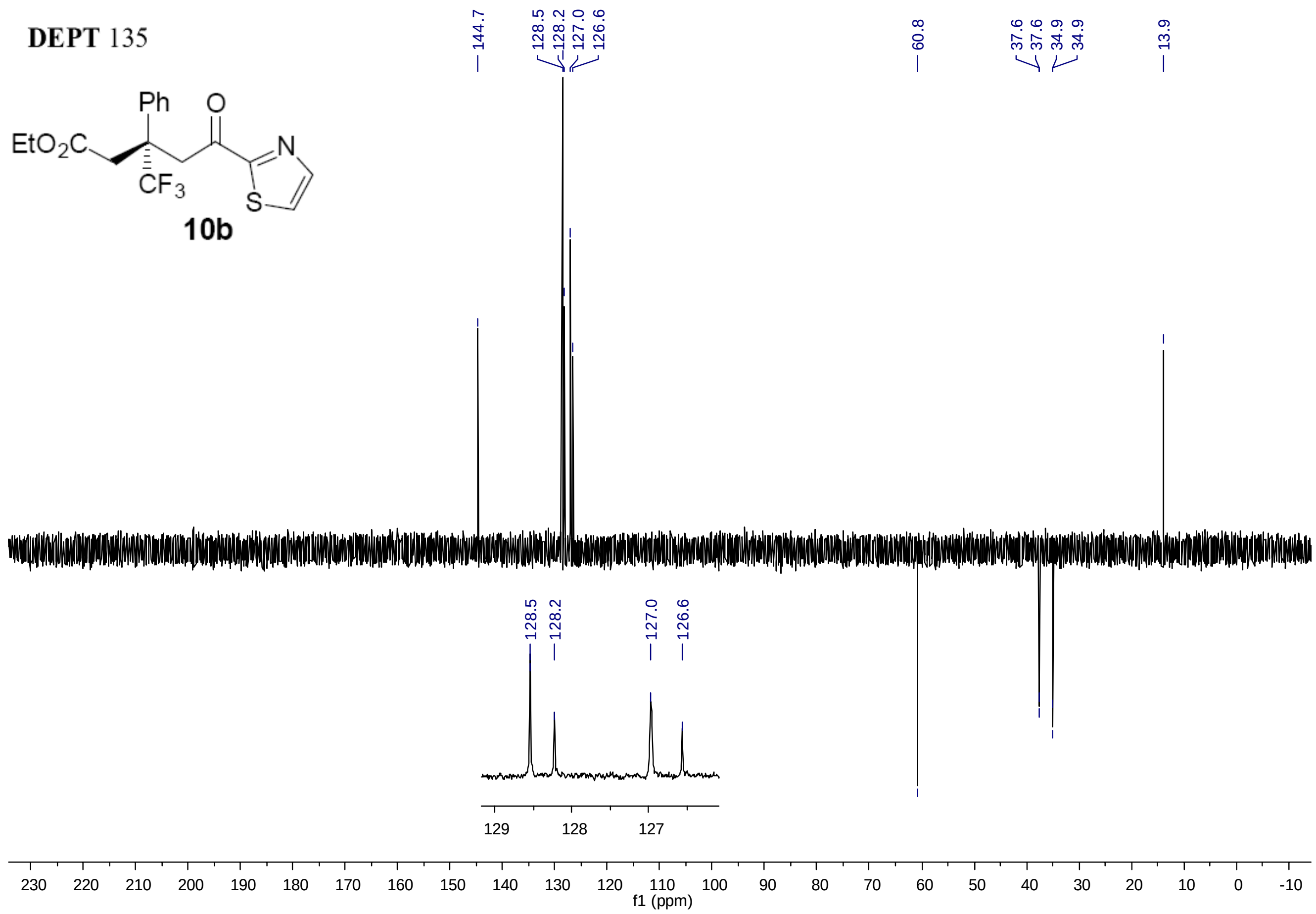

DEPT 135

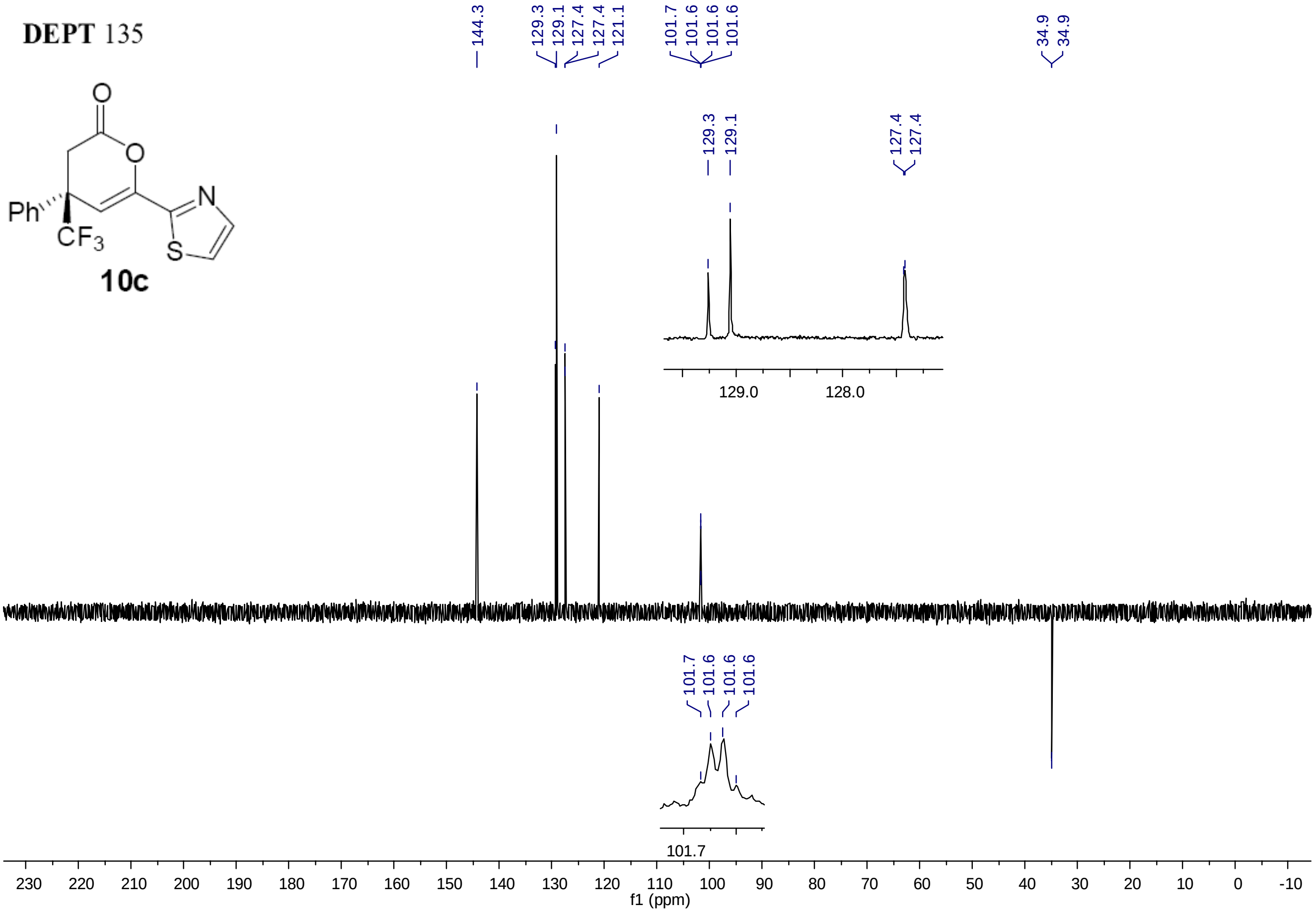

10c

DEPT 135

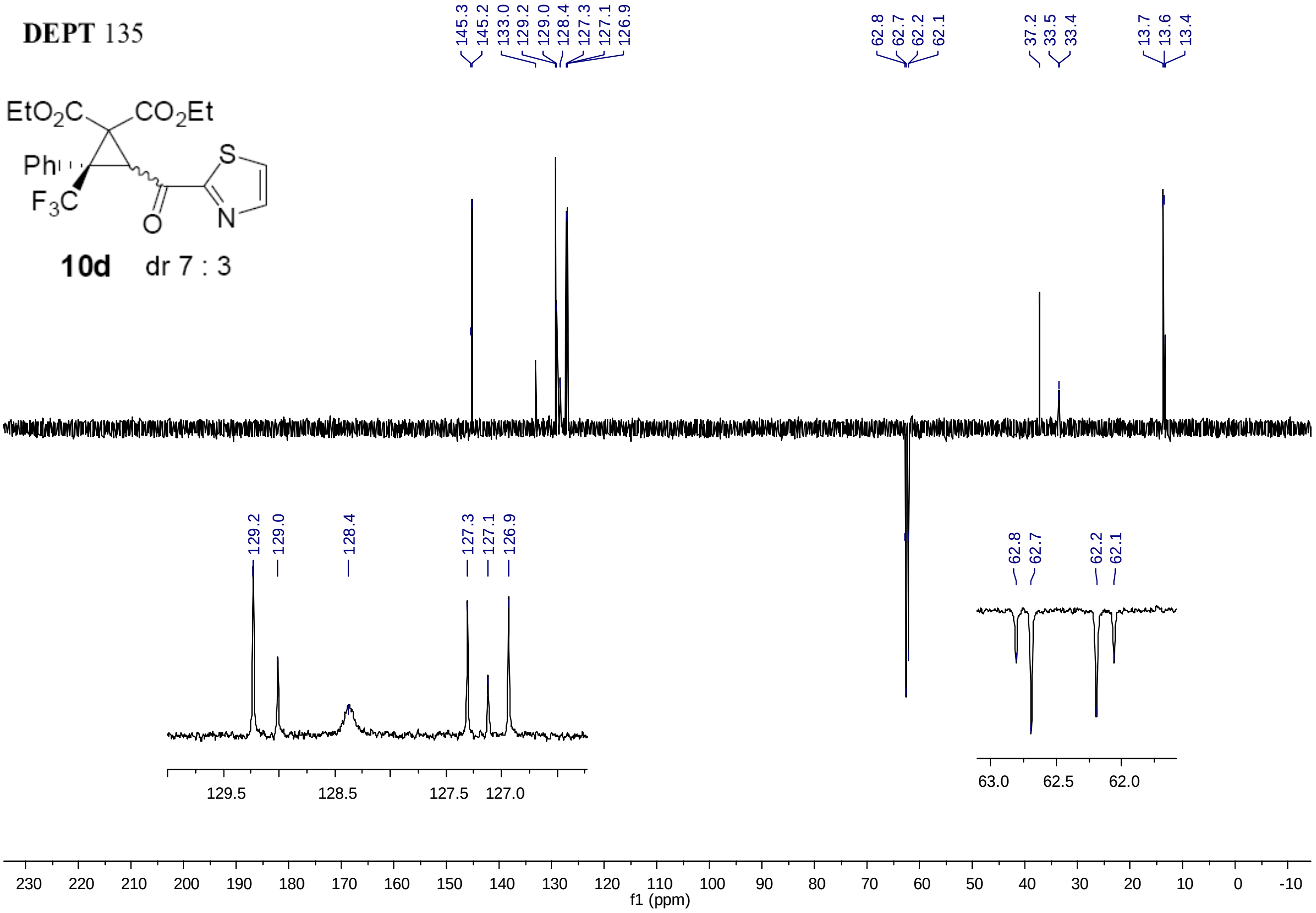

10d dr 7 : 3

DEPT 135

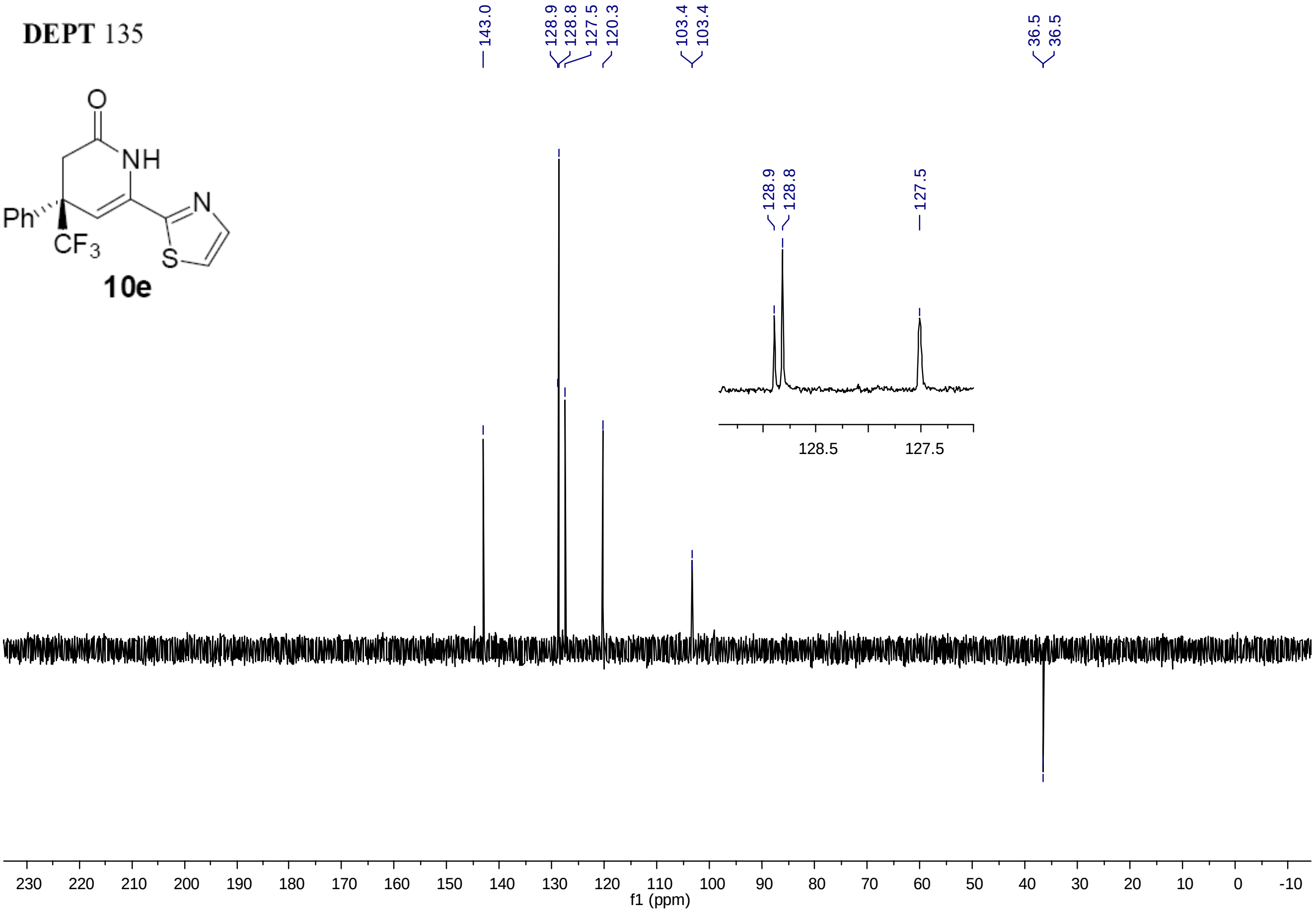

DEPT 135

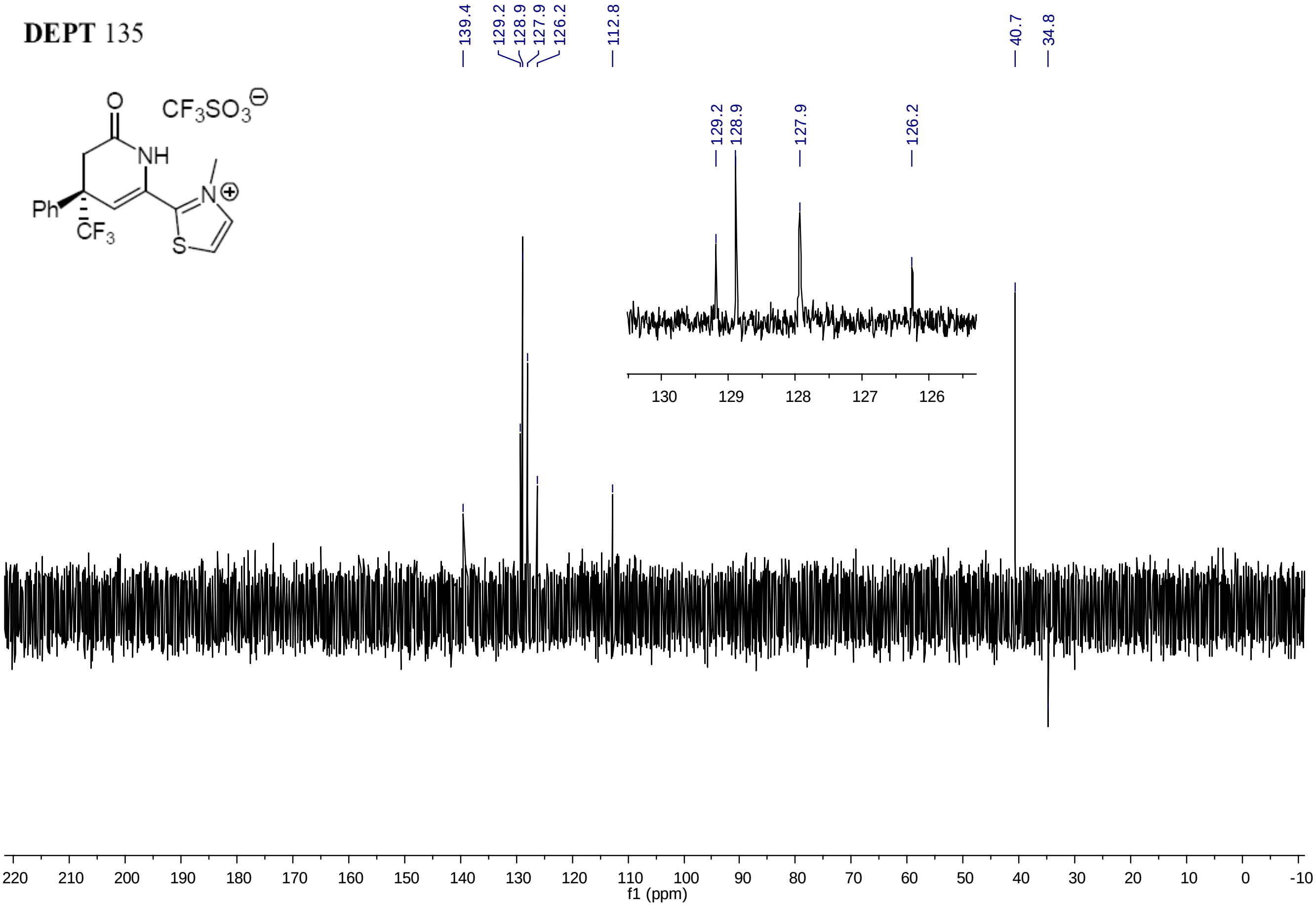

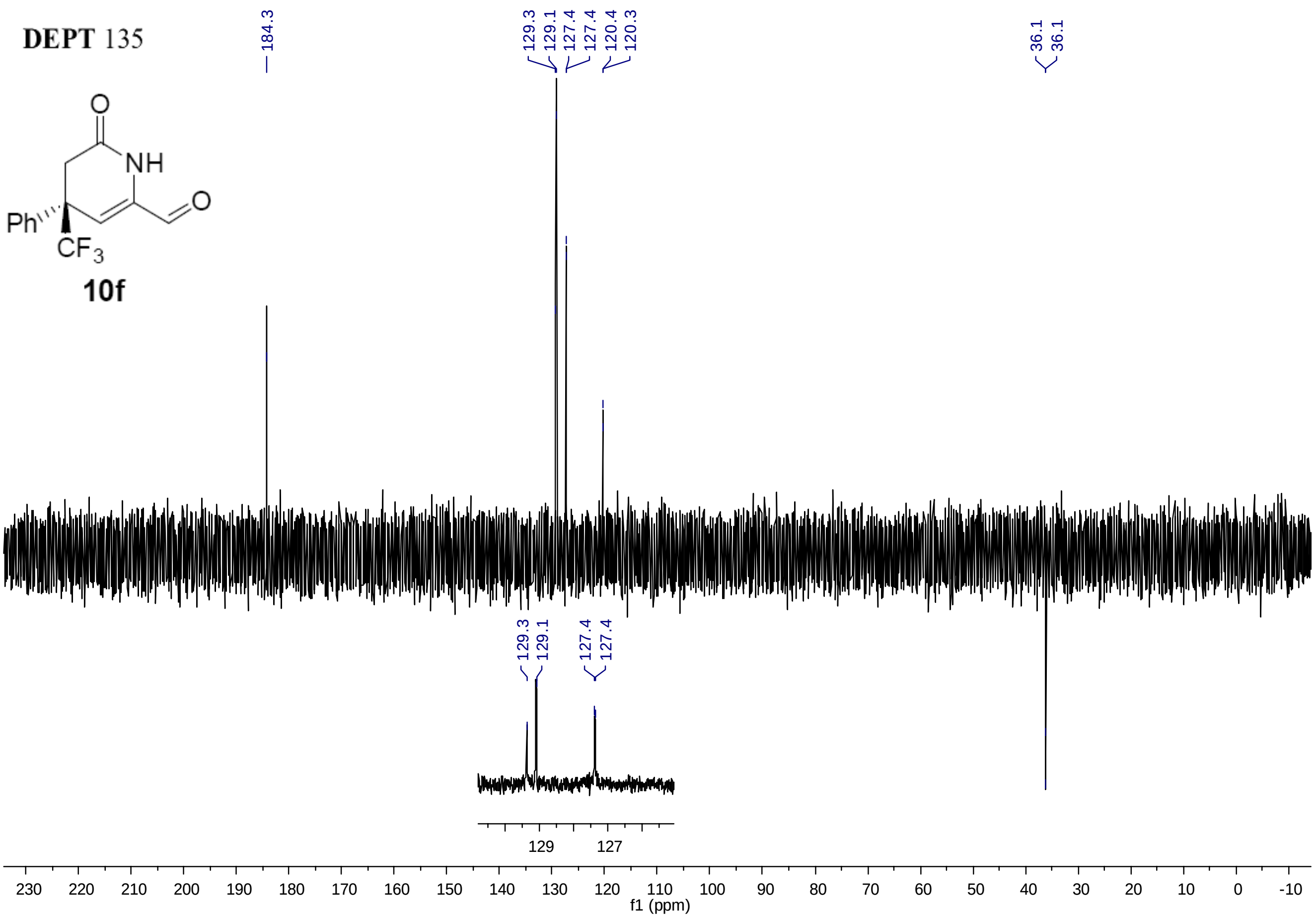
DEPT 135
10f
O
NH
Ph
CF3
184.3
129.3
129.1
127.4
127.4
120.4
120.3
36.1
36.1
129
127
f1 (ppm)

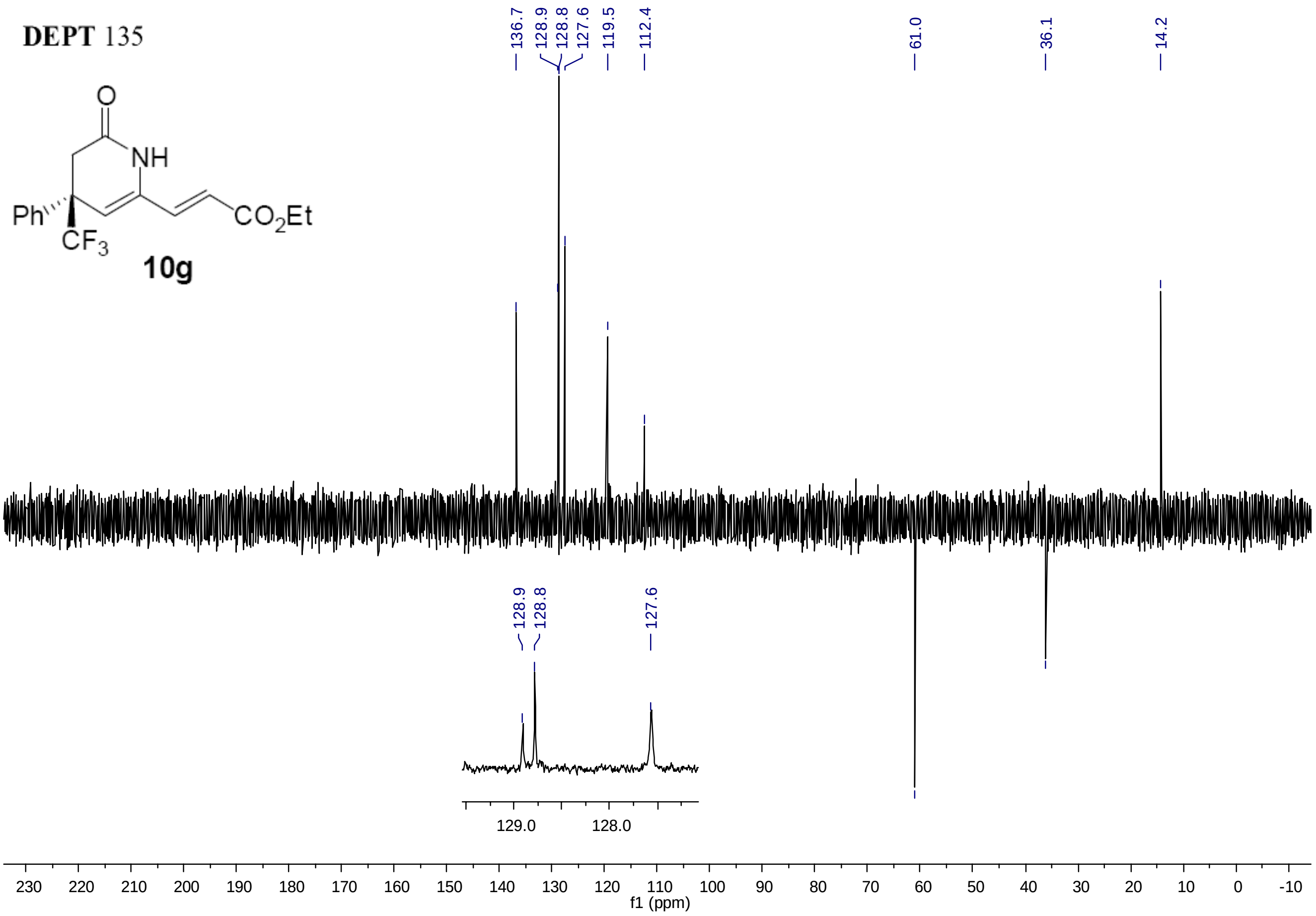
DEPT 135
10g
O
NH
Ph
CF3
CO2Et
136.7
128.9
128.8
127.6
119.5
112.4
61.0
36.1
14.2
129.0
128.0
f1 (ppm)
